# What Can We Learn from Global Sensitivity Analysis of Biochemical Systems?

**DOI:** 10.1371/journal.pone.0079244

**Published:** 2013-11-14

**Authors:** Edward Kent, Stefan Neumann, Ursula Kummer, Pedro Mendes

**Affiliations:** 1 School of Computer Science and Manchester Institute of Biotechnology, The University of Manchester, Manchester, United Kingdom; 2 Department for Modeling of Biological Processes, Das Centre for Organismal Studies Heidelberg/BIOQUANT, Heidelberg, Germany; 3 Virginia Bioinformatics Institute, Virginia Tech, Blacksburg, Virginia, United States of America; Universidad de La Laguna, Spain

## Abstract

Most biological models of intermediate size, and probably all large models, need to cope with the fact that many of their parameter values are unknown. In addition, it may not be possible to identify these values unambiguously on the basis of experimental data. This poses the question how reliable predictions made using such models are. Sensitivity analysis is commonly used to measure the impact of each model parameter on its variables. However, the results of such analyses can be dependent on an exact set of parameter values due to nonlinearity. To mitigate this problem, global sensitivity analysis techniques are used to calculate parameter sensitivities in a wider parameter space. We applied global sensitivity analysis to a selection of five signalling and metabolic models, several of which incorporate experimentally well-determined parameters. Assuming these models represent physiological reality, we explored how the results could change under increasing amounts of parameter uncertainty. Our results show that parameter sensitivities calculated with the physiological parameter values are not necessarily the most frequently observed under random sampling, even in a small interval around the physiological values. Often multimodal distributions were observed. Unsurprisingly, the range of possible sensitivity coefficient values increased with the level of parameter uncertainty, though the amount of parameter uncertainty at which the pattern of control was able to change differed among the models analysed. We suggest that this level of uncertainty can be used as a global measure of model robustness. Finally a comparison of different global sensitivity analysis techniques shows that, if high-throughput computing resources are available, then random sampling may actually be the most suitable technique.

## Introduction

By far the most frequently-used method for modelling biological systems is to describe their reaction networks through ordinary differential equations (ODEs) [1]. Rate equations are constructed to describe the time-dependent change of the value of model variables as a function of each other. Such rate equations can be used to describe various types of enzyme-catalysed biochemical reactions, such as metabolic, signalling and gene networks, among others. These models require the use of specific parameters that represent physical interactions and processes such as rate constants, Michaelis constants, and binding affinities.

A general issue in mathematical modelling is the choice of parameter values, which should reflect the properties of the real system. Unfortunately it is frequently impossible to determine or estimate what those values should be, and thus the accuracy of many parameter values is often questionable. Parameter values are obtained from a variety of different sources, including *in vitro* and *in vivo* experimental data. *In vitro* experiments do not necessarily match the conditions *in vivo*, while parameter fitting often results in ambiguous parameter sets, i.e. where groups of parameters have strong correlations and the individual values are not identifiable [2]. Sometimes models include parameter values that were estimated from experiments with different biological systems than the target one. Values may also be adopted based only on high-level constraints (like considering a rate constant to be limited by diffusion, or an equilibrium constant limited by thermodynamic constraints). All of these constitute sources of uncertainty about parameter values, and some models may be dominated by such issues. It is therefore important that models be carefully studied to expose how much the parameter values actually affect the modelling results. One should be cautious with strong conclusions that are based on model properties that depend strongly on parameters with high uncertainty.

To deal with such uncertainty, the related fields of uncertainty and sensitivity analysis have developed. Uncertainty analysis is used to quantify the uncertainty in the output of a model that is generated by uncertainty in the inputs, while sensitivity analysis is used to assess the relative importance of the inputs of a model on the output [3].

A common method for performing sensitivity analysis is to calculate sensitivity coefficients, which measure the impact of parameters on the variables of the model (state properties). These are mathematically equal to the partial derivatives of the state properties by each parameter:
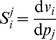
(1)where 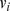 is the variable and 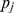 the parameter. When it is useful to remove scale from sensitivity coefficients, a scaled version of the partial derivative is used instead:
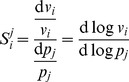
(2)


A specialised form of sensitivity analysis that is quite widely used is metabolic control analysis (MCA) [4], [5] which assesses how much systems-level properties, such as a steady-state concentrations or fluxes, depend on the velocity of individual reactions. MCA expresses these sensitivities through so-called control coefficients [6], which are scaled sensitivity coefficients taken on parameters that affect the rate of reaction linearly (such as a 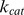 or 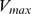, but not a 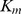). In MCA terminology [6], generic sensitivity coefficients taken on arbitrary model parameters are known as response coefficients (control coefficients are therefore special cases of response coefficients).

Sensitivity analysis can be also used as a way to assess the robustness of a model. Given the fact that most parameters depend on some properties of the environment, which is subject to stochastic variations (e.g. local fluctuations in temperature), a robust system will be resilient to relatively small perturbations in parameter values. This will be reflected in low-magnitude sensitivity coefficients [7], [8]. Conversely, a sensitive system will have high-magnitude sensitivity coefficients, indicating that for those parameters, relatively small perturbations will have a large effect on the system (this is the case in some sensory systems, such as retina cells that are extremely sensitive to light [9]).

Typically sensitivity analysis is applied to a specific operating point of the model, *i.e.* around a specific value of each parameter. This follows the definitions in Eqs. 1 and 2 which are based on the concept of infinitesimal changes of calculus. Thus each parameter is perturbed by a small magnitude while holding all other parameters constant. In this case we refer to *local* sensitivity analysis to emphasise the fact that the sensitivity coefficients depend on the specific set of parameter values used. Because all but the most trivial of kinetic models are nonlinear, the values of sensitivity coefficients are different at different operating points of a model. Therefore there is the possibility that, for a certain model, some parameters may be deemed unimportant by this type of local sensitivity analysis which may have a strong effect (large control) in other regions of parameter space. For example, after changing the expression of a particular enzyme, the distribution of control (the spectrum of sensitivity coefficients) may be completely different from the original one. Given the uncertainty surrounding many parameter values as discussed above, it is clear that the insights gained from local sensitivity analysis should be considered with a great deal of caution. After all, if the real value of some parameter is considerably different from what was assigned in the model, the entire set of sensitivity coefficients of the model will have little resemblance to those of the real system.

Global sensitivity analysis techniques attempt to avoid this weakness by calculating sensitivity coefficient values in broader regions of parameter space either surrounding the fixed initial values defined in the model or simply by selecting appropriate ranges. Therefore, while a local sensitivity analysis will generate a single sensitivity coefficient for each perturbed parameter, a global sensitivity analysis will yield a range of possible values, depending on the parameter set used. The range of potential sensitivities for a particular parameter may span several orders of magnitude, suggesting that an accurate parameter set is vital to determine whether or not the parameter has high control. If the range of potential sensitivities for a parameter contains only high-magnitude values, we can infer that the parameter has high control irrespective of the exact physiological parameter set, while only low-magnitude values would suggest that that the parameter can only exert low control. Finally, the potential sensitivities for a parameter can span both positive and negative values, indicating that the parameter could potentially exert positive or negative control on the system, depending on the parameter set used.

A common way of performing a global sensitivity analysis is to carry out a Monte Carlo simulation where derivative-based sensitivities are sampled at random [10]–[13]. For each sampled parameter set, a sensitivity analysis is performed, and the sensitivity coefficients are recorded. After taking enough random samples, plots showing the distribution of sensitivity coefficients within the parameter space can be produced. This method is relatively simple to implement, and can be performed in software such as COPASI [14]. The downside is that large numbers of samples must be taken, particularly for models with large numbers of parameters, since the parameter space grows exponentially with the number of parameters. Therefore, sampling enough parameter sets can require vast amounts of computing power. When analysing the distributions of sampled sensitivity coefficient values, it is tempting to assume that the sensitivities of the real physiological system are located around the centre of the distribution; indeed, methods based on this assumption have been previously proposed [15], [16].

An alternative method for global sensitivity analysis is to use optimisations to find the boundaries of the sensitivity values [17]. As with the sampling-based technique, a parameter space must first be defined. However, rather than sampling the entire parameter space, a numerical optimisation algorithm is used to find the upper and lower bounds on the values each sensitivity coefficient can take. For each sensitivity coefficient to be calculated, two optimisations must be run — one to maximise the sensitivity coefficient value, and another to minimise it. This technique can be much more computationally efficient than random sampling if one uses efficient global optimisation algorithms. While it is impossible to determine if the optimisation algorithm has reached a true global maximum or minimum, in practice methods such as particle swarm [18] or evolutionary algorithms are able to approximate those values fairly efficiently. Because of their efficiency in searching optima, these algorithms do not characterise the distributions of sensitivity coefficients, though. This method is not yet widely used, and its performance compared to traditional sampling-based techniques is untested.

Other popular global sensitivity analysis techniques are the sampling-based Sobol [19] and Morris [20] methods (reviewed in [10]), the variance-based extended Fourier amplitude sensitivity test (eFAST) [21] and partial rank correlation coefficients (reviewed in [3]). These techniques are able to rank the inputs of a model based on importance, though do not give a complete picture of the behaviour of each sensitivity coefficient in the chosen parameter space. In addition, previous studies have shown that the results of these methods can be quite different [22]–[24], though the reasons for this are not well understood [12].

For this study, we performed global sensitivity analysis using the derivative-based sampling and optimisation-based techniques. These methods were chosen since they provide a rich picture of the behaviour of the sensitivity coefficients of the system in a given parameter space (particularly the derivative-based sampling technique), they provide outputs which can be directly compared with a local sensitivity analysis, and they are amenable to running in parallel with little effort, giving the opportunity to vastly reduce computation times.

Global sensitivity analysis was applied to a selection of five models. These models were selected on the basis that they represent well-studied systems and most contain a large number of experimentally determined parameters. We evaluated how robust these models were to growing intervals of uncertainty in parameter values, and what kind of insights can be gained by analysing distributions of sensitivities. We also analysed how the assumed real or physiological sensitivities (published with the models) are situated within the sampled distributions. Finally, for several of the models, we compared the performance of the random sampling and optimisation-based global sensitivity analysis approaches.

## Results

Models of biochemical networks cover a wide range of stoichiometric structures and dynamics. The behaviours displayed by these models can be quite diverse due to many different factors, and accordingly it is expected that they also display different properties with respect to parameter sensitivities. Therefore, this study covers a range of models attempting to expose different scenarios regarding global parameter sensitivities. Models were chosen based on their stoichiometric structure (metabolic/signalling) and dynamics (stationary/periodic). An emphasis was also put on models which have a solid experimental basis, assuming that their parameter values are close to their physiological value. The models chosen were of stationary MAPK signalling [25], oscillatory NF
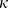
B signalling [26], oscillatory eukaryotic cell cycle [27],and metabolic models of the glycolytic pathway in *Saccharomyces cerevisiae* [28] and *Trypanosoma brucei* [29].

The behaviour of each of the models was explored using sampling-based global sensitivity analysis. In addition, we carried out analyses using the optimisation-based approach [17] on the three signalling models in order to compare the performance of the two techniques. The results for each model are summarised below, with the full output of each analysis presented in the Supporting Information.

### MAPK Model

The first model analysed is a model of MAPK signalling published by Huang *et al.* [25]. This model includes 22 components of a “canonical” MAPK signalling pathway amounting to 30 parameters. With the published parameter values, the model runs to a stable steady state. We calculated sensitivities for each parameter in the model on the steady-state concentration of doubly-phosphorylated MAPK (MAPK-PP). MAPK-PP is the final link of the signal transduction cascade, and its concentration can be considered the output of the model. If the sensitivity of any parameter on this concentration is large, it could mean that the signal would be susceptible to environmental noise (through that parameter); it would also mean that an accurate determination of the values of this parameter would be very important.

**Figure 1:**
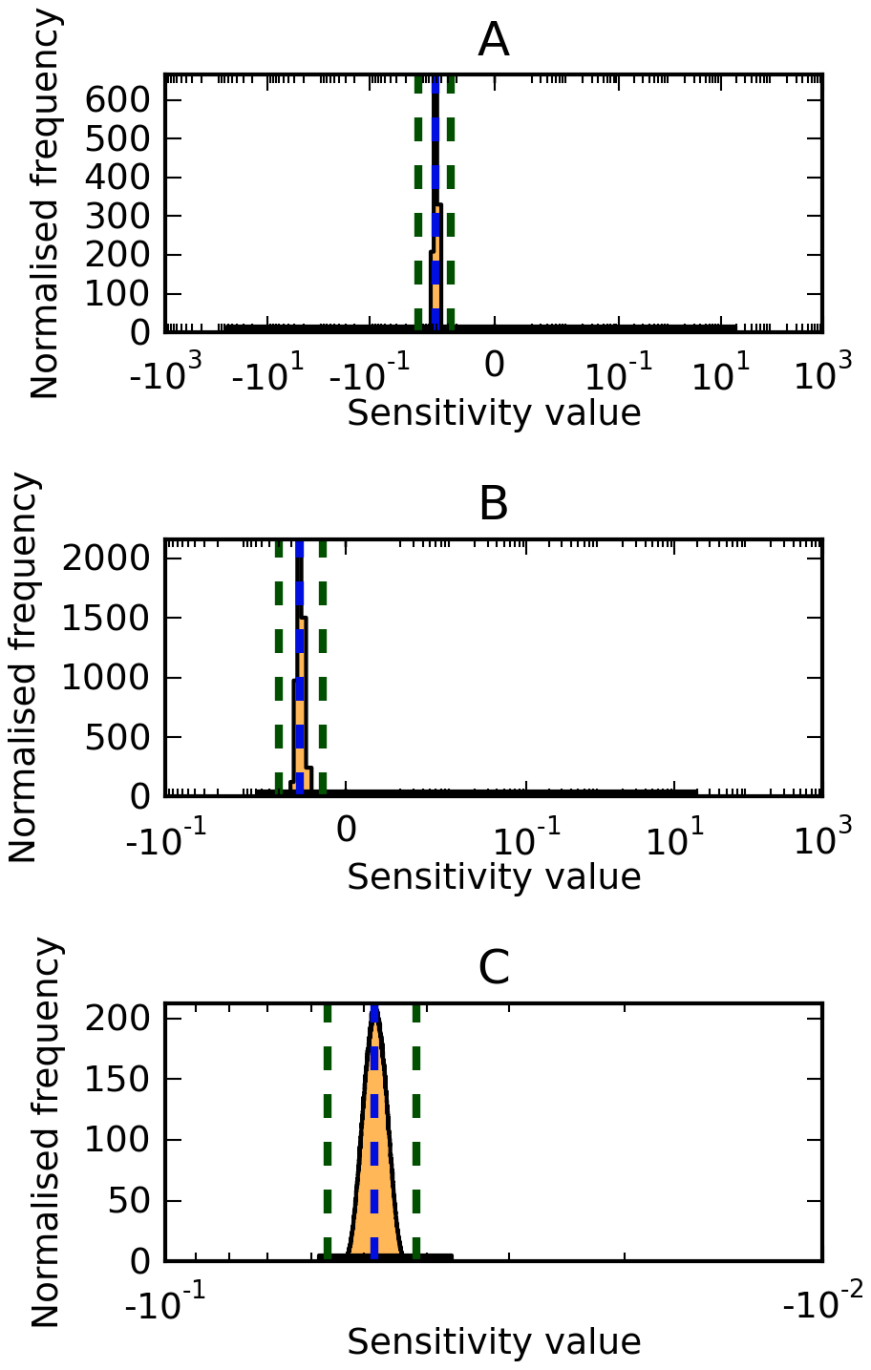
Selected global sensitivity analysis results for the MAPK model [25]. Shown are the results for parameter 2 (*MAPKKK activation.k1*) (A), parameter 5 (*binding MAPK—Pase and P-MAPK.k1*) (B), and parameter 7 (*binding MAPK—Pase and PP—MAPK.k1*) (C) in the parameter space of the reference values 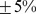. The distributions show the results of the sampling-based technique, with the sampling frequency normalised such that area under the curve is always unity. The bounds found by the optimisation technique are displayed as dashed green lines, and the local sensitivity value is shown as a dashed blue line. The full results for all parameters are shown in Table S1 in File S1.

Local sensitivity analysis of the original parameters shows that no single parameter has a large effect on the variable. The blue lines in Figure 1 depict these local sensitivity coefficients in the reference parameter set.

We carried out random sampling (uniform) of parameter values to assess the sensitivities of the parameters in a more global way. For each sensitivity coefficient, a histogram was produced showing the distribution of sampled sensitivity coefficient values. On these histograms the ‘local’ sensitivity value obtained with the reference parameter set is shown as a dotted blue line, and zero is shown as a dashed purple line. In addition, for each distribution, we calculated the normalised height of the peak (frequency/total number of samples), the sensitivity value at which the peak is located, and performed a Shapiro-Wilk Normality test. Results for selected parameters are displayed in Figure 1, and the full results are presented in Table S1 in File S1. We summarise the results below.

**Figure 2:**
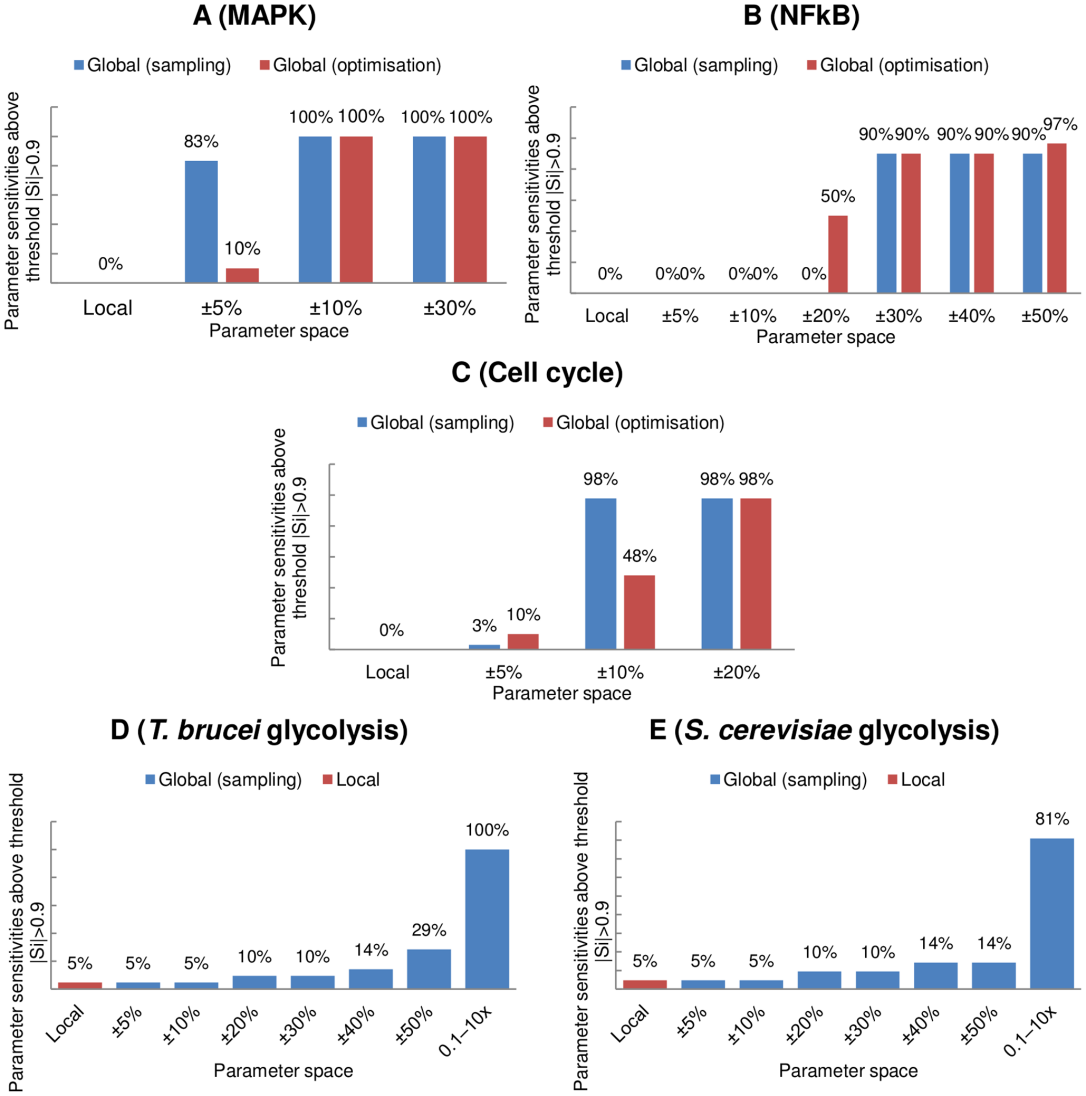
Summary of model robustness. As a benchmark for model robustness, we calculated the percentage of parameters with a potential sensitivity coefficient of magnitude 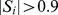. Parameter sensitivities were calculated with respect to (A) the concentration of PP-MAPK, (B) the period of cell division, (C) the period of oscillation of nuclear-NF
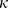
B, and (D, E) the flux through the hexokinase reactions. For (A–E), local and sampling-based global sensitivity analysis was performed, and for (A–C) optimisation-based global sensitivity analysis was also performed.

Expanding the parameter space by only 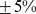 around the original parameter set shows — in contrast to the parameter set studied by Huang *et al.* [25] — that a number of parameters may be able to gain a high degree of control over [MAPK-PP] (Figure 2), with sensitivity coefficients ranging between 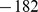 and 218. In many cases, large-magnitude sensitivity coefficients are found on one direction only (for example parameter 5 which can only gain high-magnitude positive control). In some cases, parameters could gain high-magnitude positive and negative control (for example parameter 2), while other parameters could only gain low-magnitude control close to the local sensitivity value (for example parameter 7). It should be emphasized that a 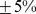 variation is a tiny variation considering natural fluctuation within every biological system, and also relative to the typical level of precision of measurements.

The distributions obtained by random sampling were Gaussian-like for some parameters (such as parameters 7 and 25), but the majority were sharp and narrow.

Larger domains of variation for parameter values were also considered. Parameter values were sampled 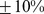 and 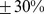 around their reference values. In both cases, the sampling method was able to find large-magnitude positive and negative sensitivity coefficients for every parameter (Figure 2), implying all parameters can, in principle, exert extremely large positive or negative control over [MAPK-PP]. It should be noted that, even though high-magnitude sensitivity coefficients occur at the edges of the distribution, the narrow distribution peaks show that, in the vast majority of cases, the sampled sensitivity coefficient values are close to that of the reference model.

Another way to study sensitivity analysis in a more global way is to ask the question of how large and how small can a sensitivity coefficient be given any possible value for the parameters (inside a certain domain) [17]. We applied this strategy using the particle swarm optimisation algorithm (see Methods). The results of the optimisations, showing the upper and lower bounds on the values a sensitivity coefficient can take, are displayed in Figure 1 as dashed green lines, superimposed on the distributions obtained by sampling.

In the case of 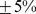 and 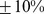 parameter variations, the optimisations performed poorly, frequently finding only low-magnitude control for parameters which the sampling technique was able to find high-magnitude control (Figure 1). This is most likely due to the optimisations having not converged to the global optimum. The optimisation method performed better than the random sampling method in the case of the 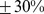 variation, often finding sensitivity coefficients several orders of magnitude larger than the sampling method (for example parameter 2, where sampling found a minimum control of approximately 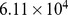 versus 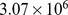 found by the optimisation). However, in these cases, both values are so large as to make little difference.

In summary, the MAPK model of Huang *et al.* [25] shows that if the parameter values had only 5% or more uncertainty, the model behaviour, and therefore any conclusions from it, could be considerably different. Full results for this model are given in Table S1 in File S1.

### NF
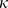
B Model

The second model represents NF
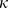
B signalling in mouse embryonic fibroblast cells, published by Ashall *et al.* [26]. The model contains 14 variables and 27 parameters. With the published parameter values, this model exhibits stable oscillations, the period and amplitude of which are thought to play a role in regulating gene expression. Therefore, a relevant sensitivity is that relating the effect of parameter values on the period of the oscillations of nuclear NF
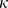
B.

**Figure 3:**
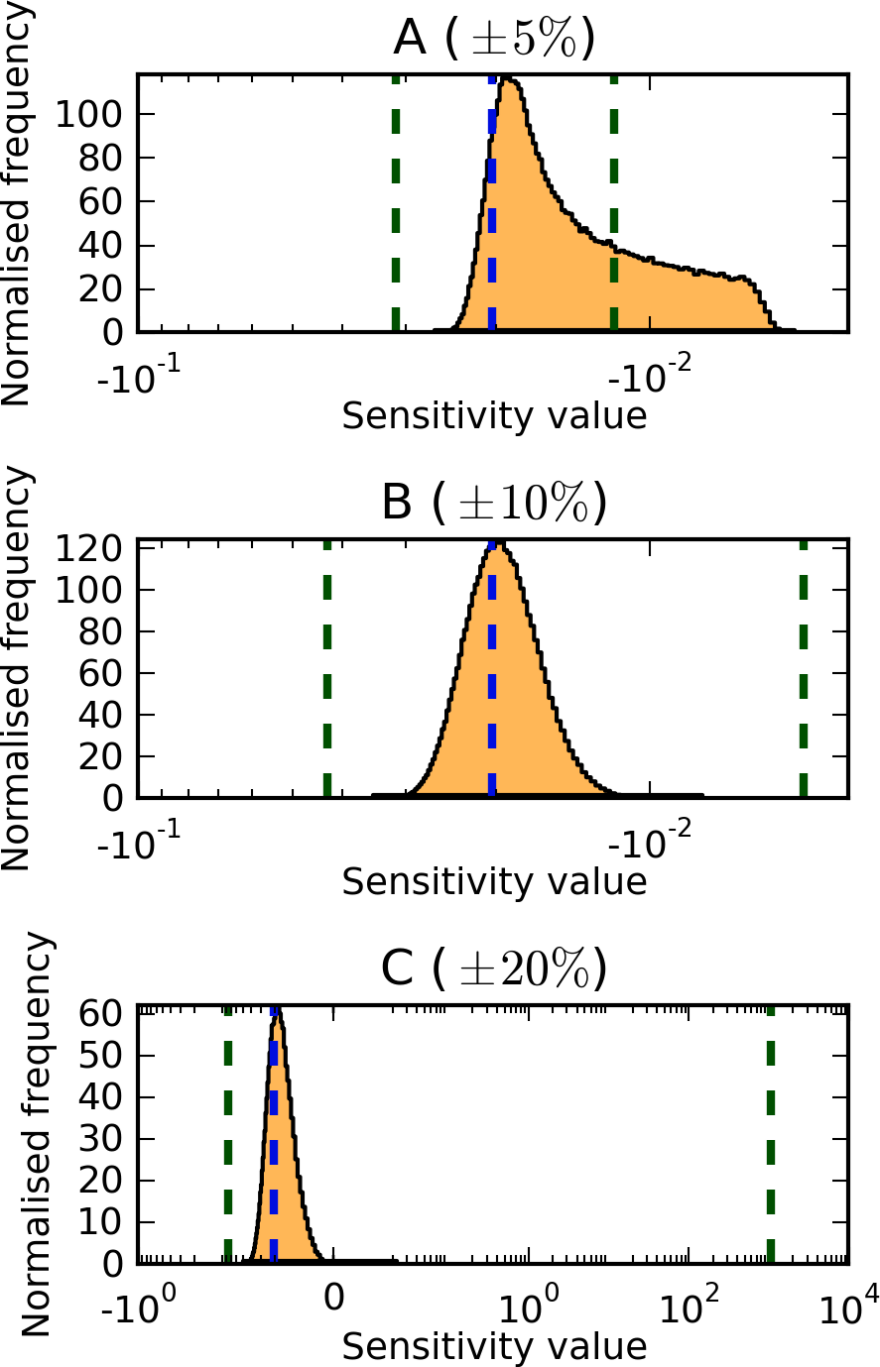
Selected global sensitivity analysis results for the NF
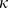
B model [26]. Shown are the results for parameter 2 (*R11 IkBa Nuc Cyto.k1*) in the parameter spaces 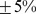 (A), 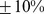 (B) and 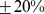 (C) of the reference values. For a description of the distributions refer to the description in Figure 1. Full results for all parameters are shown in Table S2 in File S1.

Global sensitivity analysis was performed using the sampling and optimisation techniques under parameter variations of 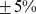
–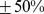 of the original values. The full results are available in Table S2 in File S1, with selected examples shown in Figure 3.

The original parameter set of [26] results in a distribution of control where no single parameter dominates. These results are presented in Table S2 in File S1 and Figure 3, where the values of these ‘local’ sensitivity coefficients are shown as dashed blue lines.

The results of both the sampling-based and optimisation-based analyses show that as the parameter space was widened to a domain that covers 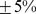 around the original parameter values, the difference in maximum and minimum control for most parameters remained small. Some parameters became able to exert both positive or negative control — this means that depending on the values of the other parameters, these particular ones could either have a positive or negative effect on the period of the oscillations. This phenomenon was verified both using the sampling and optimisation approaches. The distributions obtained by sampling appear to be Gaussian or skewed-Gaussian for many parameters; a high Shapiro-Wilk score confirms this. As can be seen in the examples displayed in Figure 3A, the peaks of the distributions do not necessarily correspond with the local sensitivity values. This means that the physiological state does not correspond to the most commonly found value that could be determined by a global sensitivity analysis (assuming the model [26] does reflect the physiological conditions).

Further exploration of this model's behaviour, by expanding the parameter space to a domain 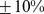 around the original parameter values, shows more parameters able to exert both positive and negative control, yet the degree of control exerted by each one remains relatively small. The distributions obtained by random sampling still appear to be Gaussian, though the peaks of the distributions now correspond to the local sensitivity coefficient values for most parameters.

Allowing parameter variation to be 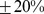 around the reference state, we observe that the oscillation period can now be extremely sensitive to a number of parameters (with sensitivities 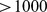), with half of all parameters able to obtain a sensitivity coefficient of magnitude 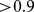 (see Figure 2). More than half of all parameters are now also able to exert both positive and negative control. The distributions of sensitivity values, as shown in Figure 3, remain mostly Gaussian-like (confirmed by high Shapiro-Wilk statistics), though sometimes appear sharp and narrow due to a large difference between the maximum and minimum values as found by the optimisation technique (for example, parameter 20).

Parameter variations of the reference state 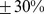 or more result in a situation where oscillatory behaviour is no longer guaranteed, and therefore the sensitivity coefficient is no longer defined. Full results for the NF
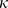
B model are given in Table S2 in File S1.

In all of the above cases, the two methods for global sensitivity produced similar results for parameter variations of 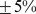 and 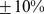, though with parameter variations of 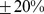, the optimisation method often found much higher-magnitude sensitivity coefficients than the sampling method.

In summary, this model was more robust with regard to the selected parameter sensitivities than the MAPK model described above. However, moderate variations of the parameter values can still drastically change the influence of individual parameters on the model output.

### Cell Cycle Model

**Figure 4:**
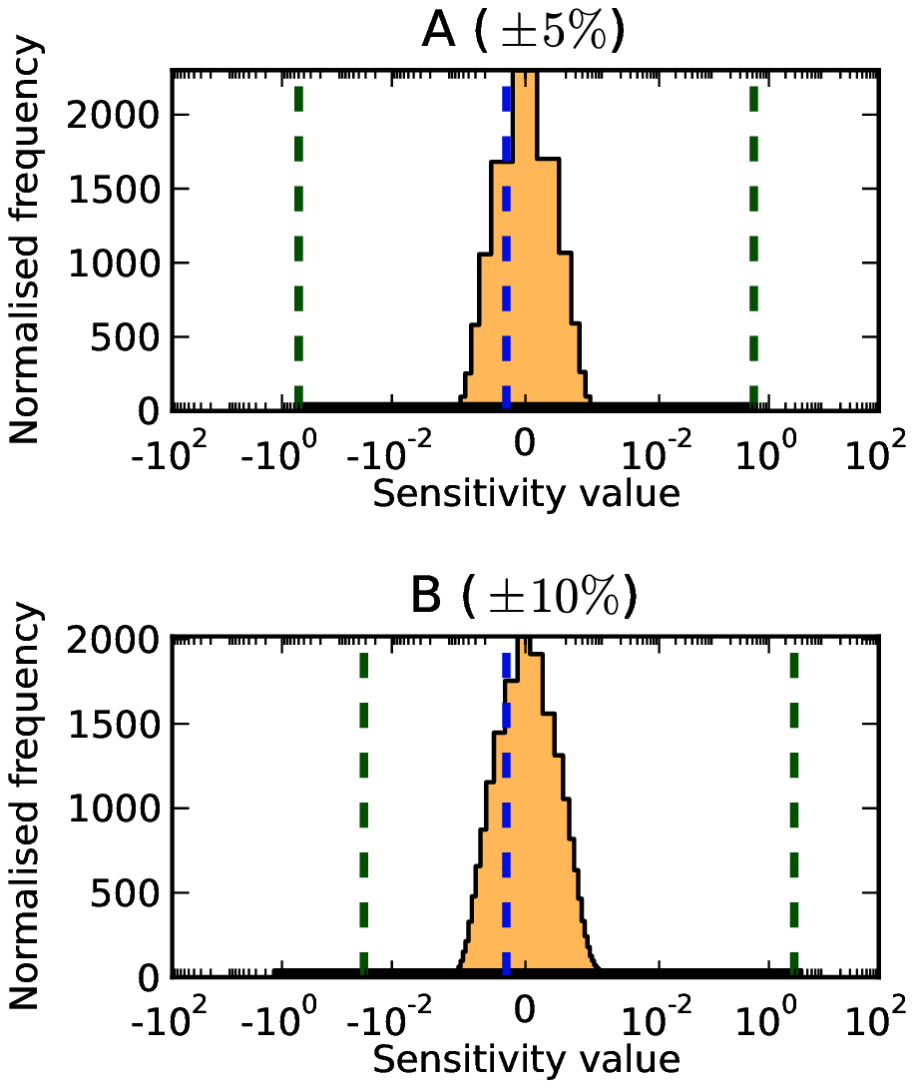
Selected global sensitivity analysis results for the cell cycle model [27]. Shown are the results for parameter 6 (*Values[J20ppx]*) under parameter variations of 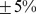 (A) and 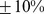 (B) of the reference values. For a description of the distributions refer to the description in Figure 1. Full results for all parameters are shown in Table S1 in File S1.

The model of yeast cell cycle by Chen *et al.* [27] represents the mechanisms of regulation of cell division. Similarly to the previous model this one also oscillates, though in this case this happens through switching in a hysteretic cycle, rather than a stable oscillation (limit cycle). Nevertheless, the period of the resulting oscillation (cell cycle) is one of the most important variables of this system. Thus we studied the sensitivity of the 142 parameters of the model towards the period of the entire cell cycle (*i.e.* time between cell divisions). Global sensitivity analyses using the sampling and optimisation methods were performed under parameter variations of 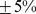 and 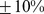. The full results are available in Table S3 in File S1, with selected examples shown in Figure 4.

Local sensitivity analysis of the parameter set published by Chen *et al.* [27] showed the control to be relatively well distributed among most parameters (with the exception of a few parameters that have very low or zero control over the period).

In contrast to the previous model, variation of parameter values within 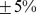 is already enough to change the distribution of control completely. In this range all parameters that have non-zero control in the reference state are now able to cause both positive and negative effects on the cell cycle period, though few are able to exert control with magnitude 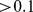. As depicted in Figure 4 and Table S3 in File S1, the distributions of sensitivity values are already sharp for all parameters, with the peaks of the distributions remaining close to the local sensitivity values.

When varying the parameters by 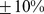 a number of sensitivities reach high-magnitude negative or positive values (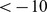 or 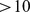), but the distribution peaks remain close to zero. Once more, the distributions are sharp and narrow for all parameters. When the variation reaches 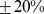 (not shown),the periodicity may be abolished completely, and the sensitivity coefficient is no longer defined.

With this model, when comparing the two global sensitivity methods, we observe that random sampling tends to find higher-magnitude coefficients than optimisation, again this is likely due to lack of convergence.

### Glycolysis models

We examined two different models of glycolysis as examples of metabolic models: one in *Saccharomyces cerevisiae* [28] and another in *Trypanosoma brucei* [29]. They differ in that the latter has a unique compartment, the glycosome, which results in separate pools of ATP between upper and lower glycolysis. Both models, in their original parameter values, exhibit stable steady state behaviour.

Since the two models display very similar behaviour in terms of sensitivity analysis, their results are reported together. For each model we calculated concentration- and flux-control coefficients. These are the same as the sensitivity coefficients of those variables with respect to the limiting rate parameters (*V*, which have a linear effect on the rates of reaction). Due to the particularities of the sensitivity distributions, the parameter variation was investigated in higher resolution than the previous examples. Domains of variation of parameter values were defined around their original parameter values by 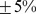, 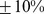, 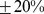, 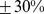, 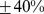, 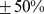, and by 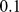
–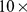. The full results of these analyses are included in Tables S4–S17 in File S2 and in Tables S18–S31 in File S3. Below is a summary of the main points.

**Figure 5:**
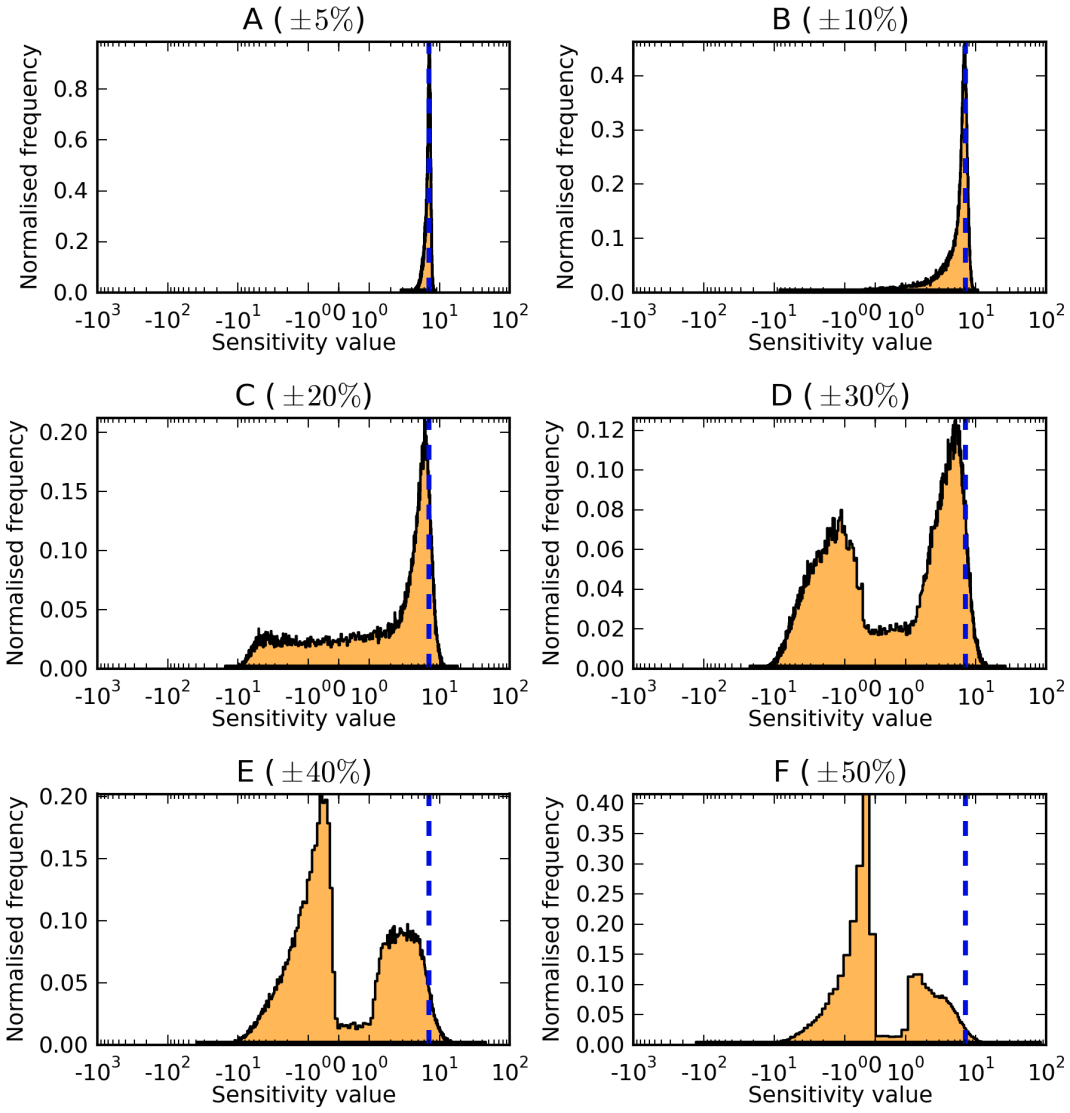
Control on concentration of adpg exerted by the glucose transport reaction in the Trypanosoma brucei model [29]. Panels A–F correspond to parameter variations of 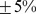 (A), 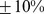 (B), 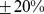 (C), 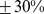 (D), 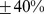 (E), and 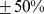 (F) of the reference parameter values. For a full description of the distributions, refer to Figure 1.

**Figure 6:**
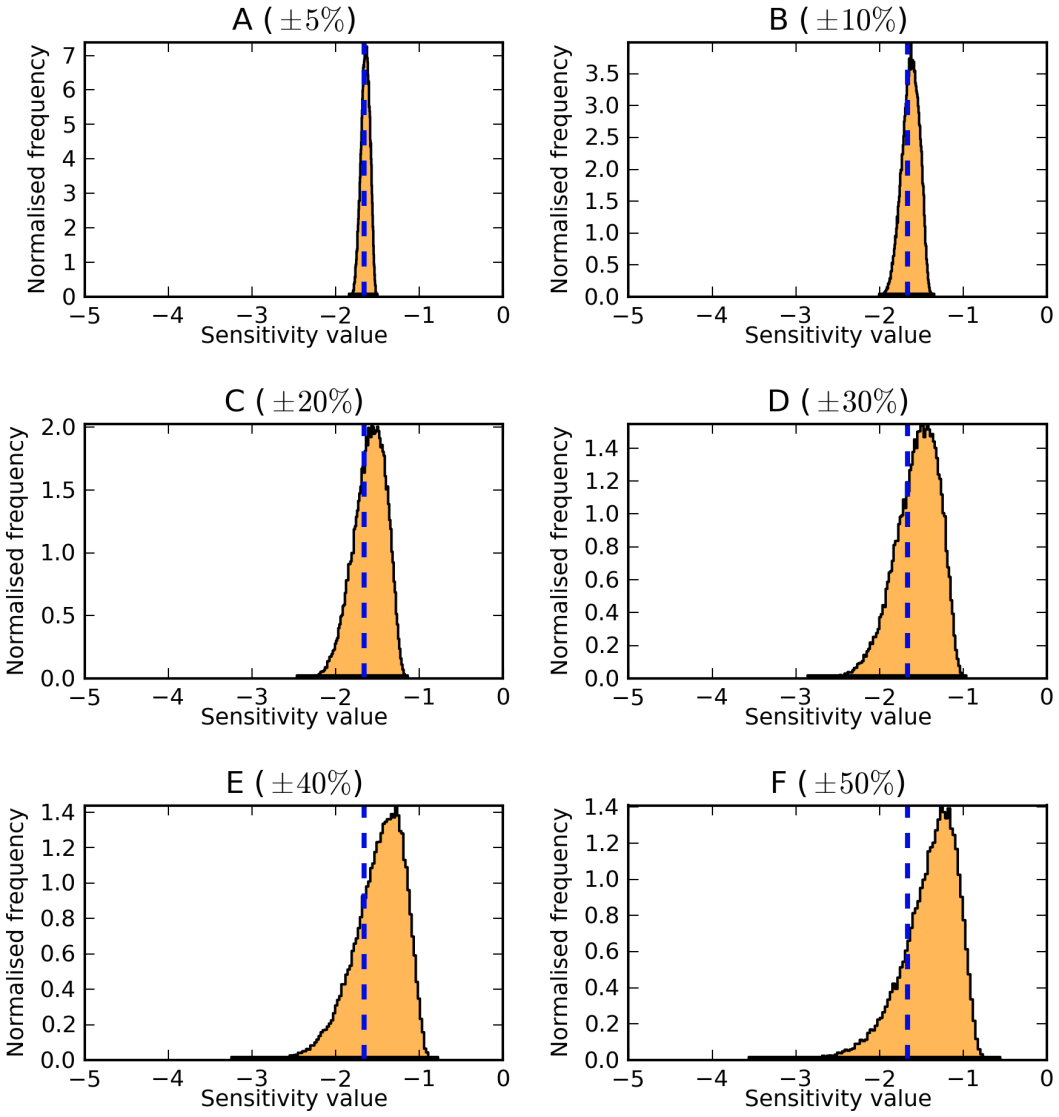
Control on the concentration of Acetaldehyde exerted by the Alcohol dehydrogenase reaction in the *S. cerevisiae* model [28]. Panels A–F correspond to parameter variations of 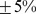 (A), 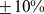 (B), 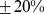 (C), 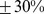 (D), 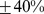 (E), and 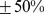 (F) of the reference parameter values. For a full description of the distributions, refer to Figure 1.

In all cases, the shapes of the distributions changed for all sensitivity coefficients as the parameter variation was increased. As the shapes changed, a number of trends were observed. When the parameter variation was confined to 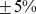, most of the sensitivity distributions have a single peak at, or near, the value of the sensitivity of the reference state (indicated as a dashed blue line in the examples displayed in Figures 5A and 6A). As the parameter variation is allowed to increase, a second peak appears in several distributions. Initially, this second peak is smaller than the peak at the reference value, but as the parameter variation is expanded further, the second peak becomes dominant. A typical example is the concentration control coefficient of adpg (the concentration of ADP in the glycosome) by the glucose transporter in the *Trypanosoma* model (Figure 5). In this case, at 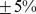 variation of parameter values, the distribution of the values of this control coefficient have a peak at the same value as the reference model [29]. When the parameter variation is increased to 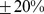, the distribution shifts slightly to the left. With 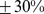 variation, a second, smaller, peak appears around zero. When the parameter variation is expanded to 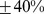, the new peak at zero already has higher frequency than the original peak, and when the variation reaches 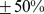, the new peak at zero is clearly dominant. Thus, it appears that, depending on the variability of the parameter values, there are two main types of models: one where glucose transport exerts high control over the concentration of adpg, and another where that metabolic step has no control over that concentration.

Other bimodal distributions of control are also observed, where the values at the peaks of the distributions are finite values (*i.e.* both are different from zero). An example is the concentration control coefficient of glycerol kinase over the concentration of glycerol, also in the *Trypanosoma* model. In this case, with only 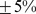 parameter variation, there is a single peak at the local sensitivity value (*circa* 0.65). As the parameter variation is expanded, a new peak appears at around 0.95. This new type of behaviour has a higher control of glycerol concentration by glycerol kinase than in the original model [29].

In other cases no new peaks are observed even at high parameter variation, but the sensitivity values at the peaks of the distributions shift away from their reference values. For example, the concentration-control coefficient of alcohol dehydrogenase on the concentration of acetaldehyde in the *S. cerevisiae* model (Figure 6). As the parameter variation expands, the peak shifts from is original position of 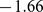 to the right, becoming closer to zero. This means that when all parameters are allowed to vary widely, there will be very few combinations of values that result in alcohol dehydrogenase having high control over acetealdehyde. Therefore this model [28] is positioned on a type of behaviour that would not be easy to generate with parameter values sampled at random — this implies that evolution has pushed the model to a statistically unlikely configuration.

**Figure 7:**
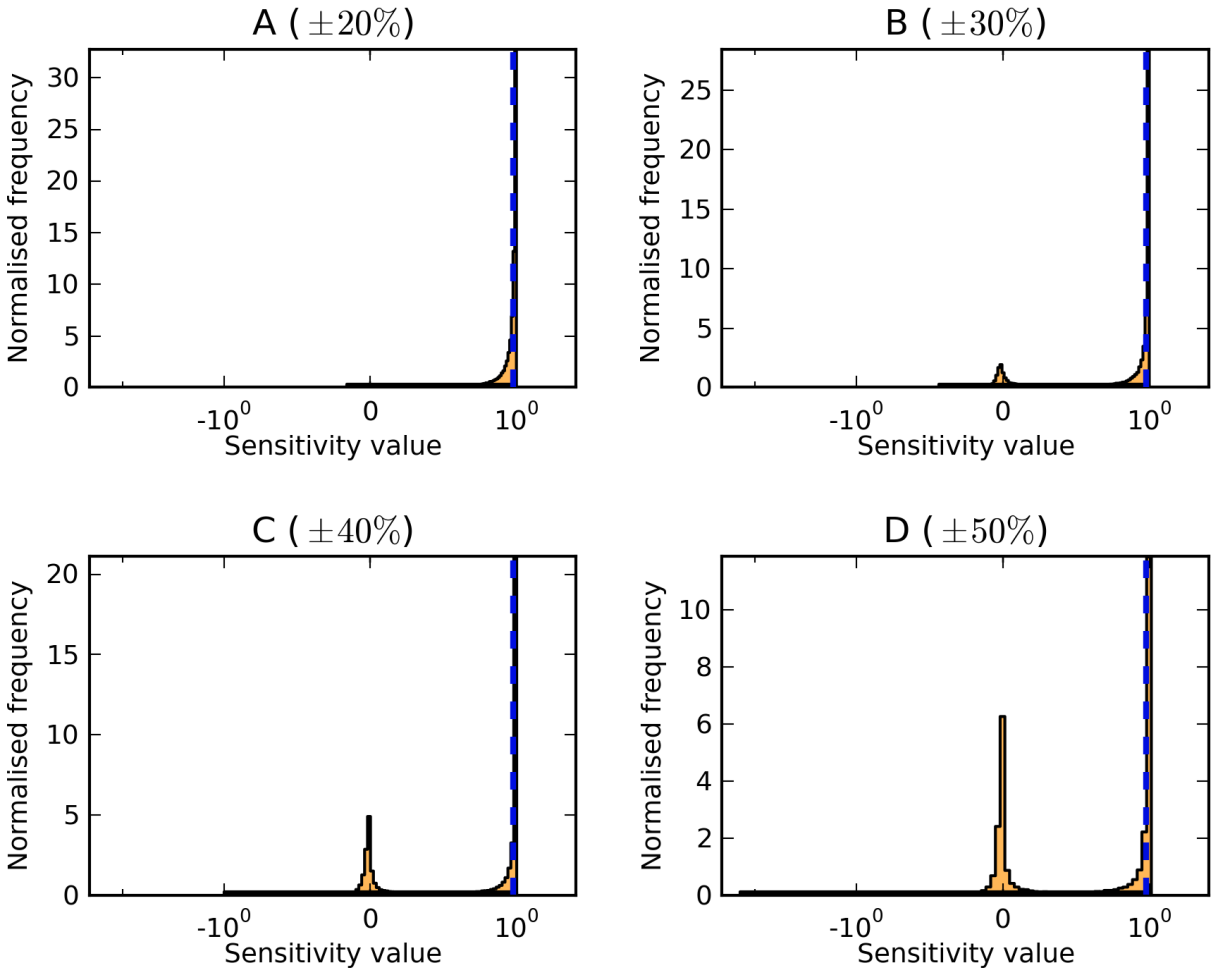
Control of upper glycolytic flux in *Trypanosoma brucei* model [29] exerted by the glucose transport reaction. Panels A–D represent the parameter variations of 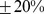 (A), 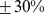 (B), 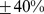 (C), and 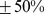 (D) of the reference parameter values. For a full description of the distributions, refer to Figure 1.

Finally, in some cases, the distributions change little as the range of parameter variation is expanded. For example, the concentration control coefficient of glycerol transport over the concentration of glycerol in the *Trypanosoma* model (Figure 7) remains at the same value as in the reference model (0.99) even as the parameter variation is expanded. This means that this particular reaction would exert a high control in most conditions. This is a strong conclusion because it is essentially independent of parameter values.

It should be noted that in the largest parameter value domain — when parameters are allowed to vary between 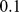
–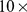 their original value — the distributions all appear to be very narrow, with long flat tails extending to one or both sides. In these cases, the bounds of the distributions are widely distant from the peak of the distribution.

As already mentioned, both models behave in very similar ways, which is likely due to their similar stoichiometric structure (they represent the same pathway). However, there are distinct differences, for example the distribution of control on the flux through the upper part of glycolysis (hexokinase reaction). With the original parameter values, the control of the flux is almost exclusively in the hexokinase reaction in the *Saccharomyces cerevisiae* model, and in the glucose transport reaction in the *Trypanosoma* model. Both models are robust to variations of parameter values of up to 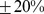, with no other reactions able to exert a high control on the flux in these cases. Parameter variations of between 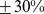 and 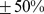 allow a number of other reactions to potentially gain a high control (notably phosphofruktokinase is able to gain control in both models), though the distribution peaks remain very close to the local sensitivity values in all cases, implying that for the distribution of control to change requires an unlikely configuration of parameters.

## Discussion

Global sensitivity analysis of several published models was carried out in order to investigate how informative the values of local sensitivities are in terms of the global behaviour of a model. Global sensitivity analysis through random parameter sampling reveals details of model behaviour in a more general sense. An alternative method for global sensitivity analysis using optimisation was compared with the sampling approach. Several interesting observations arose from this study, which are worthwhile discussing here.

One of the most popular ways to carry out global sensitivity analysis is to sample values of the model parameters from (uniform) random distributions and characterise each sample through local sensitivity analysis. The resulting data are then aggregated in the context of a distribution of values of the sensitivity coefficients and best visualised as histograms of their frequencies. One may be tempted to interpret these results by assuming that the most frequently observed value of the sensitivity would be what the model supports best [15]. The results presented here, however, indicate that such interpretation is not warranted. If the above hypothesis would be true, then the observed values of sensitivity coefficients in biological systems would be close to the peaks of the distributions obtained by parameter sampling. But in the five biological systems investigated here, this was not the case. Are these models good representations of their systems?

The MAPK model was originally defined [25] for an exploratory study and so it did not attempt to match any particular experimental results. As such this model is not relevant to the present argument.

The NF
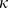
B [26] and the cell cycle models [27] have parameter values with a considerable degree of uncertainty. While the models do indeed mimic a number of properties of their corresponding biological systems, one cannot make definitive statements whether most of their parameter values are close to reality.

But in the case of the two glycolytic models, the majority of their parameter values were determined experimentally with reasonable accuracy. Most of them are enzyme kinetic parameters derived from *in vitro* experiments with purified enzymes. Thus one can reasonably assume that the parameter values in those two models are close to reality. We shall therefore use them to examine the hypothesis that the observed sensitivity values coincide with the mode of the distributions.

When the sampling is carried out in the narrow vicinity of the published parameter values the original sensitivity coefficients are indeed very close to the peaks of the distributions. As the domain of sampling becomes wider, the peaks of most distributions shift away from those reference sensitivity coefficient values. In some cases the distributions even become bimodal, which make the concept of “most probable value” even more tenuous.

Examine the case of the control coefficient for the glucose transport reaction on the concentration of adpg in the *Trypanosoma* glycolysis model [29] (Figure 5). In the published model this has a high control, as its value is 7.1. Sampling parameter values within 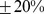 around the original values, the peak of the distribution remains close to the value of the published model. But as the parameter sampling domain expands to 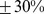, a second peak appears at a low value, indicating that for many parameter combinations the concentration of adpg has a low sensitivity to glucose transport. With a sampling domain of 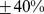, there is again only one peak, but now it is for a smaller sensitivity – 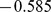. Thus, the most frequent sensitivity that is encountered at random is 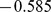 (if parameters are allowed to vary at least 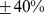), yet the physiological relevant value is 7.1! Similar scenarios can be shown for most parameter sensitivities of the two glycolytic models. Indeed, similar scenarios exist for the other three models, even if we cannot state that their reference values are “physiological”.

The importance of this observation is that one must not interpret the distributions of sensitivity coefficients delineated in global sensitivity analysis as leading to any form of likelihood with biological significance. What do the peaks of these distributions then mean? Simply that if one was to draw parameters *entirely at random* the most likely value of the sensitivity would be that one. But biology does not evolve entirely at random — natural selection guides evolution. If a certain phenotype (spectrum of sensitivity parameters) endows the organism with superior fitness, that phenotype will be selected. The fact that such phenotype is not the most frequent one bears no weight in its survival or otherwise. Even if some phenotype is frequently obtained with random sets of parameter values, there is no reason to believe that it would be particularly successful either. Therefore the peaks of the distributions are not necessarily of biological relevance and one should interpret these distributions with much care. What we do learn from them is the lower and upper bounds for the sensitivities of the respective system with given parameter ranges. This is an argument in favour of the optimisation method, which should be more efficient at finding these extrema, though other important considerations will be also be at play (see the section on method comparison below).

### Robustness

Robustness is a widely-discussed topic in systems biology [30]–[35]. It is usually defined as a property of systems which remains largely unchanged under changes in the environment. This is usually translated into a property (a variable of the models) which remains mostly unchanged in the presence of large environmental perturbations. This concept of robustness is then essentially the opposite of sensitivity as the latter measures how much a variable is affected by a parameter perturbation — thus a quantitative measure of robustness could be the inverse of the corresponding sensitivity coefficient [7], [8].

Note that the concept of robustness applies to specific properties of a system (variables of a model) and not necessarily for the entire system. Indeed some have suggested that there is some form of conservation of robustness; when some variable becomes very robust to perturbations, others become more fragile (sensitive) [30]. This also agrees with the concept that robustness is the inverse of sensitivity; it is well known that there is conservation of sensitivity in biological systems, expressed in the form of summation theorems in metabolic control analysis [4].

Measurements of single sensitivity coefficients in a small area around an operating point are not necessarily very expressive about the true robustness of a model. That can only be achieved with global sensitivity analysis, such as studied here.

As a case in point, Ihekwaba *et al.* [36] studied the robustness of sensitivity coefficients under large parameter perturbations on a similar NF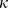 model. Parameters were individually perturbed up to 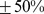. Under this parameter variation, 9 parameters were found to be able to exert control with magnitude greater than 1, with sensitivity coefficient values ranging between −20 and 10. In contrast, our analysis, under a parameter variation of only 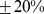 found 15 parameters able to exert a control of magnitude greater than 1, with values ranging between −500 and 1000. This difference is not surprising, since our method allowed for all parameters to vary from their original values simultaneously, whereas the technique used by Ihekwaba *et al.* varied only one parameter at a time.

In a similar study by Achar *et al.* [37], parameters of the *Trypanosoma* glycolysis model [29] were sampled from log-normal distributions, and the impact on the control of flux was measured. Their results match with ours — the situation in the reference model, in which the control on the flux through the main branch is mainly in the glucose transport reaction, is not the only possible scenario. Incorporating a degree of uncertainty surrounding the parameter values results in situations where other reactions can also gain a high degree of control, notably phosphoglycerate mutase and glyceraldehyde-3-phosphate dehydrogenase. One should therefore be cautious in drawing conclusions regarding which reaction exerts the most control on the flux based on a single set of parameter values.

Two observations that were common to all models examined are: 1) if the parameter variation in global sensitivity analysis is small enough the conclusions are the same as for the classical (local) sensitivity analysis; 2) given a sufficiently large domain of variation, all sensitivities will become potentially very large. What differs from model to model is the range of parameter variation at which the model changes from one single pattern of control to several possible patterns. A different pattern of control would result in different responses to variations in e.g. the amount of individual proteins. Thus, whereas initially the system might show insensitivity towards a change in the concentration of a specific protein, moving in parameter space can change to a different pattern of control where the protein under consideration suddenly has a large impact on the systems behavior. We argue that the size of the parameter variation domain where the distribution of control starts showing multiple patterns (such as low-magnitude and high-magnitude, zero and non-zero, positive and negative) is a measure of the overall robustness of the output of the model for which the sensitivities were calculated.

By this measure, the MAPK and cell cycle models were non-robust to changes in parameter values of as little as 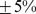, while the NF
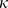
B model was robust to changes up to 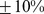. Although sensitivities were calculated against different outputs for each model, each chosen output can be considered the main function of the system. Therefore, comparisons between these models are appropriate.

For the glycolysis models, some model outputs were more robust than others. For example, in the *Trypanosoma* model, the concentration control coefficient for the glucose transport reaction on the concentration of adpg (Figure 5) was robust to variations of 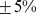, but by 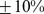 the control could be positive, zero, or negative. The control coefficient for alcohol dehydrogenase on the concentration of acetaldehyde in the *S. cerevisiae* model (Figure 6), however, was robust to parameter variations of up to 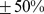.

In general, the sensitivities of the two glycolysis models showed much stronger robustness than the signalling models. These metabolic models differ from the signalling models in that they have a stronger experimentally-determined basis with regards to parameter values and regulation. The increased robustness is therefore likely to be a result of a better knowledge of the kinetic parameters and the regulation (such as feedback loops) of these systems. This implies, of course, that evolution has favoured robustness, which is perhaps not a controversial notion.

This concept of robustness is similar to that proposed by Morohashi *et al.* [35] and Coelho *et al.* [38]; they propose that model robustness can be quantified by the changes in parameter values needed to abruptly change the performance of the system (such as an oscillation period or a concentration). These techniques are limited by the numbers of parameters which can be simultaneously changed — scanning all possible combinations of parameter values within a defined range is possible for a model with few parameters, but infeasible for high-dimensional models. Of course, changing a limited number of parameters is possible in these cases, but a global picture of robustness is not observed. Our approach has the advantage that all model parameters can be varied simultaneously, even in high-dimensional models with many parameters, giving a truly global measure of model robustness.

Robustness and sensitivity analysis are also related in the field of optimal experimental design (OED). OED is a technique used to determine the necessary experiments which should be performed in order to estimate model parameters with the highest possible statistical quality. OED methods are typically based on local sensitivity analysis, and are therefore dependent on the local sensitivities being representative of the physiological system [39]. Robust experimental design aims to optimally design experiments even when there are uncertainties in the initial model parameters, and is therefore closely related to global sensitivity analysis. By identifying which parameters can potentially have a large impact on the model output, global sensitivity analysis can be used in measurement set selection, *i.e.* determining which parameters it is important to have a good experimental measurement for.

Robustness is important at many levels and biological systems do display some remarkable robust properties. For example circadian rhythms do not seem to be affected by temperature changes. Other systems require high sensitivity to a signal. For example the vision system seems to be so sensitive that a single photon can generate a nervous impulse [9]. In both cases the properties are fundamental parts of those systems and arose by selection.

But an entirely different kind of robustness is desirable in most systems: robustness against noise. Small perturbations on the parameters should not destroy the model's control structure completely. We desire systems that are stable enough not to react to small magnitude fluctuations. It is in such cases that the concept of a system's robustness makes sense. Including global sensitivity analysis can help to analyse this important property of models.

It seems critical to repeat the obvious: Sensitivity analysis of models which are heavily underdetermined should be not taken seriously unless a global analysis indicates that the results are robust towards dramatic parameter changes.

### Comparison of methods

Our results show that the performance of the optimisation-based and sampling-based techniques was mixed. In some cases the sampling-based approach found more extreme values for sensitivity coefficients, while in others the optimisation based approach was more successful. This seems to depend on the solution space of the specific models, since there were clearly different trends between the different models — the optimisation technique performed well for the NF
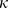
B model, but less well for MAPK and cell cycle models. However, this also indicates that none of the methods provides a guarantee for finding absolute maximal or minimal values, as it is difficult to determine if an optimisation has converged to a global optima or if enough samples have been taken. It is, however, questionable whether the exactness of the bounds is of any practical value at all. Most of the time, the problem finding the absolute maximum appears when searching a large parameter space and the resulting maximal value becomes very high. An extremely large magnitude of a control coefficient means that a tiny perturbation will result in an almost catastrophic change of behaviour, something which is not expected in most physiological cases. As also discussed by Sahle *et al.* [17], these extreme values are often singular values like bifurcation points and it might be of little importance to assess, if there the control is in the magnitude of hundreds or thousands. The general trends in the results of both methods coincided well making both methods applicable for analysing models with respect to global sensitivities.

In cases where parameter sensitivities appear robust to large changes in parameter values, one must consider whether this is a true property of the model, or rather a failure of the analysis technique to detect fragility. Neither a global optimisation or random sampling approach is guaranteed to find global optima, though the likelihood of finding such optima increases with the number of function evaluations.

A major consideration for using the sampling technique is that of sampling density — the volume of the sampling space increases exponentially with the number of parameters; therefore the sampling density in high-dimensional models is much less than in low-dimensional models, especially for larger parameter spaces. To compound this problem, as the number of parameters in a model increases, the computational cost of each sample typically becomes more expensive. There is therefore a danger that the sampling technique will underestimate the robustness of high-dimensional models.

Such a phenomenon was observed, to an degree, in our analyses. For the 142-parameter cell cycle model, 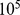 samples were taken; for the equivalent sampling density in the 27-parameter NF
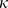
B model, only 9 samples would be needed. Since the cost of each sample in the NF
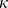
B model was significantly less, we were actually able to take 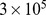 samples. Despite this, both the sampling and optimisation techniques found that the cell cycle model was less robust to small variations in parameter values than the NF
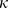
B model, though in smaller parameter spaces, the optimisation technique outperformed the sampling technique (Figure 2).

In theory, the use of a properly tuned optimisation algorithm such as Particle Swarm to search the sensitivity space of a model should be more efficient at finding global optima than a random search. However, the use of such an algorithm reveals only the bounds for parameter sensitivities; the sampling approach offers a richer picture of the distribution of sensitivities, from which substantial information can be obtained, if interpretation is done with care. In addition, the easily-parallelizible nature of the sampling-based technique enabled us to fully exploit our powerful Condor high-throughput computing pool. For the optimisation-based technique, each optimisation can be run in parallel, but unless the number of optimisations approaches the number of computing nodes, the parallel nature of the pool will not be fully exploited. Whether one technique is more suitable than another will depend on aspects such as the dimensionality of the model, and whether a distributed computing platform is available for use.

### Conclusions

Global sensitivity analysis is a methodology that allows the study of how perturbations in each parameter affect the properties of a model. In contrast to the more common local sensitivity analysis, the global version allows for large changes in parameter values. The results of these investigations reveal which parameters are sensitive and therefore should be determined with precision. If the real value of a parameter with high sensitivity is estimated poorly in a model then this could have strong consequences on the behaviour of the model, whereas if the parameter had a low sensitivity, determining an accurate value would be less important. By increasing the size of the parameter variation, we are able to see a model go from a single pattern of control (that of the reference system) to many potential patterns (at different nominal values of parameters, but all within the range of variation). We argue that the amount of variation at which the pattern of control becomes uncertain is a measure of robustness of one or more outputs of the entire system.

Systems for which multiple patterns of control appear at small variations of parameter values are very sensitive systems, while those for which this happens only at large variations can be considered robust. Up until now the concept of robustness was appropriate for each parameter in isolation. Our study suggests that global sensitivity analysis can be used as a measure of robustness for the system as a whole.

## Materials and Methods

Each model was imported into COPASI [14] (version 4.7, build 34) from an SBML [40] file, obtained from the BioModels database [41]. All analyses were then carried out with this software.

### Optimisation-based analyses

The optimisation-based sensitivity analyses were performed using the COPASI optimisation task. For a given range of parameter variation, two optimisations were prepared for each sensitivity coefficient — one to maximise and one to minimise it. The sensitivity task was configured to calculate parameter sensitivities against an appropriate systems-level property. For the MAPK model the steady-state concentration of PP-MAPK was used. For the NF
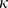
B model, the period of oscillation of nuclear NF
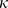
B was used; the period of oscillation was calculated using the COPASI events system, after simulating the model for a time-course of 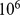 seconds. Briefly, an event is introduced that is triggered at a maximum of one of the variables and time is assigned to a variable, then in the next time the maximum is detected, the current value of time is subtracted with the previous one, thus resulting in the period of the oscillation. For the cell cycle model, the period of cell division was used; the period was already explicitly defined [27] and was calculated after simulating the model for a time-course of 1000 minutes.

Sensitivities were calculated by perturbing each parameter by 0.1% using the COPASI sensitivities task, and then and measuring the effect on the target property. In all cases, sensitivity coefficients were scaled.

For the optimisation approach the Particle Swarm optimisation algorithm [18] was used. Different algorithm settings were applied for each model, due to differences in the time taken to compute parameter sensitivities (Table 1).

**Table 1:** Optimisation algorithm settings.

Model	Iteration limit	Swarm size
MAPK	2000	50
NF 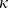 B	500	20
Cell cycle	100	10

Settings used for the Particle Swarm optimisation algorithm for each model in the sampling-based global sensitivity analysis. Different algorithm settings were used for each model due to differences in the time taken to compute parameter sensitivities for each model.

The Condor-COPASI package [42] was used to automate the process of defining optimisations for each sensitivity coefficient, and to execute the optimisations in parallel on our Condor pool (which has approximately 3500 CPUs).

### Sampling-based analyses

The sampling-based analyses were prepared using the COPASI parameter scan task. Parameters were sampled from uniform random distributions. 1,000,000 samples were taken for the MAPK model, 300,000 samples taken for NF
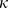
B, 100,000 for the cell cycle model, the *Trypanosoma* glycolysis model and the *Saccharomyces cerevisiae* glycolysis model (summarised in Table 2). For the MAPK, NF
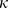
B and cell cycle models, sensitivities were calculated in the same way as the optimisation-based analyses. For the glycolysis models, control coefficients were calculated using the COPASI ‘Metabolic Control Analysis’ task.

**Table 2:** Monte Carlo sampling settings.

Model	Number of samples taken
MAPK	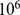
NF 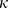 B	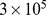
Cell cycle	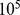
*Trypanosoma brucei* glycolysis	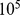
*Saccharomyces cerevisiae* glycolysis	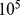

The number of random samples taken for each model for the sampling-based global sensitivity analysis.

Condor-COPASI [42] was used to split the sampling tasks for the MAPK, NF
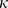
B and cell cycle models into multiple parallel jobs, each of which was run in parallel on our Condor pool. The sampling tasks for the glycolysis models were manually split into smaller parts and run on a distributed computing cluster using the COPASI command-line mode.

A Python script [43] was written to process the sampled sensitivity coefficients, and the MatPlotLib graphics plotting library [44] was used to produce the histograms.

## Supporting Information

File S1 **Tables S1—S3.** The full outputs of the global sensitivity analyses on the MAPK, NFB and Cell Cycle models using both random-sampling and optimisation-based approaches.
(PDF)

File S2 **Tables S4—S17.** The full outputs of the global sensitivity analyses on the *Trypanosoma brucei* model using the random-sampling approach.
(PDF)

File S3 **Tables S18—S31.** The full outputs of the global sensitivity analyses on the *Saccharomyces cerevisiae* model using the random-sampling approach.
(PDF)

## References

[1] Hubner K, Sahle S, Kummer U (2011) Applications and trends in systems biology in biochemistry. FEBS Journal 278: 2767–857. 21707921 10.1111/j.1742-4658.2011.08217.x

[2] Jacquez JA, Perry T (1990) Parameter estimation: local identifiability of parameters. American Journal of Physiology- Endocrinology and Metabolism 258: 727–736. 10.1152/ajpendo.1990.258.4.E727 2333964

[3] Marino S, Hogue IB, Ray CJ, Kirschner DE (2008) A methodology for performing global uncertainty and sensitivity analysis in systems biology. Journal of theoretical biology 254: 178–96. 18572196 10.1016/j.jtbi.2008.04.011 PMC2570191

[4] Kacser H, Burns JA (1973) The control of ux. Symposia of the Society for Experimental Biology 27: 65–104. 4148886

[5] Heinrich R, Rapoport TA (1974) A linear steady-state treatment of enzymatic chains. General properties, control and effector strength. European Journal of Biochemistry 42: 89–95. 4830198 10.1111/j.1432-1033.1974.tb03318.x

[6] Burns JA, Cornish-Bowden A, Groen AK, R H, Kacser H, et al (1985) Control of metabolic systems. Trends in Biochemical Science 10: 16.

[7] Quinton-Tulloch MJ (2011) Fragile robustness: principles and practice. Ph.D. thesis, University of Manchester.

[8] Westerhoff HV (2007) Multi-factorial disease and robustness: Where Systems Biology makes a difference. FEBS Journal 274: 342.

[9] Stryer L (1996) Vision: From photon to perception. Proceedings of the National Academy of Sciences USA 93: 557–559. 10.1073/pnas.93.2.557 PMC40090 9254392

[10] Saltelli A, Ratto M, Andres T, Campolongo F, Cariboni J, et al.. (2007) Global Sensitivity Analysis. The Primer. Chichester, UK: John Wiley & Sons, Ltd.

[11] Kucherenko S, Rodriguez-Fernandez M, Pantelides C, Shah N (2009) Monte Carlo evaluation of derivative-based global sensitivity measures. Reliability Engineering & System Safety 94: 1135–1148.

[12] Rodriguez-Fernandez M, Banga JR, Doyle FJ (2012) Novel global sensitivity analysis methodology accounting for the crucial role of the distribution of input parameters: application to systems biology models. International Journal of Robust and Nonlinear Control 22: 1082–1102.

[13] Murabito E, Smallbone K, Swinton J, Westerhoff HV, Steuer R (2011) A probabilistic approach to identify putative drug targets in biochemical networks. Journal of the Royal Society Interface 8: 880–95. 10.1098/rsif.2010.0540 PMC3104352 21123256

[14] Hoops S, Sahle S, Gauges R, Lee C, Pahle J, et al (2006) COPASI–a COmplex PAthway SImulator. Bioinformatics 22: 3067–74. 17032683 10.1093/bioinformatics/btl485

[15] Steuer R, Gross T, Selbig J, Blasius B (2006) Structural kinetic modeling of metabolic networks. Proceedings of the National Academy of Sciences USA 103: 11868–11873. 10.1073/pnas.0600013103 PMC1524928 16880395

[16] Hu Z, Shi P (2010) Sensitivity analysis for biomedical models. IEEE Transactions on Medical Imaging 29: 1870–81. 20562035 10.1109/TMI.2010.2053044

[17] Sahle S, Mendes P, Hoops S, Kummer U (2008) A new strategy for assessing sensitivities in biochemical models. Philosophical Transactions of the Royal Society Series A 366: 3619–31. 18632455 10.1098/rsta.2008.0108 PMC3268210

[18] Kennedy J, Eberhart R (1995) Particle swarm optimization. In: Proceedings of IEEE International Conference on Neural Networks. Perth, WA, Australia: IEEE, volume 4: , pp. 1942–1948.

[19] Sobol I (2001) Global sensitivity indices for nonlinear mathematical models and their Monte Carlo estimates. Mathematics and Computers in Simulation 55: 271–280.

[20] Morris M (1991) Factorial sampling plans for preliminary computational experiments. Technometrics 33: 161–174.

[21] Saltelli A, Tarantola S, Chan K (1999) A quantitative model-independent method for global sensitivity analysis of model output. Technometrics 37–41.

[22] Zheng Y, Rundell A (2006) Comparative study of parameter sensitivity analyses of the TCRactivated Erk-MAPK signalling pathway. IEE Proceedings Systems Biology 153: 201–211. 16986622 10.1049/ip-syb:20050088

[23] Bagheri N, Stelling J, Doyle FJ (2007) Quantitative performance metrics for robustness in circadian rhythms. Bioinformatics 23: 358–64. 17158515 10.1093/bioinformatics/btl627

[24] Kiparissides A, Kucherenko SS, Mantalaris A, Pistikopoulos EN (2009) Global sensitivity analysis challenges in biological systems modeling. Industrial & Engineering Chemistry Research 48: 7168–7180.

[25] Huang CY, Ferrell JE (1996) Ultrasensitivity in the mitogen-activated protein kinase cascade. Proceedings of the National Academy of Sciences USA 93: 10078–83. 10.1073/pnas.93.19.10078 PMC38339 8816754

[26] Ashall L, Horton CA, Nelson DE, Paszek P, Harper CV, et al (2009) Pulsatile stimulation determines timing and specificity of NF-*κ*B-dependent transcription. Science 324: 242–6. 19359585 10.1126/science.1164860 PMC2785900

[27] Chen KC, Calzone L, Csikasz-Nagy A, Cross FR, Novak B, et al (2004) Integrative analysis of cell cycle control in budding yeast. Molecular Biology of the Cell 15: 3841–62. 15169868 10.1091/mbc.E03-11-0794 PMC491841

[28] Hynne F, Danø S, Sørensen PG (2001) Full-scale model of glycolysis in *Saccharomyces cerevisiae* . Biophysical Chemistry 94: 121–63. 11744196 10.1016/s0301-4622(01)00229-0

[29] Albert MA, Haanstra JR, Hannaert V, Van Roy J, Opperdoes FR, et al (2005) Experimental and in silico analyses of glycolytic flux control in bloodstream form *Trypanosoma brucei* . Journal of Biological Chemistry 280: 28306–15. 15955817 10.1074/jbc.M502403200

[30] Zhou K, Doyle J (1998) Essentials of robust control. Prentice Hall.

[31] Kitano H, Oda K, Kimura T, Matsuoka Y, Csete M, et al (2004) Metabolic Syndrome and Robustness Tradeoffs. Diabetes 53: S6–S15. 15561923 10.2337/diabetes.53.suppl_3.s6

[32] Kitano H (2007) Towards a theory of biological robustness. Molecular Systems Biology 3: 137. 17882156 10.1038/msb4100179 PMC2013924

[33] Stelling J, Sauer U, Szallasi Z, Doyle FJ, Doyle J (2004) Robustness of cellular functions. Cell 118: 675–85. 15369668 10.1016/j.cell.2004.09.008

[34] Jacobsen E, Cedersund G (2008) Structural robustness of biochemical network models—with application to the oscillatory metabolism of activated neutrophils. IET Systems Biology 2: 39–47. 18248085 10.1049/iet-syb:20070008

[35] Morohashi M, Winn AE, Borisuk MT, Bolouri H, Doyle J, et al (2002) Robustness as a measure of plausibility in models of biochemical networks. Journal of Theoretical Biology 216: 19–30. 12076125 10.1006/jtbi.2002.2537

[36] Ihekwaba A, Broomhead D, Grimley R, Benson N, Kell D (2004) Sensitivity analysis of parameters controlling oscillatory signalling in the NF-κB pathway: the roles of IKK and IkBa. IET Systems Biology 93–103. 10.1049/sb:20045009 17052119

[37] Achcar F, Kerkhoven EJ, Bakker BM, Barrett MP, Breitling R (2012) Dynamic modelling under uncertainty: the case of *Trypanosoma brucei* energy metabolism. PLoS Computational Biology 8: e1002352. 22379410 10.1371/journal.pcbi.1002352 PMC3269904

[38] Coelho PMBM, Salvador A, Savageau MA (2009) Quantifying global tolerance of biochemical systems: design implications for moiety-transfer cycles. PLoS computational biology 5: e1000319. 19300483 10.1371/journal.pcbi.1000319 PMC2650413

[39] Yue H, Brown M, He F, Jia J, Kell DB (2008) Sensitivity analysis and robust experimental design of a signal transduction pathway system. International Journal of Chemical Kinetics 40: 730–741.

[40] Hucka M, Finney a, Sauro HM, Bolouri H, Doyle JC, et al (2003) The systems biology markup language (SBML): a medium for representation and exchange of biochemical network models. Bioinformatics 19: 524–531. 12611808 10.1093/bioinformatics/btg015

[41] Le Novère N, Bornstein B, Broicher A, Courtot M, Donizelli M, et al (2006) BioModels Database: a free, centralized database of curated, published, quantitative kinetic models of biochemical and cellular systems. Nucleic Acids Research 34: D689–91. 16381960 10.1093/nar/gkj092 PMC1347454

[42] Kent E, Hoops S, Mendes P (2012) Condor-COPASI: high-throughput computing for biochemical networks. BMC Systems Biology 6: 91. 22834945 10.1186/1752-0509-6-91 PMC3527284

[43] Python programming language official website. URL http://python.org/. Accessed 2013 Oct 1.

[44] Matplotlib: Python plotting website. URL http://matplotlib.sourceforge.net/. Accessed 2013 Oct 1.

