# Supplementary material for: What Can We Learn from Global Sensitivity Analysis of Biochemical Systems?
Source: PLoS One. 2013 Nov 14;8(11):e79244. doi: 10.1371/journal.pone.0079244 (PMC3828278; doi:10.1371/journal.pone.0079244)
Supplement: File S1 — Tables S1—S3. The full outputs of the global sensitivity analyses on the MAPK, NFB and Cell Cycle models using both random-sampling and optimisation-based approaches. (PDF) [file pone.0079244.s001.pdf]

Supplementary information (Tables S1 – S3)

Below we present the full outputs of the global sensitivity analyses on the MAPK, NFκB and Cell Cycle models using both random-sampling and optimisation-based approaches. The results of random sampling are presented as distributions for each sensitivity – distribution x axes show the sensitivity coefficient values, and the y axes show the frequency these values occurred during sampling. The initial sensitivity value is shown as a dashed blue line, zero is shown as a dotted purple line. The bounds found by the optimisation technique, where calculated, are shown on a second distribution as dashed green lines.

Table S1: Table showing distributions for the sampling-based global sensitivity analysis for the MAPK model. Local sensitivity values are shown as a dashed blue line, the best values found by the optimization-based technique are shown as dashed green lines, and 0 is shown as a dotted purple line.

ID	Name	±5%	±5% (with opt)	±10%	±10% (with opt)	±30%	±30% (with opt)
1	(MAPKKK activation).k1<br>Local<br>Min<br>Max<br>Normalized peak height<br>Peak sensitivity value<br>Shapiro-Wilk score	5.17E-03<br>-4.21E+01<br>2.00E+01<br>1.00E+00<br>-2.33E-03<br>3.846E-04	2.37E-03<br>1.26E-02	5.17E-03<br>-4.73E+03<br>2.26E+02<br>9.96E-01<br>7.47E-01<br>8.250E-03	-5.33E+02<br>3.66E-02	5.17E-03<br>-9.56E+03<br>5.69E+04<br>9.68E-01<br>-2.65E+01<br>6.026E-02	-1.64E+07<br>3.14E+05
2	(MAPKKK inactivation).k1<br>Local<br>Min<br>Max<br>Normalized peak height<br>Peak sensitivity value<br>Shapiro-Wilk score	-5.14E-03<br>-6.17E+01<br>1.99E+01<br>1.00E+00<br>-2.28E-02<br>2.345E-04	-1.13E-02<br>-2.60E-03	-5.14E-03<br>-4.48E+03<br>2.49E+02<br>9.97E-01<br>4.05E-01<br>1.294E-02	-5.35E+02<br>1.17E+03	-5.14E-03<br>-3.54E+04<br>6.11E+04<br>9.71E-01<br>-3.14E+01<br>4.865E-02	-2.54E+04<br>3.07E+06
3	(binding MAPK and PP-MAPKK).k1<br>Local<br>Min<br>Max<br>Normalized peak height<br>Peak sensitivity value<br>Shapiro-Wilk score	1.04E-03<br>-1.57E+01<br>1.99E+01<br>1.00E+00<br>1.28E-02<br>1.148E-04	5.71E-04<br>2.12E-03	1.04E-03<br>-4.57E+03<br>2.43E+02<br>9.96E-01<br>-2.86E-01<br>1.065E-02	-4.06E+03<br>1.25E+03	1.04E-03<br>-3.54E+04<br>6.81E+04<br>9.67E-01<br>1.97E+01<br>5.574E-02	-1.15E+06<br>3.61E+06
4	(binding MAPK and PP-MAPKK).k2<br>Local<br>Min<br>Max<br>Normalized peak height<br>Peak sensitivity value<br>Shapiro-Wilk score	-5.23E-04<br>-6.19E+01<br>-3.49E-04<br>1.00E+00<br>-3.13E-02<br>4.667E-05	-1.12E-03<br>-2.72E-04	-5.23E-04<br>-4.23E+03<br>2.49E+02<br>9.97E-01<br>5.76E-03<br>1.116E-02	-5.38E+02<br>9.27E+02	-5.23E-04<br>-3.54E+04<br>5.30E+04<br>9.71E-01<br>1.09E+00<br>5.223E-02	-3.23E+06<br>9.23E+05
5	(binding MAPK-Pase and P-MAPK).k1<br>Local<br>Min<br>Max<br>Normalized peak height<br>Peak sensitivity value<br>Shapiro-Wilk score	-1.54E-03<br>-5.66E-03<br>1.99E+01<br>1.00E+00<br>4.31E-03<br>1.795E-04	-2.90E-03<br>-7.41E-04	-1.54E-03<br>-4.23E+03<br>2.49E+02<br>9.98E-01<br>-7.18E-02<br>4.019E-03	-5.40E+02<br>3.48E+03	-1.54E-03<br>-3.54E+04<br>6.76E+04<br>9.83E-01<br>-2.98E+01<br>3.215E-02	-6.07E+04<br>2.95E+05
6	(binding MAPK-Pase and P-MAPK).k2<br>Local<br>Min<br>Max<br>Normalized peak height<br>Peak sensitivity value<br>Shapiro-Wilk score	7.71E-04<br>-7.71E+01<br>2.87E-03<br>1.00E+00<br>-3.57E-02<br>3.707E-05	3.88E-04<br>1.39E-03	7.71E-04<br>-4.23E+03<br>3.23E+02<br>9.98E-01<br>1.56E+00<br>7.020E-03	-7.99E+02<br>1.08E+03	7.71E-04<br>-1.08E+04<br>6.81E+04<br>9.76E-01<br>-2.08E+01<br>3.971E-02	-1.97E+07<br>3.61E+06
7	(binding MAPK-Pase and PP-MAPK).k1<br>Local<br>Min<br>Max<br>Normalized peak height<br>Peak sensitivity value<br>Shapiro-Wilk score	-4.81E-02<br>-5.83E-02<br>-3.66E-02<br>4.60E-03<br>-4.79E-02<br>9.947E-01	-5.67E-02<br>-4.15E-02	-4.81E-02<br>-4.31E+03<br>3.82E+03<br>9.99E-01<br>2.07E+00<br>1.892E-03	-5.36E+02<br>8.69E+02	-4.81E-02<br>-3.54E+04<br>5.73E+04<br>9.87E-01<br>3.32E+01<br>2.608E-02	-9.72E+06<br>3.62E+06
8	(binding MAPK-Pase and PP-MAPK).k2<br>Local<br>Min<br>Max<br>Normalized peak height<br>Peak sensitivity value<br>Shapiro-Wilk score	2.40E-02<br>1.86E-02<br>5.54E+01<br>1.00E+00<br>4.63E-02<br>3.989E-04	2.08E-02<br>2.84E-02	2.40E-02<br>-4.23E+03<br>3.23E+02<br>9.99E-01<br>1.42E+00<br>4.800E-03	1.81E-02<br>1.21E+03	2.40E-02<br>-3.54E+04<br>6.78E+04<br>9.82E-01<br>2.33E+01<br>3.113E-02	-1.28E+05<br>8.36E+05

9	(binding MAPKK-Pase and P-MAPKK).k1	Local	-4.75E-04		-4.75E-04		-4.75E-04	
		Min	-7.73E+01	-1.62E-03	-4.86E+03	-5.39E+02	-3.54E+04	-9.15E+05
		Max	7.17E-02	2.18E+02	3.23E+02	6.82E+03	5.31E+04	3.68E+06
		Normalized peak height	1.00E+00		9.96E-01		9.69E-01	
		Peak sensitivity value	3.30E-02		-1.46E+00		2.89E+01	
		Shapiro-Wilk score	1.034E-04		1.303E-02		5.703E-02	
10	(binding MAPKK-Pase and P-MAPKK).k2	Local	2.37E-04		2.37E-04		2.37E-04	
		Min	-3.68E-02	-1.82E+02	-4.23E+03	-5.39E+02	-9.57E+03	-1.62E+07
		Max	2.55E-01	7.91E-04	3.23E+02	1.18E+03	6.76E+04	3.63E+06
		Normalized peak height	5.26E-01		9.97E-01		9.70E-01	
		Peak sensitivity value	3.78E-04		1.52E+00		3.62E+01	
		Shapiro-Wilk score	1.420E-02		9.742E-03		5.090E-02	
11	(binding MAPKK-Pase and PP-MAPKK).k1	Local	-2.25E-03		-2.25E-03		-2.25E-03	
		Min	-6.22E+01	-5.08E-03	-4.57E+03	-5.40E+02	-3.54E+04	-1.42E+07
		Max	1.12E-01	-1.00E-03	1.29E+03	-4.40E-04	6.75E+04	1.19E+06
		Normalized peak height	1.00E+00		9.96E-01		9.66E-01	
		Peak sensitivity value	1.82E-02		1.00E+00		4.96E+01	
		Shapiro-Wilk score	1.329E-04		1.065E-02		5.296E-02	
12	(binding MAPKK-Pase and PP-MAPKK).k2	Local	1.12E-03		1.12E-03		1.12E-03	
		Min	-1.58E+01	5.01E-04	-4.85E+03	2.19E-04	-3.54E+04	-9.88E+02
		Max	2.47E-01	2.54E-03	2.83E+02	1.09E+03	4.50E+04	6.84E+06
		Normalized peak height	1.00E+00		9.97E-01		9.71E-01	
		Peak sensitivity value	-1.81E-03		-1.69E+00		-1.20E+00	
		Shapiro-Wilk score	1.864E-04		5.833E-03		5.703E-02	
13	(binding P-MAPKKK and MAPKK).k1	Local	2.28E-03		2.28E-03		2.28E-03	
		Min	-4.60E+01	7.97E-04	-3.64E+03	-5.28E+02	-1.58E+04	-9.56E+05
		Max	5.16E-01	6.93E-03	3.23E+02	2.39E-02	6.11E+04	5.44E+05
		Normalized peak height	1.00E+00		9.96E-01		9.68E-01	
		Peak sensitivity value	-1.90E-02		-2.04E-01		2.61E+01	
		Shapiro-Wilk score	2.031E-04		1.736E-02		5.387E-02	
14	(binding P-MAPKKK and MAPKK).k2	Local	-1.14E-03		-1.14E-03		-1.14E-03	
		Min	-1.59E+01	-3.64E-03	-1.42E+03	-4.88E+02	-1.58E+04	-5.88E+03
		Max	9.87E-01	-3.79E-04	3.23E+02	6.75E+03	6.76E+04	3.62E+06
		Normalized peak height	1.00E+00		9.96E-01		9.71E-01	
		Peak sensitivity value	-2.21E-03		8.68E-01		1.04E+01	
		Shapiro-Wilk score	2.940E-04		2.062E-02		5.049E-02	
15	(binding P-MAPKKK and P-MAPKK).k1	Local	8.99E-03		8.99E-03		8.99E-03	
		Min	5.70E-03	4.65E-03	-4.23E+03	-5.39E+02	-7.32E+03	-5.34E+03
		Max	2.00E+01	1.89E-02	2.40E+02	1.17E+03	6.79E+04	4.27E+05
		Normalized peak height	1.00E+00		9.96E-01		9.77E-01	
		Peak sensitivity value	1.57E-02		1.02E+00		1.02E+01	
		Shapiro-Wilk score	2.388E-03		1.170E-02		4.070E-02	
16	(binding P-MAPKKK and P-MAPKK).k2	Local	-4.50E-03		-4.50E-03		-4.50E-03	
		Min	-6.23E+01	-9.90E-03	-4.23E+03	-5.40E+02	-3.54E+04	-4.99E+06
		Max	2.12E-01	-2.21E-03	3.23E+02	-1.14E-03	6.11E+04	3.63E+06
		Normalized peak height	1.00E+00		9.96E-01		9.71E-01	
		Peak sensitivity value	-6.83E-03		1.57E+00		-3.68E+01	
		Shapiro-Wilk score	1.888E-04		1.135E-02		4.857E-02	
17	(binding PP-MAPKK and P-MAPK).k1	Local	3.31E-02		3.31E-02		3.31E-02	
		Min	2.70E-02	2.53E-02	-4.86E+03	1.99E-02	-3.54E+04	-1.75E+06
		Max	2.00E+01	4.60E-02	2.40E+02	1.22E+03	6.77E+04	2.48E+05
		Normalized peak height	1.00E+00		9.99E-01		9.87E-01	
		Peak sensitivity value	3.70E-02		-1.87E+00		1.23E+01	
		Shapiro-Wilk score	7.489E-03		2.204E-03		2.269E-02	
18	(binding PP-MAPKK and P-MAPK).k2	Local	-1.65E-02		-1.65E-02		-1.65E-02	
		Min	-5.85E-02	-2.42E-02	-4.23E+03	-5.39E+02	-3.54E+04	-1.41E+07
		Max	-1.32E-02	-1.20E-02	4.63E+03	-8.95E-03	5.70E+04	3.63E+06
		Normalized peak height	1.76E-02		9.98E-01		9.78E-01	
		Peak sensitivity value	-1.64E-02		-1.12E+00		3.61E+01	
		Shapiro-Wilk score	9.929E-01		3.650E-03		4.280E-02	
19	(binding of MAPKKK activator).k1	Local	1.02E-02		1.02E-02		1.02E-02	
		Min	-6.71E+01	4.89E-03	-2.94E+03	-5.40E+02	-3.54E+04	-1.17E+06
		Max	1.05E-01	2.35E-02	2.56E+02	1.17E+03	5.69E+04	5.29E+05
		Normalized peak height	1.00E+00		9.85E-01		9.66E-01	
		Peak sensitivity value	4.11E-03		1.44E+00		-2.42E+01	
		Shapiro-Wilk score	4.421E-04		2.747E-02		5.976E-02	

20	(binding of MAPKKK activator).k2<br>Local<br>Min<br>Max<br>Normalized peak height<br>Peak sensitivity value<br>Shapiro-Wilk score	-5.09E-03<br>-6.17E+01<br>2.36E-01<br>1.00E+00<br>1.93E-02<br>2.151E-04	-1.24E-02<br>-2.33E-03	-5.09E-03<br>-4.37E+03<br>3.23E+02<br>9.96E-01<br>1.39E+00<br>1.326E-02	-5.48E+02<br>1.09E+03	-5.09E-03<br>-3.54E+04<br>6.11E+04<br>9.70E-01<br>-3.14E+01<br>4.938E-02	-9.99E+02<br>3.67E+06
21	(binding of MAPKKK inactivator).k1<br>Local<br>Min<br>Max<br>Normalized peak height<br>Peak sensitivity value<br>Shapiro-Wilk score	-1.03E-02<br>-6.16E+01<br>4.66E-01<br>1.00E+00<br>1.46E-04<br>5.566E-04	-2.38E-02<br>-4.94E-03	-1.03E-02<br>-4.86E+03<br>3.23E+02<br>9.96E-01<br>-1.49E+00<br>9.646E-03	-6.38E-02<br>1.17E+03	-1.03E-02<br>-3.54E+04<br>5.69E+04<br>9.68E-01<br>-2.06E+01<br>5.684E-02	-9.97E+02<br>3.63E+06
22	(binding of MAPKKK inactivator).k2<br>Local<br>Min<br>Max<br>Normalized peak height<br>Peak sensitivity value<br>Shapiro-Wilk score	5.13E-03<br>-4.21E+01<br>5.48E-02<br>1.00E+00<br>-8.44E-03<br>3.338E-04	2.59E-03<br>1.13E-02	5.13E-03<br>-4.23E+03<br>2.83E+02<br>9.97E-01<br>1.04E+00<br>1.109E-02	-5.30E+02<br>2.98E-02	5.13E-03<br>-3.54E+04<br>6.79E+04<br>9.71E-01<br>-3.74E+01<br>4.746E-02	-1.37E+07<br>3.46E+05
23	(dephosphorylation of P-MAPK).k1<br>Local<br>Min<br>Max<br>Normalized peak height<br>Peak sensitivity value<br>Shapiro-Wilk score	-3.27E-03<br>-6.19E+01<br>-2.33E-03<br>1.00E+00<br>-3.33E-02<br>9.096E-05	-5.74E-03<br>-2.01E-03	-3.27E-03<br>-4.71E+03<br>3.23E+02<br>9.96E-01<br>-2.10E+00<br>1.072E-02	-5.56E+02<br>-1.29E-03	-3.27E-03<br>-3.54E+04<br>6.78E+04<br>9.71E-01<br>3.90E+01<br>4.788E-02	-6.93E+05<br>1.27E+06
24	(dephosphorylation of P-MAPKK).k1<br>Local<br>Min<br>Max<br>Normalized peak height<br>Peak sensitivity value<br>Shapiro-Wilk score	-2.24E-03<br>-6.03E+01<br>2.70E+00<br>1.00E+00<br>2.06E-02<br>1.528E-04	-6.88E-03<br>-7.87E-04	-2.24E-03<br>-4.87E+03<br>3.23E+02<br>9.87E-01<br>-1.64E+00<br>1.179E-02	-5.25E+02<br>1.07E+03	-2.24E-03<br>-3.54E+04<br>6.76E+04<br>9.67E-01<br>-4.17E+01<br>5.789E-02	-9.97E+02<br>3.63E+06
25	(dephosphorylation of PP-MAPK).k1<br>Local<br>Min<br>Max<br>Normalized peak height<br>Peak sensitivity value<br>Shapiro-Wilk score	-9.94E-02<br>-1.40E-01<br>-5.10E-02<br>8.36E-03<br>-9.92E-02<br>9.949E-01	-1.22E-01<br>-8.34E-02	-9.94E-02<br>-4.89E+03<br>5.86E+03<br>9.98E-01<br>-1.23E+00<br>2.678E-03	-5.24E+02<br>-7.10E-02	-9.94E-02<br>-3.54E+04<br>6.79E+04<br>9.79E-01<br>-1.71E+01<br>3.721E-02	-8.71E+06<br>3.61E+06
26	(dephosphorylation of PP-MAPKK).k1<br>Local<br>Min<br>Max<br>Normalized peak height<br>Peak sensitivity value<br>Shapiro-Wilk score	-8.63E-03<br>-6.26E+01<br>3.82E-01<br>1.00E+00<br>-2.77E-02<br>3.496E-04	-1.80E-02<br>-4.47E-03	-8.63E-03<br>-4.23E+03<br>3.23E+02<br>9.93E-01<br>1.63E+00<br>1.298E-02	-8.72E+02<br>-2.43E-03	-8.63E-03<br>-3.54E+04<br>5.69E+04<br>9.70E-01<br>-2.63E+01<br>5.287E-02	-3.32E+05<br>8.75E+05
27	(phosphorylation of MAPK).k1<br>Local<br>Min<br>Max<br>Normalized peak height<br>Peak sensitivity value<br>Shapiro-Wilk score	3.52E-03<br>2.02E-03<br>1.02E-02<br>1.18E-02<br>3.49E-03<br>9.958E-01	2.15E-03<br>6.16E-03	3.52E-03<br>-5.39E+03<br>3.80E+03<br>9.97E-01<br>-4.05E+00<br>5.574E-03	-4.84E+02<br>1.15E+03	3.52E-03<br>-3.54E+04<br>5.54E+04<br>9.70E-01<br>3.00E+01<br>5.442E-02	-2.11E+06<br>3.76E+06
28	(phosphorylation of MAPKK).k1<br>Local<br>Min<br>Max<br>Normalized peak height<br>Peak sensitivity value<br>Shapiro-Wilk score	1.34E-03<br>-4.47E+01<br>3.15E-01<br>1.00E+00<br>2.23E-02<br>1.671E-04	4.43E-04<br>4.32E-03	1.34E-03<br>-4.56E+03<br>2.83E+02<br>9.96E-01<br>-2.89E-01<br>1.332E-02	-5.16E+02<br>7.88E+02	1.34E-03<br>-3.54E+04<br>5.69E+04<br>9.68E-01<br>-2.78E+01<br>5.419E-02	-2.27E+07<br>5.76E+05
29	(phosphorylation of P-MAPK).k1<br>Local<br>Min<br>Max<br>Normalized peak height<br>Peak sensitivity value<br>Shapiro-Wilk score	1.07E-01<br>-4.14E+01<br>2.01E+01<br>1.00E+00<br>1.25E-01<br>1.093E-02	9.13E-02<br>1.28E-01	1.07E-01<br>-4.21E+03<br>3.86E+03<br>9.98E-01<br>3.72E+00<br>4.101E-03	-5.27E+02<br>1.07E+03	1.07E-01<br>-3.54E+04<br>5.30E+04<br>9.76E-01<br>-1.33E+01<br>4.601E-02	-9.24E+05<br>1.11E+06
30	(phosphorylation of P-MAPKK).k1<br>Local<br>Min<br>Max<br>Normalized peak height<br>Peak sensitivity value<br>Shapiro-Wilk score	5.25E-03<br>3.22E-03<br>2.00E+01<br>1.00E+00<br>1.32E-02<br>1.078E-03	2.56E-03<br>2.06E+02	5.25E-03<br>-4.32E+03<br>2.83E+02<br>9.97E-01<br>3.94E-01<br>9.039E-03	-5.02E+02<br>1.15E+03	5.25E-03<br>-3.54E+04<br>6.79E+04<br>9.71E-01<br>-3.49E+01<br>5.425E-02	-1.82E+06<br>9.53E+05

Table S2: Table showing distributions for the sampling-based global sensitivity analysis for the NFkB model. Local sensitivity values are shown as a dashed blue line, the best values found by the optimization-based technique are shown as dashed green lines, and 0 is shown as a dotted purple line.

ID	Name	±5%	±5% (with opt)	±10%	±10% (with opt)	±20%	±20% (with opt)
1	(R10 IkBa Cyto Nuc).k1<br>Local<br>Min<br>Max<br>Normalized peak height<br>Peak sensitivity value<br>Shapiro-Wilk score	-1.34E-01<br>-1.47E-01<br>-1.21E-01<br>3.60E-03<br>-1.35E-01<br>9.998E-01	-1.55E-01<br>-1.17E-01	-1.34E-01<br>-1.64E-01<br>-1.10E-01<br>3.72E-03<br>-1.34E-01<br>1.000E+00	-1.77E-01<br>-9.66E-02	-1.34E-01<br>-2.03E-01<br>-8.88E-02<br>3.99E-03<br>-1.34E-01<br>9.996E-01	-5.15E+02<br>-6.43E-02
2	(R11 IkBa Nuc Cyto).k1<br>Local<br>Min<br>Max<br>Normalized peak height<br>Peak sensitivity value<br>Shapiro-Wilk score	-2.03E-02<br>-2.63E-02<br>-3.43E-03<br>2.90E-03<br>-1.94E-02<br>9.416E-01	-3.13E-02<br>-1.17E-02	-2.03E-02<br>-3.47E-02<br>-7.60E-03<br>3.45E-03<br>-1.96E-02<br>9.986E-01	-4.27E-02<br>-3.02E-03	-2.03E-02<br>-4.91E-02<br>2.26E-02<br>4.61E-03<br>-1.91E-02<br>9.951E-01	-7.62E-02<br>1.06E+03
3	(R12 IKKn IKKa).k1<br>Local<br>Min<br>Max<br>Normalized peak height<br>Peak sensitivity value<br>Shapiro-Wilk score	-5.91E-02<br>-6.41E-02<br>-5.26E-02<br>3.07E-03<br>-5.86E-02<br>9.982E-01	-6.78E-02<br>-5.07E-02	-5.91E-02<br>-7.03E-02<br>-4.86E-02<br>2.84E-03<br>-5.88E-02<br>9.943E-01	-7.54E-02<br>-4.53E-02	-5.91E-02<br>-8.11E-02<br>-2.25E-02<br>3.91E-03<br>-5.65E-02<br>9.961E-01	-9.16E-02<br>3.79E-01
4	(R13 IKKa IKKi).k1<br>Local<br>Min<br>Max<br>Normalized peak height<br>Peak sensitivity value<br>Shapiro-Wilk score	4.04E-02<br>2.84E-02<br>6.62E-02<br>3.16E-03<br>4.44E-02<br>9.972E-01	2.02E-02<br>6.28E-02	4.04E-02<br>1.27E-02<br>7.20E-02<br>3.61E-03<br>4.03E-02<br>9.997E-01	-1.30E-03<br>8.80E-02	4.04E-02<br>-8.07E-02<br>1.05E-01<br>5.73E-03<br>3.76E-02<br>9.982E-01	-3.61E-02<br>1.47E-01
5	(R14 A20 dependent IKKi IKKn).k1<br>Local<br>Min<br>Max<br>Normalized peak height<br>Peak sensitivity value<br>Shapiro-Wilk score	-1.18E-01<br>-1.44E-01<br>-1.02E-01<br>3.44E-03<br>-1.21E-01<br>9.986E-01	-1.42E-01<br>-9.25E-02	-1.18E-01<br>-1.51E-01<br>-8.54E-02<br>3.42E-03<br>-1.18E-01<br>1.000E+00	-1.67E-01<br>-6.58E-02	-1.18E-01<br>-1.84E-01<br>3.91E-02<br>5.84E-03<br>-1.18E-01<br>9.993E-01	-2.30E-01<br>-2.21E-02
6	(R15 A20 transcrip).k1<br>Local<br>Min<br>Max<br>Normalized peak height<br>Peak sensitivity value<br>Shapiro-Wilk score	1.11E-01<br>9.81E-02<br>1.34E-01<br>3.28E-03<br>1.14E-01<br>9.978E-01	8.90E-02<br>1.33E-01	1.11E-01<br>8.31E-02<br>1.40E-01<br>3.65E-03<br>1.11E-01<br>9.995E-01	6.87E-02<br>1.55E-01	1.11E-01<br>-4.36E-02<br>1.66E-01<br>6.51E-03<br>1.09E-01<br>9.991E-01	2.81E-02<br>2.09E-01
7	(R16 tA20 transcrip).k1<br>Local<br>Min<br>Max<br>Normalized peak height<br>Peak sensitivity value<br>Shapiro-Wilk score	1.11E-01<br>9.81E-02<br>1.34E-01<br>3.30E-03<br>1.14E-01<br>9.978E-01	8.91E-02<br>1.32E-01	1.11E-01<br>8.30E-02<br>1.40E-01<br>3.62E-03<br>1.09E-01<br>9.991E-01	6.49E-02<br>1.55E-01	1.11E-01<br>-2.93E-02<br>1.66E-01<br>6.08E-03<br>1.09E-01<br>9.992E-01	-6.80E+00<br>2.10E-01
8	(R17 tA20 Sink).k1<br>Local<br>Min<br>Max<br>Normalized peak height<br>Peak sensitivity value<br>Shapiro-Wilk score	-1.76E-01<br>-2.05E-01<br>-1.54E-01<br>2.87E-03<br>-1.78E-01<br>9.975E-01	-2.05E-01<br>-1.45E-01	-1.76E-01<br>-2.21E-01<br>-1.28E-01<br>2.65E-03<br>-1.77E-01<br>9.958E-01	-2.34E-01<br>-1.04E-01	-1.76E-01<br>-2.58E-01<br>-7.30E-02<br>2.80E-03<br>-1.74E-01<br>9.962E-01	-2.91E-01<br>-1.94E-02
9	(R18 A20 Sink).k1<br>Local<br>Min<br>Max<br>Normalized peak height<br>Peak sensitivity value<br>Shapiro-Wilk score	-1.66E-01<br>-1.89E-01<br>-1.51E-01<br>3.40E-03<br>-1.69E-01<br>9.986E-01	-1.91E-01<br>-1.42E-01	-1.66E-01<br>-1.99E-01<br>-1.34E-01<br>3.52E-03<br>-1.66E-01<br>9.955E-01	-2.12E-01<br>-1.15E-01	-1.66E-01<br>-2.23E-01<br>9.44E-03<br>6.34E-03<br>-1.64E-01<br>9.991E-01	-2.65E-01<br>1.20E+03
10	(R19 IKKa dependent IkBa Phosphorylation).k1<br>Local<br>Min<br>Max<br>Normalized peak height<br>Peak sensitivity value<br>Shapiro-Wilk score	-2.01E-02<br>-2.58E-02<br>-1.21E-02<br>3.09E-03<br>-1.78E-02<br>9.972E-01	-3.08E-02<br>-1.10E-02	-2.01E-02<br>-3.41E-02<br>-7.39E-03<br>3.39E-03<br>-1.92E-02<br>9.985E-01	-3.98E-02<br>-2.58E-03	-2.01E-02<br>-5.50E-02<br>4.03E-02<br>6.24E-03<br>-1.85E-02<br>9.938E-01	-6.12E-02<br>1.06E+03

11	(R1 NFkB IkBa Assoc/Dissoc (backward)).k1	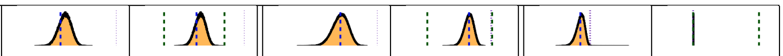					
	Local<br>Min<br>Max<br>Normalized peak height<br>Peak sensitivity value<br>Shapiro-Wilk score	-7.84E-03<br>-1.14E-02<br>-3.48E-03<br>3.76E-03<br>-7.15E-03<br>9.979E-01	-1.31E-02<br>-3.28E-03	-7.84E-03<br>-1.69E-02<br>-2.32E-03<br>3.58E-03<br>-7.65E-03<br>9.953E-01	-2.23E-02<br>2.01E-04	-7.84E-03<br>-2.77E-02<br>3.10E-02<br>7.55E-03<br>-7.16E-03<br>9.808E-01	-5.09E-02<br>1.07E+03
12	(R1 NFkB IkBa Assoc/Dissoc (forward)).k1	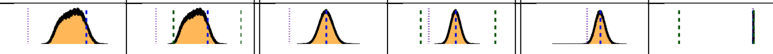					
	Local<br>Min<br>Max<br>Normalized peak height<br>Peak sensitivity value<br>Shapiro-Wilk score	1.09E-02<br>2.60E-03<br>1.47E-02<br>2.31E-03<br>9.24E-03<br>9.886E-01	3.71E-03<br>1.79E-02	1.09E-02<br>2.41E-03<br>2.04E-02<br>3.80E-03<br>1.08E-02<br>1.000E+00	-3.37E-03<br>2.71E-02	1.09E-02<br>-2.80E-02<br>3.15E-02<br>6.13E-03<br>1.06E-02<br>9.987E-01	-6.91E+00<br>5.60E-02
13	(R20 IKKa dependent NF-kB.IkBa Phosphorylation).k1	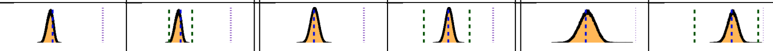					
	Local<br>Min<br>Max<br>Normalized peak height<br>Peak sensitivity value<br>Shapiro-Wilk score	-9.34E-02<br>-1.21E-01<br>-7.85E-02<br>3.10E-03<br>-9.76E-02<br>9.957E-01	-1.15E-01<br>-7.19E-02	-9.34E-02<br>-1.25E-01<br>-6.21E-02<br>3.45E-03<br>-9.38E-02<br>9.998E-01	-1.45E-01<br>-4.94E-02	-9.34E-02<br>-1.57E-01<br>-7.55E-03<br>4.32E-03<br>-9.21E-02<br>9.990E-01	-2.03E-01<br>-1.63E-02
14	(R21 pIkBa Sink).k1	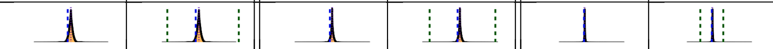					
	Local<br>Min<br>Max<br>Normalized peak height<br>Peak sensitivity value<br>Shapiro-Wilk score	-1.43E-04<br>-1.70E-03<br>1.71E-03<br>2.13E-02<br>1.49E-06<br>9.121E-01	-1.46E-03<br>1.86E-03	-1.43E-04<br>-2.58E-03<br>2.61E-03<br>4.27E-02<br>1.41E-06<br>8.637E-01	-2.09E-03<br>2.50E-03	-1.43E-04<br>-7.34E-03<br>1.13E-02<br>1.24E-01<br>7.40E-06<br>6.542E-01	-3.57E-03<br>3.25E-03
15	(R22 pIkBa.NF-kB Sink).k1	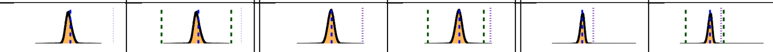					
	Local<br>Min<br>Max<br>Normalized peak height<br>Peak sensitivity value<br>Shapiro-Wilk score	-2.51E-03<br>-4.54E-03<br>-7.55E-04<br>8.88E-03<br>-2.63E-03<br>9.626E-01	-4.66E-03<br>-6.09E-04	-2.51E-03<br>-5.34E-03<br>6.65E-05<br>8.47E-03<br>-2.57E-03<br>9.872E-01	-5.07E-03<br>-5.33E-04	-2.51E-03<br>-9.50E-03<br>9.94E-03<br>1.68E-02<br>-2.43E-03<br>9.735E-01	-8.17E-03<br>6.77E-04
16	(R23 nNF-kB nIkBa Assoc/Dissoc (backward)).k1	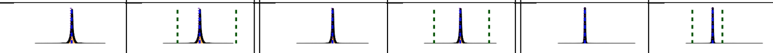					
	Local<br>Min<br>Max<br>Normalized peak height<br>Peak sensitivity value<br>Shapiro-Wilk score	4.72E-05<br>-1.65E-03<br>1.58E-03<br>3.13E-02<br>5.13E-05<br>8.798E-01	-9.82E-04<br>1.74E-03	4.72E-05<br>-2.46E-03<br>2.51E-03<br>3.83E-02<br>4.91E-05<br>8.957E-01	-1.80E-03<br>2.06E-03	4.72E-05<br>-7.67E-03<br>1.45E-02<br>9.84E-02<br>6.22E-05<br>6.716E-01	-5.80E-03<br>2.95E-03
17	(R23 nNF-kB nIkBa Assoc/Dissoc (forward)).k1	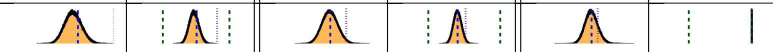					
	Local<br>Min<br>Max<br>Normalized peak height<br>Peak sensitivity value<br>Shapiro-Wilk score	-3.31E-03<br>-7.10E-03<br>-4.43E-05<br>3.77E-03<br>-3.87E-03<br>9.990E-01	-8.81E-03<br>2.02E-03	-3.31E-03<br>-1.07E-02<br>4.74E-03<br>3.76E-03<br>-3.81E-03<br>9.998E-01	-1.52E-02<br>1.40E-02	-3.31E-03<br>-2.25E-02<br>1.85E-02<br>5.09E-03<br>-4.19E-03<br>9.967E-01	-5.11E+02<br>4.80E-02
18	(R24 nIkBa Sink).k1	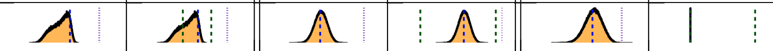					
	Local<br>Min<br>Max<br>Normalized peak height<br>Peak sensitivity value<br>Shapiro-Wilk score	-4.08E-02<br>-9.75E-02<br>-2.96E-02<br>2.69E-03<br>-4.58E-02<br>9.628E-01	-6.21E-02<br>-2.24E-02	-4.08E-02<br>-6.97E-02<br>-1.61E-02<br>3.65E-03<br>-4.10E-02<br>9.997E-01	-8.61E-02<br>-7.43E-03	-4.08E-02<br>-9.78E-02<br>1.38E-02<br>3.92E-03<br>-3.78E-02<br>9.975E-01	-1.39E-01<br>1.06E+03
19	(R2 tIkBa transcrip).k1	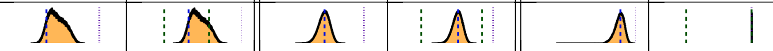					
	Local<br>Min<br>Max<br>Normalized peak height<br>Peak sensitivity value<br>Shapiro-Wilk score	-7.38E-02<br>-9.52E-02<br>-2.12E-02<br>2.58E-03<br>-6.62E-02<br>9.846E-01	-1.08E-01<br>-4.56E-02	-7.38E-02<br>-1.28E-01<br>-3.65E-02<br>3.59E-03<br>-7.35E-02<br>9.952E-01	-1.50E-01<br>-2.33E-02	-7.38E-02<br>-3.70E-01<br>-9.62E-03<br>7.05E-03<br>-7.01E-02<br>9.840E-01	-5.32E+02<br>1.33E-02
20	(R3 tIkBa dependent IkBa transcrip).k1	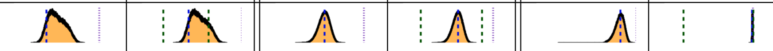					
	Local<br>Min<br>Max<br>Normalized peak height<br>Peak sensitivity value<br>Shapiro-Wilk score	-7.38E-02<br>-9.52E-02<br>-2.13E-02<br>2.54E-03<br>-6.59E-02<br>9.846E-01	-1.09E-01<br>-4.61E-02	-7.38E-02<br>-1.28E-01<br>-3.65E-02<br>3.59E-03<br>-7.29E-02<br>9.952E-01	-1.52E-01<br>-2.34E-02	-7.38E-02<br>-3.63E-01<br>-9.60E-03<br>6.95E-03<br>-7.09E-02<br>9.840E-01	-6.50E+00<br>1.48E-02
21	(R4 tIkBa Sink).k1	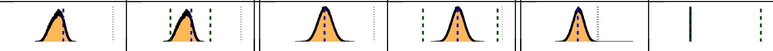					
	Local<br>Min<br>Max<br>Normalized peak height<br>Peak sensitivity value<br>Shapiro-Wilk score	-1.05E-01<br>-1.64E-01<br>-7.97E-02<br>2.72E-03<br>-1.16E-01<br>9.920E-01	-1.49E-01<br>-6.51E-02	-1.05E-01<br>-1.66E-01<br>-4.45E-02<br>3.43E-03<br>-1.03E-01<br>9.999E-01	-1.86E-01<br>-1.15E-02	-1.05E-01<br>-2.23E-01<br>1.80E-01<br>5.84E-03<br>-1.04E-01<br>9.995E-01	-2.85E-01<br>1.15E+03

22	(R5 IkBa Sink).k1	Local<br>Min<br>Max<br>Normalized peak height<br>Peak sensitivity value<br>Shapiro-Wilk score	-7.04E-02<br>-8.67E-02<br>-5.24E-02<br>3.54E-03<br>-6.73E-02<br>9.982E-01	-9.94E-02<br>-4.47E-02	-7.04E-02<br>-1.04E-01<br>-3.54E-02<br>3.41E-03<br>-6.92E-02<br>1.000E+00	-1.28E-01<br>-2.16E-02	-7.04E-02<br>-1.43E-01<br>7.97E-02<br>5.69E-03<br>-6.83E-02<br>9.993E-01	-1.94E-01<br>1.07E+03
23	(R6 NF-kB dependent IkBa degradation).k1	Local<br>Min<br>Max<br>Normalized peak height<br>Peak sensitivity value<br>Shapiro-Wilk score	-5.92E-03<br>-8.89E-03<br>-3.81E-03<br>4.96E-03<br>-6.19E-03<br>9.978E-01	-9.19E-03<br>-3.28E-03	-5.92E-03<br>-9.77E-03<br>-2.50E-03<br>4.98E-03<br>-5.80E-03<br>9.971E-01	-1.06E-02<br>-7.06E-04	-5.92E-03<br>-1.28E-02<br>1.68E-02<br>1.04E-02<br>-5.84E-03<br>9.873E-01	-1.84E-02<br>1.19E+03
24	(R7 nNF-kB.nIkBa nNF-kB).k1	Local<br>Min<br>Max<br>Normalized peak height<br>Peak sensitivity value<br>Shapiro-Wilk score	0.00E+00<br>0.00E+00<br>0.00E+00<br>1.00E+00<br>5.00E-04<br>1.000E+00	0.00E+00<br>0.00E+00	0.00E+00<br>0.00E+00<br>0.00E+00<br>1.00E+00<br>5.00E-04<br>1.000E+00	0.00E+00<br>0.00E+00	0.00E+00<br>-0.00E+00<br>-0.00E+00<br>1.00E+00<br>5.00E-04<br>1.000E+00	0.00E+00<br>0.00E+00
25	(R8 nNF-kB NF-kB transport).k1	Local<br>Min<br>Max<br>Normalized peak height<br>Peak sensitivity value<br>Shapiro-Wilk score	-3.54E-02<br>-4.32E-02<br>-3.13E-02<br>3.15E-03<br>-3.66E-02<br>9.958E-01	-4.12E-02<br>-2.99E-02	-3.54E-02<br>-4.38E-02<br>-2.75E-02<br>2.62E-03<br>-3.51E-02<br>9.950E-01	-4.84E-02<br>-2.51E-02	-3.54E-02<br>-6.11E-02<br>-1.16E-02<br>4.14E-03<br>-3.52E-02<br>9.929E-01	-4.63E-01<br>-1.65E-02
26	(R8a NF-kB nNF-kB transport).k1	Local<br>Min<br>Max<br>Normalized peak height<br>Peak sensitivity value<br>Shapiro-Wilk score	-6.59E-02<br>-7.50E-02<br>-6.07E-02<br>3.32E-03<br>-6.70E-02<br>9.962E-01	-7.58E-02<br>-5.58E-02	-6.59E-02<br>-7.83E-02<br>-5.40E-02<br>3.36E-03<br>-6.60E-02<br>9.896E-01	-8.61E-02<br>-5.05E-02	-6.59E-02<br>-9.21E-02<br>7.59E-03<br>6.86E-03<br>-6.61E-02<br>9.982E-01	-1.13E-01<br>-3.48E-02
27	(R9 nNF-kB.nIkBa NF-kB.IkBa transport).k1	Local<br>Min<br>Max<br>Normalized peak height<br>Peak sensitivity value<br>Shapiro-Wilk score	-9.45E-05<br>-2.33E-03<br>1.99E-03<br>7.10E-03<br>-1.57E-04<br>9.539E-01	-1.62E-03<br>1.67E-03	-9.45E-05<br>-2.60E-03<br>2.90E-03<br>1.95E-02<br>-8.17E-06<br>8.347E-01	-2.88E-03<br>1.91E-03	-9.45E-05<br>-7.21E-03<br>8.93E-03<br>3.84E-02<br>-6.12E-06<br>7.981E-01	-5.08E+02<br>6.30E-03

Table S3: Table showing distributions for the sampling-based global sensitivity analysis for the Cell Cycle model. Local sensitivity values are shown as a dashed blue line, the best values found by the optimization-based technique are shown as dashed green lines, and 0 is shown as a dotted purple line.

ID	Name	±5%	±5% (with opt)	±10%	±10% (with opt)
1	Values[C0]<br>Local<br>Min<br>Max<br>Normalized peak height<br>Peak sensitivity value<br>Shapiro-Wilk score	-7.66E-05<br>-5.30E-01<br>1.12E+00<br>7.99E-01<br>5.28E-04<br>1.985E-02	-2.46E-01<br>2.09E-01	-7.66E-05<br>-1.68E+00<br>1.95E+00<br>9.71E-01<br>8.91E-04<br>1.938E-02	-1.06E+02<br>8.25E-01
2	Values[CDC15T]<br>Local<br>Min<br>Max<br>Normalized peak height<br>Peak sensitivity value<br>Shapiro-Wilk score	-5.38E-05<br>-5.30E-01<br>1.11E+00<br>9.82E-01<br>3.04E-04<br>1.847E-02	-7.22E-04<br>7.34E-04	-5.38E-05<br>-1.66E+00<br>1.91E+00<br>9.71E-01<br>-6.56E-04<br>1.806E-02	-3.42E-03<br>5.35E+00
3	Values[Dn3]<br>Local<br>Min<br>Max<br>Normalized peak height<br>Peak sensitivity value<br>Shapiro-Wilk score	-9.43E-05<br>-5.30E-01<br>5.74E-01<br>8.24E-01<br>-3.88E-04<br>2.503E-02	-2.15E-03<br>3.79E-02	-9.43E-05<br>-1.59E+00<br>2.67E+00<br>9.88E-01<br>-4.36E-04<br>1.400E-02	-8.69E+01<br>9.59E-01
4	Values[ESP1T]<br>Local<br>Min<br>Max<br>Normalized peak height<br>Peak sensitivity value<br>Shapiro-Wilk score	-1.13E-04<br>-5.31E-01<br>6.46E-01<br>6.39E-01<br>-6.21E-04<br>2.379E-02	-4.26E-02<br>7.16E-04	-1.13E-04<br>-1.38E+00<br>2.27E+00<br>9.91E-01<br>-8.67E-04<br>1.475E-02	-5.96E-02<br>1.78E+00
5	Values[IET]<br>Local<br>Min<br>Max<br>Normalized peak height<br>Peak sensitivity value<br>Shapiro-Wilk score	-8.33E-05<br>-5.31E-01<br>5.23E-01<br>8.06E-01<br>-3.87E-04<br>2.236E-02	-3.79E-02<br>6.79E-04	-8.33E-05<br>-7.00E+00<br>5.67E+00<br>9.96E-01<br>8.68E-04<br>5.094E-03	-3.67E-03<br>2.23E-02
6	Values[J20ppx]<br>Local<br>Min<br>Max<br>Normalized peak height<br>Peak sensitivity value<br>Shapiro-Wilk score	-8.03E-05<br>-5.30E-01<br>4.88E-01<br>9.77E-01<br>-5.36E-05<br>2.215E-02	-4.97E-01<br>5.29E-01	-8.03E-05<br>-1.39E+00<br>3.87E+00<br>9.80E-01<br>-1.09E-03<br>1.223E-02	-3.22E-02<br>2.89E+00
7	Values[Jacdh]<br>Local<br>Min<br>Max<br>Normalized peak height<br>Peak sensitivity value<br>Shapiro-Wilk score	4.41E-05<br>-5.30E-01<br>4.46E-01<br>8.45E-01<br>2.89E-04<br>2.244E-02	-6.55E-04<br>2.75E-03	4.41E-05<br>-1.24E+00<br>3.18E+00<br>9.95E-01<br>-2.05E-04<br>9.482E-03	-1.07E-01<br>6.73E-02
8	Values[Jaiep]<br>Local<br>Min<br>Max<br>Normalized peak height<br>Peak sensitivity value<br>Shapiro-Wilk score	2.24E-05<br>-5.30E-01<br>2.77E-01<br>6.92E-01<br>-3.00E-04<br>2.395E-02	-7.07E-04<br>7.06E-04	2.24E-05<br>-2.95E+00<br>1.95E+00<br>9.94E-01<br>-1.15E-03<br>1.240E-02	-4.75E-03<br>1.57E-01
9	Values[Jamcm]<br>Local<br>Min<br>Max<br>Normalized peak height<br>Peak sensitivity value<br>Shapiro-Wilk score	-8.02E-05<br>-5.30E-01<br>7.84E-01<br>9.89E-01<br>1.22E-04<br>2.060E-02	-1.32E-01<br>1.44E-01	-8.02E-05<br>-2.45E+00<br>3.65E+00<br>9.95E-01<br>-1.54E-03<br>8.947E-03	-6.11E-03<br>8.76E-04
10	Values[Jasbf]<br>Local<br>Min<br>Max<br>Normalized peak height<br>Peak sensitivity value<br>Shapiro-Wilk score	1.34E-04<br>-5.30E-01<br>9.07E-01<br>7.41E-01<br>6.09E-04<br>1.963E-02	-2.19E-01<br>9.71E-03	1.34E-04<br>-1.24E+00<br>4.62E+00<br>9.95E-01<br>-1.43E-03<br>6.909E-03	-7.08E-02<br>2.12E-02
11	Values[Jatem]<br>Local<br>Min<br>Max<br>Normalized peak height<br>Peak sensitivity value<br>Shapiro-Wilk score	-4.58E-05<br>-5.30E-01<br>3.41E-01<br>9.28E-01<br>1.26E-04<br>2.303E-02	-1.46E-01<br>4.95E-02	-4.58E-05<br>-1.28E+00<br>5.60E+00<br>9.95E-01<br>-1.15E-05<br>6.625E-03	-3.99E+00<br>7.81E-03
12	Values[Jd2c1]<br>Local<br>Min<br>Max<br>Normalized peak height<br>Peak sensitivity value<br>Shapiro-Wilk score	-2.27E-05<br>-5.30E-01<br>6.63E-01<br>8.92E-01<br>3.57E-04<br>2.173E-02	-3.88E-02<br>3.43E-01	-2.27E-05<br>-1.34E+00<br>2.04E+00<br>5.70E-01<br>-1.63E-03<br>1.161E-02	-7.86E-04<br>9.06E-01

13	Values[Jd2f6]<br>Local<br>Min<br>Max<br>Normalized peak height<br>Peak sensitivity value<br>Shapiro-Wilk score	5.54E-05<br>-5.30E-01<br>5.40E-01<br>9.76E-01<br>1.15E-04<br>2.212E-02	-1.93E-02<br>2.16E-02	5.54E-05<br>-1.24E+00<br>1.95E+00<br>9.45E-01<br>1.28E-03<br>1.299E-02	-1.44E-01<br>7.71E-04
14	Values[Jicdh]<br>Local<br>Min<br>Max<br>Normalized peak height<br>Peak sensitivity value<br>Shapiro-Wilk score	-5.31E-05<br>-5.30E-01<br>3.85E-01<br>8.70E-01<br>2.30E-04<br>2.430E-02	-4.20E-02<br>2.11E-01	-5.31E-05<br>-2.12E+00<br>2.71E+00<br>9.94E-01<br>-5.71E-04<br>1.149E-02	-8.42E-04<br>1.63E-02
15	Values[Jiiep]<br>Local<br>Min<br>Max<br>Normalized peak height<br>Peak sensitivity value<br>Shapiro-Wilk score	-8.80E-05<br>-5.30E-01<br>6.45E-01<br>5.74E-01<br>5.18E-04<br>2.314E-02	-1.37E-02<br>6.55E-04	-8.80E-05<br>-1.52E+00<br>7.07E+00<br>9.36E-01<br>3.90E-03<br>5.633E-03	-1.12E-01<br>7.39E-04
16	Values[Jimcm]<br>Local<br>Min<br>Max<br>Normalized peak height<br>Peak sensitivity value<br>Shapiro-Wilk score	-4.14E-05<br>-5.31E-01<br>1.02E+00<br>9.11E-01<br>-5.54E-04<br>2.105E-02	-2.65E+00<br>6.83E-02	-4.14E-05<br>-1.24E+00<br>2.71E+00<br>7.93E-01<br>1.46E-03<br>1.651E-02	-3.59E-02<br>5.54E-02
17	Values[Jisbf]<br>Local<br>Min<br>Max<br>Normalized peak height<br>Peak sensitivity value<br>Shapiro-Wilk score	2.65E-04<br>-5.30E-01<br>3.89E-01<br>9.67E-01<br>-1.83E-05<br>2.425E-02	-1.97E-01<br>9.54E-04	2.65E-04<br>-2.07E+00<br>4.34E+00<br>9.95E-01<br>7.02E-04<br>1.048E-02	-1.57E+01<br>7.22E-04
18	Values[Jitem]<br>Local<br>Min<br>Max<br>Normalized peak height<br>Peak sensitivity value<br>Shapiro-Wilk score	-2.15E-04<br>-5.30E-01<br>6.46E-01<br>9.79E-01<br>-1.21E-04<br>2.218E-02	-1.50E-01<br>1.73E-02	-2.15E-04<br>-2.58E+00<br>5.96E+00<br>9.95E-01<br>3.14E-04<br>6.706E-03	-6.99E-04<br>6.32E-01
19	Values[Jn3]<br>Local<br>Min<br>Max<br>Normalized peak height<br>Peak sensitivity value<br>Shapiro-Wilk score	6.08E-05<br>-5.31E-01<br>3.10E-01<br>7.05E-01<br>3.47E-04<br>2.750E-02	-6.82E-02<br>1.50E-01	6.08E-05<br>-3.28E+00<br>8.28E+00<br>9.72E-01<br>4.95E-03<br>6.880E-03	-2.24E+00<br>2.30E+01
20	Values[Jpds]<br>Local<br>Min<br>Max<br>Normalized peak height<br>Peak sensitivity value<br>Shapiro-Wilk score	2.65E-05<br>-5.30E-01<br>5.28E-01<br>8.91E-01<br>-3.13E-04<br>2.263E-02	-1.06E-01<br>7.50E-03	2.65E-05<br>-1.24E+00<br>5.70E+00<br>9.95E-01<br>-2.21E-03<br>6.339E-03	-4.04E-01<br>2.31E-01
21	Values[Jspn]<br>Local<br>Min<br>Max<br>Normalized peak height<br>Peak sensitivity value<br>Shapiro-Wilk score	-2.94E-05<br>-5.30E-01<br>3.21E-01<br>7.69E-01<br>-2.28E-04<br>2.577E-02	-6.64E-04<br>6.86E-04	-2.94E-05<br>-1.24E+00<br>3.17E+00<br>9.93E-01<br>6.11E-04<br>1.502E-02	-7.27E-02<br>3.50E+00
22	Values[KEZ2]<br>Local<br>Min<br>Max<br>Normalized peak height<br>Peak sensitivity value<br>Shapiro-Wilk score	-3.68E-05<br>-5.30E-01<br>2.76E-01<br>6.57E-01<br>-3.37E-04<br>2.327E-02	-4.89E-02<br>1.61E-01	-3.68E-05<br>-2.01E+00<br>1.95E+00<br>9.95E-01<br>-5.15E-04<br>1.268E-02	-1.06E-02<br>8.37E-04
23	Values[KEZ]<br>Local<br>Min<br>Max<br>Normalized peak height<br>Peak sensitivity value<br>Shapiro-Wilk score	-1.50E-04<br>-5.30E-01<br>4.65E-01<br>9.62E-01<br>-1.23E-04<br>2.342E-02	-1.23E-01<br>1.27E-01	-1.50E-04<br>-1.45E+00<br>8.92E+00<br>9.96E-01<br>-2.97E-03<br>4.486E-03	-1.30E+00<br>7.91E-04
24	Values[TEM1T]<br>Local<br>Min<br>Max<br>Normalized peak height<br>Peak sensitivity value<br>Shapiro-Wilk score	-2.28E-05<br>-5.30E-01<br>3.36E-01<br>7.03E-01<br>-3.32E-04<br>2.470E-02	-1.57E-03<br>8.35E-04	-2.28E-05<br>-1.24E+00<br>3.61E+00<br>9.91E-01<br>-6.84E-04<br>1.232E-02	-1.57E-02<br>8.03E+00
25	Values[b0]<br>Local<br>Min<br>Max<br>Normalized peak height<br>Peak sensitivity value<br>Shapiro-Wilk score	-1.01E-04<br>-5.30E-01<br>3.43E-01<br>8.29E-01<br>-2.24E-04<br>2.822E-02	-4.47E-03<br>1.66E+00	-1.01E-04<br>-1.24E+00<br>5.85E+00<br>9.52E-01<br>-2.72E-03<br>8.427E-03	-6.65E-01<br>1.09E+00
26	Values[bub2h]<br>Local<br>Min<br>Max<br>Normalized peak height<br>Peak sensitivity value<br>Shapiro-Wilk score	-3.59E-05<br>-5.30E-01<br>5.16E-01<br>9.13E-01<br>-2.46E-04<br>2.178E-02	-5.54E-04<br>3.45E-01	-3.59E-05<br>-1.59E+00<br>7.14E+00<br>9.95E-01<br>-2.48E-03<br>6.096E-03	-1.81E-01<br>1.91E-02

27	Values[bub2l]<br>Local<br>Min<br>Max<br>Normalized peak height<br>Peak sensitivity value<br>Shapiro-Wilk score	-6.62E-05<br>-5.31E-01<br>5.97E-01<br>9.66E-01<br>-1.72E-04<br>2.419E-02	-4.66E+00<br>1.41E-01	-6.62E-05<br>-2.56E+00<br>4.85E+00<br>9.50E-01<br>1.52E-03<br>1.392E-02	-8.10E-01<br>1.17E-01
28	Values[ebudb5]<br>Local<br>Min<br>Max<br>Normalized peak height<br>Peak sensitivity value<br>Shapiro-Wilk score	0.00E+00<br>-5.30E-01<br>5.46E-01<br>9.85E-01<br>1.23E-04<br>1.591E-02	-2.44E-02<br>2.18E-01	0.00E+00<br>-2.12E+00<br>2.47E+00<br>9.08E-01<br>2.11E-03<br>8.588E-03	-9.97E-02<br>1.19E-02
29	Values[ebudn2]<br>Local<br>Min<br>Max<br>Normalized peak height<br>Peak sensitivity value<br>Shapiro-Wilk score	0.00E+00<br>-5.30E-01<br>5.38E-01<br>9.89E-01<br>6.26E-05<br>1.462E-02	-1.36E-01<br>7.86E-04	0.00E+00<br>-2.00E+00<br>1.95E+00<br>9.84E-01<br>1.54E-03<br>9.700E-03	-7.33E-01<br>7.18E+00
30	Values[ebudn3]<br>Local<br>Min<br>Max<br>Normalized peak height<br>Peak sensitivity value<br>Shapiro-Wilk score	0.00E+00<br>-5.30E-01<br>4.73E-01<br>9.88E-01<br>-2.40E-05<br>1.584E-02	-3.17E-01<br>5.78E-03	0.00E+00<br>-1.35E+00<br>7.08E+00<br>8.78E-01<br>-4.08E-03<br>4.758E-03	-1.69E-02<br>1.09E-01
31	Values[ec1b2]<br>Local<br>Min<br>Max<br>Normalized peak height<br>Peak sensitivity value<br>Shapiro-Wilk score	2.13E-04<br>-5.30E-01<br>4.44E-01<br>9.39E-01<br>1.59E-04<br>2.172E-02	-1.92E-01<br>7.82E-04	2.13E-04<br>-1.60E+00<br>2.53E+00<br>9.94E-01<br>9.04E-04<br>1.111E-02	-1.85E-01<br>5.75E-04
32	Values[ec1b5]<br>Local<br>Min<br>Max<br>Normalized peak height<br>Peak sensitivity value<br>Shapiro-Wilk score	-1.49E-04<br>-5.30E-01<br>5.36E-01<br>8.72E-01<br>3.10E-04<br>2.170E-02	-1.99E-01<br>2.39E-01	-1.49E-04<br>-1.24E+00<br>1.98E+00<br>9.94E-01<br>-5.94E-04<br>1.273E-02	-4.77E-02<br>2.82E-01
33	Values[ec1k2]<br>Local<br>Min<br>Max<br>Normalized peak height<br>Peak sensitivity value<br>Shapiro-Wilk score	-8.09E-05<br>-5.30E-01<br>4.78E-01<br>6.53E-01<br>-4.40E-04<br>2.333E-02	-8.36E-04<br>1.15E-01	-8.09E-05<br>-1.24E+00<br>2.92E+00<br>9.83E-01<br>1.57E-03<br>1.217E-02	-4.76E-04<br>7.27E-02
34	Values[ec1n2]<br>Local<br>Min<br>Max<br>Normalized peak height<br>Peak sensitivity value<br>Shapiro-Wilk score	-1.92E-04<br>-5.31E-01<br>6.52E-01<br>9.78E-01<br>-1.69E-04<br>2.353E-02	-4.20E-01<br>7.27E-02	-1.92E-04<br>-1.84E+00<br>7.56E+00<br>9.53E-01<br>3.57E-03<br>5.681E-03	-1.44E+00<br>2.24E-02
35	Values[ec1n3]<br>Local<br>Min<br>Max<br>Normalized peak height<br>Peak sensitivity value<br>Shapiro-Wilk score	-1.88E-04<br>-5.30E-01<br>3.24E-01<br>6.07E-01<br>-3.95E-04<br>2.418E-02	-4.32E-02<br>8.26E-04	-1.88E-04<br>-1.24E+00<br>1.95E+00<br>9.80E-01<br>8.64E-04<br>1.296E-02	-2.31E-01<br>6.83E-04
36	Values[ef6b2]<br>Local<br>Min<br>Max<br>Normalized peak height<br>Peak sensitivity value<br>Shapiro-Wilk score	-9.72E-05<br>-5.30E-01<br>3.60E-01<br>9.48E-01<br>-9.61E-05<br>2.490E-02	-3.32E-02<br>1.79E-02	-9.72E-05<br>-1.59E+00<br>5.93E+00<br>9.95E-01<br>1.74E-03<br>6.825E-03	-2.06E-01<br>9.91E-04
37	Values[ef6b5]<br>Local<br>Min<br>Max<br>Normalized peak height<br>Peak sensitivity value<br>Shapiro-Wilk score	-8.31E-06<br>-5.30E-01<br>2.98E-01<br>6.30E-01<br>3.51E-04<br>2.300E-02	-2.02E-01<br>1.51E-01	-8.31E-06<br>-2.99E+00<br>3.04E+00<br>9.95E-01<br>2.23E-04<br>1.001E-02	-1.79E-01<br>2.93E-01
38	Values[ef6k2]<br>Local<br>Min<br>Max<br>Normalized peak height<br>Peak sensitivity value<br>Shapiro-Wilk score	1.36E-05<br>-5.31E-01<br>7.81E-01<br>9.88E-01<br>-1.28E-04<br>2.049E-02	-3.22E-01<br>9.85E-02	1.36E-05<br>-1.24E+00<br>2.85E+00<br>9.94E-01<br>1.06E-03<br>1.066E-02	-6.53E-02<br>1.04E-03
39	Values[ef6n2]<br>Local<br>Min<br>Max<br>Normalized peak height<br>Peak sensitivity value<br>Shapiro-Wilk score	-1.09E-04<br>-5.31E-01<br>3.33E-01<br>9.21E-01<br>6.23E-05<br>2.530E-02	-1.75E-03<br>3.34E-01	-1.09E-04<br>-1.43E+00<br>3.45E+00<br>9.93E-01<br>-6.76E-04<br>1.235E-02	-5.80E+00<br>2.11E-02
40	Values[ef6n3]<br>Local<br>Min<br>Max<br>Normalized peak height<br>Peak sensitivity value<br>Shapiro-Wilk score	-3.80E-04<br>-5.30E-01<br>5.98E-01<br>7.47E-01<br>-4.55E-04<br>2.143E-02	-7.85E-04<br>1.61E-01	-3.80E-04<br>-1.24E+00<br>3.54E+00<br>9.95E-01<br>-3.20E-04<br>9.262E-03	-1.50E+00<br>1.01E-03

41	Values[eicdhb2]				
	Local	-6.91E-05		-6.91E-05	
	Min	-5.30E-01	-7.51E-04	-1.47E+00	-7.67E-02
	Max	2.47E-01	1.67E-01	3.09E+00	2.47E-02
	Normalized peak height	8.94E-01		9.42E-01	
	Peak sensitivity value	1.44E-04		1.96E-03	
	Shapiro-Wilk score	2.479E-02		1.145E-02	
42	Values[eicdhb5]				
	Local	5.93E-05		5.93E-05	
	Min	-5.30E-01	-7.67E-02	-2.04E+00	-1.33E-01
	Max	4.68E-01	5.93E-04	3.80E+00	1.54E-01
	Normalized peak height	8.80E-01		9.89E-01	
	Peak sensitivity value	2.67E-04		-2.09E-03	
	Shapiro-Wilk score	2.364E-02		1.003E-02	
43	Values[eicdhn2]				
	Local	-2.33E-04		-2.33E-04	
	Min	-5.30E-01	-9.04E-03	-1.41E+00	-5.90E-02
	Max	5.36E-01	4.03E-02	2.65E+00	8.73E-02
	Normalized peak height	9.79E-01		9.94E-01	
	Peak sensitivity value	2.76E-05		-5.62E-04	
	Shapiro-Wilk score	2.108E-02		1.074E-02	
44	Values[eicdhn3]				
	Local	-7.71E-05		-7.71E-05	
	Min	-5.30E-01	-1.13E-01	-1.85E+00	-1.29E-03
	Max	2.93E-01	5.71E-02	1.95E+00	9.46E-04
	Normalized peak height	8.16E-01		9.93E-01	
	Peak sensitivity value	-2.41E-04		-1.20E-03	
	Shapiro-Wilk score	2.465E-02		1.203E-02	
45	Values[eorib2]				
	Local	1.29E-04		1.29E-04	
	Min	-5.30E-01	-1.34E-01	-2.98E+00	-7.63E-02
	Max	4.18E-01	7.91E-03	6.84E+00	3.56E-02
	Normalized peak height	9.68E-01		9.96E-01	
	Peak sensitivity value	4.49E-05		-8.23E-04	
	Shapiro-Wilk score	2.227E-02		5.489E-03	
46	Values[eorib5]				
	Local	1.32E-04		1.32E-04	
	Min	-5.39E-01	-6.20E-04	-2.52E+00	-1.71E+00
	Max	8.66E-01	1.98E-01	2.21E+00	3.62E+00
	Normalized peak height	9.93E-01		9.95E-01	
	Peak sensitivity value	1.91E-05		1.17E-03	
	Shapiro-Wilk score	1.916E-02		1.149E-02	
47	Values[esbfb5]				
	Local	-2.23E-04		-2.23E-04	
	Min	-5.30E-01	-8.98E-04	-1.24E+00	-1.53E-02
	Max	4.75E-01	2.34E-01	3.81E+00	8.68E-04
	Normalized peak height	9.75E-01		9.63E-01	
	Peak sensitivity value	5.50E-05		-2.13E-03	
	Shapiro-Wilk score	2.247E-02		1.008E-02	
48	Values[esbfn2]				
	Local	-1.29E-04		-1.29E-04	
	Min	-5.38E-01	-7.80E-04	-3.62E+00	-1.47E-02
	Max	6.46E-01	3.83E-02	3.08E+00	1.15E+00
	Normalized peak height	9.66E-01		9.95E-01	
	Peak sensitivity value	1.69E-04		6.65E-04	
	Shapiro-Wilk score	2.150E-02		9.908E-03	
49	Values[esbfn3]				
	Local	-1.81E-04		-1.81E-04	
	Min	-5.29E-01	-1.43E+00	-1.24E+00	-1.33E-01
	Max	3.73E-01	6.84E-03	1.95E+00	2.24E+00
	Normalized peak height	7.37E-01		9.10E-01	
	Peak sensitivity value	4.94E-05		-5.26E-04	
	Shapiro-Wilk score	3.629E-02		2.786E-02	
50	Values[ka15”]				
	Local	-1.73E-05		-1.73E-05	
	Min	-5.30E-01	-2.58E-01	-1.24E+00	-6.79E-04
	Max	4.49E-01	2.09E+00	1.95E+00	3.88E-01
	Normalized peak height	9.71E-01		9.27E-01	
	Peak sensitivity value	4.24E-05		1.26E-03	
	Shapiro-Wilk score	2.274E-02		1.659E-02	
51	Values[ka15’]				
	Local	1.78E-04		1.78E-04	
	Min	-5.30E-01	-7.96E-04	-1.89E+00	-1.33E-01
	Max	3.53E-01	6.61E-04	3.49E+00	8.47E-04
	Normalized peak height	9.62E-01		9.95E-01	
	Peak sensitivity value	1.48E-05		9.23E-04	
	Shapiro-Wilk score	2.294E-02		1.021E-02	
52	Values[ka15p]				
	Local	1.10E-04		1.10E-04	
	Min	-5.30E-01	-6.27E-04	-2.90E+00	-4.29E-01
	Max	3.82E-01	7.24E-04	5.53E+00	8.96E-04
	Normalized peak height	9.32E-01		9.96E-01	
	Peak sensitivity value	1.51E-04		1.17E-03	
	Shapiro-Wilk score	2.274E-02		6.777E-03	
53	Values[ka20”]				
	Local	-1.09E-04		-1.09E-04	
	Min	-5.30E-01	-3.05E-02	-1.24E+00	-4.90E+00
	Max	3.73E-01	3.68E-02	4.99E+00	1.01E-03
	Normalized peak height	8.00E-01		9.95E-01	
	Peak sensitivity value	-3.45E-04		1.06E-03	
	Shapiro-Wilk score	2.454E-02		8.500E-03	
54	Values[ka20’]				
	Local	-9.51E-05		-9.51E-05	
	Min	-5.30E-01	-8.91E-04	-1.95E+00	-5.84E+00
	Max	4.28E-01	7.25E-04	3.00E+00	2.86E+02
	Normalized peak height	7.14E-01		9.94E-01	
	Peak sensitivity value	3.13E-04		8.98E-04	
	Shapiro-Wilk score	2.378E-02		1.075E-02	

55	Values[kacdh”]	Local<br>Min<br>Max<br>Normalized peak height<br>Peak sensitivity value<br>Shapiro-Wilk score	-2.29E-05<br>-5.30E-01<br>3.94E-01<br>9.32E-01<br>-1.83E-04<br>2.395E-02	-1.21E-01<br>1.91E-01	-2.29E-05<br>-2.25E+00<br>4.35E+00<br>9.95E-01<br>-2.22E-03<br>9.596E-03	-1.13E-01<br>1.03E-03
56	Values[kacdh’]	Local<br>Min<br>Max<br>Normalized peak height<br>Peak sensitivity value<br>Shapiro-Wilk score	-1.06E-04<br>-5.79E-01<br>6.48E-01<br>6.94E-01<br>5.28E-04<br>2.179E-02	-2.26E-01<br>4.32E-04	-1.06E-04<br>-1.24E+00<br>4.79E+00<br>9.95E-01<br>-6.48E-04<br>8.591E-03	-6.61E-02<br>6.93E-04
57	Values[kaiep]	Local<br>Min<br>Max<br>Normalized peak height<br>Peak sensitivity value<br>Shapiro-Wilk score	-1.49E-04<br>-5.31E-01<br>5.33E-01<br>6.66E-01<br>-5.57E-04<br>2.550E-02	-3.48E-01<br>9.24E-04	-1.49E-04<br>-1.97E+00<br>2.79E+00<br>9.83E-01<br>1.18E-03<br>1.540E-02	-7.73E+00<br>1.96E-01
58	Values[kamcm]	Local<br>Min<br>Max<br>Normalized peak height<br>Peak sensitivity value<br>Shapiro-Wilk score	-3.31E-04<br>-5.31E-01<br>3.93E-01<br>6.24E-01<br>-4.95E-04<br>3.122E-02	-8.33E-02<br>7.42E-02	-3.31E-04<br>-2.45E+00<br>1.95E+00<br>7.08E-01<br>1.55E-03<br>1.864E-02	-3.01E+00<br>4.60E-03
59	Values[kasb2]	Local<br>Min<br>Max<br>Normalized peak height<br>Peak sensitivity value<br>Shapiro-Wilk score	-5.17E-05<br>-5.30E-01<br>3.67E-01<br>7.66E-01<br>3.28E-04<br>2.323E-02	-2.02E-01<br>5.93E-04	-5.17E-05<br>-2.86E+00<br>4.42E+00<br>9.93E-01<br>2.94E-03<br>8.717E-03	-1.25E-01<br>9.90E-04
60	Values[kasb5]	Local<br>Min<br>Max<br>Normalized peak height<br>Peak sensitivity value<br>Shapiro-Wilk score	-1.71E-04<br>-5.31E-01<br>5.39E-01<br>8.49E-01<br>3.29E-04<br>2.182E-02	-7.72E-04<br>6.49E-04	-1.71E-04<br>-1.24E+00<br>1.95E+00<br>9.81E-01<br>1.08E-03<br>1.315E-02	-1.19E-01<br>6.36E-02
61	Values[kasbf]	Local<br>Min<br>Max<br>Normalized peak height<br>Peak sensitivity value<br>Shapiro-Wilk score	-2.34E-04<br>-5.29E-01<br>8.52E-01<br>6.73E-01<br>-5.88E-04<br>3.553E-02	-2.75E-02<br>8.71E-01	-2.34E-04<br>-2.26E+00<br>8.28E+00<br>9.89E-01<br>1.59E-03<br>9.907E-03	-2.70E-01<br>7.60E-01
62	Values[kasesp]	Local<br>Min<br>Max<br>Normalized peak height<br>Peak sensitivity value<br>Shapiro-Wilk score	1.92E-04<br>-5.30E-01<br>7.55E-01<br>9.91E-01<br>-1.09E-05<br>2.029E-02	-2.53E-02<br>1.71E-02	1.92E-04<br>-1.94E+00<br>7.86E+00<br>9.96E-01<br>-2.89E-03<br>5.071E-03	-1.37E-01<br>7.42E+00
63	Values[kasf2]	Local<br>Min<br>Max<br>Normalized peak height<br>Peak sensitivity value<br>Shapiro-Wilk score	1.47E-05<br>-5.30E-01<br>4.50E-01<br>7.95E-01<br>-3.01E-04<br>2.349E-02	-6.99E-04<br>4.82E-02	1.47E-05<br>-1.85E+00<br>1.95E+00<br>9.94E-01<br>-2.69E-04<br>1.230E-02	-5.47E-02<br>6.19E-02
64	Values[kasf5]	Local<br>Min<br>Max<br>Normalized peak height<br>Peak sensitivity value<br>Shapiro-Wilk score	-3.31E-04<br>-5.30E-01<br>4.29E-01<br>6.80E-01<br>-4.06E-04<br>2.311E-02	-2.17E-03<br>6.34E-04	-3.31E-04<br>-1.45E+00<br>1.95E+00<br>9.94E-01<br>7.87E-04<br>1.409E-02	-7.16E-04<br>1.45E-01
65	Values[kasrent]	Local<br>Min<br>Max<br>Normalized peak height<br>Peak sensitivity value<br>Shapiro-Wilk score	-8.15E-05<br>-5.45E-01<br>8.56E-01<br>9.78E-01<br>2.35E-04<br>1.988E-02	-2.14E-01<br>8.91E-04	-8.15E-05<br>-1.24E+00<br>4.78E+00<br>9.95E-01<br>1.28E-03<br>8.809E-03	-4.33E-01<br>9.83E-04
66	Values[kasrentp]	Local<br>Min<br>Max<br>Normalized peak height<br>Peak sensitivity value<br>Shapiro-Wilk score	2.06E-04<br>-5.30E-01<br>3.33E-01<br>6.73E-01<br>-3.47E-04<br>2.247E-02	-5.51E-02<br>5.74E-04	2.06E-04<br>-1.24E+00<br>3.31E+00<br>9.94E-01<br>-1.17E-03<br>1.128E-02	-2.87E+00<br>7.38E-02
67	Values[kaswi]	Local<br>Min<br>Max<br>Normalized peak height<br>Peak sensitivity value<br>Shapiro-Wilk score	6.85E-05<br>-5.30E-01<br>5.47E-01<br>9.69E-01<br>1.32E-04<br>2.171E-02	-1.86E-03<br>7.08E-04	6.85E-05<br>-1.67E+00<br>2.20E+00<br>9.94E-01<br>-4.68E-04<br>1.172E-02	-1.77E-01<br>3.30E-01
68	Values[kd14]	Local<br>Min<br>Max<br>Normalized peak height<br>Peak sensitivity value<br>Shapiro-Wilk score	-1.22E-04<br>-5.32E-01<br>7.50E-01<br>8.09E-01<br>-3.51E-04<br>2.312E-02	-2.45E-01<br>2.45E-01	-1.22E-04<br>-3.28E+00<br>4.31E+00<br>7.71E-01<br>-3.46E-03<br>2.636E-02	-3.55E-01<br>2.05E+00

69	Values[kd1c1]	Local	-8.44E-05		-8.44E-05	
		Min	-5.30E-01	-2.11E-02	-1.24E+00	-2.41E-01
		Max	3.37E-01	8.29E-04	1.95E+00	5.16E-02
		Normalized peak height	7.90E-01		9.86E-01	
		Peak sensitivity value	2.76E-04		1.03E-03	
		Shapiro-Wilk score	2.291E-02		1.474E-02	
70	Values[kd1f6]	Local	-9.53E-05		-9.53E-05	
		Min	-5.30E-01	-6.63E-04	-1.48E+00	-2.96E-01
		Max	3.83E-01	1.88E-02	5.54E+00	3.10E-02
		Normalized peak height	8.42E-01		9.95E-01	
		Peak sensitivity value	-2.66E-04		6.65E-04	
		Shapiro-Wilk score	2.395E-02		8.057E-03	
71	Values[kd1pds']	Local	-2.26E-05		-2.26E-05	
		Min	-5.31E-01	-1.39E-01	-2.91E+00	-1.37E-01
		Max	6.04E-01	4.50E-04	1.95E+00	6.95E-02
		Normalized peak height	8.08E-01		9.95E-01	
		Peak sensitivity value	-4.41E-04		3.13E-04	
		Shapiro-Wilk score	2.233E-02		1.366E-02	
72	Values[kd20]	Local	1.43E-04		1.43E-04	
		Min	-5.30E-01	-2.69E-01	-1.46E+00	-5.04E+01
		Max	5.31E-01	1.08E-01	1.95E+00	6.45E+00
		Normalized peak height	8.92E-01		8.24E-01	
		Peak sensitivity value	1.49E-04		1.65E-03	
		Shapiro-Wilk score	2.968E-02		2.004E-02	
73	Values[kd2c1]	Local	-2.07E-04		-2.07E-04	
		Min	-5.31E-01	-1.87E+00	-2.67E+00	-5.34E-01
		Max	2.92E-01	1.87E-01	6.77E+00	6.03E-01
		Normalized peak height	5.59E-01		9.11E-01	
		Peak sensitivity value	3.18E-04		3.93E-03	
		Shapiro-Wilk score	2.496E-02		8.875E-03	
74	Values[kd2f6]	Local	-1.27E-04		-1.27E-04	
		Min	-5.30E-01	-6.42E-01	-3.58E+00	-1.14E+00
		Max	6.94E-01	6.22E-04	5.26E+00	2.08E-01
		Normalized peak height	9.12E-01		5.92E-01	
		Peak sensitivity value	2.19E-04		4.22E-03	
		Shapiro-Wilk score	2.332E-02		9.665E-03	
75	Values[kd2pds”]	Local	-2.47E-05		-2.47E-05	
		Min	-5.30E-01	-1.01E-03	-2.71E+00	-1.33E-03
		Max	3.60E-01	4.88E-04	3.23E+00	1.85E+00
		Normalized peak height	8.89E-01		9.94E-01	
		Peak sensitivity value	-2.72E-04		8.77E-04	
		Shapiro-Wilk score	2.501E-02		1.042E-02	
76	Values[kd3c1]	Local	-1.08E-04		-1.08E-04	
		Min	-5.31E-01	-9.09E-02	-1.24E+00	-1.99E+00
		Max	6.18E-01	1.46E-01	5.87E+00	3.38E-01
		Normalized peak height	8.08E-01		9.89E-01	
		Peak sensitivity value	-4.36E-04		-7.57E-04	
		Shapiro-Wilk score	2.239E-02		7.941E-03	
77	Values[kd3f6]	Local	-7.06E-05		-7.06E-05	
		Min	-5.30E-01	-8.03E-02	-1.24E+00	-1.63E+00
		Max	3.52E-01	1.40E-01	2.82E+00	5.60E-04
		Normalized peak height	7.35E-01		9.75E-01	
		Peak sensitivity value	2.54E-04		7.93E-04	
		Shapiro-Wilk score	2.295E-02		1.235E-02	
78	Values[kd3pds”]	Local	-1.09E-04		-1.09E-04	
		Min	-5.30E-01	-2.75E-03	-1.38E+00	-9.11E-04
		Max	6.15E-01	6.25E-02	1.95E+00	7.20E-04
		Normalized peak height	8.83E-01		9.94E-01	
		Peak sensitivity value	-3.45E-04		-4.28E-04	
		Shapiro-Wilk score	2.201E-02		1.614E-02	
79	Values[kdb2”]	Local	-1.57E-04		-1.57E-04	
		Min	-5.30E-01	-6.41E-03	-1.51E+00	-1.66E+00
		Max	4.81E-01	7.82E-04	4.73E+00	2.86E-01
		Normalized peak height	9.78E-01		9.94E-01	
		Peak sensitivity value	-3.40E-05		1.17E-03	
		Shapiro-Wilk score	2.258E-02		8.442E-03	
80	Values[kdb2']	Local	-1.95E-04		-1.95E-04	
		Min	-5.30E-01	-7.27E-04	-1.28E+00	-7.92E-04
		Max	8.52E-01	2.04E-03	5.04E+00	6.74E-02
		Normalized peak height	9.87E-01		9.94E-01	
		Peak sensitivity value	-1.73E-04		2.31E-03	
		Shapiro-Wilk score	2.025E-02		7.971E-03	
81	Values[kdb2p]	Local	-5.91E-05		-5.91E-05	
		Min	-5.30E-01	-2.61E-02	-1.24E+00	-6.05E+00
		Max	3.47E-01	2.77E-02	7.28E+00	3.13E-02
		Normalized peak height	7.55E-01		9.95E-01	
		Peak sensitivity value	-3.32E-04		-1.03E-03	
		Shapiro-Wilk score	2.359E-02		5.184E-03	
82	Values[kdb5”]	Local	-1.59E-04		-1.59E-04	
		Min	-5.30E-01	-5.71E-04	-1.76E+00	-6.60E-04
		Max	3.69E-01	3.76E-02	4.27E+00	4.81E-02
		Normalized peak height	9.18E-01		8.11E-01	
		Peak sensitivity value	-1.57E-04		-2.81E-03	
		Shapiro-Wilk score	2.283E-02		9.259E-03	

83	Values[kdb5']				
	Local	1.35E-05		1.35E-05	
	Min	-5.30E-01	-2.27E-03	-1.92E+00	-7.33E-02
	Max	3.13E-01	1.28E-01	6.23E+00	5.41E-01
	Normalized peak height	8.33E-01		9.95E-01	
	Peak sensitivity value	-2.25E-04		-8.04E-04	
	Shapiro-Wilk score	2.430E-02		7.095E-03	
84	Values[kdbud]				
	Local	0.00E+00		0.00E+00	
	Min	-3.40E-04	0.00E+00	-4.97E-01	-7.54E-01
	Max	4.96E-04	0.00E+00	6.44E-01	6.26E-03
	Normalized peak height	1.00E+00		9.99E-01	
	Peak sensitivity value	-2.53E-07		1.52E-04	
	Shapiro-Wilk score	3.352E-03		1.369E-03	
85	Values[kdcdh]				
	Local	-4.34E-04		-4.34E-04	
	Min	-5.31E-01	-2.22E-03	-3.79E+00	-7.42E-01
	Max	5.72E-01	1.74E-01	2.14E+00	1.69E-02
	Normalized peak height	6.21E-01		9.65E-01	
	Peak sensitivity value	3.29E-04		-2.66E-03	
	Shapiro-Wilk score	2.325E-02		1.122E-02	
86	Values[kdib2]				
	Local	-5.02E-05		-5.02E-05	
	Min	-5.30E-01	-6.51E-02	-1.24E+00	-1.19E+00
	Max	4.43E-01	7.63E-04	1.95E+00	5.79E-02
	Normalized peak height	5.38E-01		9.85E-01	
	Peak sensitivity value	4.72E-04		1.06E-03	
	Shapiro-Wilk score	2.198E-02		1.343E-02	
87	Values[kdib5]				
	Local	-9.54E-05		-9.54E-05	
	Min	-5.31E-01	-3.19E-02	-1.24E+00	-1.41E-02
	Max	4.84E-01	5.30E-04	6.34E+00	1.31E-01
	Normalized peak height	8.50E-01		9.96E-01	
	Peak sensitivity value	-3.10E-04		-1.26E-03	
	Shapiro-Wilk score	2.254E-02		6.940E-03	
88	Values[kdiesp]				
	Local	2.32E-05		2.32E-05	
	Min	-5.30E-01	-1.00E-01	-1.42E+00	-3.83E+00
	Max	4.94E-01	1.07E-02	1.95E+00	5.30E-02
	Normalized peak height	9.43E-01		9.94E-01	
	Peak sensitivity value	-1.74E-04		4.19E-04	
	Shapiro-Wilk score	2.210E-02		1.302E-02	
89	Values[kdif2]				
	Local	-1.86E-04		-1.86E-04	
	Min	-5.30E-01	-5.62E-03	-1.24E+00	-7.91E-04
	Max	5.14E-01	7.62E-04	2.43E+00	7.59E-02
	Normalized peak height	9.36E-01		5.93E-01	
	Peak sensitivity value	-2.19E-04		-1.81E-03	
	Shapiro-Wilk score	2.074E-02		1.149E-02	
90	Values[kdif5]				
	Local	2.78E-04		2.78E-04	
	Min	-5.30E-01	-1.48E-01	-2.67E+00	-2.52E-02
	Max	4.12E-01	1.44E-03	1.95E+00	8.69E-04
	Normalized peak height	5.26E-01		9.61E-01	
	Peak sensitivity value	-4.61E-04		-1.92E-03	
	Shapiro-Wilk score	2.294E-02		1.164E-02	
91	Values[kdirent]				
	Local	-9.59E-05		-9.59E-05	
	Min	-6.28E-01	-1.55E-03	-2.12E+00	-7.73E-04
	Max	4.64E-01	9.13E-04	1.95E+00	5.39E-02
	Normalized peak height	9.26E-01		9.94E-01	
	Peak sensitivity value	2.62E-04		1.04E-03	
	Shapiro-Wilk score	2.239E-02		1.304E-02	
92	Values[kdirentp]				
	Local	2.09E-05		2.09E-05	
	Min	-5.30E-01	-2.21E-02	-1.35E+00	-3.33E-01
	Max	5.64E-01	2.74E-03	4.22E+00	2.96E+00
	Normalized peak height	9.83E-01		8.95E-01	
	Peak sensitivity value	-7.13E-05		2.45E-03	
	Shapiro-Wilk score	2.081E-02		1.023E-02	
93	Values[kdn2]				
	Local	1.43E-04		1.43E-04	
	Min	-5.30E-01	-1.48E-01	-1.75E+00	-6.79E+00
	Max	5.28E-01	1.61E+00	1.95E+00	8.15E-01
	Normalized peak height	7.52E-01		8.38E-01	
	Peak sensitivity value	5.15E-04		-1.15E-03	
	Shapiro-Wilk score	2.427E-02		2.066E-02	
94	Values[kdnet]				
	Local	1.86E-04		1.86E-04	
	Min	-5.28E-01	-5.57E-04	-3.02E+00	-1.01E+00
	Max	3.56E-01	7.23E-01	3.84E+00	4.12E-01
	Normalized peak height	6.11E-01		9.37E-01	
	Peak sensitivity value	3.85E-04		1.70E-03	
	Shapiro-Wilk score	2.885E-02		2.639E-02	
95	Values[kdori]				
	Local	1.30E-05		1.30E-05	
	Min	-5.30E-01	-6.17E-04	-1.99E+00	-4.35E+00
	Max	4.35E-01	9.48E-04	1.95E+00	9.49E-02
	Normalized peak height	9.73E-01		9.94E-01	
	Peak sensitivity value	1.21E-05		-5.72E-04	
	Shapiro-Wilk score	2.269E-02		1.332E-02	
96	Values[kdppx"]				
	Local	-2.19E-04		-2.19E-04	
	Min	-5.30E-01	-2.25E-02	-1.24E+00	-8.30E-04
	Max	5.07E-01	2.29E-01	1.95E+00	6.79E+00
	Normalized peak height	8.66E-01		9.65E-01	
	Peak sensitivity value	2.62E-04		1.10E-03	
	Shapiro-Wilk score	2.337E-02		1.901E-02	

97	Values[kdppx']				
	Local	-2.23E-04		-2.23E-04	
	Min	-5.30E-01	-6.58E-04	-1.59E+00	-2.40E-02
	Max	5.36E-01	3.78E-02	1.95E+00	3.22E+00
	Normalized peak height	7.97E-01		9.86E-01	
	Peak sensitivity value	3.34E-04		-7.91E-04	
	Shapiro-Wilk score	2.297E-02		1.474E-02	
98	Values[kdspn]				
	Local	5.82E-05		5.82E-05	
	Min	-5.29E-01	-7.02E-04	-1.75E+00	-2.57E-03
	Max	3.42E-01	3.44E+00	1.95E+00	6.48E+00
	Normalized peak height	8.84E-01		9.38E-01	
	Peak sensitivity value	2.20E-04		1.41E-04	
	Shapiro-Wilk score	2.757E-02		2.593E-02	
99	Values[kdswi]				
	Local	9.43E-05		9.43E-05	
	Min	-5.31E-01	-3.68E+00	-1.24E+00	-1.63E+00
	Max	5.21E-01	7.37E-03	1.95E+00	3.63E+01
	Normalized peak height	5.42E-01		9.25E-01	
	Peak sensitivity value	-6.41E-04		-3.47E-04	
	Shapiro-Wilk score	2.959E-02		2.894E-02	
100	Values[ki15]				
	Local	2.22E-05		2.22E-05	
	Min	-5.30E-01	-4.61E-01	-1.32E+00	-9.02E-04
	Max	4.84E-01	1.46E-01	1.95E+00	1.03E-01
	Normalized peak height	7.70E-01		9.80E-01	
	Peak sensitivity value	-3.61E-04		-1.03E-03	
	Shapiro-Wilk score	2.356E-02		1.682E-02	
101	Values[kicdh"]				
	Local	7.74E-05		7.74E-05	
	Min	-5.30E-01	-4.69E-02	-1.34E+00	-2.90E-01
	Max	5.32E-01	1.05E-02	6.67E+00	6.11E-01
	Normalized peak height	6.97E-01		9.93E-01	
	Peak sensitivity value	-4.48E-04		-2.20E-03	
	Shapiro-Wilk score	2.408E-02		6.111E-03	
102	Values[kicdh']				
	Local	5.98E-05		5.98E-05	
	Min	-5.31E-01	-2.45E-01	-2.28E+00	-1.85E+00
	Max	4.88E-01	7.56E-04	6.55E+00	2.09E-02
	Normalized peak height	5.07E-01		9.96E-01	
	Peak sensitivity value	5.06E-04		2.02E-03	
	Shapiro-Wilk score	2.285E-02		6.827E-03	
103	Values[kiiep]				
	Local	1.52E-04		1.52E-04	
	Min	-5.30E-01	-1.09E-01	-1.24E+00	-3.55E+00
	Max	4.37E-01	1.71E-01	3.88E+00	2.21E-01
	Normalized peak height	9.02E-01		9.92E-01	
	Peak sensitivity value	2.93E-04		1.89E-03	
	Shapiro-Wilk score	2.589E-02		9.396E-03	
104	Values[kimcm]				
	Local	9.37E-05		9.37E-05	
	Min	-5.29E-01	-2.28E-01	-2.36E+00	-2.79E+01
	Max	7.33E-01	3.32E+00	2.25E+00	1.91E+00
	Normalized peak height	9.22E-01		7.11E-01	
	Peak sensitivity value	3.56E-04		-1.65E-03	
	Shapiro-Wilk score	2.773E-02		1.995E-02	
105	Values[kisbf"]				
	Local	-1.03E-05		-1.03E-05	
	Min	-5.30E-01	-1.47E-01	-3.08E+00	-6.96E-04
	Max	3.25E-01	3.00E-03	7.69E+00	1.66E-02
	Normalized peak height	7.83E-01		9.94E-01	
	Peak sensitivity value	-2.79E-04		-4.55E-03	
	Shapiro-Wilk score	2.525E-02		5.743E-03	
106	Values[kisbf']				
	Local	2.33E-04		2.33E-04	
	Min	-5.32E-01	-7.73E-01	-2.14E+00	-1.05E+00
	Max	6.75E-01	9.26E-03	3.14E+00	8.48E-01
	Normalized peak height	7.50E-01		8.51E-01	
	Peak sensitivity value	-2.24E-04		1.94E-03	
	Shapiro-Wilk score	3.202E-02		2.229E-02	
107	Values[kiswi]				
	Local	1.28E-04		1.28E-04	
	Min	-5.30E-01	-2.86E-01	-2.45E+00	-4.12E-02
	Max	5.57E-01	7.33E-04	2.40E+00	2.06E-02
	Normalized peak height	9.70E-01		9.95E-01	
	Peak sensitivity value	1.30E-04		7.47E-04	
	Shapiro-Wilk score	2.185E-02		1.052E-02	
108	Values[kkpnet"]				
	Local	-1.52E-04		-1.52E-04	
	Min	-5.30E-01	-7.26E-04	-1.24E+00	-1.93E+00
	Max	5.99E-01	2.54E+00	3.75E+00	1.40E+00
	Normalized peak height	9.75E-01		9.85E-01	
	Peak sensitivity value	8.61E-05		-2.04E-04	
	Shapiro-Wilk score	2.255E-02		1.288E-02	
109	Values[kkpnet']				
	Local	-4.87E-04		-4.87E-04	
	Min	-5.49E-01	-6.91E-03	-3.81E+00	-7.34E-01
	Max	3.21E-01	6.41E-02	2.96E+00	1.19E+00
	Normalized peak height	8.77E-01		9.60E-01	
	Peak sensitivity value	2.03E-04		2.99E-03	
	Shapiro-Wilk score	2.268E-02		8.989E-03	
110	Values[kppc1]				
	Local	-7.69E-06		-7.69E-06	
	Min	-5.30E-01	-3.08E-02	-2.13E+00	-8.72E-04
	Max	3.47E-01	2.51E-03	7.45E+00	6.47E+00
	Normalized peak height	9.55E-01		8.08E-01	
	Peak sensitivity value	-3.54E-05		-4.54E-03	
	Shapiro-Wilk score	2.432E-02		5.643E-03	

111	Values[kppf6]	Local	1.53E-05		1.53E-05	
		Min	-5.30E-01	-4.53E-01	-2.33E+00	-1.11E-03
		Max	3.56E-01	3.84E-01	8.72E+00	6.22E+00
		Normalized peak height	7.65E-01		9.96E-01	
		Peak sensitivity value	3.12E-04		-1.03E-03	
		Shapiro-Wilk score	2.388E-02		5.328E-03	
112	Values[kppnet”]	Local	-1.07E-04		-1.07E-04	
		Min	-5.31E-01	-7.07E-04	-2.98E+00	-1.12E+02
		Max	4.10E-01	7.96E-04	4.63E+00	5.84E-01
		Normalized peak height	8.83E-01		9.69E-01	
		Peak sensitivity value	2.72E-04		2.60E-03	
		Shapiro-Wilk score	2.199E-02		9.534E-03	
113	Values[kppnet’]	Local	-1.10E-04		-1.10E-04	
		Min	-5.30E-01	-2.33E-02	-1.85E+00	-8.84E-01
		Max	5.49E-01	9.26E-03	1.04E+01	7.36E-04
		Normalized peak height	8.36E-01		9.96E-01	
		Peak sensitivity value	3.54E-04		-2.15E-03	
		Shapiro-Wilk score	2.210E-02		3.712E-03	
114	Values[ks14]	Local	-9.16E-05		-9.16E-05	
		Min	-5.29E-01	-2.40E-01	-2.31E+00	-2.90E+00
		Max	4.17E-01	6.46E-01	5.25E+00	4.61E-01
		Normalized peak height	6.34E-01		8.88E-01	
		Peak sensitivity value	3.20E-04		-2.77E-03	
		Shapiro-Wilk score	2.764E-02		2.229E-02	
115	Values[ks1pds”]	Local	1.72E-04		1.72E-04	
		Min	-5.30E-01	-2.45E-03	-2.81E+00	-1.95E-03
		Max	5.54E-01	5.00E-02	1.95E+00	2.23E-01
		Normalized peak height	7.58E-01		9.94E-01	
		Peak sensitivity value	-3.54E-04		-8.05E-04	
		Shapiro-Wilk score	2.192E-02		1.331E-02	
116	Values[ks20”]	Local	2.99E-05		2.99E-05	
		Min	-5.31E-01	-1.49E-02	-1.89E+00	-1.87E+00
		Max	3.41E-01	1.44E-01	1.95E+00	2.77E+00
		Normalized peak height	9.01E-01		9.90E-01	
		Peak sensitivity value	-2.16E-04		4.40E-04	
		Shapiro-Wilk score	2.733E-02		1.605E-02	
117	Values[ks20’]	Local	-9.05E-05		-9.05E-05	
		Min	-5.34E-01	-8.51E-04	-1.39E+00	-6.49E-04
		Max	6.73E-01	1.74E-01	2.40E+00	9.62E-01
		Normalized peak height	9.72E-01		9.94E-01	
		Peak sensitivity value	-1.92E-04		6.70E-04	
		Shapiro-Wilk score	2.262E-02		1.177E-02	
118	Values[ks2pds”]	Local	1.87E-05		1.87E-05	
		Min	-5.30E-01	-8.67E-04	-1.52E+00	-1.09E+01
		Max	3.25E-01	1.02E-03	4.69E+00	6.75E-01
		Normalized peak height	5.59E-01		9.94E-01	
		Peak sensitivity value	-3.06E-04		-9.60E-04	
		Shapiro-Wilk score	2.530E-02		9.439E-03	
119	Values[ksb2”]	Local	-7.18E-05		-7.18E-05	
		Min	-5.31E-01	-1.34E-01	-1.45E+00	-2.58E-01
		Max	4.50E-01	1.43E-02	5.24E+00	1.29E-01
		Normalized peak height	6.85E-01		9.88E-01	
		Peak sensitivity value	1.41E-04		9.34E-04	
		Shapiro-Wilk score	2.992E-02		1.176E-02	
120	Values[ksb2’]	Local	-2.51E-05		-2.51E-05	
		Min	-5.31E-01	-1.23E-01	-2.54E+00	-2.17E+00
		Max	3.46E-01	7.17E-04	2.81E+00	1.91E-01
		Normalized peak height	6.31E-01		9.92E-01	
		Peak sensitivity value	2.35E-04		-1.87E-03	
		Shapiro-Wilk score	2.850E-02		1.589E-02	
121	Values[ksb5”]	Local	-5.74E-05		-5.74E-05	
		Min	-5.30E-01	-1.14E-01	-1.24E+00	-6.74E-03
		Max	4.84E-01	5.74E-04	7.72E+00	8.24E-04
		Normalized peak height	7.86E-01		9.95E-01	
		Peak sensitivity value	-3.85E-04		2.49E-04	
		Shapiro-Wilk score	2.268E-02		6.334E-03	
122	Values[ksb5’]	Local	-3.90E-05		-3.90E-05	
		Min	-5.30E-01	-3.24E-01	-2.36E+00	-2.35E-02
		Max	4.28E-01	5.88E-04	2.07E+00	1.30E-01
		Normalized peak height	8.57E-01		9.94E-01	
		Peak sensitivity value	2.66E-04		-8.71E-04	
		Shapiro-Wilk score	2.391E-02		1.206E-02	
123	Values[ksbud]	Local	0.00E+00		0.00E+00	
		Min	-7.07E-01	-2.06E-02	-1.61E+00	-1.73E-03
		Max	4.41E-01	2.06E-01	3.40E+00	5.26E-01
		Normalized peak height	8.84E-01		9.95E-01	
		Peak sensitivity value	4.50E-04		-1.82E-03	
		Shapiro-Wilk score	1.354E-02		7.322E-03	
124	Values[ksc1”]	Local	-1.19E-04		-1.19E-04	
		Min	-5.38E-01	-1.02E-03	-2.17E+00	-8.77E-03
		Max	4.43E-01	2.97E+00	1.95E+00	2.06E+00
		Normalized peak height	8.81E-01		9.74E-01	
		Peak sensitivity value	2.94E-04		5.49E-04	
		Shapiro-Wilk score	2.448E-02		2.000E-02	

125	Values[ksc1’]					
		Local	2.31E-05		2.31E-05	
		Min	-5.79E-01	-7.70E-04	-1.24E+00	-9.08E-03
		Max	3.33E-01	7.99E-04	9.68E+00	4.09E-01
		Normalized peak height	9.64E-01		9.96E-01	
		Peak sensitivity value	3.32E-05		-8.31E-04	
		Shapiro-Wilk score	2.270E-02		3.942E-03	
126	Values[kscdh]					
		Local	-1.31E-04		-1.31E-04	
		Min	-5.30E-01	-6.25E-04	-3.79E+00	-4.93E-04
		Max	4.46E-01	1.00E-02	4.24E+00	4.63E+00
		Normalized peak height	5.75E-01		9.95E-01	
		Peak sensitivity value	5.92E-04		2.80E-03	
		Shapiro-Wilk score	2.501E-02		8.820E-03	
127	Values[ksf6”’]					
		Local	-2.68E-05		-2.68E-05	
		Min	-5.30E-01	-1.02E-03	-1.28E+00	-1.38E-03
		Max	3.17E-01	1.75E-01	7.38E+00	4.37E-01
		Normalized peak height	9.39E-01		9.96E-01	
		Peak sensitivity value	-8.25E-05		-5.48E-04	
		Shapiro-Wilk score	2.374E-02		5.764E-03	
128	Values[ksf6”]					
		Local	-1.94E-04		-1.94E-04	
		Min	-5.30E-01	-4.18E-02	-2.19E+00	-2.31E+01
		Max	6.68E-01	2.71E-03	7.08E+00	3.55E-01
		Normalized peak height	9.62E-01		9.95E-01	
		Peak sensitivity value	2.49E-04		4.77E-04	
		Shapiro-Wilk score	2.348E-02		4.690E-03	
129	Values[ksf6’]					
		Local	-1.03E-04		-1.03E-04	
		Min	-5.30E-01	-1.75E-02	-2.72E+00	-7.05E-04
		Max	2.87E-01	4.71E-01	2.95E+00	1.96E+00
		Normalized peak height	9.19E-01		9.95E-01	
		Peak sensitivity value	1.42E-04		-1.75E-05	
		Shapiro-Wilk score	2.435E-02		1.140E-02	
130	Values[ksn2”]					
		Local	-1.54E-04		-1.54E-04	
		Min	-5.31E-01	-5.24E-01	-1.60E+00	-7.45E+00
		Max	3.92E-01	9.48E-04	2.61E+00	4.60E-01
		Normalized peak height	6.48E-01		9.62E-01	
		Peak sensitivity value	-4.94E-04		-2.87E-04	
		Shapiro-Wilk score	2.732E-02		2.080E-02	
131	Values[ksn2’]					
		Local	0.00E+00		0.00E+00	
		Min	-0.00E+00	0.00E+00	-0.00E+00	0.00E+00
		Max	-0.00E+00	0.00E+00	-0.00E+00	0.00E+00
		Normalized peak height	1.00E+00		1.00E+00	
		Peak sensitivity value	5.00E-04		5.00E-04	
		Shapiro-Wilk score	1.000E+00		1.000E+00	
132	Values[ksnet]					
		Local	-1.15E-04		-1.15E-04	
		Min	-5.32E-01	-9.21E-01	-3.96E+00	-2.89E+00
		Max	6.86E-01	1.26E-01	2.20E+00	9.05E-02
		Normalized peak height	6.38E-01		9.30E-01	
		Peak sensitivity value	5.19E-04		-1.94E-03	
		Shapiro-Wilk score	2.507E-02		3.358E-02	
133	Values[ksori]					
		Local	-4.16E-05		-4.16E-05	
		Min	-5.30E-01	-6.27E-04	-1.24E+00	-3.54E+00
		Max	3.98E-01	1.85E-03	1.95E+00	6.53E+01
		Normalized peak height	8.56E-01		9.93E-01	
		Peak sensitivity value	2.54E-04		6.80E-04	
		Shapiro-Wilk score	2.480E-02		1.493E-02	
134	Values[kspds’]					
		Local	0.00E+00		0.00E+00	
		Min	0.00E+00	0.00E+00	-0.00E+00	0.00E+00
		Max	0.00E+00	0.00E+00	-0.00E+00	0.00E+00
		Normalized peak height	1.00E+00		1.00E+00	
		Peak sensitivity value	5.00E-04		5.00E-04	
		Shapiro-Wilk score	1.000E+00		1.000E+00	
135	Values[ksppx]					
		Local	1.69E-05		1.69E-05	
		Min	-5.30E-01	-2.33E-01	-1.24E+00	-1.70E-01
		Max	2.48E-01	1.36E-01	4.97E+00	7.43E-01
		Normalized peak height	8.08E-01		9.86E-01	
		Peak sensitivity value	2.61E-04		-2.00E-03	
		Shapiro-Wilk score	2.394E-02		1.086E-02	
136	Values[ksspn]					
		Local	-1.25E-04		-1.25E-04	
		Min	-5.32E-01	-2.42E+00	-1.39E+00	-5.22E+00
		Max	8.01E-01	2.17E-01	5.42E+00	8.51E-04
		Normalized peak height	6.53E-01		6.18E-01	
		Peak sensitivity value	3.93E-04		2.97E-03	
		Shapiro-Wilk score	2.757E-02		2.217E-02	
137	Values[ksswi”]					
		Local	7.37E-06		7.37E-06	
		Min	-5.29E-01	-1.46E-01	-2.00E+00	-1.45E+02
		Max	3.67E-01	6.14E-01	3.96E+00	7.66E+00
		Normalized peak height	8.37E-01		9.67E-01	
		Peak sensitivity value	-4.81E-05		-4.31E-04	
		Shapiro-Wilk score	2.859E-02		1.437E-02	
138	Values[ksswi’]					
		Local	-3.65E-05		-3.65E-05	
		Min	-5.30E-01	-2.18E-01	-1.46E+00	-1.01E+01
		Max	8.71E-01	8.66E-04	3.51E+00	3.52E+00
		Normalized peak height	9.91E-01		9.94E-01	
		Peak sensitivity value	1.46E-04		6.48E-04	
		Shapiro-Wilk score	2.097E-02		1.141E-02	

139	Values[lte1h]<br>Local<br>Min<br>Max<br>Normalized peak height<br>Peak sensitivity value<br>Shapiro-Wilk score	 1.54E-04<br>-5.31E-01<br>4.01E-01<br>8.10E-01<br>-3.08E-04<br>2.389E-02	 <br>-7.82E-03<br>8.33E-04	 1.54E-04<br>-1.24E+00<br>4.53E+00<br>9.95E-01<br>1.71E-03<br>8.686E-03	 <br>-4.92E-01<br>3.16E-01
140	Values[lte1l]<br>Local<br>Min<br>Max<br>Normalized peak height<br>Peak sensitivity value<br>Shapiro-Wilk score	 -4.32E-05<br>-5.29E-01<br>2.91E-01<br>9.18E-01<br>-1.46E-05<br>2.634E-02	 <br>-7.17E-04<br>3.54E-02	 -4.32E-05<br>-1.24E+00<br>1.95E+00<br>8.80E-01<br>-9.62E-04<br>2.431E-02	 <br>-1.05E-02<br>4.78E-01
141	Values[mad2h]<br>Local<br>Min<br>Max<br>Normalized peak height<br>Peak sensitivity value<br>Shapiro-Wilk score	 -3.85E-04<br>-5.30E-01<br>2.81E-01<br>9.45E-01<br>2.42E-05<br>2.374E-02	 <br>-6.31E-04<br>2.25E-01	 -3.85E-04<br>-2.82E+00<br>4.20E+00<br>9.94E-01<br>2.66E-03<br>7.722E-03	 <br>-1.37E-01<br>5.21E-01
142	Values[mad2l]<br>Local<br>Min<br>Max<br>Normalized peak height<br>Peak sensitivity value<br>Shapiro-Wilk score	 4.69E-05<br>-5.31E-01<br>3.36E-01<br>7.33E-01<br>3.27E-04<br>2.497E-02	 <br>-4.65E-02<br>5.38E-04	 4.69E-05<br>-1.47E+00<br>4.27E+00<br>9.95E-01<br>1.49E-03<br>9.193E-03	 <br>-1.10E-03<br>7.43E-04
