# Supplementary material for: What Can We Learn from Global Sensitivity Analysis of Biochemical Systems?
Source: PLoS One. 2013 Nov 14;8(11):e79244. doi: 10.1371/journal.pone.0079244 (PMC3828278; doi:10.1371/journal.pone.0079244)
Supplement: File S2 — Tables S4—S17. The full outputs of the global sensitivity analyses on the Trypanosoma brucei model using the random-sampling approach. (PDF) [file pone.0079244.s002.pdf]

Supplementary information (Tables S4 – S17)

Below we present the full outputs of the global sensitivity analyses on the *Trypanosoma brucei* model using the random-sampling approach. The results of random sampling are presented as distributions for each sensitivity – distribution x axes show the sensitivity coefficient values, and the y axes show the frequency these values occurred during sampling. The initial sensitivity value is shown as a dashed blue line, zero is shown as a dotted purple line.

Table S4: Concentration control coefficients for *Trypanosoma brucei* model with parameter variation of ±5%

	(glucosetransport)	(hexokinase)	(phosphoglycerateisomerase)	(phosphofructokinase)	(aldolase)	(triosephosphateisomerase)	(glyceraldehyde3phosphatedehydrogenase)	(glycerol3phosphatedehydrogenase)	(glycerol3phosphateoxidase)	(pyruvatetransport)	(phosphoglyceratekinase)
**adpg**											
Local	7.11E+00	5.06E-02	-3.17E-03	-2.57E-02	-5.67E-01	-9.47E-02	-2.37E+00	-1.39E+00	1.77E-01	7.55E-16	-1.91E-01
Min	2.82E+00	3.31E-02	-4.74E-03	-3.87E-02	-7.92E-01	-1.36E-01	-3.39E+00	-1.92E+00	-4.88E-02	-8.70E-15	-3.56E-01
Max	8.58E+00	6.81E-02	-1.31E-03	-1.12E-02	-2.97E-01	-3.06E-02	-1.11E+00	-6.56E-01	3.50E-01	7.64E-15	-5.38E-02
Normalized peak height	5.68E-03	3.52E-03	4.14E-03	4.15E-03	4.36E-03	4.10E-03	3.96E-03	3.80E-03	2.02E-03	1.44E-01	3.02E-03
Peak sensitivity value	7.14E+00	4.92E-02	-3.08E-03	-2.48E-02	-5.44E-01	-9.29E-02	-2.26E+00	-1.32E+00	1.80E-01	1.32E-18	-2.01E-01
Shapiro-Wilk score	9.340E-01	9.997E-01	9.965E-01	9.974E-01	9.977E-01	9.941E-01	9.985E-01	9.995E-01	9.780E-01	9.974E-01	9.962E-01
**adpc**											
Local	-5.14E-01	-3.66E-03	-1.09E-04	-7.90E-04	-1.10E-02	-3.67E-03	-6.52E-02	-3.83E-02	-3.37E-02	0.00E+00	-5.25E-03
Min	-6.60E-01	-4.63E-03	-2.85E-04	-2.08E-03	-3.38E-02	-8.14E-03	-1.83E-01	-1.03E-01	-5.19E-02	-1.14E-13	-8.34E-03
Max	-3.01E-01	-2.85E-03	-4.40E-05	-3.00E-04	-2.18E-03	-1.10E-03	-1.60E-02	-9.27E-03	-1.31E-02	1.09E-13	-2.86E-03
Normalized peak height	2.53E-03	3.46E-03	3.11E-03	3.14E-03	3.08E-03	2.69E-03	3.16E-03	3.17E-03	2.31E-03	2.15E-01	2.85E-03
Peak sensitivity value	-5.41E-01	-3.66E-03	-9.59E-05	-6.68E-04	-8.39E-03	-3.28E-03	-5.93E-02	-3.29E-02	-3.56E-02	7.63E-17	-4.96E-03
Shapiro-Wilk score	9.881E-01	9.983E-01	9.709E-01	9.710E-01	9.701E-01	9.869E-01	9.753E-01	9.775E-01	9.891E-01	8.959E-01	9.932E-01
**dihydroxyacetonephosphate**											
Local	-1.12E-01	-8.00E-04	1.87E-03	1.47E-02	2.89E-01	-7.31E-02	-6.54E-02	-3.84E-02	6.36E-02	-2.29E-17	-5.13E-03
Min	-2.42E-01	-1.58E-03	1.41E-03	1.13E-02	2.46E-01	-8.33E-02	-1.95E-01	-1.11E-01	4.04E-02	-2.28E-16	-8.75E-03
Max	1.33E-01	1.76E-03	2.55E-03	1.96E-02	3.37E-01	-6.45E-02	-1.74E-02	-1.06E-02	9.81E-02	2.08E-16	-2.89E-03
Normalized peak height	2.95E-03	4.96E-03	3.50E-03	3.47E-03	3.22E-03	3.18E-03	3.24E-03	3.21E-03	2.80E-03	6.61E-02	2.70E-03
Peak sensitivity value	-1.32E-01	-8.45E-04	1.82E-03	1.44E-02	2.85E-01	-7.31E-02	-5.22E-02	-3.11E-02	6.50E-02	8.56E-20	-4.54E-03
Shapiro-Wilk score	9.847E-01	9.535E-01	9.963E-01	9.974E-01	9.971E-01	9.975E-01	9.683E-01	9.705E-01	9.945E-01	9.920E-01	9.877E-01
**glyceraldehyde3phosphate**											
Local	-1.73E-01	-1.23E-03	1.89E-03	1.49E-02	2.93E-01	6.17E-02	-9.23E-02	-5.41E-02	5.22E-02	-3.55E-17	-7.29E-03
Min	-3.56E-01	-2.36E-03	1.40E-03	1.13E-02	2.51E-01	5.18E-02	-2.61E-01	-1.49E-01	3.24E-02	-2.73E-16	-1.21E-02
Max	1.30E-01	1.56E-03	2.52E-03	1.94E-02	3.42E-01	6.96E-02	-2.32E-02	-1.40E-02	8.37E-02	3.49E-16	-4.19E-03
Normalized peak height	2.75E-03	4.18E-03	3.33E-03	3.63E-03	3.02E-03	3.37E-03	3.12E-03	2.97E-03	3.07E-03	6.61E-02	2.60E-03
Peak sensitivity value	-1.76E-01	-1.45E-03	1.90E-03	1.48E-02	2.91E-01	6.07E-02	-7.35E-02	-4.10E-02	5.05E-02	8.09E-20	-7.67E-03
Shapiro-Wilk score	9.846E-01	9.563E-01	9.964E-01	9.975E-01	9.972E-01	9.992E-01	9.705E-01	9.728E-01	9.933E-01	9.920E-01	9.882E-01
**2phosphoglycerate**											
Local	9.33E-01	6.64E-03	1.97E-04	1.43E-03	1.99E-02	6.66E-03	1.18E-01	6.94E-02	6.11E-02	-8.41E-17	9.52E-03
Min	4.90E-01	5.02E-03	7.73E-05	5.33E-04	3.38E-03	1.93E-03	2.65E-02	1.55E-02	2.41E-02	-1.41E-14	5.34E-03
Max	1.19E+00	8.64E-03	5.59E-04	4.17E-03	6.37E-02	1.49E-02	3.46E-01	2.02E-01	9.49E-02	1.17E-14	1.50E-02
Normalized peak height	2.85E-03	3.54E-03	3.35E-03	3.59E-03	3.17E-03	2.78E-03	3.23E-03	3.11E-03	2.24E-03	1.76E-01	2.55E-03
Peak sensitivity value	9.84E-01	6.57E-03	1.63E-04	1.21E-03	1.45E-02	5.30E-03	9.13E-02	6.21E-02	5.41E-02	9.99E-18	9.90E-03
Shapiro-Wilk score	9.886E-01	9.976E-01	9.699E-01	9.701E-01	9.688E-01	9.857E-01	9.742E-01	9.762E-01	9.882E-01	8.909E-01	9.917E-01
**glucose**											
Local	3.20E+00	-1.04E+00	-1.17E-02	-7.39E-02	-1.69E-01	-2.68E-02	-6.92E-01	-4.06E-01	5.93E-02	2.20E-16	-5.57E-02
Min	1.75E+00	-1.05E+00	-1.76E-02	-1.15E-01	-6.40E-01	-8.09E-02	-2.51E+00	-1.45E+00	2.69E-03	-4.22E-15	-1.08E-01
Max	6.76E+00	-1.02E+00	-8.03E-03	-5.07E-02	-4.43E-02	-6.92E-03	-1.67E-01	-1.01E-01	4.07E-01	3.97E-15	-3.23E-02
Normalized peak height	3.04E-03	7.65E-03	3.18E-03	3.53E-03	3.91E-03	3.56E-03	3.87E-03	3.87E-03	5.20E-03	1.44E-01	3.10E-03
Peak sensitivity value	2.75E+00	-1.04E+00	-1.18E-02	-7.24E-02	-1.21E-01	-2.43E-02	-5.52E-01	-3.11E-01	2.88E-02	-1.83E-18	-5.01E-02
Shapiro-Wilk score	9.738E-01	8.998E-01	9.894E-01	9.890E-01	9.517E-01	9.720E-01	9.544E-01	9.572E-01	8.932E-01	9.777E-01	9.766E-01
**fructose6phosphate**											
Local	1.54E+00	1.10E-02	4.77E-03	-1.07E+00	-1.09E-01	6.21E-03	-1.92E-01	-1.13E-01	1.40E-01	4.79E-17	-1.51E-02
Min	1.13E+00	6.03E-03	3.46E-03	-1.09E+00	-2.13E-01	-5.42E-03	-5.87E-01	-3.40E-01	9.18E-02	-9.50E-16	-2.63E-02
Max	2.32E+00	2.82E-02	6.57E-03	-1.06E+00	-6.67E-02	1.21E-02	-5.30E-02	-3.28E-02	2.19E-01	7.03E-16	-8.62E-03
Normalized peak height	2.71E-03	3.80E-03	3.31E-03	3.69E-03	3.30E-03	3.12E-03	3.14E-03	3.20E-03	2.81E-03	1.21E-01	2.57E-03
Peak sensitivity value	1.45E+00	9.67E-03	4.74E-03	-1.07E+00	-9.79E-02	6.89E-03	-1.59E-01	-8.15E-02	1.40E-01	-5.76E-19	-1.43E-02
Shapiro-Wilk score	9.800E-01	9.510E-01	9.955E-01	9.951E-01	9.706E-01	9.780E-01	9.655E-01	9.677E-01	9.929E-01	9.724E-01	9.852E-01
**3phosphoglycerate**											
Local	3.53E+00	2.51E-02	7.47E-04	5.43E-03	7.54E-02	2.52E-02	4.48E-01	2.63E-01	2.31E-01	2.58E-22	3.60E-02
Min	1.88E+00	1.65E-02	2.54E-04	1.80E-03	1.27E-02	6.92E-03	9.33E-02	5.28E-02	8.19E-02	-1.51E-14	1.67E-02
Max	5.18E+00	4.20E-02	2.10E-03	1.52E-02	2.45E-01	5.90E-02	1.30E+00	7.35E-01	4.39E-01	1.62E-14	6.78E-02
Normalized peak height	4.14E-03	3.53E-03	3.03E-03	2.93E-03	2.97E-03	2.47E-03	2.90E-03	2.76E-03	2.35E-03	3.43E-01	2.72E-03
Peak sensitivity value	3.48E+00	2.44E-02	5.50E-04	4.50E-03	4.92E-02	1.99E-02	3.73E-01	2.08E-01	2.00E-01	3.43E-18	3.39E-02
Shapiro-Wilk score	9.992E-01	9.787E-01	9.656E-01	9.662E-01	9.637E-01	9.795E-01	9.696E-01	9.710E-01	9.873E-01	9.258E-01	9.798E-01
**glycerol**											
Local	9.22E+00	6.57E-02	-2.07E-03	-1.73E-02	-4.20E-01	-1.47E-01	-2.56E+00	-1.50E+00	-1.41E+00	1.05E-15	-2.06E-01
Min	6.46E+00	4.17E-02	-3.63E-03	-2.99E-02	-6.78E-01	-1.98E-01	-3.82E+00	-2.22E+00	-1.90E+00	-8.96E-15	-3.64E-01
Max	1.11E+01	9.98E-02	-9.11E-04	-8.11E-03	-2.28E-01	-9.96E-02	-1.68E+00	-1.01E+00	-7.51E-01	1.15E-14	-9.12E-02
Normalized peak height	4.99E-03	3.24E-03	3.48E-03	3.63E-03	3.34E-03	3.83E-03	3.52E-03	3.47E-03	3.24E-03	9.28E-02	3.14E-03
Peak sensitivity value	9.09E+00	6.67E-02	-1.88E-03	-1.63E-02	-3.95E-01	-1.41E-01	-2.49E+00	-1.43E+00	-1.44E+00	1.02E-17	-1.98E-01
Shapiro-Wilk score	9.947E-01	9.957E-01	9.908E-01	9.915E-01	9.948E-01	9.999E-01	9.911E-01	9.921E-01	9.914E-01	9.965E-01	9.915E-01
**pyruvate**											
Local	5.54E+00	3.94E-02	1.17E-03	8.51E-03	1.18E-01	3.96E-02	7.02E-01	4.12E-01	3.62E-01	-7.85E+00	5.65E-02
Min	2.59E+00	2.09E-02	3.18E-04	2.20E-03	1.69E-02	8.88E-03	1.24E-01	7.47E-02	1.17E-01	-3.33E+01	2.27E-02
Max	2.39E+01	1.85E-01	6.49E-03	4.53E-02	6.57E-01	1.73E-01	3.47E+00	2.02E+00	1.59E+00	-4.70E+00	2.43E-01
Normalized peak height	8.02E-03	7.56E-03	5.38E-03	5.37E-03	4.88E-03	4.28E-03	4.52E-03	4.60E-03	4.83E-03	7.44E-03	5.39E-03
Peak sensitivity value	4.52E+00	3.54E-02	8.64E-04	5.58E-03	7.10E-02	2.66E-02	4.37E-01	2.72E-01	2.96E-01	-6.66E+00	4.82E-02
Shapiro-Wilk score	9.079E-01	8.859E-01	9.009E-01	9.026E-01	9.057E-01	9.176E-01	9.111E-01	9.110E-01	9.177E-01	8.856E-01	9.055E-01

	(pyruvatekinase)	(atputilisation)	(glycerolkinase)	(phosphoglyceratemutase)	(enolase)	(adenylatekinasecytosol)	(adenylatekinaseglycosome)	(3phosphoglyceratetransport)	(gly3pdhapantiporter)	(glyceroltransport)
**adpg**										
Local	-2.21E-01	-4.54E-02	-2.04E-01	-1.87E+00	-3.52E-01	3.47E-18	-1.01E-17	-5.47E-06	2.02E-06	-3.41E-03
Min	-2.99E-01	-6.25E-02	-3.10E-01	-2.92E+00	-4.67E-01	-3.81E-17	-1.18E-14	-1.17E-05	-4.64E-07	-5.02E-03
Max	-5.35E-02	-1.12E-02	-7.19E-02	-5.30E-01	-9.13E-02	9.60E-18	3.14E-16	-8.68E-07	4.83E-06	-1.28E-03
Normalized peak height	2.64E-03	2.62E-03	2.78E-03	3.78E-03	2.97E-03	4.08E-01	5.34E-02	2.77E-03	2.41E-03	3.14E-03
Peak sensitivity value	-2.46E-01	-4.89E-02	-2.34E-01	-1.83E+00	-3.73E-01	-1.60E-20	1.61E-17	-5.23E-06	1.94E-06	-3.58E-03
Shapiro-Wilk score	9.739E-01	9.742E-01	9.756E-01	9.942E-01	9.683E-01	9.489E-01	4.097E-01	9.904E-01	9.897E-01	9.850E-01
**adpc**										
Local	-6.07E-03	7.27E-01	1.54E-02	-5.14E-02	-9.68E-03	9.75E-18	-4.52E-20	-1.50E-07	-3.85E-07	2.58E-04
Min	-8.87E-03	6.99E-01	6.64E-03	-1.01E-01	-1.42E-02	-2.87E-17	-7.48E-17	-2.33E-07	-6.40E-07	1.18E-04
Max	-2.39E-03	7.53E-01	2.27E-02	-1.59E-02	-3.58E-03	2.45E-17	2.20E-18	-7.05E-08	-1.74E-07	3.69E-04
Normalized peak height	2.73E-03	2.53E-03	2.61E-03	2.65E-03	2.54E-03	4.11E-01	7.56E-02	3.48E-03	2.63E-03	3.25E-03
Peak sensitivity value	-6.37E-03	7.26E-01	1.62E-02	-5.21E-02	-1.07E-02	-1.98E-20	9.96E-21	-1.49E-07	-3.93E-07	2.64E-04
Shapiro-Wilk score	9.864E-01	9.935E-01	9.908E-01	9.864E-01	9.864E-01	9.187E-01	3.674E-01	9.968E-01	9.948E-01	9.956E-01
**dihydroxyacetonephosphate**										
Local	-5.94E-03	-1.22E-03	-6.36E-03	-5.03E-02	-9.47E-03	9.33E-20	1.12E-18	-1.47E-07	7.28E-07	-1.06E-04
Min	-9.40E-03	-1.88E-03	-2.94E-02	-1.09E-01	-1.50E-02	-9.65E-19	-3.50E-17	-2.33E-07	6.24E-07	-4.10E-04
Max	-2.36E-03	-4.90E-04	-1.01E-03	-1.61E-02	-3.59E-03	2.87E-19	1.13E-15	-7.90E-08	1.07E-06	-1.68E-05
Normalized peak height	2.56E-03	2.60E-03	4.10E-03	2.65E-03	2.49E-03	4.09E-01	4.58E-02	3.25E-03	3.78E-03	3.32E-03
Peak sensitivity value	-6.23E-03	-1.37E-03	-3.81E-03	-4.16E-02	-1.01E-02	-6.10E-22	3.89E-18	-1.38E-07	7.07E-07	-6.30E-05
Shapiro-Wilk score	9.895E-01	9.899E-01	9.346E-01	9.832E-01	9.889E-01	9.486E-01	4.631E-01	9.965E-01	9.634E-01	9.484E-01
**glyceraldehyde3phosphate**										
Local	-8.44E-03	-1.73E-03	-4.30E-04	-7.14E-02	-1.34E-02	1.32E-19	1.13E-18	-2.09E-07	5.97E-07	-7.19E-06
Min	-1.26E-02	-2.55E-03	-1.99E-02	-1.50E-01	-2.05E-02	-1.20E-18	-3.84E-17	-3.35E-07	5.24E-07	-2.80E-04
Max	-3.43E-03	-7.25E-04	3.30E-03	-2.20E-02	-5.03E-03	3.53E-19	9.71E-16	-1.10E-07	8.58E-07	5.82E-05
Normalized peak height	2.51E-03	2.58E-03	5.50E-03	2.64E-03	2.43E-03	4.14E-01	4.21E-02	3.38E-03	4.55E-03	4.61E-03
Peak sensitivity value	-9.56E-03	-1.90E-03	1.41E-03	-6.57E-02	-1.45E-02	3.10E-22	-1.59E-18	-2.01E-07	6.04E-07	1.58E-05
Shapiro-Wilk score	9.872E-01	9.873E-01	8.897E-01	9.841E-01	9.868E-01	9.483E-01	5.131E-01	9.968E-01	9.692E-01	9.093E-01
**2phosphoglycerate**										
Local	-4.61E-01	-9.48E-02	-2.80E-02	9.32E-02	-7.35E-01	7.25E-18	8.21E-20	2.73E-07	6.99E-07	-4.68E-04
Min	-4.97E-01	-1.03E-01	-4.24E-02	2.74E-02	-8.13E-01	-1.41E-16	-3.70E-18	1.26E-07	3.17E-07	-7.12E-04
Max	-4.30E-01	-8.72E-02	-1.22E-02	1.86E-01	-6.65E-01	1.85E-17	1.25E-16	4.23E-07	1.16E-06	-2.30E-04
Normalized peak height	1.93E-03	3.09E-03	2.52E-03	2.55E-03	2.88E-03	4.13E-01	7.40E-02	3.61E-03	2.73E-03	3.40E-03
Peak sensitivity value	-4.56E-01	-9.49E-02	-3.02E-02	9.61E-02	-7.29E-01	-4.04E-20	-4.90E-20	2.57E-07	6.91E-07	-4.77E-04
Shapiro-Wilk score	9.760E-01	9.990E-01	9.904E-01	9.862E-01	9.968E-01	9.055E-01	3.675E-01	9.969E-01	9.941E-01	9.957E-01
**glucose**										
Local	-6.44E-02	-1.33E-02	-5.89E-02	-5.45E-01	-1.03E-01	1.01E-18	-2.56E-19	-1.60E-06	6.79E-07	-9.85E-04
Min	-1.15E-01	-2.30E-02	-3.11E-01	-1.29E+00	-1.91E-01	-1.67E-17	-5.69E-16	-2.79E-06	2.84E-08	-4.71E-03
Max	-2.72E-02	-5.62E-03	-7.06E-03	-1.77E-01	-4.07E-02	3.05E-18	2.40E-17	-7.44E-07	5.96E-06	-1.31E-04
Normalized peak height	2.66E-03	2.69E-03	4.26E-03	2.94E-03	2.70E-03	4.12E-01	1.77E-01	3.77E-03	6.71E-03	4.12E-03
Peak sensitivity value	-6.21E-02	-1.41E-02	-3.79E-02	-4.96E-01	-9.85E-02	-5.44E-21	-5.53E-20	-1.55E-06	3.81E-07	-7.11E-04
Shapiro-Wilk score	9.937E-01	9.948E-01	9.204E-01	9.747E-01	9.922E-01	9.406E-01	2.821E-01	9.937E-01	8.786E-01	9.355E-01
**fructose6phosphate**										
Local	-1.75E-02	-3.59E-03	-6.96E-03	-1.48E-01	-2.78E-02	2.74E-19	2.79E-18	-4.33E-07	1.60E-06	-1.16E-04
Min	-2.79E-02	-5.61E-03	-6.31E-02	-3.20E-01	-4.47E-02	-1.28E-18	-8.62E-17	-6.84E-07	1.37E-06	-1.00E-03
Max	-7.18E-03	-1.50E-03	2.94E-03	-4.62E-02	-1.09E-02	9.36E-19	2.57E-15	-2.26E-07	2.50E-06	5.11E-05
Normalized peak height	2.29E-03	2.49E-03	5.52E-03	2.66E-03	2.29E-03	4.12E-01	4.33E-02	3.41E-03	4.62E-03	4.93E-03
Peak sensitivity value	-1.87E-02	-3.93E-03	-2.31E-03	-1.43E-01	-3.11E-02	-4.06E-22	1.99E-19	-4.15E-07	1.57E-06	-3.15E-05
Shapiro-Wilk score	9.895E-01	9.903E-01	9.038E-01	9.813E-01	9.884E-01	9.515E-01	4.933E-01	9.966E-01	9.557E-01	9.209E-01
**3phosphoglycerate**										
Local	-4.03E-01	-8.28E-02	-1.06E-01	-3.41E+00	-6.42E-01	6.33E-18	3.11E-19	-9.98E-06	2.65E-06	-1.77E-03
Min	-4.51E-01	-9.41E-02	-1.88E-01	-5.13E+00	-7.16E-01	-1.10E-16	-1.57E-17	-1.82E-05	9.96E-07	-3.22E-03
Max	-3.54E-01	-7.29E-02	-4.15E-02	-2.50E+00	-5.66E-01	1.64E-17	5.34E-16	-4.87E-06	5.46E-06	-7.02E-04
Normalized peak height	2.77E-03	3.09E-03	2.42E-03	2.99E-03	3.26E-03	4.11E-01	7.88E-02	3.08E-03	2.76E-03	3.05E-03
Peak sensitivity value	-3.97E-01	-8.26E-02	-9.75E-02	-3.21E+00	-6.41E-01	4.13E-20	-2.11E-20	-1.01E-05	2.63E-06	-1.80E-03
Shapiro-Wilk score	9.966E-01	9.990E-01	9.892E-01	9.808E-01	9.974E-01	9.162E-01	3.573E-01	9.926E-01	9.857E-01	9.937E-01
**glycerol**										
Local	-2.39E-01	-4.91E-02	6.53E-01	-2.02E+00	-3.80E-01	3.75E-18	-1.82E-18	-5.91E-06	-1.61E-05	-9.89E-01
Min	-3.20E-01	-6.56E-02	3.79E-01	-3.14E+00	-4.90E-01	-1.12E-17	-2.20E-15	-1.16E-05	-2.59E-05	-9.96E-01
Max	-8.51E-02	-1.81E-02	8.69E-01	-7.72E-01	-1.39E-01	9.08E-18	6.19E-17	-1.76E-06	-8.94E-06	-9.82E-01
Normalized peak height	3.22E-03	2.81E-03	2.25E-03	3.80E-03	3.16E-03	4.12E-01	5.34E-02	2.60E-03	2.97E-03	2.93E-03
Peak sensitivity value	-2.55E-01	-5.17E-02	6.61E-01	-1.97E+00	-4.00E-01	-8.45E-21	-2.45E-18	-5.57E-06	-1.62E-05	-9.88E-01
Shapiro-Wilk score	9.789E-01	9.781E-01	9.886E-01	9.994E-01	9.739E-01	9.484E-01	4.156E-01	9.885E-01	9.950E-01	9.886E-01
**pyruvate**										
Local	6.54E-02	1.35E-02	-1.66E-01	5.54E-01	1.04E-01	-1.03E-18	4.87E-19	1.62E-06	4.15E-06	-2.78E-03
Min	2.00E-02	4.30E-03	-6.60E-01	1.32E-01	3.18E-02	-3.88E-18	-4.16E-17	6.30E-07	1.40E-06	-1.04E-02
Max	2.58E-01	5.39E-02	-5.81E-02	3.07E+00	4.24E-01	5.99E-18	1.29E-15	7.01E-06	1.94E-05	-9.89E-04
Normalized peak height	4.67E-03	5.09E-03	4.72E-03	5.49E-03	4.77E-03	4.12E-01	1.28E-01	6.17E-03	5.48E-03	5.13E-03
Peak sensitivity value	5.55E-02	1.12E-02	-1.46E-01	3.80E-01	7.91E-02	2.82E-21	4.15E-19	1.38E-06	3.55E-06	-2.50E-03
Shapiro-Wilk score	9.212E-01	9.225E-01	9.185E-01	9.104E-01	9.201E-01	9.487E-01	3.722E-01	9.077E-01	9.133E-01	9.152E-01

**dihydroxyacetonephosphate cytosol**																					
Local	-3.10E-01	-2.20E-03	-1.68E-04	-1.28E-03	-2.24E-02	-7.65E-03	-1.35E-01	-7.90E-02	6.75E-01	-6.46E-17	-1.08E-02	-1.25E-02	-2.58E-03	3.30E-02	-1.06E-01	-2.00E-02	1.97E-19	-9.46E-20	-3.10E-07	-8.20E-07	5.52E-04
Min	-6.80E-01	-4.36E-03	-5.23E-04	-3.96E-03	-7.17E-02	-1.74E-02	-3.74E-01	-2.24E-01	4.77E-01	-6.72E-16	-1.90E-02	-2.25E-02	-4.48E-03	1.19E-02	-2.79E-01	-3.57E-02	-9.71E-19	-2.05E-16	-5.93E-07	-1.37E-06	2.00E-04
Max	1.51E-01	1.81E-03	-4.80E-05	-3.58E-04	-3.99E-03	-2.01E-03	-2.96E-02	-1.84E-02	9.66E-01	7.08E-16	-4.72E-03	-4.26E-03	-8.98E-04	5.71E-02	-2.77E-02	-6.31E-03	6.25E-19	4.76E-18	-1.23E-07	-3.43E-07	9.67E-04
Normalized peak height	2.99E-03	4.10E-03	3.28E-03	3.45E-03	3.13E-03	2.58E-03	2.80E-03	2.93E-03	3.00E-03	6.88E-02	2.64E-03	2.78E-03	2.83E-03	2.58E-03	3.13E-03	2.70E-03	4.10E-01	1.02E-01	3.40E-03	2.34E-03	3.28E-03
Peak sensitivity value	-3.66E-01	-2.51E-03	-1.24E-04	-1.03E-03	-1.72E-02	-6.19E-03	-1.17E-01	-6.78E-02	6.28E-01	-6.46E-19	-1.11E-02	-1.23E-02	-2.59E-03	3.30E-02	-1.06E-01	-2.10E-02	2.71E-22	4.72E-20	-2.91E-07	-8.57E-07	5.26E-04
Shapiro-Wilk score	9.918E-01	9.788E-01	9.700E-01	9.709E-01	9.687E-01	9.841E-01	9.745E-01	9.757E-01	9.882E-01	9.899E-01	9.914E-01	9.958E-01	9.963E-01	9.933E-01	9.825E-01	9.945E-01	9.533E-01	3.433E-01	9.877E-01	9.892E-01	9.973E-01
**nadh**																					
Local	1.19E+00	8.44E-03	1.92E-04	1.37E-03	1.64E-02	1.21E-02	1.67E-01	-1.51E+00	-3.09E-02	2.01E-16	1.34E-02	1.55E-02	3.19E-03	-3.88E-02	1.31E-01	2.47E-02	-2.44E-19	7.06E-20	3.85E-07	-3.54E-07	-6.48E-04
Min	6.43E-01	6.15E-03	3.81E-05	1.85E-04	-6.22E-03	5.52E-03	4.13E-02	-1.61E+00	-9.88E-02	-1.11E-15	7.09E-03	6.11E-03	1.27E-03	-5.86E-02	4.12E-02	8.89E-03	-6.69E-19	-5.54E-18	1.86E-07	-9.74E-07	-9.47E-04
Max	1.56E+00	1.10E-02	5.92E-04	4.42E-03	7.44E-02	2.28E-02	4.56E-01	-1.36E+00	3.43E-02	1.23E-15	2.14E-02	2.31E-02	4.64E-03	-1.67E-02	2.64E-01	3.79E-02	3.39E-18	1.56E-16	6.25E-07	4.42E-07	-2.80E-04
Normalized peak height	2.72E-03	3.85E-03	3.30E-03	3.14E-03	3.18E-03	2.44E-03	2.98E-03	3.64E-03	3.34E-03	8.48E-02	2.63E-03	2.69E-03	2.69E-03	2.73E-03	2.67E-03	2.61E-03	4.12E-01	1.43E-01	3.53E-03	3.31E-03	3.18E-03
Peak sensitivity value	1.23E+00	8.36E-03	1.54E-04	1.02E-03	1.17E-02	1.18E-02	1.34E-01	-1.51E+00	-2.91E-02	-7.54E-19	1.29E-02	1.69E-02	3.46E-03	-4.25E-02	1.26E-01	2.59E-02	-1.83E-21	2.94E-20	3.65E-07	-4.21E-07	-6.69E-04
Shapiro-Wilk score	9.864E-01	9.989E-01	9.687E-01	9.693E-01	9.692E-01	9.866E-01	9.752E-01	9.991E-01	9.989E-01	9.720E-01	9.921E-01	9.870E-01	9.869E-01	9.908E-01	9.867E-01	9.869E-01	9.420E-01	2.789E-01	9.960E-01	9.979E-01	9.947E-01
**glycerol3phosphate**																					
Local	9.63E-01	6.85E-03	2.45E-03	1.91E-02	3.66E-01	-4.66E-02	4.02E-01	2.36E-01	-2.28E+00	2.02E-16	3.25E-02	3.76E-02	7.73E-03	-1.21E-01	3.18E-01	5.99E-02	-5.90E-19	1.45E-18	9.31E-07	-2.61E-05	-2.02E-03
Min	-3.91E-01	-4.19E-03	1.65E-03	1.33E-02	2.75E-01	-6.37E-02	7.32E-02	4.23E-02	-2.76E+00	-1.45E-15	1.51E-02	1.17E-02	2.38E-03	-2.07E-01	7.70E-02	1.75E-02	-1.84E-18	-4.64E-17	3.89E-07	-3.54E-05	-3.15E-03
Max	1.89E+00	1.25E-02	3.73E-03	2.94E-02	5.33E-01	-1.78E-02	1.09E+00	6.14E-01	-1.88E+00	1.53E-15	5.32E-02	6.01E-02	1.25E-02	-4.22E-02	6.97E-01	1.00E-01	1.08E-17	1.58E-15	1.64E-06	-1.94E-05	-8.13E-04
Normalized peak height	2.50E-03	3.66E-03	2.94E-03	3.24E-03	2.81E-03	2.98E-03	3.05E-03	2.92E-03	3.45E-03	6.30E-02	2.75E-03	2.89E-03	3.06E-03	2.67E-03	2.83E-03	2.79E-03	4.10E-01	4.86E-02	3.50E-03	3.12E-03	2.77E-03
Peak sensitivity value	1.15E+00	7.83E-03	2.34E-03	1.91E-02	3.45E-01	-4.94E-02	3.08E-01	1.74E-01	-2.28E+00	1.30E-18	3.17E-02	4.03E-02	7.97E-03	-1.19E-01	3.18E-01	6.43E-02	-9.02E-22	1.51E-18	8.69E-07	-2.58E-05	-2.16E-03
Shapiro-Wilk score	9.862E-01	9.680E-01	9.890E-01	9.899E-01	9.832E-01	9.875E-01	9.766E-01	9.781E-01	9.956E-01	9.911E-01	9.937E-01	9.940E-01	9.943E-01	9.916E-01	9.863E-01	9.931E-01	9.400E-01	4.656E-01	9.935E-01	9.965E-01	9.955E-01
**glucose6phosphate**																					
Local	1.66E+00	1.18E-02	-1.68E-01	-1.04E+00	-1.04E-01	6.02E-03	-1.84E-01	-1.08E-01	1.35E-01	4.60E-17	-1.45E-02	-1.68E-02	-3.45E-03	-6.64E-03	-1.42E-01	-2.67E-02	2.64E-19	2.69E-18	-4.16E-07	1.54E-06	-1.11E-04
Min	1.26E+00	6.65E-03	-1.85E-01	-1.06E+00	-2.02E-01	-5.30E-03	-5.51E-01	-3.33E-01	8.60E-02	-7.18E-16	-2.48E-02	-2.63E-02	-5.35E-03	-5.88E-02	-3.19E-01	-4.36E-02	-1.02E-18	-8.67E-17	-6.87E-07	1.32E-06	-8.54E-04
Max	2.38E+00	3.02E-02	-1.50E-01	-1.02E+00	-6.60E-02	1.19E-02	-4.92E-02	-2.93E-02	2.04E-01	9.29E-16	-8.36E-03	-6.79E-03	-1.40E-03	3.00E-03	-4.59E-02	-1.06E-02	7.63E-19	3.05E-15	-2.11E-07	2.34E-06	5.62E-05
Normalized peak height	2.64E-03	3.88E-03	3.02E-03	3.24E-03	3.06E-03	3.18E-03	3.15E-03	3.24E-03	2.75E-03	1.20E-01	2.56E-03	2.43E-03	2.46E-03	5.11E-03	2.62E-03	2.33E-03	4.13E-01	5.94E-02	3.44E-03	4.33E-03	4.31E-03
Peak sensitivity value	1.55E+00	1.01E-02	-1.70E-01	-1.04E+00	-9.08E-02	6.41E-03	-1.52E-01	-8.93E-02	1.37E-01	-7.08E-19	-1.40E-02	-1.86E-02	-3.47E-03	-2.90E-03	-1.27E-01	-3.07E-02	8.32E-23	-6.78E-18	-3.79E-07	1.51E-06	-3.34E-05
Shapiro-Wilk score	9.805E-01	9.529E-01	9.986E-01	9.960E-01	9.710E-01	9.785E-01	9.660E-01	9.681E-01	9.934E-01	9.713E-01	9.853E-01	9.892E-01	9.899E-01	9.050E-01	9.814E-01	9.883E-01	9.515E-01	4.208E-01	9.960E-01	9.576E-01	9.215E-01
**3phosphoglycerate cytosol**																					
Local	3.53E+00	2.51E-02	7.47E-04	5.43E-03	7.54E-02	2.52E-02	4.48E-01	2.63E-01	2.31E-01	0.00E+00	3.60E-02	-4.03E-01	-8.28E-02	-1.06E-01	-3.41E+00	-6.42E-01	6.33E-18	3.11E-19	1.03E-06	2.65E-06	-1.77E-03
Min	1.94E+00	1.63E-02	2.44E-04	1.68E-03	1.21E-02	6.47E-03	9.10E-02	5.51E-02	8.17E-02	-1.69E-14	1.79E-02	-4.51E-01	-9.37E-02	-1.91E-01	-5.11E+00	-7.20E-01	-1.51E-16	-1.70E-17	5.11E-07	9.39E-07	-3.17E-03
Max	4.90E+00	4.24E-02	2.10E-03	1.60E-02	2.42E-01	5.84E-02	1.27E+00	7.37E-01	4.39E-01	1.52E-14	6.87E-02	-3.57E-01	-7.23E-02	-4.14E-02	-2.50E+00	-5.79E-01	1.35E-17	5.81E-16	1.53E-06	5.66E-06	-7.38E-04
Normalized peak height	3.84E-03	3.58E-03	3.08E-03	3.16E-03	2.95E-03	2.62E-03	2.80E-03	2.66E-03	2.31E-03	3.40E-01	2.75E-03	2.76E-03	3.10E-03	2.45E-03	2.77E-03	3.02E-03	4.09E-01	8.56E-02	3.07E-03	2.92E-03	2.98E-03
Peak sensitivity value	3.48E+00	2.44E-02	5.98E-04	4.11E-03	5.21E-02	2.12E-02	2.98E-01	1.97E-01	2.05E-01	1.76E-18	3.38E-02	-4.00E-01	-8.28E-02	-1.02E-01	-3.27E+00	-6.40E-01	6.93E-20	5.18E-20	1.10E-06	2.69E-06	-1.77E-03
Shapiro-Wilk score	9.991E-01	9.793E-01	9.659E-01	9.666E-01	9.642E-01	9.796E-01	9.701E-01	9.712E-01	9.871E-01	9.318E-01	9.798E-01	9.966E-01	9.988E-01	9.890E-01	9.810E-01	9.971E-01	9.004E-01	3.227E-01	9.916E-01	9.857E-01	9.938E-01
**phosphoenolpyruvate**																					
Local	4.22E-01	3.01E-03	8.93E-05	6.49E-04	9.02E-03	3.02E-03	5.36E-02	3.14E-02	2.76E-02	-1.05E-16	4.31E-03	-4.91E-01	-1.01E-01	-1.27E-02	4.22E-02	7.95E-03	7.72E-18	3.72E-20	1.24E-07	3.16E-07	-2.12E-04
Min	2.39E-01	2.24E-03	3.61E-05	2.52E-04	2.02E-03	1.02E-03	1.41E-02	8.96E-03	1.20E-02	-1.23E-14	2.43E-03	-5.29E-01	-1.10E-01	-1.86E-02	1.36E-02	3.29E-03	-1.93E-17	-1.76E-18	6.21E-08	1.57E-07	-3.08E-04
Max	5.57E-01	3.88E-03	2.25E-04	1.66E-03	2.86E-02	6.61E-03	1.49E-01	8.99E-02	4.33E-02	1.32E-14	6.63E-03	-4.58E-01	-9.29E-02	-5.95E-03	8.28E-02	1.19E-02	1.95E-17	9.35E-17	2.02E-07	5.29E-07	-1.08E-04
Normalized peak height	2.64E-03	3.72E-03	3.09E-03	3.17E-03	3.14E-03	2.68E-03	3.17E-03	3.03E-03	2.39E-03	2.27E-01	2.70E-03	1.84E-03	3.23E-03	2.79E-03	2.65E-03	2.64E-03	4.09E-01	1.10E-01	3.47E-03	2.71E-03	3.28E-03
Peak sensitivity value	4.45E-01	2.95E-03	8.14E-05	5.51E-04	6.98E-03	2.83E-03	4.59E-02	2.81E-02	2.96E-02	-8.53E-18	4.16E-03	-4.84E-01	-1.01E-01	-1.29E-02	4.47E-02	8.62E-03	-4.19E-21	9.83E-20	1.20E-07	3.13E-07	-2.17E-04
Shapiro-Wilk score	9.917E-01	9.982E-01	9.707E-01	9.706E-01	9.695E-01	9.871E-01	9.747E-01	9.769E-01	9.912E-01	8.969E-01	9.939E-01	9.748E-01	9.989E-01	9.927E-01	9.868E-01	9.883E-01	9.204E-01	2.930E-01	9.958E-01	9.955E-01	9.966E-01
**bisphosphoglycerate**																					
Local	-5.89E+00	-4.19E-02	5.08E-03	4.06E-02	8.47E-01	1.55E-01	3.68E+00	2.16E+00	4.96E-03	-1.01E-15	-8.02E-02	-9.29E-02	-1.91E-02	1.64E-01	-7.86E-01	-1.48E-01	1.46E-18	3.25E-18	-2.30E-06	5.67E-08	2.74E-03
Min	-8.45E+00	-8.68E-02	3.06E-03	2.50E-02	5.68E-01	1.11E-01	2.45E+00	1.44E+00	-3.42E-01	-6.73E-15	-2.39E-01	-2.46E-01	-5.05E-02	5.70E-02	-2.41E+00	-3.97E-01	-9.44E-18	-1.10E-16	-7.91E-06	-4.48E-06	9.66E-04
Max	-4.13E+00	-2.44E-02	8.54E-03	6.69E-02	1.32E+00	2.14E-01	5.82E+00	3.25E+00	2.02E-01	7.64E-15	-3.26E-02	-2.64E-02	-5.68E-03	3.86E-01	-1.78E-01	-4.21E-02	4.86E-18	6.07E-15	-7.66E-07	1.90E-06	6.01E-03
Normalized peak height	3.41E-03	3.93E-03	3.38E-03	3.51E-03	3.16E-03	3.24E-03	3.15E-03	3.18E-03	3.50E-03	7.30E-02	3.55E-03	2.91E-03	2.85E-03	2.84E-03	2.95E-03	2.76E-03	4.11E-01	8.15E-02	4.50E-03	3.72E-03	3.05E-03
Peak sensitivity value	-5.82E+00	-4.00E-02	5.01E-03	4.05E-02	7.88E-01	1.51E-01	3.63E+00	2.19E+00	2.56E-02	1.63E-18	-6.59E-02	-9.04E-02	-1.56E-02	1.59E-01	-4.94E-01	-1.16E-01	5.31E-21	-1.55E-18	-2.20E-06	4.14E-07	2.53E-03
Shapiro-Wilk score	9.923E-01	9.810E-01	9.918E-01	9.921E-01	9.916E-01	9.951E-01	9.922E-01	9.934E-01	9.825E-01	9.780E-01	9.572E-01	9.772E-01	9.782E-01	9.843E-01	9.677E-01	9.764E-01	9.490E-01	3.856E-01	9.543E-01	9.700E-01	9.907E-01
**atpg**																					
Local	-2.48E+00	-1.77E-02	1.11E-03	8.98E-03	1.98E-01	3.30E-02	8.27E-01	4.85E-01	-6.16E-02	-2.63E-16	6.66E-02	7.71E-02	1.58E-02	7.12E-02	6.52E-01	1.23E-01	-1.21E-18	-7.32E-18	1.91E-06	-7.06E-07	1.19E-03
Min	-7.92E+00	-1.02E-01	2.44E-04	2.00E-03	4.27E-02	8.92E-03	1.89E-01	1.14E-01	-6.71E-01	-4.19E-15	3.58E-02	2.83E-02	5.86E-03	7.72E-03	1.90E-01	4.38E-02	-3.68E-18	-9.05E-15	9.21E-07	-8.01E-06	1.44E-04
Max	-6.73E-01	-3.77E-03	4.64E-03	3.81E-02	8.33E-01	1.06E-01	3.46E+00	2.03E+00	6.69E-03	4.34E-15	1.59E-01	1.54E-01	3.25E-02	5.21E-01	1.76E+00	2.59E-01	4.11E-18	2.44E-16	3.61E-06	6.04E-08	6.73E-03
Normalized peak height	3.36E-03	5.51E-03	4.09E-03	4.33E-03	4.23E-03	3.47E-03	4.24E-03	4.24E-03	7.27E-03	1.45E-01	3.55E-03	2.70E-03	2.98E-03	6.16E-03	2.92E-03	2.91E-03	4.12E-01	6.14E-02	3.45E-03	7.33E-03	4.65E-03
Peak sensitivity value	-1.96E+00	-1.25E-02	7.87E-04	6.32E-03	1.33E-01	2.66E-02	6.55E-01	3.74E-01	-2.21E-02	-3.12E-18	5.79E-02	6.53E-02	1.49E-02	3.98E-02	5.71E-01	1.16E-01	1.45E-21	-1.15E-17	1.90E-06	-2.42E-07	6.09E-04
Shapiro-Wilk score	9.633E-01	9.188E-01	9.412E-01	9.408E-01	9.384E-01	9.620E-01	9.419E-01	9.450E-01	8.738E-01	9.699E-01	9.651E-01	9.888E-01	9.899E-01	9.053E-01	9.666E-01	9.872E-01	9.539E-01	4.304E-01	9.898E-01	8.597E-01	9.218E-01
**ampc**																					
Local	-1.22E+00	-8.69E-03	-2.58E-04	-1.87E-03	-2.61E-02	-8.72E-03	-1.55E-01	-9.08E-02	-7.98E-02	-6.07E-20	-1.24E-02	-1.44E-02	1.73E+00	3.66E-02	-1.22E-01	-2.30E-02	-3.42E-16	-1.07E-19	-3.57E-07	-9.14E-07	6.12E-04
Min	-1.55E+00	-1.11E-02	-6.68E-04	-5.06E-03	-7.89E-02	-1.91E-02	-4.25E-01	-2.50E-01	-1.21E-01	-3.55E-13	-1.88E-02	-2.12E-02	1.70E+00	1.74E-02	-2.38E-01	-3.43E-02	-8.65E-16	-1.23E-16	-6.06E-07	-1.48E-06	3.20E-04
Max	-7.16E-01	-6.59E-03	-1.02E-04	-6.98E-04	-5.77E-03	-2.90E-03	-4.20E-02	-2.53E-02	-3.41E-02	2.48E-13	-7.12E-03	-6.22E-03	1.75E+00	5.57E-02	-4.03E-02	-9.66E-03	3.78E-15	5.77E-18	-1.90E-07	-4.44E-07	8.83E-04
Normalized peak height	2.46E-03	4.26E-03	3.15E-03	3.32E-03	2.94E-03	2.60E-03	3.00E-03	3.02E-03	2.24E-03	2.16E-01	2.60E-03	2.74E-03	2.63E-03	2.75E-03	2.67E-03	2.59E-03	4.09E-01	5.63E-02	3.60E-03	2.57E-03	3.09E-03
Peak sensitivity value	-1.27E+00	-8.64E-03	-2.09E-04	-1.81E-03	-2.23E-02	-9.57E-03	-1.45E-01	-7.73E-02	-8.63E-02	-3.98E-18	-1.27E-02	-1.48E-02	1.73E+00	3.92E-02	-1.21E-01	-2.53E-02	1.76E-18	3.82E-20	-3.49E-07	-9.01E-07	6.20E-04
Shapiro-Wilk score	9.870E-01	9.985E-01	9.702E-01	9.704E-01	9.695E-01	9.870E-01	9.749E-01	9.771E-01	9.894E-01	9.040E-01	9.934E-01	9.861E-01	9.930E-01	9.911E-01	9.869E-01	9.862E-01	9.301E-01	4.042E-01	9.968E-01	9.952E-01	9.953E-01
**nad**																					
Local	-2.14E-02	-1.52E-04	-3.47E-06	-2.46E-05	-2.95E-04	-2.18E-04	-3.01E-03	2.72E-02	5.57E-04	-3.62E-18	-2.42E-04	-2.80E-04	-5.76E-05	6.99E-04	-2.37E-03	-4.46E-04	4.40E-21	-1.27E-21	-6.93E-09	6.38E-09	1.17E-05
Min	-3.02E-02	-2.24E-04	-1.22E-05	-9.08E-05	-1.44E-03	-4.66E-04	-9.22E-03	2.11E-02	-5.47E-04	-2.18E-17	-4.26E-04	-4.83E-04	-9.81E-05	2.90E-04	-5.49E-03	-8.15E-04	-6.08E-20	-2.63E-18	-1.24E-08	-7.46E-09	4.94E-06
Max	-1.23E-02	-1.01E-04	-6.63E-07	-3.18E-06	9.51E-05	-8.81E-05	-6.93E-04	3.49E-02	1.88E-03	2.46E-17	-1.15E-04	-1.02E-04	-2.14E-05	1.21E-03	-7.11E-04	-1.60E-04	1.25E-20	8.98E-20	-2.95E-09	1.74E-08	1.92E-05
Normalized peak height	3.52E-03	3.62E-03	3.19E-03	3.30E-03	3.23E-03	2.53E-03	3.09E-03	2.90E-03	3.59E-03	9.24E-02	2.55E-03	2.52E-03	2.62E-03	2.51E-03	2.51E-03	2.45E-03	4.11E-01	1.38E-01	3.36E-03	3.43E-03	2.91E-03
Peak sensitivity value	-2.08E-02	-1.48E-04	-2.80E-06	-1.72E-05	-1.25E-04	-2.06E-04	-2.05E-03	2.71E-02	5.45E-04	-2.00E-20	-2.39E-04	-2.94E-04	-6.19E-05	7.15E-04	-2.28E-03	-4.94E-04	1.22E-23	-1.32E-21	-6.52E-09	6.81E-09	1.17E-05
Shapiro-Wilk score	9.989E-01	9.943E-01	9.607E-01	9.609E-01	9.589E-01	9.824E-01	9.677E-01	9.957E-01	9.997E-01	9.731E-01	9.899E-01	9.927E-01	9.935E-01	9.931E-01	9.834E-01	9.913E-01	1.000E+00	3.083E-01	9.935E-01	9.965E-01	9.970E-01
**fructose16bisphosphate**																					
Local	4.06E-01	2.89E-03	4.32E-03	3.39E-02	-1.00E-01	5.73E-03	-1.77E-01	-1.04E-01	1.29E-01	4.42E-17	-1.39E-02	-1.61E-02	-3.31E-03	-6.40E-03	-1.36E-01	-2.56E-02	2.53E-19	2.53E-18	-3.99E-07	1.48E-06	-1.07E-04
Min	2.34E-02	1.38E-04	3.27E-03	2.59E-02	-1.83E-01	-5.48E-03	-4.98E-01	-2.80E-01	8.30E-02	-6.68E-16	-2.40E-02	-2.49E-02	-4.98E-03	-5.12E-02	-2.93E-01	-4.01E-02	-1.04E-18	-7.88E-17	-6.27E-07	1.29E-06	-7.47E-04
Max	1.03E+00	1.35E-02	5.71E-03	4.39E-02	-6.30E-02	1.15E-02	-4.95E-02	-3.05E-02	2.00E-01	8.42E-16	-8.14E-03	-6.55E-03	-1.34E-03	3.14E-03	-4.37E-02	-9.84E-03	6.87E-19	2.63E-15	-1.90E-07	2.15E-06	5.06E-05
Normalized peak height	2.56E-03	4.13E-03	3.14E-03	3.32E-03	3.03E-03	3.19E-03	3.13E-03	2.83E-03	2.87E-03	1.32E-01	2.68E-03	2.54E-03	2.61E-03	4.75E-03	2.73E-03	2.39E-03	4.10E-01	6.11E-02	3.47E-03	4.29E-03	4.10E-03
Peak sensitivity value	3.61E-01	2.02E-03	4.22E-03	3.34E-02	-8.87E-02	6.84E-03	-1.55E-01	-9.35E-02	1.26E-01	3.62E-19	-1.27E-02	-1.83E-02	-3.75E-03	-1.78E-03	-1.36E-01	-2.85E-02	2.43E-22	3.78E-18	-3.77E-07	1.45E-06	-4.79E-05
Shapiro-Wilk score	9.826E-01	9.490E-01	9.959E-01	9.971E-01	9.746E-01	9.818E-01	9.700E-01	9.721E-01	9.937E-01	9.732E-01	9.874E-01	9.874E-01	9.877E-01	9.111E-01	9.836E-01	9.867E-01	9.512E-01	4.740E-01	9.968E-01	9.670E-01	9.269E-01
**ampg**																					
Local	1.67E+01	1.19E-01	-7.45E-03	-6.05E-02	-1.33E+00	-2.22E-01	-5.57E+00	-3.27E+00	4.15E-01	1.77E-15	-4.48E-01	-5.19E-01	-1.07E-01	-4.79E-01	-4.39E+00	-8.26E-01	8.15E-18	3.08E-16	-1.29E-05	4.75E-06	-8.01E-03
Min	1.30E+01	7.13E-02	-1.20E-02	-9.54E-02	-1.95E+00	-3.14E-01	-8.18E+00	-4.69E+00	-1.21E-01	-1.77E-14	-7.48E-01	-6.51E-01	-1.36E-01	-8.87E-01	-6.75E+00	-1.01E+00	-2.68E-17	-3.04E-14	-2.41E-05	-1.19E-06	-1.43E-02
Max	2.05E+01	1.85E-01	-4.36E-03	-3.56E-02	-8.35E-01	-1.41E-01	-3.53E+00	-2.08E+00	1.11E+00	2.08E-14	-2.45E-01	-2.29E-01	-4.70E-02	-1.46E-01	-1.95E+00	-3.90E-01	2.23E-17	4.01E-13	-4.83E-06	1.57E-05	-2.63E-03
Normalized peak height	4.43E-03	2.91E-03	3.46E-03	3.62E-03	3.40E-03	3.48E-03	3.18E-03	3.42E-03	2.33E-03	1.45E-01	3.07E-03	3.08E-03	2.85E-03	2.32E-03	3.86E-03	3.31E-03	4.12E-01	7.47E-02	2.65E-03	2.82E-03	2.66E-03
Peak sensitivity value	1.62E+01	1.19E-01	-7.32E-03	-5.73E-02	-1.32E+00	-2.13E-01	-5.53E+00	-3.15E+00	3.57E-01	-1.44E-17	-4.19E-01	-5.50E-01	-1.16E-01	-5.01E-01	-4.03E+00	-8.66E-01	1.09E-20	2.54E-17	-1.24E-05	4.02E-06	-8.71E-03
Shapiro-Wilk score	9.994E-01	9.951E-01	9.938E-01	9.941E-01	9.962E-01	9.961E-01	9.935E-01	9.940E-01	9.838E-01	9.971E-01	9.924E-01	9.722E-01	9.719E-01	9.881E-01	9.986E-01	9.646E-01	9.459E-01	5.970E-01	9.905E-01	9.833E-01	9.920E-01
**atpc**																					
Local	1.92E-01	1.36E-03	4.05E-05	2.94E-04	4.09E-03	1.37E-03	2.43E-02	1.43E-02	1.25E-02	2.20E-21	1.95E-03	2.26E-03	-2.71E-01	-5.75E-03	1.91E-02	3.61E-03	9.62E-18	1.69E-20	5.61E-08	1.44E-07	-9.61E-05
Min	1.07E-01	1.01E-03	1.68E-05	1.10E-04	8.92E-04	4.43E-04	6.07E-03	3.60E-03	5.36E-03	-3.48E-14	1.10E-03	9.40E-04	-2.98E-01	-8.76E-03	6.29E-03	1.46E-03	-8.03E-17	-8.08E-19	2.84E-08	6.84E-08	-1.38E-04
Max	2.61E-01	1.77E-03	1.10E-04	8.15E-04	1.32E-02	3.12E-03	7.06E-02	3.90E-02	1.97E-02	3.72E-14	3.25E-03	3.46E-03	-2.46E-01	-2.68E-03	3.74E-02	5.44E-03	2.44E-17	3.85E-17	9.60E-08	2.36E-07	-4.93E-05
Normalized peak height	2.35E-03	3.76E-03	3.52E-03	3.60E-03	3.46E-03	3.03E-03	3.53E-03	3.24E-03	2.55E-03	2.17E-01	3.58E-03	3.23E-03	2.42E-03	3.00E-03	2.83E-03	2.87E-03	4.11E-01	1.01E-01	3.87E-03	2.98E-03	3.32E-03
Peak sensitivity value	1.84E-01	1.35E-03	3.68E-05	2.70E-04	3.14E-03	1.36E-03	2.21E-02	1.36E-02	1.31E-02	-2.53E-17	1.95E-03	2.33E-03	-2.70E-01	-6.00E-03	1.89E-02	3.90E-03	-1.42E-21	3.74E-20	5.43E-08	1.45E-07	-9.77E-05
Shapiro-Wilk score	9.891E-01	9.990E-01	9.699E-01	9.697E-01	9.699E-01	9.881E-01	9.748E-01	9.770E-01	9.939E-01	9.105E-01	9.966E-01	9.908E-01	9.937E-01	9.958E-01	9.884E-01	9.906E-01	9.183E-01	3.406E-01	9.955E-01	9.969E-01	9.986E-01

glycerol3phosphate cytosol<br>Local<br>Min<br>Max<br>Normalized peak height<br>Peak sensitivity value<br>Shapiro-Wilk score	7.65E-01<br>-3.72E-01<br>1.50E+00<br>2.49E-03<br>8.61E-01<br>9.855E-01	5.45E-03<br>-4.34E-03<br>9.34E-03<br>3.92E-03<br>5.98E-03<br>9.654E-01	4.16E-04<br>1.35E-04<br>1.18E-03<br>3.33E-03<br>3.19E-04<br>9.704E-01	3.17E-03<br>9.73E-04<br>9.16E-03<br>3.32E-03<br>2.55E-03<br>9.708E-01	5.53E-02<br>1.02E-02<br>1.64E-01<br>2.96E-03<br>4.43E-02<br>9.692E-01	1.89E-02<br>5.59E-03<br>4.10E-02<br>2.55E-03<br>1.53E-02<br>9.858E-01	3.33E-01<br>7.58E-02<br>8.90E-01<br>2.98E-03<br>2.78E-01<br>9.745E-01	1.95E-01<br>4.64E-02<br>5.01E-01<br>2.85E-03<br>1.69E-01<br>9.766E-01	-1.67E+00<br>-1.83E+00<br>-1.50E+00<br>3.26E-03<br>-1.67E+00<br>9.995E-01	1.60E-16<br>-1.08E-15<br>1.38E-15<br>6.24E-02<br>5.86E-19<br>9.915E-01	2.68E-02<br>1.41E-02<br>4.28E-02<br>2.70E-03<br>2.76E-02<br>9.928E-01	3.10E-02<br>1.23E-02<br>4.67E-02<br>2.78E-03<br>3.30E-02<br>9.893E-01	6.37E-03<br>2.50E-03<br>9.34E-03<br>2.92E-03<br>6.87E-03<br>9.893E-01	-8.17E-02<br>-1.28E-01<br>-3.54E-02<br>2.46E-03<br>-8.76E-02<br>9.930E-01	2.62E-01<br>7.98E-02<br>5.34E-01<br>2.68E-03<br>2.57E-01<br>9.865E-01	4.94E-02<br>1.85E-02<br>7.53E-02<br>2.61E-03<br>5.20E-02<br>9.886E-01	-4.87E-19<br>-1.26E-18<br>4.97E-18<br>4.10E-01<br>6.06E-22<br>9.481E-01	2.34E-19<br>-1.13E-17<br>8.20E-16<br>1.56E-01<br>-5.46E-20<br>2.919E-01	7.68E-07<br>3.57E-07<br>1.23E-06<br>3.65E-03<br>7.36E-07<br>9.958E-01	2.03E-06<br>9.27E-07<br>3.46E-06<br>2.68E-03<br>1.86E-06<br>9.930E-01	-1.37E-03<br>-2.01E-03<br>-6.01E-04<br>3.02E-03<br>-1.41E-03<br>9.961E-01

Table S5: Concentration control coefficients for *Trypanosoma brucei* model with parameter variation of ±10%

	(glucosetransport)	(hexokinase)	(phosphoglycerateisomerase)	(phophofructokinase)	(aldolase)	(triosephosphateisomerase)	(glyceraldehyde3phosphatedehydrogenase)	(glycerol3phosphatedehydrogenase)	(glycerol3phosphateoxidase)	(pyruvatetransport)	(phosphoglyceratekinase)	(pyruvatekinase)	(atputilisation)	(glycerolkinase)	(phosphoglyceratemutase)	(enolase)	(adenylatekinasecytosol)	(adenylatekinaseglycosome)	(3phosphoglyceratetransport)	(gly3pdhapantiporter)	(glyceroltransport)
adpg<br>Local<br>Min<br>Max<br>Normalized peak height<br>Peak sensitivity value<br>Shapiro-Wilk score	7.11E+00<br>-8.34E+00<br>1.09E+01<br>8.91E-03<br>6.91E+00<br>8.662E-01	5.06E-02<br>-4.33E-01<br>9.59E-02<br>2.39E-02<br>4.70E-02<br>8.019E-01	-3.17E-03<br>-7.22E-03<br>3.35E-03<br>6.16E-03<br>-2.67E-03<br>9.785E-01	-2.57E-02<br>-5.53E-02<br>3.01E-02<br>6.37E-03<br>-2.12E-02<br>9.732E-01	-5.67E-01<br>-1.14E+00<br>1.19E+00<br>9.24E-03<br>-5.08E-01<br>9.390E-01	-9.47E-02<br>-1.96E-01<br>1.05E-01<br>6.27E-03<br>-8.44E-02<br>9.767E-01	-2.37E+00<br>-4.69E+00<br>4.39E+00<br>7.52E-03<br>-2.03E+00<br>9.647E-01	-1.39E+00<br>-2.74E+00<br>2.36E+00<br>7.52E-03<br>-1.15E+00<br>9.642E-01	1.77E-01<br>-1.00E+00<br>4.78E-01<br>6.01E-03<br>2.69E-01<br>9.631E-01	7.55E-16<br>-2.19E-14<br>3.04E-14<br>1.76E-01<br>5.29E-18<br>9.378E-01	-1.91E-01<br>-5.62E-01<br>1.34E-01<br>3.36E-03<br>-1.61E-01<br>9.956E-01	-2.21E-01<br>-3.37E-01<br>9.06E-02<br>3.45E-03<br>-2.66E-01<br>9.443E-01	-4.54E-02<br>-7.22E-02<br>1.78E-02<br>3.54E-03<br>-5.65E-02<br>9.448E-01	-2.04E-01<br>-3.73E-01<br>7.14E-01<br>7.50E-03<br>-2.39E-01<br>9.528E-01	-1.87E+00<br>-4.27E+00<br>1.06E+00<br>4.65E-03<br>-1.58E+00<br>9.928E-01	-3.52E-01<br>-5.35E-01<br>1.30E-01<br>4.09E-03<br>-3.95E-01<br>9.327E-01	3.47E-18<br>-1.51E-16<br>1.19E-17<br>4.09E-01<br>4.24E-20<br>7.936E-01	-1.01E-17<br>-1.53E-14<br>3.41E-16<br>9.04E-02<br>4.64E-18<br>3.612E-01	-5.47E-06<br>-1.83E-05<br>1.52E-06<br>2.58E-03<br>-5.03E-06<br>9.760E-01	2.02E-06<br>-1.80E-05<br>6.30E-06<br>6.78E-03<br>2.69E-06<br>9.778E-01	-3.41E-03<br>-6.90E-03<br>4.16E-03<br>4.76E-03<br>-3.34E-03<br>9.831E-01
adpc<br>Local<br>Min<br>Max<br>Normalized peak height<br>Peak sensitivity value<br>Shapiro-Wilk score	-5.14E-01<br>-7.06E-01<br>-1.09E-01<br>2.46E-03<br>-5.79E-01<br>9.706E-01	-3.66E-03<br>-7.37E-03<br>-2.04E-03<br>5.08E-03<br>-3.47E-03<br>9.853E-01	-1.09E-04<br>-5.86E-04<br>-1.97E-05<br>4.83E-03<br>-6.52E-05<br>9.186E-01	-7.90E-04<br>-4.45E-03<br>-1.27E-04<br>4.79E-03<br>-4.19E-04<br>9.168E-01	-1.10E-02<br>-6.65E-02<br>-2.95E-04<br>4.60E-03<br>-3.64E-03<br>9.175E-01	-3.67E-03<br>-1.21E-02<br>-3.74E-04<br>2.57E-03<br>-1.81E-03<br>9.683E-01	-6.52E-02<br>-3.14E-01<br>-4.36E-03<br>3.63E-03<br>-2.31E-02<br>9.330E-01	-3.83E-02<br>-1.77E-01<br>-2.61E-03<br>3.67E-03<br>-1.54E-02<br>9.387E-01	-3.37E-02<br>-6.55E-02<br>-5.72E-03<br>2.16E-03<br>-4.30E-02<br>9.810E-01	0.00E+00<br>-2.60E-13<br>2.66E-13<br>3.01E-01<br>1.41E-16<br>4.923E-01	-5.25E-03<br>-1.27E-02<br>-1.64E-03<br>3.07E-03<br>-5.63E-03<br>9.885E-01	-6.07E-03<br>-1.12E-02<br>-9.11E-04<br>2.66E-03<br>-6.93E-03<br>9.834E-01	7.27E-01<br>6.64E-01<br>7.75E-01<br>2.45E-03<br>7.20E-01<br>9.931E-01	1.54E-02<br>3.24E-03<br>2.78E-02<br>2.43E-03<br>1.84E-02<br>9.804E-01	-5.14E-02<br>-2.06E-01<br>-4.63E-03<br>3.72E-03<br>-4.49E-02<br>9.346E-01	-9.68E-03<br>-1.81E-02<br>-1.21E-03<br>2.50E-03<br>-1.09E-02<br>9.853E-01	9.75E-18<br>-1.91E-16<br>2.56E-17<br>4.09E-01<br>1.06E-19<br>8.686E-01	-4.52E-20<br>-2.67E-16<br>2.65E-18<br>3.06E-01<br>9.32E-20<br>1.327E-01	-1.50E-07<br>-3.59E-07<br>-3.53E-08<br>3.59E-03<br>-1.28E-07<br>9.865E-01	-3.85E-07<br>-9.07E-07<br>-8.51E-08<br>2.59E-03<br>-4.30E-07<br>9.884E-01	2.58E-04<br>5.63E-05<br>4.76E-04<br>3.09E-03<br>2.60E-04<br>9.967E-01
dihydroxyacetonephosphate<br>Local<br>Min<br>Max<br>Normalized peak height<br>Peak sensitivity value<br>Shapiro-Wilk score	-1.12E-01<br>-3.29E-01<br>3.04E-01<br>3.16E-03<br>-1.80E-01<br>9.654E-01	-8.00E-04<br>-2.27E-03<br>8.07E-03<br>9.65E-03<br>-1.01E-03<br>8.688E-01	1.87E-03<br>1.07E-03<br>3.33E-03<br>3.44E-03<br>1.84E-03<br>9.866E-01	1.47E-02<br>8.66E-03<br>2.50E-02<br>3.63E-03<br>1.41E-02<br>9.899E-01	2.89E-01<br>2.09E-01<br>4.01E-01<br>3.20E-03<br>2.80E-01<br>9.937E-01	-7.31E-02<br>-9.43E-02<br>-5.70E-02<br>3.17E-03<br>-7.21E-02<br>9.966E-01	-6.54E-02<br>-3.04E-01<br>-6.20E-03<br>3.57E-03<br>-2.89E-02<br>9.228E-01	-3.84E-02<br>-2.15E-01<br>-3.91E-03<br>4.41E-03<br>-1.50E-02<br>9.278E-01	6.36E-02<br>2.56E-02<br>1.64E-01<br>3.30E-03<br>6.55E-02<br>9.866E-01	-2.29E-17<br>-3.73E-16<br>4.18E-16<br>1.08E-01<br>2.07E-19<br>9.124E-01	-5.13E-03<br>-1.32E-02<br>-1.92E-03<br>2.78E-03<br>-4.38E-03<br>9.739E-01	-5.94E-03<br>-1.15E-02<br>-7.66E-04<br>2.54E-03<br>-6.20E-03<br>9.848E-01	-1.22E-03<br>-2.30E-03<br>-1.77E-04<br>2.53E-03<br>-1.37E-03<br>9.842E-01	-6.36E-03<br>-5.20E-02<br>6.23E-04<br>6.67E-03<br>-1.40E-03<br>8.604E-01	-5.03E-02<br>-2.38E-01<br>-4.92E-03<br>3.86E-03<br>-4.81E-02<br>9.264E-01	-9.47E-03<br>-1.98E-02<br>-1.17E-03<br>2.39E-03<br>-1.16E-02<br>9.856E-01	9.33E-20<br>-4.35E-18<br>3.51E-19<br>4.06E-01<br>2.45E-22<br>7.984E-01	1.12E-18<br>-3.49E-17<br>1.15E-15<br>6.42E-02<br>9.36E-20<br>5.712E-01	-1.47E-07<br>-3.29E-07<br>-3.83E-08<br>3.25E-03<br>-1.28E-07<br>9.847E-01	7.28E-07<br>5.28E-07<br>1.75E-06<br>5.25E-03<br>7.05E-07<br>8.928E-01	-1.06E-04<br>-6.47E-04<br>9.54E-06<br>4.68E-03<br>-3.21E-05<br>8.977E-01
glyceraldehyde3phosphate<br>Local<br>Min<br>Max<br>Normalized peak height<br>Peak sensitivity value<br>Shapiro-Wilk score	-1.73E-01<br>-4.40E-01<br>3.12E-01<br>2.46E-03<br>-2.57E-01<br>9.626E-01	-1.23E-03<br>-3.02E-03<br>8.38E-03<br>7.43E-03<br>-1.62E-03<br>8.755E-01	1.89E-03<br>1.07E-03<br>3.30E-03<br>3.42E-03<br>1.84E-03<br>9.867E-01	1.49E-02<br>8.40E-03<br>2.51E-02<br>3.66E-03<br>1.45E-02<br>9.904E-01	2.93E-01<br>2.10E-01<br>4.02E-01<br>3.13E-03<br>2.88E-01<br>9.940E-01	6.17E-02<br>4.15E-02<br>7.85E-02<br>3.54E-03<br>5.99E-02<br>9.994E-01	-9.23E-02<br>-4.12E-01<br>-8.52E-03<br>3.36E-03<br>-4.38E-02<br>9.279E-01	-5.41E-02<br>-2.33E-01<br>-5.94E-03<br>3.37E-03<br>-2.45E-02<br>9.332E-01	5.22E-02<br>2.26E-02<br>1.41E-01<br>3.33E-03<br>4.59E-02<br>9.826E-01	-3.55E-17<br>-4.71E-16<br>4.69E-16<br>1.02E-01<br>2.25E-19<br>9.282E-01	-7.29E-03<br>-1.79E-02<br>-2.72E-03<br>2.79E-03<br>-5.61E-03<br>9.791E-01	-8.44E-03<br>-1.62E-02<br>-1.23E-03<br>2.56E-03<br>-1.01E-02<br>9.825E-01	-1.73E-03<br>-3.14E-03<br>-2.69E-04<br>2.55E-03<br>-1.98E-03<br>9.811E-01	-4.30E-04<br>-4.44E-02<br>6.49E-03<br>1.24E-02<br>1.48E-03<br>8.103E-01	-7.14E-02<br>-3.55E-01<br>-6.02E-03<br>4.21E-03<br>-5.72E-02<br>9.298E-01	-1.34E-02<br>-2.54E-02<br>-1.66E-03<br>2.30E-03<br>-1.47E-02<br>9.844E-01	1.32E-19<br>-1.42E-17<br>4.79E-19<br>4.07E-01<br>7.08E-22<br>6.238E-01	1.13E-18<br>-3.64E-17<br>9.63E-16<br>6.35E-02<br>1.19E-19<br>5.744E-01	-2.09E-07<br>-4.43E-07<br>-5.04E-08<br>3.22E-03<br>-1.81E-07<br>9.866E-01	5.97E-07<br>4.57E-07<br>1.46E-06<br>6.16E-03<br>6.08E-07<br>8.638E-01	-7.19E-06<br>-6.18E-04<br>1.13E-04<br>9.71E-03<br>2.82E-05<br>8.521E-01
2phosphoglycerate<br>Local<br>Min<br>Max<br>Normalized peak height<br>Peak sensitivity value<br>Shapiro-Wilk score	9.33E-01<br>1.80E-01<br>1.34E+00<br>2.74E-03<br>1.06E+00<br>9.718E-01	6.64E-03<br>3.47E-03<br>1.37E-02<br>5.08E-03<br>6.41E-03<br>9.839E-01	1.97E-04<br>3.88E-05<br>1.03E-03<br>4.81E-03<br>1.22E-04<br>9.178E-01	1.43E-03<br>2.62E-04<br>8.67E-03<br>5.23E-03<br>7.54E-04<br>9.164E-01	1.99E-02<br>4.31E-04<br>1.18E-01<br>4.36E-03<br>6.14E-03<br>9.164E-01	6.66E-03<br>7.05E-04<br>2.14E-02<br>2.58E-03<br>4.21E-03<br>9.661E-01	1.18E-01<br>7.54E-03<br>5.46E-01<br>3.52E-03<br>4.71E-02<br>9.320E-01	6.94E-02<br>3.99E-03<br>3.21E-01<br>3.65E-03<br>3.05E-02<br>9.373E-01	6.11E-02<br>1.12E-02<br>1.24E-01<br>2.20E-03<br>7.37E-02<br>9.813E-01	-8.41E-17<br>-2.74E-14<br>2.63E-14<br>1.91E-01<br>6.41E-18<br>4.993E-01	9.52E-03<br>3.14E-03<br>2.47E-02<br>3.11E-03<br>8.32E-03<br>9.862E-01	-4.61E-01<br>-5.36E-01<br>-4.04E-01<br>1.94E-03<br>-4.48E-01<br>9.741E-01	-9.48E-02<br>-1.14E-01<br>-8.14E-02<br>3.11E-03<br>-9.62E-02<br>9.984E-01	-2.80E-02<br>-5.39E-02<br>-5.79E-03<br>2.50E-03<br>-3.43E-02<br>9.819E-01	9.32E-02<br>8.73E-03<br>3.86E-01<br>3.63E-03<br>6.48E-02<br>9.327E-01	-7.35E-01<br>-9.08E-01<br>-6.05E-01<br>3.03E-03<br>-7.23E-01<br>9.948E-01	7.25E-18<br>-3.57E-16<br>1.94E-17<br>4.07E-01<br>6.22E-21<br>7.498E-01	8.21E-20<br>-5.26E-18<br>3.19E-16<br>2.30E-01<br>9.92E-20<br>1.706E-01	2.73E-07<br>6.06E-08<br>6.32E-07<br>3.49E-03<br>2.19E-07<br>9.865E-01	6.99E-07<br>1.65E-07<br>1.66E-06<br>2.50E-03<br>7.80E-07<br>9.869E-01	-4.68E-04<br>-9.08E-04<br>-1.13E-04<br>3.09E-03<br>-4.59E-04<br>9.967E-01
glucose<br>Local<br>Min<br>Max<br>Normalized peak height<br>Peak sensitivity value<br>Shapiro-Wilk score	3.20E+00<br>1.28E+00<br>1.44E+01<br>4.67E-03<br>2.04E+00<br>9.186E-01	-1.04E+00<br>-1.05E+00<br>-7.11E-01<br>3.12E-02<br>-1.04E+00<br>5.771E-01	-1.17E-02<br>-2.80E-02<br>-5.94E-03<br>3.72E-03<br>-1.14E-02<br>9.675E-01	-7.39E-02<br>-1.86E-01<br>-3.61E-02<br>4.07E-03<br>-7.29E-02<br>9.604E-01	-1.69E-01<br>-1.72E+00<br>-1.29E-02<br>7.67E-03<br>-6.15E-02<br>8.425E-01	-2.68E-02<br>-1.66E-01<br>-1.42E-03<br>4.51E-03<br>-1.45E-02<br>9.113E-01	-6.92E-01<br>-6.80E+00<br>-4.27E-02<br>7.19E-03<br>-2.49E-01<br>8.541E-01	-4.06E-01<br>-4.14E+00<br>-2.75E-02<br>7.46E-03<br>-1.82E-01<br>8.607E-01	5.93E-02<br>-1.50E-02<br>1.53E+00<br>2.78E-02<br>5.82E-03<br>7.554E-01	2.20E-16<br>-1.16E-14<br>1.10E-14<br>1.92E-01<br>6.99E-19<br>8.258E-01	-5.57E-02<br>-2.96E-01<br>-2.06E-02<br>6.24E-03<br>-4.86E-02<br>9.107E-01	-6.44E-02<br>-1.90E-01<br>-8.18E-03<br>3.00E-03<br>-7.13E-02<br>9.858E-01	-1.33E-02<br>-3.73E-02<br>-1.71E-03<br>2.95E-03<br>-1.28E-02<br>9.875E-01	-5.89E-02<br>-1.11E+00<br>-7.72E-04<br>1.52E-02<br>-1.02E-02<br>7.920E-01	-5.45E-01<br>-3.53E+00<br>-4.67E-02<br>5.09E-03<br>-4.22E-01<br>8.915E-01	-1.03E-01<br>-3.31E-01<br>-1.21E-02<br>3.03E-03<br>-1.03E-01<br>9.827E-01	1.01E-18<br>-2.81E-17<br>3.86E-18<br>4.05E-01<br>2.95E-21<br>8.964E-01	-2.56E-19<br>-1.00E-15<br>3.20E-17<br>2.81E-01<br>3.50E-19<br>1.965E-01	-1.60E-06<br>-3.77E-06<br>-4.22E-07<br>3.10E-03<br>-1.37E-06<br>9.804E-01	6.79E-07<br>-7.94E-08<br>2.66E-05<br>3.92E-02<br>6.73E-08<br>6.996E-01	-9.85E-04<br>-1.04E-02<br>-1.27E-05<br>8.22E-03<br>-2.15E-04<br>8.535E-01
fructose6phosphate<br>Local<br>Min<br>Max<br>Normalized peak height<br>Peak sensitivity value<br>Shapiro-Wilk score	1.54E+00<br>1.00E+00<br>2.96E+00<br>2.77E-03<br>1.31E+00<br>9.545E-01	1.10E-02<br>4.12E-03<br>9.08E-02<br>9.56E-03<br>7.28E-03<br>8.233E-01	4.77E-03<br>2.48E-03<br>1.00E-02<br>3.84E-03<br>4.40E-03<br>9.825E-01	-1.07E+00<br>-1.11E+00<br>-1.05E+00<br>3.54E-03<br>-1.07E+00<br>9.926E-01	-1.09E-01<br>-3.32E-01<br>-5.09E-02<br>3.77E-03<br>-8.06E-02<br>9.322E-01	6.21E-03<br>-1.61E-02<br>1.55E-02<br>3.66E-03<br>8.96E-03<br>9.524E-01	-1.92E-01<br>-9.62E-01<br>-1.69E-02<br>4.01E-03<br>-8.35E-02<br>9.181E-01	-1.13E-01<br>-5.81E-01<br>-9.15E-03<br>4.21E-03<br>-5.40E-02<br>9.226E-01	1.40E-01<br>5.92E-02<br>4.08E-01<br>3.44E-03<br>1.32E-01<br>9.829E-01	4.79E-17<br>-1.90E-15<br>2.07E-15<br>1.83E-01<br>9.73E-19<br>8.455E-01	-1.51E-02<br>-4.09E-02<br>-5.41E-03<br>3.06E-03<br>-1.15E-02<br>9.697E-01	-1.75E-02<br>-3.53E-02<br>-2.12E-03<br>2.38E-03<br>-1.96E-02<br>9.848E-01	-3.59E-03<br>-7.14E-03<br>-4.62E-04<br>2.50E-03<br>-3.96E-03<br>9.847E-01	-6.96E-03<br>-1.40E-01<br>1.07E-02<br>1.41E-02<br>8.03E-04<br>8.173E-01	-1.48E-01<br>-7.98E-01<br>-1.18E-02<br>4.40E-03<br>-1.25E-01<br>9.221E-01	-2.78E-02<br>-5.99E-02<br>-3.26E-03<br>2.30E-03<br>-3.46E-02<br>9.849E-01	2.74E-19<br>-8.16E-18<br>9.16E-19<br>4.05E-01<br>3.88E-21<br>8.739E-01	2.79E-18<br>-8.75E-17<br>4.51E-15<br>9.70E-02<br>2.11E-18<br>4.000E-01	-4.33E-07<br>-9.32E-07<br>-1.09E-07<br>3.16E-03<br>-3.67E-07<br>9.861E-01	1.60E-06<br>1.18E-06<br>4.89E-06<br>7.25E-03<br>1.54E-06<br>8.632E-01	-1.16E-04<br>-1.54E-03<br>1.60E-04<br>8.80E-03<br>8.45E-06<br>8.592E-01
3phosphoglycerate<br>Local<br>Min<br>Max<br>Normalized peak height<br>Peak sensitivity value<br>Shapiro-Wilk score	3.53E+00<br>5.84E-01<br>8.13E+00<br>5.04E-03<br>3.25E+00<br>9.965E-01	2.51E-02<br>1.09E-02<br>9.16E-02<br>5.66E-03<br>2.23E-02<br>9.035E-01	7.47E-04<br>1.09E-04<br>5.16E-03<br>5.65E-03<br>3.09E-04<br>9.114E-01	5.43E-03<br>6.45E-04<br>3.71E-02<br>5.51E-03<br>2.63E-03<br>9.119E-01	7.54E-02<br>1.48E-03<br>5.01E-01<br>4.77E-03<br>2.52E-02<br>9.121E-01	2.52E-02<br>1.73E-03<br>1.02E-01<br>3.05E-03<br>9.27E-03<br>9.530E-01	4.48E-01<br>2.13E-02<br>2.35E+00<br>3.84E-03<br>1.62E-01<br>9.279E-01	2.63E-01<br>1.32E-02<br>1.44E+00<br>3.99E-03<br>8.25E-02<br>9.295E-01	2.31E-01<br>2.62E-02<br>8.20E-01<br>2.87E-03<br>1.35E-01<br>9.655E-01	2.58E-22<br>-2.51E-14<br>3.77E-14<br>3.71E-01<br>-1.71E-17<br>6.079E-01	3.60E-02<br>8.10E-03<br>1.57E-01<br>4.38E-03<br>2.99E-02<br>9.159E-01	-4.03E-01<br>-4.90E-01<br>-2.90E-01<br>2.97E-03<br>-3.90E-01<br>9.966E-01	-8.28E-02<br>-1.06E-01<br>-5.91E-02<br>3.61E-03<br>-8.26E-02<br>9.990E-01	-1.06E-01<br>-3.45E-01<br>-1.39E-02<br>2.96E-03<br>-1.16E-01<br>9.611E-01	-3.41E+00<br>-8.79E+00<br>-1.93E+00<br>3.99E-03<br>-2.88E+00<br>9.386E-01	-6.42E-01<br>-7.95E-01<br>-4.93E-01<br>3.22E-03<br>-6.24E-01<br>9.974E-01	6.33E-18<br>-3.38E-16<br>1.73E-17<br>4.10E-01<br>7.10E-20<br>8.259E-01	3.11E-19<br>-1.78E-17<br>1.99E-15<br>3.30E-01<br>-6.47E-19<br>1.447E-01	-9.98E-06<br>-2.94E-05<br>-2.00E-06<br>3.15E-03<br>-9.92E-06<br>9.803E-01	2.65E-06<br>4.26E-07<br>1.12E-05<br>3.57E-03<br>1.63E-06<br>9.491E-01	-1.77E-03<br>-6.43E-03<br>-2.95E-04<br>4.12E-03<br>-1.51E-03<br>9.672E-01
glycerol<br>Local<br>Min<br>Max<br>Normalized peak height<br>Peak sensitivity value<br>Shapiro-Wilk score	9.22E+00<br>1.43E+00<br>1.39E+01<br>5.99E-03<br>8.76E+00<br>9.908E-01	6.57E-02<br>2.42E-02<br>1.65E-01<br>3.81E-03<br>6.14E-02<br>9.795E-01	-2.07E-03<br>-6.01E-03<br>2.03E-03<br>5.60E-03<br>-1.54E-03<br>9.762E-01	-1.73E-02<br>-4.96E-02<br>1.47E-02<br>5.40E-03<br>-1.36E-02<br>9.755E-01	-4.20E-01<br>-1.08E+00<br>1.50E-01<br>4.87E-03<br>-3.47E-01<br>9.831E-01	-1.47E-01<br>-2.60E-01<br>-3.84E-02<br>4.41E-03<br>-1.34E-01<br>9.976E-01	-2.56E+00<br>-5.56E+00<br>-1.31E-01<br>4.71E-03<br>-2.12E+00<br>9.758E-01	-1.50E+00<br>-3.06E+00<br>-7.43E-02<br>4.38E-03<br>-1.20E+00<br>9.779E-01	-1.41E+00<br>-2.22E+00<br>-5.40E-01<br>2.74E-03<br>-1.52E+00<br>9.804E-01	1.05E-15<br>-1.60E-14<br>1.98E-14<br>1.42E-01<br>1.15E-17<br>9.730E-01	-2.06E-01<br>-5.78E-01<br>-3.13E-03<br>3.46E-03<br>-1.66E-01<br>9.774E-01	-2.39E-01<br>-3.60E-01<br>-1.74E-03<br>3.11E-03<br>-2.68E-01<br>9.578E-01	-4.91E-02<br>-7.61E-02<br>-3.45E-04<br>3.05E-03<br>-5.82E-02<br>9.558E-01	6.53E-01<br>2.65E-01<br>9.41E-01<br>1.80E-03<br>6.32E-01<br>9.690E-01	-2.02E+00<br>-5.32E+00<br>-1.27E-02<br>4.72E-03<br>-1.72E+00<br>9.930E-01	-3.80E-01<br>-5.67E-01<br>-2.42E-03<br>3.60E-03<br>-4.18E-01<br>9.494E-01	3.75E-18<br>-1.66E-16<br>1.27E-17<br>4.08E-01<br>-3.59E-20<br>7.884E-01	-1.82E-18<br>-3.17E-15<br>8.49E-17<br>1.11E-01<br>-1.42E-18<br>3.340E-01	-5.91E-06<br>-1.93E-05<br>-4.74E-08<br>2.89E-03<br>-3.56E-06<br>9.681E-01	-1.61E-05<br>-3.86E-05<br>-5.11E-06<br>3.17E-03<br>-1.51E-05<br>9.806E-01	-9.89E-01<br>-9.98E-01<br>-9.74E-01<br>2.49E-03<br>-9.90E-01<br>9.835E-01

**pyruvate**																					
Local	5.54E+00	3.94E-02	1.17E-03	8.51E-03	1.18E-01	3.96E-02	7.02E-01	4.12E-01	3.62E-01	-7.85E+00	5.65E-02	6.54E-02	1.35E-02	-1.66E-01	5.54E-01	1.04E-01	-1.03E-18	4.87E-19	1.62E-06	4.15E-06	-2.78E-03
Min	4.45E-01	1.31E-02	1.31E-04	8.28E-04	1.61E-03	1.99E-03	2.14E-02	1.33E-02	3.06E-02	-9.66E+03	9.17E-03	4.57E-03	9.60E-04	-2.34E+02	2.47E-02	6.87E-03	-1.39E-15	-1.03E-14	2.03E-07	5.14E-07	-3.55E+00
Max	5.57E+03	5.74E+01	2.78E+00	2.04E+01	2.90E+02	8.36E+01	1.59E+03	8.84E+02	5.19E+02	-3.48E+00	8.35E+01	8.75E+01	1.72E+01	-1.67E-02	7.24E+02	1.41E+02	4.31E-16	1.18E-14	2.65E-03	6.30E-03	-3.33E-04
Normalized peak height	6.37E-01	8.24E-01	8.19E-01	8.18E-01	7.89E-01	8.08E-01	7.90E-01	7.79E-01	7.34E-01	8.11E-01	7.66E-01	7.33E-01	7.20E-01	7.49E-01	6.63E-01	7.31E-01	8.39E-01	8.87E-01	8.29E-01	7.64E-01	7.47E-01
Peak sensitivity value	3.23E+00	4.18E-02	1.52E-03	1.10E-02	1.47E-01	4.38E-02	8.18E-01	4.55E-01	2.90E-01	-8.30E+00	5.09E-02	4.83E-02	9.57E-03	-1.34E-01	3.87E-01	7.72E-02	2.48E-19	-5.92E-19	1.53E-06	3.66E-06	-2.11E-03
Shapiro-Wilk score	4.728E-02	4.637E-02	4.732E-02	4.728E-02	4.596E-02	4.802E-02	4.632E-02	4.752E-02	5.048E-02	4.815E-02	5.065E-02	5.379E-02	5.325E-02	5.198E-02	5.422E-02	5.246E-02	3.886E-02	4.872E-02	4.474E-02	5.062E-02	5.010E-02
**dihydroxyacetonephosphate cytosol**																					
Local	-3.10E-01	-2.20E-03	-1.68E-04	-1.28E-03	-2.24E-02	-7.65E-03	-1.35E-01	-7.90E-02	6.75E-01	-6.46E-17	-1.08E-02	-1.25E-02	-2.58E-03	3.30E-02	-1.06E-01	-2.00E-02	1.97E-19	-9.46E-20	-3.10E-07	-8.20E-07	5.52E-04
Min	-1.13E+00	-8.38E-03	-9.98E-04	-7.48E-03	-1.57E-01	-3.02E-02	-7.07E-01	-4.03E-01	3.04E-01	-8.79E-16	-3.21E-02	-3.68E-02	-6.95E-03	4.09E-03	-6.66E-01	-6.35E-02	-1.04E-17	-7.62E-16	-1.04E-06	-1.98E-06	7.99E-05
Max	5.13E-01	9.29E-03	-1.56E-05	-1.05E-04	-2.65E-04	-4.78E-04	-4.84E-03	-3.02E-03	1.49E+00	8.39E-16	-2.23E-03	-1.11E-03	-2.15E-04	9.12E-02	-6.57E-03	-1.61E-03	7.63E-19	7.17E-18	-4.07E-08	-1.25E-07	1.42E-03
Normalized peak height	3.47E-03	6.90E-03	4.67E-03	4.55E-03	4.97E-03	2.91E-03	3.72E-03	4.01E-03	4.10E-03	1.14E-01	3.08E-03	3.23E-03	3.04E-03	2.70E-03	5.00E-03	3.41E-03	4.05E-01	4.24E-01	4.18E-03	2.45E-03	3.39E-03
Peak sensitivity value	-3.92E-01	-2.29E-03	-7.01E-05	-6.98E-04	-6.61E-03	-3.94E-03	-4.31E-02	-2.76E-02	5.96E-01	-1.38E-20	-8.72E-03	-1.11E-02	-2.28E-03	3.22E-02	-7.74E-02	-1.74E-02	-1.79E-21	-1.29E-19	-2.20E-07	-1.05E-06	4.93E-04
Shapiro-Wilk score	9.868E-01	9.410E-01	9.193E-01	9.219E-01	9.200E-01	9.603E-01	9.351E-01	9.375E-01	9.642E-01	9.248E-01	9.711E-01	9.799E-01	9.823E-01	9.803E-01	9.111E-01	9.763E-01	8.419E-01	1.278E-01	9.439E-01	9.837E-01	9.861E-01
**nadh**																					
Local	1.19E+00	8.44E-03	1.92E-04	1.37E-03	1.64E-02	1.21E-02	1.67E-01	-1.51E+00	-3.09E-02	2.01E-16	1.34E-02	1.55E-02	3.19E-03	-3.88E-02	1.31E-01	2.47E-02	-2.44E-19	7.06E-20	3.85E-07	-3.54E-07	-6.48E-04
Min	2.05E-01	4.33E-03	-1.12E-05	-2.08E-04	-1.52E-02	3.02E-03	9.83E-03	-1.72E+00	-2.17E-01	-1.34E-15	3.96E-03	1.96E-03	4.19E-04	-7.40E-02	1.05E-02	3.06E-03	-6.91E-19	-6.83E-18	7.40E-08	-1.74E-06	-1.20E-03
Max	1.77E+00	1.68E-02	1.37E-03	1.03E-02	1.48E-01	3.13E-02	7.43E-01	-1.19E+00	6.41E-02	1.28E-15	3.03E-02	3.04E-02	6.12E-03	-6.89E-03	5.64E-01	5.16E-02	6.58E-18	2.77E-16	8.97E-07	9.36E-07	-1.50E-04
Normalized peak height	2.78E-03	5.02E-03	5.09E-03	5.13E-03	4.36E-03	2.30E-03	3.26E-03	3.88E-03	3.84E-03	1.25E-01	2.67E-03	2.65E-03	2.69E-03	2.37E-03	3.66E-03	2.57E-03	4.08E-01	2.80E-01	3.42E-03	3.22E-03	3.07E-03
Peak sensitivity value	1.41E+00	7.98E-03	7.77E-05	4.40E-04	-1.77E-03	8.23E-03	6.59E-02	-1.48E+00	-3.34E-02	4.21E-19	1.16E-02	1.67E-02	3.60E-03	-4.55E-02	1.24E-01	2.80E-02	3.38E-21	1.17E-19	3.29E-07	-2.84E-07	-6.62E-04
Shapiro-Wilk score	9.662E-01	9.909E-01	9.141E-01	9.143E-01	9.169E-01	9.693E-01	9.340E-01	9.982E-01	9.897E-01	9.666E-01	9.873E-01	9.863E-01	9.847E-01	9.822E-01	9.362E-01	9.869E-01	8.966E-01	1.548E-01	9.825E-01	9.955E-01	9.959E-01
**glycerol3phosphate**																					
Local	9.63E-01	6.85E-03	2.45E-03	1.91E-02	3.66E-01	-4.66E-02	4.02E-01	2.36E-01	-2.28E+00	2.02E-16	3.25E-02	3.76E-02	7.73E-03	-1.21E-01	3.18E-01	5.99E-02	-5.90E-19	1.45E-18	9.31E-07	-2.61E-05	-2.02E-03
Min	-1.40E+00	-3.25E-02	1.16E-03	9.28E-03	2.29E-01	-7.35E-02	1.96E-02	1.19E-02	-3.39E+00	-2.81E-15	8.12E-03	4.44E-03	1.01E-03	-2.93E-01	2.46E-02	7.11E-03	-1.80E-18	-4.97E-17	1.42E-07	-4.54E-05	-4.45E-03
Max	2.45E+00	1.74E-02	5.56E-03	4.32E-02	7.61E-01	6.17E-03	1.73E+00	1.03E+00	-1.49E+00	2.15E-15	7.87E-02	8.45E-02	1.68E-02	-2.26E-02	1.64E+00	1.36E-01	2.21E-17	1.86E-15	2.54E-06	-1.43E-05	-3.77E-04
Normalized peak height	2.69E-03	6.79E-03	3.41E-03	3.63E-03	3.30E-03	3.04E-03	3.32E-03	3.27E-03	4.26E-03	1.10E-01	2.68E-03	2.87E-03	2.97E-03	2.25E-03	4.48E-03	2.60E-03	4.07E-01	8.68E-02	3.73E-03	3.09E-03	2.77E-03
Peak sensitivity value	1.56E+00	8.29E-03	2.28E-03	1.76E-02	3.31E-01	-5.22E-02	1.67E-01	1.08E-01	-2.14E+00	-8.32E-19	3.06E-02	3.54E-02	7.12E-03	-1.36E-01	2.21E-01	5.61E-02	4.32E-21	-8.81E-19	6.79E-07	-2.64E-05	-2.26E-03
Shapiro-Wilk score	9.680E-01	9.079E-01	9.714E-01	9.726E-01	9.623E-01	9.712E-01	9.412E-01	9.446E-01	9.887E-01	9.480E-01	9.884E-01	9.941E-01	9.948E-01	9.825E-01	9.314E-01	9.924E-01	8.389E-01	3.979E-01	9.711E-01	9.889E-01	9.932E-01
**glucose6phosphate**																					
Local	1.66E+00	1.18E-02	-1.68E-01	-1.04E+00	-1.04E-01	6.02E-03	-1.84E-01	-1.08E-01	1.35E-01	4.60E-17	-1.45E-02	-1.68E-02	-3.45E-03	-6.64E-03	-1.42E-01	-2.67E-02	2.64E-19	2.69E-18	-4.16E-07	1.54E-06	-1.11E-04
Min	1.08E+00	4.42E-03	-2.05E-01	-1.08E+00	-3.36E-01	-1.53E-02	-9.50E-01	-5.53E-01	5.47E-02	-2.05E-15	-3.88E-02	-3.47E-02	-6.92E-03	-1.30E-01	-6.95E-01	-5.54E-02	-3.77E-18	-8.49E-17	-9.04E-07	1.15E-06	-1.51E-03
Max	2.97E+00	1.11E-01	-1.35E-01	-1.01E+00	-4.97E-02	1.52E-02	-1.65E-02	-9.60E-03	3.33E-01	2.19E-15	-5.01E-03	-2.03E-03	-4.61E-04	8.83E-03	-1.20E-02	-3.16E-03	9.18E-19	4.79E-15	-1.10E-07	4.35E-06	1.36E-04
Normalized peak height	2.65E-03	1.11E-02	2.96E-03	3.20E-03	4.01E-03	3.63E-03	4.17E-03	3.91E-03	2.93E-03	1.64E-01	3.02E-03	2.64E-03	2.52E-03	1.36E-02	3.87E-03	2.31E-03	4.06E-01	9.44E-02	3.07E-03	6.51E-03	9.24E-03
Peak sensitivity value	1.35E+00	8.20E-03	-1.67E-01	-1.03E+00	-7.45E-02	8.86E-03	-6.83E-02	-4.79E-02	1.32E-01	-1.93E-18	-1.10E-02	-1.96E-02	-3.88E-03	7.03E-04	-9.23E-02	-3.01E-02	9.04E-22	4.10E-19	-3.44E-07	1.49E-06	1.31E-05
Shapiro-Wilk score	9.551E-01	8.268E-01	9.982E-01	9.918E-01	9.311E-01	9.523E-01	9.172E-01	9.223E-01	9.831E-01	8.440E-01	9.703E-01	9.855E-01	9.853E-01	8.175E-01	9.216E-01	9.855E-01	9.322E-01	3.769E-01	9.865E-01	8.676E-01	8.595E-01
**3phosphoglycerate cytosol**																					
Local	3.53E+00	2.51E-02	7.47E-04	5.43E-03	7.54E-02	2.52E-02	4.48E-01	2.63E-01	2.31E-01	0.00E+00	3.60E-02	-4.03E-01	-8.28E-02	-1.06E-01	-3.41E+00	-6.42E-01	6.33E-18	3.11E-19	1.03E-06	2.65E-06	-1.77E-03
Min	7.03E-01	1.06E-02	1.10E-04	7.26E-04	1.15E-03	1.67E-03	1.91E-02	1.16E-02	2.46E-02	-2.96E-14	8.70E-03	-4.89E-01	-1.04E-01	-3.48E-01	-8.93E+00	-7.91E-01	-1.92E-16	-2.13E-17	1.77E-07	4.15E-07	-5.62E-03
Max	7.39E+00	8.78E-02	4.67E-03	3.42E-02	5.10E-01	1.05E-01	2.45E+00	1.40E+00	8.44E-01	2.39E-14	1.59E-01	-2.93E-01	-5.82E-02	-1.40E-02	-1.89E+00	-4.87E-01	1.77E-17	1.66E-15	2.01E-06	1.15E-05	-2.45E-04
Normalized peak height	4.58E-03	5.65E-03	5.13E-03	5.12E-03	4.85E-03	3.30E-03	3.95E-03	3.86E-03	2.95E-03	3.69E-01	4.49E-03	2.83E-03	3.59E-03	3.12E-03	4.03E-03	3.30E-03	4.09E-01	3.05E-01	3.14E-03	3.94E-03	3.59E-03
Peak sensitivity value	3.33E+00	2.22E-02	3.95E-04	2.18E-03	2.48E-02	1.11E-02	1.40E-01	1.26E-01	1.41E-01	6.66E-18	2.50E-02	-3.99E-01	-8.29E-02	-1.14E-01	-3.01E+00	-6.25E-01	9.01E-21	-2.54E-19	1.07E-06	2.08E-06	-1.42E-03
Shapiro-Wilk score	9.967E-01	9.056E-01	9.082E-01	9.090E-01	9.097E-01	9.521E-01	9.258E-01	9.273E-01	9.649E-01	6.109E-01	9.143E-01	9.966E-01	9.988E-01	9.603E-01	9.387E-01	9.974E-01	8.509E-01	1.678E-01	9.912E-01	9.475E-01	9.664E-01
**phosphoenolpyruvate**																					
Local	4.22E-01	3.01E-03	8.93E-05	6.49E-04	9.02E-03	3.02E-03	5.36E-02	3.14E-02	2.76E-02	-1.05E-16	4.31E-03	-4.91E-01	-1.01E-01	-1.27E-02	4.22E-02	7.95E-03	7.72E-18	3.72E-20	1.24E-07	3.16E-07	-2.12E-04
Min	9.90E-02	1.71E-03	1.52E-05	9.84E-05	1.18E-04	2.23E-04	2.41E-03	1.36E-03	3.92E-03	-2.85E-14	1.32E-03	-5.74E-01	-1.21E-01	-2.37E-02	2.80E-03	7.93E-04	-5.59E-16	-2.35E-18	2.59E-08	6.20E-08	-4.06E-04
Max	6.36E-01	6.13E-03	4.82E-04	3.53E-03	5.45E-02	1.06E-02	2.65E-01	1.57E-01	5.76E-02	3.37E-14	1.02E-02	-4.30E-01	-8.65E-02	-2.13E-03	1.81E-01	1.50E-02	2.01E-17	1.24E-16	3.06E-07	7.33E-07	-4.10E-05
Normalized peak height	2.49E-03	4.87E-03	4.75E-03	4.65E-03	4.29E-03	2.75E-03	3.69E-03	3.67E-03	2.38E-03	2.26E-01	3.02E-03	1.91E-03	3.15E-03	2.61E-03	3.88E-03	2.54E-03	4.07E-01	2.07E-01	3.80E-03	2.57E-03	3.22E-03
Peak sensitivity value	4.74E-01	2.92E-03	4.77E-05	3.71E-04	2.65E-03	2.27E-03	2.35E-02	1.51E-02	3.17E-02	-1.68E-17	4.04E-03	-4.62E-01	-1.03E-01	-1.49E-02	3.62E-02	9.11E-03	1.66E-19	-7.36E-21	1.03E-07	2.97E-07	-2.14E-04
Shapiro-Wilk score	9.827E-01	9.859E-01	9.170E-01	9.152E-01	9.150E-01	9.674E-01	9.307E-01	9.367E-01	9.846E-01	5.013E-01	9.886E-01	9.735E-01	9.987E-01	9.850E-01	9.341E-01	9.881E-01	7.268E-01	1.749E-01	9.835E-01	9.893E-01	9.980E-01
**bisphosphoglycerate**																					
Local	-5.89E+00	-4.19E-02	5.08E-03	4.06E-02	8.47E-01	1.55E-01	3.68E+00	2.16E+00	4.96E-03	-1.01E-15	-8.02E-02	-9.29E-02	-1.91E-02	1.64E-01	-7.86E-01	-1.48E-01	1.46E-18	3.25E-18	-2.30E-06	5.67E-08	2.74E-03
Min	-1.12E+01	-2.08E-01	1.96E-03	1.64E-02	4.13E-01	8.44E-02	1.81E+00	1.01E+00	-6.43E-01	-1.68E-14	-7.07E-01	-4.07E-01	-7.91E-02	1.44E-02	-4.25E+00	-5.92E-01	-4.87E-17	-1.17E-16	-1.78E-05	-1.08E-05	2.67E-04
Max	-2.22E+00	-1.50E-02	1.23E-02	9.86E-02	1.75E+00	2.89E-01	7.35E+00	4.51E+00	4.27E-01	1.31E-14	-1.30E-02	-5.80E-03	-1.25E-03	5.40E-01	-3.13E-02	-8.60E-03	8.37E-18	3.56E-15	-2.49E-07	3.09E-06	8.55E-03
Normalized peak height	3.71E-03	6.32E-03	3.40E-03	3.67E-03	2.99E-03	3.57E-03	2.87E-03	3.23E-03	4.56E-03	1.34E-01	7.36E-03	3.21E-03	3.17E-03	2.69E-03	4.06E-03	3.02E-03	4.06E-01	7.33E-02	7.10E-03	5.05E-03	2.89E-03
Peak sensitivity value	-5.10E+00	-3.56E-02	4.53E-03	3.64E-02	8.08E-01	1.45E-01	3.39E+00	1.97E+00	5.14E-02	9.15E-18	-4.59E-02	-4.41E-02	-9.39E-03	1.10E-01	-2.69E-01	-5.38E-02	1.59E-20	-1.34E-18	-1.28E-06	9.80E-07	2.26E-03
Shapiro-Wilk score	9.849E-01	9.093E-01	9.775E-01	9.776E-01	9.774E-01	9.853E-01	9.803E-01	9.823E-01	9.646E-01	9.580E-01	8.684E-01	9.424E-01	9.437E-01	9.583E-01	9.234E-01	9.423E-01	8.522E-01	4.250E-01	8.539E-01	9.160E-01	9.744E-01
**atpg**																					
Local	-2.48E+00	-1.77E-02	1.11E-03	8.98E-03	1.98E-01	3.30E-02	8.27E-01	4.85E-01	-6.16E-02	-2.63E-16	6.66E-02	7.71E-02	1.58E-02	7.12E-02	6.52E-01	1.23E-01	-1.21E-18	-7.32E-18	1.91E-06	-7.06E-07	1.19E-03
Min	-2.06E+01	-9.92E-01	5.30E-05	4.57E-04	9.94E-03	1.98E-03	4.00E-02	2.52E-02	-2.73E+00	-1.59E-14	2.36E-02	8.48E-03	1.83E-03	9.83E-04	4.79E-02	1.10E-02	-5.76E-18	-1.34E-14	4.55E-07	-3.93E-05	1.89E-05
Max	-1.77E-01	-7.20E-04	1.21E-02	1.03E-01	2.96E+00	2.88E-01	1.11E+01	6.57E+00	3.21E-02	2.16E-14	4.36E-01	3.14E-01	6.40E-02	1.99E+00	4.72E+00	4.82E-01	8.45E-17	2.18E-16	5.53E-06	2.13E-07	1.57E-02
Normalized peak height	6.24E-03	4.18E-02	8.39E-03	9.09E-03	1.16E-02	6.52E-03	9.77E-03	1.03E-02	4.65E-02	2.02E-01	7.25E-03	3.61E-03	3.51E-03	2.31E-02	5.38E-03	3.32E-03	4.07E-01	1.02E-01	3.45E-03	5.24E-02	1.07E-02
Peak sensitivity value	-1.23E+00	-6.17E-03	3.83E-04	3.68E-03	7.34E-02	1.67E-02	2.88E-01	1.72E-01	3.76E-04	5.81E-18	4.61E-02	5.72E-02	1.10E-02	1.39E-02	3.40E-01	8.66E-02	-3.10E-20	6.20E-18	1.61E-06	-4.18E-09	1.83E-04
Shapiro-Wilk score	8.820E-01	6.525E-01	8.322E-01	8.237E-01	8.013E-01	8.753E-01	8.128E-01	8.196E-01	7.153E-01	7.798E-01	8.776E-01	9.668E-01	9.683E-01	7.531E-01	8.673E-01	9.633E-01	7.574E-01	3.538E-01	9.660E-01	6.608E-01	8.192E-01
**ampc**																					
Local	-1.22E+00	-8.69E-03	-2.58E-04	-1.87E-03	-2.61E-02	-8.72E-03	-1.55E-01	-9.08E-02	-7.98E-02	-6.07E-20	-1.24E-02	-1.44E-02	1.73E+00	3.66E-02	-1.22E-01	-2.30E-02	-3.42E-16	-1.07E-19	-3.57E-07	-9.14E-07	6.12E-04
Min	-1.67E+00	-1.82E-02	-1.40E-03	-1.09E-02	-1.49E-01	-2.76E-02	-7.33E-01	-4.18E-01	-1.51E-01	-5.47E-13	-2.84E-02	-2.66E-02	1.67E+00	6.88E-03	-5.04E-01	-4.24E-02	-8.51E-16	-6.90E-16	-8.24E-07	-2.03E-06	1.16E-04
Max	-2.93E-01	-5.09E-03	-4.82E-05	-3.41E-04	-7.36E-04	-8.16E-04	-9.68E-03	-6.06E-03	-1.29E-02	5.71E-13	-3.83E-03	-1.81E-03	1.77E+00	6.66E-02	-1.03E-02	-2.74E-03	4.13E-14	6.59E-18	-7.63E-08	-1.93E-07	1.10E-03
Normalized peak height	2.32E-03	5.13E-03	4.83E-03	5.09E-03	4.28E-03	2.49E-03	3.56E-03	3.34E-03	2.14E-03	3.10E-01	2.91E-03	2.85E-03	2.47E-03	2.47E-03	3.75E-03	2.56E-03	4.06E-01	3.47E-01	3.68E-03	2.50E-03	3.13E-03
Peak sensitivity value	-1.47E+00	-8.64E-03	-1.41E-04	-1.15E-03	-1.21E-02	-4.98E-03	-8.31E-02	-4.49E-02	-1.00E-01	1.02E-16	-1.36E-02	-1.63E-02	1.73E+00	4.36E-02	-1.11E-01	-2.68E-02	1.38E-17	-3.26E-20	-3.05E-07	-9.54E-07	5.88E-04
Shapiro-Wilk score	9.690E-01	9.863E-01	9.186E-01	9.170E-01	9.181E-01	9.688E-01	9.330E-01	9.393E-01	9.813E-01	4.947E-01	9.895E-01	9.837E-01	9.929E-01	9.810E-01	9.371E-01	9.856E-01	5.921E-01	1.481E-01	9.863E-01	9.895E-01	9.971E-01
**nad**																					
Local	-2.14E-02	-1.52E-04	-3.47E-06	-2.46E-05	-2.95E-04	-2.18E-04	-3.01E-03	2.72E-02	5.57E-04	-3.62E-18	-2.42E-04	-2.80E-04	-5.76E-05	6.99E-04	-2.37E-03	-4.46E-04	4.40E-21	-1.27E-21	-6.93E-09	6.38E-09	1.17E-05
Min	-3.86E-02	-3.50E-04	-2.62E-05	-1.97E-04	-3.17E-03	-7.14E-04	-1.65E-02	1.62E-02	-1.22E-03	-2.78E-17	-7.47E-04	-8.20E-04	-1.67E-04	8.41E-05	-1.15E-02	-1.38E-03	-5.38E-19	-1.66E-17	-2.16E-08	-2.06E-08	1.56E-06
Max	-5.02E-03	-6.56E-05	1.28E-07	3.32E-06	2.60E-04	-3.90E-05	-1.20E-04	4.56E-02	4.11E-03	2.88E-17	-4.87E-05	-2.35E-05	-5.06E-06	1.85E-03	-1.27E-04	-3.28E-05	1.57E-20	1.18E-19	-1.07E-09	3.36E-08	2.72E-05
Normalized peak height	3.43E-03	4.12E-03	5.52E-03	5.53E-03	5.58E-03	2.88E-03	4.16E-03	3.16E-03	4.33E-03	1.23E-01	3.11E-03	3.11E-03	3.19E-03	2.51E-03	3.98E-03	3.10E-03	4.08E-01	4.59E-01	3.81E-03	3.94E-03	3.08E-03
Peak sensitivity value	-2.02E-02	-1.41E-04	-1.15E-06	-6.97E-06	3.85E-05	-1.12E-04	-1.04E-03	2.61E-02	5.14E-04	2.15E-20	-2.17E-04	-2.87E-04	-5.98E-05	8.81E-04	-1.18E-03	-4.96E-04	-9.85E-23	-7.81E-21	-5.77E-09	6.88E-09	1.10E-05
Shapiro-Wilk score	9.990E-01	9.751E-01	8.924E-01	8.915E-01	8.901E-01	9.572E-01	9.127E-01	9.896E-01	9.913E-01	9.663E-01	9.755E-01	9.899E-01	9.912E-01	9.864E-01	9.251E-01	9.878E-01	6.025E-01	8.936E-02	9.694E-01	9.939E-01	9.942E-01
**fructose16bisphosphate**																					
Local	4.06E-01	2.89E-03	4.32E-03	3.39E-02	-1.00E-01	5.73E-03	-1.77E-01	-1.04E-01	1.29E-01	4.42E-17	-1.39E-02	-1.61E-02	-3.31E-03	-6.40E-03	-1.36E-01	-2.56E-02	2.53E-19	2.53E-18	-3.99E-07	1.48E-06	-1.07E-04
Min	-1.30E-01	-7.10E-04	2.32E-03	1.88E-02	-2.57E-01	-1.30E-02	-7.43E-01	-4.64E-01	5.18E-02	-1.34E-15	-3.44E-02	-3.07E-02	-5.97E-03	-9.05E-02	-6.78E-01	-5.09E-02	-5.16E-18	-8.03E-17	-8.84E-07	1.13E-06	-1.24E-03
Max	1.41E+00	4.02E-02	8.14E-03	5.96E-02	-4.86E-02	1.47E-02	-1.66E-02	-1.09E-02	3.29E-01	1.66E-15	-5.26E-03	-2.43E-03	-5.20E-04	9.71E-03	-1.20E-02	-3.79E-03	8.41E-19	3.57E-15	-9.77E-08	3.42E-06	1.30E-04
Normalized peak height	2.36E-03	9.06E-03	3.62E-03	3.76E-03	3.21E-03	3.30E-03	3.14E-03	3.60E-03	3.14E-03	1.82E-01	2.74E-03	2.56E-03	2.61E-03	9.99E-03	4.11E-03	2.35E-03	4.08E-01	8.99E-02	3.30E-03	5.28E-03	7.99E-03
Peak sensitivity value	1.57E-01	8.24E-04	4.19E-03	3.28E-02	-7.54E-02	8.57E-03	-7.22E-02	-4.83E-02	1.19E-01	-1.72E-19	-1.03E-02	-1.73E-02	-3.87E-03	5.47E-04	-1.20E-01	-2.94E-02	-1.99E-21	1.82E-18	-3.02E-07	1.48E-06	1.04E-05
Shapiro-Wilk score	9.586E-01	8.446E-01	9.854E-01	9.892E-01	9.445E-01	9.620E-01	9.298E-01	9.347E-01	9.846E-01	8.569E-01	9.778E-01	9.829E-01	9.811E-01	8.350E-01	9.305E-01	9.842E-01	9.159E-01	5.302E-01	9.860E-01	8.964E-01	8.723E-01
**ampg**																					
Local	1.67E+01	1.19E-01	-7.45E-03	-6.05E-02	-1.33E+00	-2.22E-01	-5.57E+00	-3.27E+00	4.15E-01	1.77E-15	-4.48E-01	-5.19E-01	-1.07E-01	-4.79E-01	-4.39E+00	-8.26E-01	8.15E-18	3.08E-16	-1.29E-05	4.75E-06	-8.01E-03
Min	4.66E+00	3.96E-02	-1.79E-02	-1.39E-01	-2.66E+00	-4.42E-01	-1.08E+01	-6.90E+00	-4.46E-01	-3.67E-14	-1.20E+00	-7.24E-01	-1.53E-01	-1.13E+00	-1.34E+01	-1.13E+00	-3.82E-16	-1.55E-13	-4.03E-05	-3.36E-06	-1.94E-02
Max	2.41E+01	3.34E-01	-9.25E-04	-1.34E-02	-4.70E-01	-5.68E-02	-2.02E+00	-1.18E+00	1.47E+00	4.25E-14	-5.63E-02	-3.34E-02	-6.93E-03	-4.48E-02	-2.56E-01	-4.99E-02	2.73E-17	3.21E-13	-9.69E-07	2.48E-05	-7.90E-04
Normalized peak height	5.02E-03	3.88E-03	4.14E-03	4.09E-03	3.59E-03	3.92E-03	3.40E-03	3.83E-03	2.42E-03	1.63E-01	3.58E-03	3.36E-03	3.39E-03	1.85E-03	5.61E-03	3.93E-03	4.07E-01	1.48E-01	2.74E-03	2.83E-03	2.61E-03
Peak sensitivity value	1.55E+01	1.20E-01	-6.37E-03	-5.16E-02	-1.22E+00	-1.91E-01	-5.00E+00	-2.91E+00	9.68E-02	-3.78E-17	-3.81E-01	-5.47E-01	-1.20E-01	-2.71E-01	-3.74E+00	-8.64E-01	1.34E-19	-1.98E-16	-9.28E-06	1.58E-06	-9.35E-03
Shapiro-Wilk score	9.979E-01	9.819E-01	9.758E-01	9.773E-01	9.876E-01	9.919E-01	9.808E-01	9.811E-01	9.564E-01	9.561E-01	9.797E-01	9.482E-01	9.469E-01	9.676E-01	9.879E-01	9.388E-01	8.108E-01	6.801E-01	9.729E-01	9.577E-01	9.843E-01

atpc	Local<br>Min<br>Max<br>Normalized peak height<br>Peak sensitivity value<br>Shapiro-Wilk score	1.92E-01<br>3.86E-02<br>3.06E-01<br>2.09E-03<br>2.21E-01<br>9.830E-01	1.36E-03<br>7.54E-04<br>2.96E-03<br>5.18E-03<br>1.36E-03<br>9.904E-01	4.05E-05<br>8.60E-06<br>2.26E-04<br>5.03E-03<br>2.59E-05<br>9.130E-01	2.94E-04<br>5.87E-05<br>1.86E-03<br>5.55E-03<br>2.05E-04<br>9.102E-01	4.09E-03<br>1.46E-04<br>2.69E-02<br>4.65E-03<br>1.90E-03<br>9.141E-01	1.37E-03<br>1.55E-04<br>4.49E-03<br>2.85E-03<br>1.08E-03<br>9.684E-01	2.43E-02<br>1.92E-03<br>1.24E-01<br>3.95E-03<br>1.33E-02<br>9.289E-01	1.43E-02<br>1.11E-03<br>6.92E-02<br>3.70E-03<br>7.88E-03<br>9.351E-01	1.25E-02<br>2.30E-03<br>2.40E-02<br>2.26E-03<br>1.42E-02<br>9.881E-01	2.20E-21<br>-1.24E-13<br>9.23E-14<br>3.14E-01<br>-1.91E-17<br>5.004E-01	1.95E-03<br>6.64E-04<br>4.71E-03<br>3.52E-03<br>1.94E-03<br>9.892E-01	2.26E-03<br>3.75E-04<br>4.10E-03<br>2.81E-03<br>2.33E-03<br>9.898E-01	-2.71E-01<br>-3.33E-01<br>-2.23E-01<br>2.47E-03<br>-2.70E-01<br>9.930E-01	-5.75E-03<br>-1.13E-02<br>-1.27E-03<br>2.79E-03<br>-6.56E-03<br>9.913E-01	1.91E-02<br>1.76E-03<br>7.81E-02<br>4.13E-03<br>1.63E-02<br>9.393E-01	3.61E-03<br>5.47E-04<br>6.74E-03<br>2.74E-03<br>3.84E-03<br>9.901E-01	9.62E-18<br>-4.46E-16<br>2.54E-17<br>4.06E-01<br>1.76E-19<br>8.070E-01	1.69E-20<br>-1.18E-18<br>7.54E-17<br>2.58E-01<br>6.96E-21<br>1.580E-01	5.61E-08<br>1.44E-08<br>1.33E-07<br>3.53E-03<br>4.95E-08<br>9.823E-01	1.44E-07<br>3.21E-08<br>3.31E-07<br>2.72E-03<br>1.34E-07<br>9.913E-01	-9.61E-05<br>-1.82E-04<br>-2.25E-05<br>3.23E-03<br>-8.55E-05<br>9.991E-01
glycerol3phosphate cytosol	Local<br>Min<br>Max<br>Normalized peak height<br>Peak sensitivity value<br>Shapiro-Wilk score	7.65E-01<br>-1.10E+00<br>1.79E+00<br>2.35E-03<br>1.16E+00<br>9.646E-01	5.45E-03<br>-2.41E-02<br>1.22E-02<br>6.18E-03<br>6.98E-03<br>9.010E-01	4.16E-04<br>5.38E-05<br>2.09E-03<br>4.30E-03<br>1.93E-04<br>9.289E-01	3.17E-03<br>3.50E-04<br>1.53E-02<br>4.06E-03<br>1.48E-03<br>9.288E-01	5.53E-02<br>1.28E-03<br>2.99E-01<br>3.92E-03<br>1.96E-02<br>9.261E-01	1.89E-02<br>1.66E-03<br>5.89E-02<br>2.44E-03<br>1.19E-02<br>9.682E-01	3.33E-01<br>1.97E-02<br>1.49E+00<br>3.30E-03<br>1.44E-01<br>9.385E-01	1.95E-01<br>1.17E-02<br>8.17E-01<br>3.12E-03<br>9.59E-02<br>9.435E-01	-1.67E+00<br>-2.00E+00<br>-1.32E+00<br>3.58E-03<br>-1.62E+00<br>9.989E-01	1.60E-16<br>-1.77E-15<br>1.81E-15<br>1.00E-01<br>-8.89E-19<br>9.476E-01	2.68E-02<br>7.67E-03<br>5.66E-02<br>2.44E-03<br>2.05E-02<br>9.891E-01	3.10E-02<br>3.94E-03<br>6.00E-02<br>2.77E-03<br>3.36E-02<br>9.881E-01	6.37E-03<br>8.36E-04<br>1.21E-02<br>2.89E-03<br>7.16E-03<br>9.871E-01	-8.17E-02<br>-1.82E-01<br>-1.44E-02<br>2.39E-03<br>-8.91E-02<br>9.867E-01	2.62E-01<br>2.34E-02<br>1.17E+00<br>3.86E-03<br>1.79E-01<br>9.367E-01	4.94E-02<br>5.43E-03<br>9.49E-02<br>2.57E-03<br>5.38E-02<br>9.887E-01	-4.87E-19<br>-1.59E-18<br>3.77E-17<br>4.07E-01<br>3.13E-21<br>7.126E-01	2.34E-19<br>-1.45E-17<br>3.77E-16<br>1.26E-01<br>1.91E-19<br>3.181E-01	7.68E-07<br>1.74E-07<br>1.83E-06<br>3.44E-03<br>6.06E-07<br>9.832E-01	2.03E-06<br>4.54E-07<br>5.37E-06<br>2.65E-03<br>1.60E-06<br>9.857E-01	-1.37E-03<br>-2.68E-03<br>-2.43E-04<br>3.06E-03<br>-1.37E-03<br>9.954E-01

Table S6: Concentration control coefficients for *Trypanosoma brucei* model with parameter variation of ±20%

		(glucosetransport)	(hexokinase)	(phosphoglycerateisomerase)	(phophofructokinase)	(aldolase)	(triosephosphateisomerase)	(glyceraldehyde3phosphatedehydrogenase)	(glycerol3phosphatedehydrogenase)	(glycerol3phosphateoxidase)	(pyruvatetransport)	(phosphoglyceratekinase)	(pyruvatekinase)	(atputilisation)	(glycerolkinase)	(phosphoglyceratemutase)	(enolase)	(adenylatekinasecytosol)	(adenylatekinaseglycosome)	(3phosphoglyceratetransport)	(gly3pdhapantiporter)	(glyceroltransport)
adpg	Local<br>Min<br>Max<br>Normalized peak height<br>Peak sensitivity value<br>Shapiro-Wilk score	7.11E+00<br>-1.49E+01<br>1.83E+01<br>7.06E-03<br>6.10E+00<br>8.762E-01	5.06E-02<br>-9.74E-01<br>1.42E-01<br>2.30E-02<br>3.53E-02<br>4.688E-01	-3.17E-03<br>-1.47E-02<br>1.25E-02<br>9.53E-03<br>-1.56E-03<br>9.674E-01	-2.57E-02<br>-1.22E-01<br>1.22E-01<br>9.90E-03<br>-1.67E-02<br>9.599E-01	-5.67E-01<br>-2.11E+00<br>2.50E+00<br>9.62E-03<br>-3.35E-01<br>8.743E-01	-9.47E-02<br>-3.36E-01<br>2.22E-01<br>6.41E-03<br>-6.84E-02<br>9.780E-01	-2.37E+00<br>-9.20E+00<br>8.62E+00<br>8.33E-03<br>-1.35E+00<br>9.188E-01	-1.39E+00<br>-5.66E+00<br>4.61E+00<br>8.27E-03<br>-9.29E-01<br>9.256E-01	1.77E-01<br>-2.56E+00<br>8.40E-01<br>1.39E-02<br>-9.04E-03<br>8.515E-01	7.55E-16<br>-7.00E-14<br>8.68E-14<br>4.04E-01<br>-1.21E-17<br>4.783E-01	-1.91E-01<br>-8.92E-01<br>4.12E-01<br>2.97E-03<br>-1.95E-01<br>9.867E-01	-2.21E-01<br>-4.06E-01<br>2.26E-01<br>3.34E-03<br>-2.34E-01<br>9.359E-01	-4.54E-02<br>-9.13E-02<br>4.85E-02<br>3.52E-03<br>-5.57E-02<br>9.321E-01	-2.04E-01<br>-5.03E-01<br>1.92E+00<br>1.08E-02<br>-2.62E-02<br>8.105E-01	-1.87E+00<br>-1.57E+01<br>1.28E+01<br>1.49E-02<br>-1.25E+00<br>9.293E-01	-3.52E-01<br>-6.49E-01<br>4.19E-01<br>4.09E-03<br>-3.58E-01<br>9.354E-01	3.47E-18<br>-1.64E-16<br>2.20E-17<br>3.90E-01<br>5.09E-22<br>7.977E-01	-1.01E-17<br>-6.78E-14<br>6.78E-16<br>3.12E-01<br>2.72E-17<br>1.130E-01	-5.47E-06<br>-4.07E-05<br>3.41E-06<br>7.68E-03<br>3.44E-08<br>9.278E-01	2.02E-06<br>-4.67E-05<br>1.80E-05<br>2.03E-02<br>-3.01E-07<br>7.813E-01	-3.41E-03<br>-1.07E-02<br>1.07E-02<br>5.83E-03<br>-7.03E-04<br>9.731E-01
adpc	Local<br>Min<br>Max<br>Normalized peak height<br>Peak sensitivity value<br>Shapiro-Wilk score	-5.14E-01<br>-7.55E-01<br>1.17E-02<br>2.84E-03<br>-6.26E-01<br>9.346E-01	-3.66E-03<br>-1.46E-02<br>5.48E-03<br>8.57E-03<br>-3.17E-03<br>9.169E-01	-1.09E-04<br>-1.47E-03<br>-6.35E-06<br>1.34E-02<br>-2.76E-05<br>8.035E-01	-7.90E-04<br>-1.12E-02<br>-3.73E-05<br>1.38E-02<br>-2.10E-04<br>7.885E-01	-1.10E-02<br>-1.24E-01<br>4.32E-04<br>1.55E-02<br>-3.76E-04<br>8.194E-01	-3.67E-03<br>-1.85E-02<br>-5.65E-05<br>5.01E-03<br>-5.28E-04<br>9.195E-01	-6.52E-02<br>-4.50E-01<br>-3.36E-04<br>8.09E-03<br>-5.05E-03<br>8.517E-01	-3.83E-02<br>-2.85E-01<br>-2.23E-04<br>8.21E-03<br>-3.50E-03<br>8.571E-01	-3.37E-02<br>-1.24E-01<br>-7.97E-04<br>3.13E-03<br>-9.47E-03<br>9.690E-01	0.00E+00<br>-7.84E-13<br>7.74E-13<br>6.15E-01<br>3.10E-16<br>3.145E-01	-5.25E-03<br>-2.86E-02<br>-5.46E-04<br>4.75E-03<br>-3.68E-03<br>9.083E-01	-6.07E-03<br>-1.62E-02<br>-1.04E-04<br>2.42E-03<br>-5.67E-03<br>9.784E-01	7.27E-01<br>5.92E-01<br>8.16E-01<br>2.50E-03<br>7.20E-01<br>9.919E-01	1.54E-02<br>3.32E-04<br>3.50E-02<br>2.31E-03<br>1.68E-02<br>9.765E-01	-5.14E-02<br>-7.17E-01<br>-3.54E-04<br>1.36E-02<br>-6.45E-03<br>6.596E-01	-9.68E-03<br>-2.85E-02<br>-1.24E-04<br>2.58E-03<br>-1.90E-03<br>9.730E-01	9.75E-18<br>-2.08E-16<br>4.33E-17<br>3.62E-01<br>-6.80E-20<br>9.093E-01	-4.52E-20<br>-1.49E-16<br>3.76E-18<br>3.64E-01<br>5.85E-21<br>2.533E-01	-1.50E-07<br>-6.18E-07<br>-6.94E-10<br>4.67E-03<br>-8.19E-08<br>9.364E-01	-3.85E-07<br>-1.43E-06<br>-2.20E-08<br>3.24E-03<br>-1.66E-07<br>9.713E-01	2.58E-04<br>8.25E-07<br>7.35E-04<br>3.17E-03<br>1.87E-04<br>9.790E-01
dihydroxyacetonephosphate	Local<br>Min<br>Max<br>Normalized peak height<br>Peak sensitivity value<br>Shapiro-Wilk score	-1.12E-01<br>-6.42E-01<br>4.90E-01<br>4.06E-03<br>-2.05E-01<br>9.609E-01	-8.00E-04<br>-1.48E-02<br>1.49E-02<br>1.70E-02<br>-9.12E-04<br>7.705E-01	1.87E-03<br>6.00E-04<br>6.62E-03<br>4.77E-03<br>1.59E-03<br>9.429E-01	1.47E-02<br>5.01E-03<br>5.30E-02<br>5.47E-03<br>1.35E-02<br>9.536E-01	2.89E-01<br>1.51E-01<br>5.57E-01<br>3.59E-03<br>2.73E-01<br>9.803E-01	-7.31E-02<br>-1.19E-01<br>-4.34E-02<br>3.45E-03<br>-6.94E-02<br>9.863E-01	-6.54E-02<br>-4.06E-01<br>-3.09E-03<br>7.89E-03<br>-7.72E-03<br>8.750E-01	-3.84E-02<br>-2.71E-01<br>-1.71E-03<br>9.21E-03<br>-5.35E-03<br>8.814E-01	6.36E-02<br>9.78E-03<br>7.23E-01<br>8.15E-03<br>3.65E-02<br>9.279E-01	-2.29E-17<br>-1.11E-15<br>1.26E-15<br>2.79E-01<br>5.71E-19<br>7.686E-01	-5.13E-03<br>-2.16E-02<br>-9.47E-04<br>4.10E-03<br>-2.78E-03<br>9.333E-01	-5.94E-03<br>-1.99E-02<br>-1.21E-04<br>2.84E-03<br>-2.25E-03<br>9.719E-01	-1.22E-03<br>-3.73E-03<br>-2.37E-05<br>2.65E-03<br>-3.92E-04<br>9.745E-01	-6.36E-03<br>-7.67E-02<br>2.98E-02<br>2.62E-02<br>-3.78E-04<br>8.307E-01	-5.03E-02<br>-7.13E-01<br>-4.48E-04<br>1.48E-02<br>-4.37E-03<br>6.881E-01	-9.47E-03<br>-3.45E-02<br>-1.54E-04<br>3.34E-03<br>-2.58E-03<br>9.657E-01	9.33E-20<br>-4.78E-18<br>3.83E-19<br>3.66E-01<br>-2.17E-21<br>8.127E-01	1.12E-18<br>-3.52E-17<br>2.96E-15<br>1.52E-01<br>-7.55E-19<br>2.578E-01	-1.47E-07<br>-6.95E-07<br>-9.33E-10<br>5.43E-03<br>-7.35E-08<br>9.282E-01	7.28E-07<br>3.76E-07<br>3.24E-06<br>6.23E-03<br>6.46E-07<br>8.623E-01	-1.06E-04<br>-1.01E-03<br>2.20E-04<br>1.53E-02<br>-8.19E-06<br>8.801E-01
glyceraldehyde3phosphate	Local<br>Min<br>Max<br>Normalized peak height<br>Peak sensitivity value<br>Shapiro-Wilk score	-1.73E-01<br>-7.05E-01<br>6.06E-01<br>4.00E-03<br>-3.21E-01<br>9.431E-01	-1.23E-03<br>-1.97E-02<br>1.69E-02<br>1.58E-02<br>-1.32E-03<br>8.125E-01	1.89E-03<br>6.15E-04<br>7.10E-03<br>5.15E-03<br>1.58E-03<br>9.442E-01	1.49E-02<br>4.97E-03<br>5.11E-02<br>5.01E-03<br>1.28E-02<br>9.539E-01	2.93E-01<br>1.45E-01<br>5.78E-01<br>3.58E-03<br>2.71E-01<br>9.796E-01	6.17E-02<br>3.12E-02<br>9.90E-02<br>3.55E-03<br>5.45E-02<br>9.940E-01	-9.23E-02<br>-5.19E-01<br>-3.62E-03<br>7.22E-03<br>-1.01E-02<br>8.808E-01	-5.41E-02<br>-3.38E-01<br>-1.77E-03<br>7.51E-03<br>-5.97E-03<br>8.866E-01	5.22E-02<br>9.34E-03<br>4.91E-01<br>7.30E-03<br>3.85E-02<br>8.962E-01	-3.55E-17<br>-7.03E-16<br>8.40E-16<br>2.13E-01<br>-1.05E-20<br>8.148E-01	-7.29E-03<br>-2.71E-02<br>-1.02E-03<br>3.72E-03<br>-3.90E-03<br>9.434E-01	-8.44E-03<br>-2.35E-02<br>-2.17E-04<br>2.51E-03<br>-2.00E-03<br>9.732E-01	-1.73E-03<br>-4.60E-03<br>-4.62E-05<br>2.43E-03<br>-4.77E-04<br>9.751E-01	-4.30E-04<br>-6.22E-02<br>3.09E-02<br>2.19E-02<br>7.69E-04<br>8.264E-01	-7.14E-02<br>-1.01E+00<br>-7.58E-04<br>1.50E-02<br>-9.31E-03<br>6.936E-01	-1.34E-02<br>-4.01E-02<br>-2.55E-04<br>2.78E-03<br>-2.62E-03<br>9.674E-01	1.32E-19<br>-1.11E-17<br>5.19E-19<br>3.66E-01<br>4.78E-22<br>5.798E-01	1.13E-18<br>-3.69E-17<br>1.77E-15<br>1.13E-01<br>1.06E-19<br>4.077E-01	-2.09E-07<br>-9.25E-07<br>-1.67E-09<br>5.09E-03<br>-8.25E-08<br>9.301E-01	5.97E-07<br>3.42E-07<br>3.17E-06<br>7.86E-03<br>5.79E-07<br>8.510E-01	-7.19E-06<br>-1.08E-03<br>4.00E-04<br>1.80E-02<br>1.51E-05<br>8.740E-01
2phosphoglycerate	Local<br>Min<br>Max<br>Normalized peak height<br>Peak sensitivity value<br>Shapiro-Wilk score	9.33E-01<br>-1.91E-02<br>1.57E+00<br>2.97E-03<br>1.10E+00<br>9.458E-01	6.64E-03<br>-1.01E-02<br>2.84E-02<br>8.63E-03<br>5.77E-03<br>9.129E-01	1.97E-04<br>1.11E-05<br>3.44E-03<br>1.72E-02<br>5.05E-05<br>8.036E-01	1.43E-03<br>6.21E-05<br>2.87E-02<br>1.91E-02<br>3.91E-04<br>7.889E-01	1.99E-02<br>-9.56E-04<br>2.39E-01<br>1.66E-02<br>6.06E-04<br>8.188E-01	6.66E-03<br>7.79E-05<br>3.80E-02<br>5.63E-03<br>8.56E-04<br>9.179E-01	1.18E-01<br>4.24E-04<br>9.18E-01<br>8.98E-03<br>9.14E-03<br>8.520E-01	6.94E-02<br>2.33E-04<br>5.81E-01<br>9.43E-03<br>7.50E-03<br>8.576E-01	6.11E-02<br>1.55E-03<br>2.57E-01<br>3.40E-03<br>1.75E-02<br>9.642E-01	-8.41E-17<br>-1.61E-13<br>1.60E-13<br>5.69E-01<br>-4.54E-17<br>3.120E-01	9.52E-03<br>8.29E-04<br>5.35E-02<br>4.79E-03<br>5.70E-03<br>9.006E-01	-4.61E-01<br>-6.44E-01<br>-3.58E-01<br>2.29E-03<br>-4.14E-01<br>9.690E-01	-9.48E-02<br>-1.37E-01<br>-7.02E-02<br>3.12E-03<br>-9.62E-02<br>9.957E-01	-2.80E-02<br>-7.10E-02<br>-9.03E-04<br>2.45E-03<br>-3.14E-02<br>9.765E-01	9.32E-02<br>5.95E-04<br>1.35E+00<br>1.48E-02<br>1.07E-02<br>6.579E-01	-7.35E-01<br>-1.10E+00<br>-4.97E-01<br>3.16E-03<br>-6.77E-01<br>9.852E-01	7.25E-18<br>-1.75E-16<br>3.32E-17<br>3.60E-01<br>1.04E-19<br>9.213E-01	8.21E-20<br>-1.55E-17<br>4.32E-16<br>4.44E-01<br>-2.85E-20<br>1.167E-01	2.73E-07<br>2.00E-09<br>1.15E-06<br>4.72E-03<br>1.34E-07<br>9.367E-01	6.99E-07<br>4.79E-08<br>2.87E-06<br>3.41E-03<br>2.61E-07<br>9.681E-01	-4.68E-04<br>-1.37E-03<br>-3.30E-06<br>3.19E-03<br>-2.58E-04<br>9.756E-01
glucose	Local<br>Min<br>Max<br>Normalized peak height<br>Peak sensitivity value<br>Shapiro-Wilk score	3.20E+00<br>1.12E+00<br>2.75E+01<br>1.25E-02<br>1.31E+00<br>8.407E-01	-1.04E+00<br>-1.07E+00<br>3.58E-01<br>5.97E-02<br>-1.04E+00<br>4.169E-01	-1.17E-02<br>-5.45E-02<br>-1.62E-03<br>5.45E-03<br>-9.64E-03<br>9.209E-01	-7.39E-02<br>-4.22E-01<br>-1.57E-02<br>6.30E-03<br>-5.86E-02<br>9.146E-01	-1.69E-01<br>-4.35E+00<br>-3.24E-03<br>3.03E-02<br>-1.85E-02<br>7.406E-01	-2.68E-02<br>-3.85E-01<br>9.09E-02<br>1.83E-02<br>-2.12E-03<br>8.084E-01	-6.92E-01<br>-1.41E+01<br>-5.30E-03<br>2.31E-02<br>-6.85E-02<br>7.474E-01	-4.06E-01<br>-9.20E+00<br>-3.26E-03<br>2.49E-02<br>-4.47E-02<br>7.491E-01	5.93E-02<br>-4.46E-01<br>3.49E+00<br>1.18E-01<br>5.19E-03<br>7.093E-01	2.20E-16<br>-1.29E-13<br>2.84E-13<br>6.71E-01<br>-1.36E-17<br>2.472E-01	-5.57E-02<br>-8.11E-01<br>-1.06E-02<br>1.43E-02<br>-2.94E-02<br>7.552E-01	-6.44E-02<br>-4.28E-01<br>-1.29E-03<br>5.26E-03<br>-1.77E-02<br>9.175E-01	-1.33E-02<br>-9.15E-02<br>-3.26E-04<br>5.12E-03<br>-3.65E-03<br>9.215E-01	-5.89E-02<br>-2.37E+00<br>-2.25E-06<br>1.02E-01<br>-1.19E-03<br>7.278E-01	-5.45E-01<br>-2.27E+01<br>-6.21E-03<br>3.98E-02<br>-6.29E-02<br>5.434E-01	-1.03E-01<br>-8.54E-01<br>-2.03E-03<br>6.62E-03<br>-2.38E-02<br>9.056E-01	1.01E-18<br>-2.68E-17<br>9.47E-18<br>3.64E-01<br>1.31E-20<br>8.574E-01	-2.56E-19<br>-7.57E-16<br>1.78E-13<br>9.93E-01<br>4.79E-17<br>9.539E-04	-1.60E-06<br>-6.95E-06<br>-7.53E-09<br>4.47E-03<br>-6.98E-07<br>9.358E-01	6.79E-07<br>-9.72E-07<br>8.37E-05<br>1.72E-01<br>8.63E-08<br>6.490E-01	-9.85E-04<br>-1.91E-02<br>-2.50E-08<br>4.62E-02<br>-2.86E-05<br>8.123E-01
fructose6phosphate	Local<br>Min<br>Max<br>Normalized peak height<br>Peak sensitivity value<br>Shapiro-Wilk score	1.54E+00<br>3.77E-01<br>6.84E+00<br>1.11E-02<br>1.09E+00<br>9.241E-01	1.10E-02<br>2.29E-03<br>1.10E+00<br>1.01E-01<br>6.13E-03<br>4.464E-01	4.77E-03<br>1.40E-03<br>2.43E-02<br>6.05E-03<br>4.14E-03<br>8.919E-01	-1.07E+00<br>-1.20E+00<br>-1.01E+00<br>4.87E-03<br>-1.07E+00<br>9.767E-01	-1.09E-01<br>-8.65E-01<br>-1.76E-02<br>9.43E-03<br>-6.55E-02<br>8.787E-01	6.21E-03<br>-7.42E-02<br>2.72E-02<br>9.24E-03<br>9.19E-03<br>9.249E-01	-1.92E-01<br>-3.09E+00<br>1.64E-01<br>2.14E-02<br>-2.34E-02<br>8.473E-01	-1.13E-01<br>-1.80E+00<br>8.02E-02<br>2.09E-02<br>-1.11E-02<br>8.478E-01	1.40E-01<br>2.72E-04<br>1.26E+00<br>7.24E-03<br>7.80E-02<br>9.257E-01	4.79E-17<br>-3.14E-14<br>3.51E-14<br>6.37E-01<br>-5.82E-18<br>3.029E-01	-1.51E-02<br>-2.18E-01<br>2.95E-03<br>1.40E-02<br>-8.20E-03<br>8.898E-01	-1.75E-02<br>-9.98E-02<br>1.48E-03<br>4.72E-03<br>-4.45E-03<br>9.689E-01	-3.59E-03<br>-2.24E-02<br>3.20E-04<br>4.89E-03<br>-9.43E-04<br>9.721E-01	-6.96E-03<br>-5.63E-01<br>5.95E-02<br>8.80E-02<br>6.20E-04<br>7.885E-01	-1.48E-01<br>-4.20E+00<br>1.90E-02<br>2.95E-02<br>-1.68E-02<br>6.596E-01	-2.78E-02<br>-1.91E-01<br>2.24E-03<br>5.92E-03<br>-6.34E-03<br>9.612E-01	2.74E-19<br>-1.06E-17<br>1.57E-18<br>3.62E-01<br>-4.06E-21<br>8.268E-01	2.79E-18<br>-1.67E-16<br>2.00E-14<br>3.42E-01<br>3.78E-18<br>9.593E-02	-4.33E-07<br>-1.95E-06<br>3.80E-08<br>4.89E-03<br>-1.90E-07<br>9.381E-01	1.60E-06<br>4.79E-09<br>1.76E-05<br>1.65E-02<br>1.41E-06<br>7.752E-01	-1.16E-04<br>-3.25E-03<br>6.34E-04<br>3.56E-02<br>6.67E-06<br>8.557E-01
3phosphoglycerate	Local<br>Min<br>Max<br>Normalized peak height<br>Peak sensitivity value<br>Shapiro-Wilk score	3.53E+00<br>-9.53E-02<br>2.10E+01<br>9.28E-03<br>2.94E+00<br>9.490E-01	2.51E-02<br>-3.71E-02<br>3.96E-01<br>1.93E-02<br>1.60E-02<br>6.064E-01	7.47E-04<br>2.88E-05<br>1.84E-02<br>2.61E-02<br>1.48E-04<br>7.484E-01	5.43E-03<br>1.62E-04<br>1.57E-01<br>3.05E-02<br>1.02E-03<br>7.482E-01	7.54E-02<br>-1.87E-03<br>1.86E+00<br>4.38E-02<br>9.22E-04<br>7.979E-01	2.52E-02<br>2.48E-04<br>2.65E-01<br>1.22E-02<br>2.77E-03<br>8.799E-01	4.48E-01<br>1.74E-03<br>7.54E+00<br>2.40E-02<br>2.81E-02<br>8.446E-01	2.63E-01<br>1.13E-03<br>4.04E+00<br>2.09E-02<br>1.53E-02<br>8.359E-01	2.31E-01<br>5.67E-03<br>2.04E+00<br>8.03E-03<br>4.12E-02<br>8.260E-01	2.58E-22<br>-8.49E-14<br>8.19E-14<br>4.58E-01<br>-6.26E-17<br>3.499E-01	3.60E-02<br>2.37E-03<br>9.14E-01<br>2.25E-02<br>1.47E-02<br>5.729E-01	-4.03E-01<br>-5.78E-01<br>-2.44E-02<br>4.20E-03<br>-3.87E-01<br>9.587E-01	-8.28E-02<br>-1.32E-01<br>-4.78E-03<br>4.85E-03<br>-8.76E-02<br>9.409E-01	-1.06E-01<br>-9.45E-01<br>-3.49E-03<br>7.71E-03<br>-2.75E-02<br>7.888E-01	-3.41E+00<br>-2.19E+01<br>-1.22E+00<br>8.59E-03<br>-2.10E+00<br>8.115E-01	-6.42E-01<br>-1.01E+00<br>-3.30E-02<br>4.90E-03<br>-5.80E-01<br>9.641E-01	6.33E-18<br>-7.79E-17<br>3.01E-17<br>3.59E-01<br>9.95E-21<br>9.347E-01	3.11E-19<br>-5.87E-17<br>1.99E-15<br>4.69E-01<br>-3.24E-19<br>9.787E-02	-9.98E-06<br>-8.33E-05<br>-2.17E-08<br>4.74E-03<br>-7.47E-06<br>9.350E-01	2.65E-06<br>1.04E-07<br>3.77E-05<br>1.20E-02<br>6.11E-07<br>7.627E-01	-1.77E-03<br>-1.48E-02<br>-1.60E-05<br>7.65E-03<br>-4.83E-04<br>8.095E-01

glycerol	Local	9.22E+00	6.57E-02	-2.07E-03	-1.73E-02	-4.20E-01	-1.47E-01	-2.56E+00	-1.50E+00	-1.41E+00	1.05E-15	-2.06E-01	-2.39E-01	-4.91E-02	6.53E-01	-2.02E+00	-3.80E-01	3.75E-18	-1.82E-18	-5.91E-06	-1.61E-05	-9.89E-01
	Min	-1.27E+00	-2.91E-01	-1.48E-02	-1.29E-01	-1.99E+00	-4.43E-01	-1.01E+01	-6.23E+00	-4.42E+00	-9.32E-14	-8.94E-01	-4.35E-01	-9.16E-02	1.57E-01	-1.53E+01	-7.05E-01	-5.51E-17	-1.24E-14	-3.57E-05	-6.93E-05	-9.99E-01
	Max	2.00E+01	2.95E-01	7.27E-03	6.28E-02	7.43E-01	1.71E-02	1.57E+00	8.48E-01	-2.73E-01	4.07E-14	7.43E-02	4.07E-02	9.32E-03	9.78E-01	1.69E+00	6.89E-02	1.87E-17	1.40E-16	5.92E-07	-1.65E-06	-9.60E-01
	Normalized peak height	4.65E-03	7.97E-03	8.78E-03	9.14E-03	6.91E-03	4.95E-03	6.01E-03	6.32E-03	4.71E-03	3.32E-01	3.21E-03	2.67E-03	2.56E-03	2.32E-03	9.41E-03	2.95E-03	3.76E-01	4.33E-01	6.69E-03	3.76E-03	4.20E-03
	Peak sensitivity value	7.62E+00	4.01E-02	-5.08E-04	-7.50E-03	-2.06E-01	-1.10E-01	-1.59E+00	-9.83E-01	-1.49E+00	3.74E-17	-1.36E-01	-2.45E-01	-5.61E-02	9.10E-01	-1.18E+00	-3.74E-01	1.08E-20	-4.13E-18	-6.70E-09	-9.32E-06	-9.97E-01
	Shapiro-Wilk score	9.652E-01	9.231E-01	9.665E-01	9.652E-01	9.749E-01	9.844E-01	9.705E-01	9.714E-01	9.802E-01	6.965E-01	9.533E-01	9.538E-01	9.487E-01	9.341E-01	9.083E-01	9.567E-01	8.693E-01	8.401E-02	9.187E-01	9.387E-01	9.460E-01
pyruvate	Local	5.54E+00	3.94E-02	1.17E-03	8.51E-03	1.18E-01	3.96E-02	7.02E-01	4.12E-01	3.62E-01	-7.85E+00	5.65E-02	6.54E-02	1.35E-02	-1.66E-01	5.54E-01	1.04E-01	-1.03E-18	4.87E-19	1.62E-06	4.15E-06	-2.78E-03
	Min	-2.26E-01	-7.71E-02	3.10E-05	1.80E-04	-6.89E-03	2.69E-04	1.63E-03	9.91E-04	5.06E-03	-8.58E+03	2.14E-03	4.48E-04	1.23E-04	-2.58E+02	1.69E-03	6.55E-04	-5.87E-16	-1.27E-14	6.51E-09	8.88E-08	-3.34E+00
	Max	5.74E+03	6.01E+01	6.40E+00	4.48E+01	5.93E+02	1.15E+02	3.18E+03	2.55E+03	8.56E+02	-2.37E+00	1.09E+02	9.02E+01	1.81E+01	-3.06E-03	1.82E+03	1.39E+02	6.76E-16	1.33E-14	3.28E-03	6.62E-03	-1.07E-05
	Normalized peak height	7.29E-01	6.77E-01	9.08E-01	9.01E-01	8.84E-01	8.68E-01	8.93E-01	9.26E-01	8.76E-01	7.85E-01	8.21E-01	7.98E-01	7.94E-01	8.18E-01	8.62E-01	7.88E-01	8.06E-01	8.79E-01	8.84E-01	8.01E-01	7.96E-01
	Peak sensitivity value	2.64E+00	1.32E-02	3.23E-03	2.26E-02	2.90E-01	5.78E-02	1.59E+00	1.27E+00	4.33E-01	-6.66E+00	5.68E-02	4.56E-02	9.19E-03	-1.32E-01	9.11E-01	7.02E-02	-2.24E-19	6.90E-18	1.65E-06	3.40E-06	-1.68E-03
	Shapiro-Wilk score	6.200E-02	5.901E-02	3.473E-02	3.512E-02	3.561E-02	4.250E-02	3.559E-02	3.295E-02	5.210E-02	6.015E-02	5.836E-02	6.075E-02	6.238E-02	5.664E-02	4.826E-02	6.286E-02	6.441E-02	3.874E-02	5.000E-02	5.325E-02	5.845E-02
dihydroxyacetonephosphate cytosol	Local	-3.10E-01	-2.20E-03	-1.68E-04	-1.28E-03	-2.24E-02	-7.65E-03	-1.35E-01	-7.90E-02	6.75E-01	-6.46E-17	-1.08E-02	-1.25E-02	-2.58E-03	3.30E-02	-1.06E-01	-2.00E-02	1.97E-19	-9.46E-20	-3.10E-07	-8.20E-07	5.52E-04
	Min	-2.73E+00	-2.38E-02	-4.60E-03	-3.15E-02	-3.83E-01	-8.92E-02	-1.44E+00	-1.19E+00	1.53E-01	-1.60E-15	-1.10E-01	-1.12E-01	-2.20E-02	4.99E-04	-2.78E+00	-2.25E-01	-7.78E-18	-7.23E-16	-4.10E-06	-5.81E-06	1.20E-05
	Max	1.35E+00	3.64E-02	8.81E-04	5.73E-03	1.89E-02	-3.95E-05	-2.88E-04	-1.34E-04	4.24E+00	2.00E-15	-4.01E-04	-8.47E-05	-1.49E-05	2.61E-01	-3.04E-04	-9.69E-05	1.41E-18	2.41E-17	-6.45E-10	-2.82E-08	3.42E-03
	Normalized peak height	6.54E-03	1.58E-02	4.16E-02	3.83E-02	3.63E-02	1.42E-02	1.62E-02	2.23E-02	9.05E-03	2.23E-01	9.01E-03	1.01E-02	9.01E-03	6.80E-03	3.77E-02	1.29E-02	3.69E-01	5.28E-01	1.62E-02	6.14E-03	6.02E-03
	Peak sensitivity value	-3.27E-01	-1.76E-03	-2.11E-05	-1.38E-04	-1.49E-04	-6.19E-04	-6.78E-03	-5.48E-03	4.00E-01	-1.79E-18	-3.19E-03	-1.26E-03	-4.44E-04	6.35E-03	-7.26E-03	-1.78E-03	1.69E-21	-1.42E-19	-5.19E-08	-1.93E-07	2.52E-04
	Shapiro-Wilk score	9.897E-01	8.919E-01	7.556E-01	7.748E-01	7.848E-01	8.540E-01	8.315E-01	8.160E-01	8.573E-01	7.529E-01	8.461E-01	8.355E-01	8.480E-01	9.044E-01	6.572E-01	8.177E-01	7.758E-01	1.217E-01	7.579E-01	9.440E-01	8.831E-01
nadh	Local	1.19E+00	8.44E-03	1.92E-04	1.37E-03	1.64E-02	1.21E-02	1.67E-01	-1.51E+00	-3.09E-02	2.01E-16	1.34E-02	1.55E-02	3.19E-03	-3.88E-02	1.31E-01	2.47E-02	-2.44E-19	7.06E-20	3.85E-07	-3.54E-07	-6.48E-04
	Min	-9.49E-02	-1.23E-02	-8.80E-05	-6.68E-04	-2.67E-02	1.45E-03	7.73E-04	-2.02E+00	-1.34E+00	-1.60E-15	9.30E-04	1.62E-04	3.56E-05	-1.27E-01	8.15E-04	1.97E-04	-1.21E-18	-9.85E-18	3.31E-09	-3.41E-06	-2.04E-03
	Max	2.38E+00	3.00E-02	3.02E-03	2.82E-02	2.60E-01	4.80E-02	1.03E+00	-1.02E+00	9.59E-02	1.42E-15	7.35E-02	5.51E-02	1.06E-02	-1.02E-03	1.81E+00	9.70E-02	1.42E-17	4.02E-16	1.78E-06	1.74E-06	-9.32E-06
	Normalized peak height	4.02E-03	7.81E-03	1.49E-02	1.79E-02	9.86E-03	4.21E-03	7.15E-03	4.09E-03	1.27E-02	1.96E-01	4.56E-03	2.84E-03	2.83E-03	2.80E-03	1.37E-02	3.32E-03	3.70E-01	3.51E-01	4.97E-03	3.78E-03	3.33E-03
	Peak sensitivity value	1.40E+00	7.50E-03	1.63E-05	3.94E-05	-6.72E-03	3.85E-03	1.57E-02	-1.44E+00	-2.70E-02	8.79E-19	6.92E-03	1.38E-02	2.94E-03	-3.84E-02	1.26E-02	4.21E-03	-5.95E-21	-1.80E-19	2.19E-07	-5.76E-07	-4.71E-04
	Shapiro-Wilk score	9.354E-01	9.299E-01	8.100E-01	8.010E-01	8.354E-01	9.307E-01	8.612E-01	9.968E-01	8.473E-01	8.870E-01	9.292E-01	9.743E-01	9.777E-01	9.787E-01	6.826E-01	9.668E-01	8.032E-01	1.775E-01	9.126E-01	9.973E-01	9.732E-01
glycerol3phosphate	Local	9.63E-01	6.85E-03	2.45E-03	1.91E-02	3.66E-01	-4.66E-02	4.02E-01	2.36E-01	-2.28E+00	2.02E-16	3.25E-02	3.76E-02	7.73E-03	-1.21E-01	3.18E-01	5.99E-02	-5.90E-19	1.45E-18	9.31E-07	-2.61E-05	-2.02E-03
	Min	-2.77E+00	-8.51E-02	6.45E-04	5.12E-03	1.65E-01	-9.32E-02	-4.22E-03	-2.40E-03	-6.95E+00	-7.33E-15	1.46E-03	3.22E-04	7.21E-05	-7.09E-01	1.09E-03	4.45E-04	-4.45E-18	-6.44E-17	2.20E-09	-8.30E-05	-7.42E-03
	Max	5.32E+00	1.23E-01	1.25E-02	8.49E-02	1.27E+00	5.30E-02	2.88E+00	1.84E+00	-1.10E+00	4.39E-15	1.52E-01	1.57E-01	3.06E-02	-4.19E-03	5.57E+00	2.97E-01	9.71E-18	2.58E-15	5.56E-06	-7.31E-06	-7.48E-05
	Normalized peak height	5.31E-03	1.87E-02	6.24E-03	5.35E-03	4.66E-03	4.30E-03	8.86E-03	9.36E-03	7.62E-03	2.76E-01	4.15E-03	3.90E-03	3.65E-03	4.42E-03	2.06E-02	4.98E-03	3.62E-01	1.30E-01	6.66E-03	4.03E-03	3.32E-03
	Peak sensitivity value	1.59E+00	7.36E-03	1.75E-03	1.50E-02	3.05E-01	-5.68E-02	2.89E-02	1.32E-02	-2.01E+00	-1.65E-18	1.66E-02	6.50E-03	1.43E-03	-3.42E-02	2.06E-02	9.49E-03	2.57E-21	4.74E-19	2.22E-07	-2.30E-05	-1.66E-03
	Shapiro-Wilk score	9.608E-01	8.677E-01	9.119E-01	9.251E-01	9.307E-01	9.652E-01	8.856E-01	8.828E-01	9.472E-01	8.213E-01	9.396E-01	9.379E-01	9.461E-01	9.453E-01	7.014E-01	9.260E-01	8.783E-01	2.991E-01	8.613E-01	9.638E-01	9.711E-01
glucose6phosphate	Local	1.66E+00	1.18E-02	-1.68E-01	-1.04E+00	-1.04E-01	6.02E-03	-1.84E-01	-1.08E-01	1.35E-01	4.60E-17	-1.45E-02	-1.68E-02	-3.45E-03	-6.64E-03	-1.42E-01	-2.67E-02	2.64E-19	2.69E-18	-4.16E-07	1.54E-06	-1.11E-04
	Min	4.90E-01	2.67E-03	-2.53E-01	-1.15E+00	-7.99E-01	-5.90E-02	-2.43E+00	-1.57E+00	-1.21E-02	-1.76E-14	-1.30E-01	-7.43E-02	-1.41E-02	-5.08E-01	-2.86E+00	-1.08E-01	-4.48E-18	-1.60E-16	-1.71E-06	-1.93E-07	-3.36E-03
	Max	5.73E+00	9.41E-01	-1.08E-01	-9.71E-01	-1.65E-02	2.23E-02	1.89E-01	1.43E-01	1.25E+00	3.82E-14	5.60E-03	2.78E-03	6.66E-04	7.37E-02	4.83E-02	4.43E-03	1.37E-18	3.26E-15	5.39E-08	1.28E-05	8.06E-04
	Normalized peak height	9.13E-03	8.26E-02	3.29E-03	3.78E-03	9.17E-03	8.25E-03	1.85E-02	2.01E-02	7.11E-03	5.81E-01	9.30E-03	3.84E-03	3.57E-03	8.02E-02	2.23E-02	3.85E-03	3.60E-01	1.16E-01	4.67E-03	1.31E-02	3.94E-02
	Peak sensitivity value	1.22E+00	5.96E-03	-1.64E-01	-1.03E+00	-6.07E-02	8.32E-03	-1.99E-02	-1.18E-02	8.06E-02	2.08E-17	-7.53E-03	-3.20E-03	-6.29E-04	7.12E-04	-1.71E-02	-4.50E-03	-7.28E-22	-1.32E-18	-1.80E-07	1.40E-06	7.85E-06
	Shapiro-Wilk score	9.254E-01	4.582E-01	9.943E-01	9.842E-01	8.788E-01	9.231E-01	8.466E-01	8.465E-01	9.235E-01	3.183E-01	8.952E-01	9.683E-01	9.720E-01	7.880E-01	6.611E-01	9.605E-01	8.892E-01	4.222E-01	9.373E-01	7.855E-01	8.510E-01
3phosphoglycerate cytosol	Local	3.53E+00	2.51E-02	7.47E-04	5.43E-03	7.54E-02	2.52E-02	4.48E-01	2.63E-01	2.31E-01	0.00E+00	3.60E-02	-4.03E-01	-8.28E-02	-1.06E-01	-3.41E+00	-6.42E-01	6.33E-18	3.11E-19	1.03E-06	2.65E-06	-1.77E-03
	Min	-1.08E-01	-4.13E-02	2.54E-05	1.40E-04	-1.80E-03	2.48E-04	9.27E-04	6.36E-04	5.64E-03	-8.92E-14	2.39E-03	-5.85E-01	-1.28E-01	-1.02E+00	-2.04E+01	-9.70E-01	-1.89E-16	-4.01E-17	3.35E-08	1.09E-07	-1.54E-02
	Max	1.82E+01	4.03E-01	2.01E-02	1.52E-01	1.61E+00	2.55E-01	6.61E+00	3.94E+00	2.16E+00	1.50E-13	8.55E-01	-1.27E-02	-3.20E-03	-3.63E-03	-1.23E+00	-2.51E-02	2.88E-17	4.07E-15	2.96E-06	3.72E-05	-7.51E-06
	Normalized peak height	7.82E-03	1.98E-02	2.85E-02	3.04E-02	3.66E-02	1.19E-02	2.05E-02	2.12E-02	8.53E-03	5.00E-01	2.03E-02	4.35E-03	4.68E-03	8.20E-03	7.55E-03	4.86E-03	3.58E-01	5.96E-01	3.29E-03	1.19E-02	8.11E-03
	Peak sensitivity value	2.94E+00	1.67E-02	1.36E-04	9.74E-04	6.11E-04	2.16E-03	3.07E-02	1.44E-02	3.69E-02	6.82E-17	1.56E-02	-3.89E-01	-8.81E-02	-2.35E-02	-2.14E+00	-5.99E-01	-5.00E-20	-1.01E-18	8.49E-07	5.73E-07	-4.45E-04
	Shapiro-Wilk score	9.521E-01	6.083E-01	7.501E-01	7.485E-01	7.968E-01	8.806E-01	8.448E-01	8.353E-01	8.300E-01	3.420E-01	5.740E-01	9.597E-01	9.425E-01	7.950E-01	8.167E-01	9.639E-01	9.045E-01	5.069E-02	9.847E-01	7.707E-01	8.142E-01
phosphoenolpyruvate	Local	4.22E-01	3.01E-03	8.93E-05	6.49E-04	9.02E-03	3.02E-03	5.36E-02	3.14E-02	2.76E-02	-1.05E-16	4.31E-03	-4.91E-01	-1.01E-01	-1.27E-02	4.22E-02	7.95E-03	7.72E-18	3.72E-20	1.24E-07	3.16E-07	-2.12E-04
	Min	-7.44E-03	-5.33E-03	6.01E-06	3.89E-05	-3.03E-04	4.81E-05	3.38E-04	1.60E-04	1.31E-03	-1.68E-13	5.29E-04	-6.80E-01	-1.46E-01	-3.26E-02	3.49E-04	1.31E-04	-3.38E-16	-4.32E-18	1.26E-09	2.05E-08	-6.74E-04
	Max	7.47E-01	1.15E-02	1.55E-03	1.24E-02	1.07E-01	1.70E-02	4.41E-01	2.70E-01	1.18E-01	1.80E-13	2.49E-02	-3.79E-01	-7.53E-02	-5.95E-04	6.77E-01	2.45E-02	3.53E-17	1.03E-16	5.56E-07	1.16E-06	-1.49E-06
	Normalized peak height	2.31E-03	8.22E-03	1.70E-02	1.81E-02	1.57E-02	5.19E-03	9.24E-03	9.22E-03	3.43E-03	5.32E-01	5.36E-03	2.13E-03	3.08E-03	2.51E-03	1.60E-02	2.57E-03	3.62E-01	3.36E-01	4.94E-03	2.96E-03	3.67E-03
	Peak sensitivity value	4.99E-01	2.84E-03	2.53E-05	1.93E-04	2.86E-04	3.11E-04	3.64E-03	2.72E-03	6.74E-03	8.64E-17	2.93E-03	-4.28E-01	-1.05E-01	-1.38E-02	5.43E-03	1.60E-03	4.46E-21	1.33E-20	6.81E-08	1.09E-07	-1.32E-04
	Shapiro-Wilk score	9.681E-01	9.202E-01	7.966E-01	7.806E-01	8.128E-01	9.147E-01	8.457E-01	8.510E-01	9.653E-01	2.981E-01	9.022E-01	9.672E-01	9.956E-01	9.794E-01	6.538E-01	9.720E-01	8.874E-01	1.883E-01	9.273E-01	9.682E-01	9.744E-01
bisphosphoglycerate	Local	-5.89E+00	-4.19E-02	5.08E-03	4.06E-02	8.47E-01	1.55E-01	3.68E+00	2.16E+00	4.96E-03	-1.01E-15	-8.02E-02	-9.29E-02	-1.91E-02	1.64E-01	-7.86E-01	-1.48E-01	1.46E-18	3.25E-18	-2.30E-06	5.67E-08	2.74E-03
	Min	-1.76E+01	-3.38E-01	3.54E-04	5.19E-03	2.58E-01	3.17E-02	1.18E+00	5.49E-01	-1.04E+00	-5.10E-14	-9.78E-01	-5.03E-01	-1.03E-01	-1.06E-01	-1.15E+01	-7.70E-01	-2.12E-17	-2.05E-16	-4.19E-05	-2.34E-05	-1.58E-03
	Max	5.82E-01	2.18E-02	2.62E-02	1.98E-01	2.88E+00	4.80E-01	1.13E+01	7.46E+00	2.84E+00	6.15E-14	-5.12E-03	-6.82E-04	-1.68E-04	8.18E-01	-2.72E-03	-9.16E-04	1.97E-17	8.00E-15	-6.11E-09	9.37E-06	1.43E-02
	Normalized peak height	4.84E-03	8.68E-03	5.60E-03	5.63E-03	4.11E-03	5.21E-03	3.56E-03	4.32E-03	1.51E-02	3.51E-01	1.18E-02	6.12E-03	5.87E-03	6.02E-03	2.29E-02	6.76E-03	3.63E-01	1.84E-01	2.01E-02	1.15E-02	6.21E-03
	Peak sensitivity value	-4.31E+00	-2.16E-02	3.05E-03	2.88E-02	5.48E-01	1.15E-01	2.31E+00	1.47E+00	5.06E-02	3.52E-17	-1.83E-02	-9.47E-03	-2.47E-03	2.89E-02	-3.16E-02	-1.28E-02	-5.88E-21	3.99E-18	-4.46E-07	1.13E-06	5.53E-04
	Shapiro-Wilk score	9.795E-01	7.768E-01	9.230E-01	9.303E-01	9.495E-01	9.419E-01	9.557E-01	9.531E-01	9.407E-01	7.027E-01	7.493E-01	8.849E-01	8.857E-01	9.057E-01	8.292E-01	8.858E-01	8.045E-01	2.737E-01	7.126E-01	8.628E-01	9.020E-01
atpg	Local	-2.48E+00	-1.77E-02	1.11E-03	8.98E-03	1.98E-01	3.30E-02	8.27E-01	4.85E-01	-6.16E-02	-2.63E-16	6.66E-02	7.71E-02	1.58E-02	7.12E-02	6.52E-01	1.23E-01	-1.21E-18	-7.32E-18	1.91E-06	-7.06E-07	1.19E-03
	Min	-3.49E+01	-2.07E+00	-2.64E-02	-1.40E-01	1.17E-03	-1.36E-01	4.85E-03	3.21E-03	-5.37E+00	-1.90E-13	1.11E-02	1.38E-03	3.54E-04	6.01E-05	5.09E-03	1.83E-03	-1.16E-17	-1.96E-13	4.66E-08	-1.19E-04	8.02E-07
	Max	-4.08E-02	-1.57E-04	3.44E-02	3.61E-01	6.31E+00	5.38E-01	1.99E+01	1.10E+01	5.27E-01	1.66E-13	9.96E-01	5.45E-01	1.20E-01	3.99E+00	3.37E+01	1.05E+00	2.34E-17	7.92E-16	1.02E-05	1.77E-06	2.88E-02
	Normalized peak height	1.51E-02	1.34E-01	7.17E-02	6.84E-02	4.10E-02	2.24E-02	2.96E-02	2.69E-02	1.73E-01	6.28E-01	1.56E-02	5.63E-03	5.83E-03	1.40E-01	5.23E-02	6.96E-03	3.63E-01	5.39E-01	5.17E-03	2.49E-01	5.94E-02
	Peak sensitivity value	-2.67E-01	-1.19E-03	6.80E-05	6.22E-04	1.69E-02	3.42E-03	7.46E-02	4.17E-02	-5.47E-04	-2.54E-17	3.33E-02	1.58E-02	4.49E-03	2.06E-03	5.56E-02	2.33E-02	-6.85E-21	-9.60E-17	8.11E-07	2.34E-08	1.52E-05
	Shapiro-Wilk score	8.231E-01	4.909E-01	8.025E-01	7.846E-01	7.316E-01	7.896E-01	7.366E-01	7.394E-01	7.047E-01	3.142E-01	7.544E-01	8.920E-01	8.964E-01	7.222E-01	5.327E-01	8.805E-01	8.498E-01	2.356E-02	9.168E-01	6.500E-01	7.941E-01
ampc	Local	-1.22E+00	-8.69E-03	-2.58E-04	-1.87E-03	-2.61E-02	-8.72E-03	-1.55E-01	-9.08E-02	-7.98E-02	-6.07E-20	-1.24E-02	-1.44E-02	1.73E+00	3.66E-02	-1.22E-01	-2.30E-02	-3.42E-16	-1.07E-19	-3.57E-07	-9.14E-07	6.12E-04
	Min	-1.73E+00	-3.19E-02	-3.86E-03	-3.37E-02	-2.87E-01	-4.25E-02	-1.08E+00	-6.77E-01	-3.87E-01	-3.62E-12	-7.25E-02	-4.03E-02	1.59E+00	1.68E-03	-1.67E+00	-6.94E-02	-9.70E-16	-8.31E-16	-1.51E-06	-3.30E-06	3.55E-06
	Max	5.80E-02	1.70E-02	-1.68E-05	-9.83E-05	1.07E-03	-1.61E-04	-8.99E-04	-4.98E-04	-3.61E-03	1.83E-12	-1.38E-03	-2.37E-04	1.81E+00	8.80E-02	-9.68E-04	-2.93E-04	1.95E-14	9.98E-18	-3.07E-09	-6.84E-08	1.69E-03
	Normalized peak height	3.50E-03	9.03E-03	1.46E-02	1.70E-02	1.46E-02	4.90E-03	8.00E-03	8.37E-03	3.85E-03	6.19E-01	5.18E-03	2.43E-03	2.60E-03	2.46E-03	1.34E-02	2.69E-03	3.66E-01	4.72E-01	4.89E-03	3.00E-03	3.13E-03
	Peak sensitivity value	-1.59E+00	-7.88E-03	-6.87E-05	-5.19E-04	-5.12E-04	-1.16E-03	-1.12E-02	-8.28E-03	-9.00E-02	2.00E-15	-1.07E-02	-1.60E-02	1.72E+00	3.67E-02	-1.85E-02	-2.11E-02	3.42E-19	3.14E-19	-1.80E-07	-3.67E-07	4.58E-04
	Shapiro-Wilk score	9.245E-01	9.185E-01	8.029E-01	7.849E-01	8.181E-01	9.197E-01	8.503E-01	8.560E-01	9.705E-01	3.002E-01	9.085E-01	9.790E-01	9.914E-01	9.764E-01	6.637E-01	9.741E-01	8.334E-01	1.185E-01	9.379E-01	9.727E-01	9.793E-01
nad	Local	-2.14E-02	-1.52E-04	-3.47E-06	-2.46E-05	-2.95E-04	-2.18E-04	-3.01E-03	2.72E-02	5.57E-04	-3.62E-18	-2.42E-04	-2.80E-04	-5.76E-05	6.99E-04	-2.37E-03	-4.46E-04	4.40E-21	-1.27E-21	-6.93E-09	6.38E-09	1.17E-05
	Min	-6.34E-02	-8.31E-04	-7.22E-05	-5.66E-04	-6.94E-03	-1.55E-03	-3.24E-02	9.31E-03	-2.74E-03	-4.65E-17	-1.98E-03	-1.48E-03	-3.11E-04	1.67E-05	-5.01E-02	-3.07E-03	-3.71E-20	-1.61E-17	-4.88E-08	-5.81E-08	1.39E-07
	Max	9.19E-04	1.64E-04	2.42E-06	1.68E-05	6.17E-04	-1.25E-05	-1.21E-05	7.19E-02	2.48E-02	4.10E-17	-1.21E-05	-2.55E-06	-5.76E-07	3.66E-03	-1.09E-05	-3.49E-06	3.09E-20	2.04E-19	-2.77E-11	8.27E-08	5.67E-05
	Normalized peak height	3.61E-03	7.70E-03	2.51E-02	2.57E-02	1.75E-02	7.17E-03	1.55E-02	3.96E-03	1.67E-02	2.59E-01	6.39E-03	4.59E-03	4.38E-03	4.34E-03	2.50E-02	6.32E-03	3.60E-01	6.02E-01	7.19E-03	6.77E-03	4.65E-03
	Peak sensitivity value	-1.66E-02	-1.08E-04	-8.02E-08	-3.61E-07	6.92E-05	-5.03E-05	-1.25E-04	2.15E-02	3.34E-04	9.77E-21	-8.78E-05	-4.31E-05	-7.57E-06	1.93E-04	-1.86E-04	-5.41E-05	2.13E-23	7.61E-22	-2.39E-09	7.30E-09	2.77E-06
	Shapiro-Wilk score	9.882E-01	8.837E-01	7.716E-01	7.645E-01	7.943E-01	8.997E-01	8.252E-01	9.571E-01	8.190E-01	8.629E-01	8.787E-01	9.326E-01	9.403E-01	9.453E-01	6.673E-01	9.194E-01	1.000E+00	1.048E-01	8.549E-01	9.927E-01	9.277E-01
fructose16bisphosphate	Local	4.06E-01	2.89E-03	4.32E-03	3.39E-02	-1.00E-01	5.73E-03	-1.77E-01	-1.04E-01	1.29E-01	4.42E-17	-1.39E-02	-1.61E-02	-3.31E-03	-6.40E-03	-1.36E-01	-2.56E-02	2.53E-19	2.53E-18	-3.99E-07	1.48E-06	-1.07E-04
	Min	-6.72E-01	-8.01E-02	1.33E-03	1.04E-02	-3.80E-01	-2.31E-02	-9.71E-01	-7.51E-01	7.85E-03	-2.54E-15	-6.31E-02	-5.10E-02	-9.81E-03	-1.28E-01	-1.94E+00	-8.80E-02	-6.34E-18	-8.23E-17	-1.57E-06	2.19E-07	-2.12E-03
	Max	1.97E+00	5.82E-02	1.54E-02	1.38E-01	-2.31E-02	2.43E-02	7.15E-02	3.80E-02	1.47E+00	3.09E-15	2.00E-03	9.22E-04	1.82E-04	7.82E-02	4.81E-02	1.45E-03	1.74E-18	8.74E-15	1.81E-08	6.32E-06	8.25E-04
	Normalized peak height	5.08E-03	5.00E-02	4.71E-03	5.51E-03	4.84E-03	5.02E-03	7.88E-03	9.66E-03	9.04E-03	3.39E-01	4.94E-03	2.99E-03	2.66E-03	3.79E-02	1.60E-02	3.34E-03	3.63E-01	2.08E-01	4.61E-03	6.97E-03	2.89E-02
	Peak sensitivity value	1.35E-02	3.50E-05	3.37E-03	3.09E-02	-6.03E-02	8.36E-03	-1.76E-02	-1.21E-02	8.62E-02	-1.48E-18	-7.54E-03	-3.42E-03	-1.10E-03	2.89E-04	-1.45E-02	-4.41E-03	-8.40E-22	1.51E-18	-1.85E-07	1.33E-06	6.43E-06
	Shapiro-Wilk score	9.283E-01	7.820E-01	9.341E-01	9.407E-01	9.173E-01	9.559E-01	8.783E-01	8.797E-01	8.924E-01	6.548E-01	9.597E-01	9.708E-01	9.734E-01	8.376E-01	7.040E-01	9.639E-01	8.526E-01	2.444E-01	9.407E-01	9.032E-01	8.604E-01

ampg	Local	1.67E+01	1.19E-01	-7.45E-03	-6.05E-02	-1.33E+00	-2.22E-01	-5.57E+00	-3.27E+00	4.15E-01	1.77E-15	-4.48E-01	-5.19E-01	-1.07E-01	-4.79E-01	-4.39E+00	-8.26E-01	8.15E-18	3.08E-16	-1.29E-05	4.75E-06	-8.01E-03
	Min	1.12E-01	1.25E-02	-3.26E-02	-2.61E-01	-4.40E+00	-7.42E-01	-1.96E+01	-1.24E+01	-3.19E+00	-1.23E-13	-1.78E+00	-8.73E-01	-1.87E-01	-1.38E+00	-3.37E+01	-1.48E+00	-1.35E-16	-9.96E-13	-8.20E-05	-8.93E-06	-3.24E-02
	Max	3.72E+01	4.88E-01	7.78E-03	4.31E-02	-2.48E-02	5.60E-02	-2.66E-02	-1.27E-02	2.76E+00	1.01E-13	-8.33E-04	-1.97E-04	-6.05E-05	-5.40E-03	-7.88E-04	-3.05E-04	4.98E-17	7.05E-13	-3.86E-09	5.14E-05	-1.60E-05
	Normalized peak height	4.31E-03	3.24E-03	6.49E-03	6.25E-03	4.47E-03	4.87E-03	4.55E-03	5.12E-03	1.10E-02	3.25E-01	3.15E-03	2.49E-03	2.48E-03	3.04E-03	8.91E-03	2.73E-03	3.71E-01	4.49E-01	9.94E-03	8.90E-03	3.91E-03
	Peak sensitivity value	1.33E+01	7.92E-02	-4.21E-03	-3.53E-02	-8.19E-01	-1.51E-01	-3.52E+00	-1.98E+00	-2.22E-02	6.71E-17	-2.49E-01	-5.50E-01	-1.12E-01	-5.56E-02	-2.44E+00	-7.73E-01	-8.61E-20	-5.47E-17	-1.27E-07	-8.10E-07	-9.71E-04
	Shapiro-Wilk score	9.888E-01	9.257E-01	9.584E-01	9.577E-01	9.632E-01	9.722E-01	9.570E-01	9.562E-01	9.560E-01	6.674E-01	9.497E-01	9.523E-01	9.459E-01	9.410E-01	8.959E-01	9.554E-01	8.837E-01	5.223E-01	9.227E-01	9.225E-01	9.531E-01
atpc	Local	1.92E-01	1.36E-03	4.05E-05	2.94E-04	4.09E-03	1.37E-03	2.43E-02	1.43E-02	1.25E-02	2.20E-21	1.95E-03	2.26E-03	-2.71E-01	-5.75E-03	1.91E-02	3.61E-03	9.62E-18	1.69E-20	5.61E-08	1.44E-07	-9.61E-05
	Min	-5.41E-03	-1.74E-03	2.60E-06	1.56E-05	-2.06E-04	3.10E-05	1.69E-04	9.49E-05	4.55E-04	-3.35E-13	2.33E-04	4.09E-05	-4.07E-01	-1.64E-02	1.83E-04	6.17E-05	-2.51E-16	-2.15E-18	3.68E-10	1.00E-08	-3.53E-04
	Max	3.94E-01	5.95E-03	6.31E-04	5.36E-03	5.00E-02	8.18E-03	2.11E-01	1.29E-01	5.00E-02	5.03E-13	1.41E-02	6.78E-03	-1.83E-01	-1.73E-04	2.78E-01	1.22E-02	4.26E-17	9.75E-17	2.73E-07	6.01E-07	-5.64E-07
	Normalized peak height	2.10E-03	8.32E-03	1.41E-02	1.62E-02	1.44E-02	5.29E-03	8.90E-03	8.93E-03	3.42E-03	5.23E-01	6.67E-03	2.79E-03	2.58E-03	2.75E-03	1.30E-02	3.01E-03	3.62E-01	4.49E-01	5.35E-03	3.67E-03	4.25E-03
	Peak sensitivity value	2.51E-01	1.30E-03	1.55E-05	9.85E-05	1.20E-04	2.22E-04	2.39E-03	1.84E-03	1.14E-02	3.78E-16	1.64E-03	2.10E-03	-2.83E-01	-6.38E-03	2.82E-03	3.00E-03	1.04E-19	-7.73E-21	2.61E-08	1.30E-07	-7.06E-05
	Shapiro-Wilk score	9.724E-01	9.292E-01	7.833E-01	7.612E-01	8.053E-01	9.130E-01	8.371E-01	8.437E-01	9.738E-01	2.821E-01	8.982E-01	9.816E-01	9.918E-01	9.835E-01	6.710E-01	9.771E-01	9.049E-01	9.406E-02	9.222E-01	9.695E-01	9.701E-01
glycerol3phosphate cytosol	Local	7.65E-01	5.45E-03	4.16E-04	3.17E-03	5.53E-02	1.89E-02	3.33E-01	1.95E-01	-1.67E+00	1.60E-16	2.68E-02	3.10E-02	6.37E-03	-8.17E-02	2.62E-01	4.94E-02	-4.87E-19	2.34E-19	7.68E-07	2.03E-06	-1.37E-03
	Min	-1.79E+00	-6.74E-02	-1.13E-03	-6.23E-03	-6.46E-02	2.18E-04	1.03E-03	6.32E-04	-2.46E+00	-3.52E-15	2.46E-03	2.61E-04	7.16E-05	-4.52E-01	1.04E-03	3.79E-04	-2.38E-18	-4.19E-17	3.06E-09	1.56E-07	-4.26E-03
	Max	2.27E+00	7.70E-02	4.21E-03	3.37E-02	4.67E-01	8.12E-02	1.84E+00	1.24E+00	-8.96E-01	4.31E-15	9.59E-02	8.77E-02	1.71E-02	-3.15E-03	3.42E+00	1.60E-01	1.62E-17	2.55E-15	3.62E-06	1.08E-05	-5.24E-05
	Normalized peak height	3.92E-03	1.59E-02	1.51E-02	1.45E-02	1.40E-02	4.32E-03	6.99E-03	7.55E-03	4.45E-03	2.11E-01	3.29E-03	2.56E-03	2.44E-03	3.98E-03	1.43E-02	3.09E-03	3.63E-01	5.57E-01	5.29E-03	4.20E-03	3.34E-03
	Peak sensitivity value	1.47E+00	6.61E-03	7.16E-05	4.88E-04	9.89E-04	2.20E-03	2.22E-02	1.36E-02	-1.59E+00	-3.67E-18	1.61E-02	1.19E-02	1.46E-03	-2.49E-02	1.98E-02	9.85E-03	-4.50E-21	7.65E-19	3.05E-07	6.39E-07	-1.14E-03
	Shapiro-Wilk score	9.307E-01	8.660E-01	8.591E-01	8.621E-01	8.643E-01	9.339E-01	8.899E-01	8.935E-01	9.965E-01	8.363E-01	9.618E-01	9.704E-01	9.738E-01	9.427E-01	7.020E-01	9.629E-01	8.212E-01	8.002E-02	9.132E-01	9.238E-01	9.776E-01

Table S7: Concentration control coefficients for *Trypanosoma brucei* model with parameter variation of ±30%

		(glucosetransport)	(hexokinase)	(phosphoglycerateisomerase)	(phophofructokinase)	(aldolase)	(triosephosphateisomerase)	(glyceraldehyde3phosphatedehydrogenase)	(glycerol3phosphatedehydrogenase)	(glycerol3phosphateoxidase)	(pyruvatetransport)	(phosphoglyceratekinase)	(pyruvatekinase)	(atputilisation)	(glycerolkinase)	(phosphoglyceratemutase)	(enolase)	(adenylatekinasecytosol)	(adenylatekinaseglycosome)	(3phosphoglyceratetransport)	(gly3pdhapantiporter)	(glyceroltransport)
adpg	Local	7.11E+00	5.06E-02	-3.17E-03	-2.57E-02	-5.67E-01	-9.47E-02	-2.37E+00	-1.39E+00	1.77E-01	7.55E-16	-1.91E-01	-2.21E-01	-4.54E-02	-2.04E-01	-1.87E+00	-3.52E-01	3.47E-18	-1.01E-17	-5.47E-06	2.02E-06	-3.41E-03
	Min	-2.20E+01	-1.23E+00	-2.53E-02	-1.86E-01	-3.51E+00	-6.23E-01	-1.54E+01	-7.69E+00	-6.48E+00	-2.13E-13	-1.01E+00	-5.15E-01	-1.12E-01	-8.26E-01	-2.00E+01	-8.51E-01	-1.45E-16	-8.06E-14	-6.74E-05	-8.66E-05	-2.18E-02
	Max	2.68E+01	3.07E-01	3.02E-02	2.38E-01	4.50E+00	3.92E-01	1.34E+01	7.41E+00	1.32E+00	1.74E-13	7.34E-01	3.81E-01	8.08E-02	2.84E+00	2.10E+01	6.89E-01	2.52E-17	7.88E-16	1.00E-05	2.32E-05	1.68E-02
	Normalized peak height	6.17E-03	2.33E-02	1.75E-02	1.61E-02	1.33E-02	9.13E-03	1.08E-02	9.39E-03	4.64E-02	6.29E-01	8.49E-03	7.70E-03	7.84E-03	2.86E-02	4.29E-02	8.93E-03	3.91E-01	3.45E-01	4.03E-02	3.98E-02	1.68E-02
	Peak sensitivity value	5.19E+00	1.82E-02	-1.11E-03	-8.38E-03	-2.53E-01	-3.72E-02	-1.03E+00	-6.31E-01	-8.66E-03	-1.07E-18	1.07E-02	7.45E-03	1.19E-03	-6.51E-03	3.81E-02	7.22E-03	3.16E-20	1.54E-17	5.70E-08	-2.64E-07	-1.42E-04
	Shapiro-Wilk score	9.662E-01	6.494E-01	8.995E-01	9.082E-01	9.483E-01	9.760E-01	9.668E-01	9.668E-01	8.584E-01	2.253E-01	9.368E-01	9.522E-01	9.460E-01	8.979E-01	8.677E-01	9.612E-01	7.543E-01	7.872E-02	8.463E-01	8.246E-01	9.752E-01
adpc	Local	-5.14E-01	-3.66E-03	-1.09E-04	-7.90E-04	-1.10E-02	-3.67E-03	-6.52E-02	-3.83E-02	-3.37E-02	0.00E+00	-5.25E-03	-6.07E-03	7.27E-01	1.54E-02	-5.14E-02	-9.68E-03	9.75E-18	-4.52E-20	-1.50E-07	-3.85E-07	2.58E-04
	Min	-8.01E-01	-2.23E-02	-3.66E-03	-3.55E-02	-1.65E-01	-2.52E-02	-5.41E-01	-3.92E-01	-3.45E-01	-1.40E-12	-4.45E-02	-2.66E-02	4.89E-01	-6.75E-03	-7.88E-01	-4.31E-02	-8.64E-16	-1.46E-16	-1.16E-06	-2.11E-06	-1.66E-05
	Max	8.14E-02	3.38E-02	-1.64E-06	-8.92E-06	9.25E-04	-1.40E-05	-3.23E-05	-1.92E-05	-4.76E-05	1.49E-12	-1.70E-04	-1.04E-05	8.44E-01	4.99E-02	-3.41E-05	-1.27E-05	5.75E-17	3.46E-17	-2.67E-11	-1.67E-09	1.07E-03
	Normalized peak height	9.29E-03	1.67E-02	3.98E-02	5.23E-02	5.87E-02	1.35E-02	2.46E-02	2.91E-02	1.33E-02	7.77E-01	6.87E-03	6.17E-03	2.63E-03	4.57E-03	3.72E-02	6.84E-03	3.36E-01	4.99E-01	8.51E-03	5.82E-03	9.64E-03
	Peak sensitivity value	-2.19E-04	-2.72E-03	-1.81E-05	-1.33E-04	1.24E-05	-1.27E-04	-8.44E-04	-6.07E-04	-2.98E-03	-6.06E-17	-2.90E-03	-3.70E-04	7.23E-01	2.06E-03	-1.22E-03	-4.22E-04	-9.99E-20	3.06E-21	-2.37E-08	-8.06E-08	1.28E-06
	Shapiro-Wilk score	9.026E-01	8.994E-01	7.295E-01	7.006E-01	7.860E-01	8.695E-01	8.121E-01	8.135E-01	8.187E-01	1.637E-01	8.294E-01	9.289E-01	9.889E-01	9.432E-01	5.977E-01	9.085E-01	8.094E-01	2.506E-01	8.586E-01	9.225E-01	8.873E-01
dihydroxyacetonephosphate	Local	-1.12E-01	-8.00E-04	1.87E-03	1.47E-02	2.89E-01	-7.31E-02	-6.54E-02	-3.84E-02	6.36E-02	-2.29E-17	-5.13E-03	-5.94E-03	-1.22E-03	-6.36E-03	-5.03E-02	-9.47E-03	9.33E-20	1.12E-18	-1.47E-07	7.28E-07	-1.06E-04
	Min	-2.95E+00	-1.23E-01	3.17E-04	2.38E-03	9.42E-02	-1.48E-01	-7.38E-01	-5.43E-01	3.95E-03	-3.01E-15	-1.02E-01	-7.98E-02	-1.68E-02	-9.30E-02	-1.36E+00	-1.53E-01	-2.58E-17	-3.95E-17	-2.95E-06	2.68E-07	-1.59E-03
	Max	1.10E+00	2.03E-02	1.32E-02	1.26E-01	7.43E-01	-3.05E-02	3.65E-02	2.61E-02	3.37E+00	4.81E-15	1.37E-03	5.72E-04	1.35E-04	1.88E-01	2.61E-03	8.76E-04	9.19E-19	2.72E-15	1.07E-08	5.33E-06	1.37E-03
	Normalized peak height	1.74E-02	6.17E-02	7.40E-03	8.62E-03	4.02E-03	3.89E-03	3.25E-02	3.45E-02	3.28E-02	4.09E-01	2.10E-02	1.82E-02	1.71E-02	8.51E-02	6.10E-02	2.45E-02	4.93E-01	1.73E-01	2.56E-02	7.54E-03	5.47E-02
	Peak sensitivity value	9.63E-03	-7.54E-04	1.43E-03	1.17E-02	2.42E-01	-6.29E-02	-4.94E-03	-3.24E-03	2.25E-02	3.75E-18	-2.10E-03	-6.73E-04	-1.28E-04	-1.21E-04	-2.15E-03	-5.88E-04	-4.73E-21	5.47E-19	-3.81E-08	6.14E-07	-4.30E-06
	Shapiro-Wilk score	9.794E-01	7.579E-01	8.802E-01	8.784E-01	9.639E-01	9.679E-01	8.501E-01	8.404E-01	5.900E-01	4.723E-01	8.363E-01	8.631E-01	8.808E-01	8.773E-01	6.442E-01	8.418E-01	2.342E-01	2.103E-01	7.149E-01	8.676E-01	8.510E-01
glyceraldehyde3phosphate	Local	-1.73E-01	-1.23E-03	1.89E-03	1.49E-02	2.93E-01	6.17E-02	-9.23E-02	-5.41E-02	5.22E-02	-3.55E-17	-7.29E-03	-8.44E-03	-1.73E-03	-4.30E-04	-7.14E-02	-1.34E-02	1.32E-19	1.13E-18	-2.09E-07	5.97E-07	-7.19E-06
	Min	-3.23E+00	-1.20E-01	3.26E-04	2.96E-03	8.58E-02	2.29E-02	-7.49E-01	-6.72E-01	2.56E-03	-1.11E-15	-1.13E-01	-6.64E-02	-1.34E-02	-7.88E-02	-1.70E+00	-1.39E-01	-1.37E-17	-4.00E-17	-3.03E-06	5.63E-08	-1.53E-03
	Max	9.52E-01	2.48E-02	1.18E-02	1.26E-01	8.14E-01	1.23E-01	-2.09E-03	-9.39E-04	4.18E+00	1.13E-15	-3.44E-04	-2.68E-05	-7.45E-06	2.05E-01	-9.96E-05	-3.69E-05	8.73E-19	1.64E-15	-3.51E-11	4.57E-06	1.47E-03
	Normalized peak height	1.63E-02	4.71E-02	6.63E-03	8.41E-03	4.47E-03	4.01E-03	2.45E-02	3.28E-02	4.92E-02	3.53E-01	1.63E-02	1.18E-02	1.04E-02	7.46E-02	6.00E-02	1.68E-02	3.92E-01	1.55E-01	2.16E-02	9.02E-03	4.23E-02
	Peak sensitivity value	3.77E-03	-1.20E-03	1.23E-03	1.17E-02	2.48E-01	4.84E-02	-5.45E-03	-3.29E-03	2.13E-02	-2.07E-19	-2.64E-03	-5.91E-04	-1.61E-04	2.87E-04	-2.64E-03	-9.38E-04	5.33E-21	-5.14E-19	-1.55E-09	5.19E-07	6.87E-06
	Shapiro-Wilk score	9.711E-01	7.949E-01	8.829E-01	8.816E-01	9.632E-01	9.753E-01	8.549E-01	8.468E-01	5.192E-01	6.584E-01	8.655E-01	8.873E-01	9.039E-01	8.914E-01	6.513E-01	8.625E-01	5.323E-01	4.553E-01	7.674E-01	8.471E-01	8.687E-01
2phosphoglycerate	Local	9.33E-01	6.64E-03	1.97E-04	1.43E-03	1.99E-02	6.66E-03	1.18E-01	6.94E-02	6.11E-02	-8.41E-17	9.52E-03	-4.61E-01	-9.48E-02	-2.80E-02	9.32E-02	-7.35E-01	7.25E-18	8.21E-20	2.73E-07	6.99E-07	-4.68E-04
	Min	-1.07E-01	-6.62E-02	2.75E-06	1.47E-05	-1.71E-03	2.63E-05	6.49E-05	3.76E-05	1.30E-04	-4.50E-13	3.30E-04	-7.85E-01	-1.71E-01	-9.52E-02	1.00E-04	-1.52E+00	-7.89E-16	-1.21E-17	4.38E-11	2.36E-09	-2.04E-03
	Max	1.90E+00	3.96E-02	5.09E-03	6.43E-02	3.49E-01	5.42E-02	1.08E+00	7.92E-01	7.69E-01	3.44E-13	8.61E-02	-3.19E-01	-6.08E-02	1.43E-02	1.74E+00	-3.81E-01	4.60E-17	2.12E-16	1.87E-06	3.88E-06	3.32E-05
	Normalized peak height	1.19E-02	1.66E-02	3.06E-02	5.03E-02	6.12E-02	1.62E-02	2.66E-02	3.09E-02	1.57E-02	6.20E-01	7.61E-03	2.63E-03	3.30E-03	4.85E-03	4.27E-02	4.21E-03	3.36E-01	3.39E-01	7.43E-03	6.29E-03	9.95E-03
	Peak sensitivity value	6.73E-04	4.82E-03	3.07E-05	2.40E-04	-1.36E-04	3.24E-04	1.69E-03	1.23E-03	5.90E-03	3.57E-16	4.15E-03	-3.92E-01	-9.85E-02	-4.69E-03	2.70E-03	-6.39E-01	-3.66E-19	1.11E-19	2.90E-08	1.21E-07	-3.00E-06
	Shapiro-Wilk score	9.195E-01	8.965E-01	7.320E-01	7.083E-01	7.862E-01	8.697E-01	8.159E-01	8.148E-01	8.025E-01	1.324E-01	8.206E-01	9.599E-01	9.888E-01	9.363E-01	5.992E-01	9.646E-01	7.664E-01	2.666E-01	8.579E-01	9.138E-01	8.789E-01
glucose	Local	3.20E+00	-1.04E+00	-1.17E-02	-7.39E-02	-1.69E-01	-2.68E-02	-6.92E-01	-4.06E-01	5.93E-02	2.20E-16	-5.57E-02	-6.44E-02	-1.33E-02	-5.89E-02	-5.45E-01	-1.03E-01	1.01E-18	-2.56E-19	-1.60E-06	6.79E-07	-9.85E-04
	Min	1.07E+00	-1.10E+00	-8.36E-02	-8.73E-01	-7.53E+00	-7.19E-01	-2.48E+01	-1.57E+01	-2.13E+00	-5.78E-13	-1.51E+00	-8.31E-01	-1.57E-01	-4.44E+00	-3.93E+01	-1.57E+00	-7.24E-17	-1.09E-15	-1.43E-05	-2.31E-06	-3.48E-02
	Max	4.03E+01	1.03E+00	1.33E-03	3.81E-03	-1.08E-03	1.82E-01	-1.42E-03	-7.12E-04	6.38E+00	5.06E-13	-3.25E-03	-2.73E-04	-5.79E-05	5.02E-03	-9.05E-04	-3.24E-04	1.57E-17	3.00E-14	-4.04E-10	1.77E-04	7.77E-05
	Normalized peak height	3.20E-02	5.82E-02	7.07E-03	1.05E-02	7.80E-02	6.64E-02	7.82E-02	8.31E-02	2.52E-01	7.89E-01	2.56E-02	1.62E-02	1.35E-02	2.38E-01	1.40E-01	2.28E-02	3.65E-01	8.42E-01	9.79E-03	2.65E-01	1.61E-01
	Peak sensitivity value	1.20E+00	-1.03E+00	-6.78E-03	-4.75E-02	-1.24E-02	-7.37E-04	-1.38E-02	-8.58E-03	2.73E-03	4.41E-16	-2.21E-02	-4.84E-03	-1.23E-03	-1.64E-03	-2.06E-02	-5.81E-03	6.37E-23	8.81E-18	-3.35E-07	1.16E-07	-9.60E-06
	Shapiro-Wilk score	8.469E-01	6.213E-01	8.747E-01	8.749E-01	7.446E-01	8.093E-01	7.451E-01	7.276E-01	7.698E-01	1.293E-01	7.185E-01	8.388E-01	8.474E-01	7.762E-01	5.280E-01	8.119E-01	6.940E-01	1.797E-02	8.536E-01	6.975E-01	7.614E-01
fructose6phosphate	Local	1.54E+00	1.10E-02	4.77E-03	-1.07E+00	-1.09E-01	6.21E-03	-1.92E-01	-1.13E-01	1.40E-01	4.79E-17	-1.51E-02	-1.75E-02	-3.59E-03	-6.96E-03	-1.48E-01	-2.78E-02	2.74E-19	2.79E-18	-4.33E-07	1.60E-06	-1.16E-04
	Min	-4.82E+00	-2.28E-02	-4.31E-04	-1.40E+00	-2.49E+00	-1.36E-01	-8.07E+00	-3.69E+00	-7.22E-02	-3.36E-13	-2.71E-01	-1.98E-01	-3.83E-02	-1.66E+00	-1.15E+01	-2.40E-01	-9.86E-18	-2.28E-16	-7.74E-06	-1.57E-06	-5.33E-03
	Max	1.46E+01	1.61E+00	5.29E-02	-8.93E-01	5.10E-02	4.43E-02	4.04E-01	2.38E-01	7.35E+00	1.07E-13	1.94E-02	9.82E-03	2.26E-03	2.51E-01	6.03E-01	1.93E-02	5.00E-18	2.28E-14	2.35E-07	3.72E-05	2.28E-03
	Normalized peak height	4.09E-02	1.35E-01	9.85E-03	8.70E-03	2.69E-02	1.51E-02	1.14E-01	8.72E-02	3.88E-02	8.75E-01	1.94E-02	1.82E-02	1.63E-02	2.20E-01	1.62E-01	1.66E-02	3.71E-01	3.61E-01	2.62E-02	2.64E-02	8.96E-02
	Peak sensitivity value	1.08E+00	4.21E-03	2.69E-03	-1.06E+00	-5.46E-02	8.13E-03	-1.52E-02	-7.39E-03	5.03E-02	1.26E-16	-5.73E-03	-1.31E-03	-3.18E-04	2.88E-05	-9.50E-03	-1.07E-03	2.17E-21	-9.15E-18	-1.16E-10	1.39E-06	6.30E-06
	Shapiro-Wilk score	8.775E-01	5.791E-01	8.148E-01	9.539E-01	7.870E-01	8.613E-01	7.386E-01	7.283E-01	6.664E-01	5.072E-02	7.587E-01	8.715E-01	8.860E-01	7.239E-01	5.333E-01	8.457E-01	7.452E-01	6.115E-02	8.027E-01	6.883E-01	7.980E-01

3phosphoglycerate

Local	3.53E+00	2.51E-02	7.47E-04	5.43E-03	7.54E-02	2.52E-02	4.48E-01	2.63E-01	2.31E-01	2.58E-22	3.60E-02	-4.03E-01	-8.28E-02	-1.06E-01	-3.41E+00	-6.42E-01	6.33E-18	3.11E-19	-9.98E-06	2.65E-06	-1.77E-03
Min	-4.85E-01	-1.76E-01	8.77E-06	4.57E-05	-4.92E-03	3.60E-05	1.15E-04	1.14E-04	9.72E-04	-2.23E-13	6.40E-04	-7.56E-01	-1.65E-01	-1.51E+00	-3.35E+01	-1.26E+00	-4.77E-16	-2.68E-15	-1.58E-04	3.94E-08	-2.90E-02
Max	3.19E+01	5.99E-01	4.85E-02	3.33E-01	3.55E+00	4.74E-01	1.22E+01	7.51E+00	4.70E+00	6.55E-13	1.01E+00	-8.70E-04	-1.25E-04	4.33E-02	-9.10E-01	-1.49E-03	4.79E-17	3.88E-15	-6.49E-11	6.22E-05	1.28E-04
Normalized peak height	2.87E-02	3.01E-02	8.79E-02	8.18E-02	1.61E-01	4.83E-02	9.56E-02	9.91E-02	2.87E-02	7.22E-01	2.67E-02	4.20E-03	4.10E-03	1.89E-02	1.30E-02	4.39E-03	3.46E-01	6.98E-01	4.24E-02	2.76E-02	3.02E-02
Peak sensitivity value	1.76E-02	1.19E-02	8.15E-05	5.44E-04	4.07E-04	7.47E-04	6.19E-03	3.87E-03	1.27E-02	3.47E-16	9.26E-03	-3.55E-01	-8.68E-02	-1.32E-02	-1.67E+00	-5.78E-01	-1.07E-19	-2.20E-18	-7.90E-08	3.19E-07	-9.04E-05
Shapiro-Wilk score	8.647E-01	6.294E-01	7.033E-01	6.993E-01	7.730E-01	8.492E-01	8.260E-01	8.055E-01	7.266E-01	1.229E-01	5.382E-01	9.406E-01	9.218E-01	6.820E-01	7.875E-01	9.486E-01	7.911E-01	4.985E-02	8.506E-01	6.601E-01	6.491E-01

glycerol

Local	9.22E+00	6.57E-02	-2.07E-03	-1.73E-02	-4.20E-01	-1.47E-01	-2.56E+00	-1.50E+00	-1.41E+00	1.05E-15	-2.06E-01	-2.39E-01	-4.91E-02	6.53E-01	-2.02E+00	-3.80E-01	3.75E-18	-1.82E-18	-5.91E-06	-1.61E-05	-9.89E-01
Min	-3.46E+00	-5.07E-01	-2.52E-02	-2.04E-01	-3.97E+00	-7.48E-01	-1.82E+01	-1.16E+01	-1.39E+01	-1.00E-13	-1.00E+00	-5.60E-01	-1.19E-01	6.82E-02	-2.73E+01	-9.17E-01	-3.37E-16	-1.46E-14	-6.54E-05	-1.72E-04	-1.00E+00
Max	3.33E+01	3.72E-01	1.40E-02	1.38E-01	1.15E+00	5.40E-02	2.46E+00	1.60E+00	-1.42E-01	6.95E-14	1.63E-01	9.38E-02	1.81E-02	1.15E+00	4.58E+00	1.17E-01	2.47E-17	2.59E-16	1.12E-06	-7.27E-07	-9.38E-01
Normalized peak height	9.95E-03	1.08E-02	1.36E-02	1.42E-02	9.90E-03	6.56E-03	1.24E-02	1.12E-02	1.46E-02	5.07E-01	9.05E-03	1.09E-02	1.05E-02	4.58E-03	4.86E-02	1.17E-02	4.72E-01	5.69E-01	6.09E-02	7.02E-03	7.83E-03
Peak sensitivity value	-1.37E-01	2.25E-02	-2.63E-04	-1.13E-03	-1.23E-01	-8.35E-02	1.32E-02	2.95E-03	-1.41E+00	1.80E-17	6.71E-03	2.55E-03	4.36E-04	9.52E-01	2.80E-03	2.69E-03	-8.08E-20	-4.69E-19	2.61E-08	-9.05E-06	-9.98E-01
Shapiro-Wilk score	9.682E-01	8.576E-01	9.431E-01	9.498E-01	9.603E-01	9.198E-01	9.247E-01	9.205E-01	8.964E-01	3.973E-01	9.055E-01	9.433E-01	9.376E-01	9.162E-01	8.143E-01	9.491E-01	6.535E-01	7.523E-02	8.359E-01	8.911E-01	8.881E-01

pyruvate

Local	5.54E+00	3.94E-02	1.17E-03	8.51E-03	1.18E-01	3.96E-02	7.02E-01	4.12E-01	3.62E-01	-7.85E+00	5.65E-02	6.54E-02	1.35E-02	-1.66E-01	5.54E-01	1.04E-01	-1.03E-18	4.87E-19	1.62E-06	4.15E-06	-2.78E-03
Min	-4.81E+01	-2.32E+01	6.91E-06	4.38E-05	-8.81E-01	5.66E-05	2.00E-04	9.95E-05	2.02E-04	-9.22E+03	8.82E-04	5.37E-05	1.13E-05	-2.86E+02	1.89E-04	5.90E-05	-1.08E-15	-3.94E-15	2.22E-10	6.85E-09	-4.74E+00
Max	3.68E+03	1.11E+02	9.01E+00	5.76E+01	1.06E+03	8.20E+01	2.82E+03	2.33E+03	2.03E+03	-1.75E+00	1.20E+02	1.15E+02	1.89E+01	6.17E+00	3.82E+03	1.47E+02	5.65E-16	1.09E-14	2.78E-03	5.69E-03	7.16E-03
Normalized peak height	5.90E-01	6.22E-01	9.39E-01	9.24E-01	6.65E-01	8.53E-01	8.95E-01	9.32E-01	9.67E-01	8.45E-01	8.72E-01	9.02E-01	8.71E-01	8.91E-01	9.29E-01	8.73E-01	7.82E-01	8.95E-01	9.09E-01	8.47E-01	8.31E-01
Peak sensitivity value	2.25E+00	-3.98E-02	4.51E-03	2.88E-02	-3.50E-01	4.10E-02	1.41E+00	1.16E+00	1.02E+00	-6.36E+00	6.09E-02	5.77E-02	9.46E-03	-1.01E-01	1.91E+00	7.36E-02	6.55E-19	-8.43E-19	1.39E-06	2.85E-06	3.70E-05
Shapiro-Wilk score	5.380E-02	4.718E-02	3.065E-02	3.621E-02	3.117E-02	4.936E-02	4.540E-02	3.683E-02	1.812E-02	5.443E-02	4.642E-02	3.662E-02	3.974E-02	3.818E-02	3.216E-02	4.005E-02	3.621E-02	4.260E-02	4.284E-02	4.249E-02	3.167E-02

dihydroxyacetonephosphate cytosol

Local	-3.10E-01	-2.20E-03	-1.68E-04	-1.28E-03	-2.24E-02	-7.65E-03	-1.35E-01	-7.90E-02	6.75E-01	-6.46E-17	-1.08E-02	-1.25E-02	-2.58E-03	3.30E-02	-1.06E-01	-2.00E-02	1.97E-19	-9.46E-20	-3.10E-07	-8.20E-07	5.52E-04
Min	-7.70E+00	-7.43E-02	-1.05E-02	-6.63E-02	-1.08E+00	-2.13E-01	-3.31E+00	-2.25E+00	7.00E-02	-5.05E-15	-2.75E-01	-2.77E-01	-5.24E-02	1.14E-04	-8.04E+00	-5.49E-01	-3.75E-18	-1.16E-14	-1.01E-05	-2.28E-05	1.93E-06
Max	3.56E+00	9.86E-02	1.65E-03	1.06E-02	7.51E-02	-8.55E-06	-2.32E-05	-1.58E-05	9.91E+00	7.49E-15	-7.22E-06	-2.15E-06	-5.87E-07	5.29E-01	-1.24E-05	-3.78E-06	3.32E-18	3.77E-17	-1.93E-11	-9.02E-09	7.33E-03
Normalized peak height	6.48E-02	4.08E-02	1.39E-01	1.11E-01	1.44E-01	7.31E-02	1.07E-01	1.17E-01	2.19E-02	5.15E-01	3.36E-02	5.88E-02	4.97E-02	2.17E-02	2.49E-01	7.65E-02	3.73E-01	9.39E-01	7.39E-02	3.33E-02	1.98E-02
Peak sensitivity value	-7.20E-03	-1.06E-03	-1.26E-05	-4.01E-05	2.72E-04	-1.15E-04	-1.68E-03	-1.14E-03	2.72E-01	-4.47E-18	-1.79E-03	-4.18E-04	-7.92E-05	1.96E-03	-4.03E-03	-8.27E-04	2.98E-21	-3.21E-18	-2.54E-08	-6.61E-08	4.96E-05
Shapiro-Wilk score	8.846E-01	8.252E-01	5.586E-01	6.057E-01	5.991E-01	6.514E-01	6.634E-01	6.119E-01	6.668E-01	4.756E-01	6.098E-01	5.742E-01	6.024E-01	7.730E-01	5.225E-01	5.499E-01	6.026E-01	5.718E-03	4.930E-01	8.114E-01	7.097E-01

nadh

Local	1.19E+00	8.44E-03	1.92E-04	1.37E-03	1.64E-02	1.21E-02	1.67E-01	-1.51E+00	-3.09E-02	2.01E-16	1.34E-02	1.55E-02	3.19E-03	-3.88E-02	1.31E-01	2.47E-02	-2.44E-19	7.06E-20	3.85E-07	-3.54E-07	-6.48E-04
Min	-1.58E+00	-6.77E-02	-4.51E-04	-3.39E-03	-6.54E-02	7.29E-04	2.28E-04	-2.63E+00	-6.21E+00	-2.81E-15	4.45E-04	1.81E-05	4.81E-06	-3.09E-01	6.42E-05	2.75E-05	-2.12E-18	-1.55E-16	5.57E-11	-6.41E-06	-4.13E-03
Max	6.72E+00	5.45E-02	6.26E-03	6.54E-02	4.04E-01	9.93E-02	1.59E+00	-7.61E-01	1.39E-01	4.41E-15	1.51E-01	1.56E-01	2.88E-02	8.65E-05	4.82E+00	2.96E-01	3.29E-18	5.71E-16	7.85E-06	3.07E-06	1.56E-07
Normalized peak height	3.71E-02	1.58E-02	4.12E-02	5.24E-02	1.85E-02	1.09E-02	2.91E-02	5.59E-03	6.13E-02	3.64E-01	9.11E-03	1.29E-02	1.03E-02	8.57E-03	8.03E-02	1.79E-02	3.43E-01	4.67E-01	2.20E-02	6.41E-03	1.07E-02
Peak sensitivity value	4.84E-03	5.43E-03	2.18E-06	-5.89E-05	-5.05E-03	2.95E-03	2.61E-03	-1.34E+00	-1.62E-02	2.26E-18	4.88E-03	1.03E-03	1.63E-04	-6.56E-03	2.47E-03	7.68E-04	1.58E-21	-2.74E-19	5.89E-08	-4.99E-07	-1.84E-05
Shapiro-Wilk score	9.092E-01	9.021E-01	7.464E-01	7.287E-01	8.170E-01	8.927E-01	8.216E-01	9.839E-01	4.900E-01	7.765E-01	8.461E-01	8.837E-01	9.020E-01	8.977E-01	6.218E-01	8.555E-01	8.115E-01	1.308E-01	7.493E-01	9.856E-01	8.744E-01

glycerol3phosphate

Local	9.63E-01	6.85E-03	2.45E-03	1.91E-02	3.66E-01	-4.66E-02	4.02E-01	2.36E-01	-2.28E+00	2.02E-16	3.25E-02	3.76E-02	7.73E-03	-1.21E-01	3.18E-01	5.99E-02	-5.90E-19	1.45E-18	9.31E-07	-2.61E-05	-2.02E-03
Min	-5.47E+00	-1.67E-01	1.70E-05	1.71E-03	1.12E-01	-1.33E-01	-1.90E-01	-9.18E-02	-9.86E+00	-1.83E-14	-3.85E-03	-2.34E-03	-5.11E-04	-8.81E-01	-1.48E-02	-2.93E-03	-6.12E-18	-1.25E-16	-9.50E-08	-1.44E-04	-1.20E-02
Max	7.85E+00	1.97E-01	2.68E-02	1.60E-01	1.96E+00	1.21E-01	4.68E+00	3.48E+00	-8.59E-01	2.03E-14	3.04E-01	2.91E-01	5.03E-02	-1.40E-03	9.45E+00	4.68E-01	8.37E-18	2.80E-14	1.41E-05	-9.35E-07	-2.55E-05
Normalized peak height	1.58E-02	2.60E-02	1.09E-02	8.14E-03	6.73E-03	6.21E-03	4.05E-02	4.61E-02	1.04E-02	5.28E-01	9.80E-03	1.56E-02	1.23E-02	7.52E-03	9.51E-02	1.75E-02	3.49E-01	7.29E-01	2.77E-02	5.08E-03	6.48E-03
Peak sensitivity value	5.24E-02	6.12E-03	1.53E-03	1.33E-02	2.64E-01	-4.77E-02	2.52E-03	2.81E-03	-1.72E+00	6.53E-18	7.40E-03	1.91E-03	4.80E-04	-1.06E-02	8.88E-03	2.02E-03	7.43E-22	1.56E-18	1.15E-08	-2.24E-05	-3.30E-04
Shapiro-Wilk score	9.827E-01	8.696E-01	8.359E-01	8.517E-01	8.835E-01	9.636E-01	8.241E-01	7.940E-01	8.423E-01	4.985E-01	8.298E-01	7.920E-01	8.130E-01	9.161E-01	6.263E-01	7.628E-01	7.427E-01	2.705E-02	6.727E-01	9.251E-01	8.960E-01

glucose6phosphate

Local	1.66E+00	1.18E-02	-1.68E-01	-1.04E+00	-1.04E-01	6.02E-03	-1.84E-01	-1.08E-01	1.35E-01	4.60E-17	-1.45E-02	-1.68E-02	-3.45E-03	-6.64E-03	-1.42E-01	-2.67E-02	2.64E-19	2.69E-18	-4.16E-07	1.54E-06	-1.11E-04
Min	-7.63E+00	-3.70E-02	-3.27E-01	-1.42E+00	-1.34E+00	-1.47E-01	-6.41E+00	-4.35E+00	-1.03E-01	-1.60E-13	-3.55E-01	-1.56E-01	-4.75E-02	-1.21E+00	-1.10E+01	-3.16E-01	-1.02E-17	-3.42E-16	-7.86E-06	-2.32E-06	-6.87E-03
Max	1.29E+01	1.68E+00	-6.94E-02	-8.94E-01	9.31E-02	4.60E-02	4.46E-01	2.62E-01	9.99E+00	1.84E-13	4.07E-02	2.75E-02	4.92E-03	3.58E-01	7.94E-01	3.99E-02	1.21E-17	5.43E-14	1.73E-07	3.56E-05	1.99E-03
Normalized peak height	4.15E-02	1.32E-01	4.02E-03	7.31E-03	1.58E-02	1.74E-02	8.81E-02	1.06E-01	5.39E-02	8.66E-01	2.79E-02	1.71E-02	2.13E-02	1.96E-01	1.55E-01	2.39E-02	3.96E-01	6.55E-01	2.82E-02	2.61E-02	1.12E-01
Peak sensitivity value	1.17E+00	5.04E-03	-1.46E-01	-1.02E+00	-5.19E-02	7.17E-03	-1.62E-02	-7.78E-03	5.39E-02	-7.77E-17	-5.38E-03	-1.22E-03	-2.47E-04	-9.58E-05	-3.04E-03	-1.23E-03	3.31E-21	1.36E-17	1.44E-10	1.22E-06	2.38E-06
Shapiro-Wilk score	8.947E-01	5.857E-01	9.863E-01	9.645E-01	7.976E-01	8.599E-01	7.488E-01	7.366E-01	6.484E-01	5.451E-02	7.757E-01	8.807E-01	8.914E-01	7.388E-01	5.398E-01	8.526E-01	6.944E-01	2.739E-02	8.124E-01	7.059E-01	7.996E-01

3phosphoglycerate cytosol

Local	3.53E+00	2.51E-02	7.47E-04	5.43E-03	7.54E-02	2.52E-02	4.48E-01	2.63E-01	2.31E-01	0.00E+00	3.60E-02	-4.03E-01	-8.28E-02	-1.06E-01	-3.41E+00	-6.42E-01	6.33E-18	3.11E-19	1.03E-06	2.65E-06	-1.77E-03
Min	-7.38E-01	-2.67E-01	9.34E-06	4.96E-05	-6.84E-03	4.74E-05	1.14E-04	8.05E-05	1.23E-03	-3.31E-13	5.50E-04	-7.17E-01	-1.61E-01	-1.47E+00	-4.26E+01	-1.30E+00	-4.03E-16	-1.75E-15	4.27E-09	4.02E-08	-2.56E-02
Max	3.96E+01	5.37E-01	4.37E-02	3.49E-01	3.20E+00	4.31E-01	1.30E+01	9.32E+00	3.76E+00	2.00E-12	1.05E+00	-1.19E-03	-2.43E-04	5.91E-02	-8.04E-01	-1.53E-03	4.01E-17	8.52E-15	4.27E-06	6.00E-05	1.50E-04
Normalized peak height	3.66E-02	3.08E-02	8.22E-02	8.72E-02	1.45E-01	4.41E-02	1.02E-01	1.17E-01	2.37E-02	7.21E-01	2.83E-02	3.74E-03	4.13E-03	1.86E-02	1.66E-02	4.66E-03	3.45E-01	8.17E-01	4.46E-03	2.75E-02	2.73E-02
Peak sensitivity value	8.37E-03	1.24E-02	7.48E-05	5.73E-04	1.17E-03	6.93E-04	6.60E-03	4.74E-03	1.06E-02	-1.11E-15	7.39E-03	-3.66E-01	-8.75E-02	-7.40E-03	-1.58E+00	-5.47E-01	1.27E-20	-2.07E-18	3.09E-07	2.50E-07	-6.87E-05
Shapiro-Wilk score	8.650E-01	6.262E-01	7.012E-01	6.951E-01	7.734E-01	8.486E-01	8.247E-01	8.018E-01	7.324E-01	4.648E-02	5.349E-01	9.399E-01	9.206E-01	6.850E-01	7.861E-01	9.479E-01	8.428E-01	2.515E-02	9.299E-01	6.677E-01	6.484E-01

phosphoenolpyruvate

Local	4.22E-01	3.01E-03	8.93E-05	6.49E-04	9.02E-03	3.02E-03	5.36E-02	3.14E-02	2.76E-02	-1.05E-16	4.31E-03	-4.91E-01	-1.01E-01	-1.27E-02	4.22E-02	7.95E-03	7.72E-18	3.72E-20	1.24E-07	3.16E-07	-2.12E-04
Min	-5.64E-02	-2.78E-02	1.01E-06	8.08E-06	-8.83E-04	1.45E-05	3.48E-05	1.91E-05	4.69E-05	-5.58E-13	1.74E-04	-8.37E-01	-1.81E-01	-4.11E-02	2.96E-05	1.08E-05	-8.13E-16	-2.53E-17	3.89E-11	9.76E-10	-1.21E-03
Max	8.82E-01	1.91E-02	2.86E-03	2.87E-02	1.69E-01	2.58E-02	5.72E-01	3.66E-01	3.29E-01	3.37E-13	4.79E-02	-3.44E-01	-6.39E-02	5.95E-03	8.47E-01	4.94E-02	5.47E-17	1.54E-16	1.39E-06	1.85E-06	1.20E-05
Normalized peak height	1.26E-02	1.56E-02	3.55E-02	4.72E-02	5.51E-02	1.51E-02	2.80E-02	2.90E-02	1.39E-02	8.28E-01	8.69E-03	2.63E-03	3.18E-03	4.31E-03	4.30E-02	8.35E-03	3.35E-01	4.87E-01	1.15E-02	5.84E-03	1.35E-02
Peak sensitivity value	3.83E-04	2.27E-03	1.67E-05	1.09E-04	5.33E-05	1.05E-04	8.93E-04	5.69E-04	2.84E-03	3.18E-17	1.73E-03	-4.00E-01	-1.01E-01	-2.44E-03	1.30E-03	3.32E-04	-3.60E-19	6.36E-20	7.33E-10	5.93E-08	-7.68E-07
Shapiro-Wilk score	9.405E-01	8.953E-01	7.210E-01	6.917E-01	7.800E-01	8.613E-01	8.091E-01	8.066E-01	8.227E-01	1.148E-01	8.241E-01	9.555E-01	9.895E-01	9.393E-01	5.908E-01	9.014E-01	7.753E-01	1.786E-01	8.333E-01	9.155E-01	8.808E-01

bisphosphoglycerate

Local	-5.89E+00	-4.19E-02	5.08E-03	4.06E-02	8.47E-01	1.55E-01	3.68E+00	2.16E+00	4.96E-03	-1.01E-15	-8.02E-02	-9.29E-02	-1.91E-02	1.64E-01	-7.86E-01	-1.48E-01	1.46E-18	3.25E-18	-2.30E-06	5.67E-08	2.74E-03
Min	-4.00E+01	-4.43E-01	-1.17E-03	-3.09E-03	7.86E-02	1.23E-02	4.04E-01	2.50E-01	-1.28E+00	-7.35E-14	-1.06E+00	-5.98E-01	-1.29E-01	-2.30E-01	-2.11E+01	-1.07E+00	-3.87E-17	-2.82E-16	-1.02E-04	-3.52E-05	-2.33E-03
Max	1.98E+00	1.36E-01	5.42E-02	4.43E-01	4.70E+00	8.67E-01	2.68E+01	1.05E+01	1.42E+01	8.28E-14	-1.53E-03	-7.58E-05	-1.88E-05	1.18E+00	-2.12E-04	-8.99E-05	3.57E-17	7.05E-15	-6.96E-11	1.59E-05	2.46E-02
Normalized peak height	2.34E-02	1.43E-02	1.20E-02	1.20E-02	6.75E-03	8.96E-03	1.00E-02	6.63E-03	6.71E-02	4.87E-01	1.62E-02	1.63E-02	1.50E-02	1.60E-02	1.06E-01	2.12E-02	3.83E-01	1.87E-01	8.41E-02	1.78E-02	2.43E-02
Peak sensitivity value	-1.89E-02	-1.26E-02	2.23E-03	2.08E-02	4.32E-01	8.36E-02	1.63E+00	1.18E+00	3.13E-02	3.71E-17	-1.06E-02	-1.57E-03	-3.41E-04	8.29E-03	-1.07E-02	-1.69E-03	-1.74E-20	1.50E-19	-5.12E-08	1.08E-06	-2.28E-05
Shapiro-Wilk score	9.362E-01	7.964E-01	8.257E-01	8.392E-01	8.909E-01	8.793E-01	8.984E-01	8.886E-01	5.823E-01	4.234E-01	7.496E-01	8.549E-01	8.544E-01	8.474E-01	7.992E-01	8.566E-01	7.112E-01	3.145E-01	6.530E-01	8.219E-01	7.784E-01

atpg

Local	-2.48E+00	-1.77E-02	1.11E-03	8.98E-03	1.98E-01	3.30E-02	8.27E-01	4.85E-01	-6.16E-02	-2.63E-16	6.66E-02	7.71E-02	1.58E-02	7.12E-02	6.52E-01	1.23E-01	-1.21E-18	-7.32E-18	1.91E-06	-7.06E-07	1.19E-03
Min	-5.43E+01	-2.76E+00	-4.49E-02	-2.99E-01	2.60E-04	-2.13E-01	9.64E-04	4.30E-04	-8.21E+00	-3.59E-13	1.69E-03	2.30E-04	6.03E-05	8.35E-06	7.92E-04	3.18E-04	-2.83E-17	-2.75E-13	2.91E-10	-2.55E-04	8.99E-08
Max	-1.31E-02	-3.37E-05	6.52E-02	4.90E-01	8.76E+00	9.11E-01	2.99E+01	1.67E+01	2.61E+00	6.03E-13	1.78E+00	8.79E-01	1.92E-01	6.96E+00	5.35E+01	1.59E+00	2.24E-16	1.24E-15	1.70E-05	3.07E-06	5.60E-02
Normalized peak height	4.69E-02	2.60E-01	2.10E-01	1.52E-01	1.10E-01	7.08E-02	9.00E-02	8.26E-02	2.34E-01	7.80E-01	2.92E-02	1.55E-02	1.48E-02	2.75E-01	1.72E-01	2.17E-02	4.50E-01	8.65E-01	1.12E-02	3.06E-01	2.09E-01
Peak sensitivity value	-9.45E-02	-1.41E-03	5.42E-05	6.19E-04	4.64E-03	1.48E-03	1.59E-02	8.77E-03	-3.74E-03	3.45E-16	2.21E-02	5.06E-03	1.50E-03	3.49E-03	2.75E-02	5.89E-03	3.90E-20	-1.11E-18	3.67E-07	1.05E-07	2.81E-05
Shapiro-Wilk score	8.073E-01	6.426E-01	8.166E-01	8.140E-01	7.159E-01	7.689E-01	7.167E-01	7.006E-01	7.532E-01	1.569E-01	7.028E-01	8.055E-01	8.151E-01	7.572E-01	5.088E-01	7.818E-01	4.744E-01	2.180E-02	8.271E-01	6.753E-01	7.298E-01

ampc

Local	-1.22E+00	-8.69E-03	-2.58E-04	-1.87E-03	-2.61E-02	-8.72E-03	-1.55E-01	-9.08E-02	-7.98E-02	-6.07E-20	-1.24E-02	-1.44E-02	1.73E+00	3.66E-02	-1.22E-01	-2.30E-02	-3.42E-16	-1.07E-19	-3.57E-07	-9.14E-07	6.12E-04
Min	-1.78E+00	-5.10E-02	-7.23E-03	-8.14E-02	-3.54E-01	-5.54E-02	-1.23E+00	-8.46E-01	-7.23E-01	-6.09E-12	-1.08E-01	-6.21E-02	1.48E+00	-2.28E-02	-1.77E+00	-1.04E-01	-9.67E-16	-4.98E-16	-3.46E-06	-5.13E-06	-8.45E-05
Max	1.50E-01	8.52E-02	-3.49E-06	-2.18E-05	2.34E-03	-3.31E-05	-9.67E-05	-4.96E-05	-1.78E-04	4.12E-12	-4.55E-04	-3.35E-05	1.85E+00	1.09E-01	-9.43E-05	-3.03E-05	5.35E-15	6.88E-17	-6.16E-11	-2.10E-09	2.67E-03
Normalized peak height	8.59E-03	1.72E-02	3.17E-02	4.76E-02	5.05E-02	1.22E-02	2.12E-02	2.40E-02	1.07E-02	8.02E-01	7.02E-03	5.32E-03	2.64E-03	4.38E-03	3.31E-02	6.48E-03	3.36E-01	5.30E-01	9.94E-03	5.57E-03	1.01E-02
Peak sensitivity value	-1.88E-03	-6.26E-03	-5.05E-05	-3.07E-04	2.32E-05	-4.48E-04	-3.16E-03	-1.32E-03	-9.94E-03	2.21E-15	-4.82E-03	-1.18E-03	1.69E+00	5.58E-03	-2.76E-03	-1.43E-03	-2.97E-18	-6.65E-20	-4.68E-08	-1.84E-07	2.33E-06
Shapiro-Wilk score	8.814E-01	8.957E-01	7.365E-01	7.055E-01	7.892E-01	8.736E-01	8.126E-01	8.176E-01	8.322E-01	1.430E-01	8.278E-01	9.320E-01	9.888E-01	9.461E-01	5.979E-01	9.119E-01	9.570E-01	1.694E-01	8.540E-01	9.287E-01	8.901E-01

nad

Local	-2.14E-02	-1.52E-04	-3.47E-06	-2.46E-05	-2.95E-04	-2.18E-04	-3.01E-03	2.72E-02	5.57E-04	-3.62E-18	-2.42E-04	-2.80E-04	-5.76E-05	6.99E-04	-2.37E-03	-4.46E-04	4.40E-21	-1.27E-21	-6.93E-09	6.38E-09	1.17E-05
Min	-1.94E-01	-2.18E-03	-1.87E-04	-1.86E-03	-1.52E-02	-3.52E-03	-5.94E-02	5.83E-03	-5.25E-03	-1.14E-16	-6.17E-03	-5.38E-03	-1.03E-03	-1.15E-04	-9.84E-02	-1.29E-02	-3.28E-19	-1.33E-17	-1.75E-07	-9.42E-08	-3.05E-07
Max	1.71E-02	1.55E-03	8.34E-06	8.43E-05	1.88E-03	-4.43E-06	-1.83E-06	1.30E-01	1.68E-01	8.09E-17	-3.50E-06	-2.74E-07	-7.78E-08	1.31E-02	-7.35E-07	-2.93E-07	6.39E-20	1.49E-18	-7.84E-13	2.54E-07	1.26E-04
Normalized peak height	4.96E-02	2.38E-02	9.27E-02	1.08E-01	4.70E-02	2.24E-02	7.96E-02	6.08E-03	1.23E-01	3.16E-01	2.33E-02	3.51E-02	3.01E-02	2.57E-02	1.25E-01	5.67E-02	3.98E-01	5.90E-01	3.85E-02	1.69E-02	2.23E-02
Peak sensitivity value	-1.00E-04	-7.01E-05	4.77E-08	-1.81E-07	6.43E-05	-2.38E-05	-3.15E-05	1.65E-02	2.00E-04	-9.60E-20	-4.97E-05	-8.34E-06	-1.62E-06	5.05E-05	-4.99E-05	-6.75E-06	1.29E-22	2.58E-21	-4.38E-10	5.89E-09	6.44E-07
Shapiro-Wilk score	9.151E-01	8.206E-01	6.767E-01	6.752E-01	7.515E-01	8.226E-01	7.756E-01	9.019E-01	3.734E-01	6.936E-01	7.403E-01	7.691E-01	7.900E-01	7.819E-01	5.874E-01	7.360E-01	6.514E-01	1.485E-01	6.277E-01	9.186E-01	7.608E-01

fructose16bisphosphate<br>Local<br>Min<br>Max<br>Normalized peak height<br>Peak sensitivity value<br>Shapiro-Wilk score	4.06E-01<br>-9.24E+00<br>2.57E+00<br>3.83E-02<br>-1.05E-02<br>8.959E-01	2.89E-03<br>-2.66E-01<br>7.57E-02<br>1.51E-01<br>-2.99E-05<br>8.094E-01	4.32E-03<br>7.79E-04<br>3.33E-02<br>7.38E-03<br>3.13E-03<br>8.704E-01	3.39E-02<br>5.87E-03<br>2.96E-01<br>8.14E-03<br>2.84E-02<br>8.711E-01	-1.00E-01<br>-6.98E-01<br>2.82E-02<br>1.04E-02<br>-5.21E-02<br>8.436E-01	5.73E-03<br>-5.71E-02<br>3.51E-02<br>1.01E-02<br>6.47E-03<br>9.335E-01	-1.77E-01<br>-2.00E+00<br>2.96E-01<br>4.22E-02<br>-1.14E-02<br>7.891E-01	-1.04E-01<br>-1.40E+00<br>1.61E-01<br>4.38E-02<br>-7.07E-03<br>7.680E-01	1.29E-01<br>-2.60E-02<br>1.13E+01<br>7.96E-02<br>3.63E-02<br>5.203E-01	4.42E-17<br>-9.50E-15<br>3.80E-15<br>6.06E-01<br>1.86E-18<br>4.709E-01	-1.39E-02<br>-2.63E-01<br>1.09E-02<br>2.03E-02<br>-5.13E-03<br>8.693E-01	-1.61E-02<br>-1.55E-01<br>5.51E-03<br>2.54E-02<br>-3.70E-05<br>8.517E-01	-3.31E-03<br>-2.76E-02<br>1.11E-03<br>2.08E-02<br>-2.60E-05<br>8.721E-01	-6.40E-03<br>-1.71E-01<br>3.80E-01<br>1.36E-01<br>3.05E-04<br>8.455E-01	-1.36E-01<br>-3.30E+00<br>3.34E-01<br>8.62E-02<br>-2.87E-03<br>5.918E-01	-2.56E-02<br>-2.39E-01<br>1.04E-02<br>2.69E-02<br>-2.22E-04<br>8.249E-01	2.53E-19<br>-1.94E-18<br>2.04E-18<br>3.67E-01<br>-1.39E-21<br>7.629E-01	2.53E-18<br>-9.37E-16<br>6.75E-15<br>2.60E-01<br>-2.71E-18<br>2.045E-01	-3.99E-07<br>-1.10E-05<br>1.06E-07<br>7.75E-02<br>1.84E-10<br>7.290E-01	1.48E-06<br>-6.71E-07<br>8.92E-06<br>7.90E-03<br>1.18E-06<br>8.953E-01	-1.07E-04<br>-2.78E-03<br>2.34E-03<br>9.22E-02<br>3.92E-06<br>7.877E-01
ampg<br>Local<br>Min<br>Max<br>Normalized peak height<br>Peak sensitivity value<br>Shapiro-Wilk score	1.67E+01<br>2.01E-02<br>6.33E+01<br>1.18E-02<br>1.78E-01<br>9.689E-01	1.19E-01<br>5.35E-03<br>7.38E-01<br>4.55E-03<br>4.16E-02<br>9.235E-01	-7.45E-03<br>-7.95E-02<br>1.48E-02<br>1.37E-02<br>3.90E-04<br>8.739E-01	-6.05E-02<br>-6.38E-01<br>7.42E-02<br>1.50E-02<br>1.92E-03<br>8.746E-01	-1.33E+00<br>-8.03E+00<br>-2.40E-03<br>1.11E-02<br>-2.25E-02<br>8.883E-01	-2.22E-01<br>-1.48E+00<br>1.00E-01<br>1.92E-02<br>-3.29E-03<br>8.832E-01	-5.57E+00<br>-3.10E+01<br>-1.29E-04<br>1.85E-02<br>-1.56E-02<br>8.715E-01	-3.27E+00<br>-1.99E+01<br>-6.12E-05<br>1.91E-02<br>-1.00E-02<br>8.682E-01	4.15E-01<br>-1.71E+01<br>3.96E+00<br>5.39E-02<br>-2.36E-02<br>8.182E-01	1.77E-15<br>-2.56E-13<br>3.90E-13<br>5.90E-01<br>1.60E-16<br>3.091E-01	-4.48E-01<br>-2.04E+00<br>-1.95E-04<br>2.04E-02<br>-3.25E-03<br>8.926E-01	-5.19E-01<br>-1.14E+00<br>-3.70E-05<br>2.44E-02<br>-6.06E-04<br>9.368E-01	-1.07E-01<br>-2.53E-01<br>-6.57E-06<br>2.46E-02<br>-3.86E-04<br>9.321E-01	-4.79E-01<br>-1.77E+00<br>-1.15E-03<br>9.46E-03<br>-2.15E-02<br>8.798E-01	-4.39E+00<br>-6.03E+01<br>-1.23E-04<br>8.32E-02<br>-3.03E-02<br>7.369E-01	-8.26E-01<br>-1.86E+00<br>-4.12E-05<br>2.80E-02<br>-9.73E-04<br>9.416E-01	8.15E-18<br>-4.92E-16<br>6.87E-17<br>4.60E-01<br>-4.44E-21<br>7.276E-01	3.08E-16<br>-2.35E-12<br>2.70E-12<br>5.75E-01<br>-1.54E-15<br>4.378E-01	-1.29E-05<br>-1.42E-04<br>-8.53E-12<br>1.35E-01<br>-7.08E-08<br>8.324E-01	4.75E-06<br>-2.56E-05<br>1.07E-04<br>2.23E-02<br>-3.64E-07<br>8.361E-01	-8.01E-03<br>-6.02E-02<br>-2.03E-06<br>3.20E-02<br>-3.21E-05<br>8.344E-01
atpc<br>Local<br>Min<br>Max<br>Normalized peak height<br>Peak sensitivity value<br>Shapiro-Wilk score	1.92E-01<br>-2.42E-02<br>4.96E-01<br>1.37E-02<br>4.82E-04<br>9.392E-01	1.36E-03<br>-1.29E-02<br>9.51E-03<br>1.59E-02<br>1.09E-03<br>8.868E-01	4.05E-05<br>5.55E-07<br>1.26E-03<br>3.17E-02<br>8.76E-06<br>7.111E-01	2.94E-04<br>4.94E-06<br>1.40E-02<br>4.68E-02<br>5.40E-05<br>6.660E-01	4.09E-03<br>-4.99E-04<br>7.28E-02<br>5.07E-02<br>-2.30E-05<br>7.733E-01	1.37E-03<br>5.33E-06<br>1.21E-02<br>1.35E-02<br>7.20E-05<br>8.551E-01	2.43E-02<br>7.17E-06<br>3.09E-01<br>2.77E-02<br>4.70E-04<br>7.977E-01	1.43E-02<br>4.54E-06<br>1.70E-01<br>2.50E-02<br>2.60E-04<br>7.987E-01	1.25E-02<br>2.03E-05<br>1.15E-01<br>9.20E-03<br>2.14E-03<br>8.579E-01	2.20E-21<br>-1.02E-12<br>6.07E-13<br>8.00E-01<br>-1.74E-16<br>1.222E-01	1.95E-03<br>9.72E-05<br>2.16E-02<br>8.64E-03<br>1.14E-03<br>8.068E-01	2.26E-03<br>1.82E-06<br>1.11E-02<br>5.13E-03<br>2.96E-04<br>9.238E-01	-2.71E-01<br>-5.07E-01<br>-1.53E-01<br>2.61E-03<br>-3.05E-01<br>9.886E-01	-5.75E-03<br>-2.33E-02<br>2.00E-03<br>4.76E-03<br>-1.76E-03<br>9.351E-01	1.91E-02<br>4.55E-06<br>3.85E-01<br>3.63E-02<br>5.83E-04<br>5.910E-01	3.61E-03<br>2.03E-06<br>1.72E-02<br>5.80E-03<br>3.90E-04<br>9.112E-01	9.62E-18<br>-6.96E-16<br>6.00E-17<br>3.33E-01<br>-1.37E-19<br>8.172E-01	1.69E-20<br>-2.19E-17<br>9.65E-17<br>5.34E-01<br>-4.22E-20<br>1.804E-01	5.61E-08<br>1.06E-11<br>6.42E-07<br>1.16E-02<br>3.31E-10<br>8.268E-01	1.44E-07<br>5.15E-10<br>9.98E-07<br>5.90E-03<br>3.49E-08<br>9.153E-01	-9.61E-05<br>-6.02E-04<br>5.24E-06<br>1.31E-02<br>-5.32E-07<br>8.662E-01
glycerol3phosphate cytosol<br>Local<br>Min<br>Max<br>Normalized peak height<br>Peak sensitivity value<br>Shapiro-Wilk score	7.65E-01<br>-2.89E+00<br>2.96E+00<br>9.24E-03<br>2.76E-02<br>9.454E-01	5.45E-03<br>-1.15E-01<br>1.65E-01<br>2.35E-02<br>5.58E-03<br>8.823E-01	4.16E-04<br>-2.97E-03<br>1.11E-02<br>5.32E-02<br>3.66E-05<br>8.121E-01	3.17E-03<br>-2.02E-02<br>6.56E-02<br>4.18E-02<br>3.24E-04<br>8.275E-01	5.53E-02<br>-1.74E-01<br>7.17E-01<br>5.81E-02<br>1.89E-04<br>8.431E-01	1.89E-02<br>5.59E-05<br>1.20E-01<br>1.23E-02<br>7.14E-04<br>9.065E-01	3.33E-01<br>-1.96E-01<br>2.38E+00<br>2.38E-02<br>3.81E-03<br>8.599E-01	1.95E-01<br>-1.59E-01<br>1.78E+00<br>2.96E-02<br>2.38E-03<br>8.494E-01	-1.67E+00<br>-3.13E+00<br>-3.41E-01<br>6.01E-03<br>-1.47E+00<br>9.807E-01	1.60E-16<br>-9.95E-15<br>1.78E-14<br>5.12E-01<br>-3.98E-18<br>5.227E-01	2.68E-02<br>-1.69E-02<br>1.37E-01<br>5.82E-03<br>7.65E-03<br>9.138E-01	3.10E-02<br>-7.54E-03<br>1.23E-01<br>7.11E-03<br>1.91E-03<br>8.942E-01	6.37E-03<br>-1.47E-03<br>2.57E-02<br>6.81E-03<br>5.74E-04<br>9.041E-01	-8.17E-02<br>-6.51E-01<br>-9.54E-04<br>7.33E-03<br>-1.04E-02<br>9.098E-01	2.62E-01<br>-1.31E-01<br>5.36E+00<br>5.78E-02<br>8.72E-03<br>6.550E-01	4.94E-02<br>-9.93E-03<br>2.40E-01<br>9.54E-03<br>3.45E-03<br>8.723E-01	-4.87E-19<br>-4.52E-18<br>4.65E-18<br>3.43E-01<br>3.73E-21<br>7.861E-01	2.34E-19<br>-7.84E-17<br>3.43E-15<br>6.33E-01<br>5.46E-19<br>6.091E-02	7.68E-07<br>-6.72E-08<br>6.09E-06<br>1.65E-02<br>3.56E-09<br>7.890E-01	2.03E-06<br>5.86E-08<br>1.50E-05<br>6.92E-03<br>3.21E-07<br>9.017E-01	-1.37E-03<br>-6.88E-03<br>-1.34E-05<br>5.45E-03<br>-2.23E-04<br>9.093E-01

Table S8: Concentration control coefficients for *Trypanosoma brucei* model with parameter variation of ±40%

	(glucosetransport)	(hexokinase)	(phosphoglycerateisomerase)	(phosphofructokinase)	(aldolase)	(triosephosphateisomerase)	(glyceraldehyde3phosphatedehydrogenase)	(glycerol3phosphatedehydrogenase)	(glycerol3phosphateoxidase)	(pyruvatetransport)	(phosphoglyceratekinase)	(pyruvatekinase)	(atputilisation)	(glycerolkinase)	(phosphoglyceratemutase)	(enolase)	(adenylatekinasecytosol)	(adenylatekinaseglycosome)	(3phosphoglyceratetransport)	(gly3pdhapantiporter)	(glyceroltransport)
adpg<br>Local<br>Min<br>Max<br>Normalized peak height<br>Peak sensitivity value<br>Shapiro-Wilk score	7.11E+00<br>-3.75E+01<br>4.59E+01<br>1.69E-02<br>-5.85E-01<br>9.818E-01	5.06E-02<br>-1.43E+00<br>5.28E-01<br>3.39E-02<br>7.05E-03<br>7.279E-01	-3.17E-03<br>-4.30E-02<br>4.35E-02<br>3.14E-02<br>-4.60E-04<br>8.028E-01	-2.57E-02<br>-4.15E-01<br>5.12E-01<br>3.88E-02<br>-3.85E-03<br>8.032E-01	-5.67E-01<br>-5.95E+00<br>7.62E+00<br>1.98E-02<br>-1.45E-01<br>9.028E-01	-9.47E-02<br>-7.25E-01<br>7.65E-01<br>2.45E-02<br>-4.41E-03<br>8.940E-01	-2.37E+00<br>-2.50E+01<br>2.35E+01<br>5.36E-02<br>2.16E-02<br>9.165E-01	-1.39E+00<br>-1.97E+01<br>1.34E+01<br>3.79E-02<br>-3.08E-03<br>9.035E-01	1.77E-01<br>-2.33E+01<br>1.83E+00<br>1.34E-01<br>3.31E-03<br>5.840E-01	7.55E-16<br>-4.51E-13<br>4.36E-13<br>8.02E-01<br>1.39E-17<br>1.379E-01	-1.91E-01<br>-1.03E+00<br>1.36E+00<br>2.97E-02<br>5.62E-03<br>8.721E-01	-2.21E-01<br>-6.19E-01<br>5.20E-01<br>3.15E-02<br>1.28E-03<br>9.298E-01	-4.54E-02<br>-1.55E-01<br>1.95E-01<br>4.22E-02<br>3.57E-04<br>9.223E-01	-2.04E-01<br>-9.20E-01<br>4.22E+00<br>7.19E-02<br>-3.20E-03<br>8.931E-01	-1.87E+00<br>-4.48E+01<br>4.02E+01<br>1.10E-01<br>-1.45E-02<br>7.749E-01	-3.52E-01<br>-1.16E+00<br>9.66E-01<br>4.01E-02<br>2.81E-03<br>9.393E-01	3.47E-18<br>-3.72E-17<br>3.67E-17<br>3.81E-01<br>2.28E-20<br>6.877E-01	-1.01E-17<br>-3.61E-13<br>3.77E-15<br>8.35E-01<br>-6.27E-17<br>1.857E-02	-5.47E-06<br>-1.16E-04<br>1.33E-05<br>1.00E-01<br>9.05E-08<br>7.415E-01	2.02E-06<br>-1.44E-04<br>4.92E-05<br>8.29E-02<br>-1.17E-07<br>7.426E-01	-3.41E-03<br>-3.07E-02<br>2.31E-02<br>3.99E-02<br>-4.09E-05<br>8.699E-01
adpc<br>Local<br>Min<br>Max<br>Normalized peak height<br>Peak sensitivity value<br>Shapiro-Wilk score	-5.14E-01<br>-8.32E-01<br>2.50E-01<br>4.19E-02<br>-6.95E-04<br>8.759E-01	-3.66E-03<br>-3.56E-02<br>1.60E-01<br>4.59E-02<br>-1.98E-03<br>7.398E-01	-1.09E-04<br>-8.25E-03<br>3.50E-07<br>1.09E-01<br>-1.20E-05<br>6.508E-01	-7.90E-04<br>-1.26E-01<br>1.32E-05<br>1.70E-01<br>-4.96E-05<br>5.883E-01	-1.10E-02<br>-2.18E-01<br>1.68E-03<br>1.12E-01<br>3.54E-05<br>7.716E-01	-3.67E-03<br>-3.64E-02<br>-2.47E-06<br>3.86E-02<br>-5.70E-05<br>8.281E-01	-6.52E-02<br>-6.25E-01<br>-8.03E-06<br>6.62E-02<br>-3.21E-04<br>7.995E-01	-3.83E-02<br>-5.13E-01<br>-3.66E-06<br>8.66E-02<br>-2.60E-04<br>7.903E-01	-3.37E-02<br>-3.75E-01<br>7.79E-03<br>2.56E-02<br>-1.21E-03<br>6.223E-01	0.00E+00<br>-7.26E-12<br>3.23E-12<br>9.22E-01<br>2.81E-15<br>5.073E-02	-5.25E-03<br>-6.10E-02<br>-5.42E-05<br>1.08E-02<br>-1.30E-03<br>7.587E-01	-6.07E-03<br>-4.22E-02<br>-1.90E-06<br>1.92E-02<br>-6.52E-05<br>8.369E-01	7.27E-01<br>3.19E-01<br>8.74E-01<br>2.82E-03<br>7.10E-01<br>9.857E-01	1.54E-02<br>-6.45E-02<br>5.51E-02<br>1.65E-02<br>3.81E-05<br>8.896E-01	-5.14E-02<br>-8.33E-01<br>-3.88E-06<br>9.45E-02<br>-4.20E-04<br>6.035E-01	-9.68E-03<br>-7.93E-02<br>-1.49E-06<br>2.77E-02<br>-1.20E-04<br>7.904E-01	9.75E-18<br>-3.21E-16<br>2.62E-16<br>3.21E-01<br>6.03E-21<br>8.872E-01	-4.52E-20<br>-1.07E-16<br>5.43E-17<br>5.42E-01<br>2.38E-20<br>2.928E-01	-1.50E-07<br>-1.41E-06<br>-4.87E-12<br>4.61E-02<br>-7.08E-10<br>7.579E-01	-3.85E-07<br>-3.56E-06<br>2.03E-07<br>1.39E-02<br>-3.59E-08<br>8.522E-01	2.58E-04<br>-1.18E-04<br>1.43E-03<br>6.84E-02<br>7.12E-07<br>7.529E-01
dihydroxyacetonephosphate<br>Local<br>Min<br>Max<br>Normalized peak height<br>Peak sensitivity value<br>Shapiro-Wilk score	-1.12E-01<br>-1.03E+01<br>2.25E+00<br>9.87E-02<br>3.26E-04<br>8.330E-01	-8.00E-04<br>-4.04E-01<br>3.66E-02<br>1.53E-01<br>-6.69E-04<br>4.980E-01	1.87E-03<br>2.05E-04<br>2.39E-02<br>1.16E-02<br>1.02E-03<br>7.990E-01	1.47E-02<br>8.91E-04<br>3.30E-01<br>1.74E-02<br>1.03E-02<br>7.503E-01	2.89E-01<br>1.91E-02<br>9.32E-01<br>4.62E-03<br>2.41E-01<br>9.470E-01	-7.31E-02<br>-1.99E-01<br>-2.18E-02<br>4.74E-03<br>-4.75E-02<br>9.455E-01	-6.54E-02<br>-1.04E+00<br>2.68E-01<br>7.25E-02<br>-4.59E-03<br>8.127E-01	-3.84E-02<br>-9.09E-01<br>2.09E-01<br>8.72E-02<br>-3.15E-03<br>7.525E-01	6.36E-02<br>-9.94E-03<br>1.07E+01<br>1.07E-01<br>1.68E-02<br>3.190E-01	-2.29E-17<br>-4.60E-15<br>4.50E-15<br>6.38E-01<br>1.63E-18<br>3.453E-01	-5.13E-03<br>-1.89E-01<br>7.61E-03<br>4.35E-02<br>-1.51E-03<br>6.131E-01	-5.94E-03<br>-2.15E-01<br>2.38E-03<br>9.19E-02<br>-1.21E-04<br>5.968E-01	-1.22E-03<br>-4.01E-02<br>9.96E-04<br>7.90E-02<br>-5.29E-05<br>6.406E-01	-6.36E-03<br>-1.30E-01<br>3.55E-01<br>1.32E-01<br>1.92E-05<br>8.915E-01	-5.03E-02<br>-2.71E+00<br>2.10E-01<br>2.15E-01<br>-1.81E-03<br>6.203E-01	-9.47E-03<br>-3.24E-01<br>7.24E-03<br>1.18E-01<br>-2.22E-04<br>5.700E-01	9.33E-20<br>-9.33E-18<br>1.76E-18<br>4.46E-01<br>2.21E-21<br>5.161E-01	1.12E-18<br>-1.43E-16<br>4.51E-15<br>3.34E-01<br>-1.63E-18<br>1.143E-01	-1.47E-07<br>-8.70E-06<br>1.26E-07<br>1.08E-01<br>-2.01E-09<br>3.998E-01	7.28E-07<br>-2.60E-07<br>8.22E-06<br>9.87E-03<br>5.33E-07<br>8.338E-01	-1.06E-04<br>-2.36E-03<br>3.31E-03<br>1.24E-01<br>-2.19E-06<br>7.726E-01
glyceraldehyde3phosphate<br>Local<br>Min<br>Max<br>Normalized peak height<br>Peak sensitivity value<br>Shapiro-Wilk score	-1.73E-01<br>-7.95E+00<br>2.66E+00<br>8.68E-02<br>1.50E-03<br>9.080E-01	-1.23E-03<br>-6.94E-01<br>5.50E-02<br>1.83E-01<br>-7.46E-04<br>5.282E-01	1.89E-03<br>1.25E-04<br>2.06E-02<br>9.79E-03<br>1.04E-03<br>8.053E-01	1.49E-02<br>9.66E-04<br>5.30E-01<br>2.73E-02<br>1.02E-02<br>7.559E-01	2.93E-01<br>1.79E-02<br>9.50E-01<br>4.52E-03<br>2.33E-01<br>9.438E-01	6.17E-02<br>1.46E-02<br>1.59E-01<br>4.88E-03<br>4.10E-02<br>9.515E-01	-9.23E-02<br>-1.21E+00<br>2.94E-01<br>6.57E-02<br>-4.34E-03<br>8.309E-01	-5.41E-02<br>-9.35E-01<br>1.16E-01<br>7.25E-02<br>-2.88E-03<br>7.852E-01	5.22E-02<br>-8.06E-02<br>7.84E+00<br>9.54E-02<br>1.84E-02<br>2.980E-01	-3.55E-17<br>-4.21E-15<br>2.19E-15<br>4.75E-01<br>2.77E-18<br>4.800E-01	-7.29E-03<br>-2.45E-01<br>1.15E-02<br>4.14E-02<br>-2.23E-03<br>6.697E-01	-8.44E-03<br>-1.77E-01<br>5.46E-03<br>6.05E-02<br>-2.91E-04<br>6.736E-01	-1.73E-03<br>-3.92E-02<br>1.02E-03<br>5.75E-02<br>-4.24E-05<br>7.084E-01	-4.30E-04<br>-1.15E-01<br>2.94E-01<br>9.82E-02<br>7.30E-05<br>8.686E-01	-7.14E-02<br>-4.92E+00<br>1.46E-01<br>2.36E-01<br>-3.13E-03<br>6.352E-01	-1.34E-02<br>-2.57E-01<br>1.18E-02<br>6.68E-02<br>-4.19E-04<br>6.358E-01	1.32E-19<br>-3.62E-18<br>2.30E-18<br>3.70E-01<br>1.54E-21<br>6.539E-01	1.13E-18<br>-4.13E-16<br>3.97E-15<br>3.34E-01<br>1.09E-18<br>1.931E-01	-2.09E-07<br>-9.99E-06<br>1.63E-07<br>1.15E-01<br>-4.29E-09<br>4.566E-01	5.97E-07<br>-6.96E-07<br>8.42E-06<br>1.36E-02<br>4.67E-07<br>8.054E-01	-7.19E-06<br>-2.34E-03<br>2.30E-03<br>8.51E-02<br>2.72E-06<br>7.854E-01
2phosphoglycerate<br>Local<br>Min<br>Max<br>Normalized peak height<br>Peak sensitivity value<br>Shapiro-Wilk score	9.33E-01<br>-4.69E-01<br>2.36E+00<br>6.64E-02<br>9.97E-04<br>8.899E-01	6.64E-03<br>-2.77E-01<br>7.14E-02<br>4.56E-02<br>3.60E-03<br>7.583E-01	1.97E-04<br>-4.44E-07<br>1.32E-02<br>1.02E-01<br>1.93E-05<br>6.410E-01	1.43E-03<br>-7.34E-05<br>2.21E-01<br>1.62E-01<br>2.58E-04<br>5.968E-01	1.99E-02<br>-4.38E-03<br>5.02E-01<br>1.30E-01<br>-7.15E-05<br>7.651E-01	6.66E-03<br>2.25E-06<br>7.41E-02<br>4.27E-02<br>1.13E-04<br>8.217E-01	1.18E-01<br>1.44E-05<br>1.43E+00<br>7.86E-02<br>7.27E-04<br>8.012E-01	6.94E-02<br>8.53E-06<br>1.12E+00<br>9.80E-02<br>5.70E-04<br>7.862E-01	6.11E-02<br>-2.14E-02<br>8.87E-01<br>3.35E-02<br>1.77E-03<br>6.010E-01	-8.41E-17<br>-3.15E-13<br>1.27E-12<br>9.20E-01<br>-3.84E-16<br>5.373E-02	9.52E-03<br>1.33E-04<br>1.41E-01<br>1.41E-02<br>2.61E-03<br>7.399E-01	-4.61E-01<br>-1.08E+00<br>-2.86E-01<br>3.75E-03<br>-3.62E-01<br>9.433E-01	-9.48E-02<br>-2.53E-01<br>-5.43E-02<br>3.94E-03<br>-1.05E-01<br>9.772E-01	-2.80E-02<br>-1.28E-01<br>8.13E-02<br>1.63E-02<br>-1.74E-04<br>8.728E-01	9.32E-02<br>7.40E-06<br>2.06E+00<br>1.19E-01<br>1.04E-03<br>6.057E-01	-7.35E-01<br>-2.02E+00<br>-3.07E-01<br>5.08E-03<br>-5.96E-01<br>9.314E-01	7.25E-18<br>-6.67E-15<br>4.12E-16<br>5.09E-01<br>-2.32E-18<br>8.857E-02	8.21E-20<br>-1.09E-16<br>2.17E-16<br>4.47E-01<br>-1.54E-19<br>2.270E-01	2.73E-07<br>3.76E-12<br>3.40E-06<br>5.35E-02<br>1.71E-09<br>7.514E-01	6.99E-07<br>-3.34E-07<br>6.39E-06<br>1.29E-02<br>7.30E-08<br>8.328E-01	-4.68E-04<br>-3.43E-03<br>2.40E-04<br>6.61E-02<br>-5.20E-07<br>7.389E-01
glucose<br>Local<br>Min<br>Max<br>Normalized peak height<br>Peak sensitivity value<br>Shapiro-Wilk score	3.20E+00<br>7.29E-01<br>7.21E+01<br>7.34E-02<br>1.12E+00<br>7.898E-01	-1.04E+00<br>-1.19E+00<br>2.45E+00<br>7.56E-02<br>-1.03E+00<br>7.153E-01	-1.17E-02<br>-2.00E-01<br>7.20E-03<br>1.68E-02<br>-4.51E-03<br>7.808E-01	-7.39E-02<br>-1.83E+00<br>3.19E-02<br>2.11E-02<br>-2.85E-02<br>7.906E-01	-1.69E-01<br>-1.30E+01<br>-3.83E-04<br>1.91E-01<br>-6.89E-03<br>6.698E-01	-2.68E-02<br>-1.65E+00<br>1.94E-01<br>1.60E-01<br>2.04E-05<br>7.239E-01	-6.92E-01<br>-4.92E+01<br>8.21E-03<br>2.09E-01<br>-1.64E-02<br>6.832E-01	-4.06E-01<br>-2.09E+01<br>3.68E-03<br>1.71E-01<br>-6.75E-03<br>6.572E-01	5.93E-02<br>-5.70E+00<br>1.00E+01<br>2.27E-01<br>9.10E-03<br>7.722E-01	2.20E-16<br>-1.61E-12<br>5.18E-13<br>9.07E-01<br>1.47E-16<br>8.253E-02	-5.57E-02<br>-1.74E+00<br>-5.37E-04<br>3.37E-02<br>-1.88E-02<br>6.350E-01	-6.44E-02<br>-9.29E-01<br>-3.66E-05<br>3.68E-02<br>-1.43E-03<br>7.204E-01	-1.33E-02<br>-2.11E-01<br>-6.01E-06<br>3.49E-02<br>-3.23E-04<br>7.345E-01	-5.89E-02<br>-9.26E+00<br>1.79E-02<br>1.68E-01<br>4.01E-03<br>7.474E-01	-5.45E-01<br>-4.79E+01<br>-7.38E-05<br>2.81E-01<br>-2.40E-02<br>5.139E-01	-1.03E-01<br>-1.88E+00<br>-4.82E-05<br>5.56E-02<br>-2.86E-03<br>6.731E-01	1.01E-18<br>-7.64E-17<br>3.33E-17<br>4.24E-01<br>-2.47E-20<br>5.258E-01	-2.56E-19<br>-6.89E-16<br>2.17E-14<br>7.66E-01<br>-5.28E-18<br>3.414E-02	-1.60E-06<br>-2.08E-05<br>-3.41E-11<br>5.54E-02<br>-1.04E-08<br>7.269E-01	6.79E-07<br>-7.68E-06<br>2.58E-04<br>2.10E-01<br>1.72E-07<br>6.445E-01	-9.85E-04<br>-5.47E-02<br>3.05E-04<br>2.08E-01<br>2.90E-06<br>6.524E-01

**fructose6phosphate**																					
Local	1.54E+00	1.10E-02	4.77E-03	-1.07E+00	-1.09E-01	6.21E-03	-1.92E-01	-1.13E-01	1.40E-01	4.79E-17	-1.51E-02	-1.75E-02	-3.59E-03	-6.96E-03	-1.48E-01	-2.78E-02	2.74E-19	2.79E-18	-4.33E-07	1.60E-06	-1.16E-04
Min	-1.22E+01	-6.11E-02	-1.81E-02	-1.78E+00	-6.84E+00	-7.31E-01	-2.03E+01	-9.48E+00	-1.28E-01	-1.43E-13	-8.07E-01	-3.89E-01	-7.35E-02	-2.93E+00	-1.98E+01	-9.67E-01	-9.15E-18	-5.06E-16	-2.17E-05	-4.38E-06	-1.42E-02
Max	3.28E+01	2.21E+00	9.42E-02	-7.23E-01	1.12E-01	7.17E-02	5.07E-01	3.24E-01	1.39E+01	1.56E-13	3.80E-02	2.69E-02	4.93E-03	4.64E-01	8.52E-01	5.11E-02	5.61E-18	5.45E-14	5.53E-07	9.32E-05	5.56E-03
Normalized peak height	9.63E-02	1.62E-01	1.68E-02	1.36E-02	7.21E-02	6.40E-02	1.89E-01	1.96E-01	8.44E-02	8.46E-01	5.82E-02	7.63E-02	5.98E-02	2.89E-01	3.51E-01	1.26E-01	4.06E-01	5.94E-01	9.82E-02	5.40E-02	2.21E-01
Peak sensitivity value	1.06E+00	3.59E-03	2.62E-03	-1.05E+00	-5.16E-02	7.08E-03	-2.35E-02	-4.55E-03	3.32E-02	1.27E-16	-4.69E-03	-2.66E-04	-5.09E-05	7.33E-04	-6.66E-03	-3.39E-04	7.16E-21	1.62E-17	7.55E-09	1.03E-06	4.21E-06
Shapiro-Wilk score	7.734E-01	6.826E-01	7.551E-01	9.020E-01	6.409E-01	7.081E-01	5.952E-01	5.765E-01	4.891E-01	5.872E-02	6.006E-01	7.071E-01	7.305E-01	6.267E-01	4.450E-01	6.533E-01	5.806E-01	3.767E-02	5.543E-01	5.555E-01	6.896E-01
**3phosphoglycerate**																					
Local	3.53E+00	2.51E-02	7.47E-04	5.43E-03	7.54E-02	2.52E-02	4.48E-01	2.63E-01	2.31E-01	2.58E-22	3.60E-02	-4.03E-01	-8.28E-02	-1.06E-01	-3.41E+00	-6.42E-01	6.33E-18	3.11E-19	-9.98E-06	2.65E-06	-1.77E-03
Min	-1.70E+00	-1.02E+00	-4.84E-07	-3.99E-05	-8.01E-02	-5.49E-03	3.38E-05	1.02E-05	-5.59E-02	-7.08E-13	2.15E-04	-9.52E-01	-2.05E-01	-2.07E+00	-6.39E+01	-1.79E+00	-4.33E-16	-1.40E-14	-4.05E-04	-6.11E-07	-4.66E-02
Max	6.28E+01	7.16E-01	7.18E-02	9.44E-01	4.86E+00	5.75E-01	1.46E+01	1.43E+01	1.10E+01	1.03E-12	1.08E+00	-1.92E-04	-4.29E-05	4.83E-01	-6.03E-01	-2.60E-04	5.74E-17	9.18E-15	-9.10E-12	1.20E-04	2.00E-03
Normalized peak height	1.20E-01	6.48E-02	1.51E-01	2.22E-01	2.29E-01	8.36E-02	1.77E-01	2.12E-01	8.93E-02	8.40E-01	3.71E-02	3.91E-03	3.54E-03	4.98E-02	2.79E-02	4.90E-03	3.45E-01	9.08E-01	1.21E-01	7.17E-02	1.20E-01
Peak sensitivity value	7.31E-03	8.85E-03	3.54E-05	4.32E-04	1.49E-03	6.13E-04	7.34E-03	7.16E-03	4.87E-03	-6.96E-16	5.06E-03	-3.42E-01	-8.89E-02	-6.07E-03	-1.27E+00	-4.80E-01	-2.40E-19	6.31E-18	-2.02E-07	1.71E-07	-2.13E-05
Shapiro-Wilk score	7.357E-01	6.753E-01	6.394E-01	6.164E-01	7.556E-01	8.160E-01	8.083E-01	7.753E-01	5.972E-01	4.441E-02	5.708E-01	9.608E-01	9.487E-01	5.869E-01	7.305E-01	9.653E-01	8.318E-01	1.885E-02	7.502E-01	5.420E-01	4.957E-01
**glycerol**																					
Local	9.22E+00	6.57E-02	-2.07E-03	-1.73E-02	-4.20E-01	-1.47E-01	-2.56E+00	-1.50E+00	-1.41E+00	1.05E-15	-2.06E-01	-2.39E-01	-4.91E-02	6.53E-01	-2.02E+00	-3.80E-01	3.75E-18	-1.82E-18	-5.91E-06	-1.61E-05	-9.89E-01
Min	-7.18E+00	-9.23E-01	-7.66E-02	-5.92E-01	-6.54E+00	-9.75E-01	-2.69E+01	-1.81E+01	-2.25E+01	-1.32E-13	-1.04E+00	-6.48E-01	-1.59E-01	1.29E-02	-5.33E+01	-1.38E+00	-4.47E-17	-1.47E-14	-1.10E-04	-2.35E-04	-1.00E+00
Max	5.71E+01	6.64E-01	2.60E-02	4.27E-01	1.51E+00	1.70E-01	4.88E+00	2.97E+00	-7.13E-02	1.90E-13	2.72E-01	1.37E-01	2.56E-02	1.62E+00	7.40E+00	3.20E-01	3.77E-17	1.71E-15	3.50E-06	-2.62E-07	-9.03E-01
Normalized peak height	4.51E-02	2.06E-02	3.73E-02	3.78E-02	1.31E-02	1.14E-02	3.35E-02	5.24E-02	2.65E-02	6.77E-01	2.38E-02	3.06E-02	3.21E-02	8.07E-03	1.43E-01	4.55E-02	4.16E-01	4.65E-01	1.56E-01	8.23E-03	1.61E-02
Peak sensitivity value	-8.08E-02	1.41E-02	9.45E-05	-3.89E-04	-5.75E-02	-2.62E-02	-5.47E-03	7.39E-03	-1.31E+00	4.51E-17	4.31E-03	1.40E-03	2.60E-04	9.54E-01	2.32E-02	2.44E-03	-2.33E-20	-7.54E-18	2.91E-08	-6.49E-06	-9.99E-01
Shapiro-Wilk score	9.145E-01	8.715E-01	8.460E-01	8.419E-01	8.995E-01	8.113E-01	8.327E-01	8.142E-01	7.101E-01	1.968E-01	8.396E-01	8.927E-01	8.893E-01	8.887E-01	6.649E-01	8.927E-01	6.851E-01	1.079E-01	7.202E-01	8.343E-01	8.120E-01
**pyruvate**																					
Local	5.54E+00	3.94E-02	1.17E-03	8.51E-03	1.18E-01	3.96E-02	7.02E-01	4.12E-01	3.62E-01	-7.85E+00	5.65E-02	6.54E-02	1.35E-02	-1.66E-01	5.54E-01	1.04E-01	-1.03E-18	4.87E-19	1.62E-06	4.15E-06	-2.78E-03
Min	-4.42E+01	-2.24E+01	-3.31E-06	-4.01E-05	-1.14E+00	-6.83E-03	4.44E-05	1.44E-05	-6.51E-02	-1.17E+04	2.11E-04	6.86E-06	1.55E-06	-3.46E+02	1.45E-05	6.50E-06	-6.58E-16	-8.35E-15	4.71E-12	-1.33E-06	-3.69E+00
Max	6.46E+03	7.29E+01	4.65E+00	5.10E+01	1.67E+03	1.10E+02	2.89E+03	3.28E+03	1.37E+03	-1.47E+00	1.89E+02	1.58E+02	3.45E+01	3.31E+01	6.94E+03	2.78E+02	7.25E-16	5.11E-15	3.47E-03	9.01E-03	2.64E-02
Normalized peak height	5.00E-01	8.52E-01	8.77E-01	9.07E-01	8.95E-01	9.12E-01	9.13E-01	9.60E-01	9.59E-01	9.04E-01	9.39E-01	9.52E-01	9.52E-01	9.12E-01	9.66E-01	9.56E-01	8.99E-01	9.13E-01	9.46E-01	9.24E-01	9.32E-01
Peak sensitivity value	4.54E+00	5.33E-03	2.32E-03	2.55E-02	-3.01E-01	4.82E-02	1.44E+00	1.64E+00	6.19E-01	-7.34E+00	9.49E-02	7.91E-02	1.72E-02	-4.73E-03	3.47E+00	1.39E-01	-1.20E-19	-1.22E-18	1.74E-06	3.18E-06	-1.53E-03
Shapiro-Wilk score	3.865E-02	4.563E-02	4.836E-02	4.222E-02	1.775E-02	3.461E-02	3.599E-02	1.907E-02	1.935E-02	4.180E-02	2.719E-02	2.528E-02	2.514E-02	2.643E-02	1.700E-02	2.431E-02	3.808E-02	3.308E-02	2.357E-02	3.099E-02	2.818E-02
**dihydroxyacetonephosphate cytosol**																					
Local	-3.10E-01	-2.20E-03	-1.68E-04	-1.28E-03	-2.24E-02	-7.65E-03	-1.35E-01	-7.90E-02	6.75E-01	-6.46E-17	-1.08E-02	-1.25E-02	-2.58E-03	3.30E-02	-1.06E-01	-2.00E-02	1.97E-19	-9.46E-20	-3.10E-07	-8.20E-07	5.52E-04
Min	-1.54E+01	-2.48E-01	-2.84E-02	-1.91E-01	-2.23E+00	-2.86E-01	-4.67E+00	-4.23E+00	4.96E-02	-2.08E-14	-6.47E-01	-6.32E-01	-8.71E-02	3.25E-05	-1.38E+01	-1.00E+00	-1.58E-17	-2.84E-15	-3.12E-05	-4.36E-05	4.61E-07
Max	7.64E+00	2.71E-01	6.30E-03	3.87E-02	1.68E-01	-1.39E-06	1.35E-01	7.84E-02	1.83E+01	1.04E-14	2.30E-03	1.06E-03	2.81E-04	8.42E-01	3.34E-02	1.26E-03	8.82E-18	7.51E-17	4.86E-08	-3.22E-09	1.51E-02
Normalized peak height	2.03E-01	1.26E-01	2.70E-01	2.82E-01	2.92E-01	1.64E-01	2.35E-01	2.61E-01	4.50E-02	7.17E-01	1.36E-01	1.94E-01	1.51E-01	5.85E-02	3.69E-01	2.45E-01	5.58E-01	7.33E-01	2.41E-01	7.96E-02	7.76E-02
Peak sensitivity value	-7.46E-03	-7.06E-04	-2.15E-05	-5.57E-05	-7.72E-04	-1.44E-04	-2.26E-03	-1.40E-03	2.05E-01	-6.43E-18	-6.16E-04	-5.25E-04	-2.47E-05	4.54E-04	-1.05E-03	-2.46E-04	-4.04E-21	8.21E-19	1.77E-09	-2.50E-08	2.32E-05
Shapiro-Wilk score	6.570E-01	6.828E-01	3.896E-01	4.589E-01	4.632E-01	5.015E-01	5.348E-01	4.748E-01	5.264E-01	1.904E-01	4.358E-01	4.029E-01	4.268E-01	6.510E-01	3.809E-01	3.717E-01	3.524E-01	4.932E-02	3.163E-01	5.761E-01	5.493E-01
**nadh**																					
Local	1.19E+00	8.44E-03	1.92E-04	1.37E-03	1.64E-02	1.21E-02	1.67E-01	-1.51E+00	-3.09E-02	2.01E-16	1.34E-02	1.55E-02	3.19E-03	-3.88E-02	1.31E-01	2.47E-02	-2.44E-19	7.06E-20	3.85E-07	-3.54E-07	-6.48E-04
Min	-5.22E+00	-2.42E-01	-1.78E-03	-1.17E-02	-1.14E-01	4.28E-04	1.14E-04	-3.20E+00	-1.31E+01	-1.09E-14	1.71E-04	5.32E-06	1.29E-06	-5.77E-01	1.48E-05	7.41E-06	-2.82E-18	-8.05E-17	4.23E-12	-9.95E-06	-5.32E-03
Max	1.37E+01	1.06E-01	1.32E-02	1.98E-01	9.30E-01	1.76E-01	2.80E+00	-4.69E-01	1.62E-01	1.28E-14	4.83E-01	2.74E-01	4.93E-02	2.11E-02	1.21E+01	4.55E-01	3.63E-17	5.04E-16	1.50E-05	6.01E-06	2.19E-05
Normalized peak height	1.23E-01	3.63E-02	1.02E-01	1.49E-01	4.15E-02	2.20E-02	1.05E-01	7.51E-03	1.48E-01	5.91E-01	3.24E-02	4.53E-02	3.37E-02	2.51E-02	2.86E-01	6.34E-02	4.50E-01	4.70E-01	1.12E-01	1.04E-02	3.50E-02
Peak sensitivity value	1.10E-02	4.85E-03	-1.30E-06	-1.01E-04	-4.51E-03	2.27E-03	1.51E-03	-1.29E+00	-1.68E-02	4.83E-18	2.83E-03	1.42E-04	7.52E-05	-2.54E-03	6.08E-03	2.35E-04	1.71E-20	-1.39E-19	7.50E-09	-4.12E-07	-7.46E-06
Shapiro-Wilk score	8.687E-01	7.691E-01	6.825E-01	6.461E-01	8.148E-01	8.405E-01	8.075E-01	9.539E-01	3.000E-01	5.874E-01	7.453E-01	7.296E-01	7.647E-01	7.253E-01	6.272E-01	6.853E-01	4.854E-01	1.961E-01	5.185E-01	9.401E-01	7.301E-01
**glycerol3phosphate**																					
Local	9.63E-01	6.85E-03	2.45E-03	1.91E-02	3.66E-01	-4.66E-02	4.02E-01	2.36E-01	-2.28E+00	2.02E-16	3.25E-02	3.76E-02	7.73E-03	-1.21E-01	3.18E-01	5.99E-02	-5.90E-19	1.45E-18	9.31E-07	-2.61E-05	-2.02E-03
Min	-9.98E+00	-4.40E-01	-3.78E-03	-1.35E-02	-3.98E-01	-1.74E-01	-2.08E+00	-1.12E+00	-1.59E+01	-7.01E-14	-1.74E-01	-1.10E-01	-2.10E-02	-1.69E+00	-4.25E+00	-1.16E-01	-1.22E-17	-1.48E-16	-4.80E-07	-3.08E-04	-1.99E-02
Max	1.45E+01	5.32E-01	4.22E-02	4.78E-01	3.44E+00	2.34E-01	5.81E+00	5.99E+00	1.56E-01	7.27E-14	5.05E-01	4.36E-01	7.42E-02	-2.74E-04	1.70E+01	8.44E-01	1.19E-17	6.53E-15	2.55E-05	1.14E-06	-5.15E-06
Normalized peak height	6.28E-02	5.91E-02	1.68E-02	2.03E-02	1.23E-02	9.22E-03	1.10E-01	1.59E-01	1.90E-02	6.74E-01	3.05E-02	6.00E-02	4.63E-02	2.07E-02	2.18E-01	7.74E-02	4.02E-01	2.66E-01	1.02E-01	8.55E-03	1.71E-02
Peak sensitivity value	3.01E-02	4.76E-03	1.30E-03	1.08E-02	2.17E-01	-4.13E-02	-1.79E-03	1.48E-03	-1.47E+00	-5.72E-17	3.21E-03	7.47E-04	8.99E-05	-6.17E-03	-6.52E-04	2.74E-04	6.93E-21	2.71E-18	8.61E-10	-1.70E-05	-1.14E-04
Shapiro-Wilk score	9.522E-01	8.390E-01	7.511E-01	7.479E-01	8.282E-01	9.492E-01	7.725E-01	7.073E-01	7.348E-01	2.177E-01	7.197E-01	6.535E-01	6.834E-01	8.911E-01	5.760E-01	6.126E-01	5.842E-01	1.203E-01	5.144E-01	8.659E-01	7.869E-01
**glucose6phosphate**																					
Local	1.66E+00	1.18E-02	-1.68E-01	-1.04E+00	-1.04E-01	6.02E-03	-1.84E-01	-1.08E-01	1.35E-01	4.60E-17	-1.45E-02	-1.68E-02	-3.45E-03	-6.64E-03	-1.42E-01	-2.67E-02	2.64E-19	2.69E-18	-4.16E-07	1.54E-06	-1.11E-04
Min	-7.19E+00	-6.32E-02	-4.09E-01	-1.74E+00	-1.50E+01	-7.45E-01	-3.04E+01	-2.34E+01	-1.54E-01	-2.15E-13	-8.48E-01	-3.27E-01	-8.84E-02	-5.21E+00	-1.95E+01	-6.53E-01	-9.14E-18	-1.41E-15	-2.11E-05	-5.27E-06	-1.55E-02
Max	7.13E+01	2.25E+00	-5.11E-02	-7.39E-01	1.80E-01	7.71E-02	6.25E-01	5.08E-01	9.39E+00	3.22E-13	3.90E-02	3.05E-02	5.69E-03	3.94E-01	1.03E+00	4.55E-02	6.26E-18	4.11E-14	4.42E-07	1.05E-04	5.24E-03
Normalized peak height	1.47E-01	1.36E-01	4.36E-03	1.04E-02	1.57E-01	6.80E-02	3.13E-01	3.34E-01	5.96E-02	9.13E-01	6.19E-02	6.40E-02	7.15E-02	3.17E-01	2.69E-01	9.55E-02	4.29E-01	5.06E-01	1.23E-01	6.38E-02	1.97E-01
Peak sensitivity value	1.17E+00	5.10E-03	-1.40E-01	-1.03E+00	-3.98E-02	6.81E-03	-1.11E-02	-5.44E-03	3.18E-02	1.70E-16	-3.99E-03	-4.44E-04	-9.76E-05	-1.02E-03	-1.23E-02	-2.74E-04	-3.75E-22	1.60E-17	4.72E-10	9.50E-07	1.12E-05
Shapiro-Wilk score	7.861E-01	6.822E-01	9.716E-01	9.190E-01	5.866E-01	7.092E-01	6.045E-01	5.710E-01	5.014E-01	4.233E-02	6.336E-01	7.206E-01	7.394E-01	6.495E-01	4.675E-01	6.720E-01	5.782E-01	5.458E-02	5.682E-01	5.789E-01	7.038E-01
**3phosphoglycerate cytosol**																					
Local	3.53E+00	2.51E-02	7.47E-04	5.43E-03	7.54E-02	2.52E-02	4.48E-01	2.63E-01	2.31E-01	0.00E+00	3.60E-02	-4.03E-01	-8.28E-02	-1.06E-01	-3.41E+00	-6.42E-01	6.33E-18	3.11E-19	1.03E-06	2.65E-06	-1.77E-03
Min	-2.21E+00	-1.89E+00	-1.32E-03	-2.38E-02	-1.01E+00	-3.31E-02	-2.71E+00	-8.01E-01	-1.61E-01	-5.55E-13	-1.32E-02	-1.00E+00	-2.24E-01	-2.55E+00	-4.89E+01	-1.80E+00	-5.52E-17	-5.04E-15	-7.46E-07	-1.98E-06	-5.19E-02
Max	4.71E+01	6.85E-01	7.27E-02	1.49E+00	4.57E+00	6.03E-01	1.51E+01	1.08E+01	8.70E+00	2.18E-12	1.07E+00	-2.66E-04	-3.79E-05	6.49E-01	-5.71E-01	-2.28E-04	5.06E-17	5.52E-15	8.03E-06	8.85E-05	8.17E-04
Normalized peak height	8.77E-02	9.43E-02	1.65E-01	2.02E-01	2.37E-01	1.13E-01	1.13E-01	1.23E-01	7.66E-02	9.05E-01	3.77E-02	4.14E-03	4.01E-03	6.15E-02	2.08E-02	4.63E-03	3.33E-01	8.10E-01	1.44E-02	5.45E-02	1.19E-01
Peak sensitivity value	-1.55E-02	8.70E-03	4.63E-05	1.16E-03	1.75E-03	3.31E-04	1.31E-02	8.12E-03	1.16E-02	8.94E-16	4.72E-03	-3.30E-01	-8.54E-02	-5.97E-03	-1.22E+00	-5.15E-01	-4.73E-20	2.57E-18	3.95E-08	1.50E-07	-5.36E-05
Shapiro-Wilk score	7.521E-01	6.785E-01	6.402E-01	6.095E-01	7.590E-01	8.106E-01	8.067E-01	7.721E-01	6.098E-01	3.074E-02	5.727E-01	9.607E-01	9.495E-01	5.840E-01	7.408E-01	9.657E-01	8.787E-01	4.123E-02	8.410E-01	5.585E-01	4.858E-01
**phosphoenolpyruvate**																					
Local	4.22E-01	3.01E-03	8.93E-05	6.49E-04	9.02E-03	3.02E-03	5.36E-02	3.14E-02	2.76E-02	-1.05E-16	4.31E-03	-4.91E-01	-1.01E-01	-1.27E-02	4.22E-02	7.95E-03	7.72E-18	3.72E-20	1.24E-07	3.16E-07	-2.12E-04
Min	-1.80E-01	-1.49E-01	-2.85E-07	-8.22E-06	-2.38E-03	-1.47E-04	1.04E-05	2.74E-06	-5.48E-03	-8.48E-13	4.40E-05	-1.12E+00	-2.49E-01	-6.80E-02	5.70E-06	2.70E-06	-3.49E-14	-4.43E-17	7.54E-13	-1.54E-07	-1.74E-03
Max	1.07E+00	3.72E-02	7.67E-03	1.29E-01	2.72E-01	4.03E-02	7.01E-01	5.75E-01	5.03E-01	8.18E-13	5.64E-02	-3.10E-01	-5.53E-02	7.44E-02	1.10E+00	7.48E-02	7.20E-17	1.56E-16	1.84E-06	3.51E-06	1.62E-04
Normalized peak height	4.79E-02	4.87E-02	1.14E-01	1.97E-01	1.31E-01	4.40E-02	7.44E-02	9.64E-02	3.55E-02	9.14E-01	1.11E-02	3.60E-03	3.67E-03	2.15E-02	1.21E-01	2.76E-02	7.06E-01	5.41E-01	5.65E-02	1.40E-02	6.19E-02
Peak sensitivity value	-4.30E-04	1.78E-03	1.12E-05	5.63E-05	-4.94E-05	7.54E-05	3.61E-04	2.90E-04	8.73E-04	5.04E-16	1.20E-03	-3.83E-01	-1.06E-01	-1.20E-04	5.55E-04	1.15E-04	-1.55E-17	8.02E-20	9.20E-10	3.08E-08	-1.22E-06
Shapiro-Wilk score	9.038E-01	7.180E-01	6.301E-01	5.641E-01	7.536E-01	8.088E-01	7.921E-01	7.734E-01	6.098E-01	5.842E-02	7.494E-01	9.399E-01	9.782E-01	8.758E-01	5.964E-01	7.795E-01	5.931E-03	1.915E-01	7.206E-01	8.369E-01	7.356E-01
**bisphosphoglycerate**																					
Local	-5.89E+00	-4.19E-02	5.08E-03	4.06E-02	8.47E-01	1.55E-01	3.68E+00	2.16E+00	4.96E-03	-1.01E-15	-8.02E-02	-9.29E-02	-1.91E-02	1.64E-01	-7.86E-01	-1.48E-01	1.46E-18	3.25E-18	-2.30E-06	5.67E-08	2.74E-03
Min	-5.38E+01	-7.83E-01	-3.97E-03	-1.78E-02	8.20E-03	-2.76E-02	8.66E-02	3.89E-02	-1.80E+00	-1.77E-13	-1.23E+00	-7.95E-01	-1.70E-01	-4.50E-01	-2.98E+01	-1.64E+00	-3.97E-17	-6.67E-16	-2.51E-04	-8.60E-05	-3.54E-03
Max	5.84E+00	1.97E-01	9.21E-02	8.15E-01	7.40E+00	1.12E+00	3.66E+01	1.60E+01	4.53E+01	1.16E-13	-4.20E-04	-1.69E-05	-3.68E-06	1.44E+00	-4.41E-05	-1.88E-05	4.29E-17	6.75E-15	-1.09E-11	3.29E-05	3.13E-02
Normalized peak height	7.53E-02	2.37E-02	2.24E-02	2.17E-02	1.20E-02	1.29E-02	1.66E-02	1.08E-02	1.94E-01	6.37E-01	2.63E-02	4.77E-02	4.02E-02	3.36E-02	1.94E-01	7.73E-02	4.21E-01	2.52E-01	3.08E-01	4.39E-02	6.81E-02
Peak sensitivity value	1.91E-02	-9.37E-03	1.65E-03	1.51E-02	3.52E-01	5.37E-02	1.46E+00	9.58E-01	5.93E-02	-2.66E-17	-5.97E-03	-4.14E-04	-8.86E-05	3.85E-03	-1.50E-02	-8.40E-04	-6.16E-21	-3.27E-18	-1.25E-07	8.72E-07	-2.29E-06
Shapiro-Wilk score	8.426E-01	7.955E-01	7.213E-01	7.201E-01	8.113E-01	8.035E-01	8.147E-01	8.056E-01	3.402E-01	2.396E-01	7.614E-01	8.411E-01	8.416E-01	7.552E-01	7.811E-01	8.405E-01	6.654E-01	2.918E-01	6.266E-01	7.376E-01	6.385E-01
**atpg**																					
Local	-2.48E+00	-1.77E-02	1.11E-03	8.98E-03	1.98E-01	3.30E-02	8.27E-01	4.85E-01	-6.16E-02	-2.63E-16	6.66E-02	7.71E-02	1.58E-02	7.12E-02	6.52E-01	1.23E-01	-1.21E-18	-7.32E-18	1.91E-06	-7.06E-07	1.19E-03
Min	-9.37E+01	-3.37E+00	-7.86E-02	-4.02E-01	4.61E-05	-4.35E-01	-4.58E-03	-1.90E-03	-1.51E+01	-1.11E-12	8.96E-04	5.90E-05	1.34E-05	1.73E-06	1.83E-04	4.92E-05	-4.17E-17	-1.85E-13	2.89E-11	-3.34E-04	2.56E-08
Max	-7.56E-03	-1.35E-05	2.67E-01	2.72E+00	2.13E+01	2.70E+00	5.91E+01	2.76E+01	3.83E+00	9.37E-13	2.92E+00	2.05E+00	3.88E-01	1.41E+01	8.60E+01	3.95E+00	1.39E-16	1.24E-15	2.95E-05	5.41E-06	8.73E-02
Normalized peak height	1.22E-01	3.06E-01	3.13E-01	2.77E-01	2.54E-01	1.66E-01	2.37E-01	2.11E-01	3.10E-01	8.98E-01	5.38E-02	7.35E-02	6.27E-02	3.33E-01	3.59E-01	1.08E-01	4.53E-01	6.64E-01	6.10E-02	3.07E-01	2.85E-01
Peak sensitivity value	-5.44E-02	-1.70E-03	1.43E-05	-4.96E-04	1.07E-02	2.18E-03	2.50E-02	1.19E-02	-4.58E-03	-4.79E-17	1.99E-02	3.13E-03	5.95E-04	7.06E-03	4.32E-02	2.02E-03	8.02E-20	2.79E-17	1.48E-08	1.52E-07	4.37E-05
Shapiro-Wilk score	7.186E-01	7.082E-01	7.645E-01	7.379E-01	6.250E-01	6.672E-01	6.380E-01	6.082E-01	7.276E-01	1.006E-01	5.869E-01	6.713E-01	6.815E-01	7.069E-01	4.641E-01	6.209E-01	4.773E-01	3.095E-02	6.970E-01	5.965E-01	6.122E-01
**ampc**																					
Local	-1.22E+00	-8.69E-03	-2.58E-04	-1.87E-03	-2.61E-02	-8.72E-03	-1.55E-01	-9.08E-02	-7.98E-02	-6.07E-20	-1.24E-02	-1.44E-02	1.73E+00	3.66E-02	-1.22E-01	-2.30E-02	-3.42E-16	-1.07E-19	-3.57E-07	-9.14E-07	6.12E-04
Min	-1.82E+00	-7.19E-02	-1.50E-02	-1.85E-01	-4.96E-01	-7.15E-02	-1.49E+00	-1.09E+00	-8.43E-01	-1.24E-11	-1.59E-01	-1.03E-01	1.36E+00	-1.05E-01	-1.81E+00	-1.74E-01	-1.74E-15	-1.95E-16	-4.18E-06	-8.90E-06	-2.78E-04
Max	3.61E-01	2.20E-01	5.94E-06	6.57E-05	6.16E-03	1.13E-03	-2.02E-05	-1.41E-05	2.54E-03	1.73E-11	-1.61E-04	-4.83E-06	1.87E+00	1.42E-01	-1.22E-05	-4.65E-06	4.90E-15	1.05E-16	6.00E-16	3.93E-08	3.74E-03
Normalized peak height	4.24E-02	2.88E-02	8.23E-02	1.30E-01	8.03E-02	3.03E-02	6.06E-02	7.38E-02	2.14E-02	8.94E-01	1.14E-02	1.75E-02	2.75E-03	1.42E-02	8.09E-02	2.34E-02	3.20E-01	5.12E-01	5.00E-02	1.24E-02	6.97E-02
Peak sensitivity value	3.10E-04	-5.25E-03	-1.66E-05	-2.12E-04	-1.17E-04	-1.46E-04	-7.64E-04	-5.58E-04	-2.96E-03	1.39E-14	-3.89E-03	-2.62E-04	1.68E+00	2.56E-04	-9.17E-04	-2.65E-04	2.72E-18	-1.29E-20	-2.09E-09	-9.03E-08	1.87E-06
Shapiro-Wilk score	8.431E-01	7.437E-01	6.532E-01	5.930E-01	7.733E-01	8.308E-01	7.955E-01	7.919E-01	6.301E-01	5.510E-02	7.602E-01	8.426E-01	9.850E-01	8.955E-01	6.012E-01	7.982E-01	9.718E-01	2.833E-01	7.549E-01	8.590E-01	7.544E-01

**nad**																					
Local	-2.14E-02	-1.52E-04	-3.47E-06	-2.46E-05	-2.95E-04	-2.18E-04	-3.01E-03	2.72E-02	5.57E-04	-3.62E-18	-2.42E-04	-2.80E-04	-5.76E-05	6.99E-04	-2.37E-03	-4.46E-04	4.40E-21	-1.27E-21	-6.93E-09	6.38E-09	1.17E-05
Min	-4.06E-01	-3.55E-03	-4.46E-04	-4.48E-03	-3.79E-02	-7.45E-03	-1.39E-01	3.39E-03	-8.61E-03	-3.72E-16	-1.22E-02	-1.34E-02	-2.34E-03	-8.15E-04	-2.69E-01	-1.86E-02	-8.30E-19	-6.81E-18	-8.98E-07	-2.08E-07	-6.22E-07
Max	1.51E-01	5.64E-03	4.11E-05	3.80E-04	3.76E-03	-1.52E-06	-8.51E-07	2.27E-01	3.97E-01	2.30E-16	-8.05E-07	-1.66E-08	-3.49E-09	2.38E-02	-4.84E-08	-2.11E-08	1.24E-19	2.51E-18	-6.06E-14	5.88E-07	3.63E-04
Normalized peak height	1.43E-01	5.90E-02	2.21E-01	2.18E-01	1.20E-01	6.26E-02	2.33E-01	9.83E-03	3.27E-01	6.44E-01	6.45E-02	1.66E-01	1.33E-01	8.19E-02	3.46E-01	1.76E-01	5.19E-01	5.23E-01	3.10E-01	4.19E-02	1.42E-01
Peak sensitivity value	-3.03E-04	-3.27E-05	-2.51E-08	-9.25E-07	2.85E-05	-2.01E-05	-7.05E-05	1.34E-02	1.06E-04	7.12E-20	-1.91E-05	-6.73E-06	-1.17E-06	3.28E-05	-1.35E-04	-9.34E-06	-3.59E-22	-1.31E-21	-4.49E-10	3.29E-09	2.87E-07
Shapiro-Wilk score	7.349E-01	6.920E-01	5.658E-01	5.682E-01	6.955E-01	7.001E-01	7.217E-01	8.193E-01	1.817E-01	4.160E-01	5.728E-01	5.415E-01	5.725E-01	5.346E-01	5.365E-01	5.166E-01	3.914E-01	2.797E-01	3.470E-01	7.285E-01	5.416E-01
**fructose16bisphosphate**																					
Local	4.06E-01	2.89E-03	4.32E-03	3.39E-02	-1.00E-01	5.73E-03	-1.77E-01	-1.04E-01	1.29E-01	4.42E-17	-1.39E-02	-1.61E-02	-3.31E-03	-6.40E-03	-1.36E-01	-2.56E-02	2.53E-19	2.53E-18	-3.99E-07	1.48E-06	-1.07E-04
Min	-1.85E+01	-8.20E-01	3.88E-04	2.29E-03	-1.51E+00	-1.28E-01	-2.56E+00	-2.12E+00	-2.91E-01	-7.16E-15	-4.87E-01	-2.74E-01	-4.37E-02	-2.50E-01	-1.22E+01	-5.92E-01	-4.66E-18	-6.90E-16	-3.52E-05	-5.82E-06	-4.76E-03
Max	5.89E+00	3.28E-01	5.91E-02	6.45E-01	1.81E-01	6.51E-02	6.60E-01	4.76E-01	2.01E+01	9.90E-15	6.83E-02	4.01E-02	7.07E-03	5.95E-01	1.61E+00	5.61E-02	2.81E-18	1.96E-14	2.95E-07	1.62E-05	7.05E-03
Normalized peak height	1.17E-01	2.68E-01	1.16E-02	1.46E-02	2.62E-02	2.19E-02	7.90E-02	1.01E-01	2.02E-01	6.86E-01	9.12E-02	1.17E-01	1.07E-01	1.84E-01	3.10E-01	1.39E-01	4.25E-01	4.65E-01	2.95E-01	1.62E-02	2.42E-01
Peak sensitivity value	-9.67E-03	3.32E-04	2.12E-03	2.06E-02	-4.40E-02	5.05E-03	-1.08E-02	-4.97E-03	2.60E-02	-4.79E-18	-2.83E-04	1.22E-05	-1.37E-05	4.37E-04	-7.45E-03	8.93E-05	-3.41E-21	-9.61E-18	-6.84E-09	7.80E-07	6.41E-06
Shapiro-Wilk score	8.046E-01	6.478E-01	8.027E-01	7.789E-01	7.297E-01	8.631E-01	6.872E-01	6.329E-01	3.302E-01	3.370E-01	6.326E-01	6.222E-01	6.652E-01	7.844E-01	4.775E-01	5.767E-01	5.856E-01	6.388E-02	4.359E-01	8.388E-01	6.810E-01
**ampg**																					
Local	1.67E+01	1.19E-01	-7.45E-03	-6.05E-02	-1.33E+00	-2.22E-01	-5.57E+00	-3.27E+00	4.15E-01	1.77E-15	-4.48E-01	-5.19E-01	-1.07E-01	-4.79E-01	-4.39E+00	-8.26E-01	8.15E-18	3.08E-16	-1.29E-05	4.75E-06	-8.01E-03
Min	5.18E-03	2.67E-03	-1.18E-01	-8.90E-01	-1.21E+01	-1.97E+00	-5.29E+01	-3.72E+01	-5.83E+01	-2.26E-13	-2.11E+00	-1.31E+00	-3.08E-01	-2.10E+00	-1.01E+02	-3.12E+00	-8.59E-17	-5.80E-12	-2.04E-04	-7.40E-05	-7.45E-02
Max	1.05E+02	1.47E+00	2.75E-02	1.61E-01	-1.63E-04	1.12E-01	5.23E-03	1.80E-03	5.73E+00	4.27E-13	-1.26E-05	-2.88E-06	-7.46E-07	-4.32E-04	-1.11E-05	-3.68E-06	7.97E-17	5.77E-12	-3.67E-13	1.62E-04	-2.18E-07
Normalized peak height	7.21E-02	1.16E-02	4.53E-02	4.24E-02	5.22E-02	6.27E-02	8.38E-02	1.04E-01	2.48E-01	6.46E-01	9.02E-02	1.17E-01	1.19E-01	2.41E-02	1.89E-01	1.51E-01	4.79E-01	6.59E-01	2.83E-01	6.30E-02	1.45E-01
Peak sensitivity value	5.76E-02	1.37E-02	2.12E-04	5.79E-04	-6.21E-03	-1.51E-03	-2.12E-02	-1.68E-02	-1.19E-03	2.40E-16	-1.07E-03	-6.58E-04	-1.55E-04	-7.79E-03	-5.07E-02	-1.56E-03	-3.32E-20	1.75E-15	-1.02E-07	2.89E-07	-3.74E-05
Shapiro-Wilk score	8.871E-01	8.970E-01	7.191E-01	7.262E-01	7.464E-01	7.407E-01	7.389E-01	7.266E-01	4.491E-01	1.863E-01	8.279E-01	8.792E-01	8.776E-01	7.841E-01	5.651E-01	8.755E-01	6.940E-01	3.653E-01	7.273E-01	7.051E-01	6.854E-01
**atpc**																					
Local	1.92E-01	1.36E-03	4.05E-05	2.94E-04	4.09E-03	1.37E-03	2.43E-02	1.43E-02	1.25E-02	2.20E-21	1.95E-03	2.26E-03	-2.71E-01	-5.75E-03	1.91E-02	3.61E-03	9.62E-18	1.69E-20	5.61E-08	1.44E-07	-9.61E-05
Min	-6.33E-02	-5.82E-02	-5.14E-07	-8.60E-06	-1.36E-03	1.54E-06	4.91E-06	1.67E-06	-6.67E-05	-2.45E-12	2.21E-05	1.06E-06	-6.45E-01	-3.32E-02	2.68E-06	1.00E-06	-5.86E-16	-6.11E-17	-1.69E-17	-2.06E-09	-1.26E-03
Max	6.26E-01	1.84E-02	2.66E-03	4.63E-02	1.01E-01	1.92E-02	4.26E-01	2.45E-01	1.71E-01	2.80E-12	3.78E-02	2.05E-02	-1.27E-01	1.13E-02	4.77E-01	2.98E-02	8.93E-17	1.10E-16	8.03E-07	1.91E-06	5.34E-05
Normalized peak height	5.30E-02	4.05E-02	7.36E-02	1.48E-01	7.66E-02	3.90E-02	7.79E-02	7.62E-02	2.15E-02	9.38E-01	1.46E-02	1.63E-02	2.66E-03	1.48E-02	9.67E-02	1.93E-02	3.23E-01	6.68E-01	5.16E-02	1.31E-02	6.37E-02
Peak sensitivity value	4.27E-04	9.03E-04	6.15E-06	6.09E-05	-7.64E-05	3.03E-05	2.18E-04	1.24E-04	5.32E-04	1.12E-15	7.59E-04	5.23E-05	-3.20E-01	-4.05E-05	2.41E-04	4.57E-05	-1.39E-19	-1.78E-20	4.01E-10	1.80E-08	-1.06E-06
Shapiro-Wilk score	8.934E-01	6.742E-01	6.201E-01	5.351E-01	7.526E-01	7.978E-01	7.747E-01	7.669E-01	6.646E-01	4.819E-02	7.381E-01	8.266E-01	9.856E-01	8.616E-01	5.885E-01	8.015E-01	8.774E-01	1.634E-01	7.133E-01	8.324E-01	7.123E-01
**glycerol3phosphate cytosol**																					
Local	7.65E-01	5.45E-03	4.16E-04	3.17E-03	5.53E-02	1.89E-02	3.33E-01	1.95E-01	-1.67E+00	1.60E-16	2.68E-02	3.10E-02	6.37E-03	-8.17E-02	2.62E-01	4.94E-02	-4.87E-19	2.34E-19	7.68E-07	2.03E-06	-1.37E-03
Min	-3.25E+00	-2.07E-01	-5.93E-03	-3.88E-02	-3.12E-01	-8.56E-03	-1.22E+00	-6.56E-01	-3.80E+00	-2.75E-14	-3.84E-02	-1.62E-02	-5.60E-03	-8.10E-01	-7.51E-01	-2.90E-02	-5.12E-18	-1.20E-16	-3.98E-07	2.51E-08	-1.15E-02
Max	3.62E+00	2.21E-01	1.45E-02	2.21E-01	1.09E+00	1.52E-01	2.79E+00	2.33E+00	-8.44E-02	7.56E-14	1.83E-01	1.57E-01	3.23E-02	-3.29E-04	6.20E+00	3.19E-01	1.88E-17	9.75E-15	1.14E-05	2.41E-05	-3.99E-06
Normalized peak height	2.60E-02	3.03E-02	1.01E-01	1.20E-01	1.08E-01	3.09E-02	8.11E-02	7.75E-02	7.60E-03	7.07E-01	1.02E-02	1.96E-02	1.81E-02	1.23E-02	1.67E-01	3.09E-02	3.93E-01	7.23E-01	5.50E-02	1.36E-02	1.33E-02
Peak sensitivity value	1.72E-02	4.63E-03	2.04E-05	3.11E-04	-8.31E-04	2.20E-04	3.11E-03	5.35E-04	-1.33E+00	-2.80E-17	4.44E-03	3.99E-04	1.38E-04	-3.97E-03	3.58E-03	4.32E-04	9.20E-22	3.66E-18	7.62E-09	1.82E-07	-1.25E-04
Shapiro-Wilk score	9.500E-01	8.698E-01	7.389E-01	7.168E-01	8.178E-01	8.752E-01	8.362E-01	8.031E-01	9.540E-01	2.031E-01	8.451E-01	7.958E-01	8.105E-01	8.860E-01	6.392E-01	7.586E-01	6.360E-01	3.132E-02	6.479E-01	8.930E-01	8.038E-01

Table S9: Concentration control coefficients for *Trypanosoma brucei* model with parameter variation of $\pm50\%$

Species	Metric	(glucosetransport)	(hexokinase)	(phosphoglucoseisomerase)	(phosphofructokinase)	(aldolase)	(triosephosphateisomerase)	(glyceraldehyde3phosphatedehydrogenase)	(glycerol3phosphatedehydrogenase)	(glycerol3phosphateoxidase)	(pyruvatetransport)
adpg	Local	7.11E+00	5.06E-02	-3.17E-03	-2.57E-02	-5.67E-01	-9.47E-02	-2.37E+00	-1.39E+00	1.77E-01	7.55E-16
	Min	-1.27E+02	-3.62E+00	-8.53E-02	-6.79E-01	-1.02E+01	-1.37E+00	-3.51E+01	-2.14E+01	-3.11E+01	-3.95E-13
	Max	8.39E+01	8.79E-01	9.71E-02	1.41E+00	1.72E+01	3.90E+00	9.62E+01	3.92E+01	4.44E+00	6.49E-13
	Normalized peak height	8.77E-02	8.69E-02	9.07E-02	1.13E-01	6.19E-02	1.23E-01	1.30E-01	1.21E-01	1.61E-01	6.55E-01
	Peak sensitivity value	-2.93E-01	3.99E-03	-2.24E-04	-2.13E-03	2.34E-02	-4.94E-03	2.19E-04	1.54E-02	1.18E-02	-5.13E-16
	Shapiro-Wilk score	8.977E-01	7.688E-01	6.590E-01	6.321E-01	7.661E-01	7.007E-01	7.762E-01	7.561E-01	3.794E-01	9.950E-02
adpc	Local	-5.14E-01	-3.66E-03	-1.09E-04	-7.90E-04	-1.10E-02	-3.67E-03	-6.52E-02	-3.83E-02	-3.37E-02	0.00E+00
	Min	-8.74E-01	-4.68E-02	-2.06E-02	-5.78E-01	-2.78E-01	-5.25E-02	-7.68E-01	-6.73E-01	-6.08E-01	-1.05E-11
	Max	4.13E-01	7.15E-01	1.26E-04	5.83E-04	5.37E-03	3.85E-03	-1.52E-06	-4.80E-07	4.37E-02	1.51E-11
	Normalized peak height	1.14E-01	1.55E-01	2.97E-01	4.92E-01	1.45E-01	8.37E-02	1.40E-01	1.73E-01	1.01E-01	9.78E-01
	Peak sensitivity value	2.05E-04	-1.53E-03	-9.21E-06	-2.84E-04	1.36E-04	-6.58E-05	-3.85E-04	-3.37E-04	-3.39E-04	-2.47E-15
	Shapiro-Wilk score	8.528E-01	3.836E-01	5.385E-01	3.378E-01	7.461E-01	7.745E-01	7.939E-01	7.625E-01	5.204E-01	1.931E-02
dihydroxyacetonephosphate	Local	-1.12E-01	-8.00E-04	1.87E-03	1.47E-02	2.89E-01	-7.31E-02	-6.54E-02	-3.84E-02	6.36E-02	-2.29E-17
	Min	-1.45E+01	-9.26E+00	-2.24E-03	5.35E-04	6.39E-03	-2.86E-01	-1.46E+00	-1.62E+00	-2.19E-01	-6.30E-15
	Max	7.42E+00	2.25E-01	3.87E-02	7.22E+00	1.30E+00	5.59E-02	2.65E+00	3.27E-01	1.47E+01	9.44E-15
	Normalized peak height	1.86E-01	6.13E-01	1.89E-02	2.79E-01	5.53E-03	7.87E-03	1.96E-01	1.45E-01	1.71E-01	6.89E-01
	Peak sensitivity value	9.55E-03	2.12E-03	6.92E-04	1.14E-02	1.97E-01	-4.45E-02	-3.54E-03	-3.30E-03	1.25E-02	5.88E-18
	Shapiro-Wilk score	6.191E-01	1.564E-01	7.127E-01	2.919E-01	9.256E-01	9.142E-01	7.626E-01	6.658E-01	2.494E-01	2.006E-01
glyceraldehyde3phosphate	Local	-1.73E-01	-1.23E-03	1.89E-03	1.49E-02	2.93E-01	6.17E-02	-9.23E-02	-5.41E-02	5.22E-02	-3.55E-17
	Min	-1.87E+01	-8.48E+00	-2.34E-04	4.32E-04	5.90E-03	1.10E-02	-1.36E+00	-1.50E+00	-2.36E-01	-7.27E-15
	Max	8.63E+00	5.68E-01	8.58E-02	6.74E+00	1.35E+00	2.01E-01	2.25E+00	8.60E-01	1.87E+01	8.54E-15
	Normalized peak height	2.23E-01	5.32E-01	4.01E-02	2.73E-01	5.49E-03	5.71E-03	1.64E-01	1.48E-01	2.48E-01	6.74E-01
	Peak sensitivity value	-5.83E-03	2.71E-03	6.69E-04	1.05E-02	1.90E-01	3.44E-02	-5.55E-03	-1.83E-03	1.97E-02	-5.35E-18
	Shapiro-Wilk score	6.665E-01	1.419E-01	7.092E-01	2.497E-01	9.225E-01	9.185E-01	7.975E-01	7.101E-01	1.992E-01	2.701E-01
2phosphoglycerate	Local	9.33E-01	6.64E-03	1.97E-04	1.43E-03	1.99E-02	6.66E-03	1.18E-01	6.94E-02	6.11E-02	-8.41E-17
	Min	-6.36E+00	-1.07E+00	-2.93E-04	-4.16E-04	-8.80E-03	-4.74E-03	3.95E-06	1.80E-06	-1.90E-01	-3.08E-12
	Max	3.41E+00	1.52E-01	2.16E-02	7.67E-01	7.42E-01	1.39E-01	3.01E+00	1.70E+00	1.95E+00	2.09E-12
	Normalized peak height	2.02E-01	1.29E-01	1.47E-01	3.20E-01	1.85E-01	1.25E-01	1.96E-01	1.94E-01	1.27E-01	9.53E-01
	Peak sensitivity value	3.39E-03	2.12E-03	2.39E-05	-3.24E-05	-1.72E-04	5.75E-05	1.51E-03	8.52E-04	1.38E-03	-2.14E-15
	Shapiro-Wilk score	8.638E-01	4.336E-01	5.302E-01	3.656E-01	7.287E-01	7.593E-01	7.948E-01	7.525E-01	4.950E-01	1.766E-02
glucose	Local	3.20E+00	-1.04E+00	-1.17E-02	-7.39E-02	-1.69E-01	-2.68E-02	-6.92E-01	-4.06E-01	5.93E-02	2.20E-16
	Min	-5.19E-01	-1.39E+00	-2.72E-01	-2.13E+00	-1.90E+01	-2.13E+00	-8.05E+01	-9.22E+01	-2.87E+01	-9.75E-13
	Max	1.90E+02	8.78E+00	1.21E-02	8.09E-02	-9.07E-05	2.91E-01	1.89E-02	2.22E-02	3.23E+01	8.95E-13
	Normalized peak height	1.58E-01	1.50E-01	4.03E-02	2.98E-02	2.55E-01	1.58E-01	3.22E-01	3.88E-01	3.57E-01	9.23E-01
	Peak sensitivity value	1.10E+00	-1.03E+00	-2.74E-04	-2.00E-03	-9.58E-03	9.79E-04	-2.14E-02	-2.40E-02	2.22E-02	-5.96E-17
	Shapiro-Wilk score	6.748E-01	7.565E-01	6.830E-01	7.006E-01	5.709E-01	6.061E-01	5.891E-01	5.191E-01	6.831E-01	8.074E-02
fructose6phosphate	Local	1.54E+00	1.10E-02	4.77E-03	-1.07E+00	-1.09E-01	6.21E-03	-1.92E-01	-1.13E-01	1.40E-01	4.79E-17
	Min	-2.54E+01	-1.42E-01	-3.81E-02	-2.30E+00	-9.84E+00	-5.93E-01	-2.66E+01	-2.30E+01	-5.99E-01	-1.76E-13
	Max	7.22E+01	3.14E+00	1.22E-01	-5.92E-01	1.91E-01	9.91E-02	6.24E-01	4.75E-01	2.69E+01	1.60E-13
	Normalized peak height	1.52E-01	1.97E-01	2.32E-02	1.82E-02	1.03E-01	5.82E-02	3.53E-01	3.86E-01	1.94E-01	9.18E-01
	Peak sensitivity value	1.10E+00	3.51E-03	1.67E-03	-1.04E+00	-4.49E-02	5.30E-03	-1.51E-02	-7.21E-03	2.01E-02	2.42E-17
	Shapiro-Wilk score	6.746E-01	7.409E-01	7.025E-01	8.408E-01	5.502E-01	6.442E-01	5.194E-01	4.944E-01	3.525E-01	5.613E-02
3phosphoglycerate	Local	3.53E+00	2.51E-02	7.47E-04	5.43E-03	7.54E-02	2.52E-02	4.48E-01	2.63E-01	2.31E-01	2.58E-22
	Min	-1.09E+01	-3.97E+00	-1.21E-03	-4.83E-03	-1.16E-01	-2.50E-02	3.38E-06	1.82E-06	-6.87E-01	-2.01E-12
	Max	8.79E+01	1.09E+00	1.70E-01	3.09E+00	7.87E+00	8.52E-01	1.91E+01	1.73E+01	1.85E+01	4.97E-13
	Normalized peak height	1.68E-01	1.83E-01	2.71E-01	2.82E-01	2.67E-01	1.23E-01	2.26E-01	2.49E-01	1.72E-01	8.80E-01
	Peak sensitivity value	-4.29E-02	6.69E-03	7.35E-05	-1.79E-04	-6.46E-05	-3.86E-05	9.53E-03	8.65E-03	1.36E-02	-9.93E-16
	Shapiro-Wilk score	5.867E-01	6.420E-01	5.424E-01	4.357E-01	7.110E-01	7.550E-01	7.702E-01	7.153E-01	4.770E-01	2.367E-02
glycerol	Local	9.22E+00	6.57E-02	-2.07E-03	-1.73E-02	-4.20E-01	-1.47E-01	-2.56E+00	-1.50E+00	-1.41E+00	1.05E-15
	Min	-2.84E+01	-6.51E+00	-1.09E-01	-6.76E-01	-8.14E+00	-2.07E+00	-3.35E+01	-2.27E+01	-4.22E+01	-2.31E-13
	Max	1.19E+02	1.07E+00	7.17E-02	4.90E+00	6.38E+00	4.03E-01	1.77E+01	6.99E+00	-2.27E-02	3.32E-13
	Normalized peak height	1.52E-01	1.07E-01	7.82E-02	2.09E-01	2.45E-02	3.02E-02	7.32E-02	9.53E-02	5.39E-02	7.86E-01
	Peak sensitivity value	-1.08E-01	1.04E-02	2.79E-04	2.09E-03	-1.99E-03	-3.05E-02	2.77E-02	4.45E-03	-1.27E+00	8.92E-17
	Shapiro-Wilk score	8.248E-01	8.351E-01	7.131E-01	4.140E-01	8.094E-01	6.950E-01	7.180E-01	6.881E-01	5.202E-01	1.012E-01
pyruvate	Local	5.54E+00	3.94E-02	1.17E-03	8.51E-03	1.18E-01	3.96E-02	7.02E-01	4.12E-01	3.62E-01	-7.85E+00
	Min	-4.20E+01	-3.41E+01	-1.63E-03	-5.86E-03	-2.41E+00	-7.69E-02	7.28E-06	9.11E-07	-5.34E-01	-1.16E+04
	Max	4.51E+03	8.20E+01	4.13E+00	5.41E+01	1.05E+03	1.79E+02	2.68E+03	2.13E+03	1.01E+03	-1.24E+00
	Normalized peak height	8.64E-01	8.70E-01	7.96E-01	8.95E-01	9.37E-01	9.30E-01	9.21E-01	9.46E-01	9.06E-01	9.23E-01
	Peak sensitivity value	1.18E+00	-5.89E-03	4.30E-04	2.12E-02	2.08E-01	1.28E-02	1.34E+00	1.06E+00	-2.72E-02	-7.01E+00
	Shapiro-Wilk score	3.430E-02	4.427E-02	4.071E-02	3.417E-02	1.850E-02	1.991E-02	2.896E-02	2.087E-02	2.143E-02	3.759E-02

Species	Metric	(phosphoglyceratekinase)	(pyruvatekinase)	(atputilisation)	(glycerolkinase)	(phosphoglyceratemutase)	(enolase)	(adenylatekinasecytosol)	(adenylatekinaseglycosome)	(3phosphoglyceratetransport)	(gly3pdhapantiporter)	(glyceroltransport)
adpg	Local	-1.91E-01	-2.21E-01	-4.54E-02	-2.04E-01	-1.87E+00	-3.52E-01	3.47E-18	-1.01E-17	-5.47E-06	2.02E-06	-3.41E-03
	Min	-1.08E+00	-9.11E-01	-2.00E-01	-9.64E-01	-8.23E+01	-1.78E+00	-2.04E-16	-6.11E-13	-2.12E-04	-5.46E-04	-5.17E-02
	Max	3.03E+00	1.09E+00	3.44E-01	1.16E+01	7.45E+01	2.27E+00	5.81E-17	3.49E-15	1.66E-05	6.62E-05	5.49E-02
	Normalized peak height	1.08E-01	1.05E-01	1.33E-01	1.32E-01	2.53E-01	1.11E-01	5.18E-01	9.42E-01	2.83E-01	2.10E-01	1.10E-01
	Peak sensitivity value	2.77E-03	6.04E-04	3.17E-04	-7.77E-04	5.99E-02	3.48E-03	9.22E-20	1.06E-16	3.30E-08	-1.98E-07	3.79E-05
	Shapiro-Wilk score	8.128E-01	8.646E-01	8.548E-01	7.827E-01	6.106E-01	8.578E-01	5.574E-01	8.911E-03	6.095E-01	5.709E-01	7.170E-01
adpc	Local	-5.25E-03	-6.07E-03	7.27E-01	1.54E-02	-5.14E-02	-9.68E-03	9.75E-18	-4.52E-20	-1.50E-07	-3.85E-07	2.58E-04
	Min	-8.60E-02	-6.37E-02	1.64E-01	-2.62E-01	-8.57E-01	-1.33E-01	-3.25E-15	-6.45E-17	-2.49E-06	-4.79E-06	-3.48E-04
	Max	-7.25E-06	-2.24E-07	8.93E-01	7.72E-02	-6.38E-07	-1.66E-07	5.00E-16	6.29E-17	6.92E-12	9.66E-07	2.75E-03
	Normalized peak height	2.06E-02	6.76E-02	2.77E-03	7.59E-02	1.78E-01	1.09E-01	3.36E-01	5.59E-01	1.26E-01	4.09E-02	1.78E-01
	Peak sensitivity value	-7.38E-04	-3.21E-05	7.22E-01	8.85E-06	-4.29E-04	-6.66E-05	-3.36E-19	3.82E-21	-1.24E-09	-3.93E-09	1.08E-06
	Shapiro-Wilk score	6.789E-01	7.183E-01	9.805E-01	7.753E-01	6.078E-01	6.320E-01	3.513E-01	3.768E-01	6.466E-01	7.674E-01	6.092E-01
dihydroxyacetonephosphate	Local	-5.13E-03	-5.94E-03	-1.22E-03	-6.36E-03	-5.03E-02	-9.47E-03	9.33E-20	1.12E-18	-1.47E-07	7.28E-07	-1.06E-04
	Min	-4.23E-01	-2.22E-01	-3.94E-02	-1.76E-01	-1.23E+01	-3.95E-01	-1.34E-17	-8.10E-16	-1.97E-05	-5.09E-06	-3.92E-03
	Max	6.30E-02	2.48E-02	8.02E-03	6.89E-01	1.36E+00	3.26E-02	6.02E-18	3.01E-15	4.71E-07	1.65E-05	7.89E-03
	Normalized peak height	1.16E-01	1.39E-01	1.27E-01	1.45E-01	4.35E-01	2.15E-01	5.79E-01	3.42E-01	2.63E-01	2.24E-02	1.88E-01
	Peak sensitivity value	-8.08E-04	-2.73E-05	-1.10E-05	2.61E-04	1.58E-04	-1.06E-04	-2.78E-21	-1.82E-18	-1.48E-09	4.33E-07	-7.67E-06
	Shapiro-Wilk score	4.376E-01	4.518E-01	4.911E-01	8.177E-01	5.079E-01	4.354E-01	3.565E-01	1.685E-01	2.393E-01	7.642E-01	6.719E-01
glyceraldehyde3phosphate	Local	-7.29E-03	-8.44E-03	-1.73E-03	-4.30E-04	-7.14E-02	-1.34E-02	1.32E-19	1.13E-18	-2.09E-07	5.97E-07	-7.19E-06
	Min	-5.77E-01	-2.45E-01	-4.49E-02	-1.62E-01	-1.54E+01	-3.73E-01	-5.56E-18	-5.85E-16	-2.61E-05	-9.13E-06	-3.86E-03
	Max	6.25E-02	1.95E-02	6.80E-03	8.89E-01	5.46E-01	2.94E-02	4.65E-18	3.76E-15	4.65E-07	1.12E-05	9.58E-03
	Normalized peak height	1.21E-01	1.53E-01	1.32E-01	2.31E-01	4.57E-01	1.77E-01	4.62E-01	3.73E-01	2.42E-01	2.83E-02	2.19E-01
	Peak sensitivity value	-1.20E-03	-2.06E-04	-4.02E-05	2.85E-04	-2.41E-03	-1.19E-04	-1.99E-21	-3.36E-19	-3.44E-10	3.33E-07	2.90E-06
	Shapiro-Wilk score	4.741E-01	5.112E-01	5.390E-01	7.316E-01	5.480E-01	4.857E-01	4.385E-01	1.292E-01	2.574E-01	7.445E-01	6.416E-01
2phosphoglycerate	Local	9.52E-03	-4.61E-01	-9.48E-02	-2.80E-02	9.32E-02	-7.35E-01	7.25E-18	8.21E-20	2.73E-07	6.99E-07	-4.68E-04
	Min	1.06E-05	-1.38E+00	-3.27E-01	-2.01E-01	1.43E-06	-3.41E+00	-6.80E-16	-1.77E-16	-1.13E-12	-3.99E-06	-6.75E-03
	Max	2.20E-01	-2.60E-01	-4.61E-02	1.84E+00	2.61E+00	-2.21E-01	5.20E-16	2.90E-16	6.34E-06	1.07E-05	1.88E-03
	Normalized peak height	2.79E-02	4.26E-03	4.08E-03	1.69E-01	2.25E-01	7.85E-03	3.21E-01	6.17E-01	1.44E-01	4.41E-02	1.98E-01
	Peak sensitivity value	1.44E-03	-3.38E-01	-1.14E-01	-1.65E-03	1.31E-03	-5.60E-01	-2.74E-19	-1.16E-19	3.17E-09	1.29E-08	-1.65E-06
	Shapiro-Wilk score	6.450E-01	9.218E-01	9.609E-01	6.780E-01	6.182E-01	8.682E-01	7.480E-01	2.218E-01	6.401E-01	7.258E-01	5.916E-01
glucose	Local	-5.57E-02	-6.44E-02	-1.33E-02	-5.89E-02	-5.45E-01	-1.03E-01	1.01E-18	-2.56E-19	-1.60E-06	6.79E-07	-9.85E-04
	Min	-3.40E+00	-2.23E+00	-4.56E-01	-2.75E+01	-8.13E+01	-5.08E+00	-2.02E-15	-2.25E-15	-3.21E-05	-1.68E-05	-1.08E-01
	Max	-5.95E-05	-5.91E-06	-1.47E-06	5.40E-02	-1.41E-05	-6.96E-06	3.56E-17	7.40E-14	-2.62E-14	4.86E-04	5.57E-04
	Normalized peak height	7.76E-02	1.74E-01	1.47E-01	2.29E-01	4.59E-01	2.67E-01	8.93E-01	9.36E-01	1.23E-01	3.39E-01	3.26E-01
	Peak sensitivity value	-8.57E-03	-1.12E-03	-2.29E-04	1.26E-02	-4.07E-02	-2.55E-03	-3.28E-19	3.69E-18	-1.61E-08	3.52E-08	-3.91E-05
	Shapiro-Wilk score	5.214E-01	5.914E-01	6.027E-01	6.206E-01	4.533E-01	5.046E-01	1.419E-02	1.194E-02	5.943E-01	5.321E-01	5.285E-01
fructose6phosphate	Local	-1.51E-02	-1.75E-02	-3.59E-03	-6.96E-03	-1.48E-01	-2.78E-02	2.74E-19	2.79E-18	-4.33E-07	1.60E-06	-1.16E-04
	Min	-2.22E+00	-1.14E+00	-2.39E-01	-9.38E+00	-2.46E+01	-1.84E+00	-1.54E-17	-1.98E-15	-2.92E-05	-9.05E-06	-2.52E-02
	Max	5.83E-02	3.90E-02	1.16E-02	1.38E+00	8.91E-01	8.66E-02	1.29E-17	5.24E-14	1.30E-06	1.44E-04	9.84E-03
	Normalized peak height	1.52E-01	2.74E-01	2.49E-01	3.67E-01	3.02E-01	2.83E-01	5.43E-01	6.29E-01	1.98E-01	8.02E-02	2.96E-01
	Peak sensitivity value	-1.97E-03	-4.00E-04	-1.13E-04	8.51E-04	-1.41E-02	-1.03E-03	-3.39E-21	5.26E-18	5.91E-09	7.91E-07	1.55E-05
	Shapiro-Wilk score	4.090E-01	5.149E-01	5.156E-01	5.102E-01	3.798E-01	4.716E-01	3.901E-01	6.278E-02	4.135E-01	4.745E-01	5.852E-01
3phosphoglycerate	Local	3.60E-02	-4.03E-01	-8.28E-02	-1.06E-01	-3.41E+00	-6.42E-01	6.33E-18	3.11E-19	-9.98E-06	2.65E-06	-1.77E-03
	Min	1.22E-05	-1.50E+00	-3.47E-01	-2.36E+00	-9.02E+01	-3.40E+00	-3.07E-15	-5.32E-15	-8.86E-04	-1.35E-05	-6.35E-02
	Max	1.13E+00	-4.99E-05	-1.10E-05	2.94E+00	-3.81E-01	-6.07E-05	9.53E-16	1.83E-14	-1.03E-12	1.48E-04	9.91E-03
	Normalized peak height	5.28E-02	1.30E-02	1.24E-02	1.41E-01	4.26E-02	2.29E-02	4.89E-01	8.22E-01	1.80E-01	9.40E-02	2.88E-01
	Peak sensitivity value	2.84E-03	-7.98E-04	-1.84E-04	-4.38E-03	-1.05E+00	-1.76E-03	5.05E-19	-9.82E-18	-4.43E-07	2.02E-07	-3.08E-05
	Shapiro-Wilk score	5.928E-01	9.717E-01	9.699E-01	5.223E-01	6.373E-01	9.497E-01	2.767E-01	1.650E-02	6.434E-01	4.455E-01	3.677E-01
glycerol	Local	-2.06E-01	-2.39E-01	-4.91E-02	6.53E-01	-2.02E+00	-3.80E-01	3.75E-18	-1.82E-18	-5.91E-06	-1.61E-05	-9.89E-01
	Min	-1.07E+00	-9.52E-01	-2.08E-01	4.69E-03	-1.16E+02	-2.47E+00	-2.29E-16	-3.05E-14	-1.96E-04	-5.83E-04	-1.00E+00
	Max	4.43E-01	3.19E-01	7.32E-02	3.21E+00	1.40E+01	5.16E-01	8.10E-17	1.51E-15	5.26E-06	-7.71E-08	-8.54E-01
	Normalized peak height	4.59E-02	7.60E-02	7.26E-02	1.79E-02	2.57E-01	1.28E-01	5.02E-01	6.74E-01	1.97E-01	1.80E-02	2.95E-02
	Peak sensitivity value	1.60E-03	3.30E-04	7.98E-05	9.67E-01	-3.37E-02	7.84E-04	1.44E-19	-5.95E-18	1.24E-07	-5.03E-06	-9.99E-01
	Shapiro-Wilk score	7.893E-01	8.186E-01	8.165E-01	8.598E-01	5.393E-01	7.995E-01	5.242E-01	5.307E-02	5.955E-01	7.597E-01	7.255E-01
pyruvate	Local	5.65E-02	6.54E-02	1.35E-02	-1.66E-01	5.54E-01	1.04E-01	-1.03E-18	4.87E-19	1.62E-06	4.15E-06	-2.78E-03
	Min	2.51E-05	6.97E-07	1.54E-07	-1.48E+02	1.43E-06	7.73E-07	-1.40E-15	-4.46E-15	1.75E-13	-2.33E-05	-4.03E+00
	Max	1.80E+02	2.21E+02	3.49E+01	2.26E+01	7.84E+03	1.77E+02	3.27E-15	6.17E-15	1.16E-03	5.22E-03	5.51E-02
	Normalized peak height	9.48E-01	9.74E-01	9.64E-01	8.91E-01	9.75E-01	9.44E-01	8.35E-01	9.04E-01	8.64E-01	8.26E-01	9.19E-01
	Peak sensitivity value	9.00E-02	1.11E-01	1.75E-02	-3.01E-02	3.92E+00	8.86E-02	-2.22E-18	1.01E-18	5.79E-07	2.51E-07	3.14E-05
	Shapiro-Wilk score	1.930E-02	1.532E-02	1.794E-02	3.400E-02	1.435E-02	2.218E-02	9.315E-03	3.738E-02	3.851E-02	3.107E-02	1.873E-02

dihydroxyacetonephosphate cytosol																					
Local	-3.10E-01	-2.20E-03	-1.68E-04	-1.28E-03	-2.24E-02	-7.65E-03	-1.35E-01	-7.90E-02	6.75E-01	-6.46E-17	-1.08E-02	-1.25E-02	-2.58E-03	3.30E-02	-1.06E-01	-2.00E-02	1.97E-19	-9.46E-20	-3.10E-07	-8.20E-07	5.52E-04
Min	-4.75E+01	-8.92E-01	-2.85E-02	-4.20E-01	-3.26E+00	-4.02E-01	-6.33E+00	-5.71E+00	2.49E-02	-1.15E-14	-1.52E+00	-6.44E-01	-9.94E-02	-1.36E-03	-2.30E+01	-1.24E+00	-1.30E-17	-1.29E-15	-4.54E-05	-6.73E-05	-9.35E-08
Max	1.38E+01	8.21E-01	2.59E-02	1.15E-01	3.47E-01	1.00E-02	2.06E-01	1.68E-01	4.89E+01	2.92E-14	1.37E-02	5.21E-03	1.80E-03	2.38E+00	4.82E-01	1.46E-02	1.87E-17	4.34E-16	7.44E-08	2.12E-08	2.51E-02
Normalized peak height	3.53E-01	3.07E-01	2.91E-01	3.93E-01	3.46E-01	2.17E-01	2.82E-01	2.89E-01	1.39E-01	6.37E-01	3.83E-01	3.91E-01	2.12E-01	1.13E-01	4.82E-01	3.21E-01	6.67E-01	6.86E-01	3.94E-01	1.23E-01	2.31E-01
Peak sensitivity value	7.73E-03	-3.76E-04	-3.15E-05	3.21E-05	-8.51E-04	-1.02E-04	-6.74E-05	7.80E-04	1.47E-01	1.99E-17	-8.61E-04	-3.02E-04	-7.71E-05	-1.65E-04	1.36E-03	1.21E-04	-6.49E-22	-5.26E-20	6.27E-09	-1.25E-08	1.25E-05
Shapiro-Wilk score	4.822E-01	5.099E-01	3.326E-01	3.619E-01	3.678E-01	4.333E-01	4.811E-01	4.204E-01	4.483E-01	1.387E-01	3.444E-01	3.425E-01	3.614E-01	5.397E-01	2.490E-01	3.047E-01	2.558E-01	1.488E-01	2.390E-01	4.463E-01	4.368E-01
nadh																					
Local	1.19E+00	8.44E-03	1.92E-04	1.37E-03	1.64E-02	1.21E-02	1.67E-01	-1.51E+00	-3.09E-02	2.01E-16	1.34E-02	1.55E-02	3.19E-03	-3.88E-02	1.31E-01	2.47E-02	-2.44E-19	7.06E-20	3.85E-07	-3.54E-07	-6.48E-04
Min	-6.91E+00	-2.07E+01	-1.44E-02	-5.98E-02	-4.63E-01	2.25E-04	5.64E-05	-5.11E+00	-3.94E+01	-4.42E-14	5.16E-05	2.86E-07	3.69E-08	-1.93E+00	6.99E-07	1.78E-07	-1.13E-17	-1.04E-15	3.39E-13	-2.45E-05	-1.35E-02
Max	3.98E+01	4.59E-01	1.09E-01	1.66E+01	1.30E+00	2.49E-01	8.06E+00	-2.11E-01	2.34E-01	2.12E-14	6.07E-01	3.60E-01	7.82E-02	1.77E-01	1.22E+01	9.30E-01	9.84E-18	2.02E-15	2.56E-05	8.53E-06	1.15E-04
Normalized peak height	2.84E-01	8.19E-01	3.10E-01	7.34E-01	6.96E-02	3.56E-02	2.57E-01	1.22E-02	3.91E-01	7.30E-01	5.50E-02	1.21E-01	1.07E-01	1.27E-01	3.66E-01	2.05E-01	4.50E-01	8.17E-01	2.42E-01	2.33E-02	1.32E-01
Peak sensitivity value	-1.31E-02	3.73E-03	-4.91E-05	-1.53E-03	-4.43E-03	1.34E-03	4.09E-03	-1.26E+00	-2.34E-02	-1.37E-17	1.57E-03	1.80E-04	3.91E-05	-1.71E-03	6.08E-03	4.65E-04	5.68E-21	4.46E-19	1.28E-08	-2.02E-07	-1.56E-06
Shapiro-Wilk score	7.668E-01	3.324E-02	5.460E-01	3.301E-02	8.062E-01	7.833E-01	8.046E-01	9.037E-01	2.104E-01	2.811E-01	6.249E-01	6.004E-01	6.375E-01	5.486E-01	6.120E-01	5.395E-01	5.186E-01	5.342E-02	3.797E-01	8.468E-01	5.659E-01
glycerol3phosphate																					
Local	9.63E-01	6.85E-03	2.45E-03	1.91E-02	3.66E-01	-4.66E-02	4.02E-01	2.36E-01	-2.28E+00	2.02E-16	3.25E-02	3.76E-02	7.73E-03	-1.21E-01	3.18E-01	5.99E-02	-5.90E-19	1.45E-18	9.31E-07	-2.61E-05	-2.02E-03
Min	-1.23E+01	-4.85E+00	-2.21E-02	-9.74E-02	-8.35E-01	-2.12E-01	-5.14E+00	-2.18E+00	-2.59E+01	-3.64E-14	-7.68E-01	-4.48E-01	-7.92E-02	-3.00E+00	-1.24E+01	-3.40E-01	-1.69E-17	-7.02E-16	-1.70E-06	-4.74E-04	-2.99E-02
Max	2.25E+01	6.15E-01	1.07E-01	3.77E+00	4.70E+00	3.60E-01	7.79E+00	6.35E+00	1.39E+00	3.14E-14	7.63E-01	6.07E-01	1.14E-01	1.42E-01	2.29E+01	1.16E+00	2.19E-17	2.08E-14	6.53E-05	8.82E-06	2.39E-05
Normalized peak height	1.48E-01	2.20E-01	4.79E-02	1.41E-01	1.68E-02	1.15E-02	2.14E-01	2.14E-01	3.40E-02	6.99E-01	9.50E-02	1.39E-01	1.64E-01	4.84E-02	4.06E-01	2.11E-01	4.83E-01	6.11E-01	2.75E-01	1.08E-02	4.11E-02
Peak sensitivity value	8.06E-03	6.20E-03	7.53E-04	8.86E-03	1.97E-01	-3.35E-02	-2.45E-04	-1.45E-03	-1.41E+00	1.16E-17	1.29E-03	1.01E-03	8.53E-05	-4.28E-03	4.25E-03	4.39E-04	-1.26E-20	-2.39E-18	8.08E-09	-1.51E-05	-5.09E-05
Shapiro-Wilk score	8.764E-01	5.798E-01	6.470E-01	4.371E-01	7.856E-01	9.354E-01	7.453E-01	6.549E-01	6.413E-01	2.426E-01	6.050E-01	5.410E-01	5.785E-01	8.458E-01	5.202E-01	4.835E-01	4.529E-01	4.617E-02	3.991E-01	7.879E-01	6.740E-01
glucose6phosphate																					
Local	1.66E+00	1.18E-02	-1.68E-01	-1.04E+00	-1.04E-01	6.02E-03	-1.84E-01	-1.08E-01	1.35E-01	4.60E-17	-1.45E-02	-1.68E-02	-3.45E-03	-6.64E-03	-1.42E-01	-2.67E-02	2.64E-19	2.69E-18	-4.16E-07	1.54E-06	-1.11E-04
Min	-1.71E+01	-1.51E-01	-5.71E-01	-2.35E+00	-7.86E+00	-7.65E-01	-2.56E+01	-1.99E+01	-5.31E-01	-2.31E-13	-1.71E+00	-7.21E-01	-2.04E-01	-5.65E+00	-3.27E+01	-1.08E+00	-1.16E-17	-8.56E-16	-2.64E-05	-1.03E-05	-1.96E-02
Max	4.39E+01	3.23E+00	-2.55E-02	-5.77E-01	2.48E-01	1.67E-01	7.80E-01	6.04E-01	1.91E+01	3.70E-13	9.27E-02	5.31E-02	1.55E-02	1.20E+00	1.12E+00	1.17E-01	1.90E-17	1.77E-13	1.13E-06	1.95E-04	1.35E-02
Normalized peak height	1.02E-01	1.81E-01	5.21E-03	1.48E-02	8.96E-02	7.72E-02	2.15E-01	2.78E-01	1.36E-01	9.47E-01	1.26E-01	1.87E-01	1.81E-01	3.30E-01	4.87E-01	2.42E-01	5.48E-01	8.44E-01	2.39E-01	1.11E-01	3.16E-01
Peak sensitivity value	1.18E+00	2.68E-03	-1.24E-01	-1.00E+00	-3.94E-02	4.98E-03	2.03E-04	5.85E-04	2.90E-02	-9.80E-18	-2.12E-03	4.53E-06	3.31E-05	1.97E-03	-1.47E-02	-3.04E-04	-6.14E-21	-5.74E-17	-1.18E-08	8.74E-07	-7.67E-07
Shapiro-Wilk score	6.935E-01	7.367E-01	9.534E-01	8.578E-01	5.582E-01	6.235E-01	5.177E-01	4.851E-01	3.955E-01	4.406E-02	4.530E-01	5.464E-01	5.637E-01	5.372E-01	3.667E-01	4.953E-01	4.083E-01	7.167E-03	4.288E-01	4.752E-01	5.855E-01
3phosphoglycerate cytosol																					
Local	3.53E+00	2.51E-02	7.47E-04	5.43E-03	7.54E-02	2.52E-02	4.48E-01	2.63E-01	2.31E-01	0.00E+00	3.60E-02	-4.03E-01	-8.28E-02	-1.06E-01	-3.41E+00	-6.42E-01	6.33E-18	3.11E-19	1.03E-06	2.65E-06	-1.77E-03
Min	-2.74E+00	-3.06E+00	-4.25E-03	-4.41E-03	-3.21E-01	-1.93E-02	7.20E-06	2.00E-06	-2.49E-01	-2.09E-12	2.65E-05	-1.33E+00	-3.12E-01	-3.28E+00	-1.11E+02	-2.67E+00	-4.04E-16	-3.51E-15	2.59E-10	-5.08E-06	-6.20E-02
Max	1.10E+02	1.95E+00	1.09E-01	2.42E+00	7.75E+00	9.90E-01	2.06E+01	2.61E+01	2.56E+01	8.62E-12	1.11E+00	-6.73E-05	-9.09E-06	2.54E+00	-3.84E-01	-7.52E-05	3.37E-16	3.47E-15	1.31E-05	1.48E-04	5.69E-03
Normalized peak height	2.33E-01	1.87E-01	1.38E-01	1.91E-01	2.46E-01	1.81E-01	2.30E-01	2.75E-01	1.73E-01	9.42E-01	5.11E-02	1.13E-02	1.08E-02	1.35E-01	5.28E-02	1.87E-02	3.75E-01	7.47E-01	3.64E-02	8.51E-02	2.80E-01
Peak sensitivity value	2.14E-02	5.84E-03	9.76E-05	1.65E-03	-1.95E-03	3.66E-04	1.03E-02	1.30E-02	2.26E-02	4.82E-15	2.81E-03	-7.32E-04	-1.65E-04	-6.32E-03	-1.10E+00	-1.41E-03	1.44E-20	-1.69E-18	1.98E-08	2.12E-07	-3.24E-05
Shapiro-Wilk score	5.876E-01	6.580E-01	5.533E-01	4.688E-01	7.064E-01	7.465E-01	7.650E-01	7.165E-01	4.291E-01	5.346E-03	5.939E-01	9.720E-01	9.695E-01	5.143E-01	6.342E-01	9.527E-01	7.248E-01	1.016E-01	7.335E-01	4.570E-01	3.642E-01
phosphoenolpyruvate																					
Local	4.22E-01	3.01E-03	8.93E-05	6.49E-04	9.02E-03	3.02E-03	5.36E-02	3.14E-02	2.76E-02	-1.05E-16	4.31E-03	-4.91E-01	-1.01E-01	-1.27E-02	4.22E-02	7.95E-03	7.72E-18	3.72E-20	1.24E-07	3.16E-07	-2.12E-04
Min	-6.72E-01	-6.15E-01	-1.87E-04	-2.00E-04	-1.56E-02	-3.87E-03	3.21E-06	6.84E-07	-3.86E-02	-4.36E-12	7.60E-06	-1.47E+00	-3.08E-01	-8.56E-02	2.59E-07	1.24E-07	-9.50E-16	-1.56E-16	-1.67E-12	-6.35E-07	-3.01E-03
Max	1.55E+00	7.92E-02	2.73E-02	4.67E-01	3.77E-01	7.69E-02	1.30E+00	9.59E-01	7.46E-01	8.83E-13	9.56E-02	-2.85E-01	-4.55E-02	3.87E-01	1.55E+00	1.61E-01	5.63E-16	2.13E-16	4.45E-06	5.61E-06	5.66E-04
Normalized peak height	1.48E-01	1.47E-01	2.81E-01	3.83E-01	1.82E-01	1.41E-01	1.77E-01	2.00E-01	1.01E-01	9.81E-01	2.27E-02	4.24E-03	3.50E-03	1.19E-01	2.36E-01	1.17E-01	3.21E-01	6.64E-01	1.65E-01	3.89E-02	1.81E-01
Peak sensitivity value	8.14E-04	1.17E-03	1.88E-05	3.36E-05	-8.84E-05	4.63E-05	6.51E-04	4.80E-04	2.46E-04	5.70E-16	6.29E-04	-3.60E-01	-1.08E-01	-2.50E-04	7.74E-04	8.08E-05	7.45E-19	1.26E-19	2.23E-09	4.90E-09	-7.03E-07
Shapiro-Wilk score	8.672E-01	3.866E-01	5.041E-01	3.379E-01	7.187E-01	7.412E-01	7.762E-01	7.365E-01	4.952E-01	1.595E-02	6.698E-01	9.178E-01	9.612E-01	7.584E-01	6.020E-01	6.147E-01	6.785E-01	1.896E-01	5.767E-01	7.358E-01	5.891E-01
bisphosphoglycerate																					
Local	-5.89E+00	-4.19E-02	5.08E-03	4.06E-02	8.47E-01	1.55E-01	3.68E+00	2.16E+00	4.96E-03	-1.01E-15	-8.02E-02	-9.29E-02	-1.91E-02	1.64E-01	-7.86E-01	-1.48E-01	1.46E-18	3.25E-18	-2.30E-06	5.67E-08	2.74E-03
Min	-9.64E+01	-4.08E+00	-1.06E-02	-6.01E-02	-2.00E-02	-6.93E-02	2.08E-02	1.50E-02	-2.43E+00	-2.26E-13	-2.61E+00	-1.06E+00	-2.46E-01	-7.30E-01	-4.41E+01	-3.01E+00	-2.11E-16	-4.63E-15	-3.08E-04	-1.06E-04	-6.59E-03
Max	1.72E+01	5.69E-01	1.22E-01	3.64E+00	1.22E+01	1.72E+00	3.86E+01	3.39E+01	9.39E+01	1.95E-13	1.17E-03	4.00E-04	1.07E-04	5.88E+00	5.60E-01	6.07E-04	1.13E-16	4.06E-15	1.78E-10	8.32E-05	5.63E-02
Normalized peak height	2.19E-01	1.13E-01	3.68E-02	9.39E-02	2.18E-02	2.27E-02	2.13E-02	2.41E-02	4.25E-01	7.00E-01	6.89E-02	8.54E-02	7.27E-02	1.03E-01	2.01E-01	1.66E-01	5.18E-01	3.08E-01	3.86E-01	7.31E-02	2.04E-01
Peak sensitivity value	-3.95E-03	-5.06E-03	8.52E-04	8.42E-03	2.68E-01	4.64E-02	1.20E+00	7.44E-01	2.51E-02	8.16E-17	-2.75E-03	-1.31E-04	-1.56E-05	6.81E-03	1.87E-03	-8.98E-04	-1.19E-19	-2.66E-18	-1.54E-07	7.60E-07	-1.36E-05
Shapiro-Wilk score	7.158E-01	7.218E-01	6.064E-01	5.215E-01	7.221E-01	7.042E-01	7.215E-01	7.078E-01	2.313E-01	1.548E-01	7.731E-01	8.322E-01	8.378E-01	6.527E-01	7.248E-01	8.147E-01	6.023E-01	3.009E-01	6.060E-01	6.703E-01	5.081E-01
atpg																					
Local	-2.48E+00	-1.77E-02	1.11E-03	8.98E-03	1.98E-01	3.30E-02	8.27E-01	4.85E-01	-6.16E-02	-2.63E-16	6.66E-02	7.71E-02	1.58E-02	7.12E-02	6.52E-01	1.23E-01	-1.21E-18	-7.32E-18	1.91E-06	-7.06E-07	1.19E-03
Min	-1.70E+02	-6.72E+00	-8.67E-02	-4.79E-01	9.36E-06	-7.79E-01	-2.94E-02	-8.57E-03	-2.92E+01	-1.48E-12	8.56E-05	1.33E-05	2.26E-06	2.77E-07	3.03E-05	1.10E-05	-1.44E-16	-2.02E-13	1.01E-12	-5.20E-04	5.73E-09
Max	-3.58E-03	-4.67E-06	1.52E-01	2.09E+00	3.86E+01	2.17E+00	9.05E+01	5.57E+01	3.53E+01	1.52E-12	8.91E+00	4.28E+00	1.52E+00	2.63E+01	1.71E+02	4.31E+00	6.21E-17	2.47E-15	5.79E-05	1.95E-05	1.45E-01
Normalized peak height	2.07E-01	3.38E-01	3.25E-01	3.27E-01	3.29E-01	2.58E-01	3.16E-01	3.42E-01	3.03E-01	9.34E-01	1.86E-01	2.60E-01	3.24E-01	3.39E-01	5.40E-01	2.33E-01	5.25E-01	5.60E-01	1.53E-01	3.35E-01	3.62E-01
Peak sensitivity value	-8.88E-02	-3.36E-03	9.79E-05	5.82E-04	1.93E-02	1.46E-03	1.59E-02	1.93E-02	2.98E-02	-4.47E-16	1.35E-02	2.15E-03	7.63E-04	1.31E-02	8.57E-02	2.16E-03	8.11E-20	-9.05E-17	2.90E-08	-1.47E-07	7.25E-05
Shapiro-Wilk score	6.052E-01	7.390E-01	7.049E-01	6.806E-01	5.133E-01	5.782E-01	5.537E-01	5.142E-01	6.521E-01	6.763E-02	4.713E-01	5.397E-01	5.258E-01	6.044E-01	3.892E-01	4.936E-01	3.785E-01	3.118E-02	5.475E-01	4.923E-01	4.929E-01
ampc																					
Local	-1.22E+00	-8.69E-03	-2.58E-04	-1.87E-03	-2.61E-02	-8.72E-03	-1.55E-01	-9.08E-02	-7.98E-02	-6.07E-20	-1.24E-02	-1.44E-02	1.73E+00	3.66E-02	-1.22E-01	-2.30E-02	-3.42E-16	-1.07E-19	-3.57E-07	-9.14E-07	6.12E-04
Min	-1.84E+00	-1.28E-01	-2.19E-01	-1.09E+01	-7.88E-01	-2.33E-01	-5.57E+00	-1.32E+00	-1.40E+00	-2.19E-11	-2.27E-01	-1.30E-01	1.17E+00	-5.83E-01	-1.86E+00	-3.87E-01	-6.43E-15	-4.86E-16	-7.13E-06	-1.08E-05	-1.65E-03
Max	2.93E+00	1.43E+01	9.02E-04	5.13E-03	1.57E-02	9.34E-03	-4.20E-06	-1.62E-06	1.36E-01	3.07E-11	-1.78E-05	-8.29E-07	1.90E+00	1.70E-01	-2.05E-06	-5.56E-07	1.77E-14	9.87E-17	3.41E-11	6.14E-06	7.04E-03
Normalized peak height	1.33E-01	7.33E-01	5.05E-01	7.04E-01	1.67E-01	1.11E-01	2.21E-01	1.45E-01	7.44E-02	9.56E-01	2.04E-02	5.13E-02	2.75E-03	6.25E-02	1.55E-01	1.13E-01	3.12E-01	5.99E-01	1.28E-01	4.50E-02	1.66E-01
Peak sensitivity value	8.09E-04	-5.85E-03	-8.56E-05	-3.12E-04	4.38E-05	-2.47E-04	-2.79E-03	-6.61E-04	-3.48E-04	-1.99E-14	-1.49E-03	-6.60E-05	1.72E+00	6.29E-04	-9.34E-04	-1.94E-04	6.75E-18	1.91E-19	-3.53E-09	-5.92E-09	9.11E-07
Shapiro-Wilk score	8.157E-01	7.087E-02	4.123E-01	7.039E-02	7.569E-01	7.843E-01	7.907E-01	7.716E-01	5.311E-01	2.199E-02	6.918E-01	7.394E-01	9.809E-01	8.035E-01	6.032E-01	6.550E-01	7.993E-01	2.049E-01	6.410E-01	7.812E-01	6.161E-01
nad																					
Local	-2.14E-02	-1.52E-04	-3.47E-06	-2.46E-05	-2.95E-04	-2.18E-04	-3.01E-03	2.72E-02	5.57E-04	-3.62E-18	-2.42E-04	-2.80E-04	-5.76E-05	6.99E-04	-2.37E-03	-4.46E-04	4.40E-21	-1.27E-21	-6.93E-09	6.38E-09	1.17E-05
Min	-2.37E+00	-1.00E-02	-1.83E-03	-2.09E-02	-6.63E-02	-1.57E-02	-2.94E-01	1.85E-03	-1.33E-02	-6.19E-16	-3.41E-02	-2.64E-02	-4.58E-03	-2.41E-03	-6.01E-01	-5.50E-02	-8.20E-19	-3.26E-17	-1.81E-06	-7.21E-07	-4.47E-06
Max	3.19E-01	2.59E-02	1.23E-04	6.82E-04	1.56E-02	-9.24E-07	-2.16E-07	4.38E-01	2.50E+00	1.12E-15	-1.82E-07	-1.40E-09	-8.70E-10	6.52E-02	-5.44E-09	-1.83E-09	6.22E-19	7.44E-17	-3.13E-15	1.10E-06	6.99E-04
Normalized peak height	3.79E-01	2.59E-01	3.76E-01	4.28E-01	1.81E-01	1.60E-01	3.62E-01	1.90E-02	8.06E-01	7.84E-01	2.41E-01	3.55E-01	3.04E-01	1.99E-01	5.11E-01	4.35E-01	6.47E-01	7.10E-01	5.26E-01	1.17E-01	3.26E-01
Peak sensitivity value	3.45E-04	-2.10E-05	5.63E-07	2.69E-06	7.35E-05	-8.79E-06	-1.47E-04	9.49E-03	5.20E-04	-2.66E-19	-1.72E-05	-1.32E-05	-2.29E-06	5.64E-05	-3.00E-04	-2.75E-05	-2.00E-22	5.07E-20	-9.03E-10	2.01E-09	1.04E-07
Shapiro-Wilk score	4.843E-01	4.580E-01	4.231E-01	3.889E-01	5.897E-01	5.452E-01	6.267E-01	7.168E-01	1.147E-01	1.267E-01	4.124E-01	3.809E-01	4.169E-01	3.536E-01	4.353E-01	3.557E-01	2.983E-01	2.883E-02	2.033E-01	5.912E-01	3.527E-01
fructose16bisphosphate																					
Local	4.06E-01	2.89E-03	4.32E-03	3.39E-02	-1.00E-01	5.73E-03	-1.77E-01	-1.04E-01	1.29E-01	4.42E-17	-1.39E-02	-1.61E-02	-3.31E-03	-6.40E-03	-1.36E-01	-2.56E-02	2.53E-19	2.53E-18	-3.99E-07	1.48E-06	-1.07E-04
Min	-2.43E+01	-4.61E+00	-5.21E-03	1.41E-03	-1.97E+00	-1.65E-01	-3.01E+00	-4.11E+00	-6.50E-01	-2.31E-14	-1.61E+00	-5.95E-01	-1.21E-01	-3.30E-01	-1.48E+01	-1.17E+00	-1.29E-17	-1.66E-15	-4.81E-05	-1.37E-05	-6.46E-03
Max	8.93E+00	3.57E-01	9.79E-02	3.51E+00	5.75E-01	1.14E-01	1.99E+00	2.76E+00	2.61E+01	1.09E-14	1.32E-01	6.59E-02	1.86E-02	1.74E+00	2.60E+00	1.84E-01	8.65E-18	5.06E-15	1.29E-06	1.88E-05	1.50E-02
Normalized peak height	2.10E-01	3.76E-01	1.92E-02	6.59E-02	3.81E-02	3.22E-02	1.27E-01	2.44E-01	2.52E-01	7.62E-01	1.99E-01	2.56E-01	2.54E-01	2.71E-01	4.61E-01	3.86E-01	6.11E-01	3.22E-01	4.70E-01	2.65E-02	4.05E-01
Peak sensitivity value	-3.99E-04	1.90E-03	1.54E-03	1.72E-02	-3.39E-02	4.73E-03	-5.89E-03	-3.10E-03	3.27E-02	-1.28E-17	-9.23E-04	1.51E-04	2.41E-05	-2.77E-04	-1.44E-03	-8.62E-05	2.11E-21	-1.28E-18	-2.02E-08	6.08E-07	2.98E-06
Shapiro-Wilk score	6.024E-01	3.968E-01	7.146E-01	5.338E-01	6.087E-01	7.422E-01	6.073E-01	5.200E-01	2.241E-01	1.990E-01	3.792E-01	4.190E-01	4.569E-01	5.923E-01	3.433E-01	3.841E-01	3.485E-01	2.440E-01	2.754E-01	7.562E-01	5.482E-01
ampg																					
Local	1.67E+01	1.19E-01	-7.45E-03	-6.05E-02	-1.33E+00	-2.22E-01	-5.57E+00	-3.27E+00	4.15E-01	1.77E-15	-4.48E-01	-5.19E-01	-1.07E-01	-4.79E-01	-4.39E+00	-8.26E-01	8.15E-18	3.08E-16	-1.29E-05	4.75E-06	-8.01E-03
Min	1.21E-03	1.24E-03	-6.43E-01	-5.28E+00	-2.61E+01	-2.65E+00	-8.50E+01	-5.02E+01	-1.04E+02	-6.44E-13	-2.21E+00	-1.92E+00	-4.08E-01	-3.19E+00	-1.39E+02	-5.58E+00	-1.58E-15	-5.08E-10	-3.67E-04	-8.95E-05	-1.07E-01
Max	1.41E+02	1.50E+00	4.63E-02	2.38E-01	-4.17E-05	1.86E-01	5.27E-03	2.52E-03	9.27E+00	7.27E-13	-1.93E-06	-9.07E-07	-1.43E-07	-1.19E-04	-3.04E-06	-7.45E-07	1.25E-16	1.00E-10	-1.34E-14	3.17E-04	-4.20E-08
Normalized peak height	1.96E-01	1.50E-02	2.16E-01	2.13E-01	2.11E-01	1.52E-01	1.99E-01	2.24E-01	4.90E-01	8.35E-01	1.90E-01	2.47E-01	2.40E-01	6.82E-02	3.24E-01	3.02E-01	6.54E-01	9.25E-01	4.09E-01	1.79E-01	3.13E-01
Peak sensitivity value	7.17E-02	9.50E-03	4.26E-04	-1.76E-03	-1.31E-02	4.60E-04	-3.73E-02	-2.26E-02	3.35E-02	9.03E-17	-1.11E-03	-9.59E-04	-2.04E-04	-4.91E-03	-6.96E-02	-2.79E-03	-5.97E-19	7.72E-15	-1.84E-07	1.37E-07	-5.35E-05
Shapiro-Wilk score	7.624E-01	8.480E-01	4.956E-01	5.018E-01	5.741E-01	5.890E-01	5.900E-01	5.550E-01	3.110E-01	9.114E-02	7.705E-01	7.925E-01	7.928E-01	6.847E-01	4.090E-01	7.631E-01	3.358E-01	1.994E-02	6.069E-01	5.513E-01	5.415E-01
atpc																					
Local	1.92E-01	1.36E-03	4.05E-05	2.94E-04	4.09E-03	1.37E-03	2.43E-02	1.43E-02	1.25E-02	2.20E-21	1.95E-03	2.26E-03	-2.71E-01	-5.75E-03	1.91E-02	3.61E-03	9.62E-18	1.69E-20	5.61E-08	1.44E-07	-9.61E-05
Min	-5.07E-01	-3.96E+00	-2.02E-04	-7.44E-04	-2.34E-03	-6.72E-04	1.62E-06	7.29E-07	-8.45E-02	-7.63E-12	3.44E-06	2.31E-07	-9.14E-01	-5.73E-02	5.14E-07	2.45E-07	-4.26E-15	-2.52E-16	-1.00E-11	-6.40E-06	-1.84E-03
Max	7.64E-01	2.76E-02	2.42E-02	3.04E+00	1.76E-01	3.79E-02	1.69E+00	4.07E-01	2.80E-01	2.03E-12	6.44E-02	4.01E-02	-9.90E-02	1.32E-01	6.10E-01	6.74E-02	3.04E-16	1.45E-16	2.12E-06	2.12E-06	2.51E-04
Normalized peak height	1.52E-01	8.18E-01	3.80E-01	8.40E-01	1.27E-01	9.92E-02	2.57E-01	1.75E-01	1.01E-01	9.78E-01	2.80E-02	6.68E-02	3.08E-03	8.81E-02	2.00E-01	9.34E-02	3.76E-01	8.01E-01	1.54E-01	8.33E-02	1.98E-01
Peak sensitivity value	5.08E-04	1.72E-03	4.59E-06	7.75E-04	6.19E-05	4.16E-05	8.47E-04	2.04E-04	1.74E-04	-9.84E-16	2.93E-04	2.03E-05	-2.96E-01	-1.12E-04	3.05E-04	3.39E-05	7.82E-19	7.05E-20	1.05E-09	5.41E-09	-1.05E-06
Shapiro-Wilk score	8.489E-01	4.300E-02	4.172E-01	4.356E-02	7.196E-01	7.317E-01	7.457E-01	7.305E-01	5.467E-01	1.874E-02	6.495E-01	7.064E-01	9.808E-01	7.637E-01	5.910E-01	6.637E-01	3.214E-01	6.322E-02	5.767E-01	7.395E-01	5.641E-01

glycerol3phosphate cytosol							
Local	7.65E-01	5.45E-03	4.16E-04	3.17E-03	5.53E-02	1.89E-02	3.33E-01
Min	-4.00E+00	-1.27E+00	-1.85E-02	-1.27E-01	-8.05E-01	-2.78E-02	-2.93E+00
Max	5.69E+00	3.90E-01	2.87E-02	1.03E+00	1.44E+00	1.78E-01	3.60E+00
Normalized peak height	7.79E-02	1.04E-01	1.92E-01	2.30E-01	1.54E-01	5.61E-02	1.92E-01
Peak sensitivity value	7.21E-03	2.94E-03	1.16E-05	-2.83E-04	-1.19E-03	7.94E-05	2.75E-03
Shapiro-Wilk score	9.381E-01	8.037E-01	6.225E-01	4.144E-01	7.877E-01	8.327E-01	8.209E-01

glycerol3phosphate cytosol							
Local	1.95E-01	-1.67E+00	1.60E-16	2.68E-02	3.10E-02	6.37E-03	-8.17E-02
Min	-1.06E+00	-4.52E+00	-2.71E-14	-5.31E-02	-2.96E-02	-5.43E-03	-1.38E+00
Max	2.75E+00	9.76E-02	3.22E-14	2.38E-01	2.48E-01	5.13E-02	-4.65E-05
Normalized peak height	1.51E-01	9.58E-03	7.10E-01	1.98E-02	6.39E-02	5.80E-02	2.94E-02
Peak sensitivity value	2.64E-04	-1.26E+00	-3.57E-19	1.74E-03	2.90E-04	4.75E-05	-2.11E-03
Shapiro-Wilk score	7.695E-01	9.252E-01	2.510E-01	7.664E-01	6.917E-01	7.102E-01	8.550E-01

glycerol3phosphate cytosol							
Local	2.62E-01	4.94E-02	-4.87E-19	2.34E-19	7.68E-07	2.03E-06	-1.37E-03
Min	-3.21E+00	-5.07E-02	-7.10E-18	-1.54E-16	-6.15E-07	1.20E-08	-1.41E-02
Max	6.55E+00	8.71E-01	8.02E-18	5.36E-15	1.71E-05	3.58E-05	-7.13E-07
Normalized peak height	1.92E-01	1.64E-01	4.02E-01	6.37E-01	1.10E-01	2.38E-02	2.70E-02
Peak sensitivity value	6.99E-03	4.19E-04	-4.04E-21	-1.95E-18	-2.73E-09	1.37E-07	-5.01E-05
Shapiro-Wilk score	6.307E-01	6.188E-01	5.412E-01	8.630E-02	5.283E-01	8.711E-01	6.899E-01

Table S10: Concentration control coefficients for *Trypanosoma brucei* model with parameter variation of 0.1 – 10×

		(glucosetransport)	(hexokinase)	(phosphoglycerateisomerase)	(phophofructokinase)	(aldolase)	(triosephosphateisomerase)	(glyceraldehyde3phosphatedehydrogenase)	(glycerol3phosphatedehydrogenase)	(glycerol3phosphateoxidase)	(pyruvatetransport)	(phosphoglyceratekinase)
adpg	Local	7.11E+00	5.06E-02	-3.17E-03	-2.57E-02	-5.67E-01	-9.47E-02	-2.37E+00	-1.39E+00	1.77E-01	7.55E-16	-1.91E-01
	Min	-3.37E+02	-5.62E+03	-1.47E+02	-1.66E+02	-2.62E+02	-2.72E+01	-2.37E+02	-3.73E+02	-2.29E+02	-2.54E-12	-7.42E+00
	Max	INF	INF	INF	INF	INF	INF	INF	INF	INF	INF	INF
	Normalized peak height	7.89E-01	6.44E-01	9.75E-01	9.92E-01	9.26E-01	8.66E-01	5.26E-01	7.13E-01	7.36E-01	9.95E-01	6.37E-01
	Peak sensitivity value	-8.74E-01	-3.12E+00	7.57E-02	-9.60E-01	8.05E-02	-3.01E-03	1.07E-01	-8.79E-02	-9.19E-02	2.53E-16	1.25E-02
	Shapiro-Wilk score	1.270E-01	8.756E-03	9.479E-03	2.590E-03	6.883E-02	1.019E-01	2.363E-01	1.707E-01	1.383E-01	4.726E-03	6.133E-01
adpc	Local	-5.14E-01	-3.66E-03	-1.09E-04	-7.90E-04	-1.10E-02	-3.67E-03	-6.52E-02	-3.83E-02	-3.37E-02	0.00E+00	-5.25E-03
	Min	-1.85E+01	-7.87E+02	-4.25E+00	-7.40E+01	-3.34E+00	-9.25E-01	-1.76E+01	-3.54E+00	-1.39E+01	-2.72E-10	-6.58E+00
	Max	INF	INF	INF	INF	INF	INF	INF	INF	INF	INF	INF
	Normalized peak height	5.40E-01	9.40E-01	9.62E-01	9.69E-01	6.21E-01	5.37E-01	4.39E-01	5.13E-01	4.22E-01	1.00E+00	6.77E-01
	Peak sensitivity value	-1.90E-03	1.77E-01	-4.09E-04	9.36E-03	1.14E-03	-5.46E-04	-6.27E-03	5.59E-03	-9.57E-03	6.70E-14	-3.99E-03
	Shapiro-Wilk score	7.475E-01	2.468E-02	4.477E-02	1.982E-02	3.441E-01	3.015E-01	7.543E-01	5.956E-01	5.916E-01	3.824E-04	1.887E-01
dihydroxyacetonephosphate	Local	-1.12E-01	-8.00E-04	1.87E-03	1.47E-02	2.89E-01	-7.31E-02	-6.54E-02	-3.84E-02	6.36E-02	-2.29E-17	-5.13E-03
	Min	-1.75E+02	-9.37E+03	-1.36E+00	-8.20E+01	-9.55E-01	-4.12E+00	-5.15E+00	-6.15E+00	-1.51E+01	-5.23E-13	-1.56E+01
	Max	INF	INF	INF	INF	INF	INF	INF	INF	INF	INF	INF
	Normalized peak height	7.53E-01	9.77E-01	9.52E-01	9.87E-01	4.48E-01	3.82E-01	6.91E-01	6.09E-01	7.45E-01	9.98E-01	8.19E-01
	Peak sensitivity value	1.01E-02	-4.66E+00	9.88E-04	-1.66E+00	3.00E-02	-2.48E-03	-3.44E-02	-1.90E-02	8.42E-02	1.72E-16	1.92E-03
	Shapiro-Wilk score	1.963E-01	3.826E-03	4.514E-02	3.541E-03	4.026E-01	4.308E-01	3.573E-01	3.624E-01	1.981E-01	1.928E-03	1.387E-01
glyceraldehyde3phosphate	Local	-1.73E-01	-1.23E-03	1.89E-03	1.49E-02	2.93E-01	6.17E-02	-9.23E-02	-5.41E-02	5.22E-02	-3.55E-17	-7.29E-03
	Min	-2.48E+02	-1.16E+04	-4.79E-01	-1.73E+02	-1.66E+01	-7.99E-01	-2.85E+01	-8.17E+00	-8.03E+00	-5.31E-12	-2.71E+01
	Max	INF	INF	INF	INF	INF	INF	INF	INF	INF	INF	INF
	Normalized peak height	7.87E-01	9.44E-01	9.19E-01	9.90E-01	5.07E-01	2.79E-01	4.99E-01	7.32E-01	5.30E-01	9.33E-01	7.96E-01
	Peak sensitivity value	-8.55E-02	5.12E+00	-6.53E-04	-2.15E+00	-1.72E-03	9.86E-03	-1.04E-02	-4.44E-02	-1.02E-01	-2.75E-15	2.31E-02
	Shapiro-Wilk score	1.810E-01	2.612E-03	4.877E-02	2.439E-03	3.823E-01	3.450E-01	5.587E-01	3.084E-01	1.439E-01	2.753E-04	1.034E-01
2phosphoglycerate	Local	9.33E-01	6.64E-03	1.97E-04	1.43E-03	1.99E-02	6.66E-03	1.18E-01	6.94E-02	6.11E-02	-8.41E-17	9.52E-03
	Min	-1.98E+02	-1.87E+03	-1.24E+00	-3.04E+01	-4.37E+00	-2.71E+02	-8.61E+00	-1.84E+00	-7.67E+00	-3.89E-11	-7.99E-01
	Max	5.42E+03	1.30E+02	6.80E+01	1.79E+03	1.41E+03	1.27E+02	8.33E+02	4.83E+03	3.97E+02	4.56E-11	1.72E+02
	Normalized peak height	9.94E-01	7.59E-01	8.56E-01	9.69E-01	9.98E-01	9.91E-01	8.78E-01	9.98E-01	5.01E-01	9.99E-01	8.63E-01
	Peak sensitivity value	1.33E+00	9.49E-01	-3.26E-02	-3.15E-01	5.72E-01	-9.84E-03	2.35E-01	5.76E-01	-1.85E-01	-1.45E-15	-2.29E-02
	Shapiro-Wilk score	7.469E-03	2.099E-02	9.810E-03	1.268E-02	3.151E-03	6.068E-03	5.544E-02	1.754E-03	4.729E-02	1.174E-03	8.270E-02
glucose	Local	3.20E+00	-1.04E+00	-1.17E-02	-7.39E-02	-1.69E-01	-2.68E-02	-6.92E-01	-4.06E-01	5.93E-02	2.20E-16	-5.57E-02
	Min	-6.55E+02	-9.82E+01	-7.70E+01	-1.24E+02	-2.61E+02	-3.48E+01	-3.68E+02	-7.65E+02	-1.55E+02	-7.47E-12	-4.32E+01
	Max	1.36E+03	1.32E+02	5.03E+00	9.57E+01	3.13E+01	2.11E+01	5.85E+01	3.11E+02	5.29E+01	9.05E-13	2.05E+02
	Normalized peak height	7.14E-01	4.30E-01	9.76E-01	7.72E-01	9.37E-01	7.65E-01	6.78E-01	6.52E-01	7.34E-01	9.82E-01	7.68E-01
	Peak sensitivity value	1.39E+00	-1.91E-02	-1.33E-02	5.97E-02	-1.08E-01	1.80E-02	-9.33E-02	4.61E-01	1.64E-02	-3.39E-15	9.37E-02
	Shapiro-Wilk score	1.986E-01	5.303E-01	2.113E-02	1.083E-01	6.755E-02	8.550E-02	1.806E-01	7.351E-02	2.262E-01	5.072E-03	8.534E-02
fructose6phosphate	Local	1.54E+00	1.10E-02	4.77E-03	-1.07E+00	-1.09E-01	6.21E-03	-1.92E-01	-1.13E-01	1.40E-01	4.79E-17	-1.51E-02
	Min	-1.07E+02	-3.06E+01	-1.60E+01	-1.75E+02	-1.15E+02	-6.81E+01	-3.73E+02	-1.66E+02	-9.61E+01	-5.01E-13	-9.25E+01
	Max	7.12E+02	2.03E+02	1.85E+01	2.48E+01	8.72E+00	3.79E+00	4.53E+01	5.50E+01	5.90E+01	1.22E-12	4.00E+00
	Normalized peak height	5.82E-01	3.28E-01	9.56E-01	6.86E-01	8.97E-01	9.42E-01	8.14E-01	8.60E-01	7.66E-01	9.71E-01	9.26E-01
	Peak sensitivity value	2.88E-01	4.80E-02	1.25E-02	-1.03E+00	-2.95E-02	1.97E-02	-7.52E-02	-3.94E-02	1.82E-02	-6.81E-16	-8.32E-03
	Shapiro-Wilk score	1.583E-01	6.231E-01	6.763E-02	2.640E-01	6.367E-02	2.459E-02	1.000E-01	1.129E-01	1.857E-01	1.255E-02	6.470E-02
3phosphoglycerate	Local	3.53E+00	2.51E-02	7.47E-04	5.43E-03	7.54E-02	2.52E-02	4.48E-01	2.63E-01	2.31E-01	2.58E-22	3.60E-02
	Min	-7.82E+02	-2.78E+03	-1.15E+00	-2.65E+01	-9.29E+00	-2.89E+01	-4.98E+01	-7.78E+00	-1.17E+02	-6.51E-11	-5.58E+00
	Max	2.70E+03	7.53E+01	7.76E+01	2.71E+03	6.75E+02	1.05E+02	1.86E+03	4.66E+03	3.12E+03	5.57E-11	1.29E+02
	Normalized peak height	8.82E-01	9.74E-01	9.69E-01	9.65E-01	9.41E-01	9.43E-01	9.69E-01	9.86E-01	9.88E-01	9.99E-01	7.60E-01
	Peak sensitivity value	-5.53E-01	-4.87E-01	-5.58E-03	-4.63E-01	-5.07E-02	9.97E-03	8.55E-01	-7.77E-01	6.18E-01	4.06E-14	-4.21E-03
	Shapiro-Wilk score	4.281E-02	2.296E-02	1.864E-02	1.315E-02	2.520E-02	4.347E-02	4.101E-02	5.133E-03	1.086E-02	5.714E-04	2.352E-01
glycerol	Local	9.22E+00	6.57E-02	-2.07E-03	-1.73E-02	-4.20E-01	-1.47E-01	-2.56E+00	-1.50E+00	-1.41E+00	1.05E-15	-2.06E-01
	Min	-5.57E+02	-1.69E+03	-5.40E+01	-2.01E+02	-1.67E+02	-8.24E+01	-4.46E+02	-2.33E+02	-3.55E+02	-7.30E-12	-1.96E+01
	Max	INF	INF	INF	INF	INF	INF	INF	INF	INF	INF	INF
	Normalized peak height	4.74E-01	5.51E-01	9.78E-01	8.74E-01	7.76E-01	6.35E-01	5.50E-01	6.25E-01	4.56E-01	9.75E-01	6.39E-01
	Peak sensitivity value	4.88E-01	-1.07E+00	2.88E-02	-5.43E-01	-9.81E-03	3.91E-02	-9.37E-02	1.95E-02	-9.55E-01	-4.03E-15	1.67E-03
	Shapiro-Wilk score	3.346E-01	6.147E-02	3.234E-02	3.638E-02	1.924E-01	1.225E-01	3.121E-01	2.541E-01	2.890E-01	4.510E-03	4.851E-01
pyruvate	Local	5.54E+00	3.94E-02	1.17E-03	8.51E-03	1.18E-01	3.96E-02	7.02E-01	4.12E-01	3.62E-01	-7.85E+00	5.65E-02
	Min	-4.15E+02	-2.81E+03	-1.74E+00	-3.48E+02	-1.59E+02	-1.02E+01	-5.94E+00	-2.71E+00	-2.37E+01	-4.04E+03	-1.01E+00
	Max	INF	INF	INF	INF	INF	INF	INF	INF	INF	INF	INF
	Normalized peak height	6.49E-01	9.81E-01	9.93E-01	9.64E-01	9.90E-01	9.02E-01	7.39E-01	6.24E-01	9.51E-01	9.73E-01	9.56E-01
	Peak sensitivity value	-1.13E+00	-5.86E-01	7.52E-02	-5.19E-01	4.30E-01	-2.07E-01	-1.75E-01	4.58E-01	4.16E-01	-3.02E+00	6.47E-02
	Shapiro-Wilk score	2.510E-02	1.857E-02	4.530E-03	1.400E-02	8.078E-03	5.096E-03	2.693E-02	2.271E-02	2.072E-02	2.393E-02	2.441E-02

		(pyruvatekinase)	(atputilisation)	(glycerolkinase)	(phosphoglyceratemutase)	(enolase)	(adenylatekinasecytosol)	(adenylatekinaseglycosome)	(3phosphoglyceratetransport)	(gly3pdhapantiporter)	(glyceroltransport)
adpg	Local	-2.21E-01	-4.54E-02	-2.04E-01	-1.87E+00	-3.52E-01	3.47E-18	-1.01E-17	-5.47E-06	2.02E-06	-3.41E-03
	Min	-1.04E+04	-5.07E+02	-3.43E+01	-2.65E+02	-2.64E+02	-8.02E-07	-9.08E-12	-1.65E-02	-2.83E-03	-7.35E-01
	Max	INF	INF	INF	INF	INF	INF	INF	INF	INF	INF
	Normalized peak height	9.99E-01	9.99E-01	6.95E-01	8.03E-01	9.27E-01	1.00E+00	1.00E+00	8.40E-01	7.97E-01	8.75E-01
	Peak sensitivity value	8.70E-01	5.95E-01	2.15E-02	2.05E-01	-4.15E-02	2.35E-10	-1.49E-16	6.92E-06	-1.65E-06	1.09E-03
	Shapiro-Wilk score	5.691E-04	6.275E-04	3.459E-01	1.201E-01	6.715E-02	5.323E-05	5.865E-04	8.023E-02	5.962E-02	1.345E-01
adpc	Local	-6.07E-03	7.27E-01	1.54E-02	-5.14E-02	-9.68E-03	9.75E-18	-4.52E-20	-1.50E-07	-3.85E-07	2.58E-04
	Min	-8.80E-01	-9.50E+02	-2.65E+00	-2.51E+00	-1.09E+00	-3.94E-07	-3.07E-12	-1.79E-04	-8.26E-05	-3.84E-01
	Max	INF	INF	INF	INF	INF	INF	INF	INF	INF	INF
	Normalized peak height	9.85E-01	4.91E-01	6.62E-01	6.41E-01	6.87E-01	1.00E+00	1.00E+00	8.56E-01	6.89E-01	9.09E-01
	Peak sensitivity value	-4.04E-01	-2.67E-01	1.17E-03	1.16E-03	-2.18E-04	-3.43E-11	-4.48E-15	-1.06E-07	1.13E-07	-1.35E-04
	Shapiro-Wilk score	3.860E-03	9.671E-02	3.113E-01	3.902E-01	2.030E-01	8.792E-05	2.182E-04	1.024E-01	1.641E-01	1.101E-01
dihydroxyacetonephosphate	Local	-5.94E-03	-1.22E-03	-6.36E-03	-5.03E-02	-9.47E-03	9.33E-20	1.12E-18	-1.47E-07	7.28E-07	-1.06E-04
	Min	-1.99E+02	-3.60E+02	-1.86E+00	-7.06E+01	-5.03E+01	-7.55E-08	-3.32E-12	-1.10E-03	-5.16E-04	-1.33E-01
	Max	INF	INF	INF	INF	INF	INF	INF	INF	INF	INF
	Normalized peak height	9.91E-01	9.81E-01	7.32E-01	6.93E-01	9.54E-01	1.00E+00	1.00E+00	9.35E-01	8.50E-01	9.09E-01
	Peak sensitivity value	4.69E-02	1.31E-01	1.60E-02	6.34E-02	-1.55E-02	1.47E-11	5.49E-15	-6.57E-07	2.25E-07	3.17E-04
	Shapiro-Wilk score	6.903E-03	3.794E-03	2.088E-01	9.649E-02	5.334E-02	4.929E-05	1.400E-04	6.911E-02	1.351E-01	7.997E-02
glyceraldehyde3phosphate	Local	-8.44E-03	-1.73E-03	-4.30E-04	-7.14E-02	-1.34E-02	1.32E-19	1.13E-18	-2.09E-07	5.97E-07	-7.19E-06
	Min	-1.65E+02	-2.81E+03	-3.33E+00	-7.39E+01	-5.35E+01	-1.57E-05	-6.61E-12	-1.72E-03	-3.17E-04	-1.85E-01
	Max	INF	INF	INF	INF	INF	INF	INF	INF	INF	INF
	Normalized peak height	9.97E-01	9.99E-01	8.14E-01	8.89E-01	9.28E-01	1.00E+00	1.00E+00	9.38E-01	7.19E-01	8.75E-01
	Peak sensitivity value	5.21E-01	4.96E-01	3.31E-02	-9.40E-03	9.36E-03	-2.35E-09	2.74E-15	-2.99E-07	-8.22E-08	-5.01E-04
	Shapiro-Wilk score	5.099E-04	3.125E-04	1.858E-01	9.670E-02	4.656E-02	6.324E-05	1.809E-04	6.445E-02	1.329E-01	7.554E-02
2phosphoglycerate	Local	-4.61E-01	-9.48E-02	-2.80E-02	9.32E-02	-7.35E-01	7.25E-18	8.21E-20	2.73E-07	6.99E-07	-4.68E-04
	Min	-1.95E+04	-8.83E+02	-7.43E+01	-4.62E+00	-4.91E+02	-9.58E-04	-3.79E-07	-1.97E-05	-8.11E-05	-8.59E-01
	Max	4.92E+01	1.41E+04	2.77E+02	5.23E+02	9.34E+00	4.13E-02	2.52E-11	1.37E-03	2.46E-03	2.68E+00
	Normalized peak height	9.95E-01	9.93E-01	9.36E-01	8.58E-01	5.30E-01	1.00E+00	1.00E+00	9.34E-01	6.55E-01	9.61E-01
	Peak sensitivity value	2.53E-01	-6.04E+00	-5.45E-02	-1.29E-01	-4.08E-01	-7.13E-06	-1.64E-10	4.53E-07	-1.14E-06	-1.79E-04
	Shapiro-Wilk score	1.686E-03	1.301E-03	2.924E-02	3.777E-02	1.691E-01	9.662E-05	3.397E-05	6.471E-02	3.968E-02	3.880E-02
glucose	Local	-6.44E-02	-1.33E-02	-5.89E-02	-5.45E-01	-1.03E-01	1.01E-18	-2.56E-19	-1.60E-06	6.79E-07	-9.85E-04
	Min	-3.07E+02	-8.98E+01	-7.16E+02	-2.77E+02	-7.45E+02	-1.51E-09	-6.34E-12	-4.01E-03	-2.10E-03	-2.38E+00
	Max	1.94E+01	1.75E+02	4.28E+01	3.28E+01	1.29E+01	5.48E-10	5.11E-12	1.59E-03	1.23E-03	6.01E-01
	Normalized peak height	9.73E-01	9.68E-01	9.26E-01	7.38E-01	9.82E-01	1.00E+00	1.00E+00	9.22E-01	9.27E-01	9.08E-01
	Peak sensitivity value	-7.11E-02	-1.11E-01	-7.55E-02	1.23E-01	-3.11E-01	3.83E-13	-1.82E-15	3.73E-07	5.88E-07	-5.97E-04
	Shapiro-Wilk score	2.699E-02	3.254E-02	6.121E-02	9.289E-02	2.016E-02	1.358E-04	3.076E-04	5.361E-02	6.119E-02	8.567E-02
fructose6phosphate	Local	-1.75E-02	-3.59E-03	-6.96E-03	-1.48E-01	-2.78E-02	2.74E-19	2.79E-18	-4.33E-07	1.60E-06	-1.16E-04
	Min	-8.20E+02	-7.73E+01	-1.14E+02	-2.49E+02	-5.01E+02	-1.93E-07	-1.26E-11	-1.59E-03	-1.41E-03	-1.45E+01
	Max	7.74E+01	1.04E+02	1.33E+01	7.84E+00	3.50E+00	3.82E-08	2.04E-11	4.08E-04	6.58E-04	4.92E-01
	Normalized peak height	9.89E-01	9.71E-01	8.25E-01	9.15E-01	9.26E-01	1.00E+00	1.00E+00	9.34E-01	9.41E-01	9.80E-01
	Peak sensitivity value	-2.44E-01	-6.33E-02	1.95E-02	2.26E-03	2.26E-01	4.24E-11	-2.33E-15	-8.29E-07	-2.37E-08	4.87E-03
	Shapiro-Wilk score	6.437E-03	2.366E-02	1.508E-01	6.461E-02	9.975E-03	4.989E-05	1.764E-04	4.390E-02	4.551E-02	7.797E-03
3phosphoglycerate	Local	-4.03E-01	-8.28E-02	-1.06E-01	-3.41E+00	-6.42E-01	6.33E-18	3.11E-19	-9.98E-06	2.65E-06	-1.77E-03
	Min	-6.29E+03	-6.21E+02	-5.01E+01	-6.00E+02	-6.82E+02	-1.21E-02	-3.29E-11	-1.58E-01	-1.52E-04	-1.21E+00
	Max	7.56E+02	3.51E+03	4.28E+02	1.82E+02	1.89E+02	4.54E-03	5.37E-09	2.96E-05	3.22E-03	4.19E+00
	Normalized peak height	9.89E-01	9.93E-01	8.99E-01	5.00E-01	8.76E-01	1.00E+00	1.00E+00	8.30E-01	9.46E-01	9.57E-01
	Peak sensitivity value	-8.21E-01	-9.24E-02	-1.28E-01	-6.45E-01	-6.87E-02	7.74E-06	2.19E-12	-4.93E-05	1.15E-06	-4.41E-04
	Shapiro-Wilk score	4.144E-03	5.203E-03	5.062E-02	1.709E-01	8.962E-02	1.556E-04	3.564E-05	8.416E-02	5.295E-02	3.761E-02
glycerol	Local	-2.39E-01	-4.91E-02	6.53E-01	-2.02E+00	-3.80E-01	3.75E-18	-1.82E-18	-5.91E-06	-1.61E-05	-9.89E-01
	Min	-2.80E+02	-1.75E+03	-5.67E+00	-7.26E+02	-2.95E+02	-4.12E-09	-2.20E-11	-7.58E-03	-1.10E-01	-1.05E+00
	Max	INF	INF	INF	INF	INF	INF	INF	INF	INF	INF
	Normalized peak height	9.43E-01	8.96E-01	3.63E-01	7.51E-01	9.22E-01	1.00E+00	1.00E+00	8.67E-01	9.26E-01	4.71E-01
	Peak sensitivity value	-9.79E-01	8.64E-01	1.00E+00	4.00E-01	-6.84E-02	2.26E-10	-3.39E-15	-2.31E-06	-2.47E-05	-1.00E+00
	Shapiro-Wilk score	1.350E-02	7.875E-03	7.367E-01	1.383E-01	9.232E-02	3.892E-05	3.493E-04	9.553E-02	5.668E-02	2.817E-01
pyruvate	Local	6.54E-02	1.35E-02	-1.66E-01	5.54E-01	1.04E-01	-1.03E-18	4.87E-19	1.62E-06	4.15E-06	-2.78E-03
	Min	-3.43E-01	-3.42E+03	-1.11E+02	-6.74E+00	-2.28E+00	-8.45E-07	-7.65E-11	-3.18E-04	-9.66E-04	-3.12E+00
	Max	INF	INF	INF	INF	INF	INF	INF	INF	INF	INF
	Normalized peak height	9.97E-01	9.93E-01	9.27E-01	9.76E-01	9.81E-01	1.00E+00	1.00E+00	9.92E-01	9.04E-01	9.67E-01
	Peak sensitivity value	1.61E+00	1.34E+00	-1.07E-01	-7.51E-01	3.49E-01	-2.82E-10	-3.64E-14	6.24E-06	-6.56E-07	3.38E-03
	Shapiro-Wilk score	1.965E-03	1.387E-03	2.516E-02	4.976E-03	7.772E-03	8.756E-05	5.323E-05	8.470E-03	2.570E-02	1.707E-02

dihydroxyacetonephosphate cytosol

Local	-3.10E-01	-2.20E-03	-1.68E-04	-1.28E-03	-2.24E-02	-7.65E-03	-1.35E-01	-7.90E-02	6.75E-01	-6.46E-17	-1.08E-02	-1.25E-02	-2.58E-03	3.30E-02	-1.06E-01	-2.00E-02	1.97E-19	-9.46E-20	-3.10E-07	-8.20E-07	5.52E-04
Min	-2.81E+02	-3.95E+15	-4.93E+07	-2.21E+15	-1.97E+04	-5.14E+01	-2.47E+01	-2.31E+04	-8.18E+01	-7.64E-13	-2.59E+07	-1.59E+06	-1.59E+06	-6.47E+00	-7.15E+01	-1.86E+02	-8.92E-11	-1.30E+09	-1.68E-03	-2.37E+00	-1.05E-01
Max	1.04E+02	2.21E+15	7.06E+10	3.95E+15	1.53E+11	1.10E+00	2.71E+02	1.42E+01	2.82E+02	4.92E-13	3.53E+08	1.10E+01	1.41E+02	2.79E+01	2.73E+01	8.90E+00	1.59E+06	5.03E-13	2.79E-04	7.94E+00	8.80E-01
Normalized peak height	8.43E-01	1.00E+00	1.00E+00	1.00E+00	1.00E+00	7.96E-01	7.61E-01	1.00E+00	4.16E-01	9.90E-01	1.00E+00	1.00E+00	1.00E+00	7.03E-01	8.90E-01	9.55E-01	1.00E+00	1.00E+00	9.48E-01	1.00E+00	8.83E-01
Peak sensitivity value	-5.69E-03	1.83E+12	-1.40E+07	-1.89E+12	7.66E+07	1.91E-02	-8.28E-03	2.64E+00	2.49E-01	3.88E-16	1.97E+04	-7.82E+02	-6.51E+02	1.10E-02	-1.24E-02	3.44E-02	7.93E+02	-6.48E+05	-8.62E-07	-2.78E-03	1.84E-04
Shapiro-Wilk score	1.494E-01	6.294E-05	3.397E-05	6.294E-05	3.630E-05	8.560E-02	2.693E-01	1.234E-04	2.717E-01	3.657E-03	3.922E-05	6.413E-05	6.384E-05	2.978E-01	8.371E-02	2.514E-02	6.360E-05	3.493E-05	6.280E-02	1.048E-04	9.611E-02

nadh

Local	1.19E+00	8.44E-03	1.92E-04	1.37E-03	1.64E-02	1.21E-02	1.67E-01	-1.51E+00	-3.09E-02	2.01E-16	1.34E-02	1.55E-02	3.19E-03	-3.88E-02	1.31E-01	2.47E-02	-2.44E-19	7.06E-20	3.85E-07	-3.54E-07	-6.48E-04
Min	-1.04E+02	-1.33E+09	-4.91E+01	-1.47E+05	-2.07E+02	-5.29E-01	-1.17E+01	-8.50E+01	-5.45E+02	-1.23E-13	-1.35E+05	-3.14E+02	-4.71E+02	-9.84E+01	-4.53E+00	-7.92E+00	-2.71E+02	-2.71E+00	-1.35E-04	-5.99E-02	-1.06E+00
Max	2.00E+02	1.89E+05	8.38E+00	1.33E+09	1.83E+02	1.29E+01	1.13E+02	6.36E+01	4.11E+01	1.72E-13	6.26E+01	1.04E+03	2.71E+02	1.65E+01	2.25E+02	5.57E+01	3.14E+02	3.12E+02	2.59E-03	5.08E-04	2.82E-01
Normalized peak height	6.18E-01	1.00E+00	9.78E-01	1.00E+00	9.25E-01	4.93E-01	3.45E-01	2.92E-01	8.17E-01	8.66E-01	1.00E+00	9.81E-01	9.77E-01	7.82E-01	7.82E-01	8.53E-01	1.00E+00	1.00E+00	8.39E-01	9.96E-01	8.88E-01
Peak sensitivity value	3.82E-02	-4.78E+05	1.46E-02	5.19E+05	-8.50E-02	-4.27E-05	-1.83E-03	-9.94E-01	-2.22E-01	-1.11E-16	-4.78E+00	2.99E-01	-1.94E-01	-6.19E-03	-5.77E-02	1.36E-03	1.10E-01	-3.45E-02	-4.87E-07	-5.03E-06	-3.79E-04
Shapiro-Wilk score	3.948E-01	5.066E-05	2.929E-02	5.066E-05	5.864E-02	3.101E-01	7.654E-01	6.086E-01	1.510E-01	7.404E-02	7.033E-05	9.635E-03	8.351E-03	2.114E-01	1.901E-01	1.370E-01	6.968E-05	5.931E-05	8.542E-02	2.248E-03	1.237E-01

glycerol3phosphate

Local	9.63E-01	6.85E-03	2.45E-03	1.91E-02	3.66E-01	-4.66E-02	4.02E-01	2.36E-01	-2.28E+00	2.02E-16	3.25E-02	3.76E-02	7.73E-03	-1.21E-01	3.18E-01	5.99E-02	-5.90E-19	1.45E-18	9.31E-07	-2.61E-05	-2.02E-03
Min	-1.41E+02	-3.91E+03	-3.62E+00	-8.95E+01	-8.00E+00	-5.75E+00	-2.82E+01	-1.63E+01	-1.18E+02	-3.97E-13	-6.46E+00	-3.41E+01	-2.66E+03	-2.54E+01	-4.62E+01	-2.98E+01	-1.60E-06	-7.14E-12	-4.14E-04	-3.14E-01	-6.31E-01
Max	INF	INF	INF	INF	INF	INF	INF	INF	INF	INF	INF	INF	INF	INF	INF	INF	INF	INF	INF	INF	INF
Normalized peak height	5.38E-01	9.37E-01	6.56E-01	9.69E-01	7.11E-01	5.93E-01	3.21E-01	4.24E-01	3.57E-01	9.27E-01	5.59E-01	9.82E-01	9.90E-01	3.08E-01	7.80E-01	8.06E-01	1.00E+00	1.00E+00	8.85E-01	9.51E-01	5.70E-01
Peak sensitivity value	-3.55E-02	-1.74E+00	2.22E-02	2.22E-01	2.74E-02	-6.72E-03	-2.27E-02	3.09E-03	-1.11E+00	-3.70E-16	-1.27E-02	-3.79E-01	-1.08E+00	1.58E-02	-3.85E-02	-4.91E-02	-5.65E-10	-1.14E-15	1.11E-07	-1.47E-06	4.65E-04
Shapiro-Wilk score	5.033E-01	2.478E-02	5.141E-02	2.127E-02	3.289E-01	4.025E-01	6.642E-01	5.377E-01	5.118E-01	1.898E-02	1.851E-01	2.752E-03	1.462E-03	5.531E-01	2.644E-01	1.207E-01	9.573E-05	2.500E-04	8.738E-02	2.387E-02	1.799E-01

glucose6phosphate

Local	1.66E+00	1.18E-02	-1.68E-01	-1.04E+00	-1.04E-01	6.02E-03	-1.84E-01	-1.08E-01	1.35E-01	4.60E-17	-1.45E-02	-1.68E-02	-3.45E-03	-6.64E-03	-1.42E-01	-2.67E-02	2.64E-19	2.69E-18	-4.16E-07	1.54E-06	-1.11E-04
Min	-1.09E+02	-1.61E+02	-3.51E+01	-6.32E+01	-1.02E+02	-1.94E+01	-4.34E+02	-1.48E+02	-1.63E+02	-4.19E-13	-4.21E+01	-1.06E+02	-1.94E+02	-8.72E+01	-1.52E+02	-8.65E+01	-3.36E-07	-2.44E-12	-3.83E-03	-7.41E-04	-1.51E+00
Max	5.01E+02	7.23E+01	4.78E-01	1.52E+02	9.55E+00	4.95E+00	2.07E+01	1.05E+02	3.14E+01	8.87E-13	6.67E+00	2.18E+02	5.68E+01	1.94E+01	5.25E+00	5.23E+00	4.47E-08	4.78E-12	5.11E-04	7.08E-04	9.16E-01
Normalized peak height	4.63E-01	3.36E-01	2.26E-01	4.59E-01	8.66E-01	8.67E-01	7.97E-01	7.63E-01	8.20E-01	9.24E-01	7.63E-01	9.81E-01	9.69E-01	8.11E-01	8.97E-01	9.28E-01	1.00E+00	1.00E+00	9.52E-01	9.29E-01	8.98E-01
Peak sensitivity value	-2.24E-03	4.13E-02	-3.84E-02	-1.01E+00	8.06E-04	-3.81E-03	3.91E-02	8.00E-02	3.23E-02	-6.06E-16	1.87E-02	-1.00E-01	6.99E-02	-1.54E-02	-2.87E-03	-3.87E-02	-2.75E-11	3.44E-16	1.04E-06	1.64E-07	-9.95E-04
Shapiro-Wilk score	2.031E-01	6.335E-01	6.007E-01	4.221E-01	6.866E-02	6.571E-02	1.142E-01	9.522E-02	1.323E-01	1.145E-02	1.110E-01	1.913E-02	1.262E-02	1.708E-01	6.546E-02	4.014E-02	4.405E-05	3.988E-04	2.338E-02	7.616E-02	5.389E-02

3phosphoglycerate cytosol

Local	3.53E+00	2.51E-02	7.47E-04	5.43E-03	7.54E-02	2.52E-02	4.48E-01	2.63E-01	2.31E-01	0.00E+00	3.60E-02	-4.03E-01	-8.28E-02	-1.06E-01	-3.41E+00	-6.42E-01	6.33E-18	3.11E-19	1.03E-06	2.65E-06	-1.77E-03
Min	-1.25E+02	-6.19E+02	-1.23E+00	-3.70E+01	-5.20E+00	-5.34E+00	-1.79E+01	-1.67E+01	-2.28E+01	-2.63E-11	-6.23E+00	-1.95E+03	-1.48E+03	-2.86E+01	-6.67E+02	-6.78E+02	-1.69E-02	-1.17E-09	-6.37E-05	-5.88E-04	-1.41E+00
Max	6.78E+02	5.33E+01	4.59E+01	6.07E+02	2.92E+02	7.19E+01	4.69E+02	4.54E+02	3.02E+03	4.63E-11	2.98E+02	2.81E+01	1.62E+03	2.08E+02	2.67E+01	6.35E+00	1.80E-01	1.17E-08	1.27E-03	3.36E-03	2.74E+00
Normalized peak height	6.33E-01	8.40E-01	5.56E-01	9.24E-01	8.98E-01	9.28E-01	3.68E-01	7.43E-01	9.79E-01	9.99E-01	8.37E-01	9.75E-01	9.89E-01	8.77E-01	4.41E-01	8.70E-01	1.00E+00	1.00E+00	7.46E-01	8.13E-01	9.34E-01
Peak sensitivity value	-1.27E-01	-1.01E-01	-2.32E-02	5.29E-02	-1.57E-03	3.02E-02	-1.63E-01	3.66E-02	7.52E-02	1.39E-14	5.73E-03	-5.24E-01	6.92E-01	-1.73E-02	-6.37E-01	-1.46E-01	-5.03E-05	4.88E-12	-4.25E-07	-1.27E-06	7.71E-04
Shapiro-Wilk score	1.035E-01	7.580E-02	2.810E-02	4.393E-02	4.641E-02	5.961E-02	1.285E-01	1.470E-01	1.492E-02	7.483E-04	1.565E-01	1.705E-02	1.549E-02	7.831E-02	1.840E-01	1.089E-01	8.339E-05	4.101E-05	8.610E-02	4.134E-02	5.979E-02

phosphoenolpyruvate

Local	4.22E-01	3.01E-03	8.93E-05	6.49E-04	9.02E-03	3.02E-03	5.36E-02	3.14E-02	2.76E-02	-1.05E-16	4.31E-03	-4.91E-01	-1.01E-01	-1.27E-02	4.22E-02	7.95E-03	7.72E-18	3.72E-20	1.24E-07	3.16E-07	-2.12E-04
Min	-6.19E+02	-8.18E+02	-1.31E+00	-2.66E+01	-3.12E+00	-6.17E+00	-1.14E+01	-1.95E+01	-6.70E+00	-1.22E-10	-5.24E+00	-1.92E+03	-1.06E+03	-4.59E+01	-1.44E+00	-1.29E+00	-1.64E-02	-3.78E-08	-4.74E-05	-1.96E-04	-7.53E-01
Max	2.36E+03	5.71E+01	1.12E+02	7.76E+02	1.46E+02	2.22E+01	6.18E+02	3.44E+02	2.84E+02	1.20E-10	5.42E+01	1.53E+02	6.67E+02	6.38E+02	8.99E+02	1.49E+02	3.01E-02	3.02E-08	8.56E-04	3.45E-03	6.03E+00
Normalized peak height	9.93E-01	9.62E-01	9.90E-01	9.81E-01	5.91E-01	8.49E-01	9.13E-01	8.67E-01	9.12E-01	9.99E-01	8.94E-01	7.92E-01	5.57E-01	9.91E-01	9.34E-01	9.41E-01	1.00E+00	1.00E+00	8.98E-01	9.26E-01	8.31E-01
Peak sensitivity value	-4.37E-02	-2.48E-01	-6.65E-03	3.05E-01	-7.11E-02	-4.78E-03	2.19E-01	-2.10E-02	1.24E-01	3.45E-14	1.94E-02	8.10E-01	3.97E-01	2.18E-01	-9.01E-02	-1.01E-02	-8.75E-06	-1.50E-11	-2.12E-08	-1.25E-06	3.36E-03
Shapiro-Wilk score	1.284E-02	2.708E-02	5.224E-03	1.831E-02	3.598E-02	6.017E-02	4.839E-02	3.738E-02	2.918E-02	5.171E-04	1.446E-01	2.726E-02	3.384E-02	7.507E-03	1.470E-02	3.747E-02	1.526E-04	6.700E-05	6.065E-02	1.435E-02	1.015E-02

bisphosphoglycerate

Local	-5.89E+00	-4.19E-02	5.08E-03	4.06E-02	8.47E-01	1.55E-01	3.68E+00	2.16E+00	4.96E-03	-1.01E-15	-8.02E-02	-9.29E-02	-1.91E-02	1.64E-01	-7.86E-01	-1.48E-01	1.46E-18	3.25E-18	-2.30E-06	5.67E-08	2.74E-03
Min	-9.50E+02	-1.54E+03	-1.01E+01	-5.79E+01	-1.59E+01	-4.83E+01	-6.92E+01	-2.95E+01	-1.43E+01	-3.96E-12	-1.11E+02	-2.65E+03	-1.01E+02	-2.10E+01	-1.55E+02	-2.93E+02	-6.33E-08	-2.37E-12	-1.98E-02	-4.07E-04	-2.91E-01
Max	INF	INF	INF	INF	INF	INF	INF	INF	INF	INF	INF	INF	INF	INF	INF	INF	INF	INF	INF	INF	INF
Normalized peak height	7.45E-01	8.92E-01	9.88E-01	9.66E-01	8.27E-01	6.42E-01	3.57E-01	5.26E-01	7.25E-01	9.91E-01	4.69E-01	9.87E-01	9.89E-01	8.48E-01	6.05E-01	8.99E-01	1.00E+00	9.96E-01	8.55E-01	9.17E-01	8.50E-01
Peak sensitivity value	-6.12E-01	2.07E-01	7.78E-02	4.04E-01	-5.90E-02	6.56E-02	4.84E-01	1.52E-01	-5.10E-02	6.40E-15	-1.00E-01	-9.06E-01	-4.80E-02	-3.14E-01	-5.70E-02	-1.35E-01	2.99E-11	-1.34E-15	-7.40E-06	1.49E-06	5.72E-04
Shapiro-Wilk score	2.150E-01	4.716E-02	1.029E-02	2.703E-02	6.860E-02	1.191E-01	2.805E-01	2.070E-01	1.007E-01	2.342E-03	6.231E-01	7.702E-03	9.278E-03	4.225E-02	2.276E-01	1.064E-01	1.283E-04	1.669E-03	1.125E-01	6.681E-02	1.241E-01

atpg

Local	-2.48E+00	-1.77E-02	1.11E-03	8.98E-03	1.98E-01	3.30E-02	8.27E-01	4.85E-01	-6.16E-02	-2.63E-16	6.66E-02	7.71E-02	1.58E-02	7.12E-02	6.52E-01	1.23E-01	-1.21E-18	-7.32E-18	1.91E-06	-7.06E-07	1.19E-03
Min	-2.49E+03	-9.08E+03	-1.74E+01	-7.67E+02	-2.25E+01	-2.68E+01	-3.82E+02	-6.91E+01	-1.70E+02	-1.20E-11	-1.15E+01	-6.61E-01	-7.81E+02	-3.06E+01	-3.32E+00	-1.51E+00	-1.17E-07	-1.81E-12	-2.38E-03	-2.02E-03	-6.40E-01
Max	INF	INF	INF	INF	INF	INF	INF	INF	INF	INF	INF	INF	INF	INF	INF	INF	INF	INF	INF	INF	INF
Normalized peak height	8.52E-01	9.96E-01	9.71E-01	8.97E-01	8.37E-01	7.95E-01	7.50E-01	8.93E-01	6.83E-01	9.96E-01	7.67E-01	9.60E-01	9.82E-01	8.66E-01	7.97E-01	9.11E-01	1.00E+00	1.00E+00	9.44E-01	9.58E-01	8.34E-01
Peak sensitivity value	-3.01E-01	-3.67E+00	-1.52E-02	-4.71E+00	-3.57E-02	-1.26E-02	1.82E-01	-5.30E-01	1.40E-01	3.05E-15	6.11E-03	-2.70E-01	2.65E-01	-2.11E-02	-2.38E-01	1.98E-02	-1.51E-10	-2.09E-15	8.87E-07	-2.99E-06	-2.43E-03
Shapiro-Wilk score	1.245E-01	9.906E-03	2.012E-02	2.917E-03	6.621E-02	9.626E-02	1.530E-01	3.068E-02	2.308E-01	3.067E-03	1.749E-01	1.926E-02	1.561E-02	8.912E-02	7.300E-02	6.027E-02	5.966E-05	4.061E-04	4.287E-02	3.063E-02	8.309E-02

ampc

Local	-1.22E+00	-8.69E-03	-2.58E-04	-1.87E-03	-2.61E-02	-8.72E-03	-1.55E-01	-9.08E-02	-7.98E-02	-6.07E-20	-1.24E-02	-1.44E-02	1.73E+00	3.66E-02	-1.22E-01	-2.30E-02	-3.42E-16	-1.07E-19	-3.57E-07	-9.14E-07	6.12E-04
Min	-4.01E+00	-1.50E+01	-2.55E+01	-1.76E+02	-7.62E+00	-2.26E+00	-3.09E+01	-1.28E+01	-1.72E+01	-1.97E-10	-3.66E+01	-1.83E+00	-2.58E-01	-1.29E+01	-4.13E+00	-2.79E+00	-9.38E-11	-3.35E-12	-4.92E-04	-5.62E-04	-6.71E-01
Max	4.23E+01	1.79E+02	2.55E-01	1.48E+01	1.13E+00	6.34E-01	1.20E+00	1.48E+00	9.91E-01	1.16E-09	1.19E+00	1.42E-01	1.99E+00	1.08E+00	1.17E+00	9.55E-01	1.46E-10	2.49E-12	1.15E-05	2.50E-05	9.60E-02
Normalized peak height	5.30E-01	8.62E-01	8.93E-01	9.22E-01	6.06E-01	6.58E-01	4.54E-01	5.05E-01	5.54E-01	1.00E+00	8.31E-01	7.74E-01	3.41E-03	6.76E-01	6.84E-01	5.30E-01	1.00E+00	1.00E+00	7.58E-01	8.13E-01	8.55E-01
Peak sensitivity value	-2.56E-03	1.35E-02	1.09E-02	1.91E-03	1.53E-03	-1.42E-03	-5.77E-03	-9.61E-04	-3.33E-03	3.04E-13	6.88E-05	-4.11E-04	4.25E-02	-3.38E-03	-2.40E-03	1.64E-03	6.39E-14	-3.17E-16	1.51E-07	6.60E-08	-3.09E-04
Shapiro-Wilk score	6.015E-01	6.089E-02	3.223E-02	3.824E-02	2.674E-01	2.067E-01	5.870E-01	4.601E-01	4.474E-01	1.649E-04	1.443E-01	1.475E-01	9.094E-01	2.699E-01	3.049E-01	1.634E-01	2.502E-04	2.508E-04	8.202E-02	8.491E-02	9.708E-02

nad

Local	-2.14E-02	-1.52E-04	-3.47E-06	-2.46E-05	-2.95E-04	-2.18E-04	-3.01E-03	2.72E-02	5.57E-04	-3.62E-18	-2.42E-04	-2.80E-04	-5.76E-05	6.99E-04	-2.37E-03	-4.46E-04	4.40E-21	-1.27E-21	-6.93E-09	6.38E-09	1.17E-05
Min	-1.17E+02	-1.33E+01	-1.26E+00	-7.19E+07	-4.06E+00	-1.83E+00	-2.11E+00	-9.54E+00	-2.98E+00	-5.20E-14	-5.79E+00	-3.28E+01	-1.39E+01	-8.17E-01	-2.80E+01	-6.92E+00	-1.04E-01	-3.76E+02	-1.44E-03	-3.25E-05	-1.55E-02
Max	9.61E+00	7.19E+07	1.09E+01	8.00E+00	3.54E+01	4.51E-02	2.58E+00	4.80E+01	1.17E+02	2.60E-14	1.68E+01	3.75E-01	7.72E+00	2.76E+01	1.39E+00	2.36E-01	1.33E-12	9.41E-01	1.03E-05	1.25E-04	2.28E-01
Normalized peak height	9.43E-01	1.00E+00	9.96E-01	1.00E+00	9.86E-01	4.72E-01	6.15E-01	7.67E-01	8.87E-01	9.88E-01	9.06E-01	9.93E-01	9.77E-01	9.27E-01	9.73E-01	9.66E-01	1.00E+00	1.00E+00	9.89E-01	9.31E-01	9.61E-01
Peak sensitivity value	3.66E-02	3.59E+04	-3.05E-03	-3.59E+04	-1.15E-02	9.04E-04	-5.87E-04	-1.21E-02	-4.42E-02	-2.22E-17	6.29E-03	-6.15E-03	2.12E-03	-7.10E-03	-9.10E-03	-3.51E-03	-5.21E-05	-1.29E-03	-6.03E-07	5.25E-09	-1.18E-05
Shapiro-Wilk score	2.557E-02	5.537E-05	2.929E-03	5.537E-05	1.137E-02	8.032E-02	2.997E-01	1.451E-01	2.735E-02	6.690E-03	6.372E-02	5.284E-03	1.884E-02	3.334E-02	1.814E-02	2.152E-02	3.904E-05	4.637E-05	1.199E-02	4.566E-02	2.160E-02

fructose16bisphosphate

Local	4.06E-01	2.89E-03	4.32E-03	3.39E-02	-1.00E-01	5.73E-03	-1.77E-01	-1.04E-01	1.29E-01	4.42E-17	-1.39E-02	-1.61E-02	-3.31E-03	-6.40E-03	-1.36E-01	-2.56E-02	2.53E-19	2.53E-18	-3.99E-07	1.48E-06	-1.07E-04
Min	-5.01E+02	-7.48E+03	-2.69E+01	-2.64E+02	-5.28E+01	-2.39E+00	-1.67E+01	-2.52E+01	-1.19E+01	-5.11E-13	-5.76E+01	-2.64E+02	-3.03E+03	-4.33E+00	-2.63E+02	-6.75E+01	-2.07E-06	-1.64E-11	-4.60E-03	-1.38E-03	-2.54E-01
Max	INF	INF	INF	INF	INF	INF	INF	INF	INF	INF	INF	INF	INF	INF	INF	INF	INF	INF	INF	INF	INF
Normalized peak height	7.86E-01	9.47E-01	6.28E-01	9.84E-01	5.63E-01	8.08E-01	3.67E-01	7.09E-01	4.94E-01	9.95E-01	9.46E-01	9.53E-01	9.49E-01	7.01E-01	8.99E-01	9.10E-01	1.00E+00	1.00E+00	9.05E-01	9.49E-01	8.01E-01
Peak sensitivity value	-1.77E-01	2.11E+00	-5.27E-02	1.42E+00	2.59E-02	1.18E-03	7.69E-02	3.47E-02	-2.11E-01	-5.00E-16	-4.94E-02	1.63E+00	1.49E+00	-2.52E-02	7.90E-02	2.47E-02	-1.07E-10	-5.67E-15	1.55E-06	1.46E-06	-1.06E-03
Shapiro-Wilk score	1.908E-01	1.120E-02	3.964E-02	1.033E-02	2.303E-01	1.883E-01	4.403E-01	2.278E-01	2.008E-01	2.545E-03	7.100E-02	9.837E-04	5.807E-04	2.012E-01	7.966E-02	4.989E-02	9.769E-05	1.791E-04	4.828E-02	6.923E-02	7.342E-02

ampg

Local	1.67E+01	1.19E-01	-7.45E-03	-6.05E-02	-1.33E+00	-2.22E-01	-5.57E+00	-3.27E+00	4.15E-01	1.77E-15	-4.48E-01	-5.19E-01	-1.07E-01	-4.79E-01	-4.39E+00	-8.26E-01	8.15E-18	3.08E-16	-1.29E-05	4.75E-06	-8.01E-03
Min	-5.80E+01	-5.41E-01	-2.64E+02	-3.41E+02	-3.89E+02	-6.68E+01	-4.08E+02	-6.08E+02	-4.70E+02	-1.02E-11	-2.01E+01	-2.67E+03	-1.83E+03	-2.99E+01	-8.13E+02	-1.95E+02	-1.51E-10	-1.46E-06	-1.74E-02	-1.99E-03	-1.70E+00
Max	INF	INF	INF	INF	INF	INF	INF	INF	INF	INF	INF	INF	INF	INF	INF	INF	INF	INF	INF	INF	INF
Normalized peak height	6.51E-01	6.59E-01	9.94E-01	9.37E-01	9.52E-01	9.21E-01	5.69E-01	7.82E-01	8.62E-01	9.96E-01	6.94E-01	9.98E-01	9.98E-01	6.84E-01	9.01E-01	8.42E-01	1.00E+00	9.98E-01	8.01E-01	8.96E-01	8.59E-01
Peak sensitivity value	-1.37E+00	5.96E-02	-7.09E-02	1.15E-01	-1.11E-01	-3.48E-02	1.78E-01	1.21E-01	2.64E-02	1.32E-15	-1.19E-02	-1.24E+00	-9.03E-01	8.40E-03	-2.99E-02	1.37E-02	3.15E-14	-3.06E-09	5.63E-06	6.07E-07	-7.58E-04
Shapiro-Wilk score	1.411E-01	1.956E-01	5.763E-03	2.003E-02	3.770E-02	5.595E-02	1.442E-01	1.122E-01	9.411E-02	3.136E-03	5.275E-01	2.212E-03	4.183E-03	3.677E-01	4.728E-02	4.351E-02	1.507E-04	7.690E-04	9.206E-02	6.624E-02	1.348E-01

atpc

Local	1.92E-01	1.36E-03	4.05E-05	2.94E-04	4.09E-03	1.37E-03	2.43E-02	1.43E-02	1.25E-02	2.20E-21	1.95E-03	2.26E-03	-2.71E-01	-5.75E-03	1.91E-02	3.61E-03	9.62E-18	1.69E-20	5.61E-08	1.44E-07	-9.61E-05
Min	-1.34E+02	-4.68E+03	-8.15E-01	-4.68E+01	-1.80E+00	-2.09E+00	-4.35E+00	-2.51E+00	-4.22E+00	-6.44E-10	-2.79E+00	-5.70E-01	-3.35E+03	-2.55E+00	-3.98E+00	-5.53E-01	-1.66E-06	-2.53E-12	-3.77E-05	-1.28E-04	-1.96E-01
Max	INF	INF	INF	INF	INF	INF	INF	INF	INF	INF	INF	INF	INF	INF	INF	INF	INF	INF	INF	INF	INF
Normalized peak height	4.87E-01	9.88E-01	9.63E-01	9.25E-01	5.54E-01	6.19E-01	4.26E-01	3.38E-01	5.60E-01	1.00E+00	7.61E-01	9.95E-01	9.96E-01	5.69E-01	6.34E-01	6.58E-01	1.00E+00	1.00E+00	9.29E-01	5.11E-01	8.54E-01
Peak sensitivity value	5.74E-02	-1.85E+00	7.56E-03	-2.06E+00	1.93E-04	1.60E-04	-3.50E-02	2.60E-02	8.48E-03	1.69E-13	5.39E-03	1.11E+00	-1.68E+00	-1.16E-02	1.07E-03	-4.35E-04	3.23E-10	-7.89E-16	6.08E-07	4.40E-07	4.59E-05
Shapiro-Wilk score	6.428E-01	1.035E-02	4.369E-02	8.142E-03	2.936E-01	2.518E-01	6.341E-01	5.038E-01	5.081E-01	3.333E-04	1.753E-01	2.659E-03	2.346E-02	2.957E-01	3.448E-01	1.774E-01	5.817E-05	2.099E-04	8.422E-02	9.421E-02	1.274E-01

glycerol3phosphate cytosol

Local	7.65E-01	5.45E-03	4.16E-04	3.17E-03	5.53E-02	1.89E-02	3.33E-01
Min	-1.06E+02	-1.14E+03	-8.90E+00	-4.14E+02	-4.50E+00	-1.45E+00	-3.62E+02
Max	INF	INF	INF	INF	INF	INF	INF
Normalized peak height	6.76E-01	9.70E-01	9.60E-01	9.20E-01	5.72E-01	5.57E-01	6.03E-01
Peak sensitivity value	-9.32E-02	-2.92E-01	-2.48E-03	-5.95E-01	-7.44E-03	6.47E-04	5.05E-02
Shapiro-Wilk score	3.918E-01	2.876E-02	4.289E-02	2.058E-02	3.107E-01	2.868E-01	3.512E-01

Local	1.95E-01	-1.67E+00	1.60E-16	2.68E-02	3.10E-02	6.37E-03	-8.17E-02
Min	-1.15E+01	-1.65E+01	-2.15E-13	-1.31E+00	-8.53E+00	-8.11E+02	-1.45E+01
Max	INF	INF	INF	INF	INF	INF	INF
Normalized peak height	3.15E-01	1.24E-01	8.83E-01	5.10E-01	9.77E-01	9.89E-01	4.47E-01
Peak sensitivity value	1.59E-02	-1.02E+00	-3.25E-18	-4.02E-03	8.69E-02	2.64E-01	-5.24E-03
Shapiro-Wilk score	5.644E-01	8.380E-01	5.799E-02	2.229E-01	9.848E-03	5.515E-03	4.880E-01

Local	2.62E-01	4.94E-02	-4.87E-19	2.34E-19	7.68E-07	2.03E-06	-1.37E-03
Min	-3.08E+01	-7.86E+00	-1.96E-08	-3.72E-12	-2.77E-04	-9.80E-05	-5.69E-01
Max	INF	INF	INF	INF	INF	INF	INF
Normalized peak height	7.44E-01	8.38E-01	1.00E+00	1.00E+00	8.50E-01	6.29E-01	8.09E-01
Peak sensitivity value	-1.38E-02	7.07E-03	7.48E-11	3.18E-14	-1.84E-07	1.16E-07	-2.22E-04
Shapiro-Wilk score	3.448E-01	2.038E-01	9.423E-05	8.637E-05	8.513E-02	2.165E-01	1.421E-01

Table S11: Flux control coefficients for *Trypanosoma brucei* model with parameter variation of ±5%

	(glucosetransport)	(hexokinase)	(phosphoglycerateisomerase)	(phophofructokinase)	(aldolase)	(triosephosphateisomerase)	(glyceraldehyde3phosphatedehydrogenase)	(glycerol3phosphatedehydrogenase)	(glycerol3phosphateoxidase)	(pyruvatetransport)
(glucose transport)<br>Local<br>Min<br>Max<br>Normalized peak height<br>Peak sensitivity value<br>Shapiro-Wilk score	9.79E-01<br>9.22E-01<br>9.92E-01<br>4.71E-03<br>9.83E-01<br>9.394E-01	6.97E-03<br>5.01E-03<br>1.18E-02<br>3.49E-03<br>6.52E-03<br>9.749E-01	7.82E-05<br>4.22E-05<br>1.88E-04<br>3.84E-03<br>7.13E-05<br>9.616E-01	4.95E-04<br>2.64E-04<br>1.19E-03<br>3.91E-03<br>4.56E-04<br>9.587E-01	1.13E-03<br>2.00E-04<br>7.08E-03<br>5.94E-03<br>7.54E-04<br>9.089E-01	1.79E-04<br>3.56E-05<br>7.59E-04<br>4.30E-03<br>1.43E-04<br>9.359E-01	4.63E-03<br>8.08E-04<br>2.67E-02<br>5.24E-03<br>3.64E-03<br>9.127E-01	2.72E-03<br>4.93E-04<br>1.59E-02<br>5.36E-03<br>1.95E-03<br>9.161E-01	-3.97E-04<br>-4.95E-03<br>-1.82E-05<br>9.16E-03<br>-1.34E-04<br>8.405E-01	-1.47E-18<br>-3.38E-17<br>3.29E-17<br>1.43E-01<br>2.31E-20<br>9.477E-01
(hexokinase)<br>Local<br>Min<br>Max<br>Normalized peak height<br>Peak sensitivity value<br>Shapiro-Wilk score	9.79E-01<br>9.16E-01<br>9.91E-01<br>4.65E-03<br>9.83E-01<br>9.407E-01	6.97E-03<br>5.06E-03<br>1.24E-02<br>3.85E-03<br>6.81E-03<br>9.753E-01	7.82E-05<br>4.17E-05<br>2.03E-04<br>4.29E-03<br>6.87E-05<br>9.616E-01	4.95E-04<br>2.56E-04<br>1.35E-03<br>4.60E-03<br>4.39E-04<br>9.584E-01	1.13E-03<br>2.38E-04<br>7.60E-03<br>6.11E-03<br>7.28E-04<br>9.104E-01	1.79E-04<br>3.69E-05<br>8.86E-04<br>4.82E-03<br>1.33E-04<br>9.368E-01	4.63E-03<br>9.24E-04<br>2.96E-02<br>5.82E-03<br>2.95E-03<br>9.137E-01	2.72E-03<br>5.49E-04<br>1.65E-02<br>5.69E-03<br>1.88E-03<br>9.167E-01	-3.97E-04<br>-5.91E-03<br>-2.25E-05<br>1.09E-02<br>-1.37E-04<br>8.420E-01	-1.47E-18<br>-3.41E-17<br>4.20E-17<br>1.48E-01<br>3.25E-20<br>9.503E-01
(phosphoglycerate isomerase)<br>Local<br>Min<br>Max<br>Normalized peak height<br>Peak sensitivity value<br>Shapiro-Wilk score	9.79E-01<br>9.26E-01<br>9.91E-01<br>4.16E-03<br>9.83E-01<br>9.398E-01	6.97E-03<br>5.08E-03<br>1.18E-02<br>3.56E-03<br>6.55E-03<br>9.747E-01	7.82E-05<br>4.13E-05<br>1.95E-04<br>3.95E-03<br>6.69E-05<br>9.610E-01	4.95E-04<br>2.59E-04<br>1.28E-03<br>4.26E-03<br>4.23E-04<br>9.580E-01	1.13E-03<br>2.56E-04<br>6.51E-03<br>5.21E-03<br>6.85E-04<br>9.094E-01	1.79E-04<br>4.05E-05<br>8.11E-04<br>4.25E-03<br>1.25E-04<br>9.355E-01	4.63E-03<br>9.50E-04<br>2.65E-02<br>5.13E-03<br>3.08E-03<br>9.125E-01	2.72E-03<br>5.74E-04<br>1.50E-02<br>4.99E-03<br>2.00E-03<br>9.156E-01	-3.97E-04<br>-4.55E-03<br>-2.23E-05<br>8.46E-03<br>-1.65E-04<br>8.405E-01	-1.47E-18<br>-3.11E-17<br>3.43E-17<br>1.44E-01<br>-3.08E-20<br>9.508E-01
(phophofructokinase)<br>Local<br>Min<br>Max<br>Normalized peak height<br>Peak sensitivity value<br>Shapiro-Wilk score	9.79E-01<br>9.12E-01<br>9.91E-01<br>5.12E-03<br>9.84E-01<br>9.408E-01	6.97E-03<br>4.91E-03<br>1.23E-02<br>3.86E-03<br>6.82E-03<br>9.752E-01	7.82E-05<br>4.32E-05<br>2.11E-04<br>4.42E-03<br>6.63E-05<br>9.618E-01	4.95E-04<br>2.62E-04<br>1.40E-03<br>4.76E-03<br>4.34E-04<br>9.588E-01	1.13E-03<br>2.36E-04<br>7.83E-03<br>6.35E-03<br>7.86E-04<br>9.099E-01	1.79E-04<br>3.86E-05<br>9.63E-04<br>5.25E-03<br>1.32E-04<br>9.356E-01	4.63E-03<br>9.44E-04<br>3.19E-02<br>6.64E-03<br>3.00E-03<br>9.127E-01	2.72E-03<br>5.61E-04<br>1.79E-02<br>6.03E-03<br>1.91E-03<br>9.162E-01	-3.97E-04<br>-6.04E-03<br>-2.02E-05<br>1.12E-02<br>-1.38E-04<br>8.411E-01	-1.47E-18<br>-3.09E-17<br>3.18E-17<br>1.45E-01<br>1.08E-20<br>9.509E-01
(aldolase)<br>Local<br>Min<br>Max<br>Normalized peak height<br>Peak sensitivity value<br>Shapiro-Wilk score	9.79E-01<br>9.26E-01<br>9.91E-01<br>4.34E-03<br>9.83E-01<br>9.412E-01	6.97E-03<br>5.06E-03<br>1.20E-02<br>3.64E-03<br>6.56E-03<br>9.750E-01	7.82E-05<br>4.18E-05<br>1.92E-04<br>3.90E-03<br>7.05E-05<br>9.624E-01	4.95E-04<br>2.66E-04<br>1.28E-03<br>4.33E-03<br>4.49E-04<br>9.593E-01	1.13E-03<br>2.47E-04<br>6.30E-03<br>5.23E-03<br>7.70E-04<br>9.109E-01	1.79E-04<br>3.94E-05<br>8.22E-04<br>4.58E-03<br>1.38E-04<br>9.364E-01	4.63E-03<br>9.83E-04<br>2.52E-02<br>4.92E-03<br>2.91E-03<br>9.138E-01	2.72E-03<br>5.63E-04<br>1.36E-02<br>4.83E-03<br>1.77E-03<br>9.174E-01	-3.97E-04<br>-4.82E-03<br>-1.64E-05<br>8.92E-03<br>-1.49E-04<br>8.433E-01	1.48E-16<br>-8.78E-16<br>9.02E-16<br>8.54E-02<br>3.67E-20<br>9.782E-01
(triosephosphate isomerase)<br>Local<br>Min<br>Max<br>Normalized peak height<br>Peak sensitivity value<br>Shapiro-Wilk score	4.14E-01<br>-2.19E-01<br>8.44E-01<br>2.59E-03<br>3.83E-01<br>9.864E-01	2.95E-03<br>-2.46E-03<br>5.02E-03<br>3.59E-03<br>3.44E-03<br>9.684E-01	2.25E-04<br>7.22E-05<br>6.09E-04<br>3.09E-03<br>1.82E-04<br>9.692E-01	1.71E-03<br>5.00E-04<br>4.72E-03<br>3.16E-03<br>1.45E-03<br>9.694E-01	3.00E-02<br>4.78E-03<br>9.21E-02<br>3.23E-03<br>2.42E-02<br>9.685E-01	1.02E-02<br>2.76E-03<br>2.27E-02<br>2.76E-03<br>9.93E-03<br>9.857E-01	1.80E-01<br>3.83E-02<br>4.95E-01<br>3.16E-03<br>1.58E-01<br>9.733E-01	1.06E-01<br>2.27E-02<br>2.89E-01<br>3.03E-03<br>1.04E-01<br>9.759E-01	9.60E-02<br>3.53E-02<br>1.56E-01<br>2.56E-03<br>9.24E-02<br>9.909E-01	8.65E-17<br>-7.07E-16<br>6.22E-16<br>6.49E-02<br>-6.44E-19<br>9.909E-01
(glyceraldehyde3phosphate dehydrogenase)<br>Local<br>Min<br>Max<br>Normalized peak height<br>Peak sensitivity value<br>Shapiro-Wilk score	7.06E-01<br>4.14E-01<br>9.10E-01<br>2.45E-03<br>7.37E-01<br>9.868E-01	5.02E-03<br>3.88E-03<br>6.31E-03<br>3.67E-03<br>4.98E-03<br>9.985E-01	1.49E-04<br>5.84E-05<br>3.87E-04<br>3.27E-03<br>1.16E-04<br>9.705E-01	1.08E-03<br>4.02E-04<br>3.00E-03<br>3.36E-03<br>8.24E-04<br>9.706E-01	1.51E-02<br>2.87E-03<br>4.69E-02<br>3.18E-03<br>1.09E-02<br>9.698E-01	5.04E-03<br>1.55E-03<br>1.10E-02<br>2.74E-03<br>5.10E-03<br>9.870E-01	8.95E-02<br>2.24E-02<br>2.42E-01<br>3.05E-03<br>7.10E-02<br>9.751E-01	5.25E-02<br>1.25E-02<br>1.40E-01<br>2.93E-03<br>4.18E-02<br>9.773E-01	4.62E-02<br>1.97E-02<br>7.13E-02<br>2.45E-03<br>4.71E-02<br>9.902E-01	1.18E-16<br>-7.60E-16<br>6.77E-16<br>1.17E-01<br>-4.38E-19<br>9.705E-01
(glycerol3phosphate dehydrogenase)<br>Local<br>Min<br>Max<br>Normalized peak height<br>Peak sensitivity value<br>Shapiro-Wilk score	7.06E-01<br>4.12E-01<br>9.05E-01<br>2.43E-03<br>7.55E-01<br>9.869E-01	5.02E-03<br>3.90E-03<br>6.29E-03<br>3.49E-03<br>5.00E-03<br>9.988E-01	1.49E-04<br>6.21E-05<br>3.89E-04<br>3.10E-03<br>1.25E-04<br>9.708E-01	1.08E-03<br>4.33E-04<br>3.00E-03<br>3.39E-03<br>9.24E-04<br>9.710E-01	1.51E-02<br>3.51E-03<br>4.54E-02<br>2.92E-03<br>1.12E-02<br>9.703E-01	5.04E-03<br>1.63E-03<br>1.10E-02<br>2.72E-03<br>4.20E-03<br>9.874E-01	8.95E-02<br>2.36E-02<br>2.42E-01<br>2.91E-03<br>7.80E-02<br>9.755E-01	5.25E-02<br>1.44E-02<br>1.36E-01<br>2.84E-03<br>4.34E-02<br>9.776E-01	4.62E-02<br>1.86E-02<br>7.04E-02<br>2.46E-03<br>4.89E-02<br>9.900E-01	1.18E-16<br>-7.72E-16<br>7.85E-16<br>1.18E-01<br>-4.50E-19<br>9.707E-01
(glycerol3phosphate oxidase)<br>Local<br>Min<br>Max<br>Normalized peak height<br>Peak sensitivity value<br>Shapiro-Wilk score	4.14E-01<br>-2.02E-01<br>8.10E-01<br>2.38E-03<br>4.54E-01<br>9.862E-01	2.95E-03<br>-2.57E-03<br>5.07E-03<br>3.62E-03<br>3.24E-03<br>9.690E-01	2.25E-04<br>7.06E-05<br>6.61E-04<br>3.57E-03<br>1.71E-04<br>9.695E-01	1.71E-03<br>5.21E-04<br>5.17E-03<br>3.72E-03<br>1.34E-03<br>9.698E-01	3.00E-02<br>6.12E-03<br>1.00E-01<br>3.32E-03<br>2.40E-02<br>9.691E-01	1.02E-02<br>3.32E-03<br>2.35E-02<br>2.82E-03<br>8.97E-03<br>9.860E-01	1.80E-01<br>4.60E-02<br>5.31E-01<br>3.25E-03<br>1.49E-01<br>9.740E-01	1.06E-01<br>2.71E-02<br>2.80E-01<br>2.96E-03<br>1.04E-01<br>9.764E-01	9.60E-02<br>4.09E-02<br>1.55E-01<br>2.34E-03<br>9.68E-02<br>9.909E-01	8.65E-17<br>-6.25E-16<br>6.00E-16<br>6.34E-02<br>4.55E-19<br>9.902E-01
(pyruvate transport)<br>Local<br>Min<br>Max<br>Normalized peak height<br>Peak sensitivity value<br>Shapiro-Wilk score	7.06E-01<br>3.76E-01<br>9.05E-01<br>2.62E-03<br>7.61E-01<br>9.872E-01	5.02E-03<br>3.90E-03<br>6.30E-03<br>3.47E-03<br>5.04E-03<br>9.987E-01	1.49E-04<br>5.71E-05<br>3.91E-04<br>3.23E-03<br>1.29E-04<br>9.711E-01	1.08E-03<br>4.00E-04<br>2.93E-03<br>3.28E-03<br>8.64E-04<br>9.710E-01	1.51E-02<br>3.30E-03<br>4.63E-02<br>3.08E-03<br>1.13E-02<br>9.697E-01	5.04E-03<br>1.74E-03<br>1.10E-02<br>2.82E-03<br>4.72E-03<br>9.873E-01	8.95E-02<br>2.39E-02<br>2.46E-01<br>3.21E-03<br>8.00E-02<br>9.751E-01	5.25E-02<br>1.35E-02<br>1.41E-01<br>3.05E-03<br>4.49E-02<br>9.776E-01	4.62E-02<br>2.00E-02<br>7.05E-02<br>2.30E-03<br>4.77E-02<br>9.905E-01	-2.22E-16<br>-8.88E-16<br>7.77E-16<br>3.42E-01<br>2.78E-19<br>9.306E-01

	(phosphoglyceratekinase)	(pyruvatekinase)	(atputilisation)	(glycerolkinase)	(phosphoglyceratemutase)	(enolase)	(adenylatekinasecytosol)	(adenylatekinaseglycosome)	(3phosphoglyceratetransport)	(gly3pdhapantiporter)	(glyceroltransport)
(glucose transport)<br>Local<br>Min<br>Max<br>Normalized peak height<br>Peak sensitivity value<br>Shapiro-Wilk score	3.73E-04<br>1.64E-04<br>1.22E-03<br>4.46E-03<br>3.07E-04<br>9.438E-01	4.31E-04<br>1.17E-04<br>1.19E-03<br>3.45E-03<br>3.49E-04<br>9.704E-01	8.87E-05<br>2.58E-05<br>2.52E-04<br>3.56E-03<br>7.80E-05<br>9.710E-01	3.95E-04<br>2.86E-05<br>3.75E-03<br>7.38E-03<br>1.76E-04<br>8.714E-01	3.65E-03<br>7.87E-04<br>1.33E-02<br>3.72E-03<br>3.10E-03<br>9.449E-01	6.87E-04<br>1.85E-04<br>1.97E-03<br>3.33E-03<br>5.66E-04<br>9.691E-01	-6.78E-21<br>-2.80E-20<br>5.03E-20<br>4.05E-01<br>3.33E-24<br>1.000E+00	1.71E-21<br>-1.86E-19<br>5.55E-18<br>2.38E-01<br>6.01E-22<br>2.425E-01	1.07E-08<br>4.24E-09<br>2.90E-08<br>4.01E-03<br>9.71E-09<br>9.743E-01	-4.55E-09<br>-6.97E-08<br>-2.16E-10<br>1.09E-02<br>-1.85E-09<br>8.222E-01	6.60E-06<br>5.07E-07<br>5.10E-05<br>6.11E-03<br>3.26E-06<br>8.899E-01
(hexokinase)<br>Local<br>Min<br>Max<br>Normalized peak height<br>Peak sensitivity value<br>Shapiro-Wilk score	3.73E-04<br>1.68E-04<br>1.41E-03<br>5.13E-03<br>3.04E-04<br>9.442E-01	4.31E-04<br>1.42E-04<br>1.34E-03<br>3.57E-03<br>3.45E-04<br>9.709E-01	8.87E-05<br>2.98E-05<br>2.87E-04<br>3.83E-03<br>7.16E-05<br>9.715E-01	3.95E-04<br>3.27E-05<br>4.24E-03<br>8.44E-03<br>1.74E-04<br>8.721E-01	3.65E-03<br>9.08E-04<br>1.35E-02<br>3.59E-03<br>3.12E-03<br>9.459E-01	6.87E-04<br>2.22E-04<br>2.31E-03<br>3.78E-03<br>5.75E-04<br>9.695E-01	-6.78E-21<br>-2.40E-20<br>3.60E-20<br>4.02E-01<br>-1.02E-23<br>1.000E+00	1.71E-21<br>-1.86E-19<br>4.27E-18<br>1.87E-01<br>-1.48E-21<br>2.956E-01	1.07E-08<br>4.07E-09<br>2.67E-08<br>3.72E-03<br>1.01E-08<br>9.747E-01	-4.55E-09<br>-8.60E-08<br>-2.51E-10<br>1.34E-02<br>-1.92E-09<br>8.252E-01	6.60E-06<br>6.52E-07<br>5.91E-05<br>7.21E-03<br>3.25E-06<br>8.903E-01
(phosphoglycerate isomerase)<br>Local<br>Min<br>Max<br>Normalized peak height<br>Peak sensitivity value<br>Shapiro-Wilk score	3.73E-04<br>1.65E-04<br>1.19E-03<br>4.11E-03<br>3.20E-04<br>9.437E-01	4.31E-04<br>1.44E-04<br>1.27E-03<br>3.51E-03<br>3.84E-04<br>9.705E-01	8.87E-05<br>3.05E-05<br>2.60E-04<br>3.51E-03<br>7.44E-05<br>9.711E-01	3.95E-04<br>4.20E-05<br>3.35E-03<br>6.47E-03<br>1.83E-04<br>8.712E-01	3.65E-03<br>9.66E-04<br>1.41E-02<br>3.97E-03<br>3.17E-03<br>9.454E-01	6.87E-04<br>2.30E-04<br>2.04E-03<br>3.44E-03<br>5.61E-04<br>9.694E-01	-6.78E-21<br>-2.30E-20<br>4.68E-20<br>4.05E-01<br>2.19E-23<br>1.000E+00	1.71E-21<br>-2.17E-19<br>6.46E-18<br>2.61E-01<br>5.06E-22<br>2.344E-01	1.07E-08<br>4.09E-09<br>2.68E-08<br>3.91E-03<br>1.01E-08<br>9.737E-01	-4.55E-09<br>-5.95E-08<br>-2.21E-10<br>9.11E-03<br>-1.61E-09<br>8.234E-01	6.60E-06<br>7.18E-07<br>4.85E-05<br>5.79E-03<br>3.08E-06<br>8.894E-01
(phophofructokinase)<br>Local<br>Min<br>Max<br>Normalized peak height<br>Peak sensitivity value<br>Shapiro-Wilk score	3.73E-04<br>1.62E-04<br>1.41E-03<br>5.33E-03<br>3.03E-04<br>9.443E-01	4.31E-04<br>1.41E-04<br>1.24E-03<br>3.38E-03<br>3.79E-04<br>9.706E-01	8.87E-05<br>2.96E-05<br>2.57E-04<br>3.55E-03<br>7.55E-05<br>9.711E-01	3.95E-04<br>3.65E-05<br>4.62E-03<br>9.26E-03<br>2.04E-04<br>8.718E-01	3.65E-03<br>9.17E-04<br>1.36E-02<br>3.91E-03<br>2.61E-03<br>9.457E-01	6.87E-04<br>2.16E-04<br>2.04E-03<br>3.37E-03<br>6.19E-04<br>9.691E-01	-6.78E-21<br>-2.79E-20<br>5.77E-20<br>4.02E-01<br>7.36E-24<br>1.000E+00	1.71E-21<br>-2.46E-19<br>5.24E-18<br>2.09E-01<br>-2.30E-21<br>2.506E-01	1.07E-08<br>4.14E-09<br>2.73E-08<br>3.74E-03<br>9.59E-09<br>9.745E-01	-4.55E-09<br>-8.04E-08<br>-2.08E-10<br>1.27E-02<br>-1.69E-09<br>8.239E-01	6.60E-06<br>6.58E-07<br>5.91E-05<br>6.98E-03<br>3.61E-06<br>8.907E-01
(aldolase)<br>Local<br>Min<br>Max<br>Normalized peak height<br>Peak sensitivity value<br>Shapiro-Wilk score	3.73E-04<br>1.65E-04<br>1.24E-03<br>4.52E-03<br>3.13E-04<br>9.441E-01	4.31E-04<br>1.37E-04<br>1.29E-03<br>3.51E-03<br>3.58E-04<br>9.704E-01	8.87E-05<br>2.85E-05<br>2.60E-04<br>3.53E-03<br>7.28E-05<br>9.710E-01	3.95E-04<br>3.28E-05<br>3.48E-03<br>6.80E-03<br>1.93E-04<br>8.733E-01	3.65E-03<br>9.42E-04<br>1.37E-02<br>3.76E-03<br>2.70E-03<br>9.452E-01	6.87E-04<br>2.16E-04<br>2.09E-03<br>3.44E-03<br>5.51E-04<br>9.690E-01	-6.78E-21<br>-3.14E-20<br>3.44E-20<br>4.02E-01<br>-2.27E-23<br>1.000E+00	1.71E-21<br>-1.90E-19<br>4.91E-18<br>2.16E-01<br>1.04E-21<br>2.694E-01	1.07E-08<br>4.28E-09<br>2.61E-08<br>3.54E-03<br>1.02E-08<br>9.739E-01	-4.55E-09<br>-6.34E-08<br>-1.52E-10<br>1.02E-02<br>-1.77E-09<br>8.252E-01	6.60E-06<br>6.25E-07<br>4.96E-05<br>6.15E-03<br>3.44E-06<br>8.909E-01
(triosephosphate isomerase)<br>Local<br>Min<br>Max<br>Normalized peak height<br>Peak sensitivity value<br>Shapiro-Wilk score	1.45E-02<br>8.11E-03<br>2.31E-02<br>2.71E-03<br>1.39E-02<br>9.934E-01	1.68E-02<br>6.07E-03<br>2.52E-02<br>2.86E-03<br>1.81E-02<br>9.880E-01	3.45E-03<br>1.29E-03<br>5.12E-03<br>2.95E-03<br>3.81E-03<br>9.883E-01	-4.42E-02<br>-7.02E-02<br>-1.82E-02<br>2.66E-03<br>-4.54E-02<br>9.945E-01	1.42E-01<br>4.12E-02<br>2.80E-01<br>2.68E-03<br>1.41E-01<br>9.865E-01	2.67E-02<br>9.37E-03<br>4.01E-02<br>2.61E-03<br>2.80E-02<br>9.876E-01	-2.64E-19<br>-6.98E-19<br>9.94E-19<br>4.05E-01<br>6.02E-23<br>9.519E-01	1.27E-19<br>-6.59E-18<br>1.73E-16<br>6.67E-02<br>-1.91E-20<br>4.203E-01	4.16E-07<br>2.04E-07<br>6.70E-07<br>3.45E-03<br>4.02E-07<br>9.964E-01	1.10E-06<br>5.09E-07<br>1.93E-06<br>2.84E-03<br>1.08E-06<br>9.941E-01	-7.39E-04<br>-1.14E-03<br>-3.17E-04<br>3.22E-03<br>-7.80E-04<br>9.970E-01
(glyceraldehyde3phosphate dehydrogenase)<br>Local<br>Min<br>Max<br>Normalized peak height<br>Peak sensitivity value<br>Shapiro-Wilk score	7.20E-03<br>4.18E-03<br>1.11E-02<br>2.76E-03<br>7.30E-03<br>9.940E-01	8.34E-03<br>3.34E-03<br>1.24E-02<br>2.81E-03<br>8.95E-03<br>9.864E-01	1.71E-03<br>7.07E-04<br>2.49E-03<br>2.90E-03<br>1.85E-03<br>9.862E-01	-2.12E-02<br>-3.13E-02<br>-1.00E-02<br>2.57E-03<br>-2.21E-02<br>9.916E-01	7.05E-02<br>2.19E-02<br>1.37E-01<br>2.72E-03<br>6.59E-02<br>9.872E-01	1.33E-02<br>5.17E-03<br>1.96E-02<br>2.66E-03<br>1.47E-02<br>9.865E-01	-1.31E-19<br>-3.42E-19<br>1.08E-18<br>4.01E-01<br>-3.75E-22<br>9.504E-01	6.21E-20<br>-3.47E-18<br>9.09E-17<br>6.82E-02<br>-2.75E-20<br>3.769E-01	2.07E-07<br>1.05E-07<br>3.28E-07<br>3.38E-03<br>2.00E-07<br>9.964E-01	5.29E-07<br>2.60E-07<br>8.71E-07<br>2.72E-03<br>5.22E-07<br>9.956E-01	-3.54E-04<br>-4.99E-04<br>-1.74E-04<br>3.19E-03<br>-3.54E-04<br>9.958E-01
(glycerol3phosphate dehydrogenase)<br>Local<br>Min<br>Max<br>Normalized peak height<br>Peak sensitivity value<br>Shapiro-Wilk score	7.20E-03<br>4.01E-03<br>1.13E-02<br>2.79E-03<br>6.78E-03<br>9.940E-01	8.34E-03<br>3.43E-03<br>1.18E-02<br>2.63E-03<br>8.98E-03<br>9.862E-01	1.71E-03<br>7.31E-04<br>2.42E-03<br>2.85E-03<br>1.83E-03<br>9.860E-01	-2.12E-02<br>-3.12E-02<br>-9.59E-03<br>2.70E-03<br>-2.26E-02<br>9.914E-01	7.05E-02<br>2.29E-02<br>1.36E-01<br>2.64E-03<br>6.92E-02<br>9.867E-01	1.33E-02<br>5.08E-03<br>1.94E-02<br>2.72E-03<br>1.35E-02<br>9.864E-01	-1.31E-19<br>-3.90E-19<br>9.58E-19<br>4.10E-01<br>3.77E-22<br>9.474E-01	6.21E-20<br>-2.71E-18<br>1.82E-16<br>1.31E-01<br>-3.21E-20<br>3.243E-01	2.07E-07<br>1.05E-07<br>3.30E-07<br>3.61E-03<br>1.96E-07<br>9.963E-01	5.29E-07<br>2.45E-07<br>8.89E-07<br>3.06E-03<br>5.64E-07<br>9.953E-01	-3.54E-04<br>-5.03E-04<br>-1.65E-04<br>3.38E-03<br>-3.53E-04<br>9.959E-01
(glycerol3phosphate oxidase)<br>Local<br>Min<br>Max<br>Normalized peak height<br>Peak sensitivity value<br>Shapiro-Wilk score	1.45E-02<br>8.26E-03<br>2.27E-02<br>2.67E-03<br>1.31E-02<br>9.934E-01	1.68E-02<br>7.22E-03<br>2.54E-02<br>2.71E-03<br>1.82E-02<br>9.884E-01	3.45E-03<br>1.49E-03<br>5.14E-03<br>2.86E-03<br>3.83E-03<br>9.885E-01	-4.42E-02<br>-7.02E-02<br>-2.11E-02<br>2.66E-03<br>-4.56E-02<br>9.942E-01	1.42E-01<br>4.70E-02<br>2.82E-01<br>2.67E-03<br>1.32E-01<br>9.862E-01	2.67E-02<br>1.11E-02<br>4.00E-02<br>2.38E-03<br>2.64E-02<br>9.875E-01	-2.64E-19<br>-7.01E-19<br>7.08E-19<br>4.08E-01<br>-1.35E-22<br>9.504E-01	1.27E-19<br>-5.80E-18<br>1.74E-16<br>6.38E-02<br>2.25E-19<br>3.405E-01	4.16E-07<br>2.15E-07<br>6.55E-07<br>3.31E-03<br>4.08E-07<br>9.963E-01	1.10E-06<br>4.74E-07<br>1.89E-06<br>2.76E-03<br>1.04E-06<br>9.939E-01	-7.39E-04<br>-1.14E-03<br>-3.56E-04<br>3.13E-03<br>-7.32E-04<br>9.972E-01
(pyruvate transport)<br>Local<br>Min<br>Max<br>Normalized peak height<br>Peak sensitivity value<br>Shapiro-Wilk score	7.20E-03<br>4.11E-03<br>1.11E-02<br>2.73E-03<br>6.98E-03<br>9.941E-01	8.34E-03<br>3.33E-03<br>1.21E-02<br>2.73E-03<br>9.12E-03<br>9.868E-01	1.71E-03<br>7.12E-04<br>2.46E-03<br>2.79E-03<br>1.88E-03<br>9.866E-01	-2.12E-02<br>-3.11E-02<br>-1.02E-02<br>2.63E-03<br>-2.11E-02<br>9.919E-01	7.05E-02<br>2.31E-02<br>1.35E-01<br>2.55E-03<br>6.17E-02<br>9.873E-01	1.33E-02<br>5.28E-03<br>1.97E-02<br>2.66E-03<br>1.45E-02<br>9.865E-01	-1.31E-19<br>-3.27E-19<br>9.63E-19<br>4.08E-01<br>-1.70E-22<br>9.483E-01	6.21E-20<br>-2.94E-18<br>1.02E-16<br>8.10E-02<br>4.16E-20<br>3.620E-01	2.07E-07<br>1.13E-07<br>3.27E-07<br>3.32E-03<br>1.95E-07<br>9.967E-01	5.29E-07<br>2.60E-07<br>8.95E-07<br>2.75E-03<br>5.28E-07<br>9.956E-01	-3.54E-04<br>-5.15E-04<br>-1.85E-04<br>3.24E-03<br>-3.51E-04<br>9.959E-01

(phosphoglycerate kinase)																					
Local	7.06E-01	5.02E-03	1.49E-04	1.08E-03	1.51E-02	5.04E-03	8.95E-02	5.25E-02	4.62E-02	2.34E-17	7.20E-03	8.34E-03	1.71E-03	-2.12E-02	7.05E-02	1.33E-02	-1.31E-19	6.21E-20	2.07E-07	5.29E-07	-3.54E-04
Min	3.97E-01	3.95E-03	5.90E-05	4.00E-04	3.24E-03	1.67E-03	2.45E-02	1.38E-02	1.95E-02	-5.75E-16	4.16E-03	3.39E-03	6.83E-04	-3.14E-02	2.20E-02	4.90E-03	-3.25E-19	-2.77E-18	1.09E-07	2.31E-07	-5.07E-04
Max	9.02E-01	6.42E-03	3.77E-04	2.84E-03	4.73E-02	1.07E-02	2.45E-01	1.41E-01	7.04E-02	5.63E-16	1.11E-02	1.23E-02	2.44E-03	-9.48E-03	1.37E-01	1.95E-02	7.01E-19	1.82E-16	3.37E-07	9.17E-07	-1.74E-04
Normalized peak height	2.43E-03	3.59E-03	3.17E-03	3.21E-03	3.13E-03	2.53E-03	3.08E-03	2.94E-03	2.32E-03	4.78E-02	2.67E-03	2.86E-03	2.93E-03	2.69E-03	2.74E-03	2.63E-03	4.12E-01	1.38E-01	3.57E-03	3.01E-03	3.28E-03
Peak sensitivity value	7.37E-01	4.99E-03	1.33E-04	1.03E-03	1.21E-02	4.42E-03	7.57E-02	4.43E-02	5.07E-02	4.47E-19	7.20E-03	8.75E-03	1.88E-03	-2.25E-02	7.41E-02	1.46E-02	-1.59E-22	-9.18E-20	1.96E-07	5.45E-07	-3.70E-04
Shapiro-Wilk score	9.869E-01	9.986E-01	9.709E-01	9.709E-01	9.699E-01	9.873E-01	9.751E-01	9.774E-01	9.900E-01	9.715E-01	9.938E-01	9.864E-01	9.862E-01	9.916E-01	9.872E-01	9.861E-01	9.483E-01	3.532E-01	9.967E-01	9.955E-01	9.958E-01
(pyruvate kinase)																					
Local	7.06E-01	5.02E-03	1.49E-04	1.08E-03	1.51E-02	5.04E-03	8.95E-02	5.25E-02	4.62E-02	-2.12E-16	7.20E-03	8.34E-03	1.71E-03	-2.12E-02	7.05E-02	1.33E-02	-1.31E-19	6.21E-20	2.07E-07	5.29E-07	-3.54E-04
Min	4.11E-01	3.91E-03	5.93E-05	4.19E-04	3.14E-03	1.64E-03	2.33E-02	1.46E-02	2.09E-02	-9.61E-16	4.28E-03	3.55E-03	7.30E-04	-3.12E-02	2.45E-02	5.41E-03	-3.30E-19	-2.85E-18	1.09E-07	2.59E-07	-5.14E-04
Max	9.02E-01	6.40E-03	3.70E-04	2.75E-03	4.49E-02	1.10E-02	2.39E-01	1.40E-01	7.00E-02	7.50E-16	1.11E-02	1.23E-02	2.50E-03	-1.05E-02	1.37E-01	1.96E-02	1.54E-18	1.37E-16	3.25E-07	8.84E-07	-1.80E-04
Normalized peak height	2.46E-03	3.67E-03	3.18E-03	3.24E-03	3.12E-03	2.83E-03	2.95E-03	3.04E-03	2.25E-03	1.34E-01	2.75E-03	2.73E-03	3.02E-03	2.51E-03	2.56E-03	2.58E-03	4.12E-01	1.01E-01	3.45E-03	2.75E-03	3.22E-03
Peak sensitivity value	7.58E-01	4.99E-03	1.29E-04	9.07E-04	1.26E-02	4.38E-03	7.85E-02	4.51E-02	5.03E-02	-6.43E-19	7.62E-03	9.01E-03	1.85E-03	-2.33E-02	6.50E-02	1.41E-02	2.33E-22	2.39E-20	1.97E-07	5.30E-07	-3.66E-04
Shapiro-Wilk score	9.867E-01	9.988E-01	9.701E-01	9.702E-01	9.693E-01	9.869E-01	9.746E-01	9.769E-01	9.902E-01	9.941E-01	9.939E-01	9.867E-01	9.865E-01	9.919E-01	9.870E-01	9.866E-01	9.442E-01	3.692E-01	9.965E-01	9.954E-01	9.961E-01
(atp utilisation)																					
Local	7.06E-01	5.02E-03	1.49E-04	1.08E-03	1.51E-02	5.04E-03	8.95E-02	5.25E-02	4.62E-02	2.20E-21	7.20E-03	8.34E-03	1.71E-03	-2.12E-02	7.05E-02	1.33E-02	-1.31E-19	6.21E-20	2.07E-07	5.29E-07	-3.54E-04
Min	3.69E-01	3.94E-03	6.16E-05	4.17E-04	3.15E-03	1.65E-03	2.26E-02	1.33E-02	1.96E-02	-1.28E-13	3.89E-03	3.51E-03	6.99E-04	-3.14E-02	2.36E-02	5.22E-03	-3.56E-19	-3.00E-18	1.08E-07	2.38E-07	-5.02E-04
Max	9.02E-01	6.33E-03	3.88E-04	2.94E-03	5.38E-02	1.12E-02	2.73E-01	1.55E-01	6.92E-02	1.41E-13	1.09E-02	1.25E-02	2.57E-03	-9.87E-03	1.35E-01	2.02E-02	5.45E-19	8.79E-17	3.29E-07	8.77E-07	-1.75E-04
Normalized peak height	2.62E-03	3.46E-03	3.25E-03	3.35E-03	3.64E-03	2.71E-03	3.40E-03	3.28E-03	2.22E-03	2.18E-01	2.74E-03	2.80E-03	2.98E-03	2.61E-03	2.65E-03	2.71E-03	4.10E-01	7.47E-02	3.49E-03	2.89E-03	3.13E-03
Peak sensitivity value	7.61E-01	5.05E-03	1.25E-04	9.30E-04	1.26E-02	4.54E-03	6.95E-02	4.17E-02	4.82E-02	2.66E-17	6.76E-03	9.07E-03	1.88E-03	-2.18E-02	7.34E-02	1.43E-02	1.60E-22	4.39E-20	1.97E-07	5.37E-07	-3.57E-04
Shapiro-Wilk score	9.867E-01	9.986E-01	9.704E-01	9.705E-01	9.696E-01	9.871E-01	9.749E-01	9.770E-01	9.900E-01	9.055E-01	9.940E-01	9.871E-01	9.868E-01	9.919E-01	9.866E-01	9.869E-01	9.496E-01	3.504E-01	9.965E-01	9.956E-01	9.960E-01
(glycerol kinase)																					
Local	9.22E+00	6.57E-02	-2.07E-03	-1.73E-02	-4.20E-01	-1.47E-01	-2.56E+00	-1.50E+00	-1.41E+00	1.05E-15	-2.06E-01	-2.39E-01	-4.91E-02	6.53E-01	-2.02E+00	-3.80E-01	3.75E-18	-1.82E-18	-5.91E-06	-1.61E-05	1.09E-02
Min	6.69E+00	4.00E-02	-3.88E-03	-3.23E-02	-6.93E-01	-1.96E-01	-3.75E+00	-2.22E+00	-1.85E+00	-9.28E-15	-3.57E-01	-3.16E-01	-6.54E-02	3.92E-01	-3.12E+00	-4.92E-01	-6.08E-17	-2.34E-15	-1.20E-05	-2.58E-05	4.86E-03
Max	1.08E+01	1.01E-01	-9.16E-04	-8.05E-03	-2.20E-01	-9.71E-02	-1.73E+00	-9.95E-01	-8.12E-01	8.47E-15	-1.03E-01	-1.04E-01	-2.14E-02	8.67E-01	-8.18E-01	-1.61E-01	9.57E-18	6.21E-17	-1.84E-06	-8.70E-06	1.82E-02
Normalized peak height	4.45E-03	3.33E-03	3.67E-03	3.90E-03	3.77E-03	3.64E-03	3.20E-03	3.51E-03	2.91E-03	1.19E-01	3.10E-03	2.72E-03	2.54E-03	2.19E-03	3.80E-03	3.04E-03	4.10E-01	6.60E-02	2.76E-03	2.96E-03	2.74E-03
Peak sensitivity value	9.05E+00	6.64E-02	-1.97E-03	-1.64E-02	-3.94E-01	-1.43E-01	-2.54E+00	-1.46E+00	-1.42E+00	-6.44E-18	-1.96E-01	-2.57E-01	-5.32E-02	6.70E-01	-1.92E+00	-3.99E-01	-2.62E-20	9.07E-19	-5.57E-06	-1.62E-05	1.03E-02
Shapiro-Wilk score	9.947E-01	9.956E-01	9.901E-01	9.907E-01	9.944E-01	9.999E-01	9.910E-01	9.918E-01	9.910E-01	9.968E-01	9.917E-01	9.790E-01	9.786E-01	9.886E-01	9.993E-01	9.736E-01	9.397E-01	4.411E-01	9.887E-01	9.949E-01	9.944E-01
(phosphoglycerate mutase)																					
Local	7.06E-01	5.02E-03	1.49E-04	1.08E-03	1.51E-02	5.04E-03	8.95E-02	5.25E-02	4.62E-02	2.10E-17	7.20E-03	8.34E-03	1.71E-03	-2.12E-02	7.05E-02	1.33E-02	-1.31E-19	6.21E-20	2.07E-07	5.29E-07	-3.54E-04
Min	4.08E-01	3.95E-03	6.02E-05	4.08E-04	2.69E-03	1.46E-03	2.03E-02	1.24E-02	1.82E-02	-5.05E-16	4.02E-03	3.16E-03	6.39E-04	-3.05E-02	2.16E-02	4.80E-03	-3.67E-19	-3.08E-18	1.07E-07	2.51E-07	-5.00E-04
Max	9.14E-01	6.20E-03	3.62E-04	2.68E-03	4.44E-02	1.05E-02	2.42E-01	1.40E-01	7.02E-02	5.20E-16	1.10E-02	1.20E-02	2.41E-03	-9.29E-03	1.33E-01	1.91E-02	3.92E-19	1.07E-16	3.38E-07	8.95E-07	-1.69E-04
Normalized peak height	2.41E-03	3.32E-03	2.99E-03	3.13E-03	3.02E-03	2.61E-03	3.02E-03	3.23E-03	2.46E-03	1.40E-01	2.72E-03	2.83E-03	2.91E-03	2.62E-03	2.55E-03	2.55E-03	4.11E-01	8.53E-02	3.55E-03	2.90E-03	3.29E-03
Peak sensitivity value	7.36E-01	5.02E-03	1.24E-04	1.03E-03	1.51E-02	4.98E-03	8.07E-02	4.45E-02	4.98E-02	1.19E-19	7.77E-03	9.14E-03	1.88E-03	-2.09E-02	6.43E-02	1.50E-02	-2.39E-22	-4.75E-20	1.95E-07	5.32E-07	-3.63E-04
Shapiro-Wilk score	9.875E-01	9.988E-01	9.714E-01	9.716E-01	9.709E-01	9.878E-01	9.760E-01	9.781E-01	9.907E-01	9.657E-01	9.944E-01	9.865E-01	9.862E-01	9.919E-01	9.877E-01	9.866E-01	9.490E-01	3.644E-01	9.964E-01	9.958E-01	9.956E-01
(enolase)																					
Local	7.06E-01	5.02E-03	1.49E-04	1.08E-03	1.51E-02	5.04E-03	8.95E-02	5.25E-02	4.62E-02	2.10E-17	7.20E-03	8.34E-03	1.71E-03	-2.12E-02	7.05E-02	1.33E-02	-1.31E-19	6.21E-20	2.07E-07	5.29E-07	-3.54E-04
Min	4.09E-01	3.93E-03	5.98E-05	4.24E-04	3.25E-03	1.73E-03	2.43E-02	1.40E-02	2.07E-02	-5.71E-16	4.24E-03	3.53E-03	7.25E-04	-3.04E-02	2.35E-02	5.58E-03	-3.61E-19	-3.08E-18	1.01E-07	2.50E-07	-5.05E-04
Max	8.99E-01	6.24E-03	3.80E-04	2.85E-03	4.51E-02	1.10E-02	2.44E-01	1.42E-01	7.01E-02	5.26E-16	1.08E-02	1.23E-02	2.47E-03	-1.00E-02	1.40E-01	1.98E-02	1.80E-18	1.69E-16	3.24E-07	8.69E-07	-1.72E-04
Normalized peak height	2.38E-03	3.44E-03	3.08E-03	3.16E-03	2.95E-03	2.70E-03	3.04E-03	2.95E-03	2.24E-03	1.40E-01	2.70E-03	2.81E-03	2.89E-03	2.65E-03	2.69E-03	2.58E-03	4.14E-01	1.27E-01	3.53E-03	2.74E-03	3.20E-03
Peak sensitivity value	7.17E-01	4.91E-03	1.26E-04	8.98E-04	1.25E-02	4.70E-03	7.59E-02	4.39E-02	4.65E-02	-4.81E-20	6.82E-03	8.94E-03	1.89E-03	-2.19E-02	7.29E-02	1.40E-02	-1.08E-21	-7.73E-20	1.98E-07	5.18E-07	-3.71E-04
Shapiro-Wilk score	9.872E-01	9.983E-01	9.707E-01	9.707E-01	9.701E-01	9.874E-01	9.753E-01	9.775E-01	9.903E-01	9.655E-01	9.938E-01	9.865E-01	9.862E-01	9.916E-01	9.871E-01	9.862E-01	9.430E-01	3.439E-01	9.964E-01	9.955E-01	9.961E-01
(adenylate kinase cytosol)																					
Local	INF	INF	INF	INF	INF	INF	INF	INF	INF	INF	INF	INF	INF	INF	INF	INF	INF	INF	INF	INF	INF
Min	-INF	-INF	-INF	-INF	-INF	-INF	-INF	-INF	-INF	-INF	-INF	-INF	-INF	-INF	-INF	-INF	-INF	-INF	-INF	-INF	-INF
Max	INF	INF	INF	INF	INF	INF	INF	INF	INF	INF	INF	INF	INF	INF	INF	INF	INF	INF	INF	INF	INF
Normalized peak height	NA	NA	NA	NA	NA	NA	NA	NA	NA	NA	NA	NA	NA	NA	NA	NA	NA	NA	NA	NA	NA
Peak sensitivity value	NAN	NAN	NAN	NAN	NAN	NAN	NAN	NAN	NAN	NAN	NAN	NAN	NAN	NAN	NAN	NAN	NAN	NAN	NAN	NAN	NAN
Shapiro-Wilk score	NA	NA	NA	NA	NA	NA	NA	NA	NA	NA	NA	NA	NA	NA	NA	NA	NA	NA	NA	NA	NA
(adenylate kinase glycosome)																					
Local	-INF	-INF	-INF	-INF	-INF	-INF	-INF	-INF	-INF	-INF	-INF	-INF	-INF	-INF	-INF	-INF	-INF	-INF	-INF	-INF	-INF
Min	-INF	-INF	-INF	-INF	-INF	-INF	-INF	-INF	-INF	-INF	-INF	-INF	-INF	-INF	-INF	-INF	-INF	-INF	-INF	-INF	-INF
Max	INF	INF	INF	INF	INF	INF	INF	INF	INF	INF	INF	INF	INF	INF	INF	INF	INF	INF	INF	INF	INF
Normalized peak height	5.98E-02	8.14E-03	8.09E-03	8.08E-03	6.08E-02	3.75E-02	5.18E-02	5.77E-02	6.74E-02	4.55E-02	6.85E-03	5.65E-02	6.02E-02	5.24E-02	5.60E-02	5.00E-02	4.17E-01	4.17E-03	8.90E-03	1.27E-02	5.87E-02
Peak sensitivity value	-4.19E-02	-1.68E-02	-5.10E-02	5.20E-03	1.15E-03	1.13E-04	7.99E-04	5.63E-03	7.84E-04	-3.70E-08	7.55E-01	7.08E-04	-1.82E-04	8.40E-04	-4.04E-03	2.33E-03	1.47E-16	-2.76E-12	1.71E-02	-8.56E-03	2.71E-05
Shapiro-Wilk score	9.779E-01	9.754E-01	9.799E-01	9.800E-01	9.734E-01	9.712E-01	9.713E-01	9.719E-01	9.048E-01	9.764E-01	9.800E-01	9.792E-01	9.811E-01	9.500E-01	9.754E-01	9.788E-01	8.038E-01	1.000E+00	9.667E-01	9.567E-01	9.571E-01
(3phosphoglycerate transport)																					
Local	7.06E-01	5.02E-03	1.49E-04	1.08E-03	1.51E-02	5.04E-03	8.95E-02	5.25E-02	4.62E-02	2.34E-17	7.20E-03	8.34E-03	1.71E-03	-2.12E-02	7.05E-02	1.33E-02	-1.31E-19	6.20E-20	2.07E-07	5.29E-07	-3.54E-04
Min	4.13E-01	3.93E-03	6.29E-05	4.35E-04	3.16E-03	1.69E-03	2.33E-02	1.35E-02	2.11E-02	-5.20E-16	4.22E-03	3.72E-03	7.83E-04	-3.10E-02	2.54E-02	5.75E-03	-3.30E-19	-2.90E-18	1.09E-07	2.43E-07	-5.22E-04
Max	9.00E-01	6.26E-03	4.15E-04	3.17E-03	4.61E-02	1.11E-02	2.47E-01	1.38E-01	7.17E-02	5.54E-16	1.09E-02	1.23E-02	2.48E-03	-1.06E-02	1.33E-01	1.91E-02	5.45E-19	1.13E-16	3.19E-07	8.62E-07	-1.83E-04
Normalized peak height	2.30E-03	3.44E-03	3.37E-03	3.71E-03	3.16E-03	2.93E-03	3.10E-03	2.87E-03	2.28E-03	4.81E-02	2.67E-03	2.77E-03	2.73E-03	2.47E-03	2.57E-03	2.57E-03	4.08E-01	9.28E-02	3.30E-03	2.75E-03	3.37E-03
Peak sensitivity value	7.41E-01	5.00E-03	1.19E-04	8.29E-04	1.00E-02	4.40E-03	7.06E-02	4.50E-02	4.89E-02	-4.00E-21	7.54E-03	9.19E-03	1.81E-03	-2.19E-02	6.60E-02	1.42E-02	2.41E-22	5.26E-20	1.96E-07	5.16E-07	-3.58E-04
Shapiro-Wilk score	9.869E-01	9.985E-01	9.704E-01	9.705E-01	9.696E-01	9.871E-01	9.748E-01	9.771E-01	9.904E-01	9.715E-01	9.940E-01	9.870E-01	9.868E-01	9.920E-01	9.868E-01	9.867E-01	9.498E-01	3.758E-01	9.965E-01	9.955E-01	9.960E-01
(gly3p dhap antiporter)																					
Local	4.14E-01	2.95E-03	2.25E-04	1.71E-03	3.00E-02	1.02E-02	1.80E-01	1.06E-01	9.60E-02	8.65E-17	1.45E-02	1.68E-02	3.45E-03	-4.42E-02	1.42E-01	2.67E-02	-2.64E-19	1.27E-19	4.16E-07	1.10E-06	-7.39E-04
Min	-2.08E-01	-2.21E-03	7.94E-05	5.60E-04	5.95E-03	3.23E-03	4.42E-02	2.67E-02	3.96E-02	-7.27E-16	8.48E-03	6.88E-03	1.39E-03	-6.98E-02	4.42E-02	1.04E-02	-8.09E-19	-6.02E-18	1.92E-07	5.17E-07	-1.10E-03
Max	8.24E-01	5.17E-03	6.21E-04	4.83E-03	9.50E-02	2.27E-02	4.93E-01	2.79E-01	1.58E-01	9.69E-16	2.25E-02	2.52E-02	5.11E-03	-2.03E-02	2.79E-01	3.98E-02	4.47E-18	1.97E-16	6.49E-07	1.93E-06	-3.52E-04
Normalized peak height	2.47E-03	3.50E-03	3.10E-03	3.13E-03	3.19E-03	2.78E-03	2.96E-03	2.94E-03	2.47E-03	8.03E-02	2.63E-03	3.00E-03	2.78E-03	2.59E-03	2.75E-03	2.55E-03	4.11E-01	7.07E-02	3.62E-03	2.81E-03	3.04E-03
Peak sensitivity value	4.16E-01	3.55E-03	1.75E-04	1.42E-03	2.22E-02	9.42E-03	1.49E-01	9.70E-02	9.81E-02	7.60E-20	1.38E-02	1.84E-02	3.57E-03	-4.22E-02	1.39E-01	3.04E-02	1.44E-21	-2.30E-19	4.02E-07	1.09E-06	-7.48E-04
Shapiro-Wilk score	9.868E-01	9.697E-01	9.699E-01	9.701E-01	9.694E-01	9.863E-01	9.742E-01	9.767E-01	9.911E-01	9.907E-01	9.938E-01	9.883E-01	9.884E-01	9.945E-01	9.867E-01	9.878E-01	9.418E-01	3.574E-01	9.964E-01	9.941E-01	9.972E-01
(glycerol transport)																					
Local	9.22E+00	6.57E-02	-2.07E-03	-1.73E-02	-4.20E-01	-1.47E-01	-2.56E+00	-1.50E+00	-1.41E+00	1.05E-15	-2.06E-01	-2.39E-01	-4.91E-02	6.53E-01	-2.02E+00	-3.80E-01	3.75E-18	-1.82E-18	-5.91E-06	-1.61E-05	1.09E-02
Min	6.85E+00	4.20E-02	-3.70E-03	-3.12E-02	-6.84E-01	-1.96E-01	-3.96E+00	-2.25E+00	-1.86E+00	-8.88E-15	-3.60E-01	-3.16E-01	-6.58E-02	3.79E-01	-3.14E+00	-5.01E-01	-1.80E-17	-2.21E-15	-1.15E-05	-2.62E-05	4.75E-03
Max	1.10E+01	1.01E-01	-9.05E-04	-8.19E-03	-2.29E-01	-1.00E-01	-1.73E+00	-9.84E-01	-8.36E-01	9.01E-15	-9.47E-02	-9.70E-02	-1.89E-02	8.78E-01	-8.33E-01	-1.49E-01	8.96E-18	6.52E-17	-2.01E-06	-8.73E-06	1.86E-02
Normalized peak height	4.54E-03	3.20E-03	3.50E-03	3.77E-03	3.55E-03	3.73E-03	3.62E-03	3.79E-03	2.80E-03	1.19E-01	3.08E-03	2.71E-03	2.69E-03	2.31E-03	3.85E-03	3.22E-03	4.11E-01	5.45E-02	2.76E-03	3.03E-03	2.79E-03
Peak sensitivity value	9.17E+00	6.65E-02	-1.94E-03	-1.63E-02	-3.93E-01	-1.45E-01	-2.52E+00	-1.41E+00	-1.48E+00	6.82E-18	-1.88E-01	-2.56E-01	-5.35E-02	5.87E-01	-1.94E+00	-4.13E-01	-9.94E-21	2.55E-18	-5.60E-06	-1.58E-05	1.16E-02
Shapiro-Wilk score	9.952E-01	9.959E-01	9.908E-01	9.914E-01	9.950E-01	9.999E-01	9.917E-01	9.925E-01	9.913E-01	9.965E-01	9.913E-01	9.790E-01	9.784E-01	9.886E-01	9.993E-01	9.738E-01	9.489E-01	4.645E-01	9.884E-01	9.949E-01	9.939E-01

Table S12: Flux control coefficients for *Trypanosoma brucei* model with parameter variation of ±10%

		(glucose transport)	(hexokinase)	(phosphoglycerate isomerase)	(phophofructokinase)	(aldolase)	(triosephosphate isomerase)	(glyceraldehyde3phosphate dehydrogenase)	(glycerol3phosphate dehydrogenase)	(glycerol3phosphate oxidase)	(pyruvate transport)	(phosphoglycerate kinase)
(glucose transport)	Local	9.79E-01	6.97E-03	7.82E-05	4.95E-04	1.13E-03	1.79E-04	4.63E-03	2.72E-03	-3.97E-04	-1.47E-18	3.73E-04
	Min	4.40E-01	3.81E-03	2.39E-05	1.54E-04	5.75E-05	3.96E-06	1.62E-04	9.67E-05	-6.78E-02	-4.62E-16	8.67E-05
	Max	9.95E-01	2.64E-02	8.22E-04	6.28E-03	7.51E-02	5.66E-03	2.65E-01	1.57E-01	9.30E-05	3.47E-16	8.53E-03
	Normalized peak height	2.37E-02	6.47E-03	1.16E-02	1.42E-02	4.98E-02	2.25E-02	4.09E-02	4.21E-02	1.41E-01	2.68E-01	2.17E-02
	Peak sensitivity value	9.89E-01	6.24E-03	5.46E-05	3.71E-04	3.95E-04	6.34E-05	1.62E-03	8.03E-04	-7.68E-05	3.95E-19	2.18E-04
	Shapiro-Wilk score	7.371E-01	8.933E-01	8.290E-01	8.067E-01	6.308E-01	7.272E-01	6.460E-01	6.501E-01	5.603E-01	6.104E-01	7.358E-01
(hexokinase)	Local	9.79E-01	6.97E-03	7.82E-05	4.95E-04	1.13E-03	1.79E-04	4.63E-03	2.72E-03	-3.97E-04	-1.47E-18	3.73E-04
	Min	3.28E-01	3.79E-03	2.56E-05	1.57E-04	5.99E-05	8.06E-06	2.22E-04	1.43E-04	-7.20E-02	-3.37E-16	8.90E-05
	Max	9.95E-01	2.63E-02	9.83E-04	7.39E-03	9.02E-02	7.81E-03	3.20E-01	1.61E-01	6.61E-05	3.60E-16	9.14E-03
	Normalized peak height	2.89E-02	6.26E-03	1.40E-02	1.66E-02	6.08E-02	3.14E-02	5.08E-02	4.33E-02	1.83E-01	2.57E-01	2.34E-02
	Peak sensitivity value	9.88E-01	6.31E-03	6.05E-05	3.78E-04	3.75E-04	5.87E-05	1.66E-03	8.68E-04	-4.20E-05	3.36E-19	2.20E-04
	Shapiro-Wilk score	7.243E-01	8.892E-01	8.193E-01	7.945E-01	6.170E-01	7.119E-01	6.291E-01	6.360E-01	5.463E-01	5.993E-01	7.291E-01
(phosphoglycerate isomerase)	Local	9.79E-01	6.97E-03	7.82E-05	4.95E-04	1.13E-03	1.79E-04	4.63E-03	2.72E-03	-3.97E-04	-1.47E-18	3.73E-04
	Min	2.00E-01	3.78E-03	2.58E-05	1.56E-04	4.46E-05	5.01E-06	1.42E-04	8.24E-05	-8.97E-02	-4.91E-16	7.45E-05
	Max	9.95E-01	2.47E-02	9.88E-04	7.90E-03	1.06E-01	9.01E-03	3.85E-01	2.26E-01	8.53E-05	7.38E-16	1.29E-02
	Normalized peak height	3.40E-02	5.73E-03	1.43E-02	1.79E-02	6.97E-02	3.58E-02	6.10E-02	6.12E-02	2.13E-01	3.37E-01	3.24E-02
	Peak sensitivity value	9.89E-01	6.09E-03	6.09E-05	4.08E-04	3.09E-04	5.45E-05	1.49E-03	8.72E-04	-4.94E-05	-8.38E-21	2.48E-04
	Shapiro-Wilk score	7.283E-01	8.923E-01	8.243E-01	7.985E-01	6.187E-01	7.152E-01	6.328E-01	6.410E-01	5.488E-01	5.829E-01	7.294E-01
(phophofructokinase)	Local	9.79E-01	6.97E-03	7.82E-05	4.95E-04	1.13E-03	1.79E-04	4.63E-03	2.72E-03	-3.97E-04	-1.47E-18	3.73E-04
	Min	5.28E-01	3.88E-03	2.45E-05	1.58E-04	5.61E-05	6.31E-06	1.89E-04	1.47E-04	-5.11E-02	-4.12E-16	8.68E-05
	Max	9.95E-01	2.52E-02	7.60E-04	5.73E-03	6.46E-02	6.12E-03	2.29E-01	1.31E-01	9.14E-05	4.96E-16	8.05E-03
	Normalized peak height	2.02E-02	5.95E-03	1.09E-02	1.29E-02	4.38E-02	2.48E-02	3.67E-02	3.58E-02	1.46E-01	2.97E-01	2.04E-02
	Peak sensitivity value	9.89E-01	6.43E-03	5.65E-05	3.89E-04	3.46E-04	6.43E-05	1.45E-03	8.66E-04	-3.66E-05	-1.83E-19	2.26E-04
	Shapiro-Wilk score	7.322E-01	8.915E-01	8.239E-01	8.005E-01	6.258E-01	7.215E-01	6.384E-01	6.452E-01	5.540E-01	5.994E-01	7.361E-01
(aldolase)	Local	9.79E-01	6.97E-03	7.82E-05	4.95E-04	1.13E-03	1.79E-04	4.63E-03	2.72E-03	-3.97E-04	1.48E-16	3.73E-04
	Min	4.19E-01	3.80E-03	2.25E-05	1.39E-04	6.09E-05	7.45E-06	1.86E-04	1.10E-04	-6.37E-02	-1.38E-15	8.09E-05
	Max	9.95E-01	2.56E-02	8.94E-04	6.37E-03	7.38E-02	6.15E-03	2.75E-01	1.56E-01	6.48E-05	1.26E-15	8.61E-03
	Normalized peak height	2.42E-02	6.02E-03	1.25E-02	1.45E-02	4.84E-02	2.41E-02	4.30E-02	4.19E-02	1.50E-01	1.30E-01	2.13E-02
	Peak sensitivity value	9.90E-01	6.17E-03	6.04E-05	3.86E-04	3.19E-04	5.96E-05	1.42E-03	8.12E-04	-3.08E-05	-4.27E-19	2.56E-04
	Shapiro-Wilk score	7.279E-01	8.900E-01	8.203E-01	7.956E-01	6.195E-01	7.183E-01	6.344E-01	6.402E-01	5.489E-01	9.748E-01	7.322E-01
(triosephosphate isomerase)	Local	4.14E-01	2.95E-03	2.25E-04	1.71E-03	3.00E-02	1.02E-02	1.80E-01	1.06E-01	9.60E-02	8.65E-17	1.45E-02
	Min	-6.23E-01	-1.44E-02	3.36E-05	2.22E-04	6.18E-04	1.04E-03	1.07E-02	6.45E-03	1.55E-02	-1.03E-15	4.37E-03
	Max	9.43E-01	6.44E-03	1.14E-03	8.93E-03	1.77E-01	3.48E-02	8.37E-01	4.64E-01	2.01E-01	1.04E-15	3.57E-02
	Normalized peak height	2.05E-03	6.05E-03	4.35E-03	4.18E-03	4.14E-03	2.69E-03	3.38E-03	3.25E-03	1.98E-03	1.08E-01	3.01E-03
	Peak sensitivity value	6.53E-01	3.76E-03	1.17E-04	7.58E-04	1.23E-02	7.23E-03	7.55E-02	4.79E-02	1.13E-01	3.07E-19	1.44E-02
	Shapiro-Wilk score	9.677E-01	8.989E-01	9.232E-01	9.218E-01	9.206E-01	9.663E-01	9.328E-01	9.385E-01	9.822E-01	9.508E-01	9.885E-01
(glyceraldehyde3phosphate dehydrogenase)	Local	7.06E-01	5.02E-03	1.49E-04	1.08E-03	1.51E-02	5.04E-03	8.95E-02	5.25E-02	4.62E-02	1.18E-16	7.20E-03
	Min	1.32E-01	2.84E-03	3.09E-05	1.92E-04	2.65E-04	4.89E-04	5.59E-03	3.20E-03	7.88E-03	-7.97E-16	2.25E-03
	Max	9.70E-01	1.00E-02	8.68E-04	6.38E-03	8.73E-02	1.62E-02	4.16E-01	2.39E-01	8.84E-02	8.60E-16	1.69E-02
	Normalized peak height	2.32E-03	4.92E-03	5.19E-03	5.06E-03	4.09E-03	2.56E-03	3.52E-03	3.37E-03	2.09E-03	1.40E-01	2.87E-03
	Peak sensitivity value	8.29E-01	4.92E-03	8.15E-05	5.79E-04	6.75E-03	3.27E-03	4.48E-02	2.46E-02	5.25E-02	6.68E-19	7.50E-03
	Shapiro-Wilk score	9.688E-01	9.874E-01	9.166E-01	9.145E-01	9.165E-01	9.681E-01	9.317E-01	9.373E-01	9.815E-01	9.650E-01	9.894E-01
(glycerol3phosphate dehydrogenase)	Local	7.06E-01	5.02E-03	1.49E-04	1.08E-03	1.51E-02	5.04E-03	8.95E-02	5.25E-02	4.62E-02	1.18E-16	7.20E-03
	Min	1.33E-01	2.65E-03	2.94E-05	1.94E-04	3.65E-04	5.56E-04	6.04E-03	4.00E-03	8.04E-03	-7.95E-16	2.18E-03
	Max	9.64E-01	1.09E-02	7.56E-04	6.11E-03	9.14E-02	1.61E-02	4.52E-01	2.47E-01	8.83E-02	7.38E-16	1.74E-02
	Normalized peak height	2.25E-03	5.61E-03	4.50E-03	4.74E-03	4.26E-03	2.43E-03	3.65E-03	3.46E-03	2.12E-03	1.40E-01	3.05E-03
	Peak sensitivity value	8.32E-01	5.08E-03	8.93E-05	5.81E-04	6.15E-03	2.84E-03	3.97E-02	2.40E-02	5.60E-02	-5.64E-19	7.65E-03
	Shapiro-Wilk score	9.697E-01	9.866E-01	9.186E-01	9.166E-01	9.178E-01	9.688E-01	9.330E-01	9.388E-01	9.817E-01	9.648E-01	9.895E-01
(glycerol3phosphate oxidase)	Local	4.14E-01	2.95E-03	2.25E-04	1.71E-03	3.00E-02	1.02E-02	1.80E-01	1.06E-01	9.60E-02	8.65E-17	1.45E-02
	Min	-6.08E-01	-1.76E-02	3.23E-05	2.20E-04	5.44E-04	9.54E-04	1.01E-02	6.35E-03	1.70E-02	-9.75E-16	4.60E-03
	Max	9.44E-01	6.60E-03	1.29E-03	9.71E-03	1.62E-01	3.31E-02	8.13E-01	4.58E-01	2.05E-01	1.05E-15	3.32E-02
	Normalized peak height	2.11E-03	6.98E-03	4.86E-03	4.52E-03	3.85E-03	2.50E-03	3.23E-03	3.21E-03	1.99E-03	9.15E-02	2.79E-03
	Peak sensitivity value	6.79E-01	3.88E-03	1.03E-04	7.94E-04	1.24E-02	7.81E-03	9.65E-02	5.27E-02	9.25E-02	8.16E-19	1.38E-02
	Shapiro-Wilk score	9.670E-01	8.961E-01	9.206E-01	9.197E-01	9.191E-01	9.655E-01	9.310E-01	9.369E-01	9.820E-01	9.518E-01	9.883E-01
(pyruvate transport)	Local	7.06E-01	5.02E-03	1.49E-04	1.08E-03	1.51E-02	5.04E-03	8.95E-02	5.25E-02	4.62E-02	-2.22E-16	7.20E-03
	Min	1.62E-01	2.87E-03	2.97E-05	2.02E-04	1.01E-04	3.73E-04	3.70E-03	2.38E-03	6.63E-03	-8.88E-16	2.25E-03
	Max	9.76E-01	1.02E-02	7.71E-04	5.93E-03	9.41E-02	1.58E-02	4.17E-01	2.48E-01	8.77E-02	8.88E-16	1.76E-02
	Normalized peak height	2.13E-03	5.10E-03	4.63E-03	4.63E-03	4.27E-03	2.41E-03	3.41E-03	3.48E-03	2.15E-03	3.45E-01	3.18E-03
	Peak sensitivity value	8.28E-01	4.92E-03	7.61E-05	6.46E-04	6.44E-03	2.85E-03	4.57E-02	3.05E-02	5.72E-02	8.88E-19	7.60E-03
	Shapiro-Wilk score	9.700E-01	9.872E-01	9.176E-01	9.158E-01	9.169E-01	9.692E-01	9.325E-01	9.382E-01	9.817E-01	9.320E-01	9.895E-01

		(pyruvate kinase)	(atputilisation)	(glycerol kinase)	(phosphoglycerate mutase)	(enolase)	(adenylate kinase cytosol)	(adenylate kinase glycosome)	(3phosphoglycerate transport)	(gly3p dhap antiporter)	(glycerol transport)
(glucose transport)	Local	4.31E-04	8.87E-05	3.95E-04	3.65E-03	6.87E-04	-6.78E-21	1.71E-21	1.07E-08	-4.55E-09	6.60E-06
	Min	3.48E-05	7.16E-06	3.49E-06	1.77E-04	4.78E-05	-7.97E-20	-7.83E-19	1.56E-09	-1.05E-06	5.95E-08
	Max	5.17E-03	1.06E-03	4.90E-02	6.83E-02	8.32E-03	3.01E-18	1.37E-17	1.19E-07	5.66E-10	3.33E-04
	Normalized peak height	9.18E-03	9.23E-03	1.01E-01	1.31E-02	9.05E-03	4.91E-01	3.98E-01	1.05E-02	1.75E-01	4.15E-02
	Peak sensitivity value	2.27E-04	4.75E-05	7.70E-05	1.57E-03	3.50E-04	-9.90E-22	5.74E-21	7.35E-09	-1.00E-09	8.91E-07
	Shapiro-Wilk score	8.617E-01	8.598E-01	5.861E-01	7.705E-01	8.606E-01	2.203E-01	1.367E-01	8.715E-01	4.934E-01	6.874E-01
(hexokinase)	Local	4.31E-04	8.87E-05	3.95E-04	3.65E-03	6.87E-04	-6.78E-21	1.71E-21	1.07E-08	-4.55E-09	6.60E-06
	Min	4.17E-05	9.49E-06	3.39E-06	2.40E-04	6.34E-05	-7.13E-20	-3.62E-18	1.94E-09	-1.42E-06	6.82E-08
	Max	5.44E-03	1.13E-03	4.90E-02	7.62E-02	8.69E-03	3.09E-19	2.38E-17	1.05E-07	4.32E-10	3.01E-04
	Normalized peak height	9.68E-03	9.55E-03	1.02E-01	1.53E-02	9.64E-03	4.06E-01	5.43E-01	8.98E-03	1.95E-01	3.83E-02
	Peak sensitivity value	2.01E-04	4.81E-05	7.69E-05	1.49E-03	3.61E-04	-4.95E-23	-9.38E-21	7.84E-09	-2.76E-10	1.12E-06
	Shapiro-Wilk score	8.584E-01	8.566E-01	5.712E-01	7.687E-01	8.581E-01	8.367E-01	8.679E-02	8.675E-01	4.780E-01	6.768E-01
(phosphoglycerate isomerase)	Local	4.31E-04	8.87E-05	3.95E-04	3.65E-03	6.87E-04	-6.78E-21	1.71E-21	1.07E-08	-4.55E-09	6.60E-06
	Min	3.38E-05	6.83E-06	2.93E-06	1.64E-04	4.63E-05	-8.60E-20	-6.66E-19	1.54E-09	-1.57E-06	5.59E-08
	Max	6.38E-03	1.50E-03	6.51E-02	7.83E-02	1.09E-02	5.57E-19	1.86E-17	1.43E-07	5.78E-10	4.08E-04
	Normalized peak height	1.14E-02	1.28E-02	1.21E-01	1.55E-02	1.18E-02	4.02E-01	5.23E-01	1.25E-02	1.96E-01	5.10E-02
	Peak sensitivity value	2.47E-04	4.93E-05	1.01E-04	1.45E-03	3.78E-04	-1.04E-22	-1.91E-21	7.55E-09	-2.08E-10	1.08E-06
	Shapiro-Wilk score	8.604E-01	8.585E-01	5.737E-01	7.714E-01	8.600E-01	7.523E-01	9.865E-02	8.694E-01	4.823E-01	6.811E-01
(phophofructokinase)	Local	4.31E-04	8.87E-05	3.95E-04	3.65E-03	6.87E-04	-6.78E-21	1.71E-21	1.07E-08	-4.55E-09	6.60E-06
	Min	4.20E-05	8.92E-06	3.65E-06	2.13E-04	6.05E-05	-8.39E-20	-4.14E-19	1.85E-09	-8.41E-07	7.52E-08
	Max	5.67E-03	1.09E-03	3.83E-02	7.07E-02	7.88E-03	2.15E-19	1.84E-17	1.01E-07	5.74E-10	3.12E-04
	Normalized peak height	9.78E-03	9.34E-03	8.58E-02	1.41E-02	8.43E-03	4.04E-01	4.06E-01	8.70E-03	1.78E-01	4.06E-02
	Peak sensitivity value	2.42E-04	5.48E-05	6.11E-05	1.52E-03	2.99E-04	3.60E-23	8.56E-21	8.27E-09	-6.89E-10	8.56E-07
	Shapiro-Wilk score	8.613E-01	8.603E-01	5.786E-01	7.712E-01	8.617E-01	8.390E-01	1.294E-01	8.715E-01	4.866E-01	6.810E-01
(aldolase)	Local	4.31E-04	8.87E-05	3.95E-04	3.65E-03	6.87E-04	-6.78E-21	1.71E-21	1.07E-08	-4.55E-09	6.60E-06
	Min	4.12E-05	8.44E-06	3.94E-06	2.00E-04	5.75E-05	-7.30E-20	-2.34E-18	1.88E-09	-1.10E-06	6.50E-08
	Max	5.21E-03	1.03E-03	4.59E-02	6.36E-02	7.90E-03	5.90E-19	5.10E-17	1.11E-07	4.60E-10	3.39E-04
	Normalized peak height	9.19E-03	8.58E-03	9.51E-02	1.23E-02	8.70E-03	4.05E-01	6.85E-01	9.74E-03	1.61E-01	4.25E-02
	Peak sensitivity value	2.30E-04	5.92E-05	7.27E-05	1.69E-03	3.99E-04	3.10E-22	-1.82E-20	7.85E-09	-1.19E-09	9.12E-07
	Shapiro-Wilk score	8.589E-01	8.570E-01	5.754E-01	7.702E-01	8.592E-01	7.800E-01	5.979E-02	8.692E-01	4.829E-01	6.785E-01
(triosephosphate isomerase)	Local	1.68E-02	3.45E-03	-4.42E-02	1.42E-01	2.67E-02	-2.64E-19	1.27E-19	4.16E-07	1.10E-06	-7.39E-04
	Min	2.22E-03	4.50E-04	-1.12E-01	1.26E-02	3.09E-03	-8.37E-19	-7.82E-18	1.03E-07	2.37E-07	-1.45E-03
	Max	3.24E-02	6.36E-03	-7.88E-03	6.12E-01	5.25E-02	1.45E-17	3.23E-16	9.26E-07	3.39E-06	-1.41E-04
	Normalized peak height	2.80E-03	2.81E-03	2.76E-03	3.83E-03	2.55E-03	4.03E-01	1.65E-01	3.23E-03	3.09E-03	2.96E-03
	Peak sensitivity value	1.83E-02	3.62E-03	-5.23E-02	1.21E-01	2.98E-02	1.03E-21	-5.42E-20	3.49E-07	9.58E-07	-7.50E-04
	Shapiro-Wilk score	9.857E-01	9.849E-01	9.902E-01	9.358E-01	9.866E-01	8.048E-01	2.554E-01	9.852E-01	9.828E-01	9.965E-01
(glyceraldehyde3phosphate dehydrogenase)	Local	8.34E-03	1.71E-03	-2.12E-02	7.05E-02	1.33E-02	-1.31E-19	6.21E-20	2.07E-07	5.29E-07	-3.54E-04
	Min	1.06E-03	2.38E-04	-3.87E-02	6.71E-03	1.63E-03	-4.87E-19	-4.13E-18	4.92E-08	1.16E-07	-6.37E-04
	Max	1.48E-02	3.00E-03	-4.35E-03	2.74E-01	2.47E-02	3.73E-18	1.61E-16	4.46E-07	1.20E-06	-7.92E-05
	Normalized peak height	2.58E-03	2.79E-03	2.64E-03	3.50E-03	2.52E-03	4.01E-01	1.72E-01	3.24E-03	2.43E-03	3.16E-03
	Peak sensitivity value	9.23E-03	1.88E-03	-2.53E-02	5.76E-02	1.40E-02	1.63E-22	-8.09E-20	1.82E-07	5.55E-07	-3.40E-04
	Shapiro-Wilk score	9.837E-01	9.819E-01	9.810E-01	9.371E-01	9.853E-01	8.879E-01	2.168E-01	9.858E-01	9.895E-01	9.973E-01
(glycerol3phosphate dehydrogenase)	Local	8.34E-03	1.71E-03	-2.12E-02	7.05E-02	1.33E-02	-1.31E-19	6.21E-20	2.07E-07	5.29E-07	-3.54E-04
	Min	1.26E-03	2.82E-04	-3.86E-02	7.21E-03	1.92E-03	-4.01E-19	-4.46E-18	4.31E-08	1.20E-07	-6.88E-04
	Max	1.49E-02	3.01E-03	-4.28E-03	2.96E-01	2.44E-02	8.70E-18	3.16E-16	4.63E-07	1.22E-06	-8.24E-05
	Normalized peak height	2.67E-03	2.74E-03	2.49E-03	3.79E-03	2.42E-03	4.04E-01	2.71E-01	3.33E-03	2.46E-03	3.35E-03
	Peak sensitivity value	9.33E-03	1.85E-03	-2.58E-02	6.08E-02	1.43E-02	3.81E-21	-1.38E-19	1.80E-07	4.60E-07	-3.32E-04
	Shapiro-Wilk score	9.832E-01	9.819E-01	9.812E-01	9.380E-01	9.851E-01	7.676E-01	1.607E-01	9.862E-01	9.896E-01	9.974E-01
(glycerol3phosphate oxidase)	Local	1.68E-02	3.45E-03	-4.42E-02	1.42E-01	2.67E-02	-2.64E-19	1.27E-19	4.16E-07	1.10E-06	-7.39E-04
	Min	2.04E-03	4.59E-04	-1.10E-01	1.18E-02	3.18E-03	-8.53E-19	-8.24E-18	9.56E-08	2.35E-07	-1.58E-03
	Max	3.20E-02	6.42E-03	-8.91E-03	5.90E-01	5.18E-02	8.72E-18	6.89E-16	9.62E-07	3.32E-06	-1.42E-04
	Normalized peak height	2.63E-03	2.79E-03	2.68E-03	3.65E-03	2.53E-03	4.04E-01	2.91E-01	3.42E-03	3.00E-03	3.46E-03
	Peak sensitivity value	1.88E-02	3.77E-03	-4.93E-02	1.06E-01	3.05E-02	4.14E-21	-2.24E-19	3.52E-07	8.55E-07	-7.52E-04
	Shapiro-Wilk score	9.861E-01	9.851E-01	9.901E-01	9.363E-01	9.866E-01	8.781E-01	1.595E-01	9.854E-01	9.824E-01	9.963E-01
(pyruvate transport)	Local	8.34E-03	1.71E-03	-2.12E-02	7.05E-02	1.33E-02	-1.31E-19	6.21E-20	2.07E-07	5.29E-07	-3.54E-04
	Min	7.63E-04	1.70E-04	-3.83E-02	4.50E-03	1.16E-03	-4.28E-19	-3.94E-18	3.58E-08	1.25E-07	-6.49E-04
	Max	1.54E-02	3.18E-03	-3.49E-03	2.83E-01	2.46E-02	1.04E-17	2.31E-16	5.20E-07	1.22E-06	-7.42E-05
	Normalized peak height	2.79E-03	3.03E-03	2.45E-03	3.60E-03	2.53E-03	4.05E-01	2.20E-01	3.85E-03	2.47E-03	3.15E-03
	Peak sensitivity value	9.48E-03	1.86E-03	-2.56E-02	5.82E-02	1.46E-02	-1.98E-21	-6.24E-20	1.82E-07	5.47E-07	-3.40E-04
	Shapiro-Wilk score	9.833E-01	9.815E-01	9.814E-01	9.371E-01	9.850E-01	7.738E-01	2.051E-01	9.859E-01	9.897E-01	9.971E-01

(phosphoglycerate kinase)	Local	7.06E-01	5.02E-03	1.49E-04	1.08E-03	1.51E-02	5.04E-03	8.95E-02	5.25E-02	4.62E-02	2.34E-17	7.20E-03	8.34E-03	1.71E-03	-2.12E-02	7.05E-02	1.33E-02	-1.31E-19	6.21E-20	2.07E-07	5.29E-07	-3.54E-04
	Min	1.26E-01	2.59E-03	2.75E-05	1.85E-04	4.18E-04	4.98E-04	5.82E-03	3.53E-03	8.24E-03	-6.42E-16	2.28E-03	1.16E-03	2.35E-04	-3.84E-02	5.97E-03	1.75E-03	-3.85E-19	-4.04E-18	4.61E-08	1.17E-07	-6.41E-04
	Max	9.67E-01	1.06E-02	8.47E-04	6.45E-03	9.34E-02	1.56E-02	4.25E-01	2.62E-01	8.71E-02	6.66E-16	1.72E-02	1.49E-02	3.10E-03	-4.40E-03	2.94E-01	2.43E-02	1.45E-17	4.56E-16	4.63E-07	1.18E-06	-7.68E-05
	Normalized peak height	2.31E-03	5.56E-03	4.89E-03	5.13E-03	4.35E-03	2.38E-03	3.54E-03	3.61E-03	2.10E-03	6.26E-02	3.00E-03	2.62E-03	2.77E-03	2.42E-03	3.69E-03	2.44E-03	4.05E-01	3.66E-01	3.33E-03	2.49E-03	3.11E-03
	Peak sensitivity value	8.20E-01	5.04E-03	8.45E-05	6.01E-04	5.67E-03	3.39E-03	4.54E-02	2.41E-02	5.57E-02	-3.50E-19	7.19E-03	9.13E-03	1.95E-03	-2.54E-02	6.28E-02	1.41E-02	-6.40E-21	-1.37E-19	1.64E-07	5.88E-07	-3.40E-04
	Shapiro-Wilk score	9.700E-01	9.862E-01	9.190E-01	9.168E-01	9.181E-01	9.694E-01	9.334E-01	9.393E-01	9.816E-01	9.691E-01	9.895E-01	9.834E-01	9.816E-01	9.812E-01	9.378E-01	9.850E-01	7.182E-01	1.400E-01	9.861E-01	9.897E-01	9.971E-01
(pyruvate kinase)	Local	7.06E-01	5.02E-03	1.49E-04	1.08E-03	1.51E-02	5.04E-03	8.95E-02	5.25E-02	4.62E-02	-2.12E-16	7.20E-03	8.34E-03	1.71E-03	-2.12E-02	7.05E-02	1.33E-02	-1.31E-19	6.21E-20	2.07E-07	5.29E-07	-3.54E-04
	Min	9.15E-02	2.74E-03	2.90E-05	2.00E-04	2.90E-04	4.90E-04	5.31E-03	3.63E-03	8.61E-03	-9.69E-16	2.29E-03	1.25E-03	2.68E-04	-3.87E-02	7.06E-03	1.77E-03	-4.49E-19	-3.74E-18	5.20E-08	1.19E-07	-6.54E-04
	Max	9.68E-01	1.04E-02	7.74E-04	5.66E-03	9.34E-02	1.57E-02	4.26E-01	2.52E-01	8.93E-02	8.43E-16	1.65E-02	1.50E-02	2.99E-03	-4.56E-03	2.75E-01	2.50E-02	3.86E-18	1.74E-16	4.91E-07	1.27E-06	-8.21E-05
	Normalized peak height	2.26E-03	5.11E-03	4.52E-03	4.50E-03	4.36E-03	2.44E-03	3.45E-03	3.53E-03	2.13E-03	1.71E-01	2.90E-03	2.60E-03	2.69E-03	2.40E-03	3.59E-03	2.50E-03	4.07E-01	1.82E-01	3.59E-03	2.60E-03	3.08E-03
	Peak sensitivity value	8.16E-01	4.96E-03	8.83E-05	6.13E-04	6.11E-03	2.84E-03	4.63E-02	2.31E-02	5.78E-02	-3.91E-19	6.94E-03	9.00E-03	1.91E-03	-2.54E-02	5.62E-02	1.48E-02	1.74E-21	8.79E-20	1.67E-07	5.36E-07	-3.47E-04
	Shapiro-Wilk score	9.692E-01	9.874E-01	9.176E-01	9.158E-01	9.171E-01	9.686E-01	9.324E-01	9.383E-01	9.815E-01	9.910E-01	9.893E-01	9.835E-01	9.820E-01	9.811E-01	9.370E-01	9.851E-01	8.967E-01	2.304E-01	9.858E-01	9.893E-01	9.971E-01
(atp utilisation)	Local	7.06E-01	5.02E-03	1.49E-04	1.08E-03	1.51E-02	5.04E-03	8.95E-02	5.25E-02	4.62E-02	2.20E-21	7.20E-03	8.34E-03	1.71E-03	-2.12E-02	7.05E-02	1.33E-02	-1.31E-19	6.21E-20	2.07E-07	5.29E-07	-3.54E-04
	Min	1.35E-01	2.80E-03	2.98E-05	1.91E-04	4.25E-04	5.18E-04	5.76E-03	3.72E-03	7.52E-03	-2.99E-13	2.25E-03	1.12E-03	2.42E-04	-3.80E-02	5.79E-03	1.65E-03	-4.80E-19	-3.83E-18	4.99E-08	1.30E-07	-6.69E-04
	Max	9.70E-01	1.05E-02	8.14E-04	6.51E-03	9.29E-02	1.61E-02	4.26E-01	2.38E-01	8.76E-02	4.50E-13	1.73E-02	1.47E-02	3.04E-03	-4.05E-03	2.79E-01	2.45E-02	8.38E-18	2.46E-16	4.98E-07	1.24E-06	-8.09E-05
	Normalized peak height	2.14E-03	5.31E-03	4.83E-03	5.34E-03	4.22E-03	2.49E-03	3.55E-03	3.27E-03	2.06E-03	3.04E-01	3.06E-03	2.65E-03	2.77E-03	2.38E-03	3.80E-03	2.42E-03	4.06E-01	2.33E-01	3.62E-03	2.47E-03	3.22E-03
	Peak sensitivity value	8.57E-01	4.85E-03	8.04E-05	6.24E-04	7.32E-03	2.78E-03	4.42E-02	2.42E-02	5.51E-02	-3.32E-16	7.45E-03	8.77E-03	1.93E-03	-2.51E-02	5.79E-02	1.50E-02	3.06E-21	5.04E-20	1.70E-07	5.79E-07	-3.44E-04
	Shapiro-Wilk score	9.692E-01	9.870E-01	9.173E-01	9.151E-01	9.168E-01	9.686E-01	9.319E-01	9.379E-01	9.817E-01	4.945E-01	9.892E-01	9.837E-01	9.822E-01	9.815E-01	9.370E-01	9.852E-01	8.304E-01	1.805E-01	9.855E-01	9.895E-01	9.975E-01
(glycerol kinase)	Local	9.22E+00	6.57E-02	-2.07E-03	-1.73E-02	-4.20E-01	-1.47E-01	-2.56E+00	-1.50E+00	-1.41E+00	1.05E-15	-2.06E-01	-2.39E-01	-4.91E-02	6.53E-01	-2.02E+00	-3.80E-01	3.75E-18	-1.82E-18	-5.91E-06	-1.61E-05	1.09E-02
	Min	2.17E+00	2.61E-02	-5.67E-03	-4.76E-02	-9.73E-01	-2.79E-01	-5.68E+00	-3.48E+00	-2.19E+00	-1.98E-14	-5.49E-01	-3.52E-01	-7.56E-02	2.63E-01	-5.53E+00	-5.48E-01	-9.84E-18	-7.21E-17	-1.90E-05	-4.07E-05	2.27E-03
	Max	1.36E+01	1.62E-01	1.77E-03	1.16E-02	5.35E-02	-4.47E-02	-5.30E-01	-3.26E-01	-5.50E-01	3.48E-14	-1.21E-02	-6.19E-03	-1.33E-03	9.43E-01	-4.86E-02	-1.06E-02	1.21E-17	7.56E-17	-2.27E-07	-4.85E-06	2.54E-02
	Normalized peak height	5.66E-03	3.59E-03	4.92E-03	4.82E-03	4.10E-03	4.48E-03	4.36E-03	4.69E-03	2.69E-03	1.63E-01	3.45E-03	2.96E-03	3.08E-03	1.76E-03	4.79E-03	3.36E-03	4.18E-01	4.95E-02	2.76E-03	3.27E-03	2.44E-03
	Peak sensitivity value	8.85E+00	6.01E-02	-1.69E-03	-1.29E-02	-3.54E-01	-1.29E-01	-2.19E+00	-1.22E+00	-1.54E+00	8.98E-18	-1.52E-01	-2.58E-01	-5.64E-02	7.42E-01	-1.67E+00	-4.06E-01	-5.62E-21	5.66E-20	-4.12E-06	-1.45E-05	1.00E-02
	Shapiro-Wilk score	9.908E-01	9.786E-01	9.767E-01	9.760E-01	9.834E-01	9.977E-01	9.761E-01	9.785E-01	9.808E-01	9.713E-01	9.764E-01	9.576E-01	9.563E-01	9.684E-01	9.930E-01	9.507E-01	9.437E-01	9.967E-01	9.680E-01	9.798E-01	9.841E-01
(phosphoglycerate mutase)	Local	7.06E-01	5.02E-03	1.49E-04	1.08E-03	1.51E-02	5.04E-03	8.95E-02	5.25E-02	4.62E-02	2.10E-17	7.20E-03	8.34E-03	1.71E-03	-2.12E-02	7.05E-02	1.33E-02	-1.31E-19	6.21E-20	2.07E-07	5.29E-07	-3.54E-04
	Min	1.34E-01	2.76E-03	3.16E-05	2.07E-04	3.16E-04	5.58E-04	5.80E-03	3.27E-03	8.00E-03	-6.67E-16	2.42E-03	1.16E-03	2.58E-04	-3.86E-02	6.75E-03	1.74E-03	-4.57E-19	-4.36E-18	4.94E-08	1.17E-07	-6.46E-04
	Max	9.68E-01	1.00E-02	8.21E-04	6.32E-03	9.43E-02	1.60E-02	4.31E-01	2.49E-01	8.84E-02	7.57E-16	1.77E-02	1.50E-02	2.99E-03	-4.47E-03	2.87E-01	2.46E-02	3.43E-18	2.54E-16	4.78E-07	1.20E-06	-7.88E-05
	Normalized peak height	2.31E-03	5.02E-03	4.78E-03	4.98E-03	4.35E-03	2.46E-03	3.44E-03	3.53E-03	2.16E-03	1.42E-01	3.05E-03	2.75E-03	2.69E-03	2.45E-03	3.67E-03	2.49E-03	4.06E-01	2.33E-01	3.42E-03	2.44E-03	3.12E-03
	Peak sensitivity value	8.63E-01	5.05E-03	8.81E-05	6.08E-04	5.91E-03	3.17E-03	3.83E-02	2.53E-02	5.33E-02	-1.28E-19	7.47E-03	9.06E-03	1.81E-03	-2.46E-02	6.18E-02	1.46E-02	3.07E-22	-1.00E-19	1.83E-07	5.15E-07	-3.42E-04
	Shapiro-Wilk score	9.696E-01	9.866E-01	9.175E-01	9.152E-01	9.169E-01	9.688E-01	9.322E-01	9.379E-01	9.817E-01	9.645E-01	9.895E-01	9.837E-01	9.819E-01	9.811E-01	9.380E-01	9.852E-01	8.951E-01	1.662E-01	9.860E-01	9.896E-01	9.973E-01
(enolase)	Local	7.06E-01	5.02E-03	1.49E-04	1.08E-03	1.51E-02	5.04E-03	8.95E-02	5.25E-02	4.62E-02	2.10E-17	7.20E-03	8.34E-03	1.71E-03	-2.12E-02	7.05E-02	1.33E-02	-1.31E-19	6.21E-20	2.07E-07	5.29E-07	-3.54E-04
	Min	1.36E-01	2.75E-03	2.77E-05	1.85E-04	2.37E-04	5.17E-04	5.40E-03	3.18E-03	7.72E-03	-7.02E-16	2.24E-03	1.14E-03	2.44E-04	-3.79E-02	6.04E-03	1.72E-03	-4.47E-19	-5.50E-18	4.82E-08	1.01E-07	-6.40E-04
	Max	9.70E-01	1.06E-02	8.18E-04	5.86E-03	9.30E-02	1.17E-01	4.39E-01	2.62E-01	8.80E-02	7.33E-16	1.63E-02	1.48E-02	3.05E-03	-4.26E-03	3.05E-01	2.41E-02	3.99E-18	5.18E-16	5.20E-07	1.19E-06	-7.48E-05
	Normalized peak height	2.25E-03	5.45E-03	4.84E-03	4.64E-03	4.26E-03	1.70E-02	3.73E-03	3.76E-03	2.20E-03	1.42E-01	2.87E-03	2.59E-03	2.77E-03	2.42E-03	3.96E-03	2.42E-03	4.06E-01	4.26E-01	3.77E-03	2.54E-03	3.07E-03
	Peak sensitivity value	8.23E-01	4.81E-03	7.87E-05	5.51E-04	5.85E-03	3.13E-03	4.33E-02	2.53E-02	5.66E-02	5.27E-19	7.74E-03	8.97E-03	1.86E-03	-2.40E-02	6.33E-02	1.45E-02	-7.26E-22	-4.06E-21	1.67E-07	5.75E-07	-3.52E-04
	Shapiro-Wilk score	9.691E-01	9.871E-01	9.177E-01	9.156E-01	9.167E-01	9.610E-01	9.321E-01	9.378E-01	9.815E-01	9.645E-01	9.893E-01	9.832E-01	9.818E-01	9.810E-01	9.376E-01	9.851E-01	8.894E-01	1.406E-01	9.861E-01	9.894E-01	9.973E-01
(adenylate kinase cytosol)	Local	INF	INF	INF	INF	INF	INF	INF	INF	INF	INF	INF	INF	INF	INF	INF	INF	INF	INF	INF	INF	INF
	Min	-INF	-INF	-INF	-INF	-INF	-INF	-INF	-INF	-INF	-INF	-INF	-INF	-INF	-INF	-INF	-INF	-INF	-INF	-INF	-INF	-INF
	Max	INF	INF	INF	INF	INF	INF	INF	INF	INF	INF	INF	INF	INF	INF	INF	INF	INF	INF	INF	INF	INF
	Normalized peak height	7.14E-02	7.14E-02	7.14E-02	7.14E-02	7.14E-02	7.14E-02	7.14E-02	7.14E-02	7.14E-02	1.43E-01	7.14E-02	7.14E-02	7.14E-02	7.14E-02	1.43E-01	7.14E-02	1.43E-01	7.14E-02	7.14E-02	7.14E-02	7.14E-02
	Peak sensitivity value	-3.91E-02	-7.21E-02	-2.21E-02	-6.21E-02	-2.45E-03	-5.43E-04	-9.23E-03	-6.92E-03	-3.26E-03	5.45E-08	-1.06E-01	-1.12E-01	-1.05E-01	-2.03E-03	-3.01E-02	-4.31E-02	-1.53E-13	-5.58E-12	-5.51E-02	-4.26E-03	-3.38E-05
	Shapiro-Wilk score	9.109E-01	9.417E-01	9.150E-01	8.846E-01	9.446E-01	9.250E-01	9.696E-01	9.695E-01	9.703E-01	9.384E-01	8.796E-01	9.571E-01	9.297E-01	9.331E-01	9.794E-01	8.932E-01	9.627E-01	7.498E-01	9.599E-01	9.711E-01	9.734E-01
(adenylate kinase glycosome)	Local	-INF	-INF	-INF	-INF	-INF	-INF	-INF	-INF	-INF	-INF	-INF	-INF	-INF	-INF	-INF	-INF	-INF	-INF	-INF	-INF	-INF
	Min	-INF	-INF	-INF	-INF	-INF	-INF	-INF	-INF	-INF	-INF	-INF	-INF	-INF	-INF	-INF	-INF	-INF	-INF	-INF	-INF	-INF
	Max	INF	INF	INF	INF	INF	INF	INF	INF	INF	INF	INF	INF	INF	INF	INF	INF	INF	INF	INF	INF	INF
	Normalized peak height	6.35E-02	1.14E-02	1.12E-02	1.34E-02	6.52E-02	3.84E-02	5.64E-02	6.44E-02	1.32E-01	7.68E-02	1.23E-02	5.88E-02	6.52E-02	6.18E-02	6.08E-02	5.29E-02	4.42E-01	6.22E-03	1.35E-02	1.76E-02	6.86E-02
	Peak sensitivity value	1.40E-03	-7.97E-02	-4.71E-03	-1.18E-03	4.48E-03	-1.85E-04	-5.54E-03	1.03E-02	7.23E-04	-1.42E-08	-2.42E-02	-9.55E-04	-2.94E-04	1.93E-03	3.34E-03	5.12E-05	-3.28E-17	-8.47E-12	1.98E-03	3.57E-03	2.32E-05
	Shapiro-Wilk score	9.647E-01	9.565E-01	9.589E-01	9.585E-01	9.448E-01	9.504E-01	9.446E-01	9.482E-01	8.183E-01	9.482E-01	9.614E-01	9.732E-01	9.730E-01	8.936E-01	9.449E-01	9.685E-01	7.306E-01	9.942E-01	9.463E-01	9.072E-01	9.061E-01
(3phosphoglycerate transport)	Local	7.06E-01	5.02E-03	1.49E-04	1.08E-03	1.51E-02	5.04E-03	8.95E-02	5.25E-02	4.62E-02	2.34E-17	7.20E-03	8.34E-03	1.71E-03	-2.12E-02	7.05E-02	1.33E-02	-1.31E-19	6.20E-20	2.07E-07	5.29E-07	-3.54E-04
	Min	1.34E-01	2.71E-03	2.91E-05	1.89E-04	2.68E-04	3.90E-04	4.31E-03	2.45E-03	6.64E-03	-6.84E-16	2.18E-03	9.73E-04	2.21E-04	-3.82E-02	5.59E-03	1.46E-03	-4.75E-19	-4.21E-18	4.48E-08	1.11E-07	-6.38E-04
	Max	9.74E-01	1.00E-02	8.13E-04	6.33E-03	9.45E-02	1.61E-02	4.34E-01	2.41E-01	8.68E-02	6.29E-16	1.75E-02	1.48E-02	3.10E-03	-3.64E-03	2.81E-01	2.47E-02	4.85E-18	1.79E-16	5.18E-07	1.26E-06	-6.63E-05
	Normalized peak height	2.24E-03	5.07E-03	4.89E-03	5.06E-03	4.42E-03	2.45E-03	3.51E-03	3.37E-03	2.12E-03	6.31E-02	3.14E-03	2.54E-03	2.84E-03	2.43E-03	3.56E-03	2.58E-03	4.04E-01	1.84E-01	3.85E-03	2.55E-03	3.12E-03
	Peak sensitivity value	8.28E-01	4.95E-03	9.46E-05	5.54E-04	5.87E-03	2.95E-03	4.02E-02	2.88E-02	5.45E-02	-6.10E-19	6.81E-03	9.02E-03	1.96E-03	-2.46E-02	6.18E-02	1.45E-02	2.16E-21	8.72E-20	1.73E-07	4.94E-07	-3.45E-04
	Shapiro-Wilk score	9.696E-01	9.870E-01	9.183E-01	9.162E-01	9.173E-01	9.690E-01	9.327E-01	9.385E-01	9.819E-01	9.687E-01	9.897E-01	9.836E-01	9.819E-01	9.816E-01	9.370E-01	9.854E-01	8.777E-01	2.096E-01	9.864E-01	9.897E-01	9.972E-01
(gly3p dhap antiporter)	Local	4.14E-01	2.95E-03	2.25E-04	1.71E-03	3.00E-02	1.02E-02	1.80E-01	1.06E-01	9.60E-02	8.65E-17	1.45E-02	1.68E-02	3.45E-03	-4.42E-02	1.42E-01	2.67E-02	-2.64E-19	1.27E-19	4.16E-07	1.10E-06	-7.39E-04
	Min	-5.99E-01	-1.53E-02	3.16E-05	2.13E-04	5.03E-04	1.06E-03	1.15E-02	6.22E-03	1.56E-02	-9.48E-16	4.18E-03	2.15E-03	4.56E-04	-1.11E-01	1.15E-02	3.39E-03	-8.24E-19	-8.30E-18	9.20E-08	2.39E-07	-1.46E-03
	Max	9.43E-01	6.49E-03	1.16E-03	8.95E-03	1.64E-01	3.30E-02	8.08E-01	4.72E-01	2.08E-01	9.62E-16	3.40E-02	3.23E-02	6.67E-03	-8.71E-03	5.44E-01	5.06E-02	2.03E-17	8.66E-16	9.82E-07	3.36E-06	-1.56E-04
	Normalized peak height	2.02E-03	6.28E-03	4.29E-03	4.17E-03	3.83E-03	2.50E-03	3.20E-03	3.22E-03	1.93E-03	1.04E-01	2.93E-03	2.68E-03	2.83E-03	2.64E-03	3.34E-03	2.37E-03	4.08E-01	3.61E-01	3.44E-03	2.97E-03	2.98E-03
	Peak sensitivity value	6.13E-01	3.78E-03	1.06E-04	9.08E-04	1.01E-02	6.57E-03	8.52E-02	4.42E-02	1.14E-01	2.08E-19	1.58E-02	1.81E-02	3.74E-03	-4.63E-02	1.14E-01	3.11E-02	9.56E-21	7.78E-21	3.31E-07	9.99E-07	-7.61E-04
	Shapiro-Wilk score	9.673E-01	8.958E-01	9.219E-01	9.208E-01	9.197E-01	9.659E-01	9.316E-01	9.376E-01	9.825E-01	9.511E-01	9.887E-01	9.862E-01	9.852E-01	9.904E-01	9.368E-01	9.869E-01	8.141E-01	1.501E-01	9.854E-01	9.829E-01	9.966E-01
(glycerol transport)	Local	9.22E+00	6.57E-02	-2.07E-03	-1.73E-02	-4.20E-01	-1.47E-01	-2.56E+00	-1.50E+00	-1.41E+00	1.05E-15	-2.06E-01	-2.39E-01	-4.91E-02	6.53E-01	-2.02E+00	-3.80E-01	3.75E-18	-1.82E-18	-5.91E-06	-1.61E-05	1.09E-02
	Min	1.08E+00	2.38E-02	-6.39E-03	-5.10E-02	-1.05E+00	-2.57E-01	-5.44E+00	-3.29E+00	-2.32E+00	-2.10E-14	-5.67E-01	-3.58E-01	-7.46E-02	2.53E-01	-5.20E+00	-5.48E-01	-1.63E-16	-5.65E-15	-2.06E-05	-3.99E-05	2.38E-03
	Max	1.36E+01	1.78E-01	2.38E-03	1.68E-02	1.60E-01	-3.15E-02	2.71E-02	1.55E-02	-5.24E-01	2.06E-14	6.97E-04	3.54E-04	7.15E-05	9.45E-01	3.36E-03	4.97E-04	1.20E-17	8.08E-17	7.86E-09	-5.22E-06	2.51E-02
	Normalized peak height	5.94E-03	3.95E-03	5.63E-03	5.51E-03	4.71E-03	4.32E-03	4.46E-03	4.77E-03	2.82E-03	1.62E-01	3.28E-03	3.07E-03	3.03E-03	1.74E-03	4.44E-03	3.65E-03	4.06E-01	1.79E-01	2.88E-03	3.19E-03	2.25E-03
	Peak sensitivity value	8.66E+00	5.97E-02	-1.52E-03	-1.28E-02	-3.51E-01	-1.34E-01	-2.09E+00	-1.27E+00	-1.53E+00	-2.83E-18	-1.85E-01	-2.64E-01	-5.74E-02	7.51E-01	-1.73E+00	-4.09E-01	-3.51E-20	-2.27E-18	-2.99E-06	-1.52E-05	1.07E-02
	Shapiro-Wilk score	9.898E-01	9.774E-01	9.774E-01	9.767E-01	9.839E-01	9.979E-01	9.771E-01	9.785E-01	9.799E-01	9.727E-01	9.772E-01	9.569E-01	9.554E-01	9.687E-01	9.927E-01	9.490E-01	8.741E-01	3.327E-01	9.688E-01	9.805E-01	9.846E-01

Table S13: Flux control coefficients for *Trypanosoma brucei* model with parameter variation of $\pm 20\%$

	(glucose transport)	(hexokinase)	(phosphoglycerate isomerase)	(phophofructokinase)	(aldolase)	(triosephosphate isomerase)	(glyceraldehyde3phosphate dehydrogenase)	(glycerol3phosphate dehydrogenase)	(glycerol3phosphate oxidase)	(pyruvate transport)	(phosphoglycerate kinase)	(pyruvate kinase)	(atputilisation)	(glycerol kinase)	(phosphoglycerate mutase)	(enolase)	(adenylate kinase cytosol)	(adenylate kinase glycosome)	(3phosphoglycerate transport)	(gly3p dhap antiporter)	(glycerol transport)
(glucose transport)<br>Local<br>Min<br>Max<br>Normalized peak height<br>Peak sensitivity value<br>Shapiro-Wilk score	9.79E-01<br>-1.60E-01<br>9.97E-01<br>5.95E-02<br>9.93E-01<br>5.232E-01	6.97E-03<br>-4.37E-02<br>4.14E-02<br>1.39E-02<br>5.16E-03<br>8.522E-01	7.82E-05<br>9.08E-06<br>2.61E-03<br>2.75E-02<br>3.90E-05<br>6.366E-01	4.95E-04<br>5.44E-05<br>2.03E-02<br>3.36E-02<br>2.47E-04<br>5.862E-01	1.13E-03<br>1.05E-05<br>2.03E-01<br>2.09E-01<br>1.12E-04<br>4.572E-01	1.79E-04<br>-3.81E-03<br>2.08E-02<br>1.64E-01<br>1.69E-05<br>4.656E-01	4.63E-03<br>1.39E-05<br>6.80E-01<br>1.78E-01<br>3.54E-04<br>4.446E-01	2.72E-03<br>1.05E-05<br>4.09E-01<br>1.80E-01<br>2.15E-04<br>4.484E-01	-3.97E-04<br>-1.75E-01<br>3.93E-03<br>2.75E-01<br>-1.07E-04<br>4.754E-01	-1.47E-18<br>-3.44E-14<br>3.44E-14<br>8.61E-01<br>2.94E-17<br>6.037E-02	3.73E-04<br>2.97E-05<br>4.60E-02<br>1.09E-01<br>1.45E-04<br>4.625E-01	4.31E-04<br>4.25E-06<br>2.69E-02<br>5.50E-02<br>7.16E-05<br>5.554E-01	8.87E-05<br>8.11E-07<br>5.25E-03<br>4.96E-02<br>1.39E-05<br>5.539E-01	3.95E-04<br>1.72E-07<br>1.38E-01<br>3.62E-01<br>6.92E-05<br>4.730E-01	3.65E-03<br>1.45E-05<br>9.52E-01<br>2.46E-01<br>4.91E-04<br>3.293E-01	6.87E-04<br>4.88E-06<br>3.98E-02<br>5.34E-02<br>1.04E-04<br>5.539E-01	-6.78E-21<br>-4.76E-19<br>5.66E-19<br>4.08E-01<br>-4.85E-22<br>4.968E-01	1.71E-21<br>-8.50E-16<br>1.69E-17<br>9.52E-01<br>3.94E-20<br>1.344E-02	1.07E-08<br>4.02E-10<br>3.17E-07<br>2.46E-02<br>3.10E-09<br>6.022E-01	-4.55E-09<br>-4.10E-06<br>1.07E-08<br>3.85E-01<br>4.59E-10<br>4.340E-01	6.60E-06<br>3.52E-09<br>8.43E-04<br>2.40E-01<br>4.25E-07<br>6.111E-01
(hexokinase)<br>Local<br>Min<br>Max<br>Normalized peak height<br>Peak sensitivity value<br>Shapiro-Wilk score	9.79E-01<br>-1.56E-01<br>9.97E-01<br>6.03E-02<br>9.93E-01<br>5.236E-01	6.97E-03<br>-4.40E-02<br>4.06E-02<br>1.40E-02<br>5.11E-03<br>8.482E-01	7.82E-05<br>8.80E-06<br>2.53E-03<br>2.74E-02<br>3.78E-05<br>6.380E-01	4.95E-04<br>5.36E-05<br>2.19E-02<br>3.58E-02<br>2.83E-04<br>5.865E-01	1.13E-03<br>1.06E-05<br>2.14E-01<br>2.18E-01<br>1.18E-04<br>4.578E-01	1.79E-04<br>-4.21E-03<br>2.11E-02<br>1.68E-01<br>1.44E-05<br>4.657E-01	4.63E-03<br>2.01E-05<br>7.07E-01<br>1.87E-01<br>3.74E-04<br>4.452E-01	2.72E-03<br>9.72E-06<br>4.24E-01<br>1.86E-01<br>2.22E-04<br>4.477E-01	-3.97E-04<br>-1.80E-01<br>2.91E-03<br>2.98E-01<br>-1.05E-04<br>4.759E-01	-1.47E-18<br>-1.69E-14<br>2.06E-14<br>8.80E-01<br>-6.97E-18<br>7.001E-02	3.73E-04<br>3.00E-05<br>5.32E-02<br>1.20E-01<br>1.63E-04<br>4.650E-01	4.31E-04<br>4.80E-06<br>2.32E-02<br>4.75E-02<br>6.28E-05<br>5.594E-01	8.87E-05<br>1.00E-06<br>5.05E-03<br>4.85E-02<br>1.36E-05<br>5.557E-01	3.95E-04<br>1.29E-07<br>1.49E-01<br>3.70E-01<br>7.46E-05<br>4.731E-01	3.65E-03<br>1.73E-05<br>9.88E-01<br>2.54E-01<br>5.11E-04<br>3.292E-01	6.87E-04<br>5.84E-06<br>4.17E-02<br>5.65E-02<br>1.10E-04<br>5.568E-01	-6.78E-21<br>-5.31E-19<br>1.88E-18<br>4.81E-01<br>-5.64E-22<br>4.423E-01	1.71E-21<br>-1.42E-15<br>1.74E-17<br>9.30E-01<br>-6.19E-19<br>9.175E-03	1.07E-08<br>3.74E-10<br>3.00E-07<br>2.34E-02<br>2.92E-09<br>6.032E-01	-4.55E-09<br>-4.04E-06<br>5.69E-09<br>4.23E-01<br>-3.73E-10<br>4.356E-01	6.60E-06<br>2.93E-09<br>8.93E-04<br>2.47E-01<br>4.49E-07<br>6.118E-01
(phosphoglycerate isomerase)<br>Local<br>Min<br>Max<br>Normalized peak height<br>Peak sensitivity value<br>Shapiro-Wilk score	9.79E-01<br>-1.52E-01<br>9.97E-01<br>6.01E-02<br>9.93E-01<br>5.212E-01	6.97E-03<br>-4.45E-02<br>3.84E-02<br>1.38E-02<br>5.58E-03<br>8.507E-01	7.82E-05<br>9.97E-06<br>2.76E-03<br>2.97E-02<br>3.88E-05<br>6.355E-01	4.95E-04<br>6.16E-05<br>2.21E-02<br>3.50E-02<br>2.71E-04<br>5.842E-01	1.13E-03<br>1.03E-05<br>2.05E-01<br>2.13E-01<br>1.13E-04<br>4.556E-01	1.79E-04<br>-4.23E-03<br>2.09E-02<br>1.30E-01<br>3.05E-05<br>4.652E-01	4.63E-03<br>2.02E-05<br>6.86E-01<br>1.83E-01<br>3.63E-04<br>4.445E-01	2.72E-03<br>1.26E-05<br>4.32E-01<br>1.91E-01<br>2.29E-04<br>4.472E-01	-3.97E-04<br>-1.75E-01<br>2.56E-03<br>3.61E-01<br>-1.79E-05<br>4.739E-01	-1.47E-18<br>-1.32E-14<br>4.46E-14<br>8.90E-01<br>1.84E-17<br>6.247E-02	3.73E-04<br>3.43E-05<br>4.68E-02<br>1.12E-01<br>1.51E-04<br>4.635E-01	4.31E-04<br>4.86E-06<br>2.29E-02<br>4.70E-02<br>6.22E-05<br>5.585E-01	8.87E-05<br>1.19E-06<br>4.70E-03<br>4.53E-02<br>1.29E-05<br>5.553E-01	3.95E-04<br>9.90E-08<br>1.30E-01<br>3.57E-01<br>6.50E-05<br>4.716E-01	3.65E-03<br>2.29E-05<br>1.02E+00<br>2.62E-01<br>5.34E-04<br>3.276E-01	6.87E-04<br>7.14E-06<br>3.67E-02<br>5.11E-02<br>9.88E-05<br>5.548E-01	-6.78E-21<br>-5.13E-19<br>5.49E-19<br>4.12E-01<br>4.67E-22<br>4.904E-01	1.71E-21<br>-1.45E-15<br>4.93E-17<br>6.57E-01<br>7.44E-19<br>8.131E-03	1.07E-08<br>4.13E-10<br>3.57E-07<br>2.73E-02<br>3.09E-09<br>6.043E-01	-4.55E-09<br>-4.68E-06<br>7.89E-09<br>3.79E-01<br>8.64E-10<br>4.320E-01	6.60E-06<br>1.72E-09<br>8.26E-04<br>2.39E-01<br>4.15E-07<br>6.105E-01
(phophofructokinase)<br>Local<br>Min<br>Max<br>Normalized peak height<br>Peak sensitivity value<br>Shapiro-Wilk score	9.79E-01<br>-1.72E-01<br>9.97E-01<br>6.16E-02<br>9.93E-01<br>5.199E-01	6.97E-03<br>-4.06E-02<br>4.01E-02<br>1.33E-02<br>5.19E-03<br>8.506E-01	7.82E-05<br>1.05E-05<br>2.57E-03<br>2.72E-02<br>4.24E-05<br>6.370E-01	4.95E-04<br>6.51E-05<br>2.26E-02<br>3.64E-02<br>2.79E-04<br>5.859E-01	1.13E-03<br>1.09E-05<br>2.02E-01<br>2.09E-01<br>1.12E-04<br>4.538E-01	1.79E-04<br>-4.11E-03<br>2.23E-02<br>1.58E-01<br>2.35E-05<br>4.649E-01	4.63E-03<br>1.78E-05<br>6.78E-01<br>1.79E-01<br>3.57E-04<br>4.426E-01	2.72E-03<br>1.08E-05<br>4.78E-01<br>2.04E-01<br>2.50E-04<br>4.450E-01	-3.97E-04<br>-1.78E-01<br>3.65E-03<br>3.89E-01<br>-8.20E-05<br>4.719E-01	-1.47E-18<br>-1.62E-14<br>1.76E-14<br>7.78E-01<br>1.57E-17<br>7.608E-02	3.73E-04<br>2.84E-05<br>4.26E-02<br>1.03E-01<br>1.35E-04<br>4.588E-01	4.31E-04<br>4.70E-06<br>2.64E-02<br>5.49E-02<br>7.06E-05<br>5.559E-01	8.87E-05<br>1.08E-06<br>4.66E-03<br>4.46E-02<br>1.74E-05<br>5.518E-01	3.95E-04<br>1.14E-07<br>1.36E-01<br>3.61E-01<br>6.83E-05<br>4.688E-01	3.65E-03<br>1.94E-05<br>9.76E-01<br>2.54E-01<br>5.08E-04<br>3.312E-01	6.87E-04<br>5.88E-06<br>3.75E-02<br>5.18E-02<br>9.96E-05<br>5.544E-01	-6.78E-21<br>-4.73E-19<br>4.71E-19<br>3.97E-01<br>2.71E-22<br>4.854E-01	1.71E-21<br>-1.98E-15<br>2.08E-17<br>9.67E-01<br>-2.14E-19<br>4.071E-03	1.07E-08<br>3.61E-10<br>3.16E-07<br>2.46E-02<br>3.68E-09<br>6.022E-01	-4.55E-09<br>-4.21E-06<br>1.16E-08<br>3.48E-01<br>1.04E-09<br>4.301E-01	6.60E-06<br>2.09E-09<br>8.33E-04<br>2.40E-01<br>4.19E-07<br>6.098E-01
(aldolase)<br>Local<br>Min<br>Max<br>Normalized peak height<br>Peak sensitivity value<br>Shapiro-Wilk score	9.79E-01<br>-1.40E-01<br>9.97E-01<br>5.82E-02<br>9.93E-01<br>5.220E-01	6.97E-03<br>-4.01E-02<br>4.16E-02<br>1.32E-02<br>4.90E-03<br>8.509E-01	7.82E-05<br>9.42E-06<br>2.49E-03<br>2.67E-02<br>3.54E-05<br>6.370E-01	4.95E-04<br>5.47E-05<br>2.12E-02<br>3.49E-02<br>2.77E-04<br>5.861E-01	1.13E-03<br>1.03E-05<br>2.17E-01<br>2.20E-01<br>1.19E-04<br>4.572E-01	1.79E-04<br>-3.95E-03<br>2.29E-02<br>1.39E-01<br>7.78E-06<br>4.661E-01	4.63E-03<br>1.71E-05<br>6.80E-01<br>1.79E-01<br>3.57E-04<br>4.450E-01	2.72E-03<br>1.06E-05<br>4.11E-01<br>1.80E-01<br>2.16E-04<br>4.474E-01	-3.97E-04<br>-1.80E-01<br>3.14E-03<br>3.81E-01<br>-5.65E-05<br>4.753E-01	1.48E-16<br>-1.79E-15<br>1.54E-15<br>2.53E-01<br>-1.02E-18<br>8.861E-01	3.73E-04<br>3.32E-05<br>4.78E-02<br>1.10E-01<br>1.53E-04<br>4.658E-01	4.31E-04<br>4.17E-06<br>2.24E-02<br>4.61E-02<br>6.02E-05<br>5.601E-01	8.87E-05<br>1.01E-06<br>4.64E-03<br>4.39E-02<br>1.26E-05<br>5.572E-01	3.95E-04<br>9.31E-08<br>1.39E-01<br>3.60E-01<br>6.95E-05<br>4.730E-01	3.65E-03<br>1.53E-05<br>9.65E-01<br>2.47E-01<br>4.98E-04<br>3.269E-01	6.87E-04<br>6.43E-06<br>3.93E-02<br>5.36E-02<br>1.05E-04<br>5.591E-01	-6.78E-21<br>-4.45E-19<br>1.02E-18<br>4.27E-01<br>2.23E-22<br>4.822E-01	1.71E-21<br>-1.10E-15<br>3.00E-17<br>9.56E-01<br>1.55E-19<br>9.255E-03	1.07E-08<br>4.50E-10<br>3.41E-07<br>2.64E-02<br>3.69E-09<br>5.996E-01	-4.55E-09<br>-4.23E-06<br>9.34E-09<br>4.49E-01<br>-1.27E-09<br>4.346E-01	6.60E-06<br>2.25E-09<br>7.88E-04<br>2.31E-01<br>3.96E-07<br>6.127E-01
(triosephosphate isomerase)<br>Local<br>Min<br>Max<br>Normalized peak height<br>Peak sensitivity value<br>Shapiro-Wilk score	4.14E-01<br>-8.75E-01<br>9.89E-01<br>3.09E-03<br>9.21E-01<br>9.243E-01	2.95E-03<br>-3.62E-02<br>5.59E-02<br>1.83E-02<br>3.82E-03<br>8.595E-01	2.25E-04<br>-7.56E-04<br>2.41E-03<br>1.58E-02<br>5.04E-05<br>8.584E-01	1.71E-03<br>-4.88E-03<br>1.80E-02<br>1.48E-02<br>3.51E-04<br>8.593E-01	3.00E-02<br>-4.15E-02<br>2.57E-01<br>1.37E-02<br>1.06E-03<br>8.638E-01	1.02E-02<br>1.57E-04<br>4.56E-02<br>4.42E-03<br>1.27E-03<br>9.308E-01	1.80E-01<br>9.22E-04<br>1.01E+00<br>6.48E-03<br>1.65E-02<br>8.845E-01	1.06E-01<br>6.00E-04<br>6.51E-01<br>6.80E-03<br>7.43E-03<br>8.897E-01	9.60E-02<br>4.18E-03<br>4.45E-01<br>3.70E-03<br>2.42E-02<br>9.537E-01	8.65E-17<br>-7.34E-15<br>2.48E-15<br>2.97E-01<br>3.96E-18<br>8.079E-01	1.45E-02<br>1.38E-03<br>5.97E-02<br>3.85E-03<br>8.06E-03<br>9.593E-01	1.68E-02<br>2.64E-04<br>4.39E-02<br>2.36E-03<br>1.71E-02<br>9.757E-01	3.45E-03<br>5.67E-05<br>8.93E-03<br>2.32E-03<br>3.82E-03<br>9.780E-01	-4.42E-02<br>-2.55E-01<br>-2.50E-03<br>4.11E-03<br>-1.65E-02<br>9.174E-01	1.42E-01<br>9.55E-04<br>1.77E+00<br>1.30E-02<br>1.25E-02<br>7.027E-01	2.67E-02<br>3.05E-04<br>7.19E-02<br>2.50E-03<br>8.14E-03<br>9.706E-01	-2.64E-19<br>-2.13E-18<br>2.33E-17<br>3.72E-01<br>-5.89E-21<br>6.293E-01	1.27E-19<br>-2.56E-17<br>1.30E-15<br>5.68E-01<br>2.06E-19<br>9.521E-02	4.16E-07<br>2.54E-09<br>1.79E-06<br>4.78E-03<br>1.34E-07<br>9.282E-01	1.10E-06<br>7.60E-08<br>6.90E-06<br>5.29E-03<br>4.00E-07<br>8.919E-01	-7.39E-04<br>-2.31E-03<br>-3.66E-05<br>3.36E-03<br>-6.09E-04<br>9.779E-01
(glyceraldehyde3phosphate dehydrogenase)<br>Local<br>Min<br>Max<br>Normalized peak height<br>Peak sensitivity value<br>Shapiro-Wilk score	7.06E-01<br>-2.22E-02<br>9.91E-01<br>3.27E-03<br>9.50E-01<br>9.245E-01	5.02E-03<br>-1.40E-02<br>1.79E-02<br>1.03E-02<br>4.35E-03<br>9.176E-01	1.49E-04<br>8.00E-06<br>1.87E-03<br>1.17E-02<br>4.61E-05<br>8.030E-01	1.08E-03<br>4.78E-05<br>2.03E-02<br>1.72E-02<br>2.81E-04<br>7.851E-01	1.51E-02<br>-7.85E-04<br>1.63E-01<br>1.45E-02<br>6.08E-04<br>8.186E-01	5.04E-03<br>1.07E-04<br>2.35E-02<br>4.55E-03<br>9.37E-04<br>9.202E-01	8.95E-02<br>5.42E-04<br>6.22E-01<br>7.91E-03<br>8.31E-03<br>8.499E-01	5.25E-02<br>2.46E-04<br>3.95E-01<br>8.60E-03<br>4.78E-03<br>8.568E-01	4.62E-02<br>1.88E-03<br>1.79E-01<br>3.07E-03<br>1.26E-02<br>9.723E-01	1.18E-16<br>-8.23E-16<br>8.54E-16<br>2.04E-01<br>-5.72E-19<br>8.869E-01	7.20E-03<br>8.32E-04<br>3.93E-02<br>4.93E-03<br>5.58E-03<br>9.066E-01	8.34E-03<br>1.41E-04<br>2.48E-02<br>2.70E-03<br>8.98E-03<br>9.798E-01	1.71E-03<br>2.99E-05<br>4.97E-03<br>2.82E-03<br>1.64E-03<br>9.815E-01	-2.12E-02<br>-4.81E-02<br>-9.50E-04<br>2.38E-03<br>-2.02E-02<br>9.776E-01	7.05E-02<br>5.38E-04<br>9.60E-01<br>1.32E-02<br>1.16E-02<br>6.666E-01	1.33E-02<br>1.79E-04<br>3.66E-02<br>2.35E-03<br>1.22E-02<br>9.753E-01	-1.31E-19<br>-8.47E-19<br>7.39E-18<br>3.67E-01<br>-3.06E-21<br>7.199E-01	6.21E-20<br>-5.99E-18<br>3.12E-16<br>3.99E-01<br>-1.13E-19<br>1.733E-01	2.07E-07<br>8.38E-10<br>8.17E-07<br>4.44E-03<br>1.11E-07<br>9.363E-01	5.29E-07<br>4.21E-08<br>1.88E-06<br>3.03E-03<br>5.64E-07<br>9.732E-01	-3.54E-04<br>-1.03E-03<br>-3.64E-06<br>3.35E-03<br>-2.22E-04<br>9.801E-01
(glycerol3phosphate dehydrogenase)<br>Local<br>Min<br>Max<br>Normalized peak height<br>Peak sensitivity value<br>Shapiro-Wilk score	7.06E-01<br>-2.27E-02<br>9.93E-01<br>3.34E-03<br>9.52E-01<br>9.239E-01	5.02E-03<br>-1.08E-02<br>1.89E-02<br>9.47E-03<br>4.48E-03<br>9.169E-01	1.49E-04<br>9.98E-06<br>2.59E-03<br>1.66E-02<br>4.49E-05<br>8.016E-01	1.08E-03<br>6.93E-05<br>2.36E-02<br>2.03E-02<br>3.17E-04<br>7.841E-01	1.51E-02<br>-8.91E-04<br>1.57E-01<br>1.35E-02<br>4.48E-04<br>8.167E-01	5.04E-03<br>7.41E-05<br>2.49E-02<br>4.51E-03<br>6.82E-04<br>9.193E-01	8.95E-02<br>5.60E-04<br>6.38E-01<br>7.63E-03<br>7.89E-03<br>8.492E-01	5.25E-02<br>2.32E-04<br>3.81E-01<br>7.99E-03<br>4.62E-03<br>8.551E-01	4.62E-02<br>1.29E-03<br>1.78E-01<br>3.13E-03<br>1.66E-02<br>9.721E-01	1.18E-16<br>-8.35E-16<br>9.17E-16<br>2.24E-01<br>-3.06E-19<br>8.838E-01	7.20E-03<br>7.22E-04<br>4.53E-02<br>5.72E-03<br>5.73E-03<br>9.063E-01	8.34E-03<br>1.34E-04<br>2.53E-02<br>2.73E-03<br>7.28E-03<br>9.797E-01	1.71E-03<br>3.16E-05<br>5.02E-03<br>2.77E-03<br>1.75E-03<br>9.814E-01	-2.12E-02<br>-5.04E-02<br>-6.61E-04<br>2.45E-03<br>-2.38E-02<br>9.776E-01	7.05E-02<br>4.62E-04<br>9.67E-01<br>1.36E-02<br>9.64E-03<br>6.633E-01	1.33E-02<br>1.77E-04<br>3.94E-02<br>2.57E-03<br>3.21E-03<br>9.752E-01	-1.31E-19<br>-7.57E-19<br>1.33E-17<br>3.78E-01<br>-6.45E-21<br>6.201E-01	6.21E-20<br>-5.14E-18<br>2.12E-16<br>3.62E-01<br>-3.86E-20<br>1.758E-01	2.07E-07<br>1.01E-09<br>8.45E-07<br>4.49E-03<br>1.02E-07<br>9.384E-01	5.29E-07<br>3.82E-08<br>2.09E-06<br>3.15E-03<br>5.61E-07<br>9.728E-01	-3.54E-04<br>-1.01E-03<br>-1.68E-06<br>3.12E-03<br>-2.71E-04<br>9.798E-01
(glycerol3phosphate oxidase)<br>Local<br>Min<br>Max<br>Normalized peak height<br>Peak sensitivity value<br>Shapiro-Wilk score	4.14E-01<br>-9.10E-01<br>9.90E-01<br>3.19E-03<br>9.26E-01<br>9.244E-01	2.95E-03<br>-3.66E-02<br>5.43E-02<br>1.79E-02<br>3.78E-03<br>8.617E-01	2.25E-04<br>-6.72E-04<br>2.26E-03<br>1.44E-02<br>4.12E-05<br>8.583E-01	1.71E-03<br>-4.37E-03<br>1.70E-02<br>1.36E-02<br>3.75E-04<br>8.596E-01	3.00E-02<br>-3.88E-02<br>2.67E-01<br>1.36E-02<br>1.10E-03<br>8.639E-01	1.02E-02<br>1.58E-04<br>4.56E-02<br>4.38E-03<br>1.54E-03<br>9.307E-01	1.80E-01<br>9.05E-04<br>1.02E+00<br>6.64E-03<br>1.76E-02<br>8.841E-01	1.06E-01<br>4.60E-04<br>6.78E-01<br>7.14E-03<br>1.03E-02<br>8.903E-01	9.60E-02<br>3.59E-03<br>3.83E-01<br>3.16E-03<br>2.81E-02<br>9.545E-01	8.65E-17<br>-3.76E-15<br>5.54E-15<br>3.33E-01<br>1.30E-18<br>8.213E-01	1.45E-02<br>1.71E-03<br>5.04E-02<br>3.53E-03<br>1.28E-02<br>9.600E-01	1.68E-02<br>2.42E-04<br>4.29E-02<br>2.39E-03<br>1.23E-02<br>9.755E-01	3.45E-03<br>5.07E-05<br>9.13E-03<br>2.44E-03<br>2.75E-03<br>9.778E-01	-4.42E-02<br>-2.71E-01<br>-2.15E-03<br>4.40E-03<br>-1.82E-02<br>9.180E-01	1.42E-01<br>7.86E-04<br>1.83E+00<br>1.38E-02<br>1.27E-02<br>7.044E-01	2.67E-02<br>3.10E-04<br>7.14E-02<br>2.37E-03<br>2.32E-02<br>9.703E-01	-2.64E-19<br>-1.21E-18<br>8.68E-18<br>3.59E-01<br>-2.45E-21<br>8.657E-01	1.27E-19<br>-2.67E-17<br>5.74E-16<br>4.12E-01<br>1.73E-20<br>1.772E-01	4.16E-07<br>1.25E-09<br>1.93E-06<br>5.31E-03<br>1.86E-07<br>9.280E-01	1.10E-06<br>8.75E-08<br>7.64E-06<br>5.38E-03<br>5.67E-07<br>8.920E-01	-7.39E-04<br>-2.21E-03<br>-3.93E-05<br>3.17E-03<br>-6.08E-04<br>9.786E-01
(pyruvate transport)<br>Local<br>Min<br>Max<br>Normalized peak height<br>Peak sensitivity value<br>Shapiro-Wilk score	7.06E-01<br>-1.34E-02<br>9.91E-01<br>3.47E-03<br>9.58E-01<br>9.254E-01	5.02E-03<br>-8.17E-03<br>1.85E-02<br>8.82E-03<br>4.53E-03<br>9.176E-01	1.49E-04<br>1.03E-05<br>2.11E-03<br>1.40E-02<br>4.29E-05<br>8.000E-01	1.08E-03<br>6.51E-05<br>1.72E-02<br>1.52E-02<br>3.31E-04<br>7.825E-01	1.51E-02<br>-5.92E-04<br>1.66E-01<br>1.48E-02<br>3.22E-04<br>8.174E-01	5.04E-03<br>8.42E-05<br>2.47E-02<br>4.73E-03<br>7.60E-04<br>9.200E-01	8.95E-02<br>6.54E-04<br>6.33E-01<br>8.09E-03<br>7.30E-03<br>8.498E-01	5.25E-02<br>3.38E-04<br>3.91E-01<br>8.34E-03<br>4.83E-03<br>8.565E-01	4.62E-02<br>1.68E-03<br>1.80E-01<br>3.28E-03<br>4.80E-02<br>9.722E-01	-2.22E-16<br>-8.88E-16<br>7.77E-16<br>3.85E-01<br>2.78E-19<br>9.207E-01	7.20E-03<br>8.51E-04<br>4.28E-02<br>5.50E-03<br>5.74E-03<br>9.074E-01	8.34E-03<br>1.30E-04<br>2.32E-02<br>2.59E-03<br>8.77E-03<br>9.793E-01	1.71E-03<br>3.15E-05<br>4.86E-03<br>2.73E-03<br>1.59E-03<br>9.812E-01	-2.12E-02<br>-5.10E-02<br>-1.02E-03<br>2.48E-03<br>-2.33E-02<br>9.776E-01	7.05E-02<br>6.10E-04<br>9.35E-01<br>1.28E-02<br>9.49E-03<br>6.692E-01	1.33E-02<br>1.74E-04<br>3.95E-02<br>2.55E-03<br>1.27E-02<br>9.751E-01	-1.31E-19<br>-7.75E-19<br>9.92E-18<br>3.62E-01<br>-1.11E-22<br>7.018E-01	6.21E-20<br>-5.77E-18<br>9.22E-16<br>6.43E-01<br>2.59E-19<br>5.304E-02	2.07E-07<br>1.87E-09<br>9.16E-07<br>5.09E-03<br>1.15E-07<br>9.373E-01	5.29E-07<br>4.44E-08<br>1.68E-06<br>2.67E-03<br>5.81E-07<br>9.728E-01	-3.54E-04<br>-9.42E-04<br>-3.63E-06<br>3.05E-03<br>-2.68E-04<br>9.790E-01

(phosphoglycerate kinase)	Local	7.06E-01	5.02E-03	1.49E-04	1.08E-03	1.51E-02	5.04E-03	8.95E-02	5.25E-02	4.62E-02	2.34E-17	7.20E-03
	Min	-1.53E-02	-8.52E-03	1.11E-05	5.45E-05	-5.72E-04	8.06E-05	5.98E-04	3.48E-04	1.46E-03	-6.36E-16	6.91E-04
	Max	9.93E-01	2.06E-02	1.97E-03	1.62E-02	1.54E-01	2.51E-02	6.22E-01	3.91E-01	2.12E-01	6.74E-16	4.23E-02
	Normalized peak height	3.22E-03	9.39E-03	1.27E-02	1.39E-02	1.33E-02	4.69E-03	7.71E-03	8.38E-03	3.71E-03	1.17E-01	5.32E-03
	Peak sensitivity value	9.49E-01	4.60E-03	4.54E-05	3.05E-04	7.42E-04	7.19E-04	9.61E-03	4.84E-03	4.85E-02	3.55E-20	5.13E-03
	Shapiro-Wilk score	9.251E-01	9.166E-01	8.006E-01	7.846E-01	8.188E-01	9.205E-01	8.503E-01	8.565E-01	9.724E-01	9.058E-01	9.047E-01
(pyruvate kinase)	Local	7.06E-01	5.02E-03	1.49E-04	1.08E-03	1.51E-02	5.04E-03	8.95E-02	5.25E-02	4.62E-02	-2.12E-16	7.20E-03
	Min	-1.42E-02	-1.17E-02	9.60E-06	5.29E-05	-7.19E-04	7.59E-05	3.70E-04	2.25E-04	1.94E-03	-8.47E-16	8.74E-04
	Max	9.92E-01	2.02E-02	2.18E-03	1.95E-02	1.64E-01	2.46E-02	6.00E-01	3.83E-01	1.84E-01	8.80E-16	4.11E-02
	Normalized peak height	3.42E-03	1.03E-02	1.45E-02	1.72E-02	1.48E-02	4.60E-03	7.47E-03	7.83E-03	3.20E-03	2.81E-01	5.03E-03
	Peak sensitivity value	9.56E-01	4.53E-03	4.54E-05	3.16E-04	3.49E-04	6.27E-04	5.47E-03	4.25E-03	4.93E-02	4.88E-20	6.28E-03
	Shapiro-Wilk score	9.250E-01	9.145E-01	8.046E-01	7.869E-01	8.192E-01	9.209E-01	8.515E-01	8.563E-01	9.720E-01	9.780E-01	9.057E-01
(atp utilisation)	Local	7.06E-01	5.02E-03	1.49E-04	1.08E-03	1.51E-02	5.04E-03	8.95E-02	5.25E-02	4.62E-02	2.20E-21	7.20E-03
	Min	-1.97E-02	-6.52E-03	9.85E-06	6.45E-05	-8.27E-04	9.54E-05	5.27E-04	3.52E-04	1.06E-03	-1.66E-12	9.28E-04
	Max	9.92E-01	2.03E-02	1.84E-03	1.68E-02	1.58E-01	2.45E-02	5.95E-01	3.73E-01	1.72E-01	1.01E-12	4.21E-02
	Normalized peak height	3.28E-03	8.73E-03	1.20E-02	1.44E-02	1.32E-02	4.72E-03	7.40E-03	7.49E-03	2.97E-03	6.27E-01	5.37E-03
	Peak sensitivity value	9.62E-01	4.77E-03	3.83E-05	3.07E-04	5.24E-04	6.21E-04	5.58E-03	4.64E-03	5.13E-02	-2.15E-16	4.36E-03
	Shapiro-Wilk score	9.248E-01	9.134E-01	8.044E-01	7.865E-01	8.180E-01	9.202E-01	8.500E-01	8.557E-01	9.725E-01	2.875E-01	9.041E-01
(glycerol kinase)	Local	9.22E+00	6.57E-02	-2.07E-03	-1.73E-02	-4.20E-01	-1.47E-01	-2.56E+00	-1.50E+00	-1.41E+00	1.05E-15	-2.06E-01
	Min	-1.42E+00	-3.06E-01	-1.20E-02	-1.05E-01	-2.02E+00	-5.28E-01	-1.06E+01	-5.97E+00	-4.37E+00	-6.12E-14	-9.04E-01
	Max	2.25E+01	2.99E-01	7.30E-03	5.45E-02	6.45E-01	1.30E-02	1.53E+00	9.07E-01	-2.92E-01	5.26E-14	1.01E-01
	Normalized peak height	5.19E-03	8.35E-03	7.92E-03	7.81E-03	6.45E-03	5.80E-03	6.51E-03	6.15E-03	4.62E-03	3.27E-01	3.55E-03
	Peak sensitivity value	7.86E+00	4.80E-02	-7.64E-04	-7.51E-03	-1.77E-01	-1.10E-01	-1.58E+00	-9.18E-01	-1.53E+00	-7.35E-18	-1.16E-01
	Shapiro-Wilk score	9.654E-01	9.244E-01	9.695E-01	9.689E-01	9.755E-01	9.842E-01	9.714E-01	9.729E-01	9.802E-01	6.763E-01	9.523E-01
(phosphoglycerate mutase)	Local	7.06E-01	5.02E-03	1.49E-04	1.08E-03	1.51E-02	5.04E-03	8.95E-02	5.25E-02	4.62E-02	2.10E-17	7.20E-03
	Min	-1.66E-02	-9.49E-03	8.12E-06	5.27E-05	-4.78E-04	9.31E-05	5.93E-04	3.10E-04	1.98E-03	-7.85E-16	8.35E-04
	Max	9.92E-01	2.00E-02	1.95E-03	1.66E-02	1.61E-01	2.42E-02	6.18E-01	3.95E-01	1.89E-01	8.35E-16	4.05E-02
	Normalized peak height	3.34E-03	9.32E-03	1.26E-02	1.47E-02	1.39E-02	4.38E-03	7.81E-03	8.17E-03	3.29E-03	1.62E-01	5.10E-03
	Peak sensitivity value	9.55E-01	4.34E-03	3.82E-05	2.92E-04	5.73E-04	6.83E-04	7.07E-03	4.85E-03	5.06E-02	-6.78E-20	6.45E-03
	Shapiro-Wilk score	9.246E-01	9.166E-01	8.018E-01	7.837E-01	8.178E-01	9.196E-01	8.494E-01	8.564E-01	9.719E-01	9.085E-01	9.062E-01
(enolase)	Local	7.06E-01	5.02E-03	1.49E-04	1.08E-03	1.51E-02	5.04E-03	8.95E-02	5.25E-02	4.62E-02	2.10E-17	7.20E-03
	Min	-1.93E-02	-6.77E-03	9.84E-06	6.13E-05	-6.41E-04	9.03E-05	4.48E-04	2.22E-04	1.81E-03	-7.65E-16	7.92E-04
	Max	9.93E-01	2.05E-02	2.25E-03	1.90E-02	1.56E-01	2.53E-02	6.30E-01	3.93E-01	1.71E-01	6.75E-16	4.25E-02
	Normalized peak height	3.42E-03	8.86E-03	1.42E-02	1.61E-02	1.36E-02	4.66E-03	8.13E-03	8.22E-03	2.98E-03	1.54E-01	5.25E-03
	Peak sensitivity value	9.62E-01	4.90E-03	4.45E-05	3.35E-04	5.34E-04	7.57E-04	7.69E-03	3.95E-03	4.94E-02	2.67E-19	5.74E-03
	Shapiro-Wilk score	9.251E-01	9.174E-01	8.022E-01	7.845E-01	8.186E-01	9.209E-01	8.499E-01	8.550E-01	9.726E-01	9.090E-01	9.054E-01
(adenylate kinase cytosol)	Local	INF	INF	INF	INF	INF	INF	INF	INF	INF	INF	INF
	Min	-INF	-INF	-INF	-INF	-INF	-INF	-INF	-INF	-INF	-INF	-INF
	Max	INF	INF	INF	INF	INF	INF	INF	INF	INF	INF	INF
	Normalized peak height	1.67E-01	1.67E-01	1.67E-01	1.67E-01	1.67E-01	1.67E-01	1.67E-01	1.67E-01	1.67E-01	5.00E-01	1.67E-01
	Peak sensitivity value	-5.42E-02	-4.42E-02	-9.49E-03	-1.68E-02	-1.21E-03	-6.46E-04	-7.33E-03	-1.14E-02	-5.49E-03	-2.94E-07	-6.01E-02
	Shapiro-Wilk score	8.904E-01	9.322E-01	9.273E-01	7.909E-01	9.219E-01	8.509E-01	8.624E-01	7.679E-01	9.515E-01	7.536E-01	9.060E-01
(adenylate kinase glycosome)	Local	-INF	-INF	-INF	-INF	-INF	-INF	-INF	-INF	-INF	-INF	-INF
	Min	-INF	-INF	-INF	-INF	-INF	-INF	-INF	-INF	-INF	-INF	-INF
	Max	INF	INF	INF	INF	INF	INF	INF	INF	INF	INF	INF
	Normalized peak height	7.65E-02	2.39E-02	2.29E-02	2.14E-02	9.11E-02	5.21E-02	8.46E-02	8.77E-02	2.73E-01	2.07E-01	1.93E-02
	Peak sensitivity value	-5.25E-02	5.18E-02	-3.55E-02	2.78E-02	-5.36E-03	2.79E-04	4.66E-04	-8.68E-03	3.90E-04	3.31E-09	5.99E-02
	Shapiro-Wilk score	8.945E-01	8.822E-01	8.902E-01	8.900E-01	8.371E-01	8.570E-01	8.354E-01	8.338E-01	6.755E-01	7.717E-01	8.956E-01
(3phosphoglycerate transport)	Local	7.06E-01	5.02E-03	1.49E-04	1.08E-03	1.51E-02	5.04E-03	8.95E-02	5.25E-02	4.62E-02	2.34E-17	7.20E-03
	Min	-1.27E-02	-8.11E-03	9.51E-06	5.07E-05	-9.30E-04	7.78E-05	5.89E-04	3.09E-04	1.56E-03	-7.41E-16	6.69E-04
	Max	9.93E-01	1.81E-02	2.09E-03	1.73E-02	1.62E-01	2.46E-02	6.09E-01	3.88E-01	1.75E-01	6.62E-16	4.14E-02
	Normalized peak height	3.33E-03	8.52E-03	1.33E-02	1.53E-02	1.41E-02	4.82E-03	7.63E-03	8.07E-03	3.13E-03	1.12E-01	5.21E-03
	Peak sensitivity value	9.52E-01	4.51E-03	4.39E-05	3.17E-04	4.53E-04	6.05E-04	7.58E-03	3.99E-03	5.35E-02	6.06E-19	5.58E-03
	Shapiro-Wilk score	9.235E-01	9.155E-01	7.990E-01	7.815E-01	8.169E-01	9.194E-01	8.484E-01	8.546E-01	9.715E-01	9.062E-01	9.046E-01
(gly3p dhap antiporter)	Local	4.14E-01	2.95E-03	2.25E-04	1.71E-03	3.00E-02	1.02E-02	1.80E-01	1.06E-01	9.60E-02	8.65E-17	1.45E-02
	Min	-9.01E-01	-3.93E-02	-5.65E-04	-3.99E-03	-3.14E-02	1.76E-04	9.44E-04	4.56E-04	4.12E-03	-1.61E-15	1.69E-03
	Max	9.88E-01	4.68E-02	2.23E-03	1.71E-02	2.49E-01	4.54E-02	1.02E+00	6.71E-01	3.98E-01	4.12E-15	4.89E-02
	Normalized peak height	3.27E-03	1.69E-02	1.40E-02	1.31E-02	1.21E-02	4.29E-03	6.42E-03	6.93E-03	3.37E-03	2.56E-01	3.04E-03
	Peak sensitivity value	9.27E-01	3.71E-03	4.93E-05	3.71E-04	5.03E-04	1.60E-03	1.78E-02	8.84E-03	2.79E-02	-2.11E-18	8.33E-03
	Shapiro-Wilk score	9.258E-01	8.600E-01	8.621E-01	8.619E-01	8.643E-01	9.322E-01	8.859E-01	8.918E-01	9.557E-01	8.338E-01	9.624E-01
(glycerol transport)	Local	9.22E+00	6.57E-02	-2.07E-03	-1.73E-02	-4.20E-01	-1.47E-01	-2.56E+00	-1.50E+00	-1.41E+00	1.05E-15	-2.06E-01
	Min	-1.30E+00	-2.95E-01	-1.35E-02	-1.24E-01	-2.11E+00	-4.31E-01	-9.41E+00	-6.41E+00	-4.41E+00	-7.04E-14	-9.06E-01
	Max	2.00E+01	2.69E-01	7.44E-03	5.55E-02	6.82E-01	1.23E-02	1.45E+00	9.04E-01	-3.08E-01	6.47E-14	7.93E-02
	Normalized peak height	4.63E-03	7.49E-03	8.65E-03	8.94E-03	6.60E-03	5.07E-03	5.60E-03	6.40E-03	4.75E-03	3.13E-01	3.36E-03
	Peak sensitivity value	8.10E+00	5.00E-02	-7.08E-04	-7.66E-03	-1.98E-01	-1.14E-01	-1.59E+00	-9.86E-01	-1.57E+00	5.51E-17	-1.21E-01
	Shapiro-Wilk score	9.666E-01	9.251E-01	9.678E-01	9.666E-01	9.746E-01	9.835E-01	9.703E-01	9.708E-01	9.798E-01	6.902E-01	9.522E-01

(phosphoglycerate kinase)	Local	8.34E-03	1.71E-03	-2.12E-02	7.05E-02	1.33E-02	-1.31E-19	6.21E-20	2.07E-07	5.29E-07	-3.54E-04
	Min	1.43E-04	3.60E-05	-4.86E-02	6.42E-04	2.16E-04	-6.21E-19	-9.14E-18	9.80E-10	4.34E-08	-1.05E-03
	Max	2.21E-02	4.53E-03	-8.55E-04	9.60E-01	3.92E-02	1.44E-18	1.98E-16	9.32E-07	1.78E-06	-3.03E-06
	Normalized peak height	2.50E-03	2.54E-03	2.29E-03	1.29E-02	2.43E-03	3.57E-01	3.29E-01	5.08E-03	2.69E-03	3.30E-03
	Peak sensitivity value	6.88E-03	1.70E-03	-2.33E-02	8.79E-03	1.08E-02	-1.01E-21	8.19E-20	1.13E-07	4.43E-07	-2.18E-04
	Shapiro-Wilk score	9.804E-01	9.820E-01	9.781E-01	6.668E-01	9.759E-01	9.223E-01	2.123E-01	9.371E-01	9.734E-01	9.795E-01
(pyruvate kinase)	Local	8.34E-03	1.71E-03	-2.12E-02	7.05E-02	1.33E-02	-1.31E-19	6.21E-20	2.07E-07	5.29E-07	-3.54E-04
	Min	1.17E-04	2.14E-05	-4.89E-02	3.79E-04	1.24E-04	-8.49E-19	-6.67E-18	1.80E-09	4.04E-08	-1.03E-03
	Max	2.17E-02	4.59E-03	-1.14E-03	9.37E-01	3.49E-02	2.59E-18	3.28E-16	8.11E-07	1.76E-06	-2.94E-06
	Normalized peak height	2.49E-03	2.51E-03	2.47E-03	1.23E-02	2.30E-03	3.58E-01	3.79E-01	4.49E-03	2.70E-03	3.24E-03
	Peak sensitivity value	8.27E-03	1.71E-03	-2.46E-02	9.28E-03	9.35E-03	1.69E-21	-1.50E-19	9.04E-08	2.17E-07	-2.23E-04
	Shapiro-Wilk score	9.796E-01	9.814E-01	9.770E-01	6.675E-01	9.752E-01	8.994E-01	1.615E-01	9.360E-01	9.735E-01	9.797E-01
(atp utilisation)	Local	8.34E-03	1.71E-03	-2.12E-02	7.05E-02	1.33E-02	-1.31E-19	6.21E-20	2.07E-07	5.29E-07	-3.54E-04
	Min	1.36E-04	4.03E-05	-5.21E-02	5.18E-04	2.05E-04	-6.79E-19	-5.13E-18	8.59E-10	2.16E-08	-9.87E-04
	Max	2.33E-02	5.42E-03	-3.33E-04	9.63E-01	4.19E-02	1.24E-17	1.98E-16	8.92E-07	1.90E-06	-6.47E-07
	Normalized peak height	2.57E-03	2.90E-03	2.73E-03	1.27E-02	2.71E-03	3.73E-01	3.45E-01	4.89E-03	2.94E-03	3.24E-03
	Peak sensitivity value	8.08E-03	1.74E-03	-2.39E-02	8.70E-03	1.28E-02	5.94E-21	5.08E-20	1.01E-07	5.41E-07	-2.38E-04
	Shapiro-Wilk score	9.801E-01	9.819E-01	9.780E-01	6.659E-01	9.755E-01	6.759E-01	1.821E-01	9.363E-01	9.734E-01	9.797E-01
(glycerol kinase)	Local	-2.39E-01	-4.91E-02	6.53E-01	-2.02E+00	-3.80E-01	3.75E-18	-1.82E-18	-5.91E-06	-1.61E-05	1.09E-02
	Min	-4.30E-01	-9.13E-02	1.71E-01	-1.58E+01	-7.07E-01	-6.56E-17	-3.76E-15	-4.02E-05	-7.25E-05	5.95E-04
	Max	4.27E-02	9.27E-03	9.76E-01	1.53E+00	7.27E-02	2.40E-17	1.74E-16	5.78E-07	-2.03E-06	4.08E-02
	Normalized peak height	2.64E-03	2.58E-03	2.31E-03	9.89E-03	2.85E-03	3.78E-01	2.00E-01	7.42E-03	4.07E-03	4.23E-03
	Peak sensitivity value	-2.52E-01	-5.32E-02	9.18E-01	-1.20E+00	-3.69E-01	-3.59E-20	-1.01E-18	-1.33E-08	-1.26E-05	3.03E-03
	Shapiro-Wilk score	9.544E-01	9.488E-01	9.336E-01	9.116E-01	9.572E-01	8.614E-01	3.137E-01	9.177E-01	9.387E-01	9.450E-01
(phosphoglycerate mutase)	Local	8.34E-03	1.71E-03	-2.12E-02	7.05E-02	1.33E-02	-1.31E-19	6.21E-20	2.07E-07	5.29E-07	-3.54E-04
	Min	1.53E-04	3.94E-05	-4.84E-02	5.83E-04	1.96E-04	-6.42E-19	-5.78E-18	2.12E-09	3.81E-08	-1.03E-03
	Max	2.34E-02	4.50E-03	-1.22E-03	9.26E-01	3.65E-02	8.59E-18	3.36E-16	9.48E-07	1.92E-06	-2.79E-06
	Normalized peak height	2.52E-03	2.49E-03	2.35E-03	1.26E-02	2.42E-03	3.61E-01	3.96E-01	5.15E-03	3.00E-03	3.23E-03
	Peak sensitivity value	5.35E-03	1.49E-03	-2.24E-02	9.38E-03	1.28E-02	-1.51E-22	-1.41E-19	1.20E-07	5.78E-07	-2.55E-04
	Shapiro-Wilk score	9.799E-01	9.816E-01	9.776E-01	6.660E-01	9.753E-01	7.888E-01	1.690E-01	9.371E-01	9.730E-01	9.787E-01
(enolase)	Local	8.34E-03	1.71E-03	-2.12E-02	7.05E-02	1.33E-02	-1.31E-19	6.21E-20	2.07E-07	5.29E-07	-3.54E-04
	Min	9.37E-05	2.23E-05	-4.82E-02	3.79E-04	1.33E-04	-8.16E-19	-6.29E-18	5.94E-10	3.83E-08	-9.32E-04
	Max	2.69E-02	4.90E-03	-1.11E-03	9.57E-01	3.80E-02	6.43E-18	1.96E-16	8.33E-07	1.77E-06	-4.87E-06
	Normalized peak height	3.02E-03	2.69E-03	2.33E-03	1.33E-02	2.54E-03	3.63E-01	3.23E-01	4.42E-03	2.73E-03	2.98E-03
	Peak sensitivity value	6.81E-03	1.71E-03	-2.38E-02	8.51E-03	1.18E-02	-3.96E-22	8.13E-20	1.08E-07	5.08E-07	-2.45E-04
	Shapiro-Wilk score	9.802E-01	9.820E-01	9.785E-01	6.630E-01	9.757E-01	8.343E-01	2.043E-01	9.392E-01	9.733E-01	9.801E-01
(adenylate kinase cytosol)	Local	INF	INF	INF	INF	INF	INF	INF	INF	INF	INF
	Min	-INF	-INF	-INF	-INF	-INF	-INF	-INF	-INF	-INF	-INF
	Max	INF	INF	INF	INF	INF	INF	INF	INF	INF	INF
	Normalized peak height	1.67E-01	1.67E-01	1.67E-01	1.67E-01	1.67E-01	1.67E-01	1.67E-01	1.67E-01	1.67E-01	1.67E-01
	Peak sensitivity value	-9.01E-02	-3.37E-02	-2.21E-04	-4.20E-02	-3.02E-02	-7.73E-13	-1.86E-13	-2.99E-02	-1.14E-03	-9.40E-06
	Shapiro-Wilk score	8.685E-01	9.628E-01	8.819E-01	8.240E-01	9.298E-01	9.547E-01	9.515E-01	9.401E-01	8.786E-01	8.170E-01
(adenylate kinase glycosome)	Local	-INF	-INF	-INF	-INF	-INF	-INF	-INF	-INF	-INF	-INF
	Min	-INF	-INF	-INF	-INF	-INF	-INF	-INF	-INF	-INF	-INF
	Max	INF	INF	INF	INF	INF	INF	INF	INF	INF	INF
	Normalized peak height	6.38E-02	6.66E-02	1.69E-01	9.17E-02	5.70E-02	3.86E-01	1.12E-02	3.86E-02	5.43E-02	1.40E-01
	Peak sensitivity value	4.06E-04	3.64E-05	-1.28E-03	-8.99E-03	-8.23E-04	-7.55E-17	-5.20E-13	6.88E-03	3.92E-03	3.12E-05
	Shapiro-Wilk score	9.263E-01	9.306E-01	7.570E-01	7.758E-01	9.236E-01	6.584E-01	9.577E-01	8.289E-01	7.943E-01	7.776E-01
(3phosphoglycerate transport)	Local	8.34E-03	1.71E-03	-2.12E-02	7.05E-02	1.33E-02	-1.31E-19	6.20E-20	2.07E-07	5.29E-07	-3.54E-04
	Min	1.45E-04	3.48E-05	-5.17E-02	5.88E-04	2.02E-04	-6.26E-19	-6.40E-18	-1.29E-09	4.33E-08	-9.99E-04
	Max	2.19E-02	4.48E-03	-6.75E-04	9.55E-01	3.64E-02	4.97E-18	1.92E-16	7.82E-07	1.79E-06	-2.32E-06
	Normalized peak height	2.46E-03	2.50E-03	2.52E-03	1.31E-02	2.46E-03	3.63E-01	3.47E-01	4.45E-03	2.75E-03	3.17E-03
	Peak sensitivity value	8.28E-03	1.66E-03	-2.35E-02	1.06E-02	1.21E-02	-1.31E-21	3.98E-20	1.25E-07	5.67E-07	-2.13E-04
	Shapiro-Wilk score	9.795E-01	9.813E-01	9.775E-01	6.632E-01	9.751E-01	8.549E-01	1.804E-01	9.374E-01	9.731E-01	9.794E-01
(gly3p dhap antiporter)	Local	1.68E-02	3.45E-03	-4.42E-02	1.42E-01	2.67E-02	-2.64E-19	1.27E-19	4.16E-07	1.10E-06	-7.39E-04
	Min	2.21E-04	5.94E-05	-3.13E-01	9.85E-04	3.13E-04	-1.40E-18	-2.96E-17	1.91E-09	8.05E-08	-2.35E-03
	Max	4.51E-02	8.92E-03	-2.44E-03	1.73E+00	6.97E-02	6.30E-18	1.31E-15	1.74E-06	6.96E-06	-3.86E-05
	Normalized peak height	2.43E-03	2.43E-03	5.07E-03	1.28E-02	2.29E-03	3.61E-01	4.85E-01	4.69E-03	5.00E-03	3.37E-03
	Peak sensitivity value	4.69E-03	3.64E-03	-1.47E-02	1.91E-02	4.51E-03	-3.26E-21	5.95E-19	1.86E-07	4.07E-07	-5.99E-04
	Shapiro-Wilk score	9.761E-01	9.783E-01	9.195E-01	7.048E-01	9.710E-01	8.729E-01	9.124E-02	9.285E-01	8.951E-01	9.784E-01
(glycerol transport)	Local	-2.39E-01	-4.91E-02	6.53E-01	-2.02E+00	-3.80E-01	3.75E-18	-1.82E-18	-5.91E-06	-1.61E-05	1.09E-02
	Min	-4.27E-01	-9.43E-02	1.58E-01	-1.21E+01	-6.86E-01	-4.55E-17	-9.40E-15	-3.66E-05	-7.86E-05	7.16E-04
	Max	4.82E-02	9.85E-03	9.80E-01	2.16E+00	6.39E-02	2.15E-17	1.90E-16	6.08E-07	-1.91E-06	3.90E-02
	Normalized peak height	2.70E-03	2.63E-03	2.38E-03	7.80E-03	2.94E-03	3.73E-01	3.68E-01	6.63E-03	4.30E-03	4.09E-03
	Peak sensitivity value	-2.58E-01	-5.63E-02	9.26E-01	-1.22E+00	-3.76E-01	2.40E-20	3.13E-18	-4.23E-08	-1.18E-05	3.26E-03
	Shapiro-Wilk score	9.546E-01	9.496E-01	9.341E-01	9.090E-01	9.579E-01	8.817E-01	1.286E-01	9.170E-01	9.377E-01	9.449E-01

Table S14: Flux control coefficients for *Trypanosoma brucei* model with parameter variation of ±30%

		(glucosetransport)	(hexokinase)	(phosphoglycerateisomerase)	(phophofructokinase)	(aldolase)	(triosephosphateisomerase)	(glyceraldehyde3phosphatedehydrogenase)	(glycerol3phosphatedehydrogenase)	(glycerol3phosphateoxidase)	(pyruvatetransport)
(glucose transport)	Local	9.79E-01	6.97E-03	7.82E-05	4.95E-04	1.13E-03	1.79E-04	4.63E-03	2.72E-03	-3.97E-04	-1.47E-18
	Min	-5.54E-01	-1.26E-01	-1.83E-04	-2.69E-04	2.21E-06	-7.15E-03	4.13E-06	1.78E-06	-2.63E-01	-5.26E-14
	Max	9.98E-01	5.85E-02	5.41E-03	7.45E-02	3.18E-01	3.37E-02	8.32E-01	6.21E-01	3.94E-02	7.44E-13
	Normalized peak height	8.53E-02	2.30E-02	5.47E-02	1.00E-01	3.38E-01	1.91E-01	2.91E-01	3.19E-01	4.12E-01	9.07E-01
	Peak sensitivity value	9.94E-01	4.42E-03	2.63E-05	1.43E-04	1.61E-04	2.84E-05	4.20E-04	3.12E-04	-6.95E-05	-3.89E-16
	Shapiro-Wilk score	6.588E-01	8.610E-01	6.682E-01	6.251E-01	6.187E-01	6.258E-01	5.805E-01	5.823E-01	6.514E-01	2.089E-02
(hexokinase)	Local	9.79E-01	6.97E-03	7.82E-05	4.95E-04	1.13E-03	1.79E-04	4.63E-03	2.72E-03	-3.97E-04	-1.47E-18
	Min	-4.37E-01	-8.64E-02	-3.01E-04	-5.85E-04	1.35E-06	-6.74E-03	2.47E-06	8.07E-07	-2.64E-01	-1.17E-13
	Max	9.98E-01	6.35E-02	5.39E-03	4.91E-02	2.89E-01	2.99E-02	8.52E-01	5.67E-01	2.21E-02	1.46E-13
	Normalized peak height	7.94E-02	1.84E-02	5.45E-02	6.94E-02	3.24E-01	2.38E-01	2.91E-01	3.04E-01	4.07E-01	9.39E-01
	Peak sensitivity value	9.95E-01	4.37E-03	2.61E-05	1.85E-04	1.46E-04	2.09E-05	4.29E-04	2.84E-04	-6.81E-05	7.81E-17
	Shapiro-Wilk score	6.587E-01	8.603E-01	6.664E-01	6.261E-01	6.170E-01	6.246E-01	5.787E-01	5.811E-01	6.522E-01	5.781E-02
(phosphoglycerate isomerase)	Local	9.79E-01	6.97E-03	7.82E-05	4.95E-04	1.13E-03	1.79E-04	4.63E-03	2.72E-03	-3.97E-04	-1.47E-18
	Min	-5.15E-01	-1.10E-01	-1.01E-04	-7.23E-05	2.36E-06	-7.56E-03	2.98E-06	1.53E-06	-2.59E-01	-1.85E-13
	Max	9.98E-01	5.14E-02	5.59E-03	7.46E-02	2.96E-01	3.27E-02	9.76E-01	5.23E-01	3.91E-02	1.28E-13
	Normalized peak height	8.62E-02	1.97E-02	5.54E-02	1.04E-01	3.29E-01	2.18E-01	3.09E-01	2.97E-01	2.50E-01	9.54E-01
	Peak sensitivity value	9.95E-01	4.59E-03	2.75E-05	1.89E-04	1.51E-04	-4.01E-06	4.91E-04	2.63E-04	1.25E-04	4.56E-17
	Shapiro-Wilk score	6.568E-01	8.611E-01	6.649E-01	6.252E-01	6.158E-01	6.235E-01	5.773E-01	5.785E-01	6.491E-01	5.461E-02
(phophofructokinase)	Local	9.79E-01	6.97E-03	7.82E-05	4.95E-04	1.13E-03	1.79E-04	4.63E-03	2.72E-03	-3.97E-04	-1.47E-18
	Min	-5.22E-01	-9.57E-02	-1.01E-04	-4.03E-04	1.85E-06	-8.70E-03	2.27E-06	1.51E-06	-2.50E-01	-1.89E-13
	Max	9.98E-01	5.36E-02	6.28E-03	5.86E-02	2.90E-01	3.45E-02	8.65E-01	5.30E-01	2.38E-02	1.11E-13
	Normalized peak height	8.46E-02	1.90E-02	6.16E-02	8.18E-02	3.24E-01	2.79E-01	2.93E-01	2.97E-01	2.91E-01	9.08E-01
	Peak sensitivity value	9.95E-01	4.57E-03	2.36E-05	1.57E-04	1.47E-04	7.09E-06	4.35E-04	2.66E-04	-1.46E-04	1.39E-16
	Shapiro-Wilk score	6.597E-01	8.605E-01	6.658E-01	6.258E-01	6.191E-01	6.246E-01	5.814E-01	5.843E-01	6.535E-01	5.665E-02
(aldolase)	Local	9.79E-01	6.97E-03	7.82E-05	4.95E-04	1.13E-03	1.79E-04	4.63E-03	2.72E-03	-3.97E-04	1.48E-16
	Min	-5.11E-01	-8.41E-02	-9.32E-05	-7.26E-05	2.72E-06	-7.41E-03	3.47E-06	1.75E-06	-2.71E-01	-9.34E-15
	Max	9.98E-01	5.20E-02	5.10E-03	4.83E-02	2.82E-01	3.29E-02	9.38E-01	6.02E-01	2.25E-02	1.56E-14
	Normalized peak height	8.64E-02	1.71E-02	5.05E-02	6.78E-02	3.26E-01	1.85E-01	3.06E-01	3.14E-01	3.75E-01	4.10E-01
	Peak sensitivity value	9.95E-01	4.17E-03	2.36E-05	1.93E-04	1.44E-04	2.88E-05	4.72E-04	3.03E-04	4.54E-05	1.22E-17
	Shapiro-Wilk score	6.564E-01	8.629E-01	6.663E-01	6.257E-01	6.147E-01	6.226E-01	5.758E-01	5.785E-01	6.491E-01	6.035E-01
(triosephosphate isomerase)	Local	4.14E-01	2.95E-03	2.25E-04	1.71E-03	3.00E-02	1.02E-02	1.80E-01	1.06E-01	9.60E-02	8.65E-17
	Min	-1.33E+00	-5.65E-02	-1.66E-03	-1.10E-02	-7.71E-02	3.79E-05	8.85E-05	4.30E-05	1.06E-03	-1.14E-14
	Max	9.96E-01	9.63E-02	5.03E-03	3.22E-02	3.79E-01	5.76E-02	1.28E+00	8.63E-01	9.15E-01	2.15E-14
	Normalized peak height	7.73E-03	2.26E-02	4.42E-02	3.64E-02	5.62E-02	9.90E-03	1.91E-02	2.15E-02	1.02E-02	5.85E-01
	Peak sensitivity value	9.76E-01	3.32E-03	2.45E-05	1.62E-04	-1.61E-04	6.43E-04	3.29E-03	1.34E-03	9.75E-03	-5.93E-18
	Shapiro-Wilk score	9.170E-01	8.676E-01	8.291E-01	8.274E-01	8.580E-01	9.064E-01	8.546E-01	8.512E-01	9.157E-01	4.161E-01
(glyceraldehyde3phosphate dehydrogenase)	Local	7.06E-01	5.02E-03	1.49E-04	1.08E-03	1.51E-02	5.04E-03	8.95E-02	5.25E-02	4.62E-02	1.18E-16
	Min	-1.15E-01	-9.40E-02	2.34E-06	1.57E-05	-1.42E-03	2.18E-05	4.75E-05	2.45E-05	7.33E-05	-7.58E-16
	Max	9.96E-01	2.90E-02	4.82E-03	7.74E-02	2.20E-01	3.55E-02	7.27E-01	4.75E-01	4.10E-01	8.27E-16
	Normalized peak height	8.92E-03	2.57E-02	3.73E-02	7.65E-02	4.88E-02	1.32E-02	2.08E-02	2.25E-02	1.03E-02	2.75E-01
	Peak sensitivity value	1.42E-03	3.85E-03	2.40E-05	2.09E-04	2.19E-05	2.88E-04	1.87E-03	7.37E-04	5.19E-03	1.46E-19
	Shapiro-Wilk score	8.735E-01	8.795E-01	7.300E-01	6.933E-01	7.883E-01	8.717E-01	8.112E-01	8.154E-01	8.397E-01	7.902E-01
(glycerol3phosphate dehydrogenase)	Local	7.06E-01	5.02E-03	1.49E-04	1.08E-03	1.51E-02	5.04E-03	8.95E-02	5.25E-02	4.62E-02	1.18E-16
	Min	-8.84E-02	-5.43E-02	1.63E-06	7.69E-06	-2.10E-03	2.25E-05	7.26E-05	3.91E-05	3.65E-05	-7.75E-16
	Max	9.97E-01	2.89E-02	4.66E-03	5.34E-02	2.29E-01	3.19E-02	7.18E-01	4.76E-01	3.70E-01	7.69E-16
	Normalized peak height	8.83E-03	1.76E-02	3.55E-02	5.38E-02	4.76E-02	1.16E-02	2.11E-02	2.28E-02	9.18E-03	2.37E-01
	Peak sensitivity value	1.11E-03	3.74E-03	2.73E-05	1.95E-04	-1.44E-04	2.61E-04	1.87E-03	7.54E-04	5.03E-03	-6.35E-19
	Shapiro-Wilk score	8.742E-01	8.852E-01	7.314E-01	6.976E-01	7.880E-01	8.723E-01	8.113E-01	8.158E-01	8.481E-01	7.905E-01
(glycerol3phosphate oxidase)	Local	4.14E-01	2.95E-03	2.25E-04	1.71E-03	3.00E-02	1.02E-02	1.80E-01	1.06E-01	9.60E-02	8.65E-17
	Min	-1.29E+00	-5.40E-02	-1.86E-03	-9.86E-03	-1.78E-01	3.28E-05	-4.45E-01	-2.21E-01	9.94E-04	-1.50E-14
	Max	1.70E+00	9.37E-02	4.20E-03	4.66E-02	3.40E-01	6.03E-02	1.32E+00	8.10E-01	9.22E-01	2.09E-14
	Normalized peak height	9.28E-03	2.21E-02	3.80E-02	4.50E-02	5.25E-02	1.04E-02	2.61E-02	2.53E-02	1.02E-02	6.03E-01
	Peak sensitivity value	9.76E-01	3.38E-03	2.21E-05	2.22E-04	1.60E-04	5.45E-04	3.06E-03	1.36E-03	8.82E-03	2.49E-18
	Shapiro-Wilk score	9.176E-01	8.698E-01	8.248E-01	8.216E-01	8.579E-01	9.072E-01	8.558E-01	8.523E-01	9.161E-01	3.636E-01
(pyruvate transport)	Local	7.06E-01	5.02E-03	1.49E-04	1.08E-03	1.51E-02	5.04E-03	8.95E-02	5.25E-02	4.62E-02	-2.22E-16
	Min	-1.34E-01	-5.02E-02	2.70E-06	2.42E-05	-1.56E-03	1.28E-05	3.76E-05	1.60E-05	4.54E-05	-8.88E-16
	Max	9.97E-01	2.92E-02	5.48E-03	5.34E-02	2.29E-01	3.20E-02	7.28E-01	5.01E-01	4.34E-01	7.77E-16
	Normalized peak height	9.07E-03	1.76E-02	4.02E-02	5.28E-02	5.43E-02	1.14E-02	2.06E-02	2.37E-02	1.13E-02	4.04E-01
	Peak sensitivity value	1.88E-03	3.88E-03	3.28E-05	2.11E-04	-5.95E-05	2.84E-04	1.86E-03	7.68E-04	5.46E-03	2.78E-19
	Shapiro-Wilk score	8.745E-01	8.936E-01	7.363E-01	7.021E-01	7.914E-01	8.735E-01	8.130E-01	8.170E-01	8.402E-01	9.119E-01

		(phosphoglyceratekinase)	(pyruvatekinase)	(atputilisation)	(glycerolkinase)	(phosphoglyceratemutase)	(enolase)	(adenylatekinasecytosol)	(adenylatekinaseglycosome)	(3phosphoglyceratetransport)	(gly3pdhapantiporter)	(glyceroltransport)
(glucose transport)	Local	3.73E-04	4.31E-04	8.87E-05	3.95E-04	3.65E-03	6.87E-04	-6.78E-21	1.71E-21	1.07E-08	-4.55E-09	6.60E-06
	Min	1.25E-05	6.86E-07	1.57E-07	-2.49E-05	2.57E-06	8.40E-07	-1.23E-18	-1.67E-15	4.09E-11	-6.18E-06	-2.54E-07
	Max	7.22E-02	3.51E-02	7.86E-03	2.03E-01	1.30E+00	6.70E-02	8.95E-19	2.97E-17	6.36E-07	6.69E-08	1.57E-03
	Normalized peak height	1.61E-01	1.18E-01	1.14E-01	3.99E-01	3.68E-01	1.56E-01	4.95E-01	8.62E-01	6.62E-02	4.07E-01	3.40E-01
	Peak sensitivity value	1.21E-04	1.83E-05	4.09E-06	7.68E-05	6.54E-04	3.43E-05	2.32E-22	-5.50E-20	9.96E-10	1.29E-09	5.32E-07
	Shapiro-Wilk score	5.741E-01	6.084E-01	6.066E-01	6.504E-01	4.055E-01	5.956E-01	4.629E-01	4.316E-02	6.475E-01	6.187E-01	6.912E-01
(hexokinase)	Local	3.73E-04	4.31E-04	8.87E-05	3.95E-04	3.65E-03	6.87E-04	-6.78E-21	1.71E-21	1.07E-08	-4.55E-09	6.60E-06
	Min	1.01E-05	4.38E-07	1.24E-07	-6.53E-05	1.07E-06	4.21E-07	-1.16E-18	-1.83E-15	2.30E-11	-7.51E-06	-2.72E-07
	Max	7.27E-02	3.97E-02	7.13E-03	2.08E-01	1.27E+00	5.95E-02	1.06E-18	4.19E-17	7.56E-07	2.73E-08	1.54E-03
	Normalized peak height	1.60E-01	1.29E-01	1.01E-01	3.83E-01	3.62E-01	1.40E-01	5.01E-01	8.63E-01	8.01E-02	4.31E-01	3.35E-01
	Peak sensitivity value	1.19E-04	2.03E-05	3.69E-06	3.90E-05	6.37E-04	3.02E-05	-1.86E-22	-2.77E-19	1.16E-09	8.99E-10	4.98E-07
	Shapiro-Wilk score	5.768E-01	6.118E-01	6.111E-01	6.510E-01	4.098E-01	6.021E-01	4.701E-01	3.865E-02	6.403E-01	6.197E-01	6.905E-01
(phosphoglycerate isomerase)	Local	3.73E-04	4.31E-04	8.87E-05	3.95E-04	3.65E-03	6.87E-04	-6.78E-21	1.71E-21	1.07E-08	-4.55E-09	6.60E-06
	Min	1.38E-05	5.63E-07	1.37E-07	-2.15E-05	1.96E-06	8.26E-07	-9.23E-19	-7.73E-16	2.59E-11	-5.79E-06	-5.12E-07
	Max	7.29E-02	3.58E-02	7.56E-03	1.89E-01	1.33E+00	6.70E-02	1.42E-18	2.56E-17	5.36E-07	4.50E-08	1.65E-03
	Normalized peak height	1.59E-01	1.18E-01	1.08E-01	3.94E-01	3.69E-01	1.54E-01	4.71E-01	8.16E-01	5.75E-02	4.02E-01	3.28E-01
	Peak sensitivity value	1.23E-04	1.84E-05	3.92E-06	7.32E-05	6.67E-04	3.43E-05	1.04E-21	-3.12E-19	1.37E-09	1.25E-09	3.11E-07
	Shapiro-Wilk score	5.699E-01	6.047E-01	6.036E-01	6.479E-01	4.061E-01	5.914E-01	4.686E-01	6.632E-02	6.438E-01	6.171E-01	6.866E-01
(phophofructokinase)	Local	3.73E-04	4.31E-04	8.87E-05	3.95E-04	3.65E-03	6.87E-04	-6.78E-21	1.71E-21	1.07E-08	-4.55E-09	6.60E-06
	Min	1.12E-05	3.11E-07	7.88E-08	-8.91E-06	7.46E-07	4.17E-07	-1.37E-18	-2.10E-15	3.12E-11	-6.57E-06	-7.45E-08
	Max	8.40E-02	3.75E-02	8.51E-03	1.89E-01	1.27E+00	6.72E-02	1.03E-18	1.12E-16	5.71E-07	3.54E-08	1.31E-03
	Normalized peak height	1.70E-01	1.21E-01	1.19E-01	3.97E-01	3.62E-01	1.53E-01	4.94E-01	8.67E-01	5.92E-02	4.49E-01	3.33E-01
	Peak sensitivity value	1.37E-04	1.91E-05	4.33E-06	8.56E-05	6.34E-04	3.40E-05	-6.96E-22	5.15E-19	1.46E-09	-9.16E-10	5.78E-07
	Shapiro-Wilk score	5.755E-01	6.110E-01	6.078E-01	6.517E-01	4.111E-01	5.980E-01	4.575E-01	3.340E-02	6.463E-01	6.215E-01	6.927E-01
(aldolase)	Local	3.73E-04	4.31E-04	8.87E-05	3.95E-04	3.65E-03	6.87E-04	-6.78E-21	1.71E-21	1.07E-08	-4.55E-09	6.60E-06
	Min	1.25E-05	8.54E-07	1.77E-07	-3.24E-05	1.79E-06	9.07E-07	-8.71E-19	-2.71E-15	1.87E-11	-6.56E-06	-3.57E-07
	Max	8.53E-02	4.61E-02	9.30E-03	1.92E-01	1.35E+00	7.15E-02	3.19E-18	2.84E-17	6.36E-07	2.95E-08	1.58E-03
	Normalized peak height	1.68E-01	1.49E-01	1.32E-01	3.93E-01	3.73E-01	1.64E-01	5.67E-01	8.76E-01	6.61E-02	4.42E-01	3.35E-01
	Peak sensitivity value	1.40E-04	2.39E-05	4.83E-06	6.38E-05	6.76E-04	3.66E-05	9.19E-22	-3.25E-19	9.72E-10	-1.10E-10	4.32E-07
	Shapiro-Wilk score	5.710E-01	6.072E-01	6.059E-01	6.478E-01	4.095E-01	5.931E-01	4.383E-01	2.308E-02	6.440E-01	6.175E-01	6.875E-01
(triosephosphate isomerase)	Local	1.45E-02	1.68E-02	3.45E-03	-4.42E-02	1.42E-01	2.67E-02	-2.64E-19	1.27E-19	4.16E-07	1.10E-06	-7.39E-04
	Min	4.13E-04	1.99E-05	4.82E-06	-4.02E-01	5.96E-05	2.03E-05	-2.21E-18	-6.21E-17	2.53E-11	2.88E-08	-3.56E-03
	Max	7.16E-02	5.88E-02	1.22E-02	-6.88E-04	2.27E+00	9.59E-02	5.77E-18	3.41E-15	2.77E-06	1.01E-05	-1.28E-05
	Normalized peak height	4.72E-03	5.53E-03	5.34E-03	8.03E-03	4.28E-02	6.44E-03	3.45E-01	6.76E-01	1.25E-02	8.65E-03	5.18E-03
	Peak sensitivity value	5.85E-03	1.22E-03	3.17E-04	-7.71E-03	3.47E-03	1.22E-03	-2.00E-21	-1.31E-18	1.41E-09	1.95E-07	-2.06E-04
	Shapiro-Wilk score	9.224E-01	9.146E-01	9.204E-01	8.870E-01	6.510E-01	8.986E-01	7.896E-01	4.117E-02	8.154E-01	8.727E-01	9.090E-01
(glyceraldehyde3phosphate dehydrogenase)	Local	7.20E-03	8.34E-03	1.71E-03	-2.12E-02	7.05E-02	1.33E-02	-1.31E-19	6.21E-20	2.07E-07	5.29E-07	-3.54E-04
	Min	3.06E-04	1.47E-05	4.00E-06	-6.73E-02	6.08E-05	1.84E-05	-9.79E-19	-3.61E-17	4.31E-11	1.29E-09	-1.57E-03
	Max	6.41E-02	3.38E-02	8.24E-03	1.15E-02	9.96E-01	5.67E-02	5.81E-18	2.84E-16	1.49E-06	2.79E-06	2.47E-05
	Normalized peak height	7.32E-03	4.92E-03	5.30E-03	4.61E-03	3.03E-02	5.79E-03	3.50E-01	5.13E-01	7.39E-03	5.33E-03	1.00E-02
	Peak sensitivity value	3.21E-03	7.75E-04	1.56E-04	-4.56E-03	1.55E-03	8.98E-04	1.94E-21	-8.64E-20	7.86E-10	1.14E-07	-1.58E-06
	Shapiro-Wilk score	8.297E-01	9.352E-01	9.396E-01	9.471E-01	5.935E-01	9.167E-01	7.747E-01	1.360E-01	8.551E-01	9.282E-01	8.910E-01
(glycerol3phosphate dehydrogenase)	Local	7.20E-03	8.34E-03	1.71E-03	-2.12E-02	7.05E-02	1.33E-02	-1.31E-19	6.21E-20	2.07E-07	5.29E-07	-3.54E-04
	Min	3.80E-04	2.12E-05	6.65E-06	-5.99E-02	7.17E-05	2.68E-05	-1.01E-18	-9.63E-17	3.90E-11	1.30E-09	-1.52E-03
	Max	6.46E-02	3.62E-02	7.74E-03	9.93E-03	9.98E-01	6.02E-02	3.31E-18	2.06E-16	1.25E-06	2.64E-06	2.96E-05
	Normalized peak height	6.97E-03	5.27E-03	4.96E-03	4.02E-03	3.21E-02	6.38E-03	3.45E-01	4.77E-01	6.36E-03	5.14E-03	1.01E-02
	Peak sensitivity value	4.39E-03	4.74E-04	1.65E-04	-3.64E-03	1.57E-03	8.99E-04	-6.69E-23	-1.19E-19	3.08E-08	1.21E-07	-2.07E-06
	Shapiro-Wilk score	8.294E-01	9.348E-01	9.395E-01	9.475E-01	5.976E-01	9.164E-01	8.066E-01	1.827E-01	8.568E-01	9.303E-01	8.897E-01
(glycerol3phosphate oxidase)	Local	1.45E-02	1.68E-02	3.45E-03	-4.42E-02	1.42E-01	2.67E-02	-2.64E-19	1.27E-19	4.16E-07	1.10E-06	-7.39E-04
	Min	-3.88E-03	-1.46E-03	-3.47E-04	-4.48E-01	-3.22E-03	-1.85E-03	-3.05E-18	-5.86E-17	-9.84E-08	2.61E-08	-3.78E-03
	Max	7.13E-02	6.02E-02	1.31E-02	-6.37E-04	2.23E+00	9.90E-02	1.24E-17	4.35E-15	3.26E-06	9.77E-06	-1.17E-05
	Normalized peak height	5.07E-03	5.59E-03	5.54E-03	8.62E-03	3.96E-02	6.68E-03	3.59E-01	7.63E-01	1.16E-02	7.93E-03	5.12E-03
	Peak sensitivity value	5.40E-03	1.28E-03	2.76E-04	-6.23E-03	4.59E-03	1.93E-03	-3.13E-21	9.86E-19	4.09E-09	2.55E-07	-1.76E-04
	Shapiro-Wilk score	9.222E-01	9.149E-01	9.203E-01	8.867E-01	6.510E-01	8.992E-01	7.115E-01	3.994E-02	8.179E-01	8.733E-01	9.095E-01
(pyruvate transport)	Local	7.20E-03	8.34E-03	1.71E-03	-2.12E-02	7.05E-02	1.33E-02	-1.31E-19	6.21E-20	2.07E-07	5.29E-07	-3.54E-04
	Min	3.26E-04	1.00E-05	2.35E-06	-6.17E-02	3.30E-05	1.29E-05	-1.58E-18	-2.59E-17	2.40E-11	2.06E-09	-1.58E-03
	Max	7.24E-02	4.16E-02	7.66E-03	1.02E-02	9.96E-01	5.96E-02	3.25E-18	1.64E-16	1.35E-06	3.52E-06	1.82E-05
	Normalized peak height	8.07E-03	6.07E-03	4.95E-03	4.11E-03	3.06E-02	6.13E-03	3.46E-01	4.38E-01	6.89E-03	6.76E-03	9.86E-03
	Peak sensitivity value	3.32E-03	5.71E-04	2.28E-04	-4.22E-03	1.53E-03	6.39E-04	-1.57E-21	-5.90E-20	2.05E-09	9.52E-08	-1.76E-06
	Shapiro-Wilk score	8.286E-01	9.342E-01	9.391E-01	9.472E-01	5.964E-01	9.161E-01	7.888E-01	2.523E-01	8.591E-01	9.293E-01	8.909E-01

(phosphoglycerate kinase)																					
Local	7.06E-01	5.02E-03	1.49E-04	1.08E-03	1.51E-02	5.04E-03	8.95E-02	5.25E-02	4.62E-02	2.34E-17	7.20E-03	8.34E-03	1.71E-03	-2.12E-02	7.05E-02	1.33E-02	-1.31E-19	6.21E-20	2.07E-07	5.29E-07	-3.54E-04
Min	-8.62E-02	-4.27E-02	3.14E-06	2.05E-05	-1.41E-03	1.91E-05	6.99E-05	4.06E-05	7.23E-05	-7.08E-16	3.59E-04	1.86E-05	4.08E-06	-6.04E-02	5.98E-05	2.23E-05	-1.31E-18	-2.04E-17	5.19E-11	1.91E-09	-1.58E-03
Max	9.97E-01	3.60E-02	3.34E-03	3.99E-02	2.12E-01	3.47E-02	7.21E-01	4.89E-01	4.25E-01	6.94E-16	6.06E-02	4.06E-02	7.50E-03	9.52E-03	9.95E-01	5.55E-02	1.42E-18	1.51E-16	1.50E-06	2.74E-06	1.50E-05
Normalized peak height	9.06E-03	1.69E-02	2.52E-02	3.98E-02	4.90E-02	1.26E-02	2.07E-02	2.40E-02	1.06E-02	1.55E-01	6.96E-03	5.80E-03	4.80E-03	3.89E-03	3.11E-02	5.76E-03	3.40E-01	3.55E-01	7.58E-03	5.38E-03	1.03E-02
Peak sensitivity value	1.00E-03	3.61E-03	3.15E-05	2.00E-04	-1.66E-05	2.44E-04	1.15E-03	7.75E-04	4.11E-03	6.41E-19	4.49E-03	4.85E-04	1.13E-04	-4.28E-03	1.55E-03	6.05E-04	-1.11E-21	8.21E-20	2.30E-09	9.65E-08	-1.69E-06
Shapiro-Wilk score	8.731E-01	9.003E-01	7.349E-01	7.045E-01	7.895E-01	8.721E-01	8.116E-01	8.158E-01	8.481E-01	8.483E-01	8.303E-01	9.342E-01	9.395E-01	9.468E-01	5.958E-01	9.157E-01	8.138E-01	2.755E-01	8.590E-01	9.297E-01	8.883E-01

(pyruvate kinase)																					
Local	7.06E-01	5.02E-03	1.49E-04	1.08E-03	1.51E-02	5.04E-03	8.95E-02	5.25E-02	4.62E-02	-2.12E-16	7.20E-03	8.34E-03	1.71E-03	-2.12E-02	7.05E-02	1.33E-02	-1.31E-19	6.21E-20	2.07E-07	5.29E-07	-3.54E-04
Min	-7.33E-02	-3.38E-02	1.97E-06	8.67E-06	-1.50E-03	2.08E-05	1.01E-04	4.34E-05	1.03E-04	-8.44E-16	3.28E-04	2.81E-05	6.63E-06	-6.14E-02	9.97E-05	3.51E-05	-9.80E-19	-2.29E-17	3.70E-11	2.11E-09	-1.41E-03
Max	9.96E-01	2.73E-02	4.39E-03	3.65E-02	2.22E-01	3.67E-02	7.14E-01	4.85E-01	4.18E-01	9.20E-16	6.68E-02	3.54E-02	7.33E-03	1.41E-02	9.96E-01	6.58E-02	6.86E-18	3.03E-16	1.72E-06	2.37E-06	2.94E-05
Normalized peak height	8.54E-03	1.33E-02	3.32E-02	3.69E-02	5.21E-02	1.32E-02	2.07E-02	2.33E-02	1.05E-02	3.44E-01	7.47E-03	5.04E-03	4.79E-03	4.36E-03	3.12E-02	6.84E-03	3.59E-01	5.08E-01	8.93E-03	4.53E-03	9.51E-03
Peak sensitivity value	1.09E-03	4.17E-03	3.05E-05	2.09E-04	-4.68E-05	2.23E-04	1.89E-03	7.70E-04	7.00E-03	-5.13E-20	3.82E-03	5.76E-04	1.35E-04	-3.53E-03	1.59E-03	7.25E-04	-3.53E-21	1.04E-19	8.96E-10	1.22E-07	-1.55E-06
Shapiro-Wilk score	8.736E-01	9.006E-01	7.298E-01	7.023E-01	7.876E-01	8.714E-01	8.108E-01	8.140E-01	8.358E-01	9.674E-01	8.278E-01	9.338E-01	9.386E-01	9.465E-01	5.963E-01	9.154E-01	7.530E-01	1.789E-01	8.592E-01	9.304E-01	8.914E-01

(atp utilisation)																					
Local	7.06E-01	5.02E-03	1.49E-04	1.08E-03	1.51E-02	5.04E-03	8.95E-02	5.25E-02	4.62E-02	2.20E-21	7.20E-03	8.34E-03	1.71E-03	-2.12E-02	7.05E-02	1.33E-02	-1.31E-19	6.21E-20	2.07E-07	5.29E-07	-3.54E-04
Min	-1.10E-01	-7.93E-02	2.06E-06	1.60E-05	-2.20E-03	2.27E-05	7.55E-05	4.19E-05	5.70E-05	-5.17E-12	2.82E-04	1.88E-05	5.58E-06	-6.24E-02	7.43E-05	2.51E-05	-1.10E-18	-3.18E-17	4.66E-11	2.29E-09	-1.48E-03
Max	9.97E-01	2.66E-02	4.34E-03	7.05E-02	2.18E-01	3.31E-02	7.18E-01	5.27E-01	4.02E-01	4.45E-12	6.42E-02	3.83E-02	7.83E-03	9.56E-03	9.96E-01	6.13E-02	4.47E-18	1.69E-16	1.32E-06	2.35E-06	1.90E-05
Normalized peak height	9.22E-03	2.28E-02	3.23E-02	6.87E-02	4.90E-02	1.18E-02	2.13E-02	2.63E-02	1.03E-02	8.38E-01	7.10E-03	5.47E-03	5.12E-03	4.24E-03	3.03E-02	6.40E-03	3.46E-01	4.20E-01	6.74E-03	4.69E-03	9.93E-03
Peak sensitivity value	1.25E-03	3.78E-03	2.59E-05	1.92E-04	-1.10E-04	2.38E-04	1.15E-03	8.32E-04	5.08E-03	3.42E-15	4.15E-03	4.21E-04	1.50E-04	-5.52E-03	1.57E-03	6.08E-04	1.37E-21	-8.16E-20	3.51E-08	1.07E-07	-1.19E-06
Shapiro-Wilk score	8.740E-01	8.925E-01	7.318E-01	6.988E-01	7.891E-01	8.719E-01	8.106E-01	8.154E-01	8.406E-01	1.089E-01	8.313E-01	9.333E-01	9.396E-01	9.470E-01	5.983E-01	9.159E-01	7.757E-01	1.972E-01	8.615E-01	9.299E-01	8.926E-01

(glycerol kinase)																					
Local	9.22E+00	6.57E-02	-2.07E-03	-1.73E-02	-4.20E-01	-1.47E-01	-2.56E+00	-1.50E+00	-1.41E+00	1.05E-15	-2.06E-01	-2.39E-01	-4.91E-02	6.53E-01	-2.02E+00	-3.80E-01	3.75E-18	-1.82E-18	-5.91E-06	-1.61E-05	1.09E-02
Min	-3.39E+00	-5.75E-01	-2.27E-02	-1.81E-01	-3.40E+00	-8.92E-01	-1.74E+01	-1.04E+01	-1.17E+01	-1.07E-13	-1.01E+00	-5.45E-01	-1.32E-01	5.08E-02	-2.96E+01	-9.36E-01	-8.86E-17	-1.05E-14	-6.70E-05	-1.34E-04	1.38E-04
Max	3.34E+01	4.07E-01	1.27E-02	1.19E-01	1.07E+00	3.65E-02	2.44E+00	2.07E+00	-9.27E-02	1.04E-13	1.30E-01	7.70E-02	1.33E-02	1.14E+00	3.70E+00	1.20E-01	2.72E-17	2.68E-16	1.01E-06	-5.72E-07	6.17E-02
Normalized peak height	1.02E-02	1.19E-02	1.29E-02	1.24E-02	8.20E-03	7.53E-03	1.18E-02	1.07E-02	1.25E-02	4.67E-01	9.03E-03	1.07E-02	1.16E-02	4.60E-03	5.86E-02	1.19E-02	3.93E-01	4.11E-01	6.49E-02	5.72E-03	7.79E-03
Peak sensitivity value	-2.04E-01	2.38E-02	-1.45E-04	-2.54E-03	-1.52E-01	-8.00E-02	1.63E-02	1.45E-02	-1.39E+00	-9.03E-17	6.62E-03	2.02E-03	5.84E-04	9.61E-01	1.22E-02	3.55E-03	5.09E-20	4.76E-18	2.33E-08	-8.93E-06	2.02E-03
Shapiro-Wilk score	9.687E-01	8.571E-01	9.451E-01	9.515E-01	9.605E-01	9.205E-01	9.287E-01	9.249E-01	9.147E-01	3.428E-01	9.023E-01	9.422E-01	9.376E-01	9.168E-01	8.098E-01	9.475E-01	7.798E-01	1.003E-01	8.326E-01	8.940E-01	8.892E-01

(phosphoglycerate mutase)																					
Local	7.06E-01	5.02E-03	1.49E-04	1.08E-03	1.51E-02	5.04E-03	8.95E-02	5.25E-02	4.62E-02	2.10E-17	7.20E-03	8.34E-03	1.71E-03	-2.12E-02	7.05E-02	1.33E-02	-1.31E-19	6.21E-20	2.07E-07	5.29E-07	-3.54E-04
Min	-5.85E-02	-3.22E-02	1.75E-06	1.65E-05	-1.40E-03	1.74E-05	7.58E-05	4.24E-05	7.65E-05	-7.45E-16	2.36E-04	2.08E-05	3.88E-06	-6.37E-02	5.12E-05	2.13E-05	-1.26E-18	-4.80E-17	4.00E-11	1.94E-09	-1.52E-03
Max	9.96E-01	2.34E-02	3.98E-03	3.61E-02	2.05E-01	3.59E-02	7.16E-01	4.86E-01	4.16E-01	7.35E-16	5.80E-02	3.46E-02	7.27E-03	1.10E-02	9.97E-01	5.80E-02	9.91E-18	2.31E-16	1.34E-06	2.79E-06	3.20E-05
Normalized peak height	8.34E-03	1.22E-02	3.01E-02	3.65E-02	4.82E-02	1.28E-02	2.02E-02	2.30E-02	1.05E-02	1.96E-01	6.51E-03	4.99E-03	4.52E-03	4.16E-03	3.00E-02	6.05E-03	3.62E-01	4.64E-01	6.87E-03	5.27E-03	1.02E-02
Peak sensitivity value	1.11E-03	3.71E-03	2.76E-05	2.15E-04	-5.99E-05	2.51E-04	1.15E-03	7.71E-04	5.27E-03	2.34E-19	3.79E-03	4.53E-04	1.24E-04	-4.46E-03	1.55E-03	8.04E-04	-4.31E-22	1.14E-19	2.05E-09	9.55E-08	-1.41E-06
Shapiro-Wilk score	8.731E-01	9.030E-01	7.310E-01	7.018E-01	7.872E-01	8.712E-01	8.108E-01	8.152E-01	8.424E-01	8.579E-01	8.282E-01	9.344E-01	9.389E-01	9.468E-01	5.976E-01	9.165E-01	6.243E-01	2.253E-01	8.613E-01	9.293E-01	8.906E-01

(enolase)																					
Local	7.06E-01	5.02E-03	1.49E-04	1.08E-03	1.51E-02	5.04E-03	8.95E-02	5.25E-02	4.62E-02	2.10E-17	7.20E-03	8.34E-03	1.71E-03	-2.12E-02	7.05E-02	1.33E-02	-1.31E-19	6.21E-20	2.07E-07	5.29E-07	-3.54E-04
Min	-8.56E-02	-6.46E-02	2.78E-06	1.38E-05	-2.45E-03	2.67E-05	7.73E-05	4.62E-05	9.07E-05	-7.27E-16	2.98E-04	2.27E-05	5.30E-06	-6.37E-02	7.77E-05	2.45E-05	-1.03E-18	-1.61E-17	5.26E-11	2.21E-09	-1.49E-03
Max	9.97E-01	2.61E-02	4.48E-03	5.83E-02	2.26E-01	3.48E-02	7.32E-01	4.80E-01	4.47E-01	6.88E-16	7.07E-02	3.45E-02	7.65E-03	1.12E-02	9.95E-01	6.75E-02	1.72E-17	1.34E-16	1.82E-06	2.74E-06	2.07E-05
Normalized peak height	8.47E-03	1.96E-02	3.40E-02	5.68E-02	5.20E-02	1.20E-02	2.05E-02	2.25E-02	1.09E-02	1.95E-01	7.83E-03	4.85E-03	4.75E-03	4.08E-03	3.04E-02	6.78E-03	3.96E-01	4.19E-01	9.16E-03	5.16E-03	9.72E-03
Peak sensitivity value	4.46E-04	3.82E-03	2.74E-05	2.18E-04	-4.89E-05	2.53E-04	1.18E-03	7.66E-04	4.78E-03	-2.34E-19	3.01E-03	1.00E-03	2.15E-04	-4.46E-03	1.57E-03	1.07E-03	-3.21E-21	-3.33E-20	6.46E-08	9.67E-08	-1.22E-06
Shapiro-Wilk score	8.742E-01	8.858E-01	7.356E-01	6.995E-01	7.907E-01	8.734E-01	8.127E-01	8.172E-01	8.364E-01	8.582E-01	8.283E-01	9.341E-01	9.391E-01	9.475E-01	5.972E-01	9.156E-01	4.729E-01	2.782E-01	8.573E-01	9.286E-01	8.896E-01

(adenylate kinase cytosol)																					
Local	INF	INF	INF	INF	INF	INF	INF	INF	INF	INF	INF	INF	INF	INF	INF	INF	INF	INF	INF	INF	INF
Min	-INF	-INF	-INF	-INF	-INF	-INF	-INF	-INF	-INF	-INF	-INF	-INF	-INF	-INF	-INF	-INF	-INF	-INF	-INF	-INF	-INF
Max	INF	INF	INF	INF	INF	INF	INF	INF	INF	INF	INF	INF	INF	INF	INF	INF	INF	INF	INF	INF	INF
Normalized peak height	1.67E-01	1.67E-01	1.67E-01	1.67E-01	8.33E-01	1.67E-01	1.67E-01	1.67E-01	1.67E-01	1.67E-01	1.67E-01	1.67E-01	1.67E-01	1.67E-01	1.67E-01	1.67E-01	1.67E-01	1.67E-01	1.67E-01	1.67E-01	1.67E-01
Peak sensitivity value	3.27E-05	-1.29E-02	-2.91E-02	-1.24E-02	-9.46E-07	-7.97E-05	-5.14E-03	-6.98E-03	-6.38E-04	-1.43E-05	-6.96E-02	-4.95E-02	-3.29E-02	-2.70E-03	-1.93E-02	-2.54E-02	-2.70E-12	-2.99E-13	-1.02E-02	-1.20E-03	-6.03E-06
Shapiro-Wilk score	8.207E-01	8.800E-01	8.434E-01	9.154E-01	4.961E-01	8.310E-01	7.631E-01	8.694E-01	8.275E-01	9.164E-01	7.066E-01	8.550E-01	8.928E-01	9.087E-01	9.069E-01	9.566E-01	8.750E-01	6.011E-01	9.371E-01	8.455E-01	9.560E-01

(adenylate kinase glycosome)																					
Local	-INF	-INF	-INF	-INF	-INF	-INF	-INF	-INF	-INF	-INF	-INF	-INF	-INF	-INF	-INF	-INF	-INF	-INF	-INF	-INF	-INF
Min	-INF	-INF	-INF	-INF	-INF	-INF	-INF	-INF	-INF	-INF	-INF	-INF	-INF	-INF	-INF	-INF	-INF	-INF	-INF	-INF	-INF
Max	INF	INF	INF	INF	INF	INF	INF	INF	INF	INF	INF	INF	INF	INF	INF	INF	INF	INF	INF	INF	INF
Normalized peak height	1.01E-01	4.59E-02	4.07E-02	4.80E-02	1.46E-01	9.40E-02	1.35E-01	1.39E-01	4.27E-01	3.49E-01	5.02E-02	7.12E-02	7.82E-02	3.03E-01	1.68E-01	6.87E-02	3.92E-01	2.40E-02	9.72E-02	1.82E-01	2.94E-01
Peak sensitivity value	-8.57E-03	-1.81E-02	-2.99E-03	1.77E-02	-2.05E-03	1.77E-03	2.17E-02	-5.16E-03	2.56E-03	1.25E-07	1.85E-02	-5.90E-04	2.50E-04	-4.73E-04	4.31E-02	-1.73E-03	4.89E-16	-1.80E-13	1.21E-02	-2.09E-04	-7.30E-06
Shapiro-Wilk score	8.030E-01	8.102E-01	8.266E-01	8.179E-01	7.105E-01	7.340E-01	7.202E-01	7.142E-01	5.613E-01	5.942E-01	8.107E-01	8.652E-01	8.609E-01	6.532E-01	5.865E-01	8.439E-01	5.223E-01	8.952E-01	6.612E-01	6.901E-01	6.517E-01

(3phosphoglycerate transport)																					
Local	7.06E-01	5.02E-03	1.49E-04	1.08E-03	1.51E-02	5.04E-03	8.95E-02	5.25E-02	4.62E-02	2.34E-17	7.20E-03	8.34E-03	1.71E-03	-2.12E-02	7.05E-02	1.33E-02	-1.31E-19	6.20E-20	2.07E-07	5.29E-07	-3.54E-04
Min	-7.37E-02	-5.28E-02	1.90E-06	1.65E-05	-1.99E-03	2.06E-05	5.64E-05	2.49E-05	4.77E-05	-6.62E-16	3.40E-04	1.87E-05	3.66E-06	-6.85E-02	4.62E-05	1.93E-05	-1.37E-18	-2.84E-17	-2.15E-08	2.27E-09	-1.99E-03
Max	9.97E-01	2.81E-02	3.81E-03	5.41E-02	2.18E-01	3.57E-02	7.00E-01	4.82E-01	4.34E-01	7.66E-16	6.36E-02	4.32E-02	7.89E-03	1.06E-02	9.96E-01	5.62E-02	2.03E-16	2.07E-16	1.97E-06	2.55E-06	3.57E-05
Normalized peak height	8.80E-03	1.72E-02	2.95E-02	5.27E-02	5.02E-02	1.32E-02	2.09E-02	2.36E-02	1.15E-02	1.70E-01	6.94E-03	6.35E-03	5.04E-03	4.27E-03	3.21E-02	5.91E-03	8.43E-01	4.85E-01	9.77E-03	5.05E-03	1.24E-02
Peak sensitivity value	6.86E-04	3.77E-03	2.28E-05	2.06E-04	-1.19E-04	2.88E-04	1.11E-03	7.49E-04	5.03E-03	-1.69E-19	3.54E-03	5.15E-04	1.26E-04	-4.86E-03	1.54E-03	8.33E-04	-3.69E-20	1.34E-20	5.52E-08	1.00E-07	-3.76E-06
Shapiro-Wilk score	8.738E-01	8.977E-01	7.320E-01	6.992E-01	7.904E-01	8.731E-01	8.128E-01	8.165E-01	8.376E-01	8.479E-01	8.317E-01	9.335E-01	9.385E-01	9.465E-01	5.954E-01	9.151E-01	1.377E-02	2.244E-01	8.554E-01	9.300E-01	8.893E-01

(gly3p dhap antiporter)																					
Local	4.14E-01	2.95E-03	2.25E-04	1.71E-03	3.00E-02	1.02E-02	1.80E-01	1.06E-01	9.60E-02	8.65E-17	1.45E-02	1.68E-02	3.45E-03	-4.42E-02	1.42E-01	2.67E-02	-2.64E-19	1.27E-19	4.16E-07	1.10E-06	-7.39E-04
Min	-1.19E+00	-7.05E-02	-1.43E-03	-9.68E-03	-9.50E-02	4.53E-05	1.04E-04	5.95E-05	1.06E-03	-1.19E-14	5.16E-04	3.36E-05	8.18E-06	-3.73E-01	8.95E-05	3.96E-05	-2.11E-18	-4.91E-17	7.81E-11	2.62E-08	-3.45E-03
Max	9.95E-01	9.23E-02	5.26E-03	4.39E-02	3.62E-01	5.79E-02	1.33E+00	8.49E-01	8.99E-01	6.07E-15	6.90E-02	5.67E-02	1.33E-02	-6.83E-04	2.15E+00	8.93E-02	1.53E-17	3.32E-15	2.79E-06	1.04E-05	-1.04E-05
Normalized peak height	7.17E-03	2.49E-02	4.27E-02	4.41E-02	5.69E-02	1.03E-02	2.05E-02	2.11E-02	9.80E-03	5.28E-01	4.54E-03	5.26E-03	5.53E-03	7.12E-03	3.96E-02	6.04E-03	3.68E-01	7.33E-01	1.26E-02	8.39E-03	4.69E-03
Peak sensitivity value	9.78E-01	3.16E-03	2.13E-05	1.50E-04	-1.19E-04	4.21E-04	2.10E-03	2.18E-03	1.32E-02	8.25E-19	6.10E-03	1.25E-03	2.95E-04	-7.94E-03	3.31E-03	1.33E-03	5.48E-21	-2.99E-19	1.47E-09	1.98E-07	-1.64E-04
Shapiro-Wilk score	9.176E-01	8.664E-01	8.303E-01	8.280E-01	8.575E-01	9.064E-01	8.551E-01	8.516E-01	9.183E-01	4.676E-01	9.200E-01	9.162E-01	9.210E-01	8.869E-01	6.513E-01	9.003E-01	6.881E-01	3.307E-02	8.148E-01	8.736E-01	9.103E-01

(glycerol transport)																					
Local	9.22E+00	6.57E-02	-2.07E-03	-1.73E-02	-4.20E-01	-1.47E-01	-2.56E+00	-1.50E+00	-1.41E+00	1.05E-15	-2.06E-01	-2.39E-01	-4.91E-02	6.53E-01	-2.02E+00	-3.80E-01	3.75E-18	-1.82E-18	-5.91E-06	-1.61E-05	1.09E-02
Min	-4.18E+00	-5.31E-01	-3.09E-02	-2.21E-01	-3.33E+00	-8.08E-01	-1.74E+01	-9.49E+00	-1.16E+01	-1.41E-13	-1.02E+00	-5.95E-01	-1.16E-01	5.46E-02	-2.97E+01	-9.36E-01	-1.26E-16	-1.82E-14	-7.82E-05	-1.25E-04	2.30E-04
Max	3.35E+01	4.06E-01	1.44E-02	1.83E-01	1.15E+00	3.94E-02	2.93E+00	1.82E+00	-1.37E-01	9.40E-14	1.42E-01	6.62E-02	1.54E-02	1.16E+00	4.79E+00	1.27E-01	3.13E-17	3.12E-16	1.10E-06	-7.51E-07	6.25E-02
Normalized peak height	1.04E-02	1.11E-02	1.52E-02	1.60E-02	8.15E-03	6.91E-03	1.25E-02	9.67E-03	1.22E-02	5.56E-01	9.22E-03	1.17E-02	1.09E-02	4.88E-03	5.90E-02	1.23E-02	4.24E-01	5.42E-01	6.73E-02	5.31E-03	7.94E-03
Peak sensitivity value	-1.59E-01	2.39E-02	-1.78E-04	-3.33E-03	-9.12E-02	-8.73E-02	1.18E-02	1.42E-02	-1.40E+00	-2.13E-17	4.79E-03	2.41E-03	5.41E-04	9.58E-01	1.10E-02	3.40E-03	2.61E-20	6.79E-18	3.24E-08	-8.16E-06	2.25E-03
Shapiro-Wilk score	9.685E-01	8.601E-01	9.379E-01	9.456E-01	9.623E-01	9.187E-01	9.278E-01	9.297E-01	9.170E-01	3.568E-01	9.017E-01	9.421E-01	9.371E-01	9.167E-01	8.021E-01	9.482E-01	7.683E-01	5.527E-02	8.310E-01	8.942E-01	8.866E-01

Table S15: Flux control coefficients for *Trypanosoma brucei* model with parameter variation of ±40%

		(glucosetransport)	(hexokinase)	(phosphoglycerateisomerase)	(phophofructokinase)	(aldolase)	(triosephosphateisomerase)	(glyceraldehyde3phosphatedehydrogenase)	(glycerol3phosphatedehydrogenase)	(glycerol3phosphateoxidase)	(pyruvatetransport)	(phosphoglyceratekinase)	(pyruvatekinase)	(atputilisation)	(glycerolkinase)	(phosphoglyceratemutase)	(enolase)	(adenylatekinasecytosol)	(adenylatekinaseglycosome)	(3phosphoglyceratetransport)	(gly3pdhapantiporter)	(glyceroltransport)
(glucose transport)	Local	9.79E-01	6.97E-03	7.82E-05	4.95E-04	1.13E-03	1.79E-04	4.63E-03	2.72E-03	-3.97E-04	-1.47E-18	3.73E-04	4.31E-04	8.87E-05	3.95E-04	3.65E-03	6.87E-04	-6.78E-21	1.71E-21	1.07E-08	-4.55E-09	6.60E-06
	Min	-1.13E+00	-2.63E-01	-3.04E-04	-1.67E-03	6.59E-07	-9.20E-03	-3.47E-04	-2.48E-04	-4.01E-01	-3.12E-13	3.12E-06	1.13E-07	1.94E-08	-6.14E-05	2.65E-07	9.08E-08	-1.64E-18	-3.96E-15	1.44E-12	-1.24E-05	-8.87E-07
	Max	9.99E-01	7.23E-02	1.17E-02	1.70E-01	4.55E-01	4.03E-02	1.03E+00	7.11E-01	2.64E-01	1.22E-13	1.01E-01	5.51E-02	1.19E-02	2.57E-01	2.20E+00	1.13E-01	2.34E-18	5.49E-17	1.17E-06	2.93E-07	2.66E-03
	Normalized peak height	1.18E-01	3.40E-02	1.15E-01	2.13E-01	3.74E-01	2.23E-01	3.04E-01	3.16E-01	4.07E-01	9.55E-01	2.24E-01	2.28E-01	2.20E-01	3.87E-01	4.49E-01	2.75E-01	5.73E-01	8.20E-01	1.82E-01	2.82E-01	3.59E-01
	Peak sensitivity value	9.96E-01	3.79E-03	1.29E-05	1.34E-04	2.28E-04	-1.64E-05	1.71E-04	1.08E-04	-2.99E-06	6.67E-17	5.35E-05	2.76E-05	5.99E-06	6.71E-05	1.10E-03	5.68E-05	2.55E-22	7.08E-19	5.87E-10	6.00E-09	4.45E-07
	Shapiro-Wilk score	7.138E-01	8.486E-01	6.240E-01	5.592E-01	6.927E-01	6.982E-01	6.458E-01	6.422E-01	7.506E-01	5.974E-02	5.970E-01	5.996E-01	6.010E-01	7.449E-01	4.878E-01	5.656E-01	4.330E-01	3.161E-02	6.352E-01	7.096E-01	6.700E-01
(hexokinase)	Local	9.79E-01	6.97E-03	7.82E-05	4.95E-04	1.13E-03	1.79E-04	4.63E-03	2.72E-03	-3.97E-04	-1.47E-18	3.73E-04	4.31E-04	8.87E-05	3.95E-04	3.65E-03	6.87E-04	-6.78E-21	1.71E-21	1.07E-08	-4.55E-09	6.60E-06
	Min	-9.97E-01	-3.72E-01	-4.70E-04	-2.53E-03	8.96E-07	-1.01E-02	-2.75E-04	-1.22E-04	-3.80E-01	-3.60E-13	3.66E-06	9.54E-08	2.13E-08	-8.58E-05	1.82E-07	8.53E-08	-2.41E-18	-2.28E-15	4.12E-12	-1.02E-05	-1.92E-06
	Max	9.99E-01	7.47E-02	1.20E-02	2.67E-01	3.56E-01	4.26E-02	1.13E+00	7.48E-01	2.16E-01	2.14E-13	1.17E-01	5.22E-02	1.49E-02	3.02E-01	1.79E+00	1.12E-01	1.21E-17	4.54E-17	1.12E-06	1.53E-07	2.79E-03
	Normalized peak height	1.11E-01	4.51E-02	1.19E-01	2.05E-01	3.54E-01	3.19E-01	3.19E-01	3.38E-01	4.00E-01	9.68E-01	2.53E-01	2.22E-01	2.42E-01	3.89E-01	4.23E-01	2.71E-01	6.52E-01	7.90E-01	1.73E-01	3.93E-01	3.21E-01
	Peak sensitivity value	9.96E-01	3.52E-03	1.24E-05	3.07E-04	1.79E-04	4.59E-06	2.92E-04	2.52E-04	-6.42E-06	-3.44E-17	6.23E-05	2.62E-05	7.48E-06	6.51E-05	8.94E-04	5.59E-05	-5.60E-21	-2.20E-20	5.62E-10	3.22E-09	-5.26E-07
	Shapiro-Wilk score	7.147E-01	8.485E-01	6.244E-01	5.592E-01	6.959E-01	7.023E-01	6.487E-01	6.452E-01	7.519E-01	5.293E-02	5.992E-01	6.015E-01	6.014E-01	7.470E-01	4.894E-01	5.726E-01	3.256E-01	5.129E-02	6.352E-01	7.126E-01	6.755E-01
(phosphoglycerate isomerase)	Local	9.79E-01	6.97E-03	7.82E-05	4.95E-04	1.13E-03	1.79E-04	4.63E-03	2.72E-03	-3.97E-04	-1.47E-18	3.73E-04	4.31E-04	8.87E-05	3.95E-04	3.65E-03	6.87E-04	-6.78E-21	1.71E-21	1.07E-08	-4.55E-09	6.60E-06
	Min	-1.12E+00	-1.85E-01	-3.28E-04	-1.43E-03	6.86E-07	-1.01E-02	-6.04E-04	-2.49E-04	-3.22E-01	-2.89E-13	4.27E-06	9.32E-08	3.20E-08	-1.23E-04	3.19E-07	1.29E-07	-2.47E-18	-2.67E-15	2.13E-12	-9.55E-06	-1.47E-06
	Max	9.99E-01	8.94E-02	8.52E-03	1.30E-01	4.03E-01	4.27E-02	1.41E+00	9.99E-01	1.79E-01	3.25E-13	9.36E-02	5.38E-02	1.17E-02	2.96E-01	1.72E+00	9.04E-02	5.71E-18	4.39E-17	1.00E-06	2.09E-07	2.48E-03
	Normalized peak height	1.16E-01	2.78E-02	8.86E-02	1.53E-01	3.63E-01	2.40E-01	3.14E-01	3.54E-01	3.96E-01	9.63E-01	2.10E-01	2.26E-01	2.16E-01	3.78E-01	4.18E-01	2.50E-01	6.03E-01	7.97E-01	1.58E-01	4.30E-01	3.29E-01
	Peak sensitivity value	9.96E-01	3.63E-03	1.29E-05	8.33E-05	2.02E-04	2.78E-05	1.02E-04	2.51E-04	-3.15E-05	-1.19E-16	5.11E-05	2.70E-05	5.87E-06	2.53E-05	8.60E-04	4.53E-05	-3.00E-21	-7.94E-19	5.03E-10	-5.18E-10	-2.29E-07
	Shapiro-Wilk score	7.161E-01	8.557E-01	6.279E-01	5.815E-01	6.955E-01	7.013E-01	6.498E-01	6.464E-01	7.532E-01	4.819E-02	6.008E-01	6.013E-01	6.026E-01	7.476E-01	4.889E-01	5.699E-01	4.137E-01	4.044E-02	6.371E-01	7.124E-01	6.755E-01
(phophofructokinase)	Local	9.79E-01	6.97E-03	7.82E-05	4.95E-04	1.13E-03	1.79E-04	4.63E-03	2.72E-03	-3.97E-04	-1.47E-18	3.73E-04	4.31E-04	8.87E-05	3.95E-04	3.65E-03	6.87E-04	-6.78E-21	1.71E-21	1.07E-08	-4.55E-09	6.60E-06
	Min	-1.66E+00	-2.99E-01	-4.80E-04	-3.13E-03	6.03E-07	-9.63E-03	-1.72E-04	-9.51E-05	-3.68E-01	-2.27E-13	5.08E-06	6.37E-08	1.67E-08	-8.03E-05	1.48E-07	4.99E-08	-1.68E-18	-1.68E-14	3.70E-12	-9.94E-06	-9.51E-07
	Max	9.99E-01	7.90E-02	1.20E-02	2.00E-01	3.71E-01	4.35E-02	1.55E+00	9.06E-01	9.42E-02	2.45E-13	9.87E-02	5.86E-02	1.62E-02	4.03E-01	1.59E+00	1.04E-01	2.49E-18	5.22E-17	8.57E-07	1.54E-07	2.20E-03
	Normalized peak height	1.34E-01	3.84E-02	1.21E-01	2.05E-01	3.59E-01	3.36E-01	3.61E-01	3.62E-01	3.54E-01	9.58E-01	2.23E-01	2.34E-01	2.52E-01	4.09E-01	4.11E-01	2.65E-01	5.77E-01	9.02E-01	1.37E-01	4.39E-01	3.40E-01
	Peak sensitivity value	9.95E-01	3.52E-03	1.29E-05	2.20E-04	1.86E-04	1.15E-05	6.02E-04	3.58E-04	1.33E-04	6.20E-17	5.45E-05	2.93E-05	8.11E-06	1.21E-04	7.93E-04	5.21E-05	1.09E-22	-6.62E-18	4.32E-10	-2.03E-09	1.49E-07
	Shapiro-Wilk score	7.153E-01	8.529E-01	6.238E-01	5.688E-01	6.936E-01	6.999E-01	6.484E-01	6.445E-01	7.517E-01	5.668E-02	5.981E-01	6.018E-01	6.019E-01	7.462E-01	4.880E-01	5.694E-01	4.270E-01	5.609E-03	6.393E-01	7.104E-01	6.735E-01
(aldolase)	Local	9.79E-01	6.97E-03	7.82E-05	4.95E-04	1.13E-03	1.79E-04	4.63E-03	2.72E-03	-3.97E-04	1.48E-16	3.73E-04	4.31E-04	8.87E-05	3.95E-04	3.65E-03	6.87E-04	-6.78E-21	1.71E-21	1.07E-08	-4.55E-09	6.60E-06
	Min	-8.92E-01	-1.92E-01	-6.24E-04	-3.59E-03	3.60E-07	-9.04E-03	-4.80E-04	-2.55E-04	-3.62E-01	-1.18E-14	4.26E-06	7.18E-08	4.24E-08	-6.79E-05	2.82E-07	9.05E-08	-2.08E-18	-1.31E-15	2.52E-12	-9.87E-06	-6.44E-07
	Max	9.99E-01	7.77E-02	1.06E-02	1.19E-01	3.71E-01	4.99E-02	1.28E+00	8.14E-01	1.06E-01	1.03E-14	1.08E-01	5.74E-02	1.87E-02	2.55E-01	1.95E+00	9.34E-02	1.80E-18	4.49E-17	8.91E-07	1.24E-07	2.67E-03
	Normalized peak height	1.08E-01	2.77E-02	1.03E-01	1.58E-01	3.61E-01	3.40E-01	3.19E-01	3.34E-01	4.04E-01	5.94E-01	2.42E-01	2.36E-01	2.73E-01	3.87E-01	4.37E-01	2.58E-01	5.46E-01	7.65E-01	1.45E-01	4.39E-01	3.68E-01
	Peak sensitivity value	9.96E-01	3.26E-03	2.16E-05	1.39E-04	1.86E-04	7.89E-06	1.61E-04	1.52E-04	-1.38E-04	3.77E-19	5.81E-05	2.88E-05	9.38E-06	5.98E-05	9.77E-04	4.68E-05	-1.32E-21	-4.42E-19	4.48E-10	-1.46E-09	6.90E-07
	Shapiro-Wilk score	7.125E-01	8.536E-01	6.290E-01	5.744E-01	6.926E-01	6.965E-01	6.466E-01	6.429E-01	7.491E-01	4.919E-01	5.957E-01	5.994E-01	5.993E-01	7.440E-01	4.838E-01	5.655E-01	4.450E-01	8.570E-02	6.378E-01	7.093E-01	6.742E-01
(triosephosphate isomerase)	Local	4.14E-01	2.95E-03	2.25E-04	1.71E-03	3.00E-02	1.02E-02	1.80E-01	1.06E-01	9.60E-02	8.65E-17	1.45E-02	1.68E-02	3.45E-03	-4.42E-02	1.42E-01	2.67E-02	-2.64E-19	1.27E-19	4.16E-07	1.10E-06	-7.39E-04
	Min	-1.74E+00	-1.14E-01	-3.28E-03	-2.87E-02	-2.36E-01	1.21E-05	-3.11E-01	-1.86E-01	3.76E-04	-1.43E-14	-4.32E-02	-1.58E-02	-2.88E-03	-6.33E-01	-3.69E+00	-3.29E-02	-3.58E-18	-9.27E-17	-1.97E-07	1.28E-08	-6.15E-03
	Max	4.41E+00	1.88E-01	7.68E-03	1.23E-01	5.23E-01	7.82E-02	1.49E+00	1.03E+00	1.26E+00	9.81E-15	1.03E-01	9.34E-02	1.78E-02	-2.52E-04	2.73E+00	1.85E-01	1.48E-17	9.25E-15	5.49E-06	1.85E-05	-1.80E-06
	Normalized peak height	3.57E-02	3.63E-02	8.61E-02	1.41E-01	1.09E-01	2.35E-02	5.76E-02	6.45E-02	1.86E-02	6.28E-01	1.11E-02	1.95E-02	1.56E-02	1.53E-02	2.10E-01	3.03E-02	4.02E-01	7.62E-01	4.67E-02	1.69E-02	1.17E-02
	Peak sensitivity value	1.01E-02	2.66E-03	1.27E-05	1.32E-04	-4.19E-04	2.08E-04	7.42E-04	4.54E-04	6.03E-03	-2.54E-18	3.21E-03	4.65E-04	9.47E-05	-5.00E-03	3.44E-03	3.66E-04	-6.72E-21	-3.87E-18	-4.55E-10	1.51E-07	-8.48E-05
	Shapiro-Wilk score	9.045E-01	8.591E-01	7.491E-01	7.090E-01	8.520E-01	8.806E-01	8.348E-01	8.204E-01	8.620E-01	3.246E-01	8.610E-01	8.279E-01	8.342E-01	8.697E-01	6.422E-01	7.947E-01	6.166E-01	2.650E-02	6.900E-01	8.685E-01	8.063E-01
(glyceraldehyde3phosphate dehydrogenase)	Local	7.06E-01	5.02E-03	1.49E-04	1.08E-03	1.51E-02	5.04E-03	8.95E-02	5.25E-02	4.62E-02	1.18E-16	7.20E-03	8.34E-03	1.71E-03	-2.12E-02	7.05E-02	1.33E-02	-1.31E-19	6.21E-20	2.07E-07	5.29E-07	-3.54E-04
	Min	-3.28E-01	-1.77E-01	-1.10E-06	-3.71E-05	-4.36E-03	3.11E-06	9.95E-06	8.01E-06	-2.49E-02	-7.60E-16	8.58E-05	3.02E-06	6.17E-07	-7.71E-02	5.54E-06	2.41E-06	-1.60E-18	-7.37E-17	2.18E-12	-5.18E-07	-3.29E-03
	Max	9.98E-01	4.08E-02	7.98E-03	1.37E-01	2.79E-01	4.84E-02	9.51E-01	5.87E-01	4.75E-01	6.89E-16	8.50E-02	5.39E-02	1.34E-02	6.00E-02	9.99E-01	1.09E-01	1.64E-18	1.72E-16	2.95E-06	4.71E-06	2.22E-04
	Normalized peak height	4.76E-02	3.64E-02	7.48E-02	1.43E-01	8.29E-02	3.23E-02	6.27E-02	6.54E-02	2.08E-02	3.25E-01	1.01E-02	1.48E-02	1.54E-02	1.46E-02	7.33E-02	2.39E-02	3.45E-01	5.48E-01	5.65E-02	1.18E-02	8.24E-02
	Peak sensitivity value	2.50E-04	3.05E-03	1.09E-05	1.69E-04	2.86E-05	1.24E-04	4.85E-04	3.01E-04	3.16E-04	1.07E-19	2.25E-03	1.38E-04	4.76E-05	-1.37E-04	5.05E-04	1.66E-04	1.33E-21	-2.42E-20	1.48E-09	6.00E-08	-1.30E-06
	Shapiro-Wilk score	8.282E-01	7.029E-01	6.518E-01	5.761E-01	7.767E-01	8.334E-01	7.962E-01	7.946E-01	6.422E-01	7.173E-01	7.619E-01	8.468E-01	8.532E-01	8.968E-01	5.974E-01	8.029E-01	6.949E-01	2.625E-01	7.550E-01	8.642E-01	7.545E-01
(glycerol3phosphate dehydrogenase)	Local	7.06E-01	5.02E-03	1.49E-04	1.08E-03	1.51E-02	5.04E-03	8.95E-02	5.25E-02	4.62E-02	1.18E-16	7.20E-03	8.34E-03	1.71E-03	-2.12E-02	7.05E-02	1.33E-02	-1.31E-19	6.21E-20	2.07E-07	5.29E-07	-3.54E-04
	Min	-3.54E-01	-2.20E-01	-3.01E-06	-4.69E-05	-3.51E-03	-4.16E-04	1.10E-05	7.09E-06	-2.79E-03	-7.81E-16	6.81E-05	2.22E-06	4.05E-07	-8.53E-02	6.62E-06	2.86E-06	-2.42E-18	-3.72E-17	2.28E-12	-6.04E-08	-2.46E-03
	Max	9.98E-01	4.42E-02	1.28E-02	1.82E-01	2.83E-01	4.42E-02	9.15E-01	6.14E-01	4.82E-01	7.62E-16	1.00E-01	6.12E-02	1.06E-02	7.89E-02	9.99E-01	1.09E-01	1.55E-17	5.62E-16	3.26E-06	4.36E-06	2.55E-04
	Normalized peak height	4.71E-02	4.36E-02	1.20E-01	1.61E-01	7.96E-02	3.02E-02	6.19E-02	7.02E-02	2.03E-02	3.31E-01	1.21E-02	1.70E-02	1.25E-02	1.43E-02	7.47E-02	2.48E-02	4.43E-01	6.43E-01	5.89E-02	1.03E-02	7.53E-02
	Peak sensitivity value	5.96E-04	3.11E-03	1.62E-05	2.26E-04	-2.07E-04	9.76E-05	4.69E-04	3.14E-04	1.33E-03	2.41E-19	2.02E-03	9.41E-05	2.68E-05	-1.96E-04	5.06E-04	1.66E-04	-3.79E-21	2.05E-19	1.63E-09	5.24E-08	-1.33E-06
	Shapiro-Wilk score	8.262E-01	6.769E-01	6.480E-01	5.610E-01	7.777E-01	8.316E-01	7.963E-01	7.927E-01	6.450E-01	7.154E-01	7.650E-01	8.437E-01	8.538E-01	8.945E-01	5.969E-01	8.063E-01	5.371E-01	9.869E-02	7.466E-01	8.618E-01	7.509E-01
(glycerol3phosphate oxidase)	Local	4.14E-01	2.95E-03	2.25E-04	1.71E-03	3.00E-02	1.02E-02	1.80E-01	1.06E-01	9.60E-02	8.65E-17	1.45E-02	1.68E-02	3.45E-03	-4.42E-02	1.42E-01	2.67E-02	-2.64E-19	1.27E-19	4.16E-07	1.10E-06	-7.39E-04
	Min	-1.64E+00	-1.38E-01	-3.33E-03	-2.45E-02	-2.81E-01	1.23E-05	-5.71E-01	-1.70E-01	4.30E-04	-9.44E-15	-5.19E-03	-3.81E-03	-8.47E-04	-6.04E-01	-1.09E+00	-2.87E-03	-3.93E-18	-6.34E-17	-6.76E-08	1.57E-08	-5.66E-03
	Max	2.04E+00	1.60E-01	1.38E-02	1.27E-01	4.87E-01	6.90E-02	1.57E+00	1.00E+00	9.81E-01	1.60E-14	1.03E-01	7.55E-02	2.05E-02	-3.01E-04	2.58E+00	1.48E-01	1.31E-17	1.59E-15	4.76E-06	1.45E-05	-9.32E-07
	Normalized peak height	2.30E-02	3.50E-02	1.25E-01	1.17E-01	1.18E-01	2.12E-02	7.33E-02	6.29E-02	1.45E-02	5.77E-01	8.68E-03	1.41E-02	1.64E-02	1.44E-02	1.14E-01	2.14E-02	3.95E-01	5.95E-01	6.49E-02	1.37E-02	1.13E-02
	Peak sensitivity value	7.63E-03	2.73E-03	1.91E-05	1.98E-04	-9.75E-06	1.85E-04	9.73E-04	1.17E-03	4.84E-03	9.77E-18	2.46E-03	3.50E-04	1.04E-04	-3.62E-03	8.60E-04	5.15E-04	-6.74E-21	3.16E-19	2.36E-09	1.24E-07	-7.73E-05
	Shapiro-Wilk score	9.043E-01	8.568E-01	7.498E-01	7.029E-01	8.484E-01	8.808E-01	8.324E-01	8.154E-01	8.640E-01	3.164E-01	8.587E-01	8.259E-01	8.294E-01	8.703E-01	6.431E-01	7.938E-01	6.194E-01	1.393E-01	6.879E-01	8.688E-01	8.056E-01
(pyruvate transport)	Local	7.06E-01	5.02E-03	1.49E-04	1.08E-03	1.51E-02	5.04E-03	8.95E-02	5.25E-02	4.62E-02	-2.22E-16	7.20E-03	8.34E-03	1.71E-03	-2.12E-02	7.05E-02	1.33E-02	-1.31E-19	6.21E-20	2.07E-07	5.29E-07	-3.54E-04
	Min	-4.18E-01	-2.57E-01	-6.28E-07	-2.45E-05	-3.96E-03	-3.92E-04	1.08E-05	7.00E-06	-4.44E-02	-8.88E-16	5.58E-05	3.48E-06	8.39E-07	-8.07E-02	5.43E-06	2.64E-06	-1.35E-18	-4.85E-17	2.87E-12	-7.31E-07	-2.41E-03
	Max	9.98E-01	4.18E-02	8.56E-03	2.03E-01	2.92E-01	4.66E-02	8.33E-01	5.89E-01	4.76E-01	8.88E-16	9.01E-02	5.40E-02	1.20E-02	1.16E-01	9.99E-01	9.14E-02	7.60E-17	9.12E-17	3.24E-06	4.54E-06	2.81E-04
	Normalized peak height	4.89E-02	4.89E-02	8.24E-02	1.76E-01	8.58E-02	3.24E-02	5.66E-02	6.79E-02	2.18E-02	4.15E-01	1.06E-02	1.47E-02	1.43E-02	2.10E-02	7.55E-02	2.05E-02	6.68E-01	3.75E-01	5.94E-02	1.24E-02	6.65E-02
	Peak sensitivity value	2.43E-04	3.09E-03	1.22E-05	7.71E-05	3.83E-05	1.01E-04	4.27E-04	3.01E-04	1.14E-03	8.88E-19	1.72E-03	1.38E-04	4.29E-05	-9.53E-05	5.05E-04	1.40E-04	5.71E-21	-6.76E-20	1.62E-09	6.80E-08	-6.13E-07
	Shapiro-Wilk score	8.265E-01	6.954E-01	6.514E-01	5.727E-01	7.729E-01	8.288E-01	7.929E-01	7.909E-01	6.372E-01	9.081E-01	7.646E-01	8.483E-01	8.550E-01	8.957E-01	5.974E-01	8.067E-01	9.236E-02	4.499E-01	7.527E-01	8.659E-01	7.557E-01

(phosphoglycerate kinase)																					
Local	7.06E-01	5.02E-03	1.49E-04	1.08E-03	1.51E-02	5.04E-03	8.95E-02	5.25E-02	4.62E-02	2.34E-17	7.20E-03	8.34E-03	1.71E-03	-2.12E-02	7.05E-02	1.33E-02	-1.31E-19	6.21E-20	2.07E-07	5.29E-07	-3.54E-04
Min	-3.92E-01	-2.90E-01	-2.71E-06	-3.92E-05	-3.25E-03	5.79E-06	1.39E-05	6.50E-06	-2.14E-02	-6.59E-16	1.15E-04	2.68E-06	5.44E-07	-7.42E-02	8.41E-06	2.46E-06	-1.84E-18	-2.35E-16	4.50E-12	-3.62E-07	-2.39E-03
Max	9.98E-01	4.18E-02	1.03E-02	2.19E-01	2.80E-01	4.61E-02	1.00E+00	6.12E-01	4.77E-01	7.70E-16	9.45E-02	4.76E-02	1.17E-02	4.87E-02	9.99E-01	9.48E-02	1.88E-18	1.85E-16	2.42E-06	3.96E-06	1.68E-04
Normalized peak height	4.85E-02	5.36E-02	9.77E-02	1.78E-01	8.75E-02	3.12E-02	6.68E-02	6.92E-02	2.10E-02	1.97E-01	1.16E-02	1.36E-02	1.35E-02	1.23E-02	7.41E-02	2.13E-02	3.47E-01	5.83E-01	5.15E-02	1.06E-02	4.71E-02
Peak sensitivity value	5.39E-04	3.16E-03	1.27E-05	7.04E-05	7.92E-06	7.50E-05	5.15E-04	3.12E-04	1.28E-03	6.07E-19	2.14E-03	7.41E-05	4.15E-05	-1.35E-04	5.08E-04	1.45E-04	-1.06E-21	-1.61E-19	1.21E-09	5.03E-08	3.34E-07
Shapiro-Wilk score	8.266E-01	6.699E-01	6.496E-01	5.591E-01	7.731E-01	8.284E-01	7.929E-01	7.910E-01	6.327E-01	8.103E-01	7.583E-01	8.462E-01	8.520E-01	8.941E-01	5.971E-01	8.043E-01	6.953E-01	1.784E-01	7.527E-01	8.637E-01	7.531E-01
(pyruvate kinase)																					
Local	7.06E-01	5.02E-03	1.49E-04	1.08E-03	1.51E-02	5.04E-03	8.95E-02	5.25E-02	4.62E-02	-2.12E-16	7.20E-03	8.34E-03	1.71E-03	-2.12E-02	7.05E-02	1.33E-02	-1.31E-19	6.21E-20	2.07E-07	5.29E-07	-3.54E-04
Min	-2.84E-01	-2.53E-01	1.38E-07	-3.22E-05	-3.27E-03	4.89E-06	1.18E-05	4.80E-06	-1.97E-02	-8.68E-16	1.06E-04	2.87E-06	6.31E-07	-8.24E-02	7.73E-06	2.91E-06	-1.55E-18	-8.36E-17	4.41E-12	-3.75E-07	-2.56E-03
Max	9.98E-01	4.43E-02	8.30E-03	2.05E-01	2.81E-01	5.26E-02	8.82E-01	5.83E-01	4.77E-01	8.54E-16	8.14E-02	5.15E-02	1.18E-02	1.00E-01	9.99E-01	1.11E-01	2.63E-18	1.44E-16	2.51E-06	4.13E-06	1.94E-04
Normalized peak height	4.44E-02	5.15E-02	7.88E-02	1.70E-01	8.93E-02	3.54E-02	5.84E-02	6.49E-02	2.10E-02	3.72E-01	1.00E-02	1.42E-02	1.39E-02	1.69E-02	7.42E-02	2.52E-02	3.47E-01	5.33E-01	4.88E-02	1.07E-02	6.44E-02
Peak sensitivity value	2.00E-04	3.09E-03	1.26E-05	7.04E-05	-4.16E-06	8.38E-05	4.53E-04	2.96E-04	1.42E-03	-7.43E-19	2.26E-03	1.83E-04	3.02E-05	-1.78E-04	5.07E-04	1.70E-04	-9.92E-22	-4.48E-20	1.26E-09	6.40E-08	-5.72E-07
Shapiro-Wilk score	8.279E-01	6.968E-01	6.502E-01	5.721E-01	7.751E-01	8.305E-01	7.956E-01	7.929E-01	6.376E-01	9.630E-01	7.627E-01	8.489E-01	8.562E-01	8.950E-01	5.963E-01	8.078E-01	6.994E-01	2.700E-01	7.615E-01	8.631E-01	7.531E-01
(atp utilisation)																					
Local	7.06E-01	5.02E-03	1.49E-04	1.08E-03	1.51E-02	5.04E-03	8.95E-02	5.25E-02	4.62E-02	2.20E-21	7.20E-03	8.34E-03	1.71E-03	-2.12E-02	7.05E-02	1.33E-02	-1.31E-19	6.21E-20	2.07E-07	5.29E-07	-3.54E-04
Min	-2.61E-01	-1.76E-01	-1.07E-06	-1.78E-05	-2.34E-03	-7.29E-04	1.21E-05	6.13E-06	-7.58E-03	-6.02E-12	4.11E-05	1.26E-06	4.28E-07	-7.57E-02	3.25E-06	1.13E-06	-1.97E-18	-3.73E-16	-7.59E-17	-1.58E-07	-2.42E-03
Max	9.98E-01	4.03E-02	6.83E-03	1.39E-01	2.99E-01	4.25E-02	8.25E-01	5.93E-01	4.71E-01	5.35E-12	9.70E-02	6.17E-02	1.12E-02	5.51E-02	9.99E-01	9.22E-02	5.92E-17	1.04E-16	2.74E-06	4.03E-06	1.69E-04
Normalized peak height	4.38E-02	3.49E-02	6.43E-02	1.39E-01	1.03E-01	3.02E-02	5.72E-02	6.81E-02	2.12E-02	9.19E-01	1.16E-02	1.77E-02	1.29E-02	1.24E-02	7.55E-02	2.16E-02	6.09E-01	6.03E-01	5.19E-02	1.00E-02	6.33E-02
Peak sensitivity value	2.45E-05	2.83E-03	1.60E-05	1.91E-04	-8.52E-05	7.13E-05	4.24E-04	3.03E-04	1.75E-03	1.28E-15	1.83E-03	9.38E-05	2.83E-05	-1.60E-04	5.03E-04	1.40E-04	1.62E-20	1.71E-19	1.37E-09	5.32E-08	-5.50E-07
Shapiro-Wilk score	8.275E-01	7.389E-01	6.564E-01	5.933E-01	7.749E-01	8.309E-01	7.952E-01	7.925E-01	6.421E-01	6.132E-02	7.638E-01	8.489E-01	8.543E-01	8.944E-01	5.955E-01	8.043E-01	1.355E-01	1.649E-01	7.664E-01	8.653E-01	7.522E-01
(glycerol kinase)																					
Local	9.22E+00	6.57E-02	-2.07E-03	-1.73E-02	-4.20E-01	-1.47E-01	-2.56E+00	-1.50E+00	-1.41E+00	1.05E-15	-2.06E-01	-2.39E-01	-4.91E-02	6.53E-01	-2.02E+00	-3.80E-01	3.75E-18	-1.82E-18	-5.91E-06	-1.61E-05	1.09E-02
Min	-7.74E+00	-1.07E+00	-4.95E-02	-3.80E-01	-6.16E+00	-1.27E+00	-2.96E+01	-1.60E+01	-2.83E+01	-2.36E-13	-1.05E+00	-6.69E-01	-1.77E-01	1.87E-02	-7.30E+01	-1.22E+00	-5.13E-16	-3.12E-14	-1.13E-04	-3.14E-04	3.12E-05
Max	7.50E+01	6.36E-01	3.17E-02	5.23E-01	1.51E+00	2.19E-01	5.47E+00	2.52E+00	-5.24E-02	2.41E-13	2.35E-01	1.17E-01	2.67E-02	1.43E+00	5.37E+00	2.35E-01	3.37E-17	3.67E-16	2.57E-06	-1.45E-07	1.02E-01
Normalized peak height	5.67E-02	2.24E-02	2.89E-02	3.26E-02	1.27E-02	1.45E-02	3.90E-02	3.26E-02	3.37E-02	7.17E-01	2.23E-02	3.04E-02	3.38E-02	7.22E-03	1.50E-01	3.98E-02	5.61E-01	7.17E-01	1.25E-01	1.06E-02	1.55E-02
Peak sensitivity value	-8.16E-02	1.11E-02	2.27E-04	-9.42E-04	-8.46E-02	-2.73E-02	2.45E-02	1.51E-02	-1.34E+00	7.85E-17	3.78E-03	9.92E-04	3.73E-04	9.68E-01	4.67E-03	1.78E-03	1.35E-19	3.16E-18	-2.76E-08	-7.21E-06	1.30E-03
Shapiro-Wilk score	9.140E-01	8.723E-01	8.538E-01	8.490E-01	8.966E-01	8.167E-01	8.316E-01	8.127E-01	6.831E-01	1.739E-01	8.428E-01	8.939E-01	8.909E-01	8.890E-01	6.646E-01	8.939E-01	4.420E-01	3.689E-02	7.236E-01	8.327E-01	8.145E-01
(phosphoglycerate mutase)																					
Local	7.06E-01	5.02E-03	1.49E-04	1.08E-03	1.51E-02	5.04E-03	8.95E-02	5.25E-02	4.62E-02	2.10E-17	7.20E-03	8.34E-03	1.71E-03	-2.12E-02	7.05E-02	1.33E-02	-1.31E-19	6.21E-20	2.07E-07	5.29E-07	-3.54E-04
Min	-2.65E-01	-2.59E-01	-6.00E-06	-9.18E-05	-2.32E-03	-1.35E-03	1.77E-05	6.87E-06	-1.04E-02	-6.20E-16	9.65E-05	3.47E-06	8.53E-07	-7.17E-02	8.64E-06	4.33E-06	-2.52E-18	-4.80E-17	3.06E-12	-1.00E-07	-2.60E-03
Max	9.98E-01	3.29E-02	1.14E-02	2.07E-01	2.84E-01	4.79E-02	8.61E-01	6.27E-01	4.73E-01	7.69E-16	8.75E-02	6.04E-02	1.13E-02	6.61E-02	9.99E-01	9.94E-02	3.07E-18	8.07E-17	3.12E-06	4.38E-06	1.61E-04
Normalized peak height	3.83E-02	4.79E-02	1.04E-01	1.92E-01	8.83E-02	3.33E-02	5.88E-02	6.94E-02	2.11E-02	2.39E-01	1.06E-02	1.68E-02	1.32E-02	1.47E-02	7.41E-02	2.23E-02	3.61E-01	3.85E-01	5.70E-02	1.06E-02	6.18E-02
Peak sensitivity value	7.60E-04	3.03E-03	1.11E-05	2.19E-04	-1.74E-04	1.08E-04	4.48E-04	3.20E-04	1.88E-03	-2.72E-20	1.98E-03	9.40E-05	2.92E-05	-1.17E-04	5.08E-04	1.53E-04	-5.45E-22	5.97E-20	1.56E-09	6.75E-08	-3.09E-07
Shapiro-Wilk score	8.269E-01	6.786E-01	6.500E-01	5.624E-01	7.742E-01	8.286E-01	7.938E-01	7.914E-01	6.413E-01	8.280E-01	7.614E-01	8.469E-01	8.549E-01	8.957E-01	5.977E-01	8.053E-01	6.799E-01	4.457E-01	7.474E-01	8.660E-01	7.518E-01
(enolase)																					
Local	7.06E-01	5.02E-03	1.49E-04	1.08E-03	1.51E-02	5.04E-03	8.95E-02	5.25E-02	4.62E-02	2.10E-17	7.20E-03	8.34E-03	1.71E-03	-2.12E-02	7.05E-02	1.33E-02	-1.31E-19	6.21E-20	2.07E-07	5.29E-07	-3.54E-04
Min	-3.49E-01	-2.36E-01	-3.42E-07	-1.49E-05	-3.27E-03	4.73E-06	7.98E-06	5.21E-06	-1.90E-02	-7.60E-16	1.10E-04	2.37E-06	7.21E-07	-7.88E-02	6.99E-06	2.35E-06	-1.97E-18	-7.29E-17	3.58E-12	-2.96E-07	-2.62E-03
Max	9.98E-01	4.16E-02	8.87E-03	1.88E-01	2.86E-01	4.51E-02	9.68E-01	6.07E-01	4.64E-01	6.42E-16	8.32E-02	5.21E-02	1.55E-02	5.15E-02	9.99E-01	1.08E-01	7.99E-18	1.48E-16	2.51E-06	4.71E-06	1.98E-04
Normalized peak height	4.49E-02	4.61E-02	8.58E-02	1.74E-01	7.57E-02	3.05E-02	6.37E-02	6.82E-02	2.01E-02	2.16E-01	9.91E-03	1.46E-02	1.79E-02	1.40E-02	7.50E-02	2.43E-02	3.89E-01	5.26E-01	4.96E-02	1.17E-02	7.48E-02
Peak sensitivity value	6.50E-04	3.17E-03	1.30E-05	7.93E-05	6.47E-05	7.24E-05	4.92E-04	3.09E-04	1.56E-03	6.11E-19	2.64E-03	1.33E-04	2.39E-05	-6.12E-05	5.07E-04	1.65E-04	-3.06E-21	4.93E-20	1.26E-09	5.71E-08	-1.13E-06
Shapiro-Wilk score	8.273E-01	7.265E-01	6.526E-01	5.871E-01	7.743E-01	8.311E-01	7.940E-01	7.914E-01	6.419E-01	8.313E-01	7.659E-01	8.469E-01	8.548E-01	8.951E-01	5.970E-01	8.063E-01	6.533E-01	3.107E-01	7.500E-01	8.651E-01	7.527E-01
(adenylate kinase cytosol)																					
Local	INF	INF	INF	INF	INF	INF	INF	INF	INF	INF	INF	INF	INF	INF	INF	INF	INF	INF	INF	INF	INF
Min	-INF	-INF	-INF	-INF	-INF	-INF	-INF	-INF	-INF	-INF	-INF	-INF	-INF	-INF	-INF	-INF	-INF	-INF	-INF	-INF	-INF
Max	INF	INF	INF	INF	INF	INF	INF	INF	INF	INF	INF	INF	INF	INF	INF	INF	INF	INF	INF	INF	INF
Normalized peak height	1.11E-01	1.11E-01	1.11E-01	1.11E-01	3.33E-01	2.22E-01	2.22E-01	1.11E-01	2.22E-01	2.22E-01	1.11E-01	1.11E-01	1.11E-01	1.11E-01	2.22E-01	1.11E-01	1.11E-01	2.22E-01	2.22E-01	1.11E-01	3.33E-01
Peak sensitivity value	-2.87E-02	-3.51E-01	-2.52E-02	-1.40E-01	-1.86E-07	4.86E-05	2.95E-06	-9.04E-04	4.01E-04	1.41E-08	-7.03E-02	-2.63E-01	-1.43E-01	-1.01E-03	-1.53E-01	-5.46E-02	-8.02E-13	1.16E-14	-1.50E-02	-7.35E-03	2.52E-08
Shapiro-Wilk score	7.164E-01	8.022E-01	9.277E-01	9.165E-01	8.542E-01	5.211E-01	6.249E-01	8.847E-01	8.824E-01	7.446E-01	8.137E-01	7.776E-01	8.707E-01	9.669E-01	8.746E-01	8.819E-01	9.267E-01	4.864E-01	8.538E-01	6.039E-01	7.511E-01
(adenylate kinase glycosome)																					
Local	-INF	-INF	-INF	-INF	-INF	-INF	-INF	-INF	-INF	-INF	-INF	-INF	-INF	-INF	-INF	-INF	-INF	-INF	-INF	-INF	-INF
Min	-INF	-INF	-INF	-INF	-INF	-INF	-INF	-INF	-INF	-INF	-INF	-INF	-INF	-INF	-INF	-INF	-INF	-INF	-INF	-INF	-INF
Max	INF	INF	INF	INF	INF	INF	INF	INF	INF	INF	INF	INF	INF	INF	INF	INF	INF	INF	INF	INF	INF
Normalized peak height	2.32E-01	1.21E-01	9.78E-02	9.44E-02	2.38E-01	2.33E-01	2.58E-01	2.19E-01	6.18E-01	3.42E-01	9.27E-02	1.09E-01	8.61E-02	4.48E-01	4.12E-01	1.09E-01	5.52E-01	5.76E-02	2.50E-01	3.67E-01	4.47E-01
Peak sensitivity value	5.01E-02	2.80E-02	1.24E-02	9.70E-03	-6.42E-03	6.94E-04	-2.82E-02	-1.65E-02	-3.40E-03	1.21E-07	3.50E-02	-1.65E-03	8.01E-05	1.45E-03	5.42E-02	-1.90E-04	8.94E-16	-1.65E-13	2.06E-02	1.22E-03	2.31E-05
Shapiro-Wilk score	6.806E-01	7.195E-01	7.512E-01	7.365E-01	5.872E-01	6.148E-01	5.790E-01	5.860E-01	3.706E-01	5.418E-01	7.346E-01	7.701E-01	7.963E-01	5.629E-01	3.891E-01	7.602E-01	3.076E-01	8.127E-01	5.034E-01	5.550E-01	5.305E-01
(3phosphoglycerate transport)																					
Local	7.06E-01	5.02E-03	1.49E-04	1.08E-03	1.51E-02	5.04E-03	8.95E-02	5.25E-02	4.62E-02	2.34E-17	7.20E-03	8.34E-03	1.71E-03	-2.12E-02	7.05E-02	1.33E-02	-1.31E-19	6.20E-20	2.07E-07	5.29E-07	-3.54E-04
Min	-4.26E-01	-2.09E-01	3.95E-07	-1.50E-05	-1.53E-02	4.01E-06	1.36E-05	4.28E-06	-8.92E-03	-6.23E-16	5.47E-05	1.39E-06	6.96E-07	-9.17E-02	6.69E-06	2.12E-06	-1.91E-18	-9.25E-17	-4.62E-08	-3.51E-07	-2.82E-03
Max	9.99E-01	4.44E-02	9.28E-03	1.72E-01	2.72E-01	4.13E-02	8.61E-01	5.97E-01	5.11E-01	7.32E-16	9.73E-02	6.14E-02	1.10E-02	6.42E-02	9.99E-01	9.56E-02	1.21E-17	2.42E-16	3.76E-06	4.15E-06	1.73E-04
Normalized peak height	5.10E-02	4.23E-02	8.97E-02	1.56E-01	9.07E-02	2.82E-02	5.86E-02	6.71E-02	2.18E-02	2.11E-01	1.19E-02	1.73E-02	1.29E-02	1.74E-02	7.50E-02	2.18E-02	4.18E-01	5.75E-01	4.03E-02	1.05E-02	6.47E-02
Peak sensitivity value	4.61E-04	3.03E-03	1.43E-05	7.10E-05	-1.75E-04	1.07E-04	4.44E-04	3.03E-04	1.22E-03	-3.03E-19	2.24E-03	9.34E-05	2.82E-05	-8.05E-05	5.06E-04	1.45E-04	5.12E-22	-8.88E-20	5.17E-09	5.15E-08	-1.74E-06
Shapiro-Wilk score	8.266E-01	7.059E-01	6.514E-01	5.751E-01	7.734E-01	8.304E-01	7.938E-01	7.916E-01	6.377E-01	8.139E-01	7.642E-01	8.490E-01	8.525E-01	8.934E-01	5.981E-01	8.075E-01	5.471E-01	2.476E-01	7.525E-01	8.638E-01	7.490E-01
(gly3p dhap antiporter)																					
Local	4.14E-01	2.95E-03	2.25E-04	1.71E-03	3.00E-02	1.02E-02	1.80E-01	1.06E-01	9.60E-02	8.65E-17	1.45E-02	1.68E-02	3.45E-03	-4.42E-02	1.42E-01	2.67E-02	-2.64E-19	1.27E-19	4.16E-07	1.10E-06	-7.39E-04
Min	-1.67E+00	-2.41E-01	-3.41E-03	-2.78E-02	-1.94E-01	8.28E-06	-3.81E-01	-3.39E-01	2.65E-04	-2.09E-14	-2.75E-02	-1.05E-02	-1.91E-03	-5.55E-01	-1.10E+00	-3.65E-02	-3.61E-18	-7.53E-17	-1.28E-07	1.67E-08	-5.84E-03
Max	1.90E+00	2.15E-01	8.28E-03	2.31E-01	5.36E-01	6.95E-02	1.60E+00	1.02E+00	9.84E-01	2.20E-14	1.24E-01	8.71E-02	2.38E-02	-1.79E-04	2.60E+00	1.49E-01	5.29E-18	3.47E-15	4.49E-06	1.49E-05	-2.26E-06
Normalized peak height	2.15E-02	5.46E-02	8.43E-02	1.53E-01	9.92E-02	2.08E-02	6.43E-02	7.96E-02	1.45E-02	6.76E-01	1.16E-02	1.67E-02	1.85E-02	1.31E-02	1.14E-01	2.52E-02	3.59E-01	6.74E-01	4.24E-02	1.31E-02	1.13E-02
Peak sensitivity value	7.93E-03	2.73E-03	1.96E-05	2.90E-04	1.23E-04	1.82E-04	8.53E-04	7.95E-04	3.71E-03	-1.22E-17	3.18E-03	2.87E-04	1.10E-04	-2.68E-03	3.66E-03	2.42E-04	-1.63E-22	1.01E-18	3.21E-09	1.73E-07	-6.94E-05
Shapiro-Wilk score	9.052E-01	8.567E-01	7.506E-01	6.673E-01	8.497E-01	8.825E-01	8.345E-01	8.183E-01	8.632E-01	2.324E-01	8.630E-01	8.285E-01	8.329E-01	8.705E-01	6.438E-01	7.950E-01	6.508E-01	6.020E-02	6.822E-01	8.699E-01	8.092E-01
(glycerol transport)																					
Local	9.22E+00	6.57E-02	-2.07E-03	-1.73E-02	-4.20E-01	-1.47E-01	-2.56E+00	-1.50E+00	-1.41E+00	1.05E-15	-2.06E-01	-2.39E-01	-4.91E-02	6.53E-01	-2.02E+00	-3.80E-01	3.75E-18	-1.82E-18	-5.91E-06	-1.61E-05	1.09E-02
Min	-7.81E+00	-9.13E-01	-4.64E-02	-5.31E-01	-5.14E+00	-9.57E-01	-2.82E+01	-1.49E+01	-2.25E+01	-3.26E-13	-1.05E+00	-6.99E-01	-1.56E-01	1.25E-02	-4.66E+01	-1.17E+00	-3.56E-17	-2.27E-14	-1.01E-04	-2.36E-04	1.87E-05
Max	4.88E+01	6.28E-01	3.81E-02	4.43E-01	1.72E+00	7.93E-02	4.82E+00	2.54E+00	-5.93E-02	1.40E-13	2.27E-01	1.18E-01	2.51E-02	1.58E+00	8.74E+00	2.22E-01	3.36E-17	4.90E-16	2.03E-06	-2.96E-07	9.07E-02
Normalized peak height	4.02E-02	1.96E-02	3.07E-02	3.52E-02	1.16E-02	1.03E-02	5.01E-02	4.63E-02	2.63E-02	7.11E-01	2.22E-02	3.20E-02	3.20E-02	7.94E-03	1.37E-01	4.02E-02	4.00E-01	5.50E-01	1.49E-01	8.10E-03	1.42E-02
Peak sensitivity value	-8.34E-02	1.09E-02	1.80E-04	2.82E-04	-6.07E-02	-2.48E-02	1.03E-02	5.61E-03	-1.33E+00	-9.22E-17	3.93E-03	8.58E-04	2.63E-04	9.62E-01	2.07E-02	1.75E-03	-2.41E-20	-9.39E-18	2.75E-08	-7.01E-06	1.15E-03
Shapiro-Wilk score	9.175E-01	8.690E-01	8.532E-01	8.578E-01	8.994E-01	8.156E-01	8.323E-01	8.154E-01	7.025E-01	1.677E-01	8.406E-01	8.918E-01	8.889E-01	8.876E-01	6.805E-01	8.924E-01	6.915E-01	5.181E-02	7.252E-01	8.348E-01	8.128E-01

Table S16: Flux control coefficients for *Trypanosoma brucei* model with parameter variation of ±50%

		(glucosetransport)	(hexokinase)	(phosphoglycerateisomerase)	(phophofructokinase)	(aldolase)	(triosephosphateisomerase)	(glyceraldehyde3phosphatedehydrogenase)	(glycerol3phosphatedehydrogenase)	(glycerol3phosphateoxidase)	(pyruvatetransport)	(phosphoglyceratekinase)	(pyruvatekinase)	(atputilisation)	(glycerolkinase)	(phosphoglyceratemutase)	(enolase)	(adenylatekinasecytosol)	(adenylatekinaseglycosome)	(3phosphoglyceratetransport)	(gly3pdhapantiporter)	(glyceroltransport)
(glucose transport)	Local	9.79E-01	6.97E-03	7.82E-05	4.95E-04	1.13E-03	1.79E-04	4.63E-03	2.72E-03	-3.97E-04	-1.47E-18	3.73E-04	4.31E-04	8.87E-05	3.95E-04	3.65E-03	6.87E-04	-6.78E-21	1.71E-21	1.07E-08	-4.55E-09	6.60E-06
	Min	-4.25E+00	-1.46E+00	-8.74E-04	-5.04E-03	1.50E-07	-1.21E-02	-1.11E-03	-9.93E-04	-6.51E-01	-8.75E-13	6.78E-07	9.76E-09	1.78E-09	-5.43E-04	1.42E-08	9.31E-09	-2.68E-18	-5.25E-15	1.20E-13	-2.02E-05	-5.73E-06
	Max	9.99E-01	1.09E-01	2.46E-02	1.14E+00	6.04E-01	1.01E-01	3.60E+00	1.56E+00	7.69E-01	1.81E-13	1.62E-01	9.79E-02	2.07E-02	9.50E-01	2.03E+00	1.78E-01	2.61E-18	1.02E-16	1.78E-06	5.19E-07	4.68E-03
	Normalized peak height	2.20E-01	1.33E-01	1.73E-01	4.32E-01	3.77E-01	3.75E-01	4.15E-01	3.47E-01	3.83E-01	9.75E-01	3.21E-01	3.15E-01	3.02E-01	3.86E-01	4.54E-01	3.48E-01	5.82E-01	7.65E-01	3.18E-01	4.26E-01	3.69E-01
	Peak sensitivity value	9.97E-01	3.08E-03	5.10E-06	9.37E-05	3.02E-04	5.40E-05	6.90E-04	-2.13E-04	3.05E-04	2.22E-16	8.18E-05	4.90E-05	1.04E-05	-6.72E-05	1.01E-03	8.91E-05	-1.48E-21	-2.60E-18	8.89E-10	-8.45E-09	1.30E-06
	Shapiro-Wilk score	7.328E-01	7.222E-01	5.479E-01	3.499E-01	7.206E-01	7.081E-01	6.858E-01	6.703E-01	8.090E-01	3.246E-02	5.688E-01	5.522E-01	5.519E-01	7.892E-01	5.290E-01	4.894E-01	3.845E-01	4.126E-02	5.877E-01	7.405E-01	6.013E-01
(hexokinase)	Local	9.79E-01	6.97E-03	7.82E-05	4.95E-04	1.13E-03	1.79E-04	4.63E-03	2.72E-03	-3.97E-04	-1.47E-18	3.73E-04	4.31E-04	8.87E-05	3.95E-04	3.65E-03	6.87E-04	-6.78E-21	1.71E-21	1.07E-08	-4.55E-09	6.60E-06
	Min	-2.21E+00	-2.93E+00	-1.11E-03	-5.91E-03	2.26E-07	-1.56E-02	-9.92E-04	-4.80E-04	-4.92E-01	-1.28E-13	3.27E-07	1.48E-08	5.37E-09	-6.50E-04	4.30E-08	9.19E-09	-5.43E-18	-6.01E-15	1.71E-13	-1.36E-05	-8.52E-06
	Max	1.01E+00	1.21E-01	1.08E-01	2.21E+00	4.65E-01	1.43E-01	2.29E+00	1.01E+00	7.50E-01	1.51E-13	1.69E-01	9.58E-02	2.23E-02	7.11E-01	3.41E+00	1.79E-01	5.68E-18	1.79E-16	2.22E-06	4.74E-07	3.67E-03
	Normalized peak height	1.29E-01	2.41E-01	4.36E-01	4.44E-01	3.62E-01	3.74E-01	3.65E-01	3.39E-01	3.48E-01	9.24E-01	3.25E-01	3.13E-01	3.08E-01	3.23E-01	5.06E-01	3.48E-01	6.55E-01	8.01E-01	3.46E-01	3.96E-01	3.55E-01
	Peak sensitivity value	9.95E-01	3.26E-03	3.10E-05	-3.79E-04	2.33E-04	-1.59E-05	1.52E-04	2.47E-05	4.88E-04	-9.86E-17	8.50E-05	4.79E-05	1.11E-05	-2.94E-04	1.70E-03	8.96E-05	-8.37E-22	2.71E-18	1.11E-09	2.76E-09	6.74E-07
	Shapiro-Wilk score	7.335E-01	5.914E-01	4.735E-01	2.315E-01	7.206E-01	7.125E-01	6.875E-01	6.707E-01	8.093E-01	6.775E-02	5.676E-01	5.465E-01	5.503E-01	7.921E-01	5.304E-01	4.877E-01	3.523E-01	3.088E-02	5.857E-01	7.426E-01	6.051E-01
(phosphoglycerate isomerase)	Local	9.79E-01	6.97E-03	7.82E-05	4.95E-04	1.13E-03	1.79E-04	4.63E-03	2.72E-03	-3.97E-04	-1.47E-18	3.73E-04	4.31E-04	8.87E-05	3.95E-04	3.65E-03	6.87E-04	-6.78E-21	1.71E-21	1.07E-08	-4.55E-09	6.60E-06
	Min	-2.60E+00	-2.39E+00	-1.03E-03	-7.68E-03	1.82E-07	-1.18E-02	-1.31E-03	-5.63E-04	-4.77E-01	-2.59E-13	3.95E-07	2.30E-08	6.00E-09	-3.32E-04	3.55E-08	2.22E-08	-2.74E-18	-2.73E-15	1.67E-13	-1.46E-05	-6.44E-06
	Max	1.00E+00	1.15E-01	5.00E-02	1.79E+00	4.90E-01	8.69E-02	2.31E+00	1.47E+00	9.49E-01	2.98E-13	1.43E-01	8.21E-02	2.37E-02	5.11E-01	3.50E+00	2.25E-01	5.92E-18	2.53E-16	2.02E-06	4.41E-07	4.19E-03
	Normalized peak height	1.45E-01	2.09E-01	3.05E-01	5.30E-01	3.64E-01	3.41E-01	3.46E-01	3.78E-01	4.01E-01	9.26E-01	3.00E-01	2.96E-01	3.14E-01	3.57E-01	5.12E-01	3.71E-01	6.17E-01	7.51E-01	3.30E-01	4.16E-01	3.45E-01
	Peak sensitivity value	9.98E-01	3.10E-03	1.40E-05	3.91E-04	2.45E-04	-1.25E-05	-1.54E-04	1.69E-04	-1.10E-04	-2.46E-16	7.21E-05	4.11E-05	1.19E-05	-7.63E-05	1.75E-03	1.13E-04	2.75E-21	9.45E-19	1.01E-09	-2.46E-09	-1.51E-07
	Shapiro-Wilk score	7.336E-01	5.646E-01	5.187E-01	2.229E-01	7.226E-01	7.091E-01	6.897E-01	6.715E-01	8.094E-01	5.104E-02	5.706E-01	5.503E-01	5.500E-01	7.949E-01	5.314E-01	4.901E-01	3.735E-01	6.259E-02	5.854E-01	7.430E-01	6.035E-01
(phophofructokinase)	Local	9.79E-01	6.97E-03	7.82E-05	4.95E-04	1.13E-03	1.79E-04	4.63E-03	2.72E-03	-3.97E-04	-1.47E-18	3.73E-04	4.31E-04	8.87E-05	3.95E-04	3.65E-03	6.87E-04	-6.78E-21	1.71E-21	1.07E-08	-4.55E-09	6.60E-06
	Min	-3.74E+00	-2.92E+00	-2.07E-03	-9.72E-03	1.71E-07	-9.71E-03	-1.26E-03	-8.07E-04	-5.31E-01	-2.39E-13	2.81E-07	1.02E-08	2.46E-09	-6.67E-04	2.42E-08	1.48E-08	-3.40E-18	-2.13E-14	6.01E-14	-1.71E-05	-5.33E-06
	Max	1.00E+00	1.14E-01	3.69E-02	2.34E+00	7.37E-01	9.03E-02	2.34E+00	1.19E+00	9.80E-01	1.94E-13	1.60E-01	1.04E-01	1.86E-02	8.50E-01	1.73E+00	1.94E-01	3.23E-18	1.35E-16	1.93E-06	4.79E-07	3.78E-03
	Normalized peak height	1.87E-01	2.41E-01	2.82E-01	5.97E-01	3.84E-01	3.80E-01	3.50E-01	3.17E-01	3.97E-01	9.57E-01	3.16E-01	3.20E-01	2.91E-01	3.56E-01	4.38E-01	3.55E-01	6.07E-01	9.46E-01	3.25E-01	4.21E-01	3.48E-01
	Peak sensitivity value	9.97E-01	3.47E-03	1.59E-05	8.72E-04	3.68E-04	3.89E-05	-8.60E-05	-2.09E-04	5.31E-05	-5.22E-17	8.02E-05	5.21E-05	9.31E-06	-2.42E-04	8.66E-04	9.72E-05	9.95E-22	-4.39E-18	9.64E-10	-3.56E-09	3.46E-07
	Shapiro-Wilk score	7.331E-01	5.908E-01	5.200E-01	2.333E-01	7.215E-01	7.112E-01	6.889E-01	6.722E-01	8.082E-01	5.858E-02	5.694E-01	5.481E-01	5.475E-01	7.918E-01	5.276E-01	4.920E-01	3.729E-01	5.252E-03	5.860E-01	7.405E-01	6.004E-01
(aldolase)	Local	9.79E-01	6.97E-03	7.82E-05	4.95E-04	1.13E-03	1.79E-04	4.63E-03	2.72E-03	-3.97E-04	1.48E-16	3.73E-04	4.31E-04	8.87E-05	3.95E-04	3.65E-03	6.87E-04	-6.78E-21	1.71E-21	1.07E-08	-4.55E-09	6.60E-06
	Min	-2.02E+00	-2.81E+00	-5.80E-04	-3.67E-03	1.98E-07	-1.37E-02	-1.22E-03	-6.08E-04	-4.96E-01	-1.50E-14	6.48E-07	1.78E-08	3.66E-09	-3.08E-04	2.66E-08	1.46E-08	-2.84E-18	-4.79E-15	1.36E-13	-1.86E-05	-2.17E-06
	Max	9.99E-01	1.04E-01	2.68E-02	2.19E+00	5.53E-01	7.84E-02	1.76E+00	9.30E-01	6.63E-01	2.36E-14	1.29E-01	9.32E-02	1.74E-02	4.88E-01	2.97E+00	1.83E-01	4.89E-18	7.12E-17	1.59E-06	1.07E-06	4.46E-03
	Normalized peak height	1.22E-01	2.20E-01	2.11E-01	4.44E-01	3.70E-01	3.28E-01	3.04E-01	3.04E-01	3.13E-01	7.01E-01	2.86E-01	3.09E-01	2.85E-01	3.57E-01	4.92E-01	3.49E-01	5.68E-01	7.89E-01	3.03E-01	4.12E-01	3.50E-01
	Peak sensitivity value	9.98E-01	3.90E-03	9.24E-06	-3.72E-04	2.77E-04	-1.65E-05	-3.34E-04	-1.43E-04	5.22E-04	-8.96E-18	6.49E-05	4.66E-05	8.69E-06	-6.37E-05	1.48E-03	9.13E-05	3.49E-21	6.48E-19	7.97E-10	2.90E-09	6.37E-08
	Shapiro-Wilk score	7.335E-01	6.079E-01	5.402E-01	2.455E-01	7.230E-01	7.133E-01	6.903E-01	6.722E-01	8.111E-01	3.176E-01	5.704E-01	5.532E-01	5.591E-01	7.951E-01	5.294E-01	4.936E-01	3.740E-01	3.896E-02	5.850E-01	7.428E-01	5.988E-01
(triosephosphate isomerase)	Local	4.14E-01	2.95E-03	2.25E-04	1.71E-03	3.00E-02	1.02E-02	1.80E-01	1.06E-01	9.60E-02	8.65E-17	1.45E-02	1.68E-02	3.45E-03	-4.42E-02	1.42E-01	2.67E-02	-2.64E-19	1.27E-19	4.16E-07	1.10E-06	-7.39E-04
	Min	-2.11E+00	-1.80E+00	-7.04E-03	-1.16E-01	-1.19E+00	-1.74E-01	-4.31E+00	-1.61E+00	5.02E-05	-4.28E-14	-2.03E+00	-6.18E-01	-1.66E-01	-2.57E+00	-4.71E+00	-1.06E+00	-9.14E-18	-1.06E-16	-3.66E-06	5.22E-09	-9.33E-03
	Max	1.62E+01	9.22E-01	5.82E-02	1.42E+00	7.47E-01	9.29E-02	2.27E+00	1.33E+00	2.12E+00	1.96E-14	1.31E-01	1.43E-01	3.93E-02	-4.08E-05	3.07E+00	2.38E-01	9.31E-18	3.20E-15	7.45E-06	3.05E-05	-4.07E-07
	Normalized peak height	1.70E-01	2.26E-01	2.66E-01	4.50E-01	2.15E-01	8.41E-02	2.29E-01	1.90E-01	4.25E-02	7.75E-01	2.04E-01	1.59E-01	2.22E-01	6.74E-02	2.52E-01	2.55E-01	4.55E-01	6.37E-01	1.43E-01	3.07E-02	2.71E-02
	Peak sensitivity value	8.53E-03	1.91E-03	4.17E-05	2.77E-04	2.95E-04	2.79E-04	2.44E-03	1.64E-03	3.22E-03	-3.45E-18	2.23E-03	7.41E-04	1.21E-04	-1.32E-03	1.15E-04	4.78E-04	5.40E-21	1.19E-18	2.46E-09	8.16E-08	-3.31E-05
	Shapiro-Wilk score	8.735E-01	7.379E-01	6.021E-01	3.311E-01	8.214E-01	8.438E-01	8.153E-01	7.846E-01	8.102E-01	1.429E-01	5.683E-01	6.945E-01	6.801E-01	8.303E-01	6.324E-01	6.392E-01	5.100E-01	1.179E-01	5.606E-01	8.443E-01	6.845E-01
(glyceraldehyde3phosphate dehydrogenase)	Local	7.06E-01	5.02E-03	1.49E-04	1.08E-03	1.51E-02	5.04E-03	8.95E-02	5.25E-02	4.62E-02	1.18E-16	7.20E-03	8.34E-03	1.71E-03	-2.12E-02	7.05E-02	1.33E-02	-1.31E-19	6.21E-20	2.07E-07	5.29E-07	-3.54E-04
	Min	-6.47E-01	-1.12E+00	-6.85E-04	-4.12E-03	-6.73E-03	-3.95E-03	3.06E-06	8.90E-07	-7.98E-02	-7.74E-16	5.75E-06	2.58E-07	5.12E-08	-9.09E-02	3.87E-07	2.44E-07	-3.98E-18	-1.06E-15	2.63E-13	-1.38E-06	-3.67E-03
	Max	9.99E-01	6.83E-02	1.88E-02	9.08E-01	3.63E-01	6.49E-02	1.15E+00	7.28E-01	6.56E-01	8.02E-16	1.31E-01	9.87E-02	1.67E-02	2.20E-01	1.00E+00	2.09E-01	5.43E-18	2.31E-16	4.20E-06	7.21E-06	5.75E-04
	Normalized peak height	7.46E-02	1.61E-01	1.78E-01	3.87E-01	1.11E-01	7.38E-02	1.31E-01	1.38E-01	5.55E-02	3.40E-01	1.95E-02	6.06E-02	4.15E-02	6.31E-02	1.43E-01	1.02E-01	4.18E-01	7.99E-01	1.27E-01	3.67E-02	1.48E-01
	Peak sensitivity value	8.83E-04	2.14E-03	5.12E-06	-1.95E-05	1.06E-04	7.96E-05	5.80E-04	3.65E-04	8.20E-05	-6.31E-19	8.56E-04	4.96E-05	8.38E-06	-6.07E-05	5.00E-04	1.05E-04	3.37E-21	-2.65E-19	2.10E-09	2.18E-09	-3.36E-07
	Shapiro-Wilk score	7.897E-01	3.751E-01	5.481E-01	3.350E-01	7.582E-01	7.865E-01	7.883E-01	7.703E-01	5.388E-01	6.614E-01	6.904E-01	7.375E-01	7.437E-01	8.127E-01	5.967E-01	6.586E-01	5.520E-01	4.630E-02	6.417E-01	7.807E-01	6.174E-01
(glycerol3phosphate dehydrogenase)	Local	7.06E-01	5.02E-03	1.49E-04	1.08E-03	1.51E-02	5.04E-03	8.95E-02	5.25E-02	4.62E-02	1.18E-16	7.20E-03	8.34E-03	1.71E-03	-2.12E-02	7.05E-02	1.33E-02	-1.31E-19	6.21E-20	2.07E-07	5.29E-07	-3.54E-04
	Min	-1.47E+00	-1.13E+00	-7.87E-04	-5.08E-03	-5.92E-03	-4.68E-03	4.23E-06	1.68E-06	-2.25E-01	-8.16E-16	1.31E-05	4.36E-07	1.26E-07	-9.91E-02	7.12E-07	3.12E-07	-2.82E-18	-9.99E-17	1.25E-13	-5.32E-06	-6.23E-03
	Max	9.99E-01	6.24E-02	2.12E-02	9.10E-01	3.77E-01	9.47E-02	2.02E+00	7.19E-01	7.13E-01	8.36E-16	1.29E-01	7.14E-02	1.60E-02	2.75E-01	1.00E+00	2.22E-01	3.44E-18	9.74E-17	6.53E-06	8.84E-06	5.57E-04
	Normalized peak height	1.32E-01	1.59E-01	2.30E-01	3.77E-01	1.41E-01	1.07E-01	1.77E-01	1.39E-01	6.97E-02	3.10E-01	1.90E-02	4.66E-02	4.07E-02	7.61E-02	1.45E-01	1.07E-01	3.96E-01	5.61E-01	1.58E-01	4.47E-02	2.06E-01
	Peak sensitivity value	3.08E-04	1.95E-03	1.42E-05	-4.65E-05	1.24E-05	4.41E-05	1.02E-03	3.61E-04	8.66E-04	7.94E-19	1.24E-03	3.61E-05	8.12E-06	-1.38E-04	5.01E-04	1.11E-04	1.53E-21	2.49E-21	3.26E-09	1.39E-08	-2.40E-06
	Shapiro-Wilk score	7.910E-01	3.300E-01	5.554E-01	2.997E-01	7.570E-01	7.876E-01	7.894E-01	7.721E-01	5.413E-01	6.584E-01	6.939E-01	7.395E-01	7.449E-01	8.054E-01	5.991E-01	6.615E-01	5.658E-01	3.305E-01	6.314E-01	7.793E-01	6.093E-01
(glycerol3phosphate oxidase)	Local	4.14E-01	2.95E-03	2.25E-04	1.71E-03	3.00E-02	1.02E-02	1.80E-01	1.06E-01	9.60E-02	8.65E-17	1.45E-02	1.68E-02	3.45E-03	-4.42E-02	1.42E-01	2.67E-02	-2.64E-19	1.27E-19	4.16E-07	1.10E-06	-7.39E-04
	Min	-2.06E+00	-2.14E+00	-5.44E-03	-5.46E-02	-9.03E-01	-7.90E-02	-1.96E+00	-1.07E+00	1.13E-04	-3.44E-14	-8.29E-02	-6.38E-02	-1.57E-02	-9.89E-01	-1.03E+00	-5.58E-02	-5.11E-18	-9.47E-17	-5.27E-07	7.44E-09	-1.28E-02
	Max	4.82E+00	2.55E-01	1.82E-02	1.67E+00	5.95E-01	1.17E-01	2.01E+00	1.32E+00	1.06E+00	1.99E-14	1.22E-01	1.12E-01	3.18E-02	-8.25E-05	2.97E+00	2.53E-01	2.81E-17	1.05E-14	9.51E-06	3.20E-05	-2.68E-07
	Normalized peak height	7.97E-02	2.20E-01	1.63E-01	4.19E-01	1.34E-01	8.81E-02	1.78E-01	1.23E-01	2.17E-02	7.10E-01	2.18E-02	6.21E-02	7.28E-02	3.06E-02	1.70E-01	8.86E-02	5.21E-01	7.64E-01	1.16E-01	3.24E-02	3.68E-02
	Peak sensitivity value	5.61E-03	2.19E-03	1.89E-05	-2.07E-04	-8.65E-04	1.34E-04	1.43E-03	-1.77E-04	2.76E-03	-1.98E-17	1.35E-03	7.36E-05	2.87E-05	-1.57E-03	-8.33E-05	2.63E-04	-8.10E-21	-4.28E-18	-1.21E-10	8.73E-08	-4.49E-05
	Shapiro-Wilk score	8.809E-01	7.060E-01	6.315E-01	2.715E-01	8.244E-01	8.474E-01	8.181E-01	7.855E-01	8.106E-01	1.382E-01	7.840E-01	7.256E-01	7.249E-01	8.427E-01	6.342E-01	6.664E-01	4.216E-01	2.597E-02	5.561E-01	8.427E-01	6.807E-01
(pyruvate transport)	Local	7.06E-01	5.02E-03	1.49E-04	1.08E-03	1.51E-02	5.04E-03	8.95E-02	5.25E-02	4.62E-02	-2.22E-16	7.20E-03	8.34E-03	1.71E-03	-2.12E-02	7.05E-02	1.33E-02	-1.31E-19	6.21E-20	2.07E-07	5.29E-07	-3.54E-04
	Min	-1.17E+00	-1.88E+00	-1.30E-03	-4.94E-03	-6.65E-03	-5.20E-03	3.13E-06	7.62E-07	-1.39E-01	-8.88E-16	8.28E-06	3.66E-07	1.33E-07	-1.02E-01	1.11E-06	3.60E-07	-3.05E-18	-1.44E-16	1.92E-13	-3.17E-06	-4.04E-03
	Max	9.99E-01	6.81E-02	2.52E-02	1.49E+00	3.45E-01	7.09E-02	1.67E+00	8.25E-01	1.02E+00	8.88E-16	1.33E-01	7.52E-02	1.95E-02	4.43E-01	1.00E+00	1.68E-01	4.43E-18	1.72E-16	3.70E-06	6.02E-06	8.26E-04
	Normalized peak height	1.04E-01	2.60E-01	2.60E-01	5.20E-01	1.35E-01	8.03E-02	1.60E-01	1.49E-01	8.10E-02	4.15E-01	1.95E-02	4.88E-02	4.82E-02	8.18E-02	1.46E-01	8.71E-02	3.99E-01	5.61E-01	1.18E-01	4.51E-02	1.38E-01
	Peak sensitivity value	1.05E-03	2.83E-03	1.63E-05	3.06E-04	-1.36E-04	8.57E-05	8.36E-04	4.13E-04	9.67E-04	8.88E-19	1.40E-03	3.80E-05	9.88E-06	-6.81E-05	5.01E-04	8.42E-05	-2.74E-21	1.26E-19	1.85E-09	5.12E-09	5.04E-07
	Shapiro-Wilk score	7.907E-01	3.164E-01	5.428E-01	2.898E-01	7.599E-01	7.880E-01	7.902E-01	7.730E-01	5.335E-01	9.079E-01	6.904E-01	7.374E-01	7.476E-01	7.974E-01	5.946E-01	6.553E-01	5.570E-01	2.834E-01	6.554E-01	7.887E-01	6.166E-01

(phosphoglycerate kinase)	Local	7.06E-01	5.02E-03	1.49E-04	1.08E-03	1.51E-02	5.04E-03	8.95E-02	5.25E-02	4.62E-02	2.34E-17	7.20E-03	8.34E-03	1.71E-03	-2.12E-02	7.05E-02	1.33E-02	-1.31E-19	6.21E-20	2.07E-07	5.29E-07	-3.54E-04
	Min	-8.37E-01	-3.75E+00	-9.16E-05	-1.53E-04	-4.86E-03	-4.70E-03	1.53E-06	9.60E-07	-4.96E-02	-7.61E-16	1.81E-05	3.64E-07	6.47E-08	-9.91E-02	9.90E-07	3.81E-07	-3.09E-18	-4.17E-16	3.83E-13	-1.47E-06	-4.24E-03
	Max	9.99E-01	6.79E-02	2.51E-02	2.91E+00	3.61E-01	6.40E-02	1.57E+00	7.22E-01	7.29E-01	8.93E-16	1.30E-01	9.63E-02	1.88E-02	2.15E-01	1.00E+00	1.76E-01	3.60E-18	2.03E-16	4.15E-06	5.60E-06	8.17E-04
	Normalized peak height	7.88E-02	3.66E-01	2.01E-01	6.67E-01	1.17E-01	7.92E-02	1.55E-01	1.39E-01	6.58E-02	2.56E-01	2.01E-02	5.89E-02	4.47E-02	5.43E-02	1.46E-01	8.99E-02	4.02E-01	6.98E-01	1.26E-01	2.67E-02	1.36E-01
	Peak sensitivity value	9.95E-04	1.10E-03	2.19E-05	1.30E-03	8.32E-05	7.14E-05	7.88E-04	3.62E-04	5.73E-04	3.73E-19	9.93E-04	4.85E-05	9.44E-06	-2.12E-04	5.01E-04	8.86E-05	-1.31E-22	-2.69E-20	2.08E-09	7.65E-09	6.78E-07
	Shapiro-Wilk score	7.901E-01	1.860E-01	5.428E-01	1.772E-01	7.580E-01	7.856E-01	7.884E-01	7.708E-01	5.350E-01	7.971E-01	6.966E-01	7.431E-01	7.458E-01	8.157E-01	5.963E-01	6.633E-01	5.676E-01	1.382E-01	6.420E-01	7.888E-01	6.157E-01
(pyruvate kinase)	Local	7.06E-01	5.02E-03	1.49E-04	1.08E-03	1.51E-02	5.04E-03	8.95E-02	5.25E-02	4.62E-02	-2.12E-16	7.20E-03	8.34E-03	1.71E-03	-2.12E-02	7.05E-02	1.33E-02	-1.31E-19	6.21E-20	2.07E-07	5.29E-07	-3.54E-04
	Min	-6.38E-01	-1.25E+00	-3.49E-04	-2.79E-04	-1.02E-02	-4.41E-03	3.66E-06	1.35E-06	-4.25E-02	-8.69E-16	2.05E-05	2.08E-07	3.99E-08	-1.04E-01	5.08E-07	1.24E-07	-3.06E-18	-7.48E-17	2.38E-13	-1.35E-06	-4.74E-03
	Max	9.99E-01	5.66E-02	3.37E-02	9.99E-01	3.53E-01	6.27E-02	1.31E+00	7.41E-01	6.92E-01	8.16E-16	1.40E-01	8.73E-02	1.84E-02	3.20E-01	1.00E+00	1.71E-01	1.05E-17	1.01E-16	3.74E-06	6.82E-06	7.90E-04
	Normalized peak height	9.54E-02	1.80E-01	2.61E-01	4.63E-01	1.03E-01	8.33E-02	1.40E-01	1.39E-01	7.04E-02	3.88E-01	2.13E-02	5.49E-02	4.43E-02	7.81E-02	1.43E-01	8.63E-02	4.59E-01	5.45E-01	1.19E-01	3.35E-02	1.26E-01
	Peak sensitivity value	-3.99E-04	2.52E-03	8.66E-06	2.20E-04	1.13E-04	4.54E-05	6.60E-04	3.72E-04	4.43E-04	-2.26E-19	1.21E-03	4.39E-05	9.24E-06	-1.17E-04	5.00E-04	8.57E-05	2.15E-21	9.98E-21	1.87E-09	6.70E-09	-3.37E-06
	Shapiro-Wilk score	7.908E-01	2.848E-01	5.495E-01	2.670E-01	7.593E-01	7.895E-01	7.910E-01	7.736E-01	5.412E-01	9.596E-01	6.882E-01	7.392E-01	7.435E-01	8.014E-01	5.972E-01	6.655E-01	5.182E-01	3.605E-01	6.474E-01	7.826E-01	6.196E-01
(atp utilisation)	Local	7.06E-01	5.02E-03	1.49E-04	1.08E-03	1.51E-02	5.04E-03	8.95E-02	5.25E-02	4.62E-02	2.20E-21	7.20E-03	8.34E-03	1.71E-03	-2.12E-02	7.05E-02	1.33E-02	-1.31E-19	6.21E-20	2.07E-07	5.29E-07	-3.54E-04
	Min	-1.02E+00	-9.59E-01	-2.37E-03	-4.95E-02	-3.37E-01	-5.66E-02	-1.23E+00	-2.63E-01	-1.54E-01	-1.02E-11	-5.62E-03	-1.29E-03	-4.45E-04	-3.04E-01	-8.57E-03	-1.58E-03	-4.59E-18	-1.29E-16	-3.26E-07	-1.08E-06	-4.05E-03
	Max	2.75E+00	1.67E-01	3.80E-02	7.45E-01	3.38E-01	6.98E-02	1.37E+00	7.48E-01	9.81E-01	8.53E-12	1.32E-01	9.45E-02	2.29E-02	2.89E-01	1.00E+00	1.83E-01	1.76E-17	1.30E-16	4.77E-06	7.15E-06	8.34E-04
	Normalized peak height	1.35E-01	1.52E-01	2.21E-01	4.28E-01	1.98E-01	1.34E-01	1.13E-01	1.17E-01	8.01E-02	9.60E-01	2.10E-02	5.18E-02	5.45E-02	9.59E-02	9.44E-02	7.48E-02	5.13E-01	5.75E-01	1.02E-01	3.11E-02	1.13E-01
	Peak sensitivity value	-1.21E-03	2.01E-03	3.43E-05	1.55E-04	-3.81E-05	9.05E-05	2.13E-03	1.85E-05	1.09E-03	-4.11E-15	1.08E-03	1.00E-04	1.07E-05	-1.72E-04	9.91E-07	1.75E-04	5.52E-21	-6.76E-20	2.88E-09	1.07E-09	-3.12E-06
	Shapiro-Wilk score	7.902E-01	3.697E-01	5.240E-01	3.399E-01	7.568E-01	7.845E-01	7.876E-01	7.712E-01	5.376E-01	3.593E-02	6.855E-01	7.343E-01	7.359E-01	7.887E-01	5.944E-01	6.513E-01	3.739E-01	3.079E-01	6.486E-01	7.789E-01	6.090E-01
(glycerol kinase)	Local	9.22E+00	6.57E-02	-2.07E-03	-1.73E-02	-4.20E-01	-1.47E-01	-2.56E+00	-1.50E+00	-1.41E+00	1.05E-15	-2.06E-01	-2.39E-01	-4.91E-02	6.53E-01	-2.02E+00	-3.80E-01	3.75E-18	-1.82E-18	-5.91E-06	-1.61E-05	1.09E-02
	Min	-2.83E+01	-7.68E+00	-1.20E-01	-9.53E-01	-8.65E+00	-1.43E+00	-3.33E+01	-3.04E+01	-2.89E+01	-2.58E-13	-1.08E+00	-1.19E+00	-2.39E-01	4.74E-03	-7.72E+01	-2.11E+00	-1.82E-16	-3.84E-14	-2.35E-04	-5.49E-04	6.12E-06
	Max	7.87E+01	8.64E-01	6.98E-02	5.51E+00	2.85E+00	1.72E-01	1.92E+01	8.89E+00	1.30E-03	2.23E-13	3.77E-01	2.54E-01	6.10E-02	3.39E+00	1.53E+01	5.21E-01	8.33E-17	2.84E-15	3.05E-06	4.19E-10	1.54E-01
	Normalized peak height	1.25E-01	1.06E-01	8.09E-02	2.36E-01	1.99E-02	1.97E-02	7.72E-02	1.06E-01	3.72E-02	7.28E-01	4.53E-02	7.77E-02	8.93E-02	1.86E-02	1.95E-01	1.10E-01	5.62E-01	6.66E-01	3.25E-01	1.64E-02	3.08E-02
	Peak sensitivity value	-7.55E-02	1.40E-02	2.41E-04	1.16E-03	-6.36E-03	-3.13E-02	2.78E-02	3.93E-04	-1.26E+00	-1.68E-16	1.47E-03	1.66E-03	2.35E-04	9.65E-01	-3.48E-02	4.76E-04	-1.53E-20	1.44E-17	7.25E-08	-5.21E-06	5.44E-04
	Shapiro-Wilk score	8.278E-01	8.308E-01	7.215E-01	4.025E-01	8.030E-01	7.122E-01	7.290E-01	6.845E-01	5.429E-01	1.175E-01	7.911E-01	8.188E-01	8.165E-01	8.581E-01	5.414E-01	8.018E-01	5.412E-01	5.001E-02	6.011E-01	7.632E-01	7.277E-01
(phosphoglycerate mutase)	Local	7.06E-01	5.02E-03	1.49E-04	1.08E-03	1.51E-02	5.04E-03	8.95E-02	5.25E-02	4.62E-02	2.10E-17	7.20E-03	8.34E-03	1.71E-03	-2.12E-02	7.05E-02	1.33E-02	-1.31E-19	6.21E-20	2.07E-07	5.29E-07	-3.54E-04
	Min	-2.42E+00	-1.09E+01	-4.73E-04	-2.76E-03	-6.05E-03	-2.49E-03	3.99E-06	1.61E-06	-4.68E-01	-6.33E-16	1.84E-05	3.18E-07	9.47E-08	-9.25E-02	9.06E-07	2.69E-07	-4.42E-18	-4.00E-16	8.77E-14	-1.06E-05	-3.73E-03
	Max	9.99E-01	7.40E-02	6.43E-02	8.34E+00	5.20E-01	9.93E-02	4.32E+00	7.98E-01	1.57E+00	6.18E-16	1.54E-01	9.30E-02	3.40E-02	8.76E-01	1.00E+00	1.93E-01	6.18E-18	1.25E-16	4.82E-06	6.32E-06	4.73E-04
	Normalized peak height	1.44E-01	7.70E-01	3.61E-01	7.92E-01	1.76E-01	9.93E-02	2.48E-01	1.48E-01	1.16E-01	2.41E-01	2.32E-02	5.90E-02	7.78E-02	1.07E-01	1.46E-01	9.55E-02	4.26E-01	6.74E-01	1.36E-01	5.08E-02	1.51E-01
	Peak sensitivity value	-5.22E-04	2.42E-03	1.32E-05	1.41E-03	-3.02E-06	1.06E-04	2.17E-03	4.01E-04	1.78E-03	5.37E-19	1.33E-03	4.68E-05	1.71E-05	-5.58E-06	5.01E-04	9.65E-05	3.93E-21	-2.11E-21	2.41E-09	1.74E-08	-4.14E-07
	Shapiro-Wilk score	7.908E-01	4.902E-02	4.962E-01	4.872E-02	7.572E-01	7.841E-01	7.869E-01	7.709E-01	5.309E-01	8.189E-01	6.876E-01	7.320E-01	7.372E-01	7.440E-01	5.947E-01	6.544E-01	5.536E-01	1.693E-01	6.411E-01	7.815E-01	6.083E-01
(enolase)	Local	7.06E-01	5.02E-03	1.49E-04	1.08E-03	1.51E-02	5.04E-03	8.95E-02	5.25E-02	4.62E-02	2.10E-17	7.20E-03	8.34E-03	1.71E-03	-2.12E-02	7.05E-02	1.33E-02	-1.31E-19	6.21E-20	2.07E-07	5.29E-07	-3.54E-04
	Min	-1.52E+00	-1.51E+00	-8.27E-05	-1.24E-04	-3.87E-02	-4.15E-03	1.82E-06	1.61E-06	-7.83E-02	-7.97E-16	1.10E-05	2.78E-07	4.99E-08	-1.13E-01	5.30E-07	1.71E-07	-2.71E-18	-1.13E-16	1.59E-13	-2.63E-06	-3.20E-03
	Max	9.99E-01	6.48E-02	1.85E-02	1.21E+00	4.17E-01	7.71E-02	1.71E+00	7.54E-01	8.74E-01	7.24E-16	1.21E-01	8.59E-02	2.22E-02	3.64E-01	1.00E+00	1.61E-01	3.48E-18	8.55E-17	3.15E-06	8.75E-06	9.10E-04
	Normalized peak height	1.30E-01	2.13E-01	1.58E-01	5.31E-01	1.31E-01	9.12E-02	1.62E-01	1.41E-01	8.02E-02	2.51E-01	1.80E-02	5.35E-02	5.39E-02	6.68E-02	1.45E-01	8.32E-02	3.90E-01	4.56E-01	1.12E-01	4.49E-02	1.29E-01
	Peak sensitivity value	-7.65E-05	2.47E-03	1.95E-05	4.80E-04	-2.47E-04	3.91E-05	8.59E-04	3.79E-04	2.85E-04	6.39E-19	1.16E-03	4.32E-05	1.12E-05	2.12E-05	5.00E-04	8.04E-05	2.01E-21	9.21E-20	1.58E-09	7.88E-09	4.28E-07
	Shapiro-Wilk score	7.908E-01	2.571E-01	5.591E-01	2.370E-01	7.560E-01	7.859E-01	7.864E-01	7.689E-01	5.345E-01	8.202E-01	6.906E-01	7.335E-01	7.423E-01	7.989E-01	5.972E-01	6.608E-01	5.785E-01	3.310E-01	6.477E-01	7.786E-01	6.150E-01
(adenylate kinase cytosol)	Local	INF	INF	INF	INF	INF	INF	INF	INF	INF	INF	INF	INF	INF	INF	INF	INF	INF	INF	INF	INF	INF
	Min	-INF	-INF	-INF	-INF	-INF	-INF	-INF	-INF	-INF	-INF	-INF	-INF	-INF	-INF	-INF	-INF	-INF	-INF	-INF	-INF	-INF
	Max	INF	INF	INF	INF	INF	INF	INF	INF	INF	INF	INF	INF	INF	INF	INF	INF	INF	INF	INF	INF	INF
	Normalized peak height	1.30E-01	4.35E-02	4.35E-02	4.35E-02	5.22E-01	3.91E-01	3.04E-01	4.78E-01	1.30E-01	2.17E-01	8.70E-02	8.70E-02	2.61E-01	1.74E-01	8.70E-02	8.70E-02	4.35E-02	1.74E-01	8.70E-02	1.74E-01	2.17E-01
	Peak sensitivity value	-4.63E-05	-1.34E-01	-4.64E-02	-1.23E-01	3.73E-06	-3.90E-07	-6.95E-06	-2.20E-05	-8.24E-05	2.71E-10	8.99E-02	-2.29E-02	-1.70E-04	-1.17E-06	-1.76E-02	-2.64E-03	-2.88E-12	-4.73E-14	4.63E-03	-2.45E-05	7.35E-09
	Shapiro-Wilk score	9.804E-01	8.512E-01	7.569E-01	8.742E-01	3.078E-01	2.248E-01	3.502E-01	3.189E-01	4.526E-01	8.717E-01	5.638E-01	7.834E-01	8.333E-01	4.309E-01	7.085E-01	7.484E-01	9.115E-01	5.462E-01	9.394E-01	3.995E-01	8.005E-01
(adenylate kinase glycosome)	Local	-INF	-INF	-INF	-INF	-INF	-INF	-INF	-INF	-INF	-INF	-INF	-INF	-INF	-INF	-INF	-INF	-INF	-INF	-INF	-INF	-INF
	Min	-INF	-INF	-INF	-INF	-INF	-INF	-INF	-INF	-INF	-INF	-INF	-INF	-INF	-INF	-INF	-INF	-INF	-INF	-INF	-INF	-INF
	Max	INF	INF	INF	INF	INF	INF	INF	INF	INF	INF	INF	INF	INF	INF	INF	INF	INF	INF	INF	INF	INF
	Normalized peak height	2.42E-01	1.95E-01	1.91E-01	1.93E-01	3.55E-01	3.24E-01	2.78E-01	3.47E-01	6.80E-01	3.21E-01	1.33E-01	1.45E-01	1.29E-01	4.92E-01	5.17E-01	1.47E-01	5.22E-01	9.69E-02	3.54E-01	3.44E-01	5.95E-01
	Peak sensitivity value	-1.73E-02	-9.78E-03	-5.89E-05	1.07E-03	9.15E-03	1.53E-03	-5.20E-02	2.84E-02	-7.16E-03	-1.78E-07	-5.35E-02	6.85E-04	-2.26E-04	6.11E-03	-3.45E-02	2.61E-03	-2.42E-16	1.67E-13	-1.65E-02	-8.63E-03	1.53E-05
	Shapiro-Wilk score	6.188E-01	6.285E-01	6.535E-01	6.323E-01	4.664E-01	5.110E-01	5.017E-01	4.739E-01	3.080E-01	5.266E-01	6.417E-01	6.947E-01	7.147E-01	5.030E-01	3.017E-01	6.768E-01	3.208E-01	7.256E-01	3.665E-01	4.842E-01	3.952E-01
(3phosphoglycerate transport)	Local	7.06E-01	5.02E-03	1.49E-04	1.08E-03	1.51E-02	5.04E-03	8.95E-02	5.25E-02	4.62E-02	2.34E-17	7.20E-03	8.34E-03	1.71E-03	-2.12E-02	7.05E-02	1.33E-02	-1.31E-19	6.20E-20	2.07E-07	5.29E-07	-3.54E-04
	Min	-1.15E+00	-1.83E+00	-2.57E-04	-1.19E-03	-6.03E-03	-3.22E-03	2.45E-06	1.13E-06	-7.95E-02	-7.63E-16	1.63E-05	5.45E-07	1.40E-07	-1.05E-01	1.56E-06	4.42E-07	-4.30E-18	-1.20E-16	-1.32E-07	-2.20E-06	-4.69E-03
	Max	9.99E-01	6.89E-02	2.64E-02	1.43E+00	4.47E-01	5.94E-02	1.52E+00	1.05E+00	7.42E-01	8.31E-16	1.29E-01	8.83E-02	2.36E-02	4.05E-01	1.00E+00	1.96E-01	6.79E-18	1.12E-16	4.70E-06	6.06E-06	4.82E-04
	Normalized peak height	1.15E-01	2.25E-01	2.03E-01	3.03E-01	1.40E-01	7.61E-02	1.52E-01	1.66E-01	6.70E-02	2.59E-01	1.90E-02	5.52E-02	5.69E-02	9.54E-02	1.43E-01	9.60E-02	4.32E-01	5.20E-01	7.47E-02	4.13E-02	1.80E-01
	Peak sensitivity value	9.11E-04	3.51E-03	2.30E-05	9.53E-04	9.33E-05	6.20E-05	7.62E-04	5.24E-04	6.15E-04	-2.39E-19	1.24E-03	4.47E-05	1.19E-05	-2.24E-04	5.01E-04	9.83E-05	-3.85E-21	-9.45E-20	5.43E-09	4.96E-09	-1.66E-06
	Shapiro-Wilk score	7.916E-01	2.877E-01	5.574E-01	2.694E-01	7.580E-01	7.877E-01	7.879E-01	7.716E-01	5.348E-01	7.976E-01	6.911E-01	7.354E-01	7.420E-01	7.872E-01	5.960E-01	6.551E-01	5.475E-01	2.963E-01	6.451E-01	7.883E-01	6.118E-01
(gly3p dhap antiporter)	Local	4.14E-01	2.95E-03	2.25E-04	1.71E-03	3.00E-02	1.02E-02	1.80E-01	1.06E-01	9.60E-02	8.65E-17	1.45E-02	1.68E-02	3.45E-03	-4.42E-02	1.42E-01	2.67E-02	-2.64E-19	1.27E-19	4.16E-07	1.10E-06	-7.39E-04
	Min	-2.07E+00	-1.63E+00	-7.24E-03	-4.76E-02	-2.96E-01	-1.04E-02	-9.24E-01	-3.83E-01	1.32E-04	-1.28E-14	-3.91E-02	-2.10E-02	-4.43E-03	-1.01E+00	-2.66E+00	-3.02E-02	-6.29E-18	-1.15E-16	-1.09E-07	8.49E-09	-1.23E-02
	Max	3.90E+00	2.56E-01	1.36E-02	1.35E+00	8.59E-01	9.69E-02	1.95E+00	1.32E+00	9.97E-01	8.30E-14	1.39E-01	1.20E-01	3.70E-02	-1.00E-04	3.02E+00	2.10E-01	1.05E-16	1.07E-14	1.12E-05	2.73E-05	-1.90E-07
	Normalized peak height	7.19E-02	1.82E-01	1.75E-01	4.39E-01	1.49E-01	4.76E-02	1.17E-01	1.20E-01	2.04E-02	7.95E-01	1.88E-02	5.33E-02	6.42E-02	3.24E-02	2.50E-01	7.64E-02	7.11E-01	8.25E-01	1.13E-01	2.77E-02	3.56E-02
	Peak sensitivity value	5.35E-03	2.38E-03	1.13E-05	7.14E-04	2.77E-04	5.78E-05	1.88E-04	1.26E-03	1.63E-03	-1.88E-19	1.42E-03	1.35E-04	2.93E-05	-1.62E-03	1.12E-03	1.85E-04	-4.72E-21	-1.42E-18	-1.25E-09	7.66E-08	-4.32E-05
	Shapiro-Wilk score	8.810E-01	7.319E-01	6.497E-01	3.039E-01	8.240E-01	8.449E-01	8.169E-01	7.836E-01	8.071E-01	9.878E-02	7.840E-01	7.266E-01	7.269E-01	8.405E-01	6.348E-01	6.746E-01	1.308E-01	2.653E-02	5.490E-01	8.450E-01	6.893E-01
(glycerol transport)	Local	9.22E+00	6.57E-02	-2.07E-03	-1.73E-02	-4.20E-01	-1.47E-01	-2.56E+00	-1.50E+00	-1.41E+00	1.05E-15	-2.06E-01	-2.39E-01	-4.91E-02	6.53E-01	-2.02E+00	-3.80E-01	3.75E-18	-1.82E-18	-5.91E-06	-1.61E-05	1.09E-02
	Min	-2.54E+01	-3.98E+00	-7.24E-02	-7.56E-01	-9.26E+00	-1.53E+00	-4.91E+01	-2.45E+01	-5.10E+01	-2.38E-13	-1.07E+00	-9.47E-01	-2.07E-01	3.81E-03	-6.25E+01	-2.33E+00	-1.54E-16	-1.81E-14	-2.31E-04	-8.79E-04	8.07E-06
	Max	7.95E+01	8.30E-01	6.27E-02	2.85E+00	3.53E+00	2.07E-01	1.18E+01	1.19E+01	2.33E-01	3.18E-13	3.53E-01	2.60E-01	4.32E-02	4.50E+00	1.28E+01	3.81E-01	1.37E-16	2.41E-15	4.56E-06	1.46E-07	1.55E-01
	Normalized peak height	1.11E-01	6.99E-02	5.85E-02	1.33E-01	2.26E-02	2.13E-02	8.77E-02	1.05E-01	6.42E-02	7.34E-01	4.31E-02	7.89E-02	7.45E-02	2.50E-02	2.09E-01	1.07E-01	5.73E-01	6.14E-01	2.63E-01	2.60E-02	3.14E-02
	Peak sensitivity value	-1.08E-01	9.72E-03	2.40E-04	3.30E-03	-1.79E-02	-3.19E-02	-1.68E-02	4.52E-03	-1.23E+00	-2.09E-16	1.57E-03	1.13E-03	1.18E-04	9.64E-01	-1.40E-02	2.20E-03	1.93E-20	6.31E-19	-2.22E-08	-3.81E-06	7.04E-04
	Shapiro-Wilk score	8.241E-01	8.610E-01	7.178E-01	5.117E-01	8.105E-01	7.074E-01	7.223E-01	6.898E-01	4.926E-01	1.153E-01	7.891E-01	8.194E-01	8.163E-01	8.574E-01	5.407E-01	7.981E-01	5.552E-01	1.190E-01	5.996E-01	7.560E-01	7.283E-01

Table S17: Flux control coefficients for *Trypanosoma brucei* model with parameter variation of 0.1 – 10×

	(glucosetransport)	(hexokinase)	(phosphoglycerateisomerase)	(phophofructokinase)	(aldolase)	(triosephosphateisomerase)	(glyceraldehyde3phosphatedehydrogenase)	(glycerol3phosphatedehydrogenase)	(glycerol3phosphateoxidase)	(pyruvatetransport)	(phosphoglyceratekinase)	(pyruvatekinase)	(atputilisation)	(glycerolkinase)	(phosphoglyceratemutase)	(enolase)	(adenylatekinasecytosol)	(adenylatekinaseglycosome)	(3phosphoglyceratetransport)	(gly3pdhapantiporter)	(glyceroltransport)
(glucose transport)<br>Local<br>Min<br>Max<br>Normalized peak height<br>Peak sensitivity value<br>Shapiro-Wilk score	9.79E-01<br>-2.17E+01<br>INF<br>3.65E-01<br>-2.69E-03<br>6.882E-01	6.97E-03<br>-5.38E+02<br>INF<br>8.74E-01<br>-1.86E-01<br>3.324E-02	7.82E-05<br>-9.05E-02<br>INF<br>6.70E-01<br>-1.41E-03<br>6.334E-02	4.95E-04<br>-1.02E+01<br>INF<br>8.56E-01<br>-1.85E-01<br>2.416E-02	1.13E-03<br>-1.28E-01<br>INF<br>4.78E-01<br>7.54E-04<br>3.417E-01	1.79E-04<br>-5.58E-01<br>INF<br>4.87E-01<br>-6.78E-04<br>2.612E-01	4.63E-03<br>-1.14E+00<br>INF<br>4.15E-01<br>1.47E-03<br>6.563E-01	2.72E-03<br>-4.94E-01<br>INF<br>3.83E-01<br>-4.15E-04<br>5.722E-01	-3.97E-04<br>-1.76E+00<br>INF<br>3.95E-01<br>4.90E-03<br>5.748E-01	-1.47E-18<br>-3.84E-13<br>INF<br>9.33E-01<br>1.25E-16<br>1.786E-02	3.73E-04<br>-2.80E-01<br>INF<br>3.66E-01<br>-2.67E-03<br>2.067E-01	4.31E-04<br>-2.44E-02<br>INF<br>9.81E-01<br>3.48E-01<br>2.618E-03	8.87E-05<br>-7.43E+02<br>INF<br>9.90E-01<br>-8.29E-02<br>1.675E-03	3.95E-04<br>-4.19E-01<br>INF<br>3.84E-01<br>-2.65E-03<br>4.302E-01	3.65E-03<br>-2.63E-01<br>INF<br>6.39E-01<br>1.19E-03<br>3.765E-01	6.87E-04<br>-5.78E-02<br>INF<br>5.51E-01<br>-6.43E-04<br>1.971E-01	-6.78E-21<br>-2.38E-07<br>INF<br>1.00E+00<br>-8.47E-11<br>2.863E-04	1.71E-21<br>-7.13E-14<br>INF<br>9.28E-01<br>-1.10E-16<br>3.246E-03	1.07E-08<br>-1.07E-06<br>INF<br>7.00E-01<br>-1.33E-07<br>8.548E-02	-4.55E-09<br>-4.95E-05<br>INF<br>5.48E-01<br>-5.18E-08<br>2.514E-01	6.60E-06<br>-1.61E-04<br>INF<br>7.50E-01<br>-5.46E-05<br>9.708E-02
(hexokinase)<br>Local<br>Min<br>Max<br>Normalized peak height<br>Peak sensitivity value<br>Shapiro-Wilk score	9.79E-01<br>-3.69E+01<br>INF<br>4.71E-01<br>-2.19E-02<br>6.347E-01	6.97E-03<br>-3.48E+02<br>INF<br>4.86E-01<br>2.47E-01<br>5.080E-02	7.82E-05<br>-2.03E+00<br>INF<br>9.74E-01<br>6.89E-03<br>1.709E-02	4.95E-04<br>-1.44E+02<br>INF<br>9.36E-01<br>4.82E-02<br>3.568E-02	1.13E-03<br>-1.48E-01<br>INF<br>4.65E-01<br>-1.09E-03<br>3.186E-01	1.79E-04<br>-2.62E-01<br>INF<br>5.03E-01<br>1.46E-05<br>2.604E-01	4.63E-03<br>-7.76E+00<br>INF<br>4.22E-01<br>6.47E-03<br>6.995E-01	2.72E-03<br>-4.64E-01<br>INF<br>4.06E-01<br>2.39E-03<br>5.813E-01	-3.97E-04<br>-4.09E+00<br>INF<br>4.14E-01<br>2.55E-03<br>5.825E-01	-1.47E-18<br>-1.40E-13<br>INF<br>8.55E-01<br>-1.89E-16<br>2.841E-02	3.73E-04<br>-1.24E-01<br>INF<br>6.26E-01<br>1.84E-03<br>2.071E-01	4.31E-04<br>-9.29E-03<br>INF<br>9.83E-01<br>3.59E-01<br>3.296E-03	8.87E-05<br>-7.37E+02<br>INF<br>9.90E-01<br>-1.12E-02<br>2.270E-03	3.95E-04<br>-1.44E+01<br>INF<br>5.11E-01<br>2.44E-03<br>4.369E-01	3.65E-03<br>-1.55E-02<br>INF<br>4.33E-01<br>-1.13E-03<br>3.754E-01	6.87E-04<br>-1.44E-02<br>INF<br>5.62E-01<br>-1.19E-03<br>1.955E-01	-6.78E-21<br>-5.35E-08<br>INF<br>1.00E+00<br>-2.49E-12<br>4.041E-04	1.71E-21<br>-3.27E-13<br>INF<br>9.92E-01<br>-3.02E-17<br>1.998E-03	1.07E-08<br>-8.33E-07<br>INF<br>7.24E-01<br>-1.97E-08<br>9.447E-02	-4.55E-09<br>-6.36E-05<br>INF<br>4.90E-01<br>-7.89E-08<br>2.145E-01	6.60E-06<br>-2.99E-04<br>INF<br>8.34E-01<br>3.91E-05<br>9.236E-02
(phosphoglycerate isomerase)<br>Local<br>Min<br>Max<br>Normalized peak height<br>Peak sensitivity value<br>Shapiro-Wilk score	9.79E-01<br>-3.80E+01<br>INF<br>4.38E-01<br>-1.24E-02<br>6.673E-01	6.97E-03<br>-2.10E+02<br>INF<br>7.36E-01<br>-6.17E-02<br>5.269E-02	7.82E-05<br>-8.98E-02<br>INF<br>6.65E-01<br>2.50E-03<br>5.950E-02	4.95E-04<br>-3.17E+00<br>INF<br>7.40E-01<br>-8.15E-02<br>3.881E-02	1.13E-03<br>-1.90E-01<br>INF<br>3.04E-01<br>-1.00E-03<br>3.472E-01	1.79E-04<br>-2.20E-01<br>INF<br>4.74E-01<br>-1.85E-04<br>2.660E-01	4.63E-03<br>-5.30E-01<br>INF<br>4.36E-01<br>3.82E-03<br>6.459E-01	2.72E-03<br>-2.02E-01<br>INF<br>4.49E-01<br>1.88E-03<br>5.587E-01	-3.97E-04<br>-1.68E+00<br>INF<br>3.95E-01<br>1.87E-03<br>5.804E-01	-1.47E-18<br>-2.18E-13<br>INF<br>8.49E-01<br>1.40E-16<br>3.782E-02	3.73E-04<br>-1.53E-01<br>INF<br>6.34E-01<br>1.03E-03<br>2.078E-01	4.31E-04<br>-9.16E-04<br>INF<br>9.73E-01<br>4.08E-02<br>3.553E-02	8.87E-05<br>-8.24E+01<br>INF<br>9.44E-01<br>2.72E-02<br>2.053E-02	3.95E-04<br>-2.42E-01<br>INF<br>4.16E-01<br>-9.39E-03<br>3.508E-01	3.65E-03<br>-2.36E-03<br>INF<br>6.15E-01<br>8.21E-04<br>3.748E-01	6.87E-04<br>-1.43E-03<br>INF<br>6.65E-01<br>-2.10E-04<br>1.986E-01	-6.78E-21<br>-1.55E-10<br>INF<br>1.00E+00<br>9.19E-14<br>7.682E-04	1.71E-21<br>-5.75E-14<br>INF<br>9.98E-01<br>-5.38E-16<br>8.211E-04	1.07E-08<br>-1.13E-06<br>INF<br>8.39E-01<br>4.55E-09<br>5.966E-02	-4.55E-09<br>-4.97E-05<br>INF<br>7.09E-01<br>-4.88E-08<br>1.134E-01	6.60E-06<br>-3.66E-04<br>INF<br>6.48E-01<br>-7.92E-05<br>9.834E-02
(phophofructokinase)<br>Local<br>Min<br>Max<br>Normalized peak height<br>Peak sensitivity value<br>Shapiro-Wilk score	9.79E-01<br>-2.71E+01<br>6.02E+00<br>2.53E-01<br>-1.90E-02<br>6.879E-01	6.97E-03<br>-2.15E+02<br>1.73E+01<br>7.55E-01<br>-2.53E-02<br>6.237E-02	7.82E-05<br>-3.14E-01<br>2.21E+01<br>9.67E-01<br>1.10E-02<br>2.270E-02	4.95E-04<br>-1.61E+01<br>2.16E+02<br>8.97E-01<br>3.95E-02<br>4.326E-02	1.13E-03<br>-5.84E-01<br>1.89E+00<br>4.64E-01<br>1.19E-03<br>3.535E-01	1.79E-04<br>-5.38E-01<br>2.23E+00<br>4.75E-01<br>-6.76E-04<br>2.656E-01	4.63E-03<br>-2.18E+00<br>1.93E+01<br>4.07E-01<br>3.17E-03<br>7.188E-01	2.72E-03<br>-6.06E-01<br>5.25E+00<br>3.86E-01<br>-2.36E-04<br>5.850E-01	-3.97E-04<br>-2.49E+00<br>4.46E+00<br>3.93E-01<br>-6.59E-05<br>5.891E-01	-1.47E-18<br>-1.27E-13<br>2.69E-13<br>8.61E-01<br>1.24E-16<br>3.210E-02	3.73E-04<br>-1.88E+00<br>6.45E+00<br>6.33E-01<br>2.80E-04<br>2.054E-01	4.31E-04<br>-1.08E-02<br>6.96E+02<br>9.81E-01<br>3.37E-01<br>4.057E-03	8.87E-05<br>-6.95E+02<br>1.97E+00<br>9.90E-01<br>2.30E-01<br>2.857E-03	3.95E-04<br>-1.49E-01<br>1.18E+01<br>4.39E-01<br>-2.89E-04<br>4.455E-01	3.65E-03<br>-2.13E+00<br>3.18E+00<br>6.70E-01<br>1.26E-03<br>3.752E-01	6.87E-04<br>-3.59E-02<br>1.63E+00<br>6.36E-01<br>-1.43E-04<br>1.966E-01	-6.78E-21<br>-1.88E-08<br>5.84E-08<br>1.00E+00<br>3.04E-11<br>5.035E-04	1.71E-21<br>-1.25E-14<br>1.02E-14<br>7.98E-01<br>3.53E-19<br>8.723E-02	1.07E-08<br>-5.95E-06<br>1.90E-04<br>7.81E-01<br>2.73E-08<br>1.203E-01	-4.55E-09<br>-6.66E-05<br>1.27E-04<br>6.09E-01<br>3.37E-08<br>1.886E-01	6.60E-06<br>-4.75E-04<br>2.92E-01<br>8.09E-01<br>-3.70E-05<br>9.320E-02
(aldolase)<br>Local<br>Min<br>Max<br>Normalized peak height<br>Peak sensitivity value<br>Shapiro-Wilk score	9.79E-01<br>-9.01E+00<br>2.21E+00<br>2.95E-01<br>-4.19E-03<br>7.236E-01	6.97E-03<br>-1.09E+03<br>4.42E+00<br>8.82E-01<br>-4.95E-01<br>1.561E-02	7.82E-05<br>-1.01E-01<br>6.18E+00<br>9.21E-01<br>2.21E-03<br>5.003E-02	4.95E-04<br>-3.29E+00<br>1.08E+03<br>9.76E-01<br>4.89E-01<br>1.139E-02	1.13E-03<br>-3.97E-02<br>2.89E+00<br>4.08E-01<br>-1.61E-04<br>3.494E-01	1.79E-04<br>-2.93E-01<br>2.16E+00<br>5.24E-01<br>6.08E-04<br>2.534E-01	4.63E-03<br>-5.48E-01<br>1.14E+01<br>3.30E-01<br>-3.87E-03<br>7.394E-01	2.72E-03<br>-1.16E-01<br>2.98E+00<br>3.47E-01<br>-1.13E-04<br>5.877E-01	-3.97E-04<br>-1.20E+00<br>5.46E+00<br>3.91E-01<br>-1.09E-03<br>5.835E-01	1.48E-16<br>-1.28E-14<br>6.54E-14<br>7.18E-01<br>3.42E-17<br>9.272E-02	3.73E-04<br>-3.05E-02<br>5.29E+00<br>5.12E-01<br>-1.20E-03<br>2.065E-01	4.31E-04<br>-1.90E-03<br>4.13E+02<br>9.78E-01<br>2.05E-01<br>6.694E-03	8.87E-05<br>-4.12E+02<br>7.87E-01<br>9.86E-01<br>1.67E-01<br>3.717E-03	3.95E-04<br>-4.64E-01<br>7.92E+00<br>4.39E-01<br>1.37E-03<br>4.678E-01	3.65E-03<br>-1.08E-02<br>2.52E+00<br>6.13E-01<br>5.54E-04<br>3.722E-01	6.87E-04<br>-1.21E-03<br>2.45E+00<br>6.82E-01<br>1.38E-05<br>1.956E-01	-6.78E-21<br>-2.54E-11<br>1.34E-09<br>1.00E+00<br>-6.25E-14<br>4.295E-04	1.71E-21<br>-1.50E-14<br>5.67E-15<br>7.90E-01<br>4.52E-19<br>9.064E-02	1.07E-08<br>-6.62E-07<br>1.48E-04<br>7.38E-01<br>7.63E-09<br>1.252E-01	-4.55E-09<br>-1.11E-04<br>1.48E-04<br>5.10E-01<br>1.29E-07<br>1.837E-01	6.60E-06<br>-1.37E-03<br>2.47E-01<br>8.15E-01<br>-7.49E-06<br>1.016E-01
(triosephosphate isomerase)<br>Local<br>Min<br>Max<br>Normalized peak height<br>Peak sensitivity value<br>Shapiro-Wilk score	4.14E-01<br>-2.35E+01<br>INF<br>5.23E-01<br>3.21E-02<br>5.203E-01	2.95E-03<br>-1.89E+03<br>INF<br>9.61E-01<br>-6.79E-01<br>5.832E-03	2.25E-04<br>-5.61E+00<br>INF<br>9.52E-01<br>6.15E-03<br>3.480E-02	1.71E-03<br>-5.30E+00<br>INF<br>9.55E-01<br>-5.75E-01<br>4.500E-03	3.00E-02<br>-3.34E+00<br>INF<br>5.21E-01<br>-4.69E-04<br>3.391E-01	1.02E-02<br>-2.49E-01<br>INF<br>4.36E-01<br>8.40E-04<br>2.993E-01	1.80E-01<br>-1.31E+01<br>INF<br>2.46E-01<br>-1.12E-02<br>6.954E-01	1.06E-01<br>-3.18E+01<br>INF<br>4.31E-01<br>-8.53E-03<br>4.332E-01	9.60E-02<br>-1.20E+00<br>INF<br>2.79E-01<br>5.32E-03<br>7.369E-01	8.65E-17<br>-1.09E-13<br>INF<br>8.45E-01<br>-2.33E-18<br>3.412E-02	1.45E-02<br>-4.75E-01<br>INF<br>6.10E-01<br>2.52E-03<br>2.186E-01	1.68E-02<br>-4.47E+00<br>INF<br>8.53E-01<br>-9.05E-02<br>1.912E-02	3.45E-03<br>-1.89E+02<br>INF<br>8.35E-01<br>-7.88E-02<br>1.089E-02	-4.42E-02<br>-1.65E+01<br>INF<br>4.41E-01<br>-2.19E-03<br>4.339E-01	1.42E-01<br>-1.04E+01<br>INF<br>6.02E-01<br>-1.30E-02<br>3.406E-01	2.67E-02<br>-2.16E+00<br>INF<br>6.09E-01<br>-2.30E-03<br>2.053E-01	-2.64E-19<br>-2.69E-09<br>INF<br>1.00E+00<br>9.74E-12<br>3.205E-04	1.27E-19<br>-3.05E-12<br>INF<br>9.84E-01<br>-1.51E-15<br>3.626E-04	4.16E-07<br>-4.62E-05<br>INF<br>8.42E-01<br>4.98E-08<br>6.612E-02	1.10E-06<br>-7.15E-06<br>INF<br>4.19E-01<br>5.63E-08<br>2.287E-01	-7.39E-04<br>-4.40E-01<br>INF<br>6.95E-01<br>1.09E-04<br>1.386E-01
(glyceraldehyde3phosphate dehydrogenase)<br>Local<br>Min<br>Max<br>Normalized peak height<br>Peak sensitivity value<br>Shapiro-Wilk score	7.06E-01<br>-INF<br>3.67E+00<br>2.24E-01<br>-6.09E-03<br>7.380E-01	5.02E-03<br>-INF<br>1.14E+01<br>8.93E-01<br>3.76E-02<br>3.102E-02	1.49E-04<br>-INF<br>5.16E+00<br>9.10E-01<br>9.63E-04<br>5.335E-02	1.08E-03<br>-INF<br>5.38E+02<br>9.11E-01<br>-1.03E-01<br>2.327E-02	1.51E-02<br>-INF<br>2.08E+00<br>3.61E-01<br>-5.45E-04<br>3.427E-01	5.04E-03<br>-INF<br>2.17E+00<br>3.64E-01<br>-7.74E-04<br>2.824E-01	8.95E-02<br>-INF<br>7.18E+00<br>3.11E-01<br>2.42E-03<br>7.669E-01	5.25E-02<br>-INF<br>2.81E+00<br>1.89E-01<br>-1.15E-03<br>5.938E-01	4.62E-02<br>-INF<br>3.12E+00<br>2.09E-01<br>1.91E-03<br>5.911E-01	1.18E-16<br>-INF<br>2.19E-14<br>7.91E-01<br>-3.95E-18<br>1.464E-01	7.20E-03<br>-INF<br>6.15E+00<br>4.63E-01<br>-2.58E-03<br>2.060E-01	8.34E-03<br>-INF<br>3.28E+02<br>9.76E-01<br>6.43E-02<br>8.524E-03	1.71E-03<br>-INF<br>1.27E+00<br>9.84E-01<br>1.17E-01<br>5.503E-03	-2.12E-02<br>-INF<br>3.82E+00<br>4.54E-01<br>1.13E-03<br>3.776E-01	7.05E-02<br>-INF<br>1.04E+00<br>4.89E-01<br>3.31E-04<br>3.852E-01	1.33E-02<br>-INF<br>1.20E+00<br>4.97E-01<br>-3.72E-04<br>2.033E-01	-1.31E-19<br>-INF<br>1.59E-09<br>1.00E+00<br>-1.92E-12<br>8.702E-04	6.21E-20<br>-INF<br>9.24E-14<br>9.90E-01<br>-3.30E-17<br>2.363E-03	2.07E-07<br>-INF<br>2.60E-04<br>5.46E-01<br>-1.03E-07<br>1.300E-01	5.29E-07<br>-INF<br>2.59E-04<br>8.08E-01<br>9.77E-08<br>1.090E-01	-3.54E-04<br>-INF<br>2.39E-01<br>8.16E-01<br>-1.16E-04<br>1.410E-01
(glycerol3phosphate dehydrogenase)<br>Local<br>Min<br>Max<br>Normalized peak height<br>Peak sensitivity value<br>Shapiro-Wilk score	7.06E-01<br>-1.62E+01<br>2.39E+00<br>2.57E-01<br>-9.51E-03<br>7.205E-01	5.02E-03<br>-2.12E+02<br>1.59E+01<br>7.89E-01<br>1.33E-02<br>4.704E-02	1.49E-04<br>-2.83E-01<br>2.76E+00<br>5.55E-01<br>-1.35E-03<br>5.954E-02	1.08E-03<br>-1.44E+01<br>2.12E+02<br>8.82E-01<br>-4.48E-03<br>3.520E-02	1.51E-02<br>-4.32E-01<br>1.30E+00<br>2.57E-01<br>-8.17E-04<br>3.397E-01	5.04E-03<br>-4.64E-01<br>1.66E+00<br>4.50E-01<br>2.00E-04<br>2.824E-01	8.95E-02<br>-9.99E-01<br>1.10E+01<br>3.40E-01<br>5.77E-03<br>7.589E-01	5.25E-02<br>-6.52E-01<br>9.13E+00<br>3.16E-01<br>-1.95E-03<br>5.728E-01	4.62E-02<br>-8.08E-01<br>1.09E+01<br>4.31E-01<br>4.81E-03<br>5.747E-01	1.18E-16<br>-1.51E-13<br>6.18E-15<br>8.37E-01<br>-2.60E-17<br>2.143E-02	7.20E-03<br>-1.38E-01<br>5.23E+00<br>4.41E-01<br>-9.46E-04<br>2.110E-01	8.34E-03<br>-4.48E-01<br>6.24E+01<br>9.68E-01<br>2.38E-02<br>5.635E-02	1.71E-03<br>-6.14E+01<br>4.84E-01<br>9.27E-01<br>1.98E-02<br>3.489E-02	-2.12E-02<br>-4.56E-01<br>3.21E+00<br>4.41E-01<br>9.56E-04<br>3.801E-01	7.05E-02<br>-7.64E-01<br>1.52E+00<br>5.58E-01<br>1.07E-03<br>3.859E-01	1.33E-02<br>-2.37E-01<br>1.04E+00<br>3.17E-01<br>6.70E-04<br>2.051E-01	-1.31E-19<br>-5.70E-11<br>1.33E-11<br>9.98E-01<br>-2.84E-14<br>1.132E-03	6.21E-20<br>-1.64E-14<br>3.02E-15<br>8.33E-01<br>-5.53E-18<br>6.487E-02	2.07E-07<br>-8.92E-06<br>2.26E-04<br>5.40E-01<br>-8.99E-08<br>1.269E-01	5.29E-07<br>-3.44E-05<br>1.09E-04<br>6.36E-01<br>5.34E-08<br>1.784E-01	-3.54E-04<br>-5.27E-02<br>1.59E-01<br>7.74E-01<br>5.91E-05<br>1.523E-01
(glycerol3phosphate oxidase)<br>Local<br>Min<br>Max<br>Normalized peak height<br>Peak sensitivity value<br>Shapiro-Wilk score	4.14E-01<br>-3.08E+01<br>INF<br>5.23E-01<br>4.88E-03<br>6.099E-01	2.95E-03<br>-2.03E+02<br>INF<br>7.56E-01<br>-4.55E-02<br>5.795E-02	2.25E-04<br>-1.44E+00<br>INF<br>8.51E-01<br>-2.23E-03<br>5.267E-02	1.71E-03<br>-6.59E+00<br>INF<br>9.07E-01<br>8.89E-02<br>4.164E-02	3.00E-02<br>-4.67E+00<br>INF<br>7.78E-01<br>-6.21E-04<br>1.508E-01	1.02E-02<br>-3.46E-01<br>INF<br>3.82E-01<br>3.37E-04<br>2.952E-01	1.80E-01<br>-2.25E+01<br>INF<br>2.86E-01<br>-1.29E-02<br>6.848E-01	1.06E-01<br>-6.35E+00<br>INF<br>3.25E-01<br>-1.99E-03<br>5.727E-01	9.60E-02<br>-4.44E-01<br>INF<br>1.53E-01<br>5.91E-04<br>7.454E-01	8.65E-17<br>-1.04E-14<br>INF<br>7.58E-01<br>-1.00E-17<br>1.651E-01	1.45E-02<br>-5.27E-01<br>INF<br>3.44E-01<br>-1.58E-03<br>2.193E-01	1.68E-02<br>-9.52E-01<br>INF<br>9.59E-01<br>3.53E-03<br>4.942E-02	3.45E-03<br>-6.88E+01<br>INF<br>9.19E-01<br>1.63E-02<br>3.043E-02	-4.42E-02<br>-6.45E+00<br>INF<br>3.63E-01<br>-3.10E-03<br>4.728E-01	1.42E-01<br>-4.07E+00<br>INF<br>5.65E-01<br>-6.14E-03<br>3.757E-01	2.67E-02<br>-3.23E+00<br>INF<br>7.25E-01<br>3.44E-04<br>2.060E-01	-2.64E-19<br>-9.25E-11<br>INF<br>9.99E-01<br>-6.11E-14<br>9.112E-04	1.27E-19<br>-2.71E-14<br>INF<br>8.50E-01<br>8.84E-18<br>2.411E-02	4.16E-07<br>-5.55E-05<br>INF<br>6.83E-01<br>-9.40E-08<br>1.078E-01	1.10E-06<br>-2.50E-06<br>INF<br>4.56E-01<br>7.78E-08<br>2.171E-01	-7.39E-04<br>-4.16E-01<br>INF<br>7.57E-01<br>-3.04E-05<br>1.524E-01
(pyruvate transport)<br>Local<br>Min<br>Max<br>Normalized peak height<br>Peak sensitivity value<br>Shapiro-Wilk score	7.06E-01<br>-1.83E+01<br>9.41E+00<br>3.55E-01<br>8.69E-03<br>6.901E-01	5.02E-03<br>-3.89E+02<br>1.57E+01<br>7.16E-01<br>1.66E-01<br>4.587E-02	1.49E-04<br>-1.00E+00<br>5.53E+00<br>9.25E-01<br>1.70E-03<br>4.610E-02	1.08E-03<br>-1.56E+01<br>3.87E+02<br>8.08E-01<br>-1.58E-01<br>3.471E-02	1.51E-02<br>-2.84E+00<br>3.24E+00<br>4.12E-01<br>-1.84E-03<br>3.319E-01	5.04E-03<br>-4.52E-01<br>1.25E+00<br>4.50E-01<br>4.89E-04<br>2.918E-01	8.95E-02<br>-7.60E+00<br>1.51E+01<br>3.46E-01<br>1.72E-03<br>7.181E-01	5.25E-02<br>-3.73E-01<br>3.63E+00<br>3.18E-01<br>1.20E-03<br>5.943E-01	4.62E-02<br>-4.01E-01<br>7.84E+00<br>3.11E-01<br>-1.02E-03<br>5.827E-01	-2.22E-16<br>-1.40E-13<br>5.60E-14<br>4.11E-01<br>-8.48E-17<br>8.340E-02	7.20E-03<br>-2.97E-01<br>6.79E+00<br>3.95E-01<br>3.94E-03<br>2.081E-01	8.34E-03<br>-5.51E-02<br>2.26E+02<br>9.75E-01<br>5.78E-02<br>1.574E-02	1.71E-03<br>-2.25E+02<br>6.18E-01<br>9.80E-01<br>5.43E-02<br>8.748E-03	-2.12E-02<br>-3.28E-01<br>3.34E+00<br>4.61E-01<br>-2.61E-04<br>3.678E-01	7.05E-02<br>-2.14E+00<br>1.51E+00<br>5.69E-01<br>7.31E-04<br>3.868E-01	1.33E-02<br>-1.78E-01<br>1.33E+00<br>3.80E-01<br>-6.68E-04<br>2.072E-01	-1.31E-19<br>-3.00E-09<br>2.58E-10<br>1.00E+00<br>-8.65E-13<br>3.973E-04	6.21E-20<br>-2.52E-14<br>2.79E-13<br>9.59E-01<br>-1.31E-16<br>1.323E-03	2.07E-07<br>-4.61E-06<br>3.13E-04<br>7.43E-01<br>-7.79E-09<br>1.077E-01	5.29E-07<br>-1.94E-05<br>1.80E-04<br>6.41E-01<br>2.52E-08<br>1.384E-01	-3.54E-04<br>-8.38E-02<br>1.24E-01<br>7.61E-01<br>6.50E-05<br>1.664E-01

(phosphoglycerate kinase)	Local	7.06E-01	5.02E-03	1.49E-04	1.08E-03	1.51E-02	5.04E-03	8.95E-02	5.25E-02	4.62E-02	2.34E-17	7.20E-03	8.34E-03	1.71E-03	-2.12E-02	7.05E-02	1.33E-02	-1.31E-19	6.21E-20	2.07E-07	5.29E-07	-3.54E-04
	Min	-INF	-INF	-INF	-INF	-INF	-INF	-INF	-INF	-INF	-INF	-INF	-INF	-INF	-INF	-INF	-INF	-INF	-INF	-INF	-INF	-INF
	Max	2.52E+00	3.95E+00	2.89E+00	1.75E+02	2.76E+00	1.48E+00	5.91E+00	1.98E+00	3.02E+00	3.00E-13	1.39E+01	2.66E+02	5.02E-01	4.96E+00	1.64E+00	1.57E+00	5.30E-09	3.35E-15	1.75E-04	1.29E-04	2.27E-01
	Normalized peak height	3.06E-01	6.01E-01	8.50E-01	8.51E-01	4.58E-01	4.29E-01	2.96E-01	2.73E-01	3.32E-01	8.10E-01	7.27E-01	9.73E-01	9.82E-01	4.91E-01	5.24E-01	6.62E-01	1.00E+00	9.15E-01	5.59E-01	4.01E-01	7.08E-01
	Peak sensitivity value	2.28E-03	8.12E-02	2.57E-04	-1.79E-02	5.08E-04	2.86E-05	2.26E-03	3.84E-04	8.09E-04	-2.49E-16	5.78E-03	-3.70E-02	1.04E-01	-1.58E-03	5.28E-04	9.48E-04	1.98E-12	1.85E-17	-5.94E-08	-4.23E-08	1.39E-04
	Shapiro-Wilk score	7.405E-01	6.060E-02	6.399E-02	4.381E-02	3.387E-01	2.905E-01	7.791E-01	6.006E-01	5.911E-01	1.148E-02	2.007E-01	1.145E-02	6.626E-03	3.571E-01	3.842E-01	2.062E-01	3.442E-04	1.034E-02	1.425E-01	1.493E-01	1.428E-01
(pyruvate kinase)	Local	7.06E-01	5.02E-03	1.49E-04	1.08E-03	1.51E-02	5.04E-03	8.95E-02	5.25E-02	4.62E-02	-2.12E-16	7.20E-03	8.34E-03	1.71E-03	-2.12E-02	7.05E-02	1.33E-02	-1.31E-19	6.21E-20	2.07E-07	5.29E-07	-3.54E-04
	Min	-1.99E+01	-8.35E+02	-2.17E-01	-1.86E+01	-7.19E-01	-2.16E-01	-1.17E+01	-1.02E+00	-9.62E-01	-8.57E-14	-2.37E-01	-8.07E-02	-3.03E+02	-4.86E-01	-1.03E+00	-7.61E-01	-1.09E-08	-2.55E-13	-1.15E-05	-1.29E-05	-7.14E-02
	Max	INF	INF	INF	INF	INF	INF	INF	INF	INF	INF	INF	INF	INF	INF	INF	INF	INF	INF	INF	INF	INF
	Normalized peak height	2.55E-01	8.89E-01	9.55E-01	9.65E-01	4.95E-01	3.94E-01	3.83E-01	1.96E-01	2.99E-01	5.88E-01	4.10E-01	9.76E-01	9.81E-01	5.05E-01	4.25E-01	6.44E-01	1.00E+00	9.08E-01	7.95E-01	7.30E-01	7.79E-01
	Peak sensitivity value	-1.52E-02	2.17E-01	-6.85E-03	1.78E-01	7.23E-04	1.90E-04	6.79E-03	2.81E-03	4.14E-04	6.72E-17	5.06E-03	7.14E-02	1.40E-01	-1.37E-03	-6.48E-04	7.28E-04	-3.43E-12	3.86E-16	5.17E-08	5.35E-08	3.43E-05
	Shapiro-Wilk score	7.163E-01	1.472E-02	1.197E-02	1.145E-02	3.357E-01	2.995E-01	7.479E-01	5.954E-01	5.918E-01	5.835E-02	2.064E-01	8.884E-03	5.126E-03	3.566E-01	3.831E-01	2.002E-01	4.976E-04	1.039E-03	1.057E-01	1.106E-01	1.644E-01
(atp utilisation)	Local	7.06E-01	5.02E-03	1.49E-04	1.08E-03	1.51E-02	5.04E-03	8.95E-02	5.25E-02	4.62E-02	2.20E-21	7.20E-03	8.34E-03	1.71E-03	-2.12E-02	7.05E-02	1.33E-02	-1.31E-19	6.21E-20	2.07E-07	5.29E-07	-3.54E-04
	Min	-2.36E+01	-3.08E+02	-1.30E-01	-4.89E-01	-2.80E-01	-3.09E-01	-5.62E-01	-4.33E-01	-1.03E+00	-8.92E-12	-1.44E-01	-3.44E-02	-4.05E+02	-5.32E-01	-1.77E-01	-9.23E-01	-4.81E-10	-7.56E-13	-7.51E-06	-3.30E-05	-7.30E-02
	Max	2.54E+00	9.37E-01	4.29E+00	3.00E+02	8.19E+00	9.90E-01	1.59E+01	4.04E+00	6.92E+00	3.83E-11	5.24E+00	4.06E+02	1.22E+00	6.47E+00	2.54E+00	1.00E+00	5.45E-07	6.68E-15	1.16E-03	6.98E-05	1.72E-01
	Normalized peak height	4.30E-01	7.10E-01	8.93E-01	8.84E-01	6.23E-01	2.65E-01	3.49E-01	2.54E-01	3.61E-01	9.95E-01	3.45E-01	9.78E-01	9.82E-01	4.68E-01	5.58E-01	6.36E-01	1.00E+00	9.97E-01	8.93E-01	5.49E-01	6.58E-01
	Peak sensitivity value	-7.45E-03	-1.44E-01	7.24E-04	-3.79E-02	3.83E-03	-5.43E-04	5.28E-03	2.26E-03	1.33E-03	-2.01E-14	-1.83E-03	1.69E-01	2.00E-01	-3.22E-03	8.95E-04	5.55E-04	-2.08E-10	2.00E-16	8.62E-08	3.03E-08	-1.22E-04
	Shapiro-Wilk score	7.025E-01	3.948E-02	6.052E-02	2.932E-02	3.203E-01	2.972E-01	7.490E-01	5.974E-01	5.855E-01	1.545E-03	2.096E-01	7.227E-03	4.409E-03	3.423E-01	3.823E-01	2.037E-01	2.776E-04	5.512E-04	3.630E-02	1.949E-01	1.434E-01
(glycerol kinase)	Local	9.22E+00	6.57E-02	-2.07E-03	-1.73E-02	-4.20E-01	-1.47E-01	-2.56E+00	-1.50E+00	-1.41E+00	1.05E-15	-2.06E-01	-2.39E-01	-4.91E-02	6.53E-01	-2.02E+00	-3.80E-01	3.75E-18	-1.82E-18	-5.91E-06	-1.61E-05	1.09E-02
	Min	-6.80E+01	-1.19E+03	-4.14E+01	-6.23E+01	-1.10E+02	-1.55E+01	-1.03E+02	-3.30E+02	-1.40E+02	-7.12E-13	-6.05E+00	-5.41E+02	-6.12E+02	-1.62E+00	-7.91E+01	-4.43E+01	-4.44E-08	-3.14E-13	-1.83E-03	-1.21E-01	-2.32E-02
	Max	INF	INF	INF	INF	INF	INF	INF	INF	INF	INF	INF	INF	INF	INF	INF	INF	INF	INF	INF	INF	INF
	Normalized peak height	4.51E-01	4.69E-01	8.31E-01	8.70E-01	5.85E-01	4.46E-01	2.84E-01	6.15E-01	3.18E-01	9.30E-01	2.85E-01	9.57E-01	9.78E-01	4.14E-01	6.78E-01	7.23E-01	1.00E+00	9.86E-01	6.96E-01	5.36E-01	4.25E-01
	Peak sensitivity value	6.30E-02	2.83E-01	-2.34E-02	-2.72E-01	-2.66E-02	8.05E-03	-2.90E-02	-1.02E-02	-1.02E+00	-2.23E-15	-9.67E-03	4.82E-01	-6.29E-03	9.99E-01	-4.35E-02	-1.13E-02	-2.00E-11	1.11E-16	-6.15E-07	5.59E-05	-5.97E-05
	Shapiro-Wilk score	3.291E-01	5.911E-02	4.346E-02	3.609E-02	1.950E-01	2.315E-01	4.757E-01	1.309E-01	3.767E-01	2.756E-03	5.637E-01	1.097E-02	8.869E-03	5.405E-01	2.307E-01	1.847E-01	3.141E-04	6.070E-03	1.579E-01	3.486E-02	2.936E-01
(phosphoglycerate mutase)	Local	7.06E-01	5.02E-03	1.49E-04	1.08E-03	1.51E-02	5.04E-03	8.95E-02	5.25E-02	4.62E-02	2.10E-17	7.20E-03	8.34E-03	1.71E-03	-2.12E-02	7.05E-02	1.33E-02	-1.31E-19	6.21E-20	2.07E-07	5.29E-07	-3.54E-04
	Min	-1.05E+01	-5.40E+02	-3.89E-01	-1.89E+01	-3.79E-01	-2.93E-01	-1.17E+00	-5.96E-01	-7.50E-01	-1.15E-13	-4.27E-01	-1.34E-01	-1.38E+02	-1.30E+00	-9.57E-02	-9.19E-02	-1.28E-09	-5.77E-12	-3.83E-05	-2.03E-05	-5.09E-02
	Max	INF	INF	INF	INF	INF	INF	INF	INF	INF	INF	INF	INF	INF	INF	INF	INF	INF	INF	INF	INF	INF
	Normalized peak height	3.60E-01	8.16E-01	7.68E-01	7.76E-01	4.36E-01	4.53E-01	2.37E-01	2.45E-01	1.69E-01	6.40E-01	5.15E-01	8.43E-01	8.88E-01	5.90E-01	4.08E-01	4.90E-01	1.00E+00	1.00E+00	7.97E-01	5.77E-01	7.75E-01
	Peak sensitivity value	-2.13E-03	1.84E-01	-4.46E-04	-2.48E-01	9.89E-04	5.42E-04	-4.32E-03	-8.81E-04	-1.45E-03	1.08E-16	-7.37E-04	-6.44E-02	-4.31E-02	6.70E-03	-2.72E-04	-3.09E-04	-4.96E-13	6.25E-16	2.56E-08	3.90E-08	4.59E-05
	Shapiro-Wilk score	7.288E-01	3.082E-02	7.007E-02	2.345E-02	3.382E-01	2.942E-01	7.429E-01	5.932E-01	5.929E-01	4.057E-02	2.072E-01	2.572E-02	1.446E-02	2.999E-01	3.830E-01	2.046E-01	5.463E-04	5.553E-04	6.832E-02	1.942E-01	1.664E-01
(enolase)	Local	7.06E-01	5.02E-03	1.49E-04	1.08E-03	1.51E-02	5.04E-03	8.95E-02	5.25E-02	4.62E-02	2.10E-17	7.20E-03	8.34E-03	1.71E-03	-2.12E-02	7.05E-02	1.33E-02	-1.31E-19	6.21E-20	2.07E-07	5.29E-07	-3.54E-04
	Min	-9.52E+00	-2.40E+02	-1.83E-01	-1.07E+00	-5.31E-01	-2.37E-01	-1.61E+00	-3.51E-01	-3.82E+00	-2.58E-14	-1.34E-01	-9.35E-02	-3.64E+02	-7.41E-01	-3.22E-01	-2.56E-01	-6.99E-10	-3.84E-14	-2.28E-05	-3.42E-05	-6.65E-02
	Max	3.46E+00	1.21E+00	1.41E+01	2.38E+02	2.11E+00	5.09E+00	1.29E+01	2.61E+00	4.82E+00	1.04E-12	3.67E+00	3.65E+02	4.84E-01	5.76E+00	2.19E+00	1.00E+00	4.56E-09	2.03E-15	1.58E-04	1.67E-04	1.43E-01
	Normalized peak height	2.84E-01	6.15E-01	8.89E-01	8.89E-01	4.36E-01	5.87E-01	3.51E-01	2.28E-01	3.47E-01	9.85E-01	5.09E-01	9.77E-01	9.73E-01	4.92E-01	5.58E-01	5.81E-01	1.00E+00	9.11E-01	7.62E-01	4.65E-01	7.89E-01
	Peak sensitivity value	4.10E-03	-1.17E-01	-4.37E-03	2.18E-03	4.00E-04	4.56E-04	6.68E-03	-7.03E-04	2.61E-04	2.11E-16	8.28E-04	8.91E-02	-6.26E-02	-2.52E-03	9.78E-04	1.87E-04	-1.40E-12	-6.83E-18	8.37E-08	-4.95E-08	-6.87E-05
	Shapiro-Wilk score	7.300E-01	6.039E-02	3.348E-02	4.459E-02	3.410E-01	2.349E-01	7.550E-01	5.941E-01	5.881E-01	2.550E-03	2.149E-01	8.271E-03	4.847E-03	3.647E-01	3.835E-01	2.062E-01	6.713E-04	1.824E-02	1.558E-01	1.607E-01	1.491E-01
(adenylate kinase cytosol)	Local	INF	INF	INF	INF	INF	INF	INF	INF	INF	INF	INF	INF	INF	INF	INF	INF	INF	INF	INF	INF	INF
	Min	-INF	-INF	-INF	-INF	-INF	-INF	-INF	-INF	-INF	-INF	-INF	-INF	-INF	-INF	-INF	-INF	-INF	-INF	-INF	-INF	-INF
	Max	INF	INF	INF	INF	INF	INF	INF	INF	INF	INF	INF	INF	INF	INF	INF	INF	INF	INF	INF	INF	INF
	Normalized peak height	6.49E-01	5.51E-01	6.06E-01	5.44E-01	7.62E-01	7.01E-01	4.25E-01	4.43E-01	5.41E-01	9.93E-01	1.77E-01	4.40E-02	2.75E-01	6.87E-01	2.60E-01	2.37E-01	4.62E-01	8.95E-01	2.86E-01	4.96E-01	9.09E-01
	Peak sensitivity value	6.29E-03	-4.88E-02	-5.39E-03	-6.57E-02	-5.92E-04	1.06E-03	-2.04E-02	6.35E-03	-4.95E-03	-2.73E-04	2.09E-02	-6.03E-04	-1.81E-03	1.08E-03	4.88E-02	3.58E-02	-2.10E-11	-4.26E-13	-4.59E-03	1.13E-03	-7.45E-05
	Shapiro-Wilk score	5.001E-01	2.429E-01	1.698E-01	1.748E-01	2.020E-01	1.994E-01	5.747E-01	5.568E-01	3.833E-01	4.659E-03	6.656E-01	8.738E-01	8.774E-01	2.828E-01	3.887E-01	4.898E-01	3.022E-02	4.463E-02	4.113E-01	4.509E-01	6.328E-02
(adenylate kinase glycosome)	Local	-INF	-INF	-INF	-INF	-INF	-INF	-INF	-INF	-INF	-INF	-INF	-INF	-INF	-INF	-INF	-INF	-INF	-INF	-INF	-INF	-INF
	Min	-INF	-INF	-INF	-INF	-INF	-INF	-INF	-INF	-INF	-INF	-INF	-INF	-INF	-INF	-INF	-INF	-INF	-INF	-INF	-INF	-INF
	Max	INF	INF	INF	INF	INF	INF	INF	INF	INF	INF	INF	INF	INF	INF	INF	INF	INF	INF	INF	INF	INF
	Normalized peak height	5.93E-01	5.08E-01	4.74E-01	4.87E-01	8.20E-01	8.86E-01	6.16E-01	5.50E-01	7.31E-01	9.69E-01	4.85E-01	9.21E-01	8.59E-01	6.58E-01	8.30E-01	8.61E-01	9.07E-01	4.72E-01	7.70E-01	7.79E-01	7.78E-01
	Peak sensitivity value	1.53E-01	-5.46E-02	2.73E-02	-4.64E-03	-5.16E-02	5.53E-02	2.99E-02	1.83E-01	-7.25E-02	-1.11E-06	9.62E-02	-3.85E-02	1.03E-01	-6.27E-03	-1.22E-01	-1.29E-01	5.26E-13	8.22E-13	-3.58E-02	-5.52E-02	-2.64E-03
	Shapiro-Wilk score	2.497E-01	3.346E-01	3.300E-01	3.172E-01	6.762E-02	2.393E-02	2.480E-01	1.892E-01	1.442E-01	9.806E-03	3.130E-01	2.128E-02	2.090E-02	3.075E-01	6.990E-02	3.658E-02	2.593E-02	2.887E-01	1.061E-01	7.959E-02	1.066E-01
(3phosphoglycerate transport)	Local	7.06E-01	5.02E-03	1.49E-04	1.08E-03	1.51E-02	5.04E-03	8.95E-02	5.25E-02	4.62E-02	2.34E-17	7.20E-03	8.34E-03	1.71E-03	-2.12E-02	7.05E-02	1.33E-02	-1.31E-19	6.20E-20	2.07E-07	5.29E-07	-3.54E-04
	Min	-1.18E+01	-9.94E+02	-2.55E-01	-8.11E+00	-1.63E+00	-2.99E-01	-1.36E+00	-4.81E-01	-5.99E-01	-5.57E-14	-2.05E-01	-2.22E-02	-2.08E+02	-5.10E-01	-1.70E-01	-1.53E-01	-9.26E-09	-7.45E-14	-2.05E-03	-3.00E-05	-5.24E-02
	Max	3.14E+00	9.30E+00	4.11E+00	9.92E+02	1.73E+00	2.35E+00	1.04E+01	3.45E+00	2.14E+00	5.28E-13	4.70E+00	2.09E+02	5.01E-01	3.08E+00	1.21E+00	1.11E+00	5.94E-09	1.48E-12	2.27E-04	7.37E-05	1.39E-01
	Normalized peak height	3.73E-01	9.54E-01	8.76E-01	9.75E-01	4.43E-01	5.27E-01	2.94E-01	3.05E-01	1.57E-01	9.62E-01	3.75E-01	9.76E-01	9.59E-01	4.25E-01	5.08E-01	5.05E-01	1.00E+00	9.98E-01	9.48E-01	5.53E-01	7.68E-01
	Peak sensitivity value	-4.33E-03	-2.31E-01	1.18E-04	3.92E-01	4.43E-05	1.07E-03	-6.99E-04	5.90E-04	-1.09E-03	5.53E-17	-1.40E-03	8.25E-02	-2.10E-02	1.16E-03	5.82E-04	-2.79E-04	1.44E-12	-5.32E-16	7.84E-07	3.58E-08	-7.38E-05
	Shapiro-Wilk score	7.294E-01	1.364E-02	6.013E-02	1.006E-02	3.398E-01	2.758E-01	7.525E-01	5.924E-01	5.963E-01	6.450E-03	2.088E-01	1.242E-02	7.289E-03	3.629E-01	3.853E-01	2.031E-01	7.223E-04	4.367E-04	1.984E-02	1.881E-01	1.492E-01
(gly3p dhap antiporter)	Local	4.14E-01	2.95E-03	2.25E-04	1.71E-03	3.00E-02	1.02E-02	1.80E-01	1.06E-01	9.60E-02	8.65E-17	1.45E-02	1.68E-02	3.45E-03	-4.42E-02	1.42E-01	2.67E-02	-2.64E-19	1.27E-19	4.16E-07	1.10E-06	-7.39E-04
	Min	-1.61E+01	-2.79E+02	-1.76E+00	-4.22E+00	-4.22E+00	-5.90E-01	-1.96E+01	-1.71E+00	-2.75E-01	-3.89E-14	-1.61E+00	-8.58E-01	-1.38E+02	-8.25E+00	-4.80E+00	-3.58E+00	-8.69E-11	-1.68E-13	-9.11E-05	-8.59E-06	-3.42E-01
	Max	INF	INF	INF	INF	INF	INF	INF	INF	INF	INF	INF	INF	INF	INF	INF	INF	INF	INF	INF	INF	INF
	Normalized peak height	4.62E-01	6.71E-01	8.82E-01	9.19E-01	5.69E-01	3.75E-01	3.92E-01	3.33E-01	1.41E-01	8.22E-01	4.27E-01	9.74E-01	8.89E-01	3.66E-01	7.25E-01	5.71E-01	1.00E+00	9.83E-01	6.71E-01	3.10E-01	7.40E-01
	Peak sensitivity value	-2.11E-03	-1.21E-01	-6.52E-04	3.63E-02	1.52E-03	1.84E-04	1.41E-02	3.99E-04	1.90E-03	1.81E-17	-1.68E-03	5.15E-02	-3.74E-02	-1.61E-03	8.38E-03	-4.28E-03	-1.92E-15	-7.24E-18	-1.72E-07	1.24E-07	-3.05E-05
	Shapiro-Wilk score	6.664E-01	3.796E-02	5.823E-02	2.788E-02	3.306E-01	3.060E-01	7.021E-01	5.888E-01	7.471E-01	6.747E-02	2.248E-01	2.385E-02	1.379E-02	4.707E-01	3.751E-01	2.044E-01	5.255E-04	2.723E-03	9.643E-02	2.300E-01	1.426E-01
(glycerol transport)	Local	9.22E+00	6.57E-02	-2.07E-03	-1.73E-02	-4.20E-01	-1.47E-01	-2.56E+00	-1.50E+00	-1.41E+00	1.05E-15	-2.06E-01	-2.39E-01	-4.91E-02	6.53E-01	-2.02E+00	-3.80E-01	3.75E-18	-1.82E-18	-5.91E-06	-1.61E-05	1.09E-02
	Min	-1.32E+02	-6.75E+02	-3.61E+01	-1.68E+01	-2.84E+01	-2.07E+01	-5.88E+01	-5.90E+01	-1.78E+02	-1.13E-12	-7.34E+00	-6.97E+01	-1.37E+03	-1.25E+00	-7.39E+01	-7.23E+01	-1.65E-07	-3.38E-13	-5.22E-03	-8.39E-02	-3.42E-03
	Max	2.34E+02	2.11E+01	2.95E+01	6.70E+02	1.95E+01	5.33E+00	1.28E+02	2.67E+01	9.01E+00	1.29E-12	2.22E+01	1.37E+03	4.44E+01	1.08E+01	3.35E+01	8.91E+00	2.17E-10	3.46E-13	2.42E-03	2.69E-05	9.83E-01
	Normalized peak height	4.96E-01	5.40E-01	9.43E-01	8.66E-01	4.32E-01	4.25E-01	2.99E-01	3.64E-01	2.88E-01	9.63E-01	4.74E-01	9.77E-01	9.91E-01	2.07E-01	6.09E-01	7.63E-01	1.00E+00	9.29E-01	8.26E-01	8.85E-01	4.46E-01
	Peak sensitivity value	-1.59E-01	-1.70E-01	-5.38E-03	5.56E-03	-6.59E-03	9.34E-03	-4.86E-02	8.70E-03	-1.01E+00	5.48E-16	1.45E-02	3.65E-02	-3.17E-02	1.00E+00	4.66E-02	1.32E-02	-3.13E-11	-2.08E-16	1.46E-06	-1.51E-05	2.92E-05
	Shapiro-Wilk score	4.807E-01	7.720E-02	3.781E-02	4.795E-02	3.173E-01	1.958E-01	4.668E-01	3.928E-01	3.407E-01	8.762E-03	5.530E-01	3.956E-03	2.443E-03	7.464E-01	2.582E-01	1.163E-01	2.801E-04	8.419E-03	9.346E-02	5.032E-02	2.865E-01
