# Supplementary material for: What Can We Learn from Global Sensitivity Analysis of Biochemical Systems?
Source: PLoS One. 2013 Nov 14;8(11):e79244. doi: 10.1371/journal.pone.0079244 (PMC3828278; doi:10.1371/journal.pone.0079244)
Supplement: File S3 — Tables S18—S31. The full outputs of the global sensitivity analyses on the Saccharomyces cerevisiae model using the random-sampling approach. (PDF) [file pone.0079244.s003.pdf]

Supplementary information (Tables S18 – S31)

Below we present the full outputs of the global sensitivity analyses on the *Saccharomyces cerevisiae* model using the random-sampling approach. The results of random sampling are presented as distributions for each sensitivity – distribution x axes show the sensitivity coefficient values, and the y axes show the frequency these values occurred during sampling. The initial sensitivity value is shown as a dashed blue line, zero is shown as a dotted purple line.

Table S18: Concentration control coefficients for *Saccharomyces cerevisiae* model with parameter variation of ±5%

		(GlucoseMixedflowtoextracellularmedium)	(Glucoseuptake)	(Hexokinase)	(Phosphoglucoisomerase)	(Phosphofructokinase)	(Aldolase)	(Triosephosphateisomerase)	(Glyceraldehyde3-phosphatedehydrogenase)	(Phosphoenolpyruvatesynthesis)	(Pyruvatekinase)	(Pyruvatedecarboxylase)	(Alcoholdehydrogenase)	(Ethanolout)	(Ethanolflow)	(Glycerolsynthesis)	(Glycerolout)	(Glycerolflow)	(Acetaldehydeout)	(Acetaldehydeflow)	(Cyanide-Acetaldehydeflow)	(Cyanideflow)	(Storage)	(ATPconsumption)	(Adenylatekinase)
Acetaldehyde	Local	2.55E-02	1.10E-02	1.99E+00	8.76E-03	1.06E-01	-5.75E-15	-8.30E-02	-1.82E-01	-1.56E-02	-4.70E-02	-7.51E-15	-1.66E+00	0.00E+00	0.00E+00	2.83E-01	-5.12E-15	7.96E-15	-6.23E-02	-3.24E-01	-9.20E-02	-7.34E-03	-7.38E-02	1.26E-01	1.05E-17
	Min	1.06E-02	5.16E-03	1.83E+00	7.11E-03	8.16E-02	-2.00E-14	-1.05E-01	-2.24E-01	-1.88E-02	-6.12E-02	-2.84E-14	-1.84E+00	0.00E+00	0.00E+00	2.30E-01	-7.01E-14	-4.92E-14	-7.47E-02	-3.72E-01	-1.07E-01	-1.14E-02	-8.38E-02	1.11E-01	-5.52E-17
	Max	7.17E-02	2.57E-02	2.16E+00	1.08E-02	1.42E-01	1.96E-14	-6.31E-02	-1.47E-01	-1.28E-02	-3.47E-02	3.31E-14	-1.50E+00	0.00E+00	0.00E+00	3.39E-01	7.43E-14	4.85E-14	-5.08E-02	-2.75E-01	-7.69E-02	-4.68E-03	-6.49E-02	1.46E-01	6.62E-16
	Normalized peak height	4.06E-03	4.35E-03	2.95E-03	3.38E-03	3.23E-03	5.47E-03	3.17E-03	3.65E-03	3.45E-03	3.63E-03	6.71E-02	2.90E-03	1.00E+00	1.00E+00	3.32E-03	5.97E-03	7.81E-03	3.32E-03	4.00E-03	3.30E-03	3.72E-03	3.80E-03	3.32E-03	3.50E-01
	Peak sensitivity value	1.88E-02	9.54E-03	1.97E+00	8.55E-03	1.01E-01	7.91E-17	-8.45E-02	-1.83E-01	-1.56E-02	-4.70E-02	-2.91E-17	-1.64E+00	5.00E-04	5.00E-04	2.83E-01	1.80E-17	6.82E-17	-6.31E-02	-3.25E-01	-9.00E-02	-7.07E-03	-7.31E-02	1.27E-01	-3.49E-19
	Shapiro-Wilk score	9.143E-01	9.559E-01	9.940E-01	9.939E-01	9.781E-01	9.970E-01	9.985E-01	9.997E-01	9.992E-01	9.983E-01	9.986E-01	9.945E-01	1.000E+00	1.000E+00	9.996E-01	9.985E-01	9.980E-01	9.985E-01	9.990E-01	9.989E-01	9.902E-01	9.993E-01	9.988E-01	7.952E-01
Cytosolic glucose	Local	2.04E+00	8.77E-01	-3.34E+00	7.30E-03	8.83E-02	4.39E-16	-2.81E-03	-6.15E-03	-5.28E-04	-1.59E-03	1.00E-15	-5.63E-02	0.00E+00	0.00E+00	9.58E-03	7.63E-16	4.37E-17	2.30E-03	1.20E-02	3.40E-03	2.71E-04	3.43E-01	2.74E-02	7.34E-18
	Min	1.13E+00	6.83E-01	-5.51E+00	4.26E-03	4.70E-02	-2.14E-15	-4.62E-03	-1.04E-02	-8.80E-04	-2.82E-03	-3.30E-15	-9.62E-02	0.00E+00	0.00E+00	5.94E-03	-8.89E-15	-6.25E-15	1.42E-03	7.85E-03	2.20E-03	1.26E-04	2.47E-01	1.93E-02	-4.04E-17
	Max	3.90E+00	1.15E+00	-2.23E+00	1.25E-02	1.71E-01	2.21E-15	-1.71E-03	-3.79E-03	-3.21E-04	-8.93E-04	2.62E-15	-3.36E-02	0.00E+00	0.00E+00	1.63E-02	8.78E-15	6.74E-15	3.72E-03	1.90E-02	5.18E-03	5.01E-04	5.08E-01	4.05E-02	1.65E-15
	Normalized peak height	3.47E-03	3.32E-03	3.65E-03	3.87E-03	4.23E-03	6.53E-02	3.70E-03	4.10E-03	3.73E-03	3.88E-03	2.32E-02	3.50E-03	1.00E+00	1.00E+00	4.05E-03	4.70E-03	4.78E-03	3.75E-03	3.88E-03	3.77E-03	3.83E-03	3.65E-03	4.07E-03	3.50E-01
	Peak sensitivity value	1.86E+00	8.74E-01	-3.16E+00	6.56E-03	8.04E-02	-5.61E-19	-2.62E-03	-5.70E-03	-4.99E-04	-1.45E-03	-4.55E-19	-5.22E-02	5.00E-04	5.00E-04	8.96E-03	2.55E-18	-2.03E-16	2.19E-03	1.10E-02	3.21E-03	2.46E-04	3.23E-01	2.69E-02	-6.04E-19
	Shapiro-Wilk score	9.700E-01	9.943E-01	9.746E-01	9.832E-01	9.725E-01	9.998E-01	9.779E-01	9.794E-01	9.784E-01	9.784E-01	9.997E-01	9.701E-01	1.000E+00	1.000E+00	9.788E-01	9.994E-01	9.994E-01	9.823E-01	9.802E-01	9.822E-01	9.705E-01	9.787E-01	9.884E-01	5.752E-01
Glycerol	Local	2.51E-02	1.08E-02	1.96E+00	8.63E-03	1.04E-01	8.13E-15	-8.18E-02	-1.79E-01	-1.53E-02	-4.63E-02	1.53E-14	-1.64E+00	0.00E+00	0.00E+00	2.79E-01	-5.98E-01	-4.02E-01	6.70E-02	3.48E-01	9.89E-02	7.89E-03	-7.27E-02	1.24E-01	1.03E-17
	Min	1.11E-02	5.11E-03	1.81E+00	6.97E-03	8.02E-02	-2.87E-14	-1.05E-01	-2.16E-01	-1.89E-02	-6.10E-02	-5.04E-14	-1.80E+00	0.00E+00	0.00E+00	2.24E-01	-6.33E-01	-4.37E-01	5.42E-02	3.04E-01	7.96E-02	5.84E-03	-8.26E-02	1.09E-01	-5.25E-17
	Max	6.53E-02	2.29E-02	2.10E+00	1.07E-02	1.43E-01	3.57E-14	-6.23E-02	-1.44E-01	-1.26E-02	-3.39E-02	4.55E-14	-1.47E+00	0.00E+00	0.00E+00	3.37E-01	-5.63E-01	-3.67E-01	8.21E-02	3.91E-01	1.18E-01	1.03E-02	-6.34E-02	1.43E-01	9.36E-16
	Normalized peak height	3.92E-03	4.02E-03	2.77E-03	3.47E-03	3.34E-03	5.41E-03	3.37E-03	3.44E-03	3.79E-03	3.69E-03	3.06E-02	3.00E-03	1.00E+00	1.00E+00	3.50E-03	2.72E-03	2.72E-03	3.60E-03	3.42E-03	3.52E-03	3.32E-03	3.85E-03	3.12E-03	3.49E-01
	Peak sensitivity value	1.96E-02	9.63E-03	1.94E+00	8.53E-03	1.01E-01	-6.90E-17	-8.20E-02	-1.81E-01	-1.52E-02	-4.66E-02	-4.57E-17	-1.61E+00	5.00E-04	5.00E-04	2.77E-01	-5.99E-01	-4.01E-01	6.63E-02	3.52E-01	1.01E-01	7.63E-03	-7.17E-02	1.23E-01	3.54E-19
	Shapiro-Wilk score	9.132E-01	9.552E-01	9.941E-01	9.941E-01	9.780E-01	9.992E-01	9.984E-01	9.997E-01	9.991E-01	9.983E-01	9.989E-01	9.948E-01	1.000E+00	1.000E+00	9.997E-01	9.975E-01	9.975E-01	9.992E-01	9.995E-01	9.999E-01	9.966E-01	9.996E-01	9.986E-01	7.056E-01
EtOH	Local	1.01E-02	4.37E-03	7.92E-01	2.32E-03	2.81E-02	-3.86E-16	9.18E-03	2.01E-02	1.72E-03	5.20E-03	-7.43E-17	1.84E-01	-1.45E-01	-8.55E-01	-3.13E-02	-1.63E-16	1.29E-15	-7.52E-03	-3.91E-02	-1.11E-02	-8.85E-04	-1.89E-02	5.21E-02	2.13E-18
	Min	4.62E-03	2.24E-03	7.33E-01	1.93E-03	2.22E-02	-1.41E-15	6.99E-03	1.53E-02	1.27E-03	3.37E-03	-2.40E-15	1.39E-01	-1.64E-01	-8.72E-01	-4.04E-02	-5.49E-15	-4.38E-15	-9.49E-03	-4.62E-02	-1.31E-02	-1.34E-03	-2.33E-02	4.35E-02	-1.26E-17
	Max	2.72E-02	9.63E-03	8.32E-01	2.89E-03	3.73E-02	1.53E-15	1.18E-02	2.58E-02	2.28E-03	7.84E-03	2.47E-15	2.41E-01	-1.28E-01	-8.36E-01	-2.37E-02	5.57E-15	4.10E-15	-5.93E-03	-3.27E-02	-9.38E-03	-5.66E-04	-1.52E-02	6.11E-02	2.03E-16
	Normalized peak height	4.15E-03	4.32E-03	3.12E-03	3.49E-03	2.82E-03	1.71E-02	3.15E-03	3.49E-03	3.39E-03	4.10E-03	5.37E-02	2.94E-03	2.54E-03	2.54E-03	3.62E-03	4.74E-03	9.46E-03	3.25E-03	2.67E-03	3.24E-03	3.24E-03	3.49E-03	2.99E-03	3.47E-01
	Peak sensitivity value	8.79E-03	3.97E-03	7.97E-01	2.34E-03	2.69E-02	-2.02E-17	9.17E-03	2.02E-02	1.70E-03	4.91E-03	3.52E-19	1.73E-01	-1.43E-01	-8.57E-01	-3.08E-02	2.93E-16	7.31E-18	-7.54E-03	-3.89E-02	-1.10E-02	-8.28E-04	-1.91E-02	5.33E-02	-1.62E-22
	Shapiro-Wilk score	9.219E-01	9.629E-01	9.840E-01	9.979E-01	9.879E-01	9.951E-01	9.971E-01	9.981E-01	9.966E-01	9.960E-01	9.993E-01	9.876E-01	9.970E-01	9.970E-01	9.978E-01	9.985E-01	9.981E-01	9.973E-01	9.956E-01	9.972E-01	9.900E-01	9.995E-01	9.964E-01	7.986E-01
ADP	Local	1.29E-03	5.55E-04	1.01E-01	-2.09E-02	-2.53E-01	6.63E-16	-3.90E-03	-8.54E-03	-7.33E-04	-2.21E-03	1.57E-15	-7.81E-02	0.00E+00	0.00E+00	1.33E-02	1.09E-15	5.21E-17	3.20E-03	1.66E-02	4.72E-03	3.76E-04	1.88E-01	3.81E-02	-4.97E-17
	Min	3.30E-04	1.72E-04	4.89E-02	-2.51E-02	-3.08E-01	-2.43E-15	-5.30E-03	-1.15E-02	-9.95E-04	-3.29E-03	-4.49E-15	-1.06E-01	0.00E+00	0.00E+00	9.49E-03	-1.17E-14	-7.76E-15	2.30E-03	1.32E-02	3.85E-03	2.27E-04	1.70E-01	3.21E-02	-4.13E-15
	Max	5.07E-03	1.91E-03	1.72E-01	-1.77E-02	-2.12E-01	3.04E-15	-2.83E-03	-6.14E-03	-5.04E-04	-1.44E-03	4.90E-15	-5.54E-02	0.00E+00	0.00E+00	1.79E-02	1.17E-14	8.57E-15	4.24E-03	2.05E-02	5.78E-03	6.08E-04	2.06E-01	4.55E-02	1.46E-15
	Normalized peak height	5.52E-03	5.25E-03	2.89E-03	3.93E-03	3.26E-03	4.04E-02	3.48E-03	3.93E-03	3.68E-03	3.69E-03	1.58E-02	3.19E-03	1.00E+00	1.00E+00	3.79E-03	4.20E-03	4.41E-03	3.93E-03	3.39E-03	3.46E-03	3.59E-03	3.48E-03	3.28E-03	3.48E-01
	Peak sensitivity value	8.26E-04	3.94E-04	8.53E-02	-2.06E-02	-2.40E-01	-6.28E-19	-3.74E-03	-8.16E-03	-7.16E-04	-2.06E-03	4.47E-19	-7.31E-02	5.00E-04	5.00E-04	1.29E-02	-1.17E-16	6.05E-18	3.13E-03	1.59E-02	4.61E-03	3.72E-04	1.87E-01	3.73E-02	1.81E-18
	Shapiro-Wilk score	8.814E-01	9.232E-01	9.806E-01	9.983E-01	9.842E-01	9.997E-01	9.952E-01	9.962E-01	9.950E-01	9.938E-01	9.998E-01	9.873E-01	1.000E+00	1.000E+00	9.959E-01	9.999E-01	9.999E-01	9.973E-01	9.966E-01	9.984E-01	9.887E-01	9.997E-01	9.986E-01	7.617E-01
Glyceraldehyde 3-phosphate	Local	2.26E-02	9.73E-03	1.76E+00	1.27E-02	1.54E-01	6.19E-15	1.56E-01	-5.55E-01	-4.76E-02	-1.44E-01	1.37E-14	-1.23E+00	0.00E+00	0.00E+00	-5.32E-01	7.05E-15	-4.91E-15	5.03E-02	2.61E-01	7.42E-02	5.92E-03	-1.10E-01	1.05E-01	1.80E-17
	Min	9.95E-03	4.86E-03	1.58E+00	9.82E-03	1.15E-01	-2.49E-14	1.25E-01	-6.51E-01	-5.63E-02	-1.85E-01	-3.32E-14	-1.53E+00	0.00E+00	0.00E+00	-6.53E-01	-9.17E-14	-7.94E-14	3.80E-02	2.09E-01	5.89E-02	4.01E-03	-1.27E-01	8.83E-02	-1.18E-16
	Max	6.44E-02	2.23E-02	1.99E+00	1.60E-02	2.11E-01	2.50E-14	1.92E-01	-4.60E-01	-4.06E-02	-1.09E-01	3.61E-14	-9.60E-01	0.00E+00	0.00E+00	-4.28E-01	9.89E-14	5.83E-14	6.42E-02	3.13E-01	9.29E-02	8.49E-03	-9.49E-02	1.22E-01	8.51E-16
	Normalized peak height	4.29E-03	3.99E-03	2.75E-03	3.70E-03	3.80E-03	4.60E-03	3.62E-03	3.69E-03	3.47E-03	3.72E-03	5.42E-02	3.17E-03	1.00E+00	1.00E+00	3.45E-03	6.56E-03	1.74E-02	3.52E-03	3.47E-03	3.52E-03	3.47E-03	3.62E-03	3.39E-03	3.47E-01
	Peak sensitivity value	1.78E-02	8.97E-03	1.72E+00	1.26E-02	1.49E-01	-1.28E-15	1.55E-01	-5.57E-01	-4.75E-02	-1.43E-01	-1.44E-17	-1.22E+00	5.00E-04	5.00E-04	-5.27E-01	5.88E-17	-1.23E-17	4.90E-02	2.59E-01	7.51E-02	5.80E-03	-1.10E-01	1.04E-01	1.91E-19
	Shapiro-Wilk score	9.139E-01	9.550E-01	9.920E-01	9.956E-01	9.808E-01	9.992E-01	9.988E-01	9.995E-01	9.991E-01	9.983E-01	9.993E-01	9.955E-01	1.000E+00	1.000E+00	9.985E-01	9.998E-01	9.991E-01	9.986E-01	9.995E-01	9.991E-01	9.942E-01	9.995E-01	9.979E-01	8.135E-01
Dihydroxyacetone phosphate	Local	2.40E-02	1.03E-02	1.88E+00	1.23E-02	1.49E-01	5.18E-15	-2.24E-01	-4.91E-01	-4.21E-02	-1.27E-01	1.04E-14	-1.03E+00	0.00E+00	0.00E+00	-4.89E-01	6.32E-15	-3.90E-15	4.23E-02	2.20E-01	6.25E-02	4.99E-03	-1.06E-01	1.13E-01	1.69E-17
	Min	1.02E-02	4.97E-03	1.70E+00	9.55E-03	1.14E-01	-2.01E-14	-2.72E-01	-5.74E-01	-4.93E-02	-1.66E-01	-2.88E-14	-1.29E+00	0.00E+00	0.00E+00	-5.90E-01	-7.55E-14	-5.69E-14	3.13E-02	1.76E-01	4.90E-02	3.30E-03	-1.24E-01	9.78E-02	-9.58E-17
	Max	6.56E-02	2.29E-02	2.08E+00	1.55E-02	2.06E-01	2.04E-14	-1.79E-01	-3.98E-01	-3.54E-02	-9.41E-02	3.03E-14	-7.93E-01	0.00E+00	0.00E+00	-3.89E-01	7.60E-14	6.00E-14	5.49E-02	2.73E-01	7.89E-02	7.38E-03	-9.19E-02	1.31E-01	9.86E-16
	Normalized peak height	4.28E-03	4.03E-03	2.98E-03	3.71E-03	3.43E-03	6.26E-03	2.90E-03	3.61E-03	3.46E-03	3.70E-03	3.49E-02	2.96E-03	1.00E+00	1.00E+00	3.43E-03	8.71E-03	1.82E-02	3.46E-03	3.58E-03	3.45E-03	3.60E-03	4.31E-03	3.33E-03	3.48E-01
	Peak sensitivity value	1.97E-02	9.26E-03	1.86E+00	1.22E-02	1.44E-01	5.36E-17	-2.27E-01	-4.88E-01	-4.22E-02	-1.27E-01	-1.87E-17	-1.02E+00	5.00E-04	5.00E-04	-4.94E-01	2.49E-17	5.70E-17	4.17E-02	2.19E-01	6.32E-02	4.75E-03	-1.05E-01	1.12E-01	-4.57E-20
	Shapiro-Wilk score	9.139E-01	9.550E-01	9.919E-01	9.950E-01	9.807E-01	9.996E-01	9.976E-01	9.997E-01	9.988E-01	9.981E-01	9.994E-01	9.955E-01	1.000E+00	1.000E+00	9.979E-01	9.996E-01	9.994E-01	9.987E-01	9.995E-01	9.990E-01	9.938E-01	9.994E-01	9.986E-01	7.646E-01
Glucose-6-Phosphate	Local	1.37E-02	5.88E-03	1.07E+00	-2.72E-02	-3.29E-01	-8.33E-16	5.10E-03	1.11E-02	9.57E-04	2.89E-03	-1.82E-15	1.02E-01	0.00E+00	0.00E+00	-1.74E-02	-1.37E-15	-6.86E-17	-4.17E-03	-2.17E-02	-6.16E-03	-4.92E-04	-7.52E-01	-4.97E-02	-2.72E-17
	Min	5.97E-03	2.96E-03	1.02E+00	-3.24E-02	-4.37E-01	-3.58E-15	3.73E-03	8.00E-03	6.62E-04	1.73E-03	-5.09E-15	7.39E-02	0.00E+00	0.00E+00	-2.31E-02	-1.41E-14	-1.05E-14	-5.61E-03	-2.71E-02	-7.68E-03	-7.28E-04	-7.75E-01	-6.56E-02	-1.33E-15
	Max	3.70E-02	1.28E-02	1.14E+00	-2.29E-02	-2.63E-01	3.68E-15	6.73E-03	1.46E-02	1.35E-03	4.50E-03	5.01E-15	1.37E-01	0.00E+00	0.00E+00	-1.22E-02	1.50E-14	1.06E-14	-2.96E-03	-1.64E-02	-4.65E-03	-3.16E-04	-7.24E-01	-3.44E-02	2.69E-16
	Normalized peak height	4.03E-03	4.08E-03	2.73E-03	3.43E-03	3.12E-03	4.64E-02	3.32E-03	3.82E-03	3.77E-03	4.00E-03	1.64E-02	3.08E-03	1.00E+00	1.00E+00	3.88E-03	4.05E-03	4.48E-03	3.75E-03	3.23E-03	3.35E-03	3.15E-03	3.52E-03	3.58E-03	3.51E-01
	Peak sensitivity value	1.22E-02	5.54E-03	1.06E+00	-2.66E-02	-3.12E-01	-3.26E-18	4.99E-03	1.11E-02	9.59E-04	2.76E-03	-3.91E-18	9.65E-02	5.00E-04	5.00E-04	-1.72E-02	-1.31E-16	4.75E-17	-4.21E-03	-2.13E-02	-6.14E-03	-4.75E-04	-7.51E-01	-5.18E-02	3.79E-19
	Shapiro-Wilk score	9.168E-01	9.580E-01	9.810E-01	9.955E-01	9.811E-01	9.997E-01	9.977E-01	9.984E-01	9.975E-01	9.957E-01	9.998E-01	9.930E-01	1.000E+00	1.000E+00	9.983E-01	9.998E-01	9.999E-01	9.985E-01	9.988E-01	9.994E-01	9.926E-01	9.979E-01	9.987E-01	8.278E-01
NAD	Local	-4.87E-03	-2.10E-03	-3.80E-01	9.66E-04	1.17E-02	-3.24E-15	-8.00E-02	-1.75E-01	-1.50E-02	-4.53E-02	-6.78E-15	6.68E-01	0.00E+00	0.00E+00	2.72E-01	-3.23E-15	2.80E-15	-2.73E-02	-1.42E-01	-4.03E-02	-3.22E-03	-9.68E-03	-2.81E-02	2.62E-18
	Min	-1.50E-02	-5.14E-03	-5.24E-01	2.38E-04	2.87E-03	-1.19E-14	-1.04E-01	-2.35E-01	-2.33E-02	-7.25E-02	-1.72E-14	4.92E-01	0.00E+00	0.00E+00	2.03E-01	-5.15E-14	-3.28E-14	-3.68E-02	-1.83E-01	-5.27E-02	-4.87E-03	-1.96E-02	-3.78E-02	-1.64E-17
	Max	-1.84E-03	-8.97E-04	-2.74E-01	2.12E-03	2.48E-02	1.20E-14	-5.92E-02	-1.27E-01	-9.10E-03	-2.57E-02	1.62E-14	9.02E-01	0.00E+00	0.00E+00	3.59E-01	4.39E-14	3.74E-14	-1.94E-02	-1.08E-01	-3.05E-02	-2.00E-03	-2.89E-03	-1.98E-02	9.40E-17
	Normalized peak height	4.66E-03	4.02E-03	2.89E-03	4.12E-03	4.02E-03	4.50E-03	3.67E-03	3.50E-03	3.54E-03	3.84E-03	7.38E-02	3.42E-03	1.00E+00	1.00E+00	3.77E-03	4.82E-03	1.36E-02	3.59E-03	3.59E-03	3.65E-03	3.37E-03	4.17E-03	3.04E-03	3.46E-01
	Peak sensitivity value	-3.55E-03	-1.68E-03	-3.59E-01	9.20E-04	1.11E-02	2.46E-16	-7.77E-02	-1.76E-01	-1.45E-02	-4.37E-02	1.41E-17	6.47E-01	5.00E-04	5.00E-04	2.72E-01	6.66E-17	2.87E-17	-2.74E-02	-1.37E-01	-4.04E-02	-2.95E-03	-9.31E-03	-2.71E-02	2.68E-20
	Shapiro-Wilk score	9.031E-01	9.444E-01	9.892E-01	9.951E-01	9.941E-01	9.993E-01	9.977E-01	9.968E-01	9.945E-01	9.947E-01	9.988E-01	9.942E-01	1.000E+00	1.000E+00	9.974E-01	9.994E-01	9.983E-01	9.978E-01	9.985E-01	9.984E-01	9.926E-01	9.957E-01	9.930E-01	8.710E-01
Extracellular acetaldehyde	Local	2.55E-02	1.10E-02	1.99E+00	8.78E-03	1.06E-01	-5.73E-15	-8.32E-02	-1.82E-01	-1.56E-02	-4.71E-02	-8.63E-15	-1.67E+00	0.00E+00	0.00E+00	2.84E-01	-5.10E-15	7.94E-15	6.82E-02	-4.25E-01	-1.21E-01	-9.62E-03	-7.39E-02	1.27E-01	1.05E-17
	Min	1.12E-02	5.36E-03	1.83E+00	7.09E-03	8.15E-02	-2.04E-14	-1.07E-01	-2.22E-01	-1.89E-02	-6.29E-02	-3.30E-14	-1.85E+00	0.00E+00	0.00E+00	2.28E-01	-6.43E-14	-4.47E-14	5.47E-02	-4.79E-01	-1.38E-01	-1.48E-02	-8.34E-02	1.10E-01	-2.81E-16
	Max	7.33E-02	2.57E-02	2.17E+00	1.08E-02	1.43E-01	2.06E-14	-6.42E-02	-1.43E-01	-1.29E-02	-3.45E-02	2.86E-14	-1.50E+00	0.00E+00	0.00E+00	3.41E-01	8.27E-14	4.92E-14	8.37E-02	-3.74E-01	-1.06E-01	-6.38E-03	-6.57E-02	1.45E-01	4.51E-16
	Normalized peak height	4.43E-03	4.36E-03	3.04E-03	3.43E-03	3.14E-03	6.08E-03	3.17E-03	3.61E-03	3.69E-03	3.89E-03	6.82E-02	3.06E-03	1.00E+00	1.00E+00	3.41E-03	5.76E-03	1.02E-02	3.56E-03	3.64E-03	3.44E-03	3.78E-03	3.56E-03	3.26E-03	3.49E-01
	Peak sensitivity value	1.94E-02	8.87E-03	1.98E+00	8.57E-03	1.01E-01	5.54E-17	-8.16E-02	-1.85E-01	-1.56E-02	-4.67E-02	-1.09E-17	-1.65E+00	5.00E-04	5.00E-04	2.86E-01	-1.54E-17	4.27E-17	6.87E-02	-4.21E-01	-1.21E-01	-9.41E-03	-7.40E-02	1.27E-01	-5.17E-20
	Shapiro-Wilk score	9.138E-01	9.563E-01	9.932E-01	9.935E-01	9.791E-01	9.962E-01	9.984E-01	9.997E-01	9.990E-01	9.985E-01	9.986E-01	9.942E-01	1.000E+00	1.000E+00	9.996E-01	9.988E-01	9.991E-01	9.992E-01	9.988E-01	9.987E-01	9.906E-01	9.995E-01	9.986E-01	8.147E-01

Phosphoenolpyruvate	Local	1.38E-02	5.93E-03	1.08E+00	6.08E-03	7.36E-02	2.62E-15	1.11E-03	2.42E-03	2.08E-04	-1.20E+00	7.14E-14	2.22E-02	0.00E+00	0.00E+00	-3.77E-03	4.71E-15	-1.00E-15	-9.07E-04	-4.72E-03	-1.34E-03	-1.07E-04	-5.20E-02	6.64E-02	9.58E-18
	Min	6.10E-03	2.92E-03	1.03E+00	5.19E-03	6.02E-02	-1.87E-14	7.93E-04	1.67E-03	1.46E-04	-1.23E+00	-2.26E-13	1.62E-02	0.00E+00	0.00E+00	-5.23E-03	-1.86E-14	-8.81E-15	-1.21E-03	-6.08E-03	-1.69E-03	-1.77E-04	-5.60E-02	5.75E-02	-1.91E-16
	Max	3.58E-02	1.24E-02	1.10E+00	7.13E-03	9.37E-02	1.70E-14	1.52E-03	3.29E-03	2.86E-04	-1.18E+00	1.90E-13	3.18E-02	0.00E+00	0.00E+00	-2.72E-03	1.50E-14	9.18E-15	-6.75E-04	-3.73E-03	-1.05E-03	-6.66E-05	-4.80E-02	7.57E-02	9.07E-16
	Normalized peak height	3.95E-03	4.00E-03	3.78E-03	3.47E-03	3.10E-03	9.92E-03	3.70E-03	4.12E-03	3.55E-03	2.67E-03	1.79E-01	3.07E-03	1.00E+00	1.00E+00	3.83E-03	4.69E-03	5.04E-03	3.47E-03	3.48E-03	3.52E-03	3.50E-03	3.38E-03	2.93E-03	3.48E-01
	Peak sensitivity value	1.04E-02	5.51E-03	1.07E+00	6.03E-03	7.11E-02	5.04E-17	1.07E-03	2.40E-03	1.99E-04	-1.20E+00	2.27E-18	2.18E-02	5.00E-04	5.00E-04	-3.69E-03	6.22E-17	-3.28E-18	-9.00E-04	-4.62E-03	-1.31E-03	-1.01E-04	-5.21E-02	6.76E-02	-3.92E-19
	Shapiro-Wilk score	9.198E-01	9.619E-01	9.978E-01	9.971E-01	9.809E-01	9.989E-01	9.941E-01	9.949E-01	9.941E-01	9.901E-01	9.589E-01	9.854E-01	1.000E+00	1.000E+00	9.946E-01	9.995E-01	9.996E-01	9.962E-01	9.948E-01	9.969E-01	9.870E-01	9.994E-01	9.980E-01	7.566E-01
Fructose-6-Phosphate	Local	1.24E-02	5.33E-03	9.67E-01	8.44E-02	-3.38E-01	-8.95E-16	5.90E-03	1.29E-02	1.11E-03	3.34E-03	-2.13E-15	1.18E-01	0.00E+00	0.00E+00	-2.01E-02	-1.59E-15	-7.98E-17	-4.83E-03	-2.51E-02	-7.13E-03	-5.69E-04	-7.58E-01	-5.75E-02	-2.80E-17
	Min	5.43E-03	2.60E-03	9.15E-01	7.28E-02	-4.53E-01	-4.21E-15	4.38E-03	9.53E-03	7.90E-04	2.11E-03	-6.15E-15	8.67E-02	0.00E+00	0.00E+00	-2.69E-02	-1.67E-14	-1.17E-14	-6.47E-03	-3.10E-02	-8.82E-03	-8.47E-04	-7.83E-01	-7.38E-02	-1.27E-15
	Max	3.27E-02	1.18E-02	1.04E+00	9.67E-02	-2.62E-01	4.15E-15	7.77E-03	1.67E-02	1.55E-03	4.85E-03	5.83E-15	1.58E-01	0.00E+00	0.00E+00	-1.47E-02	1.60E-14	1.37E-14	-3.58E-03	-1.97E-02	-5.46E-03	-3.63E-04	-7.29E-01	-4.23E-02	5.32E-16
	Normalized peak height	4.11E-03	4.37E-03	2.69E-03	3.34E-03	3.03E-03	4.64E-02	3.53E-03	3.61E-03	3.54E-03	3.58E-03	1.83E-02	3.08E-03	1.00E+00	1.00E+00	3.63E-03	3.84E-03	4.69E-03	3.53E-03	3.46E-03	3.48E-03	3.39E-03	3.49E-03	3.48E-03	3.53E-01
	Peak sensitivity value	9.75E-03	4.60E-03	9.59E-01	8.34E-02	-3.29E-01	-1.02E-18	5.80E-03	1.30E-02	1.08E-03	3.23E-03	-2.60E-18	1.16E-01	5.00E-04	5.00E-04	-1.97E-02	7.00E-16	-9.13E-18	-4.82E-03	-2.50E-02	-7.05E-03	-5.26E-04	-7.58E-01	-5.80E-02	4.06E-19
	Shapiro-Wilk score	9.178E-01	9.587E-01	9.849E-01	9.969E-01	9.817E-01	9.998E-01	9.974E-01	9.980E-01	9.971E-01	9.957E-01	9.999E-01	9.925E-01	1.000E+00	1.000E+00	9.978E-01	9.999E-01	9.999E-01	9.979E-01	9.983E-01	9.990E-01	9.929E-01	9.979E-01	9.991E-01	7.892E-01
Extracellular cyanide	Local	-1.89E-03	-8.11E-04	-1.47E-01	-6.48E-04	-7.85E-03	4.27E-16	6.15E-03	1.34E-02	1.15E-03	3.48E-03	6.32E-16	1.23E-01	0.00E+00	0.00E+00	-2.09E-02	3.66E-16	-5.96E-16	-5.03E-03	3.14E-02	-6.50E-02	7.46E-02	5.46E-03	-9.35E-03	-7.74E-19
	Min	-6.20E-03	-2.06E-03	-2.01E-01	-9.88E-04	-1.30E-02	-1.55E-15	4.24E-03	9.53E-03	7.63E-04	2.23E-03	-1.94E-15	8.75E-02	0.00E+00	0.00E+00	-2.82E-02	-5.11E-15	-3.84E-15	-6.63E-03	2.21E-02	-8.44E-02	5.65E-02	3.89E-03	-1.27E-02	-3.94E-17
	Max	-6.58E-04	-3.34E-04	-1.05E-01	-4.16E-04	-4.82E-03	1.40E-15	8.60E-03	1.80E-02	1.61E-03	5.35E-03	2.22E-15	1.68E-01	0.00E+00	0.00E+00	-1.47E-02	5.68E-15	3.50E-15	-3.80E-03	4.54E-02	-4.99E-02	9.84E-02	7.46E-03	-6.40E-03	8.58E-18
	Normalized peak height	5.05E-03	4.22E-03	3.30E-03	3.32E-03	3.53E-03	6.80E-03	3.68E-03	3.40E-03	3.60E-03	3.98E-03	6.67E-02	2.88E-03	1.00E+00	1.00E+00	3.68E-03	5.42E-03	1.02E-02	3.48E-03	3.38E-03	3.10E-03	3.15E-03	3.23E-03	3.43E-03	3.52E-01
	Peak sensitivity value	-1.43E-03	-6.82E-04	-1.38E-01	-5.87E-04	-7.21E-03	3.57E-18	5.89E-03	1.29E-02	1.10E-03	3.27E-03	4.37E-19	1.16E-01	5.00E-04	5.00E-04	-2.07E-02	6.15E-18	2.44E-18	-4.94E-03	2.90E-02	-6.26E-02	7.22E-02	5.20E-03	-8.75E-03	1.81E-20
	Shapiro-Wilk score	9.037E-01	9.444E-01	9.902E-01	9.848E-01	9.739E-01	9.967E-01	9.942E-01	9.957E-01	9.949E-01	9.931E-01	9.986E-01	9.887E-01	1.000E+00	1.000E+00	9.951E-01	9.987E-01	9.985E-01	9.972E-01	9.896E-01	9.935E-01	9.932E-01	9.932E-01	9.945E-01	8.101E-01
Pyruvate	Local	9.01E-01	3.88E-01	7.04E+01	2.28E-01	2.76E+00	-6.96E-14	4.07E-02	8.91E-02	7.64E-03	2.31E-02	-7.79E+01	8.15E-01	0.00E+00	0.00E+00	-1.39E-01	-5.18E-14	1.49E-13	-3.34E-02	-1.73E-01	-4.93E-02	-3.93E-03	-1.87E+00	4.59E+00	2.26E-16
	Min	5.57E-02	2.88E-02	8.58E+00	2.52E-02	2.95E-01	-3.55E-11	3.82E-03	8.29E-03	7.38E-04	2.11E-03	-4.19E+04	7.76E-02	0.00E+00	0.00E+00	-7.27E+01	-3.72E-11	-3.58E-11	-1.71E+01	-9.10E+01	-2.66E+01	-2.08E+00	-9.90E+02	5.55E-01	-5.94E-14
	Max	7.40E+02	2.51E+02	3.80E+04	1.20E+02	1.53E+03	8.99E-12	2.15E+01	4.68E+01	4.04E+00	1.25E+01	-9.47E+00	4.33E+02	0.00E+00	0.00E+00	-1.35E-02	3.57E-11	6.60E-11	-3.35E-03	-1.77E-02	-5.18E-03	-3.34E-04	-2.31E-01	2.38E+03	2.79E-13
	Normalized peak height	7.95E-01	7.48E-01	7.30E-01	7.16E-01	7.25E-01	6.89E-01	7.08E-01	7.06E-01	7.09E-01	7.15E-01	7.28E-01	7.09E-01	1.00E+00	1.00E+00	7.07E-01	4.47E-01	7.26E-01	7.02E-01	7.09E-01	7.19E-01	7.02E-01	7.24E-01	7.17E-01	7.53E-01
	Peak sensitivity value	4.26E-01	1.54E-01	2.76E+01	8.55E-02	1.06E+00	-1.07E-14	1.46E-02	3.17E-02	2.76E-03	8.37E-03	-3.04E+01	2.94E-01	5.00E-04	5.00E-04	-4.98E-02	2.05E-14	-5.86E-15	-1.19E-02	-6.32E-02	-1.85E-02	-1.38E-03	-7.26E-01	1.74E+00	-6.67E-17
	Shapiro-Wilk score	5.297E-02	5.479E-02	5.875E-02	5.881E-02	5.624E-02	6.071E-02	5.920E-02	5.980E-02	5.949E-02	5.962E-02	5.871E-02	6.024E-02	1.000E+00	1.000E+00	5.972E-02	1.447E-01	8.033E-02	5.930E-02	5.984E-02	5.791E-02	5.996E-02	5.875E-02	6.083E-02	5.052E-02
Extracellular ethanol	Local	1.01E-02	4.37E-03	7.92E-01	2.32E-03	2.81E-02	-3.81E-16	9.18E-03	2.01E-02	1.72E-03	5.20E-03	-1.74E-16	1.84E-01	-3.41E-16	-1.00E+00	-3.13E-02	-1.73E-16	1.29E-15	-7.52E-03	-3.91E-02	-1.11E-02	-8.85E-04	-1.89E-02	5.21E-02	2.13E-18
	Min	4.64E-03	2.17E-03	7.36E-01	1.91E-03	2.18E-02	-1.31E-15	6.90E-03	1.50E-02	1.23E-03	3.53E-03	-2.62E-15	1.39E-01	-5.35E-16	-1.00E+00	-4.13E-02	-5.61E-15	-4.51E-15	-9.40E-03	-4.69E-02	-1.30E-02	-1.30E-03	-2.31E-02	4.26E-02	-5.89E-17
	Max	2.74E-02	9.35E-03	8.33E-01	2.81E-03	3.76E-02	1.56E-15	1.19E-02	2.62E-02	2.30E-03	7.42E-03	2.55E-15	2.42E-01	5.36E-16	-1.00E+00	-2.36E-02	5.42E-15	3.48E-15	-5.85E-03	-3.24E-02	-9.38E-03	-5.57E-04	-1.48E-02	6.12E-02	1.65E-16
	Normalized peak height	4.01E-03	4.05E-03	3.05E-03	3.31E-03	3.00E-03	1.83E-02	3.28E-03	3.91E-03	3.56E-03	3.93E-03	6.03E-02	2.73E-03	6.49E-01	1.00E+00	3.80E-03	4.75E-03	8.81E-03	3.21E-03	3.20E-03	2.88E-03	3.13E-03	3.45E-03	3.18E-03	3.49E-01
	Peak sensitivity value	7.67E-03	4.14E-03	7.99E-01	2.31E-03	2.77E-02	-7.25E-19	9.16E-03	1.96E-02	1.69E-03	5.06E-03	6.32E-19	1.81E-01	9.87E-20	-1.00E+00	-3.14E-02	-8.38E-18	7.89E-18	-7.43E-03	-3.88E-02	-1.09E-02	-8.63E-04	-1.93E-02	5.36E-02	-4.31E-21
	Shapiro-Wilk score	9.212E-01	9.635E-01	9.841E-01	9.981E-01	9.884E-01	9.954E-01	9.966E-01	9.977E-01	9.964E-01	9.955E-01	9.992E-01	9.873E-01	8.089E-01	1.000E+00	9.974E-01	9.989E-01	9.985E-01	9.973E-01	9.955E-01	9.969E-01	9.905E-01	9.991E-01	9.962E-01	7.843E-01
Extracellular glycerol	Local	2.51E-02	1.08E-02	1.96E+00	8.63E-03	1.04E-01	8.14E-15	-8.18E-02	-1.79E-01	-1.53E-02	-4.63E-02	1.49E-14	-1.64E+00	0.00E+00	0.00E+00	2.79E-01	9.72E-15	-1.00E+00	6.70E-02	3.48E-01	9.89E-02	7.89E-03	-7.27E-02	1.24E-01	1.03E-17
	Min	1.12E-02	5.54E-03	1.81E+00	6.97E-03	8.08E-02	-3.26E-14	-1.04E-01	-2.19E-01	-1.87E-02	-6.00E-02	-4.89E-14	-1.81E+00	0.00E+00	0.00E+00	2.23E-01	-1.20E-13	-1.00E+00	5.30E-02	2.99E-01	8.11E-02	5.83E-03	-8.27E-02	1.08E-01	-5.41E-17
	Max	6.92E-02	2.37E-02	2.12E+00	1.08E-02	1.42E-01	3.09E-14	-6.16E-02	-1.41E-01	-1.25E-02	-3.34E-02	5.30E-14	-1.48E+00	0.00E+00	0.00E+00	3.32E-01	1.27E-13	-1.00E+00	8.18E-02	3.93E-01	1.18E-01	1.03E-02	-6.41E-02	1.42E-01	7.93E-16
	Normalized peak height	4.06E-03	3.91E-03	2.93E-03	3.61E-03	3.35E-03	4.69E-03	3.28E-03	3.73E-03	3.65E-03	3.71E-03	3.69E-02	2.96E-03	1.00E+00	1.00E+00	3.55E-03	7.39E-03	1.00E+00	3.70E-03	3.68E-03	3.55E-03	3.28E-03	3.76E-03	3.53E-03	3.48E-01
	Peak sensitivity value	2.01E-02	9.15E-03	1.94E+00	8.51E-03	9.95E-02	-2.31E-19	-8.09E-02	-1.78E-01	-1.54E-02	-4.57E-02	2.64E-17	-1.61E+00	5.00E-04	5.00E-04	2.81E-01	1.24E-16	-1.00E+00	6.67E-02	3.48E-01	9.97E-02	7.74E-03	-7.25E-02	1.24E-01	-2.81E-19
	Shapiro-Wilk score	9.161E-01	9.576E-01	9.940E-01	9.939E-01	9.790E-01	9.993E-01	9.983E-01	9.996E-01	9.992E-01	9.984E-01	9.988E-01	9.946E-01	1.000E+00	1.000E+00	9.996E-01	9.997E-01	1.000E+00	9.993E-01	9.997E-01	9.998E-01	9.970E-01	9.995E-01	9.987E-01	7.630E-01
Extracellular glucose	Local	2.57E+00	-1.40E-02	-2.53E+00	-3.55E-03	-4.30E-02	8.97E-17	-5.98E-04	-1.31E-03	-1.12E-04	-3.38E-04	2.12E-16	-1.20E-02	0.00E+00	0.00E+00	2.04E-03	1.65E-16	1.51E-17	4.89E-04	2.54E-03	7.23E-04	5.76E-05	2.55E-02	5.83E-03	-3.53E-18
	Min	1.87E+00	-4.19E-02	-3.78E+00	-6.60E-03	-8.80E-02	-4.48E-16	-1.23E-03	-2.77E-03	-2.24E-04	-7.06E-04	-6.28E-16	-2.58E-02	0.00E+00	0.00E+00	1.10E-03	-2.35E-15	-1.75E-15	2.56E-04	1.42E-03	4.08E-04	2.49E-05	1.69E-02	3.61E-03	-1.74E-16
	Max	3.85E+00	-5.64E-03	-1.84E+00	-2.09E-03	-2.38E-02	5.27E-16	-3.07E-04	-6.47E-04	-5.89E-05	-1.64E-04	6.93E-16	-6.04E-03	0.00E+00	0.00E+00	4.22E-03	2.71E-15	1.94E-15	1.01E-03	5.10E-03	1.38E-03	1.40E-04	4.09E-02	1.02E-02	2.01E-17
	Normalized peak height	2.85E-03	4.57E-03	2.80E-03	3.65E-03	3.68E-03	6.01E-02	3.87E-03	4.03E-03	3.60E-03	3.92E-03	2.17E-02	3.85E-03	1.00E+00	1.00E+00	4.07E-03	6.23E-03	6.25E-03	3.92E-03	4.00E-03	3.83E-03	4.62E-03	3.35E-03	3.45E-03	3.47E-01
	Peak sensitivity value	2.37E+00	-1.04E-02	-2.43E+00	-3.19E-03	-3.73E-02	-1.23E-19	-5.21E-04	-1.15E-03	-9.83E-05	-3.11E-04	3.00E-19	-1.03E-02	5.00E-04	5.00E-04	1.78E-03	-5.78E-18	5.02E-18	4.49E-04	2.32E-03	6.06E-04	4.89E-05	2.32E-02	5.35E-03	1.23E-20
	Shapiro-Wilk score	9.682E-01	9.203E-01	9.692E-01	9.550E-01	9.460E-01	9.990E-01	9.573E-01	9.582E-01	9.569E-01	9.561E-01	9.984E-01	9.483E-01	1.000E+00	1.000E+00	9.578E-01	9.970E-01	9.976E-01	9.606E-01	9.585E-01	9.608E-01	9.473E-01	9.789E-01	9.675E-01	8.363E-01
1-3-Bisphosphoglycerate	Local	1.07E-02	4.61E-03	8.37E-01	4.44E-02	5.38E-01	5.30E-16	8.28E-03	1.81E-02	-2.84E-01	-8.56E-01	3.50E-14	1.66E-01	0.00E+00	0.00E+00	-2.82E-02	6.06E-16	-4.86E-16	-6.78E-03	-3.52E-02	-1.00E-02	-7.98E-04	-3.98E-01	-7.28E-03	7.56E-17
	Min	5.03E-03	2.45E-03	6.69E-01	3.80E-02	4.55E-01	-9.24E-15	5.79E-03	1.24E-02	-3.26E-01	-9.16E-01	-8.88E-14	1.20E-01	0.00E+00	0.00E+00	-3.83E-02	-2.92E-14	-1.84E-14	-9.06E-03	-4.43E-02	-1.24E-02	-1.29E-03	-4.13E-01	-1.87E-02	-1.55E-15
	Max	2.70E-02	9.34E-03	9.51E-01	5.06E-02	6.66E-01	1.02E-14	1.11E-02	2.44E-02	-2.45E-01	-7.93E-01	8.99E-14	2.30E-01	0.00E+00	0.00E+00	-1.93E-02	2.40E-14	1.87E-14	-5.04E-03	-2.75E-02	-8.05E-03	-4.87E-04	-3.84E-01	8.74E-04	1.61E-14
	Normalized peak height	3.96E-03	3.88E-03	3.13E-03	3.36E-03	2.96E-03	3.54E-02	3.61E-03	3.88E-03	3.19E-03	2.91E-03	8.51E-03	3.11E-03	1.00E+00	1.00E+00	3.93E-03	3.98E-03	4.16E-03	3.76E-03	3.41E-03	3.55E-03	3.58E-03	3.46E-03	3.39E-03	3.48E-01
	Peak sensitivity value	8.69E-03	4.37E-03	8.53E-01	4.36E-02	5.30E-01	9.59E-18	8.05E-03	1.76E-02	-2.84E-01	-8.51E-01	8.54E-17	1.66E-01	5.00E-04	5.00E-04	-2.77E-02	1.44E-15	1.25E-15	-6.73E-03	-3.37E-02	-9.76E-03	-7.89E-04	-3.98E-01	-5.81E-03	8.35E-18
	Shapiro-Wilk score	9.318E-01	9.726E-01	9.789E-01	9.972E-01	9.790E-01	9.998E-01	9.937E-01	9.948E-01	9.980E-01	9.971E-01	9.973E-01	9.853E-01	1.000E+00	1.000E+00	9.946E-01	9.998E-01	9.999E-01	9.959E-01	9.950E-01	9.965E-01	9.866E-01	9.994E-01	9.899E-01	5.969E-01
Fructose 1-6-bisphosphate	Local	4.60E-02	1.98E-02	3.59E+00	2.43E-02	2.94E-01	-9.76E-02	-8.09E-02	-1.00E+00	-8.59E-02	-2.59E-01	2.20E-14	-2.18E+00	0.00E+00	0.00E+00	-9.76E-01	1.28E-14	-8.40E-15	8.90E-02	4.63E-01	1.31E-01	1.05E-02	-2.10E-01	2.16E-01	3.37E-17
	Min	1.95E-02	9.16E-03	3.23E+00	1.94E-02	2.22E-01	-1.23E-01	-1.50E-01	-1.17E+00	-1.03E-01	-3.33E-01	-6.72E-14	-2.71E+00	0.00E+00	0.00E+00	-1.20E+00	-1.85E-13	-1.11E-13	6.39E-02	3.65E-01	1.02E-01	7.11E-03	-2.41E-01	1.87E-01	-1.70E-16
	Max	1.23E-01	4.57E-02	3.99E+00	3.04E-02	3.93E-01	-7.89E-02	-1.25E-02	-8.32E-01	-7.25E-02	-1.97E-01	6.37E-14	-1.69E+00	0.00E+00	0.00E+00	-7.68E-01	1.64E-13	1.22E-13	1.14E-01	5.59E-01	1.64E-01	1.50E-02	-1.84E-01	2.50E-01	1.78E-15
	Normalized peak height	4.45E-03	4.42E-03	2.97E-03	3.58E-03	3.20E-03	3.58E-03	3.33E-03	3.45E-03	4.00E-03	3.57E-03	3.64E-02	3.02E-03	1.00E+00	1.00E+00	3.33E-03	8.82E-03	2.17E-02	3.72E-03	3.55E-03	3.65E-03	3.27E-03	3.50E-03	3.03E-03	3.49E-01
	Peak sensitivity value	3.70E-02	1.71E-02	3.52E+00	2.36E-02	2.84E-01	-9.81E-02	-7.92E-02	-9.99E-01	-8.64E-02	-2.55E-01	3.74E-17	-2.13E+00	5.00E-04	5.00E-04	-9.66E-01	-9.46E-17	4.58E-17	8.95E-02	4.58E-01	1.32E-01	1.01E-02	-2.09E-01	2.11E-01	5.90E-19
	Shapiro-Wilk score	9.134E-01	9.551E-01	9.918E-01	9.955E-01	9.811E-01	9.984E-01	9.992E-01	9.997E-01	9.992E-01	9.984E-01	9.994E-01	9.948E-01	1.000E+00	1.000E+00	9.979E-01	9.996E-01	9.995E-01	9.987E-01	9.994E-01	9.990E-01	9.937E-01	9.994E-01	9.980E-01	7.967E-01
AMP	Local	4.17E-03	1.79E-03	3.25E-01	-6.74E-02	-8.16E-01	2.40E-15	-1.26E-02	-2.76E-02	-2.37E-03	-7.14E-03	6.43E-15	-2.52E-01	0.00E+00	0.00E+00	4.30E-02	3.70E-15	2.03E-16	1.03E-02	5.37E-02	1.52E-02	1.22E-03	6.08E-01	1.23E-01	6.38E-17
	Min	1.07E-03	5.53E-04	1.60E-01	-7.81E-02	-1.00E+00	-9.40E-15	-1.73E-02	-3.82E-02	-3.33E-03	-1.02E-02	-1.41E-14	-3.49E-01	0.00E+00	0.00E+00	3.14E-02	-3.80E-14	-2.61E-14	7.76E-03	4.32E-02	1.24E-02	7.59E-04	5.94E-01	1.08E-01	-3.15E-16
	Max	1.76E-02	5.93E-03	5.86E-01	-5.90E-02	-6.97E-01	8.97E-15	-9.07E-03	-2.00E-02	-1.71E-03	-4.55E-03	1.47E-14	-1.87E-01	0.00E+00	0.00E+00	5.89E-02	3.72E-14	2.38E-14	1.34E-02	6.63E-02	1.85E-02	1.90E-03	6.22E-01	1.41E-01	2.75E-15
	Normalized peak height	5.59E-03	4.65E-03	3.07E-03	3.82E-03	2.74E-03	5.57E-02	3.50E-03	4.15E-03	3.65E-03	3.75E-03	1.78E-02	2.95E-03	1.00E+00	1.00E+00	3.84E-03	4.42E-03	3.95E-03	3.29E-03	3.37E-03	3.29E-03	3.42E-03	3.34E-03	3.12E-03	3.52E-01
	Peak sensitivity value	2.35E-03	1.37E-03	2.77E-01	-6.59E-02	-7.86E-01	-2.82E-18	-1.24E-02	-2.67E-02	-2.23E-03	-6.79E-03	1.19E-17	-2.36E-01	5.00E-04	5.00E-04	4.13E-02	-3.53E-16	-3.72E-16	1.01E-02	5.38E-02	1.51E-02	1.11E-03	6.09E-01	1.22E-01	-4.04E-19
	Shapiro-Wilk score	8.817E-01	9.219E-01	9.791E-01	9.969E-01	9.781E-01	9.996E-01	9.939E-01	9.949E-01	9.940E-01	9.938E-01	9.999E-01	9.854E-01	1.000E+00	1.000E+00	9.945E-01	9.997E-01	1.000E+00	9.964E-01	9.947E-01	9.965E-01	9.874E-01	9.989E-01	9.971E-01	8.264E-01
ATP	Local	-1.59E-03	-6.83E-04	-1.24E-01	2.57E-02	3.11E-01	-8.56E-16	4.80E-03	1.05E-02	9.01E-04	2.72E-03	-2.17E-15	9.61E-02	0.00E+00	0.00E+00	-1.64E-02	-1.37E-15	-6.97E-17	-3.93E-03	-2.04E-02	-5.81E-03	-4.63E-04	-2.32E-01	-4.68E-02	2.55E-17
	Min	-6.79E-03	-2.34E-03	-2.33E-01	2.17E-02	2.44E-01	-3.28E-15	3.32E-03	7.29E-03	6.19E-04	1.77E-03	-5.48E-15	6.77E-02	0.00E+00	0.00E+00	-2.31E-02	-1.70E-14	-1.25E-14	-5.49E-03	-2.75E-02	-7.73E-03	-7.94E-04	-2.60E-01	-5.54E-02	-1.37E-16
	Max	-3.84E-04	-1.91E-04	-5.98E-02	3.04E-02	4.15E-01	3.89E-15	6.94E-03	1.53E-02	1.30E-03	4.10E-03	6.15E-15	1.40E-01	0.00E+00	0.00E+00	-1.14E-02	1.58E-14	9.24E-15	-2.85E-03	-1.57E-02	-4.45E-03	-2.75E-04	-2.10E-01	-4.08E-02	1.37E-15
	Normalized peak height	5.69E-03	4.66E-03	3.30E-03	3.00E-03	3.00E-03	5.64E-02	3.56E-03	3.89E-03	3.70E-03	3.63E-03	1.77E-02	3.05E-03	1.00E+00	1.00E+00	3.58E-03	4.78E-03	4.73E-03	3.43E-03	3.33E-03	3.60E-03	3.65E-03	3.36E-03	3.05E-03	3.48E-01
	Peak sensitivity value	-9.63E-04	-4.35E-04	-1.12E-01	2.54E-02	3.05E-01	-1.81E-18	4.56E-03	1.04E-02	8.75E-04	2.55E-03	5.41E-18	8.90E-02	5.00E-04	5.00E-04	-1.59E-02	6.03E-16	-4.87E-17	-3.77E-03	-2.00E-02	-5.62E-03	-4.32E-04	-2.29E-01	-4.71E-02	-7.50E-19
	Shapiro-Wilk score	8.777E-01	9.174E-01	9.754E-01	9.949E-01	9.814E-01	9.996E-01	9.905E-01	9.915E-01	9.907E-01	9.907E-01	9.997E-01	9.831E-01	1.000E+00	1.000E+00	9.910E-01	9.996E-01	9.998E-01	9.935E-01	9.918E-01	9.939E-01	9.839E-01	9.977E-01	9.956E-01	8.000E-01
NADH	Local	9.35E-03	4.03E-03	7.30E-01	-1.86E-03	-2.25E-02	6.21E-15	1.54E-01	3.36E-01	2.88E-02	8.69E-02	1.30E-14	-1.28E+00	0.00E+00	0.00E+00	-5.23E-01	6.20E-15	-5.37E-15	5.25E-02	2.73E-01	7.75E-02	6.18E-03	1.86E-02	5.39E-02	-5.02E-18
	Min	3.87E-03	1.88E-03	5.98E-01	-3.30E-03	-3.93E-02	-2.48E-14	1.29E-01	2.96E-01	2.33E-02	6.56E-02	-3.63E-14	-1.39E+00	0.00E+00	0.00E+00	-5.61E-01	-8.39E-14	-7.59E-14	4.14E-02	2.32E-01	6.18E-02	4.64E-03	7.73E-03	4.41E-02	-4.06E-16
	Max	2.71E-02	8.91E-03	8.65E-01	-6.22E-04	-7.68E-03	2.45E-14	1.84E-01	3.75E-01	3.52E-02	1.14E-01	4.02E-14	-1.18E+00	0.00E+00	0.00E+00	-4.82E-01	9.34E-14	5.94E-14	6.50E-02	3.12E-01	9.47E-02	7.82E-03	3.23E-02	6.33E-02	7.69E-17
	Normalized peak height	4.31E-03	4.01E-03	3.59E-03	3.86E-03	3.74E-03	4.44E-03	3.34E-03	3.89E-03	3.62E-03	3.67E-03	7.56E-02	3.44E-03	1.00E+00	1.00E+00	3.72E-03	4.59E-03	1.35E-02	3.57E-03	3.22E-03	3.52E-03	3.36E-03	3.74E-03	3.54E-03	3.49E-01
	Peak sensitivity value	7.67E-03	3.58E-03	7.13E-01	-1.77E-03	-2.16E-02	-6.90E-16	1.54E-01	3.35E-01	2.88E-02	8.58E-02	9.61E-19	-1.27E+00	5.00E-04	5.00E-04	-5.25E-01	7.59E-16	1.82E-16	5.21E-02	2.75E-01	7.79E-02	6.02E-03	1.91E-02	5.36E-02	-1.27E-19
	Shapiro-Wilk score	9.113E-01	9.530E-01	9.982E-01	9.996E-01	9.996E-01	9.986E-01	9.987E-01	9.999E-01	9.994E-01	9.987E-01	9.972E-01	9.991E-01	1.000E+00	1.000E+00	9.992E-01	9.997E-01	9.979E-01	9.994E-01	9.972E-01	9.992E-01	9.972E-01	9.996E-01	9.991E-01	7.649E-01

Table S19: Concentration control coefficients for *Saccharomyces cerevisiae* model with parameter variation of ±10%

Metabolite	Statistic	(GlucoseMixedflowtoextracellularmedium)	(Glucoseuptake)	(Hexokinase)	(Phosphoglucoisomerase)	(Phosphofructokinase)	(Aldolase)	(Triosephosphateisomerase)	(Glyceraldehyde3-phosphatedehydrogenase)	(Phosphoenolpyruvatesynthesis)	(Pyruvatekinase)	(Pyruvatedecarboxylase)	(Alcoholdehydrogenase)
Acetaldehyde	Local	2.55E-02	1.10E-02	1.99E+00	8.76E-03	1.06E-01	-5.75E-15	-8.30E-02	-1.82E-01	-1.56E-02	-4.70E-02	-7.51E-15	-1.66E+00
	Min	5.46E-03	2.89E-03	1.65E+00	5.79E-03	6.51E-02	-2.56E-14	-1.29E-01	-2.56E-01	-2.22E-02	-7.99E-02	-4.12E-14	-2.01E+00
	Max	2.73E-01	6.72E-02	2.32E+00	1.33E-02	2.12E-01	2.75E-14	-4.62E-02	-1.11E-01	-1.05E-02	-2.51E-02	4.80E-14	-1.34E+00
	Normalized peak height	1.26E-02	8.30E-03	3.01E-03	3.95E-03	4.07E-03	8.02E-03	3.17E-03	3.39E-03	3.53E-03	3.86E-03	6.35E-02	3.01E-03
	Peak sensitivity value	1.36E-02	7.62E-03	1.90E+00	8.08E-03	9.47E-02	4.18E-16	-8.14E-02	-1.81E-01	-1.50E-02	-4.47E-02	-1.35E-17	-1.62E+00
	Shapiro-Wilk score	7.011E-01	8.398E-01	9.895E-01	9.841E-01	9.384E-01	9.823E-01	9.963E-01	9.990E-01	9.970E-01	9.935E-01	9.908E-01	9.913E-01
Cytosolic glucose	Local	2.04E+00	8.77E-01	-3.34E+00	7.30E-03	8.83E-02	4.39E-16	-2.81E-03	-6.15E-03	-5.28E-04	-1.59E-03	1.00E-15	-5.63E-02
	Min	6.41E-01	5.43E-01	-1.04E+01	4.90E-04	5.95E-03	-2.76E-15	-9.15E-03	-1.93E-02	-1.70E-03	-5.24E-03	-7.05E-15	-2.05E-01
	Max	8.70E+00	1.90E+00	-1.50E+00	2.19E-02	3.38E-01	3.14E-15	-9.86E-04	-2.36E-03	-1.80E-04	-4.96E-04	5.91E-15	-2.11E-02
	Normalized peak height	5.41E-03	4.62E-03	5.10E-03	5.66E-03	6.36E-03	7.19E-02	5.91E-03	5.57E-03	5.57E-03	5.08E-03	2.42E-02	5.55E-03
	Peak sensitivity value	1.47E+00	8.16E-01	-2.78E+00	6.31E-03	6.39E-02	6.91E-19	-2.35E-03	-5.46E-03	-4.58E-04	-1.17E-03	-6.08E-18	-4.54E-02
	Shapiro-Wilk score	8.955E-01	9.703E-01	9.169E-01	9.535E-01	9.151E-01	9.907E-01	9.335E-01	9.353E-01	9.343E-01	9.315E-01	9.821E-01	9.147E-01
Glycerol	Local	2.51E-02	1.08E-02	1.96E+00	8.63E-03	1.04E-01	8.13E-15	-8.18E-02	-1.79E-01	-1.53E-02	-4.63E-02	1.53E-14	-1.64E+00
	Min	4.94E-03	2.82E-03	1.60E+00	5.65E-03	6.46E-02	-4.61E-14	-1.27E-01	-2.57E-01	-2.16E-02	-7.83E-02	-7.50E-14	-2.00E+00
	Max	2.99E-01	7.71E-02	2.28E+00	1.30E-02	2.05E-01	4.19E-14	-4.61E-02	-1.08E-01	-9.68E-03	-2.44E-02	5.62E-14	-1.33E+00
	Normalized peak height	1.38E-02	9.23E-03	3.29E-03	3.74E-03	3.90E-03	7.08E-03	3.07E-03	3.50E-03	3.72E-03	3.70E-03	3.61E-02	3.00E-03
	Peak sensitivity value	1.30E-02	7.31E-03	1.89E+00	8.19E-03	9.98E-02	-1.10E-17	-8.04E-02	-1.84E-01	-1.46E-02	-4.37E-02	-2.25E-18	-1.60E+00
	Shapiro-Wilk score	6.947E-01	8.376E-01	9.902E-01	9.862E-01	9.385E-01	9.905E-01	9.966E-01	9.995E-01	9.968E-01	9.934E-01	9.921E-01	9.916E-01
EtOH	Local	1.01E-02	4.37E-03	7.92E-01	2.32E-03	2.81E-02	-3.86E-16	9.18E-03	2.01E-02	1.72E-03	5.20E-03	-7.43E-17	1.84E-01
	Min	2.40E-03	1.23E-03	6.27E-01	1.56E-03	1.74E-02	-1.76E-15	4.65E-03	1.01E-02	9.09E-04	2.20E-03	-3.52E-15	1.05E-01
	Max	1.06E-01	2.56E-02	8.64E-01	3.50E-03	5.34E-02	2.14E-15	1.51E-02	3.33E-02	3.09E-03	1.03E-02	3.40E-15	3.07E-01
	Normalized peak height	1.25E-02	8.10E-03	3.60E-03	3.62E-03	3.91E-03	2.01E-02	3.71E-03	4.21E-03	3.73E-03	3.92E-03	6.42E-02	2.69E-03
	Peak sensitivity value	4.84E-03	3.49E-03	8.09E-01	2.27E-03	2.61E-02	2.40E-19	8.59E-03	1.90E-02	1.60E-03	4.69E-03	-6.48E-19	1.70E-01
	Shapiro-Wilk score	7.160E-01	8.611E-01	9.561E-01	9.928E-01	9.605E-01	9.825E-01	9.910E-01	9.923E-01	9.888E-01	9.847E-01	9.978E-01	9.745E-01
ADP	Local	1.29E-03	5.55E-04	1.01E-01	-2.09E-02	-2.53E-01	6.63E-16	-3.90E-03	-8.54E-03	-7.33E-04	-2.21E-03	1.57E-15	-7.81E-02
	Min	5.87E-05	3.33E-05	1.14E-02	-2.79E-02	-4.00E-01	-3.60E-15	-6.91E-03	-1.54E-02	-1.36E-03	-4.59E-03	-6.68E-15	-1.49E-01
	Max	2.66E-02	6.57E-03	3.10E-01	-1.47E-02	-1.81E-01	3.60E-15	-1.89E-03	-4.46E-03	-3.52E-04	-9.16E-04	6.40E-15	-4.22E-02
	Normalized peak height	2.33E-02	1.25E-02	3.85E-03	3.57E-03	3.89E-03	5.00E-02	3.82E-03	4.00E-03	3.85E-03	3.95E-03	2.48E-02	3.48E-03
	Peak sensitivity value	4.69E-04	2.26E-04	6.54E-02	-2.03E-02	-2.38E-01	-2.12E-18	-3.44E-03	-8.23E-03	-6.46E-04	-1.88E-03	-4.01E-18	-6.79E-02
	Shapiro-Wilk score	6.220E-01	7.569E-01	9.599E-01	9.954E-01	9.612E-01	9.963E-01	9.868E-01	9.894E-01	9.857E-01	9.812E-01	9.941E-01	9.700E-01
Glyceraldehyde 3-phosphate	Local	2.26E-02	9.73E-03	1.76E+00	1.27E-02	1.54E-01	6.19E-15	1.56E-01	-5.55E-01	-4.76E-02	-1.44E-01	1.37E-14	-1.23E+00
	Min	4.67E-03	2.50E-03	1.44E+00	8.07E-03	9.33E-02	-2.65E-14	9.31E-02	-7.45E-01	-6.58E-02	-2.23E-01	-4.54E-14	-1.89E+00
	Max	2.80E-01	6.31E-02	2.22E+00	1.95E-02	3.04E-01	2.99E-14	2.36E-01	-3.63E-01	-3.36E-02	-7.90E-02	4.38E-14	-7.26E-01
	Normalized peak height	1.50E-02	8.45E-03	2.97E-03	3.79E-03	4.10E-03	6.10E-03	3.83E-03	3.43E-03	3.43E-03	3.38E-03	5.25E-02	3.31E-03
	Peak sensitivity value	1.14E-02	6.95E-03	1.70E+00	1.22E-02	1.35E-01	6.35E-16	1.49E-01	-5.57E-01	-4.61E-02	-1.42E-01	-3.21E-17	-1.19E+00
	Shapiro-Wilk score	6.927E-01	8.409E-01	9.828E-01	9.892E-01	9.489E-01	9.924E-01	9.971E-01	9.995E-01	9.977E-01	9.944E-01	9.954E-01	9.901E-01
Dihydroxyacetone phosphate	Local	2.40E-02	1.03E-02	1.88E+00	1.23E-02	1.49E-01	5.18E-15	-2.24E-01	-4.91E-01	-4.21E-02	-1.27E-01	1.04E-14	-1.03E+00
	Min	5.21E-03	2.75E-03	1.56E+00	7.72E-03	8.81E-02	-2.92E-14	-3.25E-01	-6.59E-01	-5.79E-02	-2.13E-01	-4.66E-14	-1.59E+00
	Max	3.90E-01	1.03E-01	2.31E+00	2.00E-02	2.92E-01	2.71E-14	-1.35E-01	-3.22E-01	-2.95E-02	-6.91E-02	3.87E-14	-5.64E-01
	Normalized peak height	1.90E-02	1.29E-02	3.14E-03	3.98E-03	4.42E-03	7.13E-03	3.20E-03	3.50E-03	3.46E-03	3.88E-03	3.77E-02	3.20E-03
	Peak sensitivity value	1.08E-02	7.73E-03	1.77E+00	1.13E-02	1.37E-01	3.95E-16	-2.29E-01	-4.90E-01	-4.07E-02	-1.28E-01	-1.35E-17	-1.05E+00
	Shapiro-Wilk score	6.983E-01	8.372E-01	9.829E-01	9.884E-01	9.446E-01	9.935E-01	9.969E-01	9.996E-01	9.978E-01	9.940E-01	9.952E-01	9.893E-01
Glucose-6-Phosphate	Local	1.37E-02	5.88E-03	1.07E+00	-2.72E-02	-3.29E-01	-8.33E-16	5.10E-03	1.11E-02	9.57E-04	2.89E-03	-1.82E-15	1.02E-01
	Min	3.06E-03	1.62E-03	8.90E-01	-4.05E-02	-6.70E-01	-4.22E-15	2.24E-03	5.36E-03	3.91E-04	9.97E-04	-7.68E-15	4.81E-02
	Max	1.50E-01	3.58E-02	1.34E+00	-1.95E-02	-2.18E-01	6.31E-15	8.59E-03	1.86E-02	1.80E-03	6.31E-03	7.04E-15	1.76E-01
	Normalized peak height	1.39E-02	8.49E-03	6.01E-03	3.81E-03	4.21E-03	5.59E-02	3.47E-03	3.80E-03	4.05E-03	3.90E-03	2.36E-02	3.35E-03
	Peak sensitivity value	7.25E-03	3.96E-03	1.04E+00	-2.61E-02	-2.90E-01	-4.13E-18	4.92E-03	1.09E-02	8.53E-04	2.69E-03	-4.64E-18	9.44E-02
	Shapiro-Wilk score	6.903E-01	8.346E-01	9.581E-01	9.892E-01	9.424E-01	9.960E-01	9.943E-01	9.954E-01	9.905E-01	9.840E-01	9.966E-01	9.829E-01
NAD	Local	-4.87E-03	-2.10E-03	-3.80E-01	9.66E-04	1.17E-02	-3.24E-15	-8.00E-02	-1.75E-01	-1.50E-02	-4.53E-02	-6.78E-15	6.68E-01
	Min	-7.01E-02	-1.79E-02	-7.04E-01	-3.67E-04	-5.70E-03	-1.55E-14	-1.33E-01	-3.21E-01	-3.30E-02	-1.04E-01	-2.58E-14	3.65E-01
	Max	-7.35E-04	-4.08E-04	-2.00E-01	3.67E-03	4.67E-02	1.60E-14	-4.36E-02	-9.09E-02	-5.32E-03	-1.35E-02	2.64E-14	1.14E+00
	Normalized peak height	1.70E-02	1.09E-02	3.10E-03	4.58E-03	5.07E-03	7.03E-03	3.48E-03	3.75E-03	3.97E-03	3.91E-03	7.02E-02	3.25E-03
	Peak sensitivity value	-2.30E-03	-1.35E-03	-3.36E-01	7.87E-04	8.69E-03	-9.19E-17	-7.55E-02	-1.64E-01	-1.37E-02	-4.08E-02	-1.02E-17	6.23E-01
	Shapiro-Wilk score	6.755E-01	8.165E-01	9.760E-01	9.812E-01	9.770E-01	9.917E-01	9.936E-01	9.887E-01	9.796E-01	9.802E-01	9.957E-01	9.853E-01
Extracellular acetaldehyde	Local	2.55E-02	1.10E-02	1.99E+00	8.78E-03	1.06E-01	-5.73E-15	-8.32E-02	-1.82E-01	-1.56E-02	-4.71E-02	-8.63E-15	-1.67E+00
	Min	5.48E-03	2.77E-03	1.68E+00	5.67E-03	6.18E-02	-2.98E-14	-1.32E-01	-2.58E-01	-2.26E-02	-8.05E-02	-4.46E-14	-2.04E+00
	Max	2.77E-01	8.80E-02	2.36E+00	1.32E-02	2.20E-01	2.56E-14	-4.59E-02	-1.09E-01	-1.03E-02	-2.60E-02	4.15E-14	-1.34E+00
	Normalized peak height	1.27E-02	1.04E-02	3.14E-03	3.65E-03	4.39E-03	8.46E-03	3.39E-03	3.82E-03	3.76E-03	3.82E-03	6.28E-02	3.05E-03
	Peak sensitivity value	1.24E-02	7.68E-03	1.95E+00	8.37E-03	9.39E-02	1.38E-17	-7.82E-02	-1.86E-01	-1.53E-02	-4.57E-02	2.37E-17	-1.65E+00
	Shapiro-Wilk score	6.966E-01	8.404E-01	9.894E-01	9.843E-01	9.370E-01	9.843E-01	9.967E-01	9.993E-01	9.967E-01	9.937E-01	9.908E-01	9.912E-01
Phosphoenolpyruvate	Local	1.38E-02	5.93E-03	1.08E+00	6.08E-03	7.36E-02	2.62E-15	1.11E-03	2.42E-03	2.08E-04	-1.20E+00	7.14E-14	2.22E-02
	Min	3.08E-03	1.64E-03	8.47E-01	4.46E-03	5.11E-02	-2.28E-14	5.84E-04	1.28E-03	1.07E-04	-1.26E+00	-2.46E-13	1.16E-02
	Max	1.59E-01	3.95E-02	1.13E+00	8.44E-03	1.31E-01	2.27E-14	2.14E-03	4.41E-03	4.26E-04	-1.16E+00	2.59E-13	4.68E-02
	Normalized peak height	1.39E-02	8.58E-03	6.70E-03	3.65E-03	3.86E-03	2.09E-02	4.01E-03	4.01E-03	4.01E-03	2.46E-03	2.03E-01	3.67E-03
	Peak sensitivity value	7.22E-03	4.68E-03	1.07E+00	5.78E-03	6.81E-02	5.65E-18	1.03E-03	2.24E-03	1.86E-04	-1.20E+00	4.47E-17	2.03E-02
	Shapiro-Wilk score	7.127E-01	8.584E-01	9.594E-01	9.925E-01	9.450E-01	9.970E-01	9.826E-01	9.836E-01	9.810E-01	9.863E-01	9.663E-01	9.626E-01

Metabolite	Statistic	(Ethanolout)	(Ethanolflow)	(Glycerolsynthesis)	(Glycerolout)	(Glycerolflow)	(Acetaldehydeout)	(Acetaldehydeflow)	(Cyanide-Acetaldehydeflow)	(Cyanideflow)	(Storage)	(ATPconsumption)	(Adenylatekinase)
Acetaldehyde	Local	0.00E+00	0.00E+00	2.83E-01	-5.12E-15	7.96E-15	-6.23E-02	-3.24E-01	-9.20E-02	-7.34E-03	-7.38E-02	1.26E-01	1.05E-17
	Min	-1.86E-15	-1.60E-15	1.79E-01	-8.77E-14	-6.11E-14	-9.16E-02	-4.27E-01	-1.23E-01	-1.73E-02	-9.58E-02	9.45E-02	-6.16E-17
	Max	1.93E-15	1.50E-15	3.96E-01	7.50E-14	6.48E-14	-4.01E-02	-2.31E-01	-6.22E-02	-2.97E-03	-4.30E-02	1.66E-01	9.14E-16
	Normalized peak height	9.23E-01	9.30E-01	3.35E-03	6.28E-03	1.68E-02	3.59E-03	3.59E-03	3.32E-03	4.20E-03	5.18E-03	3.26E-03	3.44E-01
	Peak sensitivity value	1.63E-18	-1.02E-18	2.85E-01	8.56E-17	2.20E-17	-6.11E-02	-3.21E-01	-9.20E-02	-6.20E-03	-7.24E-02	1.24E-01	3.07E-19
	Shapiro-Wilk score	2.954E-01	2.808E-01	9.995E-01	9.973E-01	9.963E-01	9.953E-01	9.971E-01	9.976E-01	9.695E-01	9.981E-01	9.975E-01	7.659E-01
Cytosolic glucose	Local	0.00E+00	0.00E+00	9.58E-03	7.63E-16	4.37E-17	2.30E-03	1.20E-02	3.40E-03	2.71E-04	3.43E-01	2.74E-02	7.34E-18
	Min	-3.45E-16	-3.59E-16	3.59E-03	-1.52E-14	-1.30E-14	9.76E-04	5.33E-03	1.54E-03	7.98E-05	1.73E-01	1.41E-02	-5.55E-17
	Max	4.04E-16	2.93E-16	3.00E-02	1.24E-14	8.53E-15	6.76E-03	3.38E-02	8.90E-03	1.05E-03	8.43E-01	6.51E-02	2.76E-16
	Normalized peak height	9.22E-01	9.27E-01	5.30E-03	7.08E-03	8.01E-03	5.21E-03	4.89E-03	4.83E-03	5.35E-03	4.60E-03	4.83E-03	3.42E-01
	Peak sensitivity value	1.96E-20	-4.61E-20	7.56E-03	2.00E-17	2.54E-17	2.03E-03	1.13E-02	3.00E-03	1.97E-04	3.14E-01	2.51E-02	1.22E-19
	Shapiro-Wilk score	2.859E-01	2.779E-01	9.345E-01	9.904E-01	9.889E-01	9.399E-01	9.379E-01	9.445E-01	9.110E-01	9.444E-01	9.551E-01	8.757E-01
Glycerol	Local	0.00E+00	0.00E+00	2.79E-01	-5.98E-01	-4.02E-01	6.70E-02	3.48E-01	9.89E-02	7.89E-03	-7.27E-02	1.24E-01	1.03E-17
	Min	-2.37E-15	-1.91E-15	1.72E-01	-6.65E-01	-4.74E-01	4.12E-02	2.53E-01	6.59E-02	4.20E-03	-9.50E-02	9.01E-02	-6.31E-17
	Max	1.94E-15	1.87E-15	3.92E-01	-5.26E-01	-3.35E-01	1.00E-01	4.34E-01	1.42E-01	1.26E-02	-4.05E-02	1.60E-01	9.92E-16
	Normalized peak height	9.24E-01	9.29E-01	3.34E-03	2.73E-03	2.73E-03	3.67E-03	3.67E-03	3.83E-03	3.25E-03	5.38E-03	3.38E-03	3.43E-01
	Peak sensitivity value	-5.13E-19	1.52E-18	2.78E-01	-5.93E-01	-4.07E-01	6.86E-02	3.50E-01	1.03E-01	7.27E-03	-7.35E-02	1.23E-01	-3.02E-19
	Shapiro-Wilk score	2.871E-01	2.802E-01	9.995E-01	9.970E-01	9.970E-01	9.979E-01	9.994E-01	9.993E-01	9.909E-01	9.983E-01	9.979E-01	8.007E-01
EtOH	Local	-1.45E-01	-8.55E-01	-3.13E-02	-1.63E-16	1.29E-15	-7.52E-03	-3.91E-02	-1.11E-02	-8.85E-04	-1.89E-02	5.21E-02	2.13E-18
	Min	-1.84E-01	-8.88E-01	-5.38E-02	-7.04E-15	-4.70E-15	-1.23E-02	-5.58E-02	-1.51E-02	-2.01E-03	-2.84E-02	3.47E-02	-1.20E-17
	Max	-1.12E-01	-8.16E-01	-1.60E-02	7.87E-15	4.55E-15	-4.50E-03	-2.68E-02	-7.84E-03	-3.45E-04	-6.36E-03	7.08E-02	1.89E-16
	Normalized peak height	2.58E-03	2.58E-03	4.00E-03	5.86E-03	9.18E-03	3.57E-03	2.92E-03	2.90E-03	3.51E-03	4.61E-03	3.12E-03	3.44E-01
	Peak sensitivity value	-1.44E-01	-8.56E-01	-3.12E-02	5.00E-18	-5.59E-18	-7.61E-03	-3.80E-02	-1.08E-02	-8.49E-04	-1.96E-02	5.49E-02	-6.43E-20
	Shapiro-Wilk score	9.955E-01	9.955E-01	9.915E-01	9.973E-01	9.946E-01	9.932E-01	9.926E-01	9.951E-01	9.709E-01	9.981E-01	9.972E-01	7.562E-01
ADP	Local	0.00E+00	0.00E+00	1.33E-02	1.09E-15	5.21E-17	3.20E-03	1.66E-02	4.72E-03	3.76E-04	1.88E-01	3.81E-02	-4.97E-17
	Min	-5.29E-16	-5.71E-16	6.37E-03	-1.42E-14	-9.14E-15	1.82E-03	1.06E-02	3.00E-03	1.32E-04	1.45E-01	2.62E-02	-4.76E-15
	Max	4.77E-16	4.42E-16	2.35E-02	1.37E-14	1.06E-14	5.37E-03	2.48E-02	6.81E-03	8.87E-04	2.22E-01	5.38E-02	2.69E-16
	Normalized peak height	9.24E-01	9.33E-01	4.04E-03	5.12E-03	5.87E-03	3.66E-03	3.30E-03	3.39E-03	3.96E-03	3.57E-03	3.60E-03	3.44E-01
	Peak sensitivity value	3.02E-19	-4.59E-19	1.22E-02	-3.99E-16	-6.58E-18	3.09E-03	1.60E-02	4.59E-03	3.15E-04	1.86E-01	3.76E-02	-4.30E-19
	Shapiro-Wilk score	2.877E-01	2.794E-01	9.884E-01	9.978E-01	9.973E-01	9.905E-01	9.916E-01	9.953E-01	9.662E-01	9.997E-01	9.972E-01	7.703E-01
Glyceraldehyde 3-phosphate	Local	0.00E+00	0.00E+00	-5.32E-01	7.05E-15	-4.91E-15	5.03E-02	2.61E-01	7.42E-02	5.92E-03	-1.10E-01	1.05E-01	1.80E-17
	Min	-1.46E-15	-1.68E-15	-7.90E-01	-1.26E-13	-7.81E-14	2.85E-02	1.63E-01	4.55E-02	2.63E-03	-1.46E-01	7.47E-02	-9.90E-17
	Max	1.63E-15	1.64E-15	-3.24E-01	1.08E-13	8.36E-14	8.32E-02	3.67E-01	1.12E-01	1.25E-02	-8.16E-02	1.45E-01	1.53E-15
	Normalized peak height	9.23E-01	9.30E-01	3.87E-03	8.58E-03	2.23E-02	3.92E-03	3.61E-03	3.60E-03	3.94E-03	3.79E-03	3.61E-03	3.43E-01
	Peak sensitivity value	-8.15E-19	7.51E-19	-5.23E-01	-9.38E-17	-4.82E-17	4.87E-02	2.64E-01	7.35E-02	5.32E-03	-1.09E-01	1.03E-01	-4.26E-19
	Shapiro-Wilk score	2.887E-01	2.798E-01	9.968E-01	9.962E-01	9.950E-01	9.954E-01	9.988E-01	9.973E-01	9.826E-01	9.982E-01	9.958E-01	8.040E-01
Dihydroxyacetone phosphate	Local	0.00E+00	0.00E+00	-4.89E-01	6.32E-15	-3.90E-15	4.23E-02	2.20E-01	6.25E-02	4.99E-03	-1.06E-01	1.13E-01	1.69E-17
	Min	-1.82E-15	-1.49E-15	-7.10E-01	-1.03E-13	-6.75E-14	2.10E-02	1.28E-01	3.75E-02	2.11E-03	-1.37E-01	8.53E-02	-1.13E-16
	Max	2.24E-15	1.57E-15	-2.96E-01	1.03E-13	9.39E-14	7.09E-02	3.33E-01	9.48E-02	9.89E-03	-6.90E-02	1.50E-01	1.59E-15
	Normalized peak height	9.24E-01	9.32E-01	3.20E-03	9.28E-03	3.05E-02	3.82E-03	3.77E-03	3.63E-03	3.55E-03	4.18E-03	3.21E-03	3.47E-01
	Peak sensitivity value	-7.21E-19	1.09E-18	-4.79E-01	-9.74E-17	3.84E-17	4.04E-02	2.15E-01	6.22E-02	4.40E-03	-1.07E-01	1.13E-01	-1.36E-19
	Shapiro-Wilk score	2.841E-01	2.743E-01	9.947E-01	9.969E-01	9.957E-01	9.944E-01	9.979E-01	9.970E-01	9.818E-01	9.985E-01	9.968E-01	7.606E-01
Glucose-6-Phosphate	Local	0.00E+00	0.00E+00	-1.74E-02	-1.37E-15	-6.86E-17	-4.17E-03	-2.17E-02	-6.16E-03	-4.92E-04	-7.52E-01	-4.97E-02	-2.72E-17
	Min	-7.26E-16	-7.26E-16	-3.13E-02	-1.91E-14	-1.74E-14	-7.39E-03	-3.29E-02	-9.50E-03	-1.13E-03	-7.89E-01	-8.18E-02	-9.74E-16
	Max	7.24E-16	7.12E-16	-7.78E-03	1.76E-14	1.41E-14	-1.80E-03	-1.04E-02	-2.74E-03	-1.87E-04	-6.79E-01	-1.82E-02	8.23E-16
	Normalized peak height	9.28E-01	9.41E-01	3.76E-03	5.18E-03	6.30E-03	4.10E-03	3.60E-03	3.83E-03	3.72E-03	3.72E-03	3.63E-03	3.43E-01
	Peak sensitivity value	-5.78E-19	-3.49E-19	-1.63E-02	-2.65E-16	3.19E-17	-3.82E-03	-2.16E-02	-5.93E-03	-4.47E-04	-7.54E-01	-5.21E-02	-5.69E-19
	Shapiro-Wilk score	2.793E-01	2.732E-01	9.945E-01	9.981E-01	9.968E-01	9.960E-01	9.989E-01	9.998E-01	9.742E-01	9.918E-01	9.987E-01	8.820E-01
NAD	Local	0.00E+00	0.00E+00	2.72E-01	-3.23E-15	2.80E-15	-2.73E-02	-1.42E-01	-4.03E-02	-3.22E-03	-9.68E-03	-2.81E-02	2.62E-18
	Min	-7.15E-16	-6.07E-16	1.49E-01	-6.36E-14	-4.22E-14	-5.03E-02	-2.54E-01	-6.83E-02	-7.59E-03	-3.00E-02	-5.19E-02	-1.98E-17
	Max	6.32E-16	5.65E-16	4.46E-01	5.78E-14	4.44E-14	-1.34E-02	-8.09E-02	-2.15E-02	-1.27E-03	1.16E-03	-1.47E-02	1.26E-16
	Normalized peak height	9.22E-01	9.29E-01	3.28E-03	6.65E-03	1.62E-02	3.97E-03	4.02E-03	3.91E-03	4.08E-03	4.02E-03	3.19E-03	3.44E-01
	Peak sensitivity value	5.51E-19	-3.89E-19	2.54E-01	4.43E-17	3.00E-17	-2.60E-02	-1.36E-01	-3.96E-02	-3.05E-03	-9.54E-03	-2.57E-02	-6.70E-20
	Shapiro-Wilk score	2.926E-01	2.857E-01	9.922E-01	9.949E-01	9.917E-01	9.917E-01	9.947E-01	9.932E-01	9.763E-01	9.829E-01	9.830E-01	8.582E-01
Extracellular acetaldehyde	Local	0.00E+00	0.00E+00	2.84E-01	-5.10E-15	7.94E-15	6.82E-02	-4.25E-01	-1.21E-01	-9.62E-03	-7.39E-02	1.27E-01	1.05E-17
	Min	-2.06E-15	-1.52E-15	1.69E-01	-9.00E-14	-5.84E-14	4.38E-02	-5.27E-01	-1.55E-01	-2.08E-02	-9.65E-02	9.28E-02	-6.45E-17
	Max	1.87E-15	1.81E-15	3.93E-01	9.64E-14	5.35E-14	9.98E-02	-3.28E-01	-9.15E-02	-4.17E-03	-3.81E-02	1.66E-01	7.06E-16
	Normalized peak height	9.23E-01	9.30E-01	3.44E-03	8.21E-03	1.01E-02	3.37E-03	3.60E-03	3.31E-03	3.87E-03	5.55E-03	3.37E-03	3.42E-01
	Peak sensitivity value	1.19E-18	-6.53E-19	2.75E-01	1.15E-16	3.34E-17	7.03E-02	-4.11E-01	-1.18E-01	-8.50E-03	-7.38E-02	1.27E-01	-1.73E-19
	Shapiro-Wilk score	2.889E-01	2.863E-01	9.996E-01	9.957E-01	9.945E-01	9.980E-01	9.977E-01	9.980E-01	9.733E-01	9.983E-01	9.976E-01	8.073E-01
Phosphoenolpyruvate	Local	0.00E+00	0.00E+00	-3.77E-03	4.71E-15	-1.00E-15	-9.07E-04	-4.72E-03	-1.34E-03	-1.07E-04	-5.20E-02	6.64E-02	9.58E-18
	Min	-8.96E-16	-7.40E-16	-6.97E-03	-1.81E-14	-1.09E-14	-1.60E-03	-7.86E-03	-2.17E-03	-2.68E-04	-6.10E-02	4.86E-02	-9.95E-17
	Max	7.90E-16	7.79E-16	-1.99E-03	1.88E-14	1.24E-14	-5.20E-04	-2.95E-03	-8.27E-04	-3.96E-05	-3.65E-02	8.72E-02	5.64E-16
	Normalized peak height	9.25E-01	9.34E-01	3.90E-03	4.93E-03	5.73E-03	3.72E-03	3.85E-03	3.97E-03	4.05E-03	4.79E-03	3.27E-03	3.44E-01
	Peak sensitivity value	-2.36E-19	4.08E-19	-3.54E-03	3.26E-16	8.73E-18	-8.66E-04	-4.61E-03	-1.30E-03	-8.96E-05	-5.24E-02	6.78E-02	-3.10E-19
	Shapiro-Wilk score	2.790E-01	2.588E-01	9.829E-01	9.971E-01	9.970E-01	9.864E-01	9.848E-01	9.897E-01	9.596E-01	9.962E-01	9.983E-01	8.717E-01

**Fructose-6-Phosphate**																								
Local	1.24E-02	5.33E-03	9.67E-01	8.44E-02	-3.38E-01	-8.95E-16	5.90E-03	1.29E-02	1.11E-03	3.34E-03	-2.13E-15	1.18E-01	0.00E+00	0.00E+00	-2.01E-02	-1.59E-15	-7.98E-17	-4.83E-03	-2.51E-02	-7.13E-03	-5.69E-04	-7.58E-01	-5.75E-02	-2.80E-17
Min	2.58E-03	1.31E-03	7.99E-01	6.40E-02	-6.95E-01	-5.28E-15	2.87E-03	6.67E-03	5.29E-04	1.31E-03	-1.19E-14	6.03E-02	-8.88E-16	-7.63E-16	-3.39E-02	-2.12E-14	-1.97E-14	-7.98E-03	-3.69E-02	-1.06E-02	-1.26E-03	-7.97E-01	-8.99E-02	-1.03E-14
Max	1.27E-01	3.19E-02	1.19E+00	1.11E-01	-2.23E-01	4.86E-15	1.02E-02	2.13E-02	2.13E-03	8.15E-03	9.41E-15	2.03E-01	8.48E-16	7.54E-16	-1.02E-02	2.30E-14	1.92E-14	-2.09E-03	-1.32E-02	-3.75E-03	-2.16E-04	-6.93E-01	-2.49E-02	1.33E-15
Normalized peak height	1.28E-02	7.84E-03	4.83E-03	3.56E-03	4.37E-03	6.04E-02	3.90E-03	3.55E-03	3.78E-03	4.57E-03	2.26E-02	3.22E-03	9.28E-01	9.40E-01	3.58E-03	5.16E-03	7.04E-03	3.71E-03	3.47E-03	3.40E-03	3.60E-03	3.69E-03	3.47E-03	3.45E-01
Peak sensitivity value	6.74E-03	3.86E-03	9.45E-01	8.14E-02	-3.13E-01	-3.44E-18	5.42E-03	1.19E-02	1.01E-03	3.17E-03	8.98E-18	1.10E-01	-4.23E-20	3.36E-19	-1.96E-02	-5.62E-16	4.13E-17	-4.69E-03	-2.52E-02	-7.06E-03	-4.50E-04	-7.57E-01	-5.69E-02	3.19E-18
Shapiro-Wilk score	6.958E-01	8.385E-01	9.737E-01	9.940E-01	9.433E-01	9.958E-01	9.935E-01	9.950E-01	9.900E-01	9.831E-01	9.952E-01	9.815E-01	2.823E-01	2.690E-01	9.937E-01	9.976E-01	9.978E-01	9.943E-01	9.975E-01	9.992E-01	9.739E-01	9.913E-01	9.993E-01	3.718E-01
**Extracellular cyanide**																								
Local	-1.89E-03	-8.11E-04	-1.47E-01	-6.48E-04	-7.85E-03	4.27E-16	6.15E-03	1.34E-02	1.15E-03	3.48E-03	6.32E-16	1.23E-01	0.00E+00	0.00E+00	-2.09E-02	3.66E-16	-5.96E-16	-5.03E-03	3.14E-02	-6.50E-02	7.46E-02	5.46E-03	-9.35E-03	-7.74E-19
Min	-2.58E-02	-6.80E-03	-2.80E-01	-1.55E-03	-2.31E-02	-1.86E-15	3.10E-03	6.41E-03	5.28E-04	1.39E-03	-2.96E-15	6.10E-02	-1.15E-16	-1.02E-16	-3.75E-02	-7.25E-15	-4.97E-15	-8.04E-03	1.51E-02	-1.10E-01	4.08E-02	2.65E-03	-1.71E-02	-4.97E-17
Max	-2.91E-04	-1.49E-04	-7.29E-02	-2.65E-04	-3.07E-03	1.67E-15	1.15E-02	2.49E-02	2.12E-03	7.99E-03	3.33E-15	2.42E-01	1.39E-16	9.45E-17	-1.04E-02	5.66E-15	4.14E-15	-2.77E-03	6.18E-02	-3.66E-02	1.29E-01	1.02E-02	-4.76E-03	4.80E-18
Normalized peak height	1.62E-02	1.06E-02	3.59E-03	4.02E-03	4.94E-03	8.58E-03	3.75E-03	3.93E-03	3.82E-03	4.29E-03	6.59E-02	3.55E-03	9.24E-01	9.31E-01	3.73E-03	7.39E-03	1.47E-02	3.39E-03	3.84E-03	3.50E-03	3.73E-03	3.64E-03	3.62E-03	3.41E-01
Peak sensitivity value	-8.14E-04	-4.98E-04	-1.32E-01	-5.52E-04	-6.28E-03	-1.18E-18	5.66E-03	1.23E-02	1.05E-03	3.17E-03	1.83E-18	1.04E-01	1.23E-19	-8.50E-20	-1.92E-02	1.16E-18	-1.72E-18	-4.96E-03	2.84E-02	-6.41E-02	6.74E-02	4.88E-03	-8.64E-03	-1.87E-20
Shapiro-Wilk score	6.699E-01	8.116E-01	9.739E-01	9.572E-01	9.218E-01	9.835E-01	9.847E-01	9.888E-01	9.841E-01	9.777E-01	9.943E-01	9.703E-01	2.874E-01	2.797E-01	9.874E-01	9.956E-01	9.951E-01	9.909E-01	9.705E-01	9.827E-01	9.815E-01	9.797E-01	9.828E-01	8.392E-01
**Pyruvate**																								
Local	9.01E-01	3.88E-01	7.04E+01	2.28E-01	2.76E+00	-6.96E-14	4.07E-02	8.91E-02	7.64E-03	2.31E-02	-7.79E+01	8.15E-01	0.00E+00	0.00E+00	-1.39E-01	-5.18E-14	1.49E-13	-3.34E-02	-1.73E-01	-4.93E-02	-3.93E-03	-1.87E+00	4.59E+00	2.26E-16
Min	1.54E-02	9.37E-03	4.70E+00	1.26E-02	1.38E-01	-3.42E-11	1.49E-03	3.28E-03	3.05E-04	7.91E-04	-3.40E+04	3.27E-02	-4.46E-15	-4.92E-15	-7.30E+01	-5.23E-11	-4.54E-11	-1.76E+01	-8.54E+01	-2.33E+01	-1.76E+00	-8.06E+02	3.01E-01	-1.03E-13
Max	5.63E+02	2.80E+02	3.02E+04	1.12E+02	1.56E+03	8.83E-12	2.25E+01	4.80E+01	4.04E+00	1.18E+01	-5.18E+00	4.20E+02	4.81E-15	4.59E-15	-5.53E-03	4.91E-11	2.45E-11	-1.53E-03	-8.03E-03	-2.25E-03	-1.19E-04	-1.21E-01	1.85E+03	5.25E-14
Normalized peak height	8.03E-01	8.43E-01	7.89E-01	8.05E-01	8.25E-01	7.48E-01	8.19E-01	8.14E-01	8.12E-01	8.04E-01	7.91E-01	8.03E-01	9.31E-01	9.47E-01	8.10E-01	7.36E-01	7.59E-01	8.14E-01	8.01E-01	7.93E-01	7.68E-01	7.88E-01	7.75E-01	7.19E-01
Peak sensitivity value	2.97E-01	1.50E-01	1.98E+01	6.85E-02	9.18E-01	-1.53E-14	1.27E-02	2.73E-02	2.33E-03	6.71E-03	-2.22E+01	2.43E-01	-3.17E-18	1.01E-18	-4.20E-02	-1.41E-14	6.22E-16	-1.03E-02	-5.07E-02	-1.39E-02	-9.98E-04	-5.24E-01	1.23E+00	7.17E-18
Shapiro-Wilk score	6.093E-02	5.535E-02	5.336E-02	5.183E-02	5.060E-02	4.709E-02	5.118E-02	5.084E-02	5.002E-02	5.104E-02	5.370E-02	5.308E-02	2.725E-01	2.508E-01	5.113E-02	7.579E-02	6.684E-02	5.352E-02	5.291E-02	5.332E-02	5.559E-02	5.189E-02	5.502E-02	7.511E-02
**Extracellular ethanol**																								
Local	1.01E-02	4.37E-03	7.92E-01	2.32E-03	2.81E-02	-3.81E-16	9.18E-03	2.01E-02	1.72E-03	5.20E-03	-1.74E-16	1.84E-01	-3.41E-16	-1.00E+00	-3.13E-02	-1.73E-16	1.29E-15	-7.52E-03	-3.91E-02	-1.11E-02	-8.85E-04	-1.89E-02	5.21E-02	2.13E-18
Min	2.23E-03	1.13E-03	6.15E-01	1.58E-03	1.78E-02	-1.63E-15	5.30E-03	1.13E-02	9.30E-04	2.20E-03	-3.45E-15	1.02E-01	-8.73E-16	-1.00E+00	-5.33E-02	-6.91E-15	-4.42E-15	-1.17E-02	-5.49E-02	-1.51E-02	-2.00E-03	-2.79E-02	3.45E-02	-1.29E-17
Max	1.19E-01	3.46E-02	8.61E-01	3.73E-03	5.56E-02	1.88E-15	1.50E-02	3.32E-02	2.95E-03	1.13E-02	2.93E-15	3.08E-01	7.89E-16	-1.00E+00	-1.84E-02	6.56E-15	5.09E-15	-4.57E-03	-2.67E-02	-7.84E-03	-3.60E-04	-2.82E-03	7.19E-02	8.93E-17
Normalized peak height	1.40E-02	1.06E-02	3.75E-03	3.95E-03	3.86E-03	2.04E-02	3.46E-03	3.71E-03	3.57E-03	4.36E-03	6.08E-02	2.79E-03	6.24E-01	1.00E+00	3.75E-03	5.95E-03	9.35E-03	3.28E-03	3.04E-03	2.94E-03	3.64E-03	5.26E-03	3.24E-03	3.45E-01
Peak sensitivity value	5.22E-03	3.12E-03	8.12E-01	2.27E-03	2.60E-02	1.31E-18	8.60E-03	1.94E-02	1.62E-03	4.86E-03	-3.14E-18	1.55E-01	5.54E-19	-1.00E+00	-2.86E-02	-4.37E-17	2.55E-19	-7.16E-03	-3.65E-02	-1.11E-02	-7.60E-04	-1.97E-02	5.29E-02	2.44E-20
Shapiro-Wilk score	7.100E-01	8.554E-01	9.572E-01	9.934E-01	9.618E-01	9.835E-01	9.909E-01	9.929E-01	9.887E-01	9.845E-01	9.984E-01	9.743E-01	8.347E-01	1.000E+00	9.912E-01	9.969E-01	9.963E-01	9.925E-01	9.922E-01	9.951E-01	9.705E-01	9.981E-01	9.974E-01	9.111E-01
**Extracellular glycerol**																								
Local	2.51E-02	1.08E-02	1.96E+00	8.63E-03	1.04E-01	8.14E-15	-8.18E-02	-1.79E-01	-1.53E-02	-4.63E-02	1.49E-14	-1.64E+00	0.00E+00	0.00E+00	2.79E-01	9.72E-15	-1.00E+00	6.70E-02	3.48E-01	9.89E-02	7.89E-03	-7.27E-02	1.24E-01	1.03E-17
Min	5.15E-03	2.80E-03	1.65E+00	5.71E-03	6.42E-02	-4.33E-14	-1.33E-01	-2.58E-01	-2.23E-02	-8.21E-02	-6.46E-14	-2.01E+00	-2.30E-15	-2.05E-15	1.76E-01	-1.36E-13	-1.00E+00	4.13E-02	2.54E-01	6.53E-02	4.37E-03	-9.40E-02	9.24E-02	-5.87E-17
Max	2.75E-01	6.16E-02	2.27E+00	1.34E-02	2.07E-01	3.57E-14	-4.60E-02	-1.15E-01	-1.02E-02	-2.38E-02	6.24E-14	-1.34E+00	2.37E-15	2.58E-15	3.86E-01	1.41E-13	-1.00E+00	9.95E-02	4.38E-01	1.41E-01	1.37E-02	-4.85E-02	1.60E-01	5.27E-16
Normalized peak height	1.30E-02	7.33E-03	3.11E-03	3.68E-03	4.20E-03	6.28E-03	3.21E-03	3.48E-03	3.81E-03	4.08E-03	4.00E-02	3.12E-03	9.24E-01	9.29E-01	3.25E-03	8.78E-03	1.00E+00	3.75E-03	3.72E-03	3.75E-03	3.52E-03	4.58E-03	3.11E-03	3.45E-01
Peak sensitivity value	1.10E-02	7.83E-03	1.94E+00	8.19E-03	9.32E-02	9.44E-16	-7.89E-02	-1.79E-01	-1.49E-02	-4.37E-02	-3.46E-17	-1.58E+00	1.97E-18	7.43E-19	2.82E-01	-8.87E-17	-1.00E+00	6.80E-02	3.55E-01	1.01E-01	7.56E-03	-7.13E-02	1.22E-01	2.33E-19
Shapiro-Wilk score	6.963E-01	8.422E-01	9.904E-01	9.848E-01	9.372E-01	9.910E-01	9.962E-01	9.993E-01	9.969E-01	9.933E-01	9.919E-01	9.920E-01	2.848E-01	2.817E-01	9.994E-01	9.977E-01	1.000E+00	9.981E-01	9.993E-01	9.996E-01	9.913E-01	9.984E-01	9.977E-01	8.755E-01
**Extracellular glucose**																								
Local	2.57E+00	-1.40E-02	-2.53E+00	-3.55E-03	-4.30E-02	8.97E-17	-5.98E-04	-1.31E-03	-1.12E-04	-3.38E-04	2.12E-16	-1.20E-02	0.00E+00	0.00E+00	2.04E-03	1.65E-16	1.51E-17	4.89E-04	2.54E-03	7.23E-04	5.76E-05	2.55E-02	5.83E-03	-3.53E-18
Min	1.41E+00	-1.93E-01	-6.29E+00	-1.42E-02	-2.50E-01	-1.04E-15	-3.44E-03	-7.57E-03	-6.42E-04	-1.96E-03	-2.81E-15	-6.43E-02	-9.42E-17	-6.75E-17	5.32E-04	-4.79E-15	-3.51E-15	1.42E-04	7.57E-04	2.29E-04	1.12E-05	-1.43E-02	2.29E-03	-3.18E-16
Max	6.49E+00	-2.36E-03	-1.39E+00	-1.32E-03	-1.40E-02	9.43E-16	-1.53E-04	-3.41E-04	-3.10E-05	-8.48E-05	2.34E-15	-3.10E-03	7.35E-17	6.71E-17	1.14E-02	4.24E-15	2.73E-15	2.35E-03	1.19E-02	2.92E-03	3.71E-04	7.25E-02	2.35E-02	9.89E-17
Normalized peak height	4.02E-03	1.62E-02	4.05E-03	5.86E-03	8.28E-03	6.95E-02	7.74E-03	7.91E-03	8.01E-03	8.07E-03	2.42E-02	7.31E-03	9.25E-01	9.30E-01	8.08E-03	1.15E-02	1.20E-02	6.92E-03	6.63E-03	5.90E-03	8.19E-03	6.45E-03	6.20E-03	3.43E-01
Peak sensitivity value	1.96E+00	-7.42E-03	-1.97E+00	-2.70E-03	-3.20E-02	8.69E-19	-4.18E-04	-1.01E-03	-7.77E-05	-2.19E-04	1.04E-18	-8.21E-03	-1.20E-20	4.29E-20	1.43E-03	1.20E-17	9.84E-18	3.68E-04	1.80E-03	5.38E-04	3.58E-05	2.05E-02	4.48E-03	1.96E-19
Shapiro-Wilk score	9.073E-01	6.993E-01	9.114E-01	8.547E-01	8.239E-01	9.663E-01	8.646E-01	8.650E-01	8.632E-01	8.632E-01	9.223E-01	8.443E-01	2.704E-01	2.662E-01	8.660E-01	9.597E-01	9.553E-01	8.730E-01	8.701E-01	8.756E-01	8.361E-01	9.380E-01	8.915E-01	7.447E-01
**1-3-Bisphosphoglycerate**																								
Local	1.07E-02	4.61E-03	8.37E-01	4.44E-02	5.38E-01	5.30E-16	8.28E-03	1.81E-02	-2.84E-01	-8.56E-01	3.50E-14	1.66E-01	0.00E+00	0.00E+00	-2.82E-02	6.06E-16	-4.86E-16	-6.78E-03	-3.52E-02	-1.00E-02	-7.98E-04	-3.98E-01	-7.28E-03	7.56E-17
Min	2.56E-03	1.28E-03	3.85E-01	3.37E-02	3.96E-01	-1.20E-14	4.18E-03	1.00E-02	-3.80E-01	-9.73E-01	-1.06E-13	8.79E-02	-6.51E-16	-7.01E-16	-5.47E-02	-3.36E-14	-2.53E-14	-1.17E-02	-6.14E-02	-1.57E-02	-1.99E-03	-4.29E-01	-3.50E-02	-1.17E-15
Max	9.23E-02	2.29E-02	1.03E+00	5.82E-02	8.95E-01	1.19E-14	1.62E-02	3.54E-02	-2.12E-01	-7.19E-01	1.18E-13	3.31E-01	7.84E-16	5.78E-16	-1.51E-02	3.27E-14	2.87E-14	-3.87E-03	-2.20E-02	-6.65E-03	-3.08E-04	-3.65E-01	7.07E-03	3.53E-15
Normalized peak height	1.08E-02	6.68E-03	3.89E-03	3.28E-03	3.60E-03	4.04E-02	4.07E-03	4.17E-03	3.24E-03	3.13E-03	8.89E-03	3.60E-03	9.25E-01	9.34E-01	4.27E-03	4.85E-03	5.78E-03	3.84E-03	3.86E-03	3.46E-03	3.97E-03	3.75E-03	3.77E-03	3.41E-01
Peak sensitivity value	6.29E-03	3.30E-03	8.68E-01	4.36E-02	4.93E-01	-9.18E-18	7.59E-03	1.74E-02	-2.93E-01	-8.47E-01	6.66E-18	1.31E-01	-1.42E-19	5.82E-19	-2.80E-02	6.80E-16	4.37E-16	-6.80E-03	-3.48E-02	-9.75E-03	-7.05E-04	-3.99E-01	-4.47E-03	1.21E-19
Shapiro-Wilk score	7.392E-01	8.821E-01	9.426E-01	9.946E-01	9.422E-01	9.978E-01	9.819E-01	9.832E-01	9.977E-01	9.977E-01	9.938E-01	9.615E-01	2.810E-01	2.774E-01	9.819E-01	9.981E-01	9.980E-01	9.855E-01	9.843E-01	9.895E-01	9.587E-01	9.993E-01	9.702E-01	8.609E-01
**Fructose 1-6-bisphosphate**																								
Local	4.60E-02	1.98E-02	3.59E+00	2.43E-02	2.94E-01	-9.76E-02	-8.09E-02	-1.00E+00	-8.59E-02	-2.59E-01	2.20E-14	-2.18E+00	0.00E+00	0.00E+00	-9.76E-01	1.28E-14	-8.40E-15	8.90E-02	4.63E-01	1.31E-01	1.05E-02	-2.10E-01	2.16E-01	3.37E-17
Min	9.69E-03	4.98E-03	2.96E+00	1.54E-02	1.74E-01	-1.65E-01	-2.13E-01	-1.34E+00	-1.17E-01	-4.32E-01	-1.04E-13	-3.36E+00	-3.33E-15	-3.01E-15	-1.41E+00	-2.04E-13	-1.63E-13	5.06E-02	2.90E-01	8.10E-02	4.68E-03	-2.71E-01	1.59E-01	-1.85E-16
Max	5.11E-01	1.25E-01	4.50E+00	3.70E-02	5.67E-01	-6.30E-02	5.53E-02	-6.83E-01	-5.94E-02	-1.44E-01	9.37E-14	-1.31E+00	2.98E-15	3.48E-15	-6.13E-01	2.07E-13	1.82E-13	1.45E-01	6.60E-01	1.97E-01	2.14E-02	-1.56E-01	2.89E-01	2.31E-15
Normalized peak height	1.33E-02	8.41E-03	3.17E-03	3.67E-03	4.10E-03	3.83E-03	3.33E-03	3.49E-03	3.47E-03	3.90E-03	3.95E-02	3.11E-03	9.28E-01	9.41E-01	3.24E-03	1.16E-02	2.22E-02	3.56E-03	3.72E-03	3.40E-03	3.78E-03	3.70E-03	3.24E-03	3.43E-01
Peak sensitivity value	2.40E-02	1.36E-02	3.40E+00	2.28E-02	2.66E-01	-9.61E-02	-8.94E-02	-9.74E-01	-8.40E-02	-2.50E-01	-4.96E-17	-1.91E+00	2.35E-18	-1.18E-18	-9.73E-01	5.99E-17	-1.58E-16	8.21E-02	4.55E-01	1.26E-01	9.27E-03	-2.09E-01	2.09E-01	6.92E-19
Shapiro-Wilk score	6.909E-01	8.363E-01	9.821E-01	9.885E-01	9.455E-01	9.944E-01	9.992E-01	9.997E-01	9.973E-01	9.948E-01	9.945E-01	9.891E-01	2.825E-01	2.683E-01	9.953E-01	9.970E-01	9.953E-01	9.951E-01	9.982E-01	9.966E-01	9.819E-01	9.983E-01	9.964E-01	8.672E-01
**AMP**																								
Local	4.17E-03	1.79E-03	3.25E-01	-6.74E-02	-8.16E-01	2.40E-15	-1.26E-02	-2.76E-02	-2.37E-03	-7.14E-03	6.43E-15	-2.52E-01	0.00E+00	0.00E+00	4.30E-02	3.70E-15	2.03E-16	1.03E-02	5.37E-02	1.52E-02	1.22E-03	6.08E-01	1.23E-01	6.38E-17
Min	1.99E-04	1.24E-04	4.08E-02	-8.65E-02	-1.34E+00	-1.19E-14	-2.31E-02	-5.13E-02	-4.61E-03	-1.55E-02	-2.29E-14	-5.17E-01	-1.89E-15	-1.76E-15	2.37E-02	-5.96E-14	-4.67E-14	5.82E-03	3.53E-02	9.99E-03	4.60E-04	5.78E-01	9.59E-02	-3.38E-16
Max	9.58E-02	2.34E-02	1.04E+00	-5.11E-02	-6.12E-01	1.28E-14	-6.90E-03	-1.54E-02	-1.21E-03	-2.85E-03	3.06E-14	-1.39E-01	1.76E-15	1.88E-15	7.76E-02	4.31E-14	3.96E-14	1.92E-02	8.86E-02	2.36E-02	3.20E-03	6.41E-01	1.65E-01	2.67E-15
Normalized peak height	2.54E-02	1.38E-02	3.67E-03	3.29E-03	3.53E-03	6.68E-02	4.06E-03	3.83E-03	4.14E-03	4.50E-03	2.63E-02	3.56E-03	9.27E-01	9.41E-01	3.79E-03	5.49E-03	6.85E-03	3.94E-03	3.67E-03	3.58E-03	4.19E-03	3.81E-03	3.15E-03	3.45E-01
Peak sensitivity value	1.30E-03	7.88E-04	2.84E-01	-6.39E-02	-7.46E-01	6.17E-18	-1.13E-02	-2.56E-02	-2.10E-03	-6.12E-03	9.33E-18	-2.31E-01	1.05E-18	-6.96E-19	3.84E-02	-9.78E-16	-3.28E-17	9.51E-03	4.96E-02	1.41E-02	1.10E-03	6.08E-01	1.22E-01	-2.50E-19
Shapiro-Wilk score	6.174E-01	7.510E-01	9.501E-01	9.946E-01	9.434E-01	9.963E-01	9.817E-01	9.839E-01	9.811E-01	9.776E-01	9.924E-01	9.622E-01	2.875E-01	2.739E-01	9.827E-01	9.973E-01	9.968E-01	9.868E-01	9.850E-01	9.900E-01	9.595E-01	9.997E-01	9.955E-01	8.550E-01
**ATP**																								
Local	-1.59E-03	-6.83E-04	-1.24E-01	2.57E-02	3.11E-01	-8.56E-16	4.80E-03	1.05E-02	9.01E-04	2.72E-03	-2.17E-15	9.61E-02	0.00E+00	0.00E+00	-1.64E-02	-1.37E-15	-6.97E-17	-3.93E-03	-2.04E-02	-5.81E-03	-4.63E-04	-2.32E-01	-4.68E-02	2.55E-17
Min	-4.34E-02	-1.06E-02	-4.77E-01	1.83E-02	1.99E-01	-4.40E-15	2.27E-03	5.03E-03	3.80E-04	1.06E-03	-1.07E-14	4.44E-02	-7.22E-16	-5.37E-16	-3.50E-02	-2.41E-14	-1.64E-14	-7.59E-03	-3.94E-02	-1.07E-02	-1.47E-03	-3.09E-01	-7.33E-02	-1.33E-16
Max	-6.82E-05	-3.80E-05	-1.53E-02	3.74E-02	6.30E-01	5.86E-15	1.04E-02	2.25E-02	1.97E-03	6.04E-03	1.17E-14	2.36E-01	7.63E-16	6.57E-16	-8.38E-03	1.88E-14	1.60E-14	-2.09E-03	-1.18E-02	-3.43E-03	-1.52E-04	-1.91E-01	-3.59E-02	2.05E-15
Normalized peak height	3.01E-02	1.62E-02	4.33E-03	3.54E-03	4.22E-03	6.52E-02	4.22E-03	4.31E-03	4.37E-03	4.26E-03	2.03E-02	4.37E-03	9.23E-01	9.32E-01	4.40E-03	6.24E-03	7.42E-03	3.81E-03	4.42E-03	3.99E-03	4.82E-03	4.13E-03	3.86E-03	3.41E-01
Peak sensitivity value	-4.80E-04	-2.96E-04	-9.87E-02	2.51E-02	2.76E-01	3.04E-18	4.46E-03	9.23E-03	8.68E-04	2.44E-03	-1.11E-17	8.49E-02	2.75E-19	-2.62E-19	-1.52E-02	1.86E-16	-5.31E-17	-3.68E-03	-1.90E-02	-5.51E-03	-3.53E-04	-2.34E-01	-4.76E-02	1.03E-18
Shapiro-Wilk score	5.974E-01	7.298E-01	9.330E-01	9.879E-01	9.428E-01	9.941E-01	9.697E-01	9.707E-01	9.676E-01	9.699E-01	9.904E-01	9.489E-01	2.862E-01	2.798E-01	9.703E-01	9.947E-01	9.941E-01	9.748E-01	9.710E-01	9.783E-01	9.486E-01	9.913E-01	9.891E-01	7.728E-01
**NADH**																								
Local	9.35E-03	4.03E-03	7.30E-01	-1.86E-03	-2.25E-02	6.21E-15	1.54E-01	3.36E-01	2.88E-02	8.69E-02	1.30E-14	-1.28E+00	0.00E+00	0.00E+00	-5.23E-01	6.20E-15	-5.37E-15	5.25E-02	2.73E-01	7.75E-02	6.18E-03	1.86E-02	5.39E-02	-5.02E-18
Min	1.85E-03	9.23E-04	4.66E-01	-5.01E-03	-5.71E-02	-3.88E-14	1.07E-01	2.56E-01	1.77E-02	4.65E-02	-4.94E-14	-1.48E+00	-1.43E-15	-1.33E-15	-6.01E-01	-9.60E-14	-6.74E-14	3.27E-02	1.94E-01	4.74E-02	3.56E-03	-2.02E-03	3.65E-02	-1.43E-15
Max	1.09E-01	2.51E-02	9.86E-01	7.03E-04	1.07E-02	2.93E-14	2.15E-01	4.17E-01	4.22E-02	1.45E-01	4.82E-14	-1.03E+00	1.59E-15	1.50E-15	-4.34E-01	9.58E-14	7.04E-14	7.95E-02	3.46E-01	1.12E-01	9.62E-03	4.24E-02	7.45E-02	3.24E-17
Normalized peak height	1.40E-02	8.16E-03	3.59E-03	4.18E-03	4.05E-03	7.35E-03	3.34E-03	3.91E-03	3.57E-03	3.61E-03	7.21E-02	3.91E-03	9.23E-01	9.29E-01	3.86E-03	5.20E-03	1.43E-02	3.82E-03	3.14E-03	3.23E-03	3.52E-03	3.62E-03	3.50E-03	3.44E-01
Peak sensitivity value	4.69E-03	2.60E-03	7.11E-01	-1.71E-03	-2.00E-02	4.97E-16	1.47E-01	3.36E-01	2.79E-02	8.35E-02	2.59E-17	-1.26E+00	-1.05E-18	-7.87E-19	-5.29E-01	-2.56E-17	2.05E-17	5.42E-02	2.80E-01	8.04E-02	5.95E-03	1.76E-02	5.22E-02	-4.59E-19
Shapiro-Wilk score	6.933E-01	8.402E-01	9.971E-01	9.993E-01	9.992E-01	9.886E-01	9.974E-01	9.998E-01	9.981E-01	9.962E-01	9.894E-01	9.987E-01	2.901E-01	2.836E-01	9.975E-01	9.983E-01	9.961E-01	9.985E-01	9.954E-01	9.992E-01	9.928E-01	9.993E-01	9.968E-01	4.649E-01

Table S20: Concentration control coefficients for *Saccharomyces cerevisiae* model with parameter variation of ±20%

	(GlucoseMixedflowtoextracellularmedium)	(Glucoseuptake)	(Hexokinase)	(Phosphoglucoisomerase)	(Phosphofructokinase)	(Aldolase)	(Triosephosphateisomerase)	(Glyceraldehyde3-phosphatedehydrogenase)	(Phosphoenolpyruvatesynthesis)	(Pyruvatekinase)	(Pyruvatedecarboxylase)	(Alcoholdehydrogenase)
**Acetaldehyde**												
Local	2.55E-02	1.10E-02	1.99E+00	8.76E-03	1.06E-01	-5.75E-15	-8.30E-02	-1.82E-01	-1.56E-02	-4.70E-02	-7.51E-15	-1.66E+00
Min	1.89E-03	1.03E-03	7.65E-02	3.79E-03	4.37E-02	-3.88E-14	-2.03E-01	-3.26E-01	-3.17E-02	-1.21E-01	-1.28E-13	-2.46E+00
Max	2.38E+00	4.45E-01	2.75E+00	2.58E-02	9.35E-01	3.89E-14	-1.88E-02	-5.59E-02	-5.99E-03	-1.09E-02	1.20E-13	-1.14E+00
Normalized peak height	1.09E-01	3.96E-02	6.07E-03	5.19E-03	1.43E-02	1.59E-02	3.71E-03	3.20E-03	4.17E-03	4.10E-03	6.91E-02	3.12E-03
Peak sensitivity value	5.46E-03	4.36E-03	1.84E+00	8.00E-03	8.07E-02	-6.65E-17	-8.24E-02	-1.73E-01	-1.49E-02	-3.99E-02	-9.83E-17	-1.49E+00
Shapiro-Wilk score	3.543E-01	5.324E-01	8.219E-01	9.474E-01	7.008E-01	9.263E-01	9.881E-01	9.973E-01	9.877E-01	9.744E-01	9.174E-01	9.832E-01
**Cytosolic glucose**												
Local	2.04E+00	8.77E-01	-3.34E+00	7.30E-03	8.83E-02	4.39E-16	-2.81E-03	-6.15E-03	-5.28E-04	-1.59E-03	1.00E-15	-5.63E-02
Min	2.36E-01	2.91E-01	-3.40E+03	-1.94E-01	-2.06E+00	-9.22E-14	-2.68E+00	-4.76E+00	-3.79E-01	-1.12E+00	-3.38E-13	-5.28E+01
Max	2.62E+03	6.68E+02	-8.21E-01	3.15E-01	4.43E+00	1.73E-13	1.02E-02	2.56E-02	1.66E-03	3.79E-03	5.85E-14	3.21E-01
Normalized peak height	6.65E-01	5.96E-01	6.36E-01	6.24E-02	6.48E-02	3.30E-01	6.42E-01	5.75E-01	5.50E-01	5.14E-01	2.44E-01	5.06E-01
Peak sensitivity value	1.54E+00	6.25E-01	-2.52E+00	4.05E-03	4.93E-02	-7.13E-17	-1.88E-03	-5.54E-03	-4.36E-04	-1.25E-03	2.84E-18	-2.49E-02
Shapiro-Wilk score	6.712E-02	4.622E-02	5.881E-02	7.328E-01	6.492E-01	3.037E-01	4.856E-02	6.541E-02	7.703E-02	8.729E-02	3.704E-01	6.209E-02
**Glycerol**												
Local	2.51E-02	1.08E-02	1.96E+00	8.63E-03	1.04E-01	8.13E-15	-8.18E-02	-1.79E-01	-1.53E-02	-4.63E-02	1.53E-14	-1.64E+00
Min	1.80E-03	1.04E-03	1.08E-01	3.82E-03	4.56E-02	-7.44E-14	-1.88E-01	-3.37E-01	-3.02E-02	-1.15E-01	-1.79E-13	-2.46E+00
Max	2.10E+00	4.56E-01	2.63E+00	2.38E-02	1.04E+00	5.84E-14	-1.84E-02	-5.62E-02	-5.45E-03	-9.11E-03	1.34E-13	-1.12E+00
Normalized peak height	9.86E-02	3.98E-02	5.71E-03	4.80E-03	1.58E-02	1.42E-02	3.40E-03	3.41E-03	3.97E-03	3.89E-03	4.87E-02	3.24E-03
Peak sensitivity value	7.05E-03	4.00E-03	1.80E+00	8.27E-03	8.47E-02	4.25E-16	-7.78E-02	-1.83E-01	-1.43E-02	-3.89E-02	-2.99E-17	-1.57E+00
Shapiro-Wilk score	3.538E-01	5.370E-01	8.166E-01	9.478E-01	7.109E-01	9.422E-01	9.890E-01	9.977E-01	9.891E-01	9.750E-01	9.361E-01	9.848E-01
**EtOH**												
Local	1.01E-02	4.37E-03	7.92E-01	2.32E-03	2.81E-02	-3.86E-16	9.18E-03	2.01E-02	1.72E-03	5.20E-03	-7.43E-17	1.84E-01
Min	9.03E-04	5.01E-04	4.38E-02	9.81E-04	1.07E-02	-2.59E-15	2.59E-03	5.65E-03	4.24E-04	9.41E-04	-6.08E-15	6.19E-02
Max	7.29E-01	1.35E-01	9.03E-01	7.50E-03	2.74E-01	2.49E-15	2.39E-02	4.97E-02	4.71E-03	1.80E-02	8.44E-15	4.93E-01
Normalized peak height	8.29E-02	2.92E-02	7.31E-03	5.87E-03	1.50E-02	2.57E-02	3.92E-03	3.90E-03	4.02E-03	4.63E-03	8.15E-02	3.77E-03
Peak sensitivity value	2.72E-03	1.91E-03	8.36E-01	2.37E-03	2.43E-02	-1.29E-18	7.62E-03	1.60E-02	1.30E-03	4.23E-03	-9.25E-19	1.33E-01
Shapiro-Wilk score	3.609E-01	5.463E-01	6.956E-01	9.587E-01	7.575E-01	9.339E-01	9.712E-01	9.772E-01	9.618E-01	9.458E-01	9.794E-01	9.315E-01
**ADP**												
Local	1.29E-03	5.55E-04	1.01E-01	-2.09E-02	-2.53E-01	6.63E-16	-3.90E-03	-8.54E-03	-7.33E-04	-2.21E-03	1.57E-15	-7.81E-02
Min	-4.53E-04	-3.64E-04	-4.48E-02	-3.59E-02	-8.54E-01	-6.94E-15	-1.38E-02	-2.96E-02	-2.40E-03	-1.06E-02	-1.38E-14	-3.07E-01
Max	5.00E-01	9.18E-02	8.19E-01	-7.87E-03	-1.36E-01	6.14E-15	-8.05E-04	-1.89E-03	-1.60E-04	-2.97E-04	1.19E-14	-1.98E-02
Normalized peak height	3.54E-01	1.64E-01	6.58E-03	3.84E-03	7.37E-03	7.85E-02	5.09E-03	5.42E-03	4.71E-03	5.89E-03	5.65E-02	5.19E-03
Peak sensitivity value	2.97E-04	5.03E-05	6.18E-02	-1.80E-02	-2.15E-01	-1.94E-18	-3.21E-03	-6.78E-03	-5.60E-04	-1.56E-03	-3.97E-18	-5.55E-02
Shapiro-Wilk score	3.017E-01	4.172E-01	8.599E-01	9.860E-01	8.463E-01	9.480E-01	9.483E-01	9.581E-01	9.455E-01	9.302E-01	9.695E-01	9.076E-01
**Glyceraldehyde 3-phosphate**												
Local	2.26E-02	9.73E-03	1.76E+00	1.27E-02	1.54E-01	6.19E-15	1.56E-01	-5.55E-01	-4.76E-02	-1.44E-01	1.37E-14	-1.23E+00
Min	1.63E-03	9.83E-04	9.11E-02	5.21E-03	5.90E-02	-3.56E-14	5.10E-02	-8.73E-01	-8.80E-02	-3.40E-01	-8.29E-14	-2.54E+00
Max	2.15E+00	3.91E-01	2.95E+00	3.03E-02	1.21E+00	4.33E-14	3.41E-01	-2.30E-01	-2.08E-02	-3.95E-02	7.80E-14	-4.05E-01
Normalized peak height	1.12E-01	3.87E-02	5.90E-03	3.95E-03	1.25E-02	1.52E-02	3.89E-03	3.03E-03	3.74E-03	3.66E-03	5.92E-02	3.24E-03
Peak sensitivity value	4.85E-03	3.91E-03	1.49E+00	1.05E-02	1.20E-01	-3.61E-17	1.40E-01	-5.67E-01	-4.34E-02	-1.24E-01	2.39E-17	-9.71E-01
Shapiro-Wilk score	3.554E-01	5.339E-01	9.133E-01	9.654E-01	7.451E-01	9.453E-01	9.906E-01	9.983E-01	9.904E-01	9.782E-01	9.585E-01	9.759E-01
**Dihydroxyacetone phosphate**												
Local	2.40E-02	1.03E-02	1.88E+00	1.23E-02	1.49E-01	5.18E-15	-2.24E-01	-4.91E-01	-4.21E-02	-1.27E-01	1.04E-14	-1.03E+00
Min	1.83E-03	1.08E-03	8.57E-02	5.16E-03	5.61E-02	-3.48E-14	-4.64E-01	-7.90E-01	-7.80E-02	-3.04E-01	-7.80E-14	-2.38E+00
Max	2.24E+00	3.62E-01	2.97E+00	3.02E-02	1.63E+00	3.67E-14	-5.64E-02	-1.80E-01	-1.89E-02	-3.08E-02	7.89E-14	-2.97E-01
Normalized peak height	1.11E-01	3.40E-02	6.65E-03	4.07E-03	1.72E-02	1.44E-02	3.34E-03	3.46E-03	3.72E-03	3.92E-03	5.13E-02	3.46E-03
Peak sensitivity value	5.18E-03	3.78E-03	1.73E+00	1.21E-02	1.20E-01	-2.65E-17	-2.18E-01	-5.03E-01	-4.24E-02	-1.11E-01	6.06E-17	-8.49E-01
Shapiro-Wilk score	3.524E-01	5.374E-01	8.780E-01	9.623E-01	7.405E-01	9.499E-01	9.925E-01	9.991E-01	9.898E-01	9.761E-01	9.583E-01	9.720E-01
**Glucose-6-Phosphate**												
Local	1.37E-02	5.88E-03	1.07E+00	-2.72E-02	-3.29E-01	-8.33E-16	5.10E-03	1.11E-02	9.57E-04	2.89E-03	-1.82E-15	1.02E-01
Min	1.04E-03	6.07E-04	5.30E-02	-6.45E-02	-3.30E+00	-8.65E-15	-2.24E-02	-5.01E-02	-4.45E-03	-8.71E-03	-1.66E-14	-7.10E-01
Max	1.63E+00	2.80E-01	4.07E+00	-1.44E-02	-1.65E-01	8.70E-15	1.29E-02	2.78E-02	3.05E-03	1.10E-02	2.63E-14	2.94E-01
Normalized peak height	1.34E-01	4.52E-02	2.86E-02	4.74E-03	1.70E-02	8.82E-02	9.85E-03	1.01E-02	1.00E-02	7.84E-03	4.60E-02	1.35E-02
Peak sensitivity value	3.49E-03	2.42E-03	1.02E+00	-2.48E-02	-2.48E-01	-2.66E-18	4.27E-03	1.01E-02	7.39E-04	2.10E-03	-7.63E-18	7.44E-02
Shapiro-Wilk score	3.528E-01	5.074E-01	7.278E-01	9.647E-01	7.317E-01	9.364E-01	9.576E-01	9.482E-01	9.603E-01	9.621E-01	9.624E-01	9.315E-01
**NAD**												
Local	-4.87E-03	-2.10E-03	-3.80E-01	9.66E-04	1.17E-02	-3.24E-15	-8.00E-02	-1.75E-01	-1.50E-02	-4.53E-02	-6.78E-15	6.68E-01
Min	-7.19E-01	-1.37E-01	-1.10E+00	-3.91E-03	-2.09E-01	-2.27E-14	-1.98E-01	-5.56E-01	-6.85E-02	-2.08E-01	-5.39E-14	1.83E-01
Max	-1.89E-04	-8.61E-05	-1.53E-02	9.21E-03	1.49E-01	1.91E-14	-2.05E-02	-5.10E-02	-1.85E-03	-3.47E-03	3.92E-14	1.67E+00
Normalized peak height	1.72E-01	6.73E-02	4.32E-03	8.43E-03	1.78E-02	1.29E-02	3.75E-03	4.68E-03	4.97E-03	5.16E-03	8.73E-02	3.50E-03
Peak sensitivity value	-1.27E-03	-5.67E-04	-2.40E-01	5.54E-04	6.06E-03	-3.47E-17	-6.46E-02	-1.56E-01	-1.25E-02	-3.63E-02	2.66E-17	5.09E-01
Shapiro-Wilk score	3.348E-01	5.024E-01	9.452E-01	9.462E-01	8.955E-01	9.384E-01	9.776E-01	9.626E-01	9.290E-01	9.293E-01	9.635E-01	9.586E-01
**Extracellular acetaldehyde**												
Local	2.55E-02	1.10E-02	1.99E+00	8.78E-03	1.06E-01	-5.73E-15	-8.32E-02	-1.82E-01	-1.56E-02	-4.71E-02	-8.63E-15	-1.67E+00
Min	1.90E-03	1.08E-03	9.64E-02	3.87E-03	4.35E-02	-3.21E-14	-1.94E-01	-3.31E-01	-3.12E-02	-1.19E-01	-1.05E-13	-2.43E+00
Max	2.24E+00	3.57E-01	2.75E+00	2.56E-02	8.95E-01	4.36E-14	-2.02E-02	-5.93E-02	-5.40E-03	-1.16E-02	8.47E-14	-1.12E+00
Normalized peak height	1.02E-01	3.26E-02	5.88E-03	5.00E-03	1.34E-02	1.64E-02	3.51E-03	3.26E-03	3.97E-03	3.82E-03	7.08E-02	3.26E-03
Peak sensitivity value	7.50E-03	4.82E-03	1.81E+00	8.03E-03	8.48E-02	-3.35E-17	-7.27E-02	-1.71E-01	-1.45E-02	-4.10E-02	-7.65E-17	-1.62E+00
Shapiro-Wilk score	3.548E-01	5.373E-01	8.263E-01	9.482E-01	7.145E-01	9.310E-01	9.882E-01	9.975E-01	9.881E-01	9.754E-01	9.274E-01	9.843E-01
**Phosphoenolpyruvate**												
Local	1.38E-02	5.93E-03	1.08E+00	6.08E-03	7.36E-02	2.62E-15	1.11E-03	2.42E-03	2.08E-04	-1.20E+00	7.14E-14	2.22E-02
Min	1.17E-03	5.28E-04	5.56E-02	3.13E-03	3.97E-02	-3.25E-14	2.68E-04	5.95E-04	5.41E-05	-1.33E+00	-2.94E-13	6.83E-03
Max	1.04E+00	2.25E-01	1.19E+00	1.30E-02	5.63E-01	3.85E-14	5.77E-03	1.50E-02	1.28E-03	-1.12E+00	3.33E-13	1.91E-01
Normalized peak height	8.63E-02	3.59E-02	1.25E-02	4.36E-03	1.44E-02	5.62E-02	7.21E-03	9.11E-03	8.13E-03	2.74E-03	2.14E-01	1.09E-02
Peak sensitivity value	3.77E-03	2.66E-03	1.05E+00	5.49E-03	6.19E-02	-2.70E-17	9.31E-04	2.08E-03	1.69E-04	-1.18E+00	-2.78E-16	1.54E-02
Shapiro-Wilk score	3.590E-01	5.423E-01	5.182E-01	9.749E-01	7.423E-01	9.610E-01	9.012E-01	8.908E-01	8.956E-01	9.723E-01	9.328E-01	8.359E-01

	(Ethanolout)	(Ethanolflow)	(Glycerolsynthesis)	(Glycerolout)	(Glycerolflow)	(Acetaldehydeout)	(Acetaldehydeflow)	(Cyanide-Acetaldehydeflow)	(Cyanideflow)	(Storage)	(ATPconsumption)	(Adenylatekinase)
**Acetaldehyde**												
Local	0.00E+00	0.00E+00	2.83E-01	-5.12E-15	7.96E-15	-6.23E-02	-3.24E-01	-9.20E-02	-7.34E-03	-7.38E-02	1.26E-01	1.05E-17
Min	-3.75E-14	-1.65E-13	8.07E-02	-1.17E-13	-1.03E-13	-1.31E-01	-5.27E-01	-1.63E-01	-3.38E-02	-1.33E-01	5.04E-02	-2.17E-16
Max	2.13E-14	5.88E-14	4.82E-01	1.02E-13	7.67E-14	-2.38E-02	-1.59E-01	-4.27E-02	-1.24E-03	1.49E-01	2.09E-01	3.51E-16
Normalized peak height	6.51E-01	7.16E-01	3.27E-03	9.23E-03	2.14E-02	3.73E-03	3.47E-03	3.58E-03	5.32E-03	1.23E-02	3.62E-03	3.14E-01
Peak sensitivity value	2.18E-17	2.65E-17	2.80E-01	7.94E-17	5.98E-17	-5.95E-02	-3.17E-01	-9.09E-02	-5.23E-03	-7.12E-02	1.27E-01	-1.65E-19
Shapiro-Wilk score	3.432E-01	5.713E-02	9.988E-01	9.879E-01	9.797E-01	9.846E-01	9.925E-01	9.927E-01	9.004E-01	8.174E-01	9.974E-01	9.139E-01
**Cytosolic glucose**												
Local	0.00E+00	0.00E+00	9.58E-03	7.63E-16	4.37E-17	2.30E-03	1.20E-02	3.40E-03	2.71E-04	3.43E-01	2.74E-02	7.34E-18
Min	-1.04E-14	-1.14E-14	-4.05E-02	-9.05E-13	-6.58E-13	-9.84E-03	-4.53E-02	-1.24E-02	-2.14E-03	1.00E-01	-5.40E-02	-6.79E-16
Max	7.52E-15	1.28E-14	7.67E+00	1.99E-13	9.19E-14	1.18E+00	7.60E+00	1.66E+00	1.71E-01	1.44E+02	1.83E+01	8.23E-16
Normalized peak height	6.74E-01	7.13E-01	5.29E-01	2.61E-01	2.62E-01	4.21E-01	4.58E-01	3.63E-01	4.43E-01	3.85E-01	5.53E-01	3.26E-01
Peak sensitivity value	-6.37E-18	4.00E-18	9.64E-03	-1.61E-16	-2.09E-17	1.45E-03	1.20E-02	3.46E-03	1.93E-04	3.17E-01	2.86E-02	7.37E-19
Shapiro-Wilk score	2.978E-01	1.655E-01	6.353E-02	3.270E-01	3.132E-01	1.170E-01	8.304E-02	1.133E-01	1.375E-01	1.030E-01	6.345E-02	6.793E-01
**Glycerol**												
Local	0.00E+00	0.00E+00	2.79E-01	-5.98E-01	-4.02E-01	6.70E-02	3.48E-01	9.89E-02	7.89E-03	-7.27E-02	1.24E-01	1.03E-17
Min	-3.68E-14	-2.06E-13	8.17E-02	-7.25E-01	-5.52E-01	2.15E-02	1.67E-01	3.90E-02	2.30E-03	-1.46E-01	4.81E-02	-6.15E-17
Max	8.52E-14	3.74E-13	4.92E-01	-4.48E-01	-2.75E-01	1.39E-01	5.03E-01	1.87E-01	1.97E-02	1.86E-01	2.04E-01	5.94E-16
Normalized peak height	6.63E-01	7.76E-01	3.34E-03	2.58E-03	2.58E-03	3.74E-03	3.43E-03	3.76E-03	3.66E-03	1.43E-02	3.72E-03	3.17E-01
Peak sensitivity value	-3.37E-17	1.32E-16	2.84E-01	-6.23E-01	-3.77E-01	6.42E-02	3.62E-01	9.69E-02	6.45E-03	-6.95E-02	1.16E-01	-2.01E-19
Shapiro-Wilk score	1.657E-01	3.508E-02	9.989E-01	9.962E-01	9.962E-01	9.914E-01	9.988E-01	9.977E-01	9.689E-01	8.218E-01	9.970E-01	9.069E-01
**EtOH**												
Local	-1.45E-01	-8.55E-01	-3.13E-02	-1.63E-16	1.29E-15	-7.52E-03	-3.91E-02	-1.11E-02	-8.85E-04	-1.89E-02	5.21E-02	2.13E-18
Min	-2.30E-01	-9.15E-01	-8.25E-02	-1.12E-14	-8.78E-15	-1.79E-02	-7.36E-02	-1.98E-02	-4.45E-03	-4.23E-02	1.53E-02	-5.37E-17
Max	-8.49E-02	-7.70E-01	-9.14E-03	1.08E-14	6.98E-15	-2.79E-03	-1.86E-02	-5.42E-03	-1.24E-04	4.96E-02	9.10E-02	8.36E-17
Normalized peak height	2.76E-03	2.76E-03	4.10E-03	8.67E-03	1.41E-02	3.45E-03	2.99E-03	3.01E-03	5.58E-03	8.97E-03	3.20E-03	3.15E-01
Peak sensitivity value	-1.53E-01	-8.47E-01	-2.61E-02	-7.11E-18	2.49E-18	-6.89E-03	-3.29E-02	-1.01E-02	-5.63E-04	-2.02E-02	5.27E-02	2.50E-20
Shapiro-Wilk score	9.905E-01	9.905E-01	9.740E-01	9.820E-01	9.758E-01	9.741E-01	9.816E-01	9.881E-01	9.078E-01	8.662E-01	9.990E-01	9.182E-01
**ADP**												
Local	0.00E+00	0.00E+00	1.33E-02	1.09E-15	5.21E-17	3.20E-03	1.66E-02	4.72E-03	3.76E-04	1.88E-01	3.81E-02	-4.97E-17
Min	-4.07E-15	-8.24E-15	2.77E-03	-2.60E-14	-1.97E-14	9.88E-04	6.22E-03	1.85E-03	5.08E-05	5.99E-02	1.78E-02	-2.91E-15
Max	2.24E-15	1.84E-14	4.21E-02	2.66E-14	1.77E-14	9.47E-03	4.31E-02	1.14E-02	1.97E-03	2.61E-01	8.10E-02	6.09E-16
Normalized peak height	6.32E-01	6.99E-01	4.64E-03	9.54E-03	1.17E-02	4.50E-03	4.47E-03	4.41E-03	5.63E-03	4.73E-03	3.78E-03	3.17E-01
Peak sensitivity value	3.16E-18	-3.67E-18	1.12E-02	1.78E-16	-1.75E-17	2.73E-03	1.49E-02	4.48E-03	2.19E-04	1.90E-01	3.90E-02	1.27E-18
Shapiro-Wilk score	6.218E-01	1.836E-01	9.558E-01	9.702E-01	9.609E-01	9.628E-01	9.673E-01	9.757E-01	8.836E-01	9.962E-01	9.884E-01	9.007E-01
**Glyceraldehyde 3-phosphate**												
Local	0.00E+00	0.00E+00	-5.32E-01	7.05E-15	-4.91E-15	5.03E-02	2.61E-01	7.42E-02	5.92E-03	-1.10E-01	1.05E-01	1.80E-17
Min	-4.71E-14	-9.10E-14	-1.03E+00	-2.75E-13	-1.63E-13	1.42E-02	9.65E-02	2.73E-02	1.14E-03	-1.94E-01	3.54E-02	-3.29E-16
Max	2.19E-14	1.45E-13	-1.90E-01	2.03E-13	1.38E-13	1.18E-01	4.68E-01	1.64E-01	1.92E-02	9.44E-02	1.98E-01	5.13E-16
Normalized peak height	6.65E-01	7.16E-01	3.43E-03	1.47E-02	3.08E-02	3.68E-03	3.43E-03	3.91E-03	4.02E-03	7.66E-03	4.10E-03	3.15E-01
Peak sensitivity value	-2.70E-17	1.01E-16	-4.74E-01	-2.16E-16	-6.18E-17	4.44E-02	2.33E-01	6.64E-02	3.95E-03	-1.09E-01	1.03E-01	-1.57E-19
Shapiro-Wilk score	2.671E-01	4.691E-02	9.902E-01	9.673E-01	9.569E-01	9.824E-01	9.953E-01	9.869E-01	9.457E-01	9.595E-01	9.895E-01	9.302E-01
**Dihydroxyacetone phosphate**												
Local	0.00E+00	0.00E+00	-4.89E-01	6.32E-15	-3.90E-15	4.23E-02	2.20E-01	6.25E-02	4.99E-03	-1.06E-01	1.13E-01	1.69E-17
Min	-4.67E-14	-9.36E-14	-9.60E-01	-1.36E-13	-1.82E-13	1.21E-02	7.05E-02	2.01E-02	8.25E-04	-1.85E-01	2.85E-02	-2.97E-16
Max	2.61E-14	2.18E-13	-1.76E-01	2.27E-13	1.33E-13	1.01E-01	4.23E-01	1.49E-01	2.11E-02	9.81E-02	2.00E-01	6.78E-16
Normalized peak height	6.86E-01	7.55E-01	3.23E-03	1.69E-02	3.95E-02	3.78E-03	3.50E-03	3.99E-03	5.12E-03	7.93E-03	4.22E-03	3.19E-01
Peak sensitivity value	-6.11E-18	8.24E-17	-4.37E-01	-3.59E-17	-3.94E-17	4.05E-02	2.16E-01	5.68E-02	3.98E-03	-1.03E-01	1.06E-01	2.60E-19
Shapiro-Wilk score	2.609E-01	4.651E-02	9.847E-01	9.664E-01	9.489E-01	9.811E-01	9.944E-01	9.867E-01	9.372E-01	9.394E-01	9.942E-01	9.052E-01
**Glucose-6-Phosphate**												
Local	0.00E+00	0.00E+00	-1.74E-02	-1.37E-15	-6.86E-17	-4.17E-03	-2.17E-02	-6.16E-03	-4.92E-04	-7.52E-01	-4.97E-02	-2.72E-17
Min	-7.90E-15	-1.63E-14	-5.04E-02	-4.82E-14	-2.93E-14	-1.09E-02	-4.64E-02	-1.44E-02	-2.19E-03	-8.20E-01	-1.28E-01	-1.80E-15
Max	3.07E-15	2.05E-14	7.51E-02	2.91E-14	2.83E-14	2.11E-02	9.64E-02	2.15E-02	4.14E-03	-4.70E-01	1.12E-01	3.70E-16
Normalized peak height	6.47E-01	7.09E-01	9.85E-03	1.23E-02	1.49E-02	1.02E-02	1.03E-02	9.13E-03	1.42E-02	5.48E-03	6.22E-03	3.14E-01
Peak sensitivity value	-4.57E-18	-6.57E-18	-1.46E-02	-3.11E-16	4.62E-19	-3.69E-03	-1.83E-02	-5.46E-03	-2.83E-04	-7.56E-01	-4.57E-02	-1.52E-19
Shapiro-Wilk score	6.118E-01	1.696E-01	9.557E-01	9.729E-01	9.612E-01	9.621E-01	9.423E-01	9.498E-01	9.184E-01	9.484E-01	9.804E-01	8.484E-01
**NAD**												
Local	0.00E+00	0.00E+00	2.72E-01	-3.23E-15	2.80E-15	-2.73E-02	-1.42E-01	-4.03E-02	-3.22E-03	-9.68E-03	-2.81E-02	2.62E-18
Min	-2.50E-14	-5.44E-14	8.05E-02	-1.08E-13	-8.71E-14	-8.16E-02	-3.23E-01	-1.11E-01	-1.42E-02	-8.79E-02	-8.56E-02	-6.94E-17
Max	2.22E-14	8.32E-14	6.31E-01	1.18E-13	8.90E-14	-6.24E-03	-4.27E-02	-1.09E-02	-5.19E-04	2.21E-02	-6.82E-03	1.76E-16
Normalized peak height	6.63E-01	7.48E-01	3.86E-03	1.32E-02	2.80E-02	4.17E-03	3.65E-03	4.40E-03	5.10E-03	6.95E-03	3.96E-03	3.17E-01
Peak sensitivity value	1.99E-17	8.93E-18	2.37E-01	1.94E-17	-9.55E-18	-2.44E-02	-1.21E-01	-3.67E-02	-1.81E-03	-5.46E-03	-2.17E-02	4.35E-20
Shapiro-Wilk score	1.675E-01	4.099E-02	9.755E-01	9.541E-01	9.375E-01	9.688E-01	9.830E-01	9.756E-01	9.245E-01	9.317E-01	9.490E-01	7.986E-01
**Extracellular acetaldehyde**												
Local	0.00E+00	0.00E+00	2.84E-01	-5.10E-15	7.94E-15	6.82E-02	-4.25E-01	-1.21E-01	-9.62E-03	-7.39E-02	1.27E-01	1.05E-17
Min	-2.69E-14	-8.64E-14	8.60E-02	-1.19E-13	-8.55E-14	2.23E-02	-6.43E-01	-1.92E-01	-4.98E-02	-1.37E-01	4.38E-02	-2.94E-16
Max	4.70E-14	1.56E-13	5.04E-01	1.07E-13	9.73E-14	1.41E-01	-2.44E-01	-6.67E-02	-1.99E-03	1.15E-01	2.13E-01	4.05E-16
Normalized peak height	6.59E-01	7.27E-01	3.45E-03	8.66E-03	2.05E-02	3.91E-03	3.76E-03	3.22E-03	5.93E-03	1.07E-02	3.93E-03	3.15E-01
Peak sensitivity value	1.87E-18	-9.28E-17	2.86E-01	1.82E-17	-6.57E-17	6.54E-02	-4.26E-01	-1.24E-01	-6.56E-03	-7.14E-02	1.21E-01	8.29E-20
Shapiro-Wilk score	3.311E-01	6.364E-02	9.989E-01	9.890E-01	9.790E-01	9.924E-01	9.947E-01	9.946E-01	9.112E-01	8.258E-01	9.966E-01	9.141E-01
**Phosphoenolpyruvate**												
Local	0.00E+00	0.00E+00	-3.77E-03	4.71E-15	-1.00E-15	-9.07E-04	-4.72E-03	-1.34E-03	-1.07E-04	-5.20E-02	6.64E-02	9.58E-18
Min	-2.65E-14	-1.37E-14	-2.06E-02	-2.52E-14	-2.00E-14	-5.22E-03	-2.66E-02	-5.76E-03	-1.06E-03	-7.69E-02	2.15E-02	-2.01E-16
Max	1.93E-14	1.67E-14	-1.06E-03	3.21E-14	2.67E-14	-2.61E-04	-1.44E-03	-4.14E-04	-1.60E-05	6.90E-02	1.09E-01	2.30E-15
Normalized peak height	6.86E-01	7.10E-01	7.52E-03	8.11E-03	1.57E-02	8.19E-03	9.22E-03	7.48E-03	1.03E-02	1.20E-02	3.50E-03	3.16E-01
Peak sensitivity value	1.79E-17	-8.83E-18	-3.05E-03	6.22E-18	6.94E-18	-7.64E-04	-4.19E-03	-1.29E-03	-6.46E-05	-5.16E-02	6.75E-02	3.22E-19
Shapiro-Wilk score	1.750E-01	2.312E-01	8.970E-01	9.796E-01	9.682E-01	9.072E-01	8.926E-01	9.150E-01	8.325E-01	7.700E-01	9.990E-01	5.671E-01

**Fructose-6-Phosphate**												
Local	1.24E-02	5.33E-03	9.67E-01	8.44E-02	-3.38E-01	-8.95E-16	5.90E-03	1.29E-02	1.11E-03	3.34E-03	-2.13E-15	1.18E-01
Min	1.05E-03	6.39E-04	3.92E-02	4.55E-02	-3.48E+00	-7.68E-15	-2.45E-02	-6.01E-02	-3.99E-03	-1.02E-02	-1.62E-14	-6.50E-01
Max	1.58E+00	2.75E-01	4.40E+00	1.43E-01	-1.61E-01	7.75E-15	1.46E-02	3.13E-02	3.34E-03	1.53E-02	1.59E-14	3.21E-01
Normalized peak height	1.45E-01	4.80E-02	2.86E-02	3.62E-03	1.72E-02	8.81E-02	9.47E-03	1.07E-02	9.07E-03	8.72E-03	3.57E-02	1.17E-02
Peak sensitivity value	3.41E-03	2.42E-03	9.39E-01	7.54E-02	-2.72E-01	-3.38E-18	4.88E-03	1.08E-02	8.13E-04	2.34E-03	1.05E-17	8.49E-02
Shapiro-Wilk score	3.486E-01	5.128E-01	7.394E-01	9.848E-01	7.167E-01	9.439E-01	9.662E-01	9.553E-01	9.610E-01	9.568E-01	9.690E-01	9.332E-01
**Extracellular cyanide**												
Local	-1.89E-03	-8.11E-04	-1.47E-01	-6.48E-04	-7.85E-03	4.27E-16	6.15E-03	1.34E-02	1.15E-03	3.48E-03	6.32E-16	1.23E-01
Min	-3.19E-01	-4.55E-02	-5.01E-01	-3.75E-03	-1.43E-01	-3.51E-15	1.27E-03	3.16E-03	2.29E-04	5.24E-04	-1.00E-14	2.98E-02
Max	-6.40E-05	-3.76E-05	-5.27E-03	-1.19E-04	-1.39E-03	3.47E-15	2.03E-02	4.03E-02	3.78E-03	1.40E-02	8.90E-15	4.52E-01
Normalized peak height	1.85E-01	5.64E-02	5.13E-03	6.87E-03	1.99E-02	2.08E-02	4.77E-03	4.10E-03	4.69E-03	5.25E-03	7.26E-02	4.94E-03
Peak sensitivity value	-5.42E-04	-2.42E-04	-9.87E-02	-4.76E-04	-4.30E-03	1.20E-17	4.93E-03	1.06E-02	7.95E-04	2.17E-03	-5.60E-18	8.53E-02
Shapiro-Wilk score	3.317E-01	4.999E-01	9.371E-01	8.738E-01	6.697E-01	9.083E-01	9.489E-01	9.616E-01	9.469E-01	9.279E-01	9.460E-01	9.220E-01
**Pyruvate**												
Local	9.01E-01	3.88E-01	7.04E+01	2.28E-01	2.76E+00	-6.96E-14	4.07E-02	8.91E-02	7.64E-03	2.31E-02	-7.79E+01	8.15E-01
Min	3.63E-03	2.07E-03	3.13E-01	5.23E-03	5.91E-02	-2.59E-11	-4.22E-03	-9.14E-03	-8.88E-04	-2.37E-03	-1.21E+05	-9.05E-02
Max	4.60E+04	6.80E+03	5.41E+04	4.68E+02	6.33E+03	7.31E-11	3.49E+01	7.40E+01	6.77E+00	1.98E+01	-2.90E+00	1.04E+03
Normalized peak height	9.96E-01	9.95E-01	9.40E-01	9.77E-01	9.76E-01	7.56E-01	9.21E-01	9.17E-01	9.24E-01	9.23E-01	9.73E-01	9.48E-01
Peak sensitivity value	2.30E+01	3.40E+00	2.74E+01	2.39E-01	3.23E+00	-4.73E-14	1.32E-02	2.78E-02	2.50E-03	7.53E-03	-6.36E+01	4.31E-01
Shapiro-Wilk score	2.183E-03	2.340E-03	1.622E-02	8.631E-03	1.081E-02	8.522E-03	2.106E-02	2.117E-02	1.819E-02	1.877E-02	1.004E-02	1.628E-02
**Extracellular ethanol**												
Local	1.01E-02	4.37E-03	7.92E-01	2.32E-03	2.81E-02	-3.81E-16	9.18E-03	2.01E-02	1.72E-03	5.20E-03	-1.74E-16	1.84E-01
Min	9.26E-04	5.27E-04	3.24E-02	8.97E-04	9.11E-03	-3.38E-15	2.24E-03	5.86E-03	4.56E-04	9.97E-04	-7.31E-15	6.64E-02
Max	7.32E-01	1.67E-01	9.01E-01	6.43E-03	2.47E-01	2.71E-15	2.30E-02	4.95E-02	5.12E-03	1.66E-02	6.80E-15	4.90E-01
Normalized peak height	8.40E-02	3.55E-02	7.17E-03	4.90E-03	1.38E-02	2.53E-02	3.99E-03	4.12E-03	4.22E-03	4.30E-03	7.99E-02	3.42E-03
Peak sensitivity value	2.75E-03	2.27E-03	8.41E-01	2.28E-03	2.30E-02	-1.01E-19	7.50E-03	1.74E-02	1.34E-03	3.84E-03	6.87E-18	1.48E-01
Shapiro-Wilk score	3.645E-01	5.465E-01	6.967E-01	9.587E-01	7.655E-01	9.302E-01	9.709E-01	9.753E-01	9.608E-01	9.453E-01	9.812E-01	9.321E-01
**Extracellular glycerol**												
Local	2.51E-02	1.08E-02	1.96E+00	8.63E-03	1.04E-01	8.14E-15	-8.18E-02	-1.79E-01	-1.53E-02	-4.63E-02	1.49E-14	-1.64E+00
Min	1.81E-03	1.07E-03	1.01E-01	3.99E-03	4.49E-02	-6.45E-14	-1.94E-01	-3.27E-01	-3.26E-02	-1.27E-01	-1.31E-13	-2.38E+00
Max	2.13E+00	3.88E-01	2.77E+00	2.23E-02	9.26E-01	6.23E-14	-1.96E-02	-5.43E-02	-5.37E-03	-1.06E-02	1.31E-13	-1.12E+00
Normalized peak height	9.71E-02	3.42E-02	6.21E-03	4.32E-03	1.42E-02	1.35E-02	3.69E-03	3.31E-03	4.13E-03	4.11E-03	5.03E-02	3.19E-03
Peak sensitivity value	7.12E-03	4.35E-03	1.80E+00	7.77E-03	8.59E-02	2.28E-16	-7.60E-02	-1.80E-01	-1.39E-02	-4.01E-02	8.00E-18	-1.55E+00
Shapiro-Wilk score	3.600E-01	5.433E-01	8.122E-01	9.504E-01	7.179E-01	9.427E-01	9.880E-01	9.976E-01	9.886E-01	9.744E-01	9.421E-01	9.843E-01
**Extracellular glucose**												
Local	2.57E+00	-1.40E-02	-2.53E+00	-3.55E-03	-4.30E-02	8.97E-17	-5.98E-04	-1.31E-03	-1.12E-04	-3.38E-04	2.12E-16	-1.20E-02
Min	9.07E-01	-1.43E+00	-1.19E+01	-6.16E-02	-1.80E+00	-6.78E-15	-2.28E-02	-5.44E-02	-4.72E-03	-9.00E-03	-8.51E-15	-5.20E-01
Max	1.29E+01	-7.09E-04	-7.47E-01	-6.02E-04	-6.62E-03	4.74E-15	4.66E-03	9.31E-03	9.34E-04	3.05E-03	1.02E-14	1.15E-01
Normalized peak height	6.24E-03	1.08E-01	6.37E-03	1.92E-02	4.32E-02	1.15E-01	4.77E-02	4.86E-02	5.11E-02	3.64E-02	8.41E-02	5.46E-02
Peak sensitivity value	1.46E+00	-2.85E-03	-1.46E+00	-1.55E-03	-1.83E-02	-2.99E-18	-2.30E-04	-5.90E-04	-4.73E-05	-1.35E-04	-6.36E-18	-4.68E-03
Shapiro-Wilk score	8.183E-01	3.491E-01	8.269E-01	6.036E-01	5.138E-01	5.726E-01	6.212E-01	6.052E-01	6.054E-01	6.398E-01	6.143E-01	5.614E-01
**1-3-Bisphosphoglycerate**												
Local	1.07E-02	4.61E-03	8.37E-01	4.44E-02	5.38E-01	5.30E-16	8.28E-03	1.81E-02	-2.84E-01	-8.56E-01	3.50E-14	1.66E-01
Min	-5.91E-01	-8.56E-02	-1.88E+00	2.49E-02	3.08E-01	-1.72E-14	2.02E-03	4.93E-03	-4.81E-01	-1.09E+00	-1.50E-13	5.20E-02
Max	8.72E-01	1.88E-01	1.13E+00	7.71E-02	2.76E+00	3.14E-14	3.48E-02	7.63E-02	-1.57E-01	-5.87E-01	1.23E-13	9.44E-01
Normalized peak height	1.52E-01	5.63E-02	1.00E-02	3.71E-03	1.02E-02	5.32E-02	6.04E-03	6.10E-03	2.96E-03	2.98E-03	1.83E-02	7.16E-03
Peak sensitivity value	3.89E-03	2.06E-03	9.48E-01	4.06E-02	4.32E-01	5.60E-18	6.56E-03	1.44E-02	-3.01E-01	-8.73E-01	4.16E-18	1.21E-01
Shapiro-Wilk score	3.602E-01	5.768E-01	8.135E-01	9.863E-01	7.751E-01	9.718E-01	9.162E-01	9.184E-01	9.940E-01	9.983E-01	9.777E-01	8.657E-01
**Fructose 1-6-bisphosphate**												
Local	4.60E-02	1.98E-02	3.59E+00	2.43E-02	2.94E-01	-9.76E-02	-8.09E-02	-1.00E+00	-8.59E-02	-2.59E-01	2.20E-14	-2.18E+00
Min	3.38E-03	1.97E-03	1.88E-01	9.00E-03	1.14E-01	-2.31E-01	-3.51E-01	-1.56E+00	-1.64E-01	-6.19E-01	-1.50E-13	-4.78E+00
Max	4.54E+00	7.72E-01	5.90E+00	6.50E-02	2.37E+00	-4.00E-02	1.50E-01	-4.21E-01	-3.93E-02	-6.95E-02	1.46E-13	-7.48E-01
Normalized peak height	1.16E-01	3.78E-02	6.52E-03	4.68E-03	1.25E-02	3.82E-03	3.23E-03	3.23E-03	3.80E-03	3.80E-03	4.63E-02	3.33E-03
Peak sensitivity value	1.02E-02	8.52E-03	3.23E+00	2.22E-02	2.30E-01	-9.25E-02	-8.63E-02	-9.52E-01	-8.13E-02	-2.35E-01	4.82E-17	-1.81E+00
Shapiro-Wilk score	3.599E-01	5.413E-01	8.993E-01	9.604E-01	7.349E-01	9.795E-01	9.991E-01	9.988E-01	9.900E-01	9.774E-01	9.621E-01	9.717E-01
**AMP**												
Local	4.17E-03	1.79E-03	3.25E-01	-6.74E-02	-8.16E-01	2.40E-15	-1.26E-02	-2.76E-02	-2.37E-03	-7.14E-03	6.43E-15	-2.52E-01
Min	-1.38E-03	-9.82E-04	-1.16E-01	-1.17E-01	-4.09E+00	-2.37E-14	-5.48E-02	-1.14E-01	-1.44E-02	-4.29E-02	-3.61E-14	-1.44E+00
Max	2.23E+00	2.26E-01	4.49E+00	-3.96E-02	-5.06E-01	3.40E-14	-3.14E-03	-7.71E-03	-5.89E-04	-1.29E-03	8.30E-14	-7.73E-02
Normalized peak height	3.09E-01	1.18E-01	1.02E-02	3.66E-03	9.99E-03	8.20E-02	6.28E-03	6.62E-03	8.06E-03	7.64E-03	6.92E-02	7.34E-03
Peak sensitivity value	-2.65E-04	2.69E-04	1.22E-01	-6.38E-02	-6.40E-01	-5.11E-18	-1.08E-02	-2.31E-02	-1.84E-03	-5.55E-03	5.87E-17	-1.83E-01
Shapiro-Wilk score	2.862E-01	3.961E-01	8.033E-01	9.867E-01	7.766E-01	9.414E-01	9.230E-01	9.223E-01	9.166E-01	9.189E-01	9.593E-01	8.674E-01
**ATP**												
Local	-1.59E-03	-6.83E-04	-1.24E-01	2.57E-02	3.11E-01	-8.56E-16	4.80E-03	1.05E-02	9.01E-04	2.72E-03	-2.17E-15	9.61E-02
Min	-1.16E+00	-2.42E-01	-2.83E+00	1.35E-02	1.40E-01	-1.38E-14	1.14E-03	2.32E-03	2.13E-04	5.51E-04	-2.64E-14	2.65E-02
Max	5.80E-04	2.77E-04	4.08E-02	5.81E-02	2.63E+00	1.67E-14	3.39E-02	8.18E-02	6.81E-03	1.56E-02	2.21E-14	9.26E-01
Normalized peak height	4.27E-01	3.16E-01	1.65E-02	4.03E-03	1.39E-02	8.72E-02	9.31E-03	1.02E-02	9.79E-03	6.93E-03	3.68E-02	1.14E-02
Peak sensitivity value	-2.51E-07	-8.61E-05	-4.68E-02	2.37E-02	2.41E-01	-5.93E-18	3.45E-03	8.56E-03	6.91E-04	2.19E-03	2.34E-17	6.38E-02
Shapiro-Wilk score	2.607E-01	3.189E-01	6.985E-01	9.608E-01	7.194E-01	8.930E-01	8.431E-01	8.341E-01	8.354E-01	8.756E-01	9.305E-01	7.770E-01
**NADH**												
Local	9.35E-03	4.03E-03	7.30E-01	-1.86E-03	-2.25E-02	6.21E-15	1.54E-01	3.36E-01	2.88E-02	8.69E-02	1.30E-14	-1.28E+00
Min	4.73E-04	2.86E-04	2.95E-02	-8.73E-03	-1.32E-01	-5.70E-14	6.26E-02	1.83E-01	1.07E-02	2.27E-02	-1.31E-13	-1.67E+00
Max	8.83E-01	1.52E-01	1.24E+00	5.52E-03	2.03E-01	3.99E-14	2.92E-01	4.89E-01	6.04E-02	2.07E-01	1.17E-13	-7.04E-01
Normalized peak height	1.12E-01	3.75E-02	4.35E-03	4.98E-03	9.74E-03	1.42E-02	3.63E-03	3.65E-03	4.11E-03	3.61E-03	8.49E-02	4.11E-03
Peak sensitivity value	1.80E-03	1.57E-03	6.55E-01	-1.41E-03	-2.11E-02	2.02E-17	1.43E-01	3.37E-01	2.82E-02	7.62E-02	1.03E-16	-1.25E+00
Shapiro-Wilk score	3.562E-01	5.340E-01	9.829E-01	9.970E-01	9.551E-01	9.304E-01	9.911E-01	9.999E-01	9.901E-01	9.838E-01	9.258E-01	9.989E-01

**Fructose-6-Phosphate**												
Local	0.00E+00	0.00E+00	-2.01E-02	-1.59E-15	-7.98E-17	-4.83E-03	-2.51E-02	-7.13E-03	-5.69E-04	-7.58E-01	-5.75E-02	-2.80E-17
Min	-2.37E-15	-1.29E-14	-5.73E-02	-3.25E-14	-3.29E-14	-1.24E-02	-5.28E-02	-1.52E-02	-2.56E-03	-8.31E-01	-1.33E-01	-3.16E-15
Max	3.52E-15	1.67E-14	8.51E-02	4.22E-14	2.52E-14	1.64E-02	8.20E-02	2.51E-02	3.34E-03	-4.77E-01	1.10E-01	6.36E-16
Normalized peak height	6.45E-01	7.08E-01	1.04E-02	1.02E-02	1.34E-02	8.55E-03	9.07E-03	9.43E-03	1.16E-02	5.59E-03	6.20E-03	3.14E-01
Peak sensitivity value	1.52E-18	1.03E-17	-1.68E-02	-1.33E-17	4.72E-18	-3.58E-03	-2.34E-02	-6.88E-03	-3.56E-04	-7.62E-01	-6.10E-02	9.19E-19
Shapiro-Wilk score	6.795E-01	2.216E-01	9.625E-01	9.750E-01	9.648E-01	9.664E-01	9.456E-01	9.493E-01	9.212E-01	9.460E-01	9.794E-01	8.246E-01
**Extracellular cyanide**												
Local	0.00E+00	0.00E+00	-2.09E-02	3.66E-16	-5.96E-16	-5.03E-03	3.14E-02	-6.50E-02	7.46E-02	5.46E-03	-9.35E-03	-7.74E-19
Min	-2.71E-15	-1.66E-14	-6.45E-02	-1.28E-14	-8.00E-15	-1.30E-02	7.78E-03	-1.85E-01	2.47E-02	-1.32E-02	-3.27E-02	-6.41E-17
Max	8.95E-16	6.83E-15	-5.00E-03	1.10E-14	1.04E-14	-1.47E-03	1.31E-01	-2.20E-02	2.27E-01	1.93E-02	-2.35E-03	2.40E-17
Normalized peak height	6.56E-01	7.67E-01	4.43E-03	1.44E-02	2.69E-02	4.03E-03	5.65E-03	4.56E-03	4.41E-03	8.41E-03	4.92E-03	3.15E-01
Peak sensitivity value	1.25E-19	6.46E-18	-1.75E-02	-5.94E-20	5.13E-18	-4.42E-03	2.20E-02	-5.41E-02	5.97E-02	3.89E-03	-8.16E-03	-3.07E-20
Shapiro-Wilk score	4.668E-01	4.980E-02	9.571E-01	9.663E-01	9.576E-01	9.685E-01	9.092E-01	9.493E-01	9.442E-01	9.471E-01	9.396E-01	7.710E-01
**Pyruvate**												
Local	0.00E+00	0.00E+00	-1.39E-01	-5.18E-14	1.49E-13	-3.34E-02	-1.73E-01	-4.93E-02	-3.93E-03	-1.87E+00	4.59E+00	2.26E-16
Min	-4.71E-14	-1.77E-13	-1.19E+02	-3.63E-10	-2.28E-10	-4.21E+01	-2.12E+02	-4.58E+01	-6.32E+00	-1.32E+03	1.51E-01	-1.32E-11
Max	1.69E-14	1.24E-13	1.41E-02	7.67E-11	1.19E-10	2.93E-03	1.57E-02	4.30E-03	3.02E-04	1.70E+02	7.65E+03	3.05E-12
Normalized peak height	6.71E-01	7.52E-01	9.20E-01	9.64E-01	8.98E-01	9.53E-01	9.52E-01	9.33E-01	9.60E-01	9.27E-01	9.75E-01	9.99E-01
Peak sensitivity value	-2.12E-17	1.43E-16	-4.53E-02	-9.32E-14	1.48E-13	-1.81E-02	-9.04E-02	-1.86E-02	-2.86E-03	-6.86E-01	3.97E+00	-1.09E-15
Shapiro-Wilk score	5.844E-01	1.396E-01	2.042E-02	7.435E-03	8.191E-03	1.389E-02	1.467E-02	1.555E-02	1.295E-02	2.249E-02	8.734E-03	8.517E-04
**Extracellular ethanol**												
Local	-3.41E-16	-1.00E+00	-3.13E-02	-1.73E-16	1.29E-15	-7.52E-03	-3.91E-02	-1.11E-02	-8.85E-04	-1.89E-02	5.21E-02	2.13E-18
Min	-3.14E-15	-1.00E+00	-7.80E-02	-8.50E-15	-7.83E-15	-1.74E-02	-7.32E-02	-1.95E-02	-4.13E-03	-4.55E-02	1.65E-02	-2.35E-17
Max	3.49E-15	-1.00E+00	-8.11E-03	9.09E-15	1.00E-14	-2.79E-03	-1.83E-02	-5.43E-03	-1.38E-04	7.64E-02	9.43E-02	1.17E-16
Normalized peak height	5.32E-01	1.00E+00	4.03E-03	6.88E-03	1.49E-02	3.54E-03	3.10E-03	2.89E-03	5.13E-03	1.10E-02	3.25E-03	3.14E-01
Peak sensitivity value	-9.74E-19	-1.00E+00	-2.82E-02	-4.77E-17	1.17E-17	-7.05E-03	-3.54E-02	-1.10E-02	-6.03E-04	-2.05E-02	5.70E-02	2.69E-20
Shapiro-Wilk score	8.831E-01	1.000E+00	9.717E-01	9.845E-01	9.726E-01	9.752E-01	9.827E-01	9.882E-01	9.088E-01	8.674E-01	9.986E-01	9.044E-01
**Extracellular glycerol**												
Local	0.00E+00	0.00E+00	2.79E-01	9.72E-15	-1.00E+00	6.70E-02	3.48E-01	9.89E-02	7.89E-03	-7.27E-02	1.24E-01	1.03E-17
Min	-5.59E-14	-3.29E-13	8.12E-02	-2.18E-13	-1.00E+00	2.14E-02	1.64E-01	3.31E-02	2.34E-03	-1.37E-01	5.20E-02	-6.67E-16
Max	5.42E-14	1.42E-13	4.92E-01	2.14E-13	-1.00E+00	1.43E-01	5.27E-01	1.83E-01	2.03E-02	1.15E-01	2.01E-01	4.62E-16
Normalized peak height	6.55E-01	7.45E-01	3.17E-03	1.25E-02	1.00E+00	3.80E-03	3.61E-03	3.63E-03	3.78E-03	1.10E-02	3.86E-03	3.15E-01
Peak sensitivity value	-2.05E-17	-1.09E-16	2.56E-01	1.76E-16	-1.00E+00	6.50E-02	3.62E-01	9.79E-02	5.92E-03	-6.92E-02	1.18E-01	-5.21E-19
Shapiro-Wilk score	2.021E-01	3.588E-02	9.988E-01	9.822E-01	1.000E+00	9.917E-01	9.988E-01	9.975E-01	9.704E-01	8.189E-01	9.974E-01	8.519E-01
**Extracellular glucose**												
Local	0.00E+00	0.00E+00	2.04E-03	1.65E-16	1.51E-17	4.89E-04	2.54E-03	7.23E-04	5.76E-05	2.55E-02	5.83E-03	-3.53E-18
Min	-2.21E-15	-1.17E-15	-1.52E-02	-3.09E-14	-1.89E-14	-4.33E-03	-1.75E-02	-4.90E-03	-6.98E-04	-7.08E-01	-3.78E-02	-6.52E-16
Max	1.04E-15	1.63E-15	7.91E-02	2.06E-14	2.07E-14	1.40E-02	7.40E-02	1.98E-02	3.36E-03	1.96E-01	9.78E-02	3.56E-16
Normalized peak height	6.81E-01	7.01E-01	4.62E-02	7.50E-02	8.90E-02	3.89E-02	3.81E-02	3.58E-02	6.90E-02	4.11E-02	2.55E-02	3.16E-01
Peak sensitivity value	-3.99E-19	1.40E-19	8.68E-04	2.39E-17	1.61E-17	2.20E-04	1.21E-03	3.74E-04	1.86E-05	1.46E-02	3.24E-03	2.19E-20
Shapiro-Wilk score	2.668E-01	1.753E-01	6.156E-01	5.952E-01	5.830E-01	6.134E-01	6.152E-01	6.381E-01	5.372E-01	3.913E-01	6.959E-01	5.594E-01
**1-3-Bisphosphoglycerate**												
Local	0.00E+00	0.00E+00	-2.82E-02	6.06E-16	-4.86E-16	-6.78E-03	-3.52E-02	-1.00E-02	-7.98E-04	-3.98E-01	-7.28E-03	7.56E-17
Min	-1.19E-14	-1.63E-14	-1.16E-01	-8.24E-14	-6.30E-14	-3.00E-02	-1.31E-01	-3.49E-02	-6.23E-03	-4.96E-01	-1.48E-01	-5.43E-16
Max	1.92E-14	3.75E-14	-8.72E-03	8.85E-14	6.08E-14	-2.15E-03	-1.42E-02	-4.41E-03	-1.22E-04	-2.88E-01	1.62E-02	4.76E-15
Normalized peak height	6.61E-01	6.98E-01	6.02E-03	1.24E-02	1.41E-02	6.37E-03	6.06E-03	6.21E-03	8.17E-03	5.55E-03	7.41E-03	3.17E-01
Peak sensitivity value	-1.73E-18	-1.70E-17	-2.66E-02	-6.27E-16	-2.49E-17	-5.34E-03	-2.91E-02	-8.73E-03	-4.79E-04	-4.00E-01	-2.17E-03	3.12E-19
Shapiro-Wilk score	2.741E-01	1.035E-01	9.214E-01	9.650E-01	9.509E-01	9.256E-01	9.189E-01	9.336E-01	8.513E-01	9.978E-01	8.796E-01	8.720E-01
**Fructose 1-6-bisphosphate**												
Local	0.00E+00	0.00E+00	-9.76E-01	1.28E-14	-8.40E-15	8.90E-02	4.63E-01	1.31E-01	1.05E-02	-2.10E-01	2.16E-01	3.37E-17
Min	-8.03E-14	-2.97E-13	-1.98E+00	-3.04E-13	-2.67E-13	2.69E-02	1.67E-01	4.63E-02	2.00E-03	-3.56E-01	7.76E-02	-2.04E-16
Max	5.81E-14	3.38E-13	-3.59E-01	4.30E-13	2.71E-13	2.24E-01	8.23E-01	2.75E-01	3.88E-02	1.35E-01	4.04E-01	3.14E-15
Normalized peak height	6.81E-01	7.56E-01	3.31E-03	1.34E-02	3.62E-02	3.92E-03	3.21E-03	3.52E-03	4.45E-03	7.02E-03	4.15E-03	3.15E-01
Peak sensitivity value	4.24E-17	-3.12E-16	-9.55E-01	3.25E-16	7.10E-17	8.22E-02	4.25E-01	1.14E-01	7.62E-03	-2.01E-01	2.08E-01	-1.60E-18
Shapiro-Wilk score	3.048E-01	3.693E-02	9.851E-01	9.649E-01	9.540E-01	9.804E-01	9.947E-01	9.871E-01	9.381E-01	9.508E-01	9.925E-01	8.231E-01
**AMP**												
Local	0.00E+00	0.00E+00	4.30E-02	3.70E-15	2.03E-16	1.03E-02	5.37E-02	1.52E-02	1.22E-03	6.08E-01	1.23E-01	6.38E-17
Min	-1.38E-14	-4.15E-14	1.19E-02	-1.34E-13	-1.03E-13	3.58E-03	2.26E-02	6.37E-03	1.75E-04	4.44E-01	7.48E-02	-1.46E-15
Max	2.40E-14	3.22E-14	1.94E-01	8.91E-14	8.04E-14	5.17E-02	2.10E-01	5.25E-02	9.11E-03	7.16E-01	2.92E-01	7.34E-15
Normalized peak height	6.66E-01	7.14E-01	6.45E-03	1.25E-02	1.53E-02	7.89E-03	6.37E-03	6.05E-03	7.79E-03	7.19E-03	4.76E-03	3.16E-01
Peak sensitivity value	-1.69E-17	-9.73E-18	3.75E-02	7.30E-16	-5.57E-17	8.70E-03	4.33E-02	1.42E-02	7.42E-04	6.08E-01	1.24E-01	-3.83E-18
Shapiro-Wilk score	5.088E-01	2.075E-01	9.242E-01	9.575E-01	9.480E-01	9.285E-01	9.224E-01	9.385E-01	8.554E-01	9.949E-01	9.786E-01	7.781E-01
**ATP**												
Local	0.00E+00	0.00E+00	-1.64E-02	-1.37E-15	-6.97E-17	-3.93E-03	-2.04E-02	-5.81E-03	-4.63E-04	-2.32E-01	-4.68E-02	2.55E-17
Min	-5.53E-15	-1.96E-14	-1.19E-01	-4.94E-14	-4.71E-14	-2.61E-02	-1.29E-01	-3.31E-02	-5.91E-03	-4.86E-01	-1.72E-01	-5.04E-16
Max	3.86E-15	2.47E-14	-3.62E-03	5.30E-14	5.58E-14	-1.14E-03	-7.70E-03	-2.23E-03	-6.44E-05	-1.56E-01	-2.82E-02	4.26E-15
Normalized peak height	6.46E-01	6.94E-01	9.77E-03	1.48E-02	2.33E-02	8.48E-03	9.42E-03	8.50E-03	1.25E-02	5.33E-03	6.70E-03	3.15E-01
Peak sensitivity value	-3.02E-18	2.06E-17	-1.23E-02	-9.44E-17	6.60E-17	-3.00E-03	-1.69E-02	-5.15E-03	-3.01E-04	-2.29E-01	-4.65E-02	-6.73E-19
Shapiro-Wilk score	5.301E-01	1.572E-01	8.413E-01	9.223E-01	9.015E-01	8.479E-01	8.313E-01	8.549E-01	7.689E-01	9.509E-01	9.070E-01	7.169E-01
**NADH**												
Local	0.00E+00	0.00E+00	-5.23E-01	6.20E-15	-5.37E-15	5.25E-02	2.73E-01	7.75E-02	6.18E-03	1.86E-02	5.39E-02	-5.02E-18
Min	-3.46E-14	-1.98E-13	-6.84E-01	-1.72E-13	-1.02E-13	1.55E-02	1.10E-01	2.52E-02	1.91E-03	-2.63E-02	2.12E-02	-1.69E-16
Max	4.58E-14	1.51E-13	-2.93E-01	1.76E-13	1.20E-13	1.15E-01	4.12E-01	1.51E-01	1.44E-02	9.90E-02	9.67E-02	1.12E-16
Normalized peak height	6.70E-01	7.71E-01	4.53E-03	9.85E-03	2.12E-02	3.74E-03	3.38E-03	3.17E-03	3.65E-03	4.64E-03	3.53E-03	3.17E-01
Peak sensitivity value	2.65E-19	-1.88E-17	-5.23E-01	5.40E-16	1.02E-16	5.28E-02	2.90E-01	7.68E-02	4.92E-03	1.67E-02	4.93E-02	1.24E-19
Shapiro-Wilk score	1.750E-01	3.418E-02	9.926E-01	9.823E-01	9.737E-01	9.939E-01	9.929E-01	9.985E-01	9.754E-01	9.925E-01	9.902E-01	9.067E-01

Table S21: Concentration control coefficients for *Saccharomyces cerevisiae* model with parameter variation of ±30%

Metabolite	Statistic	(GlucoseMixedflowtoextracellularmedium)	(Glucoseuptake)	(Hexokinase)	(Phosphoglucoisomerase)	(Phosphofructokinase)	(Aldolase)	(Triosephosphateisomerase)	(Glyceraldehyde3-phosphatedehydrogenase)	(Phosphoenolpyruvatesynthesis)	(Pyruvatekinase)	(Pyruvatedecarboxylase)	(Alcoholdehydrogenase)
Acetaldehyde	Local	2.55E-02	1.10E-02	1.99E+00	8.76E-03	1.06E-01	-5.75E-15	-8.30E-02	-1.82E-01	-1.56E-02	-4.70E-02	-7.51E-15	-1.66E+00
	Min	-1.73E-03	-3.06E-03	-8.26E-03	6.30E-04	3.49E-02	-6.47E-14	-2.84E-01	-4.02E-01	-4.16E-02	-1.72E-01	-4.10E-13	-2.85E+00
	Max	2.78E+00	9.27E-01	3.13E+00	5.35E-02	2.38E+00	5.73E-14	-5.12E-03	-2.87E-02	-2.03E-03	-2.90E-03	2.72E-13	-9.64E-01
	Normalized peak height	1.33E-01	7.54E-02	5.20E-03	8.57E-03	2.85E-02	3.05E-02	4.00E-03	3.14E-03	4.04E-03	4.59E-03	1.24E-01	3.69E-03
	Peak sensitivity value	5.22E-03	2.99E-03	1.67E+00	7.05E-03	7.36E-02	6.79E-18	-6.81E-02	-1.63E-01	-1.38E-02	-3.20E-02	2.57E-16	-1.48E+00
	Shapiro-Wilk score	4.817E-01	4.917E-01	8.374E-01	8.717E-01	4.190E-01	8.629E-01	9.739E-01	9.944E-01	9.752E-01	9.456E-01	7.019E-01	9.753E-01
Cytosolic glucose	Local	2.04E+00	8.77E-01	-3.34E+00	7.30E-03	8.83E-02	4.39E-16	-2.81E-03	-6.15E-03	-5.28E-04	-1.59E-03	1.00E-15	-5.63E-02
	Min	1.10E-01	5.77E-02	-1.56E+04	-1.94E+01	-2.53E+02	-3.87E-13	-7.11E+00	-1.41E+01	-1.30E+00	-5.54E+00	-2.29E-13	-9.51E+01
	Max	1.39E+04	1.89E+03	-5.12E-01	3.22E+00	1.51E+02	4.93E-13	5.66E-01	1.06E+00	7.13E-02	1.46E-01	4.47E-12	2.52E+01
	Normalized peak height	9.67E-01	9.06E-01	9.64E-01	6.80E-01	7.80E-01	6.68E-01	4.86E-01	7.92E-01	7.93E-01	6.13E-01	8.71E-01	6.51E-01
	Peak sensitivity value	7.06E+00	1.00E+00	-8.30E+00	-8.69E-04	8.74E-02	-1.95E-16	1.50E-03	-6.45E-03	-4.92E-04	1.03E-03	-8.65E-16	-2.15E-02
	Shapiro-Wilk score	9.640E-03	1.587E-02	1.072E-02	3.762E-02	8.865E-02	8.091E-02	2.774E-02	3.452E-02	3.049E-02	1.815E-02	1.529E-02	5.469E-02
Glycerol	Local	2.51E-02	1.08E-02	1.96E+00	8.63E-03	1.04E-01	8.13E-15	-8.18E-02	-1.79E-01	-1.53E-02	-4.63E-02	1.53E-14	-1.64E+00
	Min	-1.36E-02	-2.97E-03	-3.67E-02	8.02E-04	2.83E-02	-6.62E-14	-2.76E-01	-3.81E-01	-4.04E-02	-1.61E-01	-3.10E-13	-2.89E+00
	Max	2.58E+00	1.01E+00	3.02E+00	5.13E-02	2.39E+00	7.72E-14	-6.04E-03	-2.77E-02	-2.40E-03	-1.86E-03	3.28E-13	-9.44E-01
	Normalized peak height	1.38E-01	8.39E-02	5.29E-03	8.10E-03	2.80E-02	2.40E-02	3.80E-03	2.96E-03	4.07E-03	4.13E-03	1.10E-01	3.50E-03
	Peak sensitivity value	3.24E-03	2.61E-03	1.66E+00	7.40E-03	7.19E-02	-3.03E-18	-5.91E-02	-1.63E-01	-1.26E-02	-3.43E-02	-1.14E-16	-1.45E+00
	Shapiro-Wilk score	4.872E-01	5.002E-01	8.298E-01	8.741E-01	4.286E-01	8.943E-01	9.733E-01	9.943E-01	9.744E-01	9.486E-01	7.872E-01	9.771E-01
EtOH	Local	1.01E-02	4.37E-03	7.92E-01	2.32E-03	2.81E-02	-3.86E-16	9.18E-03	2.01E-02	1.72E-03	5.20E-03	-7.43E-17	1.84E-01
	Min	4.04E-04	2.23E-04	-3.64E-02	1.77E-04	2.34E-03	-6.90E-15	1.40E-03	2.69E-03	1.94E-04	2.44E-04	-2.79E-14	3.81E-02
	Max	8.64E-01	3.51E-01	9.20E-01	1.44E-02	8.48E-01	3.45E-15	4.11E-02	7.87E-02	8.76E-03	3.11E-02	1.84E-14	7.10E-01
	Normalized peak height	1.06E-01	6.43E-02	6.25E-03	8.24E-03	3.28E-02	3.81E-02	5.64E-03	4.71E-03	5.76E-03	6.48E-03	1.35E-01	4.48E-03
	Peak sensitivity value	1.70E-03	1.45E-03	8.61E-01	2.05E-03	2.22E-02	-2.81E-18	7.15E-03	1.60E-02	1.28E-03	3.81E-03	-1.58E-17	9.02E-02
	Shapiro-Wilk score	4.856E-01	5.066E-01	7.422E-01	8.704E-01	4.304E-01	8.695E-01	9.332E-01	9.429E-01	9.162E-01	8.857E-01	8.435E-01	8.795E-01
ADP	Local	1.29E-03	5.55E-04	1.01E-01	-2.09E-02	-2.53E-01	6.63E-16	-3.90E-03	-8.54E-03	-7.33E-04	-2.21E-03	1.57E-15	-7.81E-02
	Min	-1.15E+00	-2.53E-01	-1.30E+00	-5.02E-02	-1.99E+00	-1.01E-14	-2.76E-02	-5.27E-02	-4.32E-03	-1.82E-02	-4.81E-14	-5.28E-01
	Max	1.26E+00	2.99E-01	1.90E+00	7.44E-03	1.30E+00	1.09E-14	1.89E-02	4.40E-02	3.09E-03	4.55E-03	2.73E-14	4.63E-01
	Normalized peak height	5.69E-01	4.70E-01	2.79E-02	5.34E-03	2.41E-02	9.93E-02	1.32E-02	1.33E-02	1.09E-02	1.06E-02	1.29E-01	1.33E-02
	Peak sensitivity value	-9.36E-05	2.80E-05	2.75E-03	-1.67E-02	-2.01E-01	9.81E-18	-2.25E-03	-6.10E-03	-5.43E-04	-1.12E-03	1.44E-17	-4.00E-02
	Shapiro-Wilk score	3.896E-01	3.453E-01	7.503E-01	9.819E-01	7.435E-01	8.755E-01	8.950E-01	9.134E-01	8.964E-01	8.701E-01	8.175E-01	8.650E-01
Glyceraldehyde 3-phosphate	Local	2.26E-02	9.73E-03	1.76E+00	1.27E-02	1.54E-01	6.19E-15	1.56E-01	-5.55E-01	-4.76E-02	-1.44E-01	1.37E-14	-1.23E+00
	Min	-1.30E-01	-6.21E-02	-5.24E-01	6.53E-04	4.10E-02	-4.40E-14	2.66E-02	-1.00E+00	-1.14E-01	-4.84E-01	-3.21E-13	-3.25E+00
	Max	2.91E+00	1.12E+00	4.34E+00	5.87E-02	2.86E+00	6.57E-14	4.33E-01	-1.01E-01	-7.88E-03	-1.07E-02	3.05E-13	-2.49E-01
	Normalized peak height	1.49E-01	1.01E-01	7.56E-03	6.22E-03	2.18E-02	2.22E-02	3.95E-03	2.72E-03	4.14E-03	4.05E-03	1.28E-01	3.51E-03
	Peak sensitivity value	5.22E-03	2.14E-03	1.38E+00	9.56E-03	1.04E-01	-2.17E-17	1.23E-01	-5.76E-01	-4.43E-02	-1.07E-01	-2.09E-16	-8.66E-01
	Shapiro-Wilk score	4.847E-01	4.884E-01	9.164E-01	9.206E-01	4.788E-01	8.976E-01	9.790E-01	9.959E-01	9.810E-01	9.546E-01	7.803E-01	9.632E-01
Dihydroxyacetone phosphate	Local	2.40E-02	1.03E-02	1.88E+00	1.23E-02	1.49E-01	5.18E-15	-2.24E-01	-4.91E-01	-4.21E-02	-1.27E-01	1.04E-14	-1.03E+00
	Min	-1.80E-01	-7.79E-02	-3.58E-01	9.54E-04	4.01E-02	-3.69E-14	-6.60E-01	-9.24E-01	-1.06E-01	-4.81E-01	-1.74E-13	-2.95E+00
	Max	3.19E+00	1.12E+00	3.71E+00	5.48E-02	2.78E+00	3.41E-14	-2.42E-02	-9.65E-02	-1.01E-02	-9.76E-03	2.54E-13	-1.31E-01
	Normalized peak height	1.79E-01	1.00E-01	6.56E-03	6.29E-03	2.27E-02	1.67E-02	3.68E-03	3.13E-03	4.14E-03	4.66E-03	1.12E-01	3.78E-03
	Peak sensitivity value	3.36E-03	3.21E-03	1.52E+00	1.05E-02	9.62E-02	-5.49E-18	-2.10E-01	-4.72E-01	-3.47E-02	-8.26E-02	1.48E-16	-7.68E-01
	Shapiro-Wilk score	4.859E-01	5.029E-01	8.838E-01	9.156E-01	4.730E-01	9.067E-01	9.809E-01	9.977E-01	9.787E-01	9.516E-01	7.993E-01	9.559E-01
Glucose-6-Phosphate	Local	1.37E-02	5.88E-03	1.07E+00	-2.72E-02	-3.29E-01	-8.33E-16	5.10E-03	1.11E-02	9.57E-04	2.89E-03	-1.82E-15	1.02E-01
	Min	5.09E-04	3.03E-04	-3.68E-01	-8.95E-02	-7.57E+00	-2.18E-14	-9.18E-02	-1.59E-01	-1.53E-02	-3.15E-02	-1.55E-13	-1.75E+00
	Max	5.90E+00	1.34E+00	8.74E+00	-1.88E-03	-1.24E-01	2.86E-14	2.18E-02	4.84E-02	5.29E-03	2.28E-02	8.88E-14	4.37E-01
	Normalized peak height	3.77E-01	1.73E-01	4.79E-02	5.21E-03	3.11E-02	1.16E-01	2.03E-02	1.75E-02	1.90E-02	1.55E-02	1.85E-01	2.17E-02
	Peak sensitivity value	3.46E-03	2.31E-03	1.01E+00	-2.23E-02	-2.25E-01	3.62E-18	3.03E-03	8.49E-03	5.26E-04	1.45E-03	1.22E-16	6.24E-02
	Shapiro-Wilk score	4.623E-01	3.590E-01	6.271E-01	9.419E-01	4.951E-01	6.504E-01	6.854E-01	6.629E-01	7.159E-01	8.716E-01	5.965E-01	6.402E-01
NAD	Local	-4.87E-03	-2.10E-03	-3.80E-01	9.66E-04	1.17E-02	-3.24E-15	-8.00E-02	-1.75E-01	-1.50E-02	-4.53E-02	-6.78E-15	6.68E-01
	Min	-1.23E+00	-3.52E-01	-1.81E+00	-1.20E-02	-9.67E-01	-2.69E-14	-2.64E-01	-7.39E-01	-1.27E-01	-3.03E-01	-1.18E-13	1.21E-01
	Max	-4.88E-05	-3.80E-05	1.10E-02	1.18E-02	5.33E-01	3.06E-14	-9.95E-03	-2.59E-02	-8.01E-04	-1.33E-03	2.55E-13	2.33E+00
	Normalized peak height	3.19E-01	1.49E-01	5.76E-03	1.25E-02	5.99E-02	2.56E-02	3.85E-03	4.71E-03	7.12E-03	5.99E-03	1.62E-01	4.06E-03
	Peak sensitivity value	-6.65E-04	-5.66E-04	-1.74E-01	1.94E-04	4.24E-03	1.82E-17	-6.59E-02	-1.20E-01	-6.81E-03	-2.71E-02	-1.49E-16	3.81E-01
	Shapiro-Wilk score	4.447E-01	4.544E-01	9.148E-01	9.115E-01	4.336E-01	8.903E-01	9.564E-01	9.243E-01	8.663E-01	8.730E-01	8.006E-01	9.363E-01
Extracellular acetaldehyde	Local	2.55E-02	1.10E-02	1.99E+00	8.78E-03	1.06E-01	-5.73E-15	-8.32E-02	-1.82E-01	-1.56E-02	-4.71E-02	-8.63E-15	-1.67E+00
	Min	-4.67E-04	-7.71E-04	-3.50E-02	5.57E-04	3.34E-02	-6.79E-14	-2.86E-01	-4.03E-01	-4.45E-02	-2.29E-01	-2.36E-13	-2.82E+00
	Max	2.73E+00	9.39E-01	3.03E+00	5.83E-02	2.49E+00	5.22E-14	-5.33E-03	-3.24E-02	-1.74E-03	-2.28E-03	3.01E-13	-9.65E-01
	Normalized peak height	1.44E-01	7.52E-02	4.76E-03	8.97E-03	2.91E-02	2.91E-02	3.88E-03	3.15E-03	4.61E-03	6.11E-03	1.24E-01	3.42E-03
	Peak sensitivity value	3.64E-03	3.46E-03	1.67E+00	6.77E-03	7.15E-02	2.79E-19	-5.80E-02	-1.79E-01	-1.37E-02	-3.01E-02	3.56E-17	-1.46E+00
	Shapiro-Wilk score	4.879E-01	5.058E-01	8.416E-01	8.746E-01	4.323E-01	8.683E-01	9.724E-01	9.940E-01	9.740E-01	9.438E-01	7.005E-01	9.757E-01
Phosphoenolpyruvate	Local	1.38E-02	5.93E-03	1.08E+00	6.08E-03	7.36E-02	2.62E-15	1.11E-03	2.42E-03	2.08E-04	-1.20E+00	7.14E-14	2.22E-02
	Min	5.30E-04	3.31E-04	-2.70E-01	1.94E-04	3.17E-02	-7.00E-14	-1.11E-03	-1.95E-03	-2.20E-04	-1.45E+00	-3.69E-13	-2.33E-02
	Max	1.18E+00	5.04E-01	1.28E+00	2.34E-02	1.16E+00	6.18E-14	1.77E-02	3.59E-02	3.76E-03	-1.10E+00	3.71E-13	3.32E-01
	Normalized peak height	1.10E-01	7.07E-02	1.14E-02	6.99E-03	2.36E-02	7.21E-02	1.74E-02	1.60E-02	1.99E-02	3.79E-03	2.60E-01	1.71E-02
	Peak sensitivity value	2.31E-03	2.10E-03	1.04E+00	5.92E-03	5.27E-02	4.78E-17	7.59E-04	1.74E-03	1.40E-04	-1.17E+00	-3.28E-16	1.28E-02
	Shapiro-Wilk score	4.899E-01	5.103E-01	6.493E-01	9.333E-01	4.734E-01	8.650E-01	6.151E-01	6.059E-01	6.225E-01	9.524E-01	8.576E-01	5.761E-01

Metabolite	Statistic	(Ethanolout)	(Ethanolflow)	(Glycerolsynthesis)	(Glycerolout)	(Glycerolflow)	(Acetaldehydeout)	(Acetaldehydeflow)	(Cyanide-Acetaldehydeflow)	(Cyanideflow)	(Storage)	(ATPconsumption)	(Adenylatekinase)
Acetaldehyde	Local	0.00E+00	0.00E+00	2.83E-01	-5.12E-15	7.96E-15	-6.23E-02	-3.24E-01	-9.20E-02	-7.34E-03	-7.38E-02	1.26E-01	1.05E-17
	Min	-2.44E-13	-1.70E-12	2.76E-02	-1.49E-13	-1.63E-13	-1.89E-01	-6.63E-01	-2.03E-01	-7.87E-02	-2.05E-01	-1.56E-03	-3.98E-16
	Max	3.42E-13	2.76E-12	5.94E-01	1.53E-13	2.02E-13	-1.50E-02	-1.21E-01	-2.85E-02	-4.97E-04	4.89E-01	2.56E-01	5.08E-16
	Normalized peak height	6.66E-01	9.04E-01	3.14E-03	1.49E-02	4.25E-02	4.40E-03	3.71E-03	3.29E-03	1.02E-02	1.74E-02	3.94E-03	2.89E-01
	Peak sensitivity value	1.92E-16	1.66E-15	2.80E-01	1.49E-16	3.59E-17	-5.30E-02	-3.06E-01	-8.29E-02	-3.90E-03	-6.84E-02	1.15E-01	1.31E-19
	Shapiro-Wilk score	1.606E-01	9.244E-02	9.972E-01	9.594E-01	9.306E-01	9.671E-01	9.848E-01	9.839E-01	8.118E-01	7.233E-01	9.882E-01	8.881E-01
Cytosolic glucose	Local	0.00E+00	0.00E+00	9.58E-03	7.63E-16	4.37E-17	2.30E-03	1.20E-02	3.40E-03	2.71E-04	3.43E-01	2.74E-02	7.34E-18
	Min	-7.22E-13	-4.34E-13	-1.41E+00	-2.72E-12	-3.03E-12	-7.50E-01	-3.40E+00	-8.86E-01	-1.21E-01	5.20E-02	-1.75E+00	-1.81E-13
	Max	2.92E-14	2.09E-13	2.47E+01	1.57E-12	1.34E-12	6.68E+00	2.19E+01	6.29E+00	5.86E-01	4.09E+02	7.75E+01	1.73E-15
	Normalized peak height	8.99E-01	8.62E-01	8.10E-01	4.33E-01	7.50E-01	8.66E-01	4.99E-01	6.54E-01	7.48E-01	6.49E-01	8.41E-01	9.96E-01
	Peak sensitivity value	2.53E-16	-2.36E-16	1.11E-02	2.12E-15	-7.41E-16	3.57E-03	2.08E-02	1.14E-03	2.20E-04	2.57E-01	3.39E-02	-6.97E-18
	Shapiro-Wilk score	9.335E-03	5.045E-02	3.040E-02	8.925E-02	6.095E-02	2.498E-02	3.875E-02	3.440E-02	4.897E-02	3.299E-02	1.648E-02	1.472E-03
Glycerol	Local	0.00E+00	0.00E+00	2.79E-01	-5.98E-01	-4.02E-01	6.70E-02	3.48E-01	9.89E-02	7.89E-03	-7.27E-02	1.24E-01	1.03E-17
	Min	-2.97E-13	-2.86E-12	3.43E-02	-7.79E-01	-6.31E-01	1.22E-02	9.24E-02	1.67E-02	1.06E-03	-2.00E-01	-1.70E-03	-5.88E-16
	Max	4.91E-13	2.48E-12	5.94E-01	-3.69E-01	-2.21E-01	1.89E-01	5.80E-01	2.33E-01	2.76E-02	4.86E-01	2.59E-01	4.02E-15
	Normalized peak height	7.00E-01	9.42E-01	3.00E-03	2.64E-03	2.64E-03	3.71E-03	3.38E-03	3.46E-03	4.34E-03	1.71E-02	4.32E-03	2.92E-01
	Peak sensitivity value	1.38E-16	-1.57E-15	2.86E-01	-6.11E-01	-3.89E-01	6.27E-02	3.41E-01	9.22E-02	5.22E-03	-7.05E-02	1.19E-01	1.99E-20
	Shapiro-Wilk score	1.753E-01	1.240E-01	9.971E-01	9.943E-01	9.943E-01	9.812E-01	9.978E-01	9.934E-01	9.393E-01	7.352E-01	9.894E-01	3.366E-01
EtOH	Local	-1.45E-01	-8.55E-01	-3.13E-02	-1.63E-16	1.29E-15	-7.52E-03	-3.91E-02	-1.11E-02	-8.85E-04	-1.89E-02	5.21E-02	2.13E-18
	Min	-2.87E-01	-9.39E-01	-1.20E-01	-2.29E-14	-2.18E-14	-2.70E-02	-1.06E-01	-3.08E-02	-7.69E-03	-6.77E-02	7.95E-05	-7.29E-17
	Max	-6.12E-02	-7.13E-01	-5.14E-03	2.34E-14	2.31E-14	-1.70E-03	-1.16E-02	-3.59E-03	-5.64E-05	1.89E-01	1.23E-01	1.24E-16
	Normalized peak height	2.84E-03	2.84E-03	4.50E-03	2.15E-02	3.90E-02	4.42E-03	3.91E-03	3.97E-03	8.16E-03	1.31E-02	3.15E-03	2.89E-01
	Peak sensitivity value	-1.21E-01	-8.79E-01	-2.22E-02	-1.99E-17	1.65E-17	-6.09E-03	-3.21E-02	-8.84E-03	-4.34E-04	-1.99E-02	5.15E-02	1.92E-20
	Shapiro-Wilk score	9.814E-01	9.814E-01	9.373E-01	9.201E-01	8.874E-01	9.453E-01	9.639E-01	9.742E-01	8.335E-01	8.012E-01	9.982E-01	8.988E-01
ADP	Local	0.00E+00	0.00E+00	1.33E-02	1.09E-15	5.21E-17	3.20E-03	1.66E-02	4.72E-03	3.76E-04	1.88E-01	3.81E-02	-4.97E-17
	Min	-1.86E-14	-6.90E-14	-6.59E-02	-5.72E-14	-4.15E-14	-1.80E-02	-6.07E-02	-1.37E-02	-3.35E-03	-1.86E-01	-3.16E-02	-5.08E-15
	Max	2.57E-14	1.70E-13	8.93E-02	5.78E-14	4.88E-14	1.84E-02	7.83E-02	2.01E-02	4.11E-03	3.02E-01	1.34E-01	1.37E-15
	Normalized peak height	5.22E-01	7.85E-01	1.26E-02	2.68E-02	3.43E-02	1.32E-02	1.14E-02	1.00E-02	1.79E-02	7.19E-03	6.46E-03	2.86E-01
	Peak sensitivity value	-2.07E-17	-2.84E-17	8.55E-03	4.32E-18	-5.38E-18	2.17E-03	1.30E-02	3.70E-03	1.58E-04	1.81E-01	3.75E-02	-2.66E-18
	Shapiro-Wilk score	2.831E-01	1.439E-01	9.141E-01	8.949E-01	8.664E-01	9.172E-01	9.189E-01	9.300E-01	8.212E-01	8.903E-01	9.781E-01	7.869E-01
Glyceraldehyde 3-phosphate	Local	0.00E+00	0.00E+00	-5.32E-01	7.05E-15	-4.91E-15	5.03E-02	2.61E-01	7.42E-02	5.92E-03	-1.10E-01	1.05E-01	1.80E-17
	Min	-1.84E-13	-1.00E-12	-1.45E+00	-2.85E-13	-2.39E-13	7.52E-03	5.45E-02	1.28E-02	3.95E-04	-2.40E-01	-3.08E-02	-7.35E-16
	Max	2.61E-13	1.43E-12	-1.22E-01	3.38E-13	3.86E-13	1.75E-01	5.69E-01	2.17E-01	3.11E-02	4.25E-01	2.75E-01	9.55E-16
	Normalized peak height	6.60E-01	9.48E-01	3.47E-03	2.59E-02	5.33E-02	4.30E-03	3.26E-03	3.93E-03	5.60E-03	1.05E-02	4.89E-03	2.87E-01
	Peak sensitivity value	1.38E-16	2.36E-16	-4.95E-01	-1.32E-16	-6.89E-17	3.91E-02	2.28E-01	6.14E-02	3.02E-03	-1.09E-01	9.21E-02	-7.77E-19
	Shapiro-Wilk score	1.869E-01	1.305E-01	9.804E-01	8.992E-01	8.721E-01	9.589E-01	9.894E-01	9.731E-01	8.966E-01	8.674E-01	9.869E-01	8.926E-01
Dihydroxyacetone phosphate	Local	0.00E+00	0.00E+00	-4.89E-01	6.32E-15	-3.90E-15	4.23E-02	2.20E-01	6.25E-02	4.99E-03	-1.06E-01	1.13E-01	1.69E-17
	Min	-2.94E-13	-5.58E-13	-1.31E+00	-2.75E-13	-2.50E-13	4.45E-03	3.06E-02	6.68E-03	2.64E-04	-2.55E-01	-2.35E-02	-7.57E-16
	Max	2.13E-13	6.74E-13	-9.55E-02	2.29E-13	2.97E-13	1.68E-01	4.81E-01	1.81E-01	3.22E-02	4.67E-01	2.64E-01	2.56E-15
	Normalized peak height	6.89E-01	7.61E-01	3.61E-03	2.76E-02	5.37E-02	4.89E-03	3.24E-03	4.03E-03	6.62E-03	1.22E-02	4.55E-03	2.85E-01
	Peak sensitivity value	1.95E-16	5.84E-16	-4.58E-01	-5.77E-17	-5.83E-17	3.14E-02	2.00E-01	5.93E-02	2.74E-03	-1.04E-01	1.08E-01	-1.25E-19
	Shapiro-Wilk score	1.816E-01	1.585E-01	9.690E-01	8.955E-01	8.557E-01	9.558E-01	9.875E-01	9.718E-01	8.829E-01	8.469E-01	9.870E-01	7.258E-01
Glucose-6-Phosphate	Local	0.00E+00	0.00E+00	-1.74E-02	-1.37E-15	-6.86E-17	-4.17E-03	-2.17E-02	-6.16E-03	-4.92E-04	-7.52E-01	-4.97E-02	-2.72E-17
	Min	-2.58E-14	-1.12E-13	-7.60E-02	-1.97E-13	-2.43E-13	-1.78E-02	-7.02E-02	-1.88E-02	-4.41E-03	-8.95E-01	-1.83E-01	-2.38E-15
	Max	1.76E-14	1.69E-13	2.58E-01	1.45E-13	8.87E-14	4.64E-02	2.93E-01	6.58E-02	1.33E-02	1.67E-02	2.38E-01	7.85E-16
	Normalized peak height	5.35E-01	7.26E-01	1.75E-02	7.35E-02	1.06E-01	1.35E-02	1.71E-02	1.38E-02	2.99E-02	9.06E-03	7.03E-03	2.91E-01
	Peak sensitivity value	1.18E-17	-1.14E-16	-1.44E-02	-1.03E-16	6.08E-17	-3.29E-03	-1.78E-02	-5.14E-03	-2.43E-04	-7.47E-01	-5.10E-02	-1.01E-18
	Shapiro-Wilk score	3.064E-01	1.530E-01	7.020E-01	6.954E-01	6.826E-01	7.281E-01	6.759E-01	7.056E-01	6.526E-01	8.253E-01	9.080E-01	8.664E-01
NAD	Local	0.00E+00	0.00E+00	2.72E-01	-3.23E-15	2.80E-15	-2.73E-02	-1.42E-01	-4.03E-02	-3.22E-03	-9.68E-03	-2.81E-02	2.62E-18
	Min	-8.41E-14	-4.90E-13	5.33E-02	-2.92E-13	-1.70E-13	-1.16E-01	-4.57E-01	-1.43E-01	-1.88E-02	-2.26E-01	-1.40E-01	-1.03E-16
	Max	1.30E-13	5.23E-13	7.94E-01	3.13E-13	1.76E-13	-3.10E-03	-2.33E-02	-6.02E-03	-2.36E-04	4.34E-02	-3.22E-04	3.64E-16
	Normalized peak height	6.81E-01	9.39E-01	3.16E-03	4.15E-02	5.49E-02	4.88E-03	4.08E-03	4.29E-03	5.78E-03	1.26E-02	5.76E-03	3.21E-01
	Peak sensitivity value	-7.06E-17	-1.72E-16	2.10E-01	-2.93E-16	1.35E-16	-2.06E-02	-1.12E-01	-3.01E-02	-1.68E-03	-4.06E-03	-1.55E-02	-2.12E-19
	Shapiro-Wilk score	1.807E-01	1.443E-01	9.557E-01	8.531E-01	8.239E-01	9.340E-01	9.649E-01	9.467E-01	8.653E-01	8.605E-01	9.070E-01	6.847E-01
Extracellular acetaldehyde	Local	0.00E+00	0.00E+00	2.84E-01	-5.10E-15	7.94E-15	6.82E-02	-4.25E-01	-1.21E-01	-9.62E-03	-7.39E-02	1.27E-01	1.05E-17
	Min	-1.47E-13	-1.49E-12	3.19E-02	-1.58E-13	-1.45E-13	9.23E-03	-7.65E-01	-2.37E-01	-7.46E-02	-1.96E-01	-1.69E-03	-3.29E-16
	Max	4.66E-13	8.47E-13	5.76E-01	1.76E-13	1.55E-13	1.89E-01	-1.89E-01	-4.98E-02	-8.72E-04	4.64E-01	2.73E-01	5.87E-16
	Normalized peak height	6.82E-01	7.63E-01	2.90E-03	1.83E-02	3.56E-02	4.17E-03	3.44E-03	3.25E-03	7.55E-03	1.60E-02	4.09E-03	2.88E-01
	Peak sensitivity value	-1.02E-17	1.09E-15	2.86E-01	-1.11E-17	-4.83E-17	5.76E-02	-3.86E-01	-1.15E-01	-4.74E-03	-6.79E-02	1.14E-01	7.89E-20
	Shapiro-Wilk score	1.558E-01	1.176E-01	9.969E-01	9.635E-01	9.355E-01	9.793E-01	9.899E-01	9.898E-01	8.349E-01	7.393E-01	9.893E-01	8.737E-01
Phosphoenolpyruvate	Local	0.00E+00	0.00E+00	-3.77E-03	4.71E-15	-1.00E-15	-9.07E-04	-4.72E-03	-1.34E-03	-1.07E-04	-5.20E-02	6.64E-02	9.58E-18
	Min	-1.10E-13	-1.84E-13	-5.96E-02	-5.71E-14	-5.28E-14	-1.23E-02	-5.20E-02	-1.40E-02	-3.31E-03	-1.02E-01	2.21E-04	-2.51E-16
	Max	1.16E-13	1.13E-13	3.57E-03	8.89E-14	4.54E-14	1.15E-03	5.84E-03	1.24E-03	2.13E-04	2.50E-01	1.29E-01	9.13E-16
	Normalized peak height	6.21E-01	7.15E-01	1.71E-02	2.77E-02	4.24E-02	1.58E-02	1.43E-02	1.37E-02	2.73E-02	1.73E-02	3.48E-03	2.86E-01
	Peak sensitivity value	1.13E-16	-4.54E-17	-3.03E-03	6.28E-17	2.90E-17	-6.52E-04	-3.73E-03	-1.16E-03	-5.32E-05	-5.53E-02	6.88E-02	-5.15E-19
	Shapiro-Wilk score	1.596E-01	1.478E-01	6.315E-01	9.091E-01	8.851E-01	6.378E-01	6.147E-01	6.348E-01	5.588E-01	7.192E-01	9.900E-01	8.282E-01

Metabolite	Statistic	1	2	3	4	5	6	7	8	9	10	11	12
Fructose-6-Phosphate	Local	1.24E-02	5.33E-03	9.67E-01	8.44E-02	-3.38E-01	-8.95E-16	5.90E-03	1.29E-02	1.11E-03	3.34E-03	-2.13E-15	1.18E-01
	Min	4.68E-04	3.13E-04	-1.32E+00	3.54E-02	-7.57E+00	-3.27E-14	-8.27E-02	-1.95E-01	-2.22E-02	-3.42E-02	-6.84E-14	-1.79E+00
	Max	7.89E+00	1.38E+00	8.57E+00	1.90E-01	-1.31E-01	2.78E-14	2.33E-02	4.67E-02	5.59E-03	2.37E-02	1.14E-13	4.86E-01
	Normalized peak height	4.62E-01	1.81E-01	4.41E-02	3.89E-03	3.10E-02	1.08E-01	1.75E-02	1.81E-02	2.40E-02	1.50E-02	1.35E-01	2.01E-02
	Peak sensitivity value	4.41E-03	2.38E-03	9.37E-01	7.27E-02	-2.32E-01	-7.15E-18	3.86E-03	8.61E-03	6.26E-04	1.52E-03	-5.53E-17	5.87E-02
	Shapiro-Wilk score	4.465E-01	3.471E-01	6.149E-01	9.696E-01	4.945E-01	7.028E-01	7.158E-01	6.840E-01	7.216E-01	8.849E-01	7.160E-01	6.652E-01
Extracellular cyanide	Local	-1.89E-03	-8.11E-04	-1.47E-01	-6.48E-04	-7.85E-03	4.27E-16	6.15E-03	1.34E-02	1.15E-03	3.48E-03	6.32E-16	1.23E-01
	Min	-5.26E-01	-1.37E-01	-7.27E-01	-9.40E-03	-5.02E-01	-7.60E-15	4.51E-04	1.08E-03	1.08E-04	1.42E-04	-2.14E-14	1.53E-02
	Max	9.75E-05	6.69E-05	1.08E-03	-5.73E-05	-6.09E-04	4.17E-15	3.15E-02	5.42E-02	6.36E-03	2.55E-02	2.26E-14	6.69E-01
	Normalized peak height	2.86E-01	1.75E-01	6.56E-03	1.33E-02	5.74E-02	4.75E-02	5.87E-03	4.95E-03	6.84E-03	8.62E-03	1.24E-01	6.08E-03
	Peak sensitivity value	-1.66E-04	-1.39E-04	-7.14E-02	-3.24E-04	-3.37E-03	-1.76E-18	3.29E-03	9.07E-03	6.43E-04	1.80E-03	-2.89E-18	7.25E-02
	Shapiro-Wilk score	4.372E-01	4.390E-01	8.902E-01	7.599E-01	3.889E-01	8.005E-01	8.925E-01	9.223E-01	8.968E-01	8.457E-01	6.597E-01	8.737E-01
Pyruvate	Local	9.01E-01	3.88E-01	7.04E+01	2.28E-01	2.76E+00	-6.96E-14	4.07E-02	8.91E-02	7.64E-03	2.31E-02	-7.79E+01	8.15E-01
	Min	1.31E-03	9.71E-04	-7.50E-03	2.10E-03	3.57E-02	-6.95E-12	-3.71E-01	-1.13E+00	-6.90E-02	-1.48E-01	-4.06E+04	-5.53E+00
	Max	2.99E+04	1.04E+03	1.79E+04	9.63E+01	6.37E+03	8.56E-12	6.20E+01	1.59E+02	1.15E+01	1.38E+01	-2.09E+00	1.66E+03
	Normalized peak height	9.90E-01	9.49E-01	8.81E-01	8.95E-01	9.68E-01	4.87E-01	5.68E-01	9.67E-01	6.59E-01	7.89E-01	9.36E-01	9.52E-01
	Peak sensitivity value	1.50E+01	5.19E-01	8.96E+00	5.02E-02	3.22E+00	7.69E-15	-2.74E-02	6.94E-02	6.13E-03	-1.96E-03	-2.24E+01	2.99E-01
	Shapiro-Wilk score	3.696E-03	1.913E-02	2.646E-02	2.952E-02	1.451E-02	5.595E-02	1.460E-02	1.339E-02	1.510E-02	2.546E-02	2.306E-02	1.444E-02
Extracellular ethanol	Local	1.01E-02	4.37E-03	7.92E-01	2.32E-03	2.81E-02	-3.81E-16	9.18E-03	2.01E-02	1.72E-03	5.20E-03	-1.74E-16	1.84E-01
	Min	4.11E-04	2.03E-04	1.35E-03	4.08E-05	4.55E-04	-5.41E-15	1.06E-03	2.75E-03	2.25E-04	3.43E-04	-1.62E-14	3.90E-02
	Max	8.54E-01	3.49E-01	9.27E-01	1.69E-02	8.17E-01	3.61E-15	3.92E-02	7.83E-02	7.84E-03	3.08E-02	1.81E-14	7.26E-01
	Normalized peak height	1.06E-01	6.54E-02	5.95E-03	8.90E-03	3.20E-02	3.89E-02	5.08E-03	4.64E-03	5.20E-03	6.60E-03	1.34E-01	4.74E-03
	Peak sensitivity value	1.69E-03	1.43E-03	8.48E-01	2.18E-03	2.29E-02	4.09E-18	6.77E-03	1.50E-02	1.22E-03	2.40E-03	2.66E-18	1.15E-01
	Shapiro-Wilk score	4.848E-01	5.047E-01	7.408E-01	8.669E-01	4.260E-01	8.666E-01	9.310E-01	9.414E-01	9.158E-01	8.900E-01	8.484E-01	8.805E-01
Extracellular glycerol	Local	2.51E-02	1.08E-02	1.96E+00	8.63E-03	1.04E-01	8.14E-15	-8.18E-02	-1.79E-01	-1.53E-02	-4.63E-02	1.49E-14	-1.64E+00
	Min	-3.74E-03	-5.72E-03	-4.57E-02	9.01E-04	3.32E-02	-7.76E-14	-2.57E-01	-3.87E-01	-4.25E-02	-1.97E-01	-3.70E-13	-2.72E+00
	Max	2.60E+00	9.12E-01	2.94E+00	5.14E-02	2.43E+00	6.22E-14	-5.52E-03	-2.31E-02	-1.83E-03	-2.37E-03	3.37E-13	-9.57E-01
	Normalized peak height	1.27E-01	7.42E-02	4.96E-03	8.42E-03	2.90E-02	2.45E-02	3.63E-03	2.94E-03	4.30E-03	5.15E-03	1.25E-01	3.40E-03
	Peak sensitivity value	2.76E-03	3.00E-03	1.70E+00	7.44E-03	7.28E-02	-6.78E-17	-6.39E-02	-1.73E-01	-1.35E-02	-3.74E-02	-3.46E-16	-1.41E+00
	Shapiro-Wilk score	4.886E-01	5.039E-01	8.284E-01	8.731E-01	4.258E-01	8.943E-01	9.732E-01	9.940E-01	9.742E-01	9.460E-01	7.589E-01	9.771E-01
Extracellular glucose	Local	2.57E+00	-1.40E-02	-2.53E+00	-3.55E-03	-4.30E-02	8.97E-17	-5.98E-04	-1.31E-03	-1.12E-04	-3.38E-04	2.12E-16	-1.20E-02
	Min	6.16E-01	-1.79E+00	-1.46E+01	-9.51E-02	-7.30E+00	-2.47E-14	-8.99E-02	-2.33E-01	-1.78E-02	-4.59E-02	-2.87E-14	-2.06E+00
	Max	1.49E+01	-2.01E-04	-2.04E-02	-2.53E-04	-2.77E-03	2.99E-14	1.35E-02	2.73E-02	2.35E-03	1.07E-02	4.54E-14	2.83E-01
	Normalized peak height	6.98E-03	1.40E-01	8.07E-03	2.53E-02	1.59E-01	3.48E-01	1.63E-01	1.85E-01	1.60E-01	1.58E-01	3.59E-01	1.69E-01
	Peak sensitivity value	1.17E+00	-2.89E-03	-1.15E+00	-1.06E-03	-1.37E-02	-2.72E-17	-1.92E-04	-3.93E-04	-3.90E-05	-1.10E-04	2.75E-18	-4.17E-03
	Shapiro-Wilk score	8.265E-01	4.886E-01	8.016E-01	6.601E-01	4.165E-01	2.740E-01	4.047E-01	4.007E-01	3.884E-01	4.707E-01	3.829E-01	3.678E-01
1-3-Bisphosphoglycerate	Local	1.07E-02	4.61E-03	8.37E-01	4.44E-02	5.38E-01	5.30E-16	8.28E-03	1.81E-02	-2.84E-01	-8.56E-01	3.50E-14	1.66E-01
	Min	-3.66E+00	-8.44E-01	-6.01E+00	4.04E-04	2.62E-01	-1.06E-13	7.87E-04	2.17E-03	-7.65E-01	-1.23E+00	-1.45E-13	2.98E-02
	Max	1.06E+00	3.63E-01	1.20E+00	1.08E-01	5.81E+00	8.00E-14	1.19E-01	2.06E-01	-1.14E-01	-2.72E-01	1.60E-13	2.30E+00
	Normalized peak height	3.02E-01	1.78E-01	1.66E-02	4.92E-03	1.87E-02	5.96E-02	1.56E-02	1.26E-02	4.11E-03	4.03E-03	6.16E-02	1.42E-02
	Peak sensitivity value	1.41E-03	1.26E-03	1.00E+00	3.81E-02	3.93E-01	1.19E-17	5.47E-03	1.37E-02	-3.15E-01	-8.48E-01	9.25E-17	9.23E-02
	Shapiro-Wilk score	5.073E-01	4.096E-01	7.219E-01	9.828E-01	5.961E-01	7.197E-01	7.552E-01	7.690E-01	9.860E-01	9.994E-01	9.464E-01	7.318E-01
Fructose 1-6-bisphosphate	Local	4.60E-02	1.98E-02	3.59E+00	2.43E-02	2.94E-01	-9.76E-02	-8.09E-02	-1.00E+00	-8.59E-02	-2.59E-01	2.20E-14	-2.18E+00
	Min	-3.23E-01	-1.62E-01	-1.34E+00	1.94E-03	8.32E-02	-3.48E-01	-5.30E-01	-1.81E+00	-2.23E-01	-9.15E-01	-5.94E-13	-5.99E+00
	Max	5.76E+00	1.88E+00	7.43E+00	1.15E-01	6.42E+00	-2.51E-02	2.74E-01	-2.58E-01	-2.21E-02	-1.76E-02	6.39E-13	-4.25E-01
	Normalized peak height	1.73E-01	9.08E-02	7.08E-03	6.50E-03	2.59E-02	4.34E-03	3.54E-03	2.93E-03	4.25E-03	4.09E-03	1.18E-01	3.81E-03
	Peak sensitivity value	8.17E-03	6.45E-03	3.00E+00	1.97E-02	2.13E-01	-8.59E-02	-8.93E-02	-9.91E-01	-6.96E-02	-1.87E-01	-4.18E-16	-1.39E+00
	Shapiro-Wilk score	4.824E-01	4.934E-01	9.011E-01	9.117E-01	4.794E-01	9.539E-01	9.982E-01	9.974E-01	9.783E-01	9.498E-01	7.650E-01	9.561E-01
AMP	Local	4.17E-03	1.79E-03	3.25E-01	-6.74E-02	-8.16E-01	2.40E-15	-1.26E-02	-2.76E-02	-2.37E-03	-7.14E-03	6.43E-15	-2.52E-01
	Min	-3.08E-02	-9.24E-03	-2.52E-01	-1.47E-01	-1.06E+01	-4.06E-14	-1.44E-01	-3.10E-01	-2.26E-02	-8.07E-02	-1.60E-13	-2.51E+00
	Max	1.04E+01	1.32E+00	9.42E+00	-6.62E-04	-4.30E-01	5.21E-14	-1.58E-03	-3.68E-03	-2.54E-04	-5.58E-04	3.32E-13	-4.89E-02
	Normalized peak height	3.81E-01	2.98E-01	2.45E-02	4.83E-03	2.27E-02	9.30E-02	1.22E-02	1.20E-02	1.00E-02	1.08E-02	1.61E-01	1.05E-02
	Peak sensitivity value	5.56E-03	7.46E-04	1.37E-02	-6.01E-02	-6.08E-01	4.27E-17	-9.07E-03	-1.79E-02	-1.54E-03	-3.48E-03	-7.29E-17	-1.44E-01
	Shapiro-Wilk score	3.268E-01	2.779E-01	5.962E-01	9.846E-01	6.040E-01	7.932E-01	7.844E-01	7.885E-01	7.988E-01	8.307E-01	7.717E-01	7.463E-01
ATP	Local	-1.59E-03	-6.83E-04	-1.24E-01	2.57E-02	3.11E-01	-8.56E-16	4.80E-03	1.05E-02	9.01E-04	2.72E-03	-2.17E-15	9.61E-02
	Min	-6.16E+00	-1.08E+00	-8.78E+00	1.08E-03	1.05E-01	-4.02E-14	6.33E-04	1.48E-03	9.83E-05	1.71E-04	-8.87E-14	1.57E-02
	Max	1.28E-02	2.41E-03	9.83E-02	9.11E-02	7.51E+00	7.36E-14	1.07E-01	1.97E-01	2.60E-02	4.58E-02	1.26E-13	2.43E+00
	Normalized peak height	6.86E-01	5.67E-01	5.74E-02	5.63E-03	3.15E-02	1.25E-01	2.14E-02	1.74E-02	2.78E-02	1.58E-02	1.23E-01	2.47E-02
	Peak sensitivity value	-2.61E-03	-2.86E-04	-3.80E-03	2.21E-02	2.27E-01	2.69E-17	2.81E-03	7.63E-03	5.77E-04	1.43E-03	3.34E-17	5.08E-02
	Shapiro-Wilk score	2.788E-01	2.185E-01	4.244E-01	9.341E-01	4.826E-01	5.583E-01	5.859E-01	5.862E-01	5.806E-01	7.099E-01	6.584E-01	5.506E-01
NADH	Local	9.35E-03	4.03E-03	7.30E-01	-1.86E-03	-2.25E-02	6.21E-15	1.54E-01	3.36E-01	2.88E-02	8.69E-02	1.30E-14	-1.28E+00
	Min	1.46E-04	9.50E-05	-1.84E-01	-1.16E-02	-4.64E-01	-6.39E-14	2.39E-02	1.18E-01	5.72E-03	7.61E-03	-2.73E-13	-1.93E+00
	Max	1.20E+00	4.02E-01	1.35E+00	1.38E-02	1.00E+00	7.57E-14	4.26E-01	5.90E-01	8.02E-02	2.66E-01	3.58E-13	-3.55E-01
	Normalized peak height	1.48E-01	9.39E-02	3.60E-03	6.12E-03	2.77E-02	2.59E-02	4.29E-03	3.89E-03	3.98E-03	3.58E-03	1.62E-01	4.73E-03
	Peak sensitivity value	1.95E-03	1.10E-03	5.86E-01	-1.09E-03	-1.48E-02	-3.61E-17	1.29E-01	3.30E-01	2.44E-02	6.29E-02	2.72E-16	-1.19E+00
	Shapiro-Wilk score	4.857E-01	4.991E-01	9.821E-01	9.847E-01	4.938E-01	8.612E-01	9.788E-01	9.998E-01	9.752E-01	9.637E-01	7.592E-01	9.966E-01

Metabolite	Statistic	13	14	15	16	17	18	19	20	21	22	23	24
Fructose-6-Phosphate	Local	0.00E+00	0.00E+00	-2.01E-02	-1.59E-15	-7.98E-17	-4.83E-03	-2.51E-02	-7.13E-03	-5.69E-04	-7.58E-01	-5.75E-02	-2.80E-17
	Min	-1.21E-13	-1.96E-13	-8.83E-02	-1.86E-13	-1.00E-13	-1.75E-02	-7.40E-02	-1.93E-02	-5.15E-03	-8.96E-01	-2.05E-01	-2.02E-15
	Max	4.43E-14	9.84E-14	3.49E-01	1.58E-13	8.31E-14	5.43E-02	2.51E-01	7.19E-02	1.54E-02	1.89E-01	3.36E-01	8.45E-16
	Normalized peak height	6.15E-01	7.21E-01	2.05E-02	6.37E-02	5.38E-02	1.43E-02	1.43E-02	1.38E-02	3.09E-02	1.02E-02	8.62E-03	2.90E-01
	Peak sensitivity value	-1.21E-17	6.10E-17	-1.45E-02	-1.13E-16	-6.47E-17	-3.51E-03	-2.00E-02	-5.73E-03	-2.19E-04	-7.66E-01	-5.54E-02	-1.32E-18
	Shapiro-Wilk score	1.900E-01	1.513E-01	7.183E-01	7.593E-01	7.468E-01	7.356E-01	6.919E-01	7.171E-01	6.830E-01	8.279E-01	9.101E-01	8.514E-01
Extracellular cyanide	Local	0.00E+00	0.00E+00	-2.09E-02	3.66E-16	-5.96E-16	-5.03E-03	3.14E-02	-6.50E-02	7.46E-02	5.46E-03	-9.35E-03	-7.74E-19
	Min	-1.50E-14	-6.82E-14	-9.22E-02	-2.20E-14	-2.03E-14	-1.75E-02	2.88E-03	-2.82E-01	1.33E-02	-5.85E-02	-4.92E-02	-3.90E-17
	Max	9.97E-15	5.29E-14	-1.52E-03	4.26E-14	2.86E-14	-8.21E-04	2.07E-01	-1.23E-02	3.48E-01	4.06E-02	1.20E-04	2.12E-17
	Normalized peak height	6.34E-01	8.92E-01	5.16E-03	4.19E-02	6.47E-02	4.49E-03	7.17E-03	5.47E-03	5.45E-03	1.88E-02	6.02E-03	2.86E-01
	Peak sensitivity value	2.42E-19	2.39E-17	-1.41E-02	1.96E-17	3.56E-18	-4.15E-03	1.99E-02	-5.24E-02	5.90E-02	3.21E-03	-5.92E-03	4.48E-21
	Shapiro-Wilk score	1.487E-01	1.088E-01	9.136E-01	8.611E-01	8.220E-01	9.425E-01	8.372E-01	9.089E-01	8.986E-01	8.530E-01	8.871E-01	8.470E-01
Pyruvate	Local	0.00E+00	0.00E+00	-1.39E-01	-5.18E-14	1.49E-13	-3.34E-02	-1.73E-01	-4.93E-02	-3.93E-03	-1.87E+00	4.59E+00	2.26E-16
	Min	-6.70E-11	-2.32E-12	-2.14E+02	-3.79E-11	-2.26E-11	-5.31E+01	-2.20E+02	-6.89E+01	-9.49E+00	-7.10E+02	9.85E-04	-4.65E-14
	Max	2.13E-12	9.63E-11	1.76E+00	5.53E-11	3.45E-11	2.29E-01	1.09E+00	3.20E-01	3.25E-02	3.66E+02	2.52E+03	1.86E-12
	Normalized peak height	9.96E-01	9.94E-01	9.59E-01	5.58E-01	8.44E-01	9.52E-01	5.77E-01	9.04E-01	9.49E-01	8.60E-01	9.40E-01	9.95E-01
	Peak sensitivity value	2.38E-14	-7.11E-15	-7.59E-02	4.43E-14	-1.03E-14	-1.12E-02	9.29E-02	7.89E-03	-8.08E-04	-5.74E-01	1.26E+00	1.75E-16
	Shapiro-Wilk score	7.160E-04	1.046E-03	1.528E-02	4.877E-02	5.022E-02	1.428E-02	1.815E-02	1.558E-02	1.295E-02	2.678E-02	1.847E-02	1.168E-03
Extracellular ethanol	Local	-3.41E-16	-1.00E+00	-3.13E-02	-1.73E-16	1.29E-15	-7.52E-03	-3.91E-02	-1.11E-02	-8.85E-04	-1.89E-02	5.21E-02	2.13E-18
	Min	-8.35E-15	-1.00E+00	-1.21E-01	-3.62E-14	-1.67E-14	-2.69E-02	-1.07E-01	-2.67E-02	-7.30E-03	-8.55E-02	9.54E-05	-5.69E-17
	Max	1.06E-14	-1.00E+00	-4.93E-03	2.49E-14	2.35E-14	-1.58E-03	-1.24E-02	-3.67E-03	-5.50E-05	1.74E-01	1.19E-01	3.08E-16
	Normalized peak height	4.66E-01	1.00E+00	4.83E-03	2.71E-02	3.53E-02	4.24E-03	3.70E-03	3.70E-03	7.77E-03	1.34E-02	3.26E-03	2.92E-01
	Peak sensitivity value	-2.89E-18	-1.00E+00	-2.14E-02	-2.67E-17	7.28E-18	-6.58E-03	-2.82E-02	-1.03E-02	-4.35E-04	-2.18E-02	5.27E-02	-7.94E-20
	Shapiro-Wilk score	5.971E-01	1.000E+00	9.382E-01	9.162E-01	8.914E-01	9.459E-01	9.638E-01	9.728E-01	8.326E-01	7.974E-01	9.980E-01	7.252E-01
Extracellular glycerol	Local	0.00E+00	0.00E+00	2.79E-01	9.72E-15	-1.00E+00	6.70E-02	3.48E-01	9.89E-02	7.89E-03	-7.27E-02	1.24E-01	1.03E-17
	Min	-2.98E-13	-1.99E-12	3.01E-02	-3.20E-13	-1.00E+00	9.60E-03	8.19E-02	1.74E-02	1.19E-03	-2.00E-01	-3.52E-03	-2.65E-16
	Max	2.10E-13	1.97E-12	5.77E-01	3.26E-13	-1.00E+00	1.79E-01	5.93E-01	2.34E-01	2.99E-02	4.83E-01	2.51E-01	9.23E-16
	Normalized peak height	6.17E-01	9.37E-01	2.96E-03	2.16E-02	1.00E+00	4.09E-03	3.38E-03	3.48E-03	4.67E-03	1.77E-02	3.77E-03	2.90E-01
	Peak sensitivity value	2.02E-16	9.30E-16	2.75E-01	-3.15E-16	-1.00E+00	6.37E-02	3.56E-01	8.99E-02	5.11E-03	-7.06E-02	1.14E-01	-4.59E-19
	Shapiro-Wilk score	1.911E-01	1.315E-01	9.972E-01	9.417E-01	1.000E+00	9.802E-01	9.976E-01	9.942E-01	9.361E-01	7.382E-01	9.883E-01	7.809E-01
Extracellular glucose	Local	0.00E+00	0.00E+00	2.04E-03	1.65E-16	1.51E-17	4.89E-04	2.54E-03	7.23E-04	5.76E-05	2.55E-02	5.83E-03	-3.53E-18
	Min	-1.07E-14	-5.10E-14	-5.04E-02	-1.46E-13	-1.04E-13	-9.85E-03	-4.72E-02	-9.95E-03	-2.66E-03	-9.60E-01	-8.71E-02	-6.16E-16
	Max	9.70E-15	2.83E-14	3.35E-01	2.16E-13	9.92E-14	7.99E-02	2.62E-01	7.92E-02	1.61E-02	3.16E-01	2.76E-01	7.10E-16
	Normalized peak height	7.07E-01	8.07E-01	1.72E-01	3.48E-01	3.99E-01	1.63E-01	1.10E-01	1.15E-01	2.71E-01	4.70E-02	5.67E-02	3.19E-01
	Peak sensitivity value	1.83E-18	3.09E-17	6.71E-04	1.67E-16	-4.26E-17	1.53E-04	8.74E-04	2.53E-04	1.84E-05	1.05E-02	2.48E-03	-4.74E-19
	Shapiro-Wilk score	1.850E-01	9.235E-02	4.147E-01	2.617E-01	2.815E-01	3.989E-01	4.168E-01	4.397E-01	3.368E-01	5.217E-01	5.856E-01	5.789E-01
1-3-Bisphosphoglycerate	Local	0.00E+00	0.00E+00	-2.82E-02	6.06E-16	-4.86E-16	-6.78E-03	-3.52E-02	-1.00E-02	-7.98E-04	-3.98E-01	-7.28E-03	7.56E-17
	Min	-7.77E-14	-2.07E-13	-3.16E-01	-2.89E-13	-1.24E-13	-6.26E-02	-2.76E-01	-7.10E-02	-1.45E-02	-7.12E-01	-2.85E-01	-1.74E-15
	Max	6.74E-14	2.69E-13	-3.36E-03	1.49E-13	1.51E-13	-1.29E-03	-9.40E-03	-2.78E-03	-4.11E-05	7.65E-03	2.59E-02	3.55E-15
	Normalized peak height	5.81E-01	7.84E-01	1.16E-02	3.98E-02	3.55E-02	1.02E-02	9.19E-03	9.25E-03	1.59E-02	1.13E-02	9.96E-03	2.87E-01
	Peak sensitivity value	2.67E-17	-1.29E-16	-2.16E-02	-5.31E-17	1.44E-16	-4.99E-03	-2.85E-02	-8.54E-03	-3.37E-04	-4.01E-01	-6.64E-04	8.92E-19
	Shapiro-Wilk score	2.271E-01	1.515E-01	7.809E-01	8.197E-01	7.910E-01	7.922E-01	7.786E-01	7.907E-01	6.970E-01	8.860E-01	7.766E-01	8.926E-01
Fructose 1-6-bisphosphate	Local	0.00E+00	0.00E+00	-9.76E-01	1.28E-14	-8.40E-15	8.90E-02	4.63E-01	1.31E-01	1.05E-02	-2.10E-01	2.16E-01	3.37E-17
	Min	-5.60E-13	-1.93E-12	-2.62E+00	-6.25E-13	-5.15E-13	1.06E-02	7.45E-02	1.71E-02	7.22E-04	-5.19E-01	-8.10E-02	-9.44E-16
	Max	2.63E-13	1.11E-12	-2.38E-01	5.95E-13	4.36E-13	3.23E-01	1.05E+00	3.95E-01	5.95E-02	8.85E-01	5.29E-01	5.48E-15
	Normalized peak height	6.45E-01	7.96E-01	3.44E-03	3.04E-02	4.95E-02	4.30E-03	3.29E-03	4.15E-03	5.91E-03	1.26E-02	4.84E-03	2.90E-01
	Peak sensitivity value	2.17E-16	1.12E-15	-9.12E-01	2.17E-16	-1.44E-16	7.44E-02	3.78E-01	1.15E-01	5.51E-03	-2.04E-01	1.97E-01	-2.60E-18
	Shapiro-Wilk score	1.881E-01	1.490E-01	9.723E-01	8.984E-01	8.668E-01	9.572E-01	9.867E-01	9.705E-01	8.925E-01	8.522E-01	9.887E-01	7.066E-01
AMP	Local	0.00E+00	0.00E+00	4.30E-02	3.70E-15	2.03E-16	1.03E-02	5.37E-02	1.52E-02	1.22E-03	6.08E-01	1.23E-01	6.38E-17
	Min	-5.05E-14	-3.27E-13	6.60E-03	-2.64E-13	-2.08E-13	2.00E-03	1.44E-02	4.29E-03	6.65E-05	7.24E-02	2.68E-02	-2.53E-15
	Max	1.11E-13	5.28E-13	5.13E-01	1.96E-13	1.94E-13	1.08E-01	3.92E-01	1.01E-01	2.25E-02	1.14E+00	5.02E-01	7.41E-15
	Normalized peak height	5.47E-01	7.94E-01	1.29E-02	3.25E-02	4.08E-02	1.12E-02	9.00E-03	8.73E-03	1.65E-02	1.64E-02	6.12E-03	2.89E-01
	Peak sensitivity value	3.27E-17	1.91E-17	3.07E-02	3.64E-17	1.30E-16	7.88E-03	4.37E-02	1.27E-02	6.17E-04	6.16E-01	1.18E-01	-2.88E-18
	Shapiro-Wilk score	2.847E-01	1.547E-01	8.011E-01	7.935E-01	7.695E-01	8.045E-01	7.977E-01	8.062E-01	7.102E-01	7.363E-01	9.287E-01	7.706E-01
ATP	Local	0.00E+00	0.00E+00	-1.64E-02	-1.37E-15	-6.97E-17	-3.93E-03	-2.04E-02	-5.81E-03	-4.63E-04	-2.32E-01	-4.68E-02	2.55E-17
	Min	-2.68E-14	-7.99E-14	-3.16E-01	-3.76E-13	-2.12E-13	-8.40E-02	-3.45E-01	-8.59E-02	-2.07E-02	-8.80E-01	-3.52E-01	-7.70E-16
	Max	5.22E-14	1.13E-13	-2.38E-03	2.07E-13	2.51E-13	-6.19E-04	-4.94E-03	-1.35E-03	-2.31E-05	-1.17E-01	-2.21E-02	8.93E-16
	Normalized peak height	5.40E-01	6.85E-01	1.81E-02	9.10E-02	1.11E-01	2.01E-02	1.82E-02	1.64E-02	3.63E-02	7.88E-03	9.43E-03	2.89E-01
	Peak sensitivity value	-3.68E-17	7.32E-17	-1.32E-02	2.73E-16	1.17E-16	-2.91E-03	-1.39E-02	-4.60E-03	-1.78E-04	-2.28E-01	-4.57E-02	5.58E-19
	Shapiro-Wilk score	2.878E-01	1.599E-01	6.024E-01	5.592E-01	5.427E-01	6.028E-01	5.951E-01	6.083E-01	5.179E-01	8.403E-01	7.683E-01	9.276E-01
NADH	Local	0.00E+00	0.00E+00	-5.23E-01	6.20E-15	-5.37E-15	5.25E-02	2.73E-01	7.75E-02	6.18E-03	1.86E-02	5.39E-02	-5.02E-18
	Min	-4.68E-13	-1.15E-12	-7.72E-01	-1.79E-13	-1.94E-13	4.58E-03	3.51E-02	7.32E-03	8.65E-04	-6.48E-02	3.30E-04	-6.12E-16
	Max	3.01E-13	1.77E-12	-1.57E-01	1.80E-13	1.72E-13	1.43E-01	5.22E-01	1.95E-01	2.11E-02	2.48E-01	1.40E-01	1.08E-16
	Normalized peak height	6.90E-01	9.07E-01	5.00E-03	1.40E-02	3.19E-02	3.62E-03	3.75E-03	3.21E-03	4.31E-03	7.46E-03	4.62E-03	3.06E-01
	Peak sensitivity value	3.83E-16	1.01E-15	-5.19E-01	1.45E-17	1.19E-17	4.50E-02	2.80E-01	7.45E-02	4.77E-03	1.68E-02	4.55E-02	-2.82E-19
	Shapiro-Wilk score	1.484E-01	1.318E-01	9.806E-01	9.550E-01	9.299E-01	9.846E-01	9.909E-01	9.947E-01	9.446E-01	9.578E-01	9.892E-01	7.534E-01

Table S22: Concentration control coefficients for *Saccharomyces cerevisiae* model with parameter variation of ±40%

	(GlucoseMixedflowtoextracellularmedium)	(Glucoseuptake)	(Hexokinase)	(Phosphoglucoisomerase)	(Phosphofructokinase)	(Aldolase)	(Triosephosphateisomerase)	(Glyceraldehyde3-phosphatedehydrogenase)
Acetaldehyde<br>Local<br>Min<br>Max<br>Normalized peak height<br>Peak sensitivity value<br>Shapiro-Wilk score	2.55E-02<br>-1.44E-01<br>2.83E+00<br>1.66E-01<br>3.53E-03<br>5.602E-01	1.10E-02<br>-4.80E-02<br>1.38E+00<br>1.07E-01<br>2.58E-03<br>4.789E-01	1.99E+00<br>-9.91E-01<br>3.26E+00<br>1.05E-02<br>9.61E-03<br>8.844E-01	8.76E-03<br>8.95E-05<br>7.61E-02<br>9.72E-03<br>6.51E-03<br>8.047E-01	1.06E-01<br>2.56E-02<br>2.69E+00<br>2.60E-02<br>6.41E-02<br>5.108E-01	-5.75E-15<br>-8.88E-14<br>8.02E-14<br>5.92E-02<br>4.68E-17<br>7.784E-01	-8.30E-02<br>-4.16E-01<br>-1.23E-03<br>4.61E-03<br>-6.19E-02<br>9.463E-01	-1.82E-01<br>-4.69E-01<br>-1.52E-02<br>2.93E-03<br>-1.55E-01<br>9.884E-01
Cytosolic glucose<br>Local<br>Min<br>Max<br>Normalized peak height<br>Peak sensitivity value<br>Shapiro-Wilk score	2.04E+00<br>-2.95E+01<br>1.13E+04<br>7.67E-01<br>-1.11E+00<br>1.193E-02	8.77E-01<br>-2.39E+01<br>1.76E+03<br>7.04E-01<br>2.65E-01<br>1.906E-02	-3.34E+00<br>-1.35E+04<br>6.52E+01<br>5.87E-01<br>-9.19E+00<br>1.274E-02	7.30E-03<br>-6.13E+00<br>8.29E+00<br>5.93E-01<br>4.51E-03<br>1.655E-01	8.83E-02<br>-6.24E+01<br>1.40E+02<br>5.48E-01<br>3.17E-02<br>3.107E-01	4.39E-16<br>-6.98E-13<br>4.69E-13<br>4.95E-01<br>5.58E-16<br>1.090E-01	-2.81E-03<br>-2.55E+00<br>7.42E-01<br>5.62E-01<br>-1.58E-03<br>1.494E-01	-6.15E-03<br>-5.66E+00<br>2.06E+00<br>5.64E-01<br>-3.75E-03<br>1.457E-01
Glycerol<br>Local<br>Min<br>Max<br>Normalized peak height<br>Peak sensitivity value<br>Shapiro-Wilk score	2.51E-02<br>-1.11E-01<br>2.85E+00<br>1.88E-01<br>2.55E-03<br>5.593E-01	1.08E-02<br>-1.14E-01<br>1.41E+00<br>1.17E-01<br>2.58E-03<br>4.679E-01	1.96E+00<br>-8.79E-02<br>3.26E+00<br>8.75E-03<br>1.09E-02<br>8.778E-01	8.63E-03<br>1.15E-04<br>6.71E-02<br>8.77E-03<br>6.38E-03<br>8.112E-01	1.04E-01<br>2.69E-02<br>2.77E+00<br>2.77E-02<br>6.94E-02<br>5.139E-01	8.13E-15<br>-8.50E-14<br>1.13E-13<br>5.48E-02<br>6.92E-17<br>8.286E-01	-8.18E-02<br>-3.54E-01<br>-1.01E-03<br>4.13E-03<br>-6.66E-02<br>9.438E-01	-1.79E-01<br>-4.64E-01<br>-1.40E-02<br>2.84E-03<br>-1.80E-01<br>9.875E-01
EtOH<br>Local<br>Min<br>Max<br>Normalized peak height<br>Peak sensitivity value<br>Shapiro-Wilk score	1.01E-02<br>-1.47E-02<br>9.14E-01<br>1.28E-01<br>1.51E-03<br>5.547E-01	4.37E-03<br>-9.81E-03<br>4.63E-01<br>7.92E-02<br>8.25E-04<br>4.676E-01	7.92E-01<br>-7.72E-02<br>9.44E-01<br>5.81E-03<br>5.97E-03<br>7.959E-01	2.32E-03<br>-8.27E-04<br>2.53E-02<br>1.02E-02<br>2.11E-03<br>7.900E-01	2.81E-02<br>-8.91E-03<br>9.22E-01<br>2.76E-02<br>2.13E-02<br>4.946E-01	-3.86E-16<br>-6.55E-15<br>6.28E-15<br>5.90E-02<br>-2.37E-18<br>7.829E-01	9.18E-03<br>4.88E-04<br>5.51E-02<br>6.20E-03<br>4.23E-03<br>8.831E-01	2.01E-02<br>1.39E-03<br>9.64E-02<br>4.87E-03<br>1.31E-02<br>9.049E-01
ADP<br>Local<br>Min<br>Max<br>Normalized peak height<br>Peak sensitivity value<br>Shapiro-Wilk score	1.29E-03<br>-1.47E+00<br>3.11E+00<br>5.87E-01<br>2.03E-03<br>4.373E-01	5.55E-04<br>-3.94E-01<br>5.05E-01<br>4.87E-01<br>-9.47E-06<br>4.338E-01	1.01E-01<br>-2.17E+00<br>2.30E+00<br>6.56E-02<br>6.76E-05<br>7.225E-01	-2.09E-02<br>-7.11E-02<br>1.71E-02<br>5.83E-03<br>-1.61E-02<br>9.826E-01	-2.53E-01<br>-2.80E+00<br>1.83E+00<br>2.74E-02<br>-1.64E-01<br>7.382E-01	6.63E-16<br>-1.42E-14<br>2.17E-14<br>1.15E-01<br>1.06E-17<br>7.781E-01	-3.90E-03<br>-3.83E-02<br>3.47E-02<br>1.80E-02<br>-2.29E-03<br>8.636E-01	-8.54E-03<br>-6.09E-02<br>5.80E-02<br>1.36E-02<br>-4.95E-03<br>8.657E-01
Glyceraldehyde 3-phosphate<br>Local<br>Min<br>Max<br>Normalized peak height<br>Peak sensitivity value<br>Shapiro-Wilk score	2.26E-02<br>-6.41E-01<br>7.62E+00<br>2.70E-01<br>7.07E-03<br>5.573E-01	9.73E-03<br>-1.70E-01<br>1.66E+00<br>1.55E-01<br>1.77E-03<br>4.759E-01	1.76E+00<br>-9.85E-01<br>6.08E+00<br>1.66E-02<br>7.83E-03<br>9.369E-01	1.27E-02<br>8.79E-05<br>9.79E-02<br>8.34E-03<br>8.75E-03<br>8.571E-01	1.54E-01<br>3.25E-02<br>3.49E+00<br>2.21E-02<br>9.64E-02<br>5.545E-01	6.19E-15<br>-6.69E-14<br>6.00E-14<br>4.29E-02<br>-4.76E-17<br>8.460E-01	1.56E-01<br>1.24E-02<br>5.58E-01<br>4.17E-03<br>1.32E-01<br>9.578E-01	-5.55E-01<br>-1.55E+00<br>-6.10E-02<br>3.59E-03<br>-5.51E-01<br>9.914E-01
Dihydroxyacetone phosphate<br>Local<br>Min<br>Max<br>Normalized peak height<br>Peak sensitivity value<br>Shapiro-Wilk score	2.40E-02<br>-7.12E-01<br>4.46E+00<br>2.60E-01<br>4.28E-03<br>5.558E-01	1.03E-02<br>-9.95E-02<br>1.66E+00<br>1.30E-01<br>1.50E-03<br>4.688E-01	1.88E+00<br>-1.25E+00<br>4.93E+00<br>1.28E-02<br>1.27E-02<br>9.207E-01	1.23E-02<br>1.06E-04<br>1.11E-01<br>9.21E-03<br>9.55E-03<br>8.541E-01	1.49E-01<br>3.21E-02<br>3.53E+00<br>2.37E-02<br>1.00E-01<br>5.489E-01	5.18E-15<br>-5.59E-14<br>6.32E-14<br>5.08E-02<br>3.96E-17<br>8.433E-01	-2.24E-01<br>-8.37E-01<br>-1.02E-02<br>3.70E-03<br>-1.93E-01<br>9.614E-01	-4.91E-01<br>-1.22E+00<br>-6.95E-02<br>3.19E-03<br>-5.37E-01<br>9.954E-01
Glucose-6-Phosphate<br>Local<br>Min<br>Max<br>Normalized peak height<br>Peak sensitivity value<br>Shapiro-Wilk score	1.37E-02<br>-1.73E-01<br>1.17E+01<br>3.02E-01<br>-1.55E-03<br>4.719E-01	5.88E-03<br>-6.54E-01<br>2.25E+00<br>2.48E-01<br>2.90E-03<br>4.448E-01	1.07E+00<br>-4.68E+00<br>1.02E+01<br>5.49E-02<br>1.01E+00<br>6.746E-01	-2.72E-02<br>-1.32E-01<br>2.19E-07<br>5.81E-03<br>-2.08E-02<br>9.342E-01	-3.29E-01<br>-1.14E+01<br>4.09E-04<br>4.00E-02<br>-2.11E-01<br>5.504E-01	-8.33E-16<br>-3.43E-14<br>8.98E-14<br>1.72E-01<br>-9.48E-18<br>4.961E-01	5.10E-03<br>-1.47E-01<br>2.93E-02<br>2.67E-02<br>2.49E-03<br>6.849E-01	1.11E-02<br>-3.41E-01<br>6.74E-02<br>2.68E-02<br>5.98E-03<br>7.005E-01
NAD<br>Local<br>Min<br>Max<br>Normalized peak height<br>Peak sensitivity value<br>Shapiro-Wilk score	-4.87E-03<br>-1.65E+00<br>4.01E-02<br>2.41E-01<br>-1.32E-03<br>4.768E-01	-2.10E-03<br>-5.71E-01<br>2.07E-02<br>2.76E-01<br>-3.67E-04<br>4.139E-01	-3.80E-01<br>-1.79E+00<br>6.54E-02<br>1.90E-02<br>-2.16E-03<br>8.489E-01	9.66E-04<br>-3.11E-02<br>2.34E-02<br>2.64E-02<br>-4.37E-05<br>8.493E-01	1.17E-02<br>-1.24E+00<br>6.75E-01<br>7.03E-02<br>1.28E-03<br>4.931E-01	-3.24E-15<br>-4.12E-14<br>4.52E-14<br>6.09E-02<br>2.00E-19<br>8.214E-01	-8.00E-02<br>-3.52E-01<br>-4.73E-03<br>4.74E-03<br>-5.24E-02<br>9.253E-01	-1.75E-01<br>-7.96E-01<br>-1.34E-02<br>4.58E-03<br>-1.23E-01<br>8.930E-01
Extracellular acetaldehyde<br>Local<br>Min<br>Max<br>Normalized peak height<br>Peak sensitivity value<br>Shapiro-Wilk score	2.55E-02<br>-2.94E-01<br>2.98E+00<br>1.80E-01<br>1.96E-03<br>5.629E-01	1.10E-02<br>-1.33E-01<br>1.31E+00<br>1.13E-01<br>2.21E-03<br>4.731E-01	1.99E+00<br>-2.53E-01<br>3.45E+00<br>8.22E-03<br>7.90E-03<br>8.858E-01	8.78E-03<br>8.79E-05<br>8.29E-02<br>1.05E-02<br>6.92E-03<br>8.130E-01	1.06E-01<br>2.56E-02<br>2.83E+00<br>2.79E-02<br>6.34E-02<br>5.120E-01	-5.73E-15<br>-6.01E-14<br>7.84E-14<br>5.17E-02<br>-5.39E-17<br>7.874E-01	-8.32E-02<br>-3.80E-01<br>-7.03E-04<br>4.39E-03<br>-5.17E-02<br>9.471E-01	-1.82E-01<br>-4.58E-01<br>-1.06E-02<br>2.84E-03<br>-1.50E-01<br>9.880E-01
Phosphoenolpyruvate<br>Local<br>Min<br>Max<br>Normalized peak height<br>Peak sensitivity value<br>Shapiro-Wilk score	1.38E-02<br>-3.48E-02<br>1.26E+00<br>1.25E-01<br>2.12E-03<br>5.589E-01	5.93E-03<br>-3.98E-02<br>6.21E-01<br>8.35E-02<br>1.44E-03<br>4.922E-01	1.08E+00<br>-2.59E-01<br>1.43E+00<br>8.82E-03<br>1.02E+00<br>7.493E-01	6.08E-03<br>4.35E-05<br>4.51E-02<br>9.26E-03<br>5.02E-03<br>8.860E-01	7.36E-02<br>2.79E-02<br>1.33E+00<br>2.19E-02<br>5.47E-02<br>5.404E-01	2.62E-15<br>-1.82E-13<br>1.32E-13<br>1.13E-01<br>4.89E-17<br>6.883E-01	1.11E-03<br>-2.98E-03<br>2.45E-02<br>2.12E-02<br>5.18E-04<br>5.752E-01	2.42E-03<br>-7.70E-03<br>4.52E-02<br>1.82E-02<br>1.20E-03<br>6.108E-01

	(Phosphoenolpyruvatesynthesis)	(Pyruvatekinase)	(Pyruvatedecarboxylase)	(Alcoholdehydrogenase)	(Ethanolout)	(Ethanolflow)	(Glycerolsynthesis)	(Glycerolout)
Acetaldehyde<br>Local<br>Min<br>Max<br>Normalized peak height<br>Peak sensitivity value<br>Shapiro-Wilk score	-1.56E-02<br>-5.36E-02<br>-4.24E-04<br>4.55E-03<br>-1.12E-02<br>9.509E-01	-4.70E-02<br>-2.45E-01<br>-5.08E-04<br>5.81E-03<br>-2.33E-02<br>9.008E-01	-7.51E-15<br>-5.15E-13<br>4.48E-13<br>2.48E-01<br>-4.03E-16<br>5.308E-01	-1.66E+00<br>-3.24E+00<br>-7.73E-01<br>3.93E-03<br>-1.29E+00<br>9.617E-01	0.00E+00<br>-2.53E-13<br>3.98E-13<br>5.78E-01<br>-2.81E-16<br>2.682E-01	0.00E+00<br>-1.83E-12<br>2.03E-12<br>7.20E-01<br>1.72E-15<br>2.312E-01	2.83E-01<br>1.68E-02<br>6.71E-01<br>2.66E-03<br>2.26E-01<br>9.934E-01	-5.12E-15<br>-1.70E-13<br>2.28E-13<br>4.12E-02<br>-1.84E-16<br>9.125E-01
Cytosolic glucose<br>Local<br>Min<br>Max<br>Normalized peak height<br>Peak sensitivity value<br>Shapiro-Wilk score	-5.28E-04<br>-5.65E-01<br>1.48E-01<br>5.76E-01<br>-2.82E-04<br>1.239E-01	-1.59E-03<br>-2.81E+00<br>1.48E-01<br>6.90E-01<br>-1.27E-03<br>6.766E-02	1.00E-15<br>-7.72E-13<br>5.25E-13<br>5.79E-01<br>4.83E-16<br>1.634E-01	-5.63E-02<br>-5.39E+01<br>9.67E+00<br>5.06E-01<br>-2.53E-02<br>1.821E-01	0.00E+00<br>-2.86E-13<br>1.01E-13<br>6.51E-01<br>-1.83E-16<br>1.163E-01	0.00E+00<br>-6.43E-13<br>3.44E-13<br>8.02E-01<br>3.59E-16<br>1.216E-01	9.58E-03<br>-2.67E+00<br>8.50E+00<br>5.02E-01<br>4.17E-03<br>1.519E-01	7.63E-16<br>-9.53E-13<br>2.65E-12<br>5.11E-01<br>-1.46E-15<br>1.595E-01
Glycerol<br>Local<br>Min<br>Max<br>Normalized peak height<br>Peak sensitivity value<br>Shapiro-Wilk score	-1.53E-02<br>-5.07E-02<br>-3.65E-04<br>4.44E-03<br>-1.13E-02<br>9.514E-01	-4.63E-02<br>-2.32E-01<br>-3.72E-04<br>5.26E-03<br>-2.30E-02<br>9.081E-01	1.53E-14<br>-7.08E-13<br>5.92E-13<br>2.53E-01<br>-1.08E-16<br>6.082E-01	-1.64E+00<br>-3.08E+00<br>-7.59E-01<br>4.04E-03<br>-1.33E+00<br>9.626E-01	0.00E+00<br>-3.98E-13<br>5.12E-13<br>6.47E-01<br>-1.39E-16<br>3.198E-01	0.00E+00<br>-2.23E-12<br>2.19E-12<br>8.58E-01<br>-9.75E-17<br>2.752E-01	2.79E-01<br>1.30E-02<br>6.34E-01<br>2.64E-03<br>2.96E-01<br>9.930E-01	-5.98E-01<br>-8.23E-01<br>-2.79E-01<br>2.67E-03<br>-6.16E-01<br>9.921E-01
EtOH<br>Local<br>Min<br>Max<br>Normalized peak height<br>Peak sensitivity value<br>Shapiro-Wilk score	1.72E-03<br>1.25E-04<br>1.42E-02<br>8.51E-03<br>9.46E-04<br>8.487E-01	5.20E-03<br>1.14E-04<br>4.48E-02<br>8.97E-03<br>2.06E-03<br>8.189E-01	-7.43E-17<br>-2.47E-14<br>3.41E-14<br>2.26E-01<br>8.34E-18<br>6.487E-01	1.84E-01<br>2.54E-02<br>8.67E-01<br>5.16E-03<br>7.29E-02<br>8.463E-01	-1.45E-01<br>-3.49E-01<br>-4.32E-02<br>2.99E-03<br>-1.12E-01<br>9.697E-01	-8.55E-01<br>-9.57E-01<br>-6.51E-01<br>2.99E-03<br>-8.88E-01<br>9.697E-01	-3.13E-02<br>-2.04E-01<br>-2.49E-03<br>6.36E-03<br>-1.91E-02<br>9.007E-01	-1.63E-16<br>-5.06E-14<br>4.02E-14<br>7.33E-02<br>1.65E-17<br>7.760E-01
ADP<br>Local<br>Min<br>Max<br>Normalized peak height<br>Peak sensitivity value<br>Shapiro-Wilk score	-7.33E-04<br>-6.94E-03<br>8.53E-03<br>2.05E-02<br>-3.60E-04<br>8.305E-01	-2.21E-03<br>-2.82E-02<br>8.65E-03<br>1.66E-02<br>-6.14E-04<br>8.241E-01	1.57E-15<br>-1.29E-13<br>5.37E-14<br>2.59E-01<br>-5.06E-18<br>5.968E-01	-7.81E-02<br>-9.83E-01<br>5.35E-01<br>2.06E-02<br>-3.47E-02<br>8.513E-01	0.00E+00<br>-7.46E-14<br>3.24E-14<br>5.34E-01<br>3.75E-17<br>2.873E-01	0.00E+00<br>-1.61E-13<br>1.79E-13<br>7.64E-01<br>-1.06E-17<br>2.442E-01	1.33E-02<br>-8.31E-02<br>1.02E-01<br>1.27E-02<br>6.26E-03<br>8.838E-01	1.09E-15<br>-7.60E-14<br>6.00E-14<br>6.78E-02<br>-3.58E-17<br>7.740E-01
Glyceraldehyde 3-phosphate<br>Local<br>Min<br>Max<br>Normalized peak height<br>Peak sensitivity value<br>Shapiro-Wilk score	-4.76E-02<br>-1.74E-01<br>-5.69E-03<br>4.77E-03<br>-3.79E-02<br>9.602E-01	-1.44E-01<br>-7.35E-01<br>-2.64E-03<br>5.15E-03<br>-6.89E-02<br>9.161E-01	1.37E-14<br>-2.92E-13<br>3.35E-13<br>2.41E-01<br>-2.63E-16<br>6.397E-01	-1.23E+00<br>-5.07E+00<br>-1.22E-01<br>5.19E-03<br>-6.94E-01<br>9.484E-01	0.00E+00<br>-4.11E-13<br>3.77E-13<br>6.82E-01<br>-9.06E-17<br>3.075E-01	0.00E+00<br>-1.77E-12<br>2.31E-12<br>7.97E-01<br>1.62E-15<br>2.642E-01	-5.32E-01<br>-3.28E+00<br>-7.47E-02<br>6.54E-03<br>-4.00E-01<br>9.631E-01	7.05E-15<br>-3.46E-13<br>4.14E-13<br>6.63E-02<br>9.23E-17<br>8.189E-01
Dihydroxyacetone phosphate<br>Local<br>Min<br>Max<br>Normalized peak height<br>Peak sensitivity value<br>Shapiro-Wilk score	-4.21E-02<br>-1.54E-01<br>-5.65E-03<br>4.79E-03<br>-3.44E-02<br>9.553E-01	-1.27E-01<br>-6.99E-01<br>-3.25E-03<br>5.68E-03<br>-6.13E-02<br>9.082E-01	1.04E-14<br>-6.06E-13<br>3.84E-13<br>2.69E-01<br>-2.21E-16<br>5.839E-01	-1.03E+00<br>-3.51E+00<br>3.72E-02<br>4.08E-03<br>-6.06E-01<br>9.392E-01	0.00E+00<br>-2.38E-13<br>3.78E-13<br>6.06E-01<br>2.72E-16<br>3.068E-01	0.00E+00<br>-1.76E-12<br>1.85E-12<br>7.37E-01<br>1.53E-15<br>2.565E-01	-4.89E-01<br>-2.12E+00<br>-8.18E-02<br>4.57E-03<br>-3.43E-01<br>9.483E-01	6.32E-15<br>-7.29E-13<br>3.12E-13<br>8.45E-02<br>-6.24E-17<br>8.021E-01
Glucose-6-Phosphate<br>Local<br>Min<br>Max<br>Normalized peak height<br>Peak sensitivity value<br>Shapiro-Wilk score	9.57E-04<br>-2.92E-02<br>7.96E-03<br>3.05E-02<br>3.96E-04<br>7.046E-01	2.89E-03<br>-7.59E-02<br>3.11E-02<br>2.88E-02<br>7.40E-04<br>8.416E-01	-1.82E-15<br>-1.86E-13<br>1.57E-13<br>3.31E-01<br>-3.12E-17<br>3.756E-01	1.02E-01<br>-2.98E+00<br>5.77E-01<br>3.08E-02<br>4.10E-02<br>6.885E-01	0.00E+00<br>-1.05E-13<br>2.36E-13<br>6.44E-01<br>1.25E-16<br>1.505E-01	0.00E+00<br>-6.21E-13<br>7.01E-13<br>8.56E-01<br>5.66E-17<br>1.842E-01	-1.74E-02<br>-1.05E-01<br>4.66E-01<br>2.33E-02<br>-1.12E-02<br>7.317E-01	-1.37E-15<br>-1.73E-13<br>6.51E-13<br>2.12E-01<br>1.21E-17<br>4.919E-01
NAD<br>Local<br>Min<br>Max<br>Normalized peak height<br>Peak sensitivity value<br>Shapiro-Wilk score	-1.50E-02<br>-1.99E-01<br>-2.15E-04<br>1.13E-02<br>-4.69E-03<br>8.020E-01	-4.53E-02<br>-4.29E-01<br>-3.51E-04<br>8.78E-03<br>-1.43E-02<br>8.102E-01	-6.78E-15<br>-1.98E-13<br>2.52E-13<br>2.80E-01<br>-2.00E-16<br>6.054E-01	6.68E-01<br>5.51E-02<br>2.44E+00<br>4.11E-03<br>3.64E-01<br>9.196E-01	0.00E+00<br>-1.96E-13<br>2.15E-13<br>7.38E-01<br>3.99E-17<br>2.765E-01	0.00E+00<br>-1.26E-12<br>8.58E-13<br>8.61E-01<br>-5.49E-16<br>2.404E-01	2.72E-01<br>2.40E-02<br>1.08E+00<br>3.95E-03<br>1.84E-01<br>9.389E-01	-3.23E-15<br>-2.97E-13<br>4.85E-13<br>9.14E-02<br>3.19E-16<br>7.638E-01
Extracellular acetaldehyde<br>Local<br>Min<br>Max<br>Normalized peak height<br>Peak sensitivity value<br>Shapiro-Wilk score	-1.56E-02<br>-5.75E-02<br>-4.56E-04<br>4.67E-03<br>-1.18E-02<br>9.486E-01	-4.71E-02<br>-2.69E-01<br>-3.77E-04<br>6.15E-03<br>-2.44E-02<br>9.019E-01	-8.63E-15<br>-5.36E-13<br>2.83E-13<br>2.27E-01<br>-9.72E-17<br>5.465E-01	-1.67E+00<br>-3.14E+00<br>-7.82E-01<br>3.81E-03<br>-1.31E+00<br>9.607E-01	0.00E+00<br>-7.33E-13<br>4.03E-13<br>7.10E-01<br>-5.41E-17<br>2.573E-01	0.00E+00<br>-2.86E-12<br>2.78E-12<br>8.56E-01<br>-1.65E-15<br>1.958E-01	2.84E-01<br>7.82E-03<br>6.62E-01<br>2.78E-03<br>2.90E-01<br>9.930E-01	-5.10E-15<br>-3.39E-13<br>2.57E-13<br>4.99E-02<br>2.64E-16<br>8.960E-01
Phosphoenolpyruvate<br>Local<br>Min<br>Max<br>Normalized peak height<br>Peak sensitivity value<br>Shapiro-Wilk score	2.08E-04<br>-1.05E-03<br>6.71E-03<br>3.13E-02<br>1.04E-04<br>5.759E-01	-1.20E+00<br>-1.61E+00<br>-1.07E+00<br>4.67E-03<br>-1.13E+00<br>9.177E-01	7.14E-14<br>-5.05E-13<br>4.50E-13<br>3.49E-01<br>-1.52E-16<br>7.583E-01	2.22E-02<br>-9.61E-02<br>4.10E-01<br>2.12E-02<br>8.96E-03<br>6.113E-01	0.00E+00<br>-5.16E-13<br>2.76E-13<br>6.63E-01<br>-3.70E-16<br>2.209E-01	0.00E+00<br>-4.04E-13<br>2.23E-13<br>7.96E-01<br>8.30E-17<br>2.539E-01	-3.77E-03<br>-7.46E-02<br>1.62E-02<br>1.92E-02<br>-2.06E-03<br>6.235E-01	4.71E-15<br>-9.09E-14<br>1.34E-13<br>8.69E-02<br>4.63E-17<br>7.989E-01

	(Glycerolflow)	(Acetaldehydeout)	(Acetaldehydeflow)	(Cyanide-Acetaldehydeflow)	(Cyanideflow)	(Storage)	(ATPconsumption)	(Adenylatekinase)
Acetaldehyde<br>Local<br>Min<br>Max<br>Normalized peak height<br>Peak sensitivity value<br>Shapiro-Wilk score	7.96E-15<br>-2.75E-13<br>2.72E-13<br>7.67E-02<br>1.55E-16<br>8.611E-01	-6.23E-02<br>-2.42E-01<br>-1.12E-02<br>4.53E-03<br>-4.59E-02<br>9.425E-01	-3.24E-01<br>-7.15E-01<br>-7.71E-02<br>3.17E-03<br>-2.96E-01<br>9.794E-01	-9.20E-02<br>-2.50E-01<br>-1.31E-02<br>3.39E-03<br>-7.87E-02<br>9.739E-01	-7.34E-03<br>-1.11E-01<br>-1.55E-04<br>1.31E-02<br>-2.75E-03<br>7.323E-01	-7.38E-02<br>-3.07E-01<br>6.78E-01<br>1.63E-02<br>-6.40E-02<br>6.908E-01	1.26E-01<br>-1.15E-02<br>3.32E-01<br>5.30E-03<br>3.15E-04<br>9.866E-01	1.05E-17<br>-3.65E-16<br>4.50E-16<br>2.66E-01<br>-3.34E-19<br>8.711E-01
Cytosolic glucose<br>Local<br>Min<br>Max<br>Normalized peak height<br>Peak sensitivity value<br>Shapiro-Wilk score	4.37E-17<br>-1.57E-12<br>8.70E-13<br>5.77E-01<br>6.67E-16<br>1.899E-01	2.30E-03<br>-3.06E-01<br>5.00E+00<br>4.81E-01<br>3.85E-03<br>6.959E-02	1.20E-02<br>-1.65E+00<br>1.90E+01<br>5.60E-01<br>4.46E-03<br>8.340E-02	3.40E-03<br>-3.13E-01<br>4.82E+00<br>6.01E-01<br>2.39E-03<br>7.868E-02	2.71E-04<br>-5.61E-02<br>3.00E-01<br>3.47E-01<br>-5.86E-05<br>1.948E-01	3.43E-01<br>-1.70E+01<br>3.92E+02<br>5.39E-01<br>3.45E-01<br>4.823E-02	2.74E-02<br>-1.51E+00<br>4.07E+01<br>5.64E-01<br>3.00E-02<br>4.973E-02	7.34E-18<br>-7.81E-14<br>6.66E-15<br>9.53E-01<br>5.47E-18<br>5.121E-03
Glycerol<br>Local<br>Min<br>Max<br>Normalized peak height<br>Peak sensitivity value<br>Shapiro-Wilk score	-4.02E-01<br>-7.21E-01<br>-1.77E-01<br>2.67E-03<br>-3.84E-01<br>9.921E-01	6.70E-02<br>4.37E-03<br>2.31E-01<br>3.80E-03<br>5.02E-02<br>9.624E-01	3.48E-01<br>4.65E-02<br>6.40E-01<br>3.13E-03<br>3.46E-01<br>9.973E-01	9.89E-02<br>6.33E-03<br>3.06E-01<br>3.64E-03<br>7.76E-02<br>9.836E-01	7.89E-03<br>5.67E-04<br>3.83E-02<br>5.09E-03<br>4.51E-03<br>8.986E-01	-7.27E-02<br>-2.76E-01<br>7.78E-01<br>1.79E-02<br>-6.70E-02<br>6.802E-01	1.24E-01<br>-6.37E-03<br>3.38E-01<br>5.11E-03<br>3.39E-04<br>9.867E-01	1.03E-17<br>-5.18E-16<br>2.47E-15<br>2.83E-01<br>9.22E-21<br>5.294E-01
EtOH<br>Local<br>Min<br>Max<br>Normalized peak height<br>Peak sensitivity value<br>Shapiro-Wilk score	1.29E-15<br>-2.78E-14<br>3.82E-14<br>8.86E-02<br>-7.73E-18<br>7.348E-01	-7.52E-03<br>-3.95E-02<br>-8.44E-04<br>5.25E-03<br>-5.23E-03<br>9.097E-01	-3.91E-02<br>-1.34E-01<br>-8.76E-03<br>3.99E-03<br>-2.76E-02<br>9.459E-01	-1.11E-02<br>-4.03E-02<br>-2.37E-03<br>4.39E-03<br>-8.01E-03<br>9.513E-01	-8.85E-04<br>-1.13E-02<br>-2.07E-05<br>1.21E-02<br>-2.19E-04<br>7.690E-01	-1.89E-02<br>-1.28E-01<br>2.65E-01<br>1.36E-02<br>-2.24E-02<br>7.258E-01	5.21E-02<br>-7.95E-05<br>1.44E-01<br>4.74E-03<br>1.37E-04<br>9.935E-01	2.13E-18<br>-4.21E-17<br>8.76E-17<br>2.75E-01<br>3.26E-21<br>9.099E-01
ADP<br>Local<br>Min<br>Max<br>Normalized peak height<br>Peak sensitivity value<br>Shapiro-Wilk score	5.21E-17<br>-9.13E-14<br>5.10E-14<br>9.67E-02<br>-3.32E-17<br>7.187E-01	3.20E-03<br>-1.99E-02<br>2.58E-02<br>1.37E-02<br>1.80E-03<br>8.794E-01	1.66E-02<br>-8.64E-02<br>1.31E-01<br>1.44E-02<br>1.01E-02<br>8.569E-01	4.72E-03<br>-2.85E-02<br>3.85E-02<br>1.62E-02<br>3.48E-03<br>8.618E-01	3.76E-04<br>-6.68E-03<br>1.28E-02<br>4.76E-02<br>8.95E-05<br>7.866E-01	1.88E-01<br>-3.43E-01<br>4.30E-01<br>8.09E-03<br>1.77E-01<br>8.177E-01	3.81E-02<br>-5.55E-02<br>1.93E-01<br>6.43E-03<br>3.94E-02<br>9.787E-01	-4.97E-17<br>-2.02E-15<br>1.40E-15<br>2.67E-01<br>-4.64E-19<br>9.010E-01
Glyceraldehyde 3-phosphate<br>Local<br>Min<br>Max<br>Normalized peak height<br>Peak sensitivity value<br>Shapiro-Wilk score	-4.91E-15<br>-3.23E-13<br>4.93E-13<br>9.81E-02<br>2.19E-16<br>7.738E-01	5.03E-02<br>3.49E-03<br>2.18E-01<br>4.37E-03<br>3.77E-02<br>9.274E-01	2.61E-01<br>1.90E-02<br>6.60E-01<br>3.15E-03<br>1.83E-01<br>9.791E-01	7.42E-02<br>3.61E-03<br>2.81E-01<br>4.44E-03<br>5.64E-02<br>9.497E-01	5.92E-03<br>8.71E-05<br>4.38E-02<br>7.41E-03<br>2.38E-03<br>8.355E-01	-1.10E-01<br>-3.66E-01<br>9.32E-01<br>1.45E-02<br>-9.53E-02<br>8.220E-01	1.05E-01<br>-7.47E-02<br>4.90E-01<br>6.43E-03<br>8.10E-02<br>9.860E-01	1.80E-17<br>-9.53E-16<br>2.00E-15<br>2.72E-01<br>1.19E-18<br>7.753E-01
Dihydroxyacetone phosphate<br>Local<br>Min<br>Max<br>Normalized peak height<br>Peak sensitivity value<br>Shapiro-Wilk score	-3.90E-15<br>-3.96E-13<br>3.47E-13<br>1.02E-01<br>-8.07E-17<br>7.664E-01	4.23E-02<br>-1.81E-03<br>1.92E-01<br>4.73E-03<br>3.20E-02<br>9.226E-01	2.20E-01<br>-7.98E-03<br>5.87E-01<br>3.23E-03<br>1.58E-01<br>9.755E-01	6.25E-02<br>-2.30E-03<br>2.42E-01<br>4.28E-03<br>3.92E-02<br>9.477E-01	4.99E-03<br>-1.70E-04<br>4.15E-02<br>8.40E-03<br>1.68E-03<br>8.187E-01	-1.06E-01<br>-3.33E-01<br>8.94E-01<br>1.41E-02<br>-9.54E-02<br>7.815E-01	1.13E-01<br>-8.36E-02<br>4.26E-01<br>6.09E-03<br>2.15E-04<br>9.865E-01	1.69E-17<br>-6.74E-16<br>1.13E-15<br>2.67E-01<br>-8.45E-19<br>8.397E-01
Glucose-6-Phosphate<br>Local<br>Min<br>Max<br>Normalized peak height<br>Peak sensitivity value<br>Shapiro-Wilk score	-6.86E-17<br>-2.81E-13<br>2.31E-13<br>2.11E-01<br>-2.18E-17<br>4.925E-01	-4.17E-03<br>-2.50E-02<br>1.09E-01<br>2.30E-02<br>-2.55E-03<br>7.259E-01	-2.17E-02<br>-8.48E-02<br>4.15E-01<br>1.81E-02<br>-1.45E-02<br>7.181E-01	-6.16E-03<br>-2.30E-02<br>1.37E-01<br>1.96E-02<br>-4.01E-03<br>7.272E-01	-4.92E-04<br>-5.27E-03<br>2.86E-02<br>5.80E-02<br>-1.07E-04<br>6.386E-01	-7.52E-01<br>-1.53E+00<br>6.95E-01<br>1.51E-02<br>-7.54E-01<br>7.918E-01	-4.97E-02<br>-2.62E-01<br>4.68E-01<br>8.53E-03<br>-5.11E-02<br>9.418E-01	-2.72E-17<br>-2.31E-15<br>8.00E-16<br>3.02E-01<br>9.09E-20<br>8.083E-01
NAD<br>Local<br>Min<br>Max<br>Normalized peak height<br>Peak sensitivity value<br>Shapiro-Wilk score	2.80E-15<br>-2.69E-13<br>3.28E-13<br>1.17E-01<br>2.52E-16<br>7.467E-01	-2.73E-02<br>-1.61E-01<br>-1.53E-03<br>5.63E-03<br>-1.55E-02<br>8.894E-01	-1.42E-01<br>-5.28E-01<br>-1.27E-02<br>4.29E-03<br>-9.69E-02<br>9.416E-01	-4.03E-02<br>-1.99E-01<br>-3.09E-03<br>5.12E-03<br>-2.63E-02<br>9.082E-01	-3.22E-03<br>-2.85E-02<br>-7.91E-05<br>9.09E-03<br>-1.00E-03<br>8.056E-01	-9.68E-03<br>-3.96E-01<br>8.64E-02<br>2.13E-02<br>2.36E-04<br>7.724E-01	-2.81E-02<br>-1.64E-01<br>1.02E-03<br>6.35E-03<br>-1.27E-02<br>8.625E-01	2.62E-18<br>-1.16E-16<br>9.21E-16<br>3.78E-01<br>-4.71E-19<br>4.559E-01
Extracellular acetaldehyde<br>Local<br>Min<br>Max<br>Normalized peak height<br>Peak sensitivity value<br>Shapiro-Wilk score	7.94E-15<br>-2.08E-13<br>2.32E-13<br>7.21E-02<br>-7.74E-18<br>8.538E-01	6.82E-02<br>4.36E-03<br>2.28E-01<br>3.62E-03<br>6.29E-02<br>9.624E-01	-4.25E-01<br>-8.32E-01<br>-1.25E-01<br>3.24E-03<br>-4.09E-01<br>9.857E-01	-1.21E-01<br>-2.78E-01<br>-3.44E-02<br>3.35E-03<br>-1.19E-01<br>9.819E-01	-9.62E-03<br>-1.22E-01<br>-3.95E-04<br>1.12E-02<br>-3.62E-03<br>7.524E-01	-7.39E-02<br>-3.00E-01<br>6.44E-01<br>1.59E-02<br>-7.12E-02<br>6.883E-01	1.27E-01<br>-2.75E-02<br>3.61E-01<br>5.53E-03<br>3.29E-04<br>9.869E-01	1.05E-17<br>-3.23E-16<br>5.55E-16<br>2.72E-01<br>-4.19E-19<br>8.650E-01
Phosphoenolpyruvate<br>Local<br>Min<br>Max<br>Normalized peak height<br>Peak sensitivity value<br>Shapiro-Wilk score	-1.00E-15<br>-8.30E-14<br>9.69E-14<br>1.13E-01<br>6.56E-17<br>7.084E-01	-9.07E-04<br>-1.75E-02<br>2.38E-03<br>1.74E-02<br>-5.87E-04<br>6.311E-01	-4.72E-03<br>-5.66E-02<br>1.30E-02<br>1.37E-02<br>-3.15E-03<br>6.479E-01	-1.34E-03<br>-1.77E-02<br>4.74E-03<br>1.54E-02<br>-9.43E-04<br>6.539E-01	-1.07E-04<br>-4.93E-03<br>7.18E-04<br>4.39E-02<br>-3.04E-05<br>5.397E-01	-5.20E-02<br>-1.51E-01<br>3.23E-01<br>1.40E-02<br>-5.39E-02<br>7.264E-01	6.64E-02<br>-3.03E-03<br>1.66E-01<br>2.99E-03<br>6.31E-02<br>9.876E-01	9.58E-18<br>-3.23E-16<br>4.90E-16<br>2.73E-01<br>-8.41E-20<br>8.917E-01

Fructose-6-Phosphate	Local	1.24E-02	5.33E-03	9.67E-01	8.44E-02	-3.38E-01	-8.95E-16	5.90E-03	1.29E-02	1.11E-03	3.34E-03	-2.13E-15	1.18E-01
	Min	-7.44E-01	-4.74E-01	-2.30E+00	1.87E-02	-1.07E+01	-5.15E-14	-1.57E-01	-2.61E-01	-3.14E-02	-1.03E-01	-2.37E-13	-2.81E+00
	Max	1.14E+01	2.09E+00	1.21E+01	2.60E-01	4.00E-02	5.75E-14	3.11E-02	6.47E-02	8.94E-03	3.94E-02	2.38E-13	6.69E-01
	Normalized peak height	4.37E-01	2.74E-01	4.79E-02	4.81E-03	3.63E-02	1.44E-01	2.62E-02	1.98E-02	2.92E-02	3.38E-02	3.42E-01	2.75E-02
	Peak sensitivity value	2.49E-03	2.15E-03	9.37E-01	6.30E-02	-2.22E-01	-6.17E-19	2.65E-03	7.90E-03	5.34E-04	7.49E-04	-2.25E-16	5.09E-02
	Shapiro-Wilk score	4.664E-01	4.388E-01	6.615E-01	9.521E-01	5.608E-01	5.722E-01	7.176E-01	7.209E-01	7.365E-01	8.497E-01	3.781E-01	7.263E-01
Extracellular cyanide	Local	-1.89E-03	-8.11E-04	-1.47E-01	-6.48E-04	-7.85E-03	4.27E-16	6.15E-03	1.34E-02	1.15E-03	3.48E-03	6.32E-16	1.23E-01
	Min	-8.84E-01	-2.72E-01	-1.09E+00	-1.91E-02	-8.39E-01	-5.89E-15	1.95E-04	5.77E-04	4.04E-05	5.29E-05	-4.71E-14	8.71E-03
	Max	4.43E-03	2.66E-03	5.00E-02	-3.46E-06	-2.96E-04	7.72E-15	6.22E-02	8.70E-02	8.76E-03	4.32E-02	2.28E-14	9.14E-01
	Normalized peak height	4.55E-01	2.50E-01	3.32E-02	2.73E-02	9.11E-02	8.84E-02	1.11E-02	7.27E-03	8.54E-03	1.53E-02	2.52E-01	8.56E-03
	Peak sensitivity value	-4.55E-04	-2.25E-04	-9.38E-04	-2.23E-04	-2.39E-03	-4.06E-18	2.52E-03	7.02E-03	3.76E-04	6.79E-04	5.19E-18	5.71E-02
	Shapiro-Wilk score	4.605E-01	3.821E-01	7.990E-01	6.310E-01	4.420E-01	7.045E-01	8.174E-01	8.703E-01	8.272E-01	7.496E-01	4.648E-01	8.184E-01
Pyruvate	Local	9.01E-01	3.88E-01	7.04E+01	2.28E-01	2.76E+00	-6.96E-14	4.07E-02	8.91E-02	7.64E-03	2.31E-02	-7.79E+01	8.15E-01
	Min	-8.38E-01	-1.65E-01	-1.61E+01	1.58E-04	2.20E-02	-6.70E-11	-3.46E-01	-8.12E-01	-6.48E-02	-2.24E-01	-5.02E+04	-4.59E+00
	Max	2.08E+04	8.47E+03	4.28E+04	4.04E+02	2.72E+04	3.38E-11	2.94E+02	5.40E+02	9.28E+01	1.61E+02	-1.69E+00	7.78E+03
	Normalized peak height	9.86E-01	9.94E-01	9.55E-01	9.82E-01	9.90E-01	9.79E-01	9.91E-01	9.81E-01	9.85E-01	9.92E-01	9.64E-01	9.81E-01
	Peak sensitivity value	9.58E+00	4.07E+00	5.31E+00	2.02E-01	1.36E+01	2.07E-14	9.59E-02	-1.06E-03	-1.83E-02	1.82E-02	-2.68E+01	-7.01E-01
	Shapiro-Wilk score	5.438E-03	2.497E-03	1.061E-02	7.280E-03	4.172E-03	6.094E-03	3.362E-03	4.022E-03	1.837E-03	2.136E-03	1.043E-02	3.160E-03
Extracellular ethanol	Local	1.01E-02	4.37E-03	7.92E-01	2.32E-03	2.81E-02	-3.81E-16	9.18E-03	2.01E-02	1.72E-03	5.20E-03	-1.74E-16	1.84E-01
	Min	-3.14E-03	-2.22E-03	-2.57E+00	-1.03E-03	-6.50E-03	-9.90E-15	4.26E-04	1.48E-03	1.09E-04	1.26E-04	-2.93E-14	2.36E-02
	Max	9.04E-01	1.11E+00	9.40E-01	4.74E-02	9.24E-01	1.07E-14	5.87E-02	1.20E-01	1.50E-02	4.40E-02	3.31E-14	8.80E-01
	Normalized peak height	1.21E-01	1.65E-01	1.87E-02	1.90E-02	2.87E-02	8.86E-02	6.79E-03	5.76E-03	9.04E-03	9.18E-03	2.29E-01	5.34E-03
	Peak sensitivity value	9.46E-04	1.68E-03	5.69E-03	2.00E-03	2.19E-02	9.47E-18	5.00E-03	1.10E-02	9.53E-04	1.77E-03	-2.32E-17	8.91E-02
	Shapiro-Wilk score	5.522E-01	4.718E-01	7.949E-01	7.921E-01	4.924E-01	7.543E-01	8.792E-01	9.031E-01	8.438E-01	8.182E-01	6.492E-01	8.471E-01
Extracellular glycerol	Local	2.51E-02	1.08E-02	1.96E+00	8.63E-03	1.04E-01	8.14E-15	-8.18E-02	-1.79E-01	-1.53E-02	-4.63E-02	1.49E-14	-1.64E+00
	Min	-5.83E-02	-3.00E-02	-2.45E-01	1.05E-04	2.38E-02	-9.68E-14	-3.54E-01	-4.47E-01	-5.91E-02	-2.40E-01	-5.13E-13	-3.08E+00
	Max	3.04E+00	1.37E+00	3.35E+00	8.40E-02	2.95E+00	8.91E-14	-1.69E-03	-1.33E-02	-6.63E-04	-4.65E-04	3.82E-13	-7.22E-01
	Normalized peak height	1.84E-01	1.05E-01	8.51E-03	1.04E-02	3.02E-02	5.02E-02	4.01E-03	2.78E-03	4.81E-03	5.59E-03	2.32E-01	4.08E-03
	Peak sensitivity value	2.07E-03	2.85E-03	8.86E-03	6.10E-03	6.62E-02	-7.77E-17	-6.71E-02	-1.60E-01	-1.01E-02	-2.64E-02	6.46E-17	-1.35E+00
	Shapiro-Wilk score	5.631E-01	4.789E-01	8.787E-01	8.151E-01	5.160E-01	8.297E-01	9.446E-01	9.877E-01	9.483E-01	8.995E-01	6.227E-01	9.648E-01
Extracellular glucose	Local	2.57E+00	-1.40E-02	-2.53E+00	-3.55E-03	-4.30E-02	8.97E-17	-5.98E-04	-1.31E-03	-1.12E-04	-3.38E-04	2.12E-16	-1.20E-02
	Min	4.23E-01	-1.98E+00	-2.04E+01	-1.53E-01	-1.36E+01	-5.45E-14	-1.33E-01	-2.96E-01	-3.83E-02	-5.88E-02	-2.54E-13	-4.10E+00
	Max	2.06E+01	1.80E-02	8.72E-01	-6.27E-05	-1.36E-03	9.31E-14	1.83E-02	3.75E-02	5.33E-03	2.14E-02	2.10E-13	4.46E-01
	Normalized peak height	9.75E-03	1.53E-01	1.26E-02	4.07E-02	2.34E-01	6.15E-01	2.26E-01	2.16E-01	2.90E-01	2.04E-01	7.08E-01	2.52E-01
	Peak sensitivity value	9.78E-01	-9.31E-04	-7.97E-01	-7.51E-04	-8.14E-03	1.55E-17	-1.54E-04	-3.53E-04	-1.65E-05	-3.07E-05	9.43E-17	-1.18E-03
	Shapiro-Wilk score	7.982E-01	5.928E-01	7.503E-01	6.856E-01	4.381E-01	2.611E-01	3.891E-01	4.018E-01	3.701E-01	4.447E-01	1.760E-01	3.987E-01
1-3-Bisphosphoglycerate	Local	1.07E-02	4.61E-03	8.37E-01	4.44E-02	5.38E-01	5.30E-16	8.28E-03	1.81E-02	-2.84E-01	-8.56E-01	3.50E-14	1.66E-01
	Min	-8.06E+00	-9.67E-01	-7.60E+00	-6.64E-05	-1.03E-01	-2.91E-13	-8.49E-04	-6.50E-03	-8.71E-01	-1.42E+00	-8.74E-13	-5.67E-02
	Max	1.18E+00	6.39E-01	1.63E+00	1.66E-01	8.32E+00	1.66E-13	1.38E-01	2.42E-01	-8.04E-02	-1.44E-01	3.16E-13	2.94E+00
	Normalized peak height	3.35E-01	1.85E-01	2.73E-02	5.79E-03	2.40E-02	1.05E-01	1.56E-02	1.25E-02	3.45E-03	3.69E-03	1.88E-01	1.79E-02
	Peak sensitivity value	-8.87E-04	2.20E-03	1.01E-02	3.39E-02	3.65E-01	1.55E-16	4.65E-03	1.05E-02	-2.79E-01	-8.01E-01	1.60E-16	7.97E-02
	Shapiro-Wilk score	5.486E-01	5.165E-01	7.777E-01	9.800E-01	6.441E-01	5.260E-01	7.186E-01	7.657E-01	9.680E-01	9.986E-01	8.455E-01	7.415E-01
Fructose 1-6-bisphosphate	Local	4.60E-02	1.98E-02	3.59E+00	2.43E-02	2.94E-01	-9.76E-02	-8.09E-02	-1.00E+00	-8.59E-02	-2.59E-01	2.20E-14	-2.18E+00
	Min	-7.34E-01	-2.24E-01	-1.33E+00	9.39E-05	6.32E-02	-4.97E-01	-8.35E-01	-2.03E+00	-3.04E-01	-1.42E+00	-6.57E-13	-7.59E+00
	Max	7.62E+00	3.48E+00	1.09E+01	2.30E-01	6.75E+00	-1.96E-02	3.65E-01	-1.08E-01	-8.67E-03	-6.48E-03	6.13E-13	-9.95E-02
	Normalized peak height	2.50E-01	1.53E-01	1.46E-02	1.01E-02	2.27E-02	5.21E-03	4.11E-03	2.67E-03	4.66E-03	5.85E-03	2.27E-01	4.46E-03
	Peak sensitivity value	5.00E-03	3.56E-03	1.61E-02	1.86E-02	1.80E-01	-7.52E-02	-7.61E-02	-8.95E-01	-6.79E-02	-1.11E-01	2.46E-16	-1.32E+00
	Shapiro-Wilk score	5.582E-01	4.690E-01	9.267E-01	8.443E-01	5.507E-01	9.191E-01	9.942E-01	9.953E-01	9.580E-01	9.091E-01	6.050E-01	9.374E-01
AMP	Local	4.17E-03	1.79E-03	3.25E-01	-6.74E-02	-8.16E-01	2.40E-15	-1.26E-02	-2.76E-02	-2.37E-03	-7.14E-03	6.43E-15	-2.52E-01
	Min	-5.39E-01	-1.64E+00	-9.70E-01	-1.88E-01	-1.03E+01	-5.62E-14	-2.84E-01	-3.33E-01	-3.26E-02	-1.03E-01	-1.91E-13	-3.40E+00
	Max	1.01E+01	1.51E+00	1.06E+01	-4.50E-05	-3.84E-01	2.07E-13	3.49E-03	1.21E-02	1.47E-03	5.87E-04	2.46E-13	1.30E-01
	Normalized peak height	4.28E-01	5.01E-01	5.01E-02	4.63E-03	1.96E-02	1.09E-01	2.05E-02	1.12E-02	1.30E-02	1.38E-02	2.42E-01	1.38E-02
	Peak sensitivity value	-4.12E-03	4.38E-04	8.54E-03	-4.98E-02	-5.28E-01	3.65E-17	-6.42E-03	-1.92E-02	-1.10E-03	-2.48E-03	1.13E-16	-1.12E-01
	Shapiro-Wilk score	3.632E-01	3.929E-01	5.944E-01	9.814E-01	6.386E-01	6.720E-01	7.323E-01	7.877E-01	7.630E-01	7.617E-01	6.308E-01	7.561E-01
ATP	Local	-1.59E-03	-6.83E-04	-1.24E-01	2.57E-02	3.11E-01	-8.56E-16	4.80E-03	1.05E-02	9.01E-04	2.72E-03	-2.17E-15	9.61E-02
	Min	-1.23E+01	-2.08E+00	-9.21E+00	-1.42E-05	-1.37E-02	-7.97E-14	2.66E-04	5.97E-04	6.19E-05	6.74E-05	-1.88E-13	9.14E-03
	Max	2.14E+00	6.83E-01	3.82E+00	1.39E-01	1.26E+01	1.10E-13	1.80E-01	4.09E-01	3.82E-02	7.39E-02	2.25E-13	3.89E+00
	Normalized peak height	6.56E-01	5.67E-01	1.14E-01	6.46E-03	4.54E-02	2.18E-01	2.94E-02	2.94E-02	3.49E-02	2.35E-02	2.79E-01	3.53E-02
	Peak sensitivity value	-3.20E-03	9.60E-05	-6.04E-03	1.94E-02	1.82E-01	9.26E-17	2.52E-03	6.11E-03	3.48E-04	9.91E-04	-7.48E-18	3.44E-02
	Shapiro-Wilk score	2.878E-01	3.382E-01	4.530E-01	9.220E-01	5.548E-01	4.807E-01	5.601E-01	5.996E-01	5.683E-01	6.638E-01	4.162E-01	6.020E-01
NADH	Local	9.35E-03	4.03E-03	7.30E-01	-1.86E-03	-2.25E-02	6.21E-15	1.54E-01	3.36E-01	2.88E-02	8.69E-02	1.30E-14	-1.28E+00
	Min	-3.30E-02	-2.67E-02	-9.54E-02	-2.29E-02	-6.91E-01	-7.15E-14	7.86E-03	5.45E-02	3.14E-03	2.15E-03	-4.00E-13	-2.18E+00
	Max	1.50E+00	6.33E-01	1.61E+00	2.95E-02	1.33E+00	8.41E-14	5.36E-01	6.29E-01	1.20E-01	3.59E-01	3.59E-13	-1.45E-01
	Normalized peak height	1.86E-01	1.57E-01	9.72E-03	9.52E-03	2.88E-02	4.61E-02	4.57E-03	3.44E-03	4.90E-03	4.15E-03	2.69E-01	4.86E-03
	Peak sensitivity value	1.59E-03	6.18E-04	4.64E-03	-1.05E-03	-9.48E-03	-3.09E-17	1.16E-01	3.13E-01	2.12E-02	4.30E-02	2.98E-16	-1.12E+00
	Shapiro-Wilk score	5.607E-01	4.750E-01	9.750E-01	9.525E-01	5.376E-01	8.053E-01	9.590E-01	9.993E-01	9.498E-01	9.343E-01	6.178E-01	9.923E-01

Fructose-6-Phosphate	Local	0.00E+00	0.00E+00	-2.01E-02	-1.59E-15	-7.98E-17	-4.83E-03	-2.51E-02	-7.13E-03	-5.69E-04	-7.58E-01	-5.75E-02	-2.80E-17
	Min	-1.28E-13	-6.90E-13	-1.37E-01	-3.44E-13	-2.99E-13	-2.79E-02	-8.91E-02	-2.71E-02	-7.51E-03	-1.22E+00	-2.61E-01	-1.86E-15
	Max	1.10E-13	3.99E-13	4.44E-01	3.44E-13	1.78E-13	1.03E-01	4.24E-01	1.09E-01	2.80E-02	5.46E-01	4.70E-01	1.03E-15
	Normalized peak height	5.88E-01	8.24E-01	2.10E-02	1.73E-01	1.79E-01	2.03E-02	1.66E-02	1.60E-02	5.37E-02	1.17E-02	8.09E-03	3.02E-01
	Peak sensitivity value	-2.71E-17	2.20E-16	-1.15E-02	1.10E-16	2.35E-17	-2.73E-03	-1.59E-02	-5.37E-03	-1.17E-04	-7.63E-01	-5.63E-02	-1.05E-19
	Shapiro-Wilk score	2.712E-01	2.177E-01	7.522E-01	5.739E-01	5.570E-01	7.684E-01	7.456E-01	7.573E-01	6.890E-01	7.907E-01	9.478E-01	8.239E-01
Extracellular cyanide	Local	0.00E+00	0.00E+00	-2.09E-02	3.66E-16	-5.96E-16	-5.03E-03	3.14E-02	-6.50E-02	7.46E-02	5.46E-03	-9.35E-03	-7.74E-19
	Min	-2.39E-14	-1.28E-13	-1.60E-01	-6.23E-14	-2.86E-14	-2.49E-02	1.83E-03	-4.10E-01	7.33E-03	-1.22E-01	-7.46E-02	-2.97E-17
	Max	3.04E-14	1.27E-13	-9.66E-04	4.58E-14	3.70E-14	-4.26E-04	3.52E-01	-6.94E-03	5.23E-01	5.79E-02	7.56E-04	2.20E-17
	Normalized peak height	6.40E-01	8.59E-01	7.92E-03	1.12E-01	1.22E-01	5.63E-03	1.08E-02	6.89E-03	7.32E-03	3.04E-02	1.07E-02	2.69E-01
	Peak sensitivity value	1.92E-17	-2.42E-17	-1.15E-02	-1.09E-17	6.87E-18	-2.96E-03	1.18E-02	-3.70E-02	4.06E-02	1.90E-03	-3.57E-05	-1.15E-20
	Shapiro-Wilk score	2.044E-01	1.755E-01	8.576E-01	6.955E-01	6.597E-01	9.038E-01	7.537E-01	8.515E-01	8.375E-01	7.159E-01	8.250E-01	7.828E-01
Pyruvate	Local	0.00E+00	0.00E+00	-1.39E-01	-5.18E-14	1.49E-13	-3.34E-02	-1.73E-01	-4.93E-02	-3.93E-03	-1.87E+00	4.59E+00	2.26E-16
	Min	-2.67E-12	-1.45E-09	-1.03E+03	-5.19E-11	-4.82E-11	-1.84E+02	-9.74E+02	-2.08E+02	-3.02E+01	-1.67E+03	-4.13E-05	-1.59E-11
	Max	1.64E-12	2.67E-10	1.61E+00	1.38E-10	9.64E-11	2.32E-01	1.38E+00	4.06E-01	3.61E-02	3.11E+03	4.45E+03	1.18E-12
	Normalized peak height	7.15E-01	9.99E-01	9.83E-01	9.40E-01	9.51E-01	9.86E-01	9.83E-01	7.80E-01	9.86E-01	9.28E-01	9.79E-01	9.93E-01
	Peak sensitivity value	-1.99E-15	1.87E-13	5.31E-02	-3.75E-14	-1.53E-14	-4.42E-02	-8.34E-02	9.36E-02	-9.22E-03	-2.28E+00	2.22E+00	8.21E-15
	Shapiro-Wilk score	4.692E-02	6.996E-04	3.178E-03	1.589E-02	1.282E-02	4.425E-03	4.345E-03	5.508E-03	4.528E-03	6.852E-03	7.962E-03	6.595E-04
Extracellular ethanol	Local	-3.41E-16	-1.00E+00	-3.13E-02	-1.73E-16	1.29E-15	-7.52E-03	-3.91E-02	-1.11E-02	-8.85E-04	-1.89E-02	5.21E-02	2.13E-18
	Min	-2.74E-14	-1.00E+00	-1.84E-01	-5.92E-14	-2.39E-14	-4.33E-02	-1.29E-01	-3.66E-02	-1.18E-02	-1.37E-01	-4.74E-05	-1.58E-16
	Max	1.86E-14	-1.00E+00	-2.76E-03	8.15E-14	4.29E-14	-9.28E-04	-8.85E-03	-2.38E-03	-1.98E-05	4.98E-01	1.45E-01	1.44E-16
	Normalized peak height	3.86E-01	1.00E+00	5.63E-03	9.84E-02	8.90E-02	5.96E-03	3.82E-03	4.06E-03	1.22E-02	2.02E-02	3.89E-03	2.84E-01
	Peak sensitivity value	1.33E-17	-1.00E+00	-2.33E-02	-6.19E-18	2.12E-18	-5.32E-03	-2.58E-02	-9.76E-03	-2.38E-04	-2.25E-02	3.14E-04	1.31E-20
	Shapiro-Wilk score	4.486E-01	1.000E+00	8.993E-01	7.642E-01	7.395E-01	9.075E-01	9.446E-01	9.536E-01	7.686E-01	7.424E-01	9.935E-01	8.184E-01
Extracellular glycerol	Local	0.00E+00	0.00E+00	2.79E-01	9.72E-15	-1.00E+00	6.70E-02	3.48E-01	9.89E-02	7.89E-03	-7.27E-02	1.24E-01	1.03E-17
	Min	-6.63E-13	-3.13E-12	1.31E-02	-4.13E-13	-1.00E+00	4.74E-03	3.93E-02	6.49E-03	5.52E-04	-2.97E-01	-1.30E-02	-1.70E-15
	Max	5.33E-13	3.20E-12	6.16E-01	3.52E-13	-1.00E+00	2.35E-01	6.54E-01	2.91E-01	3.98E-02	7.52E-01	3.12E-01	2.03E-15
	Normalized peak height	7.00E-01	8.64E-01	2.58E-03	4.29E-02	1.00E+00	3.85E-03	3.34E-03	3.54E-03	5.28E-03	1.74E-02	5.48E-03	2.88E-01
	Peak sensitivity value	1.48E-16	5.02E-16	2.84E-01	-2.83E-16	-1.00E+00	4.88E-02	3.67E-01	7.91E-02	3.79E-03	-6.81E-02	1.50E-04	-1.48E-18
	Shapiro-Wilk score	3.101E-01	2.562E-01	9.930E-01	8.919E-01	1.000E+00	9.629E-01	9.974E-01	9.836E-01	8.960E-01	6.942E-01	9.869E-01	4.880E-01
Extracellular glucose	Local	0.00E+00	0.00E+00	2.04E-03	1.65E-16	1.51E-17	4.89E-04	2.54E-03	7.23E-04	5.76E-05	2.55E-02	5.83E-03	-3.53E-18
	Min	-5.26E-14	-9.23E-13	-6.90E-02	-3.60E-13	-8.23E-13	-1.68E-02	-5.68E-02	-1.45E-02	-3.30E-03	-1.08E+00	-1.39E-01	-4.68E-15
	Max	1.08E-13	3.31E-13	4.93E-01	4.69E-13	2.12E-13	1.46E-01	6.37E-01	1.71E-01	2.72E-02	8.32E-01	5.12E-01	1.49E-15
	Normalized peak height	6.71E-01	9.12E-01	2.44E-01	6.38E-01	6.27E-01	2.66E-01	2.24E-01	2.04E-01	3.49E-01	6.11E-02	8.88E-02	6.14E-01
	Peak sensitivity value	7.77E-17	4.35E-16	4.54E-04	-2.48E-17	-4.16E-16	1.33E-04	4.72E-04	2.29E-04	6.41E-06	6.92E-03	2.01E-03	9.23E-19
	Shapiro-Wilk score	6.645E-02	2.654E-02	4.110E-01	2.470E-01	1.902E-01	4.142E-01	4.329E-01	4.348E-01	3.297E-01	6.468E-01	6.085E-01	1.978E-01
1-3-Bisphosphoglycerate	Local	0.00E+00	0.00E+00	-2.82E-02	6.06E-16	-4.86E-16	-6.78E-03	-3.52E-02	-1.00E-02	-7.98E-04	-3.98E-01	-7.28E-03	7.56E-17
	Min	-3.53E-13	-1.33E-12	-4.44E-01	-5.30E-13	-3.11E-13	-9.83E-02	-2.99E-01	-9.57E-02	-2.22E-02	-8.38E-01	-4.80E-01	-2.82E-15
	Max	2.71E-13	5.88E-13	7.98E-03	2.43E-13	2.62E-13	1.16E-03	8.02E-03	1.68E-03	5.65E-04	1.61E-01	5.18E-02	2.80E-15
	Normalized peak height	6.89E-01	8.42E-01	1.40E-02	1.05E-01	1.20E-01	1.32E-02	9.30E-03	1.05E-02	2.42E-02	1.02E-02	1.29E-02	2.70E-01
	Peak sensitivity value	1.67E-16	-3.17E-16	-1.85E-02	-9.30E-17	6.32E-18	-4.36E-03	-2.75E-02	-7.43E-03	-2.66E-04	-3.97E-01	2.63E-03	1.78E-18
	Shapiro-Wilk score	2.800E-01	2.034E-01	7.638E-01	6.984E-01	6.432E-01	7.810E-01	7.992E-01	7.890E-01	6.623E-01	8.212E-01	7.812E-01	8.573E-01
Fructose 1-6-bisphosphate	Local	0.00E+00	0.00E+00	-9.76E-01	1.28E-14	-8.40E-15	8.90E-02	4.63E-01	1.31E-01	1.05E-02	-2.10E-01	2.16E-01	3.37E-17
	Min	-1.30E-12	-1.90E-12	-5.03E+00	-7.76E-13	-7.37E-13	3.57E-03	1.85E-02	5.56E-03	2.78E-04	-8.38E-01	-9.37E-02	-1.20E-15
	Max	7.13E-13	2.52E-12	-1.32E-01	1.10E-12	8.06E-13	4.01E-01	1.23E+00	4.81E-01	7.93E-02	1.65E+00	8.66E-01	1.21E-15
	Normalized peak height	7.31E-01	8.51E-01	5.54E-03	7.83E-02	1.05E-01	4.68E-03	3.34E-03	4.06E-03	7.84E-03	1.49E-02	5.65E-03	2.70E-01
	Peak sensitivity value	-3.14E-16	8.52E-17	-7.60E-01	-3.54E-16	-1.57E-16	6.57E-02	3.13E-01	1.06E-01	3.48E-03	-1.85E-01	1.95E-01	1.18E-18
	Shapiro-Wilk score	2.876E-01	2.837E-01	9.474E-01	7.974E-01	7.594E-01	9.222E-01	9.751E-01	9.464E-01	8.297E-01	7.993E-01	9.862E-01	8.763E-01
AMP	Local	0.00E+00	0.00E+00	4.30E-02	3.70E-15	2.03E-16	1.03E-02	5.37E-02	1.52E-02	1.22E-03	6.08E-01	1.23E-01	6.38E-17
	Min	-4.46E-13	-8.77E-13	-1.55E-02	-4.78E-13	-5.93E-13	-6.06E-03	-1.45E-02	-3.30E-03	-5.17E-04	-1.46E+00	-3.48E-03	-4.83E-15
	Max	4.16E-13	1.20E-12	5.71E-01	5.40E-13	3.13E-13	1.61E-01	6.05E-01	1.26E-01	2.67E-02	1.39E+00	8.14E-01	2.88E-15
	Normalized peak height	6.51E-01	7.58E-01	1.18E-02	1.03E-01	1.33E-01	1.43E-02	1.16E-02	9.45E-03	1.91E-02	2.97E-02	7.73E-03	2.70E-01
	Peak sensitivity value	1.80E-16	6.53E-16	2.35E-02	-1.78E-16	-3.56E-18	5.86E-03	3.35E-02	1.16E-02	3.96E-04	6.26E-01	1.24E-01	-3.48E-18
	Shapiro-Wilk score	2.105E-01	2.271E-01	7.892E-01	6.857E-01	6.377E-01	7.878E-01	8.069E-01	8.063E-01	6.844E-01	7.410E-01	9.348E-01	8.359E-01
ATP	Local	0.00E+00	0.00E+00	-1.64E-02	-1.37E-15	-6.97E-17	-3.93E-03	-2.04E-02	-5.81E-03	-4.63E-04	-2.32E-01	-4.68E-02	2.55E-17
	Min	-1.34E-13	-8.06E-13	-5.29E-01	-4.26E-13	-2.28E-13	-1.30E-01	-5.19E-01	-1.40E-01	-3.11E-02	-1.66E+00	-5.02E-01	-7.03E-16
	Max	7.75E-14	3.17E-13	-1.20E-03	3.12E-13	2.89E-13	-2.88E-04	-3.17E-03	-7.72E-04	-8.62E-06	2.07E-01	-1.15E-02	2.36E-15
	Normalized peak height	5.65E-01	6.35E-01	2.37E-02	1.58E-01	1.63E-01	2.50E-02	2.16E-02	2.11E-02	5.51E-02	1.37E-02	9.46E-03	2.92E-01
	Peak sensitivity value	-4.82E-17	-5.60E-16	-1.15E-02	-5.04E-17	-1.70E-16	-2.43E-03	-1.37E-02	-4.60E-03	-1.18E-04	-2.20E-01	-4.46E-02	-9.85E-19
	Shapiro-Wilk score	2.367E-01	1.723E-01	6.041E-01	5.108E-01	4.952E-01	6.227E-01	6.474E-01	6.511E-01	5.276E-01	7.842E-01	8.020E-01	8.022E-01
NADH	Local	0.00E+00	0.00E+00	-5.23E-01	6.20E-15	-5.37E-15	5.25E-02	2.73E-01	7.75E-02	6.18E-03	1.86E-02	5.39E-02	-5.02E-18
	Min	-4.41E-13	-2.99E-12	-8.78E-01	-2.14E-13	-1.69E-13	1.67E-03	1.33E-02	2.45E-03	3.75E-04	-9.68E-02	-7.17E-04	-5.90E-16
	Max	3.16E-13	2.05E-12	-7.04E-02	2.71E-13	2.62E-13	2.14E-01	6.08E-01	2.67E-01	3.20E-02	3.37E-01	1.61E-01	1.25E-16
	Normalized peak height	7.21E-01	8.39E-01	4.90E-03	4.00E-02	6.01E-02	4.28E-03	3.53E-03	3.53E-03	5.99E-03	8.21E-03	4.10E-03	2.94E-01
	Peak sensitivity value	-4.62E-17	1.95E-15	-5.32E-01	1.56E-16	1.84E-16	3.72E-02	2.89E-01	6.64E-02	3.05E-03	6.12E-03	4.27E-02	3.53E-19
	Shapiro-Wilk score	2.863E-01	2.425E-01	9.748E-01	9.085E-01	8.707E-01	9.640E-01	9.918E-01	9.849E-01	9.024E-01	8.906E-01	9.846E-01	7.087E-01

Table S23: Concentration control coefficients for *Saccharomyces cerevisiae* model with parameter variation of $\pm$50%

	(GlucoseMixedflowtoextracellularmedium)	(Glucoseuptake)	(Hexokinase)	(Phosphoglucoisomerase)	(Phosphofructokinase)	(Aldolase)	(Triosephosphateisomerase)	(Glyceraldehyde3-phosphatedehydrogenase)	(Phosphoenolpyruvatesynthesis)	(Pyruvatekinase)	(Pyruvatedecarboxylase)	(Alcoholdehydrogenase)
Acetaldehyde<br>Local<br>Min<br>Max<br>Normalized peak height<br>Peak sensitivity value<br>Shapiro-Wilk score	 2.55E-02<br>-4.51E-01<br>3.84E+00<br>2.33E-01<br>1.20E-03<br>5.995E-01	 1.10E-02<br>-1.90E-01<br>1.52E+00<br>1.32E-01<br>1.71E-03<br>4.881E-01	 1.99E+00<br>-2.88E-01<br>3.72E+00<br>3.29E-02<br>2.67E-03<br>8.969E-01	 8.76E-03<br>1.43E-05<br>1.56E-01<br>1.51E-02<br>6.17E-03<br>7.474E-01	 1.06E-01<br>1.74E-02<br>3.35E+00<br>3.02E-02<br>5.91E-02<br>6.047E-01	 -5.75E-15<br>-1.36E-13<br>1.87E-13<br>1.35E-01<br>-9.33E-17<br>6.655E-01	 -8.30E-02<br>-4.84E-01<br>-9.47E-05<br>4.76E-03<br>-4.49E-02<br>9.074E-01	 -1.82E-01<br>-5.04E-01<br>-4.27E-03<br>2.86E-03<br>-1.79E-01<br>9.785E-01	 -1.56E-02<br>-7.72E-02<br>-6.11E-05<br>5.56E-03<br>-9.43E-03<br>9.157E-01	 -4.70E-02<br>-3.01E-01<br>-5.56E-05<br>7.00E-03<br>-1.13E-02<br>8.364E-01	 -7.51E-15<br>-5.40E-13<br>3.78E-13<br>3.14E-01<br>-8.10E-17<br>4.361E-01	 -1.66E+00<br>-3.56E+00<br>-5.55E-01<br>4.74E-03<br>-1.26E+00<br>9.423E-01
Cytosolic glucose<br>Local<br>Min<br>Max<br>Normalized peak height<br>Peak sensitivity value<br>Shapiro-Wilk score	 2.04E+00<br>-1.60E+02<br>1.22E+04<br>7.61E-01<br>6.48E+00<br>9.388E-03	 8.77E-01<br>-8.08E+01<br>7.54E+03<br>9.55E-01<br>-7.44E-01<br>4.694E-03	 -3.34E+00<br>-2.48E+04<br>3.37E+02<br>9.52E-01<br>-1.54E+00<br>6.417E-03	 7.30E-03<br>-8.22E+00<br>5.90E+01<br>7.91E-01<br>1.98E-02<br>1.496E-02	 8.83E-02<br>-2.23E+02<br>4.98E+03<br>9.09E-01<br>-1.55E+00<br>5.613E-03	 4.39E-16<br>-8.34E-13<br>3.90E-13<br>6.97E-01<br>-1.57E-16<br>1.297E-01	 -2.81E-03<br>-1.94E+00<br>1.55E+01<br>8.75E-01<br>-5.14E-03<br>2.544E-02	 -6.15E-03<br>-6.70E+00<br>2.35E+01<br>8.38E-01<br>-1.28E-02<br>4.621E-02	 -5.28E-04<br>-1.24E+00<br>1.37E+00<br>5.80E-01<br>-1.48E-03<br>5.852E-02	 -1.59E-03<br>-1.05E+00<br>4.81E+00<br>8.32E-01<br>-2.39E-03<br>4.342E-02	 1.00E-15<br>-9.46E-13<br>1.13E-12<br>6.78E-01<br>8.29E-16<br>1.104E-01	 -5.63E-02<br>-1.20E+02<br>2.64E+02<br>5.22E-01<br>1.38E-01<br>4.503E-02
Glycerol<br>Local<br>Min<br>Max<br>Normalized peak height<br>Peak sensitivity value<br>Shapiro-Wilk score	 2.51E-02<br>-6.29E-02<br>3.15E+00<br>1.93E-01<br>3.05E-03<br>6.022E-01	 1.08E-02<br>-8.89E-02<br>1.58E+00<br>1.17E-01<br>2.28E-03<br>4.865E-01	 1.96E+00<br>-8.87E-01<br>3.76E+00<br>4.16E-02<br>3.26E-03<br>8.905E-01	 8.63E-03<br>1.19E-05<br>1.15E-01<br>1.20E-02<br>5.14E-03<br>7.665E-01	 1.04E-01<br>1.77E-02<br>2.96E+00<br>2.61E-02<br>6.33E-02<br>6.056E-01	 8.13E-15<br>-1.44E-13<br>1.41E-13<br>1.02E-01<br>1.19E-16<br>7.509E-01	 -8.18E-02<br>-4.92E-01<br>-1.03E-04<br>4.89E-03<br>-5.59E-02<br>9.075E-01	 -1.79E-01<br>-5.08E-01<br>-3.34E-03<br>2.80E-03<br>-1.28E-01<br>9.786E-01	 -1.53E-02<br>-8.14E-02<br>-6.22E-05<br>6.27E-03<br>-8.65E-03<br>9.068E-01	 -4.63E-02<br>-3.48E-01<br>-5.66E-05<br>8.23E-03<br>-1.24E-02<br>8.429E-01	 1.53E-14<br>-6.98E-13<br>6.32E-13<br>3.72E-01<br>-5.32E-16<br>4.920E-01	 -1.64E+00<br>-3.70E+00<br>-5.05E-01<br>5.50E-03<br>-1.20E+00<br>9.455E-01
EtOH<br>Local<br>Min<br>Max<br>Normalized peak height<br>Peak sensitivity value<br>Shapiro-Wilk score	 1.01E-02<br>-1.09E+00<br>9.33E-01<br>2.22E-01<br>5.50E-04<br>5.874E-01	 4.37E-03<br>-2.88E-01<br>6.60E-01<br>1.56E-01<br>8.88E-04<br>4.820E-01	 7.92E-01<br>-4.81E-01<br>2.17E+00<br>4.30E-02<br>3.22E-03<br>8.142E-01	 2.32E-03<br>-3.57E-03<br>4.11E-02<br>1.35E-02<br>1.85E-03<br>7.467E-01	 2.81E-02<br>-3.38E-02<br>9.72E-01<br>2.40E-02<br>2.10E-02<br>5.813E-01	 -3.86E-16<br>-1.16E-14<br>1.30E-14<br>1.17E-01<br>1.11E-19<br>6.837E-01	 9.18E-03<br>1.19E-04<br>7.70E-02<br>8.44E-03<br>4.39E-03<br>8.270E-01	 2.01E-02<br>5.50E-04<br>1.40E-01<br>7.03E-03<br>1.23E-02<br>8.663E-01	 1.72E-03<br>4.79E-05<br>2.47E-02<br>1.42E-02<br>7.02E-04<br>7.550E-01	 5.20E-03<br>5.21E-05<br>6.96E-02<br>1.66E-02<br>8.52E-04<br>7.284E-01	 -7.43E-17<br>-5.55E-14<br>3.44E-14<br>3.29E-01<br>-5.13E-18<br>5.142E-01	 1.84E-01<br>1.52E-02<br>9.45E-01<br>6.09E-03<br>7.23E-02<br>8.237E-01
ADP<br>Local<br>Min<br>Max<br>Normalized peak height<br>Peak sensitivity value<br>Shapiro-Wilk score	 1.29E-03<br>-4.16E+00<br>3.27E+00<br>5.71E-01<br>2.96E-03<br>4.125E-01	 5.55E-04<br>-9.47E-01<br>5.83E-01<br>4.03E-01<br>6.95E-04<br>5.142E-01	 1.01E-01<br>-2.61E+00<br>3.16E+00<br>1.40E-01<br>1.74E-04<br>6.871E-01	 -2.09E-02<br>-8.91E-02<br>3.35E-02<br>1.26E-02<br>5.61E-05<br>9.782E-01	 -2.53E-01<br>-3.58E+00<br>4.45E+00<br>4.18E-02<br>-1.56E-01<br>7.560E-01	 6.63E-16<br>-6.50E-14<br>2.05E-14<br>1.45E-01<br>8.70E-18<br>5.974E-01	 -3.90E-03<br>-7.27E-02<br>6.29E-02<br>3.20E-02<br>-1.05E-03<br>8.305E-01	 -8.54E-03<br>-9.48E-02<br>1.30E-01<br>2.31E-02<br>-3.08E-03<br>8.493E-01	 -7.33E-04<br>-1.18E-02<br>2.13E-02<br>4.39E-02<br>-2.01E-04<br>7.681E-01	 -2.21E-03<br>-4.67E-02<br>1.52E-02<br>3.16E-02<br>-3.64E-04<br>7.459E-01	 1.57E-15<br>-1.14E-13<br>8.19E-14<br>3.64E-01<br>-1.54E-17<br>4.841E-01	 -7.81E-02<br>-1.26E+00<br>1.07E+00<br>3.07E-02<br>-2.64E-02<br>8.522E-01
Glyceraldehyde 3-phosphate<br>Local<br>Min<br>Max<br>Normalized peak height<br>Peak sensitivity value<br>Shapiro-Wilk score	 2.26E-02<br>-2.03E+00<br>6.33E+00<br>3.79E-01<br>3.10E-03<br>5.904E-01	 9.73E-03<br>-2.07E-01<br>3.22E+00<br>1.82E-01<br>3.62E-03<br>4.785E-01	 1.76E+00<br>-1.31E+00<br>1.42E+01<br>9.37E-02<br>3.96E-03<br>9.147E-01	 1.27E-02<br>1.44E-05<br>2.92E-01<br>1.93E-02<br>8.04E-03<br>7.568E-01	 1.54E-01<br>2.39E-02<br>7.61E+00<br>3.92E-02<br>8.83E-02<br>6.361E-01	 6.19E-15<br>-1.08E-13<br>7.17E-14<br>8.04E-02<br>2.46E-17<br>7.588E-01	 1.56E-01<br>3.71E-03<br>8.25E-01<br>5.58E-03<br>7.56E-02<br>9.260E-01	 -5.55E-01<br>-3.86E+00<br>-3.48E-02<br>6.60E-03<br>-6.83E-01<br>9.818E-01	 -4.76E-02<br>-2.73E-01<br>-2.28E-03<br>6.22E-03<br>-3.30E-02<br>9.274E-01	 -1.44E-01<br>-1.07E+00<br>-1.06E-03<br>7.12E-03<br>-3.91E-02<br>8.595E-01	 1.37E-14<br>-4.16E-13<br>4.26E-13<br>3.69E-01<br>1.69E-16<br>5.072E-01	 -1.23E+00<br>-7.61E+00<br>2.88E-02<br>7.06E-03<br>-7.77E-01<br>9.219E-01
Dihydroxyacetone phosphate<br>Local<br>Min<br>Max<br>Normalized peak height<br>Peak sensitivity value<br>Shapiro-Wilk score	 2.40E-02<br>-1.22E+00<br>1.59E+01<br>4.44E-01<br>2.61E-03<br>5.890E-01	 1.03E-02<br>-2.09E-01<br>5.77E+00<br>3.21E-01<br>2.66E-03<br>4.664E-01	 1.88E+00<br>-8.30E+00<br>9.44E+01<br>1.34E-01<br>-3.15E-02<br>6.930E-01	 1.23E-02<br>1.99E-05<br>1.42E+00<br>9.32E-02<br>7.81E-03<br>5.750E-01	 1.49E-01<br>2.44E-02<br>1.90E+01<br>1.04E-01<br>9.09E-02<br>6.265E-01	 5.18E-15<br>-1.43E-10<br>3.52E-13<br>1.00E+00<br>-7.02E-15<br>7.336E-04	 -2.24E-01<br>-1.35E+00<br>-3.10E-03<br>4.99E-03<br>-1.60E-01<br>9.272E-01	 -4.91E-01<br>-1.95E+01<br>-2.47E-02<br>3.73E-02<br>-5.20E-01<br>8.138E-01	 -4.21E-02<br>-2.97E-01<br>-2.08E-03<br>8.22E-03<br>-2.79E-02<br>9.188E-01	 -1.27E-01<br>-1.24E+00<br>-5.92E-04<br>9.77E-03<br>-3.83E-02<br>8.419E-01	 1.04E-14<br>-8.79E-12<br>4.83E-13<br>6.04E-01<br>-4.27E-15<br>5.309E-02	 -1.03E+00<br>-6.00E+01<br>1.48E-01<br>5.83E-02<br>-4.83E-01<br>7.053E-01
Glucose-6-Phosphate<br>Local<br>Min<br>Max<br>Normalized peak height<br>Peak sensitivity value<br>Shapiro-Wilk score	 1.37E-02<br>-4.18E-01<br>1.50E+01<br>4.48E-01<br>6.29E-03<br>4.680E-01	 5.88E-03<br>-8.04E-01<br>1.84E+00<br>2.14E-01<br>1.07E-03<br>5.628E-01	 1.07E+00<br>-2.72E+00<br>1.34E+01<br>5.79E-02<br>2.58E-03<br>7.127E-01	 -2.72E-02<br>-2.23E-01<br>8.20E-03<br>7.89E-03<br>-1.67E-02<br>9.027E-01	 -3.29E-01<br>-1.44E+01<br>1.99E-01<br>4.37E-02<br>-2.02E-01<br>5.945E-01	 -8.33E-16<br>-7.99E-14<br>1.73E-13<br>3.07E-01<br>1.53E-17<br>4.006E-01	 5.10E-03<br>-2.62E-01<br>4.22E-02<br>4.12E-02<br>1.53E-03<br>6.840E-01	 1.11E-02<br>-3.82E-01<br>8.32E-02<br>2.75E-02<br>3.44E-03<br>7.177E-01	 9.57E-04<br>-4.01E-02<br>1.29E-02<br>4.08E-02<br>2.89E-04<br>6.989E-01	 2.89E-03<br>-7.78E-02<br>5.44E-02<br>3.88E-02<br>4.00E-04<br>7.816E-01	 -1.82E-15<br>-3.20E-13<br>4.07E-13<br>5.42E-01<br>-2.06E-16<br>2.523E-01	 1.02E-01<br>-4.37E+00<br>8.62E-01<br>4.05E-02<br>3.34E-02<br>7.268E-01
NAD<br>Local<br>Min<br>Max<br>Normalized peak height<br>Peak sensitivity value<br>Shapiro-Wilk score	 -4.87E-03<br>-2.45E+00<br>1.47E-01<br>3.94E-01<br>-1.93E-05<br>4.686E-01	 -2.10E-03<br>-8.72E-01<br>2.06E-01<br>2.97E-01<br>-6.75E-04<br>4.014E-01	 -3.80E-01<br>-2.38E+00<br>2.50E-01<br>6.90E-02<br>-1.93E-03<br>7.539E-01	 9.66E-04<br>-6.00E-02<br>3.82E-02<br>6.80E-02<br>-5.43E-05<br>7.645E-01	 1.17E-02<br>-1.71E+00<br>1.40E+00<br>1.23E-01<br>-1.61E-04<br>5.620E-01	 -3.24E-15<br>-4.63E-14<br>5.08E-14<br>1.01E-01<br>-2.61E-18<br>7.434E-01	 -8.00E-02<br>-6.55E-01<br>-1.79E-03<br>7.85E-03<br>-2.89E-02<br>8.733E-01	 -1.75E-01<br>-9.07E-01<br>-5.29E-03<br>5.04E-03<br>-4.81E-02<br>8.628E-01	 -1.50E-02<br>-3.66E-01<br>-5.63E-05<br>2.36E-02<br>-2.43E-03<br>7.318E-01	 -4.53E-02<br>-6.28E-01<br>-6.48E-05<br>1.80E-02<br>-2.26E-03<br>7.473E-01	 -6.78E-15<br>-2.96E-13<br>3.54E-13<br>4.09E-01<br>-3.13E-16<br>4.598E-01	 6.68E-01<br>3.29E-02<br>2.87E+00<br>4.72E-03<br>3.01E-01<br>8.987E-01
Extracellular acetaldehyde<br>Local<br>Min<br>Max<br>Normalized peak height<br>Peak sensitivity value<br>Shapiro-Wilk score	 2.55E-02<br>-1.31E+00<br>3.41E+00<br>2.26E-01<br>4.06E-03<br>6.041E-01	 1.10E-02<br>-6.02E-01<br>1.60E+00<br>1.70E-01<br>1.64E-03<br>4.951E-01	 1.99E+00<br>-5.90E-01<br>4.65E+00<br>3.97E-02<br>5.45E-03<br>8.971E-01	 8.78E-03<br>1.34E-05<br>1.22E-01<br>1.19E-02<br>5.20E-03<br>7.438E-01	 1.06E-01<br>1.33E-02<br>3.21E+00<br>2.73E-02<br>5.65E-02<br>6.050E-01	 -5.73E-15<br>-1.59E-13<br>1.27E-13<br>1.27E-01<br>3.53E-17<br>6.826E-01	 -8.32E-02<br>-5.69E-01<br>-9.77E-05<br>5.35E-03<br>-2.99E-02<br>9.090E-01	 -1.82E-01<br>-5.16E-01<br>-5.70E-03<br>2.79E-03<br>-9.67E-02<br>9.789E-01	 -1.56E-02<br>-8.76E-02<br>-7.63E-05<br>6.18E-03<br>-8.26E-03<br>9.156E-01	 -4.71E-02<br>-3.11E-01<br>-1.25E-04<br>7.36E-03<br>-1.05E-02<br>8.413E-01	 -8.63E-15<br>-8.38E-13<br>4.72E-13<br>3.68E-01<br>-8.24E-17<br>4.372E-01	 -1.67E+00<br>-3.85E+00<br>-5.47E-01<br>5.03E-03<br>-1.21E+00<br>9.422E-01
Phosphoenolpyruvate<br>Local<br>Min<br>Max<br>Normalized peak height<br>Peak sensitivity value<br>Shapiro-Wilk score	 1.38E-02<br>-1.03E-01<br>1.43E+00<br>1.46E-01<br>1.87E-03<br>5.967E-01	 5.93E-03<br>-2.87E-02<br>7.30E-01<br>8.65E-02<br>1.27E-03<br>5.033E-01	 1.08E+00<br>-1.96E-01<br>1.67E+00<br>2.15E-02<br>2.89E-03<br>8.000E-01	 6.08E-03<br>5.58E-06<br>6.10E-02<br>9.44E-03<br>4.37E-03<br>8.294E-01	 7.36E-02<br>1.57E-02<br>1.46E+00<br>2.06E-02<br>5.25E-02<br>6.241E-01	 2.62E-15<br>-2.97E-13<br>3.77E-13<br>1.92E-01<br>-8.42E-17<br>4.722E-01	 1.11E-03<br>-7.01E-03<br>4.01E-02<br>3.25E-02<br>3.58E-04<br>5.935E-01	 2.42E-03<br>-9.50E-03<br>5.90E-02<br>2.06E-02<br>1.15E-03<br>6.552E-01	 2.08E-04<br>-1.27E-03<br>9.17E-03<br>3.94E-02<br>6.31E-05<br>5.608E-01	 -1.20E+00<br>-2.00E+00<br>-1.05E+00<br>6.84E-03<br>-1.11E+00<br>8.639E-01	 7.14E-14<br>-6.94E-13<br>9.16E-13<br>4.44E-01<br>-5.47E-16<br>6.183E-01	 2.22E-02<br>-1.97E-01<br>4.55E-01<br>2.41E-02<br>7.58E-03<br>6.772E-01

	(Ethanolout)	(Ethanolflow)	(Glycerolsynthesis)	(Glycerolout)	(Glycerolflow)	(Acetaldehydeout)	(Acetaldehydeflow)	(Cyanide-Acetaldehydeflow)	(Cyanideflow)	(Storage)	(ATPconsumption)	(Adenylatekinase)
Acetaldehyde<br>Local<br>Min<br>Max<br>Normalized peak height<br>Peak sensitivity value<br>Shapiro-Wilk score	 0.00E+00<br>-7.54E-13<br>4.41E-13<br>5.90E-01<br>-4.59E-16<br>3.256E-01	 0.00E+00<br>-4.76E-12<br>3.18E-12<br>7.85E-01<br>-1.20E-15<br>2.681E-01	 2.83E-01<br>3.67E-03<br>6.93E-01<br>2.36E-03<br>3.37E-01<br>9.872E-01	 -5.12E-15<br>-3.47E-13<br>3.78E-13<br>1.10E-01<br>3.17E-17<br>8.150E-01	 7.96E-15<br>-2.57E-13<br>2.64E-13<br>1.23E-01<br>1.42E-16<br>7.587E-01	 -6.23E-02<br>-3.04E-01<br>-5.84E-03<br>4.74E-03<br>-4.53E-02<br>9.139E-01	 -3.24E-01<br>-8.01E-01<br>-4.85E-02<br>3.18E-03<br>-2.78E-01<br>9.765E-01	 -9.20E-02<br>-3.25E-01<br>-7.15E-03<br>3.87E-03<br>-7.79E-02<br>9.596E-01	 -7.34E-03<br>-1.56E-01<br>-2.54E-05<br>2.02E-02<br>-1.35E-03<br>6.566E-01	 -7.38E-02<br>-4.28E-01<br>8.07E-01<br>1.47E-02<br>-6.70E-02<br>7.030E-01	 1.26E-01<br>-1.87E-02<br>4.40E-01<br>1.87E-02<br>3.69E-04<br>9.830E-01	 1.05E-17<br>-1.40E-15<br>4.94E-16<br>2.69E-01<br>-6.68E-19<br>7.320E-01
Cytosolic glucose<br>Local<br>Min<br>Max<br>Normalized peak height<br>Peak sensitivity value<br>Shapiro-Wilk score	 0.00E+00<br>-3.23E-12<br>4.18E-13<br>9.26E-01<br>5.05E-16<br>1.735E-02	 0.00E+00<br>-4.93E-12<br>2.17E-12<br>8.67E-01<br>2.73E-15<br>5.904E-02	 9.58E-03<br>-4.29E+01<br>9.48E+00<br>6.02E-01<br>2.97E-02<br>3.989E-02	 7.63E-16<br>-8.47E-12<br>1.89E-12<br>7.33E-01<br>-3.84E-15<br>6.667E-02	 4.37E-17<br>-2.89E-12<br>7.44E-12<br>8.45E-01<br>-2.10E-15<br>4.696E-02	 2.30E-03<br>-7.47E+00<br>9.66E+00<br>4.98E-01<br>-6.48E-03<br>3.248E-02	 1.20E-02<br>-8.03E+01<br>2.34E+01<br>9.18E-01<br>3.15E-02<br>1.807E-02	 3.40E-03<br>-1.17E+01<br>3.18E+00<br>7.96E-01<br>3.38E-03<br>4.561E-02	 2.71E-04<br>-5.78E-01<br>2.18E-01<br>6.56E-01<br>7.95E-05<br>1.237E-01	 3.43E-01<br>-6.14E+01<br>1.80E+03<br>9.55E-01<br>8.33E-01<br>1.104E-02	 2.74E-02<br>-1.10E+02<br>5.37E+01<br>7.60E-01<br>-8.75E-03<br>2.101E-02	 7.34E-18<br>-7.76E-14<br>1.46E-13<br>9.95E-01<br>-6.25E-18<br>3.025E-03
Glycerol<br>Local<br>Min<br>Max<br>Normalized peak height<br>Peak sensitivity value<br>Shapiro-Wilk score	 0.00E+00<br>-6.10E-13<br>6.16E-13<br>6.62E-01<br>-3.24E-18<br>3.695E-01	 0.00E+00<br>-4.56E-12<br>5.22E-12<br>7.79E-01<br>2.81E-15<br>3.098E-01	 2.79E-01<br>3.55E-03<br>6.85E-01<br>2.59E-03<br>2.27E-01<br>9.868E-01	 -5.98E-01<br>-8.66E-01<br>-2.07E-01<br>2.45E-03<br>-6.32E-01<br>9.902E-01	 -4.02E-01<br>-7.93E-01<br>-1.34E-01<br>2.45E-03<br>-3.68E-01<br>9.902E-01	 6.70E-02<br>1.93E-03<br>2.82E-01<br>4.07E-03<br>5.80E-02<br>9.370E-01	 3.48E-01<br>1.79E-02<br>6.96E-01<br>2.80E-03<br>3.52E-01<br>9.974E-01	 9.89E-02<br>1.81E-03<br>3.54E-01<br>3.50E-03<br>8.55E-02<br>9.663E-01	 7.89E-03<br>2.45E-04<br>5.21E-02<br>6.91E-03<br>3.38E-03<br>8.493E-01	 -7.27E-02<br>-4.03E-01<br>7.74E-01<br>1.57E-02<br>-6.45E-02<br>7.051E-01	 1.24E-01<br>-1.53E-02<br>4.65E-01<br>2.35E-02<br>3.36E-04<br>9.828E-01	 1.03E-17<br>-4.56E-16<br>5.71E-16<br>2.60E-01<br>-4.65E-19<br>8.232E-01
EtOH<br>Local<br>Min<br>Max<br>Normalized peak height<br>Peak sensitivity value<br>Shapiro-Wilk score	 -1.45E-01<br>-4.23E-01<br>-2.94E-02<br>3.29E-03<br>-1.09E-01<br>9.563E-01	 -8.55E-01<br>-9.71E-01<br>-5.77E-01<br>3.29E-03<br>-8.91E-01<br>9.563E-01	 -3.13E-02<br>-2.47E-01<br>-8.10E-04<br>7.33E-03<br>-1.60E-02<br>8.627E-01	 -1.63E-16<br>-8.67E-14<br>6.75E-14<br>1.51E-01<br>7.17E-17<br>6.604E-01	 1.29E-15<br>-6.91E-14<br>5.78E-14<br>1.99E-01<br>-1.57E-17<br>6.205E-01	 -7.52E-03<br>-4.80E-02<br>-4.77E-04<br>5.68E-03<br>-3.78E-03<br>8.738E-01	 -3.91E-02<br>-1.52E-01<br>-5.00E-03<br>4.18E-03<br>-2.37E-02<br>9.271E-01	 -1.11E-02<br>-5.06E-02<br>-1.47E-03<br>4.90E-03<br>-7.73E-03<br>9.249E-01	 -8.85E-04<br>-1.64E-02<br>-7.48E-06<br>1.92E-02<br>-9.75E-05<br>7.147E-01	 -1.89E-02<br>-2.04E-01<br>3.87E-01<br>1.48E-02<br>-4.94E-04<br>7.331E-01	 5.21E-02<br>-2.00E-04<br>1.78E-01<br>2.32E-02<br>6.76E-05<br>9.786E-01	 2.13E-18<br>-2.18E-16<br>1.64E-15<br>4.15E-01<br>3.09E-19<br>1.816E-01
ADP<br>Local<br>Min<br>Max<br>Normalized peak height<br>Peak sensitivity value<br>Shapiro-Wilk score	 0.00E+00<br>-4.56E-14<br>5.94E-14<br>4.72E-01<br>3.86E-17<br>3.539E-01	 0.00E+00<br>-1.88E-13<br>4.08E-13<br>6.37E-01<br>2.36E-16<br>3.011E-01	 1.33E-02<br>-1.76E-01<br>1.46E-01<br>1.97E-02<br>4.36E-03<br>8.684E-01	 1.09E-15<br>-2.02E-13<br>1.49E-13<br>2.05E-01<br>8.12E-17<br>5.838E-01	 5.21E-17<br>-1.21E-13<br>1.29E-13<br>1.98E-01<br>1.12E-16<br>5.274E-01	 3.20E-03<br>-5.19E-02<br>3.66E-02<br>2.33E-02<br>1.23E-03<br>8.619E-01	 1.66E-02<br>-1.98E-01<br>1.39E-01<br>1.91E-02<br>9.44E-03<br>8.546E-01	 4.72E-03<br>-4.64E-02<br>3.84E-02<br>1.72E-02<br>2.57E-03<br>8.591E-01	 3.76E-04<br>-1.28E-02<br>1.15E-02<br>7.21E-02<br>4.90E-05<br>7.540E-01	 1.88E-01<br>-8.57E-01<br>5.39E-01<br>1.11E-02<br>1.82E-01<br>8.342E-01	 3.81E-02<br>-8.44E-02<br>2.98E-01<br>7.06E-03<br>2.93E-02<br>9.808E-01	 -4.97E-17<br>-3.43E-15<br>2.46E-15<br>2.50E-01<br>-1.61E-18<br>7.819E-01
Glyceraldehyde 3-phosphate<br>Local<br>Min<br>Max<br>Normalized peak height<br>Peak sensitivity value<br>Shapiro-Wilk score	 0.00E+00<br>-5.66E-13<br>2.87E-13<br>6.32E-01<br>1.11E-16<br>3.646E-01	 0.00E+00<br>-2.90E-12<br>2.44E-12<br>7.74E-01<br>-1.05E-15<br>3.181E-01	 -5.32E-01<br>-7.42E+00<br>-3.07E-02<br>1.20E-02<br>-3.45E-01<br>8.726E-01	 7.05E-15<br>-8.50E-13<br>1.05E-12<br>1.87E-01<br>-9.37E-17<br>6.734E-01	 -4.91E-15<br>-4.20E-13<br>4.44E-13<br>1.70E-01<br>1.07E-16<br>6.736E-01	 5.03E-02<br>-2.53E-04<br>3.14E-01<br>5.90E-03<br>2.22E-02<br>8.853E-01	 2.61E-01<br>-2.38E-03<br>7.62E-01<br>3.38E-03<br>1.79E-01<br>9.644E-01	 7.42E-02<br>-5.71E-04<br>3.47E-01<br>4.81E-03<br>4.06E-02<br>9.148E-01	 5.92E-03<br>-2.15E-04<br>6.96E-02<br>1.37E-02<br>1.15E-03<br>7.664E-01	 -1.10E-01<br>-1.10E+00<br>9.18E-01<br>1.66E-02<br>-9.03E-02<br>8.239E-01	 1.05E-01<br>-1.03E-01<br>5.80E-01<br>2.31E-02<br>1.75E-04<br>9.744E-01	 1.80E-17<br>-8.67E-16<br>9.61E-16<br>2.55E-01<br>6.26E-19<br>8.234E-01
Dihydroxyacetone phosphate<br>Local<br>Min<br>Max<br>Normalized peak height<br>Peak sensitivity value<br>Shapiro-Wilk score	 0.00E+00<br>-5.59E-13<br>4.46E-13<br>6.34E-01<br>2.50E-16<br>3.628E-01	 0.00E+00<br>-1.78E-12<br>2.40E-12<br>7.60E-01<br>-1.33E-15<br>3.077E-01	 -4.89E-01<br>-5.61E+01<br>-3.83E-02<br>9.53E-02<br>-3.47E-01<br>4.802E-01	 6.32E-15<br>-6.31E-13<br>1.23E-12<br>1.99E-01<br>1.63E-16<br>6.490E-01	 -3.90E-15<br>-4.33E-13<br>6.67E-13<br>2.01E-01<br>-1.45E-16<br>6.295E-01	 4.23E-02<br>-8.99E-03<br>3.88E-01<br>8.51E-03<br>2.06E-02<br>8.813E-01	 2.20E-01<br>-2.11E-02<br>4.62E+00<br>1.90E-02<br>1.30E-01<br>9.312E-01	 6.25E-02<br>-8.40E-03<br>4.70E-01<br>7.56E-03<br>3.68E-02<br>9.170E-01	 4.99E-03<br>-2.14E-03<br>3.16E-01<br>7.11E-02<br>8.88E-04<br>6.900E-01	 -1.06E-01<br>-2.96E+00<br>3.08E+00<br>5.10E-02<br>-9.00E-02<br>7.692E-01	 1.13E-01<br>-9.30E-02<br>2.84E+00<br>4.94E-02<br>-6.42E-04<br>9.226E-01	 1.69E-17<br>-1.26E-15<br>3.38E-15<br>2.65E-01<br>1.98E-18<br>5.978E-01
Glucose-6-Phosphate<br>Local<br>Min<br>Max<br>Normalized peak height<br>Peak sensitivity value<br>Shapiro-Wilk score	 0.00E+00<br>-2.19E-13<br>2.77E-13<br>6.69E-01<br>8.63E-17<br>2.353E-01	 0.00E+00<br>-7.81E-13<br>7.85E-13<br>6.11E-01<br>7.75E-16<br>2.386E-01	 -1.74E-02<br>-1.63E-01<br>5.95E-01<br>2.69E-02<br>-6.96E-03<br>7.469E-01	 -1.37E-15<br>-5.76E-13<br>4.80E-13<br>3.15E-01<br>-2.30E-16<br>4.103E-01	 -6.86E-17<br>-2.62E-13<br>5.95E-13<br>3.58E-01<br>-1.66E-16<br>3.809E-01	 -4.17E-03<br>-3.83E-02<br>1.74E-01<br>3.01E-02<br>-1.49E-03<br>7.394E-01	 -2.17E-02<br>-1.01E-01<br>6.18E-01<br>2.27E-02<br>-1.18E-02<br>7.614E-01	 -6.16E-03<br>-3.58E-02<br>2.17E-01<br>2.65E-02<br>-3.27E-03<br>7.664E-01	 -4.92E-04<br>-9.05E-03<br>4.67E-02<br>1.07E-01<br>-4.69E-05<br>6.236E-01	 -7.52E-01<br>-1.49E+00<br>4.69E-01<br>9.28E-03<br>-7.42E-01<br>8.460E-01	 -4.97E-02<br>-4.35E-01<br>5.17E-01<br>8.05E-03<br>-3.42E-02<br>9.693E-01	 -2.72E-17<br>-7.26E-16<br>8.19E-16<br>3.12E-01<br>-4.43E-19<br>8.842E-01
NAD<br>Local<br>Min<br>Max<br>Normalized peak height<br>Peak sensitivity value<br>Shapiro-Wilk score	 0.00E+00<br>-3.41E-13<br>2.24E-13<br>7.13E-01<br>3.02E-17<br>3.290E-01	 0.00E+00<br>-1.29E-12<br>1.17E-12<br>7.75E-01<br>3.14E-16<br>3.136E-01	 2.72E-01<br>1.37E-02<br>1.55E+00<br>5.38E-03<br>9.90E-02<br>9.176E-01	 -3.23E-15<br>-7.81E-13<br>3.71E-13<br>2.08E-01<br>-2.45E-16<br>6.101E-01	 2.80E-15<br>-3.49E-13<br>1.90E-13<br>1.81E-01<br>2.07E-16<br>6.112E-01	 -2.73E-02<br>-2.29E-01<br>-6.34E-04<br>8.13E-03<br>-1.01E-02<br>8.346E-01	 -1.42E-01<br>-6.75E-01<br>-6.43E-03<br>4.93E-03<br>-5.49E-02<br>9.078E-01	 -4.03E-02<br>-2.57E-01<br>-1.51E-03<br>6.74E-03<br>-1.95E-02<br>8.612E-01	 -3.22E-03<br>-4.32E-02<br>-3.55E-05<br>1.64E-02<br>-3.59E-04<br>7.292E-01	 -9.68E-03<br>-5.67E-01<br>1.73E-01<br>3.88E-02<br>3.47E-04<br>7.227E-01	 -2.81E-02<br>-2.44E-01<br>2.81E-03<br>1.75E-02<br>-3.44E-05<br>7.940E-01	 2.62E-18<br>-2.66E-16<br>2.90E-16<br>3.44E-01<br>1.96E-19<br>5.528E-01
Extracellular acetaldehyde<br>Local<br>Min<br>Max<br>Normalized peak height<br>Peak sensitivity value<br>Shapiro-Wilk score	 0.00E+00<br>-6.12E-13<br>4.07E-13<br>6.11E-01<br>5.88E-17<br>3.213E-01	 0.00E+00<br>-3.99E-12<br>2.45E-12<br>7.78E-01<br>4.69E-16<br>2.720E-01	 2.84E-01<br>4.11E-03<br>7.05E-01<br>2.42E-03<br>2.55E-01<br>9.877E-01	 -5.10E-15<br>-5.72E-13<br>3.76E-13<br>1.26E-01<br>-1.76E-17<br>7.875E-01	 7.94E-15<br>-2.92E-13<br>3.67E-13<br>1.25E-01<br>-3.00E-16<br>7.456E-01	 6.82E-02<br>1.73E-03<br>2.90E-01<br>4.14E-03<br>4.35E-02<br>9.379E-01	 -4.25E-01<br>-9.02E-01<br>-9.61E-02<br>3.15E-03<br>-4.09E-01<br>9.853E-01	 -1.21E-01<br>-3.33E-01<br>-2.51E-02<br>3.44E-03<br>-1.03E-01<br>9.715E-01	 -9.62E-03<br>-1.74E-01<br>-1.54E-04<br>1.59E-02<br>-3.02E-03<br>6.836E-01	 -7.39E-02<br>-3.80E-01<br>8.28E-01<br>1.51E-02<br>-6.40E-02<br>7.128E-01	 1.27E-01<br>-1.53E-02<br>4.59E-01<br>2.62E-02<br>1.32E-04<br>9.829E-01	 1.05E-17<br>-5.05E-16<br>6.88E-16<br>2.61E-01<br>1.62E-19<br>8.346E-01
Phosphoenolpyruvate<br>Local<br>Min<br>Max<br>Normalized peak height<br>Peak sensitivity value<br>Shapiro-Wilk score	 0.00E+00<br>-2.84E-13<br>3.20E-13<br>6.59E-01<br>-1.25E-16<br>3.215E-01	 0.00E+00<br>-2.93E-13<br>2.72E-13<br>7.06E-01<br>8.41E-17<br>3.173E-01	 -3.77E-03<br>-9.76E-02<br>2.44E-02<br>2.19E-02<br>-1.51E-03<br>6.587E-01	 4.71E-15<br>-3.61E-13<br>1.68E-13<br>2.04E-01<br>-2.18E-16<br>6.350E-01	 -1.00E-15<br>-1.42E-13<br>1.17E-13<br>2.06E-01<br>1.10E-16<br>5.866E-01	 -9.07E-04<br>-2.20E-02<br>7.66E-03<br>2.19E-02<br>-3.93E-04<br>6.667E-01	 -4.72E-03<br>-6.24E-02<br>3.64E-02<br>1.62E-02<br>-2.56E-03<br>7.220E-01	 -1.34E-03<br>-2.18E-02<br>5.72E-03<br>1.48E-02<br>-9.07E-04<br>7.136E-01	 -1.07E-04<br>-5.69E-03<br>1.16E-03<br>6.06E-02<br>-1.09E-05<br>5.554E-01	 -5.20E-02<br>-2.07E-01<br>3.95E-01<br>1.17E-02<br>-5.62E-02<br>7.577E-01	 6.64E-02<br>-1.17E-02<br>2.05E-01<br>6.33E-03<br>1.78E-03<br>9.836E-01	 9.58E-18<br>-3.23E-16<br>7.73E-16<br>2.51E-01<br>4.45E-19<br>7.793E-01

Fructose-6-Phosphate	Local	1.24E-02	5.33E-03	9.67E-01	8.44E-02	-3.38E-01	-8.95E-16	5.90E-03	1.29E-02	1.11E-03	3.34E-03	-2.13E-15	1.18E-01	0.00E+00	0.00E+00	-2.01E-02	-1.59E-15	-7.98E-17	-4.83E-03	-2.51E-02	-7.13E-03	-5.69E-04	-7.58E-01	-5.75E-02	-2.80E-17
	Min	-3.02E-01	-1.04E+00	-9.14E+00	1.05E-02	-1.80E+01	-1.49E-13	-2.15E-01	-5.40E-01	-4.53E-02	-8.07E-02	-2.05E-13	-4.25E+00	-1.92E-13	-6.18E-13	-1.49E-01	-3.78E-13	-4.06E-13	-3.12E-02	-1.17E-01	-3.76E-02	-1.08E-02	-1.93E+00	-4.22E-01	-1.70E-15
	Max	1.82E+01	5.31E+00	1.06E+01	3.23E-01	2.70E+00	9.38E-14	4.66E-02	9.86E-02	1.35E-02	7.10E-02	3.99E-13	7.76E-01	3.17E-13	1.88E-12	8.08E-01	4.10E-13	3.01E-13	2.32E-01	7.75E-01	1.79E-01	4.37E-02	3.76E+00	5.44E-01	1.22E-15
	Normalized peak height	4.47E-01	2.30E-01	9.03E-02	4.91E-03	5.93E-02	2.77E-01	3.31E-02	3.53E-02	4.14E-02	3.87E-02	4.88E-01	3.61E-02	6.64E-01	8.03E-01	3.13E-02	2.58E-01	2.75E-01	3.52E-02	2.43E-02	2.12E-02	9.45E-02	2.53E-02	8.07E-03	3.20E-01
	Peak sensitivity value	3.57E-03	-7.56E-04	1.01E-02	6.44E-02	-2.14E-01	8.98E-17	1.99E-03	5.04E-03	2.95E-04	2.43E-04	-1.85E-16	4.46E-02	6.00E-17	3.04E-16	-6.58E-03	2.29E-16	3.14E-16	-2.15E-03	-1.26E-02	-4.46E-03	-7.50E-05	-7.62E-01	-5.07E-02	8.84E-19
	Shapiro-Wilk score	4.495E-01	5.490E-01	6.934E-01	9.284E-01	5.992E-01	4.336E-01	7.155E-01	7.427E-01	7.250E-01	7.912E-01	3.026E-01	7.494E-01	2.241E-01	2.101E-01	7.716E-01	4.758E-01	4.528E-01	7.746E-01	7.829E-01	7.879E-01	6.507E-01	8.398E-01	9.751E-01	7.979E-01
Extracellular cyanide	Local	-1.89E-03	-8.11E-04	-1.47E-01	-6.48E-04	-7.85E-03	4.27E-16	6.15E-03	1.34E-02	1.15E-03	3.48E-03	6.32E-16	1.23E-01	0.00E+00	0.00E+00	-2.09E-02	3.66E-16	-5.96E-16	-5.03E-03	3.14E-02	-6.50E-02	7.46E-02	5.46E-03	-9.35E-03	-7.74E-19
	Min	-1.62E+00	-5.66E-01	-1.37E+00	-3.81E-02	-1.01E+00	-1.24E-14	2.84E-05	2.02E-04	1.06E-05	7.16E-06	-6.22E-14	3.46E-03	-3.89E-14	-1.88E-13	-2.02E-01	-7.77E-14	-5.66E-14	-2.98E-02	9.99E-04	-5.57E-01	3.32E-03	-2.82E-01	-1.14E-01	-7.31E-17
	Max	2.16E-02	5.42E-02	1.82E-01	4.80E-04	5.39E-03	1.54E-14	6.95E-02	1.40E-01	1.68E-02	5.67E-02	4.70E-14	1.18E+00	5.69E-14	2.23E-13	-1.97E-04	9.82E-14	7.69E-14	-1.63E-04	5.34E-01	-3.12E-03	7.18E-01	1.20E-01	1.67E-03	3.50E-17
	Normalized peak height	5.34E-01	3.91E-01	1.22E-01	6.03E-02	1.15E-01	2.09E-01	1.37E-02	1.17E-02	1.85E-02	2.77E-02	4.26E-01	1.15E-02	5.66E-01	7.88E-01	1.06E-02	2.39E-01	2.37E-01	6.74E-03	1.72E-02	8.94E-03	9.60E-03	6.71E-02	4.72E-02	2.80E-01
	Peak sensitivity value	-6.57E-04	-1.10E-04	-4.74E-04	-1.56E-04	-1.24E-03	4.14E-18	1.17E-03	5.57E-03	3.21E-04	3.76E-04	3.60E-17	3.11E-02	-4.73E-17	-9.83E-17	-6.36E-03	-3.69E-17	-6.19E-17	-2.19E-03	1.03E-02	-2.61E-02	3.30E-02	1.44E-03	-9.02E-06	4.72E-20
	Shapiro-Wilk score	4.330E-01	3.535E-01	6.827E-01	5.058E-01	4.858E-01	5.585E-01	7.282E-01	8.074E-01	7.325E-01	6.376E-01	3.024E-01	7.623E-01	2.402E-01	2.143E-01	7.944E-01	5.257E-01	4.826E-01	8.541E-01	6.841E-01	7.936E-01	7.769E-01	6.389E-01	7.439E-01	5.964E-01
Pyruvate	Local	9.01E-01	3.88E-01	7.04E+01	2.28E-01	2.76E+00	-6.96E-14	4.07E-02	8.91E-02	7.64E-03	2.31E-02	-7.79E+01	8.15E-01	0.00E+00	0.00E+00	-1.39E-01	-5.18E-14	1.49E-13	-3.34E-02	-1.73E-01	-4.93E-02	-3.93E-03	-1.87E+00	4.59E+00	2.26E-16
	Min	-1.20E+00	-1.68E+00	-5.20E+01	4.07E-05	1.61E-02	-2.49E-10	-5.91E-01	-1.16E+00	-7.85E-02	-4.92E-01	-2.79E+04	-1.23E+01	-1.53E-11	-4.76E-11	-5.09E+02	-2.35E-10	-1.35E-10	-8.48E+01	-5.43E+02	-1.80E+02	-1.51E+01	-1.14E+03	-4.33E-03	-3.54E-13
	Max	4.15E+03	7.32E+02	1.63E+04	9.50E+01	2.62E+04	5.59E-12	2.26E+02	3.23E+02	1.22E+01	2.21E+01	-1.46E+00	2.25E+03	8.70E-11	1.34E-10	1.71E+00	8.69E-11	1.01E-10	5.61E-01	1.98E+00	6.27E-01	9.59E-02	3.44E+02	8.95E+02	4.33E-14
	Normalized peak height	8.95E-01	8.81E-01	9.40E-01	9.25E-01	9.89E-01	9.59E-01	9.76E-01	9.62E-01	9.38E-01	8.19E-01	9.51E-01	9.54E-01	9.86E-01	9.77E-01	9.76E-01	9.75E-01	9.77E-01	9.45E-01	9.51E-01	9.74E-01	9.67E-01	8.62E-01	9.12E-01	9.46E-01
	Peak sensitivity value	8.81E-01	1.57E-01	5.33E+00	4.75E-02	1.31E+01	1.17E-13	-2.40E-02	-2.90E-02	1.30E-03	-7.31E-03	-1.54E+01	1.74E-01	-3.15E-14	-2.91E-14	-7.45E-02	3.19E-14	-9.00E-17	6.02E-03	7.63E-02	-4.75E-03	-2.91E-03	4.76E-01	4.43E-01	-1.52E-16
	Shapiro-Wilk score	2.271E-02	3.227E-02	2.101E-02	3.200E-02	4.920E-03	1.365E-03	4.218E-03	6.998E-03	1.593E-02	1.698E-02	1.998E-02	1.271E-02	3.143E-03	6.450E-03	7.166E-03	1.064E-02	1.117E-02	1.005E-02	8.212E-03	6.700E-03	1.016E-02	2.400E-02	3.001E-02	5.455E-03
Extracellular ethanol	Local	1.01E-02	4.37E-03	7.92E-01	2.32E-03	2.81E-02	-3.81E-16	9.18E-03	2.01E-02	1.72E-03	5.20E-03	-1.74E-16	1.84E-01	-3.41E-16	-1.00E+00	-3.13E-02	-1.73E-16	1.29E-15	-7.52E-03	-3.91E-02	-1.11E-02	-8.85E-04	-1.89E-02	5.21E-02	2.13E-18
	Min	-7.10E-01	-1.51E-01	-2.55E-01	-1.59E-03	-1.28E-02	-1.33E-14	1.17E-04	8.20E-04	6.06E-05	6.04E-05	-3.75E-14	1.54E-02	-2.72E-14	-1.00E+00	-2.34E-01	-7.80E-14	-3.86E-14	-5.51E-02	-1.52E-01	-4.88E-02	-1.58E-02	-2.22E-01	-1.43E-04	-4.84E-17
	Max	9.27E-01	5.06E-01	1.84E+00	4.41E-02	9.71E-01	1.17E-14	7.89E-02	1.40E-01	2.47E-02	6.44E-02	4.17E-14	9.37E-01	4.79E-14	-1.00E+00	-1.42E-03	7.09E-14	4.98E-14	-4.90E-04	-5.43E-03	-1.34E-03	-7.20E-06	3.14E-01	1.81E-01	1.50E-16
	Normalized peak height	2.26E-01	1.09E-01	3.87E-02	1.35E-02	2.32E-02	1.20E-01	8.54E-03	6.41E-03	1.43E-02	1.55E-02	3.30E-01	6.09E-03	3.55E-01	1.00E+00	6.54E-03	1.64E-01	1.42E-01	6.22E-03	4.16E-03	4.54E-03	1.87E-02	1.45E-02	1.92E-02	2.74E-01
	Peak sensitivity value	1.45E-03	9.58E-04	1.78E-03	1.72E-03	2.21E-02	-5.09E-18	3.78E-03	8.84E-03	7.13E-04	8.64E-04	-9.39E-18	7.11E-02	2.90E-17	-1.00E+00	-1.55E-02	-3.03E-17	-4.40E-17	-5.27E-03	-2.40E-02	-8.43E-03	-1.58E-04	-2.79E-04	1.29E-04	-7.32E-20
	Shapiro-Wilk score	5.853E-01	4.836E-01	8.158E-01	7.514E-01	5.785E-01	6.699E-01	8.211E-01	8.677E-01	7.626E-01	7.381E-01	5.140E-01	8.264E-01	4.412E-01	1.000E+00	8.615E-01	6.439E-01	6.182E-01	8.671E-01	9.280E-01	9.272E-01	7.221E-01	7.416E-01	9.787E-01	8.206E-01
Extracellular glycerol	Local	2.51E-02	1.08E-02	1.96E+00	8.63E-03	1.04E-01	8.14E-15	-8.18E-02	-1.79E-01	-1.53E-02	-4.63E-02	1.49E-14	-1.64E+00	0.00E+00	0.00E+00	2.79E-01	9.72E-15	-1.00E+00	6.70E-02	3.48E-01	9.89E-02	7.89E-03	-7.27E-02	1.24E-01	1.03E-17
	Min	-5.29E-01	-5.19E-02	-5.01E-01	8.43E-06	1.92E-02	-1.30E-13	-4.51E-01	-4.91E-01	-8.79E-02	-3.36E-01	-6.20E-13	-3.54E+00	-1.32E-12	-5.57E-12	3.21E-03	-6.20E-13	-1.00E+00	1.33E-03	1.52E-02	2.18E-03	2.82E-04	-4.39E-01	-3.15E-02	-6.00E-16
	Max	3.34E+00	1.53E+00	3.65E+00	1.37E-01	3.02E+00	1.39E-13	-1.58E-04	-3.91E-03	-9.69E-05	-1.04E-04	5.60E-13	-4.48E-01	7.77E-13	6.31E-12	6.77E-01	4.28E-13	-1.00E+00	2.78E-01	6.95E-01	3.58E-01	5.10E-02	8.69E-01	4.93E-01	5.61E-16
	Normalized peak height	2.35E-01	1.25E-01	3.46E-02	1.40E-02	2.61E-02	9.05E-02	4.67E-03	2.81E-03	6.72E-03	8.00E-03	3.67E-01	5.17E-03	5.92E-01	7.68E-01	2.33E-03	9.42E-02	1.00E+00	3.93E-03	2.84E-03	3.59E-03	7.32E-03	1.67E-02	2.90E-02	2.60E-01
	Peak sensitivity value	3.08E-03	1.08E-03	3.66E-03	6.08E-03	6.87E-02	1.26E-16	-4.19E-02	-1.29E-01	-7.34E-03	-8.32E-03	-1.05E-16	-1.19E+00	-8.89E-16	-4.52E-15	2.19E-01	-4.33E-16	-1.00E+00	4.33E-02	3.53E-01	7.57E-02	3.45E-03	-6.02E-02	2.64E-04	-1.87E-19
	Shapiro-Wilk score	5.998E-01	4.882E-01	8.909E-01	7.479E-01	6.033E-01	7.524E-01	9.065E-01	9.790E-01	9.125E-01	8.387E-01	5.020E-01	9.473E-01	3.763E-01	3.140E-01	9.873E-01	8.046E-01	1.000E+00	9.376E-01	9.974E-01	9.675E-01	8.455E-01	7.137E-01	9.835E-01	8.442E-01
Extracellular glucose	Local	2.57E+00	-1.40E-02	-2.53E+00	-3.55E-03	-4.30E-02	8.97E-17	-5.98E-04	-1.31E-03	-1.12E-04	-3.38E-04	2.12E-16	-1.20E-02	0.00E+00	0.00E+00	2.04E-03	1.65E-16	1.51E-17	4.89E-04	2.54E-03	7.23E-04	5.76E-05	2.55E-02	5.83E-03	-3.53E-18
	Min	2.73E-01	-2.14E+00	-2.48E+01	-2.15E-01	-2.16E+01	-1.15E-12	-3.50E-01	-5.66E-01	-9.14E-02	-7.60E-02	-4.81E-13	-5.86E+00	-4.25E-13	-1.07E-12	-8.05E-02	-5.40E-13	-5.01E-13	-2.01E-02	-8.09E-02	-2.60E-02	-9.13E-03	-1.33E+00	-2.24E-01	-4.97E-15
	Max	2.61E+01	9.75E-01	1.08E+00	1.48E-02	2.97E-01	1.97E-13	3.10E-02	4.67E-02	7.38E-03	2.92E-02	3.23E-13	5.17E-01	1.54E-13	1.21E-12	9.10E-01	3.83E-13	6.19E-13	2.57E-01	1.18E+00	3.07E-01	4.78E-02	1.14E+00	5.65E-01	1.46E-15
	Normalized peak height	1.33E-02	1.87E-01	3.53E-02	6.87E-02	2.11E-01	8.41E-01	3.42E-01	3.10E-01	2.77E-01	2.67E-01	7.84E-01	3.47E-01	7.94E-01	9.12E-01	2.81E-01	4.67E-01	7.06E-01	2.47E-01	3.11E-01	2.78E-01	4.23E-01	7.68E-02	9.34E-02	6.28E-01
	Peak sensitivity value	8.03E-01	-2.58E-03	-1.59E-02	-4.59E-04	-2.08E-02	-3.40E-16	-3.86E-05	-1.90E-04	-7.27E-05	-7.50E-05	6.35E-17	-2.25E-03	2.52E-16	1.97E-17	1.98E-04	4.41E-16	1.97E-16	2.33E-04	4.82E-04	1.04E-04	3.33E-06	6.33E-03	1.54E-03	1.35E-18
	Shapiro-Wilk score	7.544E-01	6.579E-01	6.788E-01	6.655E-01	4.563E-01	5.563E-02	3.772E-01	4.043E-01	3.418E-01	4.268E-01	1.319E-01	4.228E-01	6.645E-02	5.880E-02	4.120E-01	2.560E-01	1.978E-01	3.887E-01	4.401E-01	4.375E-01	3.369E-01	7.274E-01	6.380E-01	2.024E-01
1-3-Bisphosphoglycerate	Local	1.07E-02	4.61E-03	8.37E-01	4.44E-02	5.38E-01	5.30E-16	8.28E-03	1.81E-02	-2.84E-01	-8.56E-01	3.50E-14	1.66E-01	0.00E+00	0.00E+00	-2.82E-02	6.06E-16	-4.86E-16	-6.78E-03	-3.52E-02	-1.00E-02	-7.98E-04	-3.98E-01	-7.28E-03	7.56E-17
	Min	-1.05E+01	-1.20E+00	-1.15E+01	-2.68E-03	-3.71E-01	-4.18E-13	-7.86E-03	-1.55E-02	-9.44E-01	-1.70E+00	-3.74E-13	-1.54E-01	-4.06E-13	-1.06E-12	-6.46E-01	-4.43E-13	-3.21E-13	-1.05E-01	-7.38E-01	-1.30E-01	-2.52E-02	-1.05E+00	-6.00E-01	-2.45E-15
	Max	1.35E+00	6.41E-01	1.46E+00	2.05E-01	1.08E+01	3.50E-13	2.04E-01	3.34E-01	-4.86E-02	-6.33E-02	4.78E-13	4.23E+00	3.41E-13	7.48E-13	2.42E-02	5.40E-13	4.44E-13	3.21E-03	1.61E-02	5.67E-03	1.77E-03	4.93E-01	8.03E-02	4.22E-15
	Normalized peak height	4.35E-01	1.71E-01	8.84E-02	1.08E-02	2.97E-02	1.48E-01	2.50E-02	1.65E-02	3.03E-03	3.69E-03	2.88E-01	2.69E-02	6.45E-01	7.49E-01	1.92E-02	1.95E-01	2.26E-01	1.27E-02	1.92E-02	1.25E-02	3.57E-02	1.13E-02	1.56E-02	2.52E-01
	Peak sensitivity value	1.88E-03	5.88E-04	3.18E-03	1.15E-04	3.26E-01	1.66E-17	3.27E-03	8.81E-03	-2.57E-01	-8.10E-01	-3.54E-16	5.90E-02	-1.52E-16	-1.45E-16	-1.71E-02	4.38E-17	-2.02E-17	-3.79E-03	-1.90E-02	-6.74E-03	-7.99E-05	-4.09E-01	5.07E-03	-2.22E-18
	Shapiro-Wilk score	5.633E-01	6.197E-01	8.074E-01	9.645E-01	6.603E-01	4.860E-01	6.614E-01	7.525E-01	9.635E-01	9.991E-01	7.498E-01	7.255E-01	3.647E-01	2.924E-01	7.366E-01	5.507E-01	5.225E-01	7.577E-01	7.871E-01	7.830E-01	6.300E-01	8.478E-01	7.672E-01	8.597E-01
Fructose 1-6-bisphosphate	Local	4.60E-02	1.98E-02	3.59E+00	2.43E-02	2.94E-01	-9.76E-02	-8.09E-02	-1.00E+00	-8.59E-02	-2.59E-01	2.20E-14	-2.18E+00	0.00E+00	0.00E+00	-9.76E-01	1.28E-14	-8.40E-15	8.90E-02	4.63E-01	1.31E-01	1.05E-02	-2.10E-01	2.16E-01	3.37E-17
	Min	-3.57E+00	-5.87E-01	-4.08E+00	4.27E-05	4.88E-02	-6.05E-01	-1.17E+00	-2.27E+01	-4.78E-01	-1.89E+00	-1.01E-12	-5.76E+01	-1.35E-12	-4.40E-12	-5.54E+01	-1.10E-12	-7.44E-13	-2.58E-03	-1.72E-02	-2.71E-03	-2.52E-04	-4.05E+00	-2.15E-01	-6.85E-16
	Max	5.68E+01	1.48E+01	1.03E+02	1.05E+00	2.78E+01	-1.23E-02	4.57E-01	-7.01E-02	-3.93E-03	-1.19E-03	1.04E-12	2.13E-01	5.36E-13	2.61E-12	-7.39E-02	1.23E-12	2.00E-12	7.14E-01	2.88E+00	6.44E-01	3.03E-01	5.06E+00	5.49E+00	2.03E-15
	Normalized peak height	5.55E-01	3.43E-01	1.71E-01	3.53E-02	7.59E-02	5.25E-03	4.95E-03	2.15E-02	6.32E-03	7.32E-03	3.93E-01	2.75E-02	5.94E-01	7.56E-01	4.74E-02	1.62E-01	2.20E-01	7.60E-03	6.39E-03	5.04E-03	3.20E-02	3.84E-02	4.76E-02	2.53E-01
	Peak sensitivity value	2.20E-02	4.23E-03	2.69E-02	1.53E-02	1.74E-01	-7.06E-02	-6.37E-02	-1.10E+00	-6.11E-02	-7.96E-02	-3.05E-17	-1.15E+00	-7.38E-16	1.95E-15	-7.66E-01	2.69E-16	-5.00E-16	3.65E-02	3.52E-01	6.87E-02	1.72E-03	-1.69E-01	-1.61E-03	4.28E-20
	Shapiro-Wilk score	5.623E-01	4.627E-01	7.877E-01	6.642E-01	6.244E-01	8.895E-01	9.817E-01	8.431E-01	9.260E-01	8.473E-01	4.504E-01	7.816E-01	3.519E-01	3.364E-01	5.796E-01	6.884E-01	6.324E-01	8.782E-01	9.574E-01	9.129E-01	7.348E-01	7.900E-01	9.005E-01	8.249E-01
AMP	Local	4.17E-03	1.79E-03	3.25E-01	-6.74E-02	-8.16E-01	2.40E-15	-1.26E-02	-2.76E-02	-2.37E-03	-7.14E-03	6.43E-15	-2.52E-01	0.00E+00	0.00E+00	4.30E-02	3.70E-15	2.03E-16	1.03E-02	5.37E-02	1.52E-02	1.22E-03	6.08E-01	1.23E-01	6.38E-17
	Min	-3.67E-01	-4.33E-01	-4.63E+00	-3.10E-01	-1.98E+01	-1.01E-13	-4.36E-01	-7.70E-01	-7.81E-02	-1.65E-01	-5.64E-13	-6.24E+00	-1.95E-13	-1.63E-12	1.07E-03	-7.20E-13	-1.67E-12	4.56E-04	6.50E-03	1.42E-03	7.84E-06	-3.62E-01	8.88E-04	-1.27E-14
	Max	1.98E+01	2.54E+00	1.86E+01	-4.93E-06	-2.17E-01	4.08E-12	-2.14E-04	-5.67E-04	-6.72E-05	-2.85E-05	7.96E-13	-1.86E-02	5.40E-13	3.80E-12	1.14E+00	6.15E-13	4.90E-13	1.96E-01	6.96E-01	2.01E-01	3.81E-02	2.14E+00	1.10E+00	5.40E-15
	Normalized peak height	5.45E-01	3.27E-01	1.28E-01	1.36E-02	3.75E-02	4.98E-01	3.14E-02	2.34E-02	3.17E-02	2.70E-02	4.26E-01	2.50E-02	5.59E-01	6.22E-01	2.11E-02	1.92E-01	3.25E-01	1.54E-02	1.18E-02	1.28E-02	3.19E-02	1.92E-02	7.75E-03	2.51E-01
	Peak sensitivity value	5.48E-03	-1.30E-03	-4.99E-04	-1.60E-04	-4.81E-01	1.92E-15	-4.35E-03	-1.10E-02	-8.09E-04	-1.27E-03	-6.08E-16	-9.02E-02	-8.03E-17	2.61E-15	1.98E-02	4.12E-16	3.23E-17	4.85E-03	3.31E-02	1.05E-02	1.41E-04	6.03E-01	1.37E-01	5.47E-18
	Shapiro-Wilk score	3.436E-01	4.975E-01	5.669E-01	9.718E-01	6.246E-01	3.928E-02	6.759E-01	7.402E-01	6.930E-01	6.799E-01	3.996E-01	7.336E-01	3.007E-01	2.303E-01	7.388E-01	5.858E-01	4.449E-01	7.600E-01	7.995E-01	7.799E-01	6.376E-01	8.098E-01	9.354E-01	6.783E-01
ATP	Local	-1.59E-03	-6.83E-04	-1.24E-01	2.57E-02	3.11E-01	-8.56E-16	4.80E-03	1.05E-02	9.01E-04	2.72E-03	-2.17E-15	9.61E-02	0.00E+00	0.00E+00	-1.64E-02	-1.37E-15	-6.97E-17	-3.93E-03	-2.04E-02	-5.81E-03	-4.63E-04	-2.32E-01	-4.68E-02	2.55E-17
	Min	-1.70E+01	-1.52E+00	-9.88E+00	1.57E-05	6.69E-02	-1.25E-13	2.35E-05	2.35E-04	2.53E-05	2.27E-05	-4.42E-13	5.52E-03	-2.09E-13	-1.20E-12	-7.27E-01	-5.94E-13	-5.30E-13	-3.96E-01	-6.54E-01	-2.10E-01	-5.28E-02	-1.40E+00	-7.47E-01	-9.90E-16
	Max	9.85E-01	1.06E+00	1.59E+00	1.94E-01	1.81E+01	1.10E-13	4.18E-01	4.35E-01	7.35E-02	1.21E-01	3.94E-13	5.43E+00	4.82E-13	3.45E-12	-4.96E-04	7.47E-13	7.21E-13	-1.82E-04	-1.96E-03	-4.95E-04	-2.26E-06	7.57E-01	-5.67E-03	7.81E-16
	Normalized peak height	5.58E-01	5.13E-01	1.02E-01	7.17E-03	5.57E-02	2.45E-01	6.40E-02	2.85E-02	6.40E-02	4.00E-02	4.37E-01	4.67E-02	6.38E-01	8.21E-01	2.87E-02	2.96E-01	3.67E-01	6.40E-02	2.40E-02	2.62E-02	9.99E-02	1.17E-02	1.07E-02	2.92E-01
	Peak sensitivity value	2.51E-03	-3.02E-04	4.23E-03	1.76E-02	1.66E-01	1.02E-16	1.90E-03	4.37E-03	2.83E-04	5.68E-04	3.98E-16	3.00E-02	2.23E-16	-9.83E-16	-6.67E-03	-3.57E-16	-2.95E-16	-1.97E-03	-1.08E-02	-3.33E-03	-8.14E-05	-2.20E-01	-4.76E-02	-7.49E-19
	Shapiro-Wilk score	2.734E-01	4.562E-01	4.731E-01	8.936E-01	6.070E-01	4.744E-01	5.530E-01	6.249E-01	5.415E-01	5.808E-01	3.273E-01	6.391E-01	2.082E-01	1.387E-01	6.165E-01	4.502E-01	4.208E-01	6.216E-01	6.863E-01	6.743E-01	5.272E-01	8.236E-01	8.114E-01	8.794E-01
NADH	Local	9.35E-03	4.03E-03	7.30E-01	-1.86E-03	-2.25E-02	6.21E-15	1.54E-01	3.36E-01	2.88E-02	8.69E-02	1.30E-14	-1.28E+00	0.00E+00	0.00E+00	-5.23E-01	6.20E-15	-5.37E-15	5.25E-02	2.73E-01	7.75E-02	6.18E-03	1.86E-02	5.39E-02	-5.02E-18
	Min	-8.59E-02	-2.91E-02	-2.42E-01	-2.60E-02	-1.17E+00	-1.46E-13	1.18E-03	2.09E-02	5.31E-04	5.51E-04	-8.32E-13	-2.36E+00	-4.95E-13	-4.05E-12	-1.01E+00	-5.60E-13	-2.02E-13	3.56E-04	4.70E-03	5.96E-04	1.77E-04	-1.63E-01	-3.05E-03	-3.08E-16
	Max	1.98E+00	7.25E-01	1.93E+00	5.04E-02	1.40E+00	1.30E-13	8.36E-01	7.17E-01	1.66E-01	4.14E-01	6.18E-13	-5.99E-02	8.00E-13	3.85E-12	-3.38E-02	3.08E-13	2.60E-13	2.82E-01	6.89E-01	3.40E-01	3.68E-02	4.06E-01	2.39E-01	2.07E-16
	Normalized peak height	2.03E-01	1.63E-01	4.20E-02	2.05E-02	3.10E-02	1.09E-01	5.76E-03	3.55E-03	6.31E-03	4.96E-03	4.05E-01	4.98E-03	5.62E-01	7.79E-01	4.78E-03	1.08E-01	1.14E-01	4.67E-03	3.35E-03	3.96E-03	6.69E-03	1.07E-02	1.13E-02	2.75E-01
	Peak sensitivity value	1.77E-03	7.04E-04	2.05E-03	6.75E-05	-9.61E-03	2.62E-17	1.17E-01	3.25E-01	1.98E-02	2.10E-02	-2.68E-17	-1.06E+00	-6.23E-16	2.22E-15	-5.29E-01	1.11E-16	-5.96E-17	3.38E-02	2.67E-01	7.04E-02	2.17E-03	-3.49E-04	-2.64E-05	1.17E-19
	Shapiro-Wilk score	5.844E-01	4.832E-01	9.308E-01	9.045E-01	6.236E-01	7.050E-01	9.298E-01	9.980E-01	9.132E-01	8.941E-01	4.752E-01	9.873E-01	3.655E-01	3.052E-01	9.744E-01	8.237E-01	7.894E-01	9.404E-01	9.932E-01	9.670E-01	8.460E-01	8.664E-01	9.599E-01	7.770E-01

Table S24: Concentration control coefficients for *Saccharomyces cerevisiae* model with parameter variation of 0.1 – 10×

	(GlucoseMixedflowtoextracellularmedium)	(Glucoseuptake)	(Hexokinase)	(Phosphoglucoisomerase)	(Phosphofructokinase)	(Aldolase)	(Triosephosphateisomerase)	(Glyceraldehyde3-phosphatedehydrogenase)	(Phosphoenolpyruvatesynthesis)	(Pyruvatekinase)	(Pyruvatedecarboxylase)	(Alcoholdehydrogenase)
**Acetaldehyde**												
Local	2.55E-02	1.10E-02	1.99E+00	8.76E-03	1.06E-01	-5.75E-15	-8.30E-02	-1.82E-01	-1.56E-02	-4.70E-02	-7.51E-15	-1.66E+00
Min	-3.79E+00	-4.13E+00	-1.68E+03	-1.16E+00	-4.38E+01	-6.99E-11	-9.29E+00	-9.11E+01	-9.17E+01	-8.05E+01	-7.05E-11	-1.66E+03
Max	INF	INF	INF	INF	INF	INF	INF	INF	INF	INF	INF	INF
Normalized peak height	5.31E-01	2.92E-01	5.84E-01	3.15E-01	3.00E-01	9.60E-01	4.62E-01	6.55E-01	9.94E-01	8.54E-01	9.85E-01	9.68E-01
Peak sensitivity value	6.80E-03	2.13E-02	-4.86E+00	3.99E-03	3.40E-02	-2.81E-14	-5.63E-02	1.97E-02	2.34E-02	1.63E-02	3.92E-14	-1.31E+00
Shapiro-Wilk score	5.629E-01	6.775E-01	8.977E-03	4.836E-01	6.851E-01	3.581E-02	2.239E-01	7.958E-02	1.542E-03	4.869E-02	1.098E-02	2.736E-02
**Cytosolic glucose**												
Local	2.04E+00	8.77E-01	-3.34E+00	7.30E-03	8.83E-02	4.39E-16	-2.81E-03	-6.15E-03	-5.28E-04	-1.59E-03	1.00E-15	-5.63E-02
Min	-1.53E+02	-2.93E+02	-8.45E+04	-8.56E+02	-4.39E+03	-1.74E-10	-1.15E+02	-1.45E+02	-6.02E+00	-3.37E+02	-7.63E-11	-2.36E+03
Max	5.53E+04	2.71E+04	4.35E+02	6.27E+01	3.10E+02	1.78E-10	1.49E+01	6.49E+00	1.47E-01	1.90E+00	1.46E-10	1.10E+02
Normalized peak height	9.96E-01	9.95E-01	9.99E-01	9.87E-01	9.84E-01	9.79E-01	9.76E-01	4.87E-01	8.96E-01	9.91E-01	9.94E-01	9.69E-01
Peak sensitivity value	-1.48E+01	-5.26E+00	-3.20E+01	-2.21E-01	-2.20E+00	1.33E-13	-3.28E-02	-8.09E-02	2.54E-03	3.07E-02	-5.63E-14	1.61E-01
Shapiro-Wilk score	7.384E-04	1.821E-03	7.139E-04	7.792E-03	1.073E-02	8.235E-03	1.018E-02	2.374E-02	7.635E-03	4.003E-03	3.168E-03	1.095E-02
**Glycerol**												
Local	2.51E-02	1.08E-02	1.96E+00	8.63E-03	1.04E-01	8.13E-15	-8.18E-02	-1.79E-01	-1.53E-02	-4.63E-02	1.53E-14	-1.64E+00
Min	-2.59E+00	-2.58E+00	-4.32E+02	-1.92E+00	-6.03E+00	-3.93E-11	-5.61E+00	-1.01E+01	-3.72E+00	-7.77E+01	-7.28E-11	-9.53E+01
Max	6.73E+01	3.33E+01	2.12E+03	1.33E+01	2.04E+01	4.10E-11	5.17E+01	1.92E+02	5.46E+00	3.86E+00	5.77E-11	5.78E+02
Normalized peak height	5.99E-01	2.36E-01	8.76E-01	3.50E-01	1.52E-01	9.48E-01	6.63E-01	5.96E-01	8.02E-01	9.11E-01	9.68E-01	6.68E-01
Peak sensitivity value	2.84E-02	-1.26E-02	1.17E-01	3.25E-03	4.52E-02	2.40E-14	-2.87E-02	-1.12E-01	3.33E-03	-1.63E-02	5.54E-14	-6.78E-01
Shapiro-Wilk score	5.706E-01	6.778E-01	4.173E-02	4.629E-01	6.973E-01	3.227E-02	2.584E-01	8.363E-02	3.736E-02	4.822E-02	1.166E-02	7.914E-02
**EtOH**												
Local	1.01E-02	4.37E-03	7.92E-01	2.32E-03	2.81E-02	-3.86E-16	9.18E-03	2.01E-02	1.72E-03	5.20E-03	-7.43E-17	1.84E-01
Min	-1.16E+01	-9.51E+00	-2.29E+01	-2.19E+00	-1.49E+01	-2.48E-11	-2.20E-01	-8.29E+00	-3.23E-01	-1.50E-01	-1.69E-11	-1.55E+01
Max	INF	INF	INF	INF	INF	INF	INF	INF	INF	INF	INF	INF
Normalized peak height	2.66E-01	1.98E-01	9.38E-01	2.40E-01	1.83E-01	9.74E-01	9.32E-01	8.91E-01	9.79E-01	8.74E-01	9.98E-01	9.62E-01
Peak sensitivity value	-3.23E-03	8.11E-03	6.16E-01	1.22E-03	2.38E-02	-6.15E-16	1.11E-02	-3.57E-01	1.23E-03	-7.05E-03	8.24E-14	5.42E-01
Shapiro-Wilk score	6.180E-01	7.262E-01	2.898E-02	4.991E-01	7.067E-01	2.223E-02	3.185E-02	6.470E-03	1.235E-02	4.898E-02	3.293E-04	3.127E-02
**ADP**												
Local	1.29E-03	5.55E-04	1.01E-01	-2.09E-02	-2.53E-01	6.63E-16	-3.90E-03	-8.54E-03	-7.33E-04	-2.21E-03	1.57E-15	-7.81E-02
Min	-2.47E+01	-3.81E+00	-1.31E+02	-2.18E+00	-1.02E+01	-2.98E-10	-1.10E+00	-9.90E+01	-1.05E+00	-5.38E+00	-1.84E-11	-1.05E+02
Max	7.21E+00	4.58E+00	8.29E+02	2.27E+00	2.50E+01	3.60E-11	4.94E+01	8.09E+01	7.45E+00	7.90E+00	2.57E-11	6.73E+02
Normalized peak height	7.21E-01	3.33E-01	8.84E-01	2.13E-01	1.47E-01	9.80E-01	9.20E-01	9.79E-01	9.90E-01	8.66E-01	9.90E-01	8.76E-01
Peak sensitivity value	-9.84E-03	3.85E-03	1.72E-01	-1.25E-03	-1.22E-02	-1.53E-13	-1.90E-02	2.37E-02	7.87E-05	5.61E-03	3.50E-15	3.00E-01
Shapiro-Wilk score	2.114E-01	5.620E-01	2.598E-02	7.235E-01	7.274E-01	1.885E-03	2.210E-02	1.918E-02	4.535E-03	5.977E-02	6.269E-03	3.148E-02
**Glyceraldehyde 3-phosphate**												
Local	2.26E-02	9.73E-03	1.76E+00	1.27E-02	1.54E-01	6.19E-15	1.56E-01	-5.55E-01	-4.76E-02	-1.44E-01	1.37E-14	-1.23E+00
Min	-9.61E+00	-5.72E+00	-1.06E+02	-1.64E+00	-1.43E+01	-9.12E-10	-9.38E-01	-3.92E+02	-7.19E-01	-2.49E+03	-9.11E-11	-3.24E+02
Max	7.58E+02	6.70E+02	2.69E+03	1.56E+02	5.77E+02	5.90E-10	2.96E+01	5.48E+02	6.77E+00	1.23E+01	9.16E-11	1.61E+03
Normalized peak height	7.61E-01	6.98E-01	9.43E-01	8.06E-01	7.06E-01	9.97E-01	1.93E-01	8.36E-01	7.37E-01	8.82E-01	9.87E-01	4.80E-01
Peak sensitivity value	-1.88E-02	2.04E-02	1.30E+00	2.15E-02	1.52E-01	2.18E-13	2.37E-02	-4.22E-01	-3.80E-03	1.07E+00	-3.58E-14	-1.65E+00
Shapiro-Wilk score	7.906E-02	1.071E-01	4.297E-02	9.828E-02	1.443E-01	2.278E-03	4.605E-01	1.152E-01	1.448E-01	1.217E-02	1.056E-02	9.585E-02
**Dihydroxyacetone phosphate**												
Local	2.40E-02	1.03E-02	1.88E+00	1.23E-02	1.49E-01	5.18E-15	-2.24E-01	-4.91E-01	-4.21E-02	-1.27E-01	1.04E-14	-1.03E+00
Min	-1.50E+01	-2.81E+01	-6.05E+01	-1.84E+00	-2.48E+00	-3.09E-10	-1.72E+03	-5.11E+02	-6.41E-01	-1.21E+03	-6.47E-11	-2.54E+02
Max	INF	INF	INF	INF	INF	INF	INF	INF	INF	INF	INF	INF
Normalized peak height	9.31E-01	6.64E-01	5.36E-01	9.11E-01	6.93E-01	9.92E-01	8.67E-01	4.95E-01	9.46E-01	9.18E-01	9.87E-01	6.05E-01
Peak sensitivity value	5.11E-01	-2.95E-01	-4.28E-01	1.34E-02	-5.15E-02	-1.55E-13	5.05E-01	1.35E-01	-7.30E-03	4.25E-01	7.60E-14	-1.09E-01
Shapiro-Wilk score	4.162E-02	6.141E-02	4.972E-02	2.767E-02	6.366E-02	4.191E-03	1.109E-02	1.219E-01	4.665E-02	1.989E-02	4.678E-03	7.654E-02
**Glucose-6-Phosphate**												
Local	1.37E-02	5.88E-03	1.07E+00	-2.72E-02	-3.29E-01	-8.33E-16	5.10E-03	1.11E-02	9.57E-04	2.89E-03	-1.82E-15	1.02E-01
Min	-6.38E+00	-9.28E+00	-7.64E+00	-7.12E+00	-6.31E+01	-6.54E-11	-4.12E+00	-3.13E+00	-1.26E-01	-7.36E+00	-4.10E-10	-9.95E+00
Max	6.41E+01	6.93E+00	8.93E+02	5.35E-01	2.48E+00	1.51E-10	7.89E+01	1.14E+02	3.37E+00	1.74E+01	9.48E-12	2.04E+02
Normalized peak height	4.57E-01	2.21E-01	4.45E-01	1.03E-01	1.80E-01	9.85E-01	9.37E-01	8.54E-01	9.68E-01	6.65E-01	9.98E-01	4.80E-01
Peak sensitivity value	-1.06E-03	2.41E-03	9.15E-01	-4.20E-03	-1.72E-01	-5.54E-14	-1.06E-02	-1.82E-02	1.48E-03	-1.20E-02	4.41E-14	-1.66E-02
Shapiro-Wilk score	4.019E-01	7.386E-01	8.168E-02	6.938E-01	4.114E-01	7.989E-03	2.320E-02	3.315E-02	1.402E-02	4.980E-02	4.246E-04	1.013E-01
**NAD**												
Local	-4.87E-03	-2.10E-03	-3.80E-01	9.66E-04	1.17E-02	-3.24E-15	-8.00E-02	-1.75E-01	-1.50E-02	-4.53E-02	-6.78E-15	6.68E-01
Min	-1.80E+01	-1.33E+01	-2.76E+10	-3.64E+00	-1.83E+01	-2.93E-10	-2.96E+01	-5.18E+00	-1.25E+00	-1.33E+02	-2.50E-11	-1.01E+02
Max	6.07E+01	4.33E+01	2.05E+12	9.15E+00	7.25E+01	5.70E-11	2.43E-03	8.55E-02	1.00E-03	2.29E-05	4.04E-11	1.78E+02
Normalized peak height	7.66E-01	6.86E-01	1.00E+00	5.79E-01	6.73E-01	9.73E-01	3.81E-01	1.80E-02	2.59E-01	8.39E-01	9.62E-01	2.89E-01
Peak sensitivity value	-2.08E-02	-1.29E-02	4.47E+08	-3.38E-03	2.29E-02	1.24E-13	-1.24E-02	-6.64E-03	-8.75E-04	-6.63E-02	1.40E-14	3.73E-01
Shapiro-Wilk score	2.266E-01	2.707E-01	4.947E-05	2.667E-01	2.834E-01	7.763E-03	3.947E-01	9.084E-01	2.835E-01	8.740E-02	3.401E-02	5.054E-01
**Extracellular acetaldehyde**												
Local	2.55E-02	1.10E-02	1.99E+00	8.78E-03	1.06E-01	-5.73E-15	-8.32E-02	-1.82E-01	-1.56E-02	-4.71E-02	-8.63E-15	-1.67E+00
Min	-2.60E+00	-1.28E+00	-5.58E+01	-7.49E-01	-9.05E-01	-1.98E-11	-7.80E+00	-7.43E+00	-2.89E+00	-2.26E+02	-1.11E-10	-2.94E+01
Max	1.89E+02	3.50E+01	1.19E+03	1.17E+01	5.81E+01	1.98E-10	4.81E+01	4.11E+02	7.89E+00	8.79E+00	6.15E-11	5.82E+02
Normalized peak height	6.34E-01	3.99E-01	4.45E-01	3.47E-01	2.79E-01	9.60E-01	6.60E-01	9.21E-01	8.30E-01	9.62E-01	9.90E-01	5.14E-01
Peak sensitivity value	-1.12E-02	8.19E-03	-4.27E-01	4.86E-03	6.91E-02	8.69E-14	5.68E-04	-1.03E-01	3.82E-03	-3.22E-02	7.88E-15	-9.86E-01
Shapiro-Wilk score	4.278E-01	6.430E-01	4.786E-02	4.733E-01	6.663E-01	1.241E-02	3.136E-01	3.633E-02	2.867E-02	3.121E-02	8.505E-03	6.084E-02
**Phosphoenolpyruvate**												
Local	1.38E-02	5.93E-03	1.08E+00	6.08E-03	7.36E-02	2.62E-15	1.11E-03	2.42E-03	2.08E-04	-1.20E+00	7.14E-14	2.22E-02
Min	-2.84E+01	-2.24E+01	-3.80E+03	-2.83E+00	-8.07E+00	-9.83E-10	-3.85E+02	-2.14E+03	-1.05E+01	-1.62E+03	-1.87E-10	-2.52E+03
Max	4.54E+02	1.03E+03	2.75E+03	1.71E+02	3.66E+02	2.04E-09	2.29E+01	6.71E+02	5.04E+00	1.54E+01	6.04E-10	1.62E+03
Normalized peak height	4.59E-01	9.58E-01	9.84E-01	8.91E-01	5.75E-01	9.95E-01	9.65E-01	9.99E-01	9.94E-01	9.71E-01	9.95E-01	9.39E-01
Peak sensitivity value	-2.17E-01	3.50E-01	8.38E-01	3.19E-02	-2.46E-02	7.85E-13	-1.62E-01	1.58E-01	1.10E-03	-1.79E+00	-1.28E-13	-1.57E+00
Shapiro-Wilk score	5.096E-02	4.194E-02	2.422E-02	6.281E-02	8.499E-02	2.747E-03	1.488E-03	6.827E-04	6.021E-03	3.281E-02	2.491E-03	6.895E-03

	(Ethanolout)	(Ethanolflow)	(Glycerolsynthesis)	(Glycerolout)	(Glycerolflow)	(Acetaldehydeout)	(Acetaldehydeflow)	(Cyanide-Acetaldehydeflow)	(Cyanideflow)	(Storage)	(ATPconsumption)	(Adenylatekinase)
**Acetaldehyde**												
Local	0.00E+00	0.00E+00	2.83E-01	-5.12E-15	7.96E-15	-6.23E-02	-3.24E-01	-9.20E-02	-7.34E-03	-7.38E-02	1.26E-01	1.05E-17
Min	-5.10E-11	-1.73E-10	-1.97E+02	-8.20E-10	-1.13E-10	-3.38E+02	-1.46E+03	-1.55E+02	-5.52E+00	-1.38E+01	-9.38E+03	-5.50E-09
Max	INF	INF	INF	INF	INF	INF	INF	INF	INF	INF	INF	INF
Normalized peak height	9.59E-01	9.82E-01	5.91E-01	9.65E-01	9.82E-01	7.84E-01	9.97E-01	8.05E-01	6.37E-01	4.41E-01	9.89E-01	1.00E+00
Peak sensitivity value	-7.72E-16	5.13E-13	-6.19E-02	-1.43E-12	-5.89E-16	-3.08E-01	-1.08E+00	-4.35E-03	-1.08E-03	-5.04E-02	2.25E+00	2.17E-10
Shapiro-Wilk score	3.841E-02	4.380E-03	1.016E-01	4.830E-03	1.563E-02	5.901E-02	1.008E-02	5.018E-02	2.628E-01	5.832E-01	4.475E-03	4.923E-05
**Cytosolic glucose**												
Local	0.00E+00	0.00E+00	9.58E-03	7.63E-16	4.37E-17	2.30E-03	1.20E-02	3.40E-03	2.71E-04	3.43E-01	2.74E-02	7.34E-18
Min	-2.73E-11	-1.28E-10	-1.35E+01	-6.21E-10	-4.34E-10	-1.19E+01	-5.65E+01	-1.50E+01	-4.08E+00	-1.84E+02	-1.66E+01	-4.37E-12
Max	8.16E-11	9.00E-11	2.99E+02	4.44E-10	3.01E-10	3.66E+02	7.24E+02	1.89E+02	3.29E+01	2.69E+03	1.40E+03	2.26E-08
Normalized peak height	9.79E-01	9.75E-01	9.66E-01	9.69E-01	9.94E-01	9.44E-01	9.86E-01	9.74E-01	9.67E-01	7.29E-01	8.25E-01	1.00E+00
Peak sensitivity value	-1.88E-14	-9.17E-14	8.48E-02	2.88E-13	2.53E-14	-1.39E-02	6.81E-02	8.52E-03	3.16E-04	1.54E+00	-3.70E-01	6.92E-12
Shapiro-Wilk score	1.519E-02	7.679E-03	1.329E-02	2.034E-02	4.857E-03	1.299E-02	8.972E-03	8.917E-03	1.126E-02	1.385E-02	1.418E-02	4.739E-05
**Glycerol**												
Local	0.00E+00	0.00E+00	2.79E-01	-5.98E-01	-4.02E-01	6.70E-02	3.48E-01	9.89E-02	7.89E-03	-7.27E-02	1.24E-01	1.03E-17
Min	-8.15E-11	-2.48E-10	-1.94E+02	-9.99E-01	-9.97E-01	-2.70E+02	-4.09E+02	-1.24E+02	-1.39E+00	-1.56E+01	-2.11E+03	-5.88E-09
Max	1.74E-10	9.58E-10	8.40E+00	-2.65E-03	-7.29E-04	4.10E+01	4.84E+01	5.86E+00	6.92E-01	1.06E+01	4.32E+02	8.09E-09
Normalized peak height	9.43E-01	9.76E-01	5.69E-01	4.55E-03	4.55E-03	6.39E-01	8.68E-01	4.40E-01	3.48E-01	1.28E-01	9.63E-01	1.00E+00
Peak sensitivity value	1.10E-13	-5.14E-13	1.62E-02	-9.36E-01	-6.40E-02	1.73E-01	1.87E-01	9.36E-02	-5.91E-04	1.09E-03	4.10E-01	-6.17E-12
Shapiro-Wilk score	2.690E-02	6.692E-03	1.188E-01	8.194E-01	8.191E-01	9.881E-02	4.586E-02	7.172E-02	3.701E-01	6.444E-01	1.964E-02	1.655E-04
**EtOH**												
Local	-1.45E-01	-8.55E-01	-3.13E-02	-1.63E-16	1.29E-15	-7.52E-03	-3.91E-02	-1.11E-02	-8.85E-04	-1.89E-02	5.21E-02	2.13E-18
Min	-9.93E-01	-1.00E+00	-1.02E+03	-2.84E-10	-1.18E-10	-4.96E+02	-3.97E+02	-2.25E+02	-1.98E+00	-1.58E+01	-1.86E+03	-1.42E-09
Max	INF	INF	INF	INF	INF	INF	INF	INF	INF	INF	INF	INF
Normalized peak height	1.44E-03	1.44E-03	9.88E-01	9.10E-01	9.88E-01	9.74E-01	9.12E-01	9.61E-01	5.17E-01	1.84E-01	9.77E-01	1.00E+00
Peak sensitivity value	-2.66E-02	-9.73E-01	-1.26E-01	-5.15E-13	-9.18E-14	-1.62E-01	-6.62E-02	-3.45E-02	-1.22E-03	-7.43E-03	-4.35E-01	4.10E-14
Shapiro-Wilk score	9.675E-01	9.675E-01	8.679E-03	1.038E-02	5.181E-03	1.639E-02	2.695E-02	1.742E-02	2.917E-01	5.595E-01	1.515E-02	1.090E-04
**ADP**												
Local	0.00E+00	0.00E+00	1.33E-02	1.09E-15	5.21E-17	3.20E-03	1.66E-02	4.72E-03	3.76E-04	1.88E-01	3.81E-02	-4.97E-17
Min	-7.28E-11	-4.40E-10	-8.81E+01	-2.15E-10	-4.36E-11	-3.35E+02	-3.04E+02	-5.57E+01	-3.76E-01	-8.44E+00	-8.28E+02	-1.16E-09
Max	1.92E-11	8.63E-11	1.02E+02	1.11E-09	1.01E-10	5.06E+01	4.09E+01	1.35E+01	2.15E-01	3.14E+00	1.27E+02	1.31E-09
Normalized peak height	9.88E-01	9.90E-01	9.49E-01	9.88E-01	9.78E-01	9.87E-01	9.74E-01	9.05E-01	5.56E-01	6.71E-02	6.34E-01	9.99E-01
Peak sensitivity value	-8.00E-15	-2.34E-13	-5.21E-02	3.87E-13	5.50E-14	-9.11E-02	5.31E-03	-2.54E-02	-2.63E-04	-2.57E-03	-4.55E-01	-6.25E-13
Shapiro-Wilk score	7.730E-03	1.135E-03	2.477E-02	4.830E-03	9.480E-03	1.555E-02	1.812E-02	2.299E-02	3.125E-01	8.814E-01	2.112E-02	6.058E-04
**Glyceraldehyde 3-phosphate**												
Local	0.00E+00	0.00E+00	-5.32E-01	7.05E-15	-4.91E-15	5.03E-02	2.61E-01	7.42E-02	5.92E-03	-1.10E-01	1.05E-01	1.80E-17
Min	-6.72E-11	-3.08E-10	-5.54E+02	-6.25E-10	-3.21E-10	-1.05E+03	-4.44E+02	-1.14E+02	-2.30E+00	-8.11E+01	-2.69E+03	-8.38E-11
Max	8.22E-10	2.24E-10	2.19E+01	1.70E-08	2.19E-09	1.17E+02	1.25E+02	4.68E+01	1.10E+01	1.34E+02	1.28E+02	2.19E-07
Normalized peak height	9.92E-01	9.76E-01	4.80E-01	9.98E-01	9.92E-01	9.40E-01	5.75E-01	4.54E-01	6.76E-01	5.48E-01	9.69E-01	1.00E+00
Peak sensitivity value	-1.18E-13	1.67E-13	-8.34E-01	-5.20E-13	-1.05E-12	1.13E-01	3.76E-01	1.04E-01	-3.22E-03	-5.23E-02	-6.81E-03	2.57E-11
Shapiro-Wilk score	2.978E-03	1.414E-02	1.578E-01	5.112E-04	7.222E-04	3.480E-02	8.559E-02	1.044E-01	1.342E-01	3.407E-01	1.481E-02	5.007E-05
**Dihydroxyacetone phosphate**												
Local	0.00E+00	0.00E+00	-4.89E-01	6.32E-15	-3.90E-15	4.23E-02	2.20E-01	6.25E-02	4.99E-03	-1.06E-01	1.13E-01	1.69E-17
Min	-4.09E-11	-1.55E-10	-5.69E+02	-1.10E-09	-5.64E-10	-5.87E+02	-6.84E+02	-1.91E+02	-2.20E+01	-6.85E+02	-1.97E+03	-7.03E-08
Max	INF	INF	INF	INF	INF	INF	INF	INF	INF	INF	INF	INF
Normalized peak height	9.13E-01	8.79E-01	5.25E-01	9.54E-01	9.90E-01	8.27E-01	9.05E-01	8.55E-01	9.01E-01	7.35E-01	8.48E-01	1.00E+00
Peak sensitivity value	5.22E-14	1.41E-13	-9.12E-01	-8.56E-13	2.01E-13	1.47E-02	8.98E-02	4.63E-02	2.97E-03	6.96E-02	9.76E-01	-1.20E-11
Shapiro-Wilk score	3.100E-02	1.962E-02	1.369E-01	1.265E-02	4.889E-03	5.701E-02	4.453E-02	5.554E-02	8.281E-02	1.094E-01	1.735E-02	6.026E-05
**Glucose-6-Phosphate**												
Local	0.00E+00	0.00E+00	-1.74E-02	-1.37E-15	-6.86E-17	-4.17E-03	-2.17E-02	-6.16E-03	-4.92E-04	-7.52E-01	-4.97E-02	-2.72E-17
Min	-2.85E-10	-2.26E-10	-1.56E+02	-3.24E-10	-6.90E-11	-8.39E+01	-1.04E+02	-3.80E+01	-8.55E-01	-5.73E+00	-8.92E+02	-1.46E-08
Max	4.08E-11	8.93E-11	5.40E+00	4.87E-10	7.61E-11	2.74E+00	2.89E+00	1.59E+00	6.09E-01	3.85E+00	7.30E+00	2.98E-07
Normalized peak height	9.87E-01	9.90E-01	9.06E-01	9.74E-01	9.65E-01	6.06E-01	7.80E-01	8.29E-01	4.28E-01	2.18E-02	9.09E-01	1.00E+00
Peak sensitivity value	-1.23E-13	-8.69E-14	-2.00E-02	-9.97E-14	-5.17E-14	1.47E-02	-4.29E-02	-1.31E-02	6.63E-04	-9.96E-01	-3.47E-01	-1.92E-11
Shapiro-Wilk score	2.436E-03	4.246E-03	3.418E-02	2.884E-02	1.927E-02	5.523E-02	5.360E-02	4.988E-02	2.753E-01	9.670E-01	3.942E-02	5.239E-05
**NAD**												
Local	0.00E+00	0.00E+00	2.72E-01	-3.23E-15	2.80E-15	-2.73E-02	-1.42E-01	-4.03E-02	-3.22E-03	-9.68E-03	-2.81E-02	2.62E-18
Min	-3.69E-11	-4.37E-10	-1.41E-01	-2.50E-10	-4.59E-11	-2.69E+01	-1.35E+02	-5.56E+01	-2.33E+00	-1.99E+01	-2.05E+12	-1.99E+08
Max	1.37E-11	1.50E-10	2.96E+01	1.68E-10	1.95E-11	5.77E+01	3.75E+01	2.57E+01	1.08E-01	6.83E+00	2.78E+10	5.15E+09
Normalized peak height	9.41E-01	9.88E-01	4.86E-02	9.44E-01	8.81E-01	3.03E-01	4.66E-01	7.40E-01	5.98E-01	4.48E-01	1.00E+00	1.00E+00
Peak sensitivity value	-1.53E-15	1.39E-14	5.24E-02	-1.13E-13	3.06E-14	-7.88E-02	-3.74E-03	-2.79E-02	-2.14E-04	3.82E-03	-2.49E+08	1.23E+06
Shapiro-Wilk score	5.181E-02	8.280E-03	8.058E-01	3.690E-02	3.839E-02	4.955E-01	3.086E-01	3.123E-01	2.764E-01	4.345E-01	4.917E-05	5.329E-05
**Extracellular acetaldehyde**												
Local	0.00E+00	0.00E+00	2.84E-01	-5.10E-15	7.94E-15	6.82E-02	-4.25E-01	-1.21E-01	-9.62E-03	-7.39E-02	1.27E-01	1.05E-17
Min	-9.08E-11	-2.49E-10	-4.15E+02	-5.16E-10	-2.57E-10	-3.75E+02	-2.97E+02	-3.43E+02	-2.45E+00	-1.80E+01	-1.19E+03	-6.13E-09
Max	6.28E-11	4.99E-10	7.93E+00	9.91E-10	1.99E-10	1.18E+01	1.53E+01	1.24E+00	4.18E-01	8.95E+00	5.64E+01	2.92E-09
Normalized peak height	9.43E-01	9.81E-01	7.93E-01	9.81E-01	9.89E-01	6.09E-01	4.06E-01	4.92E-01	2.28E-01	1.06E-01	9.54E-01	1.00E+00
Peak sensitivity value	6.16E-14	-2.79E-13	9.49E-02	1.23E-13	-8.75E-14	3.58E-02	-4.46E-01	-3.13E-01	-2.15E-03	-7.04E-02	-1.77E-01	-9.16E-13
Shapiro-Wilk score	3.245E-02	1.003E-02	6.209E-02	1.340E-02	7.324E-03	5.932E-02	6.582E-02	5.168E-02	3.687E-01	6.538E-01	2.294E-02	3.188E-04
**Phosphoenolpyruvate**												
Local	0.00E+00	0.00E+00	-3.77E-03	4.71E-15	-1.00E-15	-9.07E-04	-4.72E-03	-1.34E-03	-1.07E-04	-5.20E-02	6.64E-02	9.58E-18
Min	-3.11E-11	-2.01E-10	-6.77E+02	-2.28E-09	-9.49E-10	-2.83E+02	-1.11E+03	-6.16E+02	-4.81E+00	-1.50E+02	-2.75E+03	-2.06E-08
Max	1.27E-10	2.73E-10	2.54E+03	1.82E-10	2.27E-10	2.69E+02	1.74E+03	5.09E+02	1.65E-01	4.54E+01	3.80E+03	4.22E-07
Normalized peak height	9.79E-01	9.69E-01	9.97E-01	9.53E-01	9.77E-01	9.35E-01	9.94E-01	9.93E-01	9.13E-01	5.57E-01	9.91E-01	1.00E+00
Peak sensitivity value	-4.01E-14	2.25E-13	-1.06E-01	1.16E-12	5.60E-13	1.56E-01	3.39E-01	1.81E-01	-1.41E-03	-4.87E-02	-1.18E-01	-5.77E-13
Shapiro-Wilk score	1.007E-02	4.374E-03	1.180E-03	5.069E-03	1.437E-03	1.857E-02	2.803E-03	2.713E-03	5.367E-02	2.416E-01	7.841E-03	9.125E-05

Fructose-6-Phosphate	Local	1.24E-02	5.33E-03	9.67E-01	8.44E-02	-3.38E-01	-8.95E-16	5.90E-03	1.29E-02
	Min	-8.08E+00	-1.14E+01	-3.34E+01	-2.27E+00	-5.96E+01	-6.00E-11	-6.26E+00	-1.73E+01
	Max	INF	INF	INF	INF	INF	INF	INF	INF
	Normalized peak height	4.52E-01	2.51E-01	4.02E-01	7.77E-02	1.25E-01	9.84E-01	8.88E-01	6.55E-01
	Peak sensitivity value	-1.50E-02	1.75E-03	-1.03E-01	1.12E-02	-1.95E-01	5.90E-14	8.24E-03	-2.30E-02
	Shapiro-Wilk score	3.761E-01	6.959E-01	7.537E-02	7.455E-01	4.718E-01	9.987E-03	1.116E-01	5.735E-02
Extracellular cyanide	Local	-1.89E-03	-8.11E-04	-1.47E-01	-6.48E-04	-7.85E-03	4.27E-16	6.15E-03	1.34E-02
	Min	-1.48E+01	-6.19E+00	-1.32E+01	-3.02E+00	-6.29E+00	-4.09E-11	-5.50E-01	-2.57E+00
	Max	3.57E-01	1.70E-01	4.39E+00	1.03E-01	1.72E-01	3.89E-11	2.84E+00	3.30E+00
	Normalized peak height	6.99E-01	5.19E-01	3.82E-01	3.95E-01	4.11E-01	9.91E-01	8.03E-01	5.25E-01
	Peak sensitivity value	1.19E-03	1.27E-03	6.93E-03	-1.74E-03	7.70E-04	2.09E-14	5.99E-04	3.07E-03
	Shapiro-Wilk score	2.319E-01	3.012E-01	3.663E-01	2.091E-01	2.768E-01	5.347E-03	1.323E-01	2.437E-01
Pyruvate	Local	9.01E-01	3.88E-01	7.04E+01	2.28E-01	2.76E+00	-6.96E-14	4.07E-02	8.91E-02
	Min	-7.24E+00	-7.31E+00	-2.31E+03	-4.20E-01	-3.57E+01	-9.92E-11	-1.34E+01	-6.64E+02
	Max	INF	INF	INF	INF	INF	INF	INF	INF
	Normalized peak height	9.95E-01	9.89E-01	9.87E-01	9.99E-01	9.85E-01	9.53E-01	9.97E-01	9.97E-01
	Peak sensitivity value	9.71E-02	8.42E-01	2.49E+00	2.80E+00	-1.56E+00	-1.28E-13	-2.33E-02	3.83E-01
	Shapiro-Wilk score	2.897E-03	7.860E-03	9.159E-03	8.863E-04	5.999E-03	1.190E-02	1.199E-03	3.351E-03
Extracellular ethanol	Local	1.01E-02	4.37E-03	7.92E-01	2.32E-03	2.81E-02	-3.81E-16	9.18E-03	2.01E-02
	Min	-3.51E+01	-1.66E+01	-3.51E+01	-3.30E+00	-9.86E+00	-3.48E-11	-3.28E+00	-9.83E+00
	Max	INF	INF	INF	INF	INF	INF	INF	INF
	Normalized peak height	5.10E-01	3.26E-01	9.75E-01	2.91E-01	1.57E-01	9.74E-01	8.95E-01	9.47E-01
	Peak sensitivity value	2.69E-03	-5.15E-04	1.05E+00	1.80E-03	2.28E-02	-7.42E-15	1.72E-02	8.08E-02
	Shapiro-Wilk score	5.920E-01	7.185E-01	1.897E-02	4.958E-01	7.121E-01	1.391E-02	6.992E-02	3.048E-02
Extracellular glycerol	Local	2.51E-02	1.08E-02	1.96E+00	8.63E-03	1.04E-01	8.14E-15	-8.18E-02	-1.79E-01
	Min	-2.88E+00	-2.94E+00	-2.41E+01	-1.97E+00	-1.75E+00	-3.87E-11	-8.29E+00	-9.57E+00
	Max	7.97E+01	4.38E+01	4.84E+03	1.08E+01	2.67E+01	5.79E-10	4.55E+01	1.26E+02
	Normalized peak height	3.39E-01	2.91E-01	5.51E-01	3.14E-01	1.53E-01	9.94E-01	4.58E-01	5.32E-01
	Peak sensitivity value	-3.21E-02	-1.38E-02	2.61E+00	2.79E-03	6.00E-02	-9.62E-14	-2.81E-02	8.11E-03
	Shapiro-Wilk score	5.431E-01	6.865E-01	1.873E-02	4.638E-01	7.066E-01	1.857E-03	3.087E-01	1.006E-01
Extracellular glucose	Local	2.57E+00	-1.40E-02	-2.53E+00	-3.55E-03	-4.30E-02	8.97E-17	-5.98E-04	-1.31E-03
	Min	-6.63E-01	-4.71E+00	-3.44E+01	-6.71E+00	-4.32E+01	-3.70E-11	-2.05E+00	-3.86E+00
	Max	4.77E+01	2.21E+00	4.21E+00	3.99E-01	6.25E-01	4.28E-11	2.35E-01	8.30E-01
	Normalized peak height	1.25E-01	2.48E-01	3.50E-01	5.80E-01	5.24E-01	9.89E-01	8.23E-01	5.05E-01
	Peak sensitivity value	3.82E-02	1.77E-03	-1.72E-02	-2.14E-03	-1.04E-02	-1.82E-14	-1.04E-03	1.83E-03
	Shapiro-Wilk score	6.193E-01	6.902E-01	4.870E-01	2.769E-01	2.275E-01	6.601E-03	1.077E-01	1.742E-01
1-3-Bisphosphoglycerate	Local	1.07E-02	4.61E-03	8.37E-01	4.44E-02	5.38E-01	5.30E-16	8.28E-03	1.81E-02
	Min	-3.00E+01	-1.80E+01	-4.04E+02	-8.49E-01	-1.56E+01	-1.08E-07	-7.41E+01	-2.42E+01
	Max	INF	INF	INF	INF	INF	INF	INF	INF
	Normalized peak height	7.89E-01	6.44E-01	7.30E-01	4.03E-01	7.23E-01	9.99E-01	9.38E-01	8.75E-01
	Peak sensitivity value	1.25E-01	-2.54E-01	-3.79E-02	3.29E-02	-1.84E-02	-1.01E-11	5.87E-03	1.97E-02
	Shapiro-Wilk score	1.012E-01	7.438E-02	7.470E-02	2.561E-01	9.143E-02	1.498E-03	5.673E-02	6.683E-02
Fructose 1-6-bisphosphate	Local	4.60E-02	1.98E-02	3.59E+00	2.43E-02	2.94E-01	-9.76E-02	-8.09E-02	-1.00E+00
	Min	-2.50E+01	-2.68E+01	-2.44E+01	-1.23E+00	-2.88E+00	-2.32E+02	-4.26E+03	-8.07E+02
	Max	INF	INF	INF	INF	INF	INF	INF	INF
	Normalized peak height	7.82E-01	6.19E-01	7.74E-01	6.69E-01	6.86E-01	9.26E-01	9.76E-01	5.78E-01
	Peak sensitivity value	1.26E-01	-6.49E-01	7.36E-01	3.06E-02	3.56E-01	-1.16E-01	-1.40E+00	-6.34E-01
	Shapiro-Wilk score	6.005E-02	5.461E-02	6.455E-02	1.469E-01	1.295E-01	4.344E-02	6.022E-03	1.091E-01
AMP	Local	4.17E-03	1.79E-03	3.25E-01	-6.74E-02	-8.16E-01	2.40E-15	-1.26E-02	-2.76E-02
	Min	-5.46E+00	-4.01E+00	-6.79E+01	-3.03E+00	-2.93E+01	-5.73E-10	-6.06E+00	-8.20E+00
	Max	5.34E+01	1.21E+01	2.00E+01	7.37E-01	3.16E+00	8.13E-11	2.35E-02	6.01E-01
	Normalized peak height	6.35E-01	3.90E-01	3.84E-01	1.19E-01	8.26E-02	9.94E-01	4.16E-01	4.25E-01
	Peak sensitivity value	-1.59E-02	-2.78E-03	1.16E-02	9.22E-04	-2.71E-01	-9.38E-14	2.23E-03	-1.98E-03
	Shapiro-Wilk score	3.424E-01	5.857E-01	6.497E-01	7.170E-01	4.599E-01	2.904E-03	2.404E-01	3.707E-01
ATP	Local	-1.59E-03	-6.83E-04	-1.24E-01	2.57E-02	3.11E-01	-8.56E-16	4.80E-03	1.05E-02
	Min	-9.58E+01	-1.17E+02	-4.62E+01	-1.50E+01	-1.31E+02	-1.38E-11	-1.87E-02	-9.89E-01
	Max	3.64E+00	4.71E+00	8.76E+09	1.25E+00	3.51E+00	2.86E-11	2.69E+01	1.15E+01
	Normalized peak height	8.73E-01	8.70E-01	1.00E+00	7.94E-01	7.61E-01	8.87E-01	3.34E-01	6.23E-02
	Peak sensitivity value	9.28E-03	2.00E-02	4.38E+06	-4.42E-03	-5.65E-02	-1.95E-14	-5.28E-03	8.18E-03
	Shapiro-Wilk score	7.011E-02	8.236E-02	4.709E-05	1.281E-01	9.707E-02	3.710E-02	5.086E-01	7.979E-01
NADH	Local	9.35E-03	4.03E-03	7.30E-01	-1.86E-03	-2.25E-02	6.21E-15	1.54E-01	3.36E-01
	Min	-5.14E+01	-2.13E+01	-1.72E+01	-3.21E+00	-1.70E+01	-7.23E-10	-2.41E-01	-7.26E+00
	Max	INF	INF	INF	INF	INF	INF	INF	INF
	Normalized peak height	5.90E-01	3.49E-01	8.81E-01	1.51E-01	2.10E-01	9.94E-01	8.89E-01	9.83E-01
	Peak sensitivity value	-1.80E-02	-8.80E-03	-1.65E+00	-2.73E-04	1.32E-01	-4.93E-13	-1.13E-03	1.19E-01
	Shapiro-Wilk score	2.646E-01	6.249E-01	2.034E-02	5.838E-01	6.028E-01	2.258E-03	4.491E-02	1.319E-02

Fructose-6-Phosphate	Local	1.11E-03	3.34E-03	-2.13E-15	1.18E-01	0.00E+00	0.00E+00	-2.01E-02	-1.59E-15
	Min	-3.00E-01	-7.98E+00	-3.18E-11	-2.09E+01	-2.72E-11	-4.53E-10	-5.90E+01	-1.44E-10
	Max	INF	INF	INF	INF	INF	INF	INF	INF
	Normalized peak height	9.86E-01	9.44E-01	9.94E-01	6.01E-01	9.64E-01	9.84E-01	6.10E-01	9.53E-01
	Peak sensitivity value	1.52E-03	4.09E-03	-1.64E-14	2.32E-01	-2.45E-16	2.28E-13	2.38E-02	-2.12E-14
	Shapiro-Wilk score	9.115E-03	4.585E-02	2.338E-03	9.462E-02	3.141E-02	1.899E-03	7.701E-02	4.753E-02
Extracellular cyanide	Local	1.15E-03	3.48E-03	6.32E-16	1.23E-01	0.00E+00	0.00E+00	-2.09E-02	3.66E-16
	Min	-9.65E-02	-1.64E+00	-5.57E-12	-3.56E+00	-1.10E-11	-1.32E-11	-2.43E+00	-3.07E-11
	Max	1.14E-01	1.78E+01	8.17E-11	8.77E+00	2.49E-11	6.97E-12	3.56E+00	8.53E-11
	Normalized peak height	9.04E-01	9.71E-01	9.97E-01	2.78E-01	9.91E-01	9.87E-01	4.82E-01	9.75E-01
	Peak sensitivity value	5.68E-05	7.59E-03	-2.76E-14	-2.89E-04	5.90E-15	4.81E-16	1.77E-03	-1.82E-14
	Shapiro-Wilk score	1.042E-01	2.357E-02	5.054E-04	4.727E-01	6.696E-03	9.657E-03	3.122E-01	1.816E-02
Pyruvate	Local	7.64E-03	2.31E-02	-7.79E+01	8.15E-01	0.00E+00	0.00E+00	-1.39E-01	-5.18E-14
	Min	-8.53E-01	-9.49E+00	-1.64E+04	-1.14E+03	-2.95E-10	-5.73E-10	-1.26E+03	-1.44E-09
	Max	INF	INF	INF	INF	INF	INF	INF	INF
	Normalized peak height	9.83E-01	9.95E-01	9.89E-01	9.89E-01	9.99E-01	9.94E-01	9.96E-01	9.87E-01
	Peak sensitivity value	-4.53E-03	1.44E-02	-9.20E+00	-9.47E-01	-5.11E-13	-1.00E-13	-3.01E-01	-3.37E-13
	Shapiro-Wilk score	4.959E-03	1.831E-03	8.128E-03	5.648E-03	2.596E-04	2.592E-03	4.312E-03	9.134E-03
Extracellular ethanol	Local	1.72E-03	5.20E-03	-1.74E-16	1.84E-01	-3.41E-16	-1.00E+00	-3.13E-02	-1.73E-16
	Min	-1.45E-02	-6.27E-02	-1.83E-11	-1.23E+01	-3.66E-11	-1.00E+00	-2.27E+02	-6.01E-10
	Max	INF	INF	INF	INF	INF	INF	INF	INF
	Normalized peak height	9.59E-01	9.65E-01	9.92E-01	7.21E-01	9.80E-01	1.00E+00	7.53E-01	9.87E-01
	Peak sensitivity value	-1.31E-03	2.04E-02	4.99E-15	1.15E+00	-6.48E-15	-1.00E+00	5.25E-02	7.31E-14
	Shapiro-Wilk score	1.210E-02	4.476E-02	5.948E-03	1.612E-02	1.533E-02	1.000E+00	4.068E-02	1.022E-02
Extracellular glycerol	Local	-1.53E-02	-4.63E-02	1.49E-14	-1.64E+00	0.00E+00	0.00E+00	2.79E-01	9.72E-15
	Min	-3.24E-01	-9.81E+01	-1.45E-09	-1.07E+01	-1.31E-10	-5.94E-10	-1.36E+02	-4.32E-10
	Max	9.62E+00	4.17E+00	1.00E-10	2.23E+03	7.90E-11	1.25E-10	1.02E+01	1.13E-09
	Normalized peak height	9.42E-01	8.51E-01	9.97E-01	9.62E-01	9.58E-01	9.77E-01	5.47E-01	9.64E-01
	Peak sensitivity value	-2.87E-04	2.58E-02	4.78E-13	-5.81E-01	-6.16E-14	-2.46E-13	3.37E-02	5.53E-13
	Shapiro-Wilk score	2.359E-02	4.601E-02	3.819E-04	3.085E-02	3.017E-02	1.139E-02	1.461E-01	1.523E-02
Extracellular glucose	Local	-1.12E-04	-3.38E-04	2.12E-16	-1.20E-02	0.00E+00	0.00E+00	2.04E-03	1.65E-16
	Min	-3.99E-01	-7.83E+00	-1.04E-11	-9.78E+00	-6.04E-12	-9.14E-12	-4.21E-01	-1.38E-10
	Max	4.52E-03	5.70E+00	1.27E-10	1.14E+00	3.93E-12	1.10E-11	5.34E+00	6.46E-11
	Normalized peak height	9.60E-01	9.76E-01	9.97E-01	4.29E-01	9.26E-01	9.68E-01	4.45E-01	9.70E-01
	Peak sensitivity value	-1.22E-04	-4.80E-03	4.76E-14	-3.84E-03	4.69E-15	-8.44E-15	3.04E-03	-6.35E-14
	Shapiro-Wilk score	2.923E-02	2.094E-02	4.103E-04	3.741E-01	2.346E-02	1.352E-02	1.777E-01	2.143E-02
1-3-Bisphosphoglycerate	Local	-2.84E-01	-8.56E-01	3.50E-14	1.66E-01	0.00E+00	0.00E+00	-2.82E-02	6.06E-16
	Min	-2.89E+00	-2.48E+03	-2.96E-08	-5.98E+01	-3.91E-11	-6.60E-11	-1.06E+02	-1.81E-09
	Max	INF	INF	INF	INF	INF	INF	INF	INF
	Normalized peak height	2.48E-02	9.04E-01	1.00E+00	6.87E-01	9.80E-01	9.95E-01	8.41E-01	9.81E-01
	Peak sensitivity value	-2.16E-03	-1.41E+00	4.42E-12	6.24E-02	-2.59E-14	-5.99E-13	-1.03E-01	-2.98E-13
	Shapiro-Wilk score	8.548E-01	4.267E-02	2.076E-04	6.545E-02	1.129E-02	7.102E-04	8.263E-02	1.271E-02
Fructose 1-6-bisphosphate	Local	-8.59E-02	-2.59E-01	2.20E-14	-2.18E+00	0.00E+00	0.00E+00	-9.76E-01	1.28E-14
	Min	-2.14E+00	-1.80E+03	-5.34E-10	-1.30E+03	-1.27E-10	-3.45E-10	-6.39E+02	-6.98E-10
	Max	INF	INF	INF	INF	INF	INF	INF	INF
	Normalized peak height	7.33E-01	6.45E-01	9.50E-01	4.65E-01	9.51E-01	9.81E-01	3.29E-01	9.74E-01
	Peak sensitivity value	-5.87E-04	8.82E-01	-4.55E-13	1.40E-01	9.45E-15	2.11E-13	-1.80E+00	2.61E-13
	Shapiro-Wilk score	1.602E-01	2.131E-02	2.780E-03	1.354E-01	4.572E-02	1.733E-02	1.482E-01	1.834E-02
AMP	Local	-2.37E-03	-7.14E-03	6.43E-15	-2.52E-01	0.00E+00	0.00E+00	4.30E-02	3.70E-15
	Min	-3.78E-01	-5.99E+01	-3.93E-11	-9.45E+00	-1.15E-11	-1.43E-11	-2.20E+00	-2.83E-10
	Max	1.28E-03	4.03E-03	3.12E-10	3.37E+00	7.34E-12	2.20E-11	6.32E+00	3.40E-10
	Normalized peak height	7.92E-01	9.70E-01	9.94E-01	6.46E-02	9.13E-01	9.38E-01	4.37E-01	9.61E-01
	Peak sensitivity value	-4.65E-05	-2.59E-02	-1.14E-13	-1.08E-02	-4.59E-15	1.00E-14	3.90E-03	5.72E-14
	Shapiro-Wilk score	1.487E-01	4.431E-02	1.096E-03	7.592E-01	8.303E-02	4.785E-02	5.019E-01	3.819E-02
ATP	Local	9.01E-04	2.72E-03	-2.17E-15	9.61E-02	0.00E+00	0.00E+00	-1.64E-02	-1.37E-15
	Min	-1.68E-02	-1.03E-02	-4.79E-11	-1.98E+01	-3.62E-11	-3.89E-10	-1.01E+01	-1.55E-11
	Max	1.30E+00	4.18E+02	5.40E-11	1.00E+01	4.70E-11	9.20E-11	2.32E+00	2.04E-11
	Normalized peak height	3.64E-01	9.80E-01	9.77E-01	1.42E-01	9.06E-01	9.86E-01	7.34E-02	8.50E-01
	Peak sensitivity value	-3.08E-04	1.99E-01	4.13E-14	-9.42E-03	3.87E-14	-6.35E-14	-1.11E-02	-1.01E-14
	Shapiro-Wilk score	2.413E-01	3.344E-02	8.139E-03	8.839E-01	4.644E-02	8.364E-03	8.992E-01	1.250E-01
NADH	Local	2.88E-02	8.69E-02	1.30E-14	-1.28E+00	0.00E+00	0.00E+00	-5.23E-01	6.20E-15
	Min	-7.20E-01	-6.55E-02	-1.93E-11	-1.30E+01	-3.17E-10	-8.96E-11	-4.21E+02	-8.85E-10
	Max	INF	INF	INF	INF	INF	INF	INF	INF
	Normalized peak height	9.87E-01	8.34E-01	9.84E-01	9.91E-01	9.87E-01	9.96E-01	9.62E-01	9.77E-01
	Peak sensitivity value	3.49E-03	-1.36E-02	-8.60E-15	2.05E-01	-8.06E-14	3.37E-13	-5.50E-02	9.80E-14
	Shapiro-Wilk score	9.424E-03	3.605E-02	1.207E-02	7.650E-03	4.736E-03	9.538E-04	1.910E-02	2.052E-02

Fructose-6-Phosphate	Local	-7.98E-17	-4.83E-03	-2.51E-02	-7.13E-03	-5.69E-04	-7.58E-01	-5.75E-02	-2.80E-17
	Min	-2.25E-10	-1.64E+02	-1.94E+02	-6.26E+01	-7.87E-01	-8.13E+00	-6.82E+02	-1.81E-08
	Max	INF	INF	INF	INF	INF	INF	INF	INF
	Normalized peak height	9.86E-01	8.00E-01	7.12E-01	9.01E-01	6.56E-01	2.03E-02	8.08E-01	1.00E+00
	Peak sensitivity value	-8.11E-14	1.13E-02	4.42E-02	-2.78E-02	-1.52E-04	-9.94E-01	-1.03E-01	4.15E-11
	Shapiro-Wilk score	7.781E-03	5.221E-02	4.189E-02	3.535E-02	3.010E-01	9.609E-01	4.239E-02	6.211E-05
Extracellular cyanide	Local	-5.96E-16	-5.03E-03	3.14E-02	-6.50E-02	7.46E-02	5.46E-03	-9.35E-03	-7.74E-19
	Min	-9.31E-12	-5.35E-01	-7.01E-01	-9.98E-01	0.00E+00	-6.74E-01	-3.15E+00	-1.37E-10
	Max	8.51E-12	1.52E+00	2.04E+00	4.66E-01	2.80E+00	2.38E+00	3.65E+00	3.29E-12
	Normalized peak height	9.77E-01	3.21E-01	1.52E-01	6.64E-02	1.23E-01	3.95E-01	4.98E-01	1.00E+00
	Peak sensitivity value	4.84E-16	-1.06E-03	2.56E-03	-1.56E-03	1.40E-03	-2.28E-04	2.41E-03	1.77E-15
	Shapiro-Wilk score	1.906E-02	5.049E-01	4.947E-01	5.535E-01	5.632E-01	3.578E-01	3.391E-01	1.041E-04
Pyruvate	Local	1.49E-13	-3.34E-02	-1.73E-01	-4.93E-02	-3.93E-03	-1.87E+00	4.59E+00	2.26E-16
	Min	-2.60E-10	-9.50E+02	-4.81E+03	-2.84E+02	-2.02E+01	-6.06E+03	-4.28E+03	-1.16E-09
	Max	INF	INF	INF	INF	INF	INF	INF	INF
	Normalized peak height	9.92E-01	9.50E-01	9.94E-01	9.96E-01	9.44E-01	6.52E-01	9.85E-01	1.00E+00
	Peak sensitivity value	-4.51E-13	2.60E-01	5.50E-01	-3.00E-01	4.94E-03	3.09E+00	4.48E-01	-5.04E-13
	Shapiro-Wilk score	2.006E-03	9.522E-03	1.763E-03	4.695E-03	1.972E-02	3.630E-03	1.256E-02	3.480E-04
Extracellular ethanol	Local	1.29E-15	-7.52E-03	-3.91E-02	-1.11E-02	-8.85E-04	-1.89E-02	5.21E-02	2.13E-18
	Min	-7.55E-10	-1.71E+03	-8.21E+02	-2.57E+02	-1.10E+00	-1.23E+01	-3.11E+03	-4.96E-09
	Max	INF	INF	INF	INF	INF	INF	INF	INF
	Normalized peak height	9.97E-01	9.95E-01	9.84E-01	9.77E-01	6.62E-01	1.29E-01	9.50E-01	1.00E+00
	Peak sensitivity value	-1.03E-13	-4.02E-01	-3.34E-01	-8.30E-02	5.96E-04	5.78E-03	-1.37E+00	-1.72E-12
	Shapiro-Wilk score	1.126E-03	5.042E-03	1.339E-02	1.376E-02	2.764E-01	5.576E-01	1.007E-02	1.017E-04
Extracellular glycerol	Local	-1.00E+00	6.70E-02	3.48E-01	9.89E-02	7.89E-03	-7.27E-02	1.24E-01	1.03E-17
	Min	-1.00E+00	-9.82E+02	-1.04E+03	-2.11E+02	-1.34E+00	-1.06E+01	-4.84E+03	-4.79E-09
	Max	-1.00E+00	4.52E+00	3.03E+00	6.24E+00	6.17E-01	1.81E+01	2.12E+01	3.55E-09
	Normalized peak height	1.00E+00	9.45E-01	9.68E-01	8.45E-01	5.26E-01	1.14E-01	9.88E-01	1.00E+00
	Peak sensitivity value	-1.00E+00	8.68E-02	4.22E-01	5.96E-02	6.96E-04	-7.56E-02	-6.32E-01	3.31E-12
	Shapiro-Wilk score	1.000E+00	2.852E-02	2.219E-02	6.605E-02	3.744E-01	6.463E-01	8.923E-03	2.335E-04
Extracellular glucose	Local	1.51E-17	4.89E-04	2.54E-03	7.23E-04	5.76E-05	2.55E-02	5.83E-03	-3.53E-18
	Min	-2.54E-11	-1.98E-01	-1.73E-01	-1.08E-01	-3.27E-02	-1.43E+00	-1.58E+00	-4.40E-10
	Max	1.51E-11	3.91E+00	4.96E+00	2.03E+00	6.55E-01	7.48E+00	9.77E+00	1.06E-10
	Normalized peak height	9.69E-01	5.57E-01	4.44E-01	5.49E-01	7.96E-01	2.41E-01	4.75E-01	1.00E+00
	Peak sensitivity value	1.57E-14	1.69E-03	-1.06E-03	-3.32E-04	-6.88E-05	4.50E-03	4.60E-03	2.13E-13
	Shapiro-Wilk score	1.757E-02	2.884E-01	2.857E-01	2.062E-01	1.293E-01	5.085E-01	3.527E-01	1.321E-04
1-3-Bisphosphoglycerate	Local	-4.86E-16	-6.78E-03	-3.52E-02	-1.00E-02	-7.98E-04	-3.98E-01	-7.28E-03	7.56E-17
	Min	-1.03E-10	-1.90E+02	-3.78E+02	-1.20E+02	-3.13E+00	-9.48E+01	-1.78E+03	-1.01E-08
	Max	INF	INF	INF	INF	INF	INF	INF	INF
	Normalized peak height	9.89E-01	7.62E-01	7.73E-01	5.56E-01	7.39E-01	3.08E-01	9.54E-01	9.99E-01
	Peak sensitivity value	9.32E-13	1.33E-02	-2.15E-01	-7.16E-02	-3.64E-04	-3.44E-01	-1.02E+00	-4.98E-12
	Shapiro-Wilk score	7.834E-04	4.436E-02	2.933E-02	3.556E-02	1.741E-01	3.756E-01	2.845E-02	1.804E-04
Fructose 1-6-bisphosphate	Local	-8.40E-15	8.90E-02	4.63E-01	1.31E-01	1.05E-02	-2.10E-01	2.16E-01	3.37E-17
	Min	-3.67E-10	-7.07E+02	-5.95E+02	-2.37E+02	-1.19E+01	-8.28E+02	-3.84E+03	-1.88E-09
	Max	INF	INF	INF	INF	INF	INF	INF	INF
	Normalized peak height	9.82E-01	7.80E-01	5.97E-01	6.54E-01	9.03E-01	7.56E-01	9.34E-01	9.99E-01
	Peak sensitivity value	1.06E-13	1.99E-01	-3.61E-02	-1.32E-01	-9.61E-04	-1.46E-01	-1.53E+00	-2.15E-12
	Shapiro-Wilk score	1.481E-02	1.152E-01	1.061E-01	8.392E-02	5.240E-02	1.966E-01	2.531E-02	3.244E-04
AMP	Local	2.03E-16	1.03E-02	5.37E-02	1.52E-02	1.22E-03	6.08E-01	1.23E-01	6.38E-17
	Min	-3.44E-11	-7.90E-01	-2.01E+00	-5.84E-01	-8.47E-02	-8.68E-01	-3.20E+00	-5.39E-04
	Max	8.98E-11	5.34E+00	4.37E+00	2.69E+00	4.29E-01	4.70E+00	6.80E+01	2.66E-10
	Normalized peak height	8.87E-01	1.54E-01	1.36E-01	2.22E-01	3.00E-01	2.70E-02	2.65E-01	1.00E+00
	Peak sensitivity value	5.94E-14	3.90E-03	5.50E-03	4.90E-04	-2.05E-04	3.90E-03	3.86E-02	-2.69E-07
	Shapiro-Wilk score	2.478E-02	6.742E-01	6.577E-01	5.232E-01	3.326E-01	9.628E-01	6.185E-01	4.709E-05
ATP	Local	-6.97E-17	-3.93E-03	-2.04E-02	-5.81E-03	-4.63E-04	-2.32E-01	-4.68E-02	2.55E-17
	Min	-2.28E-11	-3.52E+00	-7.89E+00	-3.36E+00	-1.29E-01	-3.25E+01	-8.87E+09	-2.79E-08
	Max	2.22E-11	6.02E+00	4.94E+00	2.86E+00	9.59E-01	2.74E+01	9.50E+00	1.17E+08
	Normalized peak height	9.60E-01	1.69E-01	2.09E-01	2.46E-01	5.14E-01	7.78E-01	1.00E+00	1.00E+00
	Peak sensitivity value	-1.45E-14	6.86E-03	7.89E-03	4.19E-03	-1.80E-04	-2.38E-03	-4.44E+06	5.83E+04
	Shapiro-Wilk score	3.091E-02	7.453E-01	7.579E-01	5.749E-01	2.617E-01	2.014E-01	4.709E-05	4.709E-05
NADH	Local	-5.37E-15	5.25E-02	2.73E-01	7.75E-02	6.18E-03	1.86E-02	5.39E-02	-5.02E-18
	Min	-2.11E-10	-1.59E+03	-1.23E+03	-9.45E+02	-1.66E+00	-1.28E+01	-4.42E+03	-7.25E-08
	Max	INF	INF	INF	INF	INF	INF	INF	INF
	Normalized peak height	9.58E-01	9.92E-01	9.94E-01	9.97E-01	6.73E-01	4.61E-02	9.87E-01	1.00E+00
	Peak sensitivity value	-1.18E-13	-1.98E-02	-6.18E-01	-3.38E-01	-3.38E-04	-2.28E-01	-1.03E+00	-5.08E-11
	Shapiro-Wilk score	1.297E-02	4.522E-03	9.377E-03	3.283E-03	2.731E-01	8.229E-01	1.269E-02	9.692E-05

Table S25: Flux control coefficients for *Saccharomyces cerevisiae* model with parameter variation of ±5%

		(GlucoseMixedflowtoextracellularmedium)	(Glucoseuptake)	(Hexokinase)	(Phosphoglucoisomerase)	(Phosphofructokinase)	(Aldolase)	(Triosephosphateisomerase)	(Glyceraldehyde3-phosphatedehydrogenase)	(Phosphoenolpyruvatesynthesis)	(Pyruvatekinase)	(Pyruvatedecarboxylase)	(Alcoholdehydrogenase)
(Glucose Mixed flow to extracellular medium)	Local	1.25E-02	5.36E-03	9.73E-01	1.37E-03	1.65E-02	-3.45E-17	2.30E-04	5.02E-04	4.31E-05	1.30E-04	-8.14E-17	4.60E-03
	Min	5.77E-03	2.69E-03	9.41E-01	1.04E-03	1.19E-02	-1.55E-16	1.52E-04	3.32E-04	2.72E-05	7.89E-05	-2.44E-16	2.96E-03
	Max	3.17E-02	1.14E-02	9.87E-01	1.85E-03	2.49E-02	1.63E-16	3.48E-04	7.91E-04	6.91E-05	2.10E-04	2.10E-16	7.38E-03
	Normalized peak height	3.97E-03	4.03E-03	4.11E-03	3.50E-03	3.27E-03	6.10E-02	3.65E-03	3.88E-03	4.08E-03	4.11E-03	2.31E-02	3.57E-03
	Peak sensitivity value	9.34E-03	4.77E-03	9.77E-01	1.32E-03	1.62E-02	-1.28E-19	2.18E-04	4.79E-04	4.20E-05	1.24E-04	-6.43E-20	4.43E-03
	Shapiro-Wilk score	9.211E-01	9.613E-01	9.406E-01	9.907E-01	9.800E-01	9.992E-01	9.894E-01	9.901E-01	9.890E-01	9.883E-01	9.994E-01	9.826E-01
(Glucose uptake)	Local	1.25E-02	5.36E-03	9.73E-01	1.37E-03	1.65E-02	-2.20E-17	2.30E-04	5.02E-04	4.31E-05	1.30E-04	-8.36E-17	4.60E-03
	Min	5.61E-03	2.63E-03	9.36E-01	1.02E-03	1.19E-02	-2.48E-16	1.48E-04	3.29E-04	2.79E-05	8.24E-05	-2.66E-16	3.03E-03
	Max	3.34E-02	1.23E-02	9.87E-01	1.92E-03	2.50E-02	2.41E-16	3.56E-04	7.96E-04	6.93E-05	2.10E-04	2.72E-16	7.43E-03
	Normalized peak height	4.12E-03	4.67E-03	4.54E-03	3.80E-03	3.43E-03	1.91E-02	3.70E-03	3.89E-03	3.99E-03	3.85E-03	7.75E-03	3.52E-03
	Peak sensitivity value	9.73E-03	4.63E-03	9.77E-01	1.31E-03	1.55E-02	-1.21E-19	2.25E-04	4.76E-04	4.18E-05	1.23E-04	-2.66E-19	4.32E-03
	Shapiro-Wilk score	9.194E-01	9.604E-01	9.392E-01	9.904E-01	9.791E-01	9.999E-01	9.885E-01	9.895E-01	9.881E-01	9.882E-01	9.998E-01	9.813E-01
(Hexokinase)	Local	1.25E-02	5.36E-03	9.73E-01	1.37E-03	1.65E-02	-4.14E-17	2.30E-04	5.02E-04	4.31E-05	1.30E-04	-1.06E-16	4.60E-03
	Min	5.75E-03	2.74E-03	9.38E-01	1.03E-03	1.18E-02	-1.69E-16	1.50E-04	3.10E-04	2.78E-05	8.23E-05	-2.32E-16	2.99E-03
	Max	3.27E-02	1.15E-02	9.87E-01	1.84E-03	2.53E-02	1.51E-16	3.40E-04	7.80E-04	6.57E-05	2.00E-04	2.45E-16	7.31E-03
	Normalized peak height	4.18E-03	4.00E-03	4.22E-03	3.65E-03	3.59E-03	2.42E-02	3.72E-03	4.02E-03	3.54E-03	3.55E-03	4.02E-03	3.34E-03
	Peak sensitivity value	1.05E-02	4.68E-03	9.76E-01	1.30E-03	1.58E-02	5.34E-20	2.21E-04	4.88E-04	4.26E-05	1.20E-04	4.73E-19	4.42E-03
	Shapiro-Wilk score	9.195E-01	9.606E-01	9.392E-01	9.903E-01	9.793E-01	9.997E-01	9.888E-01	9.893E-01	9.878E-01	9.887E-01	9.997E-01	9.809E-01
(Phosphoglucoisomerase)	Local	1.27E-02	5.48E-03	9.94E-01	3.42E-03	4.14E-02	4.62E-16	-6.63E-03	-1.45E-02	-1.24E-03	-3.75E-03	2.63E-15	-1.33E-01
	Min	5.72E-03	2.75E-03	9.58E-01	2.81E-03	3.23E-02	-5.74E-15	-8.32E-03	-1.82E-02	-1.59E-03	-5.27E-03	-8.89E-15	-1.65E-01
	Max	3.39E-02	1.21E-02	1.02E+00	4.28E-03	5.69E-02	5.71E-15	-5.09E-03	-1.12E-02	-9.57E-04	-2.61E-03	7.49E-15	-1.05E-01
	Normalized peak height	3.98E-03	4.15E-03	3.45E-03	3.68E-03	3.35E-03	3.08E-02	3.16E-03	3.53E-03	3.30E-03	4.00E-03	1.56E-02	2.74E-03
	Peak sensitivity value	9.71E-03	4.99E-03	9.90E-01	3.36E-03	4.01E-02	5.03E-18	-6.59E-03	-1.45E-02	-1.23E-03	-3.62E-03	-2.68E-18	-1.30E-01
	Shapiro-Wilk score	9.185E-01	9.591E-01	9.978E-01	9.950E-01	9.810E-01	9.984E-01	9.973E-01	9.989E-01	9.977E-01	9.966E-01	9.996E-01	9.897E-01
(Phosphofructokinase)	Local	1.27E-02	5.48E-03	9.94E-01	3.42E-03	4.14E-02	1.39E-15	-6.63E-03	-1.45E-02	-1.24E-03	-3.75E-03	3.85E-15	-1.33E-01
	Min	5.83E-03	2.78E-03	9.56E-01	2.82E-03	3.24E-02	-5.08E-15	-8.40E-03	-1.81E-02	-1.63E-03	-5.27E-03	-1.00E-14	-1.66E-01
	Max	3.51E-02	1.25E-02	1.02E+00	4.32E-03	5.77E-02	4.69E-15	-5.10E-03	-1.11E-02	-9.15E-04	-2.53E-03	9.70E-15	-1.05E-01
	Normalized peak height	4.11E-03	4.36E-03	3.75E-03	3.70E-03	3.55E-03	1.01E-02	3.35E-03	3.63E-03	3.76E-03	3.73E-03	4.66E-03	2.65E-03
	Peak sensitivity value	9.70E-03	4.84E-03	9.93E-01	3.41E-03	4.07E-02	-2.63E-18	-6.68E-03	-1.45E-02	-1.22E-03	-3.68E-03	-8.75E-17	-1.29E-01
	Shapiro-Wilk score	9.188E-01	9.603E-01	9.981E-01	9.947E-01	9.815E-01	1.000E+00	9.976E-01	9.991E-01	9.982E-01	9.966E-01	9.998E-01	9.894E-01
(Aldolase)	Local	1.27E-02	5.48E-03	9.94E-01	3.42E-03	4.14E-02	1.11E-15	-6.63E-03	-1.45E-02	-1.24E-03	-3.75E-03	-1.02E-14	-1.33E-01
	Min	5.50E-03	2.81E-03	9.61E-01	2.81E-03	3.17E-02	-5.77E-15	-8.25E-03	-1.78E-02	-1.61E-03	-5.18E-03	-4.12E-14	-1.66E-01
	Max	3.25E-02	1.15E-02	1.02E+00	4.22E-03	5.58E-02	5.55E-15	-5.01E-03	-1.12E-02	-9.23E-04	-2.56E-03	3.91E-14	-1.04E-01
	Normalized peak height	4.15E-03	3.98E-03	3.39E-03	3.51E-03	3.33E-03	5.11E-02	3.26E-03	3.61E-03	3.56E-03	3.71E-03	1.73E-02	2.69E-03
	Peak sensitivity value	1.12E-02	4.73E-03	9.90E-01	3.41E-03	4.06E-02	-4.45E-16	-6.40E-03	-1.41E-02	-1.22E-03	-3.55E-03	2.29E-17	-1.26E-01
	Shapiro-Wilk score	9.195E-01	9.600E-01	9.976E-01	9.955E-01	9.824E-01	9.989E-01	9.976E-01	9.990E-01	9.979E-01	9.964E-01	9.998E-01	9.893E-01
(Triosephosphate isomerase)	Local	1.01E-02	4.37E-03	7.92E-01	2.32E-03	2.81E-02	-1.03E-15	9.18E-03	2.01E-02	1.72E-03	5.20E-03	-5.10E-15	1.84E-01
	Min	4.64E-03	2.18E-03	7.30E-01	1.90E-03	2.16E-02	-2.01E-15	6.86E-03	1.50E-02	1.25E-03	3.41E-03	-1.29E-14	1.40E-01
	Max	2.68E-02	9.81E-03	8.33E-01	2.82E-03	3.76E-02	2.44E-15	1.19E-02	2.56E-02	2.34E-03	7.37E-03	1.27E-14	2.41E-01
	Normalized peak height	4.11E-03	4.53E-03	3.22E-03	3.31E-03	3.13E-03	4.58E-03	3.36E-03	3.58E-03	3.71E-03	3.73E-03	1.64E-02	2.62E-03
	Peak sensitivity value	7.54E-03	4.11E-03	7.98E-01	2.32E-03	2.70E-02	1.03E-17	8.95E-03	1.96E-02	1.71E-03	4.94E-03	-4.83E-18	1.79E-01
	Shapiro-Wilk score	9.230E-01	9.643E-01	9.844E-01	9.979E-01	9.887E-01	9.997E-01	9.968E-01	9.979E-01	9.964E-01	9.953E-01	9.999E-01	9.880E-01
(Glyceraldehyde 3-phosphate dehydrogenase)	Local	1.16E-02	4.98E-03	9.03E-01	2.92E-03	3.54E-02	-1.85E-16	5.23E-04	1.14E-03	9.81E-05	2.96E-04	-9.79E-15	1.05E-02
	Min	5.09E-03	2.51E-03	8.70E-01	2.44E-03	2.76E-02	-3.48E-15	3.73E-04	7.88E-04	6.68E-05	1.94E-04	-2.47E-14	7.38E-03
	Max	3.09E-02	1.07E-02	9.18E-01	3.55E-03	4.79E-02	3.02E-15	7.47E-04	1.67E-03	1.40E-04	4.52E-04	3.00E-14	1.53E-02
	Normalized peak height	4.53E-03	4.08E-03	4.18E-03	3.58E-03	3.21E-03	4.01E-03	3.51E-03	4.05E-03	3.58E-03	3.81E-03	4.75E-03	3.16E-03
	Peak sensitivity value	9.16E-03	4.46E-03	9.06E-01	2.90E-03	3.51E-02	-6.07E-17	5.04E-04	1.16E-03	9.59E-05	2.83E-04	-1.02E-15	1.00E-02
	Shapiro-Wilk score	9.179E-01	9.603E-01	9.585E-01	9.967E-01	9.839E-01	1.000E+00	9.936E-01	9.943E-01	9.935E-01	9.933E-01	9.997E-01	9.870E-01
(Phosphoenolpyruvate synthesis)	Local	1.16E-02	4.98E-03	9.03E-01	2.92E-03	3.54E-02	-2.35E-16	5.23E-04	1.14E-03	9.81E-05	2.96E-04	-4.52E-14	1.05E-02
	Min	5.33E-03	2.56E-03	8.68E-01	2.43E-03	2.79E-02	-2.28E-14	3.66E-04	7.70E-04	6.91E-05	1.93E-04	-1.59E-13	7.31E-03
	Max	3.09E-02	1.06E-02	9.17E-01	3.56E-03	4.78E-02	2.46E-14	7.50E-04	1.65E-03	1.41E-04	4.39E-04	1.69E-13	1.55E-02
	Normalized peak height	4.18E-03	3.86E-03	4.53E-03	3.38E-03	3.06E-03	3.89E-03	3.63E-03	4.08E-03	3.69E-03	3.73E-03	4.46E-03	3.12E-03
	Peak sensitivity value	9.10E-03	4.77E-03	9.07E-01	2.91E-03	3.49E-02	2.22E-15	5.03E-04	1.12E-03	9.38E-05	2.87E-04	-4.72E-15	9.96E-03
	Shapiro-Wilk score	9.210E-01	9.624E-01	9.600E-01	9.967E-01	9.838E-01	1.000E+00	9.931E-01	9.938E-01	9.933E-01	9.933E-01	9.995E-01	9.864E-01
(Pyruvate kinase)	Local	1.16E-02	4.98E-03	9.03E-01	2.92E-03	3.54E-02	2.24E-15	5.23E-04	1.14E-03	9.81E-05	2.96E-04	5.94E-14	1.05E-02
	Min	5.23E-03	2.55E-03	8.71E-01	2.43E-03	2.80E-02	-1.32E-14	3.74E-04	8.13E-04	6.88E-05	1.97E-04	-2.01E-13	7.16E-03
	Max	2.98E-02	1.08E-02	9.18E-01	3.54E-03	4.78E-02	1.61E-14	7.49E-04	1.71E-03	1.44E-04	4.37E-04	1.57E-13	1.55E-02
	Normalized peak height	3.82E-03	3.92E-03	4.31E-03	3.54E-03	3.01E-03	7.05E-03	3.76E-03	4.14E-03	3.76E-03	3.72E-03	1.38E-01	3.61E-03
	Peak sensitivity value	9.91E-03	4.05E-03	9.04E-01	2.92E-03	3.39E-02	-3.40E-17	5.03E-04	1.15E-03	9.49E-05	2.88E-04	-5.78E-17	1.02E-02
	Shapiro-Wilk score	9.188E-01	9.601E-01	9.602E-01	9.962E-01	9.841E-01	9.996E-01	9.937E-01	9.944E-01	9.930E-01	9.933E-01	9.606E-01	9.867E-01

		(Ethanolout)	(Ethanolflow)	(Glycerolsynthesis)	(Glycerolout)	(Glycerolflow)	(Acetaldehydeout)	(Acetaldehydeflow)	(Cyanide-Acetaldehydeflow)	(Cyanideflow)	(Storage)	(ATPconsumption)	(Adenylatekinase)
(Glucose Mixed flow to extracellular medium)	Local	0.00E+00	0.00E+00	-7.83E-04	-6.35E-17	-5.80E-18	-1.88E-04	-9.78E-04	-2.78E-04	-2.22E-05	-9.81E-03	-2.24E-03	1.36E-18
	Min	0.00E+00	0.00E+00	-1.20E-03	-8.35E-16	-5.07E-16	-2.81E-04	-1.45E-03	-4.02E-04	-3.93E-05	-1.23E-02	-2.93E-03	-8.15E-18
	Max	0.00E+00	0.00E+00	-5.22E-04	7.75E-16	5.25E-16	-1.25E-04	-6.95E-04	-1.99E-04	-1.25E-05	-7.31E-03	-1.78E-03	1.56E-16
	Normalized peak height	1.00E+00	1.00E+00	3.83E-03	4.96E-03	4.36E-03	3.77E-03	3.52E-03	4.05E-03	3.60E-03	3.63E-03	3.60E-03	3.49E-01
	Peak sensitivity value	5.00E-04	5.00E-04	-7.67E-04	-1.72E-18	2.81E-17	-1.80E-04	-9.68E-04	-2.75E-04	-2.04E-05	-1.00E-02	-2.23E-03	-1.67E-20
	Shapiro-Wilk score	1.000E+00	1.000E+00	9.897E-01	9.992E-01	9.995E-01	9.914E-01	9.908E-01	9.924E-01	9.821E-01	9.998E-01	9.964E-01	7.202E-01
(Glucose uptake)	Local	0.00E+00	0.00E+00	-7.83E-04	-6.57E-17	-3.32E-18	-1.88E-04	-9.78E-04	-2.78E-04	-2.22E-05	-9.81E-03	-2.24E-03	1.36E-18
	Min	0.00E+00	0.00E+00	-1.18E-03	-8.85E-16	-5.22E-16	-2.84E-04	-1.44E-03	-4.10E-04	-4.00E-05	-1.24E-02	-2.90E-03	-7.22E-18
	Max	0.00E+00	0.00E+00	-5.15E-04	8.33E-16	4.86E-16	-1.28E-04	-6.84E-04	-2.01E-04	-1.26E-05	-7.24E-03	-1.77E-03	7.44E-17
	Normalized peak height	1.00E+00	1.00E+00	3.84E-03	5.30E-03	4.55E-03	3.77E-03	3.77E-03	3.65E-03	3.95E-03	3.82E-03	3.40E-03	3.48E-01
	Peak sensitivity value	5.00E-04	5.00E-04	-7.34E-04	-1.11E-17	1.76E-17	-1.85E-04	-9.43E-04	-2.74E-04	-2.09E-05	-9.75E-03	-2.23E-03	-2.74E-21
	Shapiro-Wilk score	1.000E+00	1.000E+00	9.891E-01	9.991E-01	9.997E-01	9.904E-01	9.897E-01	9.914E-01	9.810E-01	9.997E-01	9.957E-01	8.026E-01
(Hexokinase)	Local	0.00E+00	0.00E+00	-7.83E-04	-6.57E-17	-3.32E-18	-1.88E-04	-9.78E-04	-2.78E-04	-2.22E-05	-9.81E-03	-2.24E-03	1.36E-18
	Min	0.00E+00	0.00E+00	-1.17E-03	-7.15E-16	-5.08E-16	-2.89E-04	-1.42E-03	-4.08E-04	-3.89E-05	-1.25E-02	-2.93E-03	-1.90E-17
	Max	0.00E+00	0.00E+00	-5.00E-04	7.71E-16	4.89E-16	-1.26E-04	-6.94E-04	-1.98E-04	-1.24E-05	-7.48E-03	-1.79E-03	2.95E-16
	Normalized peak height	1.00E+00	1.00E+00	4.07E-03	4.48E-03	4.72E-03	3.79E-03	3.49E-03	3.79E-03	3.65E-03	3.67E-03	3.49E-03	3.50E-01
	Peak sensitivity value	5.00E-04	5.00E-04	-7.69E-04	-5.12E-17	2.95E-18	-1.84E-04	-9.56E-04	-2.78E-04	-1.99E-05	-9.84E-03	-2.23E-03	-3.55E-20
	Shapiro-Wilk score	1.000E+00	1.000E+00	9.892E-01	9.994E-01	9.997E-01	9.900E-01	9.895E-01	9.917E-01	9.811E-01	9.998E-01	9.958E-01	5.438E-01
(Phosphoglucoisomerase)	Local	0.00E+00	0.00E+00	2.26E-02	1.83E-15	9.24E-17	5.43E-03	2.82E-02	8.02E-03	6.39E-04	-2.82E-02	6.46E-02	3.55E-18
	Min	0.00E+00	0.00E+00	1.73E-02	-1.92E-14	-1.33E-14	4.36E-03	2.44E-02	6.89E-03	4.16E-04	-3.28E-02	5.58E-02	-1.82E-17
	Max	0.00E+00	0.00E+00	2.85E-02	1.92E-14	1.37E-14	6.61E-03	3.26E-02	9.17E-03	9.70E-04	-2.43E-02	7.33E-02	8.69E-16
	Normalized peak height	1.00E+00	1.00E+00	3.91E-03	4.17E-03	4.43E-03	3.25E-03	2.91E-03	3.11E-03	3.33E-03	3.83E-03	2.94E-03	3.48E-01
	Peak sensitivity value	5.00E-04	5.00E-04	2.24E-02	-2.60E-16	-9.66E-17	5.40E-03	2.79E-02	7.90E-03	5.94E-04	-2.84E-02	6.51E-02	-4.29E-20
	Shapiro-Wilk score	1.000E+00	1.000E+00	9.987E-01	9.999E-01	9.999E-01	9.983E-01	9.972E-01	9.985E-01	9.926E-01	9.998E-01	9.982E-01	5.060E-01
(Phosphofructokinase)	Local	0.00E+00	0.00E+00	2.26E-02	2.02E-15	1.13E-16	5.43E-03	2.82E-02	8.02E-03	6.39E-04	-2.82E-02	6.46E-02	3.55E-18
	Min	0.00E+00	0.00E+00	1.74E-02	-2.09E-14	-1.44E-14	4.31E-03	2.43E-02	6.91E-03	4.16E-04	-3.25E-02	5.62E-02	-1.92E-17
	Max	0.00E+00	0.00E+00	2.89E-02	1.96E-14	1.46E-14	6.66E-03	3.25E-02	9.18E-03	9.26E-04	-2.44E-02	7.37E-02	1.12E-16
	Normalized peak height	1.00E+00	1.00E+00	3.81E-03	4.25E-03	4.28E-03	3.31E-03	2.85E-03	3.36E-03	3.26E-03	3.55E-03	3.10E-03	3.46E-01
	Peak sensitivity value	5.00E-04	5.00E-04	2.23E-02	4.31E-17	1.29E-16	5.38E-03	2.74E-02	7.97E-03	6.05E-04	-2.83E-02	6.52E-02	-4.19E-20
	Shapiro-Wilk score	1.000E+00	1.000E+00	9.987E-01	9.999E-01	9.999E-01	9.982E-01	9.972E-01	9.986E-01	9.926E-01	9.999E-01	9.981E-01	8.653E-01
(Aldolase)	Local	0.00E+00	0.00E+00	2.26E-02	3.98E-16	1.24E-16	5.43E-03	2.82E-02	8.02E-03	6.39E-04	-2.82E-02	6.46E-02	3.55E-18
	Min	0.00E+00	0.00E+00	1.75E-02	-2.06E-14	-1.69E-14	4.36E-03	2.43E-02	6.91E-03	4.12E-04	-3.25E-02	5.63E-02	-1.87E-17
	Max	0.00E+00	0.00E+00	2.88E-02	1.99E-14	1.37E-14	6.69E-03	3.24E-02	9.27E-03	9.20E-04	-2.45E-02	7.33E-02	5.16E-16
	Normalized peak height	1.00E+00	1.00E+00	3.76E-03	4.50E-03	4.55E-03	3.28E-03	3.09E-03	3.28E-03	3.19E-03	3.58E-03	2.99E-03	3.51E-01
	Peak sensitivity value	5.00E-04	5.00E-04	2.27E-02	-8.60E-17	-3.41E-16	5.35E-03	2.76E-02	7.96E-03	6.02E-04	-2.86E-02	6.45E-02	-2.66E-19
	Shapiro-Wilk score	1.000E+00	1.000E+00	9.987E-01	9.999E-01	9.999E-01	9.983E-01	9.970E-01	9.986E-01	9.925E-01	9.997E-01	9.984E-01	6.923E-01
(Triosephosphate isomerase)	Local	0.00E+00	0.00E+00	-3.13E-02	-1.10E-17	1.39E-15	-7.52E-03	-3.91E-02	-1.11E-02	-8.85E-04	-1.89E-02	5.21E-02	2.13E-18
	Min	0.00E+00	0.00E+00	-4.03E-02	-5.58E-15	-4.09E-15	-9.57E-03	-4.66E-02	-1.30E-02	-1.37E-03	-2.36E-02	4.39E-02	-1.18E-17
	Max	0.00E+00	0.00E+00	-2.36E-02	5.39E-15	4.65E-15	-5.89E-03	-3.25E-02	-9.28E-03	-5.47E-04	-1.50E-02	6.11E-02	1.54E-16
	Normalized peak height	1.00E+00	1.00E+00	3.50E-03	4.38E-03	5.15E-03	3.23E-03	2.85E-03	2.98E-03	3.35E-03	3.51E-03	2.97E-03	3.45E-01
	Peak sensitivity value	5.00E-04	5.00E-04	-3.06E-02	4.51E-17	8.16E-17	-7.54E-03	-3.85E-02	-1.11E-02	-8.59E-04	-1.96E-02	5.29E-02	7.33E-21
	Shapiro-Wilk score	1.000E+00	1.000E+00	9.976E-01	9.990E-01	9.987E-01	9.973E-01	9.957E-01	9.969E-01	9.904E-01	9.992E-01	9.962E-01	7.993E-01
(Glyceraldehyde 3-phosphate dehydrogenase)	Local	0.00E+00	0.00E+00	-1.78E-03	7.65E-16	6.61E-16	-4.28E-04	-2.23E-03	-6.32E-04	-5.04E-05	-2.40E-02	5.89E-02	2.91E-18
	Min	0.00E+00	0.00E+00	-2.62E-03	-1.14E-14	-8.00E-15	-5.97E-04	-2.97E-03	-8.44E-04	-8.33E-05	-2.80E-02	5.05E-02	-5.21E-17
	Max	0.00E+00	0.00E+00	-1.26E-03	1.17E-14	8.70E-15	-3.10E-04	-1.69E-03	-4.97E-04	-3.10E-05	-2.05E-02	6.84E-02	2.16E-16
	Normalized peak height	1.00E+00	1.00E+00	4.06E-03	4.21E-03	4.08E-03	3.71E-03	3.31E-03	3.38E-03	3.48E-03	3.66E-03	3.21E-03	3.46E-01
	Peak sensitivity value	5.00E-04	5.00E-04	-1.72E-03	5.54E-16	-8.17E-18	-4.21E-04	-2.17E-03	-6.41E-04	-4.70E-05	-2.43E-02	5.97E-02	-7.15E-21
	Shapiro-Wilk score	1.000E+00	1.000E+00	9.941E-01	9.999E-01	1.000E+00	9.951E-01	9.946E-01	9.958E-01	9.867E-01	9.999E-01	9.979E-01	7.465E-01
(Phosphoenolpyruvate synthesis)	Local	0.00E+00	0.00E+00	-1.78E-03	-2.42E-15	1.16E-15	-4.28E-04	-2.23E-03	-6.32E-04	-5.04E-05	-2.40E-02	5.89E-02	2.91E-18
	Min	0.00E+00	0.00E+00	-2.61E-03	-1.43E-14	-7.35E-15	-6.11E-04	-3.03E-03	-8.35E-04	-8.19E-05	-2.80E-02	5.13E-02	-3.57E-17
	Max	0.00E+00	0.00E+00	-1.21E-03	1.52E-14	8.13E-15	-3.03E-04	-1.66E-03	-4.86E-04	-3.06E-05	-2.02E-02	6.75E-02	3.42E-16
	Normalized peak height	1.00E+00	1.00E+00	4.01E-03	4.24E-03	4.53E-03	3.78E-03	3.63E-03	3.59E-03	3.36E-03	3.76E-03	3.02E-03	3.45E-01
	Peak sensitivity value	5.00E-04	5.00E-04	-1.74E-03	-2.09E-16	1.19E-16	-4.19E-04	-2.18E-03	-6.24E-04	-4.65E-05	-2.43E-02	5.93E-02	-8.32E-20
	Shapiro-Wilk score	1.000E+00	1.000E+00	9.937E-01	9.998E-01	9.999E-01	9.946E-01	9.940E-01	9.958E-01	9.874E-01	9.999E-01	9.979E-01	7.212E-01
(Pyruvate kinase)	Local	0.00E+00	0.00E+00	-1.78E-03	4.02E-15	-8.26E-16	-4.28E-04	-2.23E-03	-6.32E-04	-5.04E-05	-2.40E-02	5.89E-02	2.91E-18
	Min	0.00E+00	0.00E+00	-2.62E-03	-1.33E-14	-7.34E-15	-6.06E-04	-3.01E-03	-8.45E-04	-8.45E-05	-2.79E-02	5.09E-02	-1.17E-16
	Max	0.00E+00	0.00E+00	-1.28E-03	1.29E-14	7.27E-15	-3.10E-04	-1.70E-03	-4.90E-04	-3.14E-05	-2.05E-02	6.83E-02	1.83E-16
	Normalized peak height	1.00E+00	1.00E+00	4.04E-03	4.44E-03	5.19E-03	3.66E-03	3.42E-03	3.56E-03	3.67E-03	3.84E-03	3.08E-03	3.48E-01
	Peak sensitivity value	5.00E-04	5.00E-04	-1.73E-03	4.46E-16	4.89E-18	-4.26E-04	-2.13E-03	-6.40E-04	-4.84E-05	-2.43E-02	5.99E-02	-1.03E-19
	Shapiro-Wilk score	1.000E+00	1.000E+00	9.944E-01	9.996E-01	9.996E-01	9.947E-01	9.940E-01	9.955E-01	9.868E-01	9.999E-01	9.975E-01	7.846E-01

(Pyruvate decarboxylase)	Local	1.16E-02	4.98E-03	9.03E-01	2.92E-03	3.54E-02	-8.93E-16	5.23E-04	1.14E-03	9.81E-05	2.96E-04	-6.66E-16	1.05E-02	0.00E+00	0.00E+00	-1.78E-03	-6.65E-16	1.91E-15	-4.28E-04	-2.23E-03	-6.32E-04	-5.04E-05	-2.40E-02	5.89E-02	2.91E-18
	Min	5.26E-03	2.44E-03	8.72E-01	2.41E-03	2.79E-02	-2.91E-15	3.70E-04	7.97E-04	6.75E-05	1.99E-04	-3.77E-15	7.18E-03	0.00E+00	0.00E+00	-2.49E-03	-1.00E-14	-7.41E-15	-6.03E-04	-3.01E-03	-8.26E-04	-8.40E-05	-2.79E-02	5.05E-02	-1.08E-16
	Max	2.91E-02	1.09E-02	9.18E-01	3.58E-03	4.76E-02	2.85E-15	7.50E-04	1.62E-03	1.38E-04	4.33E-04	4.00E-15	1.55E-02	0.00E+00	0.00E+00	-1.27E-03	1.15E-14	6.51E-15	-3.08E-04	-1.67E-03	-4.74E-04	-2.87E-05	-2.03E-02	6.78E-02	1.38E-16
	Normalized peak height	4.12E-03	4.15E-03	4.13E-03	3.52E-03	2.97E-03	1.54E-02	3.63E-03	3.78E-03	3.47E-03	3.53E-03	9.48E-02	3.42E-03	1.00E+00	1.00E+00	3.57E-03	5.70E-03	1.28E-02	3.58E-03	3.65E-03	3.50E-03	3.68E-03	3.72E-03	3.07E-03	3.45E-01
	Peak sensitivity value	8.68E-03	4.24E-03	9.05E-01	2.88E-03	3.39E-02	-1.43E-18	5.04E-04	1.13E-03	9.70E-05	2.78E-04	-2.19E-16	9.98E-03	5.00E-04	5.00E-04	-1.77E-03	8.00E-17	5.35E-18	-4.16E-04	-2.24E-03	-6.31E-04	-4.77E-05	-2.45E-02	5.92E-02	7.01E-20
	Shapiro-Wilk score	9.213E-01	9.612E-01	9.600E-01	9.963E-01	9.833E-01	9.828E-01	9.935E-01	9.939E-01	9.924E-01	9.926E-01	9.966E-01	9.859E-01	1.000E+00	1.000E+00	9.936E-01	9.964E-01	9.961E-01	9.949E-01	9.941E-01	9.956E-01	9.862E-01	9.999E-01	9.975E-01	7.984E-01
(Alcohol dehydrogenase)	Local	1.01E-02	4.37E-03	7.92E-01	2.32E-03	2.81E-02	-3.80E-16	9.18E-03	2.01E-02	1.72E-03	5.20E-03	6.30E-16	1.84E-01	0.00E+00	0.00E+00	-3.13E-02	-1.84E-16	1.26E-15	-7.52E-03	-3.91E-02	-1.11E-02	-8.85E-04	-1.89E-02	5.21E-02	2.13E-18
	Min	4.66E-03	2.18E-03	7.31E-01	1.94E-03	2.21E-02	-1.42E-15	6.82E-03	1.50E-02	1.24E-03	3.31E-03	-3.68E-15	1.37E-01	0.00E+00	0.00E+00	-4.04E-02	-5.51E-15	-3.79E-15	-9.53E-03	-4.68E-02	-1.30E-02	-1.34E-03	-2.38E-02	4.36E-02	-1.15E-17
	Max	2.69E-02	9.24E-03	8.34E-01	2.84E-03	3.80E-02	1.43E-15	1.18E-02	2.61E-02	2.25E-03	7.33E-03	4.05E-15	2.39E-01	0.00E+00	0.00E+00	-2.33E-02	5.75E-15	4.06E-15	-5.90E-03	-3.25E-02	-9.32E-03	-5.63E-04	-1.49E-02	6.12E-02	1.12E-16
	Normalized peak height	4.07E-03	4.17E-03	3.03E-03	3.47E-03	3.08E-03	4.85E-03	3.50E-03	3.68E-03	3.40E-03	3.75E-03	1.85E-02	2.80E-03	1.00E+00	1.00E+00	3.57E-03	4.64E-03	9.22E-03	3.35E-03	2.77E-03	3.05E-03	3.12E-03	3.75E-03	2.95E-03	3.48E-01
	Peak sensitivity value	8.16E-03	3.91E-03	8.02E-01	2.33E-03	2.73E-02	-1.32E-17	8.91E-03	1.98E-02	1.73E-03	5.17E-03	1.69E-18	1.75E-01	5.00E-04	5.00E-04	-3.05E-02	2.69E-16	4.99E-19	-7.40E-03	-3.82E-02	-1.09E-02	-8.75E-04	-1.90E-02	5.29E-02	5.40E-20
	Shapiro-Wilk score	9.215E-01	9.633E-01	9.832E-01	9.976E-01	9.879E-01	9.952E-01	9.967E-01	9.979E-01	9.963E-01	9.957E-01	9.968E-01	9.874E-01	1.000E+00	1.000E+00	9.975E-01	9.988E-01	9.983E-01	9.971E-01	9.958E-01	9.974E-01	9.907E-01	9.994E-01	9.965E-01	8.222E-01
(Ethanol out)	Local	1.01E-02	4.37E-03	7.92E-01	2.32E-03	2.81E-02	-4.21E-16	9.18E-03	2.01E-02	1.72E-03	5.20E-03	5.13E-16	1.84E-01	2.37E-16	-8.88E-16	-3.13E-02	-1.07E-16	1.31E-15	-7.52E-03	-3.91E-02	-1.11E-02	-8.85E-04	-1.89E-02	5.21E-02	2.13E-18
	Min	4.78E-03	2.17E-03	7.24E-01	1.93E-03	2.17E-02	-1.45E-15	6.89E-03	1.50E-02	1.28E-03	3.42E-03	-3.74E-15	1.42E-01	-2.44E-15	-2.66E-15	-4.02E-02	-5.51E-15	-4.13E-15	-9.49E-03	-4.65E-02	-1.30E-02	-1.39E-03	-2.33E-02	4.35E-02	-1.25E-17
	Max	2.61E-02	9.66E-03	8.32E-01	2.86E-03	3.75E-02	1.40E-15	1.19E-02	2.61E-02	2.33E-03	7.46E-03	3.60E-15	2.39E-01	2.11E-15	2.66E-15	-2.37E-02	6.71E-15	3.84E-15	-5.83E-03	-3.23E-02	-9.40E-03	-5.54E-04	-1.51E-02	6.13E-02	1.19E-16
	Normalized peak height	3.92E-03	4.19E-03	3.19E-03	3.40E-03	3.20E-03	9.87E-02	3.22E-03	3.50E-03	3.59E-03	3.92E-03	2.56E-01	2.55E-03	8.72E-02	4.73E-01	3.34E-03	5.14E-03	8.68E-03	3.24E-03	2.92E-03	2.82E-03	3.42E-03	3.40E-03	3.02E-03	3.54E-01
	Peak sensitivity value	8.85E-03	3.87E-03	7.94E-01	2.28E-03	2.67E-02	-1.20E-18	8.93E-03	1.93E-02	1.72E-03	5.11E-03	2.88E-18	1.74E-01	-2.23E-16	2.66E-18	-3.11E-02	8.30E-18	-4.97E-18	-7.33E-03	-3.79E-02	-1.12E-02	-8.45E-04	-1.92E-02	5.34E-02	-3.03E-20
	Shapiro-Wilk score	9.233E-01	9.643E-01	9.844E-01	9.979E-01	9.886E-01	9.949E-01	9.967E-01	9.978E-01	9.967E-01	9.955E-01	9.760E-01	9.882E-01	9.969E-01	8.770E-01	9.974E-01	9.988E-01	9.985E-01	9.974E-01	9.956E-01	9.971E-01	9.902E-01	9.994E-01	9.964E-01	7.917E-01
(Ethanol flow)	Local	1.01E-02	4.37E-03	7.92E-01	2.32E-03	2.81E-02	-3.81E-16	9.18E-03	2.01E-02	1.72E-03	5.20E-03	-1.74E-16	1.84E-01	-3.41E-16	-1.11E-15	-3.13E-02	-1.73E-16	1.29E-15	-7.52E-03	-3.91E-02	-1.11E-02	-8.85E-04	-1.89E-02	5.21E-02	2.13E-18
	Min	4.68E-03	2.23E-03	7.35E-01	1.94E-03	2.20E-02	-1.43E-15	6.87E-03	1.51E-02	1.26E-03	3.36E-03	-2.47E-15	1.38E-01	-5.25E-16	-2.66E-15	-4.05E-02	-5.31E-15	-3.55E-15	-9.46E-03	-4.66E-02	-1.29E-02	-1.35E-03	-2.35E-02	4.29E-02	-1.17E-17
	Max	2.68E-02	8.81E-03	8.37E-01	2.81E-03	3.71E-02	1.46E-15	1.19E-02	2.59E-02	2.28E-03	7.74E-03	2.87E-15	2.38E-01	5.36E-16	2.55E-15	-2.40E-02	5.78E-15	3.95E-15	-5.90E-03	-3.25E-02	-9.29E-03	-5.45E-04	-1.46E-02	6.20E-02	4.03E-16
	Normalized peak height	4.07E-03	3.86E-03	3.13E-03	3.17E-03	3.17E-03	1.83E-02	3.37E-03	3.67E-03	3.29E-03	3.87E-03	6.04E-02	2.63E-03	6.48E-01	1.18E-01	3.52E-03	4.72E-03	9.48E-03	3.09E-03	2.79E-03	2.86E-03	3.39E-03	3.77E-03	3.36E-03	3.48E-01
	Peak sensitivity value	8.19E-03	3.73E-03	7.99E-01	2.28E-03	2.75E-02	3.02E-20	9.07E-03	1.99E-02	1.64E-03	5.10E-03	1.58E-18	1.73E-01	-6.04E-20	-2.20E-16	-3.08E-02	6.44E-18	-6.16E-18	-7.46E-03	-3.84E-02	-1.11E-02	-8.34E-04	-1.95E-02	5.33E-02	1.37E-19
	Shapiro-Wilk score	9.216E-01	9.634E-01	9.846E-01	9.978E-01	9.878E-01	9.952E-01	9.969E-01	9.981E-01	9.960E-01	9.951E-01	9.991E-01	9.880E-01	8.095E-01	9.923E-01	9.978E-01	9.989E-01	9.985E-01	9.974E-01	9.958E-01	9.972E-01	9.907E-01	9.993E-01	9.967E-01	6.223E-01
(Glycerol synthesis)	Local	2.51E-02	1.08E-02	1.96E+00	8.63E-03	1.04E-01	8.06E-15	-8.18E-02	-1.79E-01	-1.53E-02	-4.63E-02	1.65E-14	-1.64E+00	0.00E+00	0.00E+00	2.79E-01	8.97E-15	-6.51E-15	6.70E-02	3.48E-01	9.89E-02	7.89E-03	-7.27E-02	1.24E-01	1.03E-17
	Min	1.10E-02	5.35E-03	1.81E+00	6.98E-03	8.11E-02	-3.13E-14	-1.05E-01	-2.19E-01	-1.85E-02	-6.03E-02	-4.82E-14	-1.82E+00	0.00E+00	0.00E+00	2.17E-01	-1.25E-13	-8.41E-14	5.43E-02	3.03E-01	8.08E-02	6.00E-03	-8.25E-02	1.08E-01	-5.30E-17
	Max	6.85E-02	2.42E-02	2.12E+00	1.06E-02	1.41E-01	3.09E-14	-6.08E-02	-1.38E-01	-1.26E-02	-3.38E-02	4.26E-14	-1.48E+00	0.00E+00	0.00E+00	3.34E-01	1.18E-13	8.34E-14	8.23E-02	3.94E-01	1.19E-01	1.02E-02	-6.42E-02	1.41E-01	5.00E-16
	Normalized peak height	4.15E-03	4.30E-03	2.96E-03	3.45E-03	3.23E-03	4.37E-03	3.28E-03	3.85E-03	3.52E-03	3.82E-03	1.41E-02	3.10E-03	1.00E+00	1.00E+00	3.52E-03	5.27E-03	1.33E-02	3.50E-03	3.80E-03	3.43E-03	3.13E-03	3.60E-03	3.06E-03	3.47E-01
	Peak sensitivity value	1.92E-02	1.00E-02	1.94E+00	8.46E-03	1.00E-01	5.79E-16	-8.09E-02	-1.80E-01	-1.50E-02	-4.52E-02	-2.79E-17	-1.62E+00	5.00E-04	5.00E-04	2.84E-01	-1.30E-16	5.40E-17	6.72E-02	3.52E-01	1.00E-01	7.56E-03	-7.26E-02	1.27E-01	-1.89E-19
	Shapiro-Wilk score	9.152E-01	9.563E-01	9.941E-01	9.943E-01	9.795E-01	9.991E-01	9.983E-01	9.997E-01	9.993E-01	9.985E-01	9.988E-01	9.953E-01	1.000E+00	1.000E+00	9.997E-01	9.997E-01	9.991E-01	9.991E-01	9.997E-01	9.999E-01	9.968E-01	9.993E-01	9.984E-01	8.088E-01
(Glycerol out)	Local	2.51E-02	1.08E-02	1.96E+00	8.63E-03	1.04E-01	8.12E-15	-8.18E-02	-1.79E-01	-1.53E-02	-4.63E-02	1.55E-14	-1.64E+00	0.00E+00	0.00E+00	2.79E-01	9.24E-15	-6.55E-15	6.70E-02	3.48E-01	9.89E-02	7.89E-03	-7.27E-02	1.24E-01	1.03E-17
	Min	1.11E-02	5.34E-03	1.81E+00	6.91E-03	7.95E-02	-3.42E-14	-1.03E-01	-2.17E-01	-1.83E-02	-6.14E-02	-5.16E-14	-1.81E+00	0.00E+00	0.00E+00	2.25E-01	-1.15E-13	-7.85E-14	5.31E-02	3.01E-01	8.17E-02	5.90E-03	-8.26E-02	1.08E-01	-5.46E-17
	Max	7.06E-02	2.42E-02	2.12E+00	1.07E-02	1.40E-01	3.23E-14	-6.23E-02	-1.44E-01	-1.26E-02	-3.42E-02	4.72E-14	-1.48E+00	0.00E+00	0.00E+00	3.33E-01	1.16E-13	7.77E-14	8.13E-02	3.99E-01	1.18E-01	1.03E-02	-6.42E-02	1.43E-01	7.47E-16
	Normalized peak height	4.31E-03	4.06E-03	3.07E-03	3.62E-03	3.17E-03	4.39E-03	3.02E-03	3.50E-03	3.57E-03	3.66E-03	2.51E-02	3.09E-03	1.00E+00	1.00E+00	3.37E-03	5.36E-03	1.40E-02	3.55E-03	3.77E-03	3.40E-03	3.39E-03	3.74E-03	3.22E-03	3.49E-01
	Peak sensitivity value	2.08E-02	1.01E-02	1.94E+00	8.43E-03	9.90E-02	-1.43E-15	-8.01E-02	-1.81E-01	-1.54E-02	-4.66E-02	2.54E-17	-1.62E+00	5.00E-04	5.00E-04	2.78E-01	-5.16E-15	-9.35E-16	6.67E-02	3.52E-01	9.92E-02	7.78E-03	-7.23E-02	1.23E-01	2.65E-19
	Shapiro-Wilk score	9.132E-01	9.552E-01	9.938E-01	9.945E-01	9.795E-01	9.992E-01	9.985E-01	9.994E-01	9.993E-01	9.986E-01	9.987E-01	9.948E-01	1.000E+00	1.000E+00	9.996E-01	9.996E-01	9.991E-01	9.992E-01	9.995E-01	9.998E-01	9.971E-01	9.992E-01	9.988E-01	7.375E-01
(Glycerol flow)	Local	2.51E-02	1.08E-02	1.96E+00	8.63E-03	1.04E-01	8.14E-15	-8.18E-02	-1.79E-01	-1.53E-02	-4.63E-02	1.49E-14	-1.64E+00	0.00E+00	0.00E+00	2.79E-01	9.72E-15	-6.66E-15	6.70E-02	3.48E-01	9.89E-02	7.89E-03	-7.27E-02	1.24E-01	1.03E-17
	Min	1.13E-02	5.22E-03	1.81E+00	6.82E-03	7.97E-02	-3.24E-14	-1.05E-01	-2.18E-01	-1.88E-02	-5.93E-02	-4.56E-14	-1.81E+00	0.00E+00	0.00E+00	2.24E-01	-1.19E-13	-8.84E-14	5.42E-02	3.04E-01	8.16E-02	5.93E-03	-8.30E-02	1.08E-01	-6.82E-17
	Max	6.78E-02	2.31E-02	2.12E+00	1.07E-02	1.42E-01	3.01E-14	-6.13E-02	-1.39E-01	-1.25E-02	-3.52E-02	4.97E-14	-1.47E+00	0.00E+00	0.00E+00	3.34E-01	1.18E-13	9.94E-14	8.16E-02	3.94E-01	1.20E-01	1.03E-02	-6.47E-02	1.42E-01	4.36E-16
	Normalized peak height	4.13E-03	4.02E-03	2.88E-03	3.53E-03	3.40E-03	4.32E-03	3.28E-03	3.83E-03	3.87E-03	3.60E-03	3.77E-02	3.17E-03	1.00E+00	1.00E+00	3.40E-03	5.92E-03	1.16E-02	3.38E-03	3.60E-03	3.53E-03	3.23E-03	3.62E-03	3.18E-03	3.46E-01
	Peak sensitivity value	2.04E-02	9.46E-03	1.94E+00	8.53E-03	1.03E-01	9.95E-16	-8.12E-02	-1.77E-01	-1.51E-02	-4.66E-02	1.63E-17	-1.62E+00	5.00E-04	5.00E-04	2.74E-01	-2.47E-16	1.45E-16	6.68E-02	3.49E-01	9.82E-02	7.89E-03	-7.24E-02	1.24E-01	5.56E-20
	Shapiro-Wilk score	9.135E-01	9.551E-01	9.938E-01	9.942E-01	9.799E-01	9.990E-01	9.981E-01	9.996E-01	9.992E-01	9.981E-01	9.989E-01	9.949E-01	1.000E+00	1.000E+00	9.996E-01	9.995E-01	9.991E-01	9.992E-01	9.997E-01	9.998E-01	9.968E-01	9.995E-01	9.988E-01	8.355E-01
(Acetaldehyde out)	Local	2.51E-02	1.08E-02	1.96E+00	8.63E-03	1.04E-01	-5.92E-15	-8.18E-02	-1.79E-01	-1.53E-02	-4.63E-02	4.56E-21	-1.64E+00	0.00E+00	0.00E+00	2.79E-01	-5.24E-15	8.08E-15	6.70E-02	3.48E-01	9.89E-02	7.89E-03	-7.27E-02	1.24E-01	1.03E-17
	Min	1.12E-02	5.47E-03	1.81E+00	7.01E-03	8.00E-02	-2.01E-14	-1.03E-01	-2.18E-01	-1.84E-02	-6.02E-02	-3.71E-14	-1.81E+00	0.00E+00	0.00E+00	2.20E-01	-7.26E-14	-4.64E-14	5.44E-02	3.02E-01	8.13E-02	5.87E-03	-8.30E-02	1.06E-01	-3.74E-16
	Max	6.91E-02	2.41E-02	2.11E+00	1.05E-02	1.41E-01	1.84E-14	-6.09E-02	-1.45E-01	-1.26E-02	-3.41E-02	4.67E-14	-1.47E+00	0.00E+00	0.00E+00	3.37E-01	7.89E-14	6.43E-14	8.27E-02	3.94E-01	1.18E-01	1.04E-02	-6.47E-02	1.43E-01	6.56E-16
	Normalized peak height	4.25E-03	4.09E-03	2.76E-03	3.28E-03	3.30E-03	2.75E-02	3.15E-03	3.47E-03	3.57E-03	3.82E-03	3.35E-01	2.88E-03	1.00E+00	1.00E+00	3.63E-03	5.01E-03	7.97E-03	3.52E-03	3.80E-03	3.87E-03	3.37E-03	3.73E-03	3.53E-03	3.50E-01
	Peak sensitivity value	1.83E-02	9.72E-03	1.93E+00	8.41E-03	9.93E-02	-1.48E-17	-8.22E-02	-1.79E-01	-1.53E-02	-4.59E-02	6.86E-18	-1.61E+00	5.00E-04	5.00E-04	2.76E-01	-3.41E-15	-4.19E-17	6.68E-02	3.48E-01	9.86E-02	7.84E-03	-7.24E-02	1.24E-01	-4.24E-19
	Shapiro-Wilk score	9.135E-01	9.559E-01	9.938E-01	9.931E-01	9.784E-01	9.965E-01	9.985E-01	9.997E-01	9.991E-01	9.982E-01	9.648E-01	9.944E-01	1.000E+00	1.000E+00	9.996E-01	9.986E-01	9.993E-01	9.993E-01	9.997E-01	9.998E-01	9.970E-01	9.992E-01	9.988E-01	7.531E-01
(Acetaldehyde flow)	Local	2.55E-02	1.10E-02	1.99E+00	8.78E-03	1.06E-01	-5.73E-15	-8.32E-02	-1.82E-01	-1.56E-02	-4.71E-02	-8.63E-15	-1.67E+00	0.00E+00	0.00E+00	2.84E-01	-5.10E-15	7.94E-15	6.82E-02	5.75E-01	-1.21E-01	-9.62E-03	-7.39E-02	1.27E-01	1.05E-17
	Min	1.14E-02	5.54E-03	1.83E+00	7.02E-03	7.94E-02	-1.95E-14	-1.06E-01	-2.23E-01	-1.91E-02	-6.10E-02	-3.16E-14	-1.84E+00	0.00E+00	0.00E+00	2.27E-01	-6.96E-14	-5.23E-14	5.52E-02	5.26E-01	-1.37E-01	-1.42E-02	-8.40E-02	1.09E-01	-2.48E-16
	Max	7.35E-02	2.55E-02	2.16E+00	1.10E-02	1.42E-01	1.96E-14	-6.17E-02	-1.46E-01	-1.29E-02	-3.47E-02	2.63E-14	-1.50E+00	0.00E+00	0.00E+00	3.39E-01	6.94E-14	5.05E-14	8.23E-02	6.24E-01	-1.04E-01	-6.37E-03	-6.36E-02	1.46E-01	6.95E-16
	Normalized peak height	4.23E-03	4.21E-03	2.97E-03	3.65E-03	3.09E-03	4.59E-03	3.38E-03	3.65E-03	3.60E-03	3.58E-03	6.65E-02	2.95E-03	1.00E+00	1.00E+00	3.52E-03	5.37E-03	1.10E-02	3.47E-03	3.45E-03	3.32E-03	3.63E-03	3.82E-03	3.45E-03	3.47E-01
	Peak sensitivity value	1.83E-02	9.12E-03	1.98E+00	8.59E-03	1.03E-01	-5.43E-16	-8.32E-02	-1.82E-01	-1.55E-02	-4.67E-02	-6.90E-18	-1.66E+00	5.00E-04	5.00E-04	2.84E-01	-1.50E-17	-4.37E-17	6.88E-02	5.79E-01	-1.21E-01	-9.40E-03	-7.36E-02	1.26E-01	-4.94E-20
	Shapiro-Wilk score	9.136E-01	9.552E-01	9.936E-01	9.941E-01	9.798E-01	9.959E-01	9.983E-01	9.997E-01	9.992E-01	9.983E-01	9.987E-01	9.943E-01	1.000E+00	1.000E+00	9.996E-01	9.991E-01	9.990E-01	9.993E-01	9.989E-01	9.988E-01	9.915E-01	9.993E-01	9.985E-01	7.895E-01
(Cyanide-Acetaldehyde flow)	Local	2.36E-02	1.02E-02	1.85E+00	8.13E-03	9.84E-02	-5.30E-15	-7.71E-02	-1.69E-01	-1.45E-02	-4.36E-02	-8.00E-15	-1.54E+00	0.00E+00	0.00E+00	2.63E-01	-4.73E-15	7.34E-15	6.31E-02	-3.93E-01	8.14E-01	6.50E-02	-6.85E-02	1.17E-01	9.70E-18
	Min	1.02E-02	4.99E-03	1.71E+00	6.69E-03	7.61E-02	-1.78E-14	-9.85E-02	-2.10E-01	-1.76E-02	-5.71E-02	-2.77E-14	-1.69E+00	0.00E+00	0.00E+00	2.12E-01	-6.62E-14	-5.01E-14	4.90E-02	-4.38E-01	7.82E-01	4.96E-02	-7.81E-02	1.01E-01	-5.03E-17
	Max	6.45E-02	2.29E-02	1.98E+00	1.00E-02	1.31E-01	1.76E-14	-5.78E-02	-1.36E-01	-1.19E-02	-3.13E-02	2.76E-14	-1.39E+00	0.00E+00	0.00E+00	3.19E-01	6.37E-14	4.56E-14	7.74E-02	-3.47E-01	8.42E-01	8.46E-02	-6.05E-02	1.34E-01	1.29E-15
	Normalized peak height	4.18E-03	4.10E-03	3.06E-03	3.46E-03	3.20E-03	4.78E-03	3.33E-03	3.58E-03	3.81E-03	4.00E-03	1.92E-02	3.30E-03	1.00E+00	1.00E+00	3.36E-03	4.88E-03	1.05E-02	3.63E-03	3.91E-03	3.43E-03	3.18E-03	4.06E-03	3.43E-03	3.50E-01
	Peak sensitivity value	1.97E-02	8.57E-03	1.82E+00	7.98E-03	9.47E-02	6.43E-17	-7.82E-02	-1.70E-01	-1.45E-02	-4.26E-02	5.78E-18	-1.53E+00	5.00E-04	5.00E-04	2.58E-01	-2.22E-15	-3.93E-17	6.33E-02	-3.91E-01	8.13E-01	6.30E-02	-6.85E-02	1.17E-01	1.24E-19
	Shapiro-Wilk score	9.143E-01	9.579E-01	9.957E-01	9.956E-01	9.812E-01	9.967E-01	9.983E-01	9.997E-01	9.994E-01	9.983E-01	9.984E-01	9.964E-01	1.000E+00	1.000E+00	9.996E-01	9.986E-01	9.990E-01	9.992E-01	9.996E-01	9.978E-01	9.925E-01	9.996E-01	9.987E-01	6.729E-01
(Cyanide flow)	Local	2.36E-02	1.02E-02	1.85E+00	8.13E-03	9.84E-02	-5.36E-15	-7.71E-02	-1.69E-01	-1.45E-02	-4.36E-02	-7.92E-15	-1.54E+00	0.00E+00	0.00E+00	2.63E-01	-4.59E-15	7.47E-15	6.31E-02	-3.93E-01	8.14E-01	6.50E-02	-6.85E-02	1.17E-01	9.70E-18
	Min	9.91E-03	5.04E-03	1.71E+00	6.71E-03	7.75E-02	-1.99E-14	-9.68E-02	-2.08E-01	-1.79E-02	-5.70E-02	-2.55E-14	-1.70E+00	0.00E+00	0.00E+00	2.09E-01	-7.11E-14	-4.38E-14	5.03E-02	-4.35E-01	7.83E-01	4.79E-02	-7.70E-02	1.03E-01	-5.82E-17
	Max	6.52E-02	2.23E-02	1.98E+00	1.01E-02	1.30E-01	1.94E-14	-5.74E-02	-1.34E-01	-1.17E-02	-3.26E-02	3.16E-14	-1.39E+00	0.00E+00	0.00E+00	3.20E-01	6.75E-14	5.08E-14	7.77E-02	-3.50E-01	8.41E-01	8.52E-02	-6.13E-02	1.35E-01	4.29E-16
	Normalized peak height	4.32E-03	4.17E-03	3.10E-03	3.44E-03	3.13E-03	6.85E-03	3.12E-03	3.57E-03	3.92E-03	3.69E-03	6.67E-02	3.27E-03	1.00E+00	1.00E+00	3.87E-03	5.43E-03	9.70E-03	3.47E-03	3.59E-03	3.49E-03	3.44E-03	3.80E-03	3.34E-03	3.48E-01
	Peak sensitivity value	1.85E-02	8.99E-03	1.84E+00	7.98E-03	9.39E-02	1.87E-17	-7.54E-02	-1.67E-01	-1.46E-02	-4.25E-02	3.56E-19	-1.53E+00	5.00E-04	5.00E-04	2.63E-01	7.33E-17	-4.04E-17	6.31E-02	-3.94E-01	8.19E-01	6.21E-02	-6.80E-02	1.18E-01	-1.40E-20
	Shapiro-Wilk score	9.144E-01	9.563E-01	9.962E-01	9.952E-01	9.802E-01	9.958E-01	9.982E-01	9.996E-01	9.993E-01	9.979E-01	9.985E-01	9.970E-01	1.000E+00	1.000E+00	9.997E-01	9.991E-01	9.991E-01	9.993E-01	9.996E-01	9.980E-01	9.929E-01	9.994E-01	9.987E-01	8.595E-01
(Storage)	Local	1.21E-02	5.20E-03	9.43E-01	-1.53E-03	-1.85E-02	-1.69E-15	9.90E-03	2.16E-02	1.86E-03	5.60E-03	-3.99E-15	1.98E-01	0.00E+00	0.00E+00	-3.37E-02	-2.74E-15	-1.38E-16	-8.11E-03	-4.21E-02	-1.20E-02	-9.55E-04	1.62E-02	-9.65E-02	-1.73E-18
	Min	5.46E-03	2.53E-03	8.79E-01	-2.17E-03	-2.60E-02	-7.20E-15	7.43E-03	1.61E-02	1.36E-03	3.64E-03	-1.06E-14	1.51E-01	0.00E+00	0.00E+00	-4.39E-02	-3.55E-14	-2.19E-14	-1.01E-02	-4.98E-02	-1.41E-02	-1.47E-03	1.26E-02	-1.13E-01	-2.00E-16
	Max	3.24E-02	1.07E-02	9.89E-01	-1.01E-03	-1.21E-02	7.08E-15	1.27E-02	2.81E-02	2.51E-03	8.07E-03	9.52E-15	2.56E-01	0.00E+00	0.00E+00	-2.55E-02	2.96E-14	1.94E-14	-6.31E-03	-3.51E-02	-9.96E-03	-6.02E-04	2.05E-02	-8.33E-02	8.81E-18
	Normalized peak height	4.50E-03	3.95E-03	2.97E-03	3.77E-03	3.72E-03	2.46E-02	3.32E-03	3.79E-03	3.59E-03	3.82E-03	5.97E-03	2.77E-03	1.00E+00	1.00E+00	3.65E-03	4.95E-03	4.29E-03	3.12E-03	2.77E-03	3.15E-03	3.34E-03	3.44E-03	3.09E-03	3.50E-01
	Peak sensitivity value	1.00E-02	4.60E-03	9.52E-01	-1.54E-03	-1.82E-02	4.86E-18	9.68E-03	2.15E-02	1.84E-03	5.35E-03	2.55E-16	1.89E-01	5.00E-04	5.00E-04	-3.29E-02	7.94E-16	7.63E-16	-8.00E-03	-4.13E-02	-1.19E-02	-9.06E-04	1.64E-02	-9.90E-02	-6.21E-20
	Shapiro-Wilk score	9.214E-01	9.637E-01	9.828E-01	9.991E-01	9.967E-01	9.999E-01	9.971E-01	9.983E-01	9.967E-01	9.960E-01	9.998E-01	9.884E-01	1.000E+00	1.000E+00	9.978E-01	9.997E-01	9.999E-01	9.974E-01	9.952E-01	9.969E-01	9.909E-01	9.984E-01	9.982E-01	7.648E-01
(ATP consumption)	Local	-1.59E-03	-6.83E-04	-1.24E-01	2.57E-02	3.11E-01	-8.56E-16	4.80E-03	1.05E-02	9.01E-04	2.72E-03	-2.17E-15	9.61E-02	0.00E+00	0.00E+00	-1.64E-02	-1.37E-15	-6.97E-17	-3.93E-03	-2.04E-02	-5.81E-03	-4.63E-04	-2.32E-01	9.53E-01	2.55E-17
	Min	-6.96E-03	-2.22E-03	-2.32E-01	2.14E-02	2.45E-01	-3.72E-15	3.30E-03	7.28E-03	6.22E-04	1.76E-03	-5.12E-15	6.66E-02	0.00E+00	0.00E+00	-2.33E-02	-1.71E-14	-1.09E-14	-5.67E-03	-2.73E-02	-7.67E-03	-7.69E-04	-2.61E-01	9.44E-01	-3.63E-16
	Max	-3.53E-04	-1.92E-04	-5.73E-02	3.05E-02	4.16E-01	3.66E-15	6.70E-03	1.53E-02	1.32E-03	4.17E-03	5.25E-15	1.40E-01	0.00E+00	0.00E+00	-1.15E-02	1.50E-14	1.05E-14	-2.83E-03	-1.55E-02	-4.47E-03	-2.75E-04	-2.09E-01	9.60E-01	1.14E-15
	Normalized peak height	5.85E-03	4.48E-03	3.08E-03	3.30E-03	2.97E-03	5.63E-02	3.48E-03	3.87E-03	3.63E-03	3.90E-03	1.84E-02	3.40E-03	1.00E+00	1.00E+00	3.68E-03	4.73E-03	4.52E-03	3.70E-03	3.23E-03	3.55E-03	3.43E-03	3.45E-03	3.42E-03	3.49E-01
	Peak sensitivity value	-9.38E-04	-4.75E-04	-1.05E-01	2.54E-02	3.03E-01	-3.14E-18	4.60E-03	1.03E-02	8.69E-04	2.58E-03	-4.72E-18	9.41E-02	5.00E-04	5.00E-04	-1.61E-02	3.84E-16	-8.08E-17	-3.96E-03	-2.02E-02	-5.67E-03	-4.33E-04	-2.31E-01	9.54E-01	3.97E-19
	Shapiro-Wilk score	8.755E-01	9.174E-01	9.758E-01	9.948E-01	9.811E-01	9.996E-01	9.910E-01	9.918E-01	9.905E-01	9.916E-01	9.997E-01	9.828E-01	1.000E+00	1.000E+00	9.915E-01	9.998E-01	9.999E-01	9.927E-01	9.918E-01	9.938E-01	9.842E-01	9.979E-01	9.953E-01	8.155E-01

(Adenylate kinase)												
Local	-INF	-INF	-INF	-INF	-INF	-INF	-INF	-INF	-INF	-INF	-INF	-INF
Min	-INF	-INF	-INF	-INF	-INF	-INF	-INF	-INF	-INF	-INF	-INF	-INF
Max	INF	INF	INF	INF	INF	INF	INF	INF	INF	INF	INF	INF
Normalized peak height	NA	NA	NA	NA	NA	NA	NA	NA	NA	NA	NA	NA
Peak sensitivity value	NAN	NAN	NAN	NAN	NAN	NAN	NAN	NAN	NAN	NAN	NAN	NAN
Shapiro-Wilk score	NA	NA	NA	NA	NA	NA	NA	NA	NA	NA	NA	NA

(Adenylate kinase)												
Local	-INF	-INF	-INF	-INF	-INF	-INF	-INF	-INF	-INF	-INF	-INF	-INF
Min	-INF	-INF	-INF	-INF	-INF	-INF	-INF	-INF	-INF	-INF	-INF	-INF
Max	INF	INF	INF	INF	INF	INF	INF	INF	INF	INF	INF	INF
Normalized peak height	NA	NA	NA	NA	NA	NA	NA	NA	NA	NA	NA	NA
Peak sensitivity value	NAN	NAN	NAN	NAN	NAN	NAN	NAN	NAN	NAN	NAN	NAN	NAN
Shapiro-Wilk score	NA	NA	NA	NA	NA	NA	NA	NA	NA	NA	NA	NA

Table S26: Flux control coefficients for *Saccharomyces cerevisiae* model with parameter variation of ±10%

		(GlucoseMixedflowtoextracellularmedium)	(Glucoseuptake)	(Hexokinase)	(Phosphoglucoisomerase)	(Phosphofructokinase)	(Aldolase)	(Triosephosphateisomerase)	(Glyceraldehyde3-phosphatedehydrogenase)	(Phosphoenolpyruvatesynthesis)	(Pyruvatekinase)	(Pyruvatedecarboxylase)	(Alcoholdehydrogenase)
(Glucose Mixed flow to extracellular medium)	Local	1.25E-02	5.36E-03	9.73E-01	1.37E-03	1.65E-02	-3.45E-17	2.30E-04	5.02E-04	4.31E-05	1.30E-04	-8.14E-17	4.60E-03
	Min	2.86E-03	1.45E-03	7.92E-01	7.97E-04	8.69E-03	-2.72E-16	1.02E-04	2.10E-04	1.91E-05	5.40E-05	-4.05E-16	1.93E-03
	Max	1.32E-01	3.72E-02	9.94E-01	2.89E-03	4.40E-02	2.77E-16	5.88E-04	1.31E-03	1.17E-04	3.42E-04	6.10E-16	1.26E-02
	Normalized peak height	1.30E-02	8.88E-03	1.01E-02	4.54E-03	5.22E-03	6.91E-02	4.45E-03	4.90E-03	5.24E-03	4.72E-03	2.35E-02	4.63E-03
	Peak sensitivity value	6.94E-03	4.48E-03	9.82E-01	1.30E-03	1.50E-02	4.56E-20	2.07E-04	4.68E-04	3.89E-05	1.09E-04	3.17E-19	3.99E-03
	Shapiro-Wilk score	7.072E-01	8.521E-01	7.873E-01	9.699E-01	9.289E-01	9.916E-01	9.585E-01	9.604E-01	9.566E-01	9.604E-01	9.807E-01	9.387E-01
(Glucose uptake)	Local	1.25E-02	5.36E-03	9.73E-01	1.37E-03	1.65E-02	-2.20E-17	2.30E-04	5.02E-04	4.31E-05	1.30E-04	-8.36E-17	4.60E-03
	Min	2.79E-03	1.40E-03	8.08E-01	8.17E-04	8.59E-03	-3.84E-16	1.00E-04	2.26E-04	1.93E-05	5.23E-05	-4.83E-16	2.02E-03
	Max	1.36E-01	2.88E-02	9.94E-01	2.69E-03	4.15E-02	2.83E-16	5.40E-04	1.20E-03	1.17E-04	3.64E-04	5.60E-16	1.28E-02
	Normalized peak height	1.34E-02	7.15E-03	9.25E-03	4.21E-03	4.43E-03	2.30E-02	4.43E-03	4.34E-03	4.79E-03	4.97E-03	1.26E-02	4.84E-03
	Peak sensitivity value	6.58E-03	3.77E-03	9.81E-01	1.26E-03	1.47E-02	2.82E-19	2.10E-04	4.51E-04	3.66E-05	1.16E-04	1.36E-19	3.70E-03
	Shapiro-Wilk score	7.070E-01	8.568E-01	7.894E-01	9.712E-01	9.297E-01	9.993E-01	9.602E-01	9.602E-01	9.573E-01	9.623E-01	9.875E-01	9.398E-01
(Hexokinase)	Local	1.25E-02	5.36E-03	9.73E-01	1.37E-03	1.65E-02	-4.14E-17	2.30E-04	5.02E-04	4.31E-05	1.30E-04	-1.06E-16	4.60E-03
	Min	2.91E-03	1.53E-03	8.06E-01	8.05E-04	8.85E-03	-2.38E-16	1.03E-04	2.22E-04	1.83E-05	4.79E-05	-6.27E-16	1.98E-03
	Max	1.29E-01	3.00E-02	9.93E-01	2.74E-03	4.16E-02	2.62E-16	5.81E-04	1.34E-03	1.16E-04	3.37E-04	5.12E-16	1.39E-02
	Normalized peak height	1.23E-02	7.76E-03	9.39E-03	4.25E-03	4.55E-03	3.84E-02	4.68E-03	5.18E-03	5.02E-03	4.94E-03	9.75E-03	5.07E-03
	Peak sensitivity value	6.51E-03	3.79E-03	9.80E-01	1.28E-03	1.44E-02	1.27E-19	2.01E-04	4.49E-04	3.96E-05	1.19E-04	-3.08E-20	4.04E-03
	Shapiro-Wilk score	7.091E-01	8.509E-01	7.887E-01	9.707E-01	9.310E-01	9.919E-01	9.589E-01	9.608E-01	9.587E-01	9.624E-01	9.853E-01	9.412E-01
(Phosphoglucoisomerase)	Local	1.27E-02	5.48E-03	9.94E-01	3.42E-03	4.14E-02	4.62E-16	-6.63E-03	-1.45E-02	-1.24E-03	-3.75E-03	2.63E-15	-1.33E-01
	Min	2.92E-03	1.44E-03	8.14E-01	2.24E-03	2.58E-02	-6.52E-15	-1.04E-02	-2.18E-02	-2.01E-03	-6.97E-03	-1.19E-14	-2.04E-01
	Max	1.37E-01	3.90E-02	1.05E+00	5.28E-03	8.70E-02	5.90E-15	-3.66E-03	-8.15E-03	-7.01E-04	-1.70E-03	1.29E-14	-8.24E-02
	Normalized peak height	1.29E-02	9.73E-03	6.38E-03	3.88E-03	4.63E-03	3.77E-02	3.43E-03	3.72E-03	3.59E-03	3.81E-03	2.19E-02	2.87E-03
	Peak sensitivity value	6.74E-03	3.97E-03	9.85E-01	3.29E-03	3.76E-02	5.23E-18	-6.54E-03	-1.39E-02	-1.18E-03	-3.48E-03	7.84E-18	-1.21E-01
	Shapiro-Wilk score	7.045E-01	8.454E-01	9.768E-01	9.853E-01	9.391E-01	9.979E-01	9.936E-01	9.969E-01	9.925E-01	9.875E-01	9.943E-01	9.806E-01
(Phosphofructokinase)	Local	1.27E-02	5.48E-03	9.94E-01	3.42E-03	4.14E-02	1.39E-15	-6.63E-03	-1.45E-02	-1.24E-03	-3.75E-03	3.85E-15	-1.33E-01
	Min	2.88E-03	1.49E-03	8.24E-01	2.35E-03	2.68E-02	-5.65E-15	-1.03E-02	-2.21E-02	-1.99E-03	-7.30E-03	-1.54E-14	-2.01E-01
	Max	1.30E-01	3.35E-02	1.06E+00	5.19E-03	8.39E-02	6.12E-15	-3.84E-03	-7.82E-03	-6.88E-04	-1.75E-03	1.72E-14	-8.04E-02
	Normalized peak height	1.23E-02	8.10E-03	6.27E-03	3.91E-03	3.91E-03	1.43E-02	3.17E-03	3.78E-03	3.53E-03	4.00E-03	7.17E-03	2.67E-03
	Peak sensitivity value	6.99E-03	3.62E-03	9.81E-01	3.34E-03	3.59E-02	-5.03E-18	-6.32E-03	-1.41E-02	-1.20E-03	-3.38E-03	-9.63E-18	-1.31E-01
	Shapiro-Wilk score	7.004E-01	8.481E-01	9.762E-01	9.850E-01	9.399E-01	9.976E-01	9.937E-01	9.973E-01	9.924E-01	9.879E-01	9.957E-01	9.806E-01
(Aldolase)	Local	1.27E-02	5.48E-03	9.94E-01	3.42E-03	4.14E-02	1.11E-15	-6.63E-03	-1.45E-02	-1.24E-03	-3.75E-03	-1.02E-14	-1.33E-01
	Min	2.76E-03	1.42E-03	8.43E-01	2.34E-03	2.65E-02	-6.00E-15	-1.01E-02	-2.22E-02	-2.03E-03	-6.99E-03	-5.56E-14	-2.07E-01
	Max	1.39E-01	2.92E-02	1.06E+00	5.83E-03	8.94E-02	7.33E-15	-3.75E-03	-8.80E-03	-6.79E-04	-1.67E-03	5.17E-14	-8.12E-02
	Normalized peak height	1.35E-02	6.95E-03	5.85E-03	4.21E-03	4.54E-03	5.47E-02	3.40E-03	3.47E-03	3.80E-03	3.98E-03	1.79E-02	2.86E-03
	Peak sensitivity value	7.06E-03	4.35E-03	9.86E-01	3.41E-03	3.59E-02	-2.20E-16	-6.34E-03	-1.38E-02	-1.19E-03	-3.67E-03	6.08E-18	-1.25E-01
	Shapiro-Wilk score	7.123E-01	8.532E-01	9.798E-01	9.848E-01	9.390E-01	9.983E-01	9.938E-01	9.966E-01	9.924E-01	9.866E-01	9.979E-01	9.800E-01
(Triosephosphate isomerase)	Local	1.01E-02	4.37E-03	7.92E-01	2.32E-03	2.81E-02	-1.03E-15	9.18E-03	2.01E-02	1.72E-03	5.20E-03	-5.10E-15	1.84E-01
	Min	2.46E-03	1.26E-03	6.18E-01	1.58E-03	1.75E-02	-2.31E-15	4.90E-03	1.05E-02	8.98E-04	2.35E-03	-2.06E-14	1.07E-01
	Max	9.44E-02	2.57E-02	8.62E-01	3.49E-03	5.78E-02	2.12E-15	1.48E-02	3.30E-02	3.12E-03	1.05E-02	2.12E-14	3.08E-01
	Normalized peak height	1.13E-02	7.81E-03	3.88E-03	3.48E-03	4.25E-03	4.85E-03	3.41E-03	3.82E-03	3.91E-03	4.11E-03	2.02E-02	2.93E-03
	Peak sensitivity value	5.72E-03	3.13E-03	8.14E-01	2.29E-03	2.65E-02	-2.11E-18	8.85E-03	1.90E-02	1.58E-03	4.78E-03	1.86E-17	1.55E-01
	Shapiro-Wilk score	7.156E-01	8.628E-01	9.570E-01	9.932E-01	9.606E-01	9.991E-01	9.911E-01	9.930E-01	9.887E-01	9.845E-01	9.975E-01	9.744E-01
(Glyceraldehyde 3-phosphate dehydrogenase)	Local	1.16E-02	4.98E-03	9.03E-01	2.92E-03	3.54E-02	-1.85E-16	5.23E-04	1.14E-03	9.81E-05	2.96E-04	-9.79E-15	1.05E-02
	Min	2.79E-03	1.31E-03	7.48E-01	2.07E-03	2.27E-02	-3.42E-15	2.65E-04	5.75E-04	5.07E-05	1.25E-04	-2.67E-14	5.37E-03
	Max	1.21E-01	2.79E-02	9.29E-01	4.43E-03	7.29E-02	3.80E-15	1.06E-03	2.39E-03	2.14E-04	6.35E-04	2.75E-14	2.37E-02
	Normalized peak height	1.24E-02	7.51E-03	8.62E-03	3.73E-03	4.11E-03	4.75E-03	3.87E-03	4.39E-03	4.27E-03	4.00E-03	4.74E-03	3.82E-03
	Peak sensitivity value	5.68E-03	4.14E-03	9.06E-01	2.88E-03	3.28E-02	-1.01E-16	5.08E-04	1.05E-03	9.43E-05	2.70E-04	-7.70E-18	9.05E-03
	Shapiro-Wilk score	7.089E-01	8.571E-01	8.243E-01	9.881E-01	9.466E-01	9.985E-01	9.756E-01	9.765E-01	9.724E-01	9.746E-01	9.984E-01	9.568E-01
(Phosphoenolpyruvate synthesis)	Local	1.16E-02	4.98E-03	9.03E-01	2.92E-03	3.54E-02	-2.35E-16	5.23E-04	1.14E-03	9.81E-05	2.96E-04	-4.52E-14	1.05E-02
	Min	2.52E-03	1.17E-03	7.71E-01	2.02E-03	2.31E-02	-2.55E-14	2.56E-04	5.80E-04	4.87E-05	1.35E-04	-2.56E-13	5.30E-03
	Max	1.06E-01	2.47E-02	9.27E-01	4.47E-03	7.03E-02	2.52E-14	1.19E-03	2.46E-03	2.36E-04	6.79E-04	1.99E-13	2.27E-02
	Normalized peak height	1.11E-02	6.45E-03	7.37E-03	3.82E-03	3.84E-03	3.89E-03	4.84E-03	4.20E-03	4.77E-03	4.20E-03	6.47E-03	3.80E-03
	Peak sensitivity value	5.79E-03	3.58E-03	9.07E-01	2.82E-03	3.21E-02	-4.52E-16	4.68E-04	1.04E-03	8.88E-05	2.81E-04	-1.79E-16	9.09E-03
	Shapiro-Wilk score	7.138E-01	8.579E-01	8.275E-01	9.888E-01	9.469E-01	9.999E-01	9.754E-01	9.763E-01	9.743E-01	9.759E-01	9.942E-01	9.573E-01
(Pyruvate kinase)	Local	1.16E-02	4.98E-03	9.03E-01	2.92E-03	3.54E-02	2.24E-15	5.23E-04	1.14E-03	9.81E-05	2.96E-04	5.94E-14	1.05E-02
	Min	2.70E-03	1.36E-03	7.21E-01	2.04E-03	2.32E-02	-1.59E-14	2.50E-04	5.42E-04	4.60E-05	1.31E-04	-2.15E-13	5.28E-03
	Max	1.32E-01	3.11E-02	9.29E-01	4.35E-03	7.13E-02	1.77E-14	1.16E-03	2.53E-03	2.44E-04	6.70E-04	2.26E-13	2.36E-02
	Normalized peak height	1.36E-02	8.40E-03	9.63E-03	3.68E-03	4.13E-03	1.04E-02	4.38E-03	4.52E-03	5.14E-03	4.34E-03	1.72E-01	3.95E-03
	Peak sensitivity value	6.52E-03	3.66E-03	9.07E-01	2.82E-03	3.13E-02	-1.59E-17	4.75E-04	1.07E-03	8.84E-05	2.64E-04	-9.49E-18	9.08E-03
	Shapiro-Wilk score	7.132E-01	8.558E-01	8.286E-01	9.881E-01	9.460E-01	9.975E-01	9.751E-01	9.760E-01	9.731E-01	9.745E-01	9.686E-01	9.585E-01

		(Ethanolout)	(Ethanolflow)	(Glycerolsynthesis)	(Glycerolout)	(Glycerolflow)	(Acetaldehydeout)	(Acetaldehydeflow)	(Cyanide-Acetaldehydeflow)	(Cyanideflow)	(Storage)	(ATPconsumption)	(Adenylatekinase)
(Glucose Mixed flow to extracellular medium)	Local	0.00E+00	0.00E+00	-7.83E-04	-6.35E-17	-5.80E-18	-1.88E-04	-9.78E-04	-2.78E-04	-2.22E-05	-9.81E-03	-2.24E-03	1.36E-18
	Min	-2.99E-17	-3.03E-17	-2.03E-03	-1.24E-15	-9.30E-16	-4.47E-04	-2.32E-03	-6.55E-04	-7.70E-05	-1.60E-02	-4.17E-03	-2.32E-17
	Max	3.17E-17	3.09E-17	-3.43E-04	1.08E-15	7.32E-16	-8.98E-05	-4.87E-04	-1.38E-04	-7.00E-06	5.14E-03	-1.41E-03	9.54E-17
	Normalized peak height	9.22E-01	9.28E-01	4.81E-03	7.10E-03	7.60E-03	4.29E-03	4.59E-03	5.10E-03	5.19E-03	7.31E-03	4.47E-03	3.45E-01
	Peak sensitivity value	4.50E-21	-2.77E-20	-7.20E-04	-8.03E-18	1.58E-18	-1.72E-04	-8.94E-04	-2.58E-04	-1.77E-05	-9.68E-03	-2.17E-03	8.99E-21
	Shapiro-Wilk score	2.838E-01	2.809E-01	9.598E-01	9.921E-01	9.907E-01	9.638E-01	9.617E-01	9.671E-01	9.338E-01	9.884E-01	9.844E-01	8.064E-01
(Glucose uptake)	Local	0.00E+00	0.00E+00	-7.83E-04	-6.57E-17	-3.32E-18	-1.88E-04	-9.78E-04	-2.78E-04	-2.22E-05	-9.81E-03	-2.24E-03	1.36E-18
	Min	-2.62E-17	-3.38E-17	-1.87E-03	-8.95E-16	-8.26E-16	-4.41E-04	-2.21E-03	-5.96E-04	-8.37E-05	-1.61E-02	-4.11E-03	-3.02E-17
	Max	2.96E-17	2.70E-17	-3.55E-04	9.99E-16	7.73E-16	-8.66E-05	-4.77E-04	-1.45E-04	-7.44E-06	2.60E-03	-1.41E-03	9.01E-17
	Normalized peak height	9.23E-01	9.32E-01	4.32E-03	5.65E-03	7.44E-03	4.46E-03	4.59E-03	4.20E-03	5.52E-03	6.42E-03	3.98E-03	3.45E-01
	Peak sensitivity value	-1.47E-20	5.28E-21	-6.85E-04	1.89E-17	-4.96E-19	-1.72E-04	-9.64E-04	-2.48E-04	-1.72E-05	-9.65E-03	-2.21E-03	3.09E-20
	Shapiro-Wilk score	2.853E-01	2.756E-01	9.603E-01	9.930E-01	9.911E-01	9.640E-01	9.625E-01	9.680E-01	9.367E-01	9.893E-01	9.847E-01	8.027E-01
(Hexokinase)	Local	0.00E+00	0.00E+00	-7.83E-04	-6.57E-17	-3.32E-18	-1.88E-04	-9.78E-04	-2.78E-04	-2.22E-05	-9.81E-03	-2.24E-03	1.36E-18
	Min	-3.67E-17	-2.75E-17	-2.04E-03	-1.16E-15	-9.60E-16	-5.49E-04	-2.50E-03	-6.43E-04	-7.64E-05	-1.72E-02	-3.98E-03	-8.26E-18
	Max	3.17E-17	2.90E-17	-3.65E-04	1.02E-15	6.71E-16	-8.01E-05	-4.67E-04	-1.38E-04	-6.63E-06	2.39E-03	-1.31E-03	5.12E-17
	Normalized peak height	9.25E-01	9.33E-01	4.85E-03	6.52E-03	7.43E-03	5.77E-03	4.98E-03	4.80E-03	5.11E-03	6.84E-03	4.23E-03	3.44E-01
	Peak sensitivity value	3.20E-20	1.36E-20	-6.78E-04	2.74E-17	-3.96E-19	-1.68E-04	-8.87E-04	-2.61E-04	-1.66E-05	-9.59E-03	-2.20E-03	-2.86E-20
	Shapiro-Wilk score	2.806E-01	2.743E-01	9.604E-01	9.923E-01	9.907E-01	9.650E-01	9.623E-01	9.686E-01	9.368E-01	9.888E-01	9.850E-01	8.601E-01
(Phosphoglucoisomerase)	Local	0.00E+00	0.00E+00	2.26E-02	1.83E-15	9.24E-17	5.43E-03	2.82E-02	8.02E-03	6.39E-04	-2.82E-02	6.46E-02	3.55E-18
	Min	-9.57E-16	-7.24E-16	1.29E-02	-2.24E-14	-1.96E-14	3.48E-03	2.07E-02	5.85E-03	2.73E-04	-3.77E-02	4.85E-02	-1.93E-17
	Max	8.48E-16	8.40E-16	3.54E-02	2.21E-14	1.84E-14	8.01E-03	3.67E-02	1.08E-02	1.31E-03	-1.28E-02	8.40E-02	2.45E-16
	Normalized peak height	9.24E-01	9.32E-01	3.68E-03	4.70E-03	5.60E-03	3.20E-03	3.18E-03	3.27E-03	3.50E-03	5.32E-03	3.21E-03	3.44E-01
	Peak sensitivity value	-8.77E-19	-6.56E-19	2.17E-02	4.45E-16	-4.94E-16	5.16E-03	2.75E-02	7.90E-03	5.59E-04	-2.83E-02	6.52E-02	-1.04E-19
	Shapiro-Wilk score	2.805E-01	2.742E-01	9.959E-01	9.987E-01	9.978E-01	9.955E-01	9.960E-01	9.973E-01	9.778E-01	9.979E-01	9.985E-01	8.373E-01
(Phosphofructokinase)	Local	0.00E+00	0.00E+00	2.26E-02	2.02E-15	1.13E-16	5.43E-03	2.82E-02	8.02E-03	6.39E-04	-2.82E-02	6.46E-02	3.55E-18
	Min	-8.44E-16	-8.73E-16	1.20E-02	-2.27E-14	-1.81E-14	3.44E-03	2.02E-02	5.87E-03	2.66E-04	-3.77E-02	4.84E-02	-5.15E-17
	Max	1.01E-15	8.23E-16	3.42E-02	2.05E-14	2.59E-14	8.00E-03	3.66E-02	1.08E-02	1.38E-03	-1.59E-02	8.46E-02	6.56E-16
	Normalized peak height	9.24E-01	9.33E-01	3.57E-03	4.61E-03	7.08E-03	3.48E-03	2.98E-03	3.37E-03	3.87E-03	4.57E-03	3.17E-03	3.45E-01
	Peak sensitivity value	3.56E-19	-3.28E-19	2.18E-02	-5.53E-16	3.61E-17	5.40E-03	2.73E-02	7.84E-03	6.11E-04	-2.82E-02	6.61E-02	-1.74E-19
	Shapiro-Wilk score	2.844E-01	2.730E-01	9.961E-01	9.988E-01	9.977E-01	9.948E-01	9.959E-01	9.975E-01	9.777E-01	9.980E-01	9.986E-01	6.394E-01
(Aldolase)	Local	0.00E+00	0.00E+00	2.26E-02	3.98E-16	1.24E-16	5.43E-03	2.82E-02	8.02E-03	6.39E-04	-2.82E-02	6.46E-02	3.55E-18
	Min	-8.22E-16	-8.04E-16	1.33E-02	-2.13E-14	-2.10E-14	3.46E-03	2.06E-02	5.98E-03	2.69E-04	-3.79E-02	4.76E-02	-1.96E-17
	Max	7.11E-16	7.05E-16	3.50E-02	2.23E-14	1.91E-14	8.12E-03	3.69E-02	1.05E-02	1.36E-03	-1.55E-02	8.35E-02	2.93E-16
	Normalized peak height	9.24E-01	9.29E-01	3.78E-03	4.46E-03	6.00E-03	3.42E-03	2.92E-03	3.15E-03	3.73E-03	4.99E-03	3.28E-03	3.43E-01
	Peak sensitivity value	4.73E-19	-1.85E-19	2.18E-02	4.95E-16	-1.47E-16	5.17E-03	2.78E-02	7.85E-03	5.63E-04	-2.78E-02	6.58E-02	-7.17E-20
	Shapiro-Wilk score	2.900E-01	2.782E-01	9.958E-01	9.989E-01	9.974E-01	9.954E-01	9.962E-01	9.973E-01	9.783E-01	9.985E-01	9.984E-01	8.235E-01
(Triosephosphate isomerase)	Local	0.00E+00	0.00E+00	-3.13E-02	-1.10E-17	1.39E-15	-7.52E-03	-3.91E-02	-1.11E-02	-8.85E-04	-1.89E-02	5.21E-02	2.13E-18
	Min	-7.56E-16	-5.38E-16	-5.03E-02	-8.27E-15	-4.70E-15	-1.17E-02	-5.49E-02	-1.52E-02	-2.08E-03	-2.82E-02	3.27E-02	-1.35E-17
	Max	6.79E-16	6.01E-16	-1.73E-02	7.41E-15	4.35E-15	-4.39E-03	-2.72E-02	-7.86E-03	-3.36E-04	-7.61E-03	7.09E-02	6.26E-17
	Normalized peak height	9.32E-01	9.42E-01	3.61E-03	6.16E-03	5.71E-03	3.54E-03	2.80E-03	3.01E-03	3.75E-03	4.24E-03	3.27E-03	3.43E-01
	Peak sensitivity value	-5.19E-19	2.18E-19	-3.13E-02	4.86E-17	1.18E-16	-6.95E-03	-3.83E-02	-1.13E-02	-7.95E-04	-2.00E-02	5.47E-02	2.69E-20
	Shapiro-Wilk score	2.679E-01	2.481E-01	9.919E-01	9.961E-01	9.950E-01	9.927E-01	9.924E-01	9.950E-01	9.707E-01	9.985E-01	9.976E-01	9.262E-01
(Glyceraldehyde 3-phosphate dehydrogenase)	Local	0.00E+00	0.00E+00	-1.78E-03	7.65E-16	6.61E-16	-4.28E-04	-2.23E-03	-6.32E-04	-5.04E-05	-2.40E-02	5.89E-02	2.91E-18
	Min	-6.95E-16	-7.05E-16	-3.76E-03	-1.71E-14	-8.26E-15	-8.78E-04	-4.20E-03	-1.08E-03	-1.46E-04	-3.36E-02	4.31E-02	-1.56E-17
	Max	7.92E-16	6.55E-16	-9.38E-04	1.39E-14	1.12E-14	-2.29E-04	-1.33E-03	-3.77E-04	-1.85E-05	-1.16E-02	7.65E-02	1.25E-16
	Normalized peak height	9.23E-01	9.28E-01	4.23E-03	5.55E-03	5.08E-03	4.23E-03	3.80E-03	3.64E-03	4.95E-03	5.19E-03	2.99E-03	3.46E-01
	Peak sensitivity value	5.06E-19	4.64E-19	-1.68E-03	1.27E-16	1.29E-17	-3.89E-04	-2.02E-03	-5.98E-04	-4.34E-05	-2.44E-02	5.95E-02	-7.09E-23
	Shapiro-Wilk score	2.846E-01	2.772E-01	9.763E-01	9.985E-01	9.981E-01	9.787E-01	9.776E-01	9.818E-01	9.534E-01	9.981E-01	9.983E-01	8.834E-01
(Phosphoenolpyruvate synthesis)	Local	0.00E+00	0.00E+00	-1.78E-03	-2.42E-15	1.16E-15	-4.28E-04	-2.23E-03	-6.32E-04	-5.04E-05	-2.40E-02	5.89E-02	2.91E-18
	Min	-7.50E-16	-7.26E-16	-3.70E-03	-1.96E-14	-9.86E-15	-8.70E-04	-4.12E-03	-1.18E-03	-1.37E-04	-3.35E-02	4.33E-02	-1.66E-17
	Max	7.96E-16	6.11E-16	-9.18E-04	1.94E-14	8.53E-15	-2.16E-04	-1.28E-03	-3.75E-04	-1.90E-05	-1.41E-02	7.68E-02	1.26E-16
	Normalized peak height	9.31E-01	9.46E-01	4.29E-03	5.33E-03	5.50E-03	4.30E-03	3.84E-03	3.96E-03	4.12E-03	4.30E-03	3.03E-03	3.44E-01
	Peak sensitivity value	6.14E-19	6.53E-19	-1.72E-03	1.98E-16	1.56E-16	-4.07E-04	-2.13E-03	-5.85E-04	-4.27E-05	-2.52E-02	5.95E-02	2.66E-21
	Shapiro-Wilk score	2.722E-01	2.529E-01	9.758E-01	9.972E-01	9.963E-01	9.795E-01	9.780E-01	9.832E-01	9.546E-01	9.988E-01	9.983E-01	8.917E-01
(Pyruvate kinase)	Local	0.00E+00	0.00E+00	-1.78E-03	4.02E-15	-8.26E-16	-4.28E-04	-2.23E-03	-6.32E-04	-5.04E-05	-2.40E-02	5.89E-02	2.91E-18
	Min	-8.34E-16	-5.95E-16	-3.91E-03	-1.62E-14	-7.49E-15	-8.44E-04	-4.23E-03	-1.18E-03	-1.37E-04	-3.38E-02	4.22E-02	-5.84E-17
	Max	8.26E-16	6.52E-16	-9.04E-04	1.37E-14	8.70E-15	-2.16E-04	-1.33E-03	-3.75E-04	-1.76E-05	-1.07E-02	7.73E-02	1.25E-16
	Normalized peak height	9.30E-01	9.47E-01	4.49E-03	5.08E-03	5.28E-03	3.89E-03	3.89E-03	4.15E-03	4.31E-03	5.17E-03	3.14E-03	3.40E-01
	Peak sensitivity value	-3.03E-19	3.04E-19	-1.70E-03	-1.26E-17	4.17E-19	-3.86E-04	-2.12E-03	-6.07E-04	-4.19E-05	-2.49E-02	6.07E-02	-3.04E-20
	Shapiro-Wilk score	2.754E-01	2.536E-01	9.752E-01	9.970E-01	9.966E-01	9.796E-01	9.780E-01	9.833E-01	9.552E-01	9.978E-01	9.982E-01	8.562E-01

(Pyruvate decarboxylase)												
Local	1.16E-02	4.98E-03	9.03E-01	2.92E-03	3.54E-02	-8.93E-16	5.23E-04	1.14E-03	9.81E-05	2.96E-04	-6.66E-16	1.05E-02
Min	2.65E-03	1.38E-03	7.50E-01	2.06E-03	2.27E-02	-3.41E-15	2.60E-04	5.39E-04	4.86E-05	1.26E-04	-5.55E-15	5.19E-03
Max	1.24E-01	2.77E-02	9.27E-01	4.65E-03	7.93E-02	5.35E-15	1.16E-03	2.36E-03	2.18E-04	6.41E-04	5.44E-15	2.81E-02
Normalized peak height	1.26E-02	7.33E-03	8.34E-03	4.16E-03	4.78E-03	2.03E-02	4.74E-03	4.20E-03	4.40E-03	4.20E-03	9.38E-02	4.60E-03
Peak sensitivity value	6.34E-03	3.47E-03	9.07E-01	2.87E-03	3.29E-02	4.10E-18	4.62E-04	1.13E-03	9.66E-05	2.73E-04	-2.26E-16	9.46E-03
Shapiro-Wilk score	7.115E-01	8.545E-01	8.265E-01	9.883E-01	9.444E-01	9.616E-01	9.754E-01	9.763E-01	9.742E-01	9.760E-01	9.947E-01	9.563E-01
(Alcohol dehydrogenase)												
Local	1.01E-02	4.37E-03	7.92E-01	2.32E-03	2.81E-02	-3.80E-16	9.18E-03	2.01E-02	1.72E-03	5.20E-03	6.30E-16	1.84E-01
Min	2.38E-03	1.25E-03	6.05E-01	1.55E-03	1.70E-02	-1.79E-15	5.31E-03	1.11E-02	9.38E-04	2.26E-03	-4.45E-15	1.07E-01
Max	1.12E-01	2.41E-02	8.61E-01	3.55E-03	5.53E-02	1.82E-15	1.49E-02	3.42E-02	3.12E-03	9.98E-03	4.60E-15	3.15E-01
Normalized peak height	1.39E-02	7.26E-03	4.01E-03	3.68E-03	3.94E-03	7.19E-03	3.63E-03	4.03E-03	3.94E-03	3.72E-03	1.86E-02	3.07E-03
Peak sensitivity value	5.62E-03	3.66E-03	8.05E-01	2.30E-03	2.65E-02	2.49E-18	8.92E-03	1.94E-02	1.61E-03	4.78E-03	-1.28E-18	1.63E-01
Shapiro-Wilk score	7.202E-01	8.651E-01	9.564E-01	9.935E-01	9.616E-01	9.823E-01	9.911E-01	9.930E-01	9.885E-01	9.840E-01	9.953E-01	9.738E-01
(Ethanol out)												
Local	1.01E-02	4.37E-03	7.92E-01	2.32E-03	2.81E-02	-4.21E-16	9.18E-03	2.01E-02	1.72E-03	5.20E-03	5.13E-16	1.84E-01
Min	2.50E-03	1.24E-03	6.24E-01	1.52E-03	1.75E-02	-1.68E-15	5.05E-03	1.06E-02	9.00E-04	2.27E-03	-3.72E-15	1.06E-01
Max	1.05E-01	2.95E-02	8.62E-01	3.60E-03	5.72E-02	1.64E-15	1.50E-02	3.30E-02	3.04E-03	1.05E-02	4.31E-15	3.06E-01
Normalized peak height	1.27E-02	8.98E-03	3.66E-03	3.87E-03	4.20E-03	1.06E-01	3.53E-03	3.89E-03	3.66E-03	4.09E-03	2.55E-01	2.78E-03
Peak sensitivity value	5.64E-03	3.12E-03	7.95E-01	2.29E-03	2.65E-02	-1.16E-18	8.86E-03	1.94E-02	1.65E-03	4.76E-03	2.01E-19	1.74E-01
Shapiro-Wilk score	7.101E-01	8.522E-01	9.564E-01	9.933E-01	9.619E-01	9.853E-01	9.914E-01	9.934E-01	9.886E-01	9.846E-01	9.831E-01	9.744E-01
(Ethanol flow)												
Local	1.01E-02	4.37E-03	7.92E-01	2.32E-03	2.81E-02	-3.81E-16	9.18E-03	2.01E-02	1.72E-03	5.20E-03	-1.74E-16	1.84E-01
Min	2.25E-03	1.31E-03	6.05E-01	1.58E-03	1.82E-02	-1.89E-15	4.92E-03	1.14E-02	9.18E-04	2.26E-03	-3.68E-15	1.06E-01
Max	1.21E-01	2.78E-02	8.60E-01	3.52E-03	5.66E-02	2.17E-15	1.54E-02	3.46E-02	3.07E-03	1.02E-02	3.02E-15	3.09E-01
Normalized peak height	1.45E-02	8.75E-03	4.00E-03	3.78E-03	3.89E-03	1.98E-02	3.73E-03	4.00E-03	3.80E-03	4.05E-03	6.32E-02	2.83E-03
Peak sensitivity value	5.74E-03	3.18E-03	8.10E-01	2.35E-03	2.61E-02	-1.42E-18	8.24E-03	1.93E-02	1.58E-03	4.69E-03	2.88E-19	1.64E-01
Shapiro-Wilk score	7.207E-01	8.633E-01	9.570E-01	9.933E-01	9.613E-01	9.822E-01	9.909E-01	9.929E-01	9.891E-01	9.851E-01	9.981E-01	9.739E-01
(Glycerol synthesis)												
Local	2.51E-02	1.08E-02	1.96E+00	8.63E-03	1.04E-01	8.06E-15	-8.18E-02	-1.79E-01	-1.53E-02	-4.63E-02	1.65E-14	-1.64E+00
Min	5.75E-03	2.84E-03	1.68E+00	5.83E-03	6.55E-02	-4.78E-14	-1.28E-01	-2.60E-01	-2.23E-02	-7.86E-02	-8.71E-14	-1.98E+00
Max	2.72E-01	6.36E-02	2.29E+00	1.31E-02	2.08E-01	5.30E-14	-4.29E-02	-1.13E-01	-9.87E-03	-2.49E-02	6.30E-14	-1.34E+00
Normalized peak height	1.27E-02	7.99E-03	3.05E-03	3.61E-03	4.35E-03	7.88E-03	3.48E-03	3.61E-03	3.66E-03	3.64E-03	1.59E-02	2.99E-03
Peak sensitivity value	1.31E-02	7.19E-03	1.93E+00	8.26E-03	9.07E-02	-2.89E-16	-8.08E-02	-1.80E-01	-1.49E-02	-4.52E-02	2.84E-17	-1.61E+00
Shapiro-Wilk score	6.918E-01	8.391E-01	9.897E-01	9.851E-01	9.383E-01	9.913E-01	9.963E-01	9.994E-01	9.974E-01	9.939E-01	9.916E-01	9.913E-01
(Glycerol out)												
Local	2.51E-02	1.08E-02	1.96E+00	8.63E-03	1.04E-01	8.12E-15	-8.18E-02	-1.79E-01	-1.53E-02	-4.63E-02	1.55E-14	-1.64E+00
Min	5.01E-03	2.75E-03	1.68E+00	5.67E-03	6.54E-02	-4.51E-14	-1.28E-01	-2.63E-01	-2.23E-02	-7.85E-02	-6.60E-14	-1.99E+00
Max	2.74E-01	7.20E-02	2.30E+00	1.34E-02	2.13E-01	4.88E-14	-4.46E-02	-1.10E-01	-1.03E-02	-2.33E-02	5.95E-14	-1.34E+00
Normalized peak height	1.31E-02	8.75E-03	2.97E-03	3.80E-03	4.25E-03	7.31E-03	3.19E-03	3.65E-03	3.69E-03	3.83E-03	2.59E-02	3.26E-03
Peak sensitivity value	1.27E-02	7.77E-03	1.91E+00	8.10E-03	9.26E-02	-3.64E-16	-7.86E-02	-1.81E-01	-1.53E-02	-4.46E-02	-5.22E-17	-1.59E+00
Shapiro-Wilk score	6.993E-01	8.436E-01	9.901E-01	9.859E-01	9.388E-01	9.910E-01	9.966E-01	9.993E-01	9.970E-01	9.940E-01	9.909E-01	9.918E-01
(Glycerol flow)												
Local	2.51E-02	1.08E-02	1.96E+00	8.63E-03	1.04E-01	8.14E-15	-8.18E-02	-1.79E-01	-1.53E-02	-4.63E-02	1.49E-14	-1.64E+00
Min	5.41E-03	2.77E-03	1.62E+00	5.64E-03	6.45E-02	-5.60E-14	-1.29E-01	-2.55E-01	-2.23E-02	-7.60E-02	-8.13E-14	-1.99E+00
Max	2.74E-01	6.93E-02	2.31E+00	1.29E-02	2.08E-01	4.26E-14	-4.46E-02	-1.13E-01	-1.00E-02	-2.47E-02	6.41E-14	-1.33E+00
Normalized peak height	1.31E-02	8.42E-03	3.32E-03	3.59E-03	4.09E-03	8.04E-03	3.24E-03	3.51E-03	3.84E-03	3.68E-03	3.79E-02	2.99E-03
Peak sensitivity value	1.25E-02	7.27E-03	1.90E+00	8.10E-03	9.26E-02	2.49E-16	-7.79E-02	-1.79E-01	-1.49E-02	-4.36E-02	-6.74E-17	-1.63E+00
Shapiro-Wilk score	7.015E-01	8.448E-01	9.904E-01	9.861E-01	9.395E-01	9.916E-01	9.966E-01	9.994E-01	9.965E-01	9.940E-01	9.910E-01	9.913E-01
(Acetaldehyde out)												
Local	2.51E-02	1.08E-02	1.96E+00	8.63E-03	1.04E-01	-5.92E-15	-8.18E-02	-1.79E-01	-1.53E-02	-4.63E-02	4.56E-21	-1.64E+00
Min	5.53E-03	2.60E-03	1.63E+00	5.55E-03	6.58E-02	-2.40E-14	-1.31E-01	-2.52E-01	-2.28E-02	-7.62E-02	-5.10E-14	-2.05E+00
Max	3.16E-01	6.68E-02	2.34E+00	1.32E-02	2.06E-01	2.31E-14	-4.38E-02	-1.07E-01	-1.04E-02	-2.52E-02	4.62E-14	-1.35E+00
Normalized peak height	1.48E-02	8.24E-03	3.49E-03	3.76E-03	4.12E-03	3.05E-02	3.31E-03	3.47E-03	3.76E-03	3.56E-03	2.97E-01	3.31E-03
Peak sensitivity value	1.44E-02	8.35E-03	1.93E+00	8.16E-03	9.02E-02	-1.49E-18	-8.00E-02	-1.84E-01	-1.50E-02	-4.34E-02	-2.64E-17	-1.54E+00
Shapiro-Wilk score	7.019E-01	8.450E-01	9.904E-01	9.855E-01	9.390E-01	9.837E-01	9.969E-01	9.994E-01	9.970E-01	9.933E-01	9.801E-01	9.918E-01
(Acetaldehyde flow)												
Local	2.55E-02	1.10E-02	1.99E+00	8.78E-03	1.06E-01	-5.73E-15	-8.32E-02	-1.82E-01	-1.56E-02	-4.71E-02	-8.63E-15	-1.67E+00
Min	5.24E-03	2.87E-03	1.63E+00	5.67E-03	6.66E-02	-3.13E-14	-1.31E-01	-2.63E-01	-2.22E-02	-7.84E-02	-5.08E-14	-2.06E+00
Max	2.70E-01	7.02E-02	2.32E+00	1.36E-02	2.19E-01	2.46E-14	-4.34E-02	-1.08E-01	-9.76E-03	-2.30E-02	4.84E-14	-1.34E+00
Normalized peak height	1.23E-02	8.44E-03	3.10E-03	3.71E-03	4.70E-03	8.37E-03	3.22E-03	3.65E-03	3.85E-03	3.78E-03	6.21E-02	3.08E-03
Peak sensitivity value	1.12E-02	7.34E-03	1.92E+00	8.60E-03	9.79E-02	-6.45E-17	-8.46E-02	-1.76E-01	-1.58E-02	-4.50E-02	-4.17E-17	-1.60E+00
Shapiro-Wilk score	6.960E-01	8.360E-01	9.896E-01	9.838E-01	9.358E-01	9.825E-01	9.966E-01	9.991E-01	9.968E-01	9.939E-01	9.908E-01	9.909E-01
(Cyanide-Acetaldehyde flow)												
Local	2.36E-02	1.02E-02	1.85E+00	8.13E-03	9.84E-02	-5.30E-15	-7.71E-02	-1.69E-01	-1.45E-02	-4.36E-02	-8.00E-15	-1.54E+00
Min	5.14E-03	2.49E-03	1.57E+00	5.51E-03	6.28E-02	-2.29E-14	-1.22E-01	-2.48E-01	-2.05E-02	-7.55E-02	-4.74E-14	-1.86E+00
Max	2.52E-01	5.90E-02	2.12E+00	1.22E-02	1.90E-01	2.43E-14	-3.81E-02	-1.01E-01	-9.62E-03	-2.37E-02	4.91E-14	-1.26E+00
Normalized peak height	1.28E-02	7.51E-03	3.43E-03	3.75E-03	3.91E-03	7.52E-03	3.32E-03	3.75E-03	3.36E-03	3.81E-03	1.97E-02	3.32E-03
Peak sensitivity value	1.19E-02	6.93E-03	1.80E+00	7.64E-03	8.61E-02	-2.31E-16	-7.56E-02	-1.66E-01	-1.40E-02	-4.07E-02	1.72E-17	-1.49E+00
Shapiro-Wilk score	7.007E-01	8.434E-01	9.927E-01	9.875E-01	9.414E-01	9.824E-01	9.963E-01	9.992E-01	9.968E-01	9.943E-01	9.901E-01	9.940E-01
(Cyanide flow)												
Local	2.36E-02	1.02E-02	1.85E+00	8.13E-03	9.84E-02	-5.36E-15	-7.71E-02	-1.69E-01	-1.45E-02	-4.36E-02	-7.92E-15	-1.54E+00
Min	5.14E-03	2.68E-03	1.51E+00	5.45E-03	6.13E-02	-2.16E-14	-1.25E-01	-2.43E-01	-2.12E-02	-7.46E-02	-3.60E-14	-1.88E+00
Max	2.49E-01	6.67E-02	2.17E+00	1.20E-02	1.89E-01	2.57E-14	-4.26E-02	-1.04E-01	-9.38E-03	-2.24E-02	5.22E-14	-1.26E+00
Normalized peak height	1.28E-02	8.67E-03	3.80E-03	3.51E-03	4.18E-03	7.82E-03	3.28E-03	3.55E-03	3.77E-03	3.95E-03	6.64E-02	3.46E-03
Peak sensitivity value	1.31E-02	7.19E-03	1.81E+00	7.78E-03	8.78E-02	4.53E-17	-7.53E-02	-1.67E-01	-1.40E-02	-4.36E-02	-3.08E-17	-1.51E+00
Shapiro-Wilk score	7.014E-01	8.453E-01	9.923E-01	9.872E-01	9.398E-01	9.841E-01	9.969E-01	9.993E-01	9.973E-01	9.939E-01	9.888E-01	9.941E-01
(Storage)												
Local	1.21E-02	5.20E-03	9.43E-01	-1.53E-03	-1.85E-02	-1.69E-15	9.90E-03	2.16E-02	1.86E-03	5.60E-03	-3.99E-15	1.98E-01
Min	2.98E-03	1.41E-03	7.40E-01	-2.77E-03	-4.06E-02	-8.43E-15	5.56E-03	1.16E-02	9.72E-04	2.55E-03	-1.46E-14	1.16E-01
Max	1.17E-01	3.10E-02	1.02E+00	-4.41E-04	-6.49E-03	9.23E-15	1.64E-02	3.52E-02	3.17E-03	1.12E-02	1.85E-14	3.30E-01
Normalized peak height	1.14E-02	8.41E-03	3.93E-03	3.93E-03	4.76E-03	2.84E-02	3.52E-03	3.81E-03	3.57E-03	3.84E-03	7.69E-03	2.98E-03
Peak sensitivity value	6.13E-03	4.12E-03	9.67E-01	-1.53E-03	-1.76E-02	6.33E-19	9.78E-03	2.03E-02	1.70E-03	5.23E-03	6.44E-18	1.78E-01
Shapiro-Wilk score	7.224E-01	8.609E-01	9.559E-01	9.978E-01	9.862E-01	9.970E-01	9.916E-01	9.934E-01	9.880E-01	9.837E-01	9.950E-01	9.740E-01
(ATP consumption)												
Local	-1.59E-03	-6.83E-04	-1.24E-01	2.57E-02	3.11E-01	-8.56E-16	4.80E-03	1.05E-02	9.01E-04	2.72E-03	-2.17E-15	9.61E-02
Min	-4.10E-02	-8.67E-03	-4.57E-01	1.83E-02	2.02E-01	-4.38E-15	2.34E-03	5.28E-03	4.50E-04	1.21E-03	-1.04E-14	4.87E-02
Max	-7.37E-05	-4.03E-05	-1.41E-02	3.74E-02	6.33E-01	3.77E-15	1.01E-02	2.21E-02	2.19E-03	6.28E-03	1.15E-14	2.37E-01
Normalized peak height	2.93E-02	1.32E-02	4.32E-03	3.58E-03	4.21E-03	6.54E-02	4.02E-03	4.18E-03	4.72E-03	4.23E-03	1.90E-02	4.45E-03
Peak sensitivity value	-4.22E-04	-2.78E-04	-9.41E-02	2.53E-02	2.84E-01	-8.72E-19	4.20E-03	9.55E-03	8.25E-04	2.46E-03	-7.46E-18	8.68E-02
Shapiro-Wilk score	6.058E-01	7.374E-01	9.332E-01	9.875E-01	9.434E-01	9.959E-01	9.701E-01	9.714E-01	9.690E-01	9.719E-01	9.873E-01	9.494E-01

(Pyruvate decarboxylase)												
Local	0.00E+00	0.00E+00	-1.78E-03	-6.65E-16	1.91E-15	-4.28E-04	-2.23E-03	-6.32E-04	-5.04E-05	-2.40E-02	5.89E-02	2.91E-18
Min	-7.78E-16	-6.74E-16	-3.89E-03	-1.13E-14	-7.86E-15	-8.95E-04	-5.05E-03	-1.24E-03	-1.47E-04	-3.28E-02	4.25E-02	-4.20E-17
Max	7.39E-16	6.80E-16	-8.77E-04	1.29E-14	9.43E-15	-2.27E-04	-1.28E-03	-3.70E-04	-1.87E-05	-1.33E-02	7.70E-02	2.21E-16
Normalized peak height	9.32E-01	9.48E-01	4.81E-03	7.47E-03	9.95E-03	4.56E-03	5.07E-03	4.43E-03	4.85E-03	4.58E-03	3.15E-03	3.41E-01
Peak sensitivity value	-5.55E-19	-3.77E-19	-1.66E-03	-8.68E-18	-7.32E-19	-4.09E-04	-2.19E-03	-6.12E-04	-4.24E-05	-2.48E-02	6.19E-02	-1.14E-19
Shapiro-Wilk score	2.745E-01	2.495E-01	9.758E-01	9.929E-01	9.919E-01	9.790E-01	9.761E-01	9.816E-01	9.526E-01	9.984E-01	9.984E-01	8.551E-01
(Alcohol dehydrogenase)												
Local	0.00E+00	0.00E+00	-3.13E-02	-1.84E-16	1.26E-15	-7.52E-03	-3.91E-02	-1.11E-02	-8.85E-04	-1.89E-02	5.21E-02	2.13E-18
Min	-6.33E-16	-7.02E-16	-5.24E-02	-6.61E-15	-4.39E-15	-1.18E-02	-5.56E-02	-1.51E-02	-2.01E-03	-2.86E-02	3.46E-02	-1.22E-17
Max	6.45E-16	5.89E-16	-1.76E-02	7.73E-15	4.75E-15	-4.45E-03	-2.70E-02	-7.78E-03	-3.56E-04	-7.83E-03	7.11E-02	1.51E-16
Normalized peak height	9.31E-01	9.40E-01	3.79E-03	5.70E-03	9.49E-03	3.33E-03	3.02E-03	3.09E-03	3.95E-03	4.42E-03	3.15E-03	3.45E-01
Peak sensitivity value	4.81E-19	6.11E-19	-2.97E-02	2.69E-16	2.52E-18	-7.32E-03	-3.81E-02	-1.08E-02	-7.78E-04	-1.95E-02	5.39E-02	3.99E-20
Shapiro-Wilk score	2.707E-01	2.579E-01	9.922E-01	9.965E-01	9.954E-01	9.931E-01	9.926E-01	9.946E-01	9.689E-01	9.989E-01	9.974E-01	8.069E-01
(Ethanol out)												
Local	2.37E-16	-8.88E-16	-3.13E-02	-1.07E-16	1.31E-15	-7.52E-03	-3.91E-02	-1.11E-02	-8.85E-04	-1.89E-02	5.21E-02	2.13E-18
Min	-2.44E-15	-2.66E-15	-5.24E-02	-6.64E-15	-4.43E-15	-1.17E-02	-5.51E-02	-1.50E-02	-1.89E-03	-2.82E-02	3.58E-02	-1.27E-17
Max	2.60E-15	2.66E-15	-1.67E-02	5.90E-15	5.02E-15	-4.69E-03	-2.68E-02	-7.84E-03	-3.58E-04	-8.25E-03	7.20E-02	3.22E-16
Normalized peak height	8.44E-02	4.81E-01	3.93E-03	4.99E-03	1.09E-02	3.19E-03	3.03E-03	3.21E-03	3.51E-03	4.27E-03	3.17E-03	3.41E-01
Peak sensitivity value	-2.23E-16	2.66E-18	-2.94E-02	-2.10E-16	-1.02E-18	-7.04E-03	-3.68E-02	-1.10E-02	-7.99E-04	-1.95E-02	5.54E-02	-1.12E-19
Shapiro-Wilk score	9.970E-01	8.746E-01	9.921E-01	9.971E-01	9.956E-01	9.920E-01	9.924E-01	9.952E-01	9.709E-01	9.979E-01	9.977E-01	7.558E-01
(Ethanol flow)												
Local	-3.41E-16	-1.11E-15	-3.13E-02	-1.73E-16	1.29E-15	-7.52E-03	-3.91E-02	-1.11E-02	-8.85E-04	-1.89E-02	5.21E-02	2.13E-18
Min	-7.26E-16	-2.89E-15	-5.41E-02	-6.72E-15	-4.53E-15	-1.14E-02	-5.42E-02	-1.51E-02	-1.95E-03	-2.80E-02	3.52E-02	-1.19E-17
Max	7.82E-16	2.78E-15	-1.72E-02	6.11E-15	4.92E-15	-4.60E-03	-2.67E-02	-7.89E-03	-3.59E-04	-6.63E-03	7.11E-02	1.29E-16
Normalized peak height	6.28E-01	1.20E-01	3.96E-03	5.15E-03	9.36E-03	3.21E-03	2.83E-03	3.03E-03	3.39E-03	4.56E-03	3.10E-03	3.43E-01
Peak sensitivity value	4.10E-19	-2.23E-16	-3.09E-02	-2.94E-17	1.95E-18	-7.09E-03	-3.80E-02	-1.10E-02	-7.12E-04	-1.94E-02	5.42E-02	1.75E-22
Shapiro-Wilk score	8.326E-01	9.930E-01	9.914E-01	9.970E-01	9.951E-01	9.930E-01	9.922E-01	9.949E-01	9.707E-01	9.987E-01	9.975E-01	8.349E-01
(Glycerol synthesis)												
Local	0.00E+00	0.00E+00	2.79E-01	8.97E-15	-6.51E-15	6.70E-02	3.48E-01	9.89E-02	7.89E-03	-7.27E-02	1.24E-01	1.03E-17
Min	-2.44E-15	-2.00E-15	1.71E-01	-1.49E-13	-1.09E-13	4.27E-02	2.47E-01	6.23E-02	4.37E-03	-9.36E-02	9.33E-02	-5.75E-17
Max	2.11E-15	2.03E-15	3.91E-01	1.50E-13	8.56E-14	9.93E-02	4.37E-01	1.39E-01	1.32E-02	-4.47E-02	1.60E-01	6.68E-16
Normalized peak height	9.22E-01	9.28E-01	3.45E-03	7.14E-03	1.70E-02	3.41E-03	3.88E-03	3.70E-03	3.55E-03	4.73E-03	3.14E-03	3.41E-01
Peak sensitivity value	-9.55E-19	5.29E-19	2.77E-01	-2.69E-17	-2.51E-17	6.51E-02	3.55E-01	9.84E-02	7.25E-03	-7.16E-02	1.26E-01	2.00E-19
Shapiro-Wilk score	2.914E-01	2.859E-01	9.996E-01	9.978E-01	9.971E-01	9.981E-01	9.993E-01	9.996E-01	9.903E-01	9.988E-01	9.977E-01	7.924E-01
(Glycerol out)												
Local	0.00E+00	0.00E+00	2.79E-01	9.24E-15	-6.55E-15	6.70E-02	3.48E-01	9.89E-02	7.89E-03	-7.27E-02	1.24E-01	1.03E-17
Min	-1.98E-15	-2.13E-15	1.67E-01	-1.35E-13	-8.50E-14	4.33E-02	2.60E-01	6.45E-02	4.35E-03	-9.38E-02	9.11E-02	-6.13E-17
Max	2.46E-15	2.27E-15	4.01E-01	1.29E-13	1.09E-13	9.84E-02	4.33E-01	1.38E-01	1.33E-02	-4.24E-02	1.62E-01	6.94E-16
Normalized peak height	9.24E-01	9.29E-01	3.51E-03	6.73E-03	9.79E-03	3.73E-03	3.40E-03	3.46E-03	3.40E-03	5.09E-03	3.31E-03	3.45E-01
Peak sensitivity value	1.52E-18	-5.58E-19	2.89E-01	5.37E-17	-1.87E-16	6.85E-02	3.56E-01	9.85E-02	7.44E-03	-7.15E-02	1.27E-01	2.73E-19
Shapiro-Wilk score	2.869E-01	2.811E-01	9.996E-01	9.969E-01	9.963E-01	9.981E-01	9.993E-01	9.996E-01	9.899E-01	9.989E-01	9.978E-01	7.868E-01
(Glycerol flow)												
Local	0.00E+00	0.00E+00	2.79E-01	9.72E-15	-6.66E-15	6.70E-02	3.48E-01	9.89E-02	7.89E-03	-7.27E-02	1.24E-01	1.03E-17
Min	-2.20E-15	-2.19E-15	1.72E-01	-1.87E-13	-9.99E-14	4.00E-02	2.54E-01	6.44E-02	4.35E-03	-9.43E-02	8.96E-02	-5.60E-17
Max	2.47E-15	1.91E-15	3.93E-01	1.62E-13	1.14E-13	9.65E-02	4.35E-01	1.37E-01	1.30E-02	-4.73E-02	1.64E-01	1.48E-15
Normalized peak height	9.22E-01	9.27E-01	3.44E-03	8.18E-03	1.27E-02	3.71E-03	3.73E-03	3.84E-03	3.33E-03	4.60E-03	3.53E-03	3.44E-01
Peak sensitivity value	-9.66E-19	4.47E-19	2.84E-01	1.17E-16	-6.89E-18	6.76E-02	3.61E-01	1.01E-01	7.28E-03	-7.19E-02	1.23E-01	-1.60E-20
Shapiro-Wilk score	2.911E-01	2.905E-01	9.996E-01	9.972E-01	9.965E-01	9.977E-01	9.994E-01	9.995E-01	9.902E-01	9.987E-01	9.977E-01	6.934E-01
(Acetaldehyde out)												
Local	0.00E+00	0.00E+00	2.79E-01	-5.24E-15	8.08E-15	6.70E-02	3.48E-01	9.89E-02	7.89E-03	-7.27E-02	1.24E-01	1.03E-17
Min	-1.73E-15	-1.48E-15	1.73E-01	-8.74E-14	-5.44E-14	4.26E-02	2.56E-01	6.15E-02	4.37E-03	-9.53E-02	9.35E-02	-5.58E-17
Max	1.72E-15	1.47E-15	3.90E-01	7.21E-14	5.63E-14	9.83E-02	4.41E-01	1.41E-01	1.33E-02	-4.53E-02	1.61E-01	6.95E-16
Normalized peak height	9.25E-01	9.31E-01	3.36E-03	5.22E-03	8.81E-03	3.53E-03	3.76E-03	3.78E-03	3.47E-03	5.14E-03	3.15E-03	3.43E-01
Peak sensitivity value	-8.67E-19	3.28E-19	2.78E-01	-7.38E-17	4.85E-17	6.55E-02	3.46E-01	9.80E-02	7.26E-03	-7.19E-02	1.24E-01	1.58E-19
Shapiro-Wilk score	2.870E-01	2.815E-01	9.995E-01	9.976E-01	9.965E-01	9.977E-01	9.994E-01	9.997E-01	9.909E-01	9.987E-01	9.975E-01	7.826E-01
(Acetaldehyde flow)												
Local	0.00E+00	0.00E+00	2.84E-01	-5.10E-15	7.94E-15	6.82E-02	5.75E-01	-1.21E-01	-9.62E-03	-7.39E-02	1.27E-01	1.05E-17
Min	-1.88E-15	-1.75E-15	1.67E-01	-8.74E-14	-5.14E-14	4.30E-02	4.72E-01	-1.56E-01	-2.07E-02	-9.69E-02	9.59E-02	-3.03E-16
Max	1.79E-15	1.65E-15	4.06E-01	7.99E-14	6.60E-14	9.77E-02	6.85E-01	-9.09E-02	-4.01E-03	-4.83E-02	1.62E-01	4.00E-16
Normalized peak height	9.24E-01	9.31E-01	3.67E-03	6.37E-03	1.48E-02	3.74E-03	4.00E-03	3.35E-03	4.00E-03	4.63E-03	3.11E-03	3.45E-01
Peak sensitivity value	-7.31E-19	-1.04E-18	2.89E-01	3.22E-17	-4.06E-18	6.82E-02	5.82E-01	-1.22E-01	-8.43E-03	-7.48E-02	1.29E-01	-5.32E-20
Shapiro-Wilk score	2.857E-01	2.812E-01	9.995E-01	9.975E-01	9.968E-01	9.978E-01	9.982E-01	9.984E-01	9.731E-01	9.987E-01	9.979E-01	8.848E-01
(Cyanide-Acetaldehyde flow)												
Local	0.00E+00	0.00E+00	2.63E-01	-4.73E-15	7.34E-15	6.31E-02	-3.93E-01	8.14E-01	6.50E-02	-6.85E-02	1.17E-01	9.70E-18
Min	-2.30E-15	-1.36E-15	1.63E-01	-6.73E-14	-4.92E-14	3.92E-02	-4.77E-01	7.54E-01	3.68E-02	-8.68E-02	8.75E-02	-9.88E-17
Max	1.77E-15	1.56E-15	3.67E-01	8.87E-14	5.81E-14	9.52E-02	-3.05E-01	8.65E-01	1.09E-01	-4.44E-02	1.54E-01	1.91E-15
Normalized peak height	9.23E-01	9.30E-01	3.36E-03	6.64E-03	1.32E-02	3.48E-03	3.63E-03	3.30E-03	3.70E-03	4.49E-03	3.48E-03	3.42E-01
Peak sensitivity value	-1.25E-19	8.98E-19	2.61E-01	-1.21E-17	2.87E-17	6.46E-02	-3.82E-01	8.21E-01	5.86E-02	-6.69E-02	1.14E-01	7.11E-19
Shapiro-Wilk score	2.938E-01	2.832E-01	9.994E-01	9.974E-01	9.969E-01	9.978E-01	9.995E-01	9.947E-01	9.825E-01	9.990E-01	9.982E-01	6.513E-01
(Cyanide flow)												
Local	0.00E+00	0.00E+00	2.63E-01	-4.59E-15	7.47E-15	6.31E-02	-3.93E-01	8.14E-01	6.50E-02	-6.85E-02	1.17E-01	9.70E-18
Min	-1.67E-15	-1.70E-15	1.62E-01	-6.45E-14	-5.41E-14	3.95E-02	-4.78E-01	7.48E-01	3.63E-02	-8.79E-02	8.80E-02	-5.03E-17
Max	1.54E-15	1.31E-15	3.69E-01	8.19E-14	4.83E-14	9.62E-02	-3.09E-01	8.69E-01	1.14E-01	-4.33E-02	1.52E-01	3.71E-16
Normalized peak height	9.22E-01	9.29E-01	3.37E-03	6.83E-03	9.19E-03	3.71E-03	3.64E-03	3.62E-03	3.62E-03	4.68E-03	3.22E-03	3.46E-01
Peak sensitivity value	-1.59E-18	-4.77E-19	2.59E-01	-2.02E-17	-4.90E-17	6.23E-02	-3.87E-01	8.17E-01	5.71E-02	-6.85E-02	1.18E-01	1.11E-19
Shapiro-Wilk score	2.926E-01	2.878E-01	9.996E-01	9.967E-01	9.940E-01	9.979E-01	9.996E-01	9.948E-01	9.815E-01	9.990E-01	9.980E-01	8.813E-01
(Storage)												
Local	0.00E+00	0.00E+00	-3.37E-02	-2.74E-15	-1.38E-16	-8.11E-03	-4.21E-02	-1.20E-02	-9.55E-04	1.62E-02	-9.65E-02	-1.73E-18
Min	-1.34E-15	-1.26E-15	-5.66E-02	-4.05E-14	-2.43E-14	-1.26E-02	-5.82E-02	-1.60E-02	-2.16E-03	8.71E-03	-1.34E-01	-1.07E-16
Max	1.23E-15	1.20E-15	-1.89E-02	3.74E-14	2.93E-14	-5.07E-03	-2.95E-02	-8.60E-03	-4.00E-04	2.93E-02	-7.12E-02	1.36E-17
Normalized peak height	9.25E-01	9.33E-01	3.77E-03	5.54E-03	5.49E-03	3.21E-03	2.74E-03	2.83E-03	3.61E-03	4.21E-03	3.43E-03	3.43E-01
Peak sensitivity value	-1.30E-19	-4.48E-19	-3.18E-02	3.57E-16	2.13E-16	-7.63E-03	-4.20E-02	-1.14E-02	-8.92E-04	1.67E-02	-9.79E-02	-2.04E-20
Shapiro-Wilk score	2.846E-01	2.733E-01	9.920E-01	9.983E-01	9.975E-01	9.921E-01	9.916E-01	9.950E-01	9.710E-01	9.975E-01	9.974E-01	8.308E-01
(ATP consumption)												
Local	0.00E+00	0.00E+00	-1.64E-02	-1.37E-15	-6.97E-17	-3.93E-03	-2.04E-02	-5.81E-03	-4.63E-04	-2.32E-01	9.53E-01	2.55E-17
Min	-6.47E-16	-5.69E-16	-3.32E-02	-2.11E-14	-1.89E-14	-9.04E-03	-4.22E-02	-1.15E-02	-1.31E-03	-3.09E-01	9.31E-01	-1.28E-16
Max	6.64E-16	6.41E-16	-8.36E-03	2.08E-14	1.39E-14	-2.01E-03	-1.19E-02	-3.45E-03	-1.58E-04	-1.92E-01	9.64E-01	1.32E-15
Normalized peak height	9.22E-01	9.32E-01	3.96E-03	5.89E-03	7.37E-03	4.90E-03	4.30E-03	4.27E-03	4.59E-03	4.07E-03	3.51E-03	3.48E-01
Peak sensitivity value	2.73E-20	4.91E-19	-1.54E-02	-8.69E-16	3.18E-17	-3.64E-03	-1.98E-02	-5.56E-03	-3.89E-04	-2.27E-01	9.54E-01	-5.82E-20
Shapiro-Wilk score	2.870E-01	2.803E-01	9.713E-01	9.954E-01	9.932E-01	9.744E-01	9.716E-01	9.775E-01	9.469E-01	9.922E-01	9.891E-01	8.697E-01

(Adenylate kinase)<br>Local<br>Min<br>Max<br>Normalized peak height<br>Peak sensitivity value<br>Shapiro-Wilk score	-INF<br>-INF<br>INF<br>NA<br>NAN<br>NA	-INF<br>-INF<br>INF<br>NA<br>NAN<br>NA	-INF<br>-INF<br>INF<br>NA<br>NAN<br>NA	-INF<br>-INF<br>INF<br>NA<br>NAN<br>NA	-INF<br>-INF<br>INF<br>NA<br>NAN<br>NA	-INF<br>-INF<br>INF<br>NA<br>NAN<br>NA	-INF<br>-INF<br>INF<br>NA<br>NAN<br>NA	-INF<br>-INF<br>INF<br>NA<br>NAN<br>NA	-INF<br>-INF<br>INF<br>NA<br>NAN<br>NA	-INF<br>-INF<br>INF<br>NA<br>NAN<br>NA	-INF<br>-INF<br>INF<br>NA<br>NAN<br>NA	-INF<br>-INF<br>INF<br>NA<br>NAN<br>NA	-INF<br>-INF<br>INF<br>NA<br>NAN<br>NA	-INF<br>-INF<br>INF<br>NA<br>NAN<br>NA	-INF<br>-INF<br>INF<br>NA<br>NAN<br>NA	-INF<br>-INF<br>INF<br>NA<br>NAN<br>NA	-INF<br>-INF<br>INF<br>NA<br>NAN<br>NA	-INF<br>-INF<br>INF<br>NA<br>NAN<br>NA	-INF<br>-INF<br>INF<br>NA<br>NAN<br>NA	-INF<br>-INF<br>INF<br>NA<br>NAN<br>NA	-INF<br>-INF<br>INF<br>NA<br>NAN<br>NA	-INF<br>-INF<br>INF<br>NA<br>NAN<br>NA	-INF<br>-INF<br>INF<br>NA<br>NAN<br>NA	-INF<br>-INF<br>INF<br>NA<br>NAN<br>NA

Table S27: Flux control coefficients for *Saccharomyces cerevisiae* model with parameter variation of ±20%

	(GlucoseMixedflowtoextracellularmedium)	(Glucoseuptake)	(Hexokinase)	(Phosphoglucoisomerase)	(Phosphofructokinase)	(Aldolase)	(Triosephosphateisomerase)	(Glyceraldehyde3-phosphatedehydrogenase)	(Phosphoenolpyruvatesynthesis)	(Pyruvatekinase)	(Pyruvatedecarboxylase)	(Alcoholdehydrogenase)	(Ethanolout)	(Ethanolflow)	(Glycerolsynthesis)	(Glycerolout)	(Glycerolflow)	(Acetaldehydeout)	(Acetaldehydeflow)	(Cyanide-Acetaldehydeflow)	(Cyanideflow)	(Storage)	(ATPconsumption)	(Adenylatekinase)
(Glucose Mixed flow to extracellular medium)<br>Local<br>Min<br>Max<br>Normalized peak height<br>Peak sensitivity value<br>Shapiro-Wilk score	1.25E-02<br>1.03E-03<br>8.82E-01<br>8.01E-02<br>3.23E-03<br>3.603E-01	5.36E-03<br>6.38E-04<br>1.98E-01<br>3.48E-02<br>2.32E-03<br>5.420E-01	9.73E-01<br>5.19E-02<br>9.99E-01<br>3.27E-02<br>9.89E-01<br>4.312E-01	1.37E-03<br>4.99E-04<br>7.82E-03<br>8.25E-03<br>1.12E-03<br>8.688E-01	1.65E-02<br>5.11E-03<br>2.53E-01<br>1.87E-02<br>1.19E-02<br>6.516E-01	-3.45E-17<br>-1.19E-15<br>1.48E-15<br>9.45E-02<br>-6.00E-19<br>7.950E-01	2.30E-04<br>-4.50E-04<br>3.07E-03<br>1.83E-02<br>1.79E-04<br>7.928E-01	5.02E-04<br>-1.08E-03<br>7.35E-03<br>1.87E-02<br>4.02E-04<br>7.709E-01	4.31E-05<br>-1.32E-04<br>5.69E-04<br>1.91E-02<br>3.25E-05<br>7.754E-01	1.30E-04<br>-4.84E-04<br>1.53E-03<br>1.74E-02<br>8.96E-05<br>8.184E-01	-8.14E-17<br>-1.22E-15<br>1.21E-15<br>4.47E-02<br>-9.12E-19<br>8.838E-01	4.60E-03<br>-1.26E-02<br>7.45E-02<br>2.14E-02<br>2.97E-03<br>6.996E-01	0.00E+00<br>-2.59E-16<br>2.22E-16<br>6.42E-01<br>-8.48E-20<br>5.042E-01	0.00E+00<br>-7.96E-16<br>8.89E-16<br>7.09E-01<br>1.02E-19<br>1.600E-01	-7.83E-04<br>-1.04E-02<br>1.97E-03<br>1.89E-02<br>-6.21E-04<br>7.779E-01	-6.35E-17<br>-4.28E-15<br>9.52E-15<br>4.75E-02<br>3.57E-18<br>8.335E-01	-5.80E-18<br>-4.50E-15<br>2.38E-15<br>3.63E-02<br>1.64E-18<br>8.271E-01	-1.88E-04<br>-2.68E-03<br>3.64E-04<br>1.86E-02<br>-1.48E-04<br>7.909E-01	-9.78E-04<br>-1.04E-02<br>2.05E-03<br>1.62E-02<br>-7.15E-04<br>7.863E-01	-2.78E-04<br>-2.75E-03<br>7.64E-04<br>1.63E-02<br>-2.10E-04<br>8.197E-01	-2.22E-05<br>-4.63E-04<br>6.49E-05<br>2.33E-02<br>-1.25E-05<br>7.009E-01	-9.81E-03<br>-3.22E-02<br>9.47E-02<br>1.94E-02<br>-9.93E-03<br>6.533E-01	-2.24E-03<br>-1.53E-02<br>4.31E-03<br>1.28E-02<br>-2.24E-03<br>8.922E-01	1.36E-18<br>-2.65E-17<br>5.04E-17<br>3.15E-01<br>1.05E-20<br>9.042E-01
(Glucose uptake)<br>Local<br>Min<br>Max<br>Normalized peak height<br>Peak sensitivity value<br>Shapiro-Wilk score	1.25E-02<br>1.09E-03<br>9.08E-01<br>8.61E-02<br>3.36E-03<br>3.615E-01	5.36E-03<br>5.54E-04<br>2.12E-01<br>3.79E-02<br>2.56E-03<br>5.435E-01	9.73E-01<br>4.33E-02<br>9.98E-01<br>3.36E-02<br>9.89E-01<br>4.333E-01	1.37E-03<br>4.74E-04<br>7.50E-03<br>8.09E-03<br>1.15E-03<br>8.771E-01	1.65E-02<br>5.24E-03<br>2.59E-01<br>1.96E-02<br>1.27E-02<br>6.678E-01	-2.20E-17<br>-9.11E-16<br>7.31E-16<br>3.58E-02<br>-1.50E-19<br>9.545E-01	2.30E-04<br>-5.12E-04<br>3.36E-03<br>1.98E-02<br>1.71E-04<br>7.930E-01	5.02E-04<br>-1.29E-03<br>6.80E-03<br>1.91E-02<br>3.58E-04<br>7.876E-01	4.31E-05<br>-9.74E-05<br>4.52E-04<br>1.45E-02<br>2.91E-05<br>7.978E-01	1.30E-04<br>-2.98E-04<br>1.04E-03<br>1.22E-02<br>8.77E-05<br>8.349E-01	-8.36E-17<br>-1.25E-15<br>1.14E-15<br>2.87E-02<br>-8.88E-19<br>9.059E-01	4.60E-03<br>-1.17E-02<br>6.87E-02<br>2.00E-02<br>2.84E-03<br>7.225E-01	0.00E+00<br>-3.59E-16<br>4.70E-16<br>6.55E-01<br>-3.93E-19<br>4.503E-01	0.00E+00<br>-1.40E-15<br>6.09E-16<br>7.13E-01<br>-1.35E-19<br>1.328E-01	-7.83E-04<br>-1.02E-02<br>1.96E-03<br>1.85E-02<br>-5.68E-04<br>7.938E-01	-6.57E-17<br>-4.91E-15<br>4.04E-15<br>3.10E-02<br>-2.98E-19<br>8.659E-01	-3.32E-18<br>-4.08E-15<br>2.80E-15<br>3.65E-02<br>-3.94E-18<br>8.459E-01	-1.88E-04<br>-1.98E-03<br>5.29E-04<br>1.51E-02<br>-1.47E-04<br>8.141E-01	-9.78E-04<br>-9.89E-03<br>1.99E-03<br>1.55E-02<br>-8.28E-04<br>8.015E-01	-2.78E-04<br>-2.71E-03<br>6.37E-04<br>1.57E-02<br>-2.24E-04<br>8.267E-01	-2.22E-05<br>-6.12E-04<br>7.98E-05<br>2.95E-02<br>-1.25E-05<br>7.001E-01	-9.81E-03<br>-3.31E-02<br>1.01E-01<br>2.06E-02<br>-9.36E-03<br>6.599E-01	-2.24E-03<br>-1.17E-02<br>4.29E-03<br>1.12E-02<br>-2.07E-03<br>9.022E-01	1.36E-18<br>-2.94E-17<br>6.45E-17<br>3.14E-01<br>2.37E-20<br>9.171E-01
(Hexokinase)<br>Local<br>Min<br>Max<br>Normalized peak height<br>Peak sensitivity value<br>Shapiro-Wilk score	1.25E-02<br>1.15E-03<br>9.02E-01<br>8.61E-02<br>3.41E-03<br>3.646E-01	5.36E-03<br>6.21E-04<br>2.09E-01<br>3.74E-02<br>2.39E-03<br>5.548E-01	9.73E-01<br>5.07E-02<br>9.99E-01<br>3.40E-02<br>9.88E-01<br>4.360E-01	1.37E-03<br>4.48E-04<br>7.48E-03<br>7.81E-03<br>1.24E-03<br>8.778E-01	1.65E-02<br>4.63E-03<br>2.11E-01<br>1.61E-02<br>1.26E-02<br>6.701E-01	-4.14E-17<br>-8.79E-16<br>1.58E-15<br>8.31E-02<br>-3.94E-19<br>8.037E-01	2.30E-04<br>-4.67E-04<br>2.04E-03<br>1.31E-02<br>1.70E-04<br>8.019E-01	5.02E-04<br>-9.75E-04<br>5.24E-03<br>1.57E-02<br>3.59E-04<br>7.911E-01	4.31E-05<br>-9.25E-05<br>5.53E-04<br>1.69E-02<br>3.18E-05<br>7.882E-01	1.30E-04<br>-2.92E-04<br>1.34E-03<br>1.45E-02<br>9.17E-05<br>8.275E-01	-1.06E-16<br>-2.84E-15<br>1.12E-15<br>3.50E-02<br>8.19E-19<br>8.858E-01	4.60E-03<br>-1.32E-02<br>6.39E-02<br>1.94E-02<br>2.92E-03<br>7.168E-01	0.00E+00<br>-2.83E-16<br>2.42E-16<br>6.49E-01<br>-1.14E-19<br>4.679E-01	0.00E+00<br>-1.07E-15<br>7.94E-16<br>7.10E-01<br>4.92E-19<br>1.658E-01	-7.83E-04<br>-7.85E-03<br>1.53E-03<br>1.43E-02<br>-6.12E-04<br>7.987E-01	-6.57E-17<br>-3.50E-15<br>3.91E-15<br>2.41E-02<br>-2.80E-18<br>8.691E-01	-3.32E-18<br>-2.64E-15<br>3.14E-15<br>3.07E-02<br>-2.88E-18<br>8.506E-01	-1.88E-04<br>-2.43E-03<br>3.35E-04<br>1.67E-02<br>-1.58E-04<br>8.064E-01	-9.78E-04<br>-8.74E-03<br>1.92E-03<br>1.43E-02<br>-7.71E-04<br>7.992E-01	-2.78E-04<br>-2.43E-03<br>5.45E-04<br>1.38E-02<br>-2.31E-04<br>8.222E-01	-2.22E-05<br>-3.97E-04<br>7.83E-05<br>2.11E-02<br>-1.18E-05<br>6.978E-01	-9.81E-03<br>-3.30E-02<br>1.06E-01<br>2.15E-02<br>-9.40E-03<br>6.656E-01	-2.24E-03<br>-1.29E-02<br>4.59E-03<br>1.17E-02<br>-2.00E-03<br>9.021E-01	1.36E-18<br>-5.28E-17<br>6.17E-17<br>3.14E-01<br>5.16E-20<br>8.835E-01
(Phosphoglucoisomerase)<br>Local<br>Min<br>Max<br>Normalized peak height<br>Peak sensitivity value<br>Shapiro-Wilk score	1.27E-02<br>1.10E-03<br>9.80E-01<br>8.89E-02<br>3.55E-03<br>3.594E-01	5.48E-03<br>5.73E-04<br>1.70E-01<br>2.87E-02<br>2.70E-03<br>5.462E-01	9.94E-01<br>5.82E-02<br>1.14E+00<br>1.43E-02<br>9.74E-01<br>5.088E-01	3.42E-03<br>1.71E-03<br>1.08E-02<br>5.81E-03<br>3.19E-03<br>9.358E-01	4.14E-02<br>1.84E-02<br>3.66E-01<br>1.40E-02<br>3.35E-02<br>7.001E-01	4.62E-16<br>-1.02E-14<br>9.26E-15<br>5.49E-02<br>-8.86E-18<br>9.879E-01	-6.63E-03<br>-1.51E-02<br>-1.82E-03<br>3.59E-03<br>-5.54E-03<br>9.781E-01	-1.45E-02<br>-3.55E-02<br>-4.92E-03<br>4.26E-03<br>-1.37E-02<br>9.872E-01	-1.24E-03<br>-3.01E-03<br>-3.75E-04<br>3.86E-03<br>-1.02E-03<br>9.739E-01	-3.75E-03<br>-1.21E-02<br>-7.54E-04<br>4.66E-03<br>-2.67E-03<br>9.547E-01	2.63E-15<br>-1.91E-14<br>3.61E-14<br>4.20E-02<br>6.48E-18<br>9.711E-01	-1.33E-01<br>-2.98E-01<br>-5.12E-02<br>3.41E-03<br>-1.06E-01<br>9.563E-01	0.00E+00<br>-5.64E-15<br>1.12E-14<br>6.53E-01<br>-1.76E-18<br>5.545E-01	0.00E+00<br>-2.44E-14<br>3.37E-14<br>7.17E-01<br>2.00E-17<br>1.512E-01	2.26E-02<br>7.09E-03<br>5.15E-02<br>4.12E-03<br>1.97E-02<br>9.838E-01	1.83E-15<br>-3.78E-14<br>4.83E-14<br>8.73E-03<br>5.50E-16<br>9.809E-01	9.24E-17<br>-2.61E-14<br>3.35E-14<br>1.01E-02<br>5.90E-19<br>9.707E-01	5.43E-03<br>2.14E-03<br>1.12E-02<br>3.38E-03<br>5.26E-03<br>9.835E-01	2.82E-02<br>1.50E-02<br>4.73E-02<br>3.13E-03<br>2.69E-02<br>9.917E-01	8.02E-03<br>4.16E-03<br>1.34E-02<br>3.49E-03<br>7.48E-03<br>9.916E-01	6.39E-04<br>1.20E-04<br>2.41E-03<br>4.47E-03<br>5.04E-04<br>9.310E-01	-2.82E-02<br>-6.00E-02<br>6.70E-02<br>1.16E-02<br>-2.88E-02<br>8.023E-01	6.46E-02<br>2.32E-02<br>1.05E-01<br>3.53E-03<br>6.67E-02<br>9.991E-01	3.55E-18<br>-1.28E-16<br>1.60E-16<br>3.13E-01<br>1.32E-20<br>8.619E-01
(Phosphofructokinase)<br>Local<br>Min<br>Max<br>Normalized peak height<br>Peak sensitivity value<br>Shapiro-Wilk score	1.27E-02<br>1.06E-03<br>9.67E-01<br>8.75E-02<br>3.47E-03<br>3.578E-01	5.48E-03<br>5.82E-04<br>1.88E-01<br>3.25E-02<br>2.36E-03<br>5.460E-01	9.94E-01<br>4.86E-02<br>1.14E+00<br>1.44E-02<br>9.73E-01<br>5.072E-01	3.42E-03<br>1.67E-03<br>1.10E-02<br>5.81E-03<br>3.15E-03<br>9.356E-01	4.14E-02<br>1.81E-02<br>4.23E-01<br>1.60E-02<br>3.24E-02<br>6.985E-01	1.39E-15<br>-8.69E-15<br>6.96E-15<br>1.96E-02<br>3.52E-18<br>9.667E-01	-6.63E-03<br>-1.53E-02<br>-1.67E-03<br>3.81E-03<br>-6.02E-03<br>9.785E-01	-1.45E-02<br>-3.04E-02<br>-4.71E-03<br>3.73E-03<br>-1.28E-02<br>9.888E-01	-1.24E-03<br>-3.12E-03<br>-3.84E-04<br>4.08E-03<br>-1.10E-03<br>9.745E-01	-3.75E-03<br>-1.29E-02<br>-7.56E-04<br>4.88E-03<br>-3.24E-03<br>9.549E-01	3.85E-15<br>-3.05E-14<br>3.02E-14<br>1.77E-02<br>2.78E-18<br>9.515E-01	-1.33E-01<br>-3.00E-01<br>-4.89E-02<br>3.12E-03<br>-1.02E-01<br>9.567E-01	0.00E+00<br>-4.99E-15<br>8.71E-15<br>6.44E-01<br>-6.68E-18<br>5.625E-01	0.00E+00<br>-2.75E-14<br>3.69E-14<br>7.13E-01<br>-2.60E-17<br>1.398E-01	2.26E-02<br>7.35E-03<br>4.74E-02<br>3.64E-03<br>1.95E-02<br>9.856E-01	2.02E-15<br>-4.00E-14<br>3.53E-14<br>7.81E-03<br>4.87E-16<br>9.795E-01	1.13E-16<br>-2.80E-14<br>2.67E-14<br>9.83E-03<br>-3.65E-17<br>9.706E-01	5.43E-03<br>1.94E-03<br>1.14E-02<br>3.45E-03<br>5.06E-03<br>9.825E-01	2.82E-02<br>1.49E-02<br>4.57E-02<br>3.03E-03<br>2.77E-02<br>9.924E-01	8.02E-03<br>4.00E-03<br>1.36E-02<br>3.71E-03<br>7.47E-03<br>9.924E-01	6.39E-04<br>9.95E-05<br>2.45E-03<br>4.61E-03<br>4.42E-04<br>9.296E-01	-2.82E-02<br>-6.50E-02<br>7.81E-02<br>1.30E-02<br>-2.96E-02<br>7.998E-01	6.46E-02<br>2.52E-02<br>1.05E-01<br>3.58E-03<br>6.73E-02<br>9.993E-01	3.55E-18<br>-7.33E-17<br>1.46E-16<br>3.15E-01<br>-8.76E-20<br>9.001E-01
(Aldolase)<br>Local<br>Min<br>Max<br>Normalized peak height<br>Peak sensitivity value<br>Shapiro-Wilk score	1.27E-02<br>1.09E-03<br>9.51E-01<br>8.58E-02<br>3.46E-03<br>3.636E-01	5.48E-03<br>5.67E-04<br>2.15E-01<br>3.74E-02<br>2.61E-03<br>5.425E-01	9.94E-01<br>4.48E-02<br>1.13E+00<br>1.45E-02<br>9.69E-01<br>5.068E-01	3.42E-03<br>1.66E-03<br>1.14E-02<br>5.80E-03<br>2.98E-03<br>9.351E-01	4.14E-02<br>1.93E-02<br>4.10E-01<br>1.55E-02<br>3.35E-02<br>7.019E-01	1.11E-15<br>-9.33E-15<br>9.77E-15<br>5.94E-02<br>-2.27E-16<br>9.835E-01	-6.63E-03<br>-1.56E-02<br>-1.85E-03<br>3.79E-03<br>-5.54E-03<br>9.790E-01	-1.45E-02<br>-3.04E-02<br>-4.30E-03<br>3.64E-03<br>-1.38E-02<br>9.885E-01	-1.24E-03<br>-3.41E-03<br>-4.14E-04<br>4.80E-03<br>-1.05E-03<br>9.733E-01	-3.75E-03<br>-1.29E-02<br>-7.11E-04<br>5.01E-03<br>-2.84E-03<br>9.531E-01	-1.02E-14<br>-6.95E-14<br>8.22E-14<br>2.99E-02<br>5.04E-17<br>9.810E-01	-1.33E-01<br>-2.93E-01<br>-5.03E-02<br>3.29E-03<br>-9.56E-02<br>9.557E-01	0.00E+00<br>-5.66E-15<br>8.79E-15<br>6.45E-01<br>6.25E-20<br>5.916E-01	0.00E+00<br>-2.99E-14<br>1.67E-14<br>6.89E-01<br>-1.75E-17<br>1.597E-01	2.26E-02<br>7.57E-03<br>4.66E-02<br>3.62E-03<br>2.11E-02<br>9.850E-01	3.98E-16<br>-5.04E-14<br>4.96E-14<br>1.06E-02<br>-7.30E-16<br>9.813E-01	1.24E-16<br>-2.68E-14<br>3.34E-14<br>1.01E-02<br>6.63E-17<br>9.706E-01	5.43E-03<br>2.15E-03<br>1.14E-02<br>3.58E-03<br>5.24E-03<br>9.833E-01	2.82E-02<br>1.45E-02<br>4.40E-02<br>2.77E-03<br>2.73E-02<br>9.921E-01	8.02E-03<br>3.96E-03<br>1.38E-02<br>3.54E-03<br>7.43E-03<br>9.924E-01	6.39E-04<br>9.19E-05<br>2.44E-03<br>4.61E-03<br>4.48E-04<br>9.313E-01	-2.82E-02<br>-6.08E-02<br>8.84E-02<br>1.38E-02<br>-2.91E-02<br>7.922E-01	6.46E-02<br>2.07E-02<br>1.04E-01<br>3.58E-03<br>6.70E-02<br>9.991E-01	3.55E-18<br>-8.22E-17<br>2.74E-16<br>3.16E-01<br>-1.20E-19<br>8.633E-01
(Triosephosphate isomerase)<br>Local<br>Min<br>Max<br>Normalized peak height<br>Peak sensitivity value<br>Shapiro-Wilk score	1.01E-02<br>8.70E-04<br>7.41E-01<br>8.23E-02<br>2.72E-03<br>3.665E-01	4.37E-03<br>4.97E-04<br>1.66E-01<br>3.60E-02<br>2.07E-03<br>5.532E-01	7.92E-01<br>3.33E-02<br>9.05E-01<br>7.27E-03<br>8.23E-01<br>6.943E-01	2.32E-03<br>9.79E-04<br>6.72E-03<br>5.17E-03<br>2.18E-03<br>9.597E-01	2.81E-02<br>1.02E-02<br>2.44E-01<br>1.30E-02<br>2.46E-02<br>7.578E-01	-1.03E-15<br>-2.69E-15<br>2.87E-15<br>7.17E-03<br>-5.39E-19<br>9.924E-01	9.18E-03<br>2.63E-03<br>2.48E-02<br>4.48E-03<br>7.33E-03<br>9.715E-01	2.01E-02<br>6.46E-03<br>5.41E-02<br>4.32E-03<br>1.78E-02<br>9.769E-01	1.72E-03<br>4.24E-04<br>5.15E-03<br>4.44E-03<br>1.44E-03<br>9.617E-01	5.20E-03<br>9.31E-04<br>1.77E-02<br>4.61E-03<br>4.18E-03<br>9.449E-01	-5.10E-15<br>-2.78E-14<br>3.07E-14<br>3.13E-02<br>1.28E-17<br>9.803E-01	1.84E-01<br>6.19E-02<br>5.09E-01<br>3.79E-03<br>1.45E-01<br>9.319E-01	0.00E+00<br>-4.03E-15<br>3.60E-15<br>6.66E-01<br>2.59E-18<br>6.485E-01	0.00E+00<br>-6.28E-15<br>7.88E-15<br>7.23E-01<br>1.05E-18<br>4.122E-01	-3.13E-02<br>-8.00E-02<br>-1.03E-02<br>4.06E-03<br>-2.73E-02<br>9.731E-01	-1.10E-17<br>-1.12E-14<br>9.97E-15<br>7.58E-03<br>-3.28E-17<br>9.859E-01	1.39E-15<br>-1.35E-14<br>7.78E-15<br>1.17E-02<br>-1.45E-16<br>9.741E-01	-7.52E-03<br>-1.75E-02<br>-2.79E-03<br>3.48E-03<br>-7.26E-03<br>9.757E-01	-3.91E-02<br>-7.10E-02<br>-1.76E-02<br>3.06E-03<br>-3.63E-02<br>9.816E-01	-1.11E-02<br>-1.97E-02<br>-5.56E-03<br>3.06E-03<br>-9.79E-03<br>9.878E-01	-8.85E-04<br>-3.94E-03<br>-1.49E-04<br>4.99E-03<br>-6.89E-04<br>9.085E-01	-1.89E-02<br>-4.30E-02<br>7.58E-02<br>1.05E-02<br>-1.96E-02<br>8.710E-01	5.21E-02<br>1.29E-02<br>9.18E-02<br>3.31E-03<br>5.36E-02<br>9.990E-01	2.13E-18<br>-3.39E-17<br>9.47E-17<br>3.17E-01<br>-1.79E-20<br>9.392E-01
(Glyceraldehyde 3-phosphate dehydrogenase)<br>Local<br>Min<br>Max<br>Normalized peak height<br>Peak sensitivity value<br>Shapiro-Wilk score	1.16E-02<br>1.05E-03<br>8.33E-01<br>8.26E-02<br>3.13E-03<br>3.645E-01	4.98E-03<br>5.59E-04<br>1.86E-01<br>3.45E-02<br>2.14E-03<br>5.446E-01	9.03E-01<br>3.46E-02<br>9.39E-01<br>2.21E-02<br>9.04E-01<br>4.531E-01	2.92E-03<br>1.55E-03<br>8.19E-03<br>5.06E-03<br>2.80E-03<br>9.414E-01	3.54E-02<br>1.67E-02<br>3.29E-01<br>1.49E-02<br>2.94E-02<br>7.175E-01	-1.85E-16<br>-6.06E-15<br>5.45E-15<br>8.70E-03<br>-2.57E-17<br>9.760E-01	5.23E-04<br>-1.87E-04<br>4.51E-03<br>1.27E-02<br>3.98E-04<br>8.455E-01	1.14E-03<br>-3.92E-04<br>9.26E-03<br>1.11E-02<br>8.96E-04<br>8.384E-01	9.81E-05<br>-3.55E-05<br>7.26E-04<br>1.03E-02<br>8.14E-05<br>8.409E-01	2.96E-04<br>-9.12E-05<br>1.87E-03<br>8.57E-03<br>2.17E-04<br>8.792E-01	-9.79E-15<br>-3.59E-14<br>3.83E-14<br>1.18E-02<br>-2.50E-17<br>9.822E-01	1.05E-02<br>-3.38E-03<br>1.08E-01<br>1.31E-02<br>7.26E-03<br>7.806E-01	0.00E+00<br>-3.50E-15<br>3.60E-15<br>6.36E-01<br>-5.04E-19<br>6.830E-01	0.00E+00<br>-1.83E-14<br>1.71E-14<br>6.89E-01<br>6.34E-18<br>2.420E-01	-1.78E-03<br>-1.36E-02<br>5.98E-04<br>1.09E-02<br>-1.53E-03<br>8.416E-01	7.65E-16<br>-1.94E-14<br>2.09E-14<br>7.58E-03<br>-8.22E-17<br>9.839E-01	6.61E-16<br>-1.44E-14<br>1.44E-14<br>8.51E-03<br>4.80E-17<br>9.711E-01	-4.28E-04<br>-2.81E-03<br>1.17E-04<br>9.23E-03<br>-3.56E-04<br>8.735E-01	-2.23E-03<br>-1.49E-02<br>7.96E-04<br>1.07E-02<br>-1.84E-03<br>8.554E-01	-6.32E-04<br>-3.23E-03<br>2.18E-04<br>8.59E-03<br>-5.07E-04<br>8.809E-01	-5.04E-05<br>-6.70E-04<br>2.00E-05<br>1.39E-02<br>-3.21E-05<br>7.738E-01	-2.40E-02<br>-5.30E-02<br>7.52E-02<br>1.24E-02<br>-2.52E-02<br>8.021E-01	5.89E-02<br>2.05E-02<br>9.57E-02<br>3.20E-03<br>6.01E-02<br>9.989E-01	2.91E-18<br>-3.87E-17<br>9.57E-17<br>3.16E-01<br>6.31E-20<br>9.272E-01

**(Phosphoenolpyruvate synthesis)**												
Local	1.16E-02	4.98E-03	9.03E-01	2.92E-03	3.54E-02	-2.35E-16	5.23E-04	1.14E-03	9.81E-05	2.96E-04	-4.52E-14	1.05E-02
Min	1.04E-03	5.76E-04	3.77E-02	1.52E-03	1.72E-02	-3.27E-14	-2.16E-04	-4.83E-04	-3.72E-05	-1.79E-04	-3.14E-13	-5.01E-03
Max	8.48E-01	1.68E-01	9.39E-01	8.29E-03	2.98E-01	3.55E-14	4.41E-03	8.80E-03	7.46E-04	1.90E-03	3.18E-13	9.55E-02
Normalized peak height	8.50E-02	3.16E-02	2.19E-02	5.03E-03	1.34E-02	5.56E-03	1.16E-02	1.10E-02	1.04E-02	9.15E-03	1.49E-02	1.16E-02
Peak sensitivity value	3.16E-03	2.33E-03	9.08E-01	2.75E-03	2.86E-02	3.33E-16	4.39E-04	9.69E-04	8.15E-05	2.41E-04	-3.15E-16	7.61E-03
Shapiro-Wilk score	3.631E-01	5.463E-01	4.496E-01	9.443E-01	7.190E-01	9.961E-01	8.551E-01	8.492E-01	8.543E-01	8.852E-01	9.679E-01	7.840E-01
**(Pyruvate kinase)**												
Local	1.16E-02	4.98E-03	9.03E-01	2.92E-03	3.54E-02	2.24E-15	5.23E-04	1.14E-03	9.81E-05	2.96E-04	5.94E-14	1.05E-02
Min	9.99E-04	5.30E-04	4.39E-02	1.47E-03	1.68E-02	-2.56E-14	-2.60E-04	-4.92E-04	-3.67E-05	-1.14E-04	-3.52E-13	-3.78E-03
Max	8.34E-01	1.76E-01	9.40E-01	1.03E-02	3.55E-01	2.10E-14	4.16E-03	8.82E-03	9.83E-04	2.04E-03	2.51E-13	1.18E-01
Normalized peak height	8.24E-02	3.34E-02	2.20E-02	6.12E-03	1.57E-02	2.54E-02	1.15E-02	1.08E-02	1.37E-02	9.07E-03	1.73E-01	1.42E-02
Peak sensitivity value	3.08E-03	2.37E-03	9.06E-01	2.83E-03	2.92E-02	-5.22E-18	4.23E-04	9.47E-04	8.00E-05	2.25E-04	2.35E-16	7.76E-03
Shapiro-Wilk score	3.601E-01	5.423E-01	4.492E-01	9.410E-01	7.007E-01	9.618E-01	8.444E-01	8.372E-01	8.290E-01	8.747E-01	9.339E-01	7.640E-01
**(Pyruvate decarboxylase)**												
Local	1.16E-02	4.98E-03	9.03E-01	2.92E-03	3.54E-02	-8.93E-16	5.23E-04	1.14E-03	9.81E-05	2.96E-04	-6.66E-16	1.05E-02
Min	1.02E-03	6.21E-04	4.47E-02	1.55E-03	1.56E-02	-4.79E-15	-8.88E-05	-2.46E-04	-1.99E-05	-6.56E-05	-8.44E-15	-2.75E-03
Max	8.46E-01	1.93E-01	9.40E-01	9.71E-03	3.87E-01	3.83E-15	3.74E-03	9.89E-03	9.10E-04	1.95E-03	1.31E-14	1.25E-01
Normalized peak height	8.49E-02	3.65E-02	2.18E-02	6.02E-03	1.72E-02	2.28E-02	9.63E-03	1.14E-02	1.23E-02	8.33E-03	9.65E-02	1.53E-02
Peak sensitivity value	3.13E-03	2.06E-03	9.07E-01	2.87E-03	2.88E-02	-3.54E-19	4.30E-04	8.33E-04	7.81E-05	2.22E-04	-2.21E-16	7.55E-03
Shapiro-Wilk score	3.604E-01	5.511E-01	4.484E-01	9.433E-01	7.079E-01	9.172E-01	8.496E-01	8.339E-01	8.335E-01	8.786E-01	9.688E-01	7.685E-01
**(Alcohol dehydrogenase)**												
Local	1.01E-02	4.37E-03	7.92E-01	2.32E-03	2.81E-02	-3.80E-16	9.18E-03	2.01E-02	1.72E-03	5.20E-03	6.30E-16	1.84E-01
Min	8.64E-04	5.70E-04	3.26E-02	9.09E-04	1.04E-02	-2.75E-15	2.63E-03	5.74E-03	4.64E-04	8.29E-04	-6.72E-15	6.31E-02
Max	7.52E-01	1.44E-01	9.01E-01	6.99E-03	2.10E-01	3.51E-15	2.51E-02	4.93E-02	4.79E-03	1.86E-02	6.10E-15	4.86E-01
Normalized peak height	8.36E-02	3.05E-02	7.36E-03	5.47E-03	1.14E-02	1.57E-02	4.19E-03	3.90E-03	4.17E-03	4.74E-03	2.53E-02	3.80E-03
Peak sensitivity value	2.74E-03	2.22E-03	8.44E-01	2.19E-03	2.38E-02	-5.27E-18	7.78E-03	1.68E-02	1.35E-03	3.77E-03	-5.80E-18	1.28E-01
Shapiro-Wilk score	3.626E-01	5.474E-01	6.977E-01	9.583E-01	7.583E-01	9.325E-01	9.693E-01	9.737E-01	9.605E-01	9.452E-01	9.870E-01	9.296E-01
**(Ethanol out)**												
Local	1.01E-02	4.37E-03	7.92E-01	2.32E-03	2.81E-02	-4.21E-16	9.18E-03	2.01E-02	1.72E-03	5.20E-03	5.13E-16	1.84E-01
Min	8.99E-04	4.98E-04	4.25E-02	7.01E-04	1.03E-02	-2.89E-15	2.66E-03	5.88E-03	4.99E-04	9.73E-04	-6.48E-15	6.41E-02
Max	7.29E-01	1.57E-01	9.02E-01	7.54E-03	2.18E-01	3.32E-15	2.31E-02	5.13E-02	5.77E-03	1.83E-02	8.54E-15	5.01E-01
Normalized peak height	8.06E-02	3.33E-02	7.28E-03	5.82E-03	1.17E-02	1.31E-01	3.78E-03	3.99E-03	4.85E-03	4.58E-03	2.77E-01	3.61E-03
Peak sensitivity value	2.72E-03	1.83E-03	8.41E-01	2.14E-03	2.44E-02	1.61E-18	7.64E-03	1.90E-02	1.49E-03	3.74E-03	3.98E-18	1.30E-01
Shapiro-Wilk score	3.654E-01	5.490E-01	6.983E-01	9.559E-01	7.543E-01	9.385E-01	9.703E-01	9.746E-01	9.617E-01	9.463E-01	9.821E-01	9.310E-01
**(Ethanol flow)**												
Local	1.01E-02	4.37E-03	7.92E-01	2.32E-03	2.81E-02	-3.81E-16	9.18E-03	2.01E-02	1.72E-03	5.20E-03	-1.74E-16	1.84E-01
Min	9.67E-04	5.62E-04	3.95E-02	1.06E-03	1.07E-02	-2.63E-15	2.39E-03	5.17E-03	4.94E-04	9.47E-04	-7.07E-15	6.54E-02
Max	7.34E-01	1.48E-01	9.06E-01	6.63E-03	2.07E-01	2.51E-15	2.42E-02	4.77E-02	5.05E-03	1.97E-02	5.85E-15	4.94E-01
Normalized peak height	8.43E-02	3.18E-02	7.15E-03	4.95E-03	1.13E-02	2.64E-02	3.91E-03	3.85E-03	4.51E-03	5.16E-03	7.96E-02	3.68E-03
Peak sensitivity value	2.80E-03	1.96E-03	8.39E-01	2.27E-03	2.33E-02	-1.07E-18	6.92E-03	1.72E-02	1.42E-03	3.84E-03	1.13E-18	1.38E-01
Shapiro-Wilk score	3.628E-01	5.459E-01	6.909E-01	9.616E-01	7.629E-01	9.297E-01	9.712E-01	9.756E-01	9.608E-01	9.454E-01	9.826E-01	9.323E-01
**(Glycerol synthesis)**												
Local	2.51E-02	1.08E-02	1.96E+00	8.63E-03	1.04E-01	8.06E-15	-8.18E-02	-1.79E-01	-1.53E-02	-4.63E-02	1.65E-14	-1.64E+00
Min	1.79E-03	1.05E-03	1.06E-01	3.94E-03	4.25E-02	-6.10E-14	-2.05E-01	-3.18E-01	-3.18E-02	-1.16E-01	-1.16E-13	-2.36E+00
Max	2.14E+00	4.50E-01	2.64E+00	2.54E-02	9.27E-01	5.34E-14	-1.86E-02	-5.87E-02	-5.67E-03	-1.24E-02	1.23E-13	-1.13E+00
Normalized peak height	9.91E-02	3.94E-02	6.03E-03	5.32E-03	1.39E-02	1.26E-02	3.85E-03	3.32E-03	4.12E-03	3.74E-03	2.88E-02	3.24E-03
Peak sensitivity value	7.14E-03	4.42E-03	1.72E+00	7.68E-03	7.47E-02	-7.48E-17	-7.37E-02	-1.84E-01	-1.39E-02	-4.42E-02	-1.16E-16	-1.53E+00
Shapiro-Wilk score	3.593E-01	5.429E-01	8.126E-01	9.507E-01	7.154E-01	9.433E-01	9.886E-01	9.978E-01	9.883E-01	9.748E-01	9.418E-01	9.848E-01
**(Glycerol out)**												
Local	2.51E-02	1.08E-02	1.96E+00	8.63E-03	1.04E-01	8.12E-15	-8.18E-02	-1.79E-01	-1.53E-02	-4.63E-02	1.55E-14	-1.64E+00
Min	1.94E-03	1.09E-03	1.03E-01	3.97E-03	4.21E-02	-6.15E-14	-1.92E-01	-3.29E-01	-3.12E-02	-1.20E-01	-1.36E-13	-2.45E+00
Max	2.08E+00	5.50E-01	2.72E+00	2.72E-02	1.05E+00	7.25E-14	-1.86E-02	-5.83E-02	-5.31E-03	-1.01E-02	1.77E-13	-1.11E+00
Normalized peak height	9.83E-02	4.84E-02	6.29E-03	5.51E-03	1.65E-02	1.42E-02	3.66E-03	3.30E-03	3.96E-03	4.14E-03	3.86E-02	3.34E-03
Peak sensitivity value	7.14E-03	4.65E-03	1.81E+00	7.54E-03	7.79E-02	2.39E-16	-7.23E-02	-1.75E-01	-1.39E-02	-3.46E-02	5.63E-17	-1.53E+00
Shapiro-Wilk score	3.581E-01	5.365E-01	8.121E-01	9.469E-01	7.081E-01	9.424E-01	9.881E-01	9.971E-01	9.888E-01	9.748E-01	9.323E-01	9.847E-01
**(Glycerol flow)**												
Local	2.51E-02	1.08E-02	1.96E+00	8.63E-03	1.04E-01	8.14E-15	-8.18E-02	-1.79E-01	-1.53E-02	-4.63E-02	1.49E-14	-1.64E+00
Min	1.75E-03	1.02E-03	1.20E-01	3.88E-03	4.55E-02	-5.51E-14	-1.84E-01	-3.29E-01	-3.03E-02	-1.20E-01	-1.45E-13	-2.41E+00
Max	2.09E+00	3.86E-01	2.71E+00	2.36E-02	1.09E+00	5.33E-14	-1.75E-02	-5.80E-02	-5.13E-03	-9.28E-03	1.34E-13	-1.12E+00
Normalized peak height	9.83E-02	3.54E-02	6.09E-03	5.05E-03	1.69E-02	1.14E-02	3.29E-03	3.41E-03	3.86E-03	4.24E-03	5.17E-02	3.23E-03
Peak sensitivity value	6.96E-03	4.67E-03	1.74E+00	8.10E-03	7.63E-02	3.30E-16	-8.06E-02	-1.82E-01	-1.44E-02	-4.30E-02	-7.33E-17	-1.56E+00
Shapiro-Wilk score	3.624E-01	5.424E-01	8.160E-01	9.503E-01	7.015E-01	9.414E-01	9.887E-01	9.975E-01	9.892E-01	9.753E-01	9.411E-01	9.849E-01
**(Acetaldehyde out)**												
Local	2.51E-02	1.08E-02	1.96E+00	8.63E-03	1.04E-01	-5.92E-15	-8.18E-02	-1.79E-01	-1.53E-02	-4.63E-02	4.56E-21	-1.64E+00
Min	1.57E-03	9.36E-04	9.96E-02	3.87E-03	4.34E-02	-3.49E-14	-2.03E-01	-3.35E-01	-3.02E-02	-1.18E-01	-1.61E-13	-2.41E+00
Max	2.20E+00	3.50E-01	2.75E+00	2.34E-02	8.87E-01	4.19E-14	-1.89E-02	-5.21E-02	-5.93E-03	-9.35E-03	1.59E-13	-1.12E+00
Normalized peak height	1.02E-01	3.14E-02	6.09E-03	4.98E-03	1.31E-02	4.50E-02	3.74E-03	3.28E-03	3.93E-03	4.20E-03	2.85E-01	3.14E-03
Peak sensitivity value	7.07E-03	4.25E-03	1.78E+00	7.44E-03	8.18E-02	-1.21E-17	-7.34E-02	-1.77E-01	-1.39E-02	-4.30E-02	6.39E-17	-1.53E+00
Shapiro-Wilk score	3.566E-01	5.483E-01	8.143E-01	9.519E-01	7.177E-01	9.295E-01	9.885E-01	9.974E-01	9.890E-01	9.752E-01	9.503E-01	9.844E-01
**(Acetaldehyde flow)**												
Local	2.55E-02	1.10E-02	1.99E+00	8.78E-03	1.06E-01	-5.73E-15	-8.32E-02	-1.82E-01	-1.56E-02	-4.71E-02	-8.63E-15	-1.67E+00
Min	1.79E-03	1.14E-03	8.60E-02	3.90E-03	4.69E-02	-3.59E-14	-1.82E-01	-3.42E-01	-3.03E-02	-1.17E-01	-1.42E-13	-2.50E+00
Max	2.23E+00	4.50E-01	2.75E+00	2.79E-02	9.67E-01	3.36E-14	-2.09E-02	-6.15E-02	-5.53E-03	-1.16E-02	9.18E-14	-1.13E+00
Normalized peak height	1.02E-01	3.91E-02	5.97E-03	5.67E-03	1.42E-02	1.37E-02	3.13E-03	3.40E-03	3.91E-03	3.93E-03	7.02E-02	3.57E-03
Peak sensitivity value	7.37E-03	4.50E-03	1.84E+00	8.05E-03	8.51E-02	-1.94E-16	-7.69E-02	-1.82E-01	-1.47E-02	-4.06E-02	1.02E-16	-1.53E+00
Shapiro-Wilk score	3.596E-01	5.323E-01	8.266E-01	9.451E-01	7.081E-01	9.249E-01	9.884E-01	9.975E-01	9.882E-01	9.731E-01	9.248E-01	9.836E-01
**(Cyanide-Acetaldehyde flow)**												
Local	2.36E-02	1.02E-02	1.85E+00	8.13E-03	9.84E-02	-5.30E-15	-7.71E-02	-1.69E-01	-1.45E-02	-4.36E-02	-8.00E-15	-1.54E+00
Min	1.75E-03	1.08E-03	1.04E-01	3.52E-03	4.30E-02	-3.02E-14	-1.86E-01	-3.09E-01	-2.93E-02	-1.15E-01	-1.00E-13	-2.17E+00
Max	1.91E+00	3.40E-01	2.43E+00	2.40E-02	9.23E-01	3.47E-14	-1.49E-02	-5.23E-02	-4.64E-03	-9.11E-03	1.31E-13	-1.05E+00
Normalized peak height	9.43E-02	3.34E-02	6.48E-03	5.43E-03	1.56E-02	1.53E-02	3.65E-03	3.27E-03	4.37E-03	4.16E-03	4.53E-02	3.21E-03
Peak sensitivity value	6.52E-03	4.30E-03	1.71E+00	7.55E-03	7.60E-02	-2.84E-17	-7.06E-02	-1.62E-01	-1.34E-02	-3.95E-02	-8.66E-17	-1.50E+00
Shapiro-Wilk score	3.580E-01	5.391E-01	7.678E-01	9.531E-01	7.246E-01	9.253E-01	9.895E-01	9.973E-01	9.905E-01	9.748E-01	9.225E-01	9.883E-01
**(Cyanide flow)**												
Local	2.36E-02	1.02E-02	1.85E+00	8.13E-03	9.84E-02	-5.36E-15	-7.71E-02	-1.69E-01	-1.45E-02	-4.36E-02	-7.92E-15	-1.54E+00
Min	1.88E-03	1.15E-03	7.44E-02	3.68E-03	4.38E-02	-3.96E-14	-1.94E-01	-3.05E-01	-2.92E-02	-1.09E-01	-7.90E-14	-2.19E+00
Max	1.96E+00	3.87E-01	2.43E+00	2.11E-02	7.99E-01	4.12E-14	-1.54E-02	-5.67E-02	-4.65E-03	-7.60E-03	8.56E-14	-1.05E+00
Normalized peak height	9.65E-02	3.65E-02	6.95E-03	4.55E-03	1.33E-02	1.76E-02	3.72E-03	3.11E-03	3.97E-03	4.03E-03	7.15E-02	3.50E-03
Peak sensitivity value	6.78E-03	4.05E-03	1.77E+00	6.97E-03	7.51E-02	6.76E-17	-6.98E-02	-1.62E-01	-1.32E-02	-3.83E-02	-7.39E-17	-1.48E+00
Shapiro-Wilk score	3.620E-01	5.443E-01	7.659E-01	9.548E-01	7.207E-01	9.242E-01	9.889E-01	9.974E-01	9.889E-01	9.772E-01	9.291E-01	9.887E-01

**(Phosphoenolpyruvate synthesis)**												
Local	0.00E+00	0.00E+00	-1.78E-03	-2.42E-15	1.16E-15	-4.28E-04	-2.23E-03	-6.32E-04	-5.04E-05	-2.40E-02	5.89E-02	2.91E-18
Min	-3.04E-14	-2.25E-14	-1.45E-02	-2.86E-14	-1.66E-14	-3.53E-03	-1.37E-02	-3.53E-03	-6.30E-04	-5.07E-02	2.18E-02	-4.14E-17
Max	4.46E-14	2.44E-14	7.99E-04	2.96E-14	1.48E-14	1.88E-04	8.62E-04	2.24E-04	3.20E-05	6.84E-02	9.85E-02	3.20E-16
Normalized peak height	7.39E-01	7.49E-01	1.15E-02	8.06E-03	8.90E-03	1.11E-02	9.45E-03	9.36E-03	1.28E-02	1.15E-02	3.35E-03	3.17E-01
Peak sensitivity value	-5.02E-18	1.45E-17	-1.47E-03	-1.90E-18	4.64E-17	-3.74E-04	-1.92E-03	-6.12E-04	-3.06E-05	-2.43E-02	5.84E-02	2.60E-20
Shapiro-Wilk score	9.791E-02	1.310E-01	8.511E-01	9.788E-01	9.749E-01	8.659E-01	8.548E-01	8.880E-01	7.891E-01	8.076E-01	9.990E-01	8.083E-01
**(Pyruvate kinase)**												
Local	0.00E+00	0.00E+00	-1.78E-03	4.02E-15	-8.26E-16	-4.28E-04	-2.23E-03	-6.32E-04	-5.04E-05	-2.40E-02	5.89E-02	2.91E-18
Min	-4.31E-14	-1.67E-14	-1.30E-02	-2.36E-14	-1.61E-14	-3.42E-03	-1.92E-02	-4.15E-03	-6.34E-04	-5.24E-02	1.67E-02	-8.06E-17
Max	2.22E-14	1.32E-14	7.37E-04	2.79E-14	1.74E-14	2.17E-04	8.74E-04	2.61E-04	2.27E-05	6.70E-02	9.69E-02	2.09E-16
Normalized peak height	7.31E-01	7.47E-01	1.03E-02	8.84E-03	1.04E-02	1.10E-02	1.33E-02	1.05E-02	1.30E-02	1.15E-02	3.56E-03	3.15E-01
Peak sensitivity value	8.81E-18	1.02E-17	-1.51E-03	-1.49E-16	-5.85E-18	-3.85E-04	-1.90E-03	-5.00E-04	-2.95E-05	-2.48E-02	5.92E-02	5.89E-20
Shapiro-Wilk score	1.325E-01	1.838E-01	8.414E-01	9.764E-01	9.699E-01	8.484E-01	8.373E-01	8.709E-01	7.684E-01	8.033E-01	9.992E-01	8.692E-01
**(Pyruvate decarboxylase)**												
Local	0.00E+00	0.00E+00	-1.78E-03	-6.65E-16	1.91E-15	-4.28E-04	-2.23E-03	-6.32E-04	-5.04E-05	-2.40E-02	5.89E-02	2.91E-18
Min	-4.21E-15	-1.93E-14	-1.50E-02	-1.80E-14	-1.68E-14	-3.72E-03	-1.72E-02	-3.91E-03	-7.13E-04	-5.19E-02	1.66E-02	-7.03E-17
Max	6.12E-15	1.90E-14	3.96E-04	1.74E-14	2.19E-14	1.21E-04	5.72E-04	1.37E-04	1.53E-05	6.96E-02	1.00E-01	8.78E-17
Normalized peak height	6.65E-01	7.45E-01	1.14E-02	1.06E-02	3.00E-02	1.17E-02	1.15E-02	9.50E-03	1.43E-02	1.18E-02	3.78E-03	3.13E-01
Peak sensitivity value	-2.72E-20	-9.51E-18	-1.59E-03	1.24E-17	-8.45E-18	-3.85E-04	-2.02E-03	-5.54E-04	-2.88E-05	-2.50E-02	5.94E-02	-2.76E-20
Shapiro-Wilk score	6.578E-01	2.142E-01	8.404E-01	9.799E-01	9.642E-01	8.612E-01	8.435E-01	8.745E-01	7.757E-01	8.089E-01	9.990E-01	9.179E-01
**(Alcohol dehydrogenase)**												
Local	0.00E+00	0.00E+00	-3.13E-02	-1.84E-16	1.26E-15	-7.52E-03	-3.91E-02	-1.11E-02	-8.85E-04	-1.89E-02	5.21E-02	2.13E-18
Min	-2.34E-15	-7.59E-15	-8.50E-02	-7.94E-15	-8.38E-15	-1.78E-02	-7.19E-02	-1.96E-02	-3.77E-03	-4.31E-02	1.37E-02	-2.15E-17
Max	3.01E-15	1.47E-14	-9.13E-03	9.16E-15	1.05E-14	-2.61E-03	-1.85E-02	-5.57E-03	-1.38E-04	6.01E-02	9.20E-02	8.13E-17
Normalized peak height	6.63E-01	7.29E-01	4.36E-03	6.86E-03	1.58E-02	3.59E-03	2.91E-03	3.12E-03	4.53E-03	9.84E-03	3.29E-03	3.16E-01
Peak sensitivity value	1.54E-18	1.02E-17	-3.12E-02	4.27E-19	1.38E-17	-6.43E-03	-3.32E-02	-1.05E-02	-5.72E-04	-2.10E-02	5.15E-02	1.03E-21
Shapiro-Wilk score	6.719E-01	2.871E-01	9.695E-01	9.835E-01	9.736E-01	9.752E-01	9.807E-01	9.875E-01	9.067E-01	8.668E-01	9.989E-01	9.108E-01
**(Ethanol out)**												
Local	2.37E-16	-8.88E-16	-3.13E-02	-1.07E-16	1.31E-15	-7.52E-03	-3.91E-02	-1.11E-02	-8.85E-04	-1.89E-02	5.21E-02	2.13E-18
Min	-3.33E-15	-1.15E-14	-8.12E-02	-1.10E-14	-8.63E-15	-1.77E-02	-7.33E-02	-2.00E-02	-4.39E-03	-4.42E-02	1.48E-02	-7.35E-17
Max	2.78E-15	7.55E-15	-9.18E-03	1.21E-14	6.91E-15	-2.66E-03	-1.82E-02	-4.86E-03	-1.14E-04	7.07E-02	9.13E-02	1.52E-16
Normalized peak height	7.12E-02	5.02E-01	3.97E-03	9.38E-03	1.69E-02	3.73E-03	3.10E-03	3.25E-03	5.46E-03	1.03E-02	3.10E-03	3.17E-01
Peak sensitivity value	-2.21E-16	-2.88E-18	-2.79E-02	-1.67E-18	-1.49E-18	-6.23E-03	-3.40E-02	-1.00E-02	-6.59E-04	-2.00E-02	5.29E-02	4.76E-20
Shapiro-Wilk score	9.984E-01	8.769E-01	9.708E-01	9.800E-01	9.723E-01	9.743E-01	9.812E-01	9.884E-01	9.076E-01	8.677E-01	9.989E-01	8.755E-01
**(Ethanol flow)**												
Local	-3.41E-16	-1.11E-15	-3.13E-02	-1.73E-16	1.29E-15	-7.52E-03	-3.91E-02	-1.11E-02	-8.85E-04	-1.89E-02	5.21E-02	2.13E-18
Min	-1.43E-15	-5.77E-15	-8.79E-02	-8.83E-15	-7.48E-15	-1.82E-02	-7.42E-02	-1.96E-02	-3.69E-03	-4.49E-02	1.42E-02	-4.11E-17
Max	2.38E-15	5.44E-15	-9.00E-03	9.64E-15	9.08E-15	-2.60E-03	-1.85E-02	-5.25E-03	-1.49E-04	6.18E-02	9.01E-02	1.78E-16
Normalized peak height	5.38E-01	1.24E-01	4.28E-03	7.09E-03	1.36E-02	3.73E-03	3.18E-03	3.32E-03	4.63E-03	9.37E-03	3.18E-03	3.17E-01
Peak sensitivity value	-1.09E-18	-2.17E-16	-2.66E-02	-7.61E-18	-3.19E-18	-7.21E-03	-3.35E-02	-1.09E-02	-6.07E-04	-1.99E-02	5.18E-02	-5.40E-20
Shapiro-Wilk score	8.960E-01	9.922E-01	9.721E-01	9.816E-01	9.745E-01	9.767E-01	9.818E-01	9.877E-01	9.078E-01	8.623E-01	9.990E-01	8.797E-01
**(Glycerol synthesis)**												
Local	0.00E+00	0.00E+00	2.79E-01	8.97E-15	-6.51E-15	6.70E-02	3.48E-01	9.89E-02	7.89E-03	-7.27E-02	1.24E-01	1.03E-17
Min	-3.51E-14	-1.90E-13	8.35E-02	-2.06E-13	-2.03E-13	2.33E-02	1.63E-01	3.62E-02	2.16E-03	-1.30E-01	4.08E-02	-2.53E-16
Max	8.86E-14	5.35E-13	4.99E-01	2.08E-13	1.55E-13	1.32E-01	5.12E-01	1.88E-01	1.93E-02	1.52E-01	2.04E-01	8.76E-16
Normalized peak height	6.71E-01	8.14E-01	3.51E-03	1.02E-02	2.41E-02	3.49E-03	3.70E-03	3.53E-03	3.51E-03	1.21E-02	3.84E-03	3.11E-01
Peak sensitivity value	-7.16E-19	-1.81E-16	2.73E-01	-2.02E-16	1.42E-16	6.72E-02	3.59E-01	9.62E-02	6.28E-03	-7.13E-02	1.22E-01	-2.39E-19
Shapiro-Wilk score	1.793E-01	3.199E-02	9.990E-01	9.825E-01	9.747E-01	9.913E-01	9.990E-01	9.974E-01	9.671E-01	8.165E-01	9.972E-01	7.984E-01
**(Glycerol out)**												
Local	0.00E+00	0.00E+00	2.79E-01	9.24E-15	-6.55E-15	6.70E-02	3.48E-01	9.89E-02	7.89E-03	-7.27E-02	1.24E-01	1.03E-17
Min	-4.91E-14	-3.35E-13	8.01E-02	-2.32E-13	-2.35E-13	2.43E-02	1.38E-01	2.93E-02	2.13E-03	-1.30E-01	4.31E-02	-2.23E-16
Max	4.90E-14	1.43E-13	5.03E-01	2.32E-13	1.62E-13	1.38E-01	5.06E-01	1.88E-01	2.22E-02	2.13E-01	2.05E-01	3.80E-15
Normalized peak height	6.58E-01	7.56E-01	3.24E-03	1.32E-02	2.63E-02	3.58E-03	3.81E-03	3.85E-03	4.21E-03	1.48E-02	3.83E-03	3.15E-01
Peak sensitivity value	-2.65E-17	-2.42E-17	2.87E-01	1.14E-16	1.98E-16	6.17E-02	3.54E-01	9.76E-02	6.85E-03	-7.11E-02	1.15E-01	9.29E-19
Shapiro-Wilk score	1.849E-01	3.280E-02	9.988E-01	9.802E-01	9.714E-01	9.913E-01	9.985E-01	9.975E-01	9.702E-01	8.124E-01	9.975E-01	3.702E-01
**(Glycerol flow)**												
Local	0.00E+00	0.00E+00	2.79E-01	9.72E-15	-6.66E-15	6.70E-02	3.48E-01	9.89E-02	7.89E-03	-7.27E-02	1.24E-01	1.03E-17
Min	-1.15E-13	-1.21E-12	8.71E-02	-1.90E-13	-2.24E-13	2.24E-02	1.64E-01	3.66E-02	2.49E-03	-1.31E-01	3.71E-02	-1.47E-16
Max	6.22E-14	1.91E-13	4.86E-01	3.26E-13	1.93E-13	1.30E-01	5.20E-01	1.81E-01	2.01E-02	1.27E-01	2.03E-01	5.65E-16
Normalized peak height	6.69E-01	8.97E-01	3.23E-03	1.25E-02	2.90E-02	3.56E-03	3.56E-03	3.39E-03	3.59E-03	1.12E-02	3.95E-03	3.13E-01
Peak sensitivity value	2.63E-18	-2.68E-16	2.79E-01	-1.53E-16	1.87E-16	6.71E-02	3.42E-01	9.70E-02	6.85E-03	-7.22E-02	1.20E-01	-2.76E-19
Shapiro-Wilk score	1.745E-01	1.463E-02	9.989E-01	9.820E-01	9.714E-01	9.910E-01	9.986E-01	9.974E-01	9.682E-01	8.220E-01	9.975E-01	8.986E-01
**(Acetaldehyde out)**												
Local	0.00E+00	0.00E+00	2.79E-01	-5.24E-15	8.08E-15	6.70E-02	3.48E-01	9.89E-02	7.89E-03	-7.27E-02	1.24E-01	1.03E-17
Min	-4.10E-14	-1.71E-13	8.46E-02	-1.11E-13	-8.71E-14	2.42E-02	1.66E-01	3.68E-02	2.33E-03	-1.32E-01	4.55E-02	-2.52E-16
Max	4.01E-14	1.21E-13	4.83E-01	1.08E-13	9.21E-14	1.35E-01	5.33E-01	1.82E-01	2.03E-02	1.05E-01	2.00E-01	7.98E-16
Normalized peak height	6.67E-01	7.37E-01	3.08E-03	7.70E-03	1.52E-02	3.62E-03	3.74E-03	3.43E-03	3.79E-03	1.04E-02	3.47E-03	3.15E-01
Peak sensitivity value	1.30E-17	5.23E-17	2.75E-01	2.27E-17	8.56E-17	6.24E-02	3.56E-01	1.00E-01	6.05E-03	-6.89E-02	1.22E-01	-4.75E-19
Shapiro-Wilk score	2.123E-01	5.302E-02	9.989E-01	9.884E-01	9.825E-01	9.910E-01	9.989E-01	9.978E-01	9.681E-01	8.281E-01	9.966E-01	8.400E-01
**(Acetaldehyde flow)**												
Local	0.00E+00	0.00E+00	2.84E-01	-5.10E-15	7.94E-15	6.82E-02	5.75E-01	-1.21E-01	-9.62E-03	-7.39E-02	1.27E-01	1.05E-17
Min	-4.59E-14	-2.07E-13	8.63E-02	-1.02E-13	-7.95E-14	2.72E-02	3.49E-01	-1.93E-01	-3.94E-02	-1.30E-01	5.10E-02	-1.89E-16
Max	4.26E-14	1.72E-13	5.09E-01	1.25E-13	9.76E-14	1.35E-01	7.43E-01	-6.64E-02	-1.54E-03	1.39E-01	2.12E-01	2.18E-15
Normalized peak height	6.66E-01	7.57E-01	3.51E-03	8.76E-03	1.87E-02	3.32E-03	3.74E-03	3.36E-03	5.15E-03	1.19E-02	3.74E-03	3.18E-01
Peak sensitivity value	-8.05E-18	-6.59E-17	2.76E-01	-1.22E-16	-8.11E-17	6.37E-02	5.70E-01	-1.20E-01	-6.71E-03	-7.19E-02	1.19E-01	-4.39E-19
Shapiro-Wilk score	2.877E-01	4.467E-02	9.990E-01	9.882E-01	9.803E-01	9.910E-01	9.948E-01	9.950E-01	9.147E-01	8.104E-01	9.970E-01	6.059E-01
**(Cyanide-Acetaldehyde flow)**												
Local	0.00E+00	0.00E+00	2.63E-01	-4.73E-15	7.34E-15	6.31E-02	-3.93E-01	8.14E-01	6.50E-02	-6.85E-02	1.17E-01	9.70E-18
Min	-3.00E-14	-1.73E-13	6.71E-02	-1.14E-13	-8.43E-14	2.14E-02	-5.59E-01	6.56E-01	2.13E-02	-1.22E-01	3.49E-02	-2.74E-16
Max	5.00E-14	1.46E-13	4.68E-01	1.06E-13	8.11E-14	1.31E-01	-2.44E-01	9.06E-01	1.89E-01	1.32E-01	1.90E-01	5.99E-16
Normalized peak height	6.65E-01	7.57E-01	3.31E-03	1.02E-02	2.14E-02	3.63E-03	3.38E-03	3.76E-03	4.37E-03	1.18E-02	3.99E-03	3.15E-01
Peak sensitivity value	1.89E-17	-1.23E-16	2.67E-01	2.69E-17	-3.84E-17	6.08E-02	-3.95E-01	8.18E-01	5.83E-02	-6.71E-02	1.12E-01	3.06E-19
Shapiro-Wilk score	2.396E-01	3.994E-02	9.987E-01	9.893E-01	9.804E-01	9.918E-01	9.989E-01	9.861E-01	9.502E-01	8.112E-01	9.982E-01	8.731E-01
**(Cyanide flow)**												
Local	0.00E+00	0.00E+00	2.63E-01	-4.59E-15	7.47E-15	6.31E-02	-3.93E-01	8.14E-01	6.50E-02	-6.85E-02	1.17E-01	9.70E-18
Min	-1.78E-14	-1.01E-13	7.00E-02	-1.15E-13	-7.98E-14	2.04E-02	-5.72E-01	6.56E-01	2.12E-02	-1.33E-01	3.70E-02	-2.09E-16
Max	2.67E-14	1.99E-13	4.54E-01	1.05E-13	9.59E-14	1.33E-01	-2.40E-01	9.00E-01	1.81E-01	1.30E-01	1.85E-01	5.80E-16
Normalized peak height	6.50E-01	7.36E-01	3.15E-03	9.99E-03	1.50E-02	3.46E-03	3.65E-03	3.71E-03	4.16E-03	1.23E-02	3.51E-03	3.16E-01
Peak sensitivity value	-6.29E-18	1.02E-17	2.52E-01	-1.56E-16	7.82E-17	6.38E-02	-3.97E-01	8.26E-01	5.38E-02	-6.91E-02	1.19E-01	-6.95E-21
Shapiro-Wilk score	3.840E-01	4.908E-02	9.985E-01	9.862E-01	9.760E-01	9.910E-01	9.986E-01	9.866E-01	9.501E-01	8.124E-01	9.983E-01	8.386E-01

(Storage)																								
Local	1.21E-02	5.20E-03	9.43E-01	-1.53E-03	-1.85E-02	-1.69E-15	9.90E-03	2.16E-02	1.86E-03	5.60E-03	-3.99E-15	1.98E-01	0.00E+00	0.00E+00	-3.37E-02	-2.74E-15	-1.38E-16	-8.11E-03	-4.21E-02	-1.20E-02	-9.55E-04	1.62E-02	-9.65E-02	-1.73E-18
Min	1.15E-03	6.67E-04	4.55E-02	-5.16E-03	-1.63E-01	-1.22E-14	2.73E-03	6.13E-03	5.07E-04	9.17E-04	-2.75E-14	6.82E-02	-1.09E-14	-4.24E-14	-8.86E-02	-6.22E-14	-5.36E-14	-1.82E-02	-7.56E-02	-2.13E-02	-4.13E-03	-2.94E-03	-1.85E-01	-8.96E-16
Max	8.64E-01	1.81E-01	1.09E+00	2.46E-03	6.45E-02	1.28E-14	2.34E-02	5.89E-02	5.23E-03	2.24E-02	4.03E-14	5.13E-01	8.63E-15	2.80E-14	-1.04E-02	8.56E-14	4.16E-14	-2.88E-03	-2.07E-02	-5.69E-03	-1.44E-04	1.09E-01	-5.15E-02	2.41E-17
Normalized peak height	8.29E-02	3.22E-02	7.46E-03	5.82E-03	1.36E-02	3.94E-02	3.63E-03	4.33E-03	4.05E-03	5.61E-03	1.77E-02	3.53E-03	6.45E-01	7.10E-01	4.16E-03	1.13E-02	1.15E-02	3.40E-03	3.01E-03	3.17E-03	4.68E-03	9.67E-03	3.40E-03	3.37E-01
Peak sensitivity value	3.31E-03	2.38E-03	9.87E-01	-1.59E-03	-1.53E-02	-1.72E-19	8.40E-03	1.95E-02	1.76E-03	4.37E-03	-1.65E-17	1.42E-01	-1.23E-18	1.68E-17	-3.05E-02	-9.41E-16	2.53E-16	-6.92E-03	-3.73E-02	-1.13E-02	-5.92E-04	1.65E-02	-9.89E-02	-2.90E-19
Shapiro-Wilk score	3.647E-01	5.527E-01	6.766E-01	9.896E-01	9.148E-01	9.486E-01	9.741E-01	9.780E-01	9.613E-01	9.434E-01	9.708E-01	9.355E-01	6.177E-01	1.926E-01	9.734E-01	9.737E-01	9.639E-01	9.759E-01	9.821E-01	9.880E-01	9.142E-01	8.426E-01	9.915E-01	2.689E-01
(ATP consumption)																								
Local	-1.59E-03	-6.83E-04	-1.24E-01	2.57E-02	3.11E-01	-8.56E-16	4.80E-03	1.05E-02	9.01E-04	2.72E-03	-2.17E-15	9.61E-02	0.00E+00	0.00E+00	-1.64E-02	-1.37E-15	-6.97E-17	-3.93E-03	-2.04E-02	-5.81E-03	-4.63E-04	-2.32E-01	9.53E-01	2.55E-17
Min	-1.02E+00	-1.53E-01	-3.09E+00	1.34E-02	1.47E-01	-1.09E-14	1.18E-03	2.20E-03	2.29E-04	4.12E-04	-2.29E-14	2.47E-02	-4.14E-15	-2.35E-14	-1.22E-01	-7.39E-14	-4.45E-14	-3.33E-02	-1.56E-01	-3.36E-02	-7.02E-03	-5.07E-01	8.26E-01	-2.70E-16
Max	5.89E-04	3.81E-04	4.33E-02	6.24E-02	2.92E+00	1.03E-14	3.02E-02	8.30E-02	7.63E-03	2.32E-02	2.76E-14	9.84E-01	7.37E-15	1.97E-14	-4.19E-03	4.87E-14	3.73E-14	-1.15E-03	-7.14E-03	-2.21E-03	-6.18E-05	-1.57E-01	9.73E-01	2.51E-15
Normalized peak height	3.68E-01	1.83E-01	1.76E-02	4.31E-03	1.49E-02	8.79E-02	8.13E-03	1.03E-02	1.07E-02	1.01E-02	3.17E-02	1.23E-02	6.48E-01	7.15E-01	9.68E-03	1.81E-02	2.01E-02	1.15E-02	1.11E-02	8.70E-03	1.53E-02	5.59E-03	6.79E-03	3.16E-01
Peak sensitivity value	7.59E-05	-1.56E-04	-3.99E-02	2.34E-02	2.40E-01	9.62E-18	3.63E-03	8.06E-03	6.85E-04	2.04E-03	-4.78E-18	6.64E-02	-1.80E-18	9.41E-18	-1.31E-02	2.15E-16	-3.13E-17	-3.42E-03	-1.79E-02	-4.90E-03	-2.81E-04	-2.29E-01	9.54E-01	-1.13E-18
Shapiro-Wilk score	2.700E-01	3.513E-01	7.035E-01	9.607E-01	7.257E-01	8.969E-01	8.460E-01	8.359E-01	8.339E-01	8.686E-01	9.316E-01	7.833E-01	5.199E-01	1.671E-01	8.404E-01	9.204E-01	9.089E-01	8.519E-01	8.340E-01	8.585E-01	7.779E-01	9.523E-01	9.132E-01	7.947E-01
(Adenylate kinase)																								
Local	-INF	-INF	-INF	-INF	-INF	-INF	-INF	-INF	-INF	-INF	-INF	-INF	-INF	-INF	-INF	-INF	-INF	-INF	-INF	-INF	-INF	-INF	-INF	-INF
Min	-INF	-INF	-INF	-INF	-INF	-INF	-INF	-INF	-INF	-INF	-INF	-INF	-INF	-INF	-INF	-INF	-INF	-INF	-INF	-INF	-INF	-INF	-INF	-INF
Max	INF	INF	INF	INF	INF	INF	INF	INF	INF	INF	INF	INF	INF	INF	INF	INF	INF	INF	INF	INF	INF	INF	INF	INF
Normalized peak height	NA	NA	NA	NA	NA	NA	NA	NA	NA	NA	NA	NA	NA	NA	NA	NA	NA	NA	NA	NA	NA	NA	NA	NA
Peak sensitivity value	NAN	NAN	NAN	NAN	NAN	NAN	NAN	NAN	NAN	NAN	NAN	NAN	NAN	NAN	NAN	NAN	NAN	NAN	NAN	NAN	NAN	NAN	NAN	NAN
Shapiro-Wilk score	NA	NA	NA	NA	NA	NA	NA	NA	NA	NA	NA	NA	NA	NA	NA	NA	NA	NA	NA	NA	NA	NA	NA	NA

Table S28: Flux control coefficients for *Saccharomyces cerevisiae* model with parameter variation of ±30%

	(GlucoseMixedflowtoextracellularmedium)	(Glucoseuptake)	(Hexokinase)	(Phosphoglucoisomerase)	(Phosphofructokinase)	(Aldolase)	(Triosephosphateisomerase)	(Glyceraldehyde3-phosphatedehydrogenase)	(Phosphoenolpyruvatesynthesis)	(Pyruvatekinase)	(Pyruvatedecarboxylase)	(Alcoholdehydrogenase)	(Ethanolout)	(Ethanolflow)	(Glycerolsynthesis)	(Glycerolout)	(Glycerolflow)	(Acetaldehydeout)	(Acetaldehydeflow)	(Cyanide-Acetaldehydeflow)	(Cyanideflow)	(Storage)	(ATPconsumption)	(Adenylatekinase)
(Glucose Mixed flow to extracellular medium)																								
Local	1.25E-02	5.36E-03	9.73E-01	1.37E-03	1.65E-02	-3.45E-17	2.30E-04	5.02E-04	4.31E-05	1.30E-04	-8.14E-17	4.60E-03	0.00E+00	0.00E+00	-7.83E-04	-6.35E-17	-5.80E-18	-1.88E-04	-9.78E-04	-2.78E-04	-2.22E-05	-9.81E-03	-2.24E-03	1.36E-18
Min	5.22E-04	3.42E-04	-1.24E-02	2.01E-04	2.20E-03	-4.99E-15	-1.66E-03	-3.58E-03	-4.65E-04	-2.54E-03	-1.13E-14	-6.69E-02	-3.62E-15	-7.82E-15	-4.80E-02	-2.44E-14	-3.34E-14	-9.38E-03	-3.89E-02	-9.92E-03	-2.31E-03	-6.16E-02	-3.60E-02	-3.42E-17
Max	9.73E-01	4.56E-01	1.00E+00	1.75E-02	7.88E-01	6.43E-15	1.42E-02	3.08E-02	2.65E-03	5.39E-03	1.08E-14	2.40E-01	1.02E-15	1.51E-14	5.97E-03	6.52E-14	2.83E-14	1.67E-03	6.86E-03	1.96E-03	5.15E-04	2.51E-01	2.18E-02	1.01E-16
Normalized peak height	9.70E-02	6.84E-02	2.95E-02	1.29E-02	4.39E-02	2.24E-01	5.72E-02	5.42E-02	6.02E-02	5.04E-02	2.67E-01	5.60E-02	5.80E-01	8.59E-01	5.62E-02	3.25E-01	3.24E-01	4.52E-02	3.87E-02	3.70E-02	1.00E-01	2.76E-02	2.31E-02	2.87E-01
Peak sensitivity value	1.98E-03	1.48E-03	9.93E-01	1.06E-03	1.05E-02	-3.67E-18	1.40E-04	3.20E-04	2.25E-05	6.12E-05	-1.89E-18	1.94E-03	-2.09E-18	-5.14E-19	-4.81E-04	-2.38E-18	1.40E-17	-1.18E-04	-6.68E-04	-2.07E-04	-6.57E-06	-8.64E-03	-1.96E-03	4.97E-20
Shapiro-Wilk score	4.890E-01	5.105E-01	5.781E-01	7.361E-01	3.547E-01	3.158E-01	3.999E-01	4.011E-01	3.929E-01	5.347E-01	4.076E-01	3.732E-01	2.527E-01	1.348E-01	4.149E-01	2.975E-01	2.700E-01	4.320E-01	4.278E-01	4.480E-01	3.423E-01	6.339E-01	6.755E-01	8.665E-01
(Glucose uptake)																								
Local	1.25E-02	5.36E-03	9.73E-01	1.37E-03	1.65E-02	-2.20E-17	2.30E-04	5.02E-04	4.31E-05	1.30E-04	-8.36E-17	4.60E-03	0.00E+00	0.00E+00	-7.83E-04	-6.57E-17	-3.32E-18	-1.88E-04	-9.78E-04	-2.78E-04	-2.22E-05	-9.81E-03	-2.24E-03	1.36E-18
Min	4.87E-04	3.22E-04	-1.04E-02	2.21E-04	2.38E-03	-5.49E-15	-2.63E-03	-4.27E-03	-4.03E-04	-1.86E-03	-8.10E-15	-5.26E-02	-3.92E-15	-7.54E-15	-4.24E-02	-2.58E-14	-2.09E-14	-1.01E-02	-3.63E-02	-9.38E-03	-2.37E-03	-5.36E-02	-3.24E-02	-1.88E-17
Max	9.76E-01	4.53E-01	1.00E+00	1.96E-02	8.18E-01	7.97E-15	1.38E-02	2.70E-02	3.61E-03	6.64E-03	1.04E-14	2.38E-01	4.43E-15	1.31E-14	7.87E-03	2.67E-14	2.44E-14	1.56E-03	7.89E-03	2.07E-03	4.11E-04	2.39E-01	1.59E-02	1.27E-16
Normalized peak height	9.98E-02	6.81E-02	3.03E-02	1.43E-02	4.79E-02	1.24E-01	6.03E-02	5.11E-02	7.78E-02	5.47E-02	1.67E-01	5.60E-02	6.64E-01	8.40E-01	5.30E-02	2.02E-01	2.42E-01	4.88E-02	3.83E-02	3.54E-02	1.04E-01	2.74E-02	1.82E-02	2.88E-01
Peak sensitivity value	1.95E-03	1.91E-03	9.95E-01	1.18E-03	1.01E-02	-2.09E-18	1.22E-04	2.85E-04	2.06E-05	6.49E-05	-2.14E-19	2.14E-03	-3.63E-19	-2.28E-18	-4.56E-04	-5.01E-18	1.28E-17	-1.05E-04	-7.18E-04	-1.81E-04	-7.83E-06	-8.74E-03	-1.98E-03	4.53E-20
Shapiro-Wilk score	4.864E-01	5.195E-01	5.765E-01	7.342E-01	3.564E-01	4.309E-01	4.020E-01	3.979E-01	3.972E-01	5.366E-01	5.224E-01	3.760E-01	1.870E-01	1.199E-01	4.129E-01	3.462E-01	3.292E-01	4.251E-01	4.289E-01	4.542E-01	3.461E-01	6.425E-01	6.685E-01	8.577E-01
(Hexokinase)																								
Local	1.25E-02	5.36E-03	9.73E-01	1.37E-03	1.65E-02	-4.14E-17	2.30E-04	5.02E-04	4.31E-05	1.30E-04	-1.06E-16	4.60E-03	0.00E+00	0.00E+00	-7.83E-04	-6.57E-17	-3.32E-18	-1.88E-04	-9.78E-04	-2.78E-04	-2.22E-05	-9.81E-03	-2.24E-03	1.36E-18
Min	4.83E-04	3.19E-04	4.87E-03	1.75E-04	2.71E-03	-5.66E-15	-2.70E-03	-5.83E-03	-6.07E-04	-2.87E-03	-2.46E-14	-6.54E-02	-1.04E-15	-4.10E-15	-4.17E-02	-3.93E-14	-2.45E-14	-1.03E-02	-3.93E-02	-1.02E-02	-2.33E-03	-5.34E-02	-3.46E-02	-3.55E-17
Max	9.79E-01	4.33E-01	1.00E+00	1.63E-02	8.18E-01	4.79E-15	1.33E-02	2.72E-02	3.74E-03	5.22E-03	2.04E-14	2.47E-01	1.67E-15	1.25E-14	9.79E-03	2.66E-14	2.08E-14	2.28E-03	8.49E-03	2.38E-03	5.08E-04	2.56E-01	2.07E-02	4.85E-17
Normalized peak height	9.75E-02	6.48E-02	2.88E-02	1.21E-02	4.72E-02	2.00E-01	5.76E-02	5.24E-02	8.29E-02	5.36E-02	3.33E-01	6.11E-02	5.51E-01	7.39E-01	5.19E-02	2.14E-01	2.51E-01	5.14E-02	4.18E-02	3.77E-02	1.04E-01	2.74E-02	2.12E-02	2.89E-01
Peak sensitivity value	1.95E-03	1.83E-03	9.92E-01	1.02E-03	1.05E-02	-3.51E-18	1.15E-04	2.39E-04	2.03E-05	6.88E-05	3.12E-18	2.01E-03	-6.98E-19	-6.57E-18	-4.30E-04	-2.90E-17	1.40E-18	-1.26E-04	-6.19E-04	-2.03E-04	-6.78E-06	-9.36E-03	-2.05E-03	-7.72E-21
Shapiro-Wilk score	4.851E-01	5.088E-01	5.766E-01	7.321E-01	3.574E-01	3.262E-01	3.995E-01	3.974E-01	3.745E-01	5.391E-01	3.418E-01	3.758E-01	2.719E-01	1.381E-01	4.113E-01	3.485E-01	3.519E-01	4.254E-01	4.288E-01	4.514E-01	3.479E-01	6.268E-01	6.716E-01	9.163E-01
(Phosphoglucoisomerase)																								
Local	1.27E-02	5.48E-03	9.94E-01	3.42E-03	4.14E-02	4.62E-16	-6.63E-03	-1.45E-02	-1.24E-03	-3.75E-03	2.63E-15	-1.33E-01	0.00E+00	0.00E+00	2.26E-02	1.83E-15	9.24E-17	5.43E-03	2.82E-02	8.02E-03	6.39E-04	-2.82E-02	6.46E-02	3.55E-18
Min	4.83E-04	2.88E-04	-1.36E-02	4.65E-04	1.49E-02	-1.35E-14	-2.52E-02	-3.68E-02	-4.56E-03	-1.83E-02	-5.42E-14	-3.89E-01	-4.28E-14	-1.61E-13	2.08E-04	-4.45E-14	-5.49E-14	5.50E-05	2.54E-04	7.11E-05	1.05E-05	-8.76E-02	2.14E-04	-5.60E-17
Max	1.11E+00	4.04E-01	1.22E+00	2.08E-02	9.86E-01	1.25E-14	-3.57E-05	-2.06E-04	-1.15E-05	-1.26E-05	6.30E-14	-2.51E-03	3.86E-14	2.08E-13	6.06E-02	5.50E-14	7.32E-14	1.60E-02	5.81E-02	1.74E-02	4.22E-03	2.13E-01	1.27E-01	1.04E-16
Normalized peak height	1.11E-01	6.17E-02	1.13E-02	8.22E-03	2.83E-02	6.19E-02	4.94E-03	3.72E-03	4.81E-03	5.82E-03	1.06E-01	4.12E-03	5.42E-01	7.34E-01	3.60E-03	1.43E-02	2.90E-02	4.33E-03	3.85E-03	4.25E-03	6.47E-03	1.54E-02	3.41E-03	2.87E-01
Peak sensitivity value	2.15E-03	1.70E-03	9.64E-01	3.06E-03	2.99E-02	-5.10E-18	-4.34E-03	-1.17E-02	-8.79E-04	-2.12E-03	3.41E-17	-8.85E-02	1.99E-17	-6.16E-17	1.92E-02	8.53E-18	1.68E-18	4.09E-03	2.42E-02	6.61E-03	3.45E-04	-2.91E-02	6.23E-02	6.28E-20
Shapiro-Wilk score	4.877E-01	5.070E-01	6.398E-01	8.523E-01	4.114E-01	9.696E-01	9.618E-01	9.824E-01	9.500E-01	9.115E-01	8.187E-01	9.353E-01	2.559E-01	1.593E-01	9.779E-01	9.432E-01	9.086E-01	9.751E-01	9.936E-01	9.927E-01	8.778E-01	7.306E-01	9.897E-01	9.426E-01
(Phosphofructokinase)																								
Local	1.27E-02	5.48E-03	9.94E-01	3.42E-03	4.14E-02	1.39E-15	-6.63E-03	-1.45E-02	-1.24E-03	-3.75E-03	3.85E-15	-1.33E-01	0.00E+00	0.00E+00	2.26E-02	2.02E-15	1.13E-16	5.43E-03	2.82E-02	8.02E-03	6.39E-04	-2.82E-02	6.46E-02	3.55E-18
Min	4.86E-04	3.23E-04	-1.07E-02	3.49E-04	1.38E-02	-1.66E-14	-2.54E-02	-4.17E-02	-4.79E-03	-2.33E-02	-7.92E-14	-3.73E-01	-2.73E-14	-1.29E-13	1.74E-04	-5.80E-14	-6.18E-14	5.96E-05	3.51E-04	6.09E-05	5.32E-06	-9.71E-02	1.87E-04	-8.36E-17
Max	1.10E+00	4.45E-01	1.21E+00	2.26E-02	9.89E-01	1.26E-14	-3.54E-05	-1.14E-04	-9.20E-06	-5.59E-06	3.84E-14	-2.59E-03	3.13E-14	1.33E-13	7.08E-02	5.98E-14	4.79E-14	1.59E-02	5.27E-02	1.63E-02	3.94E-03	2.37E-01	1.35E-01	2.34E-16
Normalized peak height	1.12E-01	6.74E-02	1.12E-02	9.01E-03	2.79E-02	2.89E-02	4.96E-03	4.15E-03	5.30E-03	7.40E-03	7.97E-02	4.00E-03	5.08E-01	6.69E-01	4.42E-03	1.79E-02	2.55E-02	4.11E-03	3.54E-03	3.90E-03	6.30E-03	1.73E-02	3.82E-03	2.87E-01
Peak sensitivity value	2.14E-03	1.88E-03	9.55E-01	3.05E-03	3.28E-02	-6.48E-18	-4.84E-03	-1.14E-02	-8.97E-04	-1.74E-03	3.24E-17	-8.68E-02	-2.92E-17	-5.76E-17	1.68E-02	-5.40E-18	5.20E-17	4.47E-03	2.35E-02	7.14E-03	3.18E-04	-2.95E-02	6.51E-02	1.29E-19
Shapiro-Wilk score	4.868E-01	5.106E-01	6.401E-01	8.437E-01	4.102E-01	9.235E-01	9.599E-01	9.815E-01	9.533E-01	9.096E-01	8.623E-01	9.368E-01	2.737E-01	1.698E-01	9.773E-01	9.355E-01	9.011E-01	9.743E-01	9.938E-01	9.922E-01	8.769E-01	7.402E-01	9.893E-01	8.772E-01
(Aldolase)																								
Local	1.27E-02	5.48E-03	9.94E-01	3.42E-03	4.14E-02	1.11E-15	-6.63E-03	-1.45E-02	-1.24E-03	-3.75E-03	-1.02E-14	-1.33E-01	0.00E+00	0.00E+00	2.26E-02	3.98E-16	1.24E-16	5.43E-03	2.82E-02	8.02E-03	6.39E-04	-2.82E-02	6.46E-02	3.55E-18
Min	-1.32E-01	-1.79E-01	1.52E-03	-5.28E-05	-2.26E-03	-2.89E-14	-2.31E-02	-4.21E-02	-4.06E-03	-2.00E-02	-1.14E-13	-4.11E-01	-3.56E-14	-1.34E-13	2.02E-04	-7.13E-14	-5.08E-14	1.01E-04	4.43E-04	9.92E-05	1.43E-05	-1.27E-01	2.45E-04	-4.14E-17
Max	1.10E+00	4.68E-01	1.48E+00	2.42E-02	9.84E-01	3.62E-14	-4.74E-05	-2.01E-04	-1.26E-05	-1.23E-05	9.80E-14	-3.34E-03	5.17E-14	1.25E-13	6.59E-02	5.96E-14	6.55E-14	1.57E-02	5.32E-02	1.68E-02	3.87E-03	2.34E-01	1.28E-01	1.16E-16
Normalized peak height	1.22E-01	9.48E-02	1.39E-02	9.62E-03	2.85E-02	6.00E-02	4.65E-03	4.11E-03	4.28E-03	6.01E-03	8.27E-02	4.45E-03	5.33E-01	6.75E-01	3.99E-03	1.79E-02	2.52E-02	3.99E-03	3.51E-03	4.07E-03	6.05E-03	1.86E-02	3.51E-03	2.88E-01
Peak sensitivity value	2.41E-03	1.93E-03	9.61E-01	2.97E-03	3.18E-02	-2.07E-16	-4.24E-03	-1.30E-02	-8.76E-04	-1.86E-03	8.09E-17	-8.88E-02	-1.32E-17	6.97E-17	1.82E-02	-1.05E-17	-1.22E-17	4.04E-03	2.45E-02	6.90E-03	2.98E-04	-3.01E-02	6.13E-02	-4.16E-20
Shapiro-Wilk score	4.881E-01	5.200E-01	6.389E-01	8.564E-01	4.100E-01	8.740E-01	9.616E-01	9.808E-01	9.515E-01	9.141E-01	9.273E-01	9.346E-01	2.466E-01	1.706E-01	9.764E-01	9.393E-01	8.925E-01	9.753E-01	9.940E-01	9.918E-01	8.725E-01	7.513E-01	9.891E-01	9.300E-01
(Triosephosphate isomerase)																								
Local	1.01E-02	4.37E-03	7.92E-01	2.32E-03	2.81E-02	-1.03E-15	9.18E-03	2.01E-02	1.72E-03	5.20E-03	-5.10E-15	1.84E-01	0.00E+00	0.00E+00	-3.13E-02	-1.10E-17	1.39E-15	-7.52E-03	-3.91E-02	-1.11E-02	-8.85E-04	-1.89E-02	5.21E-02	2.13E-18
Min	4.68E-04	2.58E-04	-1.31E-02	1.00E-04	1.12E-03	-7.91E-15	7.75E-04	2.32E-03	2.45E-04	3.67E-04	-4.24E-14	3.98E-02	-1.56E-14	-4.58E-14	-1.42E-01	-1.95E-14	-1.78E-14	-2.57E-02	-1.08E-01	-2.70E-02	-6.86E-03	-6.97E-02	2.30E-04	-5.56E-17
Max	8.54E-01	3.54E-01	9.21E-01	1.55E-02	7.96E-01	1.10E-14	3.55E-02	9.24E-02	7.82E-03	2.55E-02	4.11E-14	7.27E-01	1.93E-14	6.07E-14	-3.55E-03	3.25E-14	2.00E-14	-1.56E-03	-1.27E-02	-3.69E-03	-5.28E-05	1.91E-01	1.20E-01	1.03E-16
Normalized peak height	1.05E-01	6.41E-02	5.81E-03	8.67E-03	3.16E-02	1.99E-02	4.52E-03	5.46E-03	5.46E-03	5.52E-03	8.32E-02	4.44E-03	5.50E-01	6.35E-01	5.48E-03	1.99E-02	2.27E-02	4.44E-03	3.77E-03	3.48E-03	7.46E-03	1.35E-02	3.33E-03	2.94E-01
Peak sensitivity value	1.75E-03	1.50E-03	8.51E-01	2.12E-03	2.14E-02	8.44E-18	6.80E-03	1.84E-02	1.18E-03	2.91E-03	-3.32E-17	1.07E-01	-1.35E-17	-3.28E-17	-2.71E-02	-1.19E-19	1.46E-17	-6.37E-03	-3.09E-02	-8.88E-03	-3.76E-04	-2.13E-02	5.15E-02	-3.42E-20
Shapiro-Wilk score	4.871E-01	4.973E-01	7.438E-01	8.669E-01	4.235E-01	9.184E-01	9.325E-01	9.409E-01	9.142E-01	8.881E-01	9.341E-01	8.795E-01	3.796E-01	1.939E-01	9.364E-01	9.243E-01	8.934E-01	9.458E-01	9.638E-01	9.735E-01	8.333E-01	7.923E-01	9.981E-01	9.180E-01

**(Glyceraldehyde 3-phosphate dehydrogenase)**												
Local	1.16E-02	4.98E-03	9.03E-01	2.92E-03	3.54E-02	-1.85E-16	5.23E-04	1.14E-03	9.81E-05	2.96E-04	-9.79E-15	1.05E-02
Min	-3.11E-03	-2.40E-03	-1.91E-02	2.12E-04	1.21E-02	-1.68E-14	-1.62E-03	-3.51E-03	-3.51E-04	-1.38E-03	-5.10E-14	-4.51E-02
Max	9.11E-01	4.06E-01	9.49E-01	1.74E-02	8.70E-01	1.57E-14	1.60E-02	3.26E-02	3.26E-03	6.25E-03	5.28E-14	2.74E-01
Normalized peak height	1.02E-01	6.50E-02	1.40E-02	8.21E-03	2.92E-02	2.33E-02	3.07E-02	2.77E-02	3.30E-02	2.32E-02	5.47E-02	2.91E-02
Peak sensitivity value	1.92E-03	1.48E-03	9.02E-01	2.56E-03	2.54E-02	3.79E-17	3.07E-04	7.71E-04	5.91E-05	1.67E-04	9.28E-18	5.43E-03
Shapiro-Wilk score	4.893E-01	5.086E-01	5.991E-01	8.467E-01	4.169E-01	9.122E-01	4.991E-01	4.972E-01	4.912E-01	6.514E-01	9.458E-01	4.641E-01
**(Phosphoenolpyruvate synthesis)**												
Local	1.16E-02	4.98E-03	9.03E-01	2.92E-03	3.54E-02	-2.35E-16	5.23E-04	1.14E-03	9.81E-05	2.96E-04	-4.52E-14	1.05E-02
Min	4.12E-04	3.14E-04	3.76E-04	3.83E-04	1.25E-02	-2.11E-13	-2.46E-03	-4.23E-03	-4.23E-04	-2.20E-03	-6.57E-13	-5.69E-02
Max	9.08E-01	4.10E-01	9.53E-01	1.76E-02	8.73E-01	2.37E-13	1.34E-02	2.91E-02	3.74E-03	6.21E-03	4.35E-13	2.68E-01
Normalized peak height	9.81E-02	6.63E-02	1.47E-02	7.98E-03	2.92E-02	3.28E-02	2.78E-02	2.65E-02	3.91E-02	2.61E-02	6.81E-02	2.88E-02
Peak sensitivity value	1.77E-03	1.75E-03	9.02E-01	3.01E-03	2.75E-02	1.56E-16	3.57E-04	7.95E-04	5.80E-05	1.64E-04	-2.51E-16	5.87E-03
Shapiro-Wilk score	4.862E-01	5.036E-01	5.968E-01	8.502E-01	4.141E-01	8.127E-01	4.931E-01	4.922E-01	4.891E-01	6.436E-01	9.215E-01	4.540E-01
**(Pyruvate kinase)**												
Local	1.16E-02	4.98E-03	9.03E-01	2.92E-03	3.54E-02	2.24E-15	5.23E-04	1.14E-03	9.81E-05	2.96E-04	5.94E-14	1.05E-02
Min	4.60E-04	2.54E-04	-1.31E-02	2.18E-04	1.26E-02	-4.94E-14	-1.71E-03	-3.58E-03	-2.80E-04	-1.23E-03	-3.66E-13	-3.25E-02
Max	9.26E-01	3.64E-01	9.50E-01	1.99E-02	8.78E-01	4.54E-14	1.63E-02	3.22E-02	3.48E-03	5.93E-03	3.17E-13	2.69E-01
Normalized peak height	1.02E-01	5.88E-02	1.38E-02	9.30E-03	3.00E-02	4.93E-02	3.30E-02	2.78E-02	3.64E-02	2.21E-02	2.35E-01	2.76E-02
Peak sensitivity value	1.85E-03	1.53E-03	9.03E-01	2.69E-03	2.60E-02	-2.04E-17	3.58E-04	7.36E-04	5.66E-05	1.86E-04	-2.38E-16	5.72E-03
Shapiro-Wilk score	4.874E-01	5.190E-01	5.971E-01	8.493E-01	4.144E-01	8.789E-01	4.876E-01	4.899E-01	4.735E-01	6.397E-01	8.514E-01	4.549E-01
**(Pyruvate decarboxylase)**												
Local	1.16E-02	4.98E-03	9.03E-01	2.92E-03	3.54E-02	-8.93E-16	5.23E-04	1.14E-03	9.81E-05	2.96E-04	-6.66E-16	1.05E-02
Min	4.92E-04	2.43E-04	-3.40E-05	3.78E-04	1.25E-02	-8.80E-15	-2.17E-03	-3.62E-03	-4.92E-04	-2.11E-03	-3.84E-14	-6.90E-02
Max	9.13E-01	3.88E-01	9.52E-01	2.19E-02	8.93E-01	8.69E-15	1.85E-02	3.07E-02	3.34E-03	5.85E-03	3.50E-14	2.60E-01
Normalized peak height	1.00E-01	6.31E-02	1.37E-02	1.01E-02	2.97E-02	4.27E-02	3.73E-02	2.69E-02	3.61E-02	2.46E-02	1.07E-01	3.04E-02
Peak sensitivity value	1.86E-03	1.60E-03	8.98E-01	2.80E-03	2.61E-02	7.01E-18	3.39E-04	7.90E-04	5.79E-05	1.47E-04	-2.16E-16	5.89E-03
Shapiro-Wilk score	4.866E-01	5.106E-01	5.968E-01	8.469E-01	4.168E-01	8.618E-01	4.928E-01	4.862E-01	4.915E-01	6.605E-01	7.888E-01	4.615E-01
**(Alcohol dehydrogenase)**												
Local	1.01E-02	4.37E-03	7.92E-01	2.32E-03	2.81E-02	-3.80E-16	9.18E-03	2.01E-02	1.72E-03	5.20E-03	6.30E-16	1.84E-01
Min	3.65E-04	2.63E-04	1.94E-03	2.18E-05	2.59E-04	-4.88E-15	1.36E-03	2.87E-03	2.51E-04	3.67E-04	-1.58E-14	3.66E-02
Max	8.59E-01	3.16E-01	9.23E-01	1.33E-02	8.26E-01	3.54E-15	4.28E-02	7.91E-02	7.24E-03	2.63E-02	1.46E-14	7.00E-01
Normalized peak height	1.06E-01	5.86E-02	5.98E-03	7.41E-03	3.19E-02	2.16E-02	5.52E-03	4.63E-03	5.15E-03	5.62E-03	6.66E-02	4.75E-03
Peak sensitivity value	1.65E-03	1.37E-03	8.48E-01	2.16E-03	2.30E-02	-5.50E-18	6.69E-03	1.46E-02	1.25E-03	2.74E-03	-1.44E-17	1.12E-01
Shapiro-Wilk score	4.842E-01	4.960E-01	7.401E-01	8.719E-01	4.285E-01	8.729E-01	9.325E-01	9.437E-01	9.146E-01	8.911E-01	9.277E-01	8.777E-01
**(Ethanol out)**												
Local	1.01E-02	4.37E-03	7.92E-01	2.32E-03	2.81E-02	-4.21E-16	9.18E-03	2.01E-02	1.72E-03	5.20E-03	5.13E-16	1.84E-01
Min	4.24E-04	2.58E-04	1.40E-03	1.19E-04	1.41E-03	-4.90E-15	1.33E-03	2.96E-03	2.19E-04	4.22E-04	-1.55E-14	3.92E-02
Max	8.56E-01	3.31E-01	9.22E-01	1.61E-02	8.02E-01	3.95E-15	3.81E-02	7.79E-02	9.99E-03	2.92E-02	1.13E-14	7.02E-01
Normalized peak height	1.08E-01	6.23E-02	5.73E-03	8.80E-03	3.16E-02	1.45E-01	5.08E-03	4.68E-03	6.46E-03	6.31E-03	3.39E-01	4.41E-03
Peak sensitivity value	1.71E-03	1.42E-03	8.54E-01	2.11E-03	2.26E-02	-1.08E-18	5.83E-03	1.39E-02	1.33E-03	3.03E-03	-4.95E-18	1.20E-01
Shapiro-Wilk score	4.791E-01	5.032E-01	7.422E-01	8.695E-01	4.277E-01	8.863E-01	9.329E-01	9.428E-01	9.176E-01	8.867E-01	9.197E-01	8.812E-01
**(Ethanol flow)**												
Local	1.01E-02	4.37E-03	7.92E-01	2.32E-03	2.81E-02	-3.81E-16	9.18E-03	2.01E-02	1.72E-03	5.20E-03	-1.74E-16	1.84E-01
Min	-3.23E-03	-4.81E-03	-1.54E-03	2.44E-04	2.74E-03	-4.40E-15	1.27E-03	2.98E-03	2.59E-04	4.00E-04	-1.74E-14	3.82E-02
Max	8.63E-01	3.92E-01	9.21E-01	1.40E-02	8.15E-01	4.62E-15	3.85E-02	7.60E-02	8.16E-03	3.37E-02	1.97E-14	7.03E-01
Normalized peak height	1.03E-01	7.36E-02	6.25E-03	7.61E-03	3.13E-02	3.92E-02	5.14E-03	4.84E-03	5.70E-03	7.11E-03	1.35E-01	4.42E-03
Peak sensitivity value	1.53E-03	1.35E-03	8.52E-01	2.11E-03	2.02E-02	-3.77E-18	6.69E-03	1.44E-02	1.19E-03	2.85E-03	-1.15E-17	1.00E-01
Shapiro-Wilk score	4.829E-01	5.010E-01	7.419E-01	8.722E-01	4.278E-01	8.656E-01	9.350E-01	9.442E-01	9.172E-01	8.882E-01	8.473E-01	8.819E-01
**(Glycerol synthesis)**												
Local	2.51E-02	1.08E-02	1.96E+00	8.63E-03	1.04E-01	8.06E-15	-8.18E-02	-1.79E-01	-1.53E-02	-4.63E-02	1.65E-14	-1.64E+00
Min	-2.63E-03	-1.68E-03	-3.26E-02	9.11E-04	3.49E-02	-6.95E-14	-2.42E-01	-3.86E-01	-3.92E-02	-1.73E-01	-2.66E-13	-2.79E+00
Max	2.59E+00	8.61E-01	3.01E+00	4.40E-02	2.42E+00	7.18E-14	-5.11E-03	-2.82E-02	-1.85E-03	-1.54E-03	3.07E-13	-9.56E-01
Normalized peak height	1.40E-01	7.08E-02	4.94E-03	7.13E-03	2.87E-02	2.14E-02	3.42E-03	3.02E-03	3.86E-03	4.69E-03	9.44E-02	3.67E-03
Peak sensitivity value	3.85E-03	3.07E-03	1.58E+00	7.26E-03	6.96E-02	-6.21E-17	-6.87E-02	-1.64E-01	-1.18E-02	-2.65E-02	-6.40E-17	-1.51E+00
Shapiro-Wilk score	4.884E-01	5.026E-01	8.304E-01	8.783E-01	4.291E-01	8.934E-01	9.721E-01	9.941E-01	9.769E-01	9.496E-01	7.821E-01	9.773E-01
**(Glycerol out)**												
Local	2.51E-02	1.08E-02	1.96E+00	8.63E-03	1.04E-01	8.12E-15	-8.18E-02	-1.79E-01	-1.53E-02	-4.63E-02	1.55E-14	-1.64E+00
Min	7.37E-04	4.67E-04	-6.92E-03	9.09E-04	2.99E-02	-7.04E-14	-3.03E-01	-4.19E-01	-4.19E-02	-1.69E-01	-4.20E-13	-2.68E+00
Max	2.59E+00	1.08E+00	3.02E+00	4.81E-02	2.31E+00	6.61E-14	-5.36E-03	-2.71E-02	-1.78E-03	-1.29E-03	3.62E-13	-8.46E-01
Normalized peak height	1.36E-01	8.64E-02	4.94E-03	7.79E-03	2.87E-02	2.28E-02	4.08E-03	3.35E-03	4.17E-03	4.29E-03	1.08E-01	3.73E-03
Peak sensitivity value	4.63E-03	3.17E-03	1.66E+00	7.12E-03	7.21E-02	-2.99E-17	-6.74E-02	-1.75E-01	-1.31E-02	-3.28E-02	-5.74E-17	-1.48E+00
Shapiro-Wilk score	4.886E-01	5.051E-01	8.281E-01	8.810E-01	4.263E-01	8.930E-01	9.730E-01	9.945E-01	9.756E-01	9.485E-01	7.677E-01	9.784E-01
**(Glycerol flow)**												
Local	2.51E-02	1.08E-02	1.96E+00	8.63E-03	1.04E-01	8.14E-15	-8.18E-02	-1.79E-01	-1.53E-02	-4.63E-02	1.49E-14	-1.64E+00
Min	-9.87E-04	-5.26E-04	-7.69E-02	8.22E-04	3.45E-02	-7.52E-14	-2.83E-01	-4.05E-01	-4.34E-02	-1.59E-01	-3.41E-13	-2.84E+00
Max	2.68E+00	8.97E-01	3.18E+00	4.52E-02	2.33E+00	6.78E-14	-4.91E-03	-2.72E-02	-1.89E-03	-1.66E-03	2.78E-13	-9.48E-01
Normalized peak height	1.38E-01	7.28E-02	5.41E-03	7.59E-03	2.78E-02	2.32E-02	3.85E-03	3.18E-03	4.22E-03	4.41E-03	1.17E-01	3.58E-03
Peak sensitivity value	3.03E-03	2.61E-03	1.67E+00	7.23E-03	7.47E-02	-5.98E-17	-7.06E-02	-1.62E-01	-1.38E-02	-3.01E-02	2.38E-16	-1.51E+00
Shapiro-Wilk score	4.880E-01	4.980E-01	8.303E-01	8.747E-01	4.252E-01	8.950E-01	9.726E-01	9.941E-01	9.758E-01	9.505E-01	7.771E-01	9.776E-01
**(Acetaldehyde out)**												
Local	2.51E-02	1.08E-02	1.96E+00	8.63E-03	1.04E-01	-5.92E-15	-8.18E-02	-1.79E-01	-1.53E-02	-4.63E-02	4.56E-21	-1.64E+00
Min	7.43E-04	5.48E-04	-4.35E-02	6.62E-04	3.25E-02	-4.52E-14	-2.87E-01	-3.84E-01	-3.94E-02	-1.83E-01	-2.64E-13	-2.73E+00
Max	2.71E+00	9.35E-01	2.98E+00	4.41E-02	2.44E+00	8.08E-14	-5.88E-03	-3.18E-02	-1.85E-03	-2.04E-03	3.02E-13	-9.46E-01
Normalized peak height	1.36E-01	7.49E-02	4.88E-03	6.96E-03	2.93E-02	5.81E-02	3.84E-03	2.95E-03	4.13E-03	4.74E-03	3.11E-01	3.28E-03
Peak sensitivity value	4.81E-03	2.89E-03	1.59E+00	6.98E-03	6.98E-02	-3.54E-17	-6.96E-02	-1.57E-01	-1.31E-02	-3.24E-02	1.71E-16	-1.48E+00
Shapiro-Wilk score	4.916E-01	5.067E-01	8.322E-01	8.826E-01	4.350E-01	8.582E-01	9.742E-01	9.944E-01	9.759E-01	9.487E-01	8.018E-01	9.770E-01
**(Acetaldehyde flow)**												
Local	2.55E-02	1.10E-02	1.99E+00	8.78E-03	1.06E-01	-5.73E-15	-8.32E-02	-1.82E-01	-1.56E-02	-4.71E-02	-8.63E-15	-1.67E+00
Min	-1.63E-03	-4.38E-03	-4.81E-02	6.88E-04	3.20E-02	-5.82E-14	-2.69E-01	-3.86E-01	-4.61E-02	-1.94E-01	-2.09E-13	-2.97E+00
Max	3.05E+00	9.80E-01	3.04E+00	4.91E-02	2.44E+00	4.59E-14	-4.97E-03	-2.88E-02	-1.81E-03	-1.82E-03	3.03E-13	-9.57E-01
Normalized peak height	1.46E-01	7.89E-02	5.15E-03	7.95E-03	2.81E-02	2.79E-02	3.82E-03	2.98E-03	4.72E-03	4.84E-03	1.30E-01	3.59E-03
Peak sensitivity value	2.94E-03	3.00E-03	1.64E+00	6.71E-03	7.18E-02	-4.64E-17	-7.35E-02	-1.71E-01	-1.36E-02	-3.49E-02	-2.42E-16	-1.43E+00
Shapiro-Wilk score	4.891E-01	4.988E-01	8.412E-01	8.722E-01	4.313E-01	8.653E-01	9.726E-01	9.941E-01	9.744E-01	9.476E-01	7.209E-01	9.758E-01

**(Glyceraldehyde 3-phosphate dehydrogenase)**												
Local	0.00E+00	0.00E+00	-1.78E-03	7.65E-16	6.61E-16	-4.28E-04	-2.23E-03	-6.32E-04	-5.04E-05	-2.40E-02	5.89E-02	2.91E-18
Min	-2.57E-14	-1.15E-13	-4.63E-02	-3.19E-14	-2.74E-14	-1.01E-02	-4.22E-02	-1.14E-02	-2.76E-03	-8.04E-02	1.83E-04	-6.18E-17
Max	3.01E-14	8.97E-14	6.11E-03	3.72E-14	6.00E-14	1.25E-03	5.74E-03	1.39E-03	2.38E-04	2.17E-01	1.21E-01	2.57E-16
Normalized peak height	5.19E-01	6.66E-01	2.64E-02	1.46E-02	3.07E-02	2.30E-02	2.11E-02	2.02E-02	4.70E-02	1.63E-02	3.48E-03	2.92E-01
Peak sensitivity value	1.24E-17	-3.06E-17	-1.20E-03	-1.25E-16	-1.82E-17	-3.33E-04	-1.86E-03	-5.27E-04	-2.14E-05	-2.46E-02	6.13E-02	1.52E-19
Shapiro-Wilk score	2.803E-01	1.565E-01	5.171E-01	9.389E-01	9.012E-01	5.272E-01	5.175E-01	5.383E-01	4.371E-01	7.428E-01	9.928E-01	8.513E-01
**(Phosphoenolpyruvate synthesis)**												
Local	0.00E+00	0.00E+00	-1.78E-03	-2.42E-15	1.16E-15	-4.28E-04	-2.23E-03	-6.32E-04	-5.04E-05	-2.40E-02	5.89E-02	2.91E-18
Min	-1.36E-13	-2.80E-13	-4.33E-02	-5.46E-14	-2.66E-14	-1.13E-02	-4.11E-02	-1.12E-02	-2.37E-03	-8.65E-02	2.93E-04	-1.19E-16
Max	4.17E-13	1.01E-13	7.66E-03	5.15E-14	3.22E-14	8.66E-04	5.64E-03	1.65E-03	3.85E-04	2.23E-01	1.24E-01	1.01E-16
Normalized peak height	8.49E-01	7.71E-01	2.54E-02	2.15E-02	2.35E-02	2.50E-02	2.04E-02	2.02E-02	4.36E-02	1.74E-02	3.39E-03	2.92E-01
Peak sensitivity value	6.66E-17	-1.56E-16	-1.23E-03	2.77E-19	1.25E-18	-2.96E-04	-1.87E-03	-5.51E-04	-2.10E-05	-2.50E-02	6.57E-02	3.03E-20
Shapiro-Wilk score	1.454E-01	1.466E-01	5.117E-01	9.350E-01	8.995E-01	5.167E-01	5.070E-01	5.288E-01	4.285E-01	7.406E-01	9.923E-01	8.970E-01
**(Pyruvate kinase)**												
Local	0.00E+00	0.00E+00	-1.78E-03	4.02E-15	-8.26E-16	-4.28E-04	-2.23E-03	-6.32E-04	-5.04E-05	-2.40E-02	5.89E-02	2.91E-18
Min	-1.57E-13	-1.01E-13	-4.94E-02	-8.07E-14	-3.25E-14	-1.24E-02	-4.22E-02	-1.15E-02	-2.38E-03	-8.67E-02	1.28E-04	-5.86E-17
Max	1.19E-13	7.09E-14	4.46E-03	3.24E-14	4.81E-14	1.93E-03	5.63E-03	1.65E-03	2.26E-04	2.03E-01	1.25E-01	2.79E-16
Normalized peak height	6.87E-01	6.64E-01	2.76E-02	2.89E-02	3.77E-02	2.93E-02	2.13E-02	2.07E-02	4.29E-02	1.58E-02	3.63E-03	2.88E-01
Peak sensitivity value	2.82E-17	6.44E-17	-1.38E-03	-2.68E-17	6.11E-18	-3.03E-04	-1.67E-03	-4.98E-04	-2.54E-05	-2.49E-02	6.52E-02	-4.90E-20
Shapiro-Wilk score	1.496E-01	1.738E-01	5.069E-01	9.158E-01	8.664E-01	5.156E-01	5.113E-01	5.396E-01	4.360E-01	7.636E-01	9.922E-01	8.585E-01
**(Pyruvate decarboxylase)**												
Local	0.00E+00	0.00E+00	-1.78E-03	-6.65E-16	1.91E-15	-4.28E-04	-2.23E-03	-6.32E-04	-5.04E-05	-2.40E-02	5.89E-02	2.91E-18
Min	-1.50E-14	-1.17E-13	-5.39E-02	-4.58E-14	-4.58E-14	-1.03E-02	-3.91E-02	-1.05E-02	-2.67E-03	-8.20E-02	1.40E-04	-1.07E-16
Max	1.18E-14	5.43E-14	7.63E-03	4.37E-14	3.64E-14	2.02E-03	8.33E-03	1.76E-03	3.29E-04	2.13E-01	1.22E-01	1.08E-16
Normalized peak height	5.33E-01	6.71E-01	3.08E-02	3.04E-02	5.68E-02	2.57E-02	2.06E-02	1.97E-02	4.82E-02	1.58E-02	3.56E-03	2.88E-01
Peak sensitivity value	-1.19E-17	8.01E-17	-1.14E-03	-4.19E-17	-1.71E-17	-3.07E-04	-1.65E-03	-5.23E-04	-2.34E-05	-2.37E-02	6.03E-02	-9.95E-20
Shapiro-Wilk score	3.634E-01	1.602E-01	5.025E-01	8.976E-01	8.704E-01	5.280E-01	5.203E-01	5.408E-01	4.363E-01	7.466E-01	9.932E-01	8.850E-01
**(Alcohol dehydrogenase)**												
Local	0.00E+00	0.00E+00	-3.13E-02	-1.84E-16	1.26E-15	-7.52E-03	-3.91E-02	-1.11E-02	-8.85E-04	-1.89E-02	5.21E-02	2.13E-18
Min	-1.12E-14	-4.09E-14	-1.23E-01	-1.85E-14	-2.09E-14	-2.79E-02	-1.02E-01	-2.91E-02	-6.99E-03	-6.96E-02	1.13E-04	-6.30E-17
Max	7.28E-15	4.87E-14	-4.91E-03	2.54E-14	2.11E-14	-1.60E-03	-1.25E-02	-3.78E-03	-4.82E-05	1.72E-01	1.15E-01	1.20E-16
Normalized peak height	5.26E-01	6.36E-01	4.77E-03	2.06E-02	3.72E-02	4.61E-03	3.63E-03	3.59E-03	7.53E-03	1.27E-02	3.26E-03	2.91E-01
Peak sensitivity value	-7.08E-18	-1.33E-17	-2.60E-02	-8.65E-18	1.17E-17	-6.12E-03	-3.47E-02	-9.62E-03	-3.64E-04	-2.02E-02	5.33E-02	-5.26E-20
Shapiro-Wilk score	4.527E-01	1.895E-01	9.410E-01	9.181E-01	8.892E-01	9.459E-01	9.636E-01	9.738E-01	8.254E-01	7.911E-01	9.978E-01	8.999E-01
**(Ethanol out)**												
Local	2.37E-16	-8.88E-16	-3.13E-02	-1.07E-16	1.31E-15	-7.52E-03	-3.91E-02	-1.11E-02	-8.85E-04	-1.89E-02	5.21E-02	2.13E-18
Min	-1.03E-14	-6.93E-14	-1.32E-01	-2.90E-14	-3.53E-14	-3.17E-02	-1.11E-01	-2.95E-02	-7.52E-03	-7.47E-02	2.23E-04	-3.72E-17
Max	7.64E-15	5.42E-14	-5.04E-03	2.37E-14	3.39E-14	-1.61E-03	-1.22E-02	-3.84E-03	-5.18E-05	1.83E-01	1.19E-01	1.18E-16
Normalized peak height	6.31E-02	4.92E-01	4.87E-03	2.38E-02	5.63E-02	4.87E-03	3.76E-03	3.87E-03	7.79E-03	1.31E-02	3.26E-03	2.93E-01
Peak sensitivity value	-2.16E-16	4.31E-17	-2.27E-02	-5.93E-18	1.70E-18	-6.23E-03	-3.38E-02	-9.04E-03	-4.51E-04	-2.08E-02	5.51E-02	4.61E-20
Shapiro-Wilk score	9.789E-01	5.119E-01	9.388E-01	9.149E-01	8.726E-01	9.455E-01	9.649E-01	9.735E-01	8.337E-01	8.047E-01	9.983E-01	9.226E-01
**(Ethanol flow)**												
Local	-3.41E-16	-1.11E-15	-3.13E-02	-1.73E-16	1.29E-15	-7.52E-03	-3.91E-02	-1.11E-02	-8.85E-04	-1.89E-02	5.21E-02	2.13E-18
Min	-1.02E-14	-4.53E-14	-1.37E-01	-3.06E-14	-1.84E-14	-2.94E-02	-1.12E-01	-2.66E-02	-6.92E-03	-7.70E-02	1.14E-04	-7.29E-17
Max	1.55E-14	3.65E-14	-5.45E-03	2.46E-14	2.08E-14	-1.54E-03	-1.33E-02	-3.52E-03	-5.55E-05	2.18E-01	1.20E-01	3.41E-16
Normalized peak height	4.67E-01	1.18E-01	5.01E-03	2.58E-02	3.48E-02	4.80E-03	3.98E-03	3.46E-03	7.11E-03	1.58E-02	3.19E-03	2.92E-01
Peak sensitivity value	-2.79E-18	-2.53E-16	-2.21E-02	2.05E-17	-1.61E-17	-5.85E-03	-3.41E-02	-1.01E-02	-4.78E-04	-2.17E-02	5.42E-02	1.37E-19
Shapiro-Wilk score	5.555E-01	6.080E-01	9.397E-01	9.137E-01	8.786E-01	9.460E-01	9.636E-01	9.732E-01	8.344E-01	7.930E-01	9.981E-01	7.418E-01
**(Glycerol synthesis)**												
Local	0.00E+00	0.00E+00	2.79E-01	8.97E-15	-6.51E-15	6.70E-02	3.48E-01	9.89E-02	7.89E-03	-7.27E-02	1.24E-01	1.03E-17
Min	-4.25E-13	-2.15E-12	2.88E-02	-3.15E-13	-2.44E-13	1.01E-02	8.54E-02	1.59E-02	1.24E-03	-2.04E-01	-2.18E-03	-2.06E-16
Max	2.79E-13	1.30E-12	5.57E-01	2.71E-13	2.45E-13	1.79E-01	6.07E-01	2.39E-01	2.80E-02	4.23E-01	2.58E-01	1.39E-15
Normalized peak height	6.81E-01	9.53E-01	2.75E-03	1.88E-02	3.59E-02	3.55E-03	3.71E-03	3.59E-03	4.21E-03	1.65E-02	3.94E-03	2.86E-01
Peak sensitivity value	-2.83E-16	7.62E-18	2.52E-01	9.04E-17	-1.21E-16	5.77E-02	3.62E-01	9.38E-02	5.03E-03	-7.09E-02	1.23E-01	-5.18E-19
Shapiro-Wilk score	1.780E-01	1.443E-01	9.970E-01	9.478E-01	9.163E-01	9.803E-01	9.975E-01	9.934E-01	9.376E-01	7.328E-01	9.877E-01	6.839E-01
**(Glycerol out)**												
Local	0.00E+00	0.00E+00	2.79E-01	9.24E-15	-6.55E-15	6.70E-02	3.48E-01	9.89E-02	7.89E-03	-7.27E-02	1.24E-01	1.03E-17
Min	-3.07E-13	-1.26E-12	3.17E-02	-3.49E-13	-2.79E-13	1.14E-02	7.22E-02	1.65E-02	1.24E-03	-1.96E-01	-6.82E-04	-1.88E-16
Max	3.34E-13	9.11E-13	5.74E-01	3.90E-13	2.54E-13	1.97E-01	5.95E-01	2.33E-01	2.83E-02	5.47E-01	2.51E-01	6.40E-16
Normalized peak height	6.70E-01	9.11E-01	2.98E-03	2.08E-02	3.06E-02	4.00E-03	3.56E-03	3.69E-03	4.25E-03	1.92E-02	3.88E-03	2.85E-01
Peak sensitivity value	2.36E-16	3.71E-16	2.74E-01	-2.47E-16	-1.69E-16	5.68E-02	3.46E-01	1.02E-01	4.90E-03	-6.82E-02	1.11E-01	3.87E-19
Shapiro-Wilk score	1.757E-01	1.472E-01	9.973E-01	9.442E-01	9.153E-01	9.800E-01	9.975E-01	9.933E-01	9.374E-01	7.370E-01	9.886E-01	8.814E-01
**(Glycerol flow)**												
Local	0.00E+00	0.00E+00	2.79E-01	9.72E-15	-6.66E-15	6.70E-02	3.48E-01	9.89E-02	7.89E-03	-7.27E-02	1.24E-01	1.03E-17
Min	-2.00E-13	-1.82E-12	3.07E-02	-3.62E-13	-2.76E-13	1.22E-02	9.09E-02	1.85E-02	1.16E-03	-2.22E-01	-3.89E-03	-1.46E-16
Max	3.55E-13	1.89E-12	5.88E-01	3.13E-13	3.29E-13	1.85E-01	5.91E-01	2.46E-01	2.82E-02	4.64E-01	2.52E-01	2.85E-16
Normalized peak height	6.18E-01	8.71E-01	3.01E-03	2.02E-02	4.06E-02	3.80E-03	3.37E-03	3.47E-03	4.05E-03	1.73E-02	3.85E-03	2.88E-01
Peak sensitivity value	-2.55E-16	1.32E-15	3.19E-01	-1.76E-16	1.12E-17	6.00E-02	3.29E-01	9.68E-02	4.93E-03	-6.96E-02	1.18E-01	-7.16E-20
Shapiro-Wilk score	1.883E-01	1.396E-01	9.971E-01	9.418E-01	9.178E-01	9.801E-01	9.976E-01	9.936E-01	9.377E-01	7.259E-01	9.875E-01	9.395E-01
**(Acetaldehyde out)**												
Local	0.00E+00	0.00E+00	2.79E-01	-5.24E-15	8.08E-15	6.70E-02	3.48E-01	9.89E-02	7.89E-03	-7.27E-02	1.24E-01	1.03E-17
Min	-1.72E-13	-8.31E-13	3.35E-02	-1.77E-13	-1.32E-13	1.17E-02	9.41E-02	1.57E-02	1.28E-03	-2.10E-01	-3.75E-04	-1.99E-16
Max	2.12E-13	1.09E-12	5.74E-01	2.34E-13	1.75E-13	1.80E-01	6.06E-01	2.40E-01	3.01E-02	4.66E-01	2.59E-01	2.50E-15
Normalized peak height	6.08E-01	8.23E-01	3.01E-03	1.75E-02	3.00E-02	3.64E-03	3.49E-03	3.66E-03	4.61E-03	1.71E-02	4.07E-03	2.92E-01
Peak sensitivity value	-1.55E-16	6.77E-16	2.65E-01	1.48E-16	4.38E-17	6.65E-02	3.68E-01	8.70E-02	4.93E-03	-6.82E-02	1.16E-01	-1.14E-18
Shapiro-Wilk score	2.280E-01	1.314E-01	9.973E-01	9.610E-01	9.397E-01	9.805E-01	9.975E-01	9.933E-01	9.402E-01	7.342E-01	9.888E-01	5.399E-01
**(Acetaldehyde flow)**												
Local	0.00E+00	0.00E+00	2.84E-01	-5.10E-15	7.94E-15	6.82E-02	5.75E-01	-1.21E-01	-9.62E-03	-7.39E-02	1.27E-01	1.05E-17
Min	-1.84E-13	-1.09E-12	3.29E-02	-1.43E-13	-1.67E-13	1.12E-02	2.28E-01	-2.31E-01	-9.29E-02	-2.06E-01	-8.68E-04	-2.66E-16
Max	2.33E-13	9.14E-13	5.87E-01	2.06E-13	1.42E-13	1.93E-01	8.05E-01	-4.93E-02	-7.59E-04	4.68E-01	2.78E-01	5.21E-16
Normalized peak height	6.16E-01	8.33E-01	2.96E-03	1.88E-02	3.65E-02	3.98E-03	3.69E-03	3.21E-03	9.33E-03	1.71E-02	4.09E-03	2.91E-01
Peak sensitivity value	1.78E-16	-6.95E-16	2.68E-01	-1.10E-16	2.38E-17	5.70E-02	5.87E-01	-1.13E-01	-6.15E-03	-6.97E-02	1.28E-01	-2.07E-19
Shapiro-Wilk score	1.799E-01	1.193E-01	9.970E-01	9.619E-01	9.309E-01	9.807E-01	9.898E-01	9.895E-01	8.271E-01	7.323E-01	9.892E-01	8.687E-01

(Cyanide-Acetaldehyde flow)	Local	2.36E-02	1.02E-02	1.85E+00	8.13E-03	9.84E-02	-5.30E-15	-7.71E-02	-1.69E-01	-1.45E-02	-4.36E-02	-8.00E-15	-1.54E+00	0.00E+00	0.00E+00	2.63E-01	-4.73E-15	7.34E-15	6.31E-02	-3.93E-01	8.14E-01	6.50E-02	-6.85E-02	1.17E-01	9.70E-18
	Min	-3.94E-03	-1.65E-03	-2.43E-02	3.58E-04	2.92E-02	-5.11E-14	-2.50E-01	-3.81E-01	-4.01E-02	-1.58E-01	-2.32E-13	-2.44E+00	-1.62E-13	-1.51E-12	2.64E-02	-1.78E-13	-1.19E-13	8.68E-03	-6.37E-01	5.60E-01	1.17E-02	-1.80E-01	-1.96E-03	-3.56E-16
	Max	2.47E+00	8.22E-01	2.71E+00	4.26E-02	2.21E+00	4.98E-14	-5.58E-03	-2.41E-02	-1.67E-03	-1.01E-03	2.41E-13	-8.30E-01	2.64E-13	1.09E-12	5.33E-01	1.91E-13	1.47E-13	1.69E-01	-1.72E-01	9.31E-01	2.75E-01	4.03E-01	2.59E-01	1.03E-15
	Normalized peak height	1.28E-01	7.19E-02	5.22E-03	7.52E-03	2.98E-02	2.61E-02	3.47E-03	3.24E-03	4.45E-03	4.34E-03	1.16E-01	3.68E-03	6.32E-01	8.27E-01	2.84E-03	2.16E-02	3.61E-02	3.57E-03	3.38E-03	3.99E-03	5.43E-03	1.62E-02	4.13E-03	2.88E-01
	Peak sensitivity value	4.74E-03	2.88E-03	1.60E+00	6.50E-03	6.96E-02	-6.83E-18	-5.67E-02	-1.67E-01	-1.25E-02	-3.12E-02	1.52E-16	-1.34E+00	-5.80E-17	-1.04E-15	2.74E-01	3.88E-17	3.73E-17	5.56E-02	-4.06E-01	8.28E-01	4.32E-02	-6.75E-02	1.10E-01	3.87E-19
	Shapiro-Wilk score	4.899E-01	5.055E-01	7.996E-01	8.841E-01	4.255E-01	8.582E-01	9.739E-01	9.941E-01	9.763E-01	9.510E-01	7.313E-01	9.809E-01	1.703E-01	1.170E-01	9.968E-01	9.600E-01	9.369E-01	9.812E-01	9.973E-01	9.741E-01	9.051E-01	7.420E-01	9.880E-01	7.327E-01
(Cyanide flow)	Local	2.36E-02	1.02E-02	1.85E+00	8.13E-03	9.84E-02	-5.36E-15	-7.71E-02	-1.69E-01	-1.45E-02	-4.36E-02	-7.92E-15	-1.54E+00	0.00E+00	0.00E+00	2.63E-01	-4.59E-15	7.47E-15	6.31E-02	-3.93E-01	8.14E-01	6.50E-02	-6.85E-02	1.17E-01	9.70E-18
	Min	-9.00E-03	-3.23E-03	-5.24E-02	3.30E-04	2.92E-02	-7.57E-14	-2.68E-01	-3.96E-01	-3.80E-02	-1.54E-01	-3.41E-13	-2.56E+00	-1.81E-13	-8.07E-13	2.79E-02	-1.84E-13	-1.08E-13	9.11E-03	-6.53E-01	5.57E-01	1.05E-02	-1.80E-01	-3.43E-03	-2.94E-16
	Max	2.59E+00	9.02E-01	2.68E+00	4.11E-02	2.29E+00	6.46E-14	-5.24E-03	-2.31E-02	-2.01E-03	-1.14E-03	2.44E-13	-8.63E-01	2.67E-13	8.35E-13	5.37E-01	2.03E-13	1.24E-13	1.69E-01	-1.94E-01	9.41E-01	2.88E-01	4.30E-01	2.52E-01	3.88E-16
	Normalized peak height	1.32E-01	7.82E-02	5.12E-03	7.56E-03	2.92E-02	3.69E-02	3.70E-03	3.28E-03	3.99E-03	4.35E-03	1.28E-01	3.74E-03	6.37E-01	9.21E-01	2.95E-03	2.17E-02	2.63E-02	3.38E-03	3.28E-03	3.89E-03	5.29E-03	1.65E-02	4.20E-03	2.88E-01
	Peak sensitivity value	2.70E-03	2.66E-03	1.63E+00	6.71E-03	6.65E-02	-1.27E-17	-6.33E-02	-1.73E-01	-1.26E-02	-3.11E-02	1.60E-16	-1.38E+00	4.38E-17	-5.47E-17	2.65E-01	-4.25E-17	1.05E-16	5.73E-02	-3.71E-01	8.32E-01	3.84E-02	-6.18E-02	1.11E-01	-2.38E-19
	Shapiro-Wilk score	4.878E-01	5.022E-01	7.993E-01	8.825E-01	4.298E-01	8.589E-01	9.724E-01	9.938E-01	9.764E-01	9.484E-01	6.928E-01	9.810E-01	1.567E-01	1.280E-01	9.967E-01	9.563E-01	9.379E-01	9.804E-01	9.974E-01	9.741E-01	9.085E-01	7.314E-01	9.878E-01	8.739E-01
(Storage)	Local	1.21E-02	5.20E-03	9.43E-01	-1.53E-03	-1.85E-02	-1.69E-15	9.90E-03	2.16E-02	1.86E-03	5.60E-03	-3.99E-15	1.98E-01	0.00E+00	0.00E+00	-3.37E-02	-2.74E-15	-1.38E-16	-8.11E-03	-4.21E-02	-1.20E-02	-9.55E-04	1.62E-02	-9.65E-02	-1.73E-18
	Min	5.43E-04	3.41E-04	-1.86E-02	-8.28E-03	-3.71E-01	-2.03E-14	1.16E-03	2.43E-03	2.59E-04	3.63E-04	-8.15E-14	4.13E-02	-5.24E-14	-3.00E-13	-1.23E-01	-8.34E-14	-8.76E-14	-2.79E-02	-9.70E-02	-2.68E-02	-7.36E-03	-2.94E-02	-2.54E-01	-9.83E-17
	Max	1.04E+00	4.18E-01	1.15E+00	1.52E-02	6.63E-01	1.91E-14	3.68E-02	7.44E-02	8.20E-03	3.24E-02	7.55E-14	7.50E-01	4.82E-14	4.00E-13	-5.43E-03	1.25E-13	8.89E-14	-1.60E-03	-1.47E-02	-4.16E-03	-6.67E-05	2.44E-01	-2.56E-02	7.82E-17
	Normalized peak height	1.06E-01	6.39E-02	6.16E-03	1.06E-02	3.93E-02	4.84E-02	4.64E-03	4.37E-03	5.29E-03	6.41E-03	7.72E-02	4.83E-03	5.26E-01	7.89E-01	4.47E-03	1.96E-02	2.53E-02	4.47E-03	3.18E-03	3.20E-03	7.69E-03	1.30E-02	3.68E-03	2.97E-01
	Peak sensitivity value	2.11E-03	1.80E-03	1.01E+00	-1.24E-03	-1.38E-02	1.42E-17	7.21E-03	1.69E-02	1.19E-03	2.43E-03	4.67E-17	1.10E-01	-3.64E-17	8.43E-17	-2.60E-02	-2.50E-17	3.62E-17	-5.90E-03	-3.44E-02	-1.02E-02	-4.06E-04	1.86E-02	-8.92E-02	8.61E-20
	Shapiro-Wilk score	4.901E-01	5.154E-01	7.340E-01	9.214E-01	4.658E-01	9.002E-01	9.435E-01	9.549E-01	9.214E-01	8.850E-01	8.370E-01	8.921E-01	2.837E-01	1.484E-01	9.471E-01	9.200E-01	8.910E-01	9.508E-01	9.689E-01	9.754E-01	8.449E-01	7.505E-01	9.828E-01	8.665E-01
(ATP consumption)	Local	-1.59E-03	-6.83E-04	-1.24E-01	2.57E-02	3.11E-01	-8.56E-16	4.80E-03	1.05E-02	9.01E-04	2.72E-03	-2.17E-15	9.61E-02	0.00E+00	0.00E+00	-1.64E-02	-1.37E-15	-6.97E-17	-3.93E-03	-2.04E-02	-5.81E-03	-4.63E-04	-2.32E-01	9.53E-01	2.55E-17
	Min	-5.90E+00	-1.36E+00	-8.11E+00	1.39E-03	1.15E-01	-5.17E-14	5.59E-04	1.28E-03	1.03E-04	1.72E-04	-1.61E-13	1.58E-02	-2.87E-14	-1.26E-13	-3.98E-01	-2.25E-13	-1.52E-13	-8.67E-02	-3.05E-01	-9.10E-02	-1.94E-02	-1.08E+00	6.51E-01	-1.43E-15
	Max	1.33E-01	1.72E-01	1.42E+00	9.30E-02	7.30E+00	5.57E-14	1.15E-01	2.14E-01	2.16E-02	3.64E-02	1.64E-13	2.11E+00	3.68E-14	1.33E-13	-2.10E-03	3.67E-13	2.19E-13	-6.56E-04	-4.70E-03	-1.37E-03	-2.13E-05	-1.94E-02	9.78E-01	1.02E-15
	Normalized peak height	4.30E-01	6.14E-01	5.70E-02	5.91E-03	3.11E-02	1.41E-01	2.33E-02	1.86E-02	2.35E-02	1.24E-02	1.56E-01	2.03E-02	5.38E-01	7.15E-01	2.28E-02	9.33E-02	8.71E-02	2.06E-02	1.57E-02	1.69E-02	3.37E-02	1.14E-02	9.11E-03	2.92E-01
	Peak sensitivity value	-3.11E-03	-3.95E-04	-1.04E-02	2.16E-02	2.12E-01	4.31E-17	2.91E-03	6.27E-03	5.66E-04	1.53E-03	-4.09E-17	4.40E-02	2.56E-17	9.51E-17	-1.14E-02	-6.68E-17	1.60E-16	-3.19E-03	-1.36E-02	-4.11E-03	-1.86E-04	-2.21E-01	9.48E-01	-3.07E-19
	Shapiro-Wilk score	2.645E-01	2.135E-01	4.311E-01	9.357E-01	4.832E-01	5.427E-01	5.870E-01	5.830E-01	5.807E-01	7.278E-01	6.698E-01	5.560E-01	2.882E-01	1.601E-01	5.922E-01	6.019E-01	5.752E-01	6.084E-01	5.963E-01	6.115E-01	5.391E-01	8.336E-01	7.764E-01	8.735E-01
(Adenylate kinase)	Local	-INF	-INF	-INF	-INF	-INF	-INF	-INF	-INF	-INF	-INF	-INF	-INF	-INF	-INF	-INF	-INF	-INF	-INF	-INF	-INF	-INF	-INF	-INF	-INF
	Min	-INF	-INF	-INF	-INF	-INF	-INF	-INF	-INF	-INF	-INF	-INF	-INF	-INF	-INF	-INF	-INF	-INF	-INF	-INF	-INF	-INF	-INF	-INF	-INF
	Max	INF	INF	INF	INF	INF	INF	INF	INF	INF	INF	INF	INF	INF	INF	INF	INF	INF	INF	INF	INF	INF	INF	INF	INF
	Normalized peak height	NA	NA	NA	NA	NA	NA	NA	NA	NA	NA	NA	NA	NA	NA	NA	NA	NA	NA	NA	NA	NA	NA	NA	NA
	Peak sensitivity value	NAN	NAN	NAN	NAN	NAN	NAN	NAN	NAN	NAN	NAN	NAN	NAN	NAN	NAN	NAN	NAN	NAN	NAN	NAN	NAN	NAN	NAN	NAN	NAN
	Shapiro-Wilk score	NA	NA	NA	NA	NA	NA	NA	NA	NA	NA	NA	NA	NA	NA	NA	NA	NA	NA	NA	NA	NA	NA	NA	NA

Table S29: Flux control coefficients for *Saccharomyces cerevisiae* model with parameter variation of ±40%

		(GluconseMixedflowtoextracellularmedium)	(Glucoseuptake)	(Hexokinase)	(Phosphoglucoisomerase)	(Phosphofructokinase)	(Aldolase)	(Triosephosphateisomerase)	(Glyceraldehyde3-phosphatedehydrogenase)	(Phosphoenolpyruvatesynthesis)	(Pyruvatekinase)	(Pyruvatedecarboxylase)	(Alcoholdehydrogenase)	(Ethanolout)	(Ethanolflow)	(Glycerolsynthesis)	(Glycerolout)	(Glycerolflow)	(Acetaldehydeout)	(Acetaldehydeflow)	(Cyanide-Acetaldehydeflow)	(Cyanideflow)	(Storage)	(ATPconsumption)	(Adenylatekinase)
(Glucose Mixed flow to extra-cellular medium)	Local	1.25E-02	5.36E-03	9.73E-01	1.37E-03	1.65E-02	-3.45E-17	2.30E-04	5.02E-04	4.31E-05	1.30E-04	-8.14E-17	4.60E-03	0.00E+00	0.00E+00	-7.83E-04	-6.35E-17	-5.80E-18	-1.88E-04	-9.78E-04	-2.78E-04	-2.22E-05	-9.81E-03	-2.24E-03	1.36E-18
	Min	-6.41E-03	-5.67E-03	-1.38E-01	2.40E-05	8.74E-04	-1.08E-14	-7.08E-03	-8.76E-03	-1.12E-03	-4.37E-03	-2.96E-14	-9.77E-02	-2.25E-14	-1.82E-13	-6.12E-02	-6.20E-14	-4.76E-14	-1.54E-02	-4.66E-02	-1.41E-02	-3.49E-03	-1.34E-01	-4.35E-02	-5.56E-17
	Max	9.90E-01	5.10E-01	1.01E+00	3.26E-02	9.43E-01	1.26E-14	2.22E-02	4.05E-02	6.41E-03	7.09E-03	2.31E-14	2.85E-01	2.18E-14	1.07E-13	1.52E-02	1.00E-13	4.10E-14	3.84E-03	1.42E-02	4.66E-03	9.02E-04	3.09E-01	3.82E-02	9.68E-17
	Normalized peak height	1.03E-01	6.90E-02	2.76E-02	1.82E-02	4.35E-02	4.17E-01	8.87E-02	6.27E-02	1.21E-01	6.51E-02	5.25E-01	6.40E-02	6.98E-01	8.95E-01	6.21E-02	4.95E-01	4.52E-01	6.34E-02	3.97E-02	4.34E-02	1.52E-01	2.62E-02	1.99E-02	2.80E-01
	Peak sensitivity value	1.06E-03	1.30E-03	9.97E-01	9.19E-04	8.88E-03	1.72E-18	8.61E-05	1.75E-04	1.95E-05	3.35E-05	-7.36E-18	1.60E-03	1.91E-17	7.45E-17	-3.87E-04	-1.30E-17	1.47E-17	-9.31E-05	-5.20E-04	-1.55E-04	-5.16E-06	-7.90E-03	-1.89E-03	-3.09E-20
	Shapiro-Wilk score	5.587E-01	5.032E-01	6.837E-01	6.589E-01	4.550E-01	3.179E-01	4.361E-01	4.644E-01	4.268E-01	5.408E-01	2.407E-01	4.657E-01	8.039E-02	4.840E-02	4.712E-01	3.215E-01	3.049E-01	4.795E-01	5.038E-01	5.112E-01	3.922E-01	6.249E-01	7.416E-01	8.010E-01
(Glucose uptake)	Local	1.25E-02	5.36E-03	9.73E-01	1.37E-03	1.65E-02	-2.20E-17	2.30E-04	5.02E-04	4.31E-05	1.30E-04	-8.36E-17	4.60E-03	0.00E+00	0.00E+00	-7.83E-04	-6.57E-17	-3.32E-18	-1.88E-04	-9.78E-04	-2.78E-04	-2.22E-05	-9.81E-03	-2.24E-03	1.36E-18
	Min	-4.22E-02	-1.71E-02	-9.11E-02	2.38E-05	7.78E-04	-1.40E-14	-6.69E-03	-1.15E-02	-1.04E-03	-4.71E-03	-4.78E-14	-1.11E-01	-4.20E-14	-8.50E-14	-5.83E-02	-5.64E-14	-6.46E-14	-1.53E-02	-5.09E-02	-1.61E-02	-3.38E-03	-1.09E-01	-4.33E-02	-3.49E-17
	Max	9.87E-01	5.23E-01	1.01E+00	2.89E-02	9.45E-01	1.35E-14	1.81E-02	4.08E-02	4.34E-03	8.07E-03	3.59E-14	2.73E-01	2.05E-14	1.81E-13	1.83E-02	6.53E-14	5.20E-14	3.43E-03	1.59E-02	4.07E-03	6.12E-04	3.05E-01	3.45E-02	8.13E-17
	Normalized peak height	1.14E-01	7.23E-02	2.79E-02	1.61E-02	4.36E-02	2.03E-01	7.39E-02	6.69E-02	8.67E-02	7.18E-02	4.65E-01	6.14E-02	8.34E-01	7.55E-01	6.02E-02	3.80E-01	4.36E-01	6.00E-02	4.38E-02	4.54E-02	1.38E-01	2.44E-02	1.94E-02	2.74E-01
	Peak sensitivity value	1.55E-03	9.88E-04	9.97E-01	1.02E-03	9.75E-03	1.96E-19	1.03E-04	1.79E-04	1.50E-05	4.20E-05	-2.68E-17	1.53E-03	1.19E-17	1.29E-16	-3.24E-04	3.76E-17	4.33E-17	-9.73E-05	-5.69E-04	-1.33E-04	-4.36E-06	-8.48E-03	-1.87E-03	-7.74E-21
	Shapiro-Wilk score	5.601E-01	4.990E-01	6.821E-01	6.587E-01	4.538E-01	3.717E-01	4.450E-01	4.673E-01	4.228E-01	5.383E-01	2.226E-01	4.614E-01	8.221E-02	5.158E-02	4.754E-01	3.190E-01	3.014E-01	4.745E-01	5.035E-01	5.090E-01	3.916E-01	6.242E-01	7.440E-01	7.924E-01
(Hexokinase)	Local	1.25E-02	5.36E-03	9.73E-01	1.37E-03	1.65E-02	-4.14E-17	2.30E-04	5.02E-04	4.31E-05	1.30E-04	-1.06E-16	4.60E-03	0.00E+00	0.00E+00	-7.83E-04	-6.57E-17	-3.32E-18	-1.88E-04	-9.78E-04	-2.78E-04	-2.22E-05	-9.81E-03	-2.24E-03	1.36E-18
	Min	-2.29E-02	-2.22E-02	-6.46E-02	6.14E-05	9.44E-04	-1.59E-14	-5.65E-03	-9.00E-03	-9.26E-04	-6.03E-03	-3.97E-14	-1.05E-01	-2.81E-14	-9.86E-14	-5.59E-02	-5.57E-14	-4.91E-14	-1.46E-02	-4.82E-02	-1.45E-02	-3.39E-03	-9.71E-02	-4.76E-02	-3.77E-17
	Max	9.88E-01	5.10E-01	1.01E+00	2.76E-02	9.33E-01	1.03E-14	2.14E-02	3.49E-02	5.32E-03	9.54E-03	5.35E-14	2.84E-01	4.55E-14	1.13E-13	1.65E-02	8.53E-14	3.86E-14	4.68E-03	1.74E-02	5.01E-03	7.19E-04	3.10E-01	3.93E-02	5.90E-17
	Normalized peak height	1.05E-01	6.99E-02	2.68E-02	1.54E-02	4.15E-02	3.86E-01	8.07E-02	5.54E-02	1.02E-01	8.75E-02	5.05E-01	6.15E-02	8.32E-01	8.58E-01	5.80E-02	4.07E-01	3.73E-01	6.26E-02	4.29E-02	4.55E-02	1.36E-01	2.42E-02	2.16E-02	2.73E-01
	Peak sensitivity value	1.85E-03	1.51E-03	9.97E-01	7.64E-04	7.94E-03	-3.42E-18	1.01E-04	1.89E-04	1.43E-05	4.88E-05	2.91E-17	1.57E-03	-2.10E-17	8.06E-17	-3.59E-04	-4.44E-17	3.45E-17	-8.19E-05	-5.32E-04	-1.35E-04	-6.04E-06	-8.64E-03	-1.90E-03	-4.74E-20
	Shapiro-Wilk score	5.544E-01	5.040E-01	6.817E-01	6.661E-01	4.546E-01	3.341E-01	4.457E-01	4.696E-01	4.204E-01	5.380E-01	2.196E-01	4.663E-01	6.268E-02	5.532E-02	4.775E-01	3.381E-01	3.171E-01	4.812E-01	5.048E-01	5.130E-01	3.971E-01	6.266E-01	7.424E-01	8.271E-01
(Phosphoglucoisomerase)	Local	1.27E-02	5.48E-03	9.94E-01	3.42E-03	4.14E-02	4.62E-16	-6.63E-03	-1.45E-02	-1.24E-03	-3.75E-03	2.63E-15	-1.33E-01	0.00E+00	0.00E+00	2.26E-02	1.83E-15	9.24E-17	5.43E-03	2.82E-02	8.02E-03	6.39E-04	-2.82E-02	6.46E-02	3.55E-18
	Min	-6.74E-04	-7.96E-04	-8.99E-02	3.28E-05	1.13E-02	-1.77E-14	-3.82E-02	-6.44E-02	-6.69E-03	-2.51E-02	-6.26E-14	-4.71E-01	-5.70E-14	-1.60E-13	-3.02E-04	-9.30E-14	-6.22E-14	-1.36E-04	-4.16E-04	-1.23E-04	-1.22E-05	-1.45E-01	-4.15E-04	-4.98E-17
	Max	1.20E+00	5.79E-01	1.29E+00	3.84E-02	1.02E+00	1.72E-14	8.42E-05	1.86E-04	2.09E-05	6.15E-05	9.49E-14	2.45E-03	4.51E-14	3.43E-13	9.51E-02	7.16E-14	6.34E-14	2.24E-02	6.18E-02	2.17E-02	5.03E-03	3.13E-01	1.64E-01	1.28E-16
	Normalized peak height	1.32E-01	8.19E-02	8.80E-03	1.13E-02	2.42E-02	7.01E-02	6.35E-03	5.14E-03	6.20E-03	7.92E-03	2.12E-01	4.92E-03	4.49E-01	6.70E-01	4.74E-03	6.17E-02	6.94E-02	4.72E-03	3.29E-03	3.95E-03	8.40E-03	1.64E-02	4.34E-03	2.76E-01
	Peak sensitivity value	1.12E-03	1.23E-03	9.48E-01	2.89E-03	2.70E-02	-9.07E-18	-3.11E-03	-9.34E-03	-5.33E-04	-2.64E-05	-1.04E-17	-6.46E-02	-4.81E-17	1.66E-16	1.59E-02	5.47E-17	3.05E-17	4.07E-03	1.87E-02	6.01E-03	1.47E-04	-2.99E-02	1.62E-04	-8.87E-20
	Shapiro-Wilk score	5.586E-01	4.901E-01	7.386E-01	7.874E-01	4.991E-01	9.414E-01	9.284E-01	9.698E-01	9.171E-01	8.548E-01	6.872E-01	9.245E-01	3.564E-01	2.806E-01	9.645E-01	8.459E-01	8.023E-01	9.622E-01	9.939E-01	9.909E-01	8.147E-01	7.079E-01	9.872E-01	9.066E-01
(Phosphofructokinase)	Local	1.27E-02	5.48E-03	9.94E-01	3.42E-03	4.14E-02	1.39E-15	-6.63E-03	-1.45E-02	-1.24E-03	-3.75E-03	3.85E-15	-1.33E-01	0.00E+00	0.00E+00	2.26E-02	2.02E-15	1.13E-16	5.43E-03	2.82E-02	8.02E-03	6.39E-04	-2.82E-02	6.46E-02	3.55E-18
	Min	-2.75E-01	-9.19E-01	-8.62E-02	-1.32E-02	-5.48E-01	-1.52E-14	-3.18E-02	-6.32E-02	-6.27E-03	-2.56E-02	-6.20E-14	-4.79E-01	-7.55E-14	-2.01E-13	8.44E-06	-7.20E-14	-9.42E-14	2.07E-06	1.71E-05	4.21E-06	1.51E-07	-5.29E-01	6.02E-06	-1.25E-16
	Max	1.19E+00	5.66E-01	3.34E+00	4.02E-02	9.99E-01	1.61E-14	-1.38E-06	-8.23E-06	-3.11E-07	-1.79E-07	1.14E-13	-7.13E-05	4.82E-14	2.43E-13	8.88E-02	8.17E-14	9.79E-14	2.17E-02	6.91E-02	2.00E-02	5.55E-03	3.07E-01	1.57E-01	1.36E-16
	Normalized peak height	1.53E-01	1.65E-01	2.18E-02	1.54E-02	3.60E-02	4.83E-02	5.50E-03	4.87E-03	5.92E-03	1.29E-02	2.15E-01	4.72E-03	4.86E-01	7.00E-01	4.67E-03	6.45E-02	9.85E-02	4.54E-03	3.45E-03	3.81E-03	9.10E-03	2.77E-02	4.67E-03	2.88E-01
	Peak sensitivity value	1.12E-03	2.26E-03	9.53E-01	2.78E-03	2.83E-02	5.76E-18	-3.35E-03	-9.26E-03	-7.31E-04	-1.30E-05	-1.44E-17	-6.20E-02	2.51E-17	4.48E-17	1.61E-02	2.34E-17	6.05E-18	3.50E-03	2.06E-02	6.09E-03	1.31E-04	-2.88E-02	8.44E-05	1.77E-20
	Shapiro-Wilk score	5.551E-01	4.821E-01	7.365E-01	7.880E-01	4.975E-01	8.712E-01	9.304E-01	9.704E-01	9.212E-01	8.523E-01	7.311E-01	9.253E-01	3.428E-01	2.760E-01	9.661E-01	8.497E-01	7.907E-01	9.630E-01	9.934E-01	9.911E-01	8.126E-01	7.007E-01	9.862E-01	8.738E-01

(Aldolase)												
Local	1.27E-02	5.48E-03	9.94E-01	3.42E-03	4.14E-02	1.11E-15	-6.63E-03	-1.45E-02	-1.24E-03	-3.75E-03	-1.02E-14	-1.33E-01
Min	-3.15E-03	-2.20E-03	-1.75E+00	2.07E-05	1.22E-02	-4.04E-14	-3.14E-02	-4.71E-02	-6.63E-03	-2.65E-02	-1.24E-13	-4.99E-01
Max	1.24E+00	6.35E-01	1.26E+00	4.01E-02	1.79E+00	5.12E-14	1.02E-03	6.27E-03	1.57E-03	7.48E-04	1.69E-13	1.40E-01
Normalized peak height	1.40E-01	9.08E-02	1.91E-02	1.13E-02	4.03E-02	5.66E-02	5.31E-03	4.20E-03	7.95E-03	9.20E-03	1.82E-01	6.32E-03
Peak sensitivity value	1.20E-03	1.30E-03	9.40E-01	2.72E-03	2.73E-02	-2.48E-16	-3.54E-03	-9.55E-03	-6.89E-04	-2.70E-05	1.44E-16	-6.49E-02
Shapiro-Wilk score	5.590E-01	4.842E-01	7.409E-01	7.874E-01	5.024E-01	7.351E-01	9.298E-01	9.688E-01	9.190E-01	8.479E-01	8.369E-01	9.227E-01
(Triosephosphate isomerase)												
Local	1.01E-02	4.37E-03	7.92E-01	2.32E-03	2.81E-02	-1.03E-15	9.18E-03	2.01E-02	1.72E-03	5.20E-03	-5.10E-15	1.84E-01
Min	-3.15E-03	-3.19E-03	-1.81E-01	-1.20E-03	-4.11E-02	-2.30E-14	4.89E-04	1.61E-03	1.35E-04	1.16E-04	-9.36E-14	2.26E-02
Max	9.12E-01	4.54E-01	9.40E-01	2.86E-02	9.31E-01	1.74E-14	6.12E-02	1.10E-01	1.49E-02	4.47E-02	8.93E-14	8.70E-01
Normalized peak height	1.26E-01	7.89E-02	6.00E-03	1.17E-02	2.85E-02	3.99E-02	7.02E-03	5.60E-03	8.30E-03	9.05E-03	1.87E-01	5.55E-03
Peak sensitivity value	9.69E-04	1.15E-03	7.81E-03	2.07E-03	2.06E-02	-1.05E-17	5.19E-03	1.29E-02	9.97E-04	1.83E-03	-4.14E-17	7.72E-02
Shapiro-Wilk score	5.505E-01	4.717E-01	7.962E-01	8.023E-01	4.927E-01	7.806E-01	8.801E-01	9.020E-01	8.530E-01	8.173E-01	8.551E-01	8.449E-01
(Glyceraldehyde 3-phosphate dehydrogenase)												
Local	1.16E-02	4.98E-03	9.03E-01	2.92E-03	3.54E-02	-1.85E-16	5.23E-04	1.14E-03	9.81E-05	2.96E-04	-9.79E-15	1.05E-02
Min	-2.15E-03	-2.72E-03	-1.31E-01	1.27E-05	1.06E-02	-5.14E-14	-6.65E-03	-8.13E-03	-1.92E-03	-5.07E-03	-1.07E-13	-9.29E-02
Max	9.38E-01	4.92E-01	9.63E-01	2.74E-02	9.63E-01	9.54E-14	2.54E-02	4.62E-02	7.58E-03	8.75E-03	6.16E-14	3.05E-01
Normalized peak height	1.17E-01	7.37E-02	1.08E-02	1.01E-02	2.51E-02	1.00E-01	4.56E-02	3.47E-02	7.28E-02	3.70E-02	1.51E-01	3.11E-02
Peak sensitivity value	1.14E-03	9.87E-04	9.03E-01	2.55E-03	2.63E-02	6.22E-17	2.87E-04	5.40E-04	5.33E-05	1.32E-04	6.38E-17	4.76E-03
Shapiro-Wilk score	5.572E-01	4.861E-01	7.012E-01	7.934E-01	5.006E-01	7.063E-01	5.019E-01	5.356E-01	4.894E-01	6.314E-01	8.670E-01	5.336E-01
(Phosphoenolpyruvate synthesis)												
Local	1.16E-02	4.98E-03	9.03E-01	2.92E-03	3.54E-02	-2.35E-16	5.23E-04	1.14E-03	9.81E-05	2.96E-04	-4.52E-14	1.05E-02
Min	-2.64E-03	-4.74E-03	-3.89E-01	4.73E-05	1.02E-02	-2.56E-13	-6.21E-03	-8.26E-03	-1.13E-03	-4.23E-03	-5.93E-13	-8.42E-02
Max	9.36E-01	4.96E-01	9.63E-01	3.01E-02	9.73E-01	2.87E-13	2.51E-02	4.46E-02	5.14E-03	1.05E-02	3.86E-13	2.98E-01
Normalized peak height	1.11E-01	7.48E-02	1.33E-02	1.01E-02	2.64E-02	4.00E-02	4.53E-02	3.32E-02	4.90E-02	3.91E-02	1.58E-01	3.01E-02
Peak sensitivity value	1.58E-03	1.02E-03	9.02E-01	2.56E-03	2.51E-02	2.24E-16	2.62E-04	6.00E-04	5.20E-05	1.25E-04	-2.81E-16	4.61E-03
Shapiro-Wilk score	5.550E-01	4.852E-01	7.009E-01	7.899E-01	5.047E-01	6.439E-01	5.102E-01	5.399E-01	4.948E-01	6.209E-01	8.558E-01	5.341E-01
(Pyruvate kinase)												
Local	1.16E-02	4.98E-03	9.03E-01	2.92E-03	3.54E-02	2.24E-15	5.23E-04	1.14E-03	9.81E-05	2.96E-04	5.94E-14	1.05E-02
Min	-1.53E-02	-2.58E-02	-6.41E-02	3.10E-05	8.64E-03	-1.45E-13	-5.82E-03	-9.10E-03	-9.18E-04	-3.91E-03	-4.53E-13	-8.64E-02
Max	9.42E-01	5.10E-01	9.74E-01	3.14E-02	9.64E-01	1.30E-13	2.61E-02	4.90E-02	7.15E-03	1.08E-02	4.41E-13	3.04E-01
Normalized peak height	1.17E-01	7.98E-02	1.05E-02	1.12E-02	2.62E-02	1.08E-01	4.72E-02	3.59E-02	6.40E-02	4.11E-02	3.20E-01	3.10E-02
Peak sensitivity value	1.47E-03	1.23E-03	9.03E-01	2.46E-03	2.54E-02	-8.92E-17	2.33E-04	6.26E-04	5.39E-05	1.15E-04	-3.47E-16	5.07E-03
Shapiro-Wilk score	5.562E-01	4.870E-01	7.002E-01	7.864E-01	4.992E-01	6.786E-01	5.089E-01	5.358E-01	4.919E-01	6.245E-01	7.410E-01	5.314E-01
(Pyruvate decarboxylase)												
Local	1.16E-02	4.98E-03	9.03E-01	2.92E-03	3.54E-02	-8.93E-16	5.23E-04	1.14E-03	9.81E-05	2.96E-04	-6.66E-16	1.05E-02
Min	-1.53E-02	-9.46E-03	-3.23E-01	6.61E-05	9.90E-03	-1.23E-14	-3.42E-03	-8.01E-03	-7.43E-04	-2.89E-03	-5.71E-14	-1.03E-01
Max	9.42E-01	5.05E-01	9.60E-01	2.81E-02	9.60E-01	1.37E-14	2.52E-02	4.02E-02	4.90E-03	8.49E-03	6.66E-14	3.00E-01
Normalized peak height	1.23E-01	7.91E-02	1.25E-02	9.79E-03	2.57E-02	6.52E-02	4.21E-02	3.11E-02	4.43E-02	3.01E-02	1.19E-01	3.27E-02
Peak sensitivity value	1.42E-03	1.09E-03	9.01E-01	2.47E-03	2.46E-02	-5.49E-18	2.81E-04	6.06E-04	4.43E-05	1.20E-04	-2.35E-16	4.45E-03
Shapiro-Wilk score	5.543E-01	4.849E-01	7.006E-01	7.886E-01	5.013E-01	7.517E-01	4.950E-01	5.363E-01	4.916E-01	6.311E-01	6.380E-01	5.329E-01
(Alcohol dehydrogenase)												
Local	1.01E-02	4.37E-03	7.92E-01	2.32E-03	2.81E-02	-3.80E-16	9.18E-03	2.01E-02	1.72E-03	5.20E-03	6.30E-16	1.84E-01
Min	-6.70E-04	-7.55E-04	-1.25E-01	-1.04E-03	-1.09E-02	-6.06E-15	5.27E-04	1.45E-03	1.29E-04	1.50E-04	-2.99E-14	2.50E-02
Max	9.19E-01	4.51E-01	9.38E-01	2.80E-02	9.17E-01	8.15E-15	5.42E-02	1.01E-01	1.72E-02	5.26E-02	3.79E-14	8.59E-01
Normalized peak height	1.20E-01	7.70E-02	5.95E-03	1.14E-02	2.75E-02	3.96E-02	6.38E-03	5.15E-03	1.03E-02	1.05E-02	1.60E-01	5.66E-03
Peak sensitivity value	1.63E-03	1.28E-03	4.58E-03	2.11E-03	2.20E-02	3.76E-18	4.52E-03	1.15E-02	8.39E-04	1.65E-03	2.36E-17	8.79E-02
Shapiro-Wilk score	5.523E-01	4.671E-01	7.957E-01	7.999E-01	4.927E-01	7.739E-01	8.780E-01	9.085E-01	8.473E-01	8.134E-01	7.864E-01	8.457E-01
(Ethanol out)												
Local	1.01E-02	4.37E-03	7.92E-01	2.32E-03	2.81E-02	-4.21E-16	9.18E-03	2.01E-02	1.72E-03	5.20E-03	5.13E-16	1.84E-01
Min	-6.11E-03	-5.45E-03	-1.23E-01	-1.22E-03	-9.26E-03	-7.38E-15	5.59E-04	1.58E-03	1.19E-04	1.71E-04	-2.42E-14	2.75E-02
Max	8.99E-01	4.42E-01	9.49E-01	2.49E-02	9.30E-01	7.63E-15	5.74E-02	1.14E-01	1.42E-02	4.64E-02	3.78E-14	8.82E-01
Normalized peak height	1.18E-01	7.76E-02	5.90E-03	1.06E-02	2.72E-02	1.73E-01	6.35E-03	5.81E-03	8.51E-03	9.27E-03	4.25E-01	5.32E-03
Peak sensitivity value	1.58E-03	1.05E-03	4.96E-03	2.02E-03	2.31E-02	-3.29E-18	4.68E-03	1.38E-02	9.16E-04	2.04E-03	1.01E-17	7.92E-02
Shapiro-Wilk score	5.508E-01	4.711E-01	7.954E-01	7.995E-01	4.941E-01	7.675E-01	8.810E-01	9.030E-01	8.529E-01	8.147E-01	7.277E-01	8.444E-01
(Ethanol flow)												
Local	1.01E-02	4.37E-03	7.92E-01	2.32E-03	2.81E-02	-3.81E-16	9.18E-03	2.01E-02	1.72E-03	5.20E-03	-1.74E-16	1.84E-01
Min	-2.20E-02	-1.57E-02	-7.67E-02	-9.61E-04	-1.09E-02	-1.06E-14	5.64E-04	1.41E-03	1.35E-04	1.17E-04	-5.20E-14	2.23E-02
Max	9.04E-01	4.50E-01	9.39E-01	2.79E-02	9.18E-01	9.25E-15	5.45E-02	1.14E-01	1.71E-02	5.01E-02	6.49E-14	8.59E-01
Normalized peak height	1.21E-01	7.79E-02	5.76E-03	1.15E-02	2.82E-02	7.36E-02	6.11E-03	6.18E-03	1.04E-02	1.03E-02	2.53E-01	5.34E-03
Peak sensitivity value	1.62E-03	1.25E-03	5.06E-03	2.12E-03	2.12E-02	3.41E-18	5.23E-03	1.27E-02	8.75E-04	2.19E-03	3.82E-17	9.13E-02
Shapiro-Wilk score	5.529E-01	4.760E-01	7.959E-01	8.057E-01	4.976E-01	7.363E-01	8.811E-01	9.065E-01	8.479E-01	8.161E-01	6.026E-01	8.474E-01
(Glycerol synthesis)												
Local	2.51E-02	1.08E-02	1.96E+00	8.63E-03	1.04E-01	8.06E-15	-8.18E-02	-1.79E-01	-1.53E-02	-4.63E-02	1.65E-14	-1.64E+00
Min	-3.23E-01	-2.84E-02	-2.06E+00	8.90E-05	2.57E-02	-1.22E-13	-3.82E-01	-4.52E-01	-5.28E-02	-2.53E-01	-5.81E-13	-3.08E+00
Max	3.07E+00	1.74E+00	3.25E+00	8.42E-02	2.69E+00	1.24E-13	-1.56E-03	-1.11E-02	-8.57E-04	-3.86E-04	4.71E-13	-6.37E-01
Normalized peak height	1.67E-01	1.36E-01	1.14E-02	1.10E-02	2.69E-02	5.91E-02	4.32E-03	2.72E-03	4.43E-03	6.03E-03	2.30E-01	4.12E-03
Peak sensitivity value	4.33E-03	2.51E-03	1.06E-02	7.03E-03	6.16E-02	1.85E-17	-6.91E-02	-1.44E-01	-1.03E-02	-2.30E-02	5.15E-16	-1.37E+00
Shapiro-Wilk score	5.575E-01	4.765E-01	8.793E-01	8.092E-01	5.145E-01	8.219E-01	9.459E-01	9.879E-01	9.516E-01	9.027E-01	6.069E-01	9.647E-01
(Glycerol out)												
Local	2.51E-02	1.08E-02	1.96E+00	8.63E-03	1.04E-01	8.12E-15	-8.18E-02	-1.79E-01	-1.53E-02	-4.63E-02	1.55E-14	-1.64E+00
Min	-1.37E-01	-3.54E-02	-2.68E-01	1.42E-04	2.37E-02	-1.12E-13	-3.60E-01	-4.66E-01	-5.39E-02	-2.71E-01	-4.80E-13	-3.12E+00
Max	3.02E+00	1.17E+00	3.34E+00	7.80E-02	2.84E+00	1.23E-13	-1.47E-03	-1.42E-02	-6.51E-04	-5.48E-04	4.22E-13	-7.00E-01
Normalized peak height	1.83E-01	9.06E-02	8.61E-03	9.90E-03	2.88E-02	6.06E-02	4.16E-03	2.97E-03	4.47E-03	5.85E-03	2.27E-01	4.23E-03
Peak sensitivity value	3.31E-03	2.73E-03	1.23E-02	6.33E-03	6.17E-02	6.46E-17	-5.55E-02	-1.77E-01	-1.04E-02	-2.61E-02	1.48E-18	-1.37E+00
Shapiro-Wilk score	5.648E-01	4.734E-01	8.799E-01	8.102E-01	5.174E-01	8.199E-01	9.460E-01	9.879E-01	9.517E-01	9.041E-01	6.038E-01	9.636E-01
(Glycerol flow)												
Local	2.51E-02	1.08E-02	1.96E+00	8.63E-03	1.04E-01	8.14E-15	-8.18E-02	-1.79E-01	-1.53E-02	-4.63E-02	1.49E-14	-1.64E+00
Min	-7.83E-02	-3.97E-02	-7.29E-01	4.75E-05	2.29E-02	-1.20E-13	-3.97E-01	-4.69E-01	-5.25E-02	-2.32E-01	-7.23E-13	-3.14E+00
Max	3.04E+00	1.34E+00	3.40E+00	7.89E-02	2.82E+00	8.61E-14	-1.39E-03	-1.40E-02	-4.24E-04	-6.31E-04	1.01E-12	-7.42E-01
Normalized peak height	1.54E-01	1.10E-01	9.89E-03	9.80E-03	2.86E-02	5.74E-02	4.39E-03	2.90E-03	4.92E-03	5.19E-03	2.86E-01	4.06E-03
Peak sensitivity value	4.28E-03	2.32E-03	8.44E-03	6.24E-03	6.34E-02	1.10E-17	-6.84E-02	-1.93E-01	-1.08E-02	-3.03E-02	4.41E-16	-1.34E+00
Shapiro-Wilk score	5.621E-01	4.762E-01	8.786E-01	8.191E-01	5.120E-01	8.382E-01	9.453E-01	9.883E-01	9.523E-01	9.039E-01	5.725E-01	9.648E-01

(Aldolase)												
Local	0.00E+00	0.00E+00	2.26E-02	3.98E-16	1.24E-16	5.43E-03	2.82E-02	8.02E-03	6.39E-04	-2.82E-02	6.46E-02	3.55E-18
Min	-9.61E-14	-2.67E-13	-8.25E-03	-1.11E-13	-8.78E-14	-2.37E-03	-1.64E-02	-3.08E-03	-4.63E-04	-1.46E-01	-1.99E-02	-4.86E-17
Max	1.09E-13	2.33E-13	8.43E-02	8.68E-14	6.05E-14	1.98E-02	6.78E-02	2.31E-02	6.13E-03	3.18E-01	1.59E-01	1.77E-16
Normalized peak height	5.38E-01	6.93E-01	5.11E-03	6.33E-02	7.27E-02	4.62E-03	4.26E-03	4.64E-03	1.04E-02	1.55E-02	4.67E-03	2.83E-01
Peak sensitivity value	6.18E-17	-1.46E-16	1.24E-02	-2.03E-17	3.86E-17	3.96E-03	2.02E-02	6.28E-03	1.86E-04	-2.86E-02	1.02E-04	-8.49E-20
Shapiro-Wilk score	3.007E-01	2.875E-01	9.642E-01	8.503E-01	7.994E-01	9.626E-01	9.935E-01	9.909E-01	8.117E-01	6.967E-01	9.875E-01	8.820E-01
(Triosephosphate isomerase)												
Local	0.00E+00	0.00E+00	-3.13E-02	-1.10E-17	1.39E-15	-7.52E-03	-3.91E-02	-1.11E-02	-8.85E-04	-1.89E-02	5.21E-02	2.13E-18
Min	-3.77E-14	-9.05E-14	-1.67E-01	-4.41E-14	-4.08E-14	-4.09E-02	-1.27E-01	-3.67E-02	-1.14E-02	-1.35E-01	-1.14E-04	-6.25E-17
Max	3.02E-14	7.92E-14	-2.40E-03	4.71E-14	2.97E-14	-8.62E-04	-8.67E-03	-2.31E-03	-2.05E-05	2.59E-01	1.47E-01	1.31E-16
Normalized peak height	4.95E-01	5.65E-01	5.22E-03	5.49E-02	6.42E-02	5.53E-03	3.70E-03	4.08E-03	1.20E-02	1.31E-02	4.35E-03	2.75E-01
Peak sensitivity value	-2.01E-18	1.60E-17	-1.96E-02	1.33E-18	-2.08E-17	-4.92E-03	-2.83E-02	-9.41E-03	-2.43E-04	-1.98E-02	1.06E-04	2.27E-20
Shapiro-Wilk score	3.938E-01	3.155E-01	9.000E-01	7.777E-01	7.489E-01	9.047E-01	9.444E-01	9.499E-01	7.683E-01	7.385E-01	9.931E-01	8.773E-01
(Glyceraldehyde 3-phosphate dehydrogenase)												
Local	0.00E+00	0.00E+00	-1.78E-03	7.65E-16	6.61E-16	-4.28E-04	-2.23E-03	-6.32E-04	-5.04E-05	-2.40E-02	5.89E-02	2.91E-18
Min	-3.40E-14	-2.03E-13	-6.97E-02	-4.35E-14	-5.25E-14	-1.53E-02	-5.06E-02	-1.53E-02	-5.11E-03	-1.32E-01	-9.32E-06	-7.10E-17
Max	4.59E-14	1.41E-13	1.84E-02	4.66E-14	5.51E-14	3.65E-03	1.52E-02	4.38E-03	6.46E-04	2.75E-01	1.57E-01	1.57E-16
Normalized peak height	4.50E-01	6.82E-01	3.41E-02	3.92E-02	6.53E-02	3.00E-02	2.17E-02	2.28E-02	9.12E-02	1.43E-02	4.12E-03	2.85E-01
Peak sensitivity value	-2.37E-17	5.76E-17	-8.94E-04	-7.84E-18	-4.91E-17	-2.91E-04	-1.42E-03	-3.83E-04	-1.34E-05	-2.32E-02	2.26E-04	5.31E-20
Shapiro-Wilk score	3.476E-01	2.688E-01	5.398E-01	8.593E-01	7.896E-01	5.575E-01	5.758E-01	5.849E-01	4.630E-01	7.102E-01	9.903E-01	8.534E-01
(Phosphoenolpyruvate synthesis)												
Local	0.00E+00	0.00E+00	-1.78E-03	-2.42E-15	1.16E-15	-4.28E-04	-2.23E-03	-6.32E-04	-5.04E-05	-2.40E-02	5.89E-02	2.91E-18
Min	-3.28E-13	-2.89E-13	-7.32E-02	-1.21E-13	-5.85E-14	-1.62E-02	-5.04E-02	-1.72E-02	-4.04E-03	-1.42E-01	-9.99E-06	-9.53E-17
Max	4.14E-13	1.97E-13	1.71E-02	8.06E-14	5.71E-14	2.26E-03	1.01E-02	4.15E-03	6.81E-04	2.88E-01	1.52E-01	1.68E-16
Normalized peak height	6.79E-01	7.63E-01	3.58E-02	6.31E-02	7.70E-02	2.85E-02	2.00E-02	2.53E-02	7.30E-02	1.52E-02	4.37E-03	2.82E-01
Peak sensitivity value	3.23E-16	3.62E-17	-9.92E-04	9.88E-17	5.25E-17	-2.83E-04	-1.33E-03	-4.43E-04	-1.45E-05	-2.41E-02	6.58E-05	3.19E-20
Shapiro-Wilk score	2.751E-01	2.698E-01	5.428E-01	8.446E-01	7.771E-01	5.589E-01	5.790E-01	5.855E-01	4.662E-01	7.067E-01	9.894E-01	8.609E-01
(Pyruvate kinase)												
Local	0.00E+00	0.00E+00	-1.78E-03	4.02E-15	-8.26E-16	-4.28E-04	-2.23E-03	-6.32E-04	-5.04E-05	-2.40E-02	5.89E-02	2.91E-18
Min	-2.06E-13	-1.68E-13	-6.83E-02	-1.27E-13	-8.03E-14	-1.61E-02	-5.16E-02	-1.55E-02	-4.63E-03	-1.40E-01	-1.39E-05	-5.96E-17
Max	2.55E-13	1.39E-13	1.88E-02	6.51E-14	8.81E-14	3.66E-03	1.16E-02	3.21E-03	8.24E-04	2.86E-01	1.54E-01	2.56E-16
Normalized peak height	6.82E-01	6.64E-01	3.43E-02	7.89E-02	1.12E-01	3.03E-02	2.15E-02	2.18E-02	8.70E-02	1.45E-02	4.20E-03	2.87E-01
Peak sensitivity value	1.40E-16	-9.17E-17	-1.09E-03	-8.76E-17	-6.82E-17	-3.02E-04	-1.45E-03	-4.05E-04	-1.34E-05	-2.42E-02	6.31E-05	-2.18E-20
Shapiro-Wilk score	2.469E-01	2.712E-01	5.442E-01	7.991E-01	7.115E-01	5.509E-01	5.747E-01	5.821E-01	4.565E-01	7.130E-01	9.896E-01	8.611E-01
(Pyruvate decarboxylase)												
Local	0.00E+00	0.00E+00	-1.78E-03	-6.65E-16	1.91E-15	-4.28E-04	-2.23E-03	-6.32E-04	-5.04E-05	-2.40E-02	5.89E-02	2.91E-18
Min	-2.90E-14	-1.48E-13	-6.74E-02	-1.33E-13	-5.68E-14	-1.43E-02	-4.92E-02	-1.66E-02	-4.08E-03	-1.31E-01	-1.22E-05	-7.94E-17
Max	4.13E-14	2.94E-13	1.22E-02	7.58E-14	8.24E-14	2.62E-03	1.34E-02	3.27E-03	4.90E-04	2.95E-01	1.51E-01	1.98E-15
Normalized peak height	4.82E-01	6.86E-01	3.20E-02	1.12E-01	1.03E-01	2.62E-02	2.09E-02	2.43E-02	7.12E-02	1.49E-02	3.99E-03	3.26E-01
Peak sensitivity value	1.55E-17	-2.00E-16	-9.73E-04	3.28E-18	6.78E-17	-2.35E-04	-1.31E-03	-4.23E-04	-1.45E-05	-2.51E-02	2.14E-04	-9.49E-20
Shapiro-Wilk score	3.963E-01	2.655E-01	5.404E-01	7.498E-01	6.961E-01	5.550E-01	5.753E-01	5.825E-01	4.599E-01	7.060E-01	9.897E-01	1.765E-01
(Alcohol dehydrogenase)												
Local	0.00E+00	0.00E+00	-3.13E-02	-1.84E-16	1.26E-15	-7.52E-03	-3.91E-02	-1.11E-02	-8.85E-04	-1.89E-02	5.21E-02	2.13E-18
Min	-4.34E-14	-1.24E-13	-1.76E-01	-5.42E-14	-5.01E-14	-3.99E-02	-1.36E-01	-3.50E-02	-1.14E-02	-1.22E-01	-4.24E-05	-3.88E-17
Max	2.34E-14	8.87E-14	-2.38E-03	3.69E-14	3.53E-14	-7.74E-04	-7.92E-03	-2.29E-03	-1.83E-05	2.72E-01	1.45E-01	1.18E-16
Normalized peak height	4.76E-01	6.07E-01	5.42E-03	6.57E-02	9.98E-02	5.60E-03	3.94E-03	3.67E-03	1.19E-02	1.32E-02	3.81E-03	2.77E-01
Peak sensitivity value	-2.26E-17	-3.31E-17	-1.79E-02	-4.16E-17	-1.59E-17	-5.68E-03	-2.39E-02	-9.37E-03	-2.98E-04	-2.20E-02	1.76E-04	6.93E-21
Shapiro-Wilk score	4.377E-01	3.030E-01	9.028E-01	7.659E-01	7.392E-01	9.085E-01	9.432E-01	9.522E-01	7.674E-01	7.310E-01	9.937E-01	9.096E-01
(Ethanol out)												
Local	2.37E-16	-8.88E-16	-3.13E-02	-1.07E-16	1.31E-15	-7.52E-03	-3.91E-02	-1.11E-02	-8.85E-04	-1.89E-02	5.21E-02	2.13E-18
Min	-2.50E-14	-9.33E-14	-1.72E-01	-5.87E-14	-5.63E-14	-3.94E-02	-1.30E-01	-3.55E-02	-1.13E-02	-1.42E-01	-6.85E-05	-8.07E-17
Max	2.24E-14	7.28E-14	-2.56E-03	5.21E-14	4.50E-14	-8.05E-04	-8.15E-03	-2.47E-03	-1.96E-05	2.75E-01	1.51E-01	1.42E-16
Normalized peak height	6.00E-02	4.53E-01	5.37E-03	8.13E-02	1.08E-01	5.26E-03	3.74E-03	3.99E-03	1.27E-02	1.38E-02	4.34E-03	2.78E-01
Peak sensitivity value	-2.28E-16	4.42E-19	-2.01E-02	-2.45E-17	-3.37E-17	-5.61E-03	-2.64E-02	-8.61E-03	-2.74E-04	-2.40E-02	1.59E-04	-8.10E-20
Shapiro-Wilk score	8.752E-01	4.616E-01	8.997E-01	7.747E-01	7.417E-01	9.100E-01	9.449E-01	9.510E-01	7.747E-01	7.374E-01	9.935E-01	8.161E-01
(Ethanol flow)												
Local	-3.41E-16	-1.11E-15	-3.13E-02	-1.73E-16	1.29E-15	-7.52E-03	-3.91E-02	-1.11E-02	-8.85E-04	-1.89E-02	5.21E-02	2.13E-18
Min	-2.35E-14	-1.01E-13	-1.87E-01	-3.01E-14	-5.09E-14	-3.96E-02	-1.30E-01	-3.63E-02	-1.06E-02	-1.17E-01	-3.73E-05	-1.24E-16
Max	2.69E-14	6.62E-14	-2.37E-03	6.89E-14	4.66E-14	-9.70E-04	-8.90E-03	-2.01E-03	-2.14E-05	2.52E-01	1.46E-01	1.22E-16
Normalized peak height	3.87E-01	1.06E-01	6.22E-03	7.38E-02	1.01E-01	5.25E-03	3.99E-03	3.96E-03	1.14E-02	1.25E-02	3.79E-03	2.83E-01
Peak sensitivity value	-1.93E-17	-2.13E-16	-2.06E-02	4.08E-17	3.54E-17	-5.24E-03	-2.84E-02	-9.96E-03	-2.06E-04	-2.03E-02	3.55E-05	9.50E-21
Shapiro-Wilk score	4.481E-01	4.538E-01	9.035E-01	7.739E-01	7.207E-01	9.094E-01	9.423E-01	9.505E-01	7.680E-01	7.477E-01	9.932E-01	8.644E-01
(Glycerol synthesis)												
Local	0.00E+00	0.00E+00	2.79E-01	8.97E-15	-6.51E-15	6.70E-02	3.48E-01	9.89E-02	7.89E-03	-7.27E-02	1.24E-01	1.03E-17
Min	-5.24E-13	-3.46E-12	1.10E-02	-3.90E-13	-2.58E-13	3.68E-03	4.36E-02	5.47E-03	5.78E-04	-2.67E-01	-1.19E-02	-2.58E-16
Max	4.47E-13	2.09E-12	6.41E-01	6.98E-13	4.45E-13	2.40E-01	6.50E-01	2.98E-01	4.16E-02	6.75E-01	3.33E-01	6.00E-16
Normalized peak height	6.32E-01	8.56E-01	2.58E-03	5.68E-02	6.98E-02	3.90E-03	3.27E-03	3.70E-03	5.63E-03	1.59E-02	5.26E-03	2.69E-01
Peak sensitivity value	-3.46E-16	-1.62E-15	2.85E-01	-3.33E-16	2.18E-16	5.17E-02	3.64E-01	9.50E-02	3.84E-03	-6.48E-02	3.23E-04	-1.80E-19
Shapiro-Wilk score	2.983E-01	2.563E-01	9.923E-01	8.849E-01	8.591E-01	9.624E-01	9.976E-01	9.831E-01	8.983E-01	6.963E-01	9.869E-01	8.342E-01
(Glycerol out)												
Local	0.00E+00	0.00E+00	2.79E-01	9.24E-15	-6.55E-15	6.70E-02	3.48E-01	9.89E-02	7.89E-03	-7.27E-02	1.24E-01	1.03E-17
Min	-4.47E-13	-2.36E-12	1.27E-02	-4.68E-13	-4.29E-13	3.62E-03	3.88E-02	5.17E-03	6.23E-04	-2.58E-01	-8.27E-03	-2.14E-16
Max	5.89E-13	2.67E-12	6.26E-01	4.64E-13	3.37E-13	2.32E-01	6.37E-01	2.85E-01	3.72E-02	6.32E-01	3.45E-01	4.29E-16
Normalized peak height	6.78E-01	8.61E-01	2.48E-03	5.66E-02	6.80E-02	3.74E-03	3.19E-03	3.59E-03	5.20E-03	1.50E-02	4.87E-03	2.68E-01
Peak sensitivity value	-1.53E-16	5.76E-16	2.23E-01	1.58E-17	-3.83E-16	5.72E-02	3.33E-01	9.64E-02	4.37E-03	-7.12E-02	3.34E-05	-1.27E-19
Shapiro-Wilk score	2.960E-01	2.652E-01	9.930E-01	8.878E-01	8.450E-01	9.617E-01	9.974E-01	9.829E-01	8.969E-01	6.821E-01	9.870E-01	8.628E-01
(Glycerol flow)												
Local	0.00E+00	0.00E+00	2.79E-01	9.72E-15	-6.66E-15	6.70E-02	3.48E-01	9.89E-02	7.89E-03	-7.27E-02	1.24E-01	1.03E-17
Min	-4.96E-13	-5.39E-12	1.20E-02	-4.19E-13	-3.34E-13	3.65E-03	3.61E-02	6.19E-03	5.79E-04	-2.99E-01	-9.06E-03	-4.21E-16
Max	4.46E-13	2.74E-12	6.34E-01	5.75E-13	3.34E-13	2.33E-01	6.50E-01	2.78E-01	4.01E-02	6.72E-01	3.26E-01	9.57E-16
Normalized peak height	6.29E-01	8.62E-01	2.48E-03	5.70E-02	6.47E-02	3.81E-03	3.13E-03	3.35E-03	5.83E-03	1.69E-02	5.94E-03	2.70E-01
Peak sensitivity value	-2.33E-16	1.21E-15	2.01E-01	1.24E-16	-5.65E-17	5.71E-02	3.74E-01	9.51E-02	3.68E-03	-6.44E-02	1.67E-04	1.05E-19
Shapiro-Wilk score	3.100E-01	2.437E-01	9.929E-01	8.874E-01	8.477E-01	9.604E-01	9.976E-01	9.840E-01	8.946E-01	6.929E-01	9.873E-01	7.763E-01

(Acetaldehyde out)<br>Local<br>Min<br>Max<br>Normalized peak height<br>Peak sensitivity value<br>Shapiro-Wilk score	2.51E-02<br>-7.03E-02<br>2.91E+00<br>1.90E-01<br>2.65E-03<br>5.620E-01	1.08E-02<br>-8.15E-02<br>1.47E+00<br>1.11E-01<br>3.06E-03<br>4.768E-01	1.96E+00<br>-2.27E-01<br>3.20E+00<br>8.56E-03<br>7.79E-03<br>8.778E-01	8.63E-03<br>1.20E-04<br>7.66E-02<br>9.90E-03<br>6.43E-03<br>8.156E-01	1.04E-01<br>2.47E-02<br>2.63E+00<br>2.64E-02<br>6.26E-02<br>5.128E-01	-5.92E-15<br>-8.03E-14<br>1.15E-13<br>8.52E-02<br>-5.84E-17<br>7.895E-01	-8.18E-02<br>-4.17E-01<br>-1.15E-03<br>4.73E-03<br>-5.17E-02<br>9.480E-01	-1.79E-01<br>-4.51E-01<br>-1.49E-02<br>2.76E-03<br>-1.56E-01<br>9.881E-01	-1.53E-02<br>-5.77E-02<br>-4.28E-04<br>4.73E-03<br>-1.01E-02<br>9.504E-01	-4.63E-02<br>-2.46E-01<br>-3.49E-04<br>5.66E-03<br>-2.19E-02<br>9.016E-01	4.56E-21<br>-6.95E-13<br>4.75E-13<br>3.73E-01<br>1.80E-16<br>6.619E-01	-1.64E+00<br>-2.98E+00<br>-7.86E-01<br>3.68E-03<br>-1.37E+00<br>9.660E-01	0.00E+00<br>-4.31E-13<br>8.05E-13<br>7.27E-01<br>8.79E-17<br>3.261E-01	0.00E+00<br>-1.88E-12<br>3.21E-12<br>8.59E-01<br>7.38E-16<br>2.245E-01	2.79E-01<br>1.10E-02<br>6.23E-01<br>2.52E-03<br>2.35E-01<br>9.932E-01	-5.24E-15<br>-2.68E-13<br>2.24E-13<br>3.75E-02<br>-2.41E-16<br>9.075E-01	8.08E-15<br>-2.21E-13<br>5.04E-13<br>8.50E-02<br>1.16E-16<br>8.468E-01	6.70E-02<br>4.28E-03<br>2.25E-01<br>3.59E-03<br>5.60E-02<br>9.616E-01	3.48E-01<br>3.97E-02<br>6.32E-01<br>2.94E-03<br>3.39E-01<br>9.972E-01	9.89E-02<br>7.47E-03<br>2.95E-01<br>3.50E-03<br>8.44E-02<br>9.833E-01	7.89E-03<br>5.70E-04<br>4.05E-02<br>5.51E-03<br>4.66E-03<br>8.979E-01	-7.27E-02<br>-2.77E-01<br>7.21E-01<br>1.72E-02<br>-6.56E-02<br>6.978E-01	1.24E-01<br>-9.46E-03<br>3.74E-01<br>6.07E-03<br>3.18E-04<br>9.866E-01	1.03E-17<br>-2.93E-16<br>2.20E-15<br>2.80E-01<br>-3.12E-19<br>5.531E-01
(Acetaldehyde flow)<br>Local<br>Min<br>Max<br>Normalized peak height<br>Peak sensitivity value<br>Shapiro-Wilk score	2.55E-02<br>-2.44E-01<br>3.18E+00<br>1.70E-01<br>4.18E-03<br>5.641E-01	1.10E-02<br>-4.62E-02<br>1.42E+00<br>1.07E-01<br>1.51E-03<br>4.752E-01	1.99E+00<br>-7.23E-01<br>3.50E+00<br>9.40E-03<br>1.02E-02<br>8.858E-01	8.78E-03<br>1.01E-04<br>8.16E-02<br>9.64E-03<br>5.68E-03<br>8.053E-01	1.06E-01<br>2.61E-02<br>2.68E+00<br>2.72E-02<br>6.19E-02<br>5.121E-01	-5.73E-15<br>-9.90E-14<br>9.01E-14<br>6.70E-02<br>2.97E-17<br>7.721E-01	-8.32E-02<br>-4.28E-01<br>-1.74E-03<br>4.73E-03<br>-6.55E-02<br>9.482E-01	-1.82E-01<br>-4.71E-01<br>-1.04E-02<br>2.95E-03<br>-1.60E-01<br>9.885E-01	-1.56E-02<br>-5.38E-02<br>-7.11E-04<br>4.26E-03<br>-1.32E-02<br>9.495E-01	-4.71E-02<br>-2.55E-01<br>-6.28E-04<br>5.65E-03<br>-2.39E-02<br>9.052E-01	-8.63E-15<br>-4.59E-13<br>3.80E-13<br>2.46E-01<br>-2.61E-16<br>5.409E-01	-1.67E+00<br>-3.21E+00<br>-6.43E-01<br>4.28E-03<br>-1.30E+00<br>9.619E-01	0.00E+00<br>-4.17E-13<br>3.85E-13<br>6.03E-01<br>3.56E-16<br>2.640E-01	0.00E+00<br>-2.56E-12<br>2.49E-12<br>8.60E-01<br>6.36E-16<br>2.136E-01	2.84E-01<br>1.45E-02<br>6.37E-01<br>2.54E-03<br>2.85E-01<br>9.934E-01	-5.10E-15<br>-2.28E-13<br>2.14E-13<br>4.56E-02<br>1.77E-17<br>9.076E-01	7.94E-15<br>-2.60E-13<br>4.57E-13<br>7.47E-02<br>3.50E-16<br>8.332E-01	6.82E-02<br>4.14E-03<br>2.38E-01<br>3.99E-03<br>5.54E-02<br>9.608E-01	5.75E-01<br>1.64E-01<br>8.51E-01<br>3.28E-03<br>5.54E-01<br>9.873E-01	-1.21E-01<br>-2.86E-01<br>-3.52E-02<br>3.26E-03<br>-1.20E-01<br>9.818E-01	-9.62E-03<br>-1.26E-01<br>-3.25E-04<br>1.13E-02<br>-3.52E-03<br>7.554E-01	-7.39E-02<br>-2.68E-01<br>6.97E-01<br>1.68E-02<br>-6.65E-02<br>6.867E-01	1.27E-01<br>-2.92E-02<br>3.19E-01<br>5.89E-03<br>2.37E-04<br>9.862E-01	1.05E-17<br>-1.73E-16<br>1.68E-15<br>2.73E-01<br>-4.01E-20<br>6.422E-01
(Cyanide-Acetaldehyde flow)<br>Local<br>Min<br>Max<br>Normalized peak height<br>Peak sensitivity value<br>Shapiro-Wilk score	2.36E-02<br>-4.00E-02<br>2.68E+00<br>1.80E-01<br>2.18E-03<br>5.606E-01	1.02E-02<br>-3.88E-02<br>1.27E+00<br>1.04E-01<br>2.47E-03<br>4.798E-01	1.85E+00<br>-3.14E-01<br>2.99E+00<br>8.42E-03<br>1.18E-02<br>8.581E-01	8.13E-03<br>8.05E-05<br>7.33E-02<br>1.03E-02<br>5.83E-03<br>8.193E-01	9.84E-02<br>2.51E-02<br>2.62E+00<br>2.83E-02<br>6.02E-02<br>5.147E-01	-5.30E-15<br>-9.51E-14<br>9.59E-14<br>6.72E-02<br>8.28E-17<br>7.680E-01	-7.71E-02<br>-3.45E-01<br>-1.30E-03<br>3.89E-03<br>-5.38E-02<br>9.465E-01	-1.69E-01<br>-4.29E-01<br>-8.17E-03<br>2.73E-03<br>-1.65E-01<br>9.872E-01	-1.45E-02<br>-5.03E-02<br>-5.79E-04<br>4.35E-03<br>-1.21E-02<br>9.511E-01	-4.36E-02<br>-2.37E-01<br>-3.75E-04<br>5.69E-03<br>-1.66E-02<br>9.052E-01	-8.00E-15<br>-3.52E-13<br>3.65E-13<br>2.29E-01<br>3.58E-16<br>5.573E-01	-1.54E+00<br>-2.90E+00<br>-4.92E-01<br>4.69E-03<br>-1.34E+00<br>9.707E-01	0.00E+00<br>-4.08E-13<br>5.35E-13<br>6.82E-01<br>-8.27E-17<br>2.608E-01	0.00E+00<br>-2.95E-12<br>1.70E-12<br>7.85E-01<br>1.90E-15<br>2.077E-01	2.63E-01<br>9.56E-03<br>5.91E-01<br>2.42E-03<br>3.20E-01<br>9.919E-01	-4.73E-15<br>-2.54E-13<br>2.68E-13<br>5.16E-02<br>-9.40E-17<br>9.033E-01	7.34E-15<br>-1.51E-13<br>1.96E-13<br>5.89E-02<br>1.43E-16<br>8.652E-01	6.31E-02<br>2.98E-03<br>2.18E-01<br>3.73E-03<br>4.85E-02<br>9.638E-01	-3.93E-01<br>-7.03E-01<br>-1.39E-01<br>3.11E-03<br>-3.79E-01<br>9.965E-01	8.14E-01<br>4.48E-01<br>9.52E-01<br>4.04E-03<br>8.35E-01<br>9.619E-01	6.50E-02<br>6.84E-03<br>4.34E-01<br>6.91E-03<br>3.44E-02<br>8.554E-01	-6.85E-02<br>-2.66E-01<br>6.81E-01<br>1.70E-02<br>-6.20E-02<br>6.994E-01	1.17E-01<br>-1.42E-02<br>3.02E-01<br>6.62E-03<br>1.90E-04<br>9.861E-01	9.70E-18<br>-3.01E-16<br>6.87E-16<br>2.70E-01<br>-2.27E-19<br>8.407E-01
(Cyanide flow)<br>Local<br>Min<br>Max<br>Normalized peak height<br>Peak sensitivity value<br>Shapiro-Wilk score	2.36E-02<br>-5.59E-02<br>2.61E+00<br>1.40E-01<br>3.99E-03<br>5.600E-01	1.02E-02<br>-6.80E-02<br>1.16E+00<br>9.74E-02<br>2.59E-03<br>4.767E-01	1.85E+00<br>-1.22E-01<br>3.10E+00<br>7.75E-03<br>8.49E-03<br>8.595E-01	8.13E-03<br>7.83E-05<br>7.00E-02<br>9.38E-03<br>5.92E-03<br>8.173E-01	9.84E-02<br>2.35E-02<br>2.51E+00<br>2.80E-02<br>6.20E-02<br>5.154E-01	-5.36E-15<br>-7.20E-14<br>6.87E-14<br>5.77E-02<br>-1.15E-17<br>7.864E-01	-7.71E-02<br>-3.49E-01<br>-7.75E-04<br>4.20E-03<br>-5.50E-02<br>9.472E-01	-1.69E-01<br>-4.31E-01<br>-1.22E-02<br>2.96E-03<br>-1.59E-01<br>9.876E-01	-1.45E-02<br>-5.81E-02<br>-3.38E-04<br>5.47E-03<br>-1.04E-02<br>9.501E-01	-4.36E-02<br>-2.17E-01<br>-3.72E-04<br>5.20E-03<br>-2.19E-02<br>9.072E-01	-7.92E-15<br>-4.09E-13<br>3.80E-13<br>2.64E-01<br>-3.84E-16<br>5.505E-01	-1.54E+00<br>-3.07E+00<br>-6.20E-01<br>4.91E-03<br>-1.20E+00<br>9.694E-01	0.00E+00<br>-3.39E-13<br>3.85E-13<br>6.24E-01<br>1.11E-16<br>2.559E-01	0.00E+00<br>-1.65E-12<br>1.69E-12<br>6.70E-01<br>1.61E-15<br>2.332E-01	2.63E-01<br>7.46E-03<br>6.04E-01<br>2.56E-03<br>2.67E-01<br>9.928E-01	-4.59E-15<br>-2.21E-13<br>2.21E-13<br>4.49E-02<br>2.05E-16<br>9.045E-01	7.47E-15<br>-1.25E-13<br>1.53E-13<br>5.84E-02<br>6.42E-17<br>8.689E-01	6.31E-02<br>3.46E-03<br>2.31E-01<br>4.29E-03<br>5.60E-02<br>9.618E-01	-3.93E-01<br>-7.11E-01<br>-1.35E-01<br>3.22E-03<br>-3.53E-01<br>9.966E-01	8.14E-01<br>4.56E-01<br>9.51E-01<br>3.87E-03<br>8.33E-01<br>9.615E-01	6.50E-02<br>7.00E-03<br>4.38E-01<br>7.24E-03<br>3.61E-02<br>8.551E-01	-6.85E-02<br>-3.06E-01<br>6.29E-01<br>1.69E-02<br>-6.52E-02<br>6.970E-01	1.17E-01<br>-6.09E-03<br>3.25E-01<br>5.44E-03<br>2.33E-05<br>9.874E-01	9.70E-18<br>-4.81E-16<br>5.09E-16<br>2.71E-01<br>-1.85E-19<br>8.739E-01
(Storage)<br>Local<br>Min<br>Max<br>Normalized peak height<br>Peak sensitivity value<br>Shapiro-Wilk score	1.21E-02<br>-2.26E-02<br>1.19E+00<br>1.19E-01<br>1.11E-03<br>5.650E-01	5.20E-03<br>-2.50E-02<br>5.32E-01<br>7.48E-02<br>1.44E-03<br>5.191E-01	9.43E-01<br>-3.37E-01<br>1.31E+00<br>6.36E-03<br>1.04E+00<br>7.983E-01	-1.53E-03<br>-1.56E-02<br>2.33E-02<br>1.20E-02<br>-1.24E-03<br>8.942E-01	-1.85E-02<br>-6.22E-01<br>8.94E-01<br>3.81E-02<br>-1.48E-02<br>4.912E-01	-1.69E-15<br>-3.26E-14<br>7.20E-14<br>6.74E-02<br>7.14E-18<br>7.928E-01	9.90E-03<br>4.39E-04<br>6.82E-02<br>7.44E-03<br>5.35E-03<br>8.924E-01	2.16E-02<br>1.52E-03<br>9.84E-02<br>4.54E-03<br>1.32E-02<br>9.159E-01	1.86E-03<br>1.25E-04<br>1.26E-02<br>7.29E-03<br>8.31E-04<br>8.558E-01	5.60E-03<br>1.56E-04<br>5.73E-02<br>1.05E-02<br>2.13E-03<br>8.092E-01	-3.99E-15<br>-3.24E-13<br>1.98E-13<br>2.44E-01<br>2.36E-16<br>5.879E-01	1.98E-01<br>2.57E-02<br>9.05E-01<br>5.54E-03<br>9.55E-02<br>8.559E-01	0.00E+00<br>-3.07E-13<br>1.43E-13<br>5.82E-01<br>-1.56E-16<br>2.732E-01	0.00E+00<br>-3.95E-13<br>3.05E-13<br>6.39E-01<br>2.52E-16<br>2.675E-01	-3.37E-02<br>-2.12E-01<br>-2.79E-03<br>6.18E-03<br>-2.05E-02<br>9.074E-01	-2.74E-15<br>-2.77E-13<br>1.67E-13<br>7.55E-02<br>-5.76E-17<br>7.796E-01	-1.38E-16<br>-2.09E-13<br>1.64E-13<br>9.05E-02<br>-2.32E-17<br>7.583E-01	-8.11E-03<br>-3.62E-02<br>-1.01E-03<br>4.34E-03<br>-6.23E-03<br>9.201E-01	-4.21E-02<br>-1.22E-01<br>-8.77E-03<br>3.43E-03<br>-2.95E-02<br>9.516E-01	-1.20E-02<br>-3.72E-02<br>-2.48E-03<br>3.88E-03<br>-9.02E-03<br>9.539E-01	-9.55E-04<br>-1.23E-02<br>-1.70E-05<br>1.22E-02<br>-2.31E-04<br>7.767E-01	1.62E-02<br>-6.60E-02<br>3.38E-01<br>1.15E-02<br>1.87E-02<br>7.077E-01	-9.65E-02<br>-3.47E-01<br>-1.09E-02<br>3.94E-03<br>-9.40E-02<br>9.665E-01	-1.73E-18<br>-1.12E-16<br>4.66E-17<br>2.86E-01<br>-6.43E-20<br>8.227E-01
(ATP consumption)<br>Local<br>Min<br>Max<br>Normalized peak height<br>Peak sensitivity value<br>Shapiro-Wilk score	-1.59E-03<br>-1.02E+01<br>3.52E-01<br>6.26E-01<br>-1.55E-03<br>2.922E-01	-6.83E-04<br>-1.38E+00<br>4.28E-01<br>4.13E-01<br>6.58E-04<br>3.395E-01	-1.24E-01<br>-9.61E+00<br>1.83E+00<br>1.01E-01<br>-2.58E-03<br>4.515E-01	2.57E-02<br>8.75E-05<br>1.60E-01<br>7.18E-03<br>1.90E-02<br>9.242E-01	3.11E-01<br>9.31E-02<br>1.12E+01<br>4.16E-02<br>1.99E-01<br>5.561E-01	-8.56E-16<br>-6.55E-14<br>1.07E-13<br>1.93E-01<br>-7.48E-17<br>5.028E-01	4.80E-03<br>2.89E-04<br>1.93E-01<br>3.17E-02<br>2.31E-03<br>5.728E-01	1.05E-02<br>6.84E-04<br>3.36E-01<br>2.43E-02<br>5.22E-03<br>6.133E-01	9.01E-04<br>4.97E-05<br>4.61E-02<br>4.10E-02<br>3.49E-04<br>5.662E-01	2.72E-03<br>1.09E-04<br>7.21E-02<br>2.22E-02<br>1.01E-03<br>6.599E-01	-2.17E-15<br>-1.79E-13<br>4.02E-13<br>3.20E-01<br>-1.25E-16<br>3.686E-01	9.61E-02<br>9.98E-03<br>3.02E+00<br>2.81E-02<br>4.16E-02<br>6.062E-01	0.00E+00<br>-2.00E-13<br>1.75E-13<br>6.80E-01<br>-3.52E-17<br>1.880E-01	0.00E+00<br>-7.84E-13<br>5.43E-13<br>8.30E-01<br>-3.39E-17<br>1.576E-01	-1.64E-02<br>-4.78E-01<br>-1.20E-03<br>2.19E-02<br>-1.10E-02<br>6.149E-01	-1.37E-15<br>-1.21E-12<br>3.40E-13<br>2.48E-01<br>5.94E-16<br>4.516E-01	-6.97E-17<br>-5.56E-13<br>3.00E-13<br>2.45E-01<br>-7.01E-17<br>4.674E-01	-3.93E-03<br>-1.34E-01<br>-3.69E-04<br>2.51E-02<br>-2.58E-03<br>6.192E-01	-2.04E-02<br>-4.33E-01<br>-3.48E-03<br>1.78E-02<br>-1.19E-02<br>6.470E-01	-5.81E-03<br>-1.38E-01<br>-9.51E-04<br>2.04E-02<br>-3.62E-03<br>6.460E-01	-4.63E-04<br>-2.63E-02<br>-8.67E-06<br>4.70E-02<br>-1.01E-04<br>5.390E-01	-2.32E-01<br>-1.14E+00<br>2.76E-01<br>1.02E-02<br>-2.30E-01<br>7.848E-01	9.53E-01<br>5.39E-01<br>9.82E-01<br>8.42E-03<br>9.58E-01<br>7.953E-01	2.55E-17<br>-2.70E-15<br>1.68E-15<br>3.03E-01<br>1.58E-18<br>6.933E-01
(Adenylate kinase)<br>Local<br>Min<br>Max<br>Normalized peak height<br>Peak sensitivity value<br>Shapiro-Wilk score	-INF<br>-INF<br>INF<br>NA<br>NAN<br>NA	-INF<br>-INF<br>INF<br>NA<br>NAN<br>NA	-INF<br>-INF<br>INF<br>NA<br>NAN<br>NA	-INF<br>-INF<br>INF<br>NA<br>NAN<br>NA	-INF<br>-INF<br>INF<br>NA<br>NAN<br>NA	-INF<br>-INF<br>INF<br>NA<br>NAN<br>NA	-INF<br>-INF<br>INF<br>NA<br>NAN<br>NA	-INF<br>-INF<br>INF<br>NA<br>NAN<br>NA	-INF<br>-INF<br>INF<br>NA<br>NAN<br>NA	-INF<br>-INF<br>INF<br>NA<br>NAN<br>NA	-INF<br>-INF<br>INF<br>NA<br>NAN<br>NA	-INF<br>-INF<br>INF<br>NA<br>NAN<br>NA	-INF<br>-INF<br>INF<br>NA<br>NAN<br>NA	-INF<br>-INF<br>INF<br>NA<br>NAN<br>NA	-INF<br>-INF<br>INF<br>NA<br>NAN<br>NA	-INF<br>-INF<br>INF<br>NA<br>NAN<br>NA	-INF<br>-INF<br>INF<br>NA<br>NAN<br>NA	-INF<br>-INF<br>INF<br>NA<br>NAN<br>NA	-INF<br>-INF<br>INF<br>NA<br>NAN<br>NA	-INF<br>-INF<br>INF<br>NA<br>NAN<br>NA	-INF<br>-INF<br>INF<br>NA<br>NAN<br>NA	-INF<br>-INF<br>INF<br>NA<br>NAN<br>NA	-INF<br>-INF<br>INF<br>NA<br>NAN<br>NA	-INF<br>-INF<br>INF<br>NA<br>NAN<br>NA

Table S30: Flux control coefficients for *Saccharomyces cerevisiae* model with parameter variation of $\pm$50%

	(GlucoseMixedflowtoextracellularmedium)	(Glucoseuptake)	(Hexokinase)	(Phosphoglucoisomerase)	(Phosphofructokinase)	(Aldolase)	(Triosephosphateisomerase)	(Glyceraldehyde3-phosphatedehydrogenase)	(Phosphoenolpyruvatesynthesis)	(Pyruvatekinase)	(Pyruvatedecarboxylase)	(Alcoholdehydrogenase)	(Ethanolout)	(Ethanolflow)	(Glycerolsynthesis)	(Glycerolout)	(Glycerolflow)	(Acetaldehydeout)	(Acetaldehydeflow)	(Cyanide-Acetaldehydeflow)	(Cyanideflow)	(Storage)	(ATPconsumption)	(Adenylatekinase)
(Glucose Mixed flow to extra-cellular medium)<br>Local<br>Min<br>Max<br>Normalized peak height<br>Peak sensitivity value<br>Shapiro-Wilk score	1.25E-02<br>-9.45E-02<br>9.95E-01<br>1.37E-01<br>8.87E-04<br>5.956E-01	5.36E-03<br>-3.87E-02<br>5.75E-01<br>8.12E-02<br>9.12E-04<br>5.213E-01	9.73E-01<br>-5.92E-01<br>1.15E+00<br>4.01E-02<br>1.00E+00<br>7.356E-01	1.37E-03<br>1.02E-05<br>4.44E-02<br>2.06E-02<br>7.42E-04<br>6.315E-01	1.65E-02<br>2.27E-04<br>1.00E+00<br>3.77E-02<br>7.74E-03<br>5.599E-01	-3.45E-17<br>-3.13E-14<br>2.22E-13<br>7.97E-01<br>-5.31E-17<br>1.084E-01	2.30E-04<br>-8.37E-03<br>3.63E-02<br>1.23E-01<br>5.69E-05<br>5.012E-01	5.02E-04<br>-2.05E-02<br>4.89E-02<br>7.94E-02<br>1.48E-04<br>5.518E-01	4.31E-05<br>-1.91E-03<br>9.68E-03<br>1.63E-01<br>8.34E-06<br>4.654E-01	1.30E-04<br>-9.27E-03<br>1.25E-02<br>1.25E-01<br>1.90E-05<br>5.408E-01	-8.14E-17<br>-4.08E-14<br>6.92E-14<br>6.17E-01<br>-4.39E-17<br>1.901E-01	4.60E-03<br>-2.07E-01<br>3.04E-01<br>7.81E-02<br>1.09E-03<br>5.698E-01	0.00E+00<br>-7.10E-14<br>3.82E-14<br>6.96E-01<br>4.95E-17<br>1.041E-01	0.00E+00<br>-2.26E-13<br>1.97E-13<br>8.60E-01<br>1.77E-18<br>1.018E-01	-7.83E-04<br>-7.46E-02<br>3.10E-02<br>7.16E-02<br>-1.84E-04<br>5.522E-01	-6.35E-17<br>-9.93E-14<br>1.08E-13<br>3.84E-01<br>-1.03E-16<br>3.453E-01	-5.80E-18<br>-7.09E-14<br>1.62E-13<br>4.77E-01<br>-1.11E-16<br>2.855E-01	-1.88E-04<br>-2.11E-02<br>7.81E-03<br>7.93E-02<br>-5.31E-05<br>5.602E-01	-9.78E-04<br>-5.99E-02<br>2.76E-02<br>4.81E-02<br>-3.57E-04<br>6.128E-01	-2.78E-04<br>-1.80E-02<br>6.97E-03<br>4.65E-02<br>-1.35E-04<br>6.061E-01	-2.22E-05<br>-6.61E-03<br>1.69E-03<br>2.49E-01<br>-5.42E-06<br>4.472E-01	-9.81E-03<br>-1.82E-01<br>3.67E-01<br>2.34E-02<br>-6.98E-03<br>6.490E-01	-2.24E-03<br>-5.94E-02<br>8.63E-02<br>2.37E-02<br>-1.75E-03<br>8.214E-01	1.36E-18<br>-4.96E-17<br>9.18E-17<br>2.74E-01<br>-5.62E-20<br>7.228E-01
(Glucose uptake)<br>Local<br>Min<br>Max<br>Normalized peak height<br>Peak sensitivity value<br>Shapiro-Wilk score	1.25E-02<br>-2.87E-02<br>9.96E-01<br>1.14E-01<br>1.52E-03<br>5.912E-01	5.36E-03<br>-4.55E-02<br>5.78E-01<br>8.16E-02<br>9.41E-04<br>5.170E-01	9.73E-01<br>-1.86E-01<br>1.02E+00<br>2.93E-02<br>9.99E-01<br>7.336E-01	1.37E-03<br>8.55E-06<br>4.62E-02<br>2.18E-02<br>5.86E-04<br>6.265E-01	1.65E-02<br>2.41E-04<br>9.68E-01<br>3.73E-02<br>8.47E-03<br>5.572E-01	-2.20E-17<br>-4.48E-14<br>3.19E-14<br>3.87E-01<br>2.20E-17<br>3.376E-01	2.30E-04<br>-1.17E-02<br>3.01E-02<br>1.16E-01<br>7.01E-05<br>4.987E-01	5.02E-04<br>-1.37E-02<br>4.80E-02<br>7.10E-02<br>1.60E-04<br>5.512E-01	4.31E-05<br>-1.74E-03<br>1.05E-02<br>1.81E-01<br>1.26E-05<br>4.518E-01	1.30E-04<br>-1.10E-02<br>1.40E-02<br>1.40E-01<br>2.79E-05<br>5.349E-01	-8.36E-17<br>-5.26E-14<br>5.96E-13<br>6.32E-01<br>3.15E-16<br>4.561E-02	4.60E-03<br>-2.23E-01<br>3.14E-01<br>7.91E-02<br>1.15E-03<br>5.649E-01	0.00E+00<br>-3.42E-14<br>8.06E-14<br>6.55E-01<br>-5.46E-17<br>1.060E-01	0.00E+00<br>-1.75E-13<br>1.55E-13<br>8.17E-01<br>9.69E-17<br>1.096E-01	-7.83E-04<br>-7.33E-02<br>2.34E-02<br>6.89E-02<br>-2.18E-04<br>5.528E-01	-6.57E-17<br>-8.55E-14<br>1.02E-13<br>4.82E-01<br>-6.39E-17<br>3.386E-01	-3.32E-18<br>-9.99E-14<br>7.96E-14<br>6.00E-01<br>1.22E-17<br>3.002E-01	-1.88E-04<br>-1.87E-02<br>5.37E-03<br>6.60E-02<br>-6.15E-05<br>5.574E-01	-9.78E-04<br>-5.87E-02<br>2.96E-02<br>4.85E-02<br>-4.03E-04<br>6.061E-01	-2.78E-04<br>-1.85E-02<br>7.53E-03<br>4.76E-02<br>-1.24E-04<br>5.981E-01	-2.22E-05<br>-5.77E-03<br>1.36E-03<br>2.30E-01<br>-1.74E-06<br>4.441E-01	-9.81E-03<br>-1.84E-01<br>3.71E-01<br>2.35E-02<br>-8.61E-03<br>6.483E-01	-2.24E-03<br>-5.30E-02<br>8.00E-02<br>2.17E-02<br>-1.49E-03<br>8.195E-01	1.36E-18<br>-5.97E-17<br>1.09E-15<br>4.18E-01<br>5.46E-19<br>1.992E-01
(Hexokinase)<br>Local<br>Min<br>Max<br>Normalized peak height<br>Peak sensitivity value<br>Shapiro-Wilk score	1.25E-02<br>-8.55E-03<br>9.95E-01<br>1.34E-01<br>9.87E-04<br>5.884E-01	5.36E-03<br>-2.45E-02<br>5.61E-01<br>7.78E-02<br>9.59E-04<br>5.173E-01	9.73E-01<br>-3.94E-01<br>1.02E+00<br>3.22E-02<br>1.00E+00<br>7.331E-01	1.37E-03<br>6.53E-06<br>4.77E-02<br>2.26E-02<br>7.45E-04<br>6.210E-01	1.65E-02<br>3.47E-04<br>9.75E-01<br>3.79E-02<br>7.66E-03<br>5.610E-01	-4.14E-17<br>-3.38E-14<br>3.73E-13<br>7.23E-01<br>1.70E-16<br>6.077E-02	2.30E-04<br>-1.10E-02<br>2.80E-02<br>1.08E-01<br>5.45E-05<br>5.068E-01	5.02E-04<br>-1.92E-02<br>4.97E-02<br>7.81E-02<br>1.43E-04<br>5.545E-01	4.31E-05<br>-1.60E-03<br>8.06E-03<br>1.32E-01<br>1.63E-05<br>4.633E-01	1.30E-04<br>-9.18E-03<br>1.45E-02<br>1.26E-01<br>3.52E-05<br>5.353E-01	-1.06E-16<br>-6.27E-14<br>3.29E-13<br>6.67E-01<br>-1.75E-16<br>1.032E-01	4.60E-03<br>-1.89E-01<br>3.04E-01<br>7.10E-02<br>9.53E-04<br>5.697E-01	0.00E+00<br>-5.83E-14<br>3.62E-14<br>7.87E-01<br>-1.98E-18<br>1.155E-01	0.00E+00<br>-1.57E-13<br>3.11E-13<br>8.52E-01<br>-1.08E-16<br>9.901E-02	-7.83E-04<br>-9.45E-02<br>2.86E-02<br>8.32E-02<br>-2.28E-04<br>5.580E-01	-6.57E-17<br>-1.29E-13<br>8.37E-14<br>5.47E-01<br>-2.76E-17<br>3.424E-01	-3.32E-18<br>-5.37E-14<br>2.02E-13<br>4.97E-01<br>1.18E-16<br>2.605E-01	-1.88E-04<br>-2.16E-02<br>6.33E-03<br>7.54E-02<br>-5.98E-05<br>5.593E-01	-9.78E-04<br>-5.96E-02<br>2.01E-02<br>4.25E-02<br>-4.57E-04<br>6.149E-01	-2.78E-04<br>-1.99E-02<br>6.43E-03<br>4.93E-02<br>-8.82E-05<br>6.074E-01	-2.22E-05<br>-5.44E-03<br>2.17E-03<br>1.90E-01<br>2.30E-07<br>4.470E-01	-9.81E-03<br>-2.13E-01<br>3.55E-01<br>2.33E-02<br>-8.54E-03<br>6.484E-01	-2.24E-03<br>-6.05E-02<br>6.89E-02<br>2.14E-02<br>-1.56E-03<br>8.221E-01	1.36E-18<br>-4.57E-17<br>9.44E-17<br>2.72E-01<br>2.87E-20<br>7.239E-01

(Phosphoglucoisomerase)	Local	1.27E-02	5.48E-03	9.94E-01	3.42E-03	4.14E-02	4.62E-16	-6.63E-03	-1.45E-02	-1.24E-03	-3.75E-03	2.63E-15	-1.33E-01
	Min	-2.87E-02	-3.31E-02	-3.10E-01	6.92E-06	8.59E-03	-5.19E-14	-4.59E-02	-5.74E-02	-8.62E-03	-3.60E-02	-9.12E-14	-5.34E-01
	Max	1.29E+00	6.46E-01	1.31E+00	6.94E-02	1.06E+00	2.70E-14	1.13E-04	4.53E-04	3.14E-05	1.69E-05	7.01E-14	5.83E-03
	Normalized peak height	1.84E-01	9.19E-02	2.55E-02	1.58E-02	2.05E-02	9.11E-02	1.78E-02	1.41E-02	1.77E-02	4.19E-02	3.24E-01	1.16E-02
	Peak sensitivity value	9.41E-04	1.16E-03	1.67E-03	2.75E-03	2.81E-02	3.85E-17	-4.75E-05	-3.87E-05	-7.50E-06	-1.13E-06	2.41E-17	-3.84E-04
	Shapiro-Wilk score	5.927E-01	4.972E-01	7.828E-01	7.289E-01	5.879E-01	8.716E-01	8.688E-01	9.396E-01	8.534E-01	7.685E-01	5.700E-01	9.000E-01
(Phosphofructokinase)	Local	1.27E-02	5.48E-03	9.94E-01	3.42E-03	4.14E-02	1.39E-15	-6.63E-03	-1.45E-02	-1.24E-03	-3.75E-03	3.85E-15	-1.33E-01
	Min	-9.22E-02	-1.85E-01	-9.61E-02	7.92E-06	8.94E-03	-1.95E-14	-4.80E-02	-6.48E-02	-8.63E-03	-3.61E-02	-1.00E-13	-5.63E-01
	Max	1.26E+00	6.16E-01	1.33E+00	5.71E-02	1.00E+00	2.52E-14	9.71E-07	7.50E-06	5.48E-07	2.20E-07	9.92E-14	6.69E-05
	Normalized peak height	1.56E-01	1.07E-01	2.27E-02	1.26E-02	1.87E-02	8.47E-02	2.78E-02	1.43E-02	2.24E-02	5.45E-02	3.62E-01	1.24E-02
	Peak sensitivity value	1.52E-03	1.19E-03	1.78E-03	2.66E-03	2.63E-02	-2.14E-17	-2.30E-05	-2.49E-05	-3.77E-06	-1.78E-05	3.41E-17	-2.15E-04
	Shapiro-Wilk score	5.958E-01	5.010E-01	7.828E-01	7.311E-01	5.912E-01	7.793E-01	8.678E-01	9.366E-01	8.578E-01	7.582E-01	5.773E-01	9.018E-01
(Aldolase)	Local	1.27E-02	5.48E-03	9.94E-01	3.42E-03	4.14E-02	1.11E-15	-6.63E-03	-1.45E-02	-1.24E-03	-3.75E-03	-1.02E-14	-1.33E-01
	Min	-1.15E-01	-3.13E-02	-1.35E-01	6.03E-06	8.64E-03	-1.53E-13	-5.34E-02	-7.32E-02	-8.52E-03	-3.44E-02	-1.69E-13	-6.05E-01
	Max	1.25E+00	6.35E-01	1.29E+00	6.26E-02	1.02E+00	9.08E-14	1.17E-05	6.77E-05	1.33E-05	1.97E-06	2.01E-13	1.85E-03
	Normalized peak height	1.82E-01	9.14E-02	2.42E-02	1.38E-02	2.01E-02	1.13E-01	2.49E-02	1.64E-02	1.66E-02	5.15E-02	2.88E-01	1.33E-02
	Peak sensitivity value	9.12E-04	9.86E-04	1.49E-03	2.67E-03	2.74E-02	-3.23E-16	-1.50E-05	-4.22E-05	-8.04E-06	-1.52E-05	5.94E-17	-2.71E-04
	Shapiro-Wilk score	5.956E-01	5.005E-01	7.833E-01	7.348E-01	5.949E-01	6.480E-01	8.655E-01	9.322E-01	8.542E-01	7.602E-01	7.232E-01	8.987E-01
(Triosephosphate isomerase)	Local	1.01E-02	4.37E-03	7.92E-01	2.32E-03	2.81E-02	-1.03E-15	9.18E-03	2.01E-02	1.72E-03	5.20E-03	-5.10E-15	1.84E-01
	Min	-3.82E-02	-7.38E-02	-4.84E-01	-1.78E-03	-3.29E-02	-3.14E-14	1.19E-04	7.27E-04	3.83E-05	3.34E-05	-6.55E-14	1.47E-02
	Max	9.36E-01	5.10E-01	9.86E-01	3.45E-02	9.64E-01	2.36E-14	8.13E-02	1.60E-01	2.12E-02	7.08E-02	7.11E-14	9.41E-01
	Normalized peak height	1.47E-01	9.67E-02	2.71E-02	1.13E-02	2.36E-02	4.90E-02	8.92E-03	7.15E-03	1.21E-02	1.65E-02	2.90E-01	5.53E-03
	Peak sensitivity value	1.22E-03	5.64E-04	6.63E-04	1.87E-03	2.14E-02	-1.52E-17	3.57E-03	1.10E-02	6.41E-04	9.88E-04	-1.06E-17	6.79E-02
	Shapiro-Wilk score	5.833E-01	4.832E-01	8.138E-01	7.550E-01	5.811E-01	7.114E-01	8.197E-01	8.653E-01	7.637E-01	7.264E-01	7.604E-01	8.259E-01
(Glyceraldehyde 3-phosphate dehydrogenase)	Local	1.16E-02	4.98E-03	9.03E-01	2.92E-03	3.54E-02	-1.85E-16	5.23E-04	1.14E-03	9.81E-05	2.96E-04	-9.79E-15	1.05E-02
	Min	-3.73E-02	-5.60E-02	-1.89E-01	4.21E-06	7.28E-03	-9.65E-14	-7.36E-03	-1.31E-02	-1.32E-03	-7.79E-03	-1.17E-13	-1.48E-01
	Max	9.52E-01	5.25E-01	9.96E-01	4.51E-02	9.82E-01	9.51E-14	3.62E-02	5.43E-02	1.03E-02	1.55E-02	1.37E-13	3.15E-01
	Normalized peak height	1.50E-01	8.77E-02	2.05E-02	1.19E-02	2.14E-02	1.35E-01	5.80E-02	3.68E-02	8.11E-02	6.19E-02	2.55E-01	3.30E-02
	Peak sensitivity value	7.79E-04	6.08E-04	2.00E-03	2.33E-03	2.53E-02	7.82E-18	2.04E-04	4.38E-04	3.12E-05	7.57E-05	2.61E-17	3.38E-03
	Shapiro-Wilk score	5.870E-01	4.960E-01	7.493E-01	7.400E-01	5.922E-01	6.389E-01	5.449E-01	6.007E-01	5.001E-01	6.047E-01	7.528E-01	6.167E-01
(Phosphoenolpyruvate synthesis)	Local	1.16E-02	4.98E-03	9.03E-01	2.92E-03	3.54E-02	-2.35E-16	5.23E-04	1.14E-03	9.81E-05	2.96E-04	-4.52E-14	1.05E-02
	Min	-4.26E-02	-2.04E-02	-1.39E-01	4.71E-06	6.08E-03	-9.53E-13	-9.12E-03	-1.10E-02	-1.77E-03	-6.67E-03	-7.59E-13	-1.39E-01
	Max	9.57E-01	5.28E-01	9.75E-01	5.16E-02	9.82E-01	9.00E-13	3.65E-02	5.49E-02	1.11E-02	2.08E-02	1.09E-12	3.17E-01
	Normalized peak height	1.52E-01	8.20E-02	1.96E-02	1.32E-02	2.14E-02	1.31E-01	5.97E-02	3.63E-02	9.27E-02	7.37E-02	2.88E-01	3.28E-02
	Peak sensitivity value	9.22E-04	7.47E-04	2.11E-03	2.41E-03	2.51E-02	3.97E-16	2.11E-04	4.11E-04	3.29E-05	7.94E-05	6.32E-16	4.11E-03
	Shapiro-Wilk score	5.905E-01	4.996E-01	7.487E-01	7.452E-01	5.900E-01	5.115E-01	5.435E-01	5.981E-01	4.948E-01	5.989E-01	7.507E-01	6.147E-01
(Pyruvate kinase)	Local	1.16E-02	4.98E-03	9.03E-01	2.92E-03	3.54E-02	2.24E-15	5.23E-04	1.14E-03	9.81E-05	2.96E-04	5.94E-14	1.05E-02
	Min	-3.05E-02	-4.50E-02	-8.89E-01	9.14E-06	7.73E-03	-2.95E-13	-7.05E-03	-1.33E-02	-1.86E-03	-6.44E-03	-4.59E-13	-1.44E-01
	Max	9.61E-01	7.73E-01	9.82E-01	4.76E-02	9.81E-01	9.59E-12	3.72E-02	5.29E-02	8.18E-03	1.24E-02	4.65E-13	3.29E-01
	Normalized peak height	1.53E-01	1.14E-01	3.02E-02	1.24E-02	2.07E-02	7.62E-01	5.86E-02	3.52E-02	7.12E-02	5.20E-02	3.89E-01	3.27E-02
	Peak sensitivity value	7.66E-04	1.17E-03	2.15E-03	2.41E-03	2.67E-02	-3.42E-15	1.88E-04	5.20E-04	2.99E-05	7.13E-05	-4.11E-16	3.46E-03
	Shapiro-Wilk score	5.855E-01	4.952E-01	7.486E-01	7.396E-01	5.934E-01	1.701E-02	5.457E-01	6.064E-01	5.099E-01	6.152E-01	6.283E-01	6.177E-01
(Pyruvate decarboxylase)	Local	1.16E-02	4.98E-03	9.03E-01	2.92E-03	3.54E-02	-8.93E-16	5.23E-04	1.14E-03	9.81E-05	2.96E-04	-6.66E-16	1.05E-02
	Min	-8.38E-02	-3.73E-02	-3.13E-01	6.61E-06	6.53E-03	-2.10E-14	-5.99E-03	-1.18E-02	-1.43E-03	-6.15E-03	-6.84E-14	-1.30E-01
	Max	9.53E-01	5.43E-01	1.05E+00	4.90E-02	9.84E-01	4.71E-14	2.84E-02	4.85E-02	1.05E-02	1.54E-02	1.48E-13	3.35E-01
	Normalized peak height	1.53E-01	8.87E-02	2.26E-02	1.25E-02	2.14E-02	1.49E-01	4.47E-02	3.28E-02	8.32E-02	5.78E-02	2.01E-01	3.18E-02
	Peak sensitivity value	7.31E-04	7.33E-04	1.12E-03	2.29E-03	2.56E-02	-2.46E-17	1.78E-04	4.31E-04	3.90E-05	8.11E-05	2.52E-17	3.84E-03
	Shapiro-Wilk score	5.882E-01	5.001E-01	7.495E-01	7.339E-01	5.929E-01	6.409E-01	5.503E-01	6.051E-01	5.021E-01	5.968E-01	5.160E-01	6.160E-01
(Alcohol dehydrogenase)	Local	1.01E-02	4.37E-03	7.92E-01	2.32E-03	2.81E-02	-3.80E-16	9.18E-03	2.01E-02	1.72E-03	5.20E-03	6.30E-16	1.84E-01
	Min	-1.49E-01	-9.71E-02	-1.31E-01	-2.26E-03	-2.70E-02	-2.60E-14	5.51E-05	6.43E-04	1.85E-05	3.76E-05	-5.12E-14	1.53E-02
	Max	9.29E-01	5.03E-01	1.08E+00	4.16E-02	9.65E-01	1.20E-14	8.21E-02	1.50E-01	2.94E-02	6.77E-02	7.17E-14	9.32E-01
	Normalized peak height	1.82E-01	9.56E-02	2.35E-02	1.40E-02	2.46E-02	1.00E-01	8.50E-03	7.25E-03	1.69E-02	1.67E-02	2.74E-01	5.52E-03
	Peak sensitivity value	9.25E-04	1.01E-03	1.60E-03	2.02E-03	1.91E-02	1.90E-17	4.03E-03	9.39E-03	6.51E-04	9.51E-04	5.33E-18	6.44E-02
	Shapiro-Wilk score	5.823E-01	4.803E-01	8.136E-01	7.451E-01	5.792E-01	6.858E-01	8.245E-01	8.653E-01	7.677E-01	7.289E-01	6.636E-01	8.247E-01
(Ethanol out)	Local	1.01E-02	4.37E-03	7.92E-01	2.32E-03	2.81E-02	-4.21E-16	9.18E-03	2.01E-02	1.72E-03	5.20E-03	5.13E-16	1.84E-01
	Min	-1.88E-02	-8.37E-03	-2.43E+00	-2.29E-03	-2.38E-02	-1.96E-14	7.14E-05	6.58E-04	2.96E-05	3.04E-05	-2.98E-14	1.54E-02
	Max	9.35E-01	1.02E+00	9.68E-01	5.49E-02	1.31E+00	1.46E-14	7.27E-02	1.36E-01	2.21E-02	5.78E-02	4.20E-14	9.52E-01
	Normalized peak height	1.61E-01	1.41E-01	5.16E-02	1.68E-02	3.08E-02	2.18E-01	7.90E-03	6.20E-03	1.30E-02	1.38E-02	5.03E-01	5.69E-03
	Peak sensitivity value	7.56E-04	1.38E-03	3.90E-03	1.80E-03	1.95E-02	-1.44E-17	3.08E-03	1.08E-02	6.15E-04	8.68E-04	9.50E-19	6.55E-02
	Shapiro-Wilk score	5.870E-01	4.801E-01	8.123E-01	7.408E-01	5.799E-01	7.009E-01	8.253E-01	8.650E-01	7.702E-01	7.351E-01	6.307E-01	8.257E-01
(Ethanol flow)	Local	1.01E-02	4.37E-03	7.92E-01	2.32E-03	2.81E-02	-3.81E-16	9.18E-03	2.01E-02	1.72E-03	5.20E-03	-1.74E-16	1.84E-01
	Min	-1.05E+00	-7.04E-01	-1.95E-01	-4.25E-02	-5.51E-01	-1.32E-14	1.26E-04	7.68E-04	4.07E-05	3.70E-05	-7.66E-14	1.53E-02
	Max	9.30E-01	4.92E-01	2.59E+00	3.96E-02	9.66E-01	1.21E-14	8.09E-02	1.36E-01	2.21E-02	6.52E-02	3.42E-14	9.47E-01
	Normalized peak height	2.51E-01	1.72E-01	4.99E-02	2.42E-02	3.50E-02	1.22E-01	8.81E-03	6.45E-03	1.28E-02	1.49E-02	3.37E-01	5.79E-03
	Peak sensitivity value	7.61E-04	1.33E-03	1.55E-03	1.77E-03	2.17E-02	7.49E-18	3.64E-03	9.50E-03	6.90E-04	9.82E-04	1.05E-17	7.08E-02
	Shapiro-Wilk score	5.822E-01	4.784E-01	8.149E-01	7.429E-01	5.832E-01	6.647E-01	8.222E-01	8.648E-01	7.766E-01	7.302E-01	4.919E-01	8.255E-01
(Glycerol synthesis)	Local	2.51E-02	1.08E-02	1.96E+00	8.63E-03	1.04E-01	8.06E-15	-8.18E-02	-1.79E-01	-1.53E-02	-4.63E-02	1.65E-14	-1.64E+00
	Min	-2.51E-01	-6.43E-02	-1.01E+00	1.30E-05	2.03E-02	-1.93E-13	-5.07E-01	-5.37E-01	-7.80E-02	-3.53E-01	-5.52E-13	-3.50E+00
	Max	3.20E+00	1.71E+00	3.57E+00	1.14E-01	3.03E+00	1.87E-13	-7.56E-05	-4.35E-03	-5.26E-05	-8.25E-05	7.24E-13	-5.56E-01
	Normalized peak height	2.43E-01	1.31E-01	3.76E-02	1.19E-02	2.67E-02	1.05E-01	4.99E-03	2.91E-03	5.54E-03	8.47E-03	3.50E-01	4.83E-03
	Peak sensitivity value	2.44E-03	2.24E-03	1.66E-03	6.00E-03	6.09E-02	-1.53E-16	-4.74E-02	-1.83E-01	-8.74E-03	-1.09E-02	-3.53E-16	-1.15E+00
	Shapiro-Wilk score	5.998E-01	4.940E-01	8.916E-01	7.491E-01	6.066E-01	7.228E-01	9.097E-01	9.783E-01	9.141E-01	8.417E-01	5.094E-01	9.459E-01

(Phosphoglucoisomerase)	Local	0.00E+00	0.00E+00	2.26E-02	1.83E-15	9.24E-17	5.43E-03	2.82E-02	8.02E-03	6.39E-04	-2.82E-02	6.46E-02	3.55E-18
	Min	-1.90E-13	-4.06E-13	-5.58E-04	-1.15E-13	-9.50E-14	-1.22E-04	-7.90E-04	-1.05E-04	-1.57E-05	-2.19E-01	-4.86E-04	-8.53E-17
	Max	6.25E-14	3.59E-13	8.78E-02	1.39E-13	9.64E-14	3.06E-02	8.53E-02	2.59E-02	6.42E-03	3.89E-01	2.08E-01	1.63E-16
	Normalized peak height	5.22E-01	6.23E-01	1.16E-02	1.77E-01	1.91E-01	1.85E-02	8.80E-03	1.09E-02	1.89E-02	1.52E-02	2.25E-02	2.95E-01
	Peak sensitivity value	1.79E-17	-2.93E-16	1.05E-04	1.72E-17	2.49E-17	1.64E-05	2.79E-05	1.17E-05	3.88E-07	-4.41E-04	3.39E-05	1.12E-19
	Shapiro-Wilk score	3.656E-01	3.408E-01	9.329E-01	7.144E-01	6.593E-01	9.220E-01	9.861E-01	9.781E-01	7.424E-01	7.175E-01	9.831E-01	8.654E-01
(Phosphofructokinase)	Local	0.00E+00	0.00E+00	2.26E-02	2.02E-15	1.13E-16	5.43E-03	2.82E-02	8.02E-03	6.39E-04	-2.82E-02	6.46E-02	3.55E-18
	Min	-6.14E-14	-3.00E-13	-9.35E-06	-1.32E-13	-9.26E-14	-1.33E-06	-9.95E-06	-3.49E-06	-5.07E-07	-2.49E-01	-1.91E-06	-6.91E-17
	Max	6.84E-14	3.27E-13	9.82E-02	1.08E-13	1.07E-13	2.58E-02	7.34E-02	2.50E-02	8.05E-03	3.55E-01	2.04E-01	1.22E-16
	Normalized peak height	4.60E-01	6.31E-01	1.61E-02	1.81E-01	1.73E-01	1.58E-02	7.70E-03	9.93E-03	3.14E-02	1.67E-02	2.76E-02	3.03E-01
	Peak sensitivity value	-2.94E-17	-2.07E-16	3.98E-05	-2.02E-17	-8.86E-17	1.16E-05	1.00E-04	8.99E-06	3.52E-06	-1.21E-04	1.00E-04	-6.33E-21
	Shapiro-Wilk score	3.924E-01	3.440E-01	9.316E-01	7.199E-01	6.702E-01	9.271E-01	9.859E-01	9.784E-01	7.378E-01	7.163E-01	9.834E-01	8.820E-01
(Aldolase)	Local	0.00E+00	0.00E+00	2.26E-02	3.98E-16	1.24E-16	5.43E-03	2.82E-02	8.02E-03	6.39E-04	-2.82E-02	6.46E-02	3.55E-18
	Min	-9.07E-14	-3.98E-13	-8.74E-05	-2.88E-13	-1.08E-13	-3.48E-05	-3.46E-04	-6.40E-05	-7.37E-06	-2.22E-01	-4.27E-05	-1.36E-16
	Max	1.05E-13	4.33E-13	1.06E-01	1.46E-13	8.97E-14	2.51E-02	8.06E-02	2.89E-02	7.84E-03	3.74E-01	1.94E-01	3.38E-16
	Normalized peak height	4.61E-01	7.06E-01	1.58E-02	1.54E-01	1.69E-01	1.19E-02	8.41E-03	1.01E-02	2.91E-02	1.58E-02	2.32E-02	3.21E-01
	Peak sensitivity value	9.17E-17	2.04E-16	7.22E-05	2.13E-16	-1.55E-18	2.81E-05	9.97E-05	8.38E-06	4.40E-06	-1.13E-04	5.43E-05	-6.61E-20
	Shapiro-Wilk score	3.639E-01	3.472E-01	9.291E-01	6.761E-01	6.719E-01	9.257E-01	9.855E-01	9.770E-01	7.364E-01	7.174E-01	9.830E-01	7.895E-01
(Triosephosphate isomerase)	Local	0.00E+00	0.00E+00	-3.13E-02	-1.10E-17	1.39E-15	-7.52E-03	-3.91E-02	-1.11E-02	-8.85E-04	-1.89E-02	5.21E-02	2.13E-18
	Min	-5.74E-14	-1.83E-13	-2.43E-01	-8.35E-14	-6.99E-14	-5.02E-02	-1.52E-01	-4.70E-02	-1.79E-02	-1.84E-01	-1.92E-04	-6.58E-17
	Max	8.56E-14	1.60E-13	-1.21E-03	6.28E-14	5.13E-14	-4.03E-04	-5.53E-03	-1.49E-03	-7.19E-06	3.02E-01	1.76E-01	3.88E-16
	Normalized peak height	4.68E-01	6.01E-01	7.15E-03	1.26E-01	1.44E-01	5.96E-03	4.10E-03	4.30E-03	2.12E-02	1.21E-02	2.50E-02	2.98E-01
	Peak sensitivity value	4.04E-17	1.46E-16	-1.66E-02	-1.11E-17	-5.52E-17	-4.86E-03	-3.04E-02	-8.52E-03	-1.05E-04	-1.81E-02	7.23E-05	1.49E-19
	Shapiro-Wilk score	4.225E-01	3.629E-01	8.613E-01	6.777E-01	6.375E-01	8.697E-01	9.267E-01	9.290E-01	7.235E-01	7.327E-01	9.785E-01	6.773E-01
(Glyceraldehyde 3-phosphate dehydrogenase)	Local	0.00E+00	0.00E+00	-1.78E-03	7.65E-16	6.61E-16	-4.28E-04	-2.23E-03	-6.32E-04	-5.04E-05	-2.40E-02	5.89E-02	2.91E-18
	Min	-7.45E-14	-2.42E-13	-8.54E-02	-7.96E-14	-8.22E-14	-2.38E-02	-6.03E-02	-1.85E-02	-5.48E-03	-1.96E-01	-7.08E-05	-1.17E-16
	Max	4.31E-14	2.96E-13	1.87E-02	9.51E-14	6.07E-14	4.95E-03	1.87E-02	5.29E-03	1.48E-03	3.26E-01	1.98E-01	6.64E-17
	Normalized peak height	4.31E-01	5.80E-01	3.45E-02	1.16E-01	1.41E-01	3.88E-02	2.23E-02	2.28E-02	1.11E-01	1.27E-02	2.00E-02	2.80E-01
	Peak sensitivity value	-1.94E-17	-2.56E-16	-6.88E-04	-3.78E-17	-6.06E-17	-1.57E-04	-1.06E-03	-3.52E-04	-9.47E-06	-2.57E-02	2.81E-05	-7.18E-20
	Shapiro-Wilk score	4.070E-01	3.254E-01	6.013E-01	7.405E-01	6.509E-01	6.167E-01	6.671E-01	6.648E-01	4.881E-01	7.157E-01	9.836E-01	8.844E-01
(Phosphoenolpyruvate synthesis)	Local	0.00E+00	0.00E+00	-1.78E-03	-2.42E-15	1.16E-15	-4.28E-04	-2.23E-03	-6.32E-04	-5.04E-05	-2.40E-02	5.89E-02	2.91E-18
	Min	-3.74E-13	-2.56E-13	-9.73E-02	-8.21E-14	-1.16E-13	-2.01E-02	-6.51E-02	-1.95E-02	-5.12E-03	-2.11E-01	-9.13E-05	-7.52E-17
	Max	4.32E-13	3.44E-13	2.58E-02	1.42E-13	1.02E-13	5.06E-03	1.83E-02	5.07E-03	1.09E-03	3.11E-01	1.97E-01	1.57E-16
	Normalized peak height	6.06E-01	7.22E-01	4.07E-02	1.58E-01	1.71E-01	3.22E-02	2.33E-02	2.44E-02	1.02E-01	1.33E-02	1.86E-02	2.86E-01
	Peak sensitivity value	-3.38E-16	4.12E-18	-8.82E-04	-2.48E-17	1.07E-16	-1.59E-04	-1.28E-03	-3.27E-04	-9.77E-06	-2.67E-04	7.43E-06	4.64E-20
	Shapiro-Wilk score	3.502E-01	3.407E-01	5.973E-01	7.505E-01	6.417E-01	6.122E-01	6.625E-01	6.608E-01	4.933E-01	7.244E-01	9.834E-01	8.074E-01
(Pyruvate kinase)	Local	0.00E+00	0.00E+00	-1.78E-03	4.02E-15	-8.26E-16	-4.28E-04	-2.23E-03	-6.32E-04	-5.04E-05	-2.40E-02	5.89E-02	2.91E-18
	Min	-3.21E-13	-4.82E-13	-8.76E-02	-1.62E-13	-1.11E-13	-1.87E-02	-5.97E-02	-2.05E-02	-6.03E-03	-2.16E-01	-5.37E-05	-8.98E-17
	Max	2.49E-13	3.11E-13	1.99E-02	2.15E-13	2.22E-13	5.04E-03	2.49E-02	6.43E-03	2.00E-03	3.54E-01	1.87E-01	9.97E-16
	Normalized peak height	6.97E-01	6.97E-01	3.62E-02	1.98E-01	2.60E-01	3.15E-02	2.34E-02	2.56E-02	1.27E-01	1.46E-02	2.23E-02	3.36E-01
	Peak sensitivity value	6.31E-17	-2.36E-16	-9.31E-04	1.04E-16	-4.58E-17	-1.88E-04	-1.36E-03	-3.57E-04	-9.73E-06	-4.52E-04	3.96E-05	-1.36E-19
	Shapiro-Wilk score	3.094E-01	3.037E-01	6.049E-01	6.525E-01	5.455E-01	6.188E-01	6.665E-01	6.603E-01	4.924E-01	7.236E-01	9.832E-01	4.584E-01
(Pyruvate decarboxylase)	Local	0.00E+00	0.00E+00	-1.78E-03	-6.65E-16	1.91E-15	-4.28E-04	-2.23E-03	-6.32E-04	-5.04E-05	-2.40E-02	5.89E-02	2.91E-18
	Min	-4.67E-14	-2.57E-13	-8.43E-02	-1.50E-13	-1.06E-13	-2.31E-02	-6.14E-02	-1.99E-02	-5.79E-03	-1.82E-01	-1.71E-04	-7.26E-17
	Max	6.41E-14	3.33E-13	1.85E-02	1.40E-13	1.19E-13	5.53E-03	1.77E-02	4.94E-03	1.39E-03	3.40E-01	1.91E-01	2.08E-16
	Normalized peak height	4.51E-01	6.44E-01	3.35E-02	1.87E-01	2.25E-01	3.66E-02	2.11E-02	2.51E-02	1.13E-01	1.28E-02	2.20E-02	2.87E-01
	Peak sensitivity value	1.30E-18	2.15E-16	-9.59E-04	-1.04E-16	4.04E-17	-1.80E-04	-1.19E-03	-4.11E-04	-1.10E-05	-2.54E-02	1.16E-04	-3.71E-20
	Shapiro-Wilk score	4.174E-01	3.149E-01	6.066E-01	6.061E-01	5.473E-01	6.161E-01	6.701E-01	6.629E-01	4.979E-01	7.164E-01	9.832E-01	8.246E-01
(Alcohol dehydrogenase)	Local	0.00E+00	0.00E+00	-3.13E-02	-1.84E-16	1.26E-15	-7.52E-03	-3.91E-02	-1.11E-02	-8.85E-04	-1.89E-02	5.21E-02	2.13E-18
	Min	-4.27E-14	-2.10E-13	-2.81E-01	-1.23E-13	-5.20E-14	-5.35E-02	-1.54E-01	-4.63E-02	-1.55E-02	-2.58E-01	-1.97E-04	-1.24E-16
	Max	4.47E-14	1.49E-13	-1.13E-03	1.21E-13	6.93E-14	-4.81E-04	-5.48E-03	-1.45E-03	-6.85E-06	2.95E-01	1.83E-01	1.65E-16
	Normalized peak height	4.35E-01	6.15E-01	7.84E-03	1.72E-01	1.89E-01	6.43E-03	4.00E-03	4.39E-03	1.85E-02	1.40E-02	2.42E-02	2.84E-01
	Peak sensitivity value	-2.56E-17	1.25E-16	-1.50E-02	-1.22E-16	7.81E-18	-4.91E-03	-2.04E-02	-7.84E-03	-1.23E-04	-4.86E-04	7.90E-05	1.48E-20
	Shapiro-Wilk score	4.793E-01	3.542E-01	8.584E-01	6.165E-01	6.007E-01	8.747E-01	9.274E-01	9.261E-01	7.120E-01	7.345E-01	9.784E-01	8.023E-01
(Ethanol out)	Local	2.37E-16	-8.88E-16	-3.13E-02	-1.07E-16	1.31E-15	-7.52E-03	-3.91E-02	-1.11E-02	-8.85E-04	-1.89E-02	5.21E-02	2.13E-18
	Min	-2.76E-14	-2.29E-13	-2.51E-01	-5.79E-14	-5.10E-14	-5.15E-02	-1.61E-01	-4.77E-02	-1.46E-02	-1.96E-01	-1.54E-04	-8.98E-17
	Max	3.15E-14	2.13E-13	-9.47E-04	6.64E-14	7.27E-14	-4.45E-04	-5.41E-03	-1.11E-03	-6.23E-06	5.66E-01	1.85E-01	1.28E-16
	Normalized peak height	5.81E-02	4.11E-01	7.37E-03	1.40E-01	1.93E-01	6.27E-03	4.22E-03	4.52E-03	1.76E-02	1.93E-02	1.94E-02	2.76E-01
	Peak sensitivity value	-2.16E-16	1.89E-16	-1.46E-02	-3.95E-17	1.26E-17	-4.56E-03	-2.56E-02	-7.66E-03	-1.59E-04	-6.27E-04	1.24E-04	-3.91E-20
	Shapiro-Wilk score	8.137E-01	4.225E-01	8.570E-01	6.626E-01	6.047E-01	8.720E-01	9.253E-01	9.255E-01	7.083E-01	7.388E-01	9.788E-01	8.170E-01
(Ethanol flow)	Local	-3.41E-16	-1.11E-15	-3.13E-02	-1.73E-16	1.29E-15	-7.52E-03	-3.91E-02	-1.11E-02	-8.85E-04	-1.89E-02	5.21E-02	2.13E-18
	Min	-6.17E-14	-2.12E-13	-2.25E-01	-8.45E-14	-8.82E-14	-5.34E-02	-1.54E-01	-4.67E-02	-1.36E-02	-3.25E-01	-3.29E-04	-1.32E-16
	Max	3.75E-14	1.38E-13	-1.29E-03	7.15E-14	6.33E-14	-4.56E-04	-5.63E-03	-1.22E-03	-7.18E-06	2.94E-01	1.82E-01	1.18E-16
	Normalized peak height	3.49E-01	1.69E-01	6.18E-03	1.72E-01	2.18E-01	6.38E-03	4.12E-03	4.62E-03	1.69E-02	1.55E-02	1.83E-02	2.81E-01
	Peak sensitivity value	-1.43E-17	-3.32E-16	-1.53E-02	-3.10E-17	1.30E-17	-4.14E-03	-2.57E-02	-9.20E-03	-1.64E-04	-1.65E-04	1.28E-04	-8.67E-21
	Shapiro-Wilk score	4.158E-01	4.389E-01	8.596E-01	6.500E-01	5.846E-01	8.712E-01	9.257E-01	9.233E-01	7.184E-01	7.355E-01	9.783E-01	8.071E-01
(Glycerol synthesis)	Local	0.00E+00	0.00E+00	2.79E-01	8.97E-15	-6.51E-15	6.70E-02	3.48E-01	9.89E-02	7.89E-03	-7.27E-02	1.24E-01	1.03E-17
	Min	-9.19E-13	-5.59E-12	4.09E-03	-6.62E-13	-4.81E-13	1.35E-03	1.49E-02	2.60E-03	2.17E-04	-4.21E-01	-2.80E-02	-3.09E-16
	Max	8.67E-13	7.09E-12	7.05E-01	6.25E-13	3.62E-13	2.86E-01	6.86E-01	3.49E-01	6.07E-02	8.94E-01	4.29E-01	5.32E-16
	Normalized peak height	6.81E-01	7.80E-01	2.38E-03	1.20E-01	1.35E-01	4.20E-03	2.72E-03	3.56E-03	8.15E-03	1.61E-02	2.74E-02	2.54E-01
	Peak sensitivity value	-4.15E-16	-2.77E-15	3.00E-01	2.33E-16	1.97E-16	4.45E-02	3.52E-01	8.42E-02	2.85E-03	-6.66E-02	1.41E-04	4.80E-20
	Shapiro-Wilk score	3.597E-01	3.092E-01	9.870E-01	7.980E-01	7.704E-01	9.371E-01	9.972E-01	9.670E-01	8.485E-01	7.224E-01	9.829E-01	8.774E-01

(Glycerol out) Local	2.51E-02	1.08E-02	1.96E+00	8.63E-03	1.04E-01	8.12E-15	-8.18E-02	-1.79E-01	-1.53E-02	-4.63E-02	1.55E-14	-1.64E+00	0.00E+00	0.00E+00	2.79E-01	9.24E-15	-6.55E-15	6.70E-02	3.48E-01	9.89E-02	7.89E-03	-7.27E-02	1.24E-01	1.03E-17
(Glycerol out) Min	-7.46E-01	-5.28E-01	-3.00E+00	1.94E-05	1.03E-02	-1.60E-13	-4.76E-01	-4.94E-01	-8.04E-02	-3.34E-01	-1.28E-12	-3.74E+00	-1.07E-12	-5.70E-12	2.77E-03	-4.97E-13	-3.42E-13	1.18E-03	1.92E-02	2.25E-03	2.19E-04	-4.20E-01	-1.40E-02	-2.48E-16
(Glycerol out) Max	3.11E+00	2.96E+00	3.76E+00	1.48E-01	3.02E+00	1.29E-13	-1.42E-04	-5.54E-03	-1.22E-04	-9.56E-05	9.15E-13	-5.61E-01	8.62E-13	4.10E-12	6.82E-01	4.20E-13	4.68E-13	2.96E-01	6.96E-01	3.49E-01	5.47E-02	1.36E+00	4.24E-01	1.98E-14
(Glycerol out) Normalized peak height	1.78E-01	2.06E-01	5.48E-02	1.49E-02	2.76E-02	1.03E-01	4.64E-03	2.72E-03	5.72E-03	8.06E-03	4.06E-01	4.98E-03	5.95E-01	7.75E-01	2.44E-03	7.81E-02	1.32E-01	3.98E-03	2.87E-03	3.48E-03	7.35E-03	2.23E-02	2.66E-02	6.43E-01
(Glycerol out) Peak sensitivity value	4.10E-03	1.28E-03	4.23E-03	5.11E-03	5.70E-02	7.82E-17	-4.61E-02	-1.17E-01	-7.79E-03	-1.33E-02	-9.87E-16	-1.19E+00	8.25E-16	-4.76E-16	2.96E-01	-4.38E-16	1.21E-16	4.70E-02	3.40E-01	8.15E-02	3.24E-03	-6.42E-02	2.31E-04	2.69E-18
(Glycerol out) Shapiro-Wilk score	6.020E-01	4.862E-01	8.916E-01	7.449E-01	6.055E-01	7.413E-01	9.069E-01	9.791E-01	9.155E-01	8.386E-01	4.843E-01	9.457E-01	3.654E-01	3.240E-01	9.870E-01	8.228E-01	7.727E-01	9.354E-01	9.975E-01	9.667E-01	8.404E-01	7.112E-01	9.833E-01	2.444E-02
(Glycerol flow) Local	2.51E-02	1.08E-02	1.96E+00	8.63E-03	1.04E-01	8.14E-15	-8.18E-02	-1.79E-01	-1.53E-02	-4.63E-02	1.49E-14	-1.64E+00	0.00E+00	0.00E+00	2.79E-01	9.72E-15	-6.66E-15	6.70E-02	3.48E-01	9.89E-02	7.89E-03	-7.27E-02	1.24E-01	1.03E-17
(Glycerol flow) Min	-3.02E-01	-8.63E-02	-7.71E-01	1.82E-05	1.91E-02	-1.60E-13	-6.14E-01	-5.00E-01	-7.98E-02	-3.49E-01	-4.98E-13	-3.48E+00	-8.46E-13	-3.55E-12	2.69E-03	-7.78E-13	-3.91E-13	8.73E-04	1.83E-02	1.77E-03	2.17E-04	-3.70E-01	-2.66E-02	-2.72E-16
(Glycerol flow) Max	3.70E+00	1.42E+00	3.66E+00	1.46E-01	3.01E+00	2.25E-13	-1.60E-04	-4.25E-03	-6.61E-05	-9.98E-05	6.66E-13	-4.81E-01	6.54E-13	6.64E-12	6.94E-01	8.03E-13	3.35E-13	2.82E-01	6.95E-01	3.56E-01	5.47E-02	9.22E-01	5.04E-01	3.58E-16
(Glycerol flow) Normalized peak height	1.84E-01	1.20E-01	3.91E-02	1.47E-02	2.75E-02	1.27E-01	5.93E-03	2.74E-03	5.84E-03	8.54E-03	3.76E-01	4.76E-03	6.03E-01	7.79E-01	2.42E-03	1.26E-01	1.29E-01	3.94E-03	2.83E-03	3.58E-03	7.16E-03	1.64E-02	3.01E-02	2.53E-01
(Glycerol flow) Peak sensitivity value	4.90E-04	1.63E-03	3.10E-03	5.49E-03	5.95E-02	1.39E-16	-4.65E-02	-1.43E-01	-9.03E-03	-7.60E-03	5.19E-16	-1.18E+00	5.25E-16	4.28E-16	2.65E-01	5.65E-16	1.39E-16	4.18E-02	3.40E-01	7.24E-02	2.86E-03	-6.02E-02	1.54E-04	-1.32E-19
(Glycerol flow) Shapiro-Wilk score	6.030E-01	4.873E-01	8.915E-01	7.380E-01	6.068E-01	7.356E-01	9.055E-01	9.785E-01	9.131E-01	8.404E-01	5.029E-01	9.489E-01	3.712E-01	3.074E-01	9.861E-01	7.823E-01	7.601E-01	9.379E-01	9.969E-01	9.675E-01	8.445E-01	7.110E-01	9.825E-01	8.738E-01
(Acetaldehyde out) Local	2.51E-02	1.08E-02	1.96E+00	8.63E-03	1.04E-01	-5.92E-15	-8.18E-02	-1.79E-01	-1.53E-02	-4.63E-02	4.56E-21	-1.64E+00	0.00E+00	0.00E+00	2.79E-01	-5.24E-15	8.08E-15	6.70E-02	3.48E-01	9.89E-02	7.89E-03	-7.27E-02	1.24E-01	1.03E-17
(Acetaldehyde out) Min	-1.90E-01	-1.19E-01	-3.97E-01	1.19E-05	1.91E-02	-2.28E-13	-5.12E-01	-5.11E-01	-7.70E-02	-2.99E-01	-7.47E-13	-3.64E+00	-9.45E-13	-4.17E-12	4.45E-03	-5.04E-13	-4.61E-13	1.08E-03	1.25E-02	1.70E-03	2.24E-04	-4.24E-01	-2.91E-02	-3.44E-16
(Acetaldehyde out) Max	3.14E+00	1.72E+00	3.94E+00	1.23E-01	3.22E+00	1.46E-13	-8.90E-05	-5.04E-03	-7.47E-05	-1.03E-04	1.21E-12	-5.83E-01	7.27E-13	2.99E-12	6.87E-01	6.60E-13	2.79E-13	3.05E-01	7.08E-01	3.66E-01	5.26E-02	8.75E-01	4.43E-01	5.26E-16
(Acetaldehyde out) Normalized peak height	2.26E-01	1.51E-01	3.70E-02	1.32E-02	2.87E-02	1.36E-01	5.27E-03	2.80E-03	5.54E-03	7.34E-03	4.53E-01	5.22E-03	6.36E-01	7.70E-01	2.30E-03	1.20E-01	1.37E-01	4.38E-03	3.05E-03	3.94E-03	7.00E-03	1.65E-02	2.07E-02	2.58E-01
(Acetaldehyde out) Peak sensitivity value	1.50E-03	1.62E-03	3.44E-03	5.49E-03	5.91E-02	7.35E-17	-5.16E-02	-1.38E-01	-8.73E-03	-9.52E-03	4.36E-16	-1.22E+00	4.85E-16	1.36E-15	2.89E-01	-5.11E-16	-2.58E-16	4.71E-02	3.28E-01	6.78E-02	2.92E-03	-6.47E-02	-7.06E-05	-4.12E-19
(Acetaldehyde out) Shapiro-Wilk score	6.015E-01	4.872E-01	8.910E-01	7.352E-01	6.076E-01	6.761E-01	9.099E-01	9.793E-01	9.093E-01	8.420E-01	5.592E-01	9.466E-01	4.062E-01	2.915E-01	9.869E-01	7.929E-01	7.567E-01	9.381E-01	9.973E-01	9.670E-01	8.449E-01	7.065E-01	9.829E-01	8.334E-01
(Acetaldehyde flow) Local	2.55E-02	1.10E-02	1.99E+00	8.78E-03	1.06E-01	-5.73E-15	-8.32E-02	-1.82E-01	-1.56E-02	-4.71E-02	-8.63E-15	-1.67E+00	0.00E+00	0.00E+00	2.84E-01	-5.10E-15	7.94E-15	6.82E-02	5.75E-01	-1.21E-01	-9.62E-03	-7.39E-02	1.27E-01	1.05E-17
(Acetaldehyde flow) Min	-2.59E-01	-1.49E-01	-5.44E+00	1.86E-05	1.82E-02	-1.13E-13	-6.16E-01	-5.19E-01	-7.92E-02	-3.84E-01	-4.85E-13	-3.94E+00	-4.82E-13	-4.46E-12	3.45E-03	-7.21E-13	-3.60E-13	1.26E-03	1.09E-01	-3.52E-01	-1.75E-01	-3.95E-01	-1.43E-02	-5.27E-16
(Acetaldehyde flow) Max	3.56E+00	2.57E+00	4.13E+00	1.50E-01	3.13E+00	1.20E-13	-1.86E-04	-3.99E-03	-7.79E-05	-1.05E-04	5.62E-13	-5.50E-01	6.26E-13	4.55E-12	6.89E-01	4.06E-13	2.35E-13	2.91E-01	9.09E-01	-2.18E-02	-1.46E-04	1.71E+00	4.52E-01	3.72E-16
(Acetaldehyde flow) Normalized peak height	2.46E-01	1.93E-01	6.97E-02	1.44E-02	2.58E-02	1.01E-01	6.03E-03	2.91E-03	5.89E-03	9.44E-03	3.45E-01	5.16E-03	5.59E-01	7.83E-01	2.37E-03	1.27E-01	1.32E-01	4.14E-03	3.00E-03	3.57E-03	1.77E-02	2.60E-02	2.24E-02	2.55E-01
(Acetaldehyde flow) Peak sensitivity value	2.78E-03	2.14E-03	3.51E-03	5.18E-03	6.01E-02	-9.92E-17	-4.66E-02	-1.39E-01	-9.38E-03	-1.14E-02	-5.96E-17	-1.20E+00	5.05E-16	7.03E-16	2.61E-01	4.95E-16	1.32E-16	3.70E-02	6.16E-01	-1.14E-01	-2.51E-03	-6.54E-02	3.42E-04	-1.15E-19
(Acetaldehyde flow) Shapiro-Wilk score	6.050E-01	4.863E-01	8.956E-01	7.485E-01	6.032E-01	6.808E-01	9.089E-01	9.792E-01	9.111E-01	8.391E-01	4.323E-01	9.440E-01	3.211E-01	2.783E-01	9.873E-01	7.921E-01	7.656E-01	9.367E-01	9.850E-01	9.710E-01	6.750E-01	7.055E-01	9.829E-01	8.485E-01
(Cyanide-Acetaldehyde flow) Local	2.36E-02	1.02E-02	1.85E+00	8.13E-03	9.84E-02	-5.30E-15	-7.71E-02	-1.69E-01	-1.45E-02	-4.36E-02	-8.00E-15	-1.54E+00	0.00E+00	0.00E+00	2.63E-01	-4.73E-15	7.34E-15	6.31E-02	-3.93E-01	8.14E-01	6.50E-02	-6.85E-02	1.17E-01	9.70E-18
(Cyanide-Acetaldehyde flow) Min	-2.03E-01	-9.12E-02	-5.52E+00	1.59E-05	1.74E-02	-1.10E-13	-5.01E-01	-4.89E-01	-7.85E-02	-3.35E-01	-7.97E-13	-3.34E+00	-6.61E-13	-3.99E-12	1.89E-03	-3.27E-13	-2.29E-13	9.08E-04	-7.55E-01	3.34E-01	3.29E-03	-3.51E-01	-1.61E-02	-1.05E-15
(Cyanide-Acetaldehyde flow) Max	3.07E+00	3.03E+00	3.68E+00	1.15E-01	3.10E+00	1.41E-12	-9.93E-05	-3.55E-03	-3.28E-05	-5.36E-05	4.28E-13	-3.82E-01	4.71E-13	5.48E-12	6.56E-01	4.33E-13	2.79E-13	2.79E-01	-9.40E-02	9.68E-01	5.69E-01	1.97E+00	3.59E-01	4.98E-16
(Cyanide-Acetaldehyde flow) Normalized peak height	2.14E-01	1.86E-01	7.52E-02	1.22E-02	2.91E-02	3.76E-01	5.12E-03	2.68E-03	6.17E-03	8.30E-03	3.83E-01	5.19E-03	6.42E-01	7.87E-01	2.47E-03	1.17E-01	1.27E-01	3.96E-03	2.88E-03	4.05E-03	9.03E-03	2.96E-02	2.47E-02	2.69E-01
(Cyanide-Acetaldehyde flow) Peak sensitivity value	1.24E-03	1.01E-03	4.93E-03	4.90E-03	5.89E-02	3.85E-16	-3.84E-02	-1.45E-01	-9.17E-03	-1.06E-02	-5.66E-16	-1.10E+00	-8.20E-17	2.24E-15	2.92E-01	1.94E-16	1.50E-16	3.72E-02	-3.73E-01	8.48E-01	2.62E-02	-6.41E-02	2.31E-04	5.38E-19
(Cyanide-Acetaldehyde flow) Shapiro-Wilk score	6.057E-01	4.878E-01	8.772E-01	7.635E-01	6.056E-01	3.084E-01	9.103E-01	9.784E-01	9.128E-01	8.392E-01	4.350E-01	9.565E-01	3.352E-01	2.797E-01	9.861E-01	8.215E-01	7.665E-01	9.372E-01	9.955E-01	9.490E-01	7.960E-01	7.066E-01	9.830E-01	7.620E-01
(Cyanide flow) Local	2.36E-02	1.02E-02	1.85E+00	8.13E-03	9.84E-02	-5.36E-15	-7.71E-02	-1.69E-01	-1.45E-02	-4.36E-02	-7.92E-15	-1.54E+00	0.00E+00	0.00E+00	2.63E-01	-4.59E-15	7.47E-15	6.31E-02	-3.93E-01	8.14E-01	6.50E-02	-6.85E-02	1.17E-01	9.70E-18
(Cyanide flow) Min	-2.83E-01	-1.97E-01	-5.36E-01	2.11E-05	1.82E-02	-1.74E-13	-4.64E-01	-4.78E-01	-7.86E-02	-2.87E-01	-8.57E-13	-3.08E+00	-5.94E-13	-3.16E-12	3.08E-03	-4.43E-13	-2.65E-13	1.04E-03	-7.49E-01	3.51E-01	2.36E-03	-3.72E-01	-2.04E-02	-2.96E-16
(Cyanide flow) Max	2.83E+00	1.64E+00	3.23E+00	1.30E-01	2.98E+00	1.01E-13	-1.07E-04	-2.38E-03	-4.19E-05	-1.67E-05	6.34E-13	-2.65E-01	7.60E-13	6.43E-12	6.60E-01	3.97E-13	4.57E-13	3.04E-01	-9.98E-02	9.65E-01	5.66E-01	9.28E-01	3.78E-01	3.18E-16
(Cyanide flow) Normalized peak height	2.28E-01	1.31E-01	3.13E-02	1.43E-02	2.83E-02	1.33E-01	4.75E-03	2.77E-03	6.02E-03	7.29E-03	4.31E-01	5.22E-03	6.70E-01	7.84E-01	2.27E-03	1.24E-01	1.41E-01	4.75E-03	3.09E-03	4.02E-03	9.04E-03	1.69E-02	2.70E-02	2.55E-01
(Cyanide flow) Peak sensitivity value	1.54E-03	2.38E-03	4.17E-03	5.02E-03	5.81E-02	5.56E-17	-2.63E-02	-1.40E-01	-8.96E-03	-1.11E-02	-7.35E-16	-1.14E+00	-1.95E-16	1.94E-16	2.78E-01	8.82E-17	-2.34E-16	4.45E-02	-3.99E-01	8.56E-01	2.91E-02	-6.30E-02	1.41E-04	-8.42E-20
(Cyanide flow) Shapiro-Wilk score	6.041E-01	4.896E-01	8.773E-01	7.631E-01	6.053E-01	6.597E-01	9.096E-01	9.781E-01	9.099E-01	8.421E-01	3.961E-01	9.608E-01	3.242E-01	2.831E-01	9.861E-01	8.133E-01	7.501E-01	9.374E-01	9.958E-01	9.452E-01	7.835E-01	7.114E-01	9.830E-01	8.875E-01
(Storage) Local	1.21E-02	5.20E-03	9.43E-01	-1.53E-03	-1.85E-02	-1.69E-15	9.90E-03	2.16E-02	1.86E-03	5.60E-03	-3.99E-15	1.98E-01	0.00E+00	0.00E+00	-3.37E-02	-2.74E-15	-1.38E-16	-8.11E-03	-4.21E-02	-1.20E-02	-9.55E-04	1.62E-02	-9.65E-02	-1.73E-18
(Storage) Min	-2.46E-01	-1.83E-01	-3.99E+00	-2.56E-02	-9.88E-01	-5.21E-14	-7.30E-04	-1.62E-03	-9.89E-05	-9.01E-04	-3.40E-13	-2.30E-02	-1.07E-13	-7.70E-13	-2.31E-01	-4.09E-13	-5.70E-13	-5.01E-02	-1.55E-01	-5.37E-02	-1.71E-02	-1.59E-01	-5.03E-01	-2.68E-16
(Storage) Max	1.59E+00	2.35E+00	1.82E+00	4.21E-02	1.19E+00	1.30E-13	1.04E-01	1.39E-01	2.94E-02	6.52E-02	2.00E-13	1.10E+00	1.85E-13	6.86E-13	2.66E-03	5.22E-13	2.65E-13	1.56E-03	4.21E-03	2.40E-03	1.16E-04	1.57E+00	2.23E-02	2.03E-15
(Storage) Normalized peak height	1.56E-01	1.84E-01	5.73E-02	1.51E-02	4.05E-02	1.25E-01	9.88E-03	6.15E-03	1.55E-02	1.44E-02	3.53E-01	6.49E-03	4.50E-01	6.50E-01	6.42E-03	1.84E-01	2.21E-01	5.81E-03	4.21E-03	5.36E-03	1.90E-02	3.43E-02	4.95E-03	4.97E-01
(Storage) Peak sensitivity value	6.80E-04	5.43E-04	3.75E-03	-1.02E-03	-1.38E-02	-8.46E-17	4.14E-03	1.14E-02	5.65E-04	9.17E-04	2.41E-16	7.74E-02	-5.45E-17	-4.84E-16	-1.45E-02	3.30E-16	2.87E-16	-5.44E-03	-2.72E-02	-9.03E-03	-1.67E-04	2.15E-02	-7.45E-02	-8.47E-19
(Storage) Shapiro-Wilk score	6.007E-01	5.345E-01	8.289E-01	8.715E-01	6.014E-01	6.981E-01	8.397E-01	8.805E-01	7.755E-01	7.188E-01	4.542E-01	8.308E-01	3.752E-01	3.252E-01	8.711E-01	6.395E-01	6.016E-01	8.765E-01	9.316E-01	9.304E-01	7.178E-01	7.092E-01	9.384E-01	1.199E-01
(ATP consumption) Local	-1.59E-03	-6.83E-04	-1.24E-01	2.57E-02	3.11E-01	-8.56E-16	4.80E-03	1.05E-02	9.01E-04	2.72E-03	-2.17E-15	9.61E-02	0.00E+00	0.00E+00	-1.64E-02	-1.37E-15	-6.97E-17	-3.93E-03	-2.04E-02	-5.81E-03	-4.63E-04	-2.32E-01	9.53E-01	2.55E-17
(ATP consumption) Min	-1.62E+01	-2.41E+00	-9.67E+00	4.55E-06	3.28E-02	-4.11E-13	-1.88E-03	-5.22E-03	-1.06E-03	-2.95E-04	-3.71E-13	-4.41E-02	-3.11E-13	-9.52E-13	-6.92E-01	-5.85E-13	-4.68E-13	-3.51E-01	-5.70E-01	-1.81E-01	-3.76E-02	-1.94E+00	4.10E-01	-3.16E-14
(ATP consumption) Max	3.00E+00	6.15E-01	3.70E+00	1.78E-01	1.75E+01	1.38E-13	2.36E-01	5.44E-01	5.44E-02	1.42E-01	7.74E-13	4.32E+00	3.05E-13	1.32E-12	7.44E-03	6.14E-13	5.85E-13	2.67E-03	1.89E-02	2.96E-03	1.52E-04	4.62E-01	1.01E+00	2.29E-15
(ATP consumption) Normalized peak height	5.94E-01	5.20E-01	1.11E-01	6.52E-03	5.21E-02	4.00E-01	3.84E-02	3.65E-02	4.75E-02	4.68E-02	4.86E-01	3.81E-02	6.39E-01	7.55E-01	2.84E-02	2.93E-01	3.48E-01	5.82E-02	2.07E-02	2.28E-02	7.61E-02	1.25E-02	8.98E-03	5.10E-01
(ATP consumption) Peak sensitivity value	-6.96E-03	6.90E-05	-7.38E-03	2.03E-02	1.81E-01	-8.04E-17	1.80E-03	3.83E-03	2.99E-04	7.75E-04	-4.50E-16	3.22E-02	1.72E-16	-6.96E-16	-8.29E-03	-2.44E-17	1.71E-16	-1.75E-03	-1.09E-02	-3.37E-03	-5.53E-05	-2.36E-01	9.57E-01	5.09E-18
(ATP consumption) Shapiro-Wilk score	2.893E-01	4.517E-01	4.785E-01	8.936E-01	6.169E-01	3.923E-01	5.666E-01	6.398E-01	5.580E-01	5.852E-01	2.484E-01	6.469E-01	2.046E-01	2.052E-01	6.362E-01	4.395E-01	3.863E-01	6.299E-01	6.968E-01	6.812E-01	5.377E-01	8.215E-01	8.123E-01	4.255E-02
(Adenylate kinase) Local	-INF	-INF	-INF	-INF	-INF	-INF	-INF	-INF	-INF	-INF	-INF	-INF	-INF	-INF	-INF	-INF	-INF	-INF	-INF	-INF	-INF	-INF	-INF	-INF
(Adenylate kinase) Min	-INF	-INF	-INF	-INF	-INF	-INF	-INF	-INF	-INF	-INF	-INF	-INF	-INF	-INF	-INF	-INF	-INF	-INF	-INF	-INF	-INF	-INF	-INF	-INF
(Adenylate kinase) Max	INF	INF	INF	INF	INF	INF	INF	INF	INF	INF	INF	INF	INF	INF	INF	INF	INF	INF	INF	INF	INF	INF	INF	INF
(Adenylate kinase) Normalized peak height	NA	NA	NA	NA	NA	NA	NA	NA	NA	NA	NA	NA	NA	NA	NA	NA	NA	NA	NA	NA	NA	NA	NA	NA
(Adenylate kinase) Peak sensitivity value	NAN	NAN	NAN	NAN	NAN	NAN	NAN	NAN	NAN	NAN	NAN	NAN	NAN	NAN	NAN	NAN	NAN	NAN	NAN	NAN	NAN	NAN	NAN	NAN
(Adenylate kinase) Shapiro-Wilk score	NA	NA	NA	NA	NA	NA	NA	NA	NA	NA	NA	NA	NA	NA	NA	NA	NA	NA	NA	NA	NA	NA	NA	NA

Table S31: Flux control coefficients for *Saccharomyces cerevisiae* model with parameter variation of 0.1 – 10×

	(GluconseMixedflowtoextracellularmedium)	(Glucoseuptake)	(Hexokinase)	(Phosphoglucoisomerase)	(Phosphofructokinase)	(Aldolase)	(Triosephosphateisomerase)	(Glyceraldehyde3-phosphatedehydrogenase)	(Phosphoenolpyruvatesynthesis)	(Pyruvatekinase)	(Pyruvatedecarboxylase)	(Alcoholdehydrogenase)	(Ethanolout)	(Ethanolflow)	(Glycerolsynthesis)	(Glycerolout)	(Glycerolflow)	(Acetaldehydeout)	(Acetaldehydeflow)	(Cyanide-Acetaldehydeflow)	(Cyanideflow)	(Storage)	(ATPconsumption)	(Adenylatekinase)
(Glucose Mixed flow to extracellular medium) Local	1.25E-02	5.36E-03	9.73E-01	1.37E-03	1.65E-02	-3.45E-17	2.30E-04	5.02E-04	4.31E-05	1.30E-04	-8.14E-17	4.60E-03	0.00E+00	0.00E+00	-7.83E-04	-6.35E-17	-5.80E-18	-1.88E-04	-9.78E-04	-2.78E-04	-2.22E-05	-9.81E-03	-2.24E-03	1.36E-18
(Glucose Mixed flow to extracellular medium) Min	-1.00E-01	-3.33E-01	-1.32E+01	-2.71E-01	-9.66E-01	-1.33E-11	-2.04E-01	-7.69E+00	-7.92E-03	-9.48E-01	-9.54E-12	-9.90E+00	-8.06E-12	-1.02E-11	-7.40E+02	-7.80E-11	-1.11E-11	-5.09E+02	-1.75E+02	-8.58E+01	-4.64E-01	-1.26E+01	-1.78E+03	-1.96E-09
(Glucose Mixed flow to extracellular medium) Max	1.02E+00	1.01E+01	1.78E+03	9.68E-01	6.42E+00	2.18E-11	3.80E+01	7.01E+02	2.98E+00	2.46E+00	2.52E-12	6.26E+02	4.05E-12	2.05E-11	9.59E+00	4.53E-10	1.87E-11	6.14E+00	4.21E+00	2.00E+00	2.94E-02	2.15E+00	1.24E+01	1.87E-12
(Glucose Mixed flow to extracellular medium) Normalized peak height	7.78E-02	1.90E-01	8.56E-01	1.79E-01	1.16E-01	9.71E-01	9.59E-01	9.71E-01	9.60E-01	8.99E-01	9.75E-01	9.03E-01	9.48E-01	9.61E-01	9.73E-01	8.83E-01	9.33E-01	8.28E-01	8.92E-01	8.72E-01	6.74E-01	2.24E-01	7.81E-01	1.00E+00
(Glucose Mixed flow to extracellular medium) Peak sensitivity value	9.36E-04	6.68E-03	2.04E-01	1.04E-04	4.29E-03	-2.77E-15	6.70E-03	-2.52E-01	-4.44E-04	7.24E-05	-3.53E-15	-3.71E-02	9.38E-16	-1.07E-14	2.21E-01	-2.61E-13	1.15E-14	2.17E-01	1.11E-02	2.38E-02	2.34E-05	2.21E-03	8.08E-01	8.88E-13
(Glucose Mixed flow to extracellular medium) Shapiro-Wilk score	6.352E-01	7.430E-01	1.956E-02	4.731E-01	7.064E-01	1.276E-02	7.987E-03	1.600E-03	1.107E-02	4.352E-02	8.873E-03	1.460E-02	3.486E-02	1.490E-02	1.944E-03	8.058E-03	3.107E-02	4.947E-03	2.093E-02	1.583E-02	1.599E-01	5.521E-01	9.301E-03	4.663E-04

(Glucose uptake)	Local	1.25E-02	5.36E-03	9.73E-01	1.37E-03	1.65E-02	-2.20E-17	2.30E-04	5.02E-04	4.31E-05	1.30E-04	-8.36E-17	4.60E-03	0.00E+00	0.00E+00	-7.83E-04	-6.57E-17	-3.32E-18	-1.88E-04	-9.78E-04	-2.78E-04	-2.22E-05	-9.81E-03	-2.24E-03	1.36E-18
	Min	-3.43E-01	-5.97E-01	-3.22E+00	-1.31E-01	-9.71E-02	-1.21E-11	-2.14E-01	-1.07E+00	-2.79E-03	-2.79E-01	-2.65E-12	-2.98E+00	-2.43E-11	-1.11E-11	-3.86E+01	-1.03E-10	-1.24E-11	-5.25E+01	-2.78E+01	-9.23E+00	-5.48E-01	-5.38E+00	-1.58E+02	-5.05E-12
	Max	1.00E+00	1.48E+00	1.60E+02	9.51E-01	2.68E+00	4.10E-11	1.26E+01	3.82E+01	4.45E+00	2.66E+00	4.68E-12	7.04E+01	6.98E-12	1.69E-11	2.99E+00	2.10E-10	9.69E-11	1.34E+00	6.78E-01	2.32E-01	5.33E-02	4.70E-01	3.01E+00	1.95E-11
	Normalized peak height	8.72E-02	1.03E-01	2.78E-01	1.94E-01	7.30E-02	9.70E-01	8.19E-01	8.91E-01	9.73E-01	8.95E-01	9.59E-01	4.77E-01	9.48E-01	9.64E-01	6.78E-01	9.62E-01	9.59E-01	4.50E-01	5.11E-01	6.73E-01	6.49E-01	1.52E-01	6.73E-01	9.99E-01
	Peak sensitivity value	-5.86E-05	1.30E-03	-4.54E-02	3.61E-04	1.57E-03	1.04E-14	-3.43E-03	9.78E-03	-5.63E-04	1.33E-03	-2.31E-15	-9.65E-03	-1.35E-14	-6.33E-15	1.45E-02	5.64E-14	4.79E-14	-3.00E-02	7.95E-03	-1.39E-04	1.27E-04	-7.74E-04	3.26E-02	-3.36E-15
	Shapiro-Wilk score	6.338E-01	7.562E-01	1.515E-01	4.746E-01	7.080E-01	1.138E-02	3.694E-02	3.022E-02	7.454E-03	4.421E-02	1.713E-02	1.036E-01	1.647E-02	1.956E-02	4.029E-02	2.086E-02	5.297E-03	4.735E-02	8.917E-02	8.317E-02	1.351E-01	5.737E-01	6.326E-02	1.595E-03
(Hexokinase)	Local	1.25E-02	5.36E-03	9.73E-01	1.37E-03	1.65E-02	-4.14E-17	2.30E-04	5.02E-04	4.31E-05	1.30E-04	-1.06E-16	4.60E-03	0.00E+00	0.00E+00	-7.83E-04	-6.57E-17	-3.32E-18	-1.88E-04	-9.78E-04	-2.78E-04	-2.22E-05	-9.81E-03	-2.24E-03	1.36E-18
	Min	-5.51E-01	-6.46E-01	-3.09E+00	-3.55E-02	-3.67E-01	-1.01E-10	-2.41E-01	-8.58E-01	-5.73E-03	-3.01E-01	-1.67E-11	-2.34E+00	-2.26E-11	-1.45E-10	-6.49E+01	-2.14E-10	-3.33E-11	-1.55E+02	-6.19E+01	-5.65E+01	-1.24E+00	-6.73E+00	-1.75E+03	-2.29E-11
	Max	1.00E+00	1.04E+00	1.75E+03	9.42E-01	3.40E+00	4.72E-11	5.46E+00	6.37E+01	2.39E+00	2.37E+00	1.99E-11	2.25E+02	1.61E-11	4.03E-11	2.31E+00	1.43E-10	1.16E-11	1.15E+00	1.62E+00	2.64E-01	1.66E-02	5.18E-01	3.02E+00	1.18E-10
	Normalized peak height	1.14E-01	9.50E-02	5.51E-01	1.69E-01	6.37E-02	9.83E-01	8.37E-01	9.28E-01	9.67E-01	7.92E-01	9.91E-01	8.04E-01	9.69E-01	9.88E-01	8.86E-01	9.67E-01	9.61E-01	9.34E-01	7.73E-01	8.65E-01	8.21E-01	1.66E-01	9.34E-01	9.99E-01
	Peak sensitivity value	2.01E-04	3.30E-04	-4.53E-01	1.37E-04	-2.10E-04	-4.39E-14	1.52E-03	1.34E-02	2.67E-04	-9.39E-04	-6.19E-15	4.68E-02	1.13E-14	6.18E-14	-6.29E-03	-3.13E-14	1.03E-14	-2.68E-02	-2.99E-03	8.96E-03	-2.83E-04	-6.80E-04	3.92E-01	5.14E-14
	Shapiro-Wilk score	6.322E-01	7.539E-01	2.195E-02	4.712E-01	7.133E-01	5.316E-03	5.263E-02	1.791E-02	1.496E-02	4.137E-02	3.730E-03	4.671E-02	1.289E-02	3.640E-03	2.345E-02	1.665E-02	1.850E-02	2.210E-02	4.865E-02	2.258E-02	7.891E-02	5.716E-01	1.134E-02	1.239E-03
(Phosphoglucoisomerase)	Local	1.27E-02	5.48E-03	9.94E-01	3.42E-03	4.14E-02	4.62E-16	-6.63E-03	-1.45E-02	-1.24E-03	-3.75E-03	2.63E-15	-1.33E-01	0.00E+00	0.00E+00	2.26E-02	1.83E-15	9.24E-17	5.43E-03	2.82E-02	8.02E-03	6.39E-04	-2.82E-02	6.46E-02	3.55E-18
	Min	-5.53E-02	-2.90E-01	-7.80E+00	-2.66E-01	-2.25E-01	-3.55E-08	-4.59E-01	-1.65E+00	-1.16E-02	-4.27E+00	-4.50E-11	-4.05E+00	-1.33E-11	-1.59E-11	-5.27E+01	-4.15E-11	-1.42E-11	-5.62E+01	-7.20E+01	-2.16E+01	-5.22E-01	-7.16E+00	-4.45E+02	-1.67E-12
	Max	4.77E+00	3.07E+00	4.47E+02	2.32E+00	3.54E+00	3.49E-10	1.28E+01	3.90E+01	5.37E+00	2.10E+00	6.28E-11	1.01E+02	6.08E-12	7.52E-11	3.62E+00	3.72E-10	6.34E-12	3.16E+00	2.48E+00	6.63E-01	1.84E-01	5.54E-01	7.29E+00	2.40E-10
	Normalized peak height	2.20E-01	1.80E-01	5.46E-01	1.03E-01	4.77E-02	1.00E+00	8.62E-01	8.55E-01	9.78E-01	8.66E-01	9.93E-01	4.73E-01	9.48E-01	9.81E-01	7.24E-01	9.68E-01	9.25E-01	6.65E-01	6.92E-01	8.00E-01	6.89E-01	8.43E-02	5.42E-01	9.99E-01
	Peak sensitivity value	1.71E-04	5.68E-04	1.55E-01	1.93E-03	9.98E-01	8.63E-12	-2.03E-03	-4.13E-03	1.85E-03	1.03E-03	2.49E-14	5.49E-03	2.81E-15	-1.67E-14	-1.71E-02	8.72E-14	6.69E-15	-1.34E-02	-1.72E-02	6.69E-03	2.52E-04	1.72E-03	-1.76E-01	-9.92E-14
	Shapiro-Wilk score	6.417E-01	7.558E-01	9.166E-02	5.194E-01	7.157E-01	4.191E-04	4.345E-02	4.335E-02	5.632E-03	6.175E-02	2.359E-03	7.911E-02	2.986E-02	5.715E-03	4.609E-02	8.437E-03	3.309E-02	5.622E-02	4.736E-02	4.693E-02	1.842E-01	5.925E-01	4.144E-02	4.439E-04
(Phosphofructokinase)	Local	1.27E-02	5.48E-03	9.94E-01	3.42E-03	4.14E-02	1.39E-15	-6.63E-03	-1.45E-02	-1.24E-03	-3.75E-03	3.85E-15	-1.33E-01	0.00E+00	0.00E+00	2.26E-02	2.02E-15	1.13E-16	5.43E-03	2.82E-02	8.02E-03	6.39E-04	-2.82E-02	6.46E-02	3.55E-18
	Min	-2.56E-02	-4.14E-02	-9.03E+00	-4.68E-02	-4.36E-01	-2.36E-11	-4.23E-01	-4.16E+00	-1.04E-02	-5.34E+00	-7.93E-12	-5.83E+00	-7.29E-12	-2.88E-11	-2.22E+01	-3.87E-10	-1.40E-11	-6.80E+01	-6.71E+01	-2.84E+01	-8.21E-01	-4.72E+00	-5.64E+02	-2.64E-11
	Max	3.53E+00	3.66E+00	5.66E+02	9.40E-01	2.51E+00	8.75E-12	4.96E+00	2.18E+01	2.50E+00	1.97E+00	1.48E-10	1.61E+02	2.83E-11	4.77E-11	6.04E+00	8.78E-11	1.36E-11	2.26E+00	2.62E+00	7.24E-01	1.12E+00	1.16E+00	8.49E+00	4.72E-12
	Normalized peak height	2.32E-01	2.03E-01	4.49E-01	8.54E-02	6.17E-02	9.50E-01	7.60E-01	7.29E-01	9.47E-01	9.03E-01	9.93E-01	6.88E-01	9.67E-01	9.80E-01	6.56E-01	9.70E-01	8.43E-01	6.38E-01	7.98E-01	8.25E-01	8.19E-01	7.27E-02	8.67E-01	9.99E-01
	Peak sensitivity value	1.10E-03	1.16E-03	-1.29E-01	3.40E-05	9.99E-01	1.04E-14	-1.19E-03	4.32E-03	8.88E-04	-1.99E-03	-4.97E-14	-7.96E-02	-4.56E-15	7.40E-15	-6.05E-03	1.22E-13	1.33E-14	-2.17E-02	4.78E-03	1.02E-02	2.49E-04	1.50E-03	1.91E-01	-2.46E-15
	Shapiro-Wilk score	6.415E-01	7.518E-01	6.042E-02	5.215E-01	7.145E-01	1.367E-02	8.883E-02	6.089E-02	1.417E-02	5.827E-02	9.845E-04	5.830E-02	1.437E-02	9.842E-03	7.751E-02	8.675E-03	2.839E-02	3.660E-02	4.179E-02	3.924E-02	1.009E-01	5.966E-01	3.007E-02	1.330E-03
(Aldolase)	Local	1.27E-02	5.48E-03	9.94E-01	3.42E-03	4.14E-02	1.11E-15	-6.63E-03	-1.45E-02	-1.24E-03	-3.75E-03	-1.02E-14	-1.33E-01	0.00E+00	0.00E+00	2.26E-02	3.98E-16	1.24E-16	5.43E-03	2.82E-02	8.02E-03	6.39E-04	-2.82E-02	6.46E-02	3.55E-18
	Min	-6.17E-02	-2.73E-01	-3.83E+00	-4.07E-04	-3.47E-03	-1.30E-09	-4.56E-01	-2.40E+00	-1.63E-02	-5.30E+00	-3.44E-10	-3.47E+00	-8.42E-12	-3.44E-11	-8.48E+01	-9.42E-11	-1.08E-11	-1.28E+02	-3.94E+02	-3.32E+02	-3.03E-01	-7.70E+00	-8.84E+02	-1.48E-09
	Max	5.73E+00	2.16E+00	8.86E+02	1.01E+00	4.01E+00	1.74E-09	2.25E+01	8.43E+01	2.33E+00	2.24E+00	1.64E-11	8.42E+02	1.11E-11	3.00E-11	3.46E+00	5.95E-11	8.87E-11	2.54E+00	6.62E-01	2.17E-01	4.93E-02	5.35E-01	3.82E+00	1.36E-10
	Normalized peak height	2.02E-01	1.75E-01	6.11E-01	8.96E-02	7.10E-02	9.88E-01	8.93E-01	9.33E-01	9.13E-01	9.04E-01	9.61E-01	7.79E-01	9.29E-01	9.67E-01	8.14E-01	9.29E-01	9.74E-01	9.20E-01	9.47E-01	9.79E-01	6.02E-01	1.07E-01	9.45E-01	1.00E+00
	Peak sensitivity value	-8.76E-04	8.26E-04	1.72E-01	1.00E-04	9.99E-01	6.47E-13	-9.07E-03	-1.42E-02	-1.07E-03	-5.91E-04	5.91E-15	3.33E-01	-5.41E-15	2.04E-14	-2.40E-02	-3.70E-14	2.84E-14	-8.75E-03	7.00E-02	5.06E-02	1.16E-04	-3.66E-03	-1.70E-01	-3.23E-13
	Shapiro-Wilk score	6.383E-01	7.529E-01	3.172E-02	5.165E-01	7.163E-01	6.460E-03	2.017E-02	1.706E-02	1.201E-02	5.825E-02	6.273E-03	1.155E-02	3.754E-02	1.350E-02	2.089E-02	3.169E-02	6.959E-03	2.024E-02	9.041E-03	3.388E-03	2.305E-01	5.931E-01	1.718E-02	4.959E-04
(Triosephosphate isomerase)	Local	1.01E-02	4.37E-03	7.92E-01	2.32E-03	2.81E-02	-1.03E-15	9.18E-03	2.01E-02	1.72E-03	5.20E-03	-5.10E-15	1.84E-01	0.00E+00	0.00E+00	-3.13E-02	-1.10E-17	1.39E-15	-7.52E-03	-3.91E-02	-1.11E-02	-8.85E-04	-1.89E-02	5.21E-02	2.13E-18
	Min	-5.94E+00	-9.44E+00	-4.86E+00	-1.26E+00	-4.25E+00	-3.37E-09	-6.62E-03	-2.48E+00	-2.66E-02	-3.35E-02	-2.46E-10	-3.62E+00	-5.98E-12	-8.43E-11	-4.90E+01	-4.41E-11	-1.13E-11	-1.31E+02	-1.31E+02	-2.20E+01	-4.99E-01	-4.13E+00	-3.92E+02	-1.23E-07
	Max	1.58E+00	2.41E+00	3.94E+02	9.37E-01	2.07E+00	3.15E-10	2.03E+01	3.64E+01	1.48E+00	1.48E+01	1.99E-11	1.74E+02	1.72E-11	6.74E-11	3.56E+00	1.77E-10	2.75E-11	1.35E+00	2.06E+00	1.13E+00	7.93E-02	8.26E-01	4.86E+00	1.74E-11
	Normalized peak height	2.38E-01	2.46E-01	5.72E-01	1.43E-01	5.61E-02	9.96E-01	7.86E-01	3.77E-01	8.40E-01	8.88E-01	9.21E-01	2.98E-01	9.43E-01	9.86E-01	4.72E-01	8.64E-01	8.67E-01	7.49E-01	5.65E-01	5.10E-01	4.99E-01	6.61E-02	7.23E-01	1.00E+00
	Peak sensitivity value	4.37E-03	-1.68E-03	1.25E-01	2.55E-04	2.98E-02	-2.63E-13	3.53E-03	-1.11E-02	-1.69E-04	3.64E-03	1.29E-13	2.04E-01	3.69E-15	-2.63E-14	9.72E-03	1.00E-13	1.80E-14	-3.98E-02	-4.09E-03	-1.32E-02	-2.16E-04	1.93E-03	-9.61E-02	-4.40E-11
	Shapiro-Wilk score	6.216E-01	7.258E-01	7.883E-02	5.012E-01	7.162E-01	1.698E-03	7.330E-02	9.340E-02	2.956E-02	5.637E-02	1.242E-03	1.002E-01	1.810E-02	5.951E-03	1.017E-01	2.502E-02	2.057E-02	4.291E-02	5.678E-02	9.517E-02	3.505E-01	5.649E-01	3.903E-02	3.814E-04
(Glyceraldehyde 3-phosphate dehydrogenase)	Local	1.16E-02	4.98E-03	9.03E-01	2.92E-03	3.54E-02	-1.85E-16	5.23E-04	1.14E-03	9.81E-05	2.96E-04	-9.79E-15	1.05E-02	0.00E+00	0.00E+00	-1.78E-03	7.65E-16	6.61E-16	-4.28E-04	-2.23E-03	-6.32E-04	-5.04E-05	-2.40E-02	5.89E-02	2.91E-18
	Min	-3.87E-01	-3.04E-01	-6.18E+00	-1.51E-02	-2.04E-01	-9.54E-08	-8.23E-02	-5.17E+00	-5.05E-03	-1.92E-01	-4.14E-08	-6.10E+00	-2.68E-11	-2.25E-11	-3.84E+01	-7.21E-11	-6.83E-12	-5.09E+01	-5.50E+01	-1.57E+01	-2.82E-01	-4.43E+00	-4.29E+02	-5.32E-10
	Max	1.02E+00	1.68E+00	4.31E+02	9.42E-01	2.17E+00	5.80E-08	1.10E+01	2.93E+01	1.25E+00	4.40E+00	8.10E-11	1.14E+02	1.11E-11	1.56E-10	6.20E+00	1.49E-10	1.08E-11	1.15E+00	2.11E+00	2.83E+00	8.51E-03	3.84E-01	6.14E+00	1.19E-10
	Normalized peak height	1.38E-01	9.46E-02	5.54E-01	6.93E-02	2.16E-02	9.99E-01	8.55E-01	8.68E-01	9.54E-01	8.45E-01	1.00E+00	6.56E-01	9.68E-01	9.88E-01	8.47E-01	9.54E-01	9.23E-01	7.97E-01	7.61E-01	4.51E-01	5.86E-01	7.38E-02	5.28E-01	1.00E+00
	Peak sensitivity value	4.49E-05	1.39E-03	1.63E-01	-2.66E-04	3.20E-02	-5.80E-11	1.09E-03	1.22E-02	5.94E-04	-1.02E-03	1.88E-11	5.94E-02	2.40E-16	4.25E-14	-2.20E-02	-2.36E-14	4.06E-15	-1.57E-02	-2.87E-02	-1.11E-02	-6.74E-05	1.08E-03	-1.73E-01	-9.61E-14
	Shapiro-Wilk score	6.379E-01	7.516E-01	7.495E-02	5.122E-01	7.183E-01	1.049E-03	3.725E-02	5.162E-02	2.101E-02	4.939E-02	3.896E-04	9.176E-02	1.230E-02	3.058E-03	5.650E-02	2.733E-02	3.950E-02	7.059E-02	6.069E-02	5.308E-02	2.014E-01	5.867E-01	3.538E-02	5.785E-04
(Phosphoenolpyruvate synthesis)	Local	1.16E-02	4.98E-03	9.03E-01	2.92E-03	3.54E-02	-2.35E-16	5.23E-04	1.14E-03	9.81E-05	2.96E-04	-4.52E-14	1.05E-02	0.00E+00	0.00E+00	-1.78E-03	-2.42E-15	1.16E-15	-4.28E-04	-2.23E-03	-6.32E-04	-5.04E-05	-2.40E-02	5.89E-02	2.91E-18
	Min	-1.89E-01	-8.12E-01	-2.84E+00	-3.60E-02	-4.20E-01	-1.25E-08	-1.16E-01	-2.82E+00	-1.24E-02	-3.42E+00	-4.47E-10	-2.80E+00	-1.07E-11	-1.58E-11	-6.71E+01	-3.66E-11	-2.70E-11	-8.41E+01	-9.58E+01	-4.78E+01	-9.04E-01	-4.13E+00	-4.32E+02	-2.24E-12
	Max	1.02E+00	2.56E+00	4.34E+02	9.87E-01	2.06E+00	5.63E-08	9.97E+00	6.28E+01	2.57E+00	2.48E+00	1.34E-09	1.70E+02	6.20E-11	3.65E-11	3.91E+00	3.67E-11	2.29E-11	1.16E+00	1.46E+00	6.45E-01	1.72E-02	5.69E-01	2.76E+00	4.90E-12
	Normalized peak height	1.47E-01	1.95E-01	4.94E-01	1.02E-01	2.20E-02	9.95E-01	8.40E-01	9.30E-01	9.25E-01	7.75E-01	9.82E-01	7.33E-01	9.47E-01	9.54E-01	9.09E-01	8.71E-01	9.38E-01	7.50E-01	7.22E-01	9.02E-01	6.39E-01	8.88E-02	8.11E-01	9.98E-01
	Peak sensitivity value	3.21E-04	1.09E-03	4.83E-03	3.47E-04	3.49E-02	1.82E-11	4.72E-04	3.18E-02	-7.56E-04	-2.36E-03	-6.46E-13	5.04E-02	-2.36E-14	-1.80E-14	-3.19E-02	8.76E-15	1.76E-14	1.04E-02	-5.01E-02	-8.78E-03	1.73E-04	-1.81E-03	-6.80E-02	-4.10E-16
	Shapiro-Wilk score	6.404E-01	7.531E-01	7.515E-02	5.072E-01	7.174E-01	3.185E-03	3.426E-02	1.829E-02	1.124E-02	5.071E-02	8.032E-03	4.265E-02	1.367E-02	1.306E-02	2.319E-02	7.496E-02	2.447E-02	3.039E-02	3.322E-02	2.790E-02	1.281E-01	5.821E-01	3.586E-02	3.516E-03
(Pyruvate kinase)	Local	1.16E-02	4.98E-03	9.03E-01	2.92E-03	3.54E-02	2.24E-15	5.23E-04	1.14E-03	9.81E-05	2.96E-04	5.94E-14	1.05E-02	0.00E+00	0.00E+00	-1.78E-03	4.02E-15	-8.26E-16	-4.28E-04	-2.23E-03	-6.32E-04	-5.04E-05	-2.40E-02	5.89E-02	2.91E-18
	Min	-INF	-INF	-INF	-INF	-INF	-INF	-INF	-INF	-INF	-INF	-INF	-INF	-INF	-INF	-INF	-INF	-INF	-INF	-INF	-INF	-INF	-INF	-INF	-INF
	Max	1.05E+00	1.11E+00	7.79E+02	9.64E-01	4.03E+00	7.97E-11	1.22E+01	4.19E+01	6.11E+00	2.77E+00	2.48E-11	1.17E+02	4.74E-12	1.67E-11	3.01E+00	1.85E-10	1.51E-11	1.18E+00	1.04E+00	4.13E-01	2.01E-02	9.07E-01	2.87E+00	5.61E-12
	Normalized peak height	1.12E-01	1.33E-01	3.97E-01	6.77E-02	3.85E-02	9.98E-01	6.42E-01	8.46E-01	9.83E-01	8.72E-01	9.97E-01	4.57E-01	9.24E-01	9.33E-01	8.74E-01	9.39E-01	9.13E-01	7.24E-01	6.76E-01	8.87E-01	6.91E-01	1.10E-01	9.19E-01	1.00E+00
	Peak sensitivity value	-2.90E-04	1.29E-04	4.28E-01	-3.22E-04	3.24E-02	-2.19E-12	-5.02E-03	1.95E-03	5.00E-04	7.44E-05	9.00E-13	-1.30E-02	-4.10E-16	-9.25E-15	-2.31E-02	-4.38E-14	-2.47E-14	1.11E-03	-3.40E-02	-9.73E-03	-1.42E-04	-2.04E-03	1.39E-01	-3.12E-12
	Shapiro-Wilk score	6.389E-01	7.513E-01	4.145E-02	5.189E-01	7.200E-01	5.661E-04	2.552E-02	2.897E-02	6.213E-03	5.336E-02	5.170E-04	6.497E-02	5.118E-02	3.505E-02	3.652E-02	2.643E-02	1.413E-02	4.803E-02	4.700E-02	3.143E-02	1.594E-01	5.790E-01	1.958E-02	3.809E-04
(Pyruvate decarboxylase)	Local	1.16E-02	4.98E-03	9.03E-01	2.92E-03	3.54E-02	-8.93E-16	5.23E-04	1.14E-03	9.81E-05	2.96E-04	-6.66E-16	1.05E-02	0.00E+00	0.00E+00	-1.78E-03	-6.65E-16	1.91E-15	-4.28E-04	-2.23E-03	-6.32E-04	-5.04E-05	-2.40E-02	5.89E-02	2.91E-18
	Min	-1.95E-01	-1.10E+00	-1.40E+01	-7.25E-03	-6.28E-01	-5.85E-12	-9.55E-02	-3.73E+00	-8.14E-02	-5.25E-01	-1.04E-11	-6.64E+00	-9.87E-12	-2.92E-11	-2.62E+02	-1.32E-10	-1.91E-11	-1.24E+03	-1.48E+03	-2.48E+02	-7.81E-01	-6.85E+00	-4.04E+03	-9.33E-12
	Max	1.33E+00	1.06E+00	4.04E+03	1.68E+00	3.42E+00	2.72E-11	8.79E+01	1.73E+02	4.92E+00	2.91E+00	3.49E-12	2.97E+03	3.45E-12	1.82E-11	6.07E+00	2.54E-10	1.63E-11	1.29E+00	2.97E+00	5.12E+00	2.52E+00	1.25E+00	1.29E+01	1.02E-09
	Normalized peak height	1.47E-01	1.58E-01	9.70E-01	1.19E-01	3.45E-02	9.18E-01	9.85E-01	9.79E-01	9.83E-01	7.80E-01	9.49E-01	9.93E-01	8.49E-01	9.63E-01	9.50E-01	9.53E-01	8.27E-01	9.95E-01	7.94E-01	9.86E-01	8.73E-01	8.75E-02	9.89E-01	1.00E+00
	Peak sensitivity value	7.70E-04	5.37E-04	2.09E-01	3.47E-04	3.02E-02	1.26E-14	3.65E-02	7.22E-02	1.03E-03	-1.08E-03	-4.69E-15	7.94E-01	-5.95E-15	-6.80E-15	3.36E-02	9.52E-14	-1.73E-14	-5.66E-01	-7.45E-01	-7.91E-02	-2.77E-04	2.03E-03	-1.29E+00	4.65E-13
	Shapiro-Wilk score	6.356E-01	7.531E-01	7.851E-03	5.107E-01	7.195E-01	2.306E-02	3.501E-03	7.044E-03	8.749E-03	5.371E-02	1.254E-02	2.746E-03	4.020E-02	1.924E-02	6.871E-03	2.063E-02	2.982E-02	2.088E-03	2.346E-03	5.893E-03	4.207E-02	5.797E-01	5.062E-03	4.117E-04
(Alcohol dehydrogenase)	Local	1.01E-02	4.37E-03	7.92E-01	2.32E-03	2.81E-02	-3.80E-16	9.18E-03	2.01E-02	1.72E-03	5.20E-03	6.30E-16	1.84E-01	0.00E+00	0.00E+00	-3.13E-02	-1.84E-16	1.26E-15	-7.52E-03	-3.91E-02	-1.11E-02	-8.85E-04	-1.89E-02	5.21E-02	2.13E-18
	Min	-4.13E+00	-4.13E+00	-4.54E+00	-6.54E-01	-9.77E+00	-5.67E-12	-8.78E-03	-9.78E-01	-1.91E-02	-9.57E-04	-5.39E-12	-2.45E+00	-1.74E-11	-6.95E-11	-1.39E+02	-1.58E-10	-8.62E-11	-4.24E+01	-5.44E+01	-5.99E+01	-3.89E-01	-7.77E+00	-3.73E+02	-5.48E-12
	Max	1.43E+00	1.77E+00	3.74E+02	9.59E-01	3.88E+00	4.09E-12	6.60E+01	1.36E+02	5.82E+00	1.22E+01	2.19E-11	1.38E+02	2.23E-11	3.18E-11	2.40E+00	8.37E-11	7.60E-11	1.00E+00	1.73E+00	3.09E-01	1.20E-03	8.61E-01	3.38E+00	7.56E-10
	Normalized peak height	2.83E-01	2.37E-01	3.85E-01	1.04E-01	1.18E-01	9.07E-01	9.32E-01	9.03E-01	9.70E-01	8.91E-01	9.72E-01	2.74E-01	9.66E-01	9.78E-01	8.64E-01	9.19E-01	9.87E-01	4.17E-01	3.67E-01	8.20E-01	4.51E-01	9.26E-02	7.75E-01	1.00E+00
	Peak sensitivity value	1.26E-03	9.11E-04	1.97E-01	-1.33E-04	2.69E-02	-1.46E-15	2.42E-02	5.25E-02	1.39E-03	5.14E-03	-9.72E-15	1.60E-01	-5.53E-15	2.77E-14	-6.44E-02	-9.97E-14	2.63E-14	-1.70E-02	-4.16E-02	-2.19E-02	-1.62E-04	2.50E-03	1.83E-01	2.32E-13
	Shapiro-Wilk score	6.219E-01	7.386E-01	9.463E-02	5.085E-01	7.062E-01	5.033E-02	1.660E-02	2.412E-02	1.033E-02	5.659E-02	4.448E-03	1.351E-01	1.360E-02	7.756E-03	2.908E-02	2.149E-02	5.084E-03	8.889E-02	1.009E-01	3.910E-02	3.459E-01	5.619E-01	4.455E-02	7.527E-04
(Ethanol out)	Local	1.01E-02	4.37E-03	7.92E-01	2.32E-03	2.81E-02	-4.21E-16	9.18E-03	2.01E-02	1.72E-03	5.20E-03	5.13E-16	1.84E-01	2.37E-16	-8.88E-16	-3.13E-02	-1.07E-16	1.31E-15	-7.52E-03	-3.91E-02	-1.11E-02	-8.85E-04	-1.89E-02	5.21E-02	2.13E-18
	Min	-5.65E+00	-8.59E+00	-5.01E+00	-1.01E+00	-4.56E+00	-1.00E-11	-1.40E-03	-2.51E+00	-4.56E-03	-6.99E-04	-1.52E-12	-3.74E+00	-1.01E-11	-6.11E-12	-2.82E+01	-2.97E-10	-2.70E-11	-9.55E+01	-1.77E+02	-6.40E+01	-4.82E-01	-8.33E+00	-4.17E+02	-2.68E-11
	Max	1.29E+00	2.06E+00	4.18E+02	9.34E-01	4.16E+00	2.31E-11	5.16E+00	2.78E+01	6.87E+00	1.47E+01	2.53E-11	3.37E+02	1.37E-11	6.74E-11	3.72E+00	1.04E-10	1.60E-10	1.71E+00	9.90E-01	9.93E-01	1.50E-03	8.62E-01	4.83E+00	2.39E-11
	Normalized peak height	2.93E-01	2.86E-01	4.09E-01	1.06E-01	7.85E-02	8.75E-01	5.74E-01	5.22E-01	9.47E-01	9.08E-01	9.84E-01	6.20E-01	9.50E-01	9.70E-01	5.16E-01	9.53E-01	9.88E-01	4.23E-01	5.91E-01	7.99E-01	4.81E-01	9.63E-02	8.74E-01	9.99E-01
	Peak sensitivity value	8.41E-04	7.61E-04	-1.41E-01	1.20E-03	2.88E-02	-1.54E-14	1.18E-03	1.71E-02	-1.12E-03	6.65E-03	-3.40E-15	1.70E-01	7.97E-16	2.17E-14	-4.60E-03	-1.44E-13	-1.37E-14	9.45E-03	1.17E-02	-1.45E-02	-1.91E-04	2.69E-03	-2.18E-02	-2.15E-14
	Shapiro-Wilk score	6.188E-01	7.238E-01	7.396E-02	5.049E-01	7.182E-01	1.612E-02	2.037E-01	1.083E-01	6.657E-03	5.507E-02	3.092E-03	6.594E-02	2.861E-02	7.814E-03	1.307E-01	1.214E-02	3.036E-03	4.867E-02	4.178E-02	4.135E-02	3.244E-01	5.557E-01	3.362E-02	1.429E-03

**(Ethanol flow)**												
Local	1.01E-02	4.37E-03	7.92E-01	2.32E-03	2.81E-02	-3.81E-16	9.18E-03	2.01E-02	1.72E-03	5.20E-03	-1.74E-16	1.84E-01
Min	-5.27E+00	-2.91E+00	-6.04E+00	-1.29E+00	-5.60E+00	-1.36E-10	-2.78E-02	-3.32E+00	-8.31E-03	-1.17E-03	-1.45E-12	-4.61E+00
Max	1.37E+00	1.65E+00	1.14E+03	9.01E-01	9.31E+00	6.15E-12	1.34E+01	3.90E+01	1.94E+00	7.47E+00	4.60E-11	2.65E+02
Normalized peak height	2.13E-01	1.53E-01	7.71E-01	1.72E-01	1.31E-01	9.85E-01	7.69E-01	6.57E-01	9.18E-01	8.48E-01	9.92E-01	4.87E-01
Peak sensitivity value	-1.42E-03	2.86E-03	2.44E-01	9.81E-04	2.84E-02	-5.01E-14	5.69E-03	8.66E-03	4.41E-04	2.57E-03	-4.56E-16	1.02E-01
Shapiro-Wilk score	6.222E-01	7.366E-01	3.255E-02	5.114E-01	7.135E-01	1.440E-03	9.181E-02	1.117E-01	2.184E-02	6.670E-02	1.777E-03	6.147E-02
**(Glycerol synthesis)**												
Local	2.51E-02	1.08E-02	1.96E+00	8.63E-03	1.04E-01	8.06E-15	-8.18E-02	-1.79E-01	-1.53E-02	-4.63E-02	1.65E-14	-1.64E+00
Min	-1.44E+00	-9.36E-01	-2.40E+00	-1.01E-01	-1.73E-01	-1.73E-11	-4.09E+00	-2.90E+00	-1.55E-01	-3.68E+01	-1.32E-11	-6.95E+00
Max	1.53E+01	1.77E+01	7.13E+03	3.35E+00	1.04E+01	1.46E-11	7.75E+01	8.16E+01	3.13E+00	2.02E+00	1.35E-11	5.77E+02
Normalized peak height	4.06E-01	3.37E-01	9.89E-01	1.37E-01	6.22E-02	9.06E-01	5.05E-01	3.51E-01	6.73E-01	8.73E-01	8.87E-01	6.06E-01
Peak sensitivity value	7.60E-03	5.60E-03	1.16E+00	3.81E-04	4.29E-02	9.09E-15	3.25E-02	1.84E-02	1.06E-03	-1.71E-02	1.21E-14	-8.23E-01
Shapiro-Wilk score	6.105E-01	6.921E-01	3.778E-03	4.979E-01	7.107E-01	4.197E-02	7.622E-02	8.961E-02	3.672E-02	6.042E-02	3.010E-02	4.749E-02
**(Glycerol out)**												
Local	2.51E-02	1.08E-02	1.96E+00	8.63E-03	1.04E-01	8.12E-15	-8.18E-02	-1.79E-01	-1.53E-02	-4.63E-02	1.55E-14	-1.64E+00
Min	-1.37E+00	-5.45E-01	-5.43E+00	-3.60E-02	-1.38E+00	-2.57E-11	-3.51E+00	-9.49E+00	-2.21E-01	-4.84E+01	-8.80E-11	-6.43E+00
Max	3.33E+01	2.17E+01	2.99E+03	6.56E+00	1.63E+01	5.07E-12	1.77E+01	2.52E+02	2.53E+00	2.00E+00	9.50E-12	2.77E+03
Normalized peak height	3.82E-01	2.92E-01	5.65E-01	1.78E-01	1.03E-01	9.15E-01	5.81E-01	6.48E-01	6.35E-01	7.98E-01	9.82E-01	6.81E-01
Peak sensitivity value	-4.27E-03	-1.33E-03	-9.32E-01	3.06E-04	4.17E-02	2.98E-15	-5.30E-03	3.59E-02	9.08E-04	1.09E-02	-6.45E-15	5.12E-01
Shapiro-Wilk score	5.398E-01	6.823E-01	1.138E-02	4.719E-01	7.001E-01	3.833E-02	3.485E-01	2.174E-02	4.299E-02	4.811E-02	4.784E-03	5.615E-03
**(Glycerol flow)**												
Local	2.51E-02	1.08E-02	1.96E+00	8.63E-03	1.04E-01	8.14E-15	-8.18E-02	-1.79E-01	-1.53E-02	-4.63E-02	1.49E-14	-1.64E+00
Min	-4.85E-01	-3.51E-01	-1.07E+00	-2.22E-02	-9.21E-03	-2.44E-11	-4.30E+00	-5.54E+00	-1.83E-01	-2.49E+01	-5.14E-11	-7.64E+00
Max	1.24E+01	9.25E+00	1.04E+03	9.20E+00	8.82E+00	8.77E-12	6.15E+01	3.52E+01	2.29E+00	1.86E+00	4.24E-12	3.24E+02
Normalized peak height	2.70E-01	2.18E-01	7.07E-01	2.29E-01	5.17E-02	9.15E-01	6.56E-01	4.04E-01	5.29E-01	8.19E-01	9.60E-01	3.91E-01
Peak sensitivity value	-1.41E-03	-4.29E-04	4.93E-01	8.93E-04	3.93E-02	-2.23E-15	6.84E-03	-1.89E-02	-1.32E-03	-5.18E-03	-1.21E-14	-8.41E-01
Shapiro-Wilk score	6.256E-01	7.335E-01	3.306E-02	4.327E-01	7.202E-01	4.277E-02	1.066E-01	2.530E-01	5.332E-02	7.638E-02	9.090E-03	9.089E-02
**(Acetaldehyde out)**												
Local	2.51E-02	1.08E-02	1.96E+00	8.63E-03	1.04E-01	-5.92E-15	-8.18E-02	-1.79E-01	-1.53E-02	-4.63E-02	4.56E-21	-1.64E+00
Min	-9.83E-01	-6.39E-01	-2.94E+00	-1.31E-01	-5.02E-01	-8.37E-12	-5.15E+00	-4.64E+00	-2.23E-01	-6.81E+01	-9.02E-11	-7.92E+00
Max	3.09E+01	2.88E+01	7.33E+02	4.58E+00	8.22E+00	1.15E-11	1.84E+01	3.12E+01	5.25E+00	1.99E+00	1.15E-11	1.74E+02
Normalized peak height	2.79E-01	2.80E-01	4.42E-01	1.35E-01	5.09E-02	8.48E-01	5.86E-01	3.03E-01	9.17E-01	8.91E-01	9.77E-01	2.17E-01
Peak sensitivity value	1.98E-02	-6.14E-03	3.73E-01	3.70E-03	4.25E-02	-4.33E-15	-4.95E-03	-1.20E-03	-1.17E-03	-7.67E-03	-3.11E-15	-7.23E-01
Shapiro-Wilk score	5.744E-01	6.152E-01	4.596E-02	4.931E-01	7.165E-01	8.294E-02	3.237E-01	1.859E-01	1.896E-02	4.044E-02	7.160E-03	1.624E-01
**(Acetaldehyde flow)**												
Local	2.55E-02	1.10E-02	1.99E+00	8.78E-03	1.06E-01	-5.73E-15	-8.32E-02	-1.82E-01	-1.56E-02	-4.71E-02	-8.63E-15	-1.67E+00
Min	-2.96E-01	-8.43E-01	-2.53E+01	-1.41E-01	-1.63E-01	-8.82E-11	-5.47E+00	-3.32E+00	-2.01E+00	-4.75E+01	-9.00E-12	-6.05E+00
Max	1.23E+01	2.17E+01	1.26E+03	5.80E+00	9.75E+00	1.56E-11	8.92E+00	6.31E+01	3.46E+00	8.89E+00	1.01E-10	4.70E+02
Normalized peak height	3.01E-01	3.15E-01	5.36E-01	1.60E-01	5.70E-02	9.00E-01	4.06E-01	4.63E-01	6.89E-01	8.40E-01	9.82E-01	4.27E-01
Peak sensitivity value	4.60E-04	7.52E-04	-1.75E-01	4.26E-03	5.03E-02	4.77E-14	3.33E-03	-3.46E-02	2.11E-03	5.37E-03	-3.08E-14	-5.81E-01
Shapiro-Wilk score	6.111E-01	6.387E-01	2.750E-02	4.784E-01	7.140E-01	1.163E-02	4.327E-01	1.257E-01	2.827E-02	5.041E-02	3.641E-03	6.347E-02
**(Cyanide-Acetaldehyde flow)**												
Local	2.36E-02	1.02E-02	1.85E+00	8.13E-03	9.84E-02	-5.30E-15	-7.71E-02	-1.69E-01	-1.45E-02	-4.36E-02	-8.00E-15	-1.54E+00
Min	-1.04E+00	-7.72E-01	-3.15E+00	-2.98E-02	-2.16E-01	-5.12E-11	-3.27E+00	-7.23E+00	-2.43E-01	-4.09E+01	-6.91E-12	-5.54E+00
Max	1.43E+01	2.27E+01	9.10E+02	2.22E+00	1.23E+01	4.20E-12	1.07E+01	2.13E+01	1.46E+00	6.20E+00	2.58E-10	1.74E+02
Normalized peak height	2.77E-01	2.14E-01	5.66E-01	1.23E-01	7.94E-02	9.37E-01	5.03E-01	2.34E-01	7.39E-01	8.19E-01	9.86E-01	2.18E-01
Peak sensitivity value	-2.55E-03	1.51E-02	5.06E-02	5.42E-04	4.04E-02	1.24E-14	-1.11E-03	-2.18E-02	4.84E-05	7.35E-03	1.08E-13	-7.93E-01
Shapiro-Wilk score	5.734E-01	5.972E-01	4.358E-02	4.932E-01	7.033E-01	1.509E-02	4.253E-01	2.164E-01	6.179E-02	4.336E-02	1.304E-03	1.454E-01
**(Cyanide flow)**												
Local	2.36E-02	1.02E-02	1.85E+00	8.13E-03	9.84E-02	-5.36E-15	-7.71E-02	-1.69E-01	-1.45E-02	-4.36E-02	-7.92E-15	-1.54E+00
Min	-5.55E-01	-2.75E-01	-1.74E+01	-2.11E-01	-1.00E+00	-5.91E-12	-4.02E+00	-1.16E+01	-1.40E-01	-5.99E+01	-1.35E-11	-1.20E+01
Max	INF	INF	INF	INF	INF	INF	INF	INF	INF	INF	INF	INF
Normalized peak height	3.16E-01	3.60E-01	9.98E-01	1.85E-01	1.03E-01	8.33E-01	5.84E-01	9.96E-01	9.07E-01	8.93E-01	9.74E-01	9.98E-01
Peak sensitivity value	-2.60E-04	2.38E-03	1.43E+00	2.65E-03	3.69E-02	-7.05E-15	5.52E-02	-5.55E+00	5.34E-04	-3.12E-02	1.84E-15	-2.59E+00
Shapiro-Wilk score	6.105E-01	6.088E-01	7.570E-04	4.780E-01	6.770E-01	5.816E-02	2.864E-02	4.930E-04	1.066E-02	4.196E-02	1.612E-02	8.305E-04
**(Storage)**												
Local	1.21E-02	5.20E-03	9.43E-01	-1.53E-03	-1.85E-02	-1.69E-15	9.90E-03	2.16E-02	1.86E-03	5.60E-03	-3.99E-15	1.98E-01
Min	-INF	-INF	-INF	-INF	-INF	-INF	-INF	-INF	-INF	-INF	-INF	-INF
Max	7.48E+00	6.56E+00	4.30E+03	1.20E+00	4.58E+00	1.87E-10	3.97E+01	2.18E+02	9.87E+00	1.38E+01	1.84E-11	1.53E+03
Normalized peak height	2.63E-01	2.94E-01	9.23E-01	1.72E-01	4.74E-02	9.80E-01	8.69E-01	9.23E-01	9.35E-01	8.89E-01	9.38E-01	9.34E-01
Peak sensitivity value	-8.22E-04	7.25E-03	-1.23E-02	-6.16E-04	-8.51E-03	-5.70E-14	1.37E-02	6.03E-02	-2.72E-03	6.80E-03	-6.64E-15	4.49E-01
Shapiro-Wilk score	6.082E-01	6.951E-01	1.033E-02	6.711E-01	8.474E-01	7.672E-03	5.002E-02	1.548E-02	8.493E-03	5.864E-02	2.632E-02	1.325E-02
**(ATP consumption)**												
Local	-1.59E-03	-6.83E-04	-1.24E-01	2.57E-02	3.11E-01	-8.56E-16	4.80E-03	1.05E-02	9.01E-04	2.72E-03	-2.17E-15	9.61E-02
Min	-3.25E+01	-4.31E+00	-1.15E+01	-2.22E-01	-3.15E+00	-6.47E-11	-2.40E-03	-1.34E+00	-3.61E-04	-2.26E-05	-1.27E-11	-3.25E+00
Max	1.20E+00	1.18E+00	5.19E+02	2.34E+00	3.40E+01	7.13E-11	5.45E+00	5.65E+01	4.90E+00	2.25E+01	1.45E-11	3.27E+02
Normalized peak height	5.61E-01	2.18E-01	5.86E-01	7.14E-02	1.19E-01	9.63E-01	6.01E-01	7.91E-01	9.76E-01	9.53E-01	9.68E-01	5.65E-01
Peak sensitivity value	6.93E-03	-3.16E-04	-1.43E-01	-7.99E-04	1.36E-01	-3.16E-14	3.25E-04	1.49E-02	2.09E-03	1.12E-02	2.39E-15	2.13E-01
Shapiro-Wilk score	2.692E-01	6.409E-01	7.788E-02	5.832E-01	6.119E-01	1.541E-02	1.825E-01	5.734E-02	1.130E-02	3.859E-02	1.671E-02	5.191E-02
**(Adenylate kinase)**												
Local	-INF	-INF	-INF	-INF	-INF	-INF	-INF	-INF	-INF	-INF	-INF	-INF
Min	-INF	-INF	-INF	-INF	-INF	-INF	-INF	-INF	-INF	-INF	-INF	-INF
Max	INF	INF	INF	INF	INF	INF	INF	INF	INF	INF	INF	INF
Normalized peak height	7.42E-01	6.77E-01	6.13E-01	4.84E-01	5.48E-01	4.52E-01	5.16E-01	4.19E-01	5.48E-01	3.87E-01	5.48E-01	3.87E-01
Peak sensitivity value	-5.96E-04	-7.22E-04	-1.74E-04	-4.03E-04	3.42E-04	-1.82E-03	4.60E-04	-1.72E-03	8.04E-04	-3.08E-04	-7.42E-06	-3.42E-04
Shapiro-Wilk score	3.049E-01	1.940E-01	2.578E-01	3.460E-01	2.674E-01	2.188E-01	1.852E-01	3.672E-01	2.220E-01	3.058E-01	5.463E-01	3.032E-01

**(Ethanol flow)**												
Local	-3.41E-16	-1.11E-15	-3.13E-02	-1.73E-16	1.29E-15	-7.52E-03	-3.91E-02	-1.11E-02	-8.85E-04	-1.89E-02	5.21E-02	2.13E-18
Min	-2.44E-11	-6.11E-11	-5.43E+01	-2.40E-10	-4.64E-11	-7.20E+01	-1.60E+02	-4.92E+01	-7.02E-01	-1.85E+01	-1.14E+03	-1.90E-10
Max	8.57E-11	2.14E-11	6.23E+00	3.75E-10	1.79E-11	8.38E-01	2.26E+00	7.51E-01	2.85E-02	1.46E+00	4.39E+00	3.89E-11
Normalized peak height	9.74E-01	9.79E-01	7.26E-01	9.79E-01	9.66E-01	4.01E-01	5.83E-01	8.05E-01	5.49E-01	1.94E-01	9.08E-01	9.99E-01
Peak sensitivity value	-4.25E-14	4.60E-15	-2.90E-02	-4.38E-14	-1.80E-14	-1.44E-04	-1.01E-01	-2.34E-02	-3.56E-04	-8.16E-03	3.97E-01	2.57E-14
Shapiro-Wilk score	4.089E-03	1.011E-02	1.119E-01	9.099E-03	8.827E-03	7.336E-02	3.071E-02	4.461E-02	3.261E-01	5.390E-01	1.630E-02	1.209E-03
**(Glycerol synthesis)**												
Local	0.00E+00	0.00E+00	2.79E-01	8.97E-15	-6.51E-15	6.70E-02	3.48E-01	9.89E-02	7.89E-03	-7.27E-02	1.24E-01	1.03E-17
Min	-8.35E-11	-8.70E-11	-9.84E+01	-1.09E-10	-1.55E-11	-3.91E+02	-1.48E+02	-9.38E+01	-6.49E-01	-5.12E+00	-7.13E+03	-4.47E-10
Max	1.78E-11	3.58E-11	2.09E+00	2.10E-10	2.32E-11	9.51E-01	1.24E+00	8.11E-01	5.19E-01	1.57E+00	1.14E+01	4.87E-11
Normalized peak height	8.80E-01	9.43E-01	4.89E-01	8.65E-01	9.11E-01	5.04E-01	3.73E-01	5.53E-01	3.59E-01	7.15E-02	9.87E-01	9.99E-01
Peak sensitivity value	-4.61E-14	-1.88E-14	2.85E-02	-1.32E-13	4.85E-16	-2.96E-02	1.26E-01	6.42E-03	-5.19E-05	1.10E-03	7.34E-01	-1.75E-13
Shapiro-Wilk score	2.195E-02	3.380E-02	9.851E-02	4.010E-02	4.280E-02	2.479E-02	6.447E-02	6.426E-02	3.715E-01	6.478E-01	2.405E-03	1.042E-03
**(Glycerol out)**												
Local	0.00E+00	0.00E+00	2.79E-01	9.24E-15	-6.55E-15	6.70E-02	3.48E-01	9.89E-02	7.89E-03	-7.27E-02	1.24E-01	1.03E-17
Min	-2.56E-11	-6.84E-11	-2.53E+02	-3.24E-10	-7.35E-12	-7.05E+02	-1.92E+03	-1.42E+02	-2.66E-01	-6.85E+00	-2.99E+03	-1.43E-11
Max	2.59E-11	2.28E-10	4.61E+00	7.71E-11	1.59E-11	2.30E+00	2.26E+00	1.04E+00	5.57E-01	2.75E+00	4.03E+00	1.21E-10
Normalized peak height	9.16E-01	9.70E-01	7.13E-01	9.20E-01	8.80E-01	5.39E-01	8.21E-01	4.47E-01	3.53E-01	9.18E-02	9.82E-01	9.99E-01
Peak sensitivity value	-8.38E-15	1.24E-14	1.09E-01	-1.01E-13	5.15E-16	-1.74E-01	-6.29E-01	-3.70E-02	1.23E-04	-9.28E-04	-4.61E-01	-2.02E-14
Shapiro-Wilk score	5.128E-02	1.321E-02	3.577E-02	2.192E-02	6.310E-02	1.051E-02	2.899E-03	3.199E-02	3.804E-01	6.473E-01	6.338E-03	5.381E-04
**(Glycerol flow)**												
Local	0.00E+00	0.00E+00	2.79E-01	9.72E-15	-6.66E-15	6.70E-02	3.48E-01	9.89E-02	7.89E-03	-7.27E-02	1.24E-01	1.03E-17
Min	-1.61E-11	-2.03E-11	-6.99E+01	-1.77E-09	-1.50E-11	-7.43E+01	-9.18E+01	-1.62E+02	-2.88E-01	-4.68E+00	-1.04E+03	-7.70E-12
Max	6.78E-11	2.20E-10	9.46E-01	8.45E-11	9.75E-11	9.54E-01	9.02E-01	8.35E-01	5.21E-01	1.09E+00	2.55E+00	4.84E-09
Normalized peak height	8.96E-01	9.62E-01	3.39E-01	9.85E-01	9.62E-01	1.96E-01	2.36E-01	5.34E-01	2.72E-01	7.02E-02	9.44E-01	1.00E+00
Peak sensitivity value	-3.49E-14	4.35E-14	-1.07E-02	1.73E-14	-6.35E-15	1.27E-02	1.14E-01	1.02E-01	-1.45E-04	5.60E-04	-5.84E-02	-4.22E-13
Shapiro-Wilk score	3.242E-02	1.506E-02	1.853E-01	2.594E-03	1.003E-02	1.773E-01	1.035E-01	2.004E-02	3.855E-01	6.486E-01	1.696E-02	3.868E-04
**(Acetaldehyde out)**												
Local	0.00E+00	0.00E+00	2.79E-01	-5.24E-15	8.08E-15	6.70E-02	3.48E-01	9.89E-02	7.89E-03	-7.27E-02	1.24E-01	1.03E-17
Min	-2.63E-11	-8.87E-11	-4.47E+01	-4.20E-11	-2.31E-11	-5.49E+01	-1.20E+02	-5.02E+01	-7.71E-01	-8.18E+00	-7.31E+02	-3.09E-11
Max	6.90E-11	1.60E-10	2.79E+00	8.80E-11	1.04E-11	1.70E+00	1.04E+00	8.48E-01	4.95E-01	1.35E+00	4.99E+00	1.75E-11
Normalized peak height	9.48E-01	9.71E-01	3.66E-01	8.11E-01	9.03E-01	1.97E-01	3.00E-01	3.97E-01	2.90E-01	9.15E-02	8.85E-01	9.98E-01
Peak sensitivity value	2.28E-15	3.17E-14	1.11E-02	5.56E-14	-9.97E-15	2.79E-02	1.31E-01	5.25E-03	7.77E-04	-5.40E-04	2.08E-01	1.99E-14
Shapiro-Wilk score	2.684E-02	1.349E-02	2.263E-01	6.542E-02	4.348E-02	2.452E-01	9.133E-02	1.242E-01	3.619E-01	6.392E-01	2.174E-02	1.639E-03
**(Acetaldehyde flow)**												
Local	0.00E+00	0.00E+00	2.84E-01	-5.10E-15	7.94E-15	6.82E-02	5.75E-01	-1.21E-01	-9.62E-03	-7.39E-02	1.27E-01	1.05E-17
Min	-2.01E-11	-2.06E-10	-6.58E+01	-2.38E-10	-3.64E-11	-3.02E+02	-1.24E+02	-8.10E+01	-2.15E+00	-6.09E+00	-1.26E+03	-4.94E-12
Max	2.96E-11	6.93E-11	4.55E+00	6.77E-11	3.59E-11	2.42E+00	2.40E+00	1.22E+00	1.35E-06	1.53E+00	2.63E+01	7.29E-11
Normalized peak height	9.28E-01	9.68E-01	4.08E-01	9.27E-01	9.45E-01	6.69E-01	2.02E-01	2.90E-01	3.66E-01	8.34E-02	9.56E-01	9.99E-01
Peak sensitivity value	-9.63E-15	-8.78E-14	1.08E-02	3.93E-15	1.78E-14	1.34E-01	4.34E-01	-1.38E-01	-1.07E-03	-3.68E-04	-1.02E-01	-4.11E-15
Shapiro-Wilk score	4.509E-02	1.137E-02	1.900E-01	3.267E-02	2.504E-02	3.729E-02	1.130E-01	9.029E-02	3.619E-01	6.541E-01	1.325E-02	7.052E-04
**(Cyanide-Acetaldehyde flow)**												
Local	0.00E+00	0.00E+00	2.63E-01	-4.73E-15	7.34E-15	6.31E-02	-3.93E-01	8.14E-01	6.50E-02	-6.85E-02	1.17E-01	9.70E-18
Min	-5.96E-11	-3.65E-11	-2.36E+01	-8.61E-11	-6.58E-11	-1.09E+02	-8.25E+01	-2.60E+01	-1.92E-01	-3.61E+00	-9.08E+02	-3.79E-11
Max	1.71E-11	6.32E-11	2.59E+00	1.64E-09	1.23E-10	1.72E+00	3.95E-01	1.03E+00	9.98E-01	1.30E+00	2.81E+00	8.48E-10
Normalized peak height	9.46E-01	9.42E-01	2.95E-01	9.78E-01	9.77E-01	2.66E-01	1.38E-01	7.14E-02	6.36E-02	7.81E-02	6.21E-01	1.00E+00
Peak sensitivity value	1.39E-14	3.24E-14	3.87E-03	-5.21E-13	-4.62E-14	1.11E-01	-5.59E-01	8.30E-01	9.14E-04	-1.69E-03	-3.80E-01	-2.78E-13
Shapiro-Wilk score	2.756E-02	2.448E-02	2.969E-01	2.521E-03	6.485E-03	1.275E-01	1.403E-01	3.824E-01	5.554E-01	6.361E-01	2.158E-02	4.739E-04
**(Cyanide flow)**												
Local	0.00E+00	0.00E+00	2.63E-01	-4.59E-15	7.47E-15	6.31E-02	-3.93E-01	8.14E-01	6.50E-02	-6.85E-02	1.17E-01	9.70E-18
Min	-1.65E-11	-5.02E-11	-1.22E+04	-1.32E-09	-1.98E-11	-1.22E+04	-6.33E+03	-2.14E+02	-2.35E-01	-8.86E+00	-3.76E+04	-1.15E-11
Max	INF	INF	INF	INF	INF	INF	INF	INF	INF	INF	INF	INF
Normalized peak height	9.04E-01	9.86E-01	9.83E-01	9.84E-01	9.51E-01	1.00E+00	9.98E-01	4.77E-01	1.24E-01	9.13E-02	9.98E-01	9.99E-01
Peak sensitivity value	3.64E-15	-8.76E-16	6.03E+00	-5.87E-14	6.65E-15	-3.86E+00	-1.65E+00	9.04E-01	1.46E-03	1.90E-03	-3.76E+00	-2.08E-15
Shapiro-Wilk score	6.844E-02	3.924E-03	5.426E-04	2.759E-03	2.082E-02	6.611E-04	1.070E-03	4.177E-02	5.525E-01	6.257E-01	6.341E-04	2.519E-03
**(Storage)**												
Local	0.00E+00	0.00E+00	-3.37E-02	-2.74E-15	-1.38E-16	-8.11E-03	-4.21E-02	-1.20E-02	-9.55E-04	1.62E-02	-9.65E-02	-1.73E-18
Min	-INF	-INF	-INF	-INF	-INF	-INF	-INF	-INF	-INF	-INF	-INF	-INF
Max	2.07E-11	2.14E-11	3.30E+00	1.37E-10	3.52E-11	1.91E+00	9.07E-01	3.67E-01	8.04E-03	1.05E+00	4.20E+00	6.08E-11
Normalized peak height	9.34E-01	9.61E-01	8.94E-01	8.58E-01	9.55E-01	9.65E-01	8.11E-01	5.84E-01	4.47E-01	7.30E-02	7.14E-01	9.99E-01
Peak sensitivity value	3.13E-15	-1.59E-13	-8.65E-02	3.04E-13	4.64E-14	-6.00E-02	7.81E-02	7.36E-02	1.20E-04	6.06E-04	-2.25E+00	2.13E-14
Shapiro-Wilk score	3.823E-02	3.292E-03	2.218E-02	3.267E-02	1.572E-02	7.148E-03	1.417E-02	1.210E-02	2.888E-01	8.039E-01	8.201E-03	1.270E-03
**(ATP consumption)**												
Local	0.00E+00	0.00E+00	-1.64E-02	-1.37E-15	-6.97E-17	-3.93E-03	-2.04E-02	-5.81E-03	-4.63E-04	-2.32E-01	9.53E-01	2.55E-17
Min	-6.61E-12	-1.94E-11	-6.36E+01	-1.74E-10	-3.35E-11	-1.13E+02	-1.59E+02	-5.87E+01	-5.06E-01	-6.85E+00	-5.17E+02	-1.69E-10
Max	6.19E-12	2.37E-11	2.80E+00	1.68E-10	1.54E-11	9.41E-01	1.93E+00	4.88E-01	1.71E-02	1.95E+00	4.20E+00	1.91E-11
Normalized peak height	8.98E-01	9.21E-01	7.77E-01	9.22E-01	8.78E-01	7.39E-01	7.15E-01	8.34E-01	3.92E-01	2.21E-02	7.94E-01	9.99E-01
Peak sensitivity value	-2.24E-16	1.74E-14	-2.45E-02	4.54E-14	2.05E-14	-2.31E-02	-8.64E-02	-1.45E-02	1.07E-04	-2.26E-01	8.07E-01	3.68E-15
Shapiro-Wilk score	7.280E-02	3.421E-02	7.540E-02	4.401E-02	3.986E-02	4.569E-02	3.537E-02	2.412E-02	3.157E-01	8.249E-01	4.514E-02	8.334E-04
**(Adenylate kinase)**												
Local	-INF	-INF	-INF	-INF	-INF	-INF	-INF	-INF	-INF	-INF	-INF	-INF
Min	-INF	-INF	-INF	-INF	-INF	-INF	-INF	-INF	-INF	-INF	-INF	-INF
Max	INF	INF	INF	INF	INF	INF	INF	INF	INF	INF	INF	INF
Normalized peak height	7.10E-01	8.39E-01	5.48E-01	9.68E-01	9.68E-01	3.87E-01	5.16E-01	3.23E-01	4.84E-01	6.13E-01	2.90E-01	4.52E-01
Peak sensitivity value	-5.78E-20	-3.69E-20	3.22E-04	2.53E-09	2.23E-11	-9.12E-05	-7.46E-05	7.51E-06	-2.79E-07	-1.29E-04	1.34E-05	-3.60E-16
Shapiro-Wilk score	2.066E-01	1.823E-01	2.626E-01	1.756E-01	1.756E-01	2.900E-01	3.642E-01	3.055E-01	3.520E-01	2.766E-01	3.868E-01	2.783E-01
